# Refining the relationship between gut microbiota and common hematologic malignancies: insights from a bidirectional Mendelian randomization study

**DOI:** 10.3389/fcimb.2024.1412035

**Published:** 2024-06-14

**Authors:** Pengyin Chen, Jiaxin Guo, Wei Wang, Anhua Feng, Lili Qin, Yuyuan Hu, Nannan Lyu, Haiying Wang

**Affiliations:** ^1^ Department of Hematology, Affiliated Hospital of Shandong Second Medical University, Weifang, China; ^2^ School of Clinical Medicine, Shandong Second Medical University, Weifang, China

**Keywords:** gut microbiota, MR, hematologic malignancies, IVW, SNPs

## Abstract

**Background:**

The relationship between gut microbiota and hematologic malignancies has attracted considerable attention. As research progresses, it has become increasingly clear that the composition of gut microbiota may influence the onset and progression of hematologic malignancies. However, our understanding of this association remains limited.

**Methods:**

In our study, we classified gut microbiota into five groups based on information at the phylum, class, order, family, and genus levels. Subsequently, we obtained data related to common hematologic malignancies from the IEU Open GWAS project. We then employed a bidirectional Mendelian Randomization (MR) approach to determine whether there is a causal relationship between gut microbiota and hematologic malignancies. Additionally, we conducted bidirectional MR analyses to ascertain the directionality of this causal relationship.

**Results:**

Through forward and reverse MR analyses, we found the risk of lymphoid leukemia was significantly associated with the abundance of phylum Cyanobacteria, order Methanobacteriales, class Methanobacteria, family Peptococcaceae, family Methanobacteriaceae, and genera Lachnospiraceae UCG010, Methanobrevibacter, Eubacterium brachy group, and Butyrivibrio. The risk of myeloid leukemia was significantly associated with the abundance of phylum Actinobacteria, phylum Firmicutes, order Bifidobacteriales, order Clostridiales, class Actinobacteria, class Gammaproteobacteria, class Clostridia, family Bifidobacteriaceae, and genera Fusicatenibacter, Eubacterium hallii group, Blautia, Collinsella, Ruminococcus gauvreauii group, and Bifidobacterium. The risk of Hodgkin lymphoma was significantly associated with the abundance of family Clostridiales vadinBB60 group, genus Peptococcus, and genus Ruminococcaceae UCG010. The risk of malignant plasma cell tumor was significantly associated with the abundance of genera Romboutsia and Eubacterium rectale group. The risk of diffuse large B-cell lymphoma was significantly associated with the abundance of genera Erysipelatoclostridium and Eubacterium coprostanoligenes group. The risk of mature T/NK cell lymphomas was significantly associated with the abundance of phylum Verrucomicrobia, genus Ruminococcaceae UCG013, genus Lachnoclostridium, and genus Eubacterium rectale group. Lastly, the risk of myeloproliferative neoplasms was significantly associated with the abundance of genus Coprococcus 3 and Eubacterium hallii group.

**Conclusion:**

Our study provided new evidence for the causal relationship between gut microbiota and hematologic malignancies, offering novel insights and approaches for the prevention and treatment of these tumors.

## 1 Introduction

Hematologic malignancies are tumors of the bone marrow and lymphatic system caused by disruption of normal hematopoietic function, accounting for 6.5% of all cancers worldwide (Tietsche de Moraes Hungria et al., 2019; Kocarnik et al., 2022). Common types include lymphoid leukemia, myeloid leukemia, Hodgkin lymphoma, malignant plasma cell tumor, follicular lymphoma, diffuse large B-cell lymphoma, mature T/NK cell lymphomas, and myeloproliferative neoplasms, among others. The rapid aging of the population poses challenges to healthcare systems and increases the burden of hematologic malignancies. Approximately 1.2 million new cases of hematologic malignancies are diagnosed worldwide each year, resulting in around 690,000 deaths. Hematologic malignancies continue to be the most common type of cancer in children (Song et al., 2020). The gut microbiota, also known as the gut microbiome, refers to the collective term for micro-organisms residing in the human intestine, including bacteria, fungi, viruses, etc. These microorganisms coexist with humans and play vital roles in health and disease development (Adak and Khan, 2019). The gut microbiota is essential in regulating immune system functions, maintaining immune balance, and preventing abnormal immune responses, potentially reducing the incidence of hematologic cancers (Zhou et al., 2021). Certain gut microbes have anti-inflammatory properties that can mitigate chronic inflammation, a factor closely linked to cancer onset and progression (Guevara-Ramírez et al., 2023). The gut microbiota can influence the metabolism and efficacy of anticancer drugs; by modulating the microbiota composition, it may be possible to enhance therapeutic effects and reduce side effects (Uribe-Herranz et al., 2023). A healthy gut microbiota helps maintain the integrity of the intestinal barrier, preventing harmful substances and pathogens from entering the bloodstream, thereby reducing damage to the bone marrow and lymphatic system (Wang et al., 2022). However, the gut microbiota can also have adverse effects on the body. An imbalance in the gut microbiota (such as dysbiosis) can lead to immune dysfunction, increasing the risk of infections and inflammation, thereby promoting the occurrence and progression of hematologic cancers (Cheng et al., 2020). Certain pathogenic microorganisms may directly or indirectly participate in the formation and development of hematologic malignancies. For example, some bacteria and viruses may promote tumor growth by inducing chronic inflammation or immunosuppression. Additionally, the gut microbiota may interact with chemotherapeutic drugs, affecting their efficacy and toxicity (Leigh et al., 2022; Wang et al., 2023).

Some studies have explored changes in gut microbiota in mouse models and patients with hematologic malignancies and found that the composition of microbiota may vary with different types of hematologic diseases. Kostic et al. demonstrated that the relative abundance of Prevotella maculosa and Edwardsiella tarda was lower in patients with acute lymphoblastic leukemia (ALL) (Kostic et al., 2013; Schirmer et al., 2016; Liu et al., 2020). Additionally, Chua et al. found lower abundance of Faecalibacterium in ALL patients (Chua et al., 2017). Another study reported that patients with chronic lymphoid leukemia had higher abundance of Bacteroides, Parabacteroides, Acinetobacter, and Prevotella, while Lachnospiraceae and Ruminococcaceae were less abundant (Faitová et al., 2022). Yuan et al. characterized the gut microbiota of 25 untreated diffuse large B-cell lymphoma patients, finding higher abundance of Proteobacteria at the phylum level, and significant increases in Clostridium butyricum and Escherichia coli species. According to Zhang et al., multiple myeloma (MM) patients had higher abundance of Clostridium leptum and Pseudomonas aeruginosa in their gut microbiota. Specifically, the level of Clostridium leptum was higher in MM patients at late stages of the disease (Zhang et al., 2019). Currently, most observational studies mainly analyze the composition and changes of gut microbiota in patient feces. However, traditional observational studies have inherent limitations, such as inadequate study design to determine causality and difficulty controlling other interfering factors (Boyko, 2013). Although randomized controlled trials (RCTs) are the gold standard for verifying causality, conducting RCTs may be challenging due to the diversity and complexity of gut microbiota (Steeger et al., 2021). The composition and function of gut microbiota are influenced by many factors, including individual genetic background, dietary habits, lifestyle, and environmental factors (Wan et al., 2019; Wan et al., 2020). In addition, the dynamic changes and interactions of microbiota also increase the complexity of RCTs. Therefore, new methods are needed to explore the causal relationship between gut microbiota and hematologic malignancies.

MR is a highly useful experimental design method that utilizes genetic variation existing in nature to randomly allocate individuals to different environmental exposure conditions. This method, similar to randomized controlled trials, can better assess the impact of environmental factors on specific phenotypes or disease occurrences (Smith and Ebrahim, 2003; Sleiman and Grant, 2010). In our study, we utilized MR methods to comprehensively analyze eight common hematologic malignancies from the IEU Open GWAS project through two-sample MR analysis. These malignancies include myeloid leukemia, lymphoid leukemia, Hodgkin lymphoma, diffuse large B-cell lymphoma, follicular lymphoma, myeloproliferative neoplasms, mature T/NK cell lymphomas, and malignant plasma cell tumor. By studying the causal relationship between gut microbiota and these hematologic malignancies, we hope to gain a deeper understanding of the pathogenesis of these diseases and provide more effective strategies for their future prevention and treatment.

## 2 Methods

### 2.1 Study design

**Figure 1:**
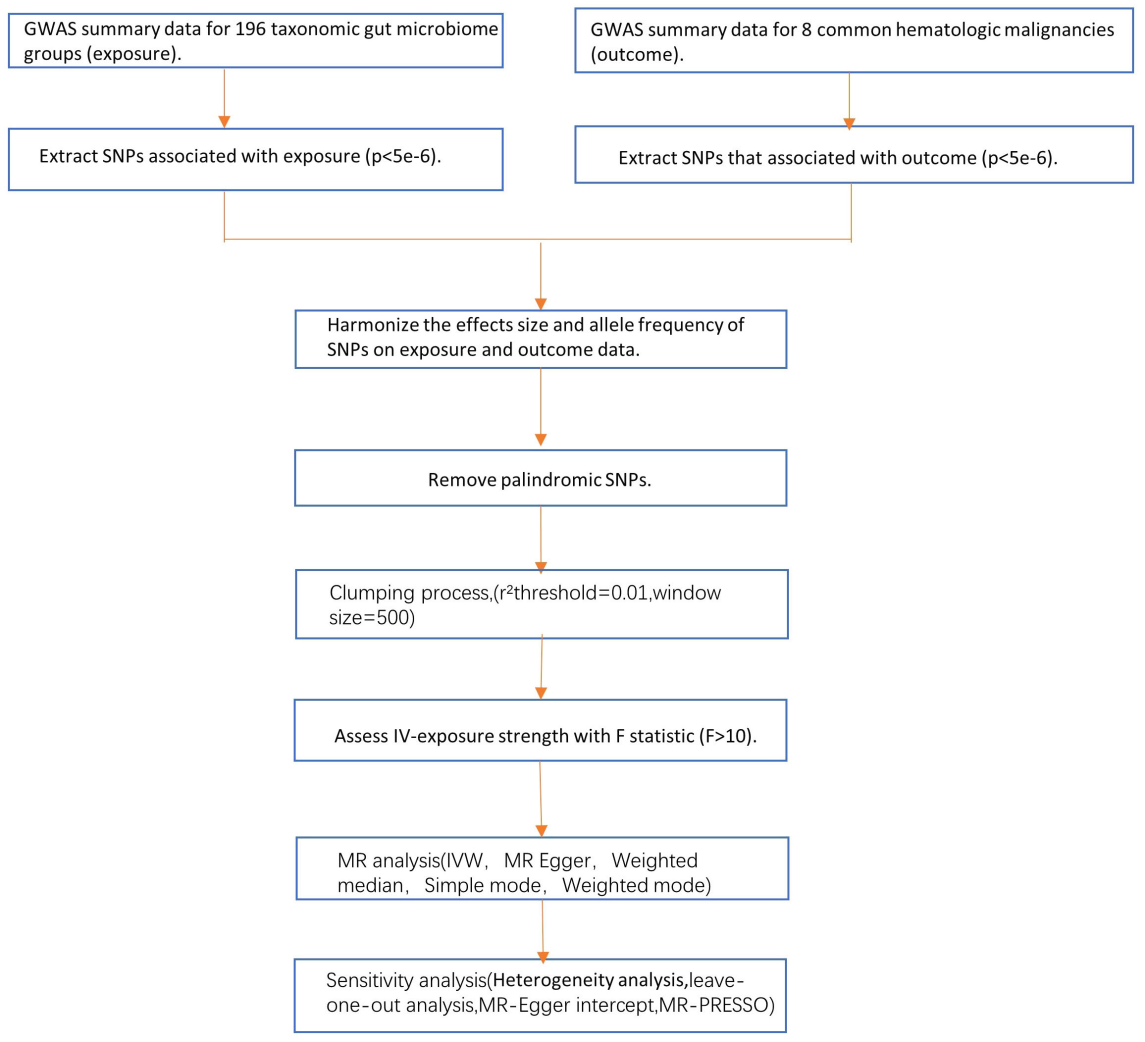
The mind map of the research design for this study.

**Figure 2:**
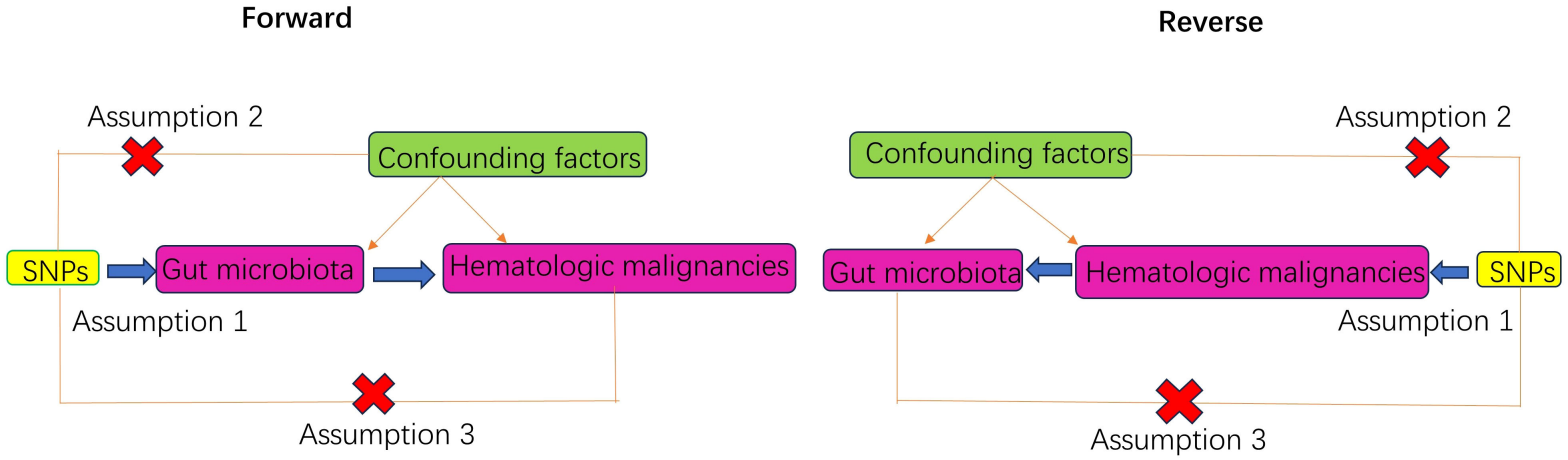
Principle diagram of MR analysis.

In the forward MR analysis, we regarded gut microbiota as the exposure factor, while eight common hematologic malignancies were considered as the outcomes. In the reverse MR analysis, we treated the eight hematologic malignancies as the exposure factors and gut microbiota as the outcomes. We rigorously selected instrumental variables (IVs) through a strict quality control process and conducted analyses using MR techniques. Throughout this analysis, we operated based on three critical assumptions: (1) significant correlation between IVs and exposure factors; (2) independence of IVs from any potential confounding factors; (3) IVs only affect outcomes through exposure factors without any other influence (Sekula et al., 2016; Bowden and Holmes, 2019) (**Figures 1**, **2**).

### 2.2 Data sources

The MiBioGen International Consortium GWAS dataset provided SNP data for gut microbiota and is one of the largest multi-ethnic GWAS datasets to date. The study encompassed 18,340 participants from 24 cohorts, with the majority being of European ancestry. By collecting both 16S ribosomal RNA gene sequencing profiles and genotype data from these participants, the study aimed to explore the relationship between human autosomal genetic variation and gut microbiome composition. The analysis covered a total of 211 taxonomic groups, including 35 families, 20 orders, 16 classes, 9 phyla, and 131 genera (Kurilshikov et al., 2021). As 15 bacterial traits lacked specific species names, we excluded these traits and ultimately selected 196 bacterial traits for further analysis. We obtained data related to eight common hematologic malignancies from the IEU Open GWAS project. Except for the SNP data for myeloproliferative neoplasms, which was not sourced from the FinnGen database, all others were sourced from the FinnGen database (Kurki et al., 2023). Detailed information is provided in **Supplementary Table 1**.

### 2.3 Instrumental variable selection

To ensure the stability and reliability of MR analysis results, we employed the following criteria for IV selection: (1) SNPs with p-values below the genome-wide statistical significance threshold (p < 5 × 10^-6^) were chosen as IVs; (2) to avoid linkage disequilibrium (LD), we used a clumping procedure to select independent SNPs (r2 < 0.01, distance > 500 kb); (3) IVs closely correlated with outcomes (p < 5 × 10^-6^) were removed; (4) to ensure that the effect of SNPs on exposure corresponded to their effect on outcomes, we eliminated palindromic SNPs (e.g., those with A/T or G/C alleles); (5) we calculated the F statistic to prevent weak IVs from influencing outcomes, where F=[R2*(N-2)]/(1-R2), with R2=[2β²EAF*(1-EAF)]/[2β²EAF*(1-EAF)+2SE²NEAF(1-EAF)], and EAF representing the ef-fect allele frequency (Davey Smith and Hemani, 2014; Gupta et al., 2017), with IVs selected having an F value greater than 10.

### 2.4 Statistical analysis

All analyses were performed using R version 4.3.2. We utilized several R packages, including “TwoSampleMR” (version 0.5.10), “GagnonMR”, “ieugwasr”, “data.table”, “dplyr”, “plyr”, among others. In our analysis, p-values less than 0.05 indicated potential causal relationships, while p-values less than 0.01 denoted significant causal relationships. The MR methods employed included Weighted mode (Hartwig et al., 2017), Weighted median (Bowden et al., 2016), MR Egger (Bowden et al., 2015), and Inverse variance weighted (IVW) (Burgess et al., 2013), Simple mode with IVW being the primary analysis method. Additionally, we conducted a series of sensitivity analyses, including heterogeneity testing, pleiotropy testing, and leave-one-out sensitivity testing. Cochran’s Q test was used to assess the degree of heterogeneity in the study (Greco et al., 2015). For pleiotropy testing, we utilized both the MR-Egger intercept method and the MR Pleiotropy RESidual Sum and Outlier (MR-PRESSO) global test to assess potential influences of other factors on the level of causal effect (Burgess et al., 2013).. Furthermore, we performed leave-one-out analysis and generated plots to evaluate whether MR conclusions depended on specific SNPs. If certain SNPs were found to significantly influence significant MR conclusions, we considered excluding these instrumental variables to further validate the robustness of the results.

## 3 Results

### 3.1 Selection of SNPs

To analyze the influence of 196 gut microbial taxa on hematological malignancies, a total of 1407 SNPs related to gut microbiota were selected at the levels of phylum, class, order, family, and genus, which were 66, 153, 121, 245, and 822, respectively, with a significance level of 5 × 10^-6^ (**Supplementary Table 2**). To analyze the influence of hematological malignancies on gut microbiota, a total of 82 SNPs related to hematological malignancies were selected at eight levels, including lymphoid leukemia, myeloid leukemia, Hodgkin lymphoma, malignant plasma cell tumor, follicular lymphoma, diffuse large B-cell lymphoma, mature T/NK-cell lymphomas, and myeloproliferative neoplasms, which were 16, 8, 10, 7, 12, 8, 10, and 11, respectively, with a significance level of 5 × 10^-6^ (**Supplementary Table 3**). After statistical calculation, all SNP F values were greater than 10, indicating that the results of the analysis were not affected by weak instrumental variables.

### 3.2 Bidirectional causal relationship between gut microbiota and common hematological malignancies

We conducted bidirectional MR analysis of gut microbiota and hematological malignancies, and all analysis results are shown in **Supplementary Tables 4** and **5**.

#### 3.2.1 Lymphoid leukemia

In the forward MR analysis, using the IVW method, it was observed that the relative abundance of class Methanobacteria (OR = 2.371, 95% CI: 1.387-4.055, p = 0.002), family Desulfovibrionaceae (OR = 1.864, 95% CI: 1.002-3.466, p = 0.049), family Methanobacteriaceae (OR = 2.371, 95% CI: 1.387-4.055, p = 0.002), family Peptococcaceae (OR = 2.055, 95% CI: 1.191-3.545, p = 0.01), family Prevotellaceae (OR = 2.102, 95% CI: 1.103-4.004, p = 0.024), genus Clostridium innocuum group (OR = 1.89, 95% CI: 1.033-3.455, p = 0.039), genus Methanobrevibacter (OR = 2.369, 95% CI: 1.337-4.196, p = 0.003), genus Ruminiclostridium 9 (OR = 2.318, 95% CI: 1.082-4.965, p = 0.031), order Desulfovibrionales (OR = 1.874, 95% CI: 1.008-3.484, p = 0.047), and order Methanobacteriales (OR = 2.371, 95% CI: 1.387-4.055, p = 0.002) was positively associated with the risk of lymphoid leukemia. Conversely, the relative abundance of class Negativicutes (OR = 0.452, 95% CI: 0.220-0.929, p = 0.031), family Lactobacillaceae (OR = 0.505, 95% CI: 0.262-0.972, p = 0.041), genus Coprococcus 3 (OR = 0.263, 95% CI: 0.089-0.777, p = 0.016), genus Family XIII UCG001 (OR = 0.363, 95% CI: 0.145-0.906, p = 0.03), genus Holdemania (OR = 0.532, 95% CI: 0.306-0.926, p = 0.026), genus Ruminiclostridium 6 (OR = 0.505, 95% CI: 0.265-0.960, p = 0.037), and order Selenomonadales (OR = 0.452, 95% CI: 0.220-0.929, p = 0.031) was inversely associated with the risk of lymphoid leukemia.

In the reverse MR analysis, using the IVW method, it was observed that the susceptibility to lymphoid leukemia was positively associated with the relative abundance of family Enterobacteriaceae (OR = 1.032, 95% CI: 1.004-1.062, p = 0.026), genus Eubacterium brachy group (OR = 1.083, 95% CI: 1.029-1.139, p = 0.002), genus Butyrivibrio (OR = 1.09, 95% CI: 1.028-1.155, p = 0.004), genus Escherichia Shigella (OR = 1.031, 95% CI: 1.001-1.062, p = 0.044), genus Lachnospiraceae NC2004 group (OR = 1.043, 95% CI: 1.000-1.088, p = 0.05), and order Enterobacteriales (OR = 1.032, 95% CI: 1.004-1.062, p = 0.026). Conversely, the susceptibility to lymphoid leukemia was negatively associated with the relative abundance of genus Anaerofilum (OR = 0.954, 95% CI: 0.911-0.999, p = 0.046), genus Fusicatenibacter (OR = 0.974, 95% CI: 0.950-0.998, p = 0.036), and genus Lachnospiraceae UCG010 (OR = 0.957, 95% CI: 0.930-0.985, p = 0.002).

#### 3.2.2 Myeloid leukemia

In the forward MR analysis, using the IVW method, it was observed that the relative abundance of class Gammaproteobacteria (OR = 9.035, 95%CI: 1.835-44.483, p = 0.007), genus Lachnospiraceae UCG008 (OR = 1.985, 95%CI: 1.019-3.868, p = 0.044), and genus Slackia (OR = 2.366, 95%CI: 1.024-5.470, p = 0.044) was positively correlated with the risk of myeloid leukemia. Conversely, the relative abundance of class Coriobacteriia (OR = 0.216, 95%CI: 0.062-0.749, p = 0.016), family Coriobacteriaceae (OR = 0.216, 95%CI: 0.062-0.749, p = 0.016), genus Dorea (OR = 0.246, 95%CI: 0.067-0.896, p = 0.033), genus Prevotella 9 (OR = 0.399, 95%CI: 0.182-0.873, p = 0.021), genus Turicibacter (OR = 0.349, 95%CI: 0.151-0.806, p = 0.014), and order Coriobacteriales (OR = 0.216, 95%CI: 0.062-0.749, p = 0.016) was negatively correlated with the risk of myeloid leukemia.

In the reverse MR analysis, using the IVW method, it was observed that the susceptibility to myeloid leukemia was positively associated with the relative abundance of class Actinobacteria (OR = 1.054, 95%CI: 1.024-1.085, p = 4.00E-04), class Clostridia (OR = 1.04, 95%CI: 1.012-1.070, p = 0.005), family Bifidobacteriaceae (OR = 1.057, 95%CI: 1.025-1.090, p = 4.00E-04), family Lachnospiraceae (OR = 1.039, 95%CI: 1.011-1.069, p = 0.006), genus Eubacterium hallii group (OR = 1.048, 95%CI: 1.017-1.080, p = 0.002), genus Ruminococcus gauvreauii group (OR = 1.048, 95%CI: 1.014-1.082, p = 0.005), genus Bifidobacterium (OR = 1.056, 95%CI: 1.024-1.089, p = 0.001), genus Blautia (OR = 1.05, 95%CI: 1.015-1.086, p = 0.005), genus Collinsella (OR = 1.043, 95%CI: 1.011-1.077, p = 0.008), genus Fusicatenibacter (OR = 1.046, 95%CI: 1.016-1.076, p = 0.002), genus Slackia (OR = 1.055, 95%CI: 1.006-1.107, p = 0.029), order Bifidobacteriales (OR = 1.057, 95%CI: 1.025-1.090, p = 4.00E-04), order Clostridiales (OR = 1.041, 95%CI: 1.012-1.070, p = 0.005), phylum Actinobacteria (OR = 1.047, 95%CI: 1.018-1.076, p = 0.001), and phylum Firmicutes (OR = 1.049, 95%CI: 1.021-1.079, p = 0.001). Conversely, the susceptibility to myeloid leukemia was negatively associated with the relative abundance of class Bacteroidia (OR = 0.966, 95%CI: 0.940-0.994, p = 0.016), genus Ruminococcus gnavus group (OR = 0.951, 95%CI: 0.908-0.997, p = 0.038), order Bacteroidales (OR = 0.966, 95%CI: 0.940-0.994, p = 0.016), and phylum Bacteroidetes (OR = 0.965, 95%CI: 0.938-0.992, p = 0.011).

#### 3.2.3 Hodgkin’s lymphoma

In the forward MR analysis, using the IVW method, a positive correlation was observed between the relative abundance of class Gammaproteobacteria (OR = 4.303, 95%CI: 1.061-17.451, p = 0.041) and the risk of Hodgkin’s lymphoma. Conversely, a negative correlation was found between the relative abundance of genus Peptococcus (OR = 0.554, 95%CI: 0.360-0.854, p = 0.007) and the risk of Hodgkin’s lymphoma.

In the reverse MR analysis, using the IVW method, the susceptibility to Hodgkin’s lymphoma was positively correlated with the relative abundance of various taxa including family Clostridiales vadin BB60 group (OR = 1.055, 95%CI: 1.021-1.091, p = 0.001), genus Eubacterium ventriosum group (OR = 1.03, 95%CI: 1.002-1.059, p = 0.033), genus Odoribacter (OR = 1.032, 95%CI: 1.004-1.061, p = 0.027), genus Ruminiclostridium 9 (OR = 1.03, 95%CI: 1.004-1.057, p = 0.026), genus Ruminococcaceae NK4A214 group (OR = 1.029, 95%CI: 1.001-1.058, p = 0.039), genus Ruminococcaceae UCG003 (OR = 1.035, 95%CI: 1.006-1.064, p = 0.017), genus Ruminococcaceae UCG005 (OR = 1.036, 95%CI: 1.008-1.065, p = 0.011), genus Ruminococcaceae UCG010 (OR = 1.042, 95%CI: 1.011-1.073, p = 0.007), and genus Ruminococcaceae UCG013 (OR = 1.036, 95%CI: 1.009-1.063, p = 0.008). Additionally, the susceptibility to Hodgkin’s lymphoma was negatively correlated with the relative abundance of phylum Verrucomicrobia (OR = 0.969, 95%CI: 0.940-0.999, p = 0.044).

#### 3.2.4 Malignant plasma cell tumor

In the forward MR analysis, using the IVW method, a positive correlation was observed between the relative abundance of genus Lachnospiraceae UCG010 (OR = 2.491, 95%CI: 1.091-5.685, p = 0.03) and the risk of malignant plasma cell tumor. Conversely, a negative correlation was found between the relative abundance of class Lentisphaeria (OR = 0.577, 95%CI: 0.368-0.905, p = 0.017), genus Dorea (OR = 0.356, 95%CI: 0.152-0.832, p = 0.017), genus Lactococcus (OR = 0.666, 95%CI: 0.454-0.978, p = 0.038), genus Rikenellaceae RC9 gut group (OR = 0.627, 95%CI: 0.421-0.935, p = 0.022), genus Romboutsia (OR = 0.115, 95%CI: 0.036-0.370, p = 3.00E-04), genus Ruminococcaceae UCG014 (OR = 0.513, 95%CI: 0.266-0.992, p = 0.047), and order Victivallales (OR = 0.577, 95%CI: 0.368-0.905, p = 0.017), and the risk of malignant plasma cell tumor.

In the reverse MR analysis, using the IVW method, the susceptibility to malignant plasma cell tumor was positively correlated with the relative abundance of genus Eubacterium rectale group (OR = 1.055, 95%CI: 1.015-1.097, p = 0.007) and genus Candidatus Soleaferrea (OR = 1.077, 95%CI: 1.009-1.149, p = 0.026), while it was negatively correlated with the relative abundance of family Defluviitaleaceae (OR = 0.935, 95%CI: 0.883-0.989, p = 0.019) and genus Defluviitaleaceae UCG011 (OR = 0.934, 95%CI: 0.883-0.988, p = 0.018).

#### 3.2.5 Follicular lymphoma

In the forward MR analysis, using the IVW method, a positive correlation was observed between the relative abundance of class Clostridia (OR = 2.964, 95%CI: 1.209-7.269, p = 0.018), genus Adlercreutzia (OR = 2.021, 95%CI: 1.104-3.699, p = 0.023), genus Phascolarctobacterium (OR = 1.994, 95%CI: 1.017-3.909, p = 0.044), genus Sutterella (OR = 3.177, 95%CI: 1.215-8.307, p = 0.018), order Mollicutes RF9 (OR = 2.514, 95%CI: 1.029-6.138, p = 0.043), genus Ruminococcaceae UCG005 (OR = 1.805, 95%CI: 1.001-3.254, p = 0.05), and the risk of follicular lymphoma. Conversely, a negative correlation was found between the relative abundance of family Alcaligenaceae (OR = 0.456, 95%CI: 0.232-0.894, p = 0.022) and the risk of follicular lymphoma.

In the reverse MR analysis, using the IVW method, the susceptibility to follicular lymphoma was positively correlated with the relative abundance of family Clostridiaceae 1 (OR = 1.034, 95%CI: 1.003-1.066, p = 0.033), genus Allisonella (OR = 1.085, 95%CI: 1.002-1.174, p = 0.044), genus Clostridium sensu stricto 1 (OR = 1.032, 95%CI: 1.000-1.064, p = 0.048), and genus Rikenellaceae RC9 gut group (OR = 1.093, 95%CI: 1.020-1.170, p = 0.011). Additionally, the susceptibility to follicular lymphoma was negatively correlated with the relative abundance of family Defluviitaleaceae (OR = 0.956, 95%CI: 0.918-0.995, p = 0.027), genus Defluviitaleaceae UCG011 (OR = 0.955, 95%CI: 0.918-0.994, p = 0.026), and genus Faecalibacterium (OR = 0.972, 95%CI: 0.945-0.999, p = 0.039).

#### 3.2.6 Diffuse large B-cell lymphoma

In the forward MR analysis, using the IVW method, a negative correlation was observed between the relative abundance of genus Eubacterium coprostanoligenes group (OR = 0.123, 95%CI: 0.029-0.518, p = 0.004), class Alphaproteobacteria (OR = 0.2, 95%CI: 0.048-0.838, p = 0.028), genus Erysipelatoclostridium (OR = 0.34, 95%CI: 0.129-0.900, p = 0.03), phylum Cyanobacteria (OR = 0.359, 95%CI: 0.141-0.916, p = 0.032), and the risk of diffuse large B-cell lymphoma.

In the reverse MR analysis, using the IVW method, the susceptibility to diffuse large B-cell lymphoma was negatively correlated with the relative abundance of family Veillonellaceae (OR = 0.98, 95%CI: 0.963-0.997, p = 0.025), genus Blautia (OR = 0.982, 95%CI: 0.966-0.998, p = 0.029), genus Eggerthella (OR = 0.963, 95%CI: 0.930-0.997, p = 0.034), genus Erysipelatoclostridium (OR = 0.965, 95%CI: 0.943-0.987, p = 0.002), genus Fusicatenibacter (OR = 0.982, 95%CI: 0.966-0.999, p = 0.041), and genus Ruminococcaceae UCG009 (OR = 0.973, 95%CI: 0.949-0.999, p = 0.041).

#### 3.2.7 Mature T/NK cell lymphomas

In the forward MR analysis, using the IVW method, a positive correlation was observed between the relative abundance of family Rhodospirillaceae (OR = 3.55, 95%CI: 1.108-11.373, p = 0.033), genus Eubacterium rectale group (OR = 4.202, 95%CI: 1.017-17.360, p = 0.047), genus Ruminococcus gnavus group (OR = 3.211, 95%CI: 1.134-9.092, p = 0.028), genus Anaerostipes (OR = 7.171, 95%CI: 1.504-34.196, p = 0.013), genus Escherichia.Shigella (OR = 7.711, 95%CI: 1.331-44.666, p = 0.023), and the risk of mature T/NK cell lymphomas. Conversely, a negative correlation was found between the relative abundance of genus Erysipelatoclostridium (OR = 0.292, 95%CI: 0.093-0.917, p = 0.035) and genus Lachnospiraceae UCG001 (OR = 0.282, 95%CI: 0.089-0.891, p = 0.031), and the risk of mature T/NK cell lymphomas.

In the reverse MR analysis, using the IVW method, the susceptibility to mature T/NK cell lymphomas was positively correlated with the relative abundance of class Bacteroidia (OR = 1.026, 95%CI: 1.004-1.048, p = 0.019), family Lachnospiraceae (OR = 1.023, 95%CI: 1.002-1.045, p = 0.034), genus Eubacterium rectale group (OR = 1.031, 95%CI: 1.009-1.054, p = 0.006), genus Lachnoclostridium (OR = 1.033, 95%CI: 1.011-1.055, p = 0.004), order Bacteroidales (OR = 1.026, 95%CI: 1.004-1.048, p = 0.019), phylum Bacteroidetes (OR = 1.026, 95%CI: 1.005-1.049, p = 0.017). Additionally, the susceptibility to mature T/NK cell lymphomas was negatively correlated with the relative abundance of class Mollicutes (OR = 0.969, 95%CI: 0.943-0.996, p = 0.024), class Verrucomicrobiae (OR = 0.973, 95%CI: 0.947-0.999, p = 0.038), family Pasteurellaceae (OR = 0.969, 95%CI: 0.941-0.999, p = 0.041), family Veillonellaceae (OR = 0.973, 95%CI: 0.951-0.995, p = 0.017), family Verrucomicrobiaceae (OR = 0.973, 95%CI: 0.947-0.998, p = 0.038), genus Akkermansia (OR = 0.972, 95%CI: 0.947-0.998, p = 0.037), genus Ruminococcaceae UCG005 (OR = 0.977, 95%CI: 0.955-0.999, p = 0.044), genus Ruminococcaceae UCG010 (OR = 0.974, 95%CI: 0.949-0.999, p = 0.042), order Pasteurellales (OR = 0.969, 95%CI: 0.941-0.999, p = 0.041), order Verrucomicrobiales (OR = 0.973, 95%CI: 0.947-0.999, p = 0.038), phylum Tenericutes (OR = 0.969, 95%CI: 0.943-0.996, p = 0.024), and phylum Verrucomicrobia (OR = 0.966, 95%CI: 0.941-0.991, p = 0.008).

#### 3.2.8 Myeloproliferative neoplasms

In the forward MR analysis, using the IVW method, a positive correlation was observed between the relative abundance of family Bifidobacteriaceae (OR = 1.621, 95%CI: 1.009-2.603, p = 0.046), genus Bifidobacterium (OR = 1.532, 95%CI: 1.002-2.343, p = 0.049), order Bifidobacteriales (OR = 1.621, 95%CI: 1.009-2.603, p = 0.046), phylum Firmicutes (OR = 3.077, 95%CI: 1.161-8.156, p = 0.024), and the risk of myeloproliferative neoplasms. Conversely, a negative correlation was found between the relative abundance of genus Eubacterium hallii group (OR = 0.395, 95%CI: 0.203-0.767, p = 0.006), genus Coprococcus 3 (OR = 0.227, 95%CI: 0.075-0.686, p = 0.009), genus Haemophilus (OR = 0.527, 95%CI: 0.311-0.893, p = 0.017), and the risk of myeloproliferative neoplasms.

In the reverse MR analysis, using the IVW method, the susceptibility to myeloproliferative neoplasms was positively correlated with the relative abundance of genus Slackia (OR = 1.047, 95%CI: 1.009-1.086, p = 0.015). Additionally, the susceptibility to myeloproliferative neoplasms was negatively correlated with the relative abundance of class Erysipelotrichia (OR = 0.971, 95%CI: 0.949-0.993, p = 0.01), family Erysipelotrichaceae (OR = 0.971, 95%CI: 0.949-0.993, p = 0.01), genus Eubacterium ruminantium group (OR = 0.963, 95%CI: 0.932-0.994, p = 0.022), genus Collinsella (OR = 0.974, 95%CI: 0.951-0.998, p = 0.032), genus Coprococcus 1 (OR = 0.978, 95%CI: 0.957-1.000, p = 0.048), genus Dorea (OR = 0.974, 95%CI: 0.953-0.995, p = 0.016), genus Sellimonas (OR = 0.944, 95%CI: 0.898-0.993, p = 0.027), genus Tyzzerella 3 (OR = 0.940, 95%CI: 0.887-0.995, p = 0.034), and order Erysipelotrichales (OR = 0.971, 95%CI: 0.949-0.993, p = 0.01).

### 3.3 Significant associations between gut microbiota and hematologic malignancies

**Figure 3:**
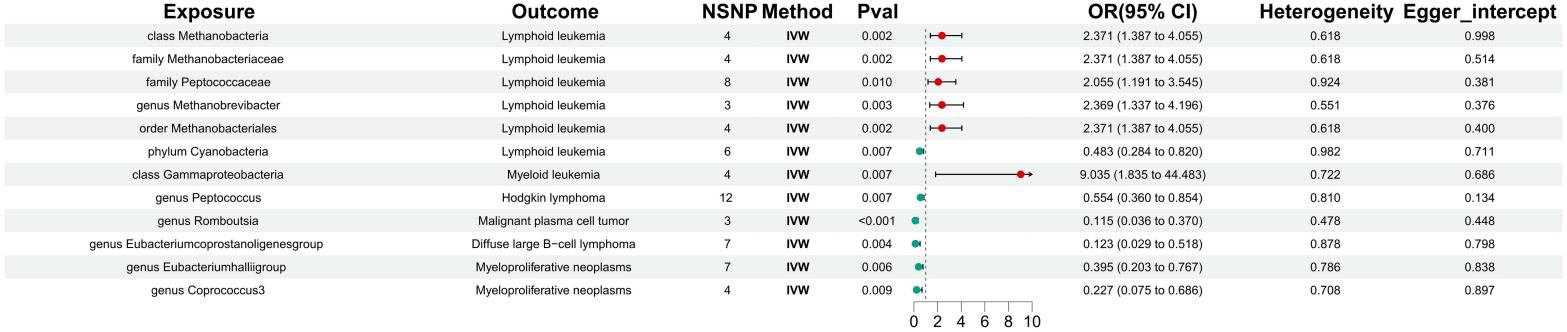
The significant correlation between gut microbiota and hematologic malignancies.

In the forward MR analysis, class Methanobacteria, family Methanobacteriaceae, fami-ly.Peptococcaceae, genus Methanobrevibacter, and order Methanobacteriales were significantly associated with a higher risk of lymphoid leukemia. Conversely, phylum Cyanobacteria was significantly associated with a lower risk of lymphoid leukemia. Additionally, class Gammaproteobacteria was linked to a higher risk of myeloid leukemia, while genus Peptococcus was significantly associated with a lower risk of Hodgkin’s lymphoma. Genus Romboutsia was significantly associated with a lower risk of malignant plasma cell tumor, and genus Eubacterium coprostanoligenes group was linked to a lower risk of diffuse large B-cell lymphoma. Notably, genus Eubacterium hallii group and genus Coprococcus3 were significantly associated with a lower risk of myeloproliferative neoplasms (**Figure 3**).

**Figure 4:**
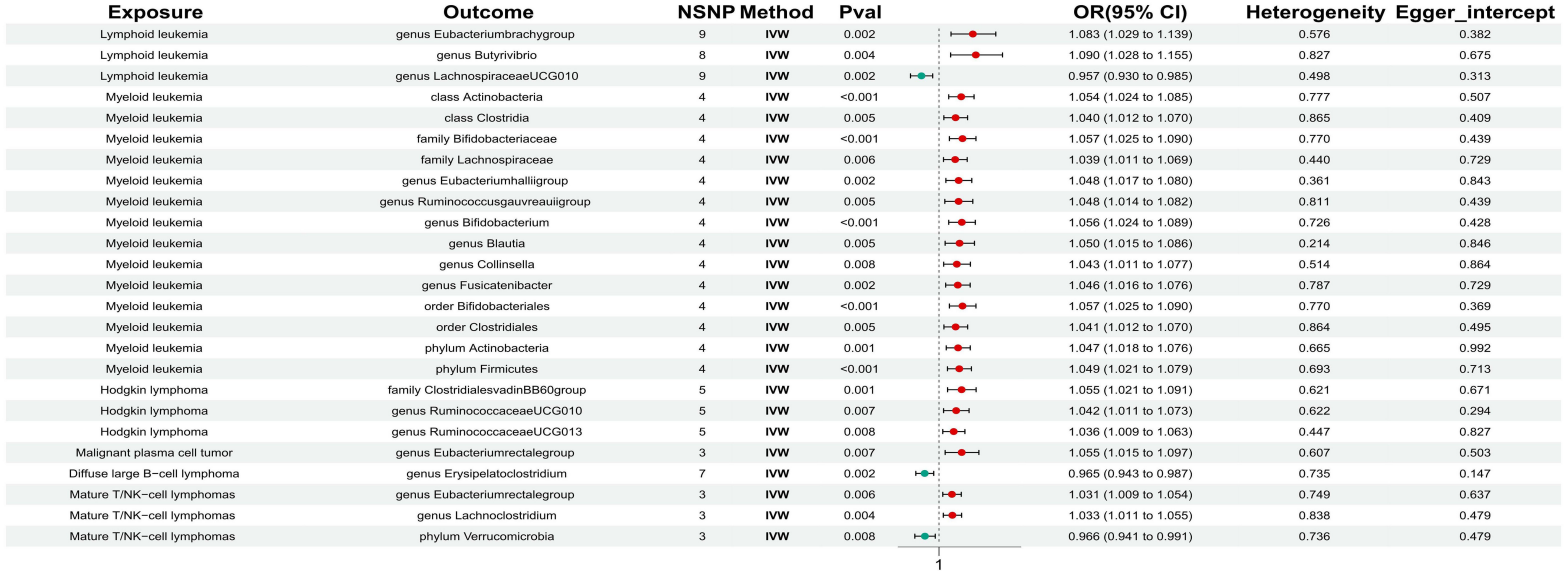
The significant correlation between hematologic malignancies and gut microbiota.

In reverse MR analysis, the risk of lymphoid leukemia was significantly associated with higher relative abundances of genus Eubacterium brachy group and genus Butyrivibrio, along with lower relative abundance of genus Lachnospiraceae UCG010. The risk of myeloid leukemia was significantly associated with higher relative abundances of class Actinobacteria, class Clostridia, family Bifidobacteriaceae, family Lachnospiraceae, genus Eubacterium hallii group, genus Ruminococcus gauvreauii group, genus Bifidobacterium, genus Blautia, genus Collinsella, genus Fusicatenibacter, order Bifidobacteriales, order Clostridiales, phylum Actinobacteria, and phylum Firmicutes. The risk of Hodgkin’s lymphoma was significantly associated with higher relative abundances of family Clostridiales vadin BB60 group, genus Ruminococcaceae UCG010, and genus Ruminococcaceae UCG013. The risk of malignant plasma cell tumor was significantly associated with higher relative abundance of genus Eubacterium rectale group, while the risk of diffuse large B-cell lymphoma was significantly associated with lower relative abundance of genus Erysipelatoclostridium. The risk of mature T/NK-cell lymphoma was significantly associated with higher relative abundances of genus Eubacterium rectale group and genus Lachnoclostridium, along with lower relative abundance of phylum Verrucomicrobia (**Figure 4**).

**Figure 5:**
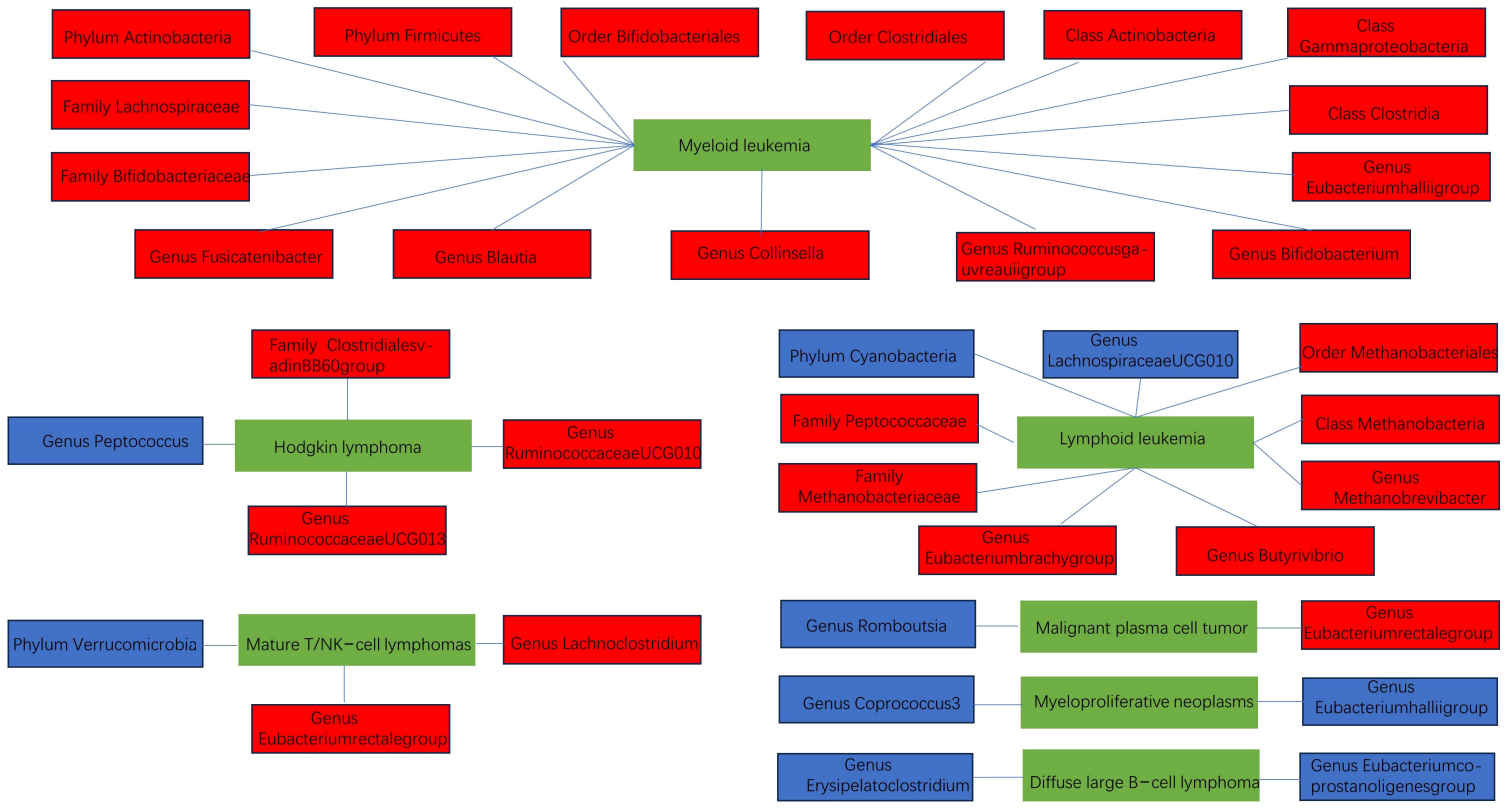
The schematic diagram of the relationship between various hematologic malignancies and the gut microbiota (blue indicates negative correlation, red indicates positive correlation).

Through the above forward and reverse MR analyses, we summarized the relationship structure between hematologic malignancies and gut microbiota in a schematic diagram, as shown in **Figure 5**.

### 3.4 Sensitivity analysis

During the heterogeneity analysis, we found that myeloproliferative neoplasms and genus Tyzzerella3 exhibited heterogeneity in the Cochran’s Q statistic in both the IVW and MR-Egger analyses. This suggested potential differences or other unconsidered factors between these two variables. However, it was encouraging that the intercept term in the MR-Egger method did not detect potential pleiotropy. This indicated that the observed heterogeneity was primarily due to biological differences or other unknown factors rather than methodological issues or the influence of confounding factors. It is worth noting that for other associations between tumors and gut microbiota, neither the IVW nor the MR-Egger Cochran’s Q statistics showed heterogeneity. Additionally, the MR-Egger intercept term and the MR-PRESSO global test did not detect potential horizontal pleiotropy (**Supplementary Tables 6–8**). This suggested that the associations between other hematologic neoplasms and gut microbiota may have been more consistent and less influenced by unknown factors. Furthermore, the results shown in the funnel plots were consistent with the Cochran’s Q statistic results, further strengthening our conclusions (**Supplementary Figures 1–8**). We also conducted leave-one-out tests, reanalyzing the data each time one SNP was removed, and the results remained stable, providing further support for the reliability of our findings (**Supplementary Figures 1-8**).

## 4 Discussion

In our study, we delved into the causal relationship between the gut microbiota and hematologic malignancies. Employing MR for the first time, we comprehensively analyzed the potential links between the composition of gut microbiota and various hematologic malignancies. Through bidirectional MR analysis, we identified a series of gut microbiota associated with hematologic malignancies, including lymphoid leukemia, myeloid leukemia, Hodgkin’s lymphoma, malignant plasma cell tumor, follicular lymphoma, diffuse large B-cell lymphoma, mature T/NK cell lymphomas, and marrow proliferative neoplasms.

Our research found that lymphoid leukemia led to a decrease in the relative abundance of genus Lachnospiraceae UCG010. Furthermore, researchers found that the reduction of Lachnospiraceae, a member of the Lachnospiraceae family, might have various impacts on the development of leukemia (Vacca et al., 2020; Masetti et al., 2021; Zhou et al., 2021). Firstly, Lachnospiraceae is associated with the host’s resistance to high radiation doses. Its reduction might lower the host’s resistance to high radiation doses, increasing the risk of leukemia development. Secondly, Lachnospiraceae is linked to the recovery of hematopoietic function and butyrate-mediated gastrointestinal repair. Since leukemia treatment often damages the hematopoietic and gastrointestinal systems, the reduction of Lachnospiraceae might affect these repair processes, thereby influencing the patient’s recovery and treatment outcomes. Additionally, studies have reported that the abundance of Lachnospiraceae is correlated with a reduction in the side effects of graft-versus-host disease (GVHD) in transplant patients. This suggests that the reduction of Lachnospiraceae might increase the risk of treatment-related complications in leukemia patients, further affecting treatment outcomes and survival rates (Ma et al., 2020; Ma et al., 2021). We also found that lymphoid leukemia led to an increase in the relative abundance of family Enterobacteriaceae. Similarly, studies have found that high abundance of Enterobacteriaceae in leukemia patients is associated with a slower rate of NK and B-cell recon-stitution. We also observed elevated levels of C-reactive protein (CRP) in these patients, indicating higher levels of inflammation in their bodies. This inflammation may be related to systemic inflammation during the gastrointestinal toxicity phase after chemotherapy, caused by pathogen-associated molecular patterns (PAMPs) released by translocated bacteria, inducing the release of inflammatory cytokines by binding with Toll-like receptors expressed by immune cells, epithelial cells, and other tissue cells, thereby affecting the patient’s treatment outcomes (Ingham et al., 2019).Our study revealed that myeloid leukemia led to an increase in the relative abundance of phylum Actinobacteria, while genus Prevotella 9 was identified as a protective factor against myeloid leukemia. Similarly, Yu et al. conducted sequencing analysis of gut microbiota composition through Illumina of 16S rRNA and found that compared to the healthy control group, patients with myeloid leukemia had an increase in the relative abundance of phylum Actinobacteria and a decrease in the relative abundance of genus Prevotella 9 (Yu et al., 2021).

Our research also revealed many previously unexplored or less-reported gut microbiota that are causally related to hematologic malignancies. For instance, our study showed associations between the relative abundance of phylum Cyanobacteria, phylum Verrucomicrobia, and order Bifidobacteriales, and the occurrence of hematologic malignancies. These microbiota may play important roles in the development of hematologic malignancies, although their exact mechanisms require further investigation. To explore these mechanisms, we provide the following explanations. Firstly, there are crucial interactions between the microbiota and the host immune system in the gut (Round and Mazmanian, 2009). Intestinal dendritic cells play a significant role by sensing bacteria in the gut and transmitting this information to the body’s immune system. This interaction influences the regulation of the intestinal immune system and to some extent determines the inflammatory environment for tumor development. For example, if Th17 cells are overly active or Treg cells cause immune suppression, it may accelerate cancer development (Knochelmann et al., 2018). Th17 cells in the intestine help protect the intestinal mucosal barrier, and the differentiation of these cells is influenced by gut microbiota. These bacteria induce Th17 cells to produce certain cytokines, such as IL-17, which may worsen the tumor microenvironment and lead to cancer deterioration (Chang, 2019; Rossi et al., 2021; Zhang et al., 2022). Secondly, gut microbiota produce short-chain fatty acids (SCFAs) by breaking down insoluble dietary fibers (Hou et al., 2022), including butyric acid, propionic acid, and acetic acid (den Besten et al., 2013), which play critical roles in regulating gut function and the immune system (Martin-Gallausiaux et al., 2021; Mirzaei et al., 2021). Butyric acid, as an anti-inflammatory molecule, can inhibit the production of pro-inflammatory cytokines by neutrophils stimulated under lipopolysaccharide (LPS), such as TNF-α and cytokine-induced neutrophil chemoattractant-2, while also alleviating LPS-induced NF-κB activation (Vinolo et al., 2011). Additionally, butyric acid suppresses histone deacetylase (HDAC) in colon cells and immune cells, reducing the production of pro-inflammatory cytokines. Therefore, SCFAs may play an important role in inhibiting the growth of hematologic malignancies by regulating the NF-κB pathway, inhibiting the release of inflammatory factors, and inducing the expression of anti-inflammatory cytokines.

During the development of hematologic malignancies, the interaction between gut microbiota and the host is a complex network regulated and influenced by various external factors. Nutrition is one significant factor, as different types of diets directly affect the composition and metabolic activity of gut microbiota. For example, a plant-based diet rich in dietary fiber can promote the growth of beneficial bacteria (Iyama et al., 2014; Makki et al., 2018), while a high-fat diet may lead to a reduction in beneficial microbial communities (Fava et al., 2013), thereby altering the community structure and function of gut microbiota (Alexander et al., 2017; Han et al., 2017; Myhrstad et al., 2020). Besides nutritional factors, drugs and treatments also greatly influence the composition and activity of gut microbiota. The use of antibiotics disrupts the balance of gut microbiota, potentially leading to the loss of probiotics and an increase in harmful bacteria, thereby affecting the normal function of the immune system (Dunn et al., 2022). Additionally, anticancer drugs and immunotherapy drugs may also affect the development and treatment outcomes of tumors by modulating gut microbiota (Sochacka-Ćwikła et al., 2021). Lifestyle factors also affect the composition and function of gut microbiota. For example, exercise, sleep quality, and stress levels are associated with the diversity and stability of microbiota, thereby affecting the activity of the host immune system and the process of tumor development (Jang et al., 2019). Environmental factors such as pollutants, chemicals, and microbial exposures may alter the composition and activity of gut microbiota. For example, prolonged exposure to heavy metals or toxic chemicals may lead to imbalance of gut microbiota, reducing beneficial microbial communities and increasing harmful microbial communities, thereby exacerbating the risk of tumor development (Kaur and Rawal, 2023; Porru et al., 2024).

Our study has several significant advantages. Firstly, traditional observational studies were susceptible to observational bias, and unaccounted confounding factors might have interfered with the results. However, through MR analysis, we effectively controlled confounding factors by randomly allocating treatment and control groups, thus avoiding such biases. Secondly, our study utilized publicly available large-scale GWAS summary statistics data, offering an efficient alternative to avoid additional experimental costs and ensuring the reliability of genetic information. We conducted a comprehensive investigation of eight common hematologic malignancies, providing strong support for the uni-versality and reliability of the research findings. Furthermore, through MR analysis, we could identify specific biomarkers or biological pathways related to gut microbiota, which could have served as potential targets for treating hematologic malignancies. This provided valuable clues and directions for future research and drug development. Lastly, based on our research findings, we could have developed further clinical trial plans to evaluate the effects of regulating gut microbiota on the treatment outcomes of hematologic malignancy patients. Such personalized treatment plans would have been more reliable and effective, contributing to improved survival rates and quality of life for patients.

However, our study also has some limitations. Firstly, we primarily studied the European population, which might have limited our comprehensive assessment of the relationship between gut microbiota and hematologic malignancies among different races, thereby affecting the universality of the research results. Secondly, the lack of support from *in vivo* or *in vitro* models made it difficult for us to directly assess the direct connection between gut microbiota and the origin of hematologic malignancies, which might have affected our interpretation and application of the research results. Thirdly, we used strict thresholds in the IV selection stage, which might have excluded some genetic predispositions related to the association between gut microbiota and tumors, thereby affecting the integrity and accuracy of the results. Finally, the lack of stratified summary data such as gender and age might have limited our in-depth analysis of the relationship between gut microbiota and tumors among different populations, affecting the thoroughness of the results.

## 5 Conclusions

In conclusion, our study provided a comprehensive assessment of the causal relationship between gut microbiota and common hematologic malignancies, revealing the complex and diverse associations between various tumor types and gut microbiota. Specifically, we found potential causal relationships between gut microbiota and various hematologic malignancies such as lymphoid leukemia, myeloid leukemia, Hodgkin’s lymphoma, multiple myeloma, follicular lymphoma, diffuse large B-cell lymphoma, mature T/NK cell lymphomas, and myeloproliferative neoplasms. This finding not only expanded our understanding of the pathogenesis of tumors but also provided new insights into the application of gut microbiota in tumor prevention, diagnosis, and treatment.

## Data availability statement

The original contributions presented in the study are included in the article/**Supplementary Material**. Further inquiries can be directed to the corresponding authors.

## Ethics statement

No further ethical approval is necessary, as this involves a re-analysis of data already in the public domain.

## Author contributions

PC: Writing – review & editing, Writing – original draft, Visualization, Validation, Methodology, Formal analysis, Conceptualization. JG: Writing – review & editing, Formal analysis, Conceptualization. WW: Writing – review & editing, Formal analysis, Conceptualization. AF: Writing – review & editing, Formal analysis, Conceptualization. LQ: Writing – review & editing, Conceptualization. YH: Writing – review & editing, Methodology. NL: Writing – review & editing, Conceptualization. HW: Writing – review & editing, Validation, Methodology, Investigation, Funding acquisition.
