# Supplementary material for: Refining the relationship between gut microbiota and common hematologic malignancies: insights from a bidirectional Mendelian randomization study
Source: Front Cell Infect Microbiol. 2024 Jun 14;14:1412035. doi: 10.3389/fcimb.2024.1412035 (PMC11224959; doi:10.3389/fcimb.2024.1412035)
Supplement: Supplementary file 1 [file DataSheet_1.pdf]

Supplementary figures:

Supplementary figure 1.Sensitivity analysis of the impact of gut microbiota on hematologic malignancies using MR.(forest plot)
Supplementary figure 2.Sensitivity analysis of the impact of gut microbiota on hematologic malignancies using MR.(funnel plot)
Supplementary figure 3.Sensitivity analysis of the impact of gut microbiota on hematologic malignancies using MR.(leaveoneout plot)
Supplementary figure 4.Sensitivity analysis of the impact of gut microbiota on hematologic malignancies using MR.(scatter plot)
Supplementary figure 5.Sensitivity analysis of the impact of hematologic malignancies on gut microbiota using MR.(forest plot)
Supplementary figure 6.Sensitivity analysis of the impact of hematologic malignancies on gut microbiota using MR.(funnel plot)
Supplementary figure 7.Sensitivity analysis of the impact of hematologic malignancies on gut microbiota tumors using MR.(leaveoneout plot)
Supplementary figure 8.Sensitivity analysis of the impact of hematologic malignancies on gut microbiota using MR.(scatter plot)

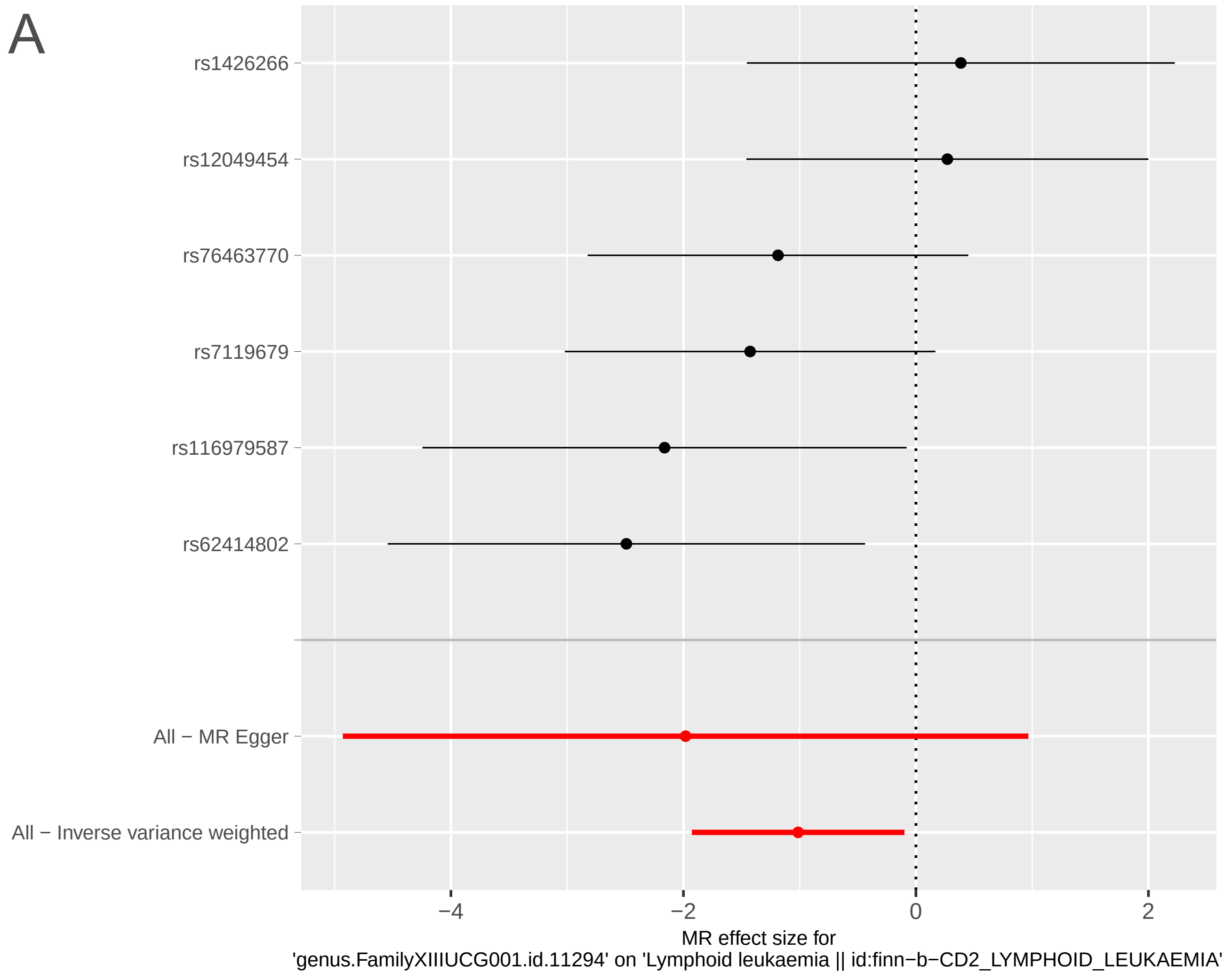
A
rs1426266
rs12049454
rs76463770
rs7119679
rs116979587
rs62414802
All − MR Egger
All − Inverse variance weighted
−4
−2
0
2
MR effect size for
'genus.FamilyXIIIUCG001.id.11294' on 'Lymphoid leukaemia || id:finn−b−CD2_LYMPHOID_LEUKAEMIA'

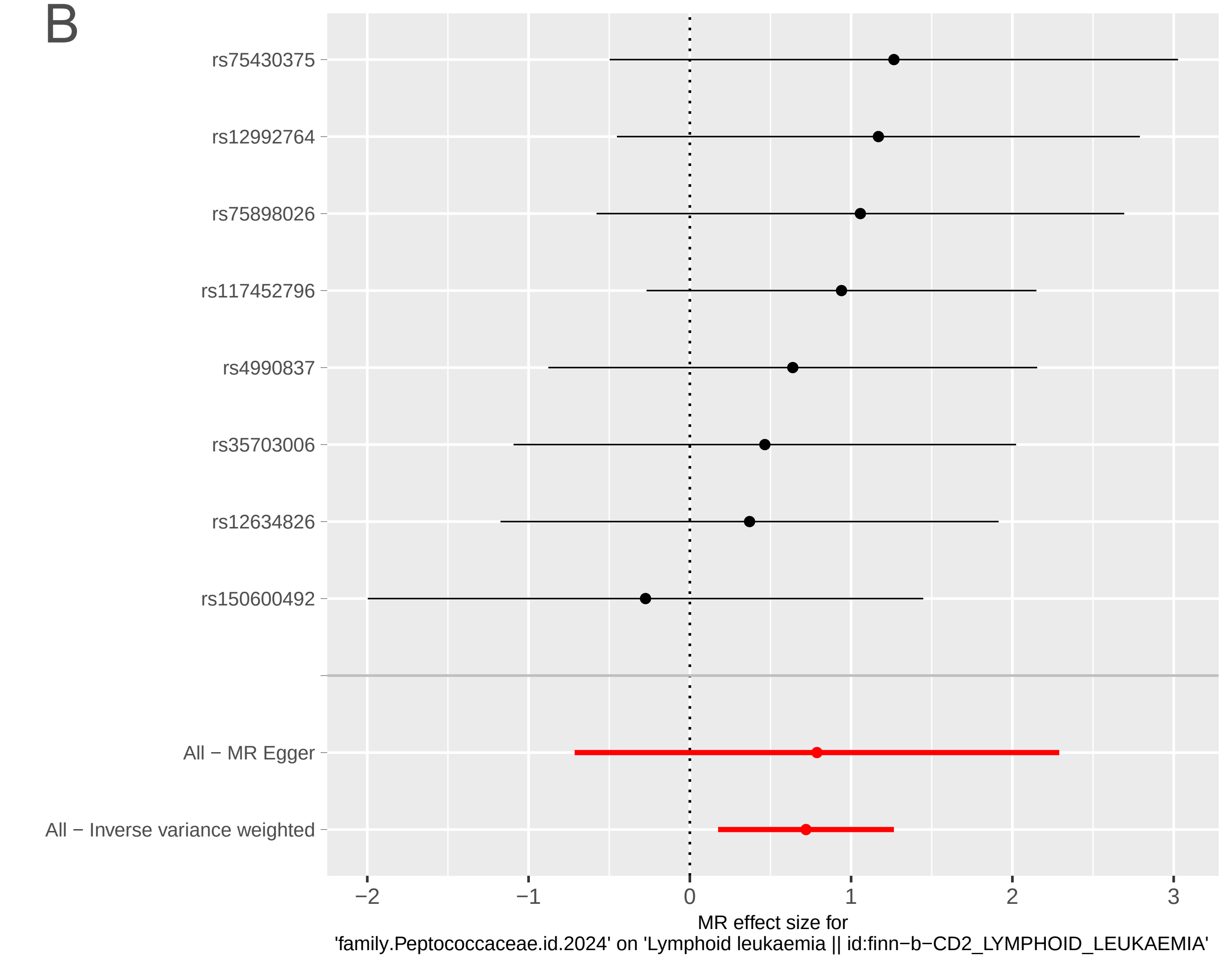
B
rs75430375
rs12992764
rs75898026
rs117452796
rs4990837
rs35703006
rs12634826
rs150600492
All − MR Egger
All − Inverse variance weighted
−2
−1
0
1
2
3
MR effect size for
'family.Peptococcaceae.id.2024' on 'Lymphoid leukaemia || id:finn−b−CD2_LYMPHOID_LEUKAEMIA'

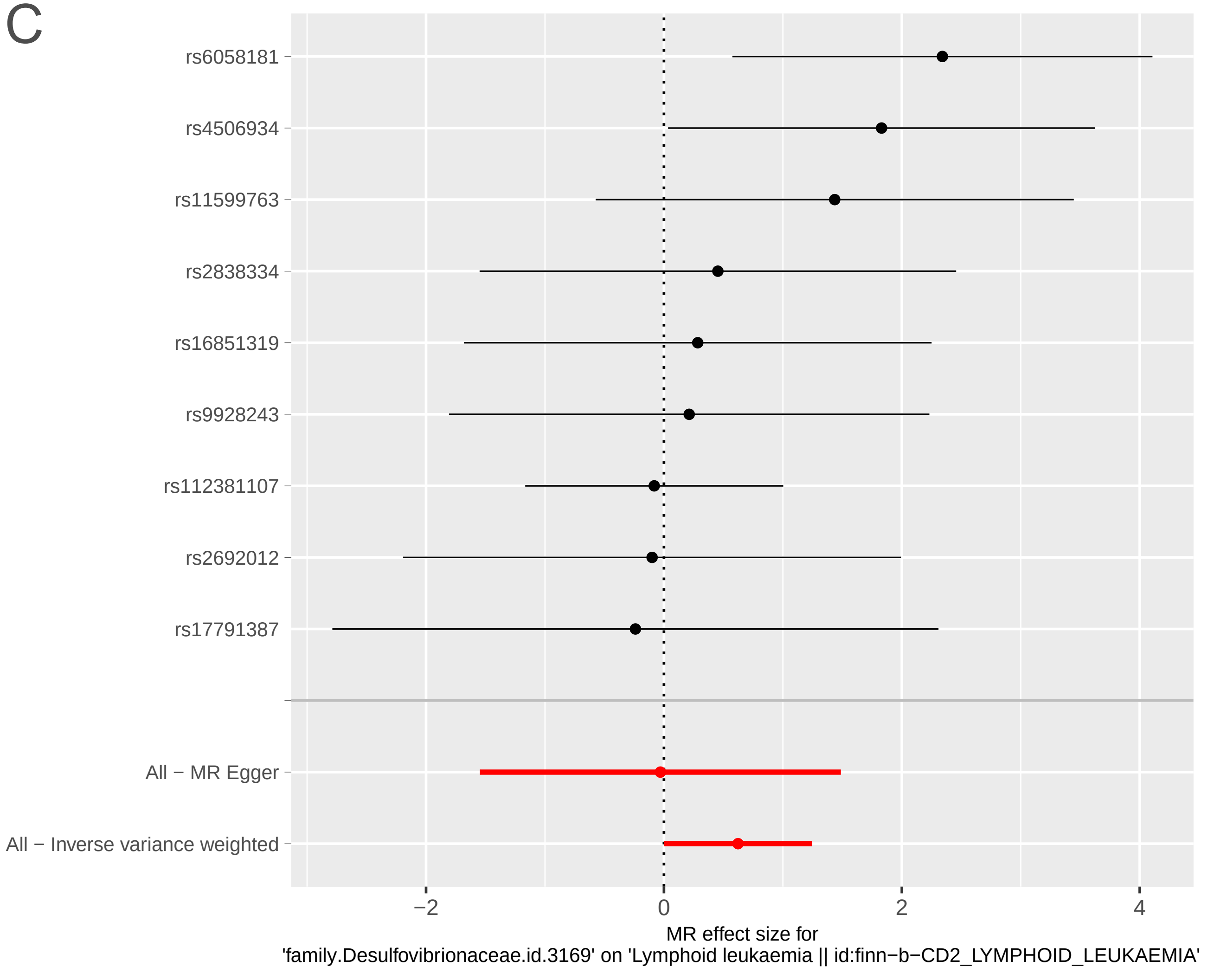
C
rs6058181
rs4506934
rs11599763
rs2838334
rs16851319
rs9928243
rs112381107
rs2692012
rs17791387
All − MR Egger
All − Inverse variance weighted
−2
0
2
4
MR effect size for
'family.Desulfovibrionaceae.id.3169' on 'Lymphoid leukaemia || id:finn−b−CD2_LYMPHOID_LEUKAEMIA'

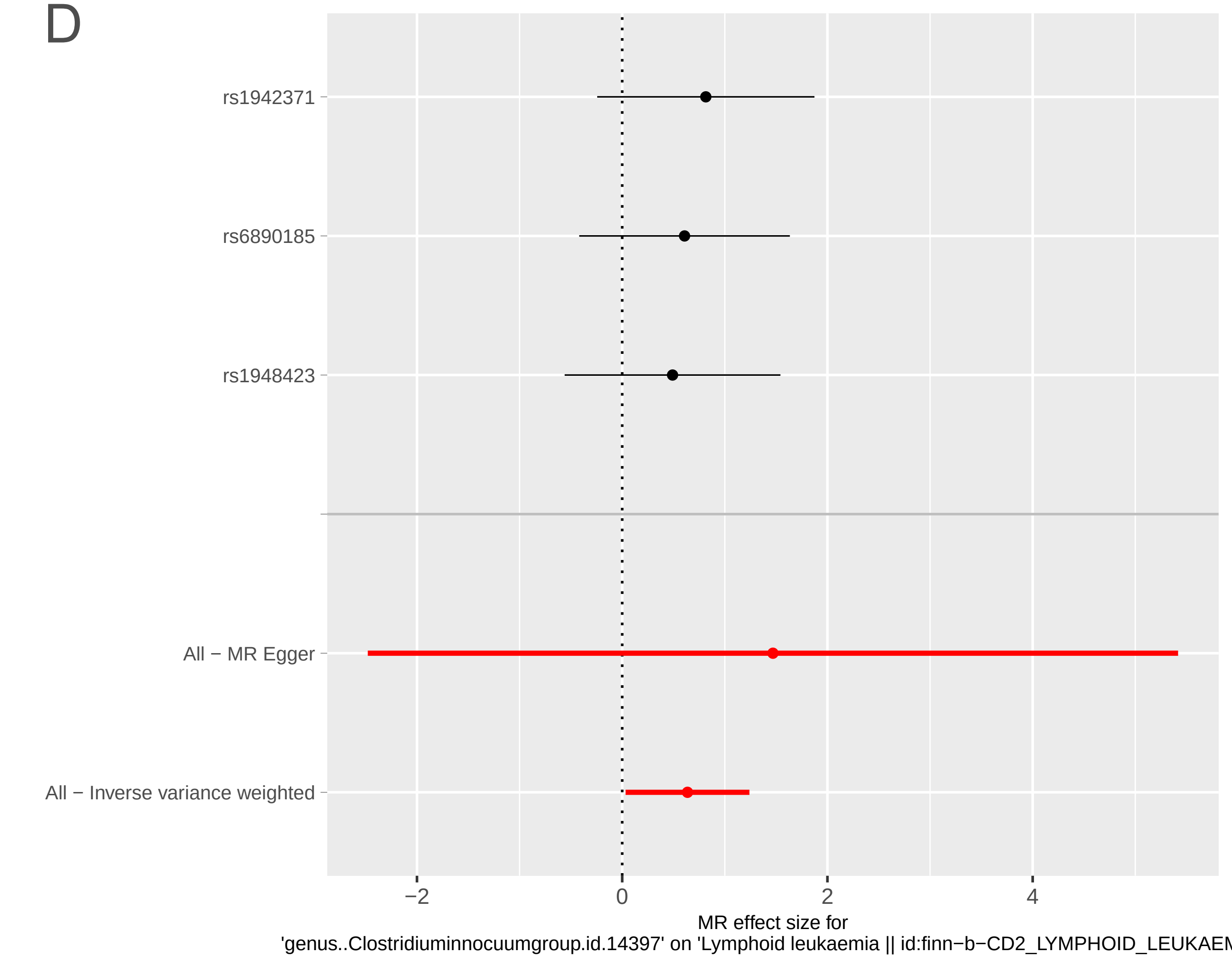
D
rs1942371
rs6890185
rs1948423
All − MR Egger
All − Inverse variance weighted
−2
0
2
4
MR effect size for
'genus..Clostridiuminnocuumgroup.id.14397' on 'Lymphoid leukaemia || id:finn−b−CD2_LYMPHOID_LEUKAEM

E

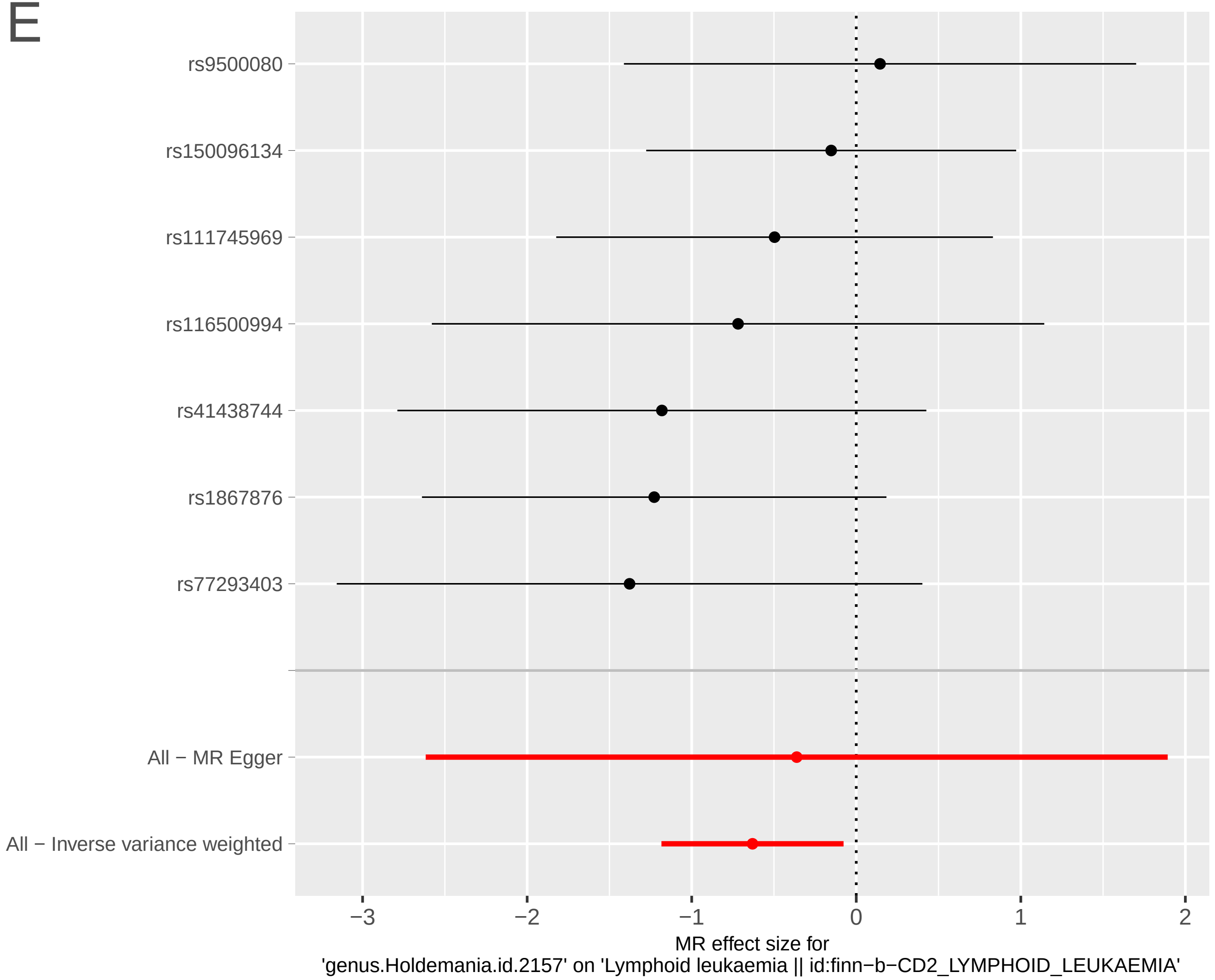

rs9500080
rs150096134
rs111745969
rs116500994
rs41438744
rs1867876
rs77293403
All − MR Egger
All − Inverse variance weighted
−3
−2
−1
0
1
2
MR effect size for
'genus.Holdemania.id.2157' on 'Lymphoid leukaemia || id:finn−b−CD2_LYMPHOID_LEUKAEMIA'

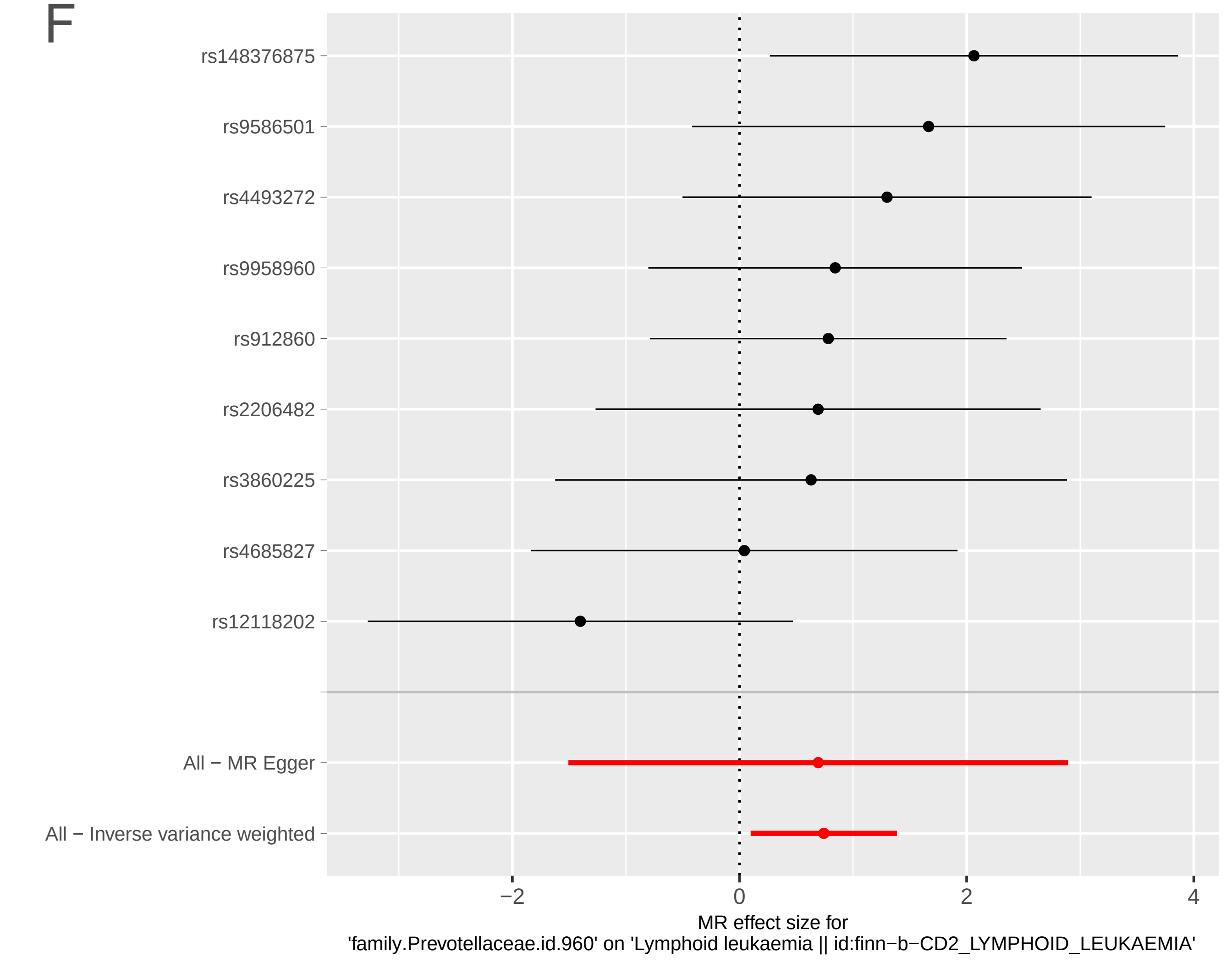
F
rs148376875
rs9586501
rs4493272
rs9958960
rs912860
rs2206482
rs3860225
rs4685827
rs12118202
All − MR Egger
All − Inverse variance weighted
−2
0
2
4
MR effect size for
'family.Prevotellaceae.id.960' on 'Lymphoid leukaemia || id:finn−b−CD2_LYMPHOID_LEUKAEMIA'

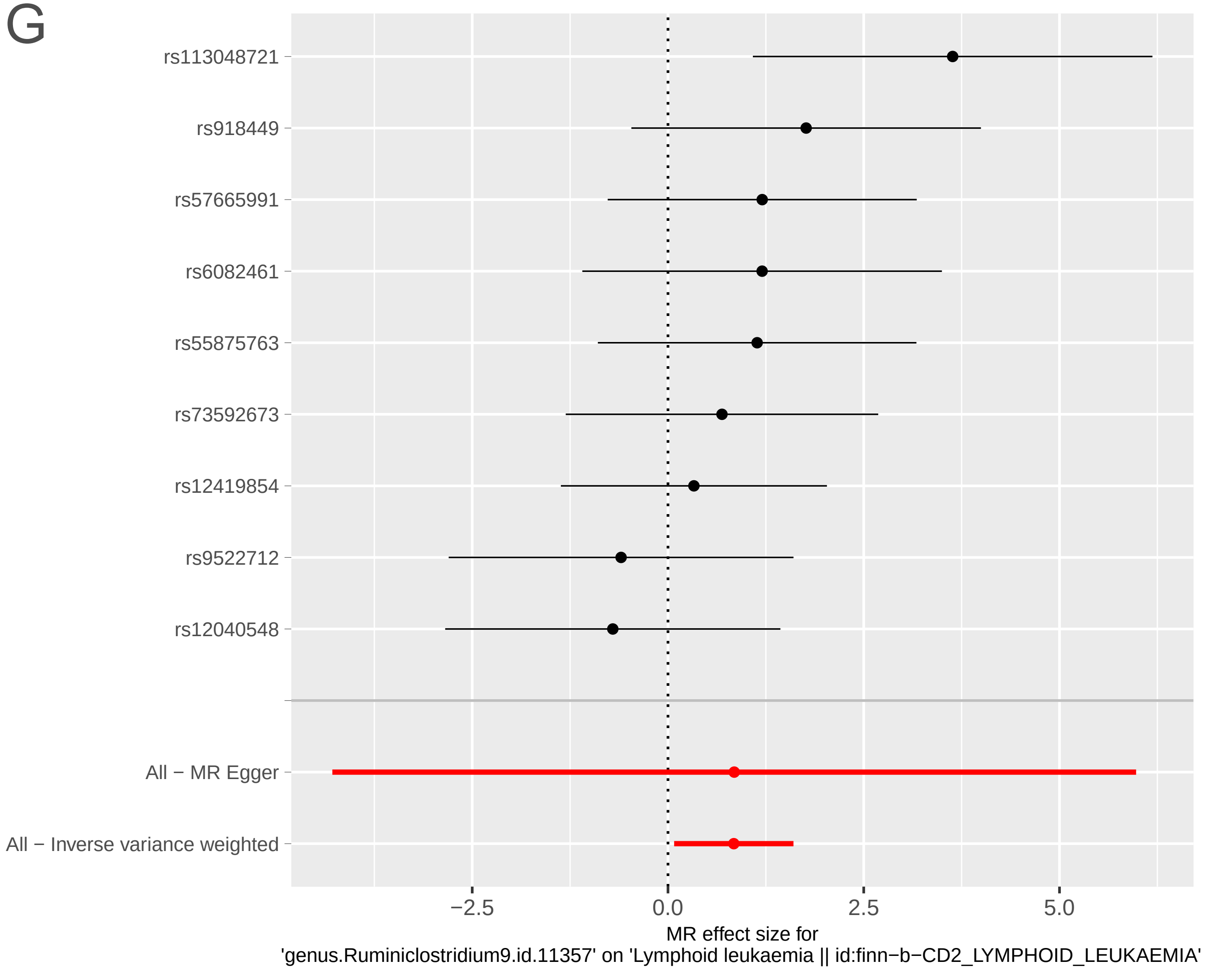
G
rs113048721
rs918449
rs57665991
rs6082461
rs55875763
rs73592673
rs12419854
rs9522712
rs12040548
All − MR Egger
All − Inverse variance weighted
−2.5
0.0
2.5
5.0
MR effect size for
'genus.Ruminiclostridium9.id.11357' on 'Lymphoid leukaemia || id:finn−b−CD2_LYMPHOID_LEUKAEMIA'

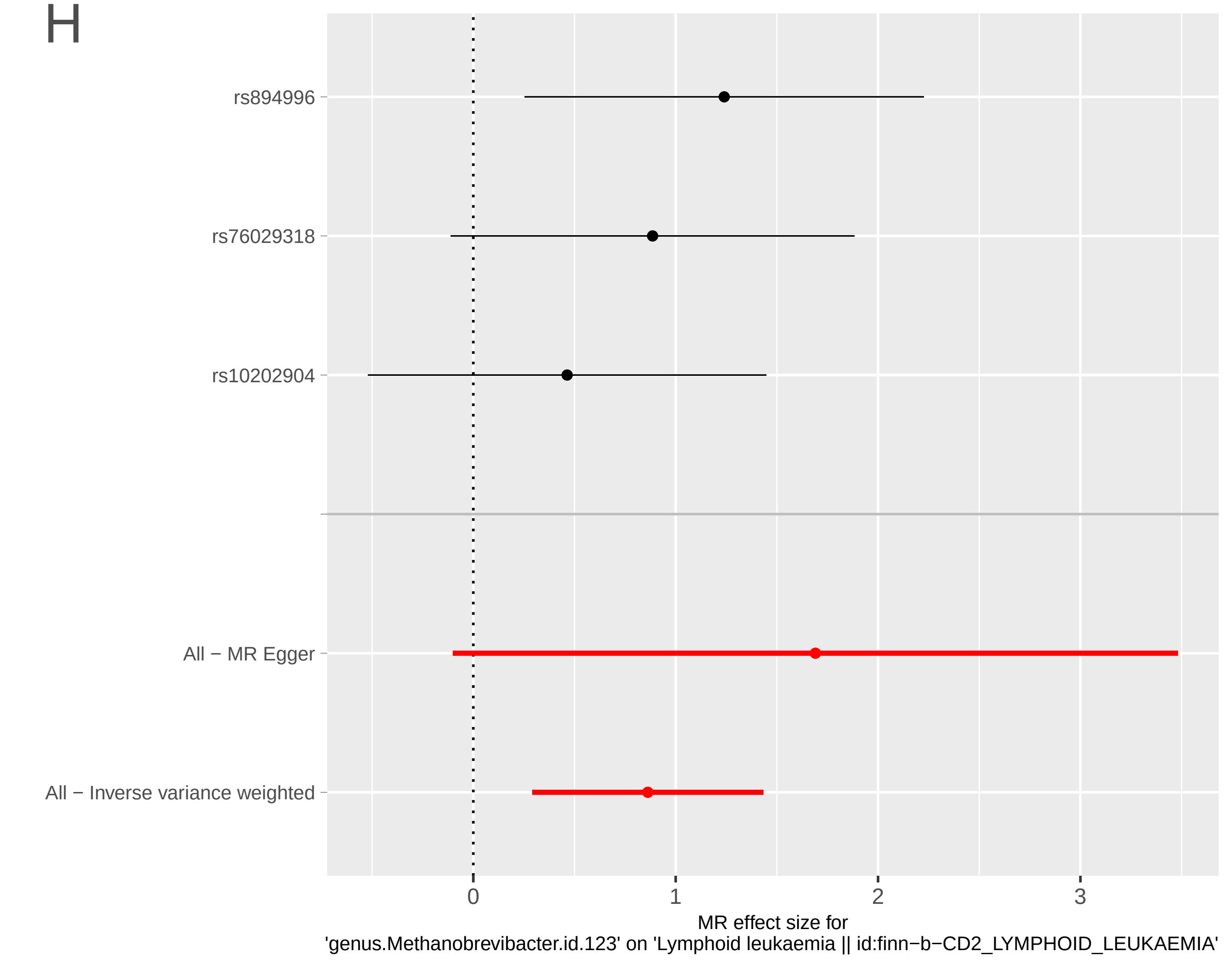

H
rs894996
rs76029318
rs10202904
All − MR Egger
All − Inverse variance weighted
0
1
2
3
MR effect size for
'genus.Methanobrevibacter.id.123' on 'Lymphoid leukaemia || id:finn−b−CD2_LYMPHOID_LEUKAEMIA'

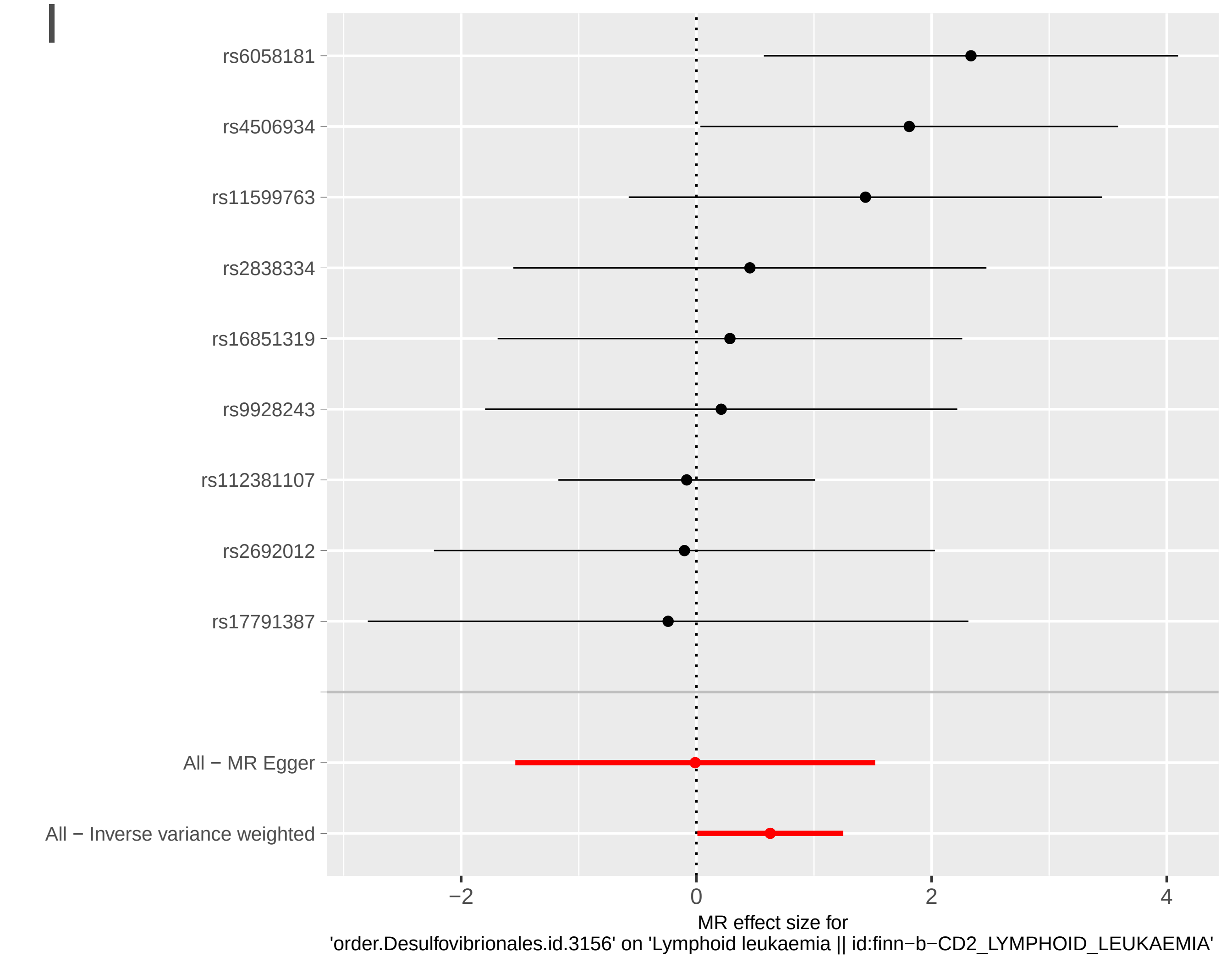
rs6058181
rs4506934
rs11599763
rs2838334
rs16851319
rs9928243
rs112381107
rs2692012
rs17791387
All − MR Egger
All − Inverse variance weighted
−2
0
2
4
MR effect size for
'order.Desulfovibrionales.id.3156' on 'Lymphoid leukaemia || id:finn−b−CD2_LYMPHOID_LEUKAEMIA'

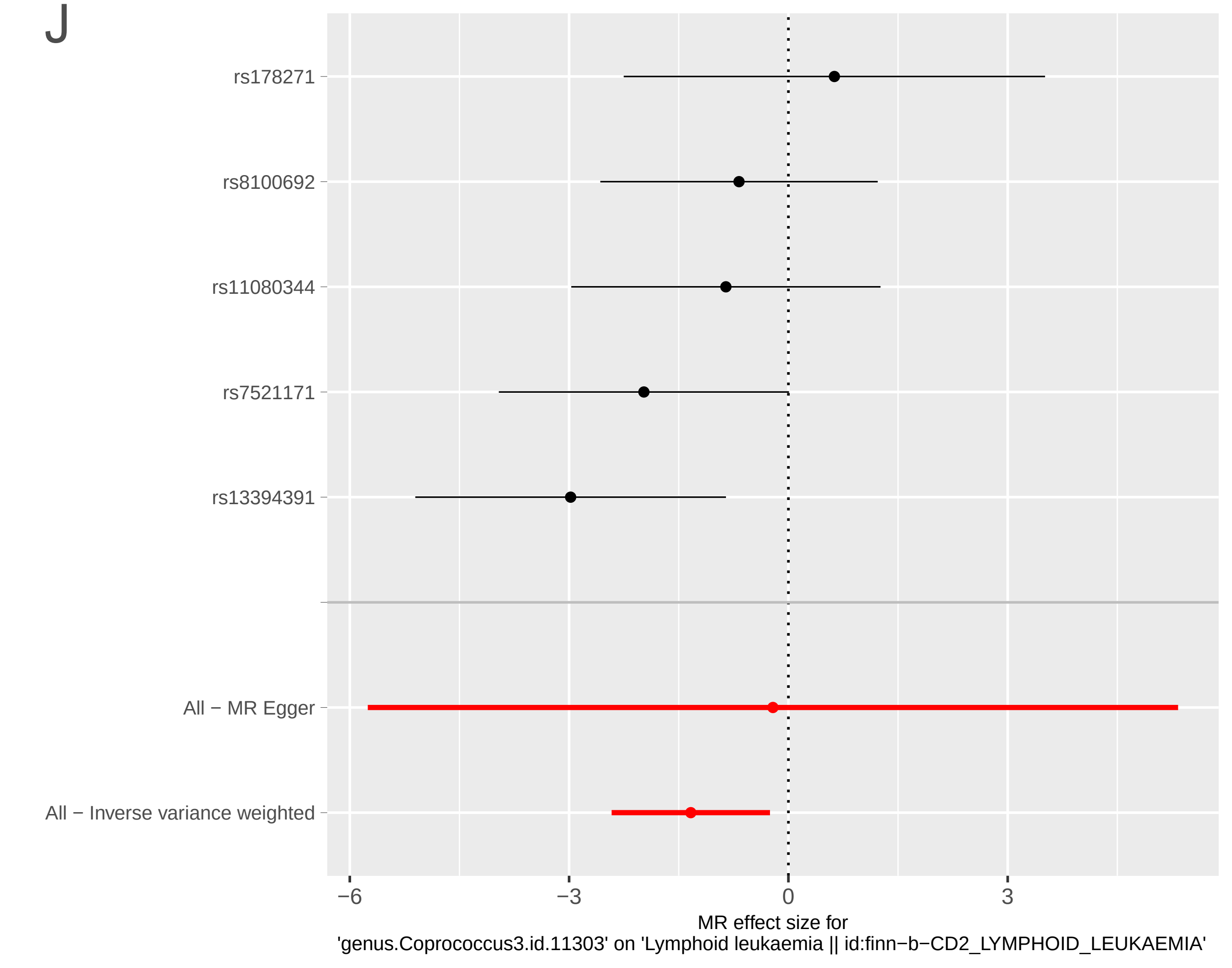
J
rs178271
rs8100692
rs11080344
rs7521171
rs13394391
All − MR Egger
All − Inverse variance weighted
−6
−3
0
3
MR effect size for
'genus.Coprococcus3.id.11303' on 'Lymphoid leukaemia || id:finn−b−CD2_LYMPHOID_LEUKAEMIA'

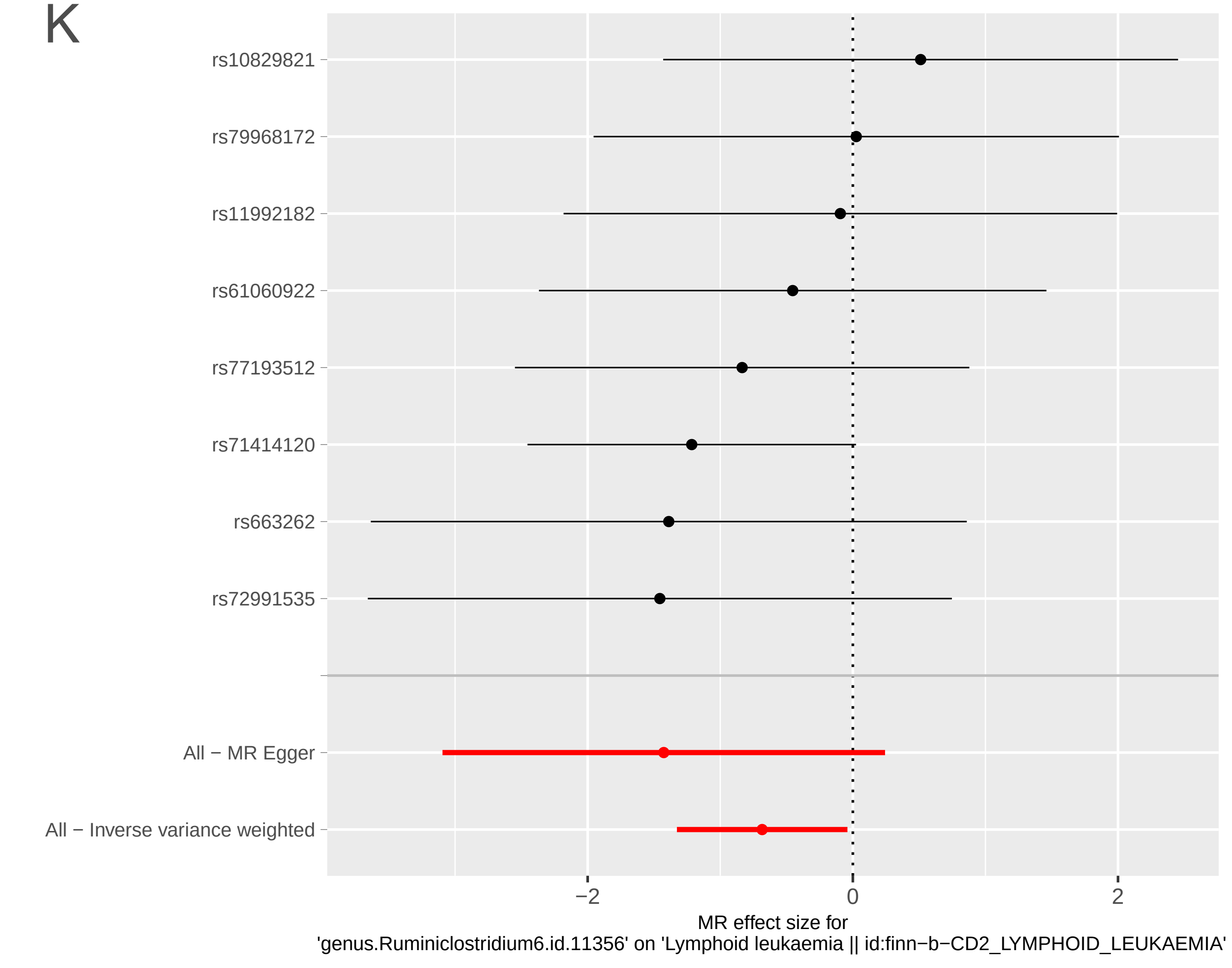
K
rs10829821
rs79968172
rs11992182
rs61060922
rs77193512
rs71414120
rs663262
rs72991535
All − MR Egger
All − Inverse variance weighted
−2
0
2
MR effect size for
'genus.Ruminiclostridium6.id.11356' on 'Lymphoid leukaemia || id:finn−b−CD2_LYMPHOID_LEUKAEMIA'

L

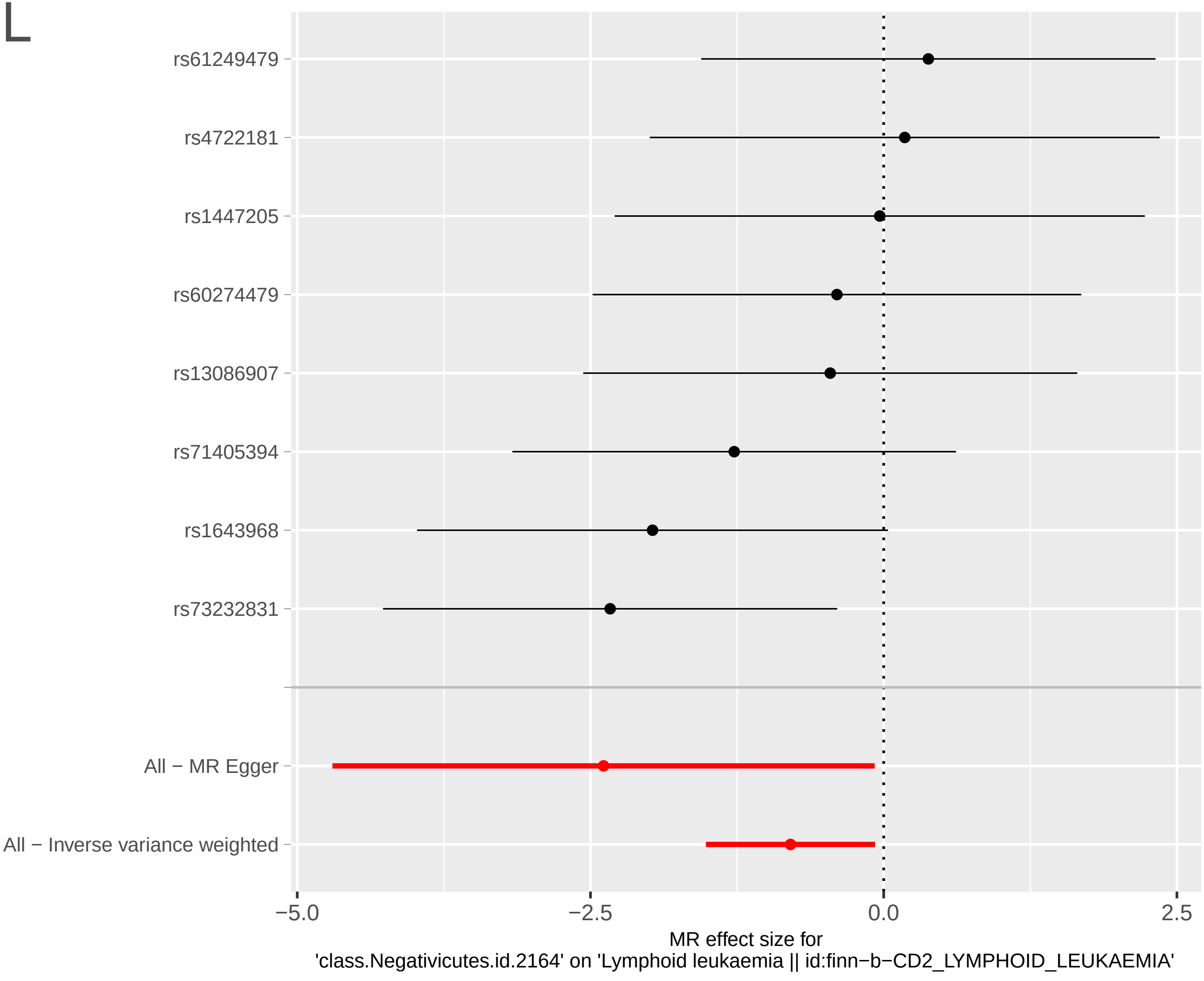
rs61249479
rs4722181
rs1447205
rs60274479
rs13086907
rs71405394
rs1643968
rs73232831
All − MR Egger
All − Inverse variance weighted
−5.0
−2.5
0.0
2.5
MR effect size for
'class.Negativicutes.id.2164' on 'Lymphoid leukaemia || id:finn−b−CD2_LYMPHOID_LEUKAEMIA'

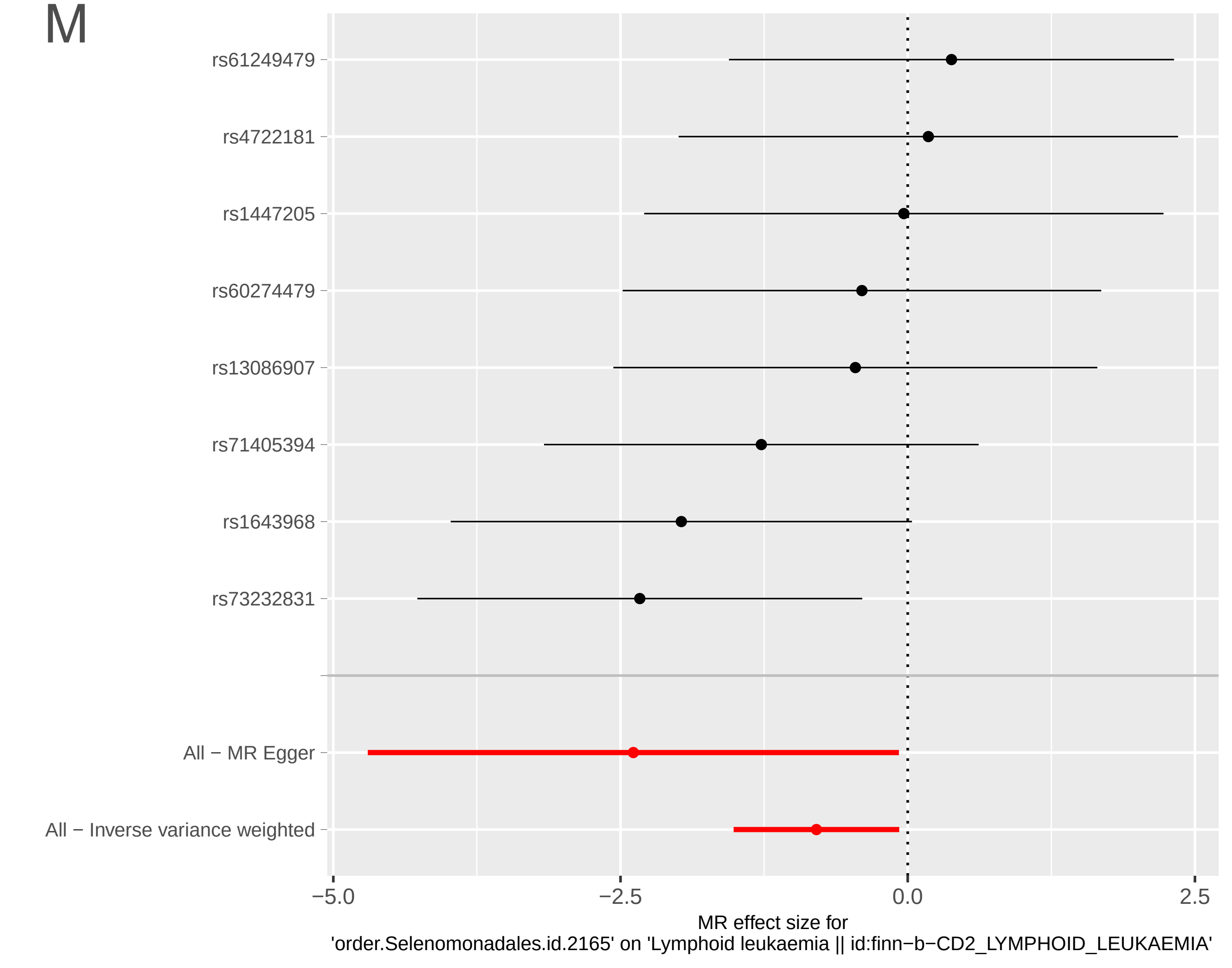
M
rs61249479
rs4722181
rs1447205
rs60274479
rs13086907
rs71405394
rs1643968
rs73232831
All − MR Egger
All − Inverse variance weighted
−5.0
−2.5
0.0
2.5
MR effect size for
'order.Selenomonadales.id.2165' on 'Lymphoid leukaemia || id:finn−b−CD2_LYMPHOID_LEUKAEMIA'

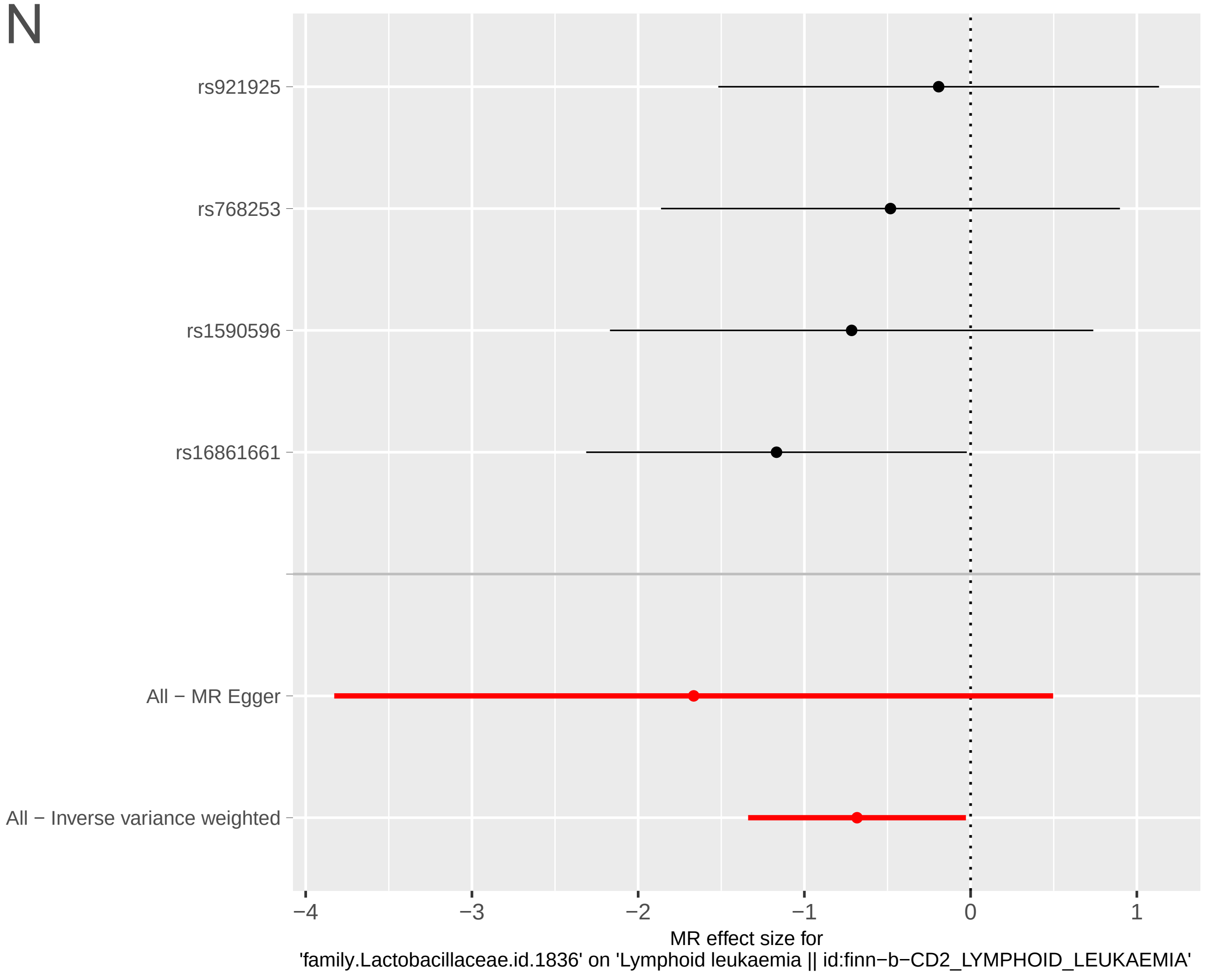
N
rs921925
rs768253
rs1590596
rs16861661
All − MR Egger
All − Inverse variance weighted
−4
−3
−2
−1
0
1
MR effect size for
'family.Lactobacillaceae.id.1836' on 'Lymphoid leukaemia || id:finn−b−CD2_LYMPHOID_LEUKAEMIA'

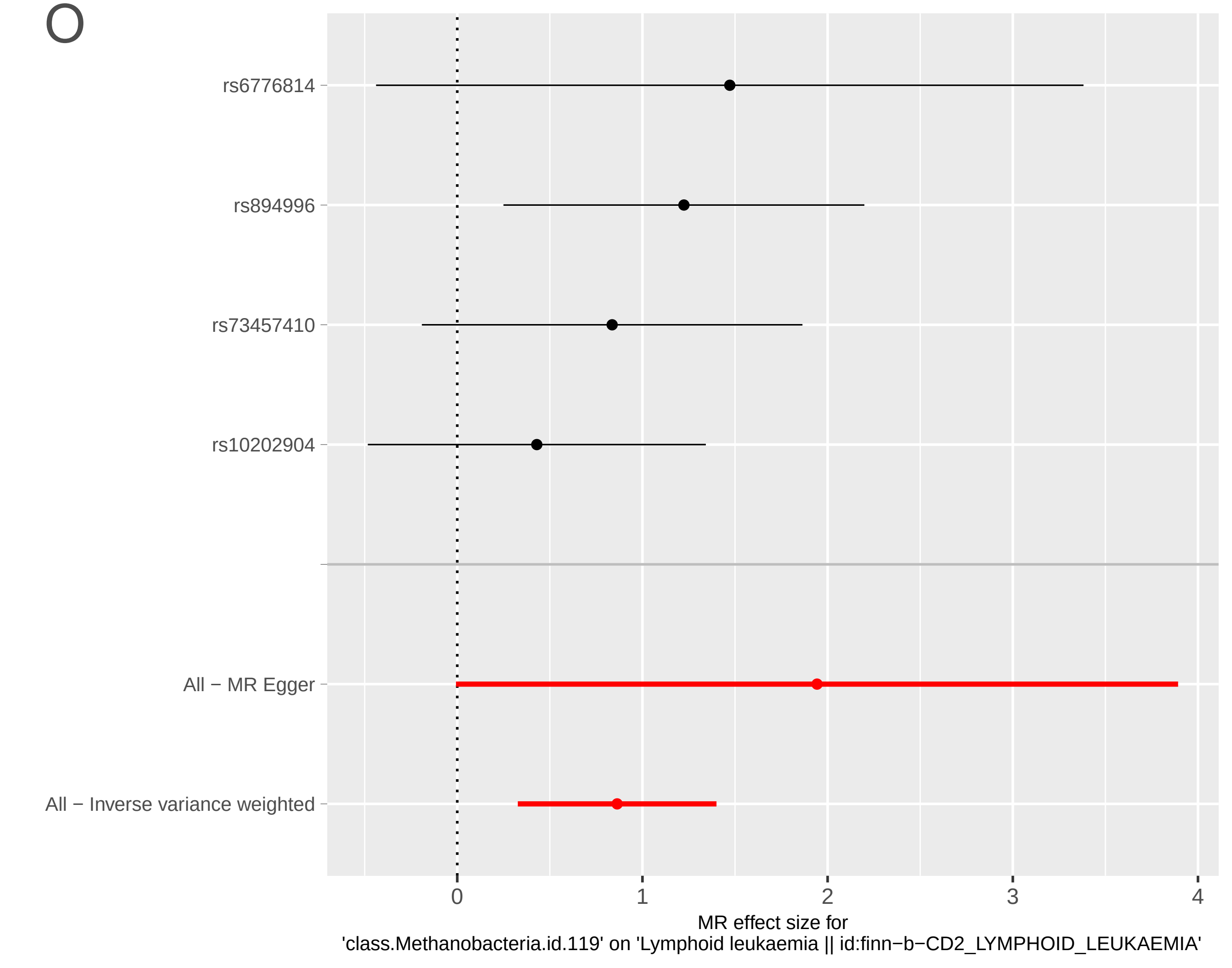
O
rs6776814
rs894996
rs73457410
rs10202904
All − MR Egger
All − Inverse variance weighted
0
1
2
3
4
MR effect size for
'class.Methanobacteria.id.119' on 'Lymphoid leukaemia || id:finn−b−CD2_LYMPHOID_LEUKAEMIA'

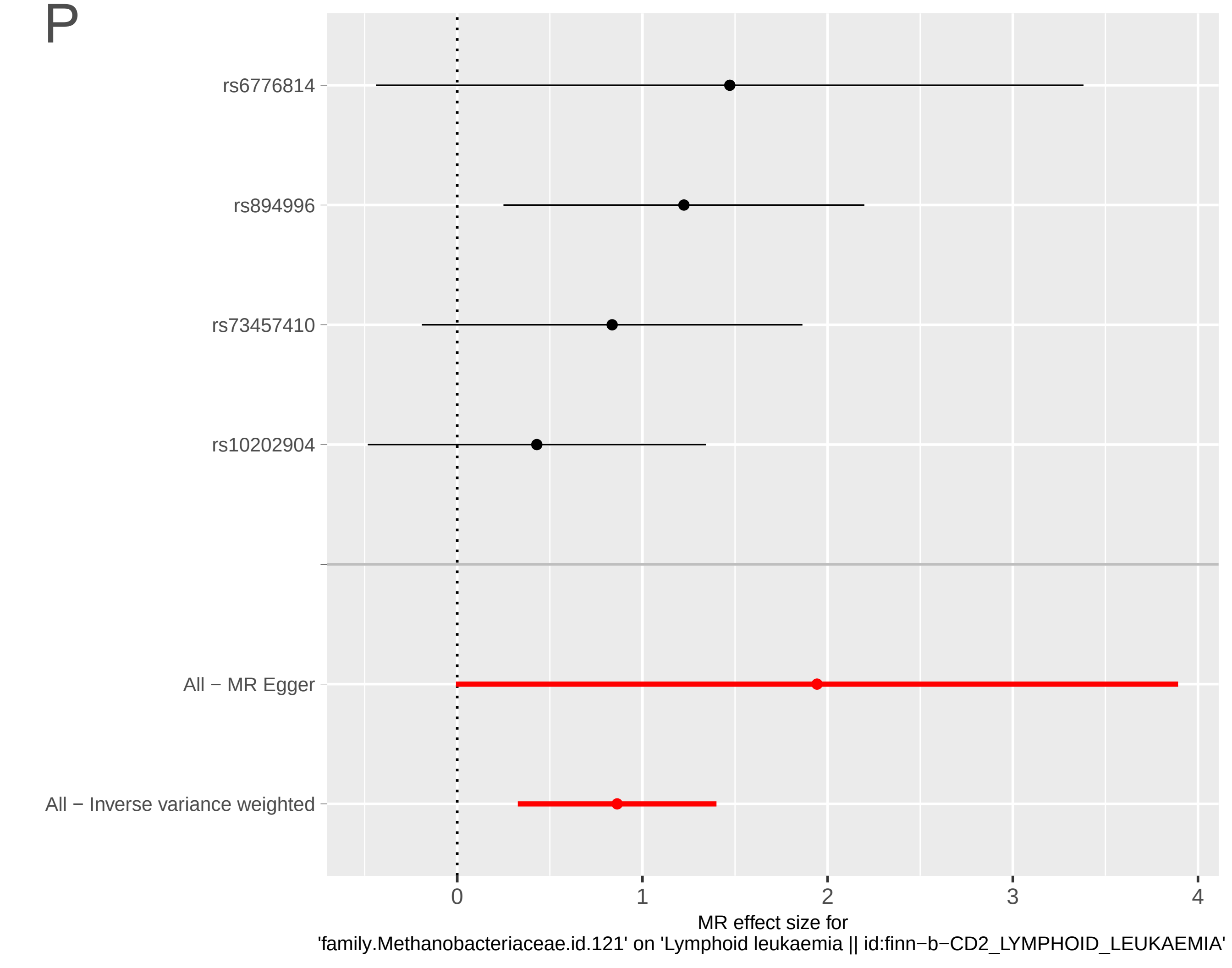
P
rs6776814
rs894996
rs73457410
rs10202904
All − MR Egger
All − Inverse variance weighted
0
1
2
3
4
MR effect size for
'family.Methanobacteriaceae.id.121' on 'Lymphoid leukaemia || id:finn−b−CD2_LYMPHOID_LEUKAEMIA'

Q

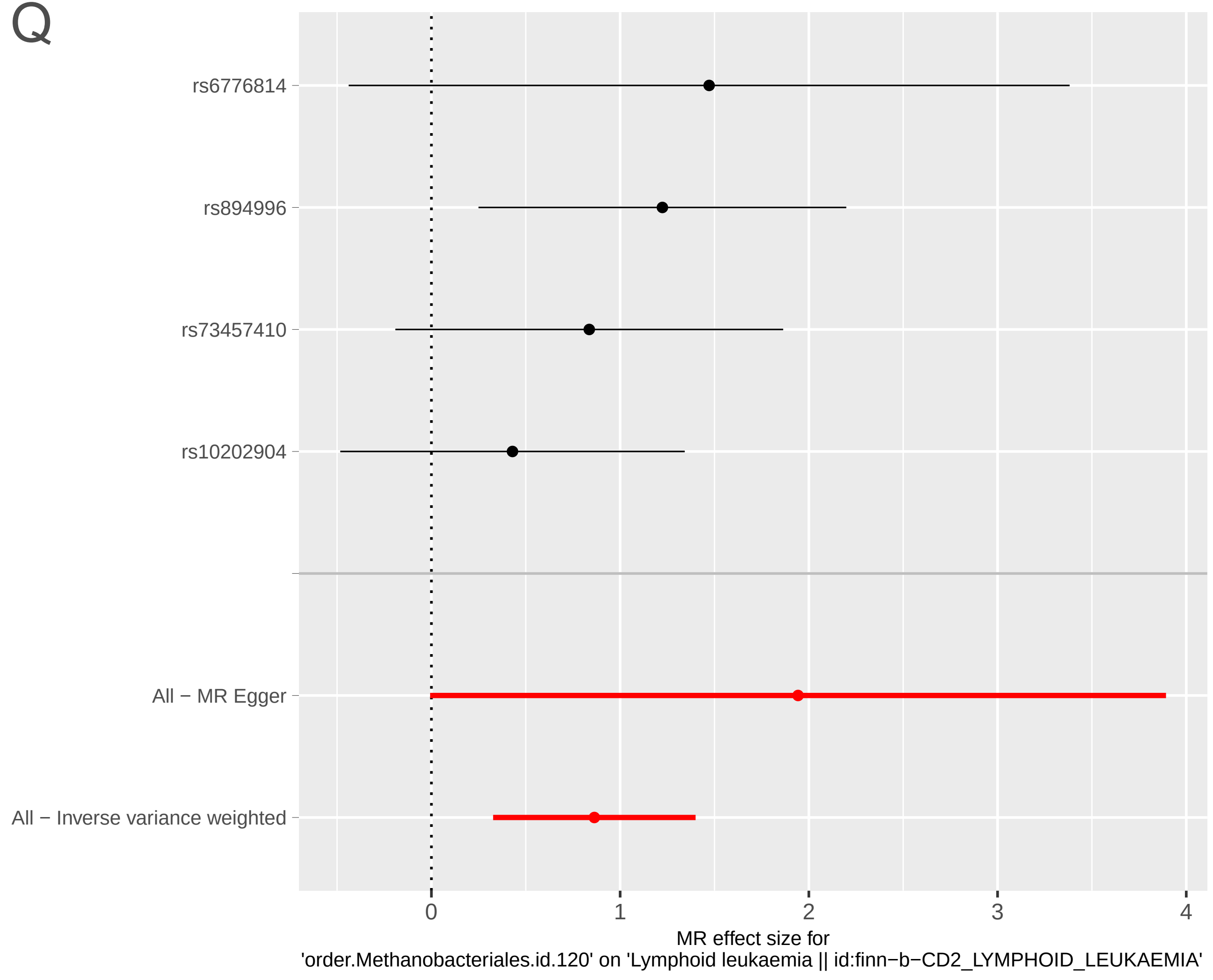

rs6776814
rs894996
rs73457410
rs10202904
All − MR Egger
All − Inverse variance weighted
0
1
2
3
4
MR effect size for
'order.Methanobacteriales.id.120' on 'Lymphoid leukaemia || id:finn−b−CD2_LYMPHOID_LEUKAEMIA'

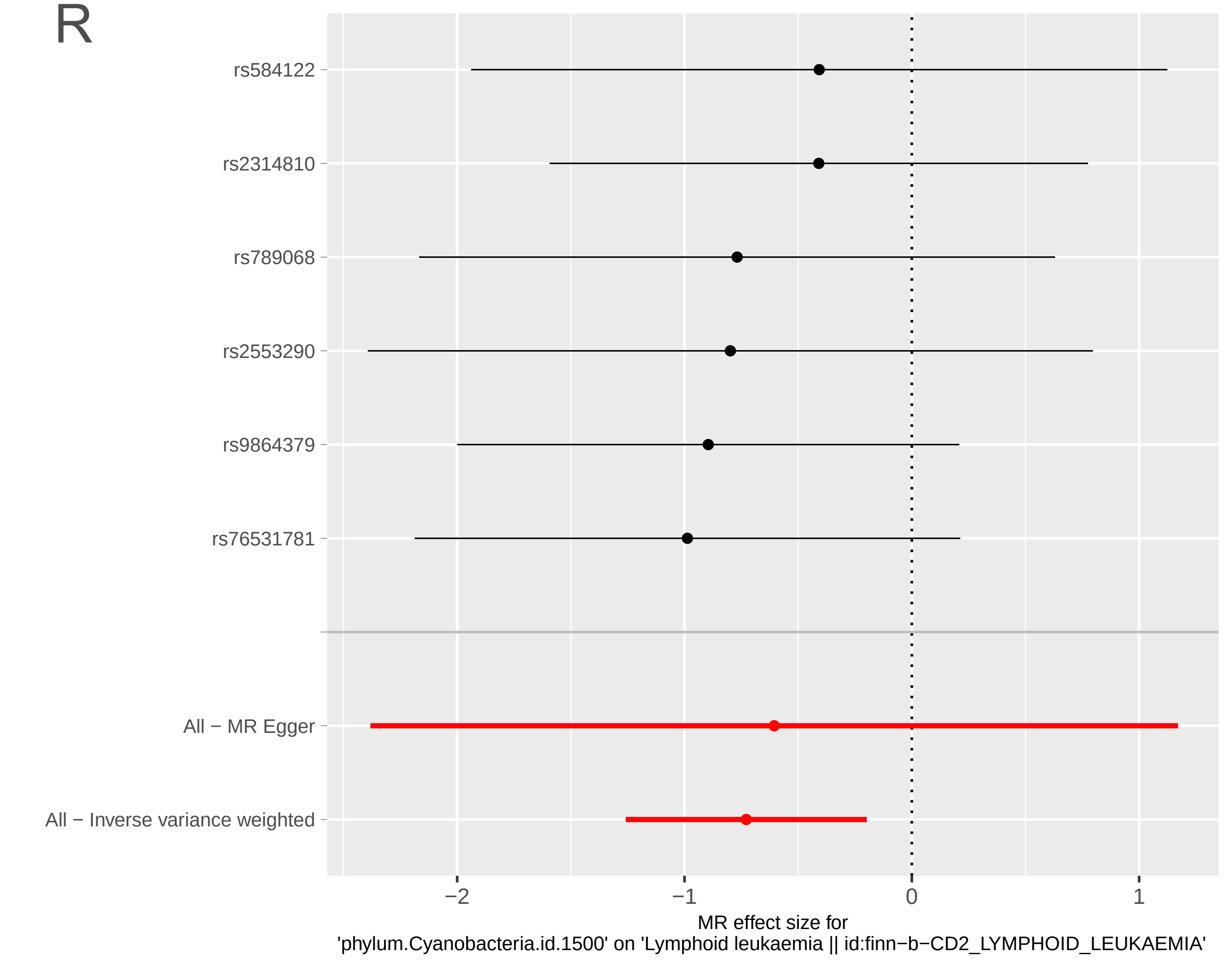
R
rs584122
rs2314810
rs789068
rs2553290
rs9864379
rs76531781
All − MR Egger
All − Inverse variance weighted
−2
−1
0
1
MR effect size for
'phylum.Cyanobacteria.id.1500' on 'Lymphoid leukaemia || id:finn−b−CD2_LYMPHOID_LEUKAEMIA'

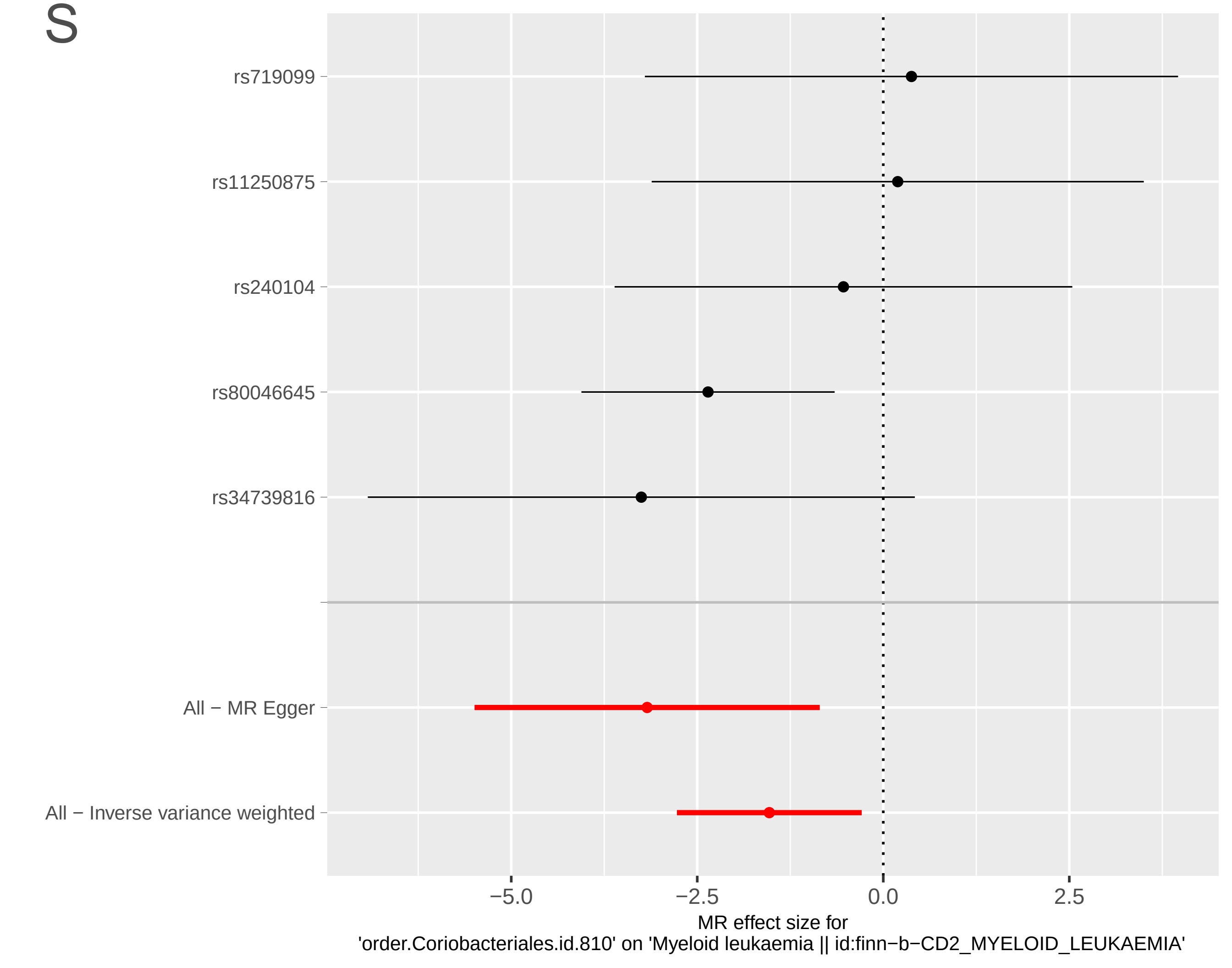
S
rs719099
rs11250875
rs240104
rs80046645
rs34739816
All − MR Egger
All − Inverse variance weighted
−5.0
−2.5
0.0
2.5
MR effect size for
'order.Coriobacteriales.id.810' on 'Myeloid leukaemia || id:finn−b−CD2_MYELOID_LEUKAEMIA'

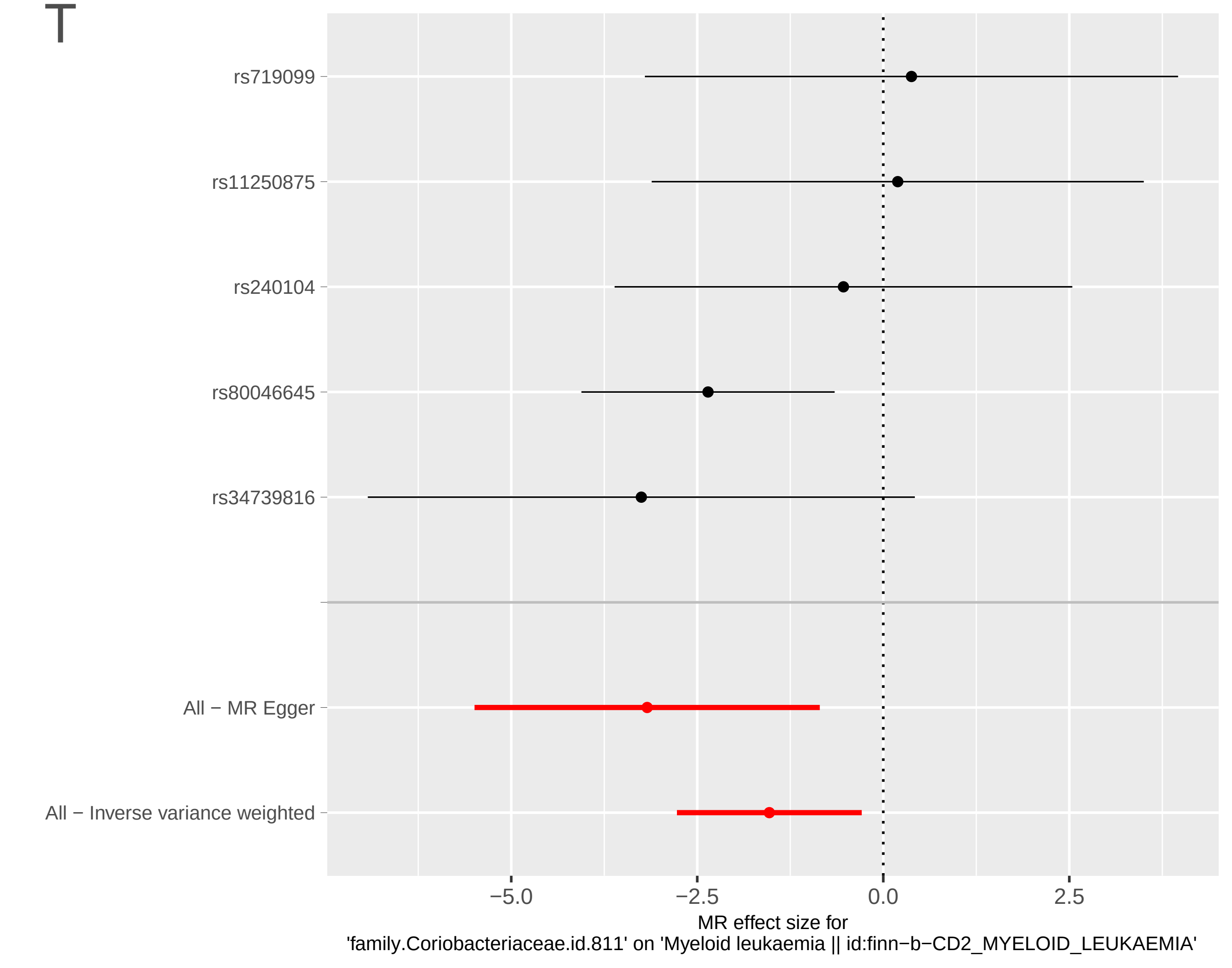
T
rs719099
rs11250875
rs240104
rs80046645
rs34739816
All − MR Egger
All − Inverse variance weighted
−5.0
−2.5
0.0
2.5
MR effect size for
'family.Coriobacteriaceae.id.811' on 'Myeloid leukaemia || id:finn−b−CD2_MYELOID_LEUKAEMIA'

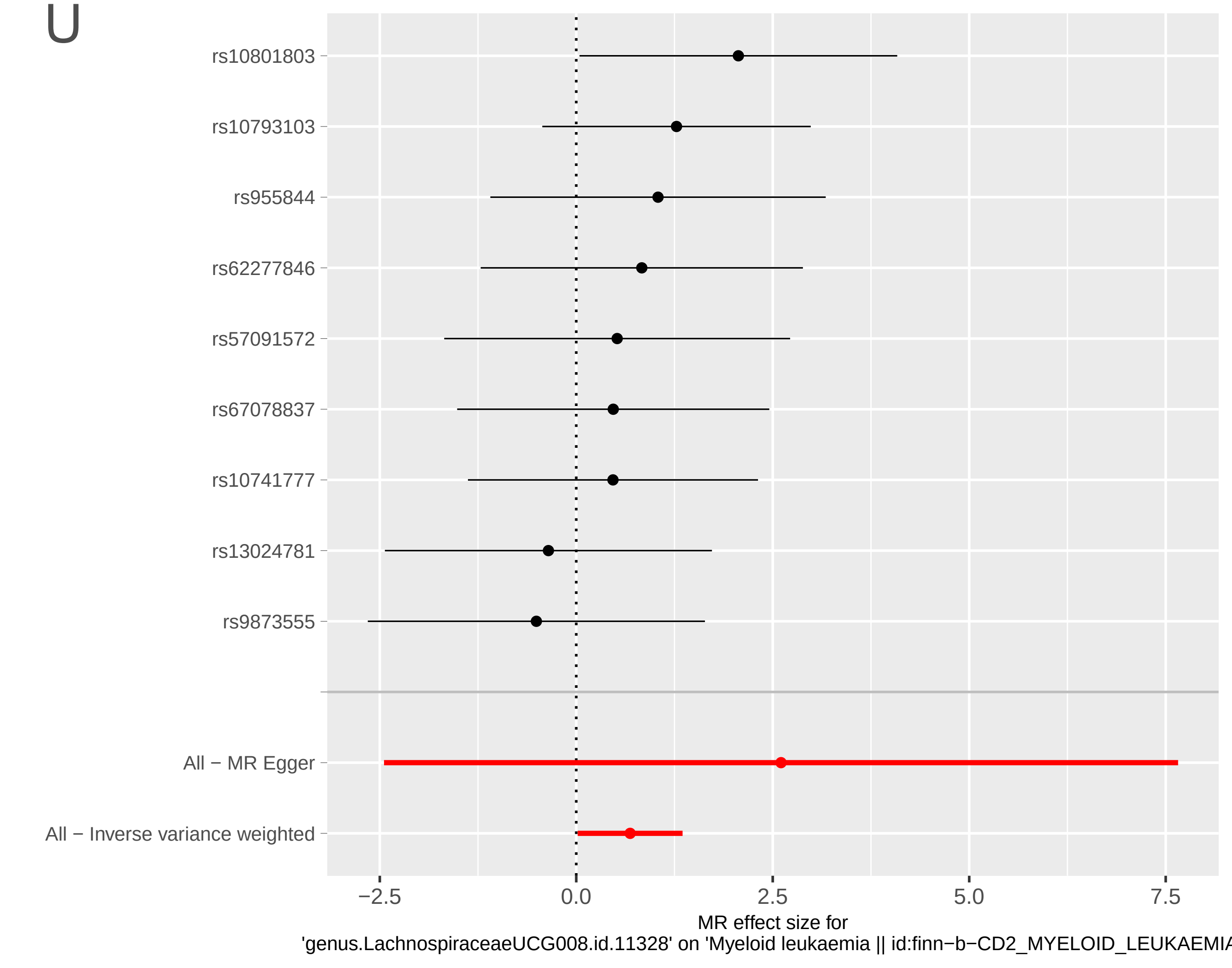

U
rs10801803
rs10793103
rs955844
rs62277846
rs57091572
rs67078837
rs10741777
rs13024781
rs9873555
All − MR Egger
All − Inverse variance weighted
−2.5
0.0
2.5
5.0
7.5
MR effect size for
'genus.LachnospiraceaeUCG008.id.11328' on 'Myeloid leukaemia || id:finn−b−CD2_MYELOID_LEUKAEMIA

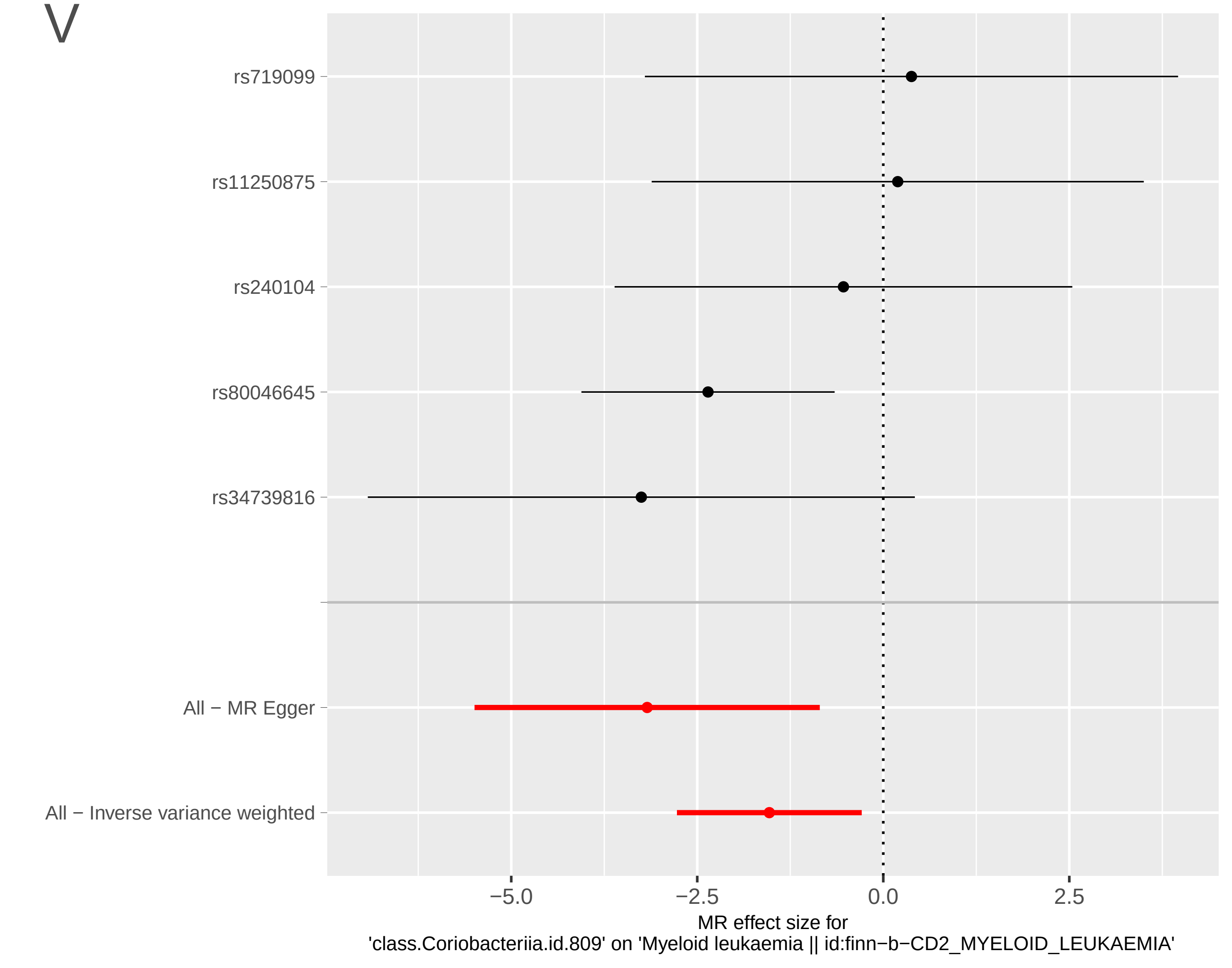
V
rs719099
rs11250875
rs240104
rs80046645
rs34739816
All − MR Egger
All − Inverse variance weighted
−5.0
−2.5
0.0
2.5
MR effect size for
'class.Coriobacteriia.id.809' on 'Myeloid leukaemia || id:finn−b−CD2_MYELOID_LEUKAEMIA'

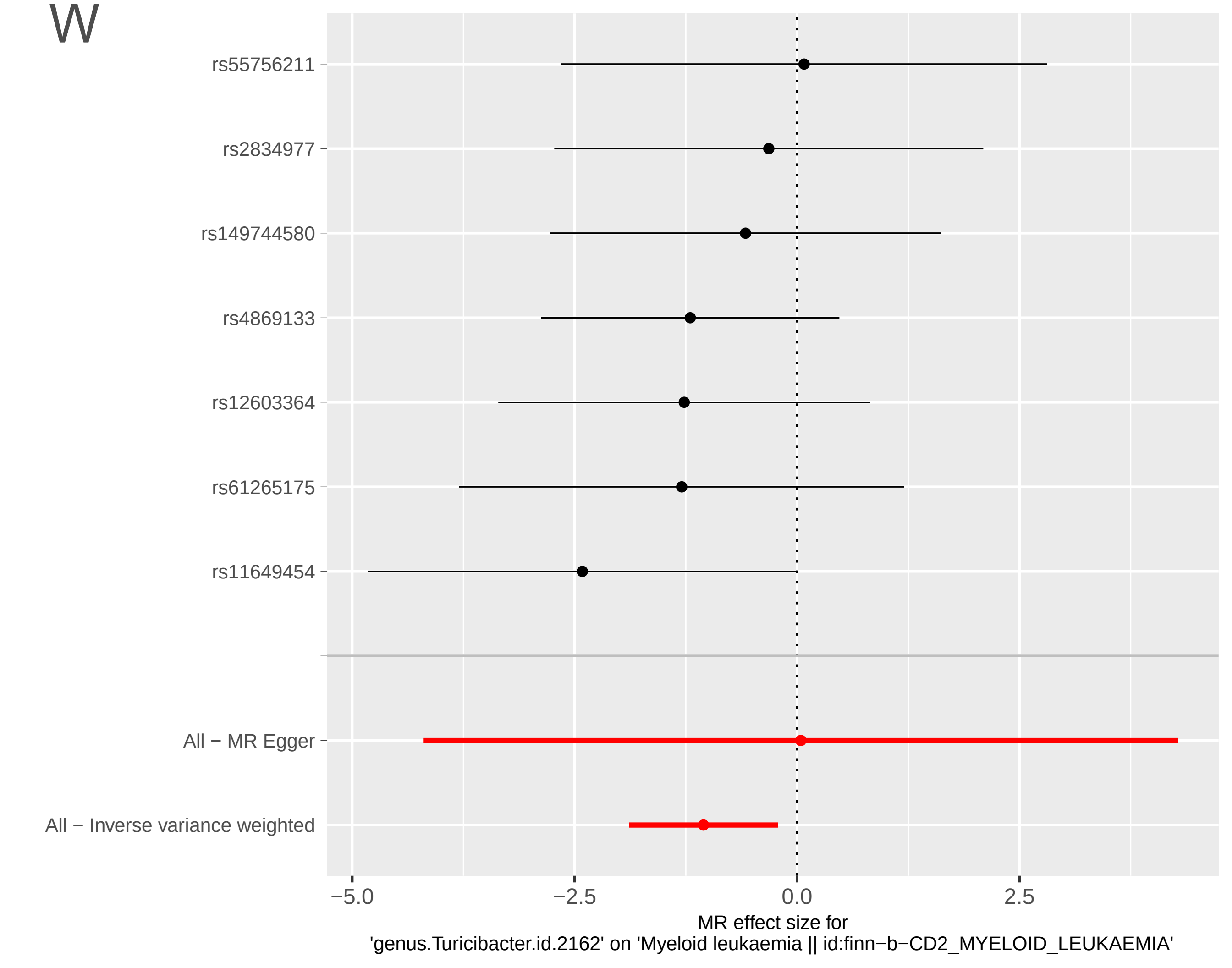
W
rs55756211
rs2834977
rs149744580
rs4869133
rs12603364
rs61265175
rs11649454
All − MR Egger
All − Inverse variance weighted
−5.0
−2.5
0.0
2.5
MR effect size for
'genus.Turicibacter.id.2162' on 'Myeloid leukaemia || id:finn−b−CD2_MYELOID_LEUKAEMIA'

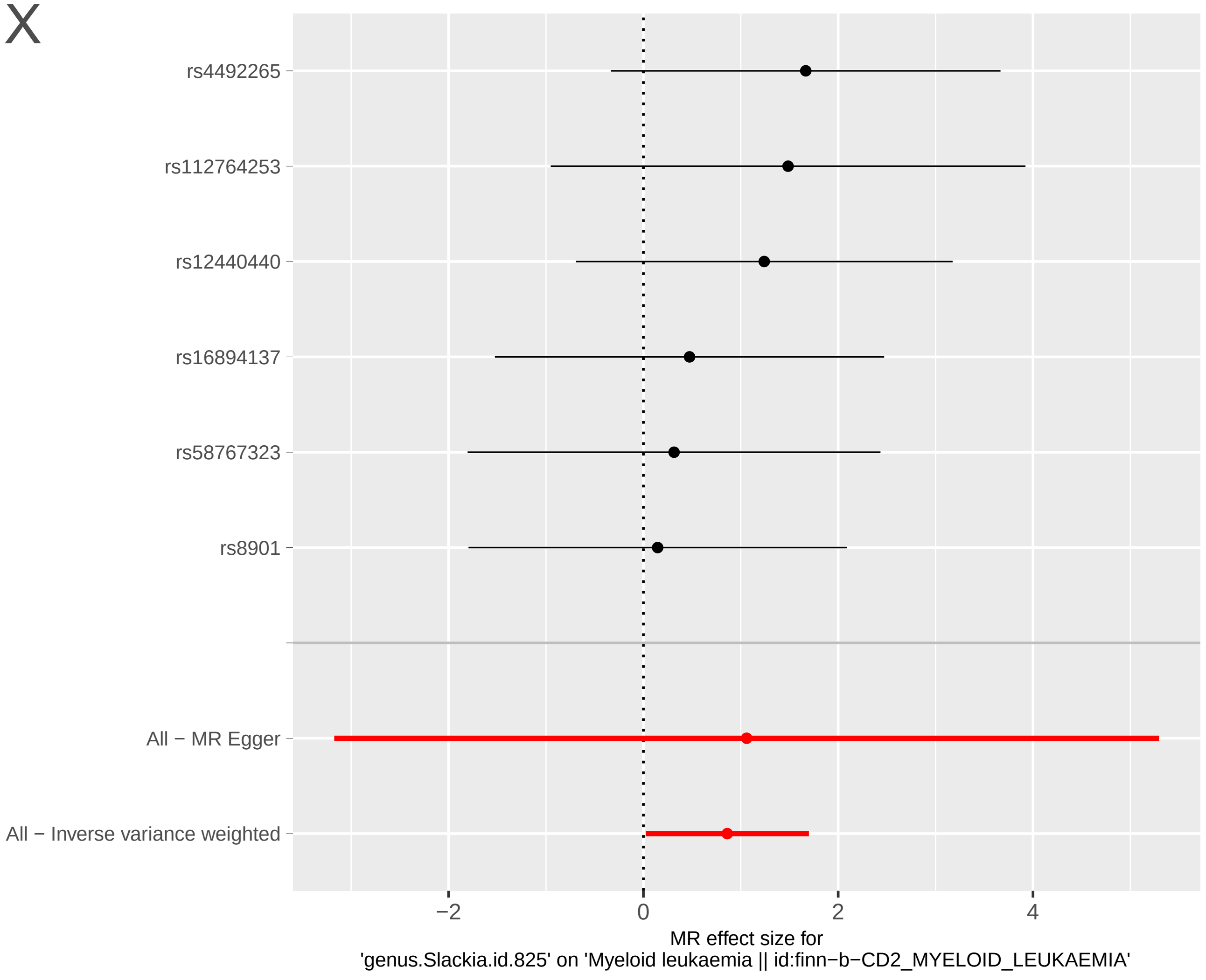
X
rs4492265
rs112764253
rs12440440
rs16894137
rs58767323
rs8901
All − MR Egger
All − Inverse variance weighted
−2
0
2
4
MR effect size for
'genus.Slackia.id.825' on 'Myeloid leukaemia || id:finn−b−CD2_MYELOID_LEUKAEMIA'

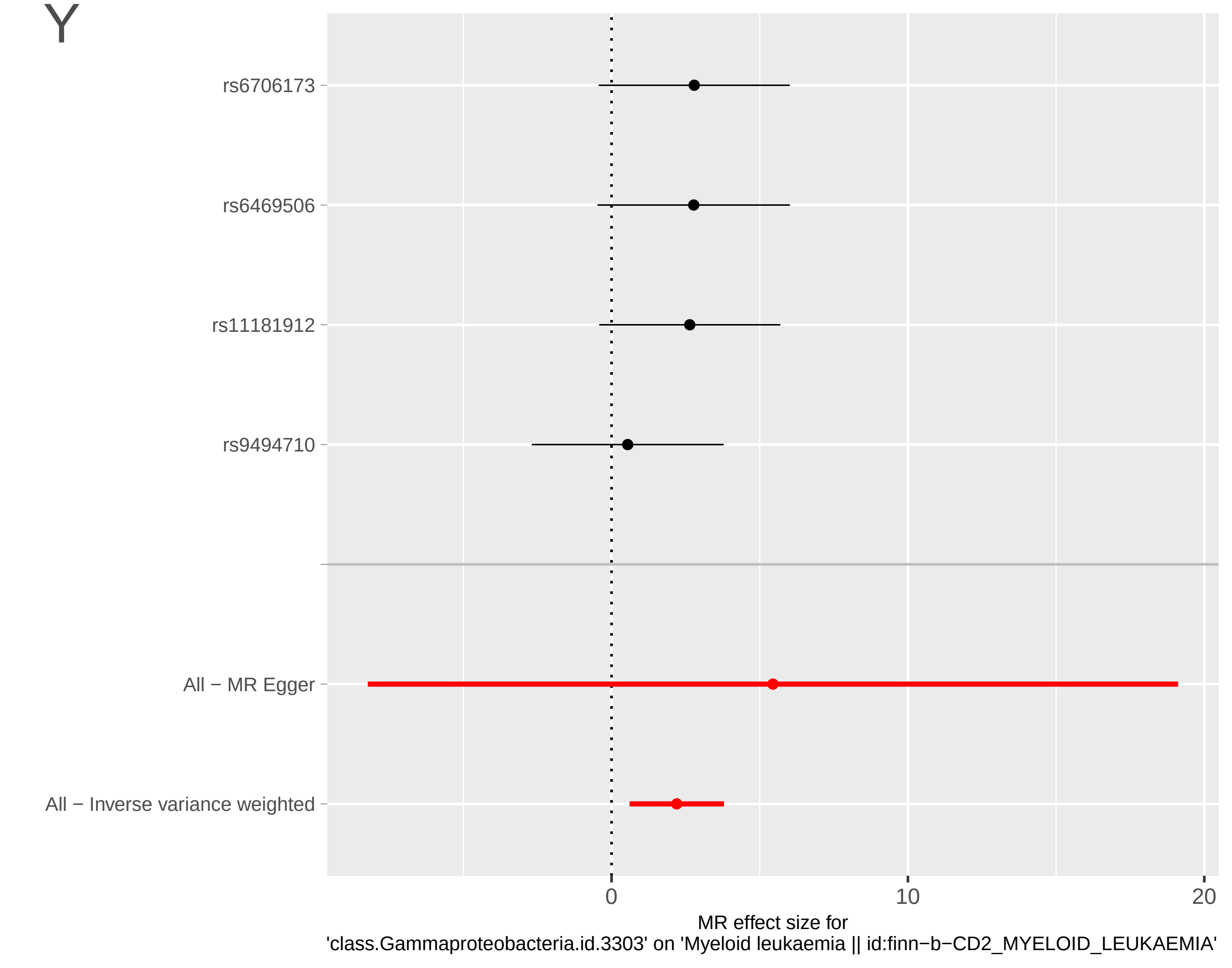
Y
rs6706173
rs6469506
rs11181912
rs9494710
All − MR Egger
All − Inverse variance weighted
0
10
20
MR effect size for
'class.Gammaproteobacteria.id.3303' on 'Myeloid leukaemia || id:finn−b−CD2_MYELOID_LEUKAEMIA'

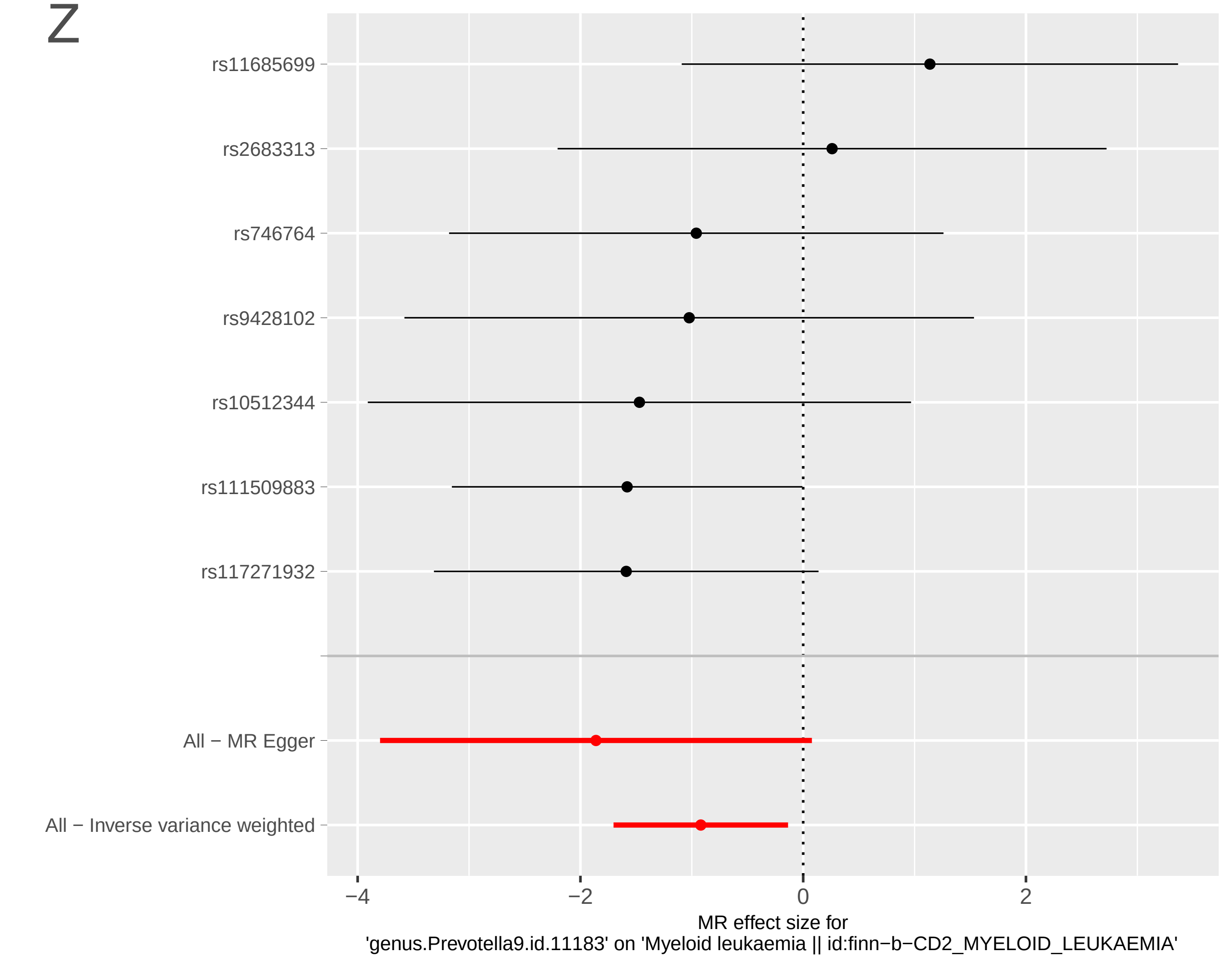
Z
rs11685699
rs2683313
rs746764
rs9428102
rs10512344
rs111509883
rs117271932
All − MR Egger
All − Inverse variance weighted
−4
−2
0
2
MR effect size for
'genus.Prevotella9.id.11183' on 'Myeloid leukaemia || id:finn−b−CD2_MYELOID_LEUKAEMIA'

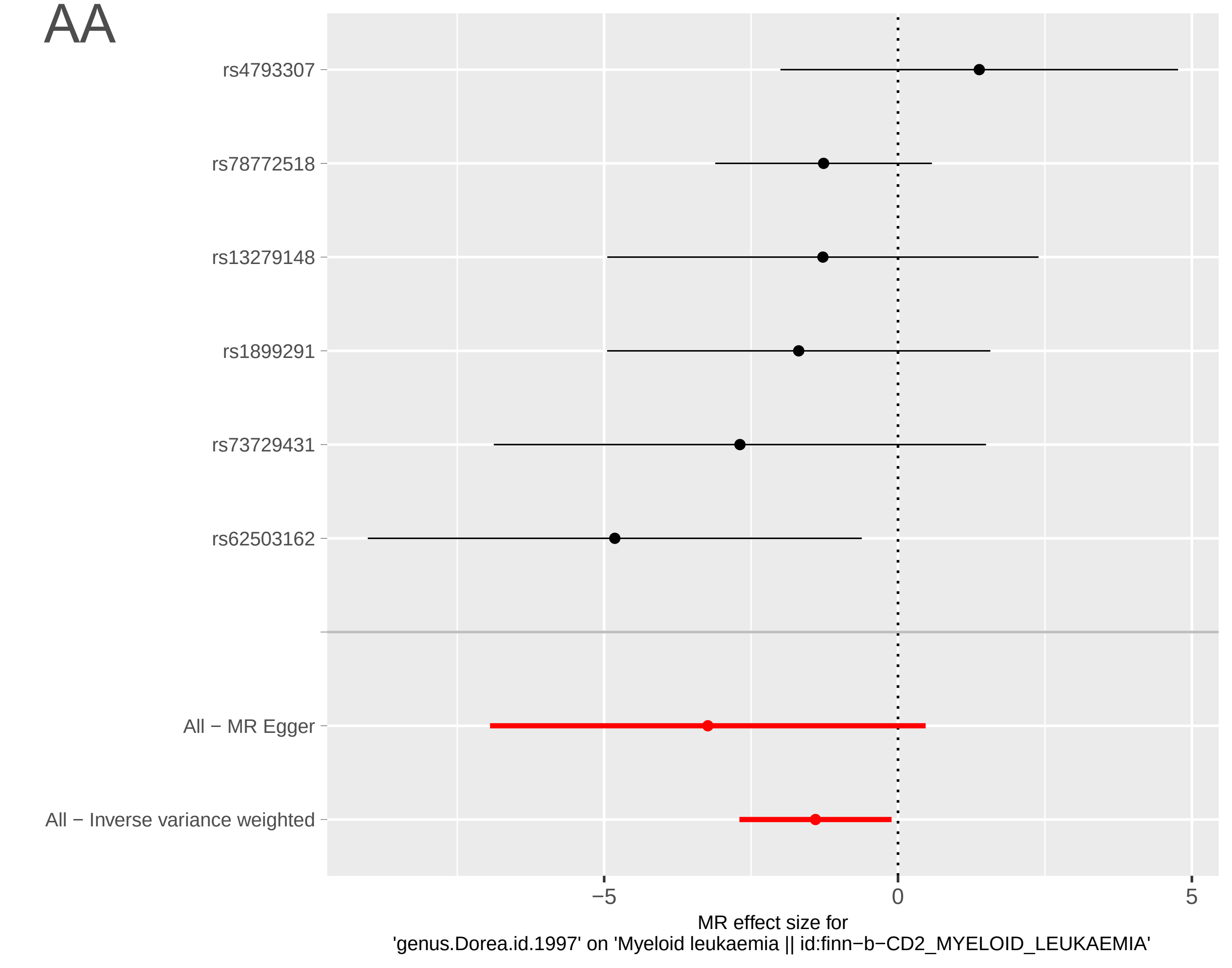

AA
rs4793307
rs78772518
rs13279148
rs1899291
rs73729431
rs62503162
All − MR Egger
All − Inverse variance weighted
−5
0
5
MR effect size for
'genus.Dorea.id.1997' on 'Myeloid leukaemia || id:finn−b−CD2_MYELOID_LEUKAEMIA'

AB

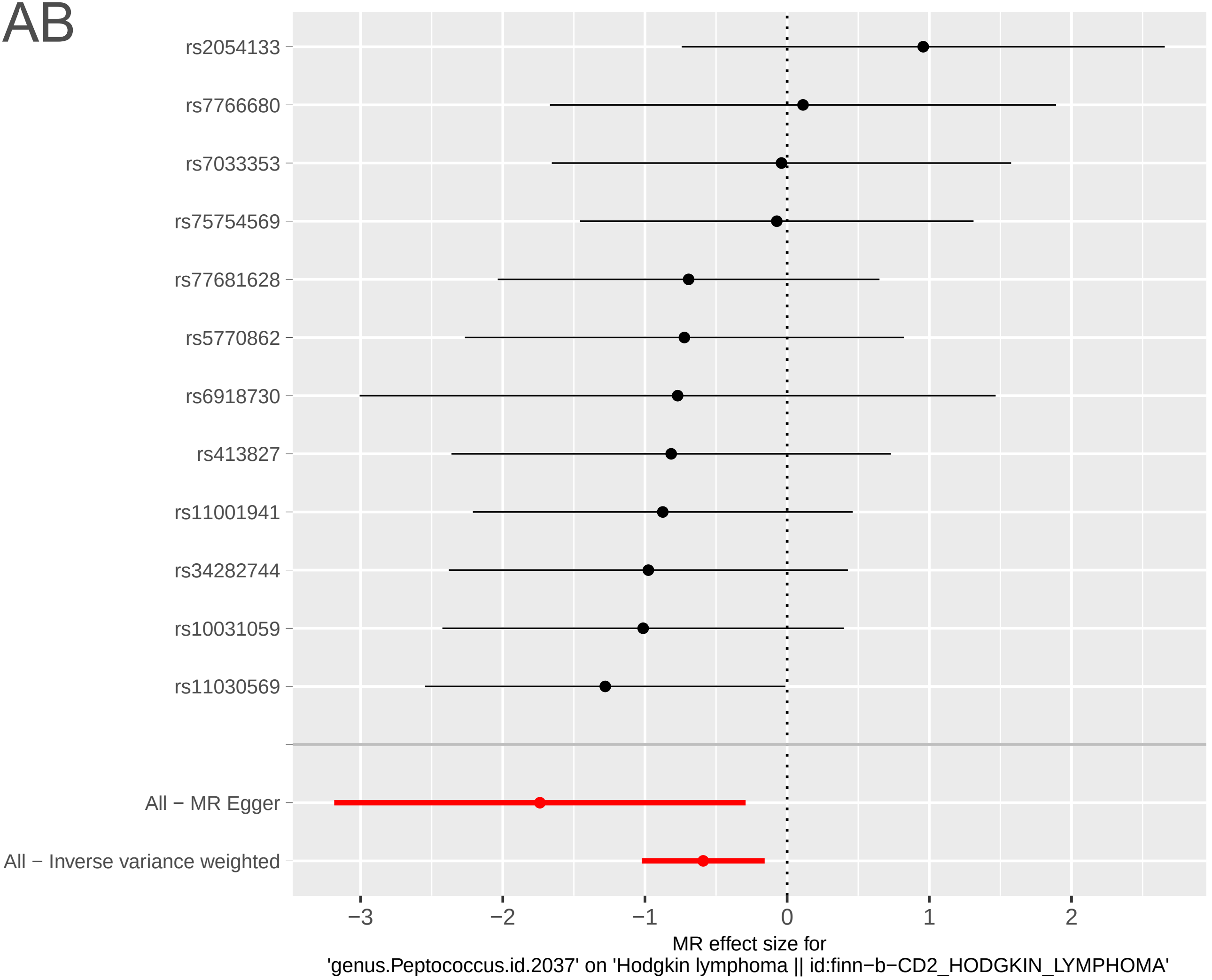
rs2054133
rs7766680
rs7033353
rs75754569
rs77681628
rs5770862
rs6918730
rs413827
rs11001941
rs34282744
rs10031059
rs11030569
All − MR Egger
All − Inverse variance weighted
−3
−2
−1
0
1
2
MR effect size for
'genus.Peptococcus.id.2037' on 'Hodgkin lymphoma || id:finn−b−CD2_HODGKIN_LYMPHOMA'

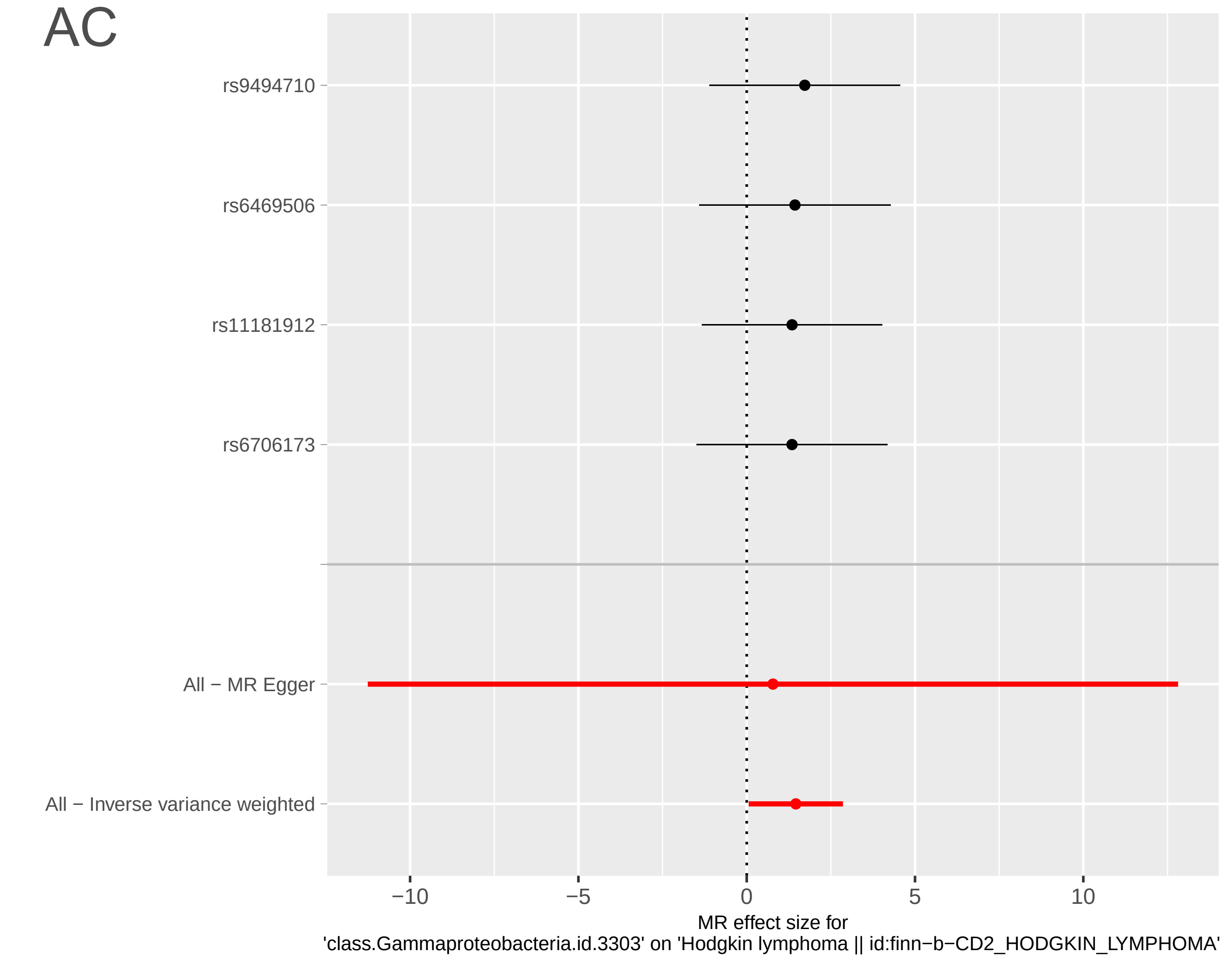
AC
rs9494710
rs6469506
rs11181912
rs6706173
All − MR Egger
All − Inverse variance weighted
−10
−5
0
5
10
MR effect size for
'class.Gammaproteobacteria.id.3303' on 'Hodgkin lymphoma || id:finn−b−CD2_HODGKIN_LYMPHOMA'

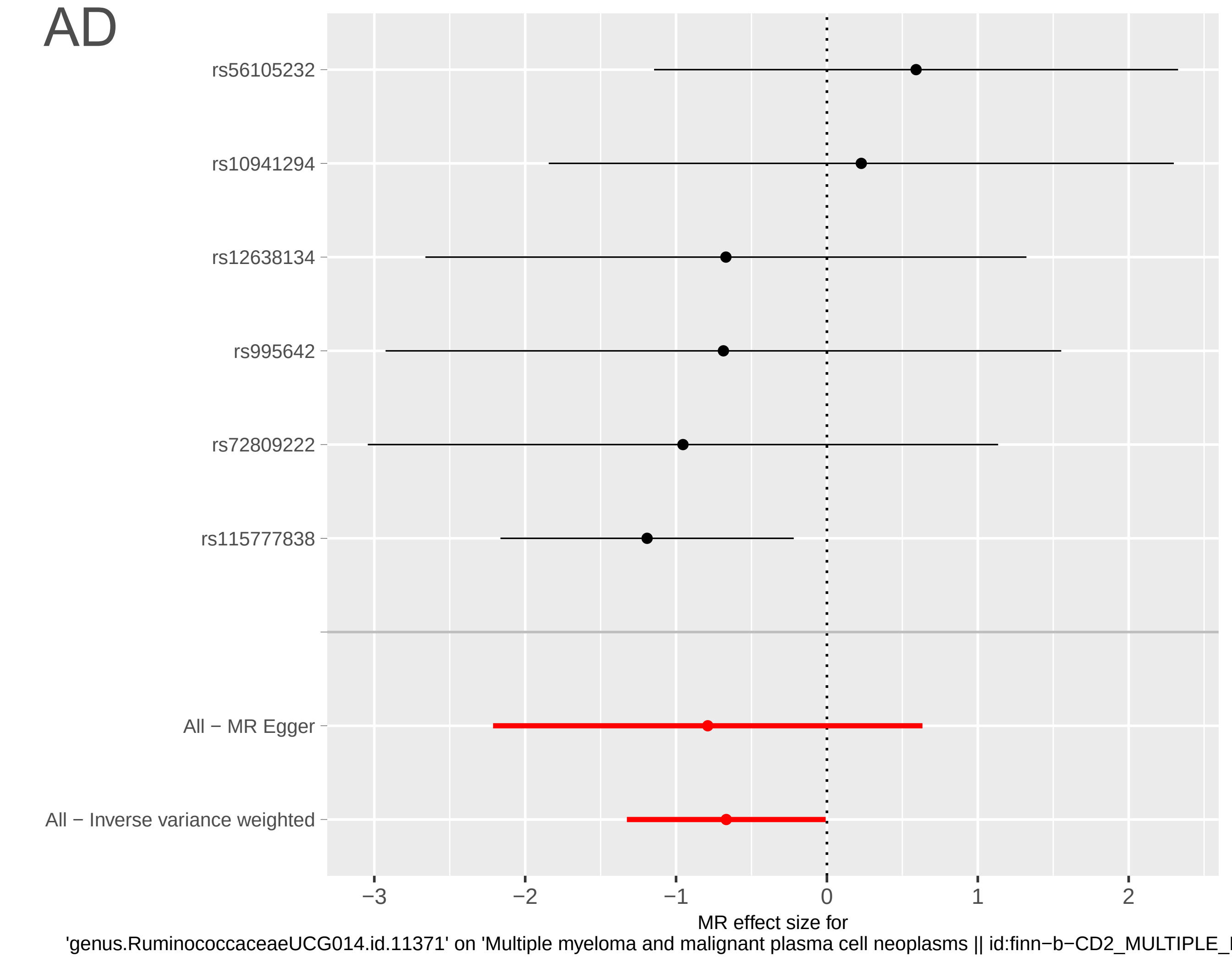
AD
rs56105232
rs10941294
rs12638134
rs995642
rs72809222
rs115777838
All − MR Egger
All − Inverse variance weighted
−3
−2
−1
0
1
2
MR effect size for
'genus.RuminococcaceaeUCG014.id.11371' on 'Multiple myeloma and malignant plasma cell neoplasms || id:finn−b−CD2_MULTIPLE_

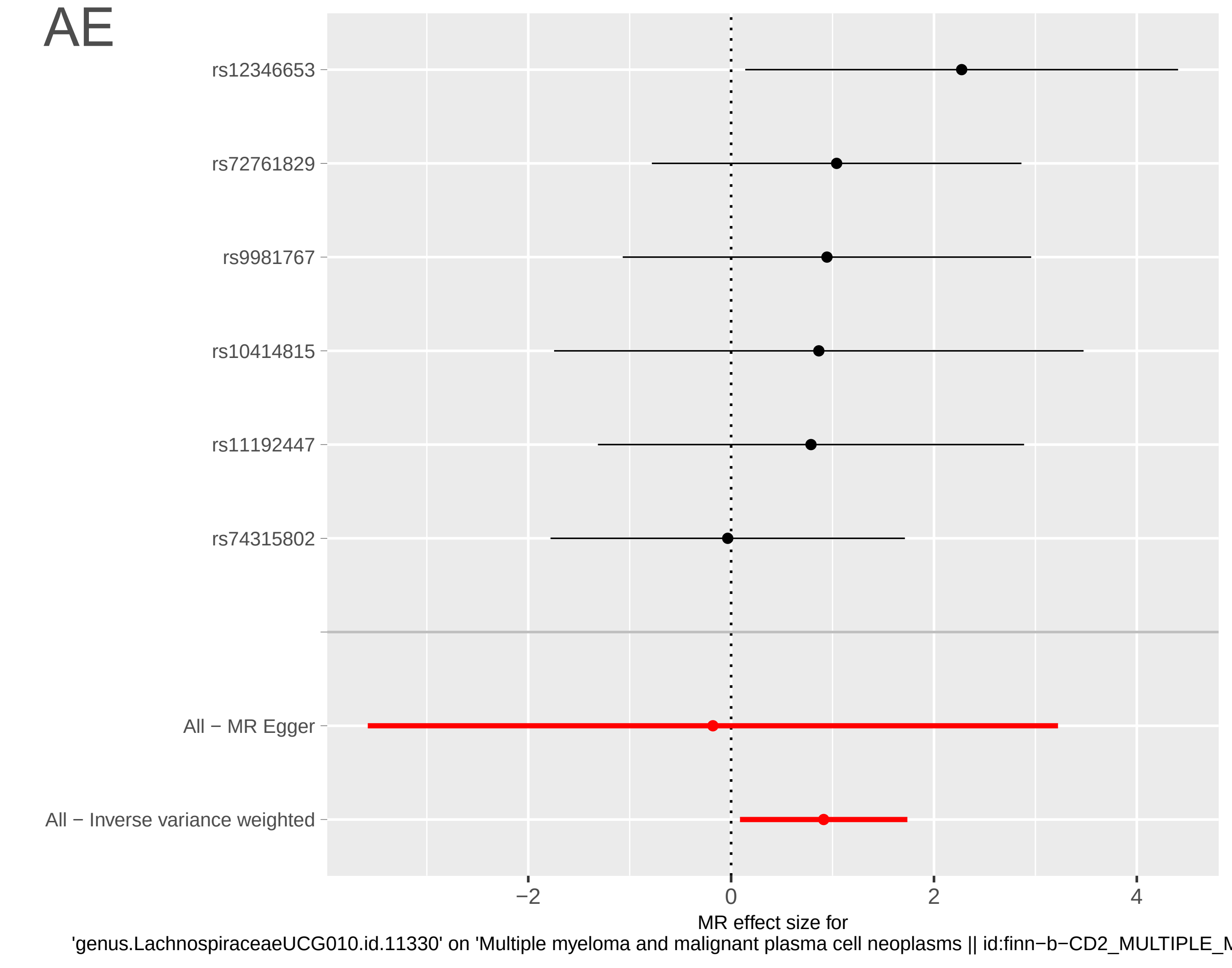
AE
rs12346653
rs72761829
rs9981767
rs10414815
rs11192447
rs74315802
All − MR Egger
All − Inverse variance weighted
−2
0
2
4
MR effect size for
'genus.LachnospiraceaeUCG010.id.11330' on 'Multiple myeloma and malignant plasma cell neoplasms || id:finn−b−CD2_MULTIPLE_M

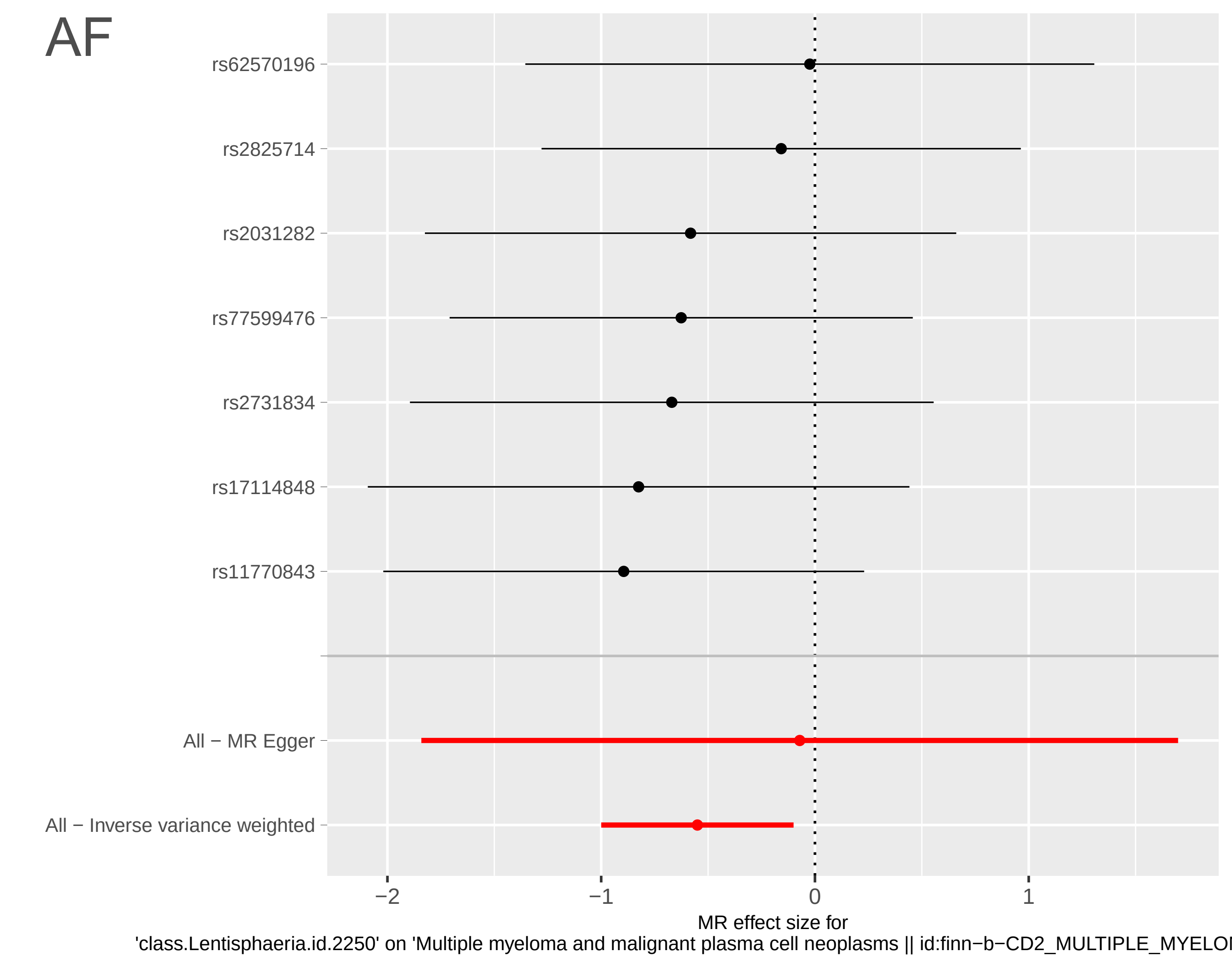
AF
rs62570196
rs2825714
rs2031282
rs77599476
rs2731834
rs17114848
rs11770843
All − MR Egger
All − Inverse variance weighted
−2
−1
0
1
MR effect size for
'class.Lentisphaeria.id.2250' on 'Multiple myeloma and malignant plasma cell neoplasms || id:finn−b−CD2_MULTIPLE_MYELO

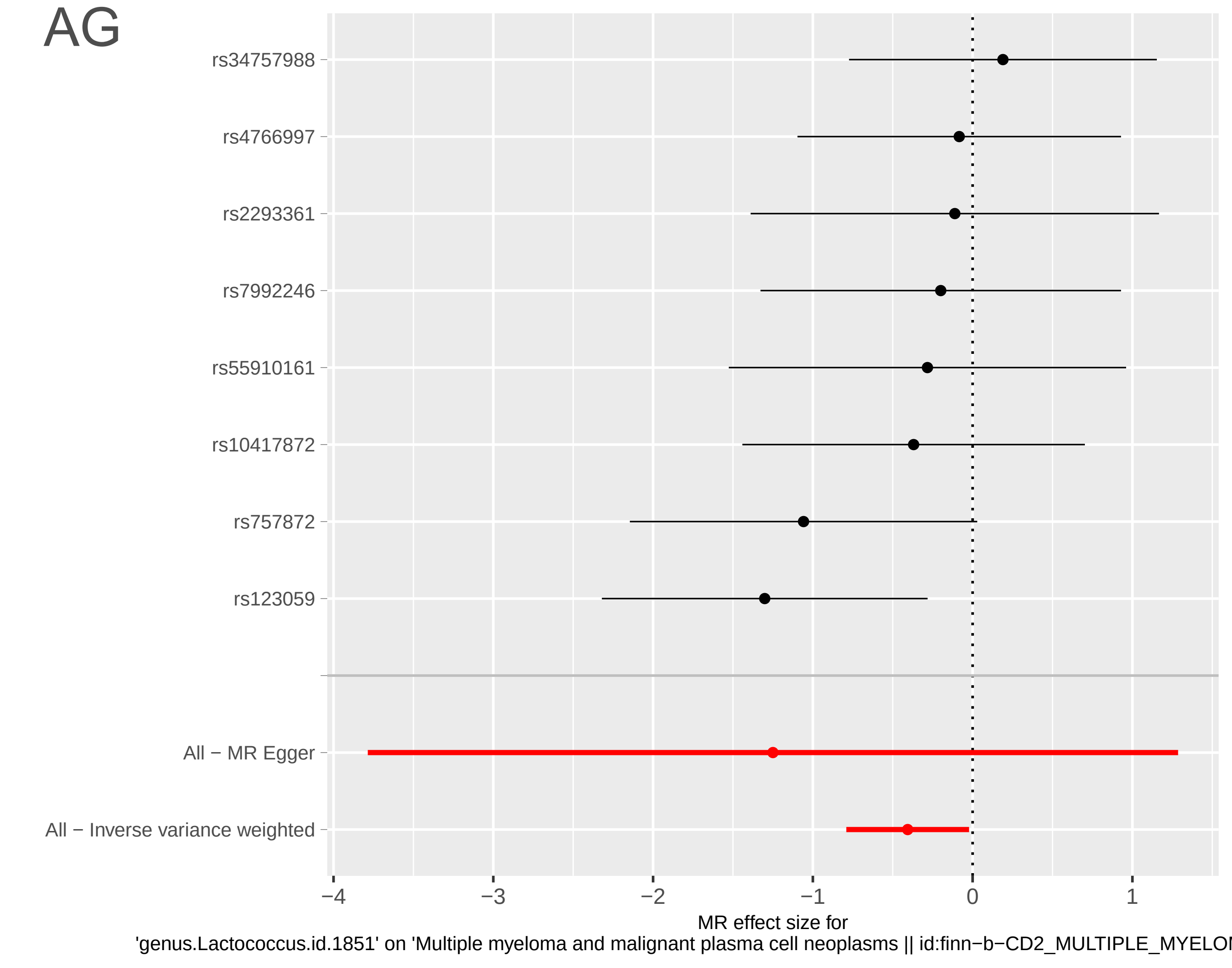
AG
rs34757988
rs4766997
rs2293361
rs7992246
rs55910161
rs10417872
rs757872
rs123059
All − MR Egger
All − Inverse variance weighted
−4
−3
−2
−1
0
1
MR effect size for
'genus.Lactococcus.id.1851' on 'Multiple myeloma and malignant plasma cell neoplasms || id:finn−b−CD2_MULTIPLE_MYELO

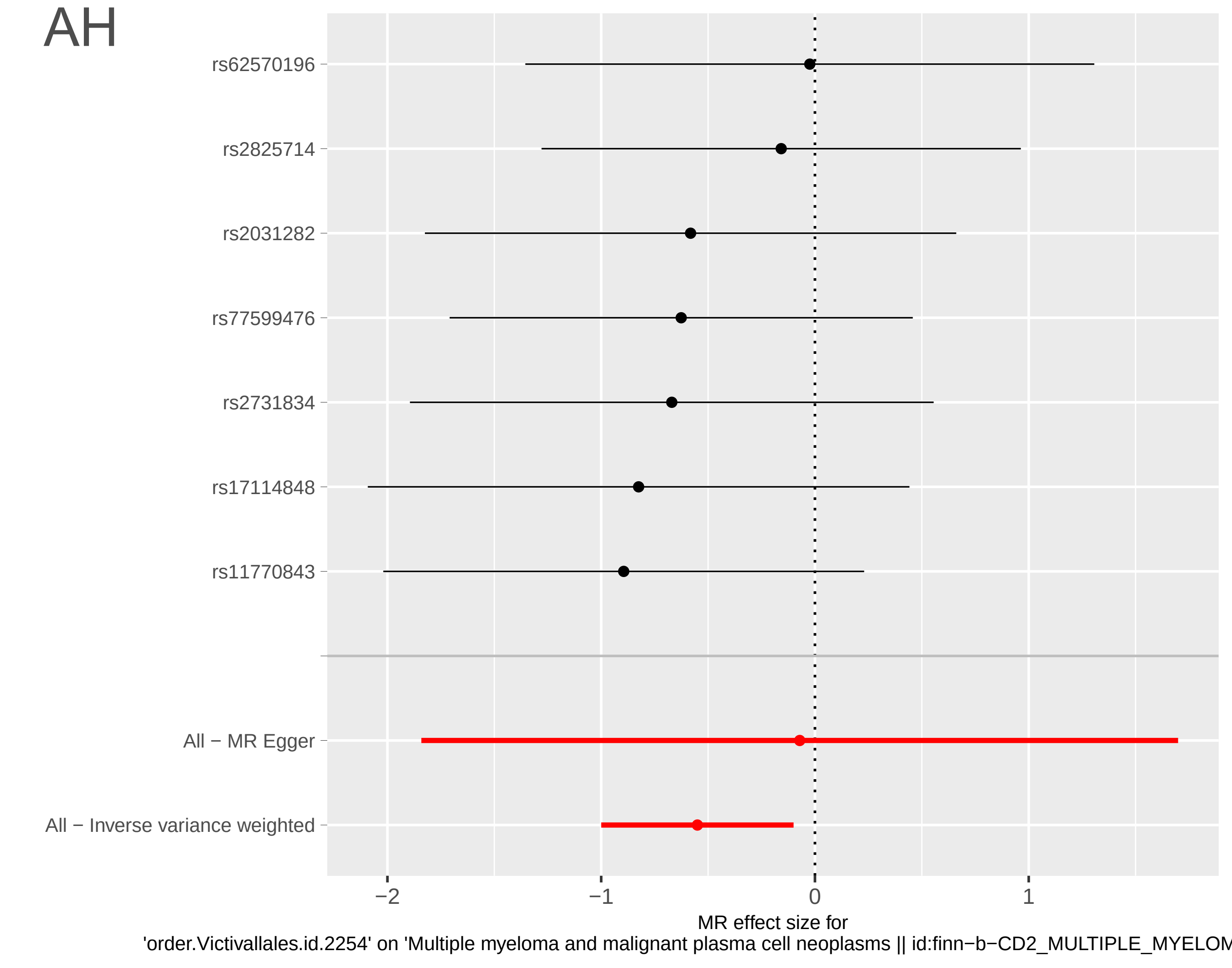
AH
rs62570196
rs2825714
rs2031282
rs77599476
rs2731834
rs17114848
rs11770843
All − MR Egger
All − Inverse variance weighted
−2
−1
0
1
MR effect size for
'order.Victivallales.id.2254' on 'Multiple myeloma and malignant plasma cell neoplasms || id:finn−b−CD2_MULTIPLE_MYELOM

AI

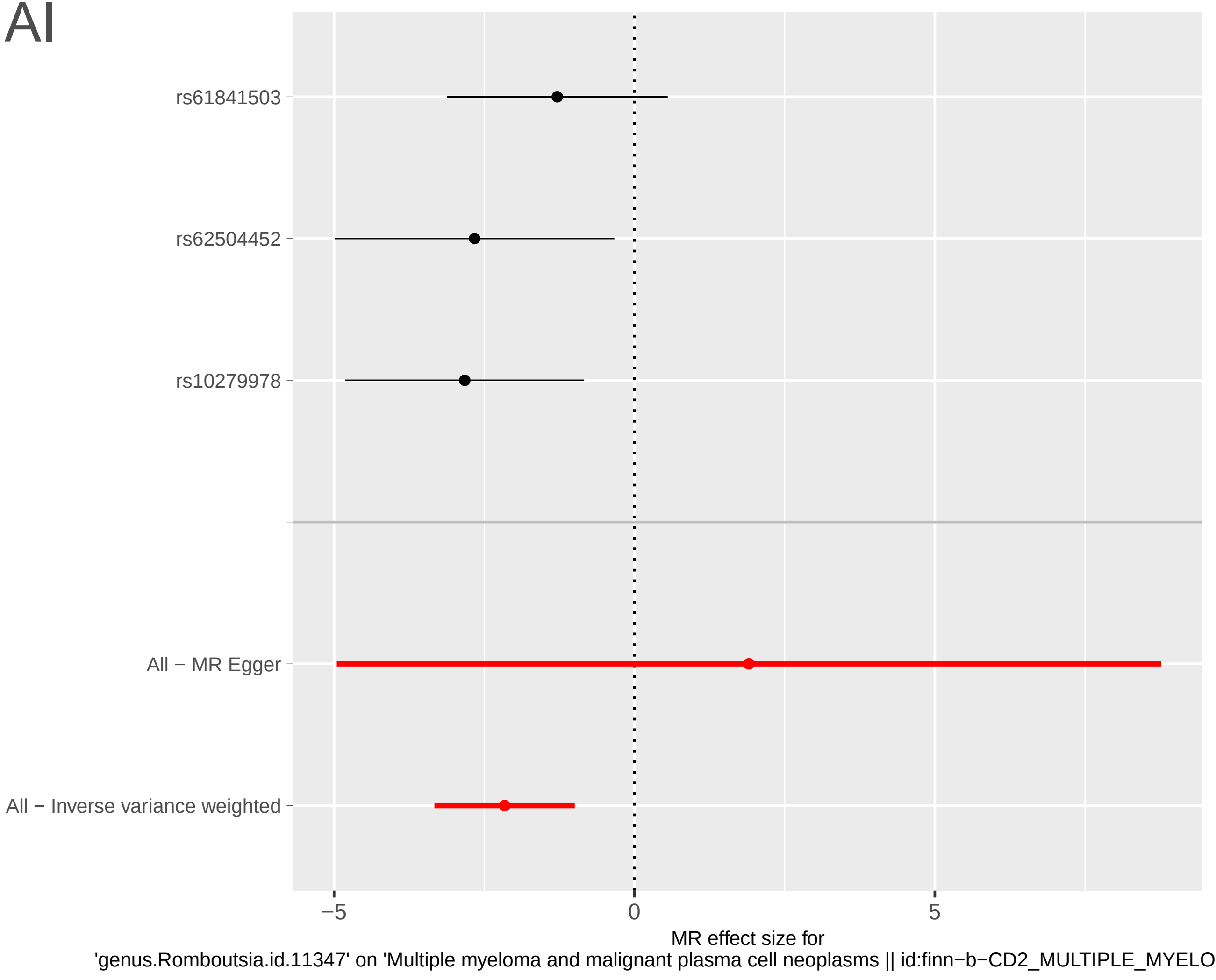
rs61841503
rs62504452
rs10279978
All − MR Egger
All − Inverse variance weighted
−5
0
5
MR effect size for
'genus.Romboutsia.id.11347' on 'Multiple myeloma and malignant plasma cell neoplasms || id:finn−b−CD2_MULTIPLE_MYELO

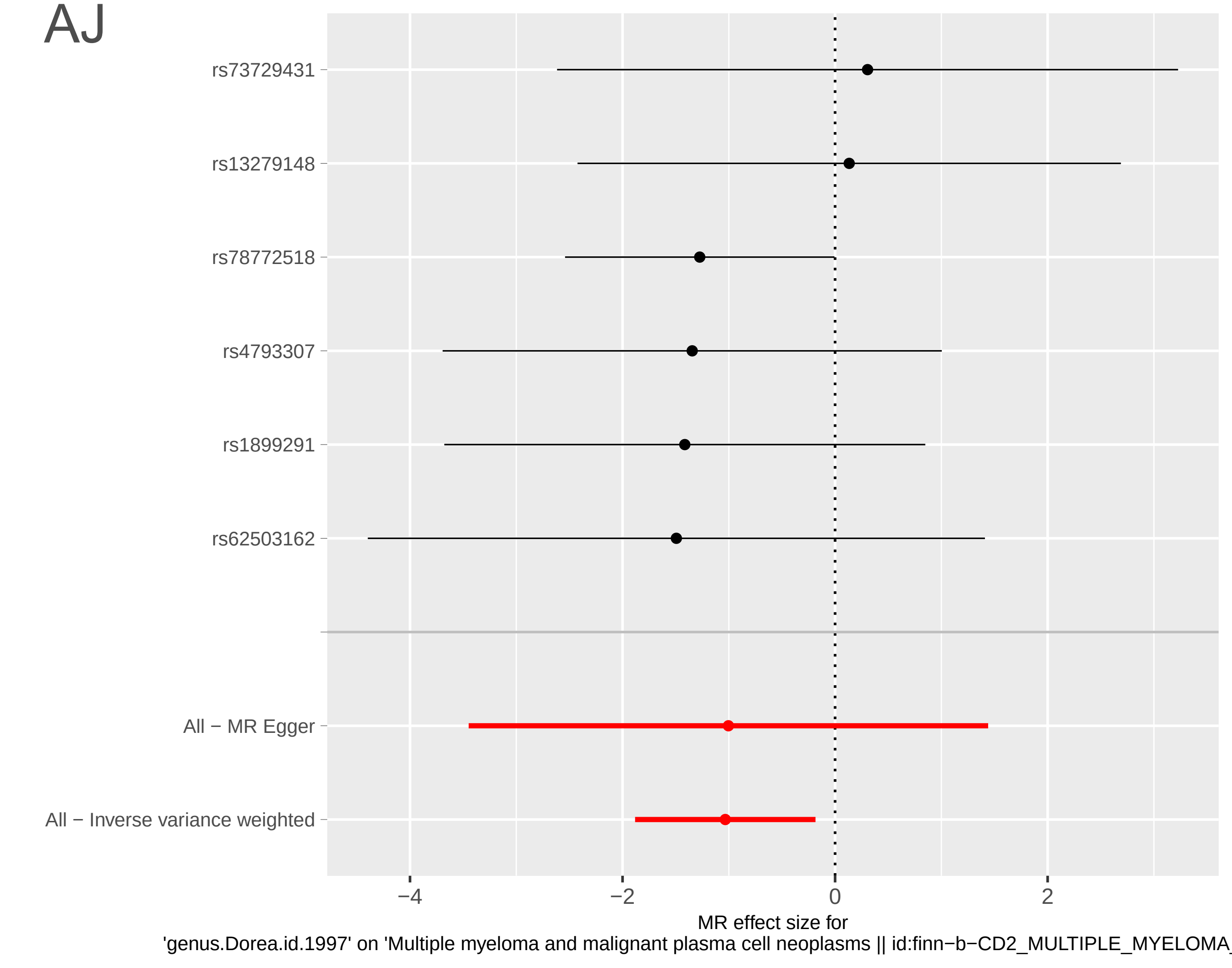
AJ
rs73729431
rs13279148
rs78772518
rs4793307
rs1899291
rs62503162
All − MR Egger
All − Inverse variance weighted
−4
−2
0
2
MR effect size for
'genus.Dorea.id.1997' on 'Multiple myeloma and malignant plasma cell neoplasms || id:finn−b−CD2_MULTIPLE_MYELOMA_

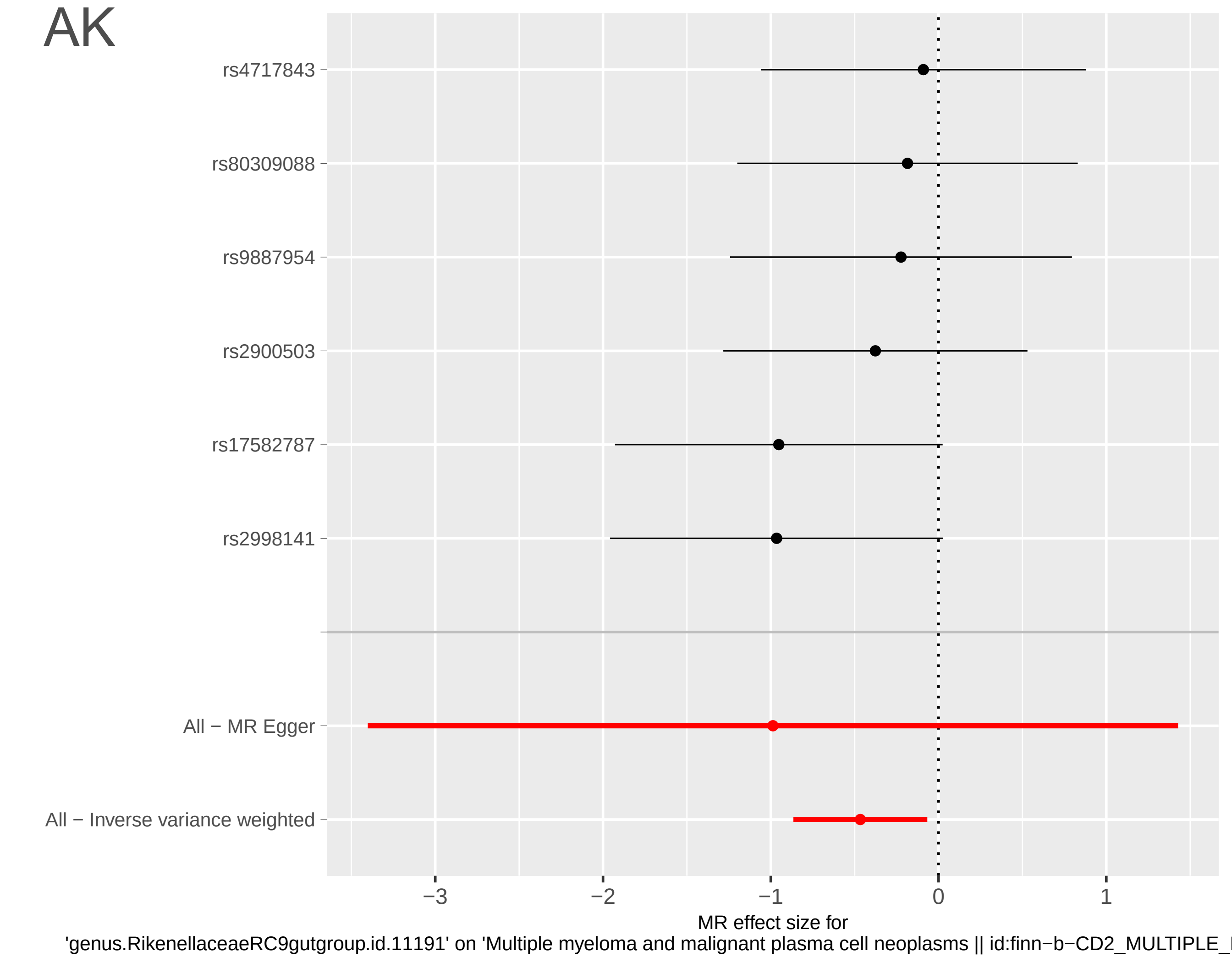
AK
rs4717843
rs80309088
rs9887954
rs2900503
rs17582787
rs2998141
All − MR Egger
All − Inverse variance weighted
−3
−2
−1
0
1
MR effect size for
'genus.RikenellaceaeRC9gutgroup.id.11191' on 'Multiple myeloma and malignant plasma cell neoplasms || id:finn−b−CD2_MULTIPLE_

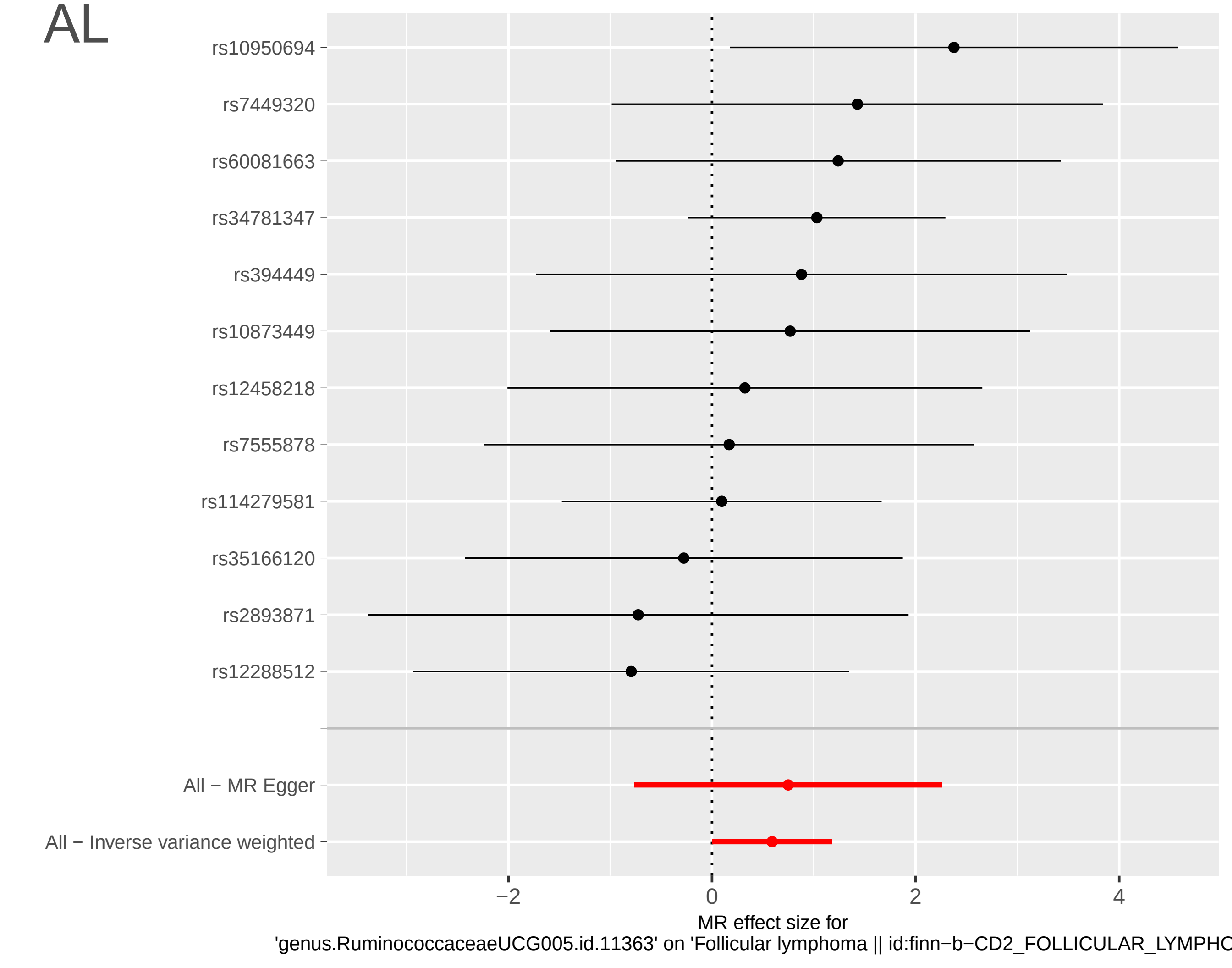
AL
rs10950694
rs7449320
rs60081663
rs34781347
rs394449
rs10873449
rs12458218
rs7555878
rs114279581
rs35166120
rs2893871
rs12288512
All − MR Egger
All − Inverse variance weighted
−2
0
2
4
MR effect size for
'genus.RuminococcaceaeUCG005.id.11363' on 'Follicular lymphoma || id:finn−b−CD2_FOLLICULAR_LYMPHO

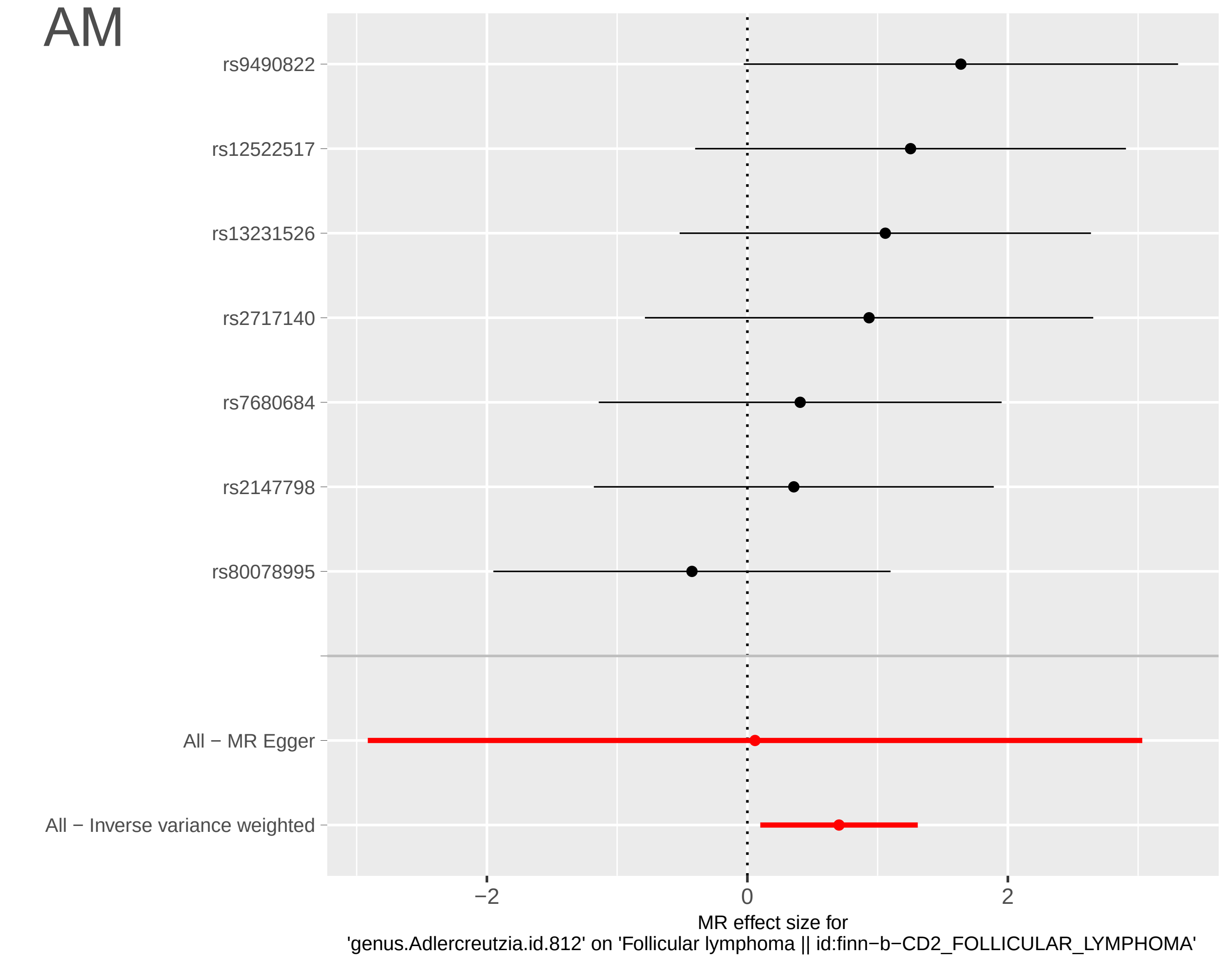
AM
rs9490822
rs12522517
rs13231526
rs2717140
rs7680684
rs2147798
rs80078995
All − MR Egger
All − Inverse variance weighted
−2
0
2
MR effect size for
'genus.Adlercreutzia.id.812' on 'Follicular lymphoma || id:finn−b−CD2_FOLLICULAR_LYMPHOMA'

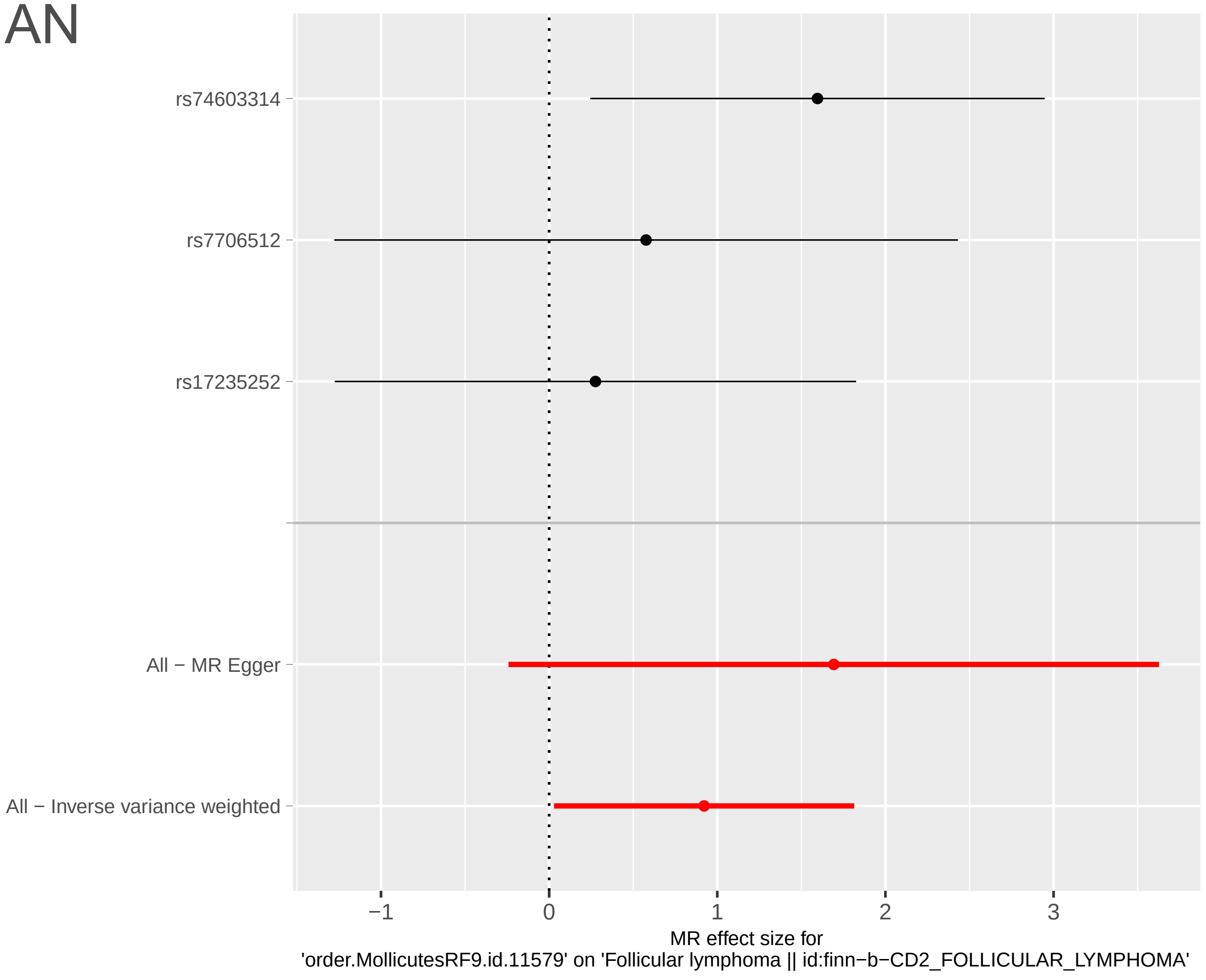
AN
rs74603314
rs7706512
rs17235252
All − MR Egger
All − Inverse variance weighted
−1
0
1
2
3
MR effect size for
'order.MollicutesRF9.id.11579' on 'Follicular lymphoma || id:finn−b−CD2_FOLLICULAR_LYMPHOMA'

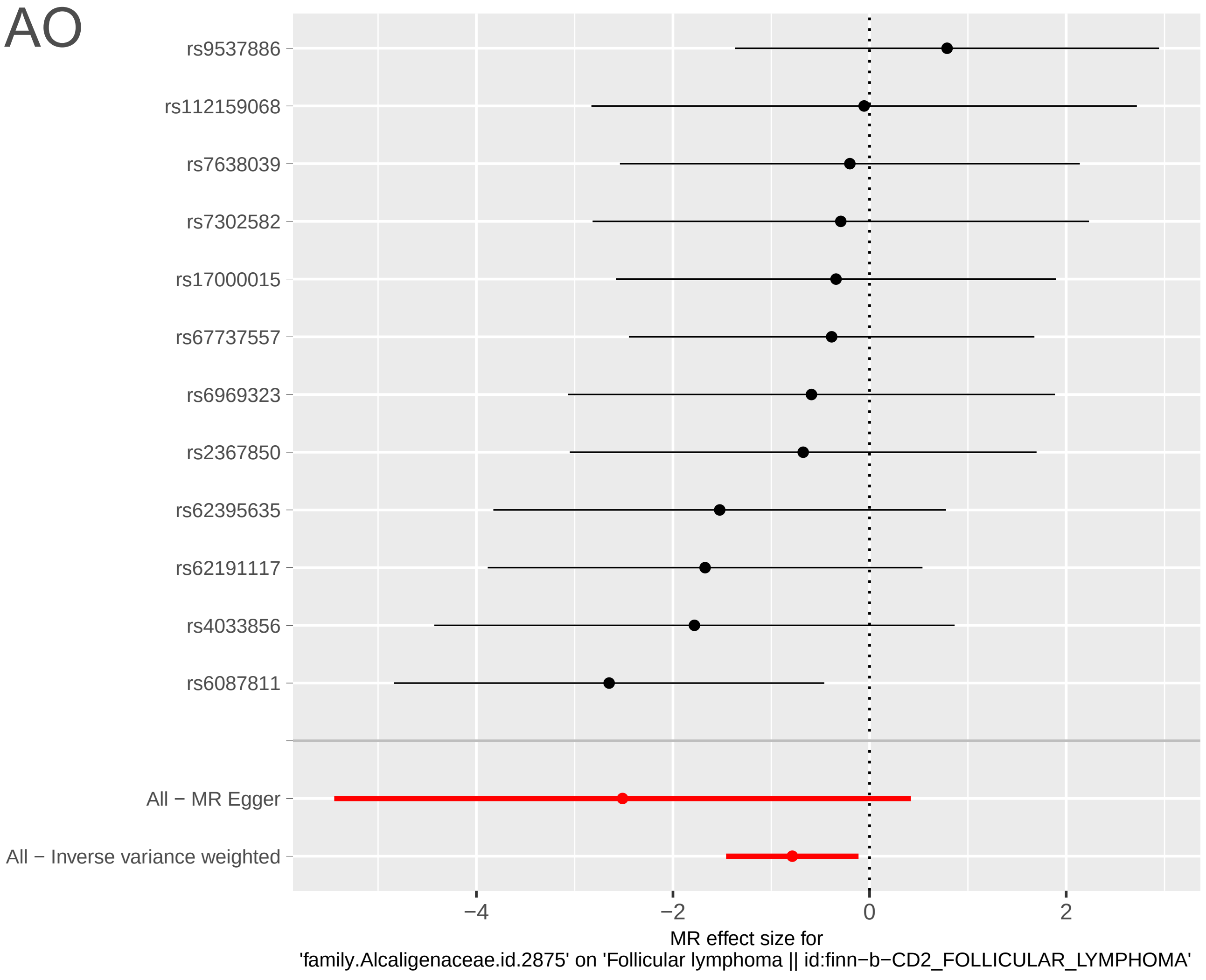
AO
rs9537886
rs112159068
rs7638039
rs7302582
rs17000015
rs67737557
rs6969323
rs2367850
rs62395635
rs62191117
rs4033856
rs6087811
All − MR Egger
All − Inverse variance weighted
−4
−2
0
2
MR effect size for
'family.Alcaligenaceae.id.2875' on 'Follicular lymphoma || id:finn−b−CD2_FOLLICULAR_LYMPHOMA'

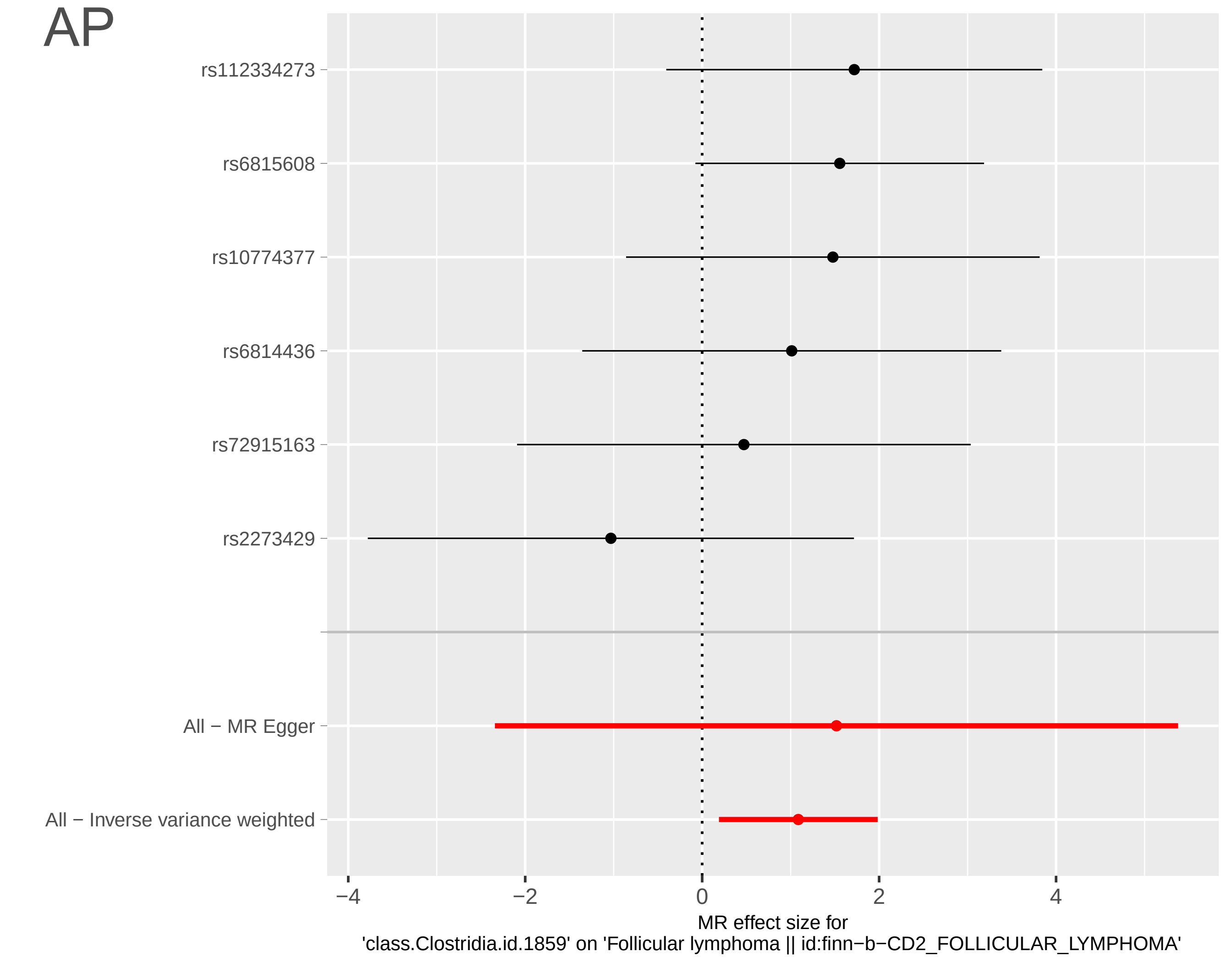
AP
rs112334273
rs6815608
rs10774377
rs6814436
rs72915163
rs2273429
All − MR Egger
All − Inverse variance weighted
−4
−2
0
2
4
MR effect size for
'class.Clostridia.id.1859' on 'Follicular lymphoma || id:finn−b−CD2_FOLLICULAR_LYMPHOMA'

AQ

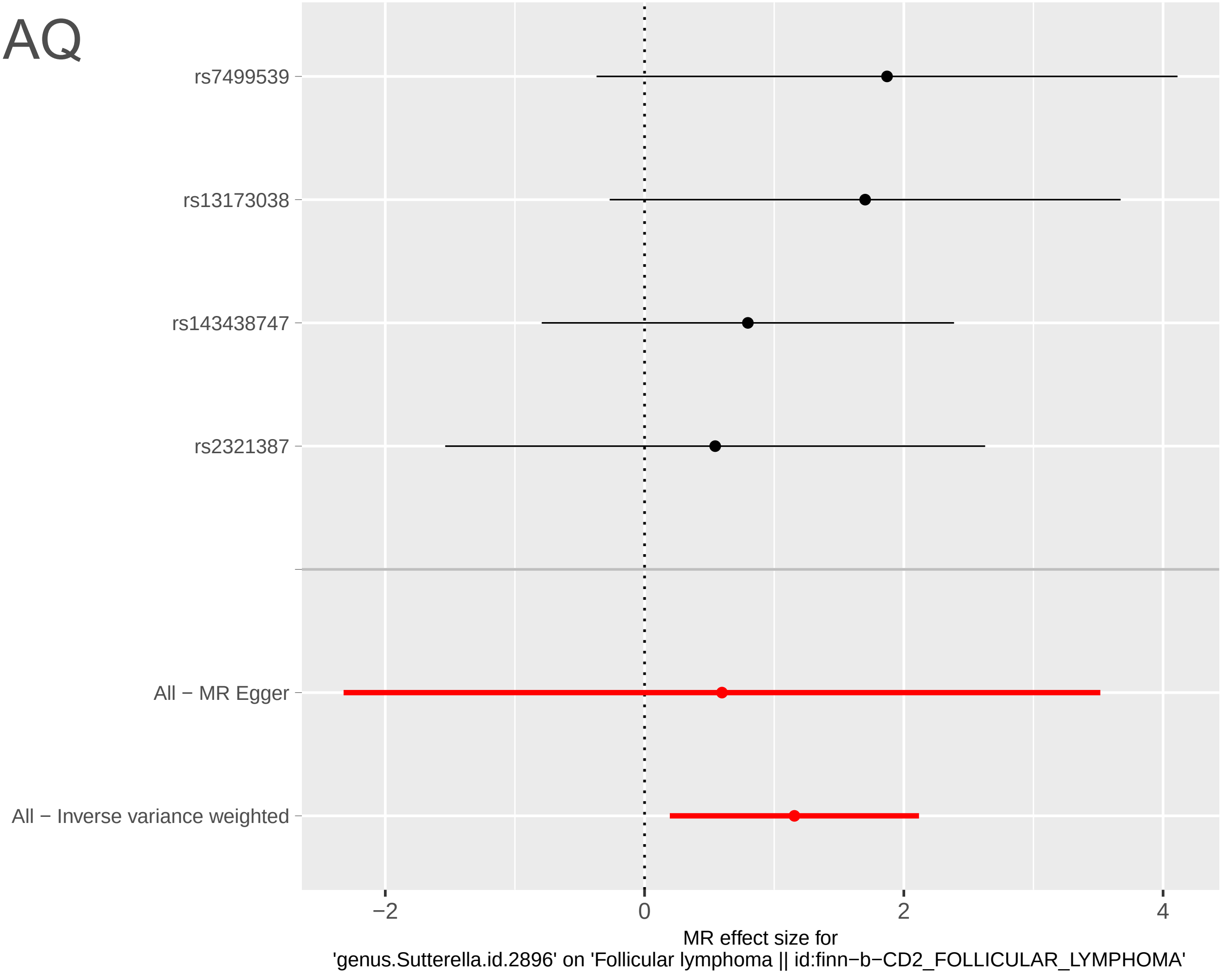

rs7499539
rs13173038
rs143438747
rs2321387
All − MR Egger
All − Inverse variance weighted
−2
0
2
4
MR effect size for
'genus.Sutterella.id.2896' on 'Follicular lymphoma || id:finn−b−CD2_FOLLICULAR_LYMPHOMA'

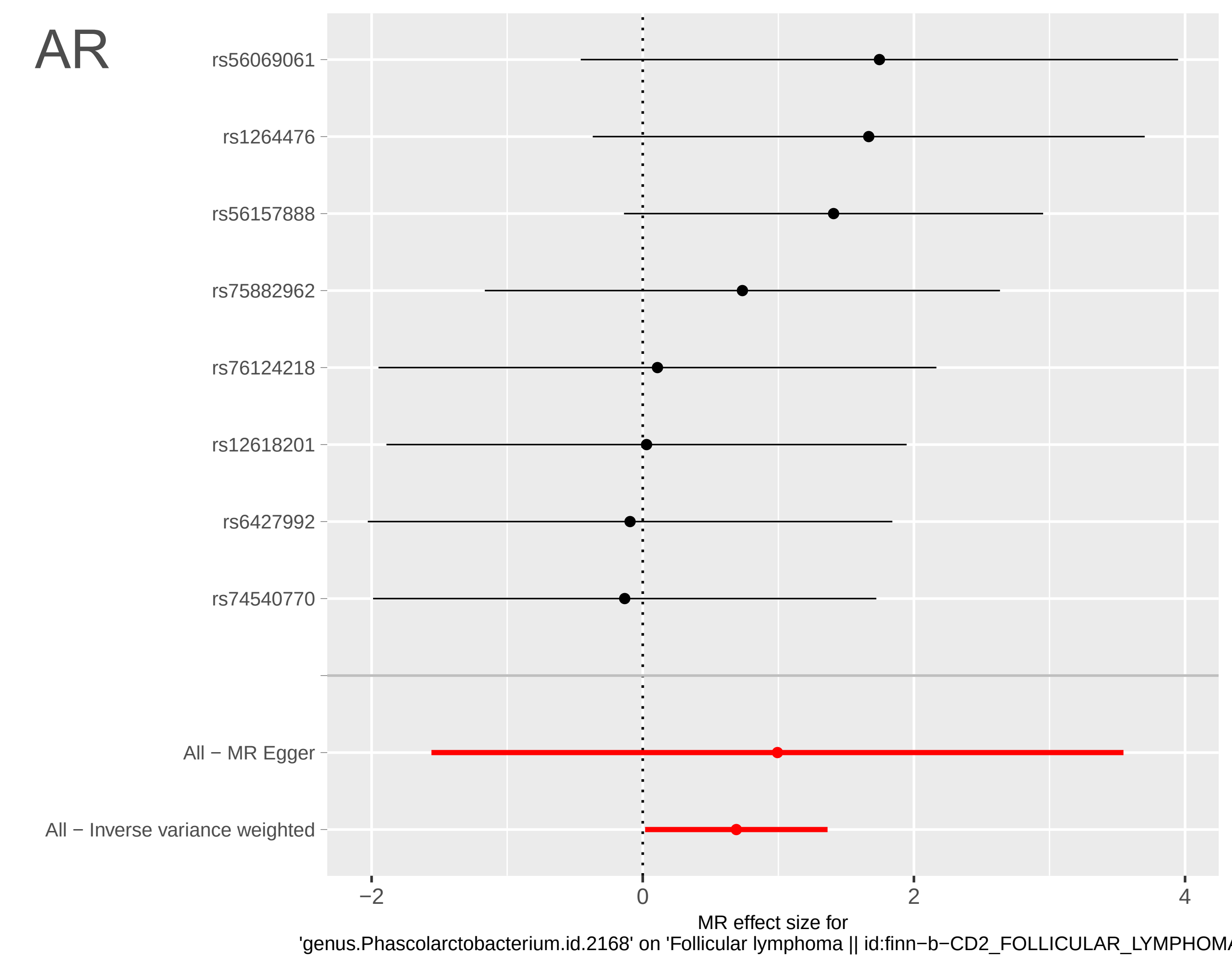
AR
rs56069061
rs1264476
rs56157888
rs75882962
rs76124218
rs12618201
rs6427992
rs74540770
All − MR Egger
All − Inverse variance weighted
−2
0
2
4
MR effect size for
'genus.Phascolarctobacterium.id.2168' on 'Follicular lymphoma || id:finn−b−CD2_FOLLICULAR_LYMPHOMA

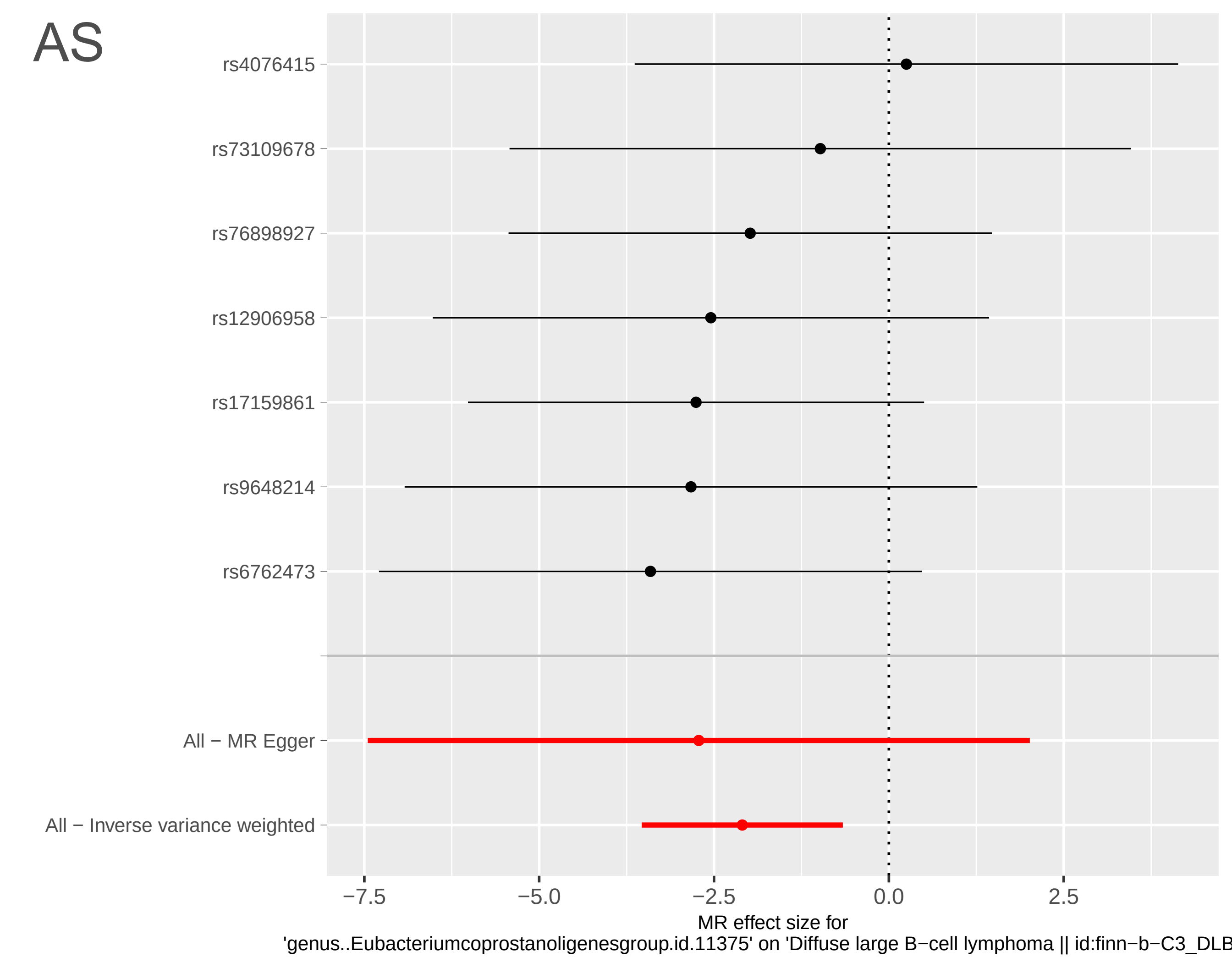

AS
rs4076415
rs73109678
rs76898927
rs12906958
rs17159861
rs9648214
rs6762473
All − MR Egger
All − Inverse variance weighted
−7.5
−5.0
−2.5
0.0
2.5
MR effect size for
'genus..Eubacteriumcoprostanoligenesgroup.id.11375' on 'Diffuse large B−cell lymphoma || id:finn−b−C3_DLB

AT

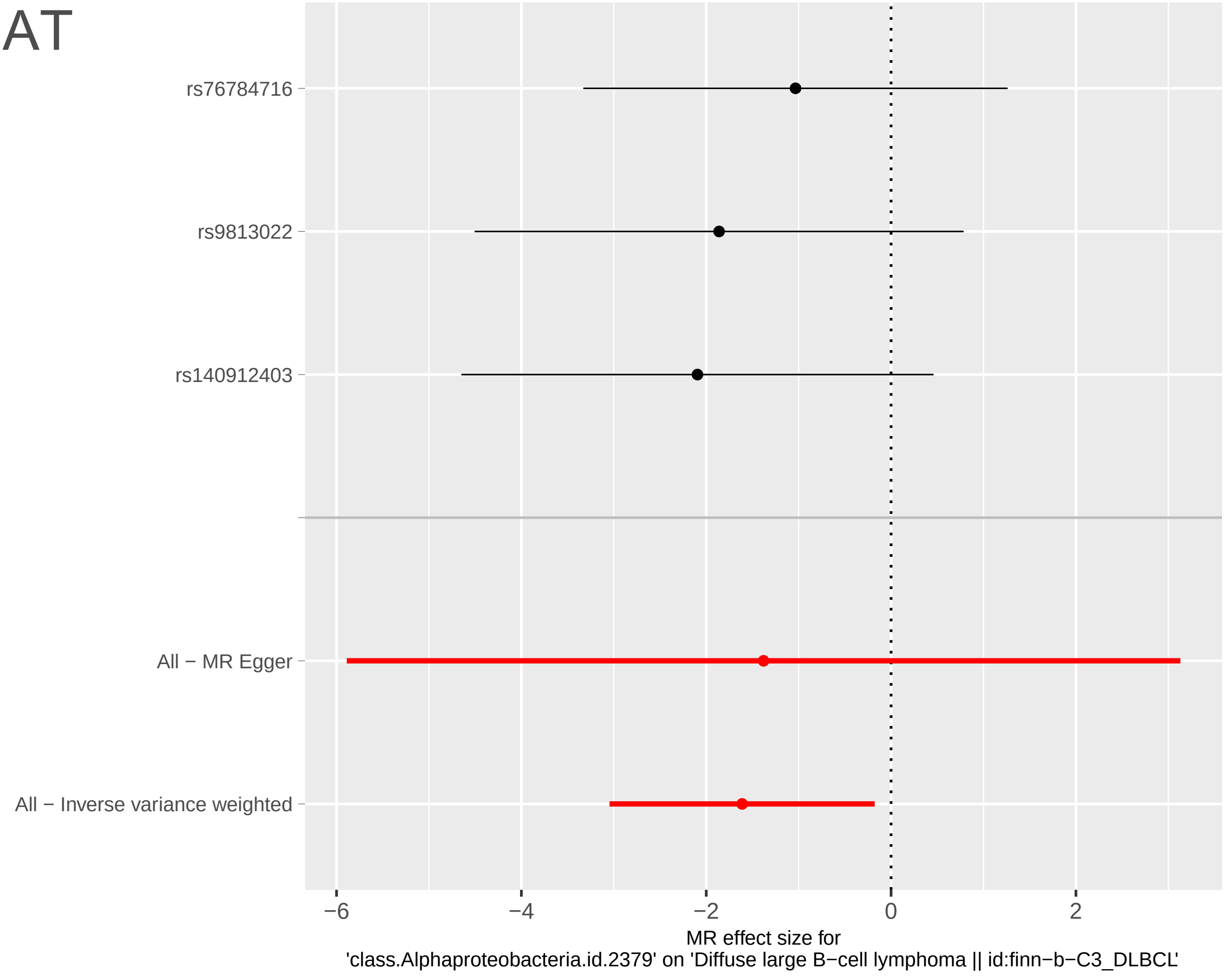
rs76784716
rs9813022
rs140912403
All − MR Egger
All − Inverse variance weighted
−6
−4
−2
0
2
MR effect size for
'class.Alphaproteobacteria.id.2379' on 'Diffuse large B−cell lymphoma || id:finn−b−C3_DLBCL'

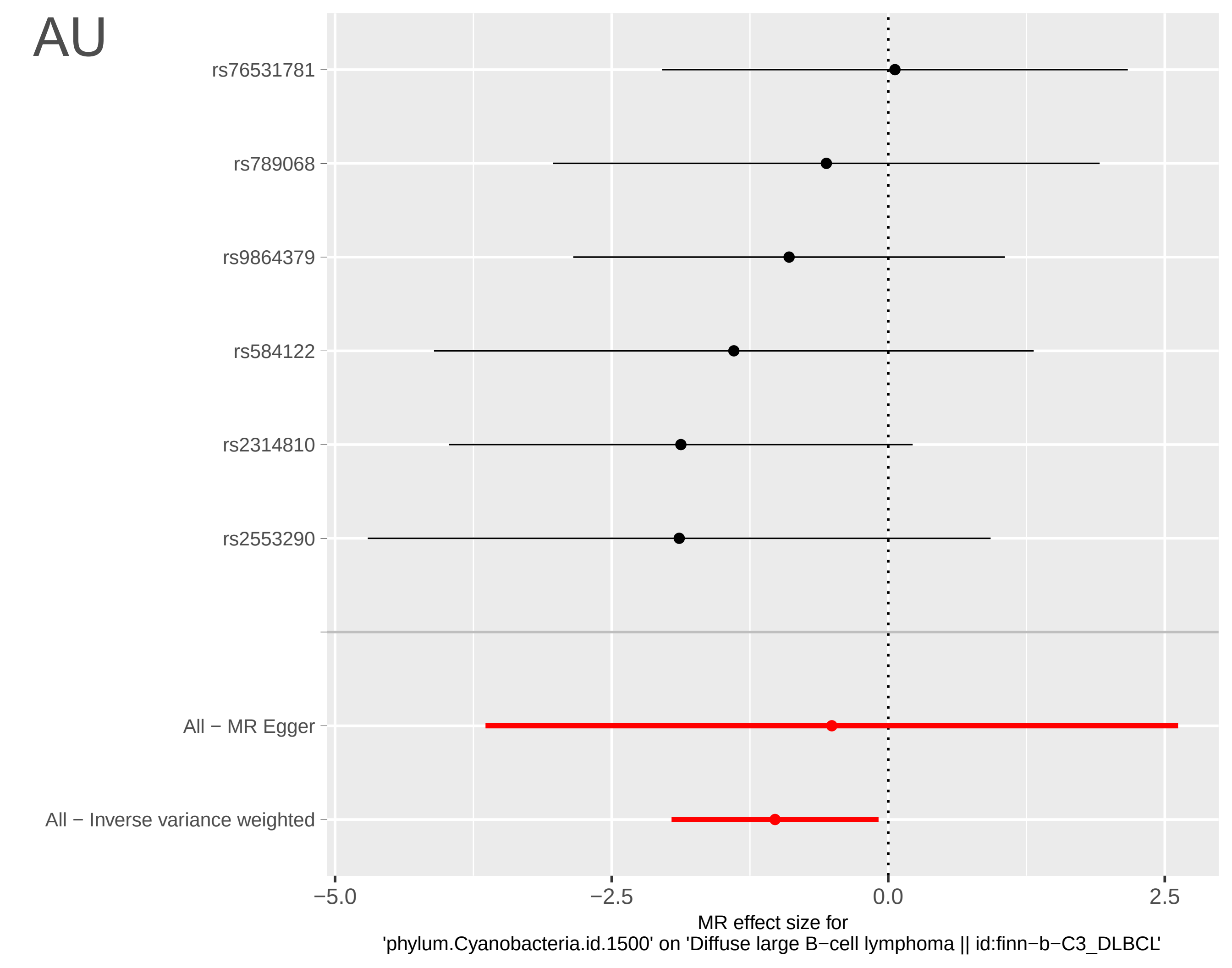
AU
rs76531781
rs789068
rs9864379
rs584122
rs2314810
rs2553290
All − MR Egger
All − Inverse variance weighted
−5.0
−2.5
0.0
2.5
MR effect size for
'phylum.Cyanobacteria.id.1500' on 'Diffuse large B−cell lymphoma || id:finn−b−C3_DLBCL'

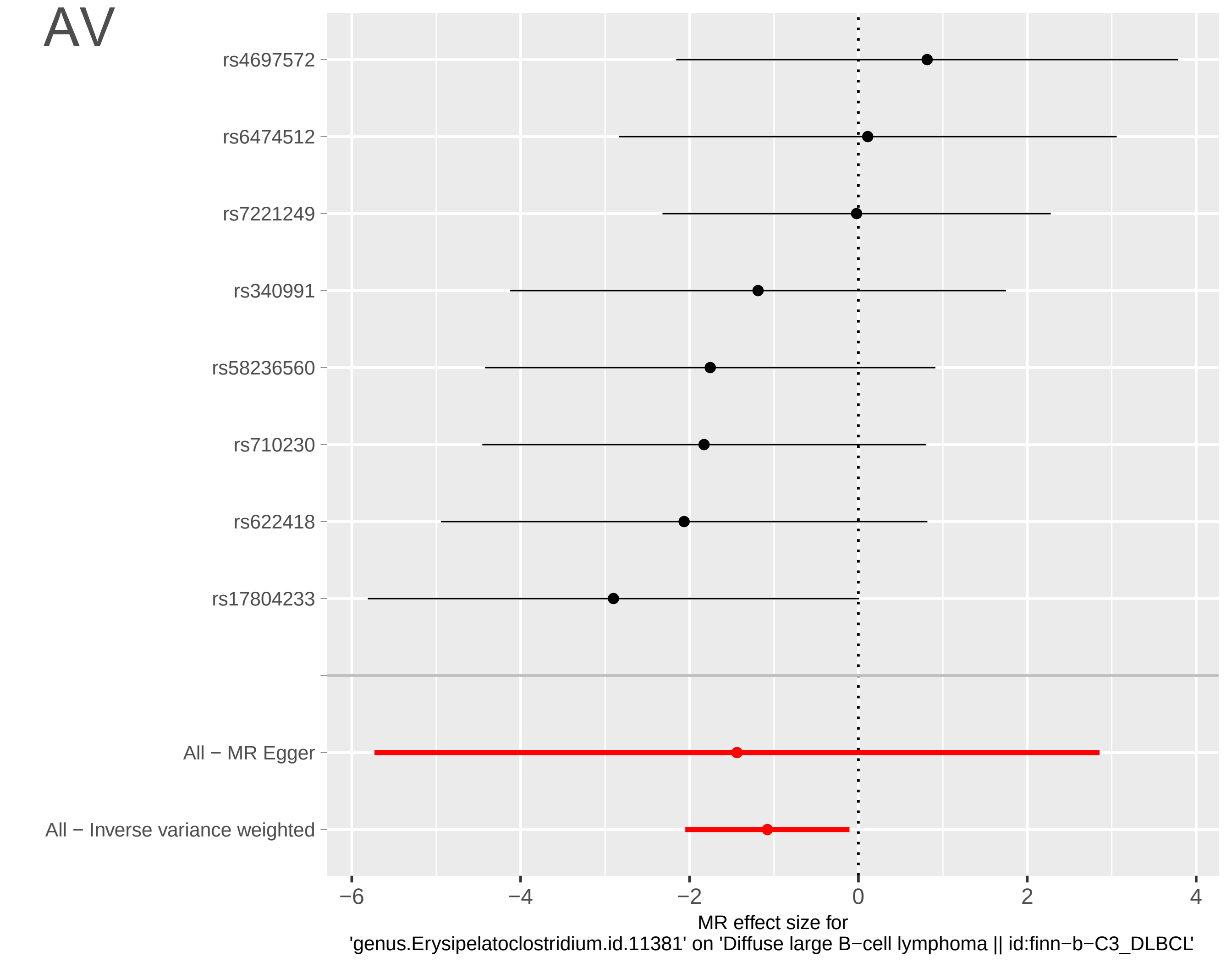
AV
rs4697572
rs6474512
rs7221249
rs340991
rs58236560
rs710230
rs622418
rs17804233
All − MR Egger
All − Inverse variance weighted
−6
−4
−2
0
2
4
MR effect size for
'genus.Erysipelatoclostridium.id.11381' on 'Diffuse large B−cell lymphoma || id:finn−b−C3_DLBCL'

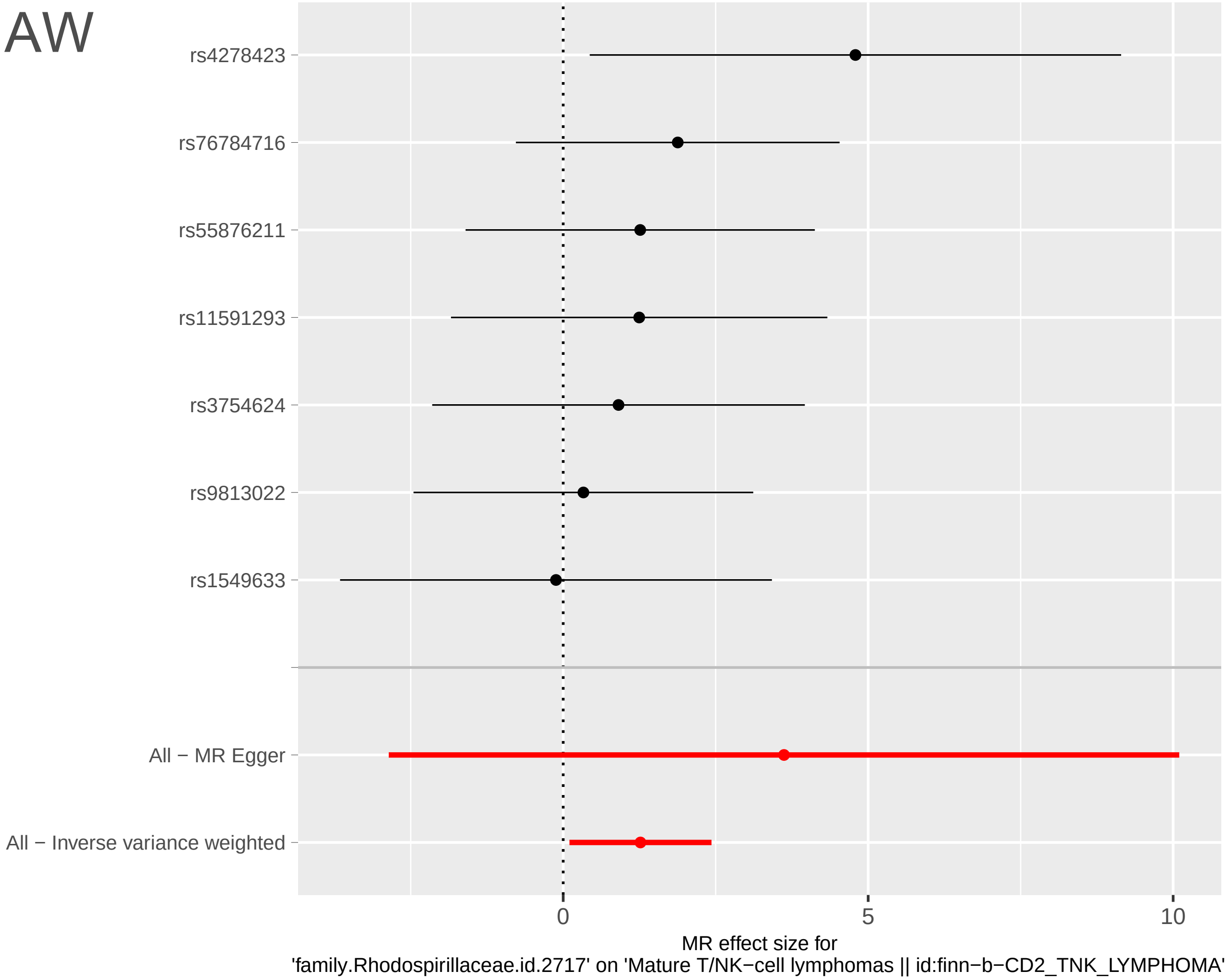
AW
rs4278423
rs76784716
rs55876211
rs11591293
rs3754624
rs9813022
rs1549633
All − MR Egger
All − Inverse variance weighted
0
5
10
MR effect size for
'family.Rhodospirillaceae.id.2717' on 'Mature T/NK−cell lymphomas || id:finn−b−CD2_TNK_LYMPHOMA'

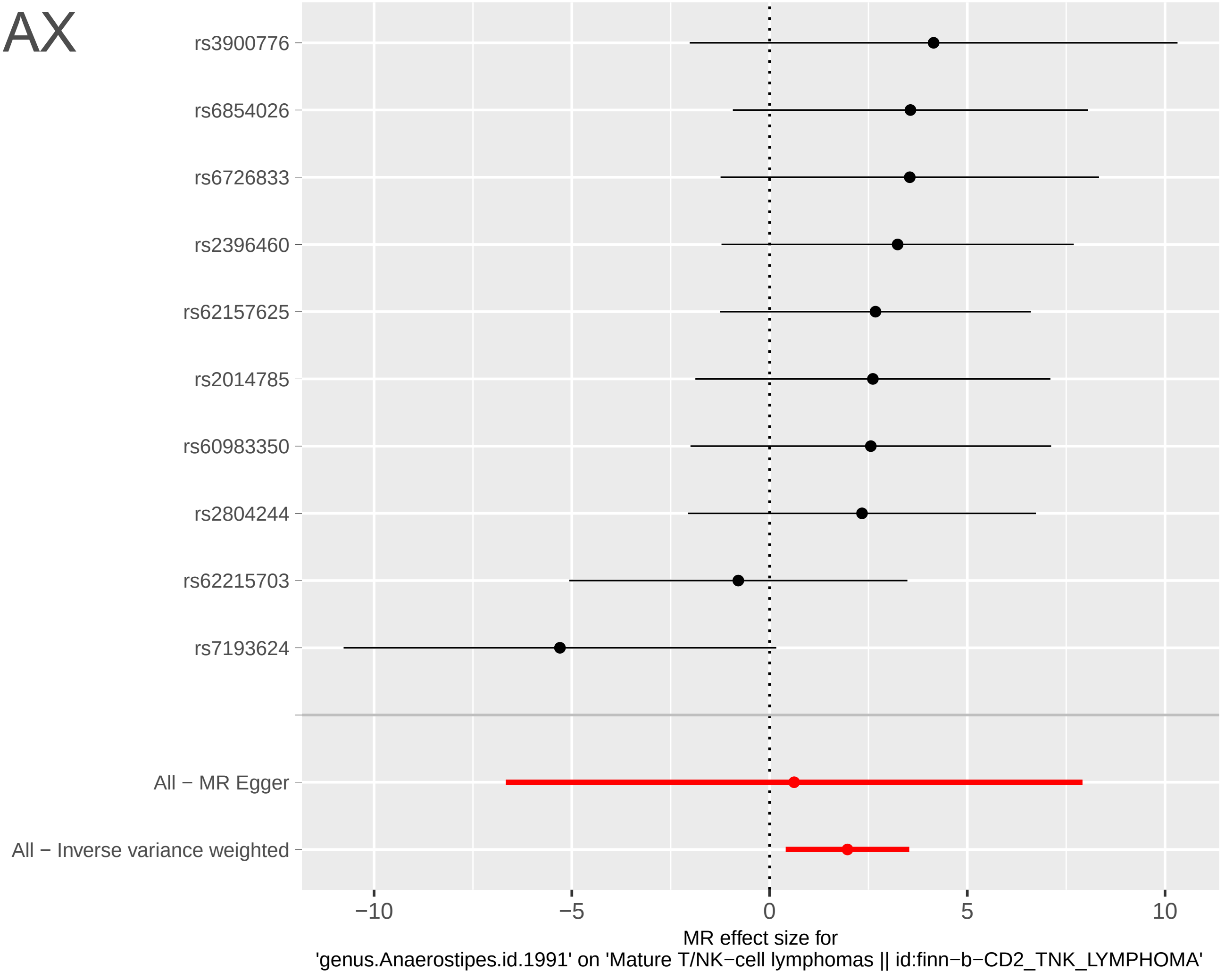
AX
rs3900776
rs6854026
rs6726833
rs2396460
rs62157625
rs2014785
rs60983350
rs2804244
rs62215703
rs7193624
All − MR Egger
All − Inverse variance weighted
−10
−5
0
5
10
MR effect size for
'genus.Anaerostipes.id.1991' on 'Mature T/NK−cell lymphomas || id:finn−b−CD2_TNK_LYMPHOMA'

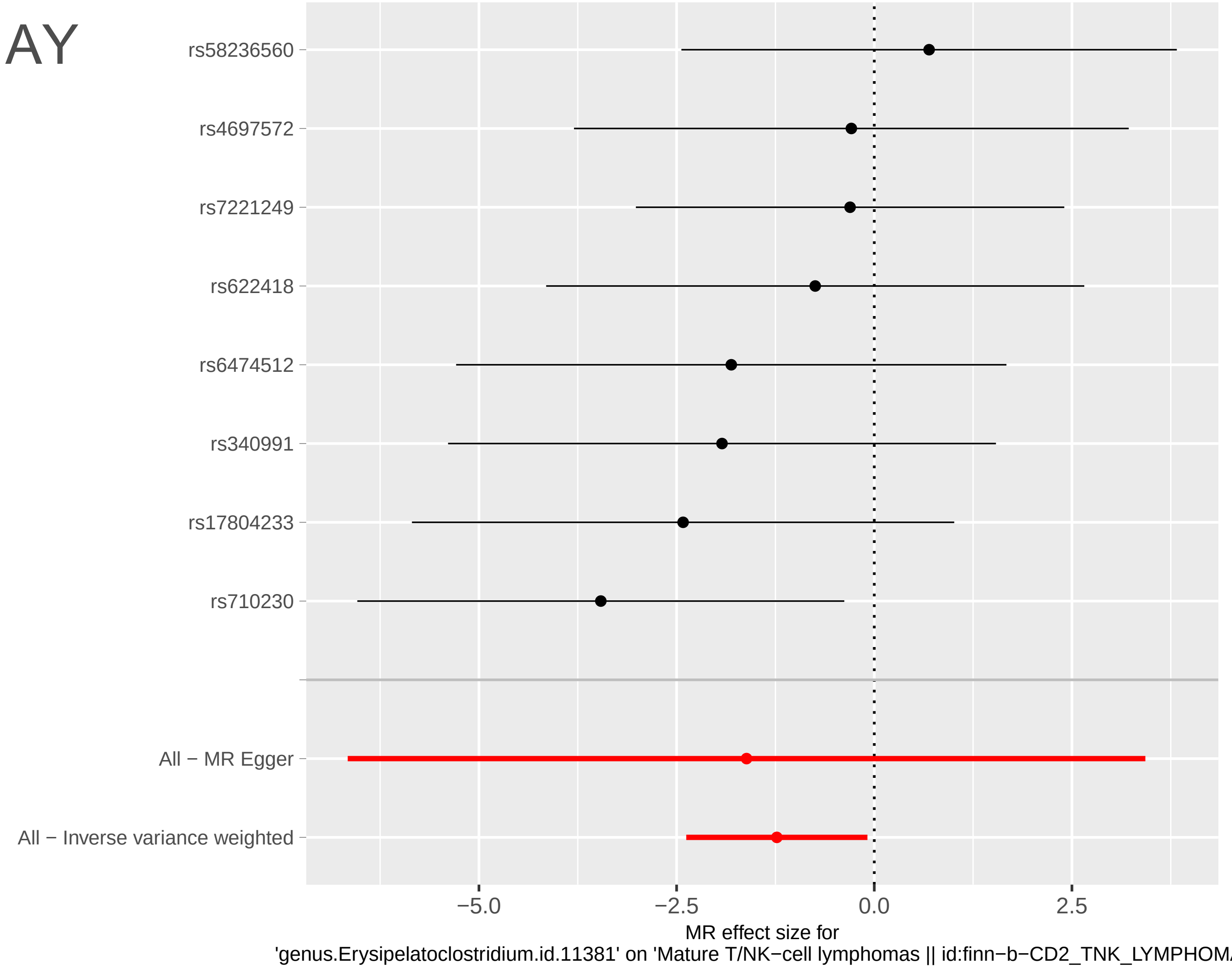
AY
rs58236560
rs4697572
rs7221249
rs622418
rs6474512
rs340991
rs17804233
rs710230
All − MR Egger
All − Inverse variance weighted
−5.0
−2.5
0.0
2.5
MR effect size for
'genus.Erysipelatoclostridium.id.11381' on 'Mature T/NK−cell lymphomas || id:finn−b−CD2_TNK_LYMPHOM

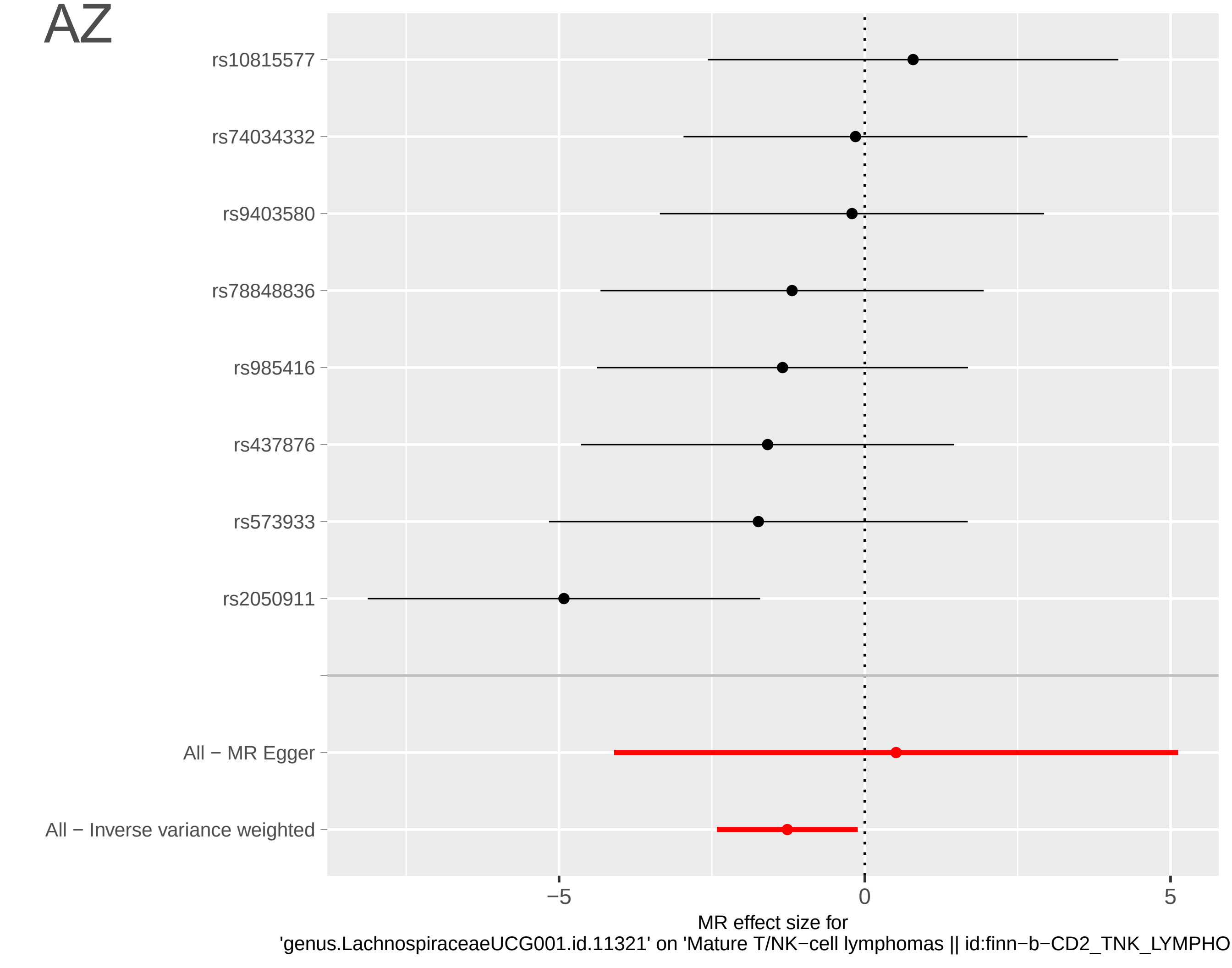
AZ
rs10815577
rs74034332
rs9403580
rs78848836
rs985416
rs437876
rs573933
rs2050911
All − MR Egger
All − Inverse variance weighted
−5
0
5
MR effect size for
'genus.LachnospiraceaeUCG001.id.11321' on 'Mature T/NK−cell lymphomas || id:finn−b−CD2_TNK_LYMPHO

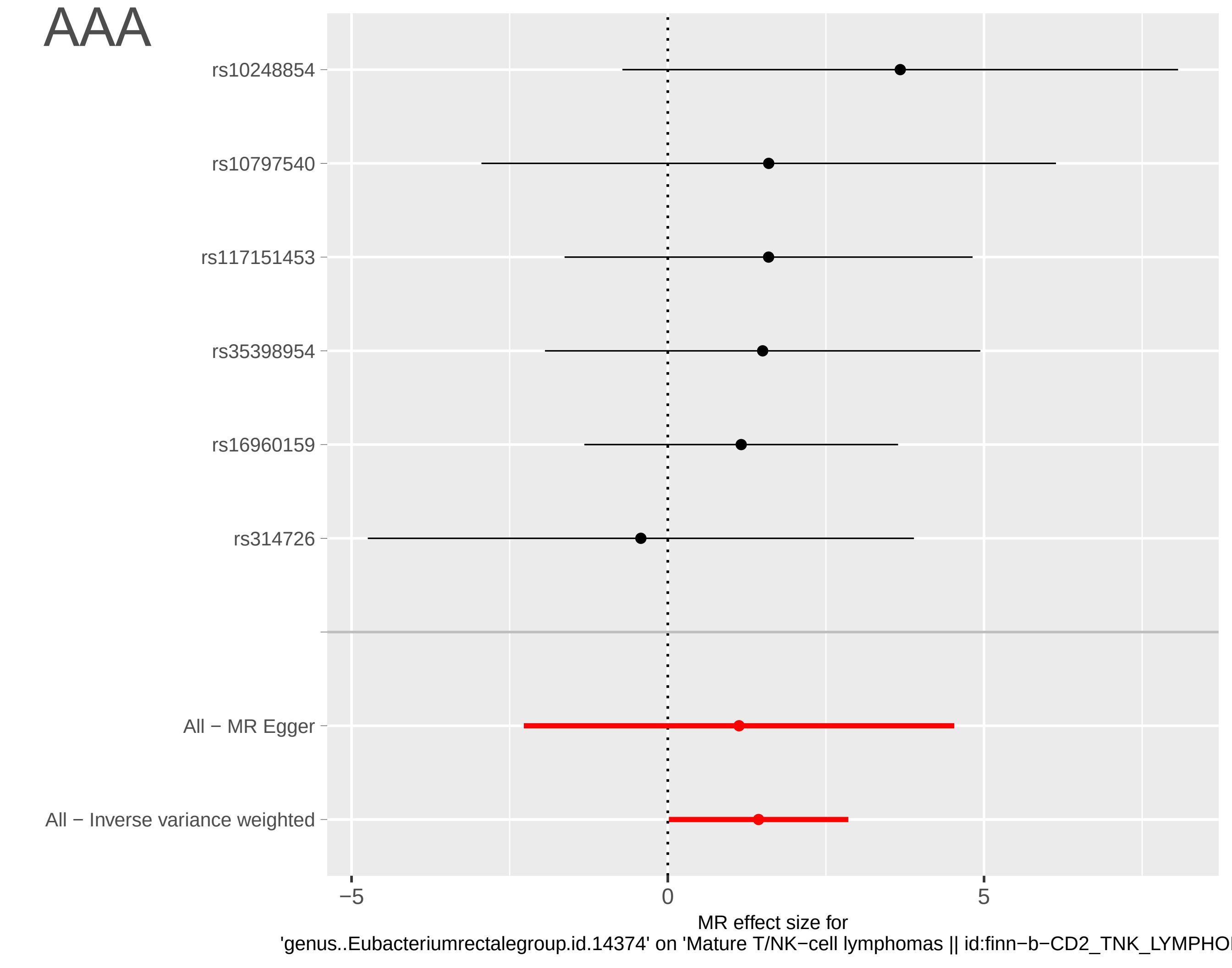
AAA
rs10248854
rs10797540
rs117151453
rs35398954
rs16960159
rs314726
All − MR Egger
All − Inverse variance weighted
−5
0
5
MR effect size for
'genus..Eubacteriumrectalegroup.id.14374' on 'Mature T/NK−cell lymphomas || id:finn−b−CD2_TNK_LYMPHO

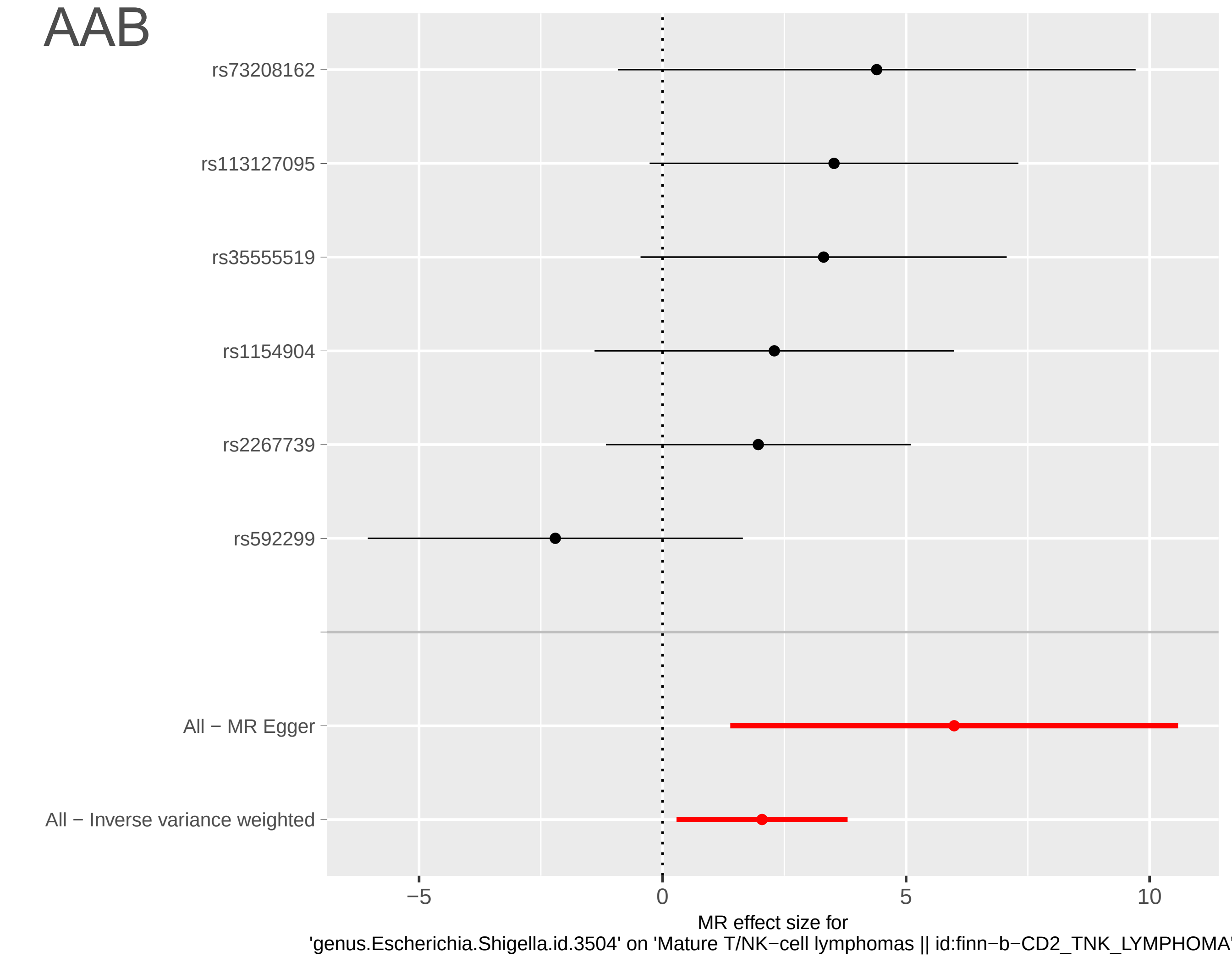
AAB
rs73208162
rs113127095
rs35555519
rs1154904
rs2267739
rs592299
All − MR Egger
All − Inverse variance weighted
−5
0
5
10
MR effect size for
'genus.Escherichia.Shigella.id.3504' on 'Mature T/NK−cell lymphomas || id:finn−b−CD2_TNK_LYMPHOMA'

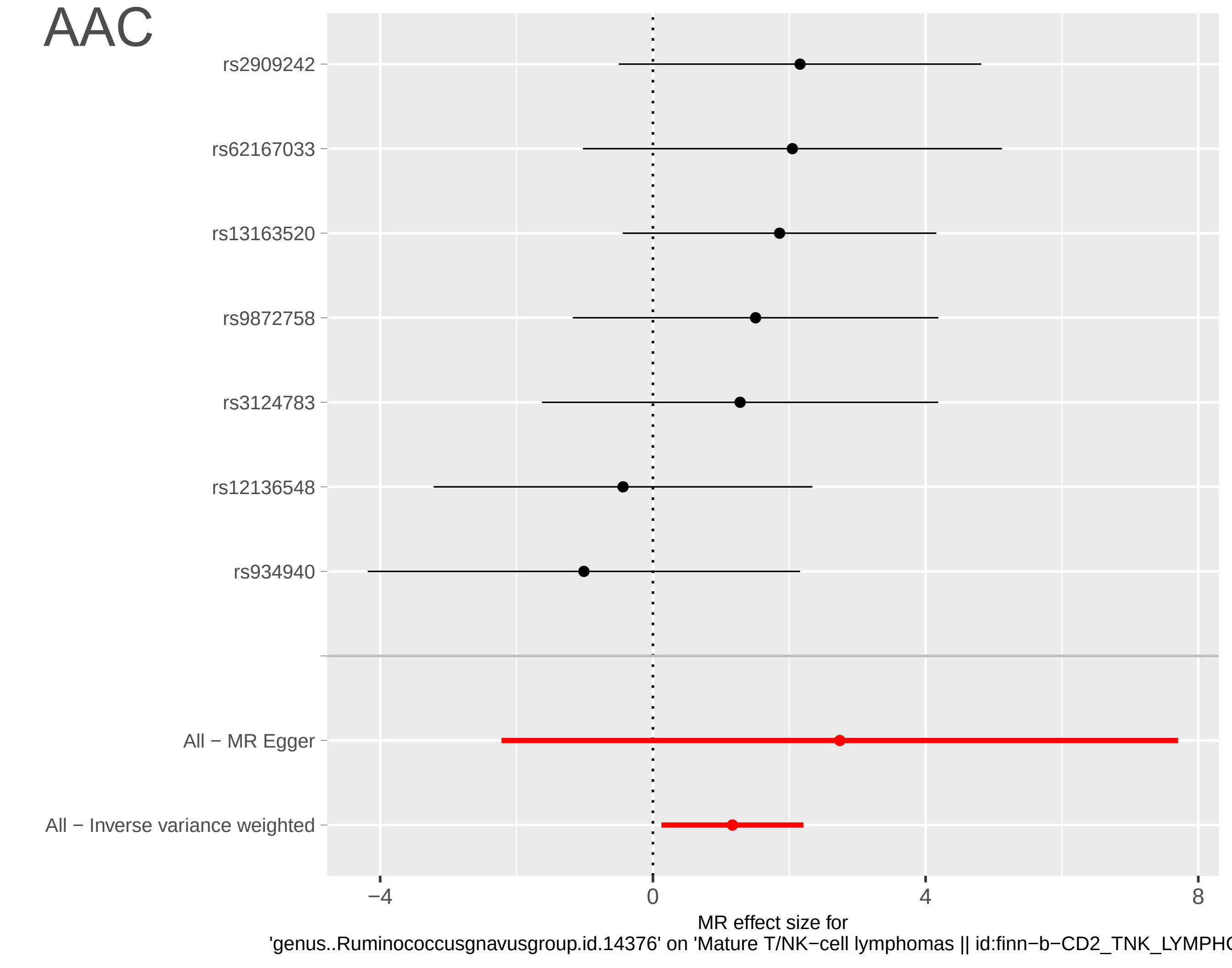

AAC
rs2909242
rs62167033
rs13163520
rs9872758
rs3124783
rs12136548
rs934940
All − MR Egger
All − Inverse variance weighted
−4
0
4
8
MR effect size for
'genus..Ruminococcusgnavusgroup.id.14376' on 'Mature T/NK−cell lymphomas || id:finn−b−CD2_TNK_LYMPHO

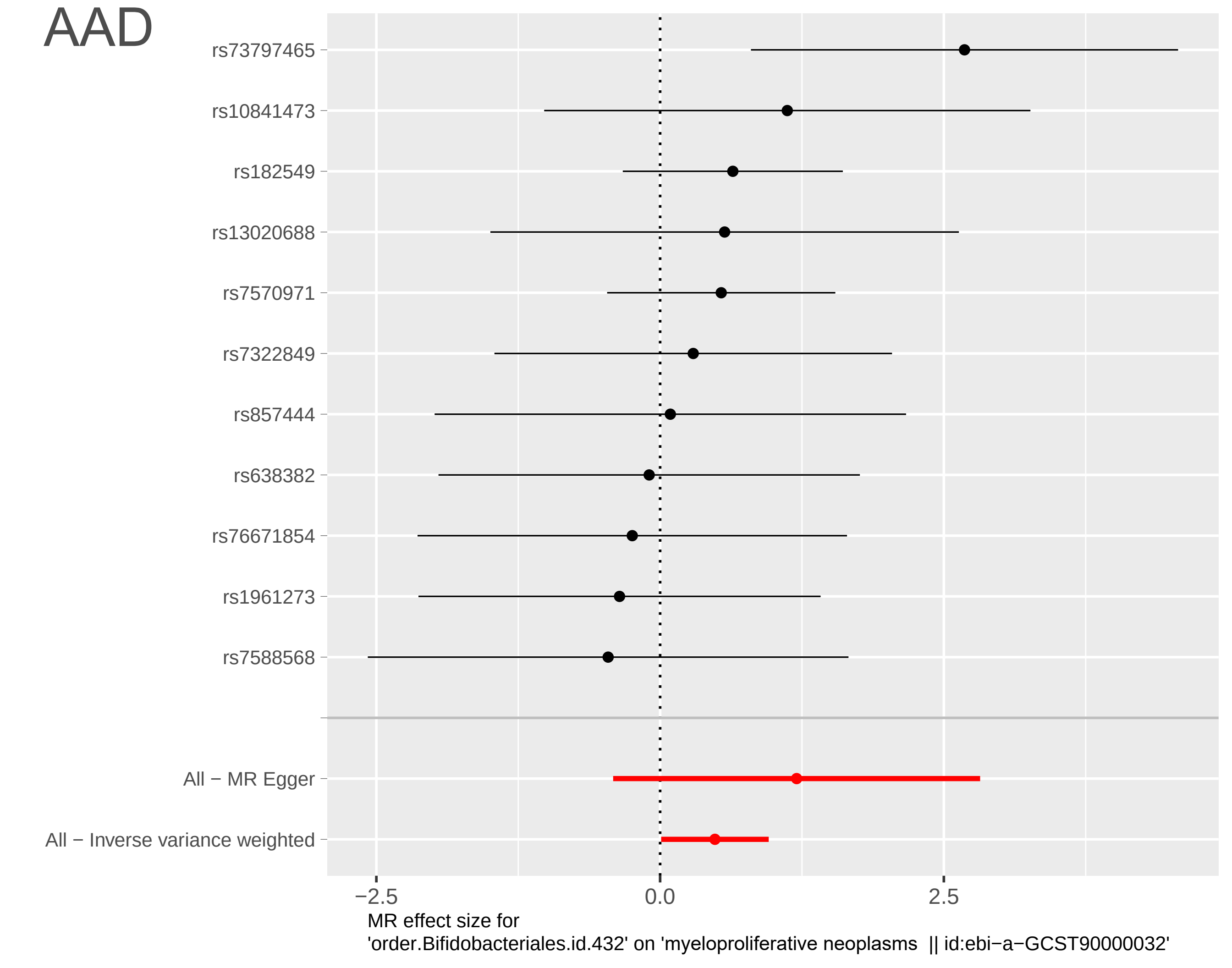
AAD
rs73797465
rs10841473
rs182549
rs13020688
rs7570971
rs7322849
rs857444
rs638382
rs76671854
rs1961273
rs7588568
All − MR Egger
All − Inverse variance weighted
−2.5
0.0
2.5
MR effect size for
'order.Bifidobacteriales.id.432' on 'myeloproliferative neoplasms || id:ebi−a−GCST90000032'

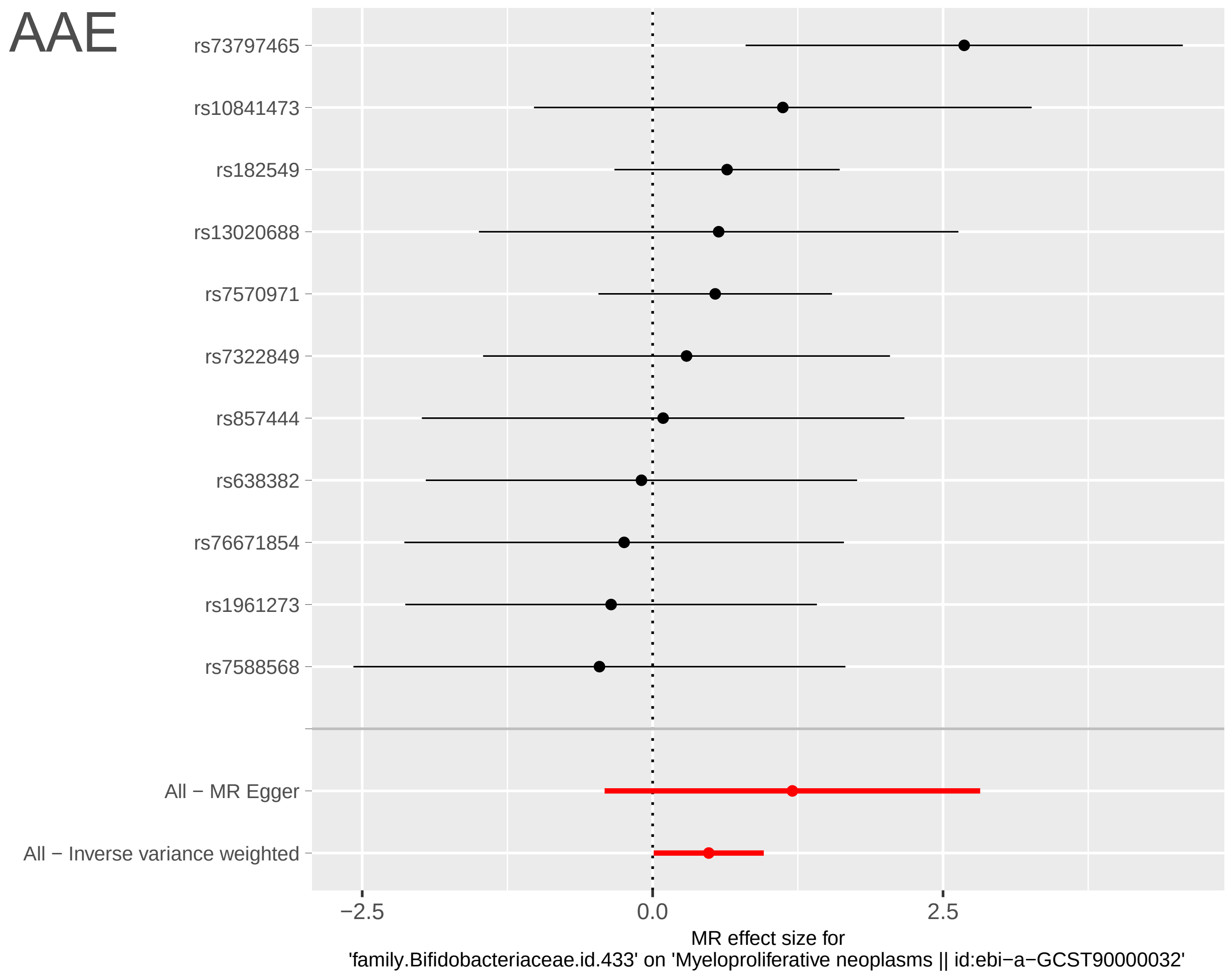

AAE
rs73797465
rs10841473
rs182549
rs13020688
rs7570971
rs7322849
rs857444
rs638382
rs76671854
rs1961273
rs7588568
All − MR Egger
All − Inverse variance weighted
−2.5
0.0
2.5
MR effect size for
'family.Bifidobacteriaceae.id.433' on 'Myeloproliferative neoplasms || id:ebi−a−GCST90000032'

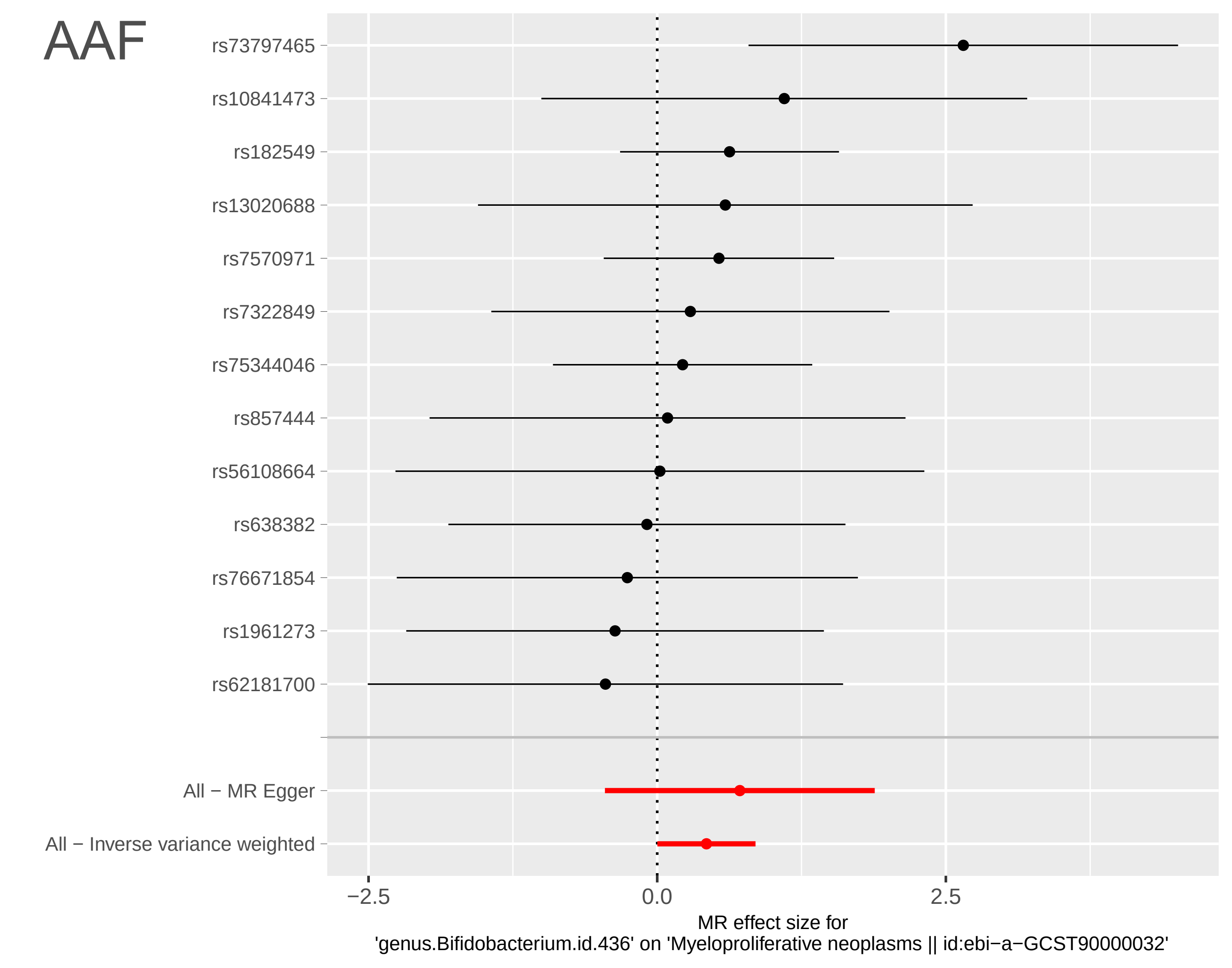
AAF
rs73797465
rs10841473
rs182549
rs13020688
rs7570971
rs7322849
rs75344046
rs857444
rs56108664
rs638382
rs76671854
rs1961273
rs62181700
All − MR Egger
All − Inverse variance weighted
−2.5
0.0
2.5
MR effect size for
'genus.Bifidobacterium.id.436' on 'Myeloproliferative neoplasms || id:ebi−a−GCST90000032'

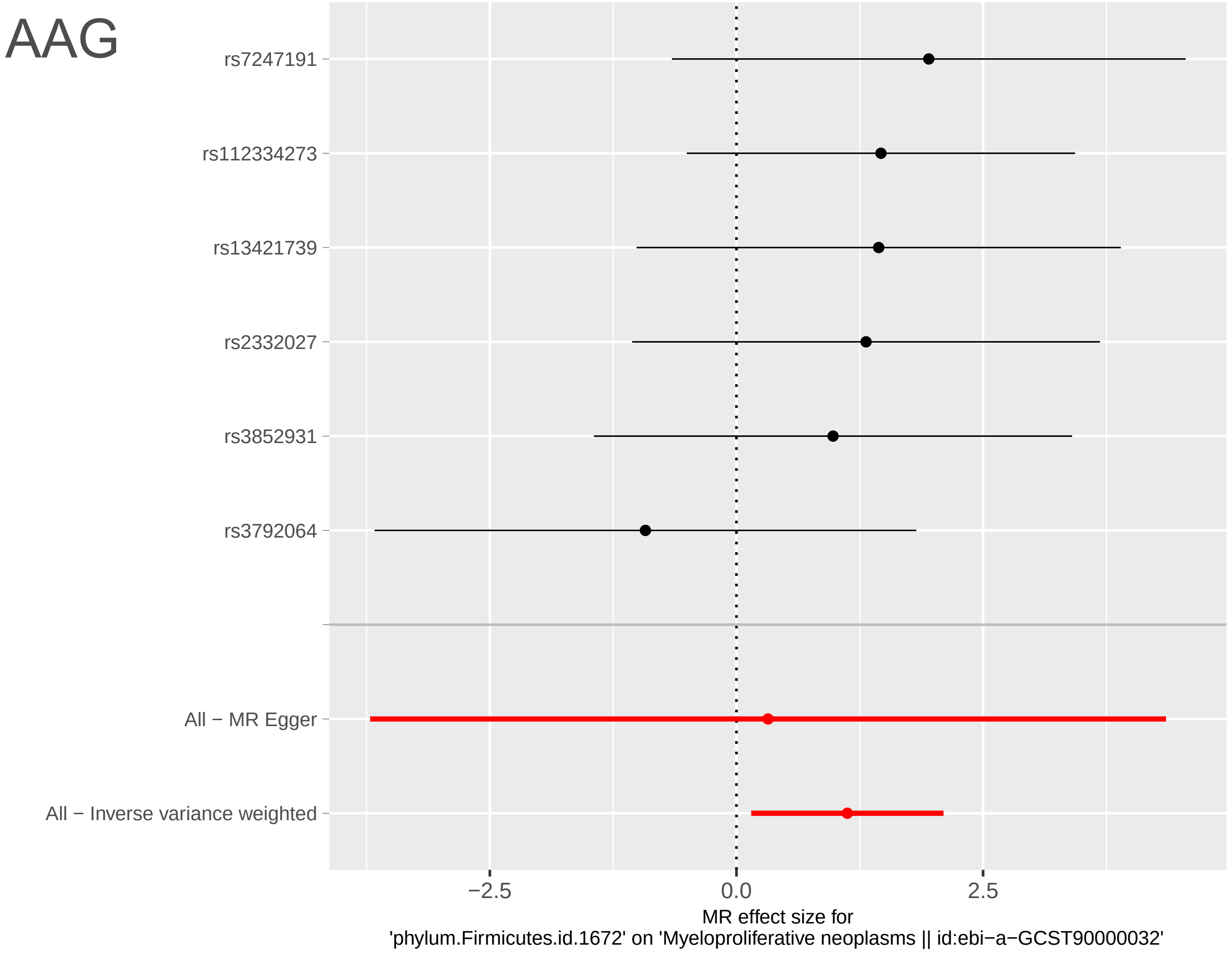
AAG
rs7247191
rs112334273
rs13421739
rs2332027
rs3852931
rs3792064
All − MR Egger
All − Inverse variance weighted
−2.5
0.0
2.5
MR effect size for
'phylum.Firmicutes.id.1672' on 'Myeloproliferative neoplasms || id:ebi−a−GCST90000032'

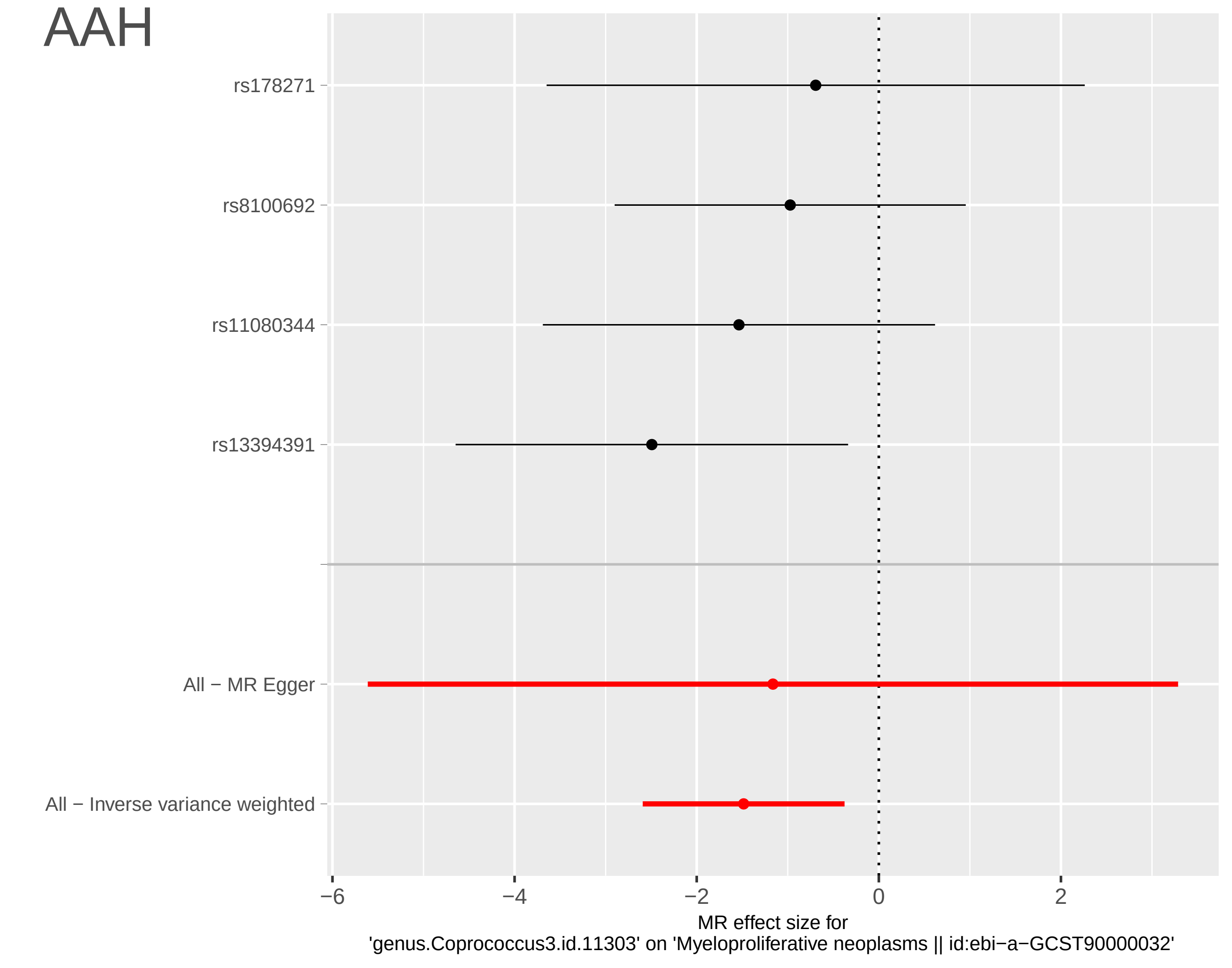
AAH
rs178271
rs8100692
rs11080344
rs13394391
All − MR Egger
All − Inverse variance weighted
−6
−4
−2
0
2
MR effect size for
'genus.Coprococcus3.id.11303' on 'Myeloproliferative neoplasms || id:ebi−a−GCST90000032'

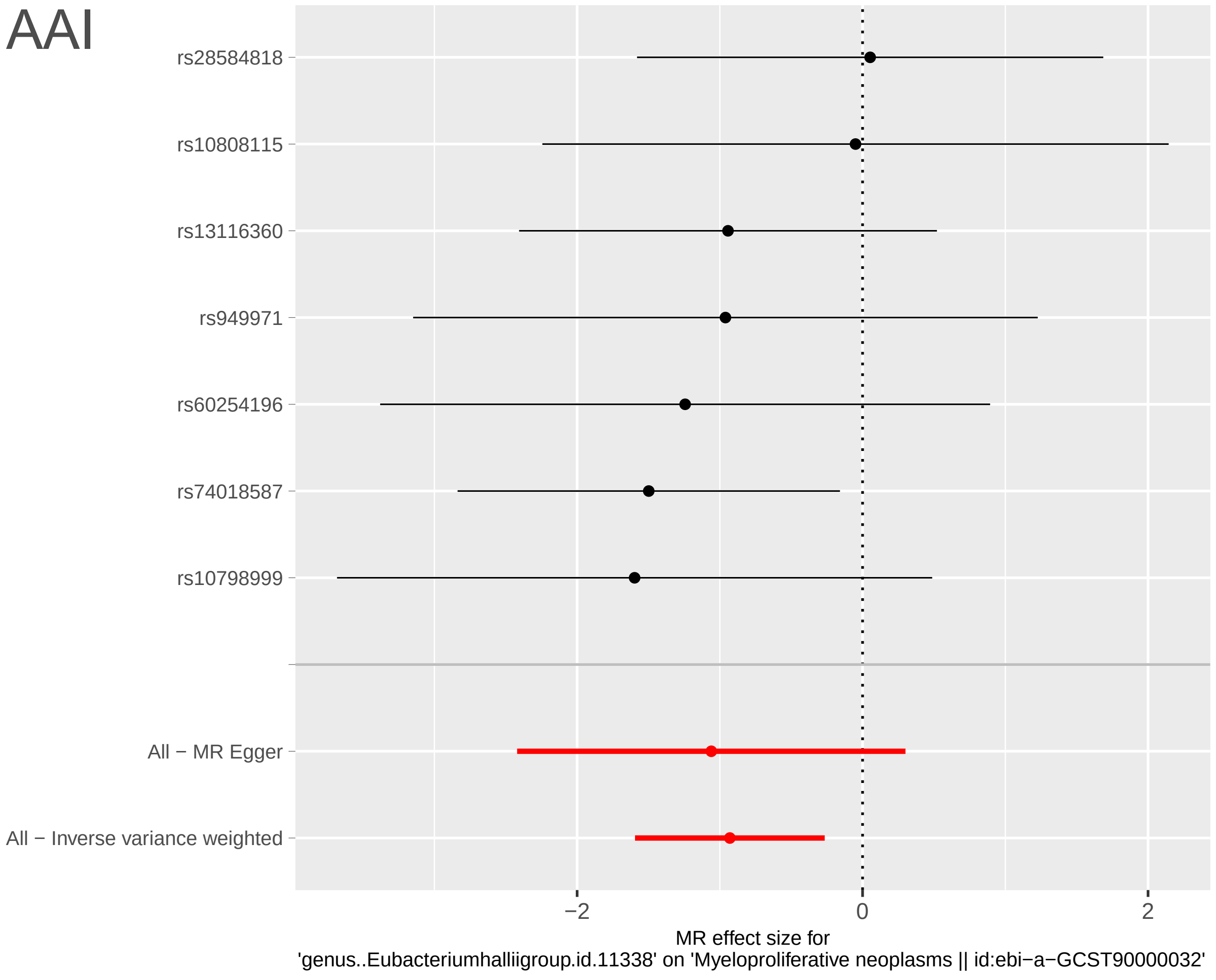
AAI
rs28584818
rs10808115
rs13116360
rs949971
rs60254196
rs74018587
rs10798999
All − MR Egger
All − Inverse variance weighted
−2
0
2
MR effect size for
'genus..Eubacteriumhalliigroup.id.11338' on 'Myeloproliferative neoplasms || id:ebi−a−GCST90000032'

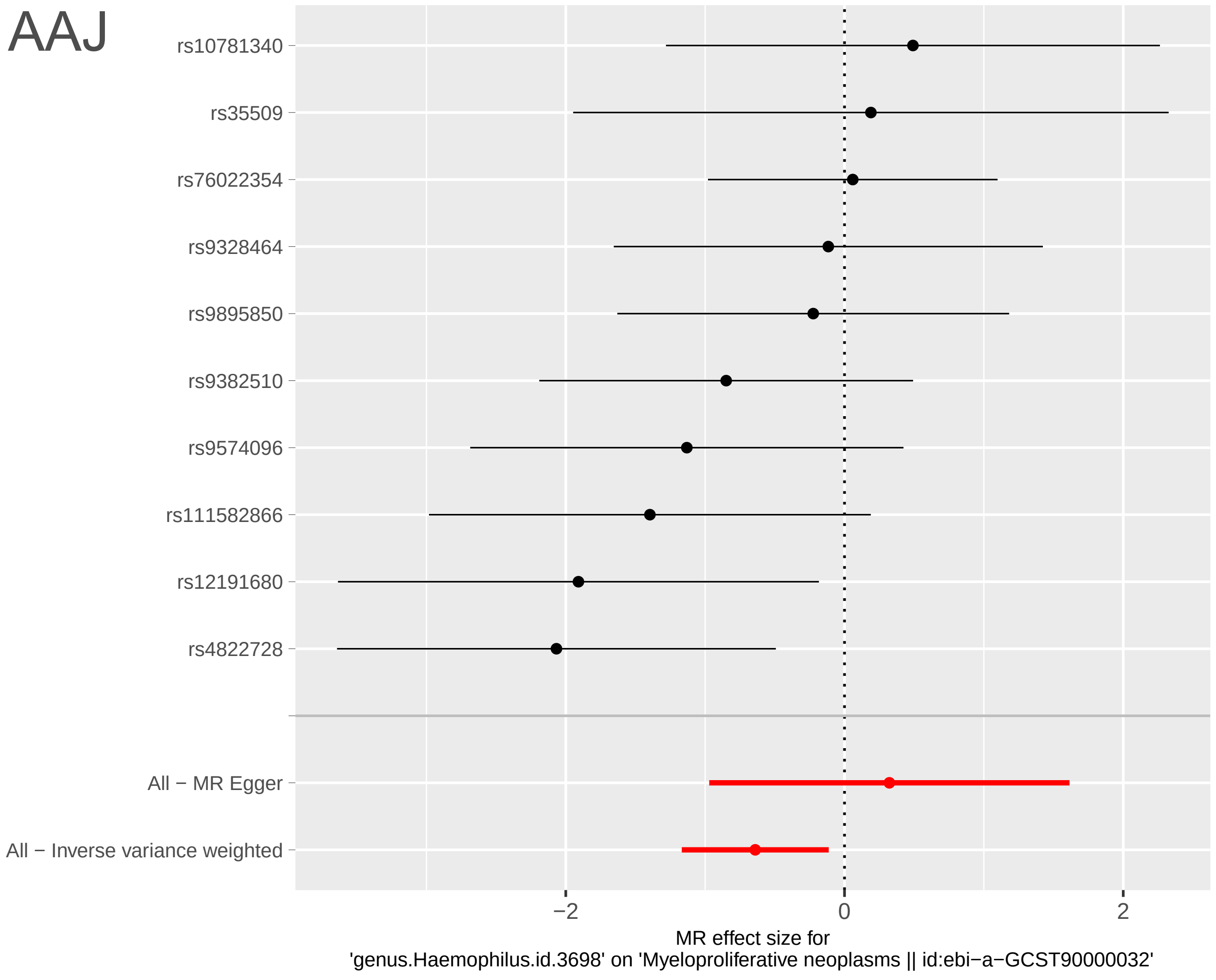
AAJ
rs10781340
rs35509
rs76022354
rs9328464
rs9895850
rs9382510
rs9574096
rs111582866
rs12191680
rs4822728
All − MR Egger
All − Inverse variance weighted
−2
0
2
MR effect size for
'genus.Haemophilus.id.3698' on 'Myeloproliferative neoplasms || id:ebi−a−GCST90000032'

Supplementary figure 1.Sensitivity analysis of the impact of gut microbiota on hematologic malignancies using MR. (forest plot)

A.Forest plot between genus.FamilyXIIIUCG001 and lymphoid leukemia

B.Forest plot between family.Peptococcaceae and lymphoid leukemia**.**

C.Forest plot between family.Desulfovibrionaceae and lymphoid leukemia

D.Forest plot between genus..Clostridiuminnocuumgroup and lymphoid leukemia.

E.Forest plot between genus.Holdemania and lymphoid leukemia.

F.Forest plot between family.Prevotellaceae and lymphoid leukemia.

G.Forest plot between genus.Ruminiclostridium9. and lymphoid leukemia.

H.Forest plot between genus.Methanobrevibacter and lymphoid leukemia.

I.Forest plot between order.Desulfovibrionales. and lymphoid leukemia.

J.Forest plot between genus.Coprococcus3 and lymphoid leukemia.

K.Forest plot between genus.Ruminiclostridium6 and lymphoid leukemia.

L.Forest plot between class.Negativicutes and lymphoid leukemia.

M.Forest plot between order.Selenomonadales. and lymphoid leukemia.

N.Forest plot between family.Lactobacillaceae. and lymphoid leukemia.

O.Forest plot between class.Methanobacteria and lymphoid leukemia.

P.Forest plot between family.Methanobacteriaceae and lymphoid leukemia.

Q.Forest plot between order.Methanobacteriales and lymphoid leukemia.

R.Forest plot between phylum.Cyanobacteria and lymphoid leukemia**.**

S.Forest plot between order.Coriobacteriales and myeloid leukemia**.**

T.Forest plot between family.Coriobacteriaceae. and myeloid leukemia**.**

U.Forest plot between genus.LachnospiraceaeUCG008 and myeloid leukemia.

V.Forest plot between class.Coriobacteriia. and myeloid leukemia**.**

W.Forest plot between genus.Turicibacter and myeloid leukemia**.**

X.Forest plot between genus.Slackia. and myeloid leukemia.

Y.Forest plot between class.Gammaproteobacteria and myeloid leukemia.

Z.Forest plot between genus.Prevotella9 and myeloid leukemia.

AA. Forest plot between genus.Dorea and myeloid leukemia.

AB.Forest plot between genus.Peptococcus. and Hodgkin lymphoma

AC.Forest plot between class.Gammaproteobacteria and Hodgkin lymphoma.

AD.Forest plot between genus.RuminococcaceaeUCG014 and malignant plasma cell tumor**.**

AE.Forest plot between genus.LachnospiraceaeUCG010. and malignant plasma cell tumor.

AF.Forest plot between class.Lentisphaeria and malignant plasma cell tumor.

AG.Forest plot between genus.Lactococcus. and malignant plasma cell tumor.

AH.Forest plot between order.Victivallales. and malignant plasma cell tumor.

AI.Forest plot between genus.Romboutsia and malignant plasma cell tumor.

AJ.Forest plot between genus.Dorea and malignant plasma cell tumor.

AK.Forest plot between genus.RikenellaceaeRC9gutgroup and malignant plasma cell tumor**.**

AL. Forest plot between genus.RuminococcaceaeUCG005 and follicular lymphoma.

AM.Forest plot between genus.Adlercreutzia and follicular lymphoma.

AN.Forest plot between order.MollicutesRF9 and follicular lymphoma.

AO.Forest plot between family.Alcaligenaceae and follicular lymphoma.

AP.Forest plot between class.Clostridia and follicular lymphoma.

AQ. Forest plot between genus.Sutterella and follicular lymphoma.

AR.Forest plot between genus.Phascolarctobacterium and follicular lymphoma.

AS.Forest plot between genus..Eubacteriumcoprostanoligenesgroup and diffuse large B-cell lymphoma.

AT.Forest plot between class.Alphaproteobacteria. and diffuse large B-cell lymphoma.

AU.Forest plot between phylum.Cyanobacter and diffuse large B-cell lymphoma.

AV.Forest plot between genus.Erysipelatoclostridium and diffuse large B-cell lymphoma.

AW.Forest plot between family.Rhodospirillaceae. and mature T/NK-cell lymphomas.

AX.Forest plot between genus.Anaerostipes and mature T/NK-cell lymphomas.

AY.Forest plot between genus.Erysipelatoclostridium. and mature T/NK-cell lymphomas.

AZ.Forest plot between genus.LachnospiraceaeUCG001.. and mature T/NK-cell lymphomas.

AAA.Forest plot between genus..Eubacteriumrectalegroup. and mature T/NK-cell lymphomas.

AAB. Forest plot between genus.Escherichia.Shigella. and mature T/NK-cell lymphomas.

AAC.Forest plot between genus..Ruminococcusgnavusgroup and mature T/NK-cell lymphomas.

AAD. Forest plot between order.Bifidobacteriales. and myeloproliferative neoplasms.

AAE. Forest plot between family.Bifidobacteriaceae.. and myeloproliferative neoplasms.

AAF. Forest plot between genus.Bifidobacterium. and myeloproliferative neoplasms.

AAG.Forest plot between phylum.Firmicutes. and myeloproliferative neoplasms.

AAH.Forest plot between genus.Coprococcus3. and myeloproliferative neoplasms.

AAI.Forest plot between genus..Eubacteriumhalliigroup. and myeloproliferative neoplasms.

AAJ.Forest plot between genus.Haemophilus. and myeloproliferative neoplasms

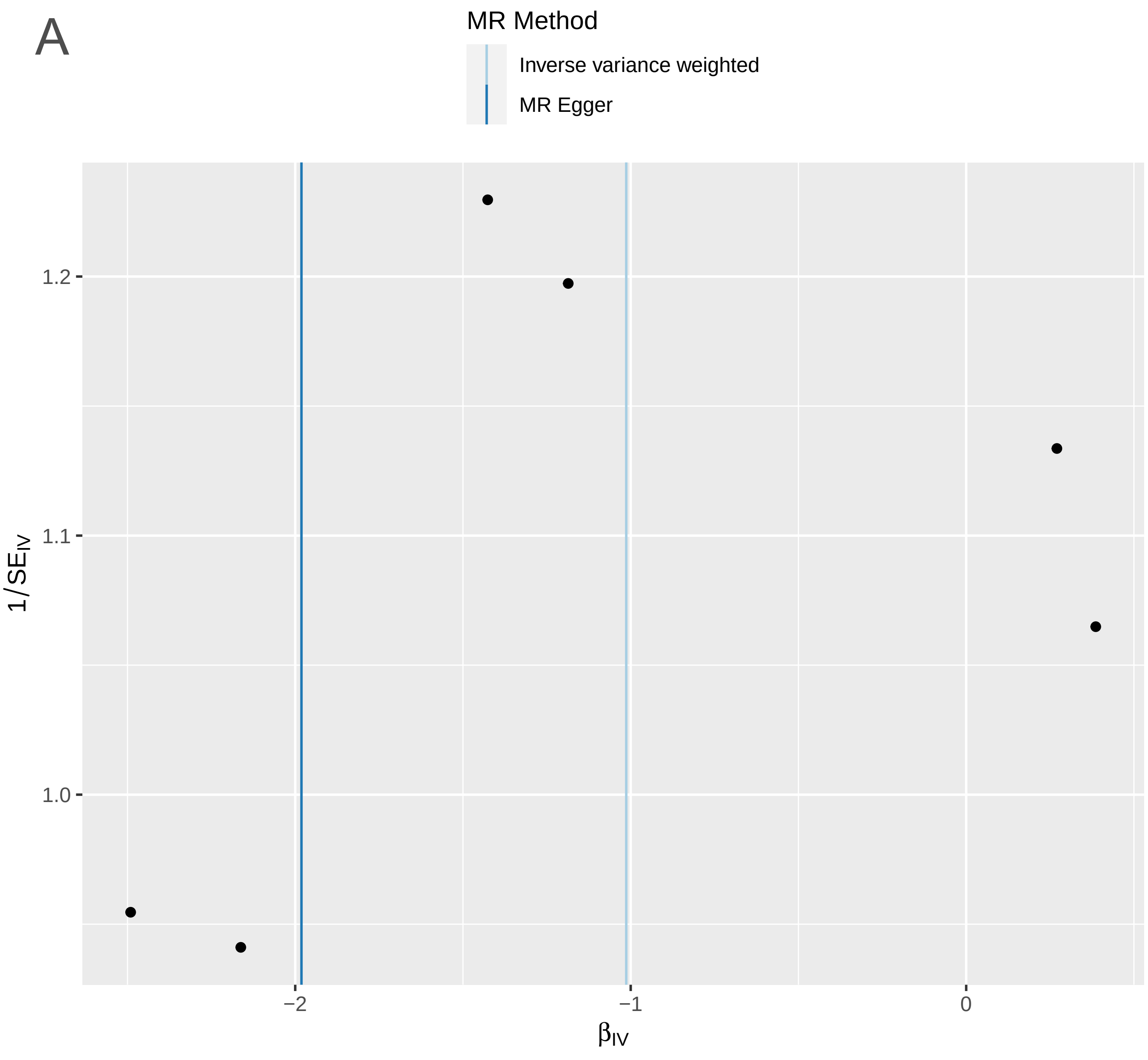

A
MR Method
Inverse variance weighted
MR Egger
1.2
1.1
1.0
1/SE_IV
−2
−1
0
β_IV

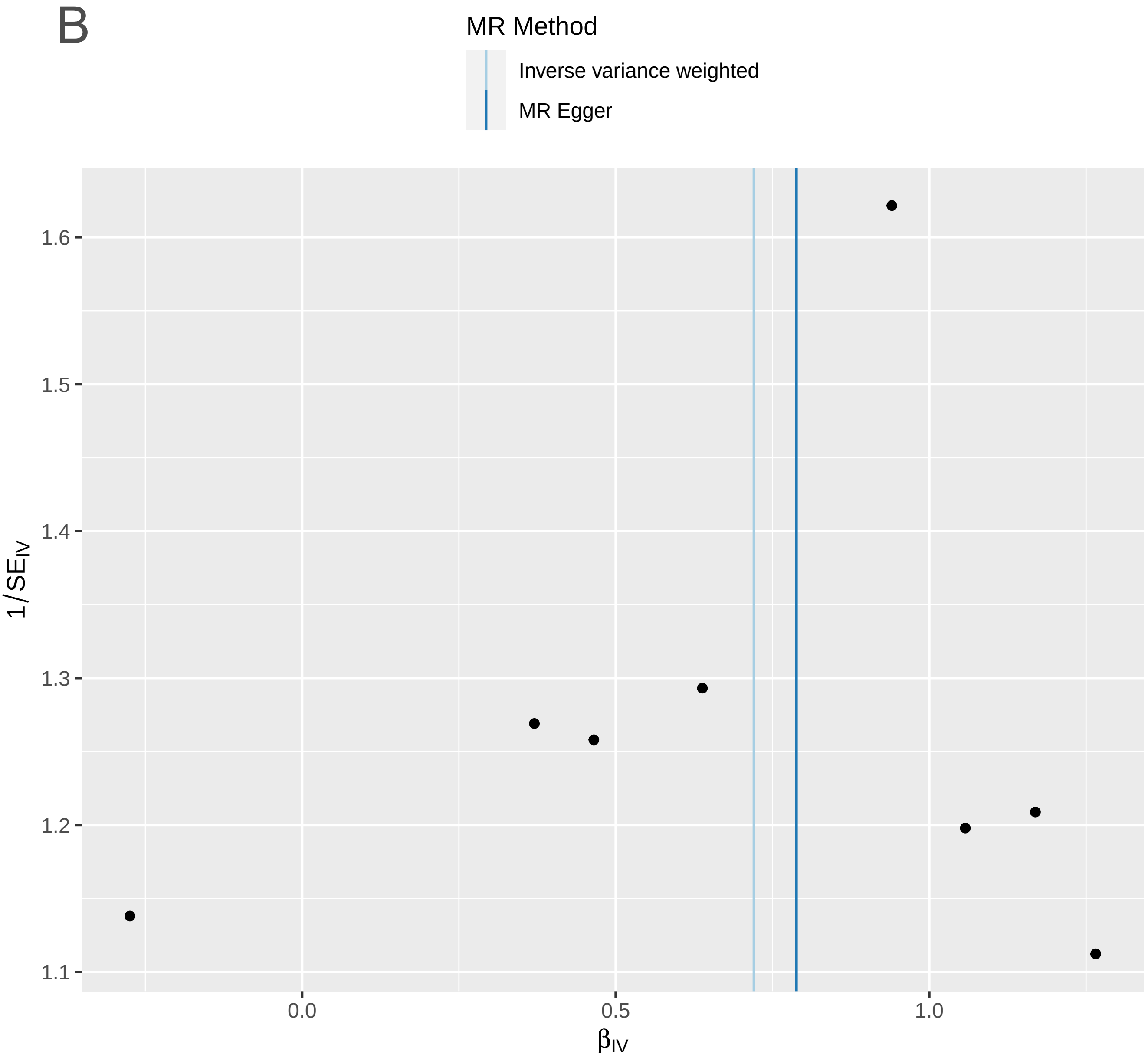

B
MR Method
Inverse variance weighted
MR Egger
1.6
1.5
1.4
1.3
1.2
1.1
1/SE_IV
0.0
0.5
1.0
β_IV

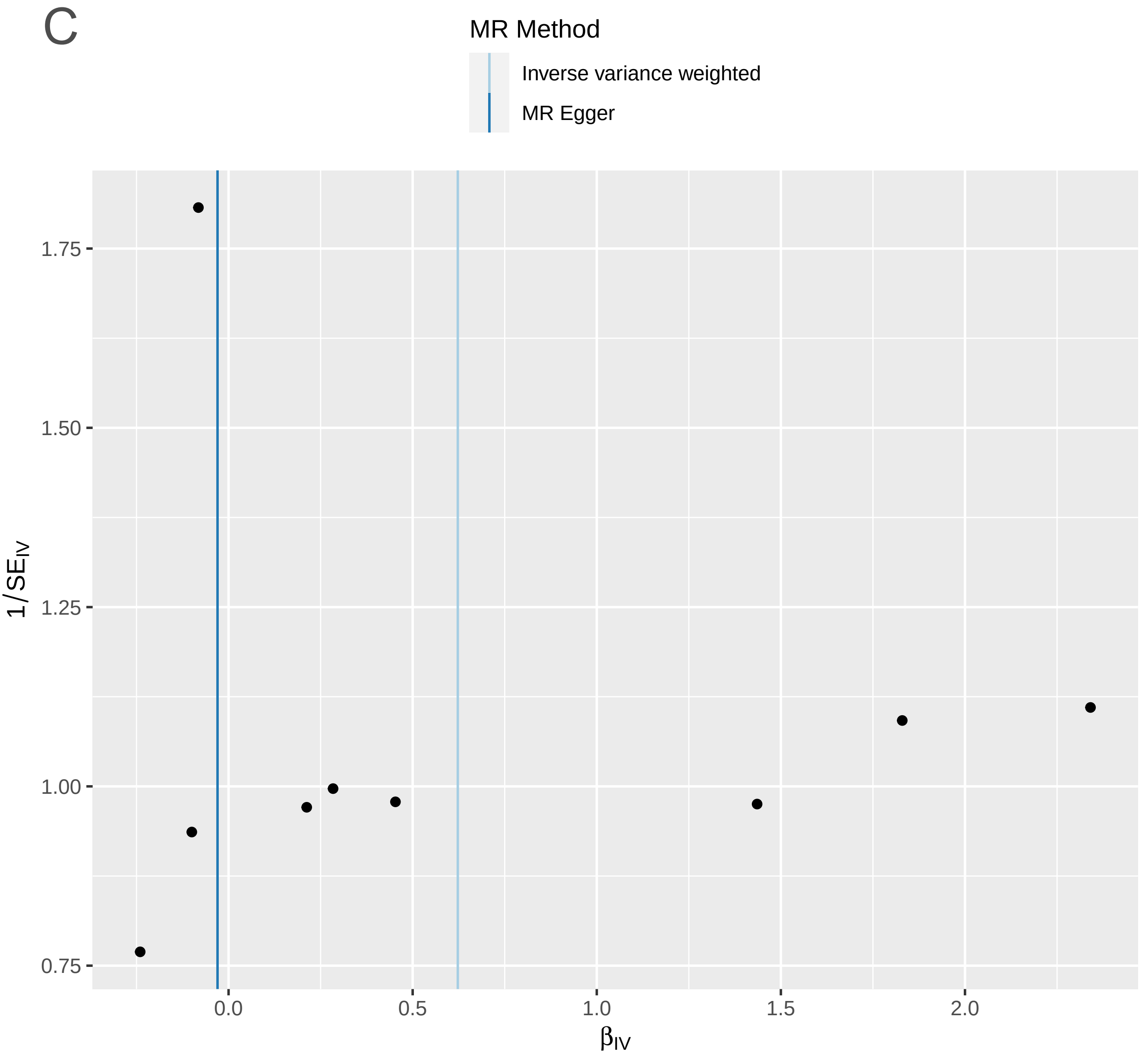
C
MR Method
Inverse variance weighted
MR Egger
1.75
1.50
1.25
1.00
0.75
1/SE$_{IV}$
0.0
0.5
1.0
1.5
2.0
β$_{IV}$

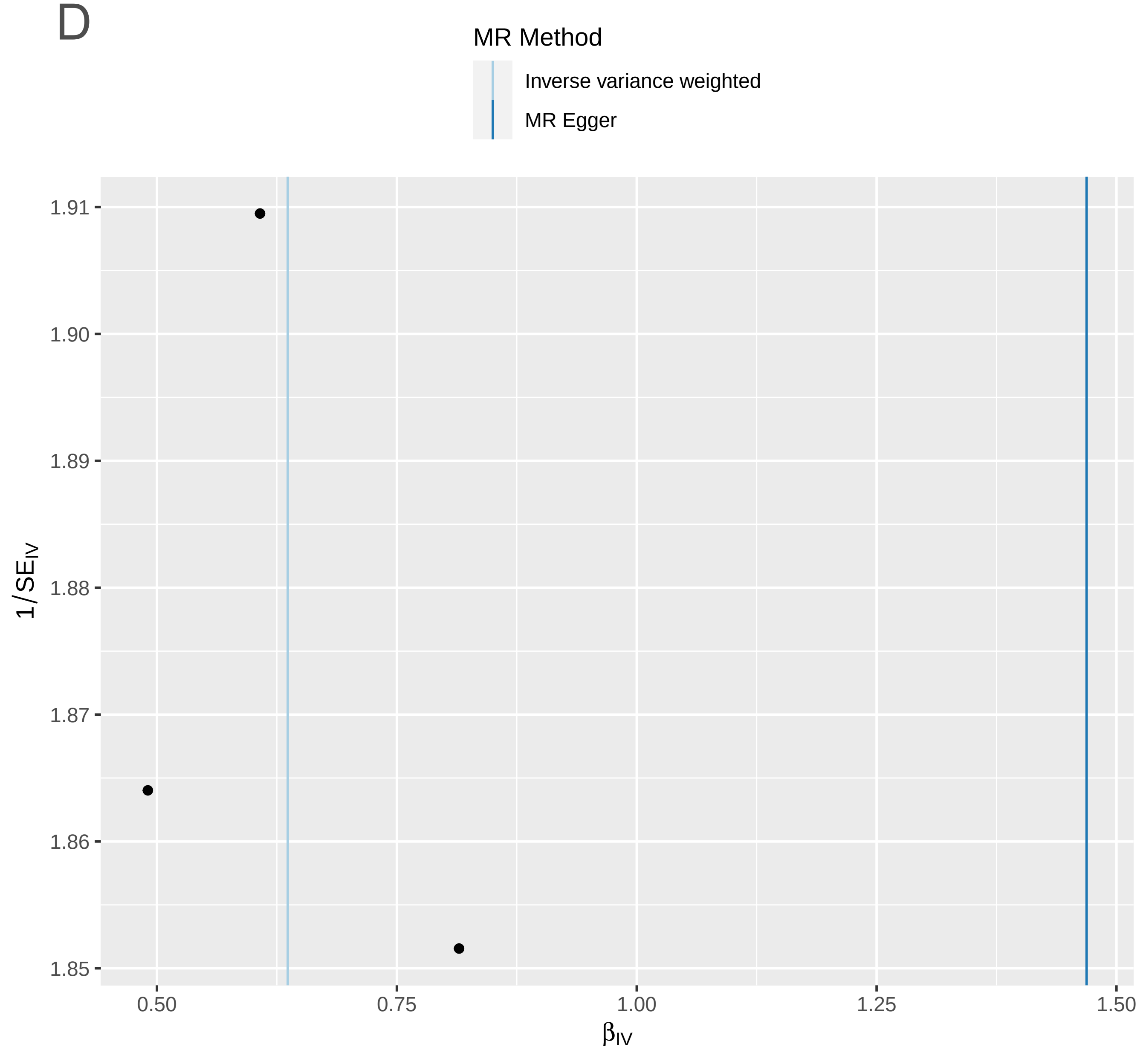

D
MR Method
Inverse variance weighted
MR Egger
1.91
1.90
1.89
1.88
1.87
1.86
1.85
1/SE_IV
0.50
0.75
1.00
1.25
1.50
β_IV

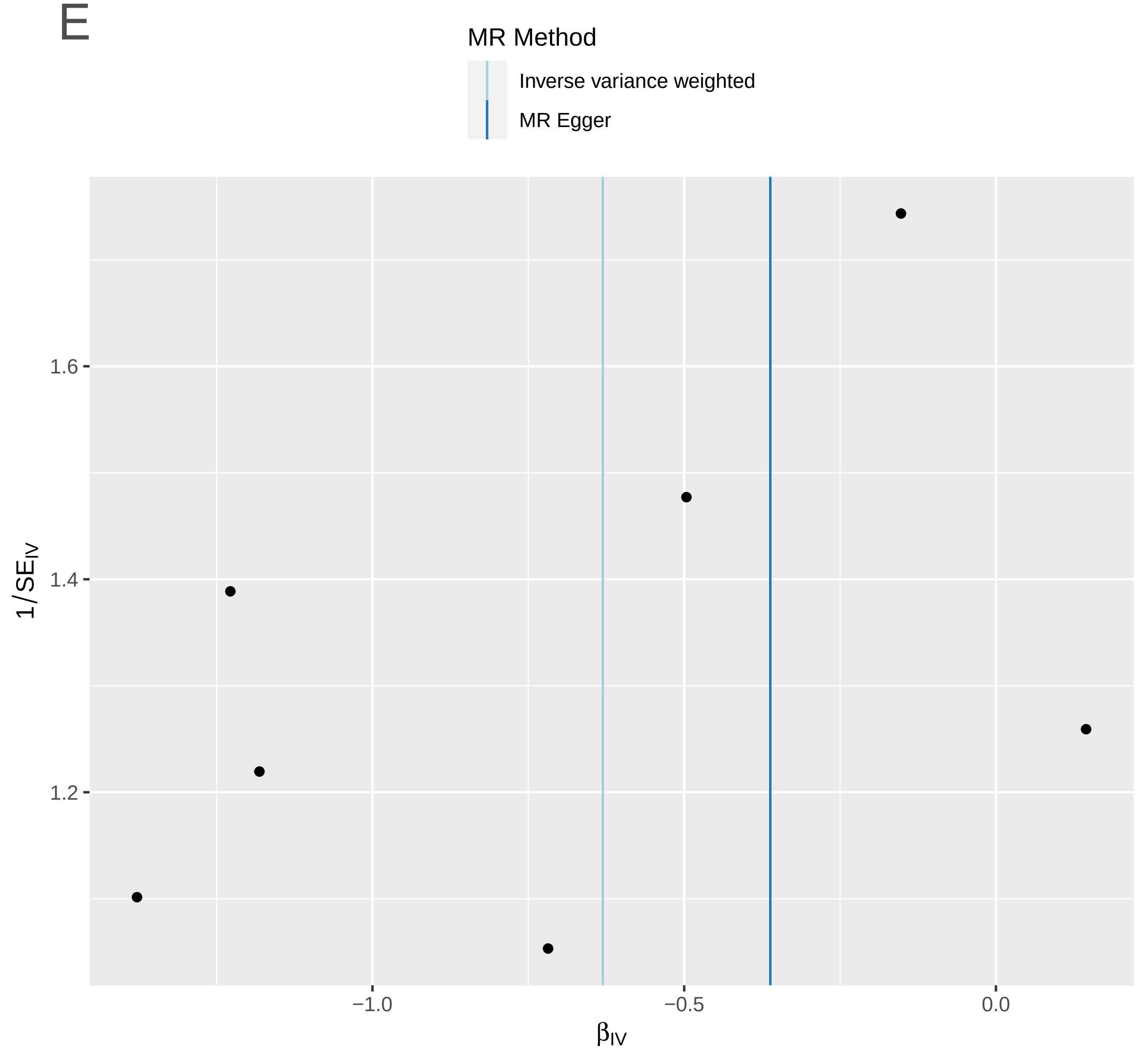

E
MR Method
Inverse variance weighted
MR Egger
1.6
1.4
1.2
1/SE_IV
−1.0
−0.5
0.0
β_IV

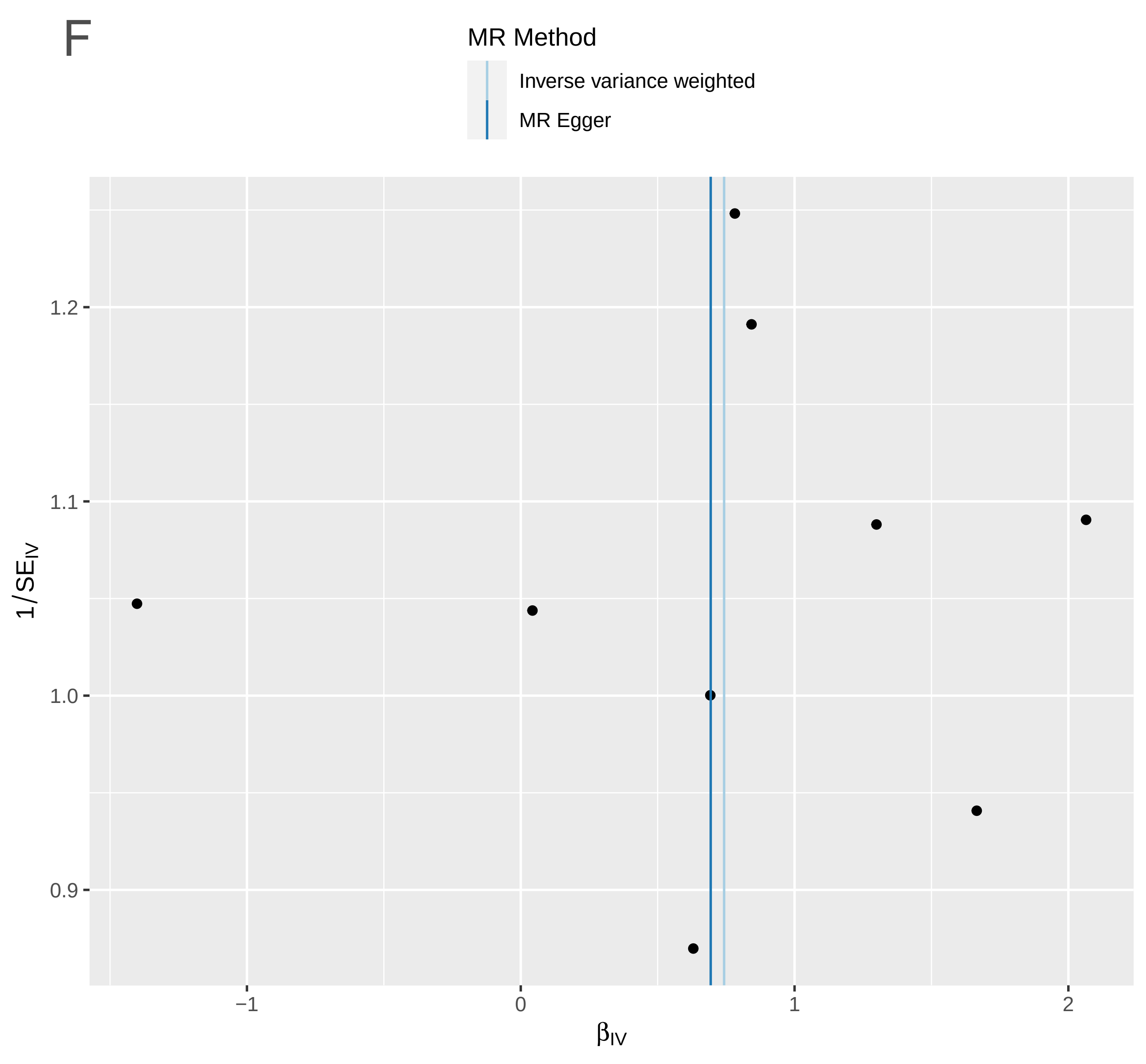
F
MR Method
Inverse variance weighted
MR Egger
1.2
1.1
1.0
0.9
−1
0
1
2
$1/SE_{IV}$
β_{IV}

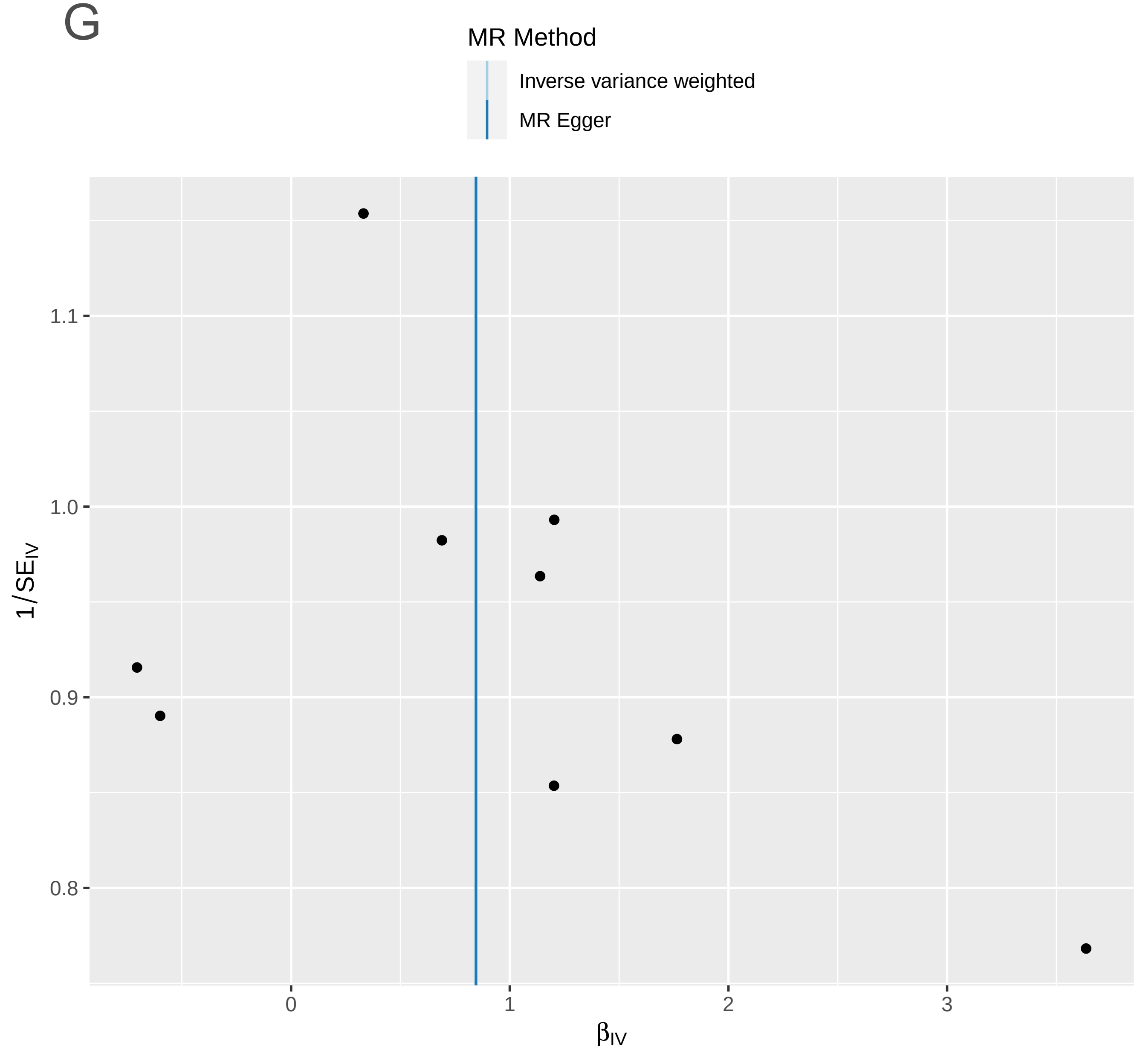
G
MR Method
Inverse variance weighted
MR Egger
1.1
1.0
0.9
0.8
$1/SE_{IV}$
0
1
2
3
β_{IV}

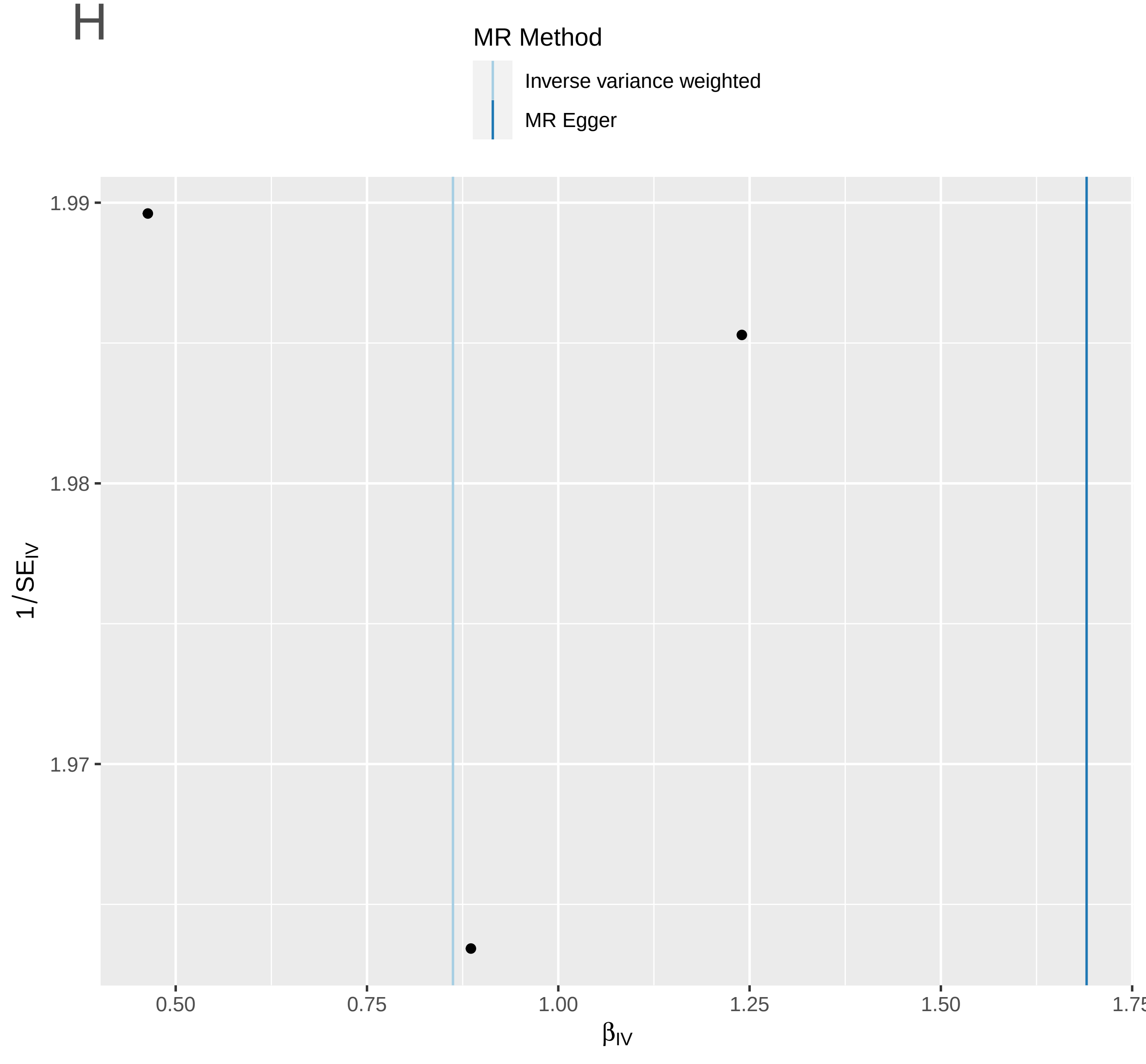

H
MR Method
Inverse variance weighted
MR Egger
1.99
1.98
1.97
$1/SE_{IV}$
0.50
0.75
1.00
1.25
1.50
1.75
β_{IV}

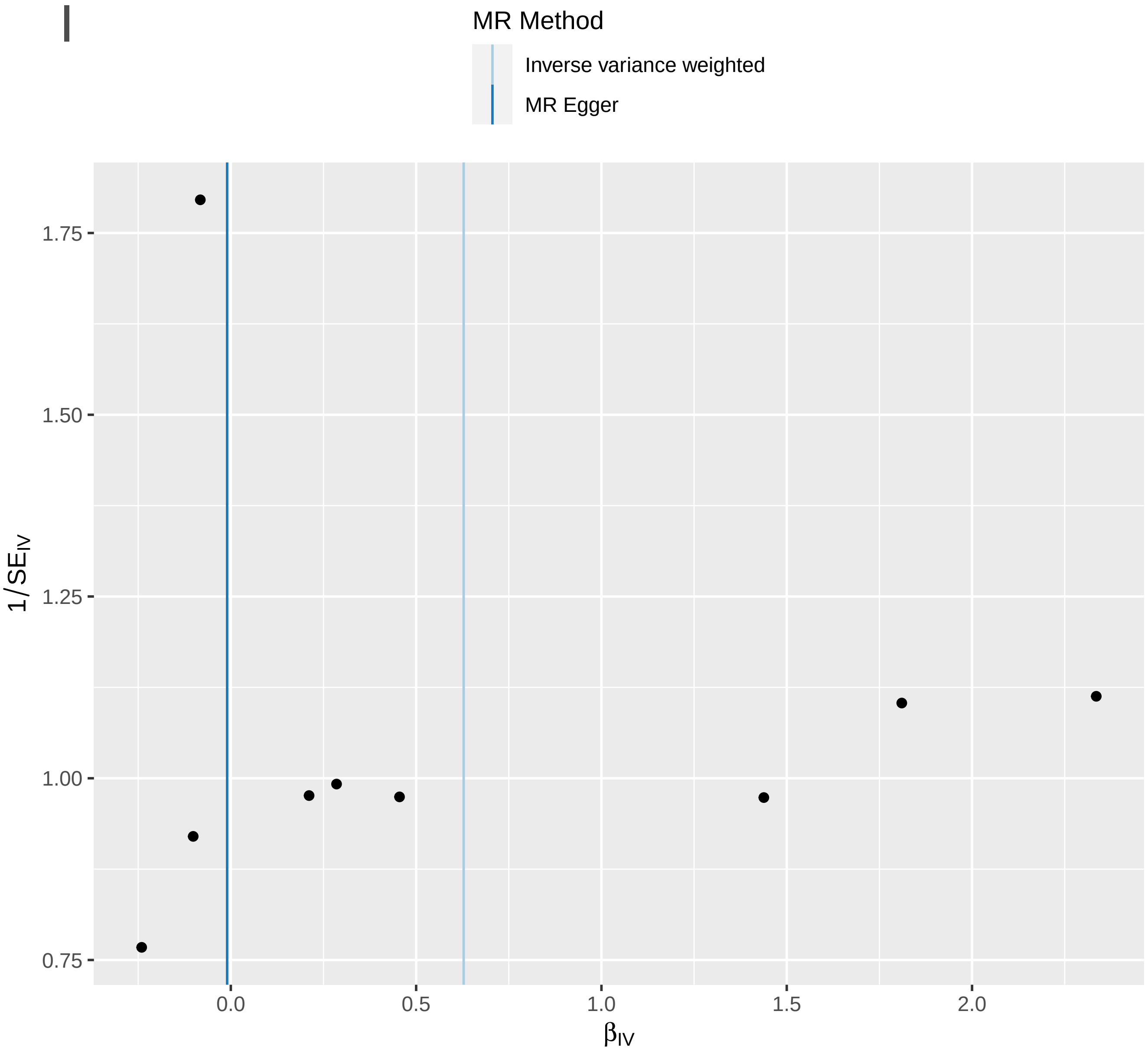

MR Method
Inverse variance weighted
MR Egger
1.75
1.50
1.25
1.00
0.75
$1/SE_{IV}$
0.0
0.5
1.0
1.5
2.0
β_{IV}

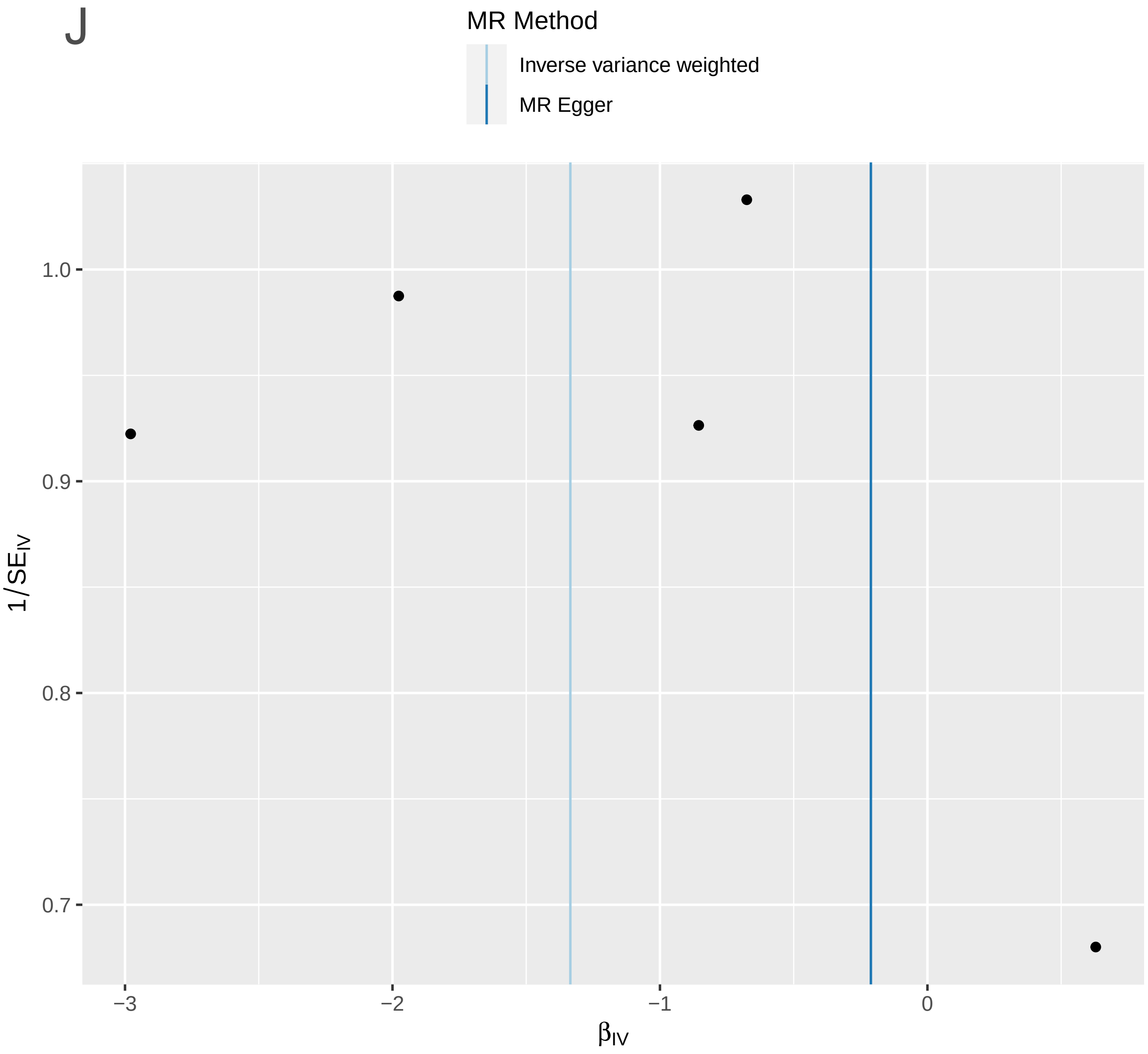
J
MR Method
Inverse variance weighted
MR Egger
1.0
0.9
0.8
0.7
1/SE_IV
−3
−2
−1
0
β_IV

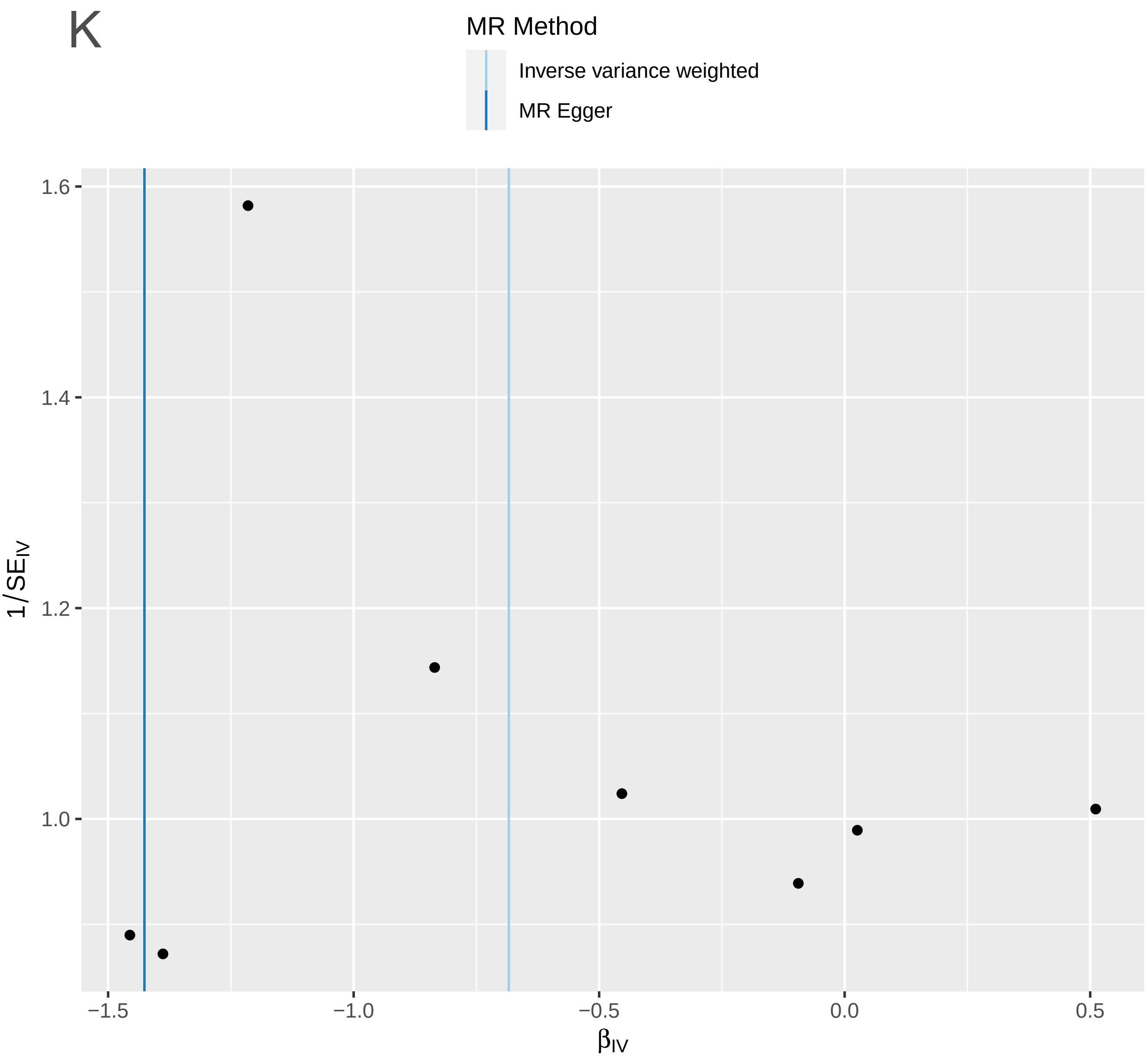
K
MR Method
Inverse variance weighted
MR Egger
1.6
1.4
1.2
1.0
1/SE_IV
−1.5
−1.0
−0.5
0.0
0.5
β_IV

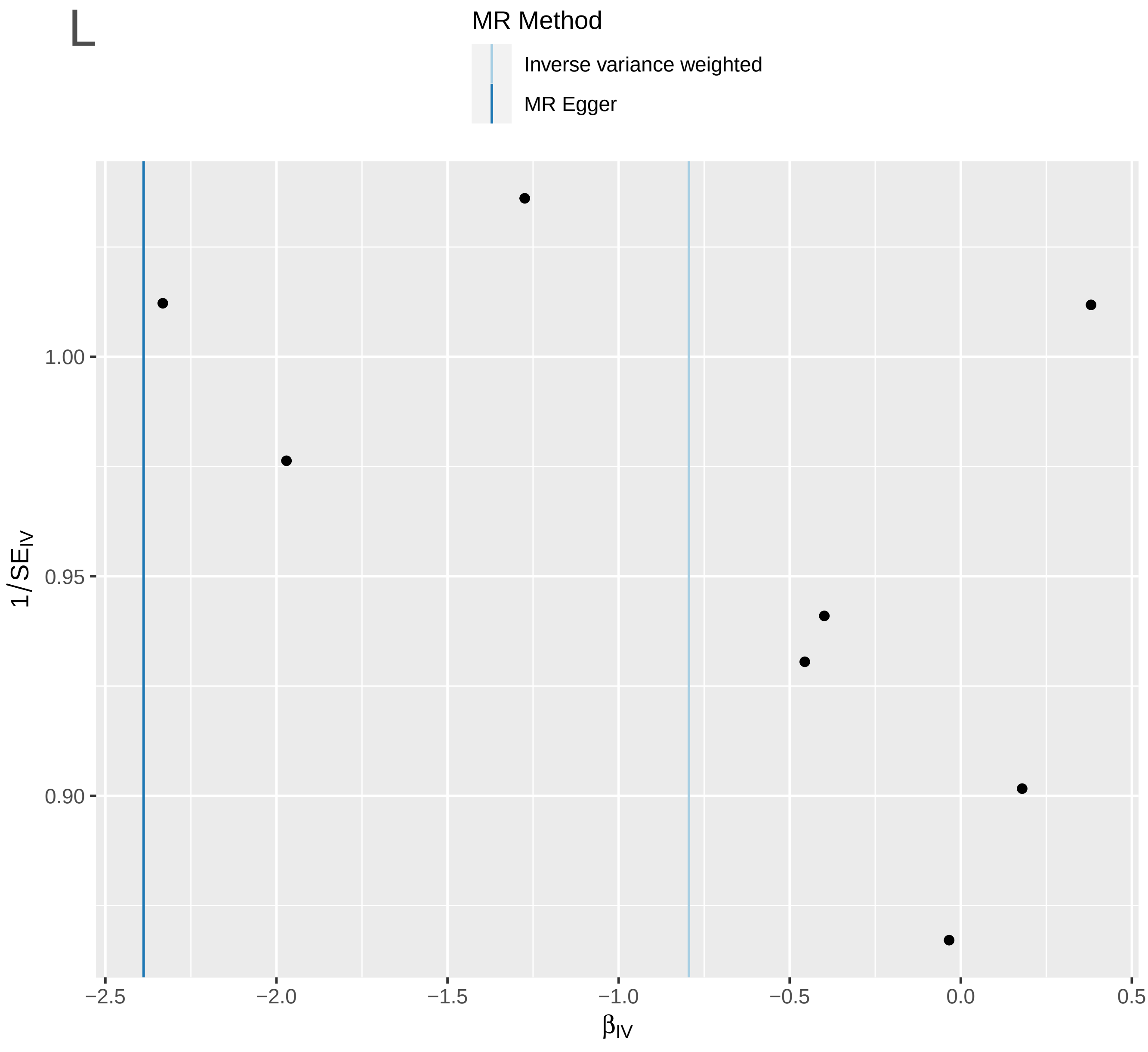
L
MR Method
Inverse variance weighted
MR Egger
1/SE_IV
1.00
0.95
0.90
−2.5
−2.0
−1.5
−1.0
−0.5
0.0
0.5
β_IV

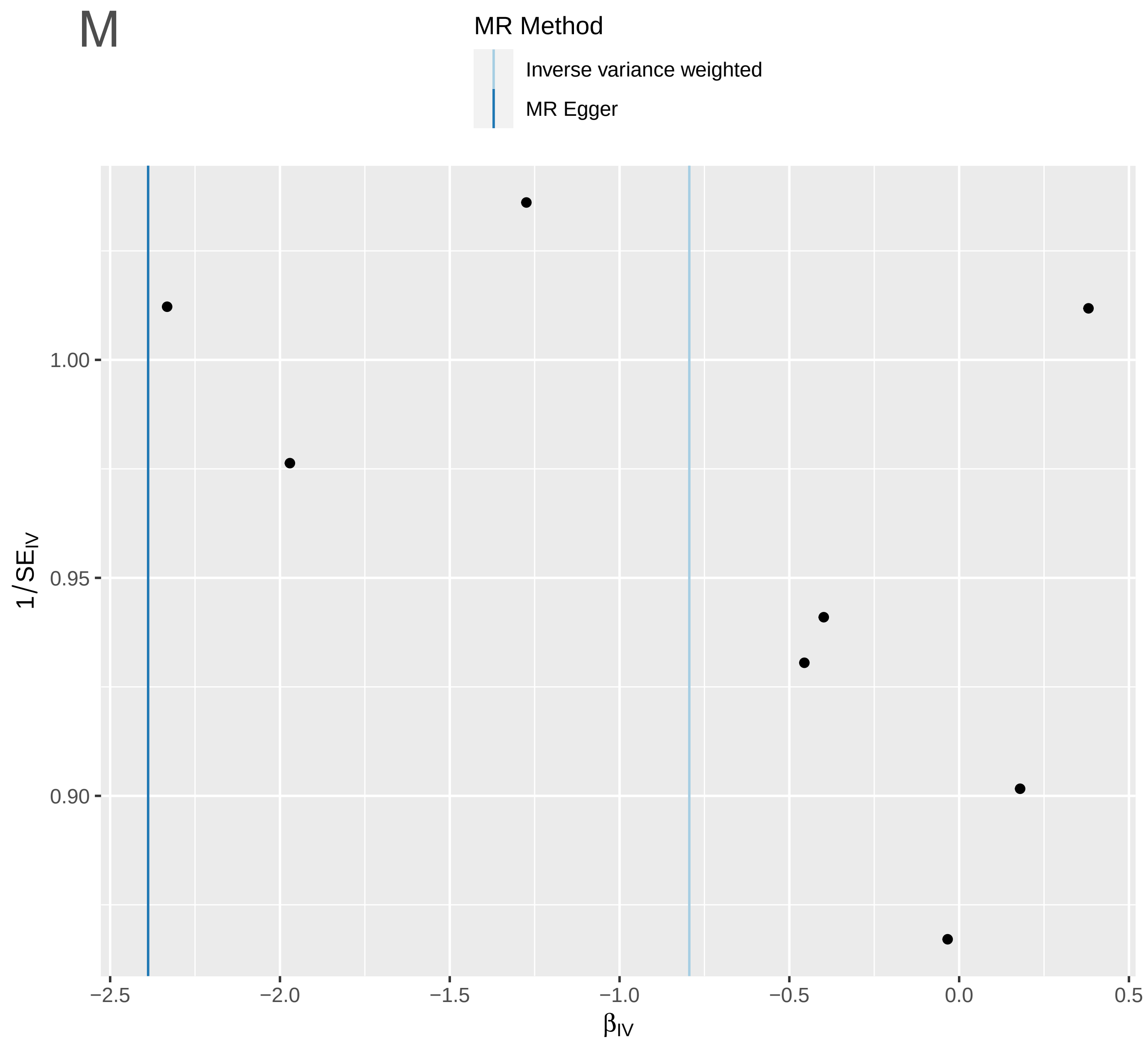
M
MR Method
Inverse variance weighted
MR Egger
1/SE_{IV}
1.00
0.95
0.90
−2.5
−2.0
−1.5
−1.0
−0.5
0.0
0.5
β_{IV}

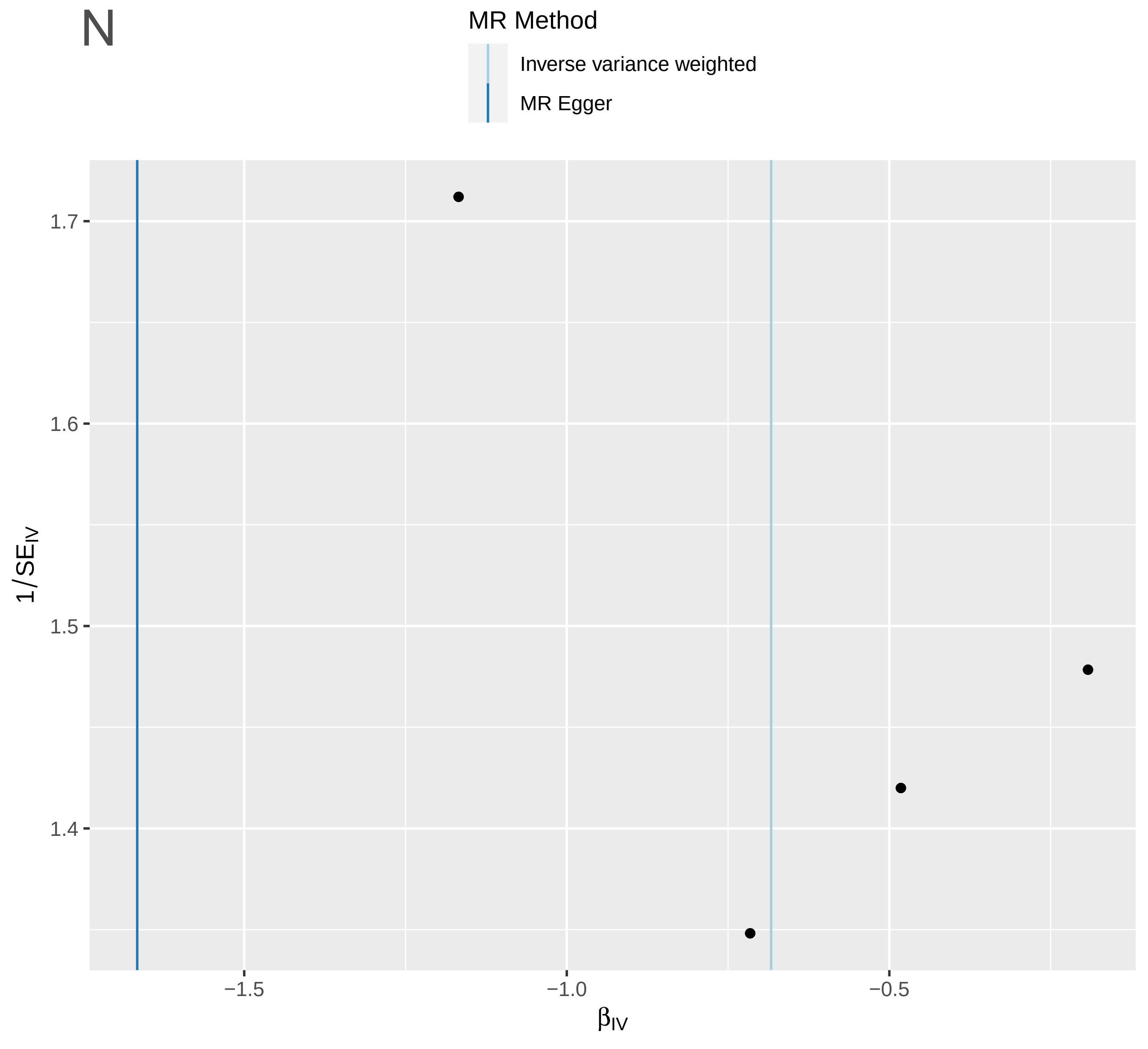
N
MR Method
Inverse variance weighted
MR Egger
1.7
1.6
1.5
1.4
1/SE$_{IV}$
−1.5
−1.0
−0.5
β$_{IV}$

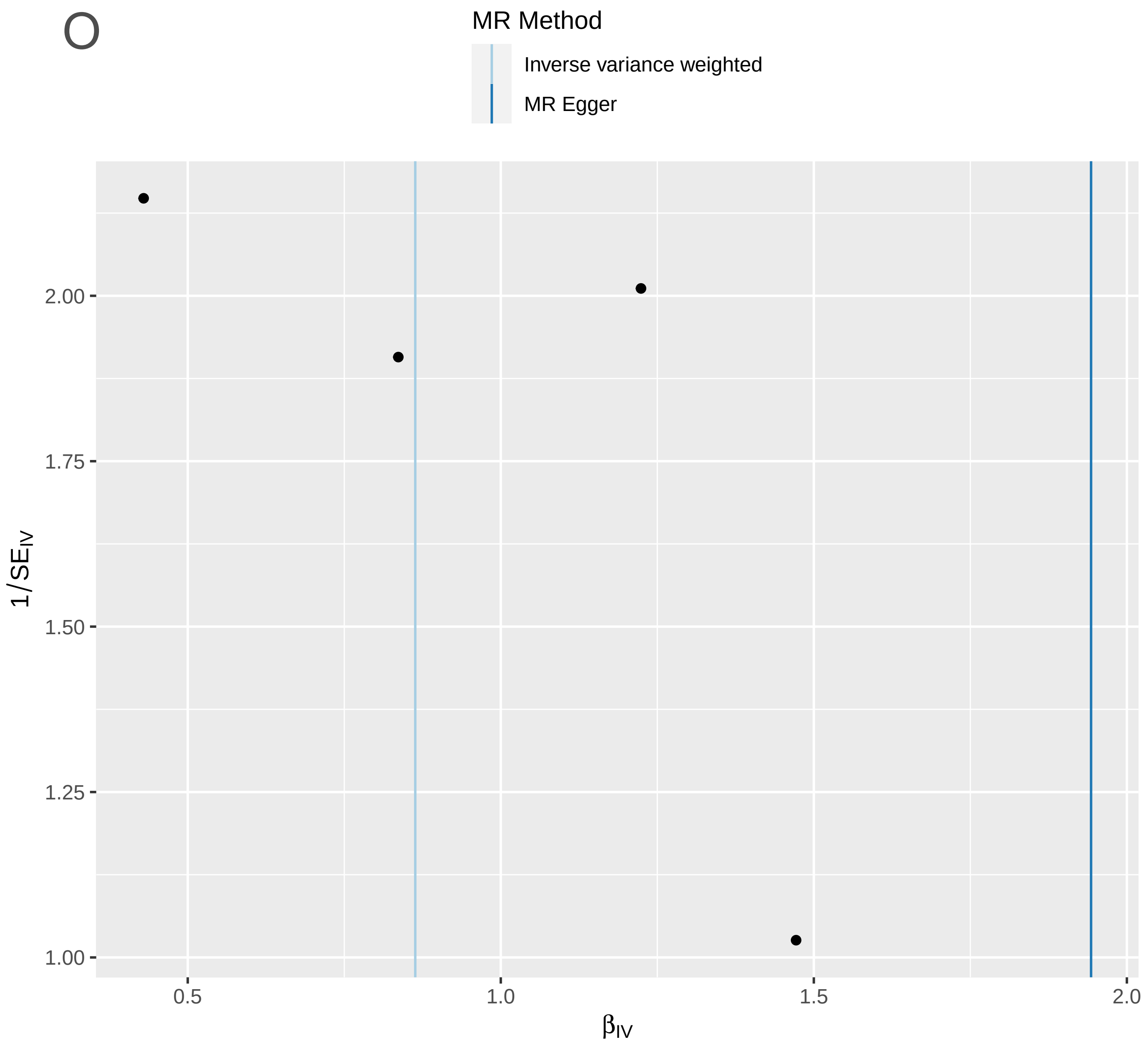
O
MR Method
Inverse variance weighted
MR Egger
2.00
1.75
1.50
1.25
1.00
1/SE_IV
0.5
1.0
1.5
2.0
β_IV

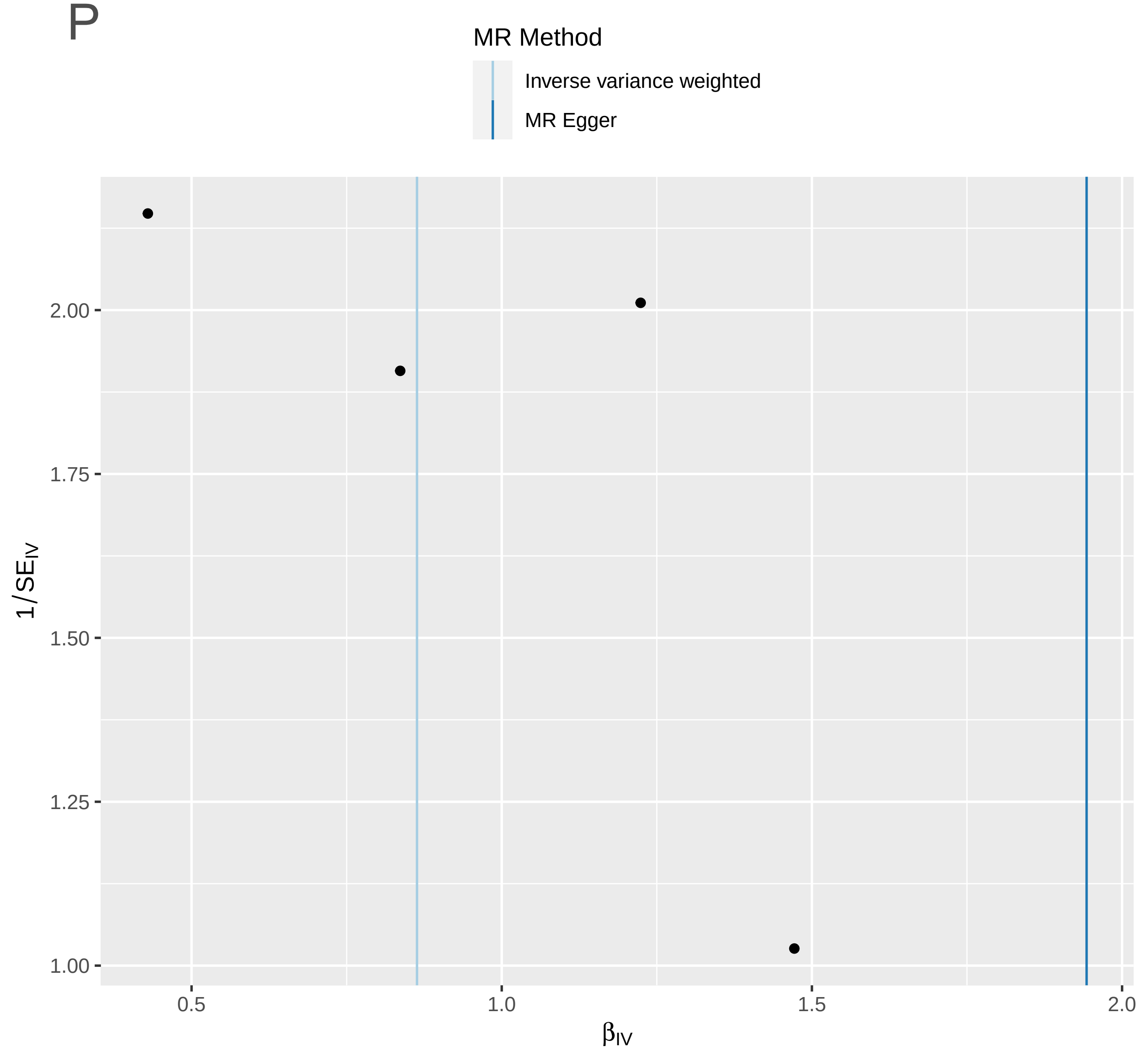
P
MR Method
Inverse variance weighted
MR Egger
2.00
1.75
1.50
1.25
1.00
1/SE_IV
0.5
1.0
1.5
2.0
β_IV

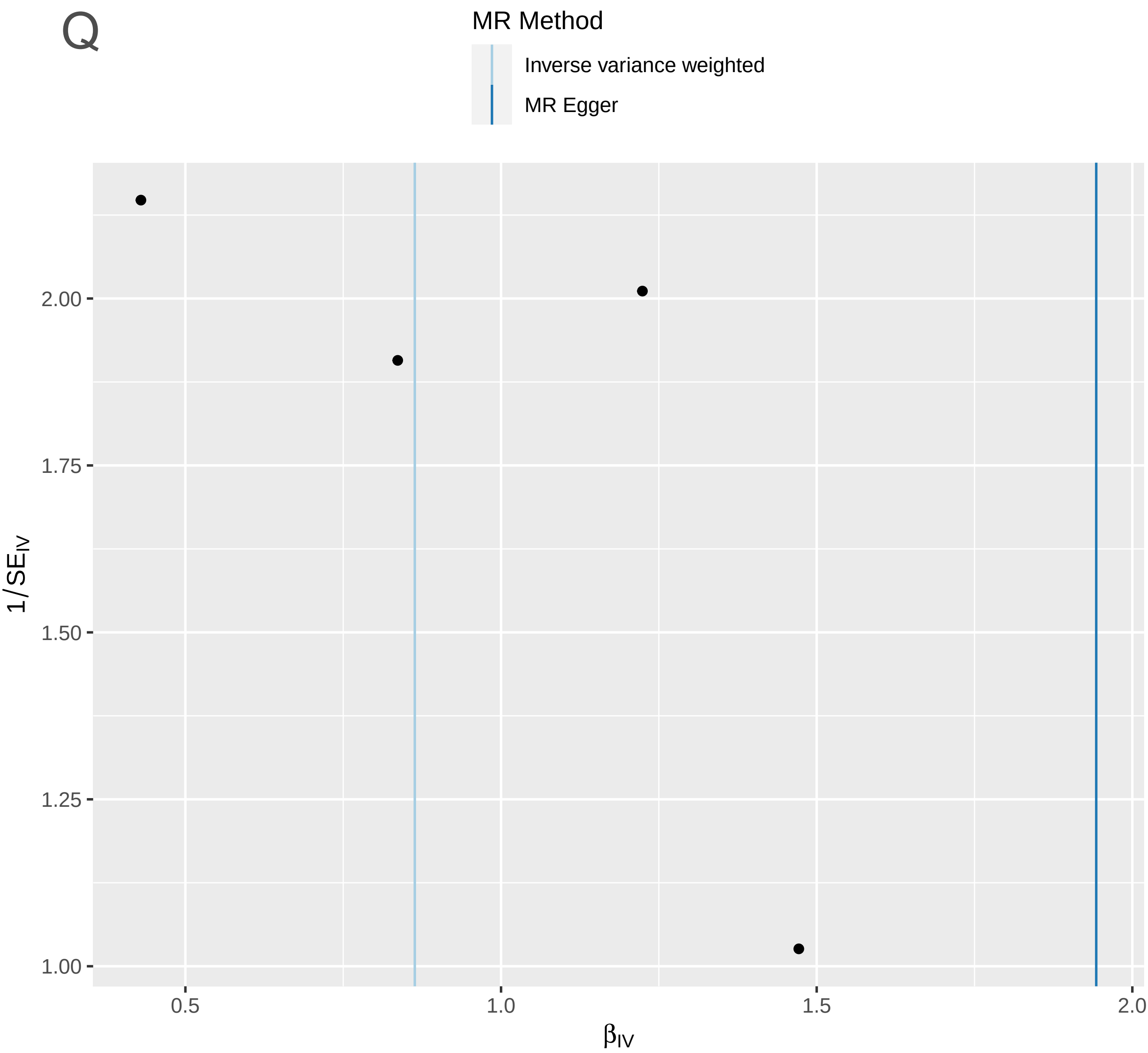

Q
MR Method
Inverse variance weighted
MR Egger
2.00
1.75
1.50
1.25
1.00
$1/SE_{IV}$
0.5
1.0
1.5
2.0
β_{IV}

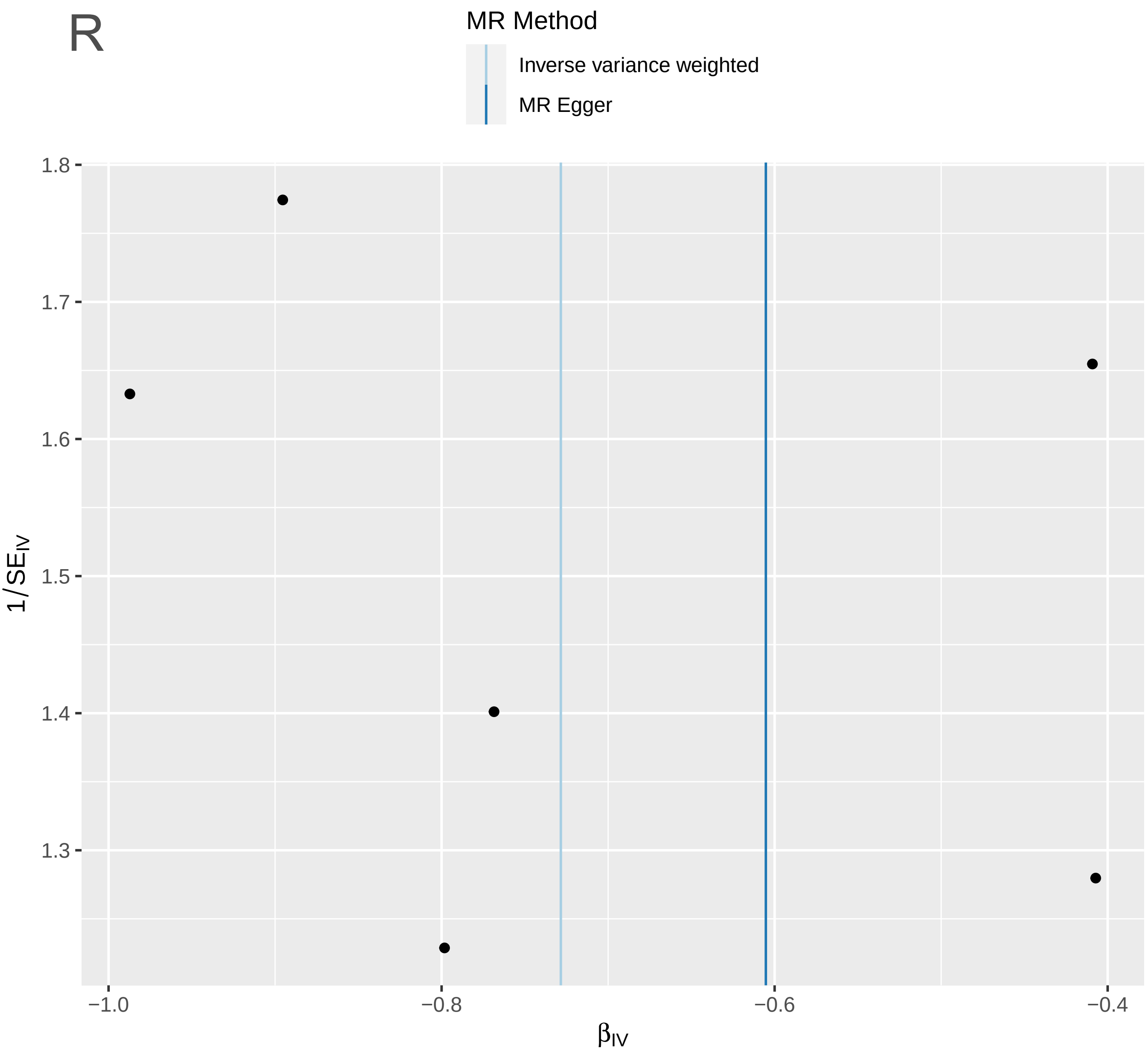
R
MR Method
Inverse variance weighted
MR Egger
1.8
1.7
1.6
1.5
1.4
1.3
1/SE_IV
−1.0
−0.8
−0.6
−0.4
β_IV

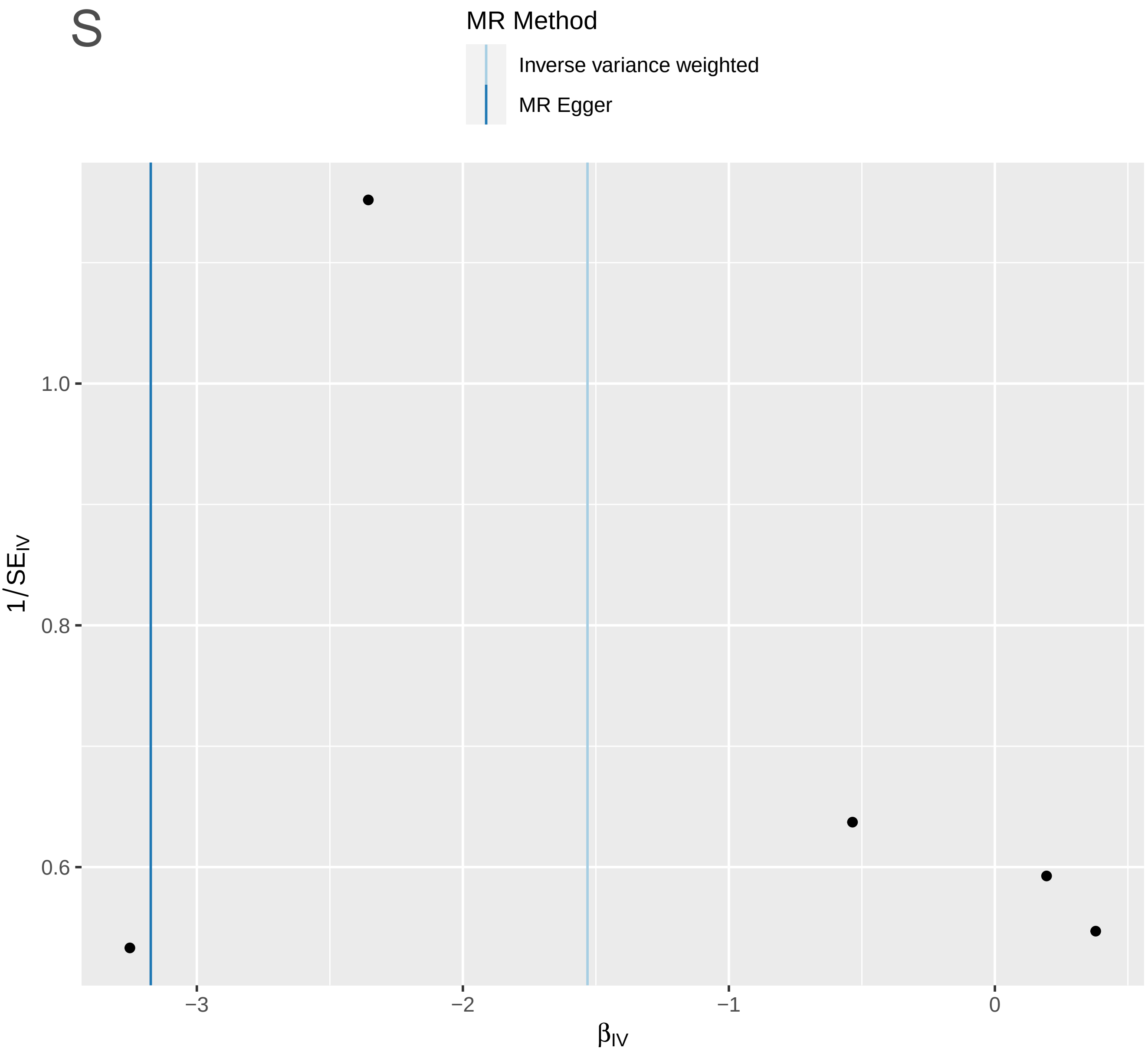
S
MR Method
Inverse variance weighted
MR Egger
1.0
0.8
0.6
1/SE_IV
−3
−2
−1
0
β_IV

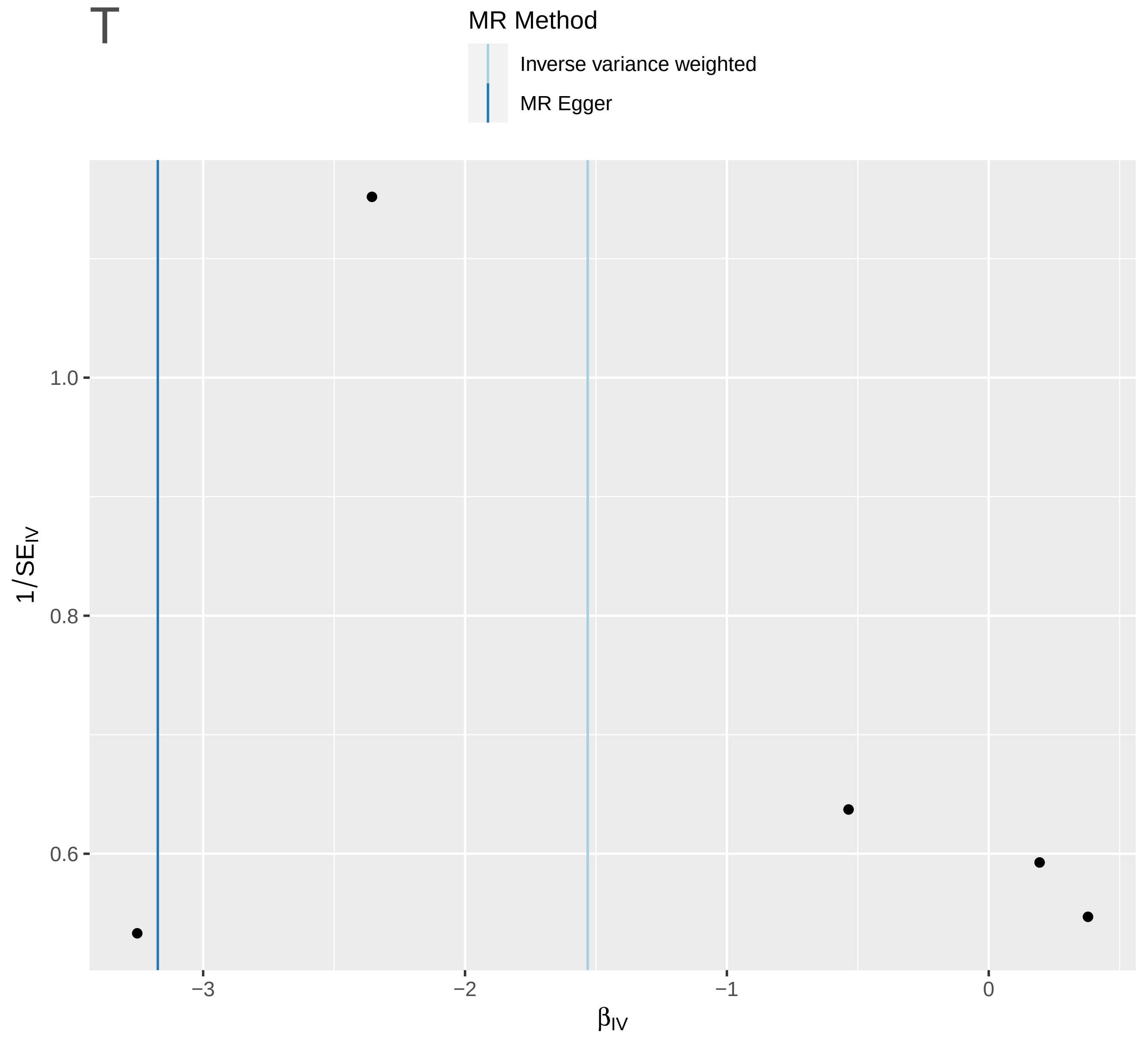
T
MR Method
Inverse variance weighted
MR Egger
1.0
0.8
0.6
$1/SE_{IV}$
−3
−2
−1
0
β_{IV}

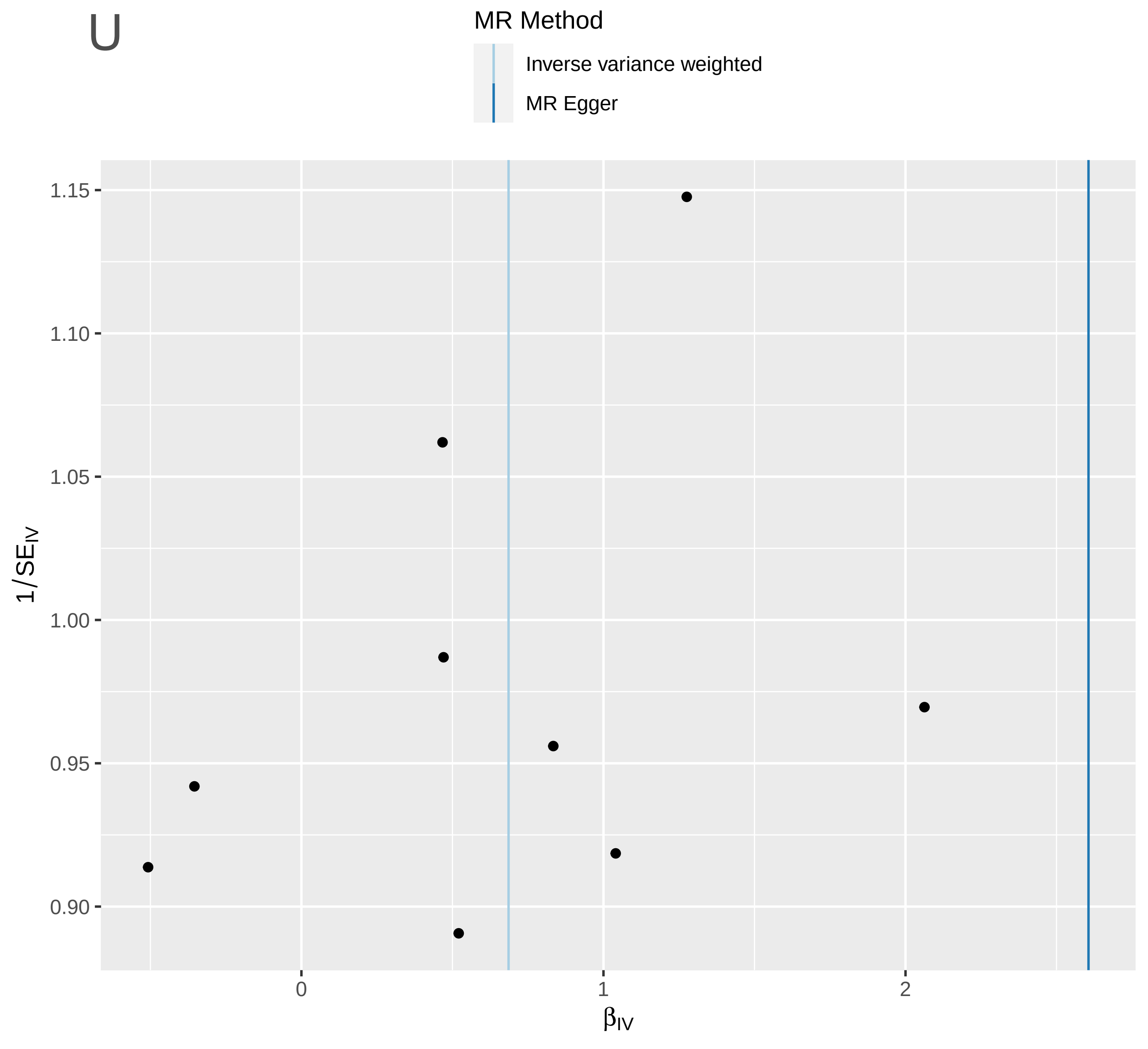

U
MR Method
Inverse variance weighted
MR Egger
1.15
1.10
1.05
1.00
0.95
0.90
$1/SE_{IV}$
0
1
2
β_{IV}

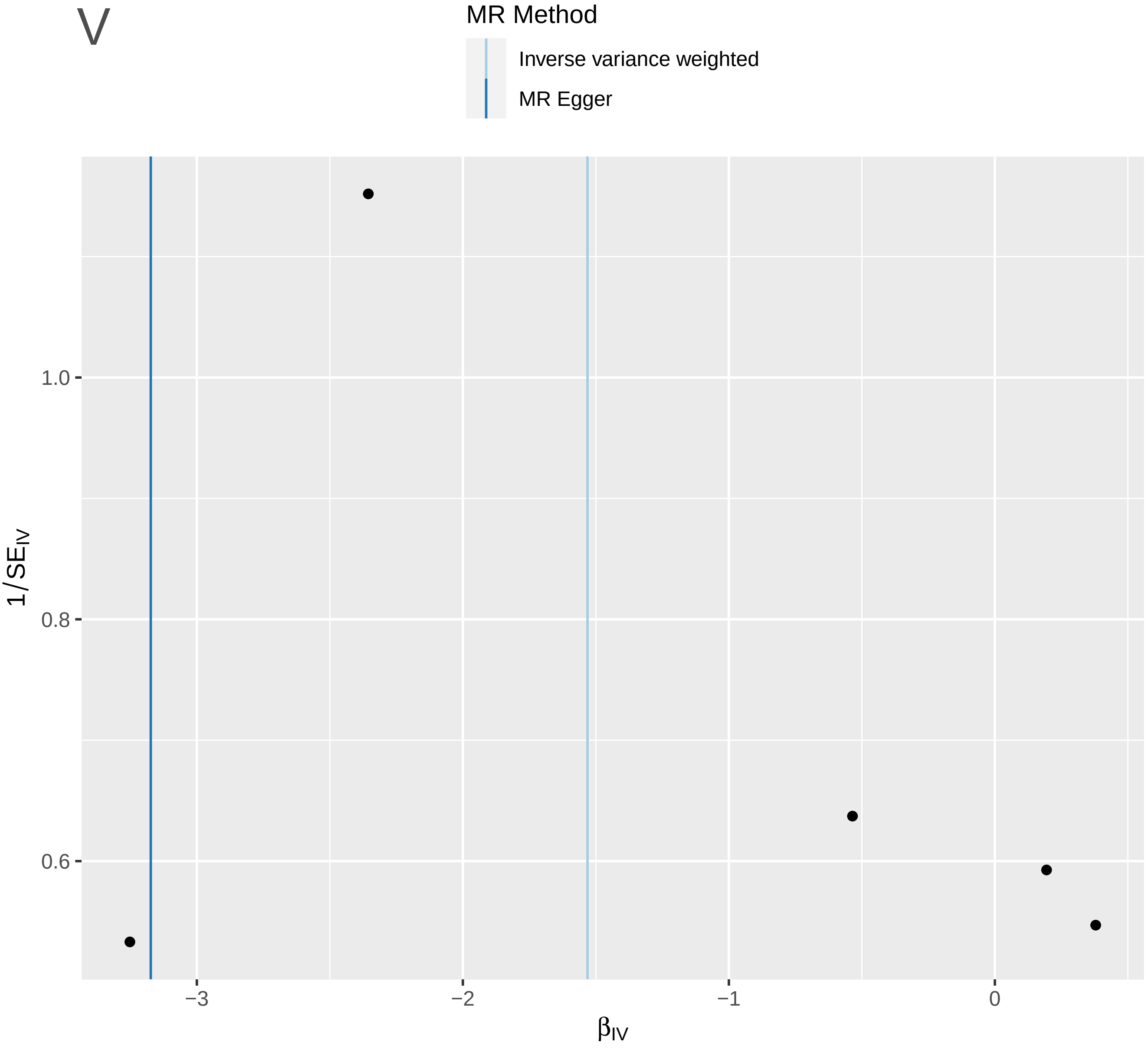
V
MR Method
Inverse variance weighted
MR Egger
1.0
0.8
0.6
1/SE_IV
−3
−2
−1
0
β_IV

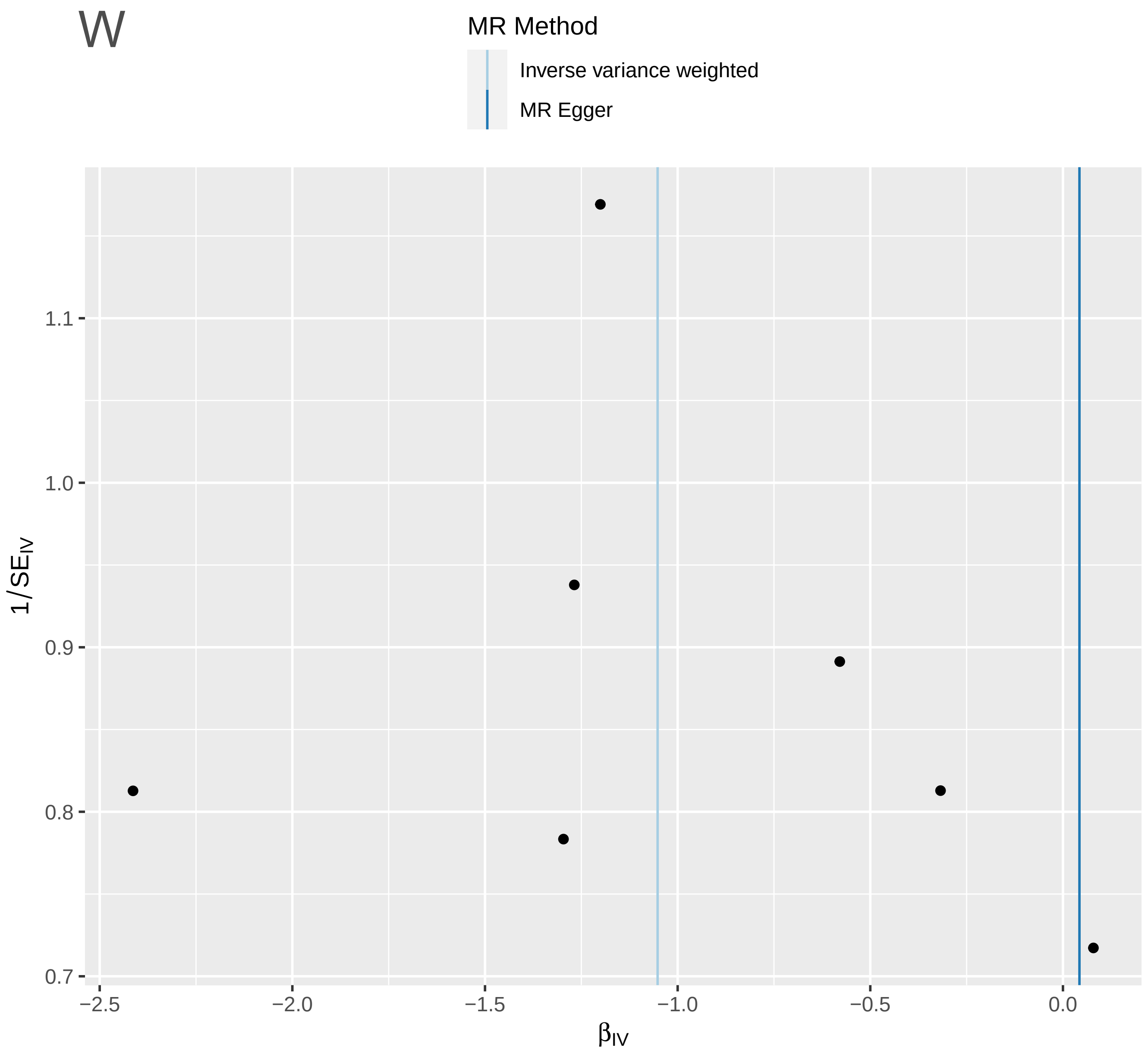
W
MR Method
Inverse variance weighted
MR Egger
1.1
1.0
0.9
0.8
0.7
1/SE_IV
−2.5
−2.0
−1.5
−1.0
−0.5
0.0
β_IV

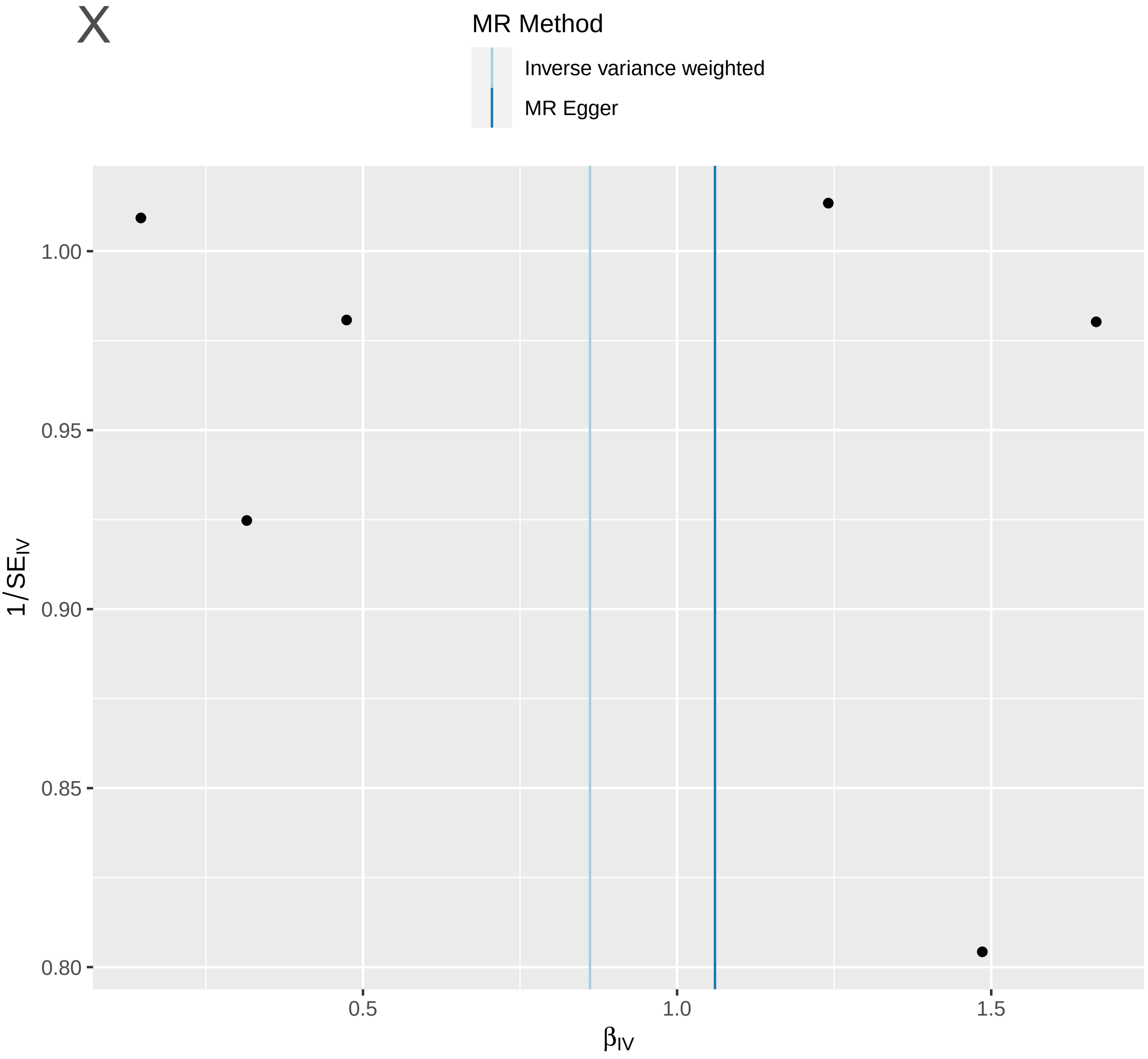
X
MR Method
Inverse variance weighted
MR Egger
1.00
0.95
0.90
0.85
0.80
$1/SE_{IV}$
0.5
1.0
1.5
β_{IV}

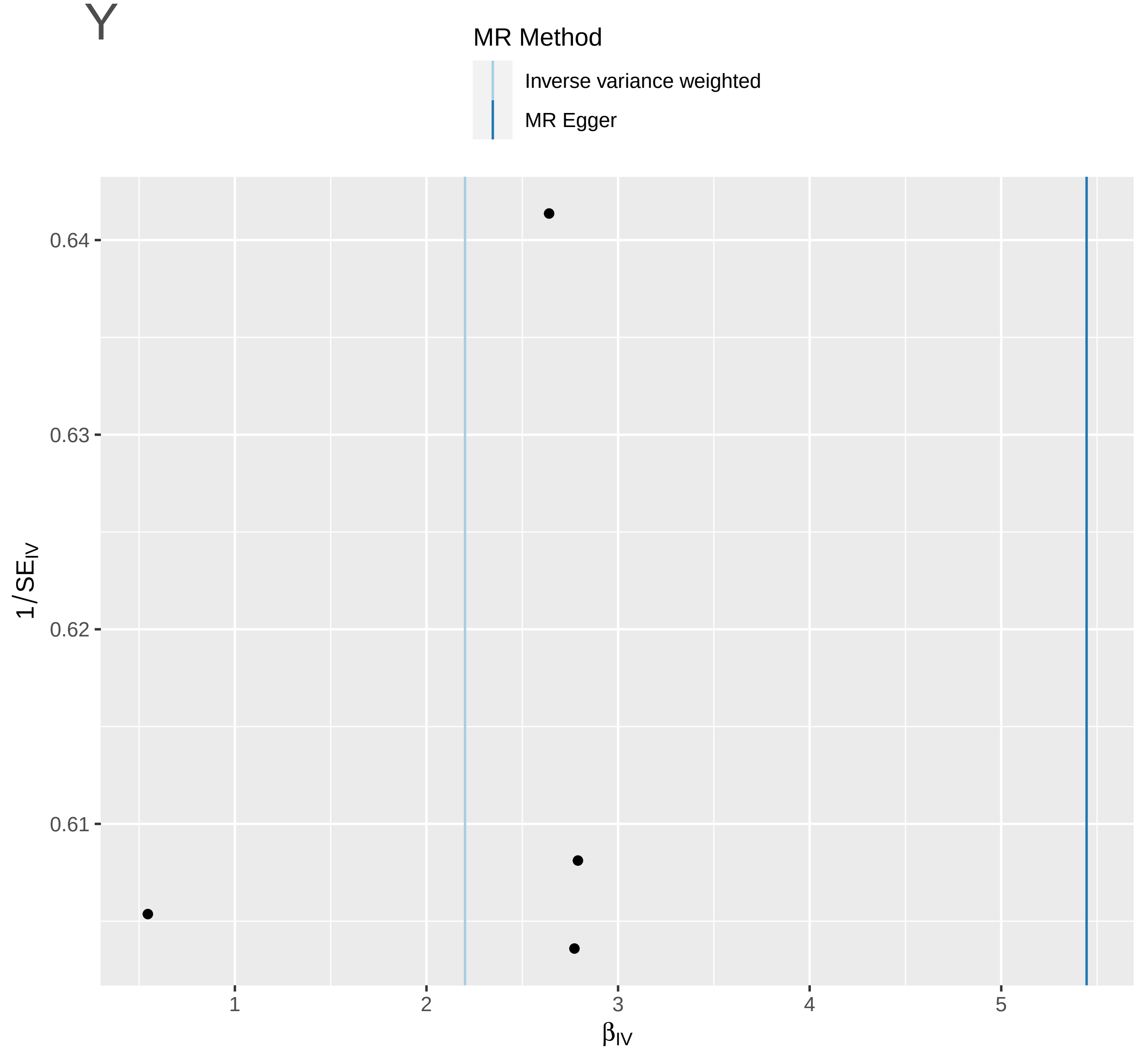
Y
MR Method
Inverse variance weighted
MR Egger
0.64
0.63
0.62
0.61
1/SE_IV
1
2
3
4
5
β_IV

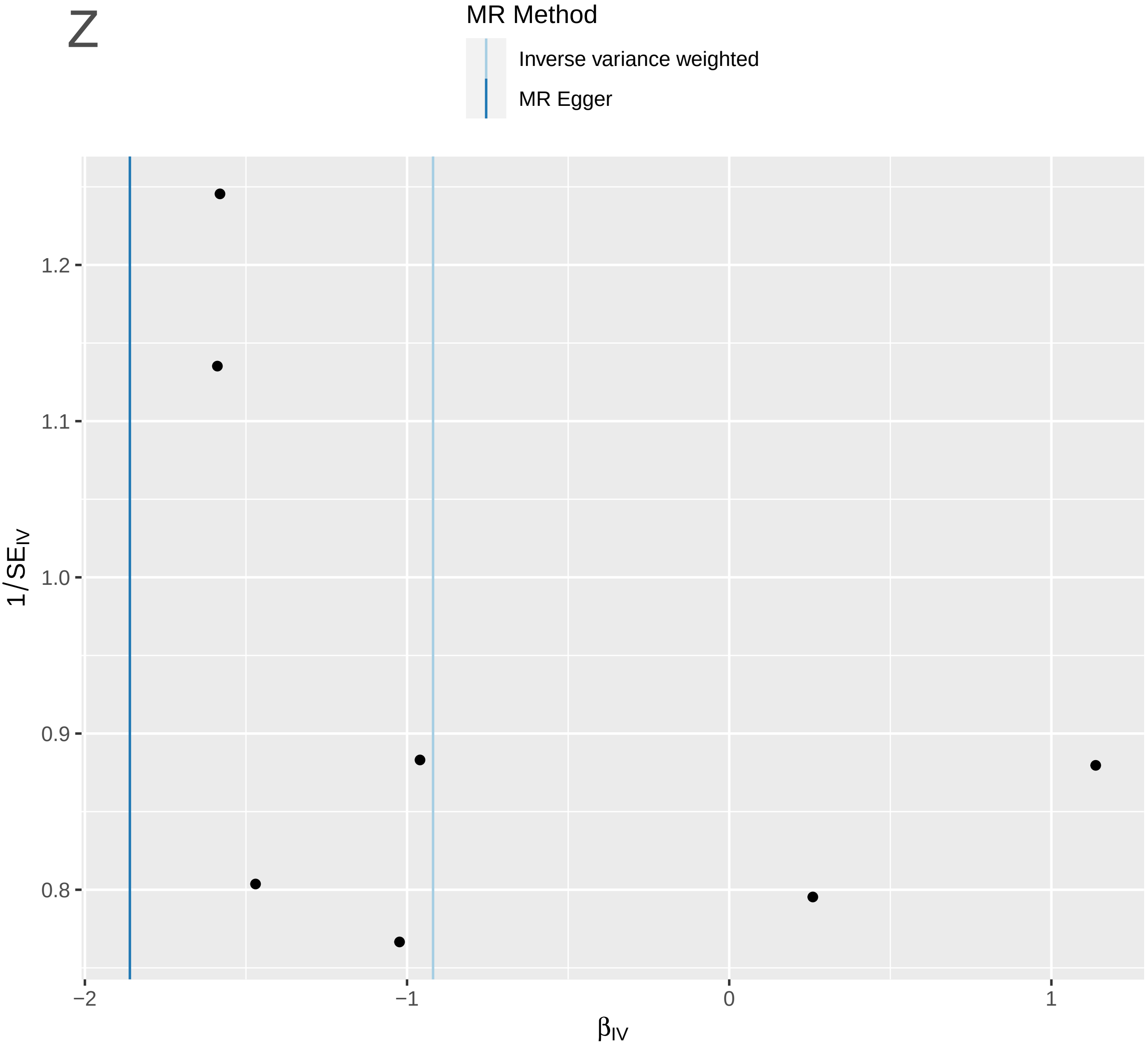
Z
MR Method
Inverse variance weighted
MR Egger
1.2
1.1
1.0
0.9
0.8
$1/SE_{IV}$
−2
−1
0
1
β_{IV}

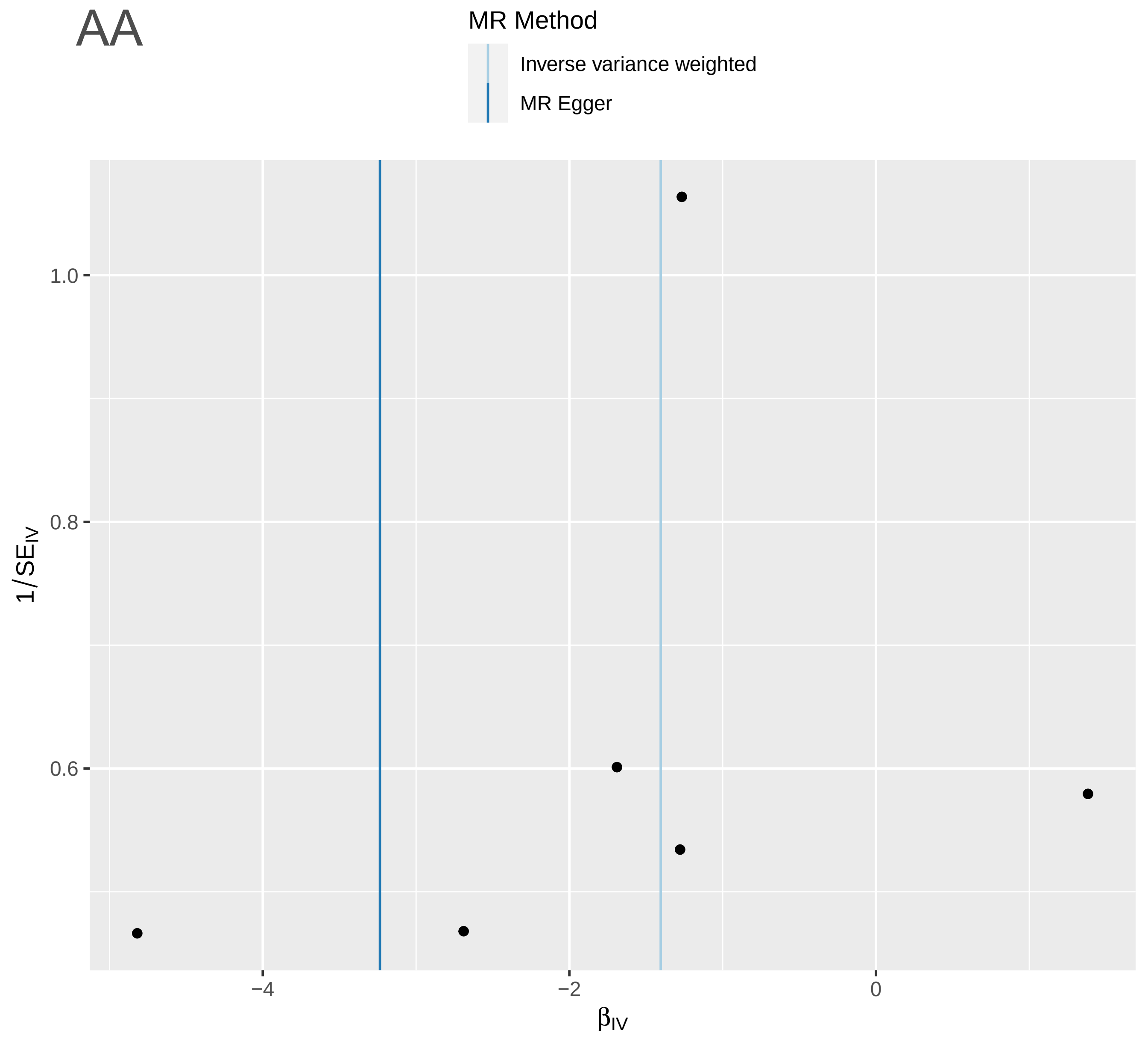
AA
MR Method
Inverse variance weighted
MR Egger
1.0
0.8
0.6
−4
−2
0
$1/SE_{IV}$
β_{IV}

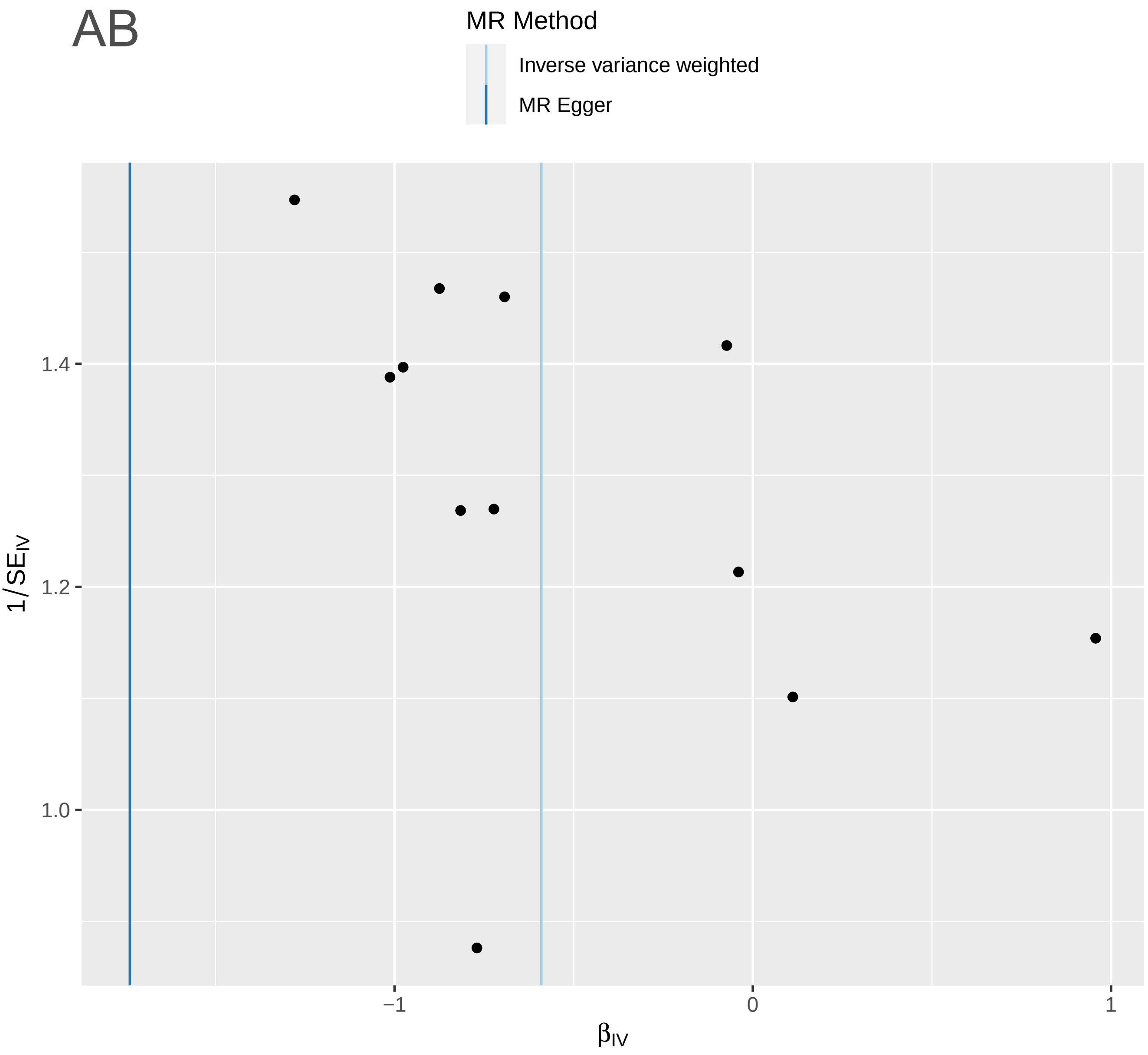
AB
MR Method
Inverse variance weighted
MR Egger
1.4
1.2
1.0
1/SE$_{IV}$
−1
0
1
β$_{IV}$

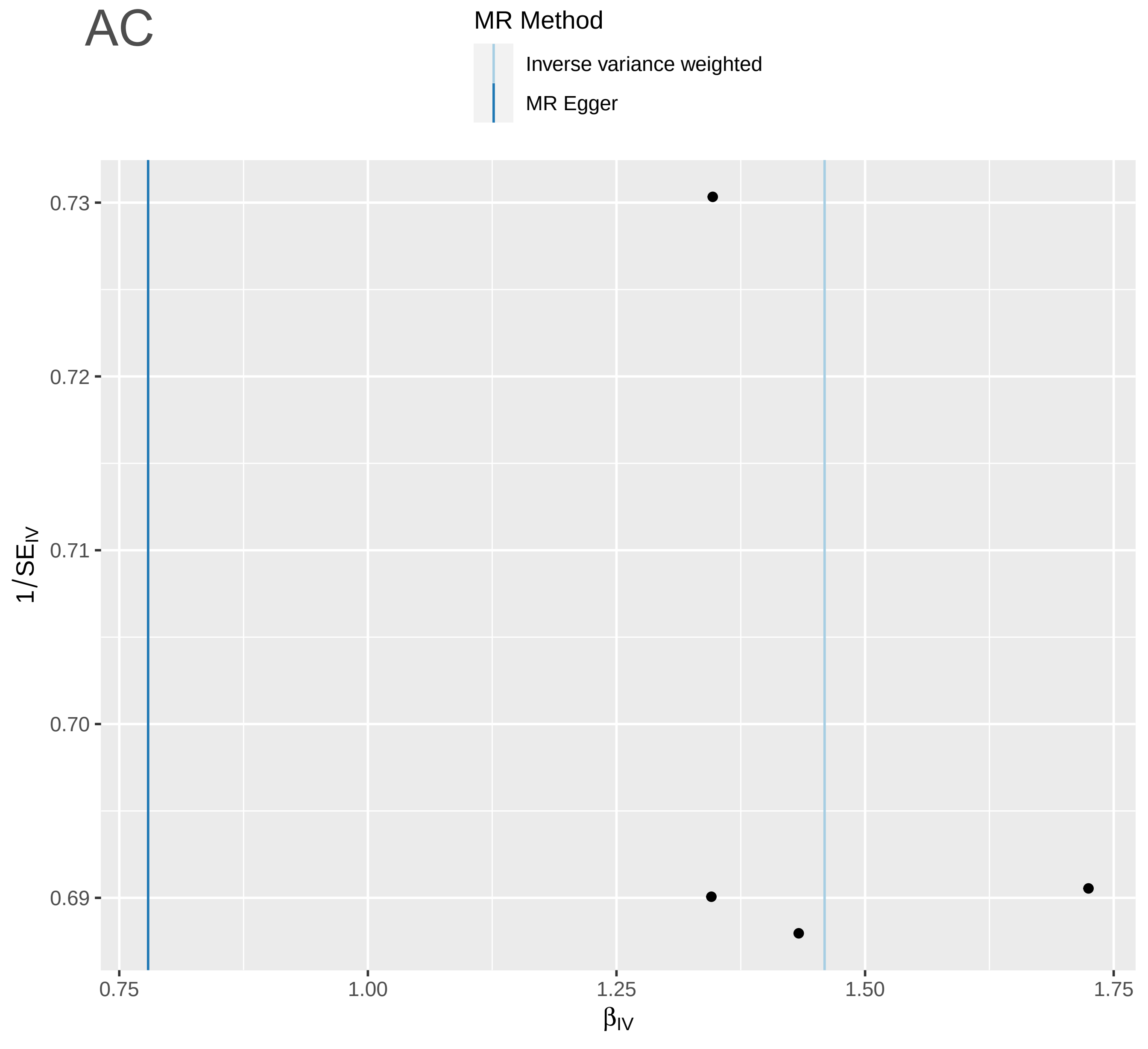

AC
MR Method
Inverse variance weighted
MR Egger
0.73
0.72
0.71
0.70
0.69
$1/SE_{IV}$
0.75
1.00
1.25
1.50
1.75
β_{IV}

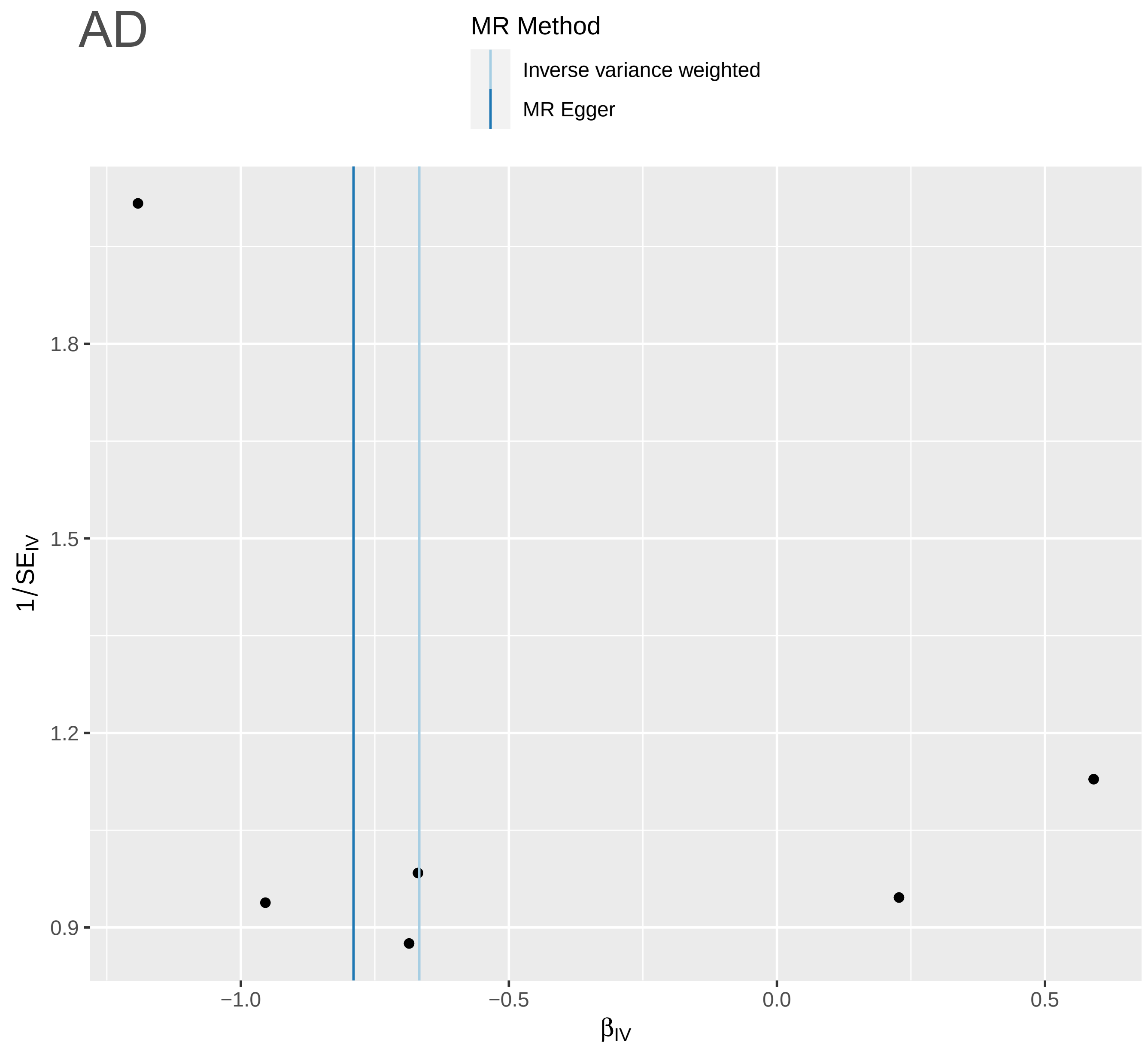
AD
MR Method
Inverse variance weighted
MR Egger
1.8
1.5
1.2
0.9
1/SE$_{IV}$
−1.0
−0.5
0.0
0.5
β$_{IV}$

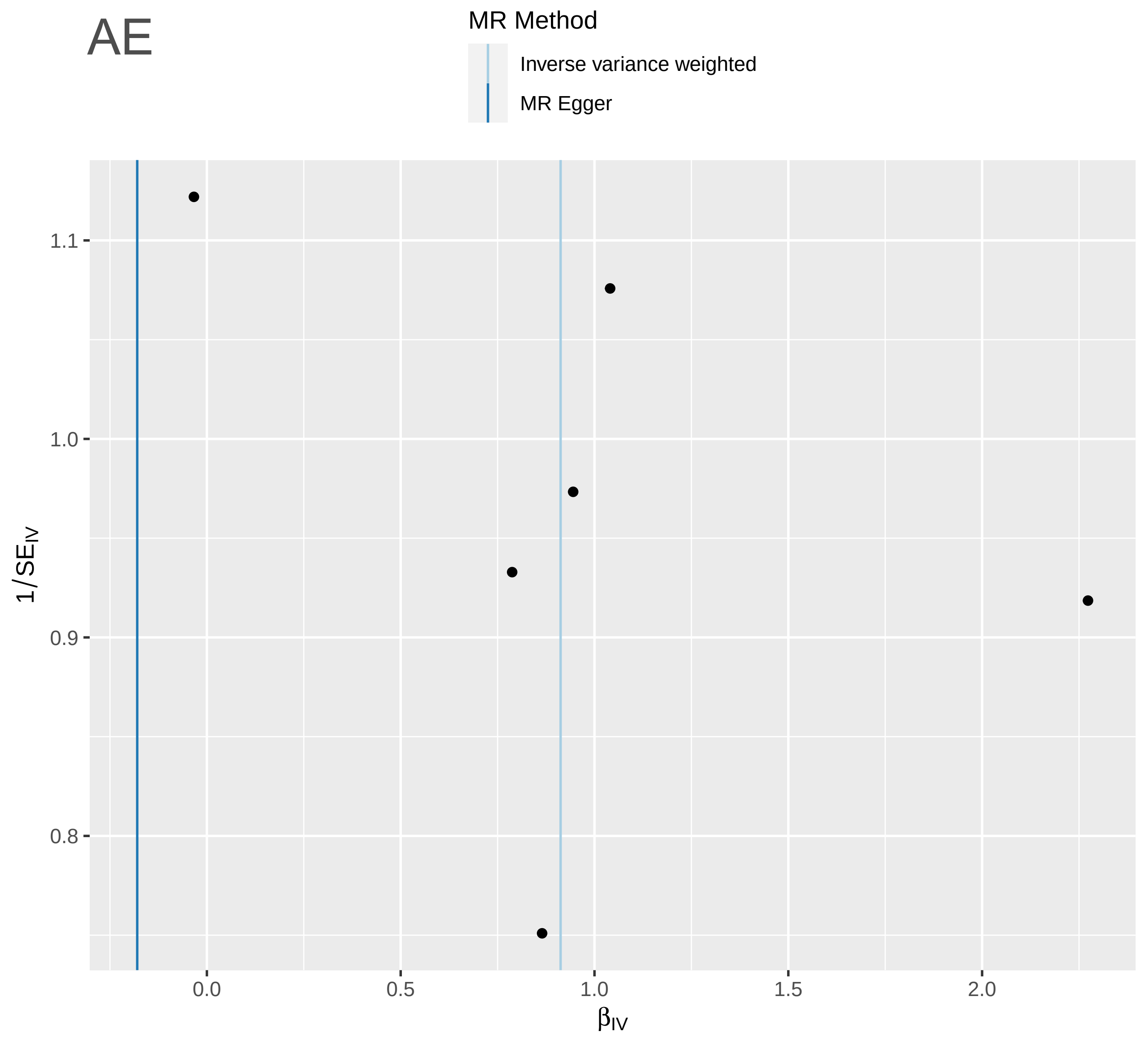
AE
MR Method
Inverse variance weighted
MR Egger
1.1
1.0
0.9
0.8
1/SE_IV
0.0
0.5
1.0
1.5
2.0
β_IV

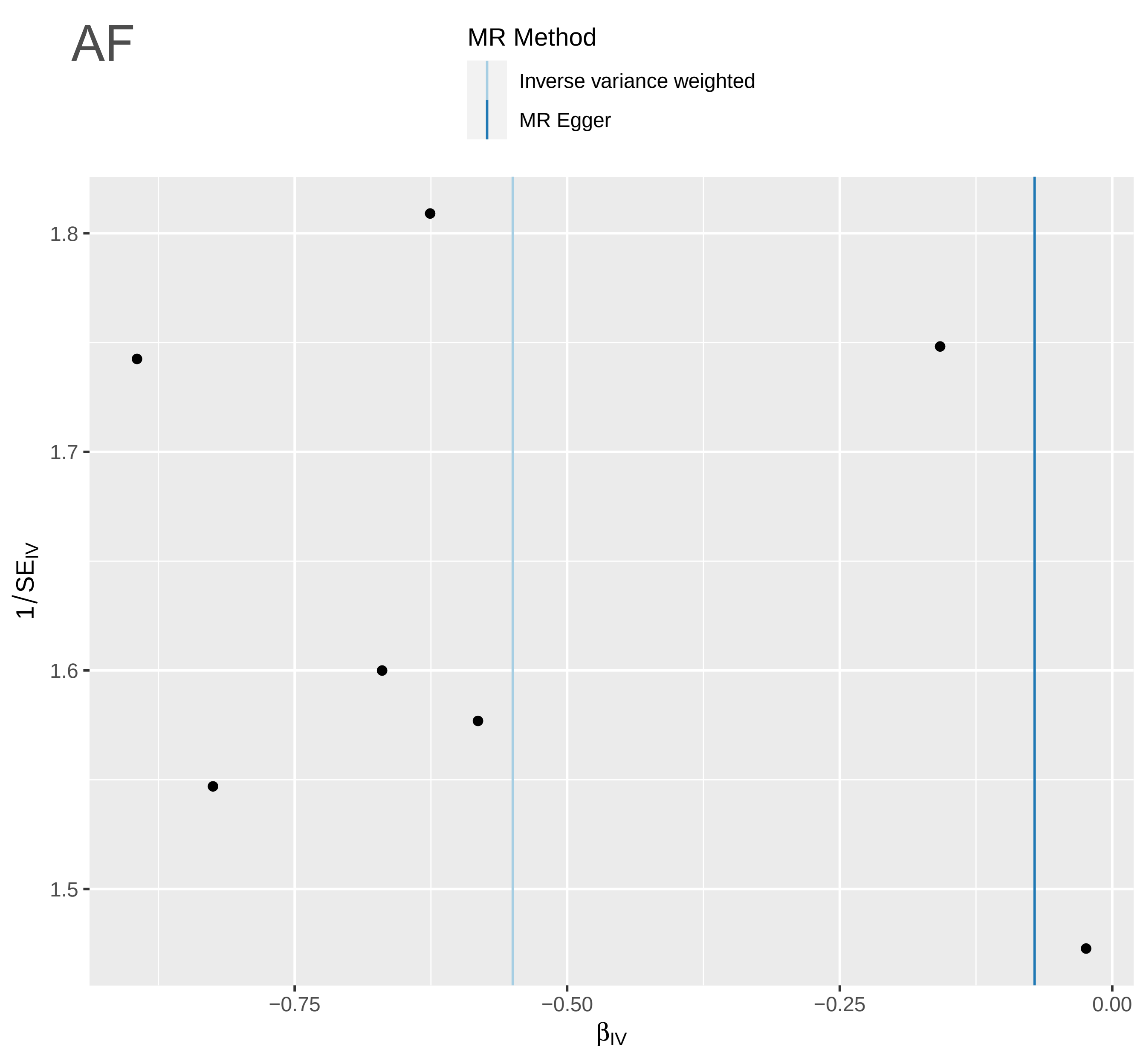
AF
MR Method
Inverse variance weighted
MR Egger
1.8
1.7
1.6
1.5
1/SE_{IV}
−0.75
−0.50
−0.25
0.00
β_{IV}

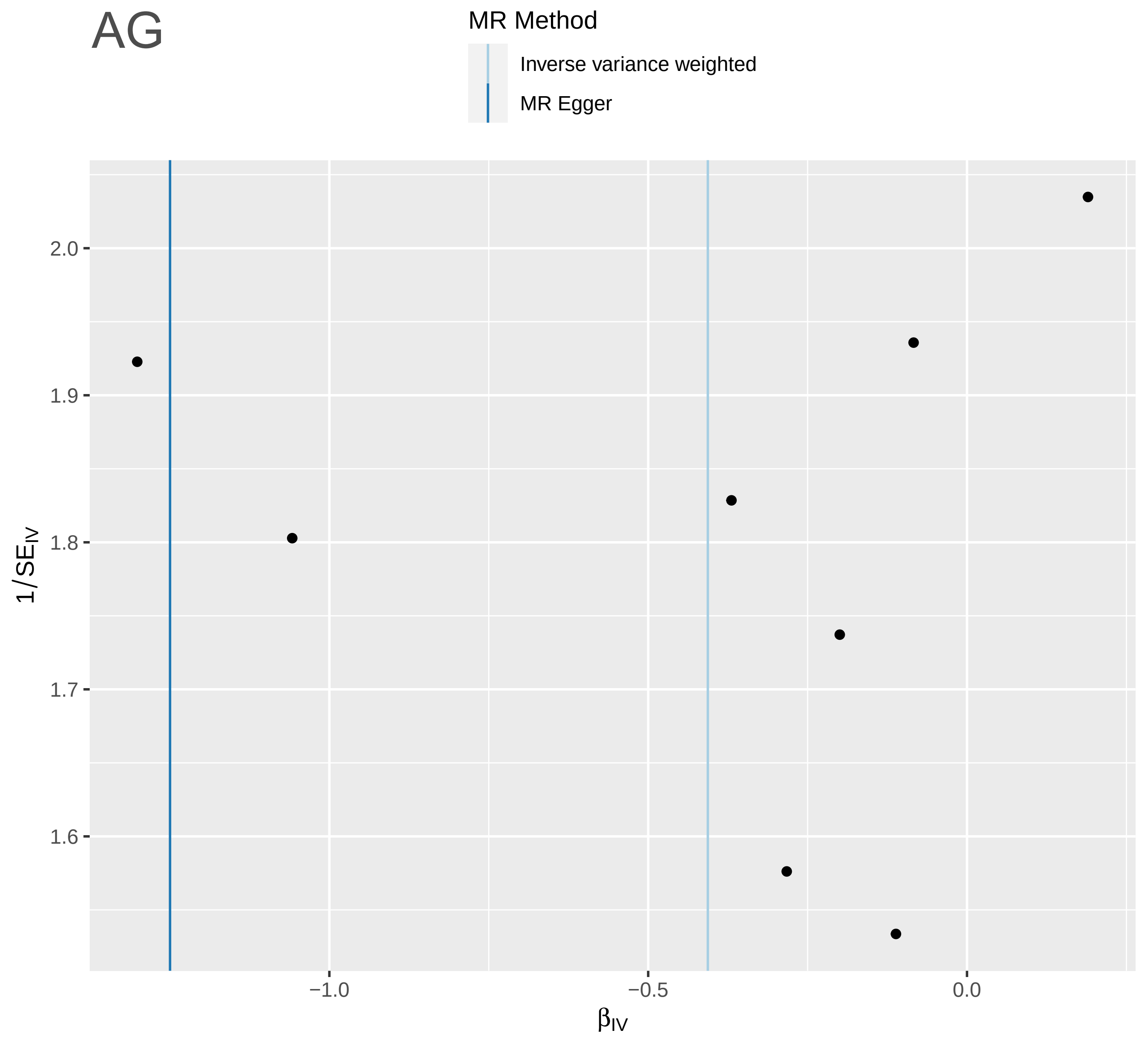
AG
MR Method
Inverse variance weighted
MR Egger
2.0
1.9
1.8
1.7
1.6
1/SE_IV
−1.0
−0.5
0.0
β_IV

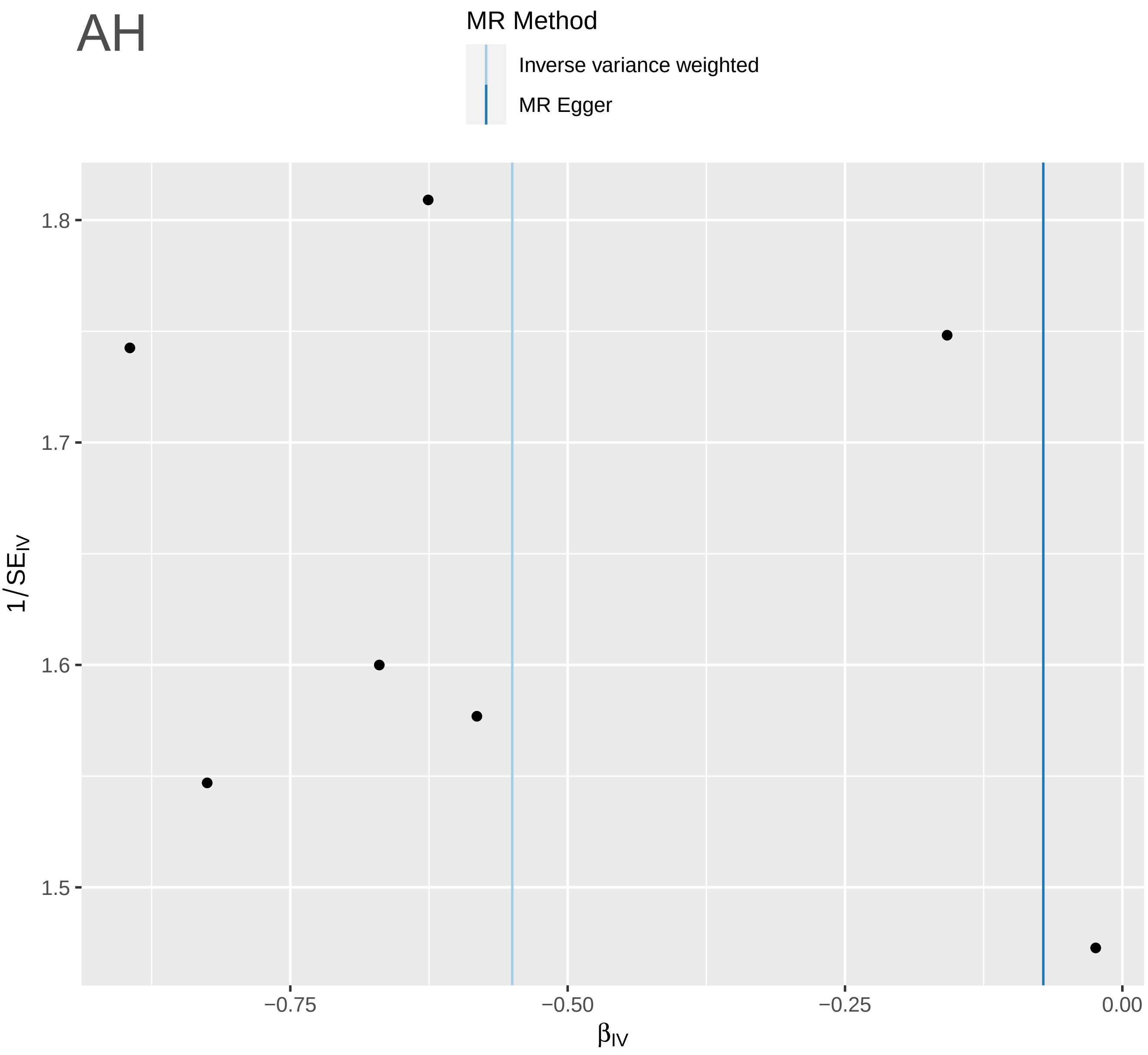
AH
MR Method
Inverse variance weighted
MR Egger
1.8
1.7
1.6
1.5
1/SE$_{IV}$
−0.75
−0.50
−0.25
0.00
β$_{IV}$

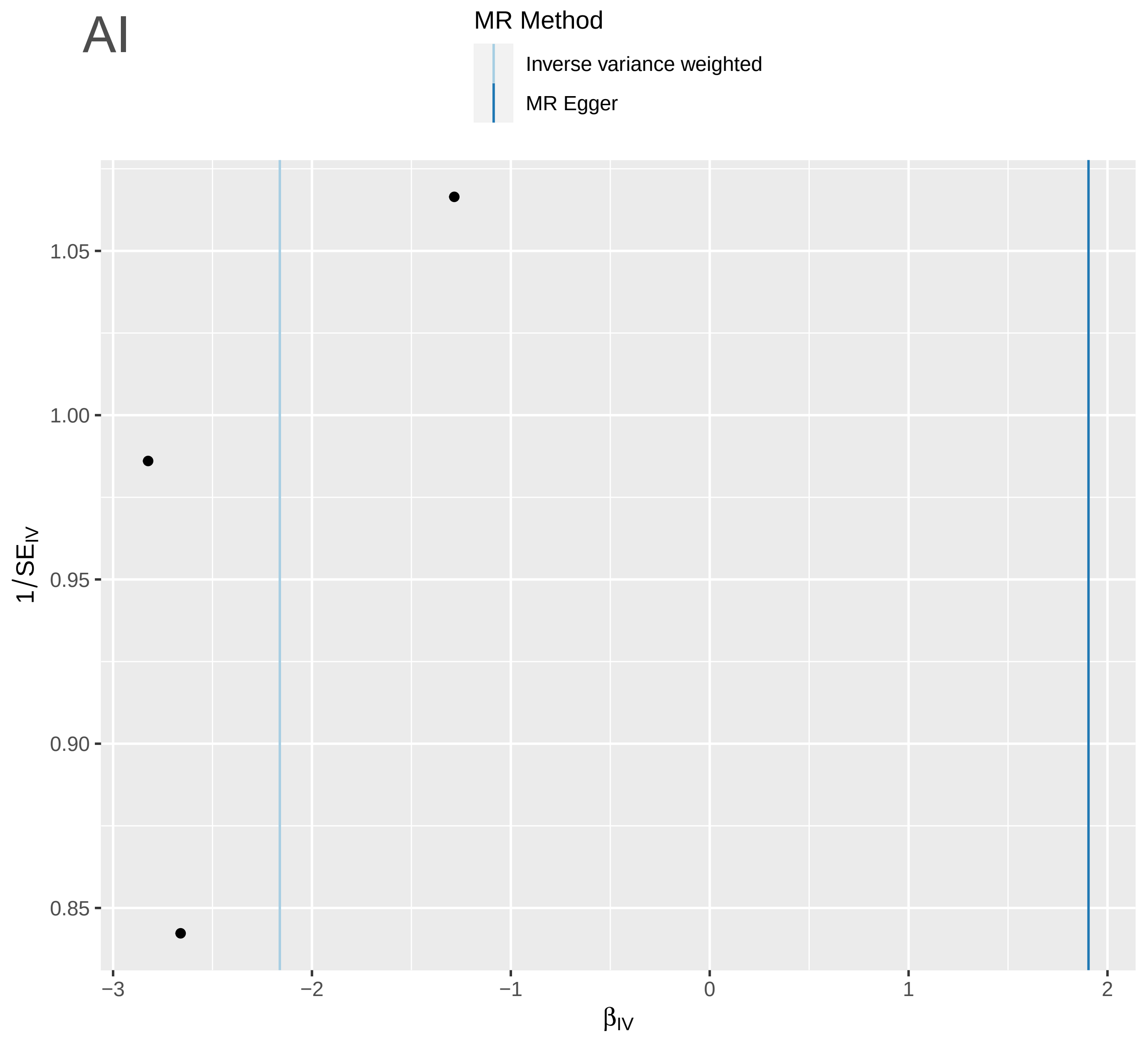

AI
MR Method
Inverse variance weighted
MR Egger
1.05
1.00
0.95
0.90
0.85
1/SE_IV
−3
−2
−1
0
1
2
β_IV

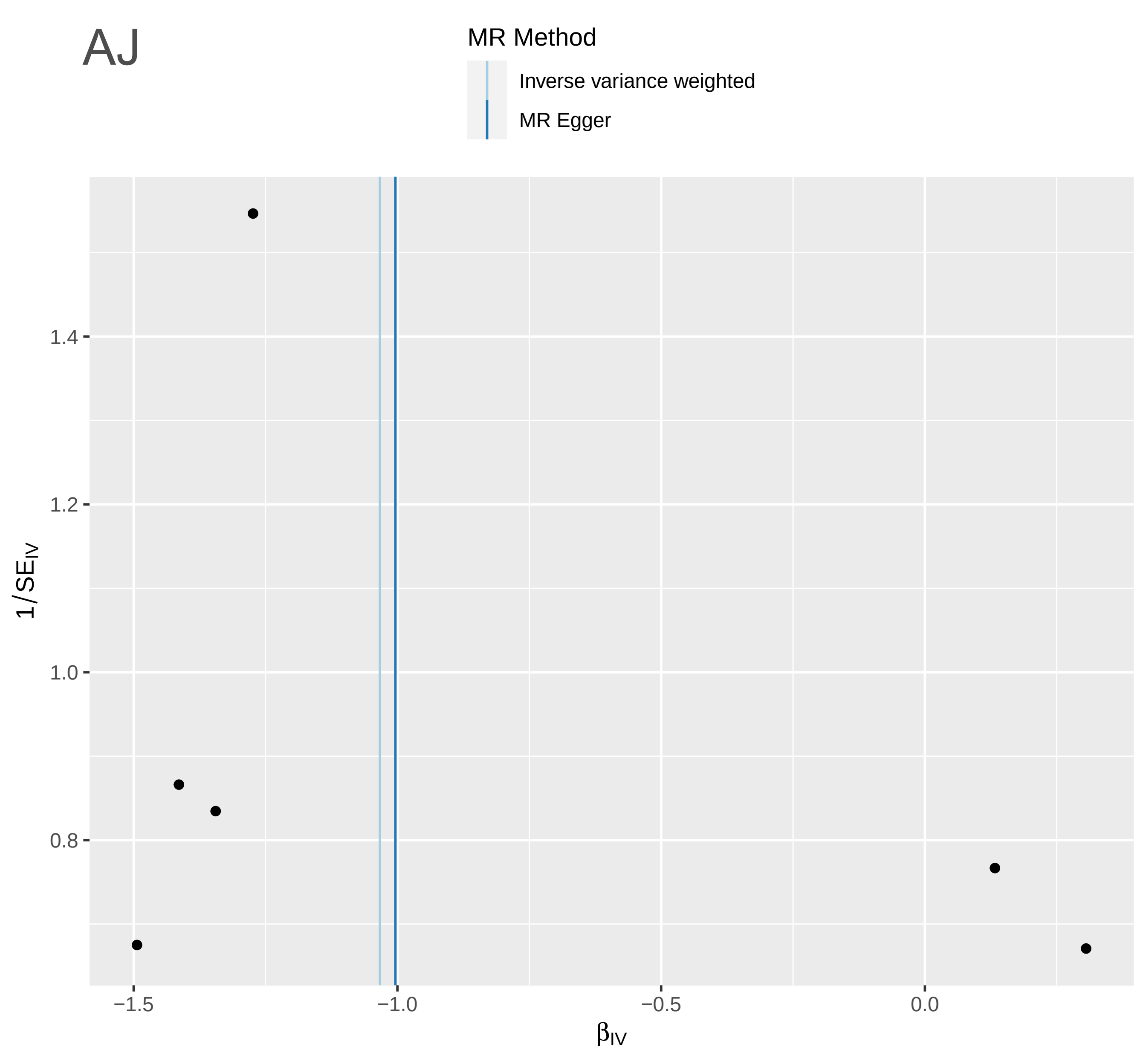
AJ
MR Method
Inverse variance weighted
MR Egger
1.4
1.2
1.0
0.8
$1/SE_{IV}$
−1.5
−1.0
−0.5
0.0
β_{IV}

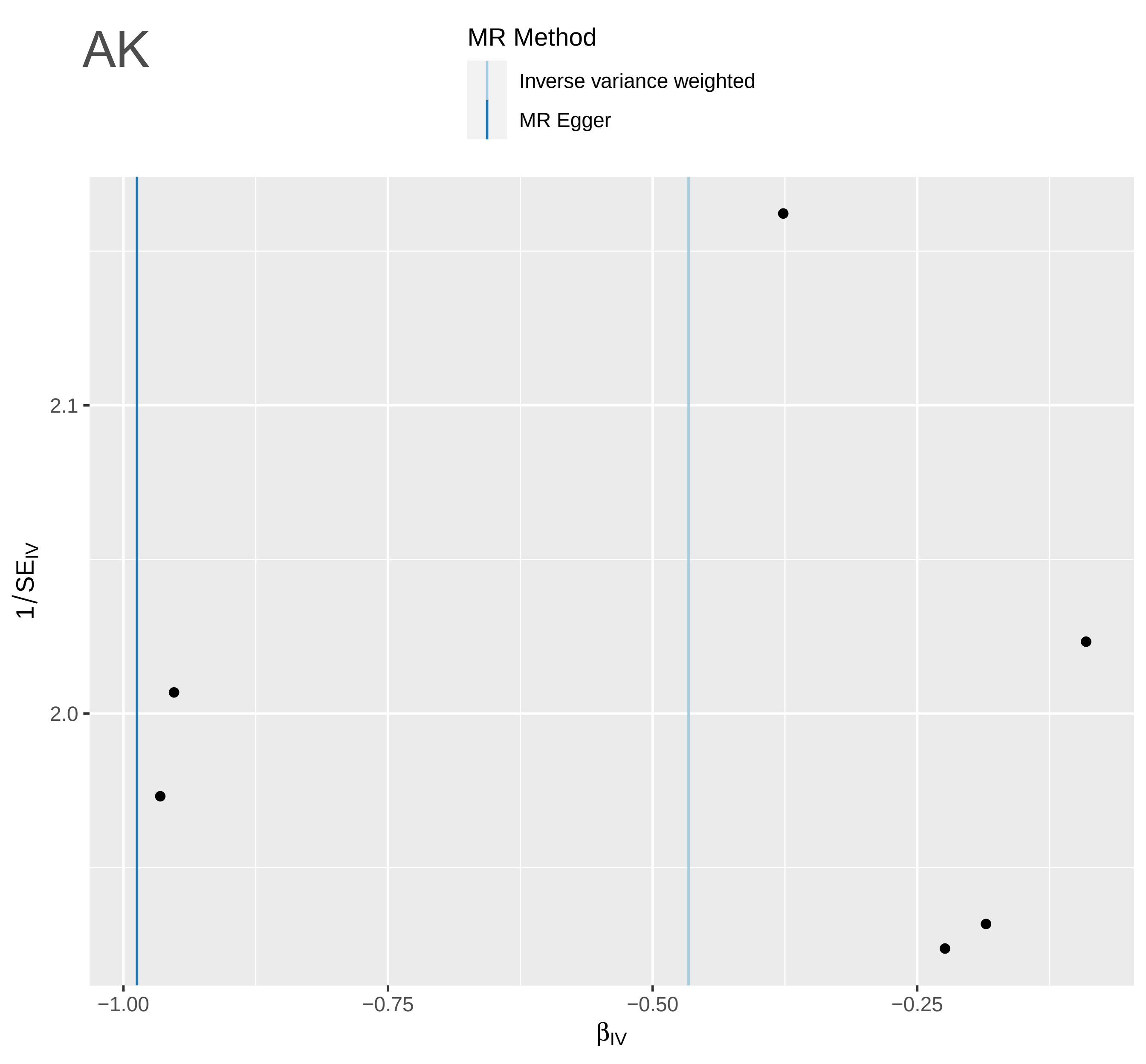

AK
MR Method
Inverse variance weighted
MR Egger
2.1
2.0
1/SE_IV
−1.00
−0.75
−0.50
−0.25
β_IV

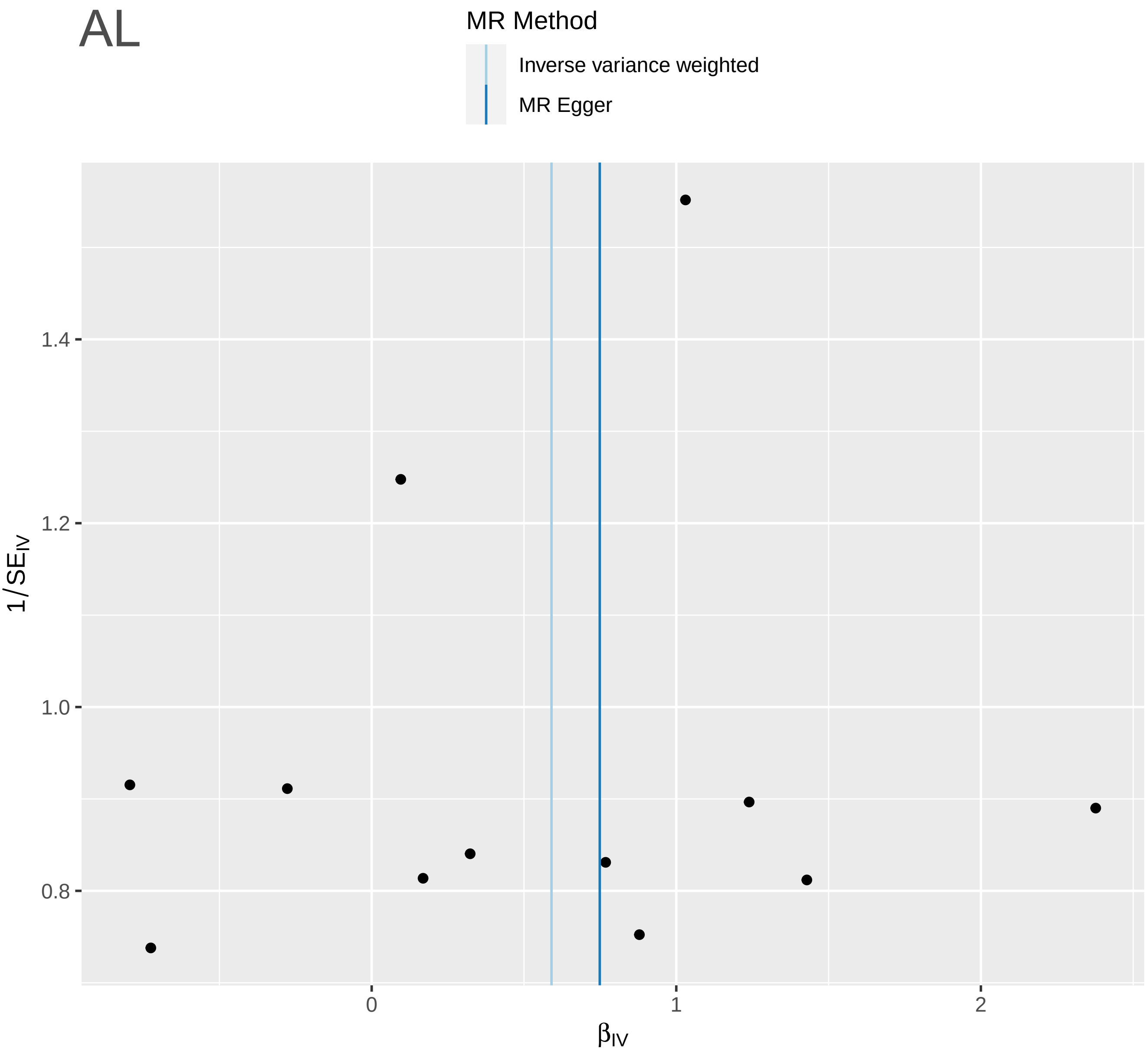
AL
MR Method
Inverse variance weighted
MR Egger
1.4
1.2
1.0
0.8
0
1
2
1/SE_IV
β_IV

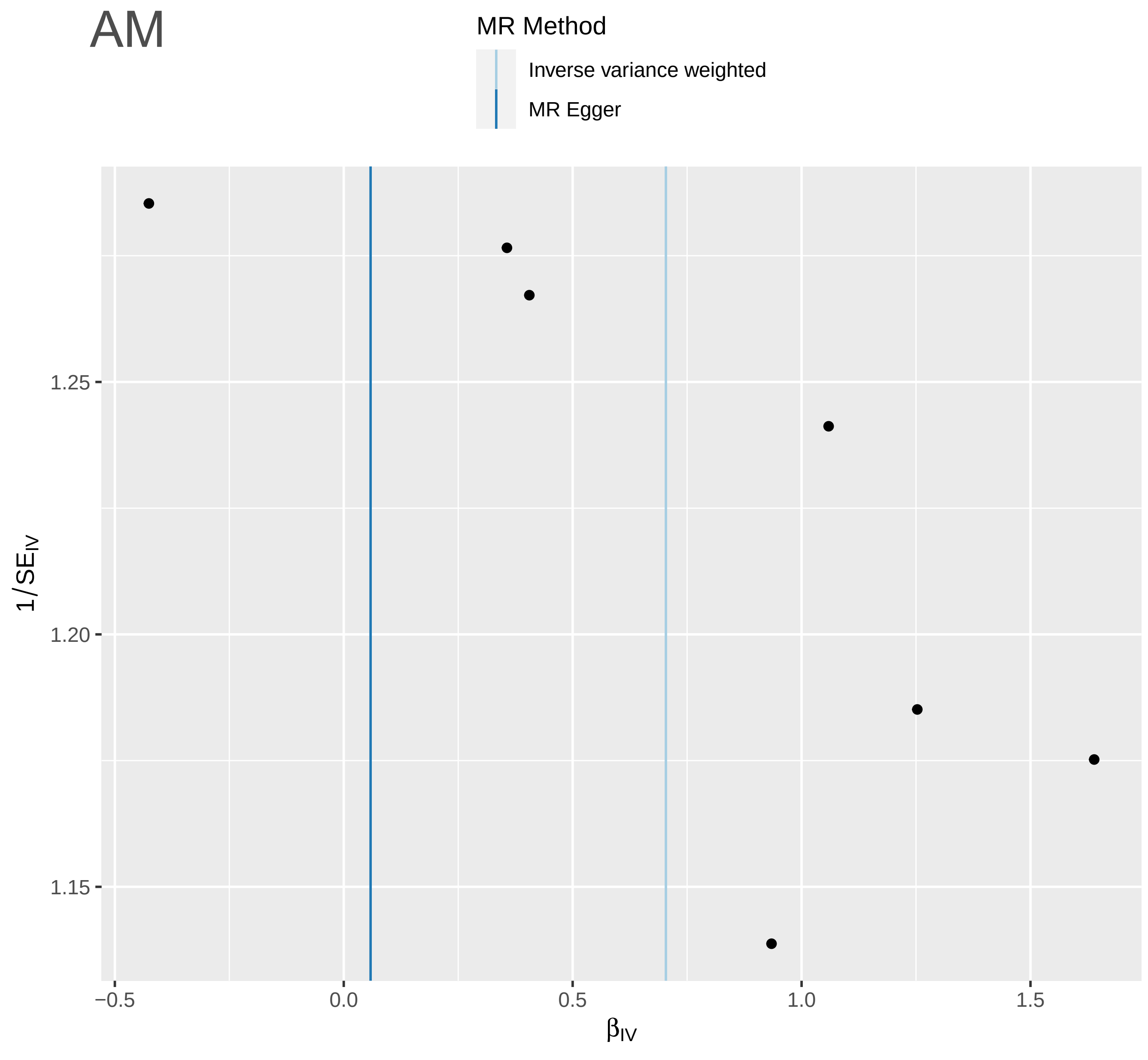

AM
MR Method
Inverse variance weighted
MR Egger
1.25
1.20
1.15
$1/SE_{IV}$
−0.5
0.0
0.5
1.0
1.5
β_{IV}

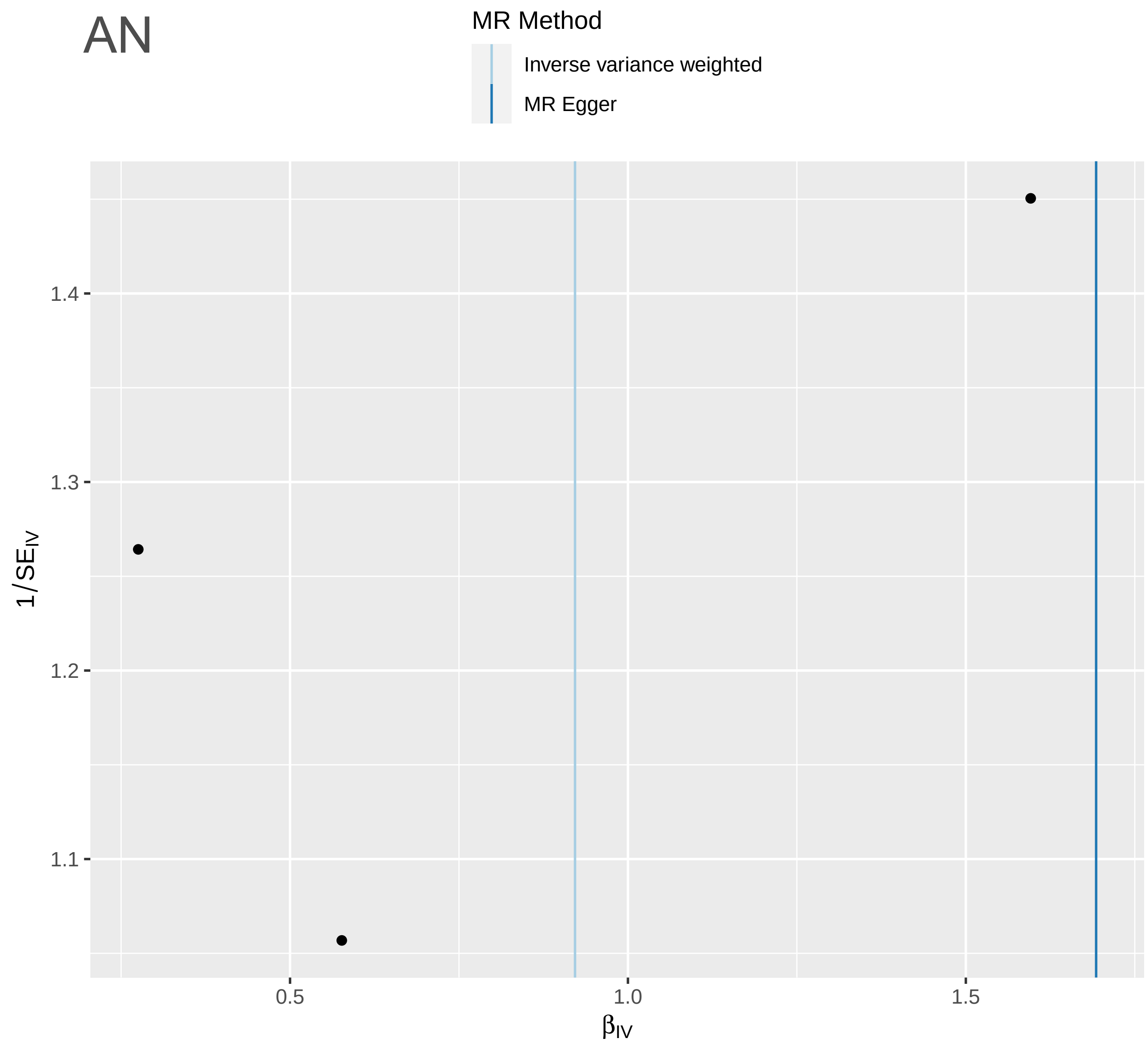
AN
MR Method
Inverse variance weighted
MR Egger
1.4
1.3
1.2
1.1
0.5
1.0
1.5
$1/SE_{IV}$
β_{IV}

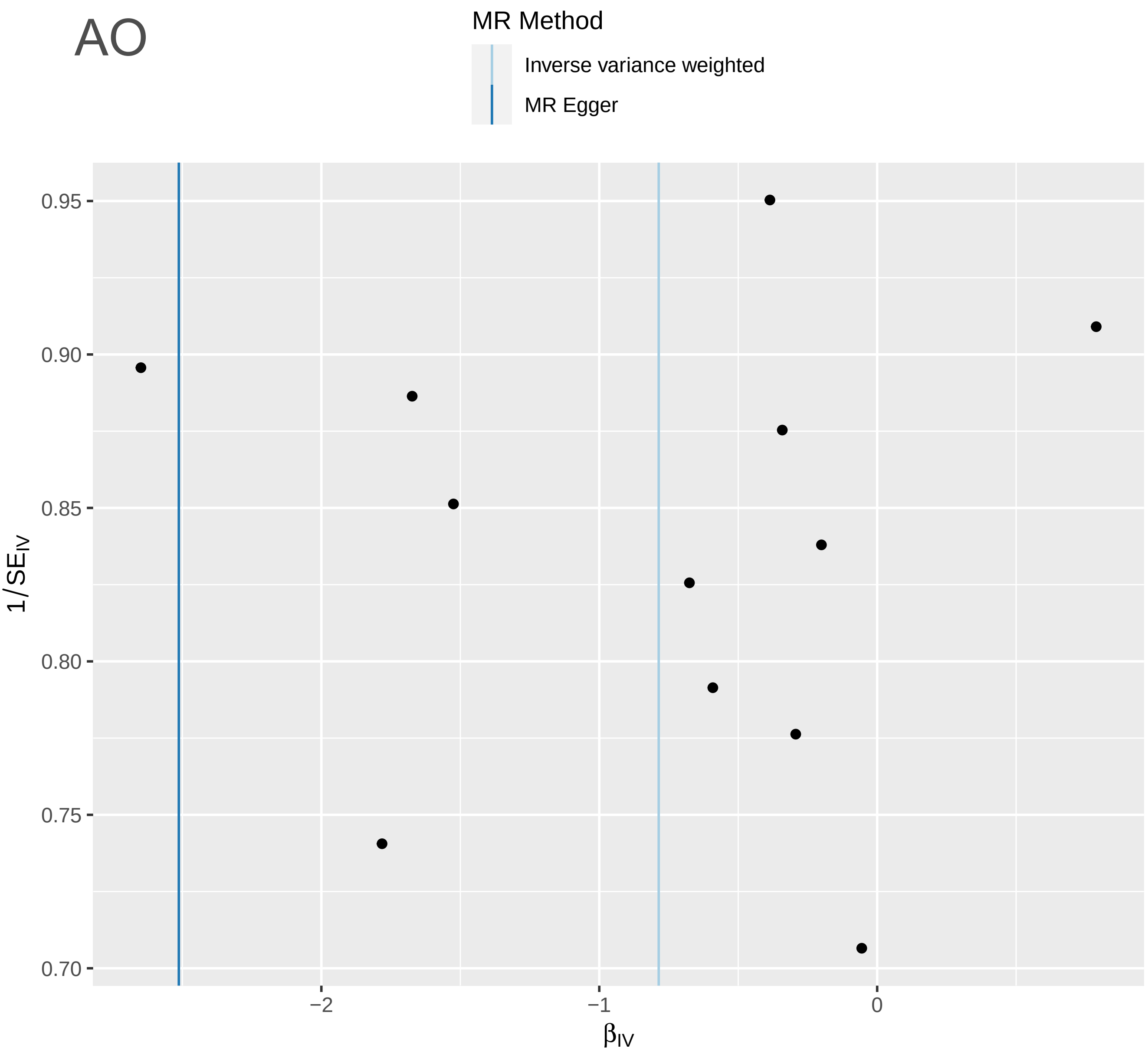
AO
MR Method
Inverse variance weighted
MR Egger
0.95
0.90
0.85
0.80
0.75
0.70
1/SE$_{IV}$
−2
−1
0
β$_{IV}$

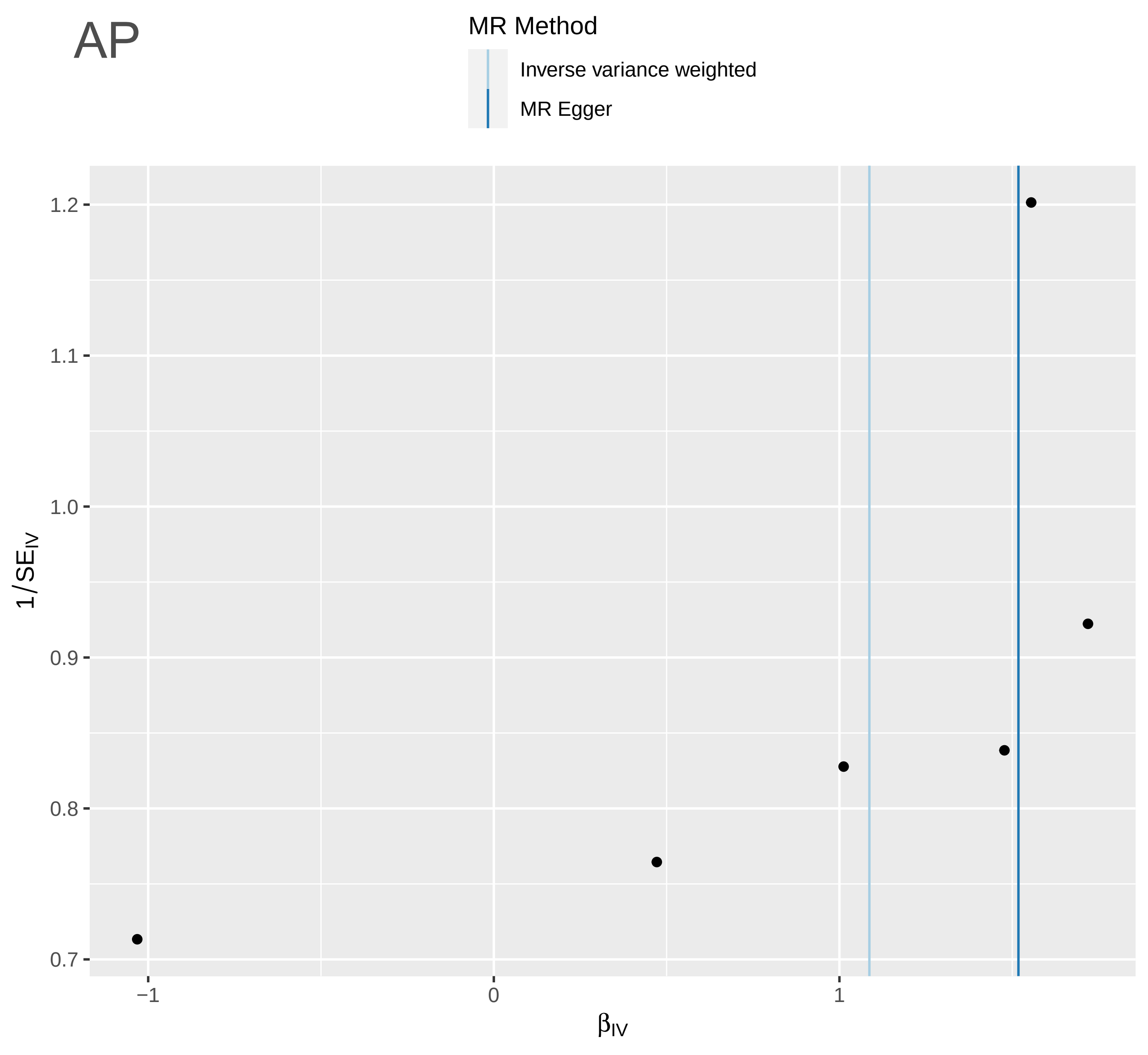
AP
MR Method
Inverse variance weighted
MR Egger
1.2
1.1
1.0
0.9
0.8
0.7
1/SE_IV
−1
0
1
β_IV

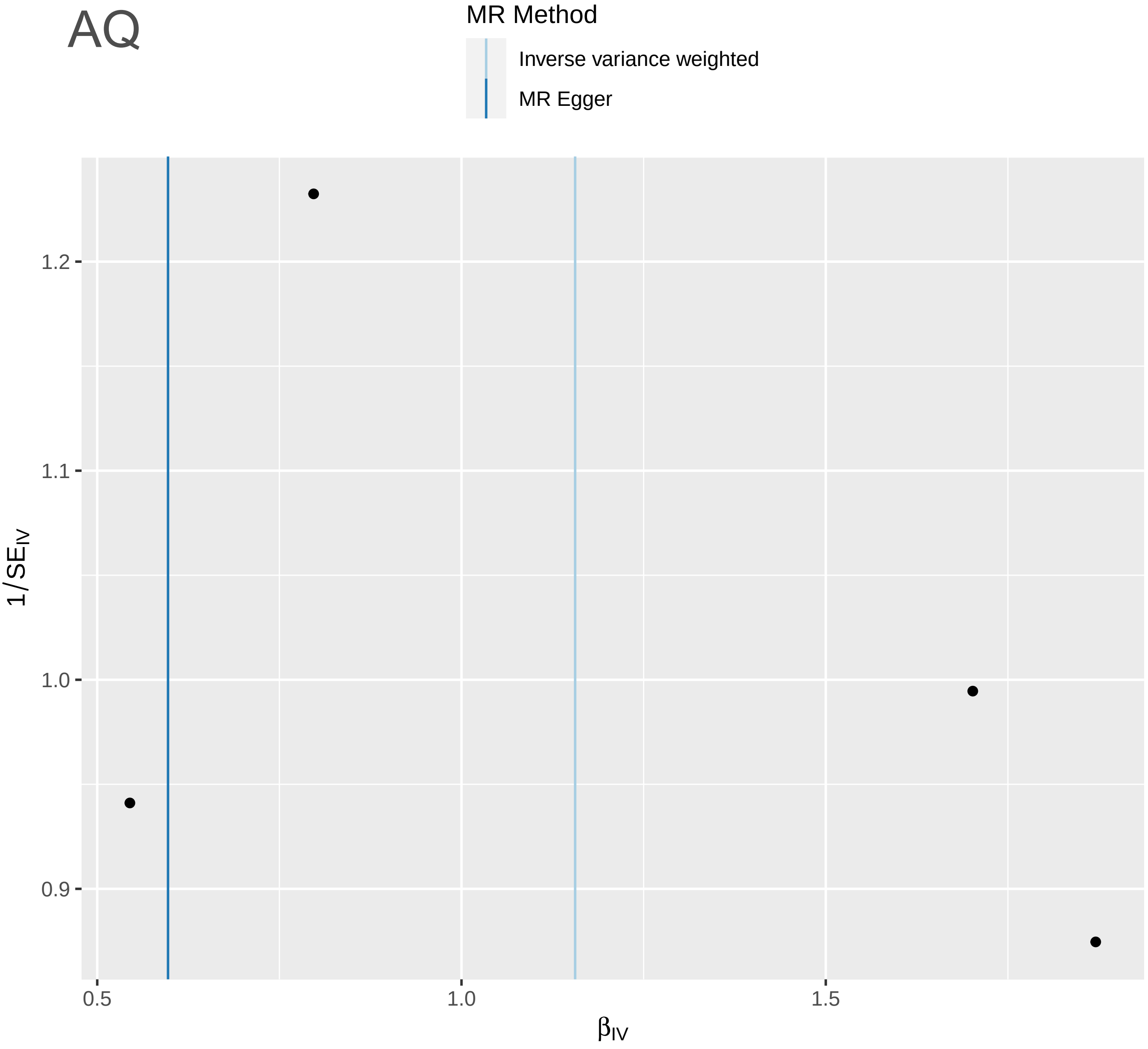
AQ
MR Method
Inverse variance weighted
MR Egger
1.2
1.1
1.0
0.9
$1/SE_{IV}$
0.5
1.0
1.5
β_{IV}

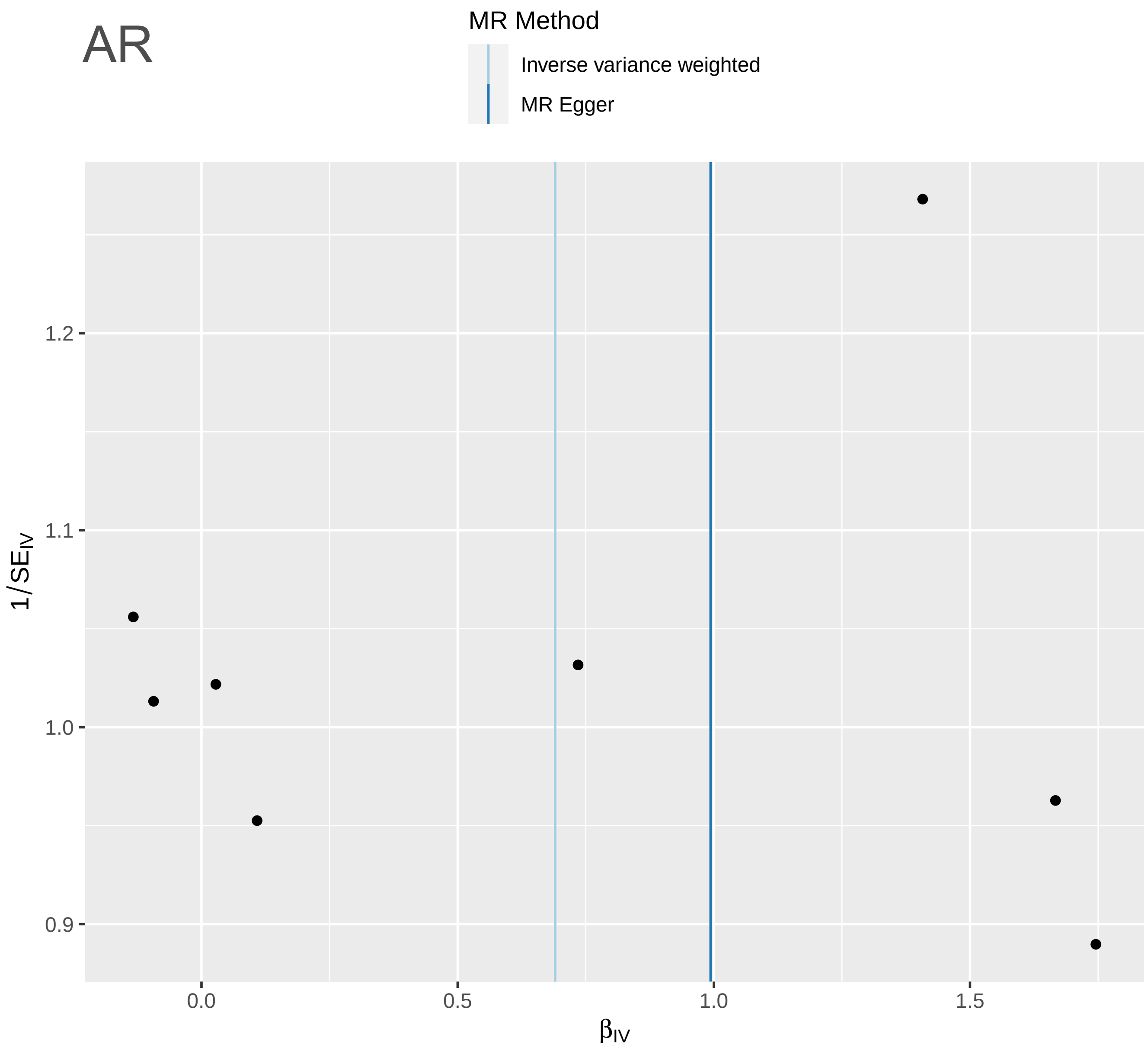

AR
MR Method
Inverse variance weighted
MR Egger
1.2
1.1
1.0
0.9
$1/SE_{IV}$
0.0
0.5
1.0
1.5
β_{IV}

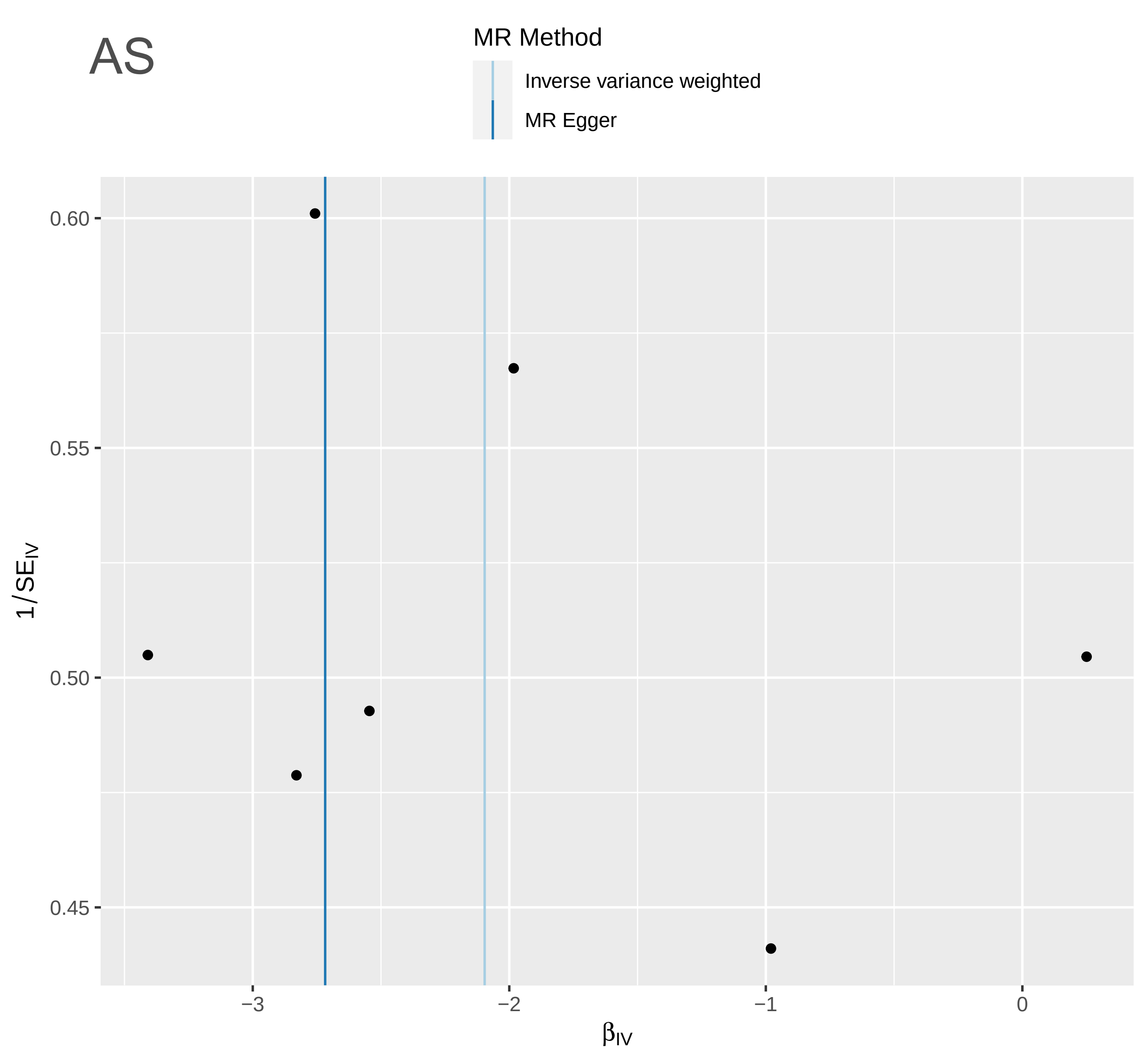
AS
MR Method
Inverse variance weighted
MR Egger
0.60
0.55
0.50
0.45
1/SE_IV
−3
−2
−1
0
β_IV

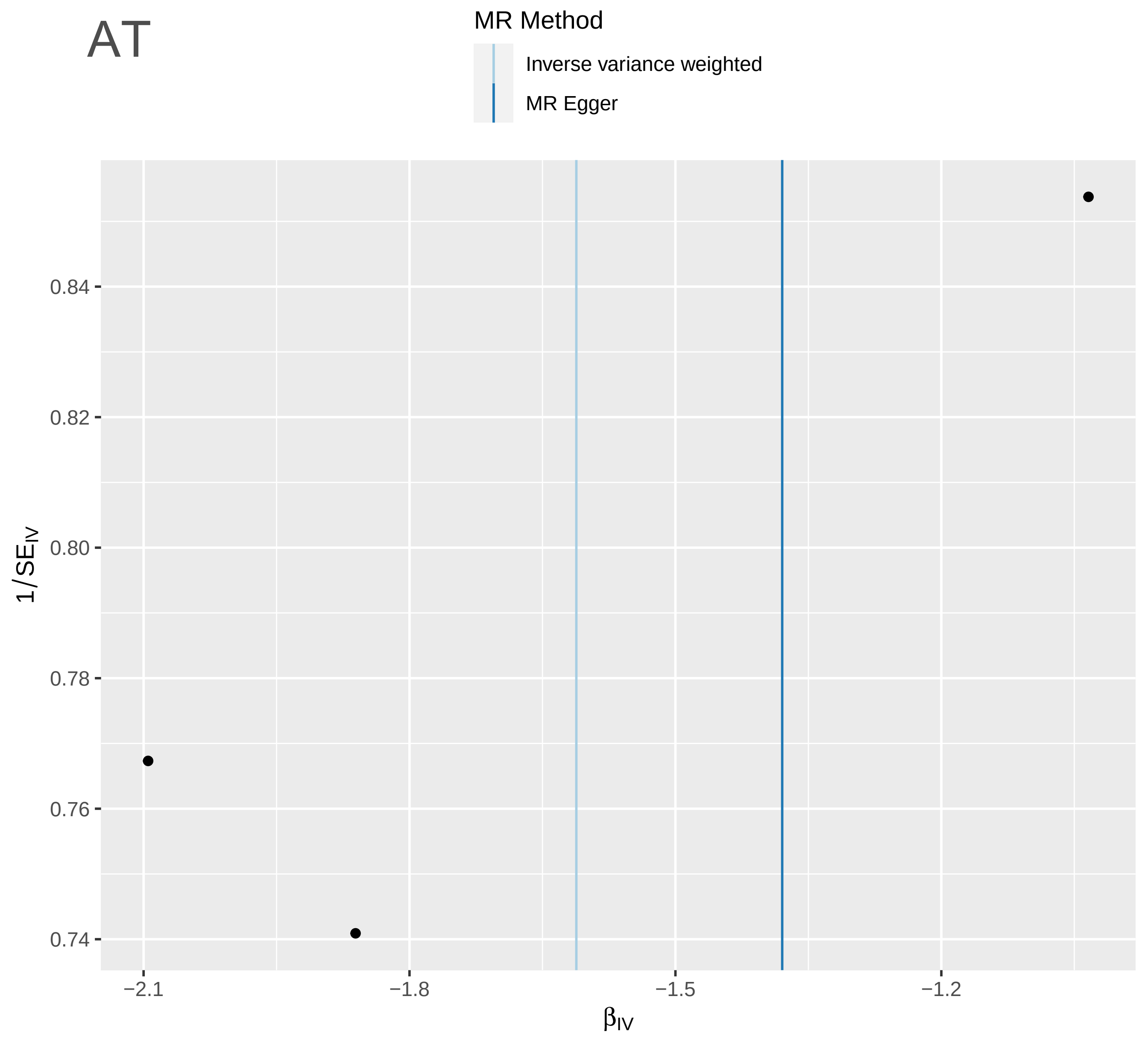

AT
MR Method
Inverse variance weighted
MR Egger
0.84
0.82
0.80
0.78
0.76
0.74
1/SE_IV
−2.1
−1.8
−1.5
−1.2
β_IV

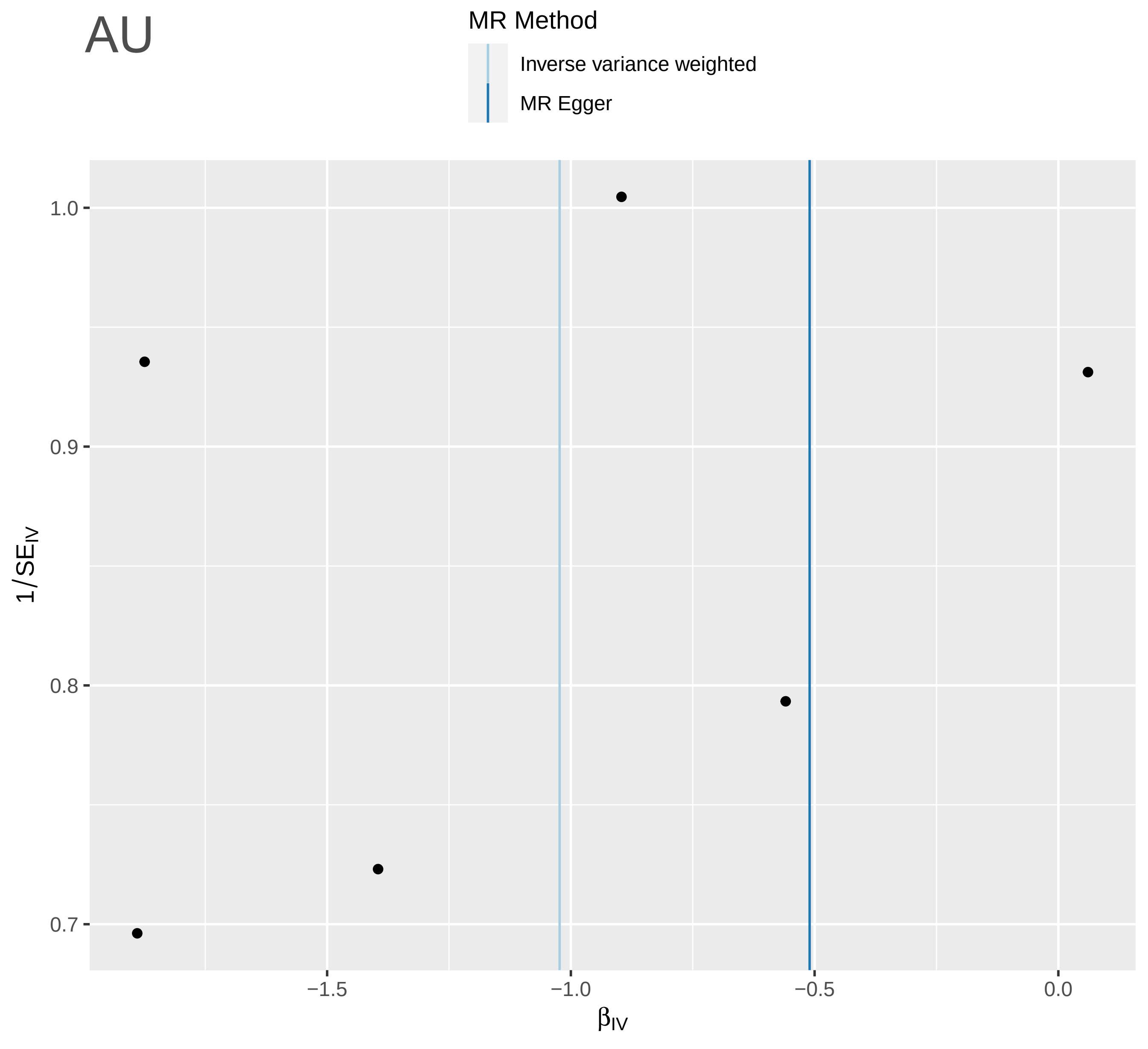

AU
MR Method
Inverse variance weighted
MR Egger
1.0
0.9
0.8
0.7
1/SE_IV
−1.5
−1.0
−0.5
0.0
β_IV

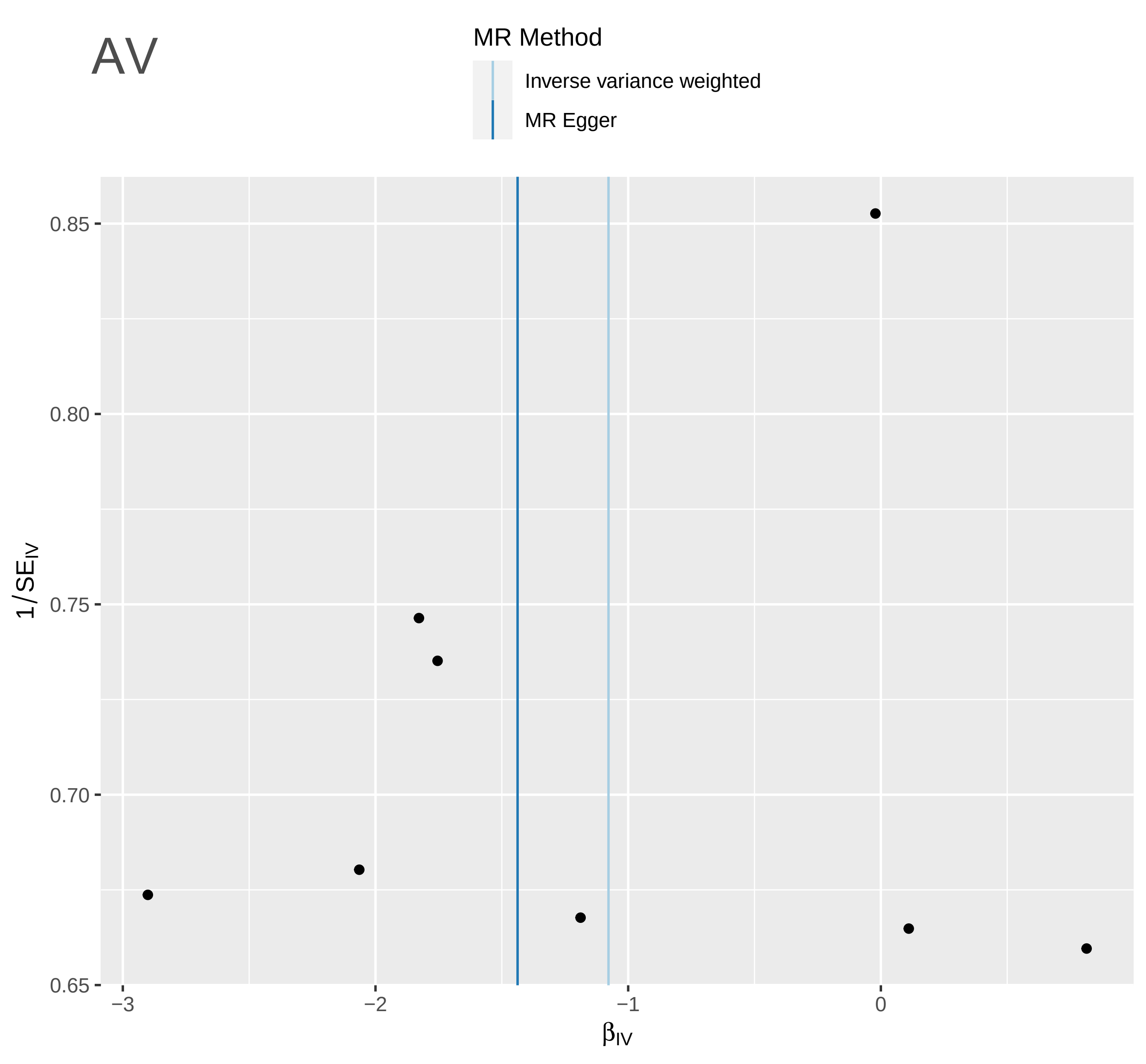
AV
MR Method
Inverse variance weighted
MR Egger
0.85
0.80
0.75
0.70
0.65
1/SE_IV
−3
−2
−1
0
β_IV

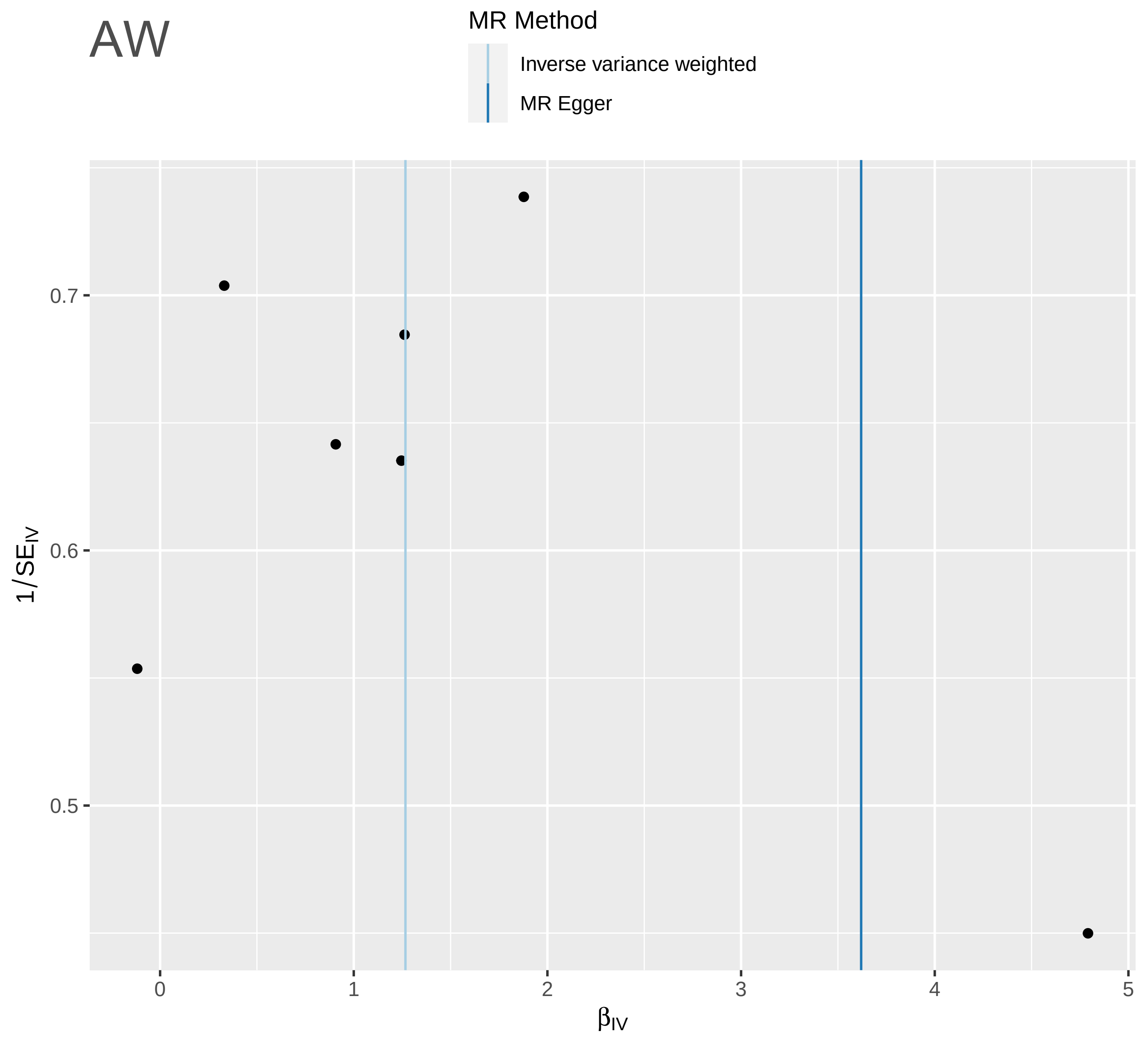

AW
MR Method
Inverse variance weighted
MR Egger
0.7
0.6
0.5
1/SE$_{IV}$
0
1
2
3
4
5
β$_{IV}$

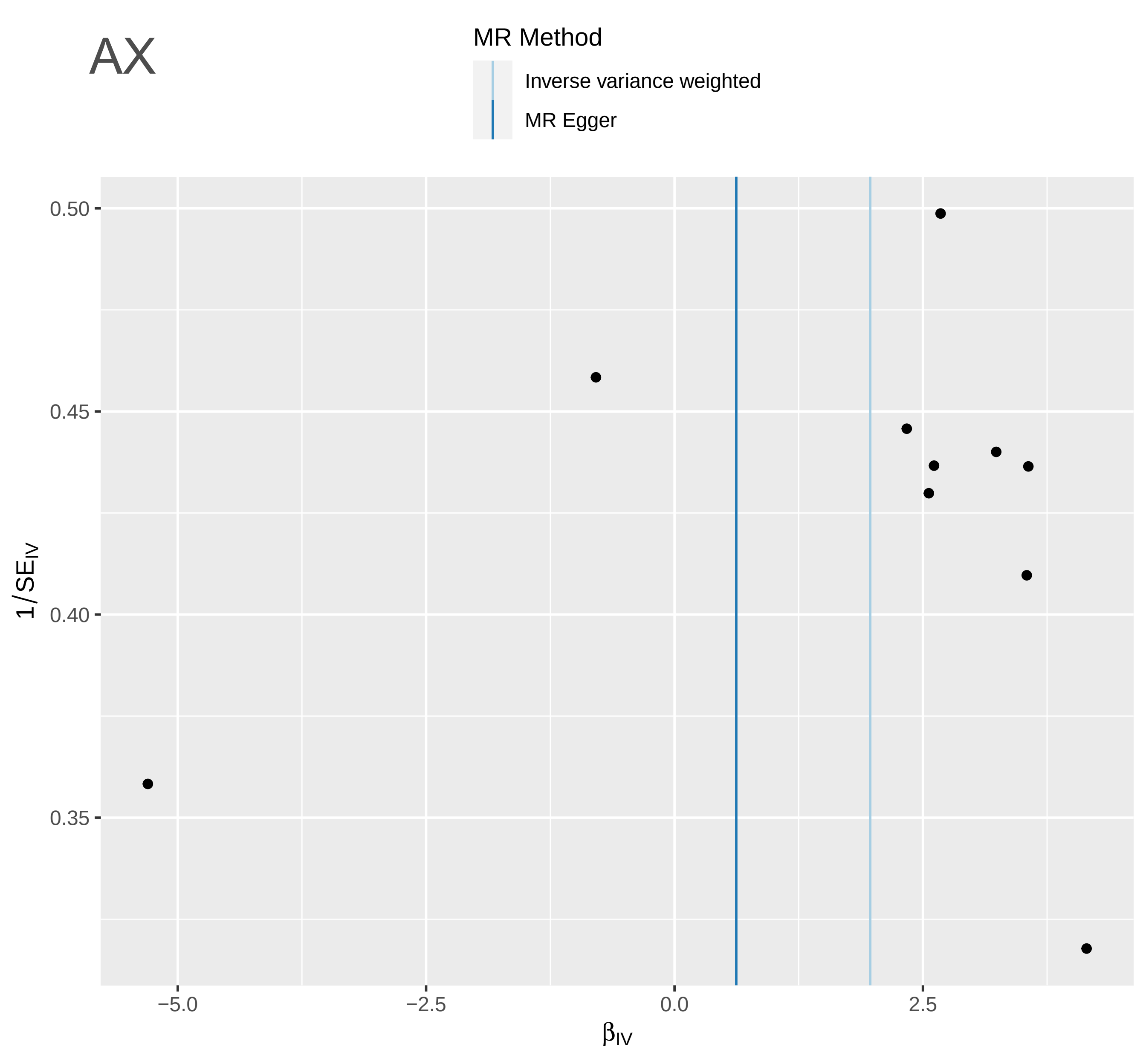
AX
MR Method
Inverse variance weighted
MR Egger
0.50
0.45
0.40
0.35
1/SE_IV
−5.0
−2.5
0.0
2.5
β_IV

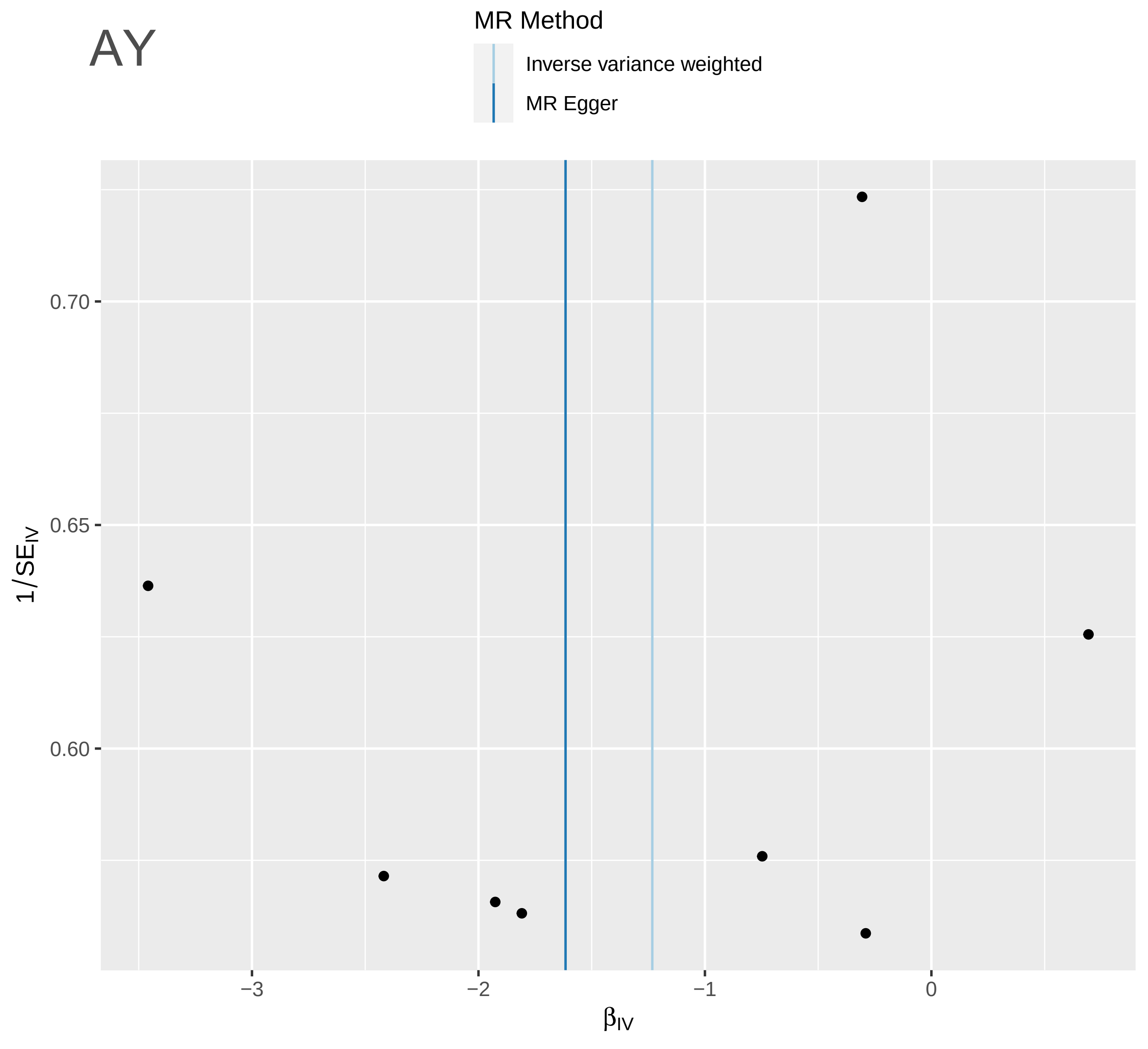
AY
MR Method
Inverse variance weighted
MR Egger
0.70
0.65
0.60
1/SE$_{IV}$
−3
−2
−1
0
β$_{IV}$

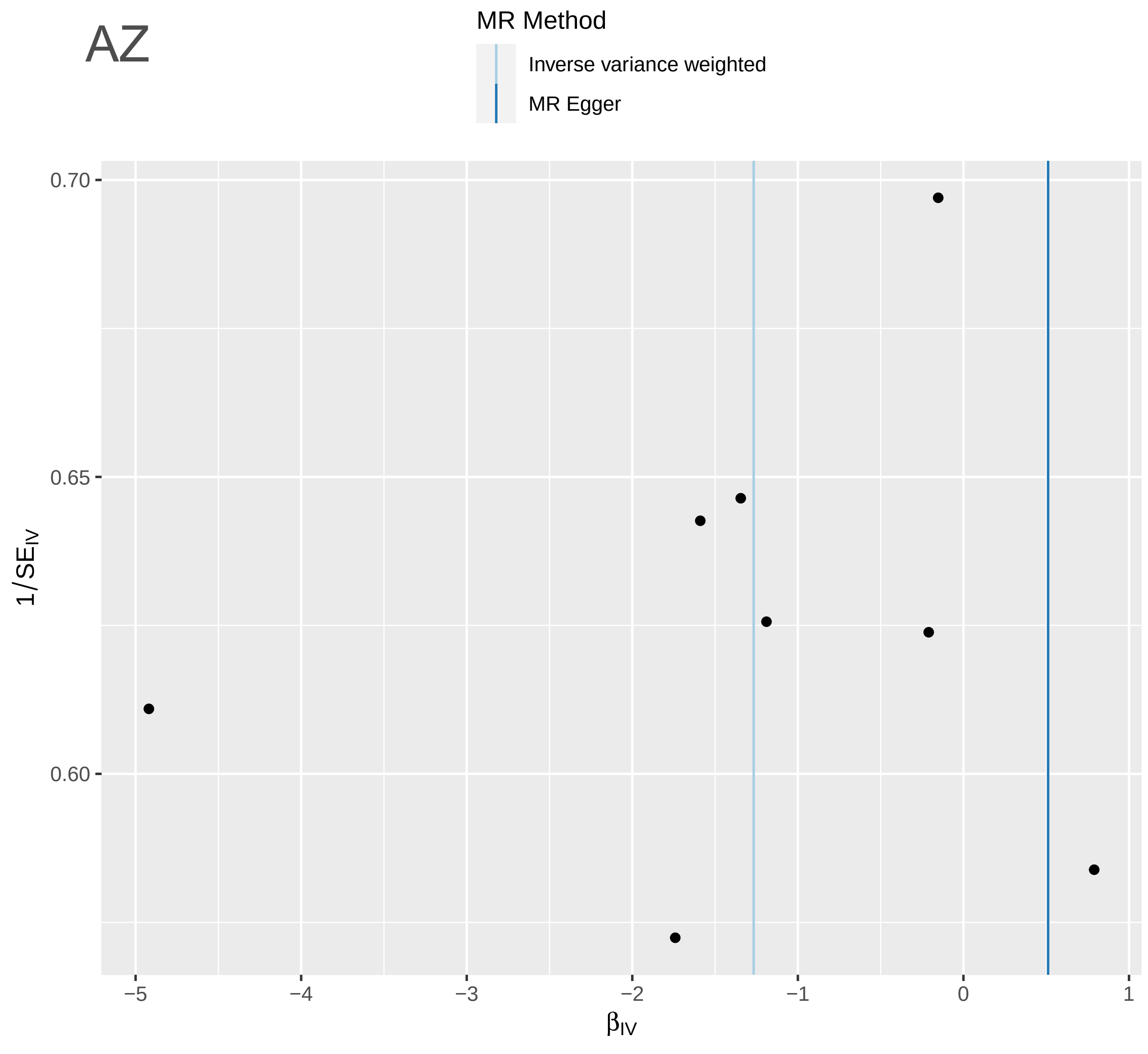
AZ
MR Method
Inverse variance weighted
MR Egger
0.70
0.65
0.60
1/SE$_{IV}$
−5
−4
−3
−2
−1
0
1
β$_{IV}$

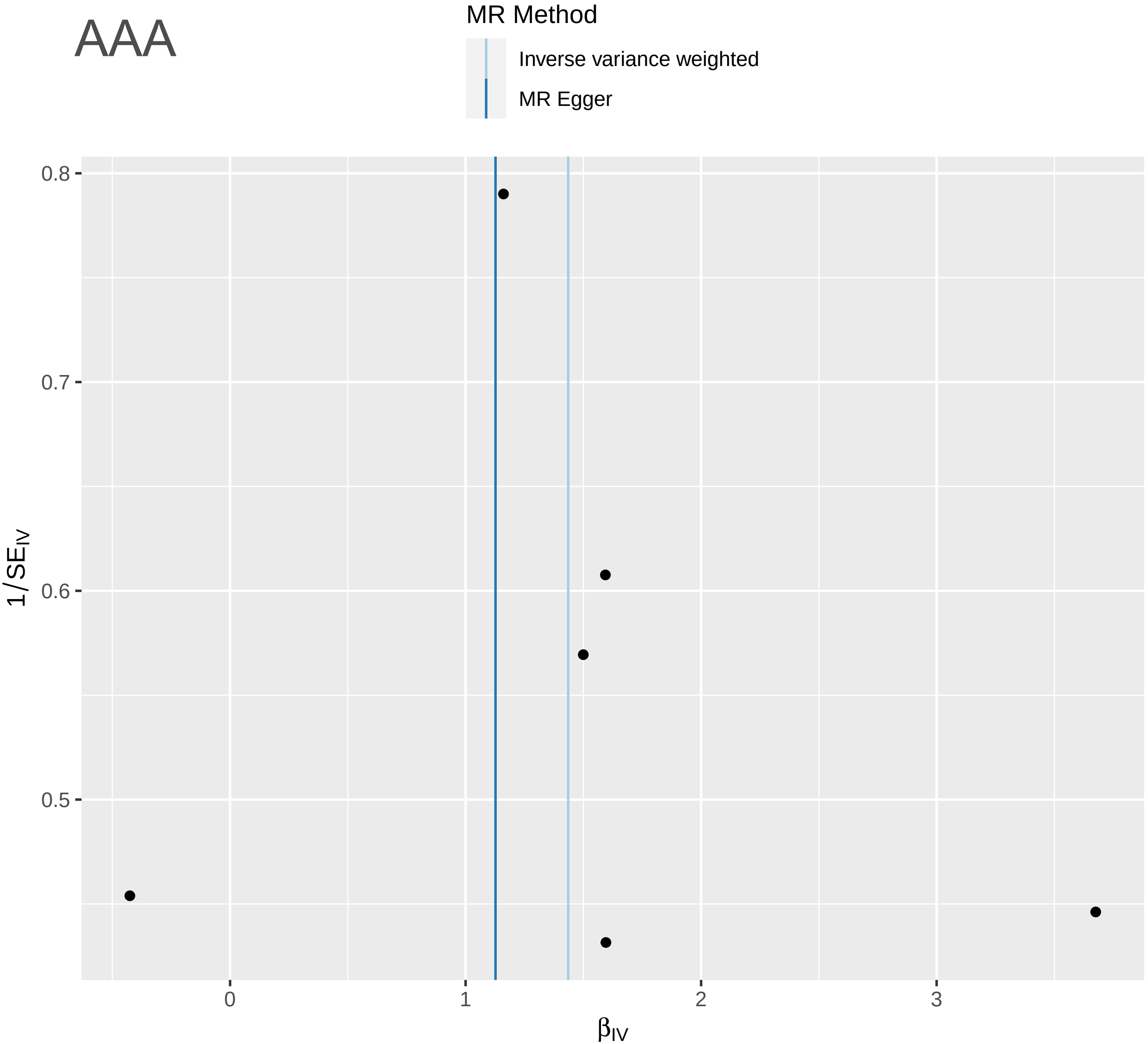
AAA
MR Method
Inverse variance weighted
MR Egger
0.8
0.7
0.6
0.5
1/SE_IV
0
1
2
3
β_IV

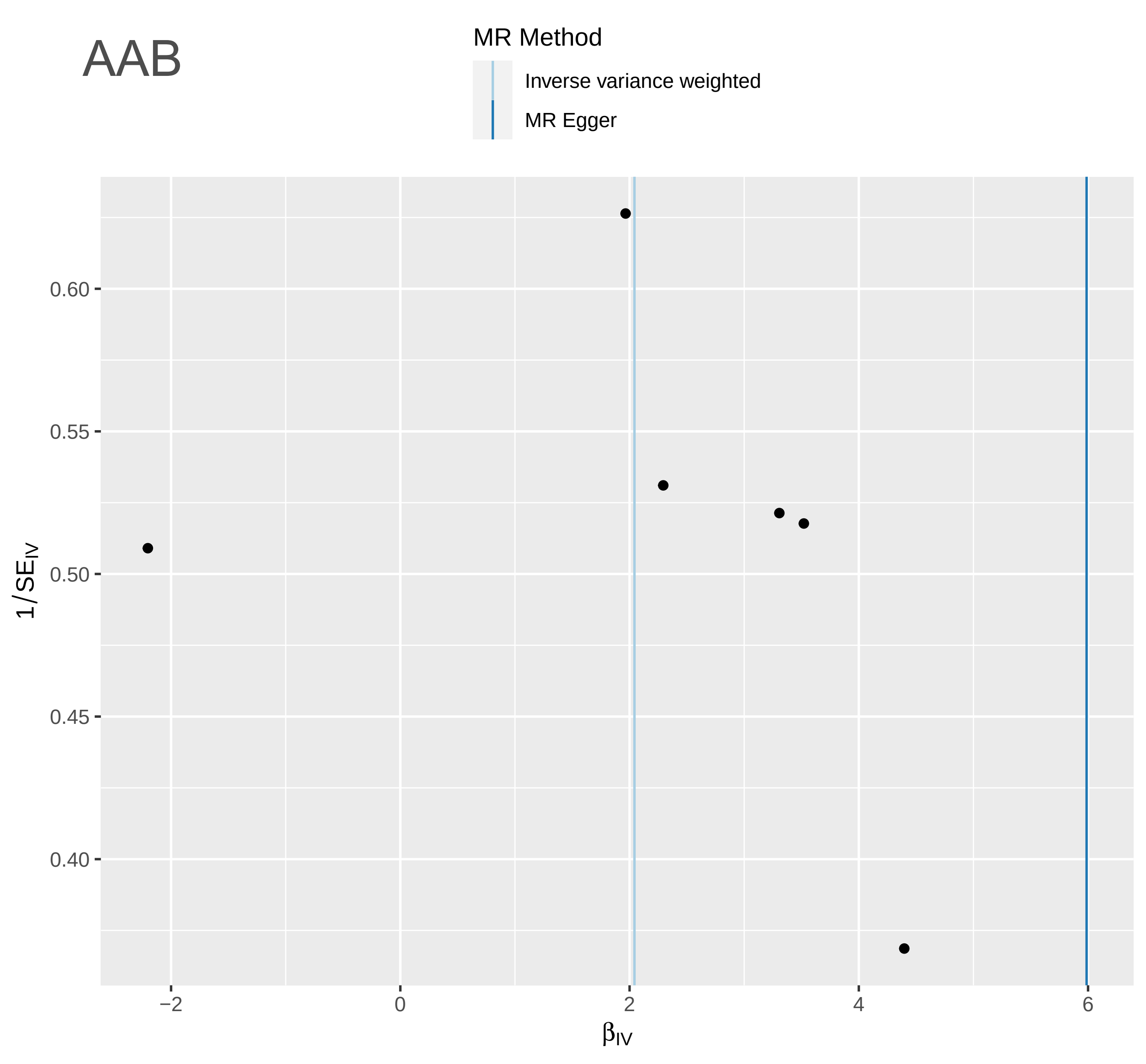

AAB
MR Method
Inverse variance weighted
MR Egger
0.60
0.55
0.50
0.45
0.40
1/SE_IV
−2
0
2
4
6
β_IV

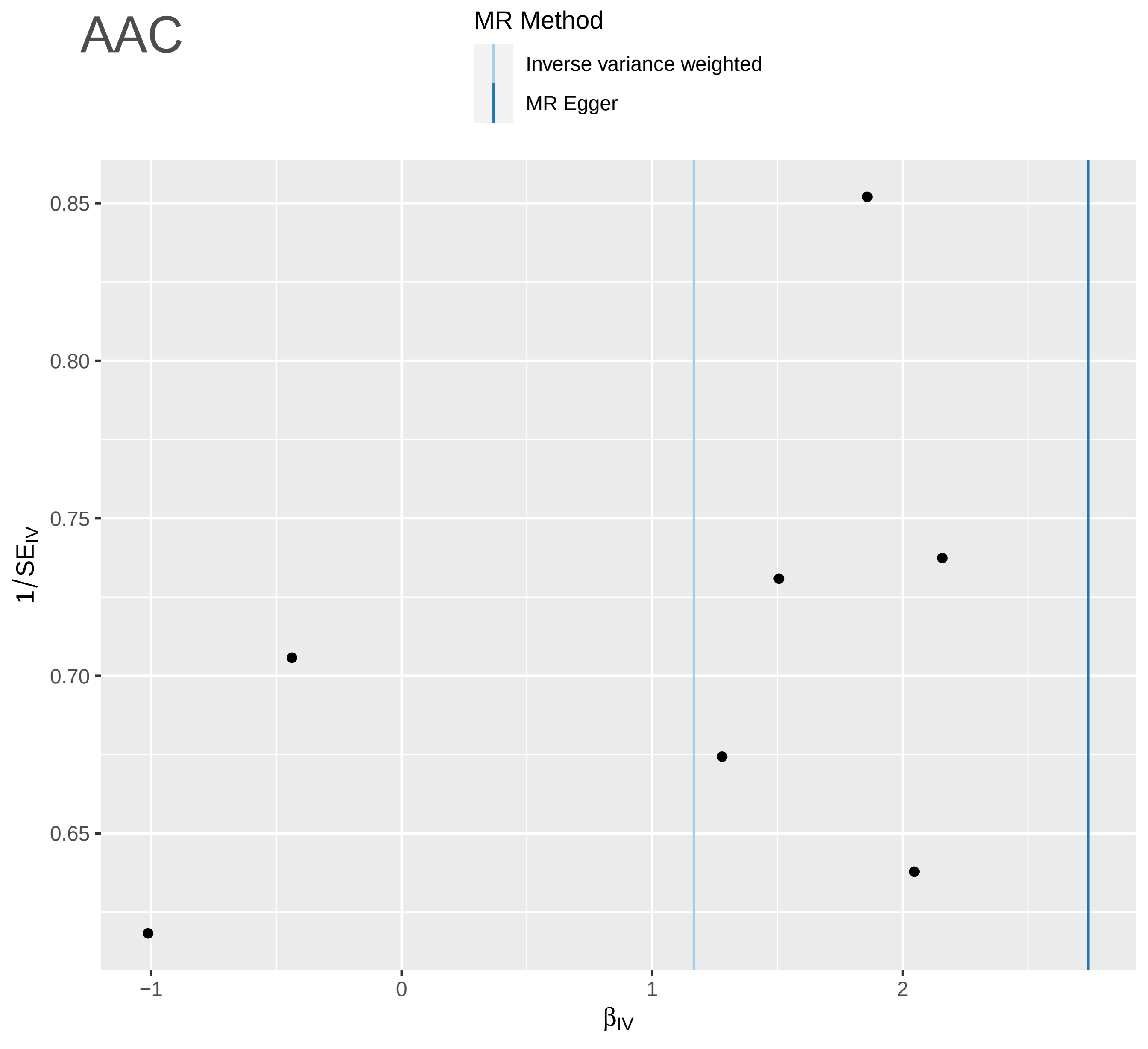
AAC
MR Method
Inverse variance weighted
MR Egger
0.85
0.80
0.75
0.70
0.65
1/SE_{IV}
−1
0
1
2
$β_{IV}$

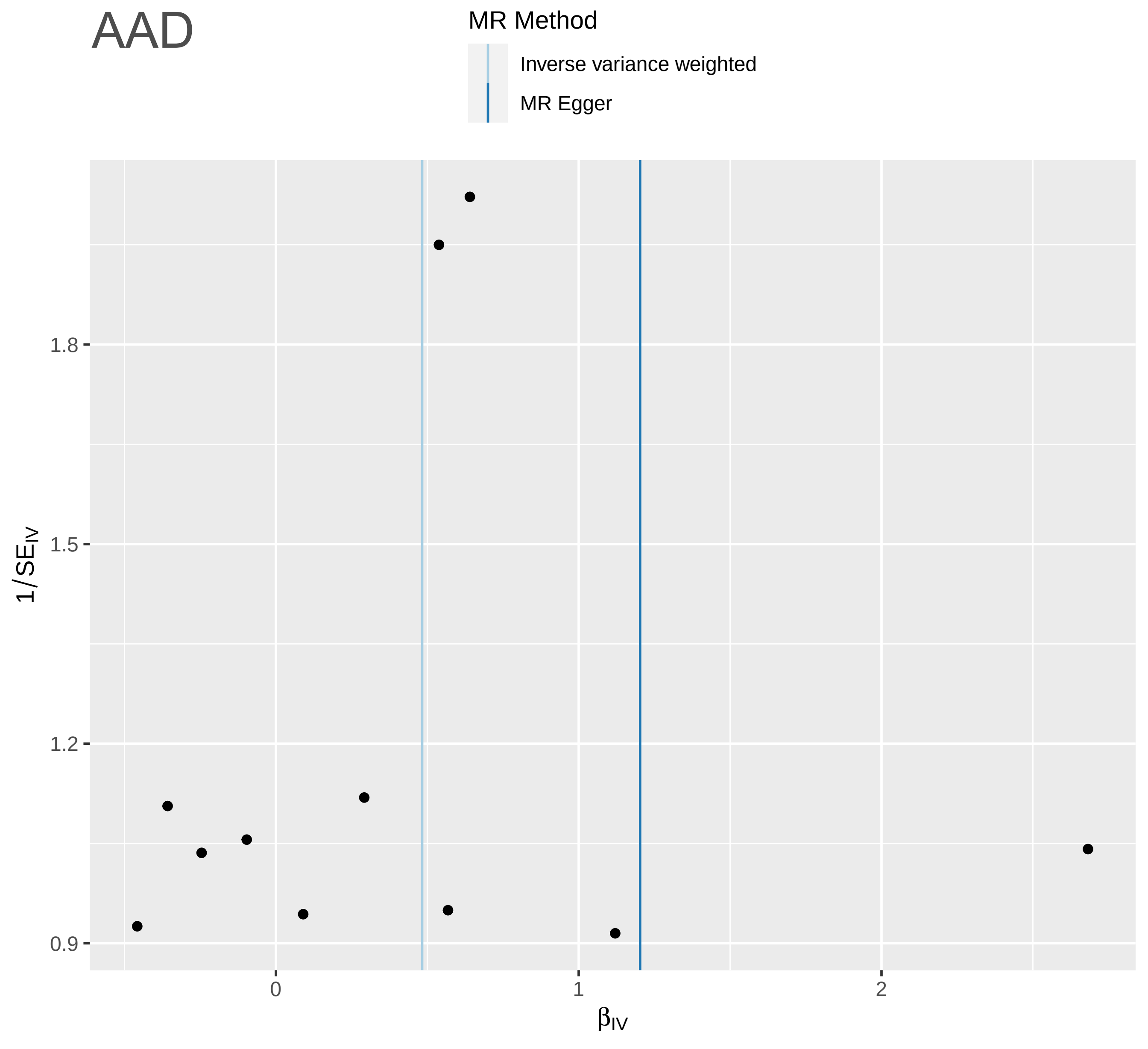

AAD
MR Method
Inverse variance weighted
MR Egger
1.8
1.5
1.2
0.9
1/SE_IV
0
1
2
β_IV

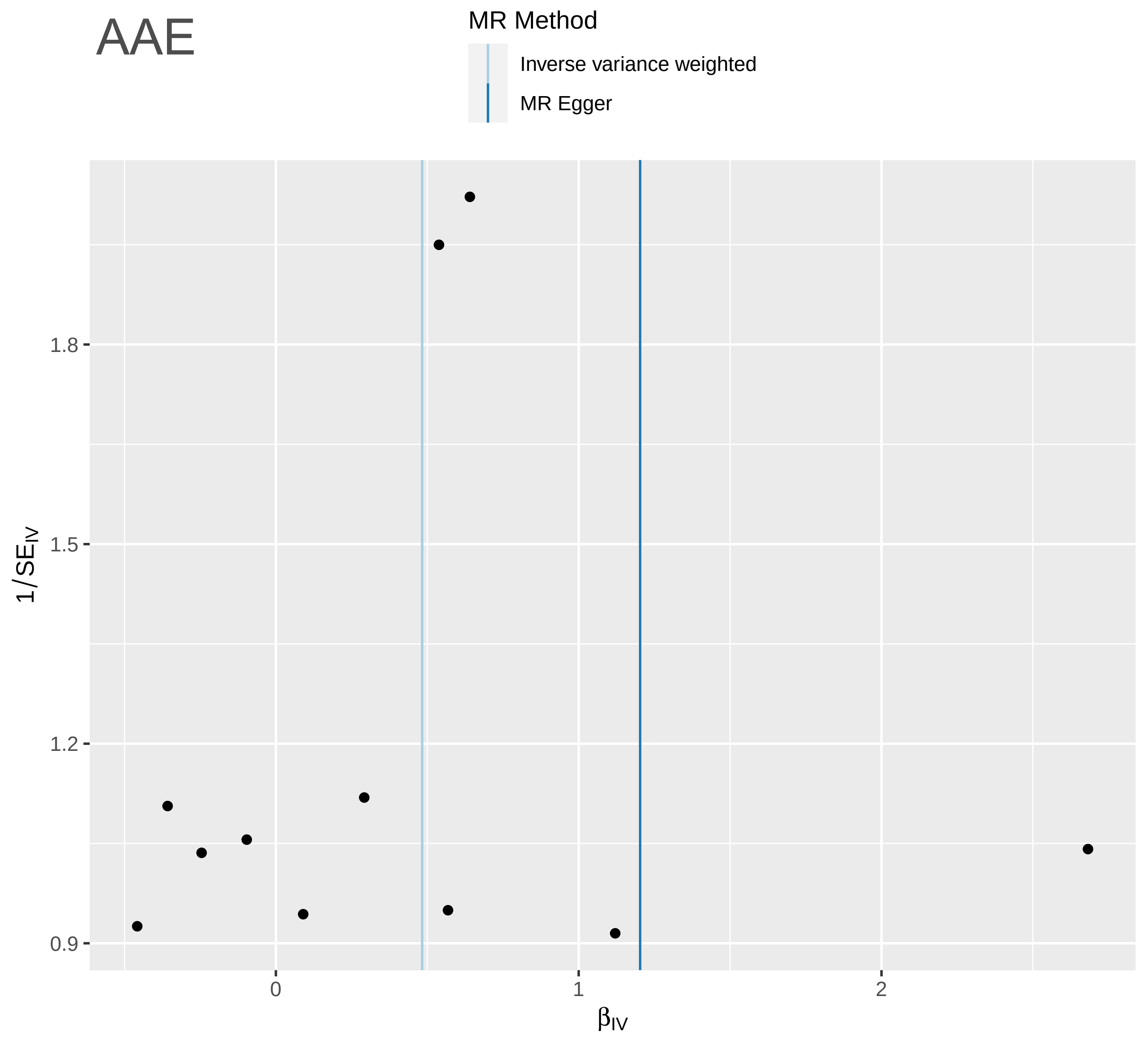
AAE
MR Method
Inverse variance weighted
MR Egger
1.8
1.5
1.2
0.9
0
1
2
$1/SE_{IV}$
β_{IV}

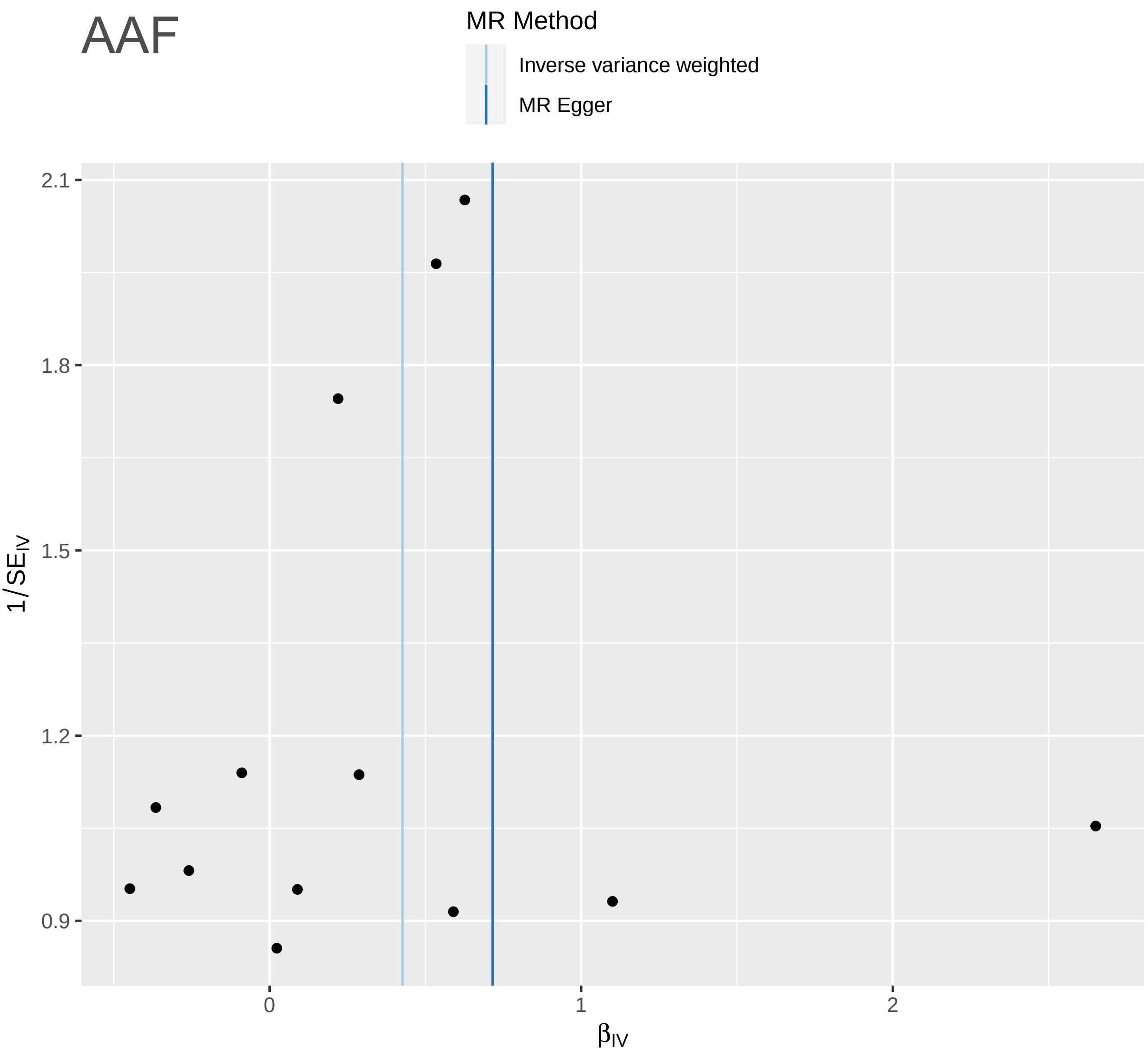
AAF
MR Method
Inverse variance weighted
MR Egger
2.1
1.8
1.5
1.2
0.9
1/SE_IV
0
1
2
β_IV

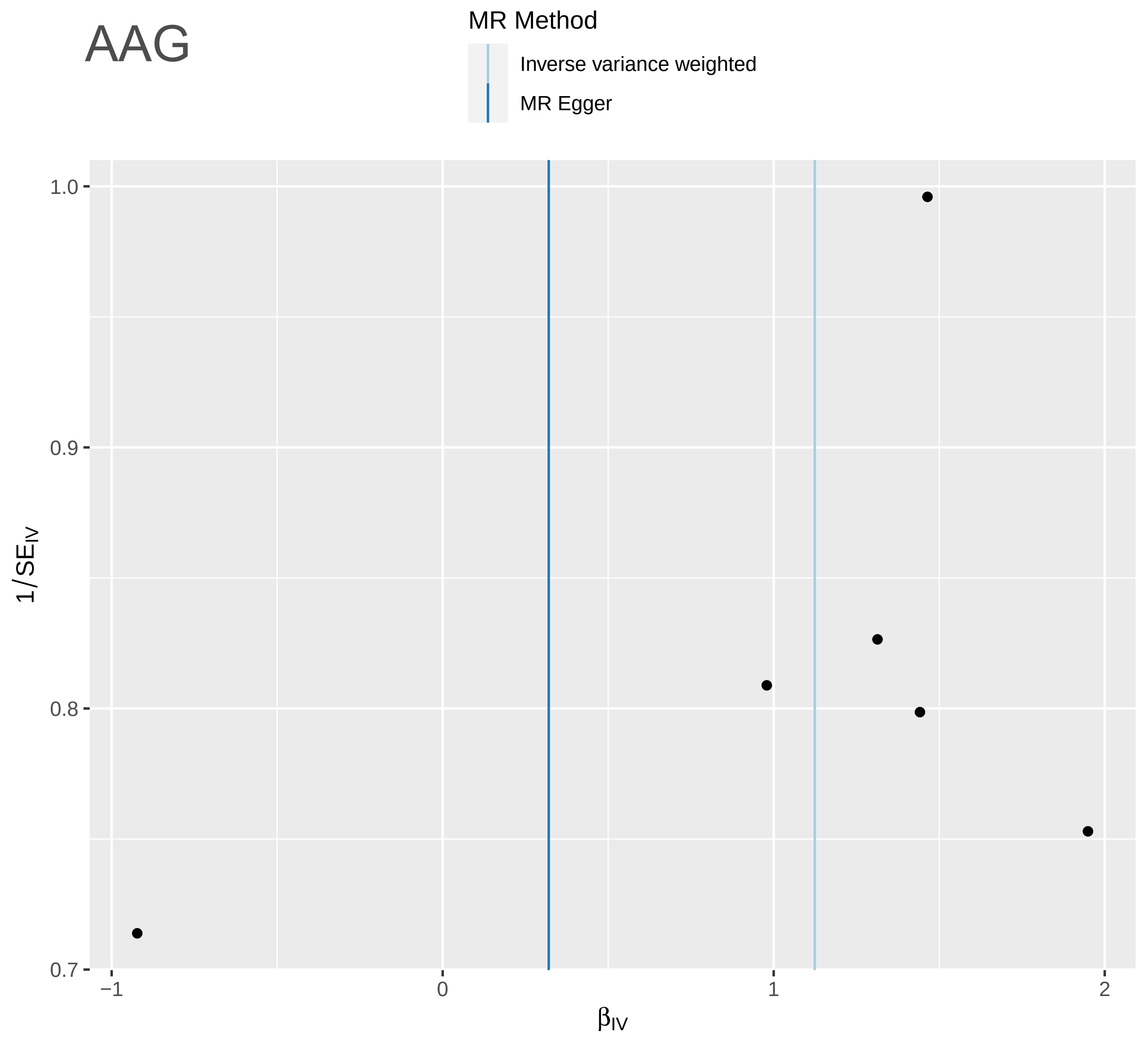

AAG
MR Method
Inverse variance weighted
MR Egger
1.0
0.9
0.8
0.7
−1
0
1
2
$1/SE_{IV}$
β_{IV}

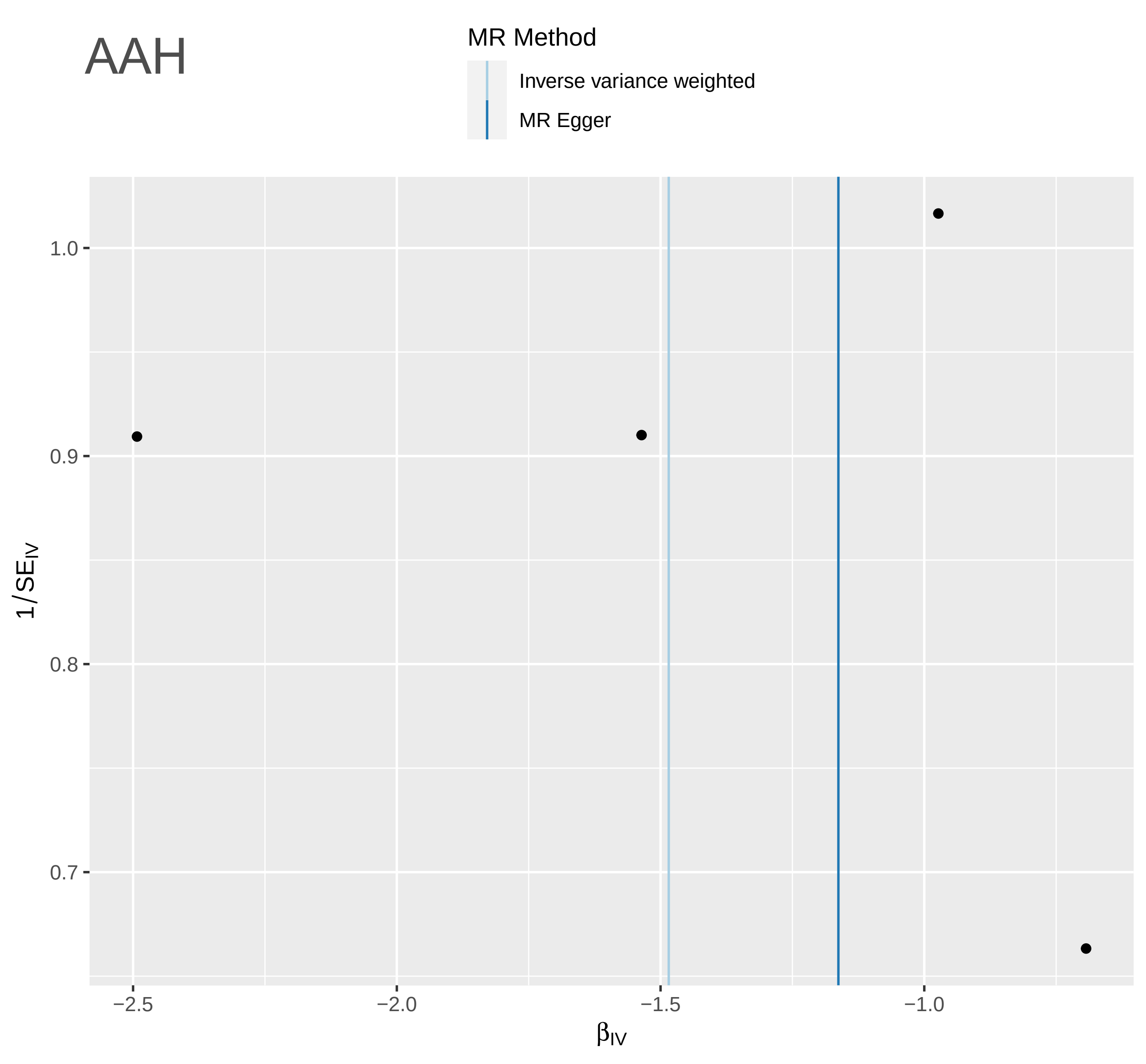
AAH
MR Method
Inverse variance weighted
MR Egger
1.0
0.9
0.8
0.7
1/SE_IV
−2.5
−2.0
−1.5
−1.0
β_IV

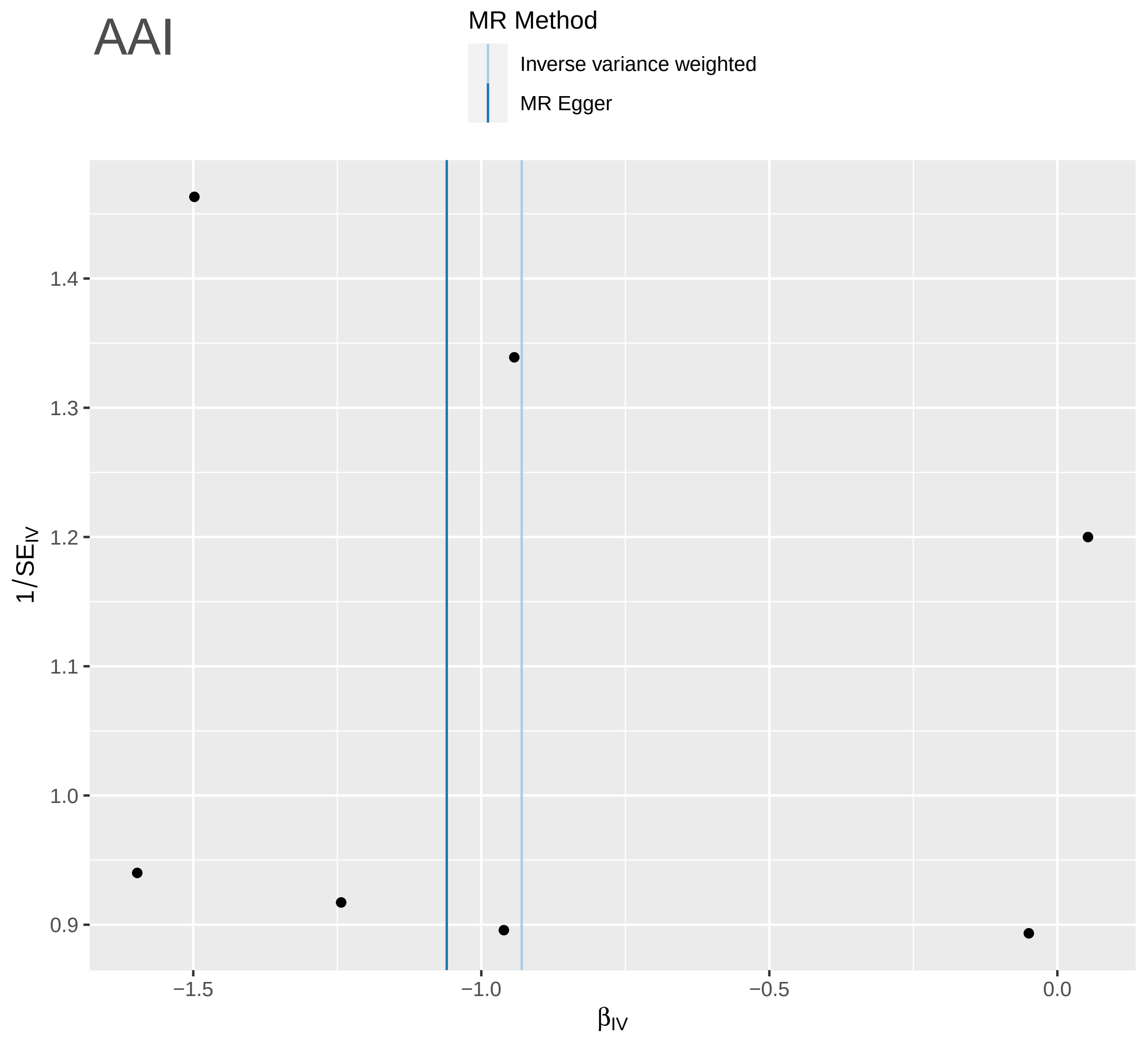

AAI
MR Method
Inverse variance weighted
MR Egger
1.4
1.3
1.2
1.1
1.0
0.9
$1/SE_{IV}$
−1.5
−1.0
−0.5
0.0
β_{IV}

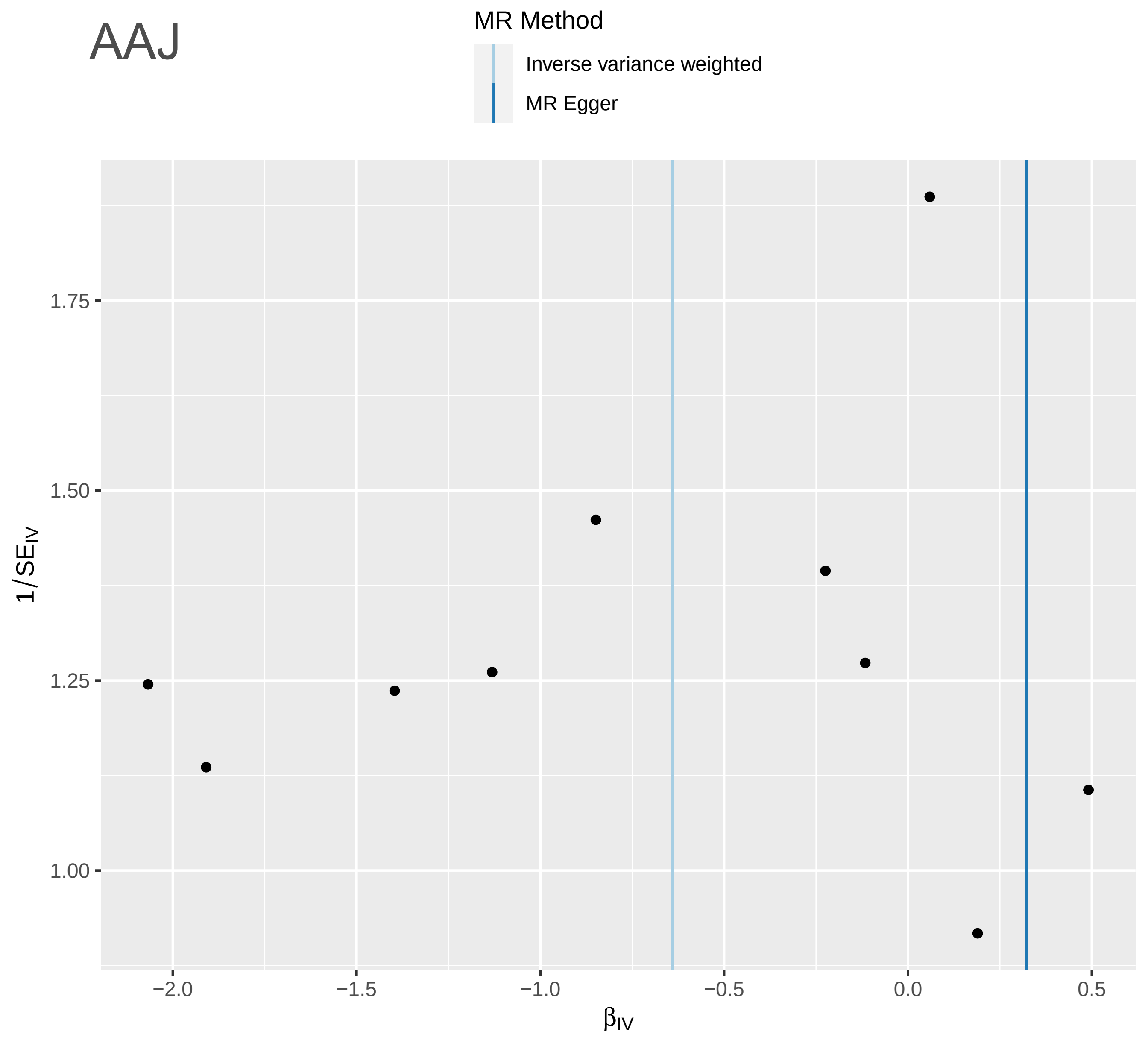

AAJ
MR Method
Inverse variance weighted
MR Egger
1.75
1.50
1.25
1.00
1/SE_IV
−2.0
−1.5
−1.0
−0.5
0.0
0.5
β_IV

Supplementary figure 2.Sensitivity analysis of the impact of gut microbiota on hematologic malignancies using MR. (funnel plot)

A.Funnel plot between genus.FamilyXIIIUCG001 and lymphoid leukemia

B.Funnel plot between family.Peptococcaceae and lymphoid leukemia**.**

C.Funnel plot between family.Desulfovibrionaceae and lymphoid leukemia

D.Funnel plot between genus..Clostridiuminnocuumgroup and lymphoid leukemia.

E.Funnel plot between genus.Holdemania and lymphoid leukemia.

F.Funnel plot between family.Prevotellaceae and lymphoid leukemia.

G.Funnel plot between genus.Ruminiclostridium9. and lymphoid leukemia.

H.Funnel plot between genus.Methanobrevibacter and lymphoid leukemia.

I.Funnel plot between order.Desulfovibrionales. and lymphoid leukemia.

J.Funnel plot between genus.Coprococcus3 and lymphoid leukemia.

K.Funnel plot between genus.Ruminiclostridium6 and lymphoid leukemia.

L.Funnel plot between class.Negativicutes and lymphoid leukemia.

M.Funnel plot between order.Selenomonadales. and lymphoid leukemia.

N.Funnel plot between family.Lactobacillaceae. and lymphoid leukemia.

O.Funnel plot between class.Methanobacteria and lymphoid leukemia.

P.Funnel plot between family.Methanobacteriaceae and lymphoid leukemia.

Q.Funnel plot between order.Methanobacteriales and lymphoid leukemia.

R.Funnel plot between phylum.Cyanobacteria and lymphoid leukemia**.**

S.Funnel plot between order.Coriobacteriales and myeloid leukemia**.**

T.Funnel plot between family.Coriobacteriaceae. and myeloid leukemia**.**

U.Funnel plot between genus.LachnospiraceaeUCG008 and myeloid leukemia.

V.Funnel plot between class.Coriobacteriia. and myeloid leukemia**.**

W.Funnel plot between genus.Turicibacter and myeloid leukemia**.**

X.Funnel plot between genus.Slackia. and myeloid leukemia.

Y.Funnel plot between class.Gammaproteobacteria and myeloid leukemia.

Z.Funnel plot between genus.Prevotella9 and myeloid leukemia.

AA. Funnel plot between genus.Dorea and myeloid leukemia.

AB.Funnel plot between genus.Peptococcus. and Hodgkin lymphoma

AC.Funnel plot between class.Gammaproteobacteria and Hodgkin lymphoma.

AD.Funnel plot between genus.RuminococcaceaeUCG014 and malignant plasma cell tumor**.**

AE.Funnel plot between genus.LachnospiraceaeUCG010. and malignant plasma cell tumor.

AF.Funnel plot between class.Lentisphaeria and malignant plasma cell tumor.

AG.Funnel plot between genus.Lactococcus. and malignant plasma cell tumor.

AH.Funnel plot between order.Victivallales. and malignant plasma cell tumor.

AI.Funnel plot between genus.Romboutsia and malignant plasma cell tumor.

AJ.Funnel plot between genus.Dorea and malignant plasma cell tumor.

AK.Funnel plot between genus.RikenellaceaeRC9gutgroup and malignant plasma cell tumor**.**

AL. Funnel plot between genus.RuminococcaceaeUCG005 and follicular lymphoma.

AM.Funnel plot between genus.Adlercreutzia and follicular lymphoma.

AN.Funnel plot between order.MollicutesRF9 and follicular lymphoma.

AO.Funnel plot between family.Alcaligenaceae and follicular lymphoma.

AP.Funnel plot between class.Clostridia and follicular lymphoma.

AQ. Funnel plot between genus.Sutterella and follicular lymphoma.

AR.Funnel plot between genus.Phascolarctobacterium and follicular lymphoma.

AS.Funnel plot between genus..Eubacteriumcoprostanoligenesgroup and diffuse large B-cell lymphoma.

AT.Funnel plot between class.Alphaproteobacteria. and diffuse large B-cell lymphoma.

AU.Funnel plot between phylum.Cyanobacter and diffuse large B-cell lymphoma.

AV.Funnel plot between genus.Erysipelatoclostridium and diffuse large B-cell lymphoma.

AW.Funnel plot between family.Rhodospirillaceae. and mature T/NK-cell lymphomas.

AX.Funnel plot between genus.Anaerostipes and mature T/NK-cell lymphomas.

AY.Funnel plot between genus.Erysipelatoclostridium. and mature T/NK-cell lymphomas.

AZ.Funnel plot between genus.LachnospiraceaeUCG001.. and mature T/NK-cell lymphomas.

AAA.Funnel plot between genus..Eubacteriumrectalegroup. and mature T/NK-cell lymphomas.

AAB. Funnel plot between genus.Escherichia.Shigella. and mature T/NK-cell lymphomas.

AAC.Funnel plot between genus..Ruminococcusgnavusgroup and mature T/NK-cell lymphomas.

AAD. Funnel plot between order.Bifidobacteriales. and myeloproliferative neoplasms.

AAE. Funnel plot between family.Bifidobacteriaceae.. and myeloproliferative neoplasms.

AAF. Funnel plot between genus.Bifidobacterium. and myeloproliferative neoplasms.

AAG.Funnel plot between phylum.Firmicutes. and myeloproliferative neoplasms.

AAH.Funnel plot between genus.Coprococcus3. and myeloproliferative neoplasms.

AAI.Funnel plot between genus..Eubacteriumhalliigroup. and myeloproliferative neoplasms.

AAJ.Funnel plot between genus.Haemophilus. and myeloproliferative neoplasms

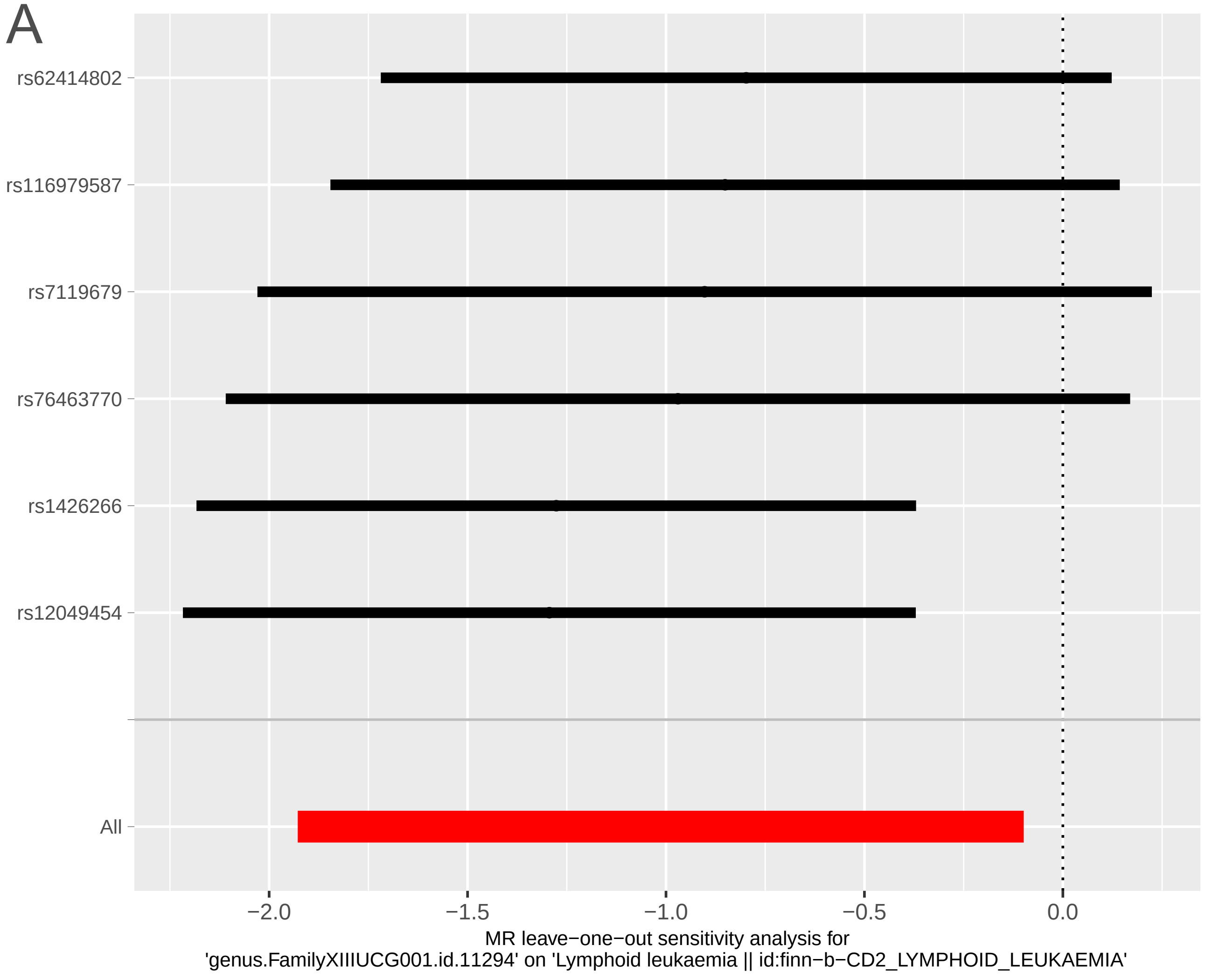
A
rs62414802
rs116979587
rs7119679
rs76463770
rs1426266
rs12049454
All
−2.0
−1.5
−1.0
−0.5
0.0
MR leave−one−out sensitivity analysis for
'genus.FamilyXIIIUCG001.id.11294' on 'Lymphoid leukaemia || id:finn−b−CD2_LYMPHOID_LEUKAEMIA'

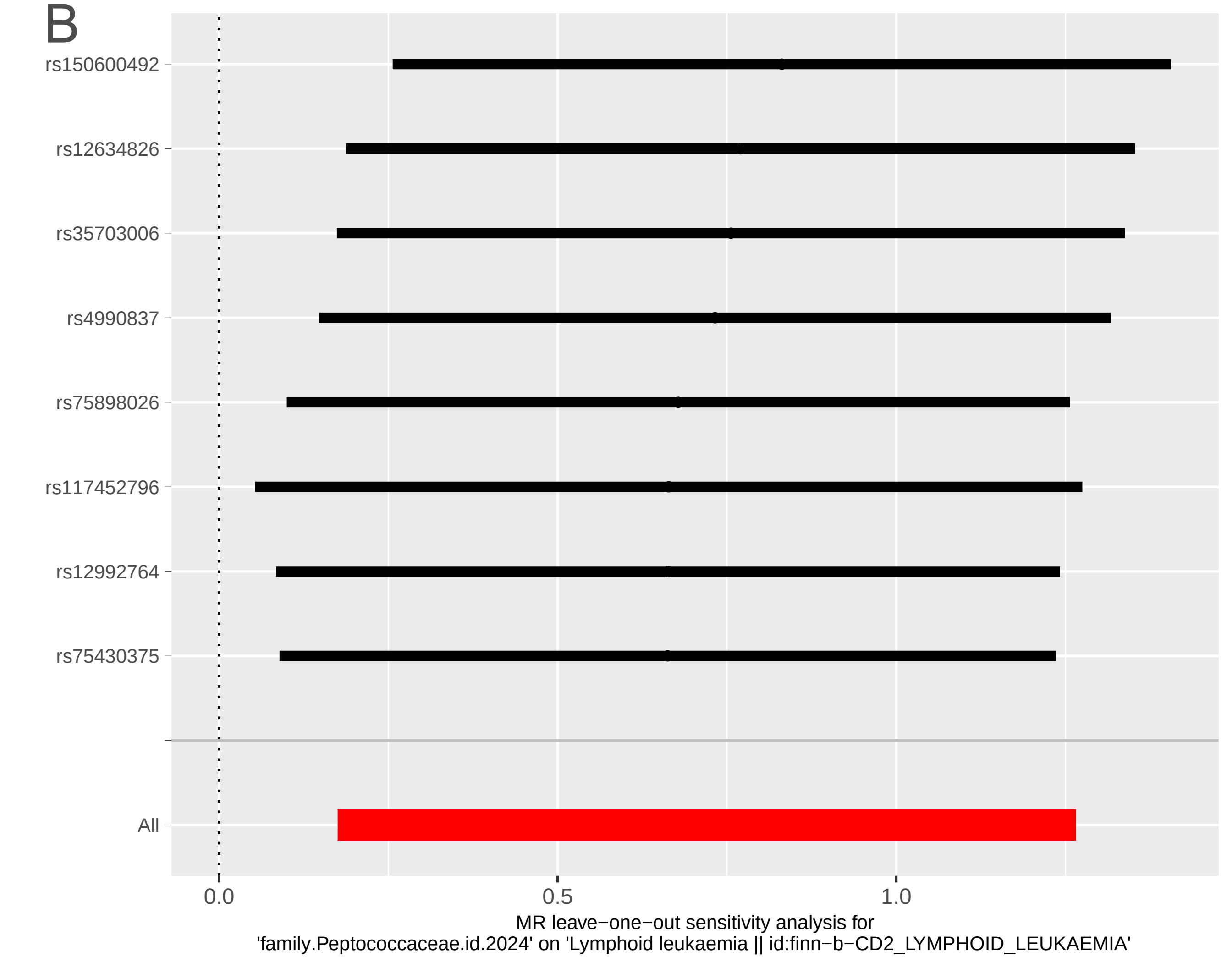
B
rs150600492
rs12634826
rs35703006
rs4990837
rs75898026
rs117452796
rs12992764
rs75430375
All
0.0
0.5
1.0
MR leave−one−out sensitivity analysis for
'family.Peptococcaceae.id.2024' on 'Lymphoid leukaemia || id:finn−b−CD2_LYMPHOID_LEUKAEMIA'

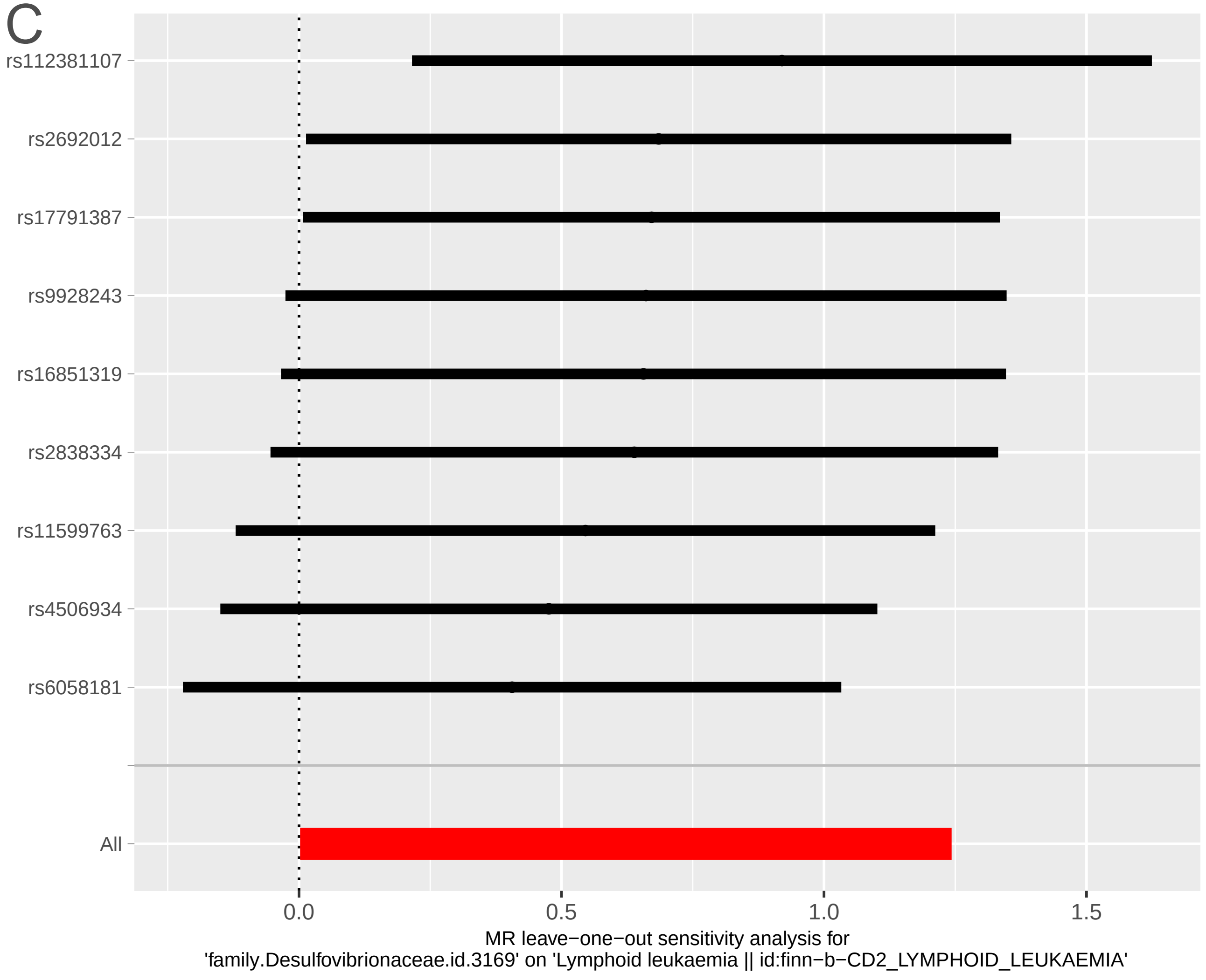
C
rs112381107
rs2692012
rs17791387
rs9928243
rs16851319
rs2838334
rs11599763
rs4506934
rs6058181
All
0.0
0.5
1.0
1.5
MR leave−one−out sensitivity analysis for
'family.Desulfovibrionaceae.id.3169' on 'Lymphoid leukaemia || id:finn−b−CD2_LYMPHOID_LEUKAEMIA'

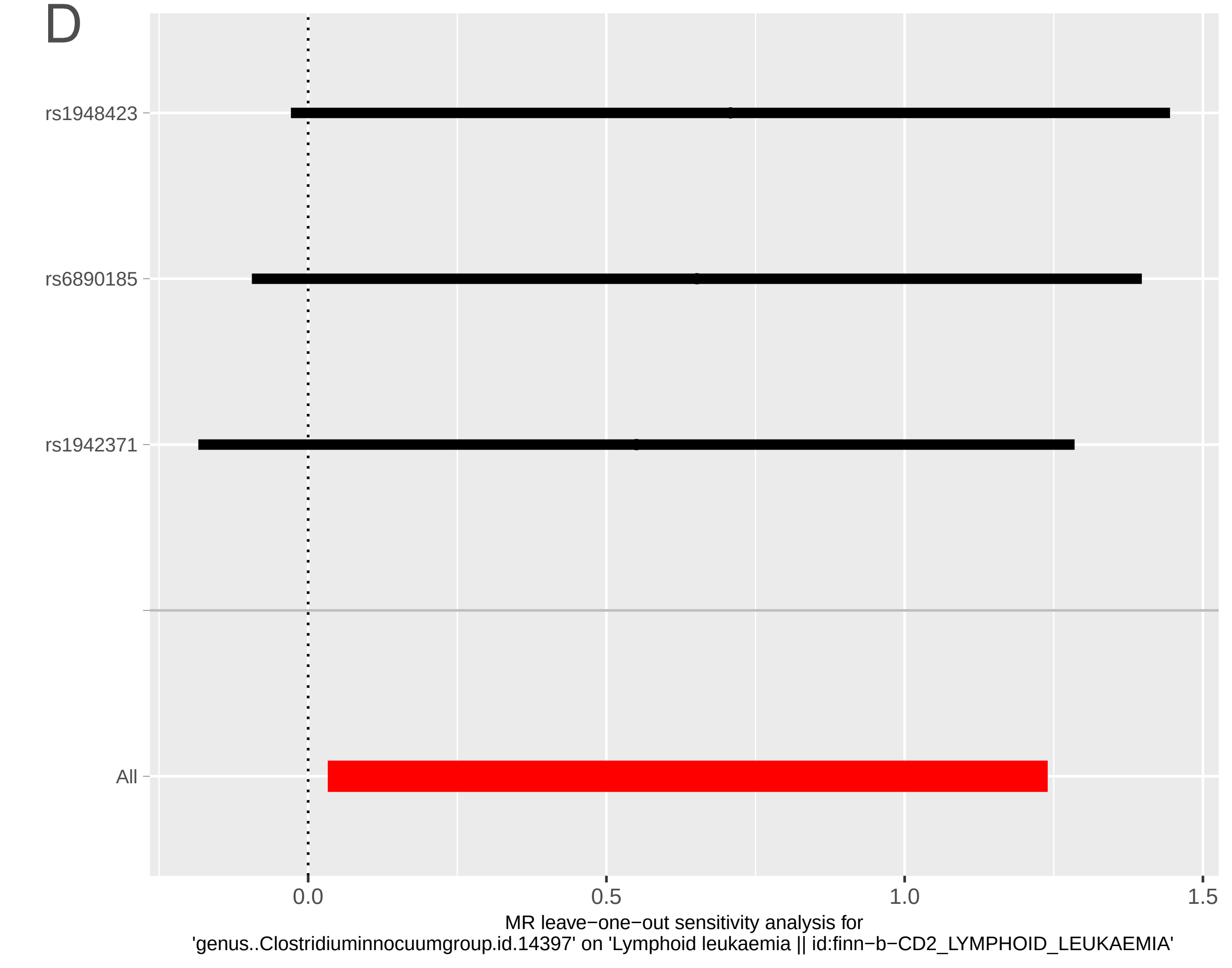
D
rs1948423
rs6890185
rs1942371
All
0.0
0.5
1.0
1.5
MR leave−one−out sensitivity analysis for
'genus..Clostridiuminnocuumgroup.id.14397' on 'Lymphoid leukaemia || id:finn−b−CD2_LYMPHOID_LEUKAEMIA'

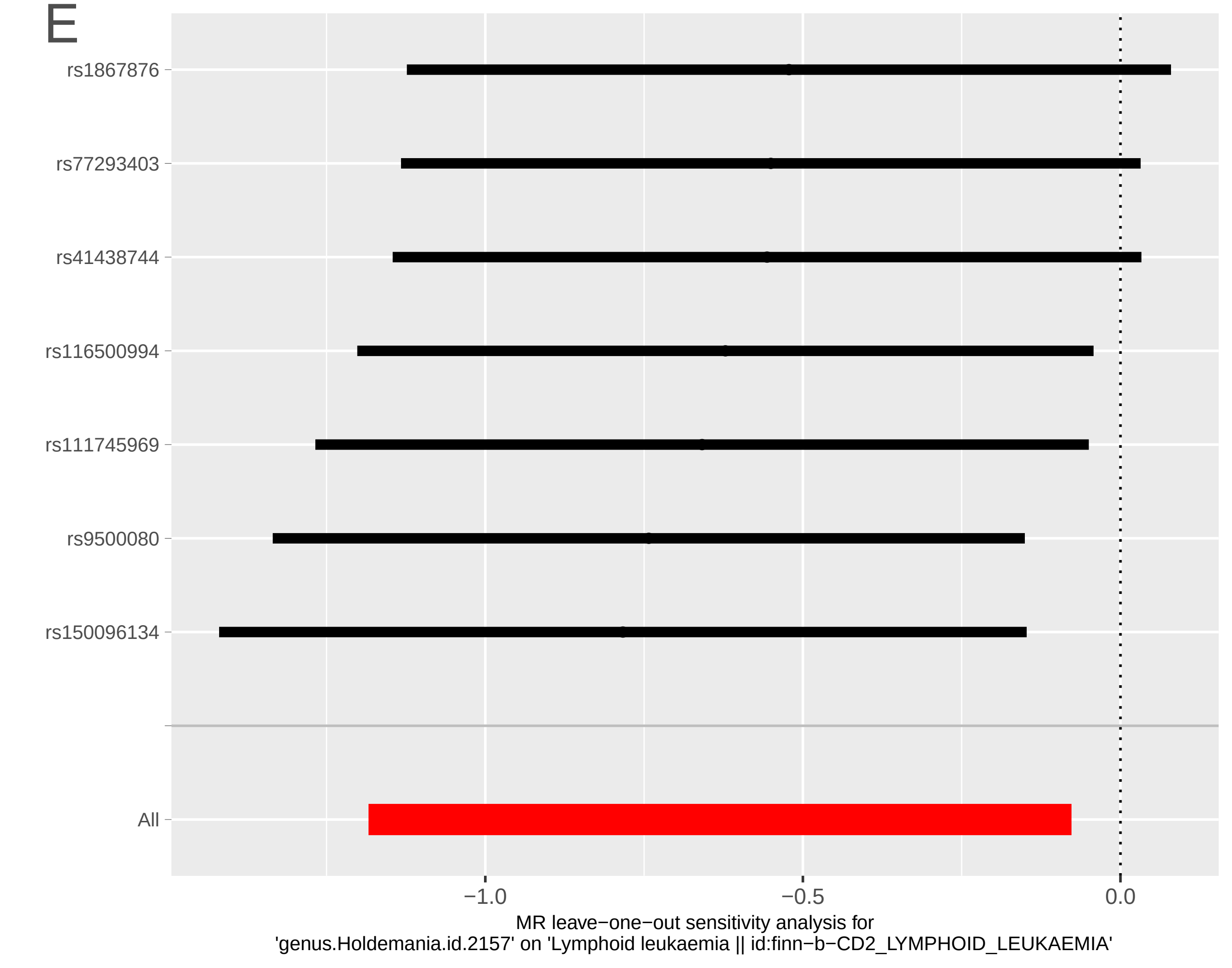
E
rs1867876
rs77293403
rs41438744
rs116500994
rs111745969
rs9500080
rs150096134
All
−1.0
−0.5
0.0
MR leave−one−out sensitivity analysis for
'genus.Holdemania.id.2157' on 'Lymphoid leukaemia || id:finn−b−CD2_LYMPHOID_LEUKAEMIA'

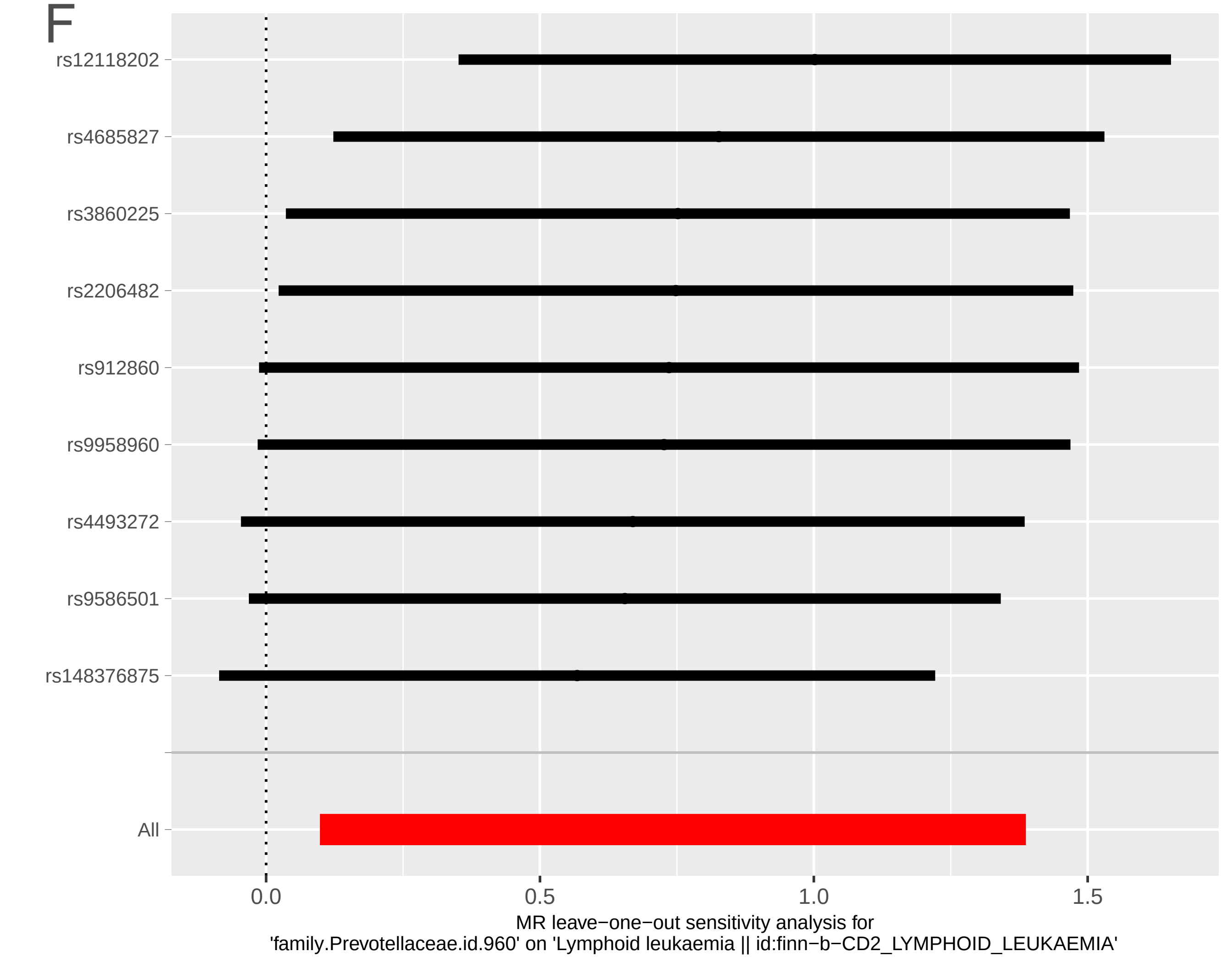
F
rs12118202
rs4685827
rs3860225
rs2206482
rs912860
rs9958960
rs4493272
rs9586501
rs148376875
All
0.0
0.5
1.0
1.5
MR leave−one−out sensitivity analysis for
'family.Prevotellaceae.id.960' on 'Lymphoid leukaemia || id:finn−b−CD2_LYMPHOID_LEUKAEMIA'

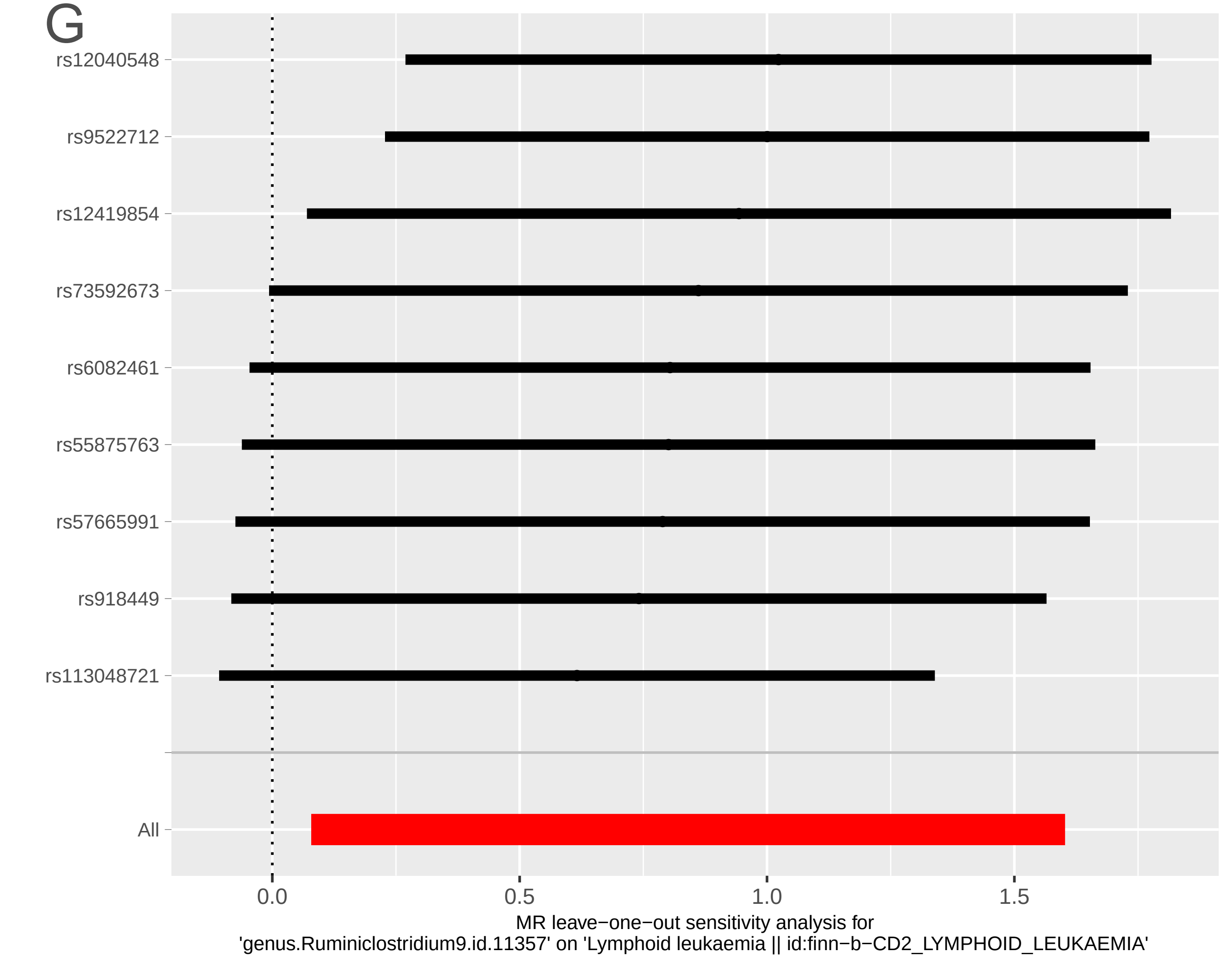
G
rs12040548
rs9522712
rs12419854
rs73592673
rs6082461
rs55875763
rs57665991
rs918449
rs113048721
All
0.0
0.5
1.0
1.5
MR leave−one−out sensitivity analysis for
'genus.Ruminiclostridium9.id.11357' on 'Lymphoid leukaemia || id:finn−b−CD2_LYMPHOID_LEUKAEMIA'

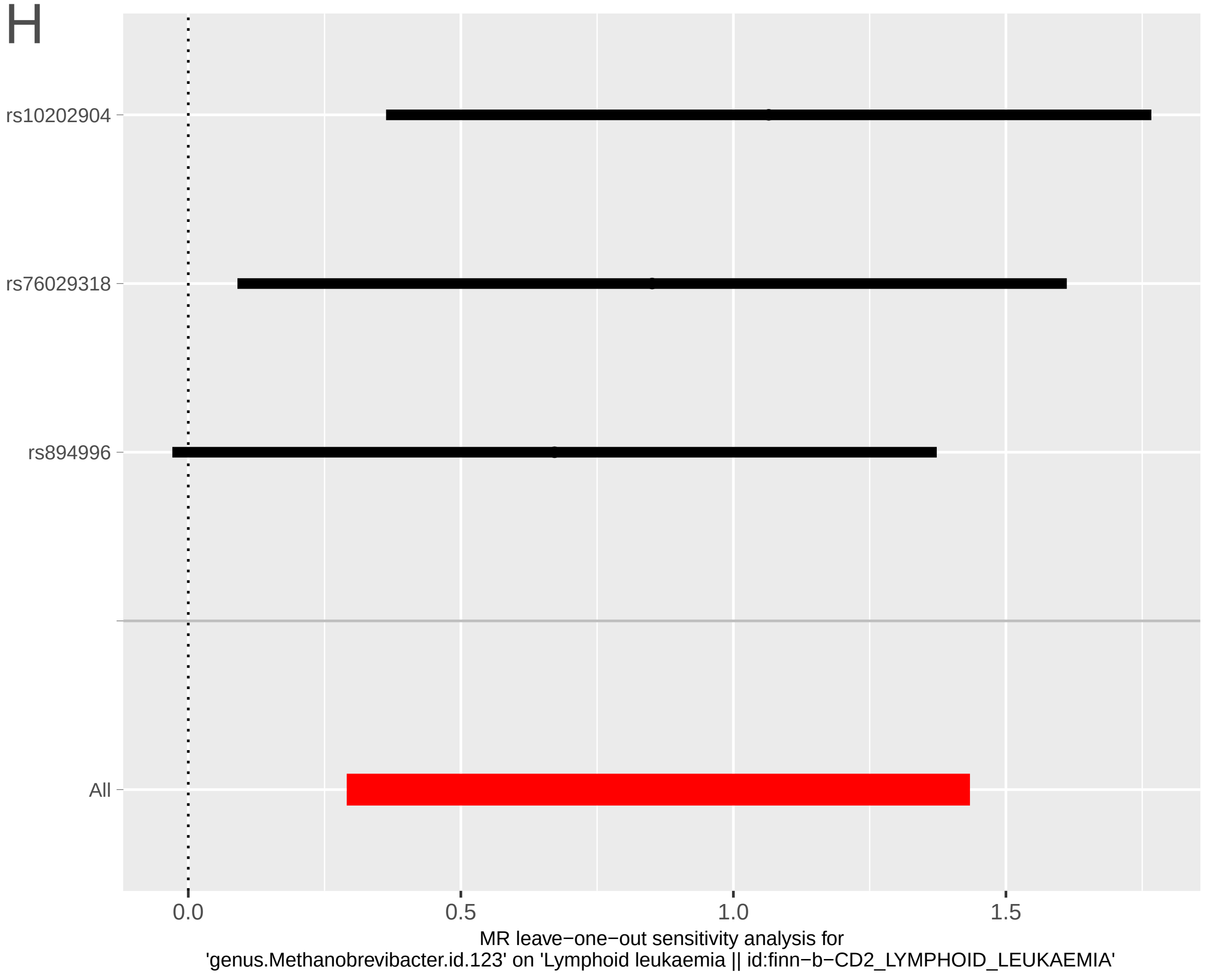
H
rs10202904
rs76029318
rs894996
All
0.0
0.5
1.0
1.5
MR leave−one−out sensitivity analysis for
'genus.Methanobrevibacter.id.123' on 'Lymphoid leukaemia || id:finn−b−CD2_LYMPHOID_LEUKAEMIA'

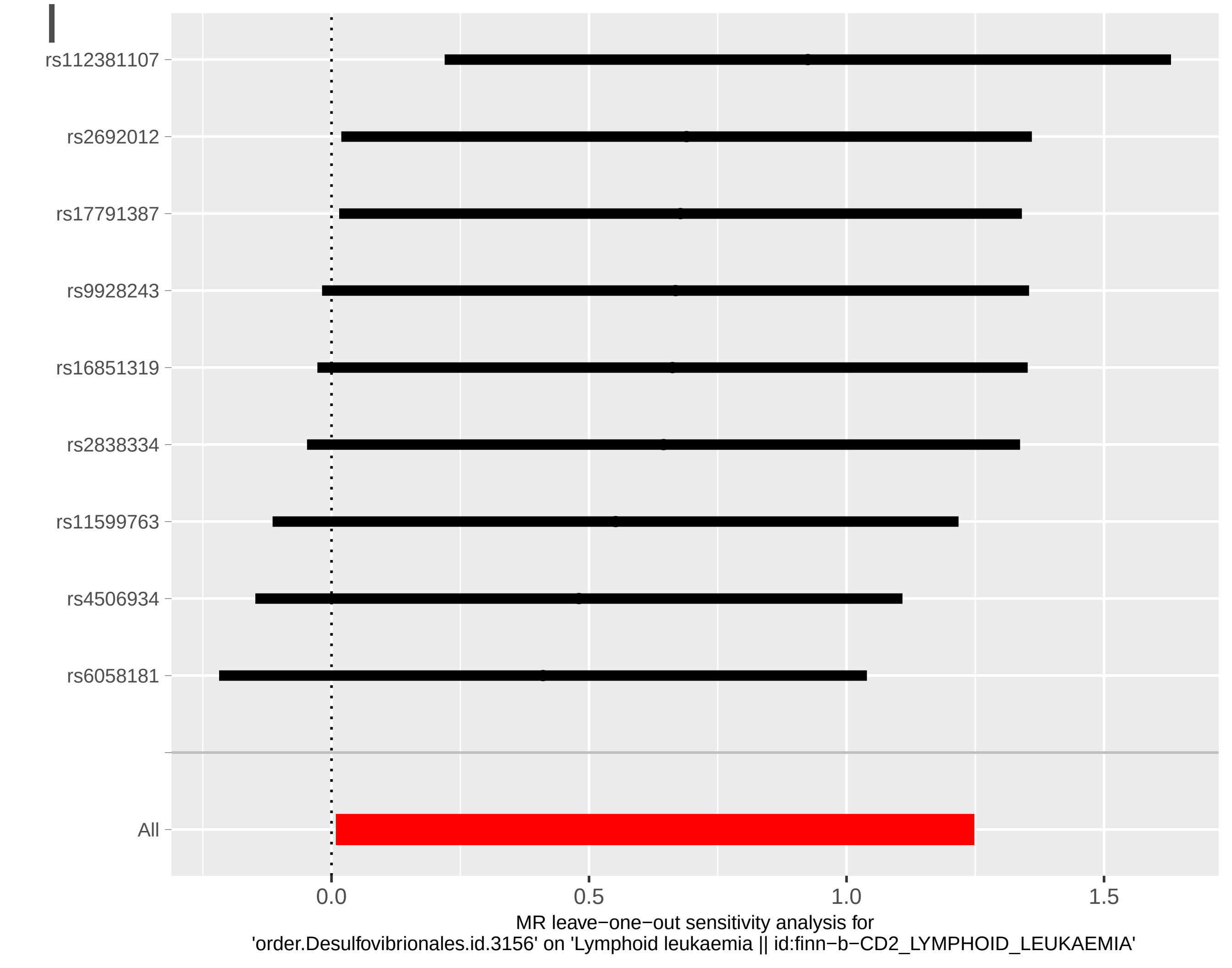

rs112381107
rs2692012
rs17791387
rs9928243
rs16851319
rs2838334
rs11599763
rs4506934
rs6058181
All
0.0
0.5
1.0
1.5
MR leave−one−out sensitivity analysis for
'order.Desulfovibrionales.id.3156' on 'Lymphoid leukaemia || id:finn−b−CD2_LYMPHOID_LEUKAEMIA'

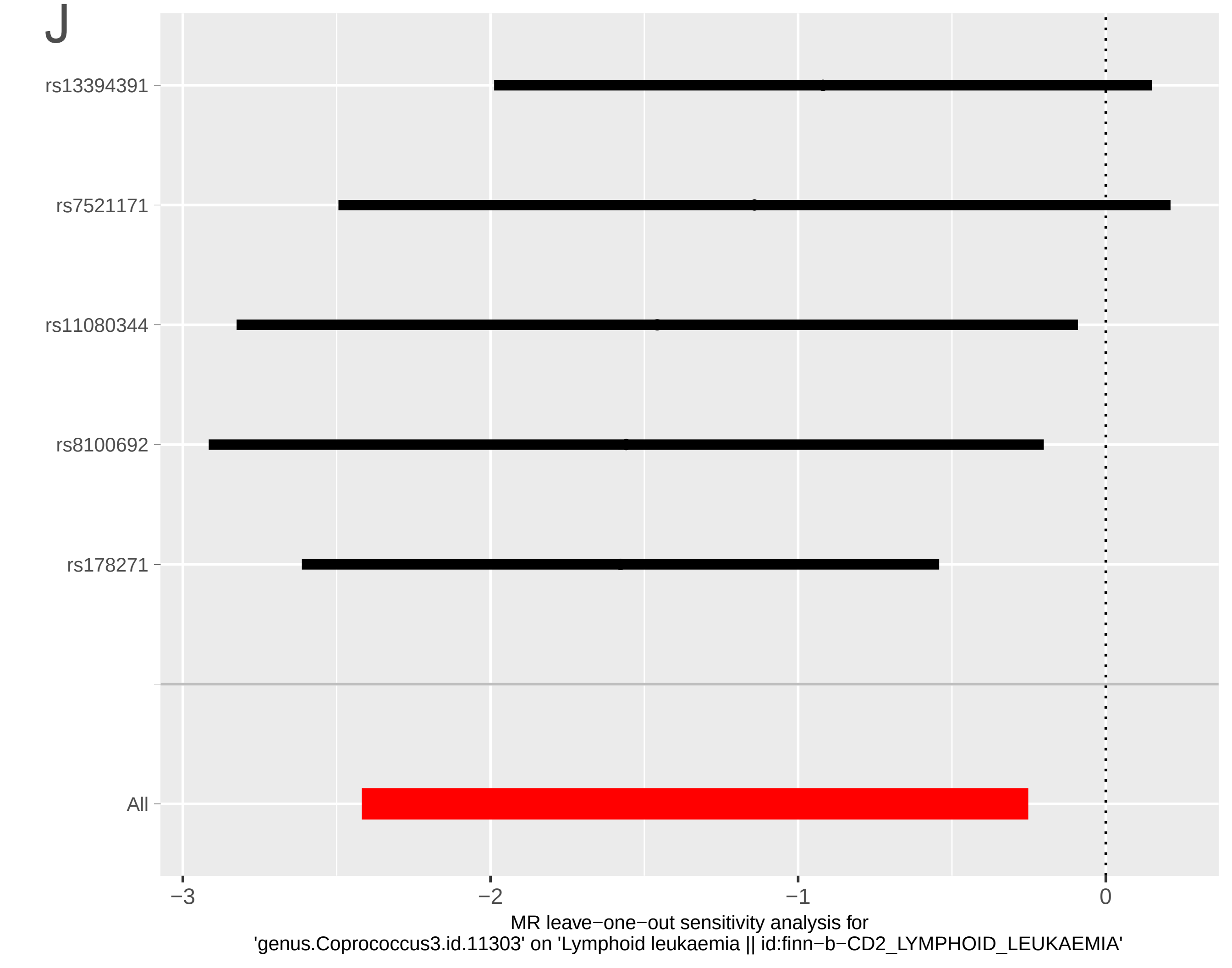
J
rs13394391
rs7521171
rs11080344
rs8100692
rs178271
All
−3
−2
−1
0
MR leave−one−out sensitivity analysis for
'genus.Coprococcus3.id.11303' on 'Lymphoid leukaemia || id:finn−b−CD2_LYMPHOID_LEUKAEMIA'

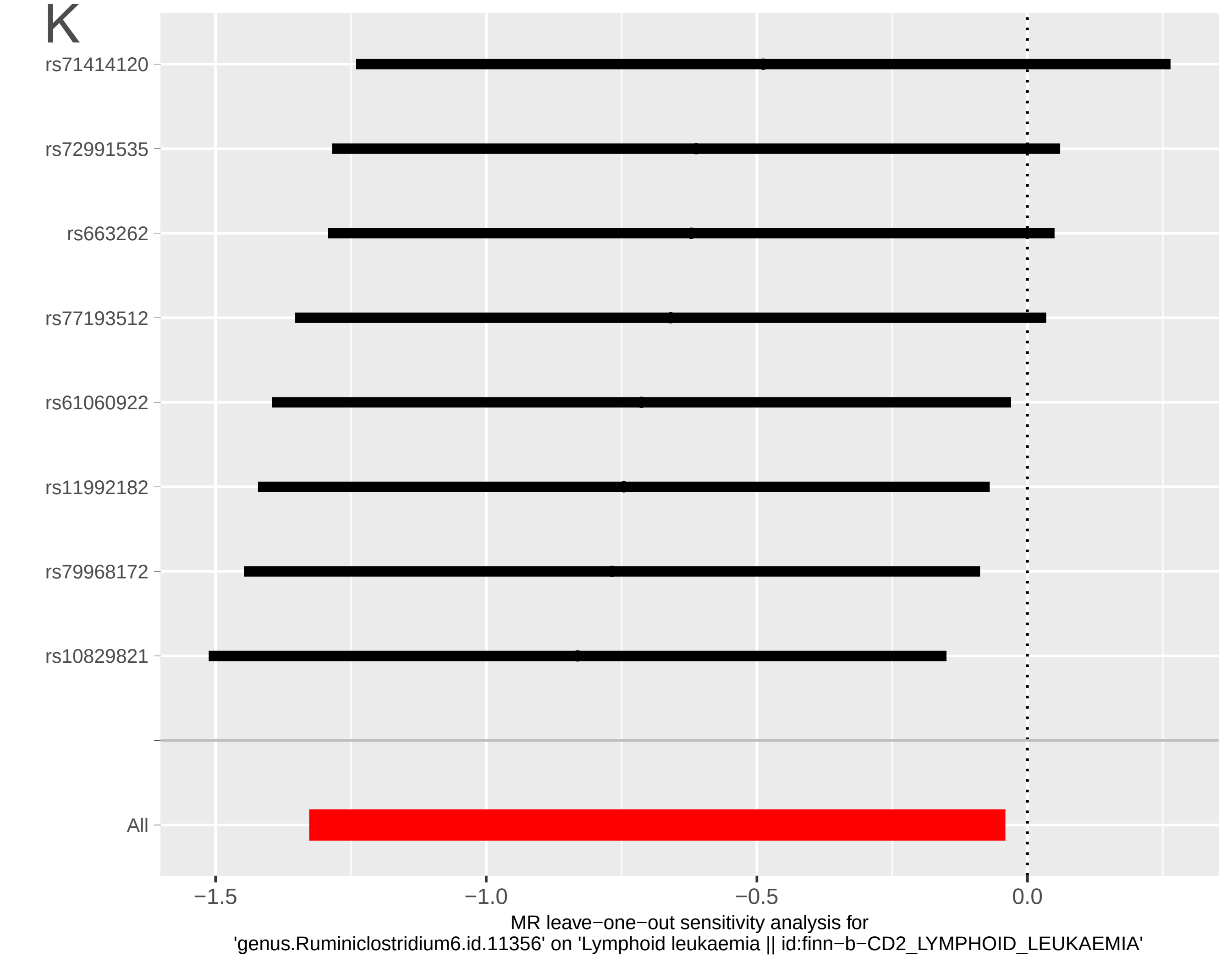
K
rs71414120
rs72991535
rs663262
rs77193512
rs61060922
rs11992182
rs79968172
rs10829821
All
−1.5
−1.0
−0.5
0.0
MR leave−one−out sensitivity analysis for
'genus.Ruminiclostridium6.id.11356' on 'Lymphoid leukaemia || id:finn−b−CD2_LYMPHOID_LEUKAEMIA'

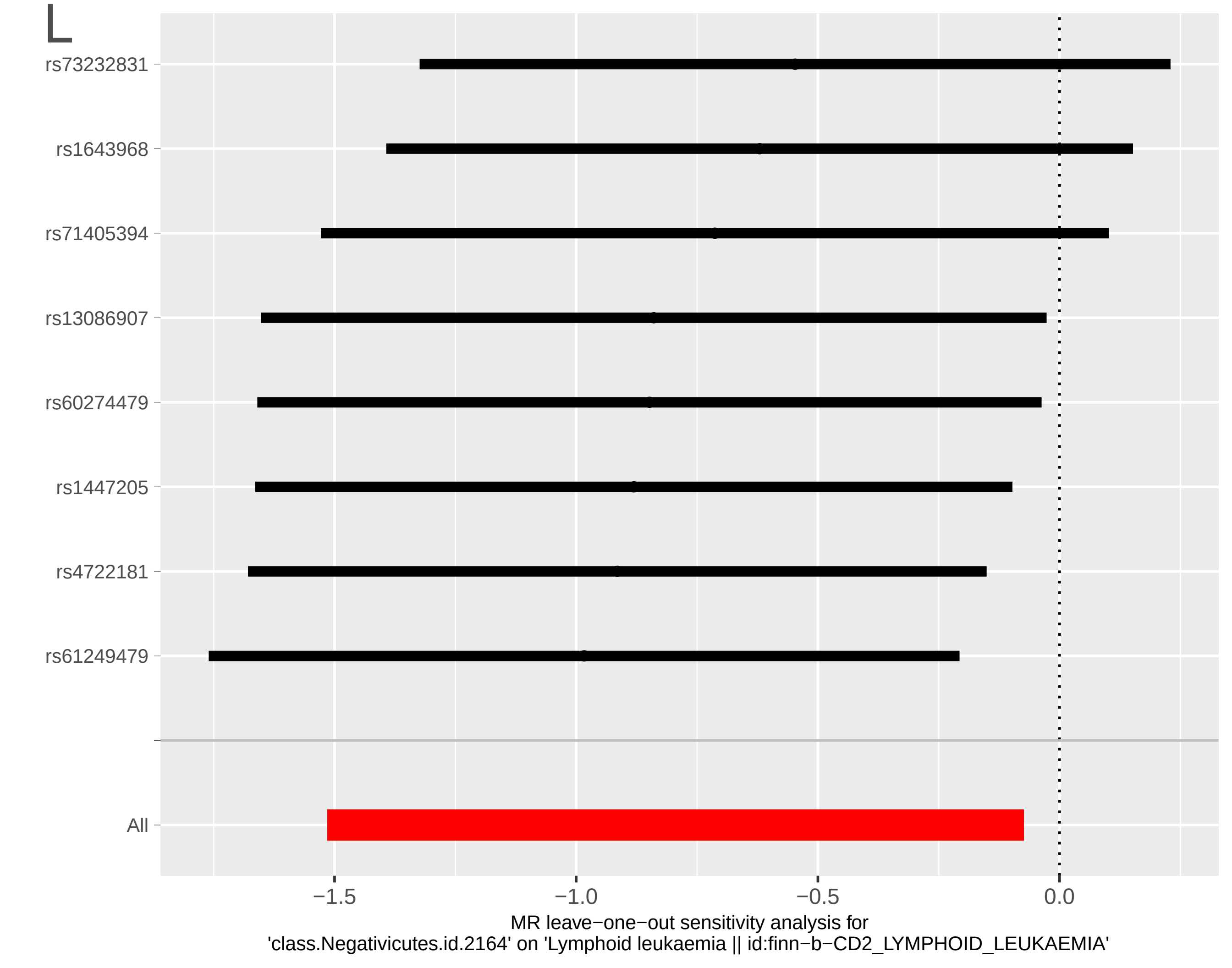
L
rs73232831
rs1643968
rs71405394
rs13086907
rs60274479
rs1447205
rs4722181
rs61249479
All
−1.5
−1.0
−0.5
0.0
MR leave−one−out sensitivity analysis for
'class.Negativicutes.id.2164' on 'Lymphoid leukaemia || id:finn−b−CD2_LYMPHOID_LEUKAEMIA'

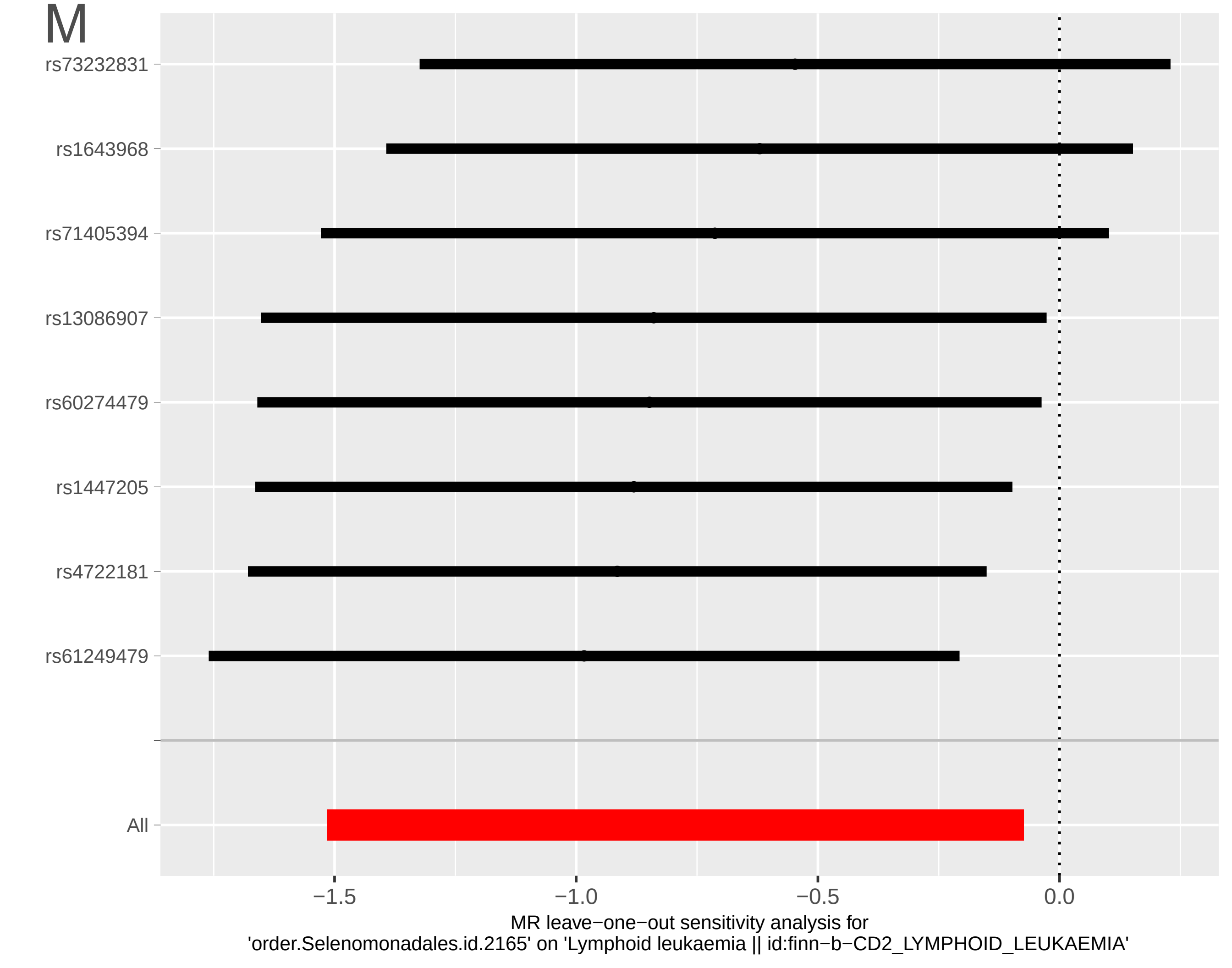
M
rs73232831
rs1643968
rs71405394
rs13086907
rs60274479
rs1447205
rs4722181
rs61249479
All
−1.5
−1.0
−0.5
0.0
MR leave−one−out sensitivity analysis for
'order.Selenomonadales.id.2165' on 'Lymphoid leukaemia || id:finn−b−CD2_LYMPHOID_LEUKAEMIA'

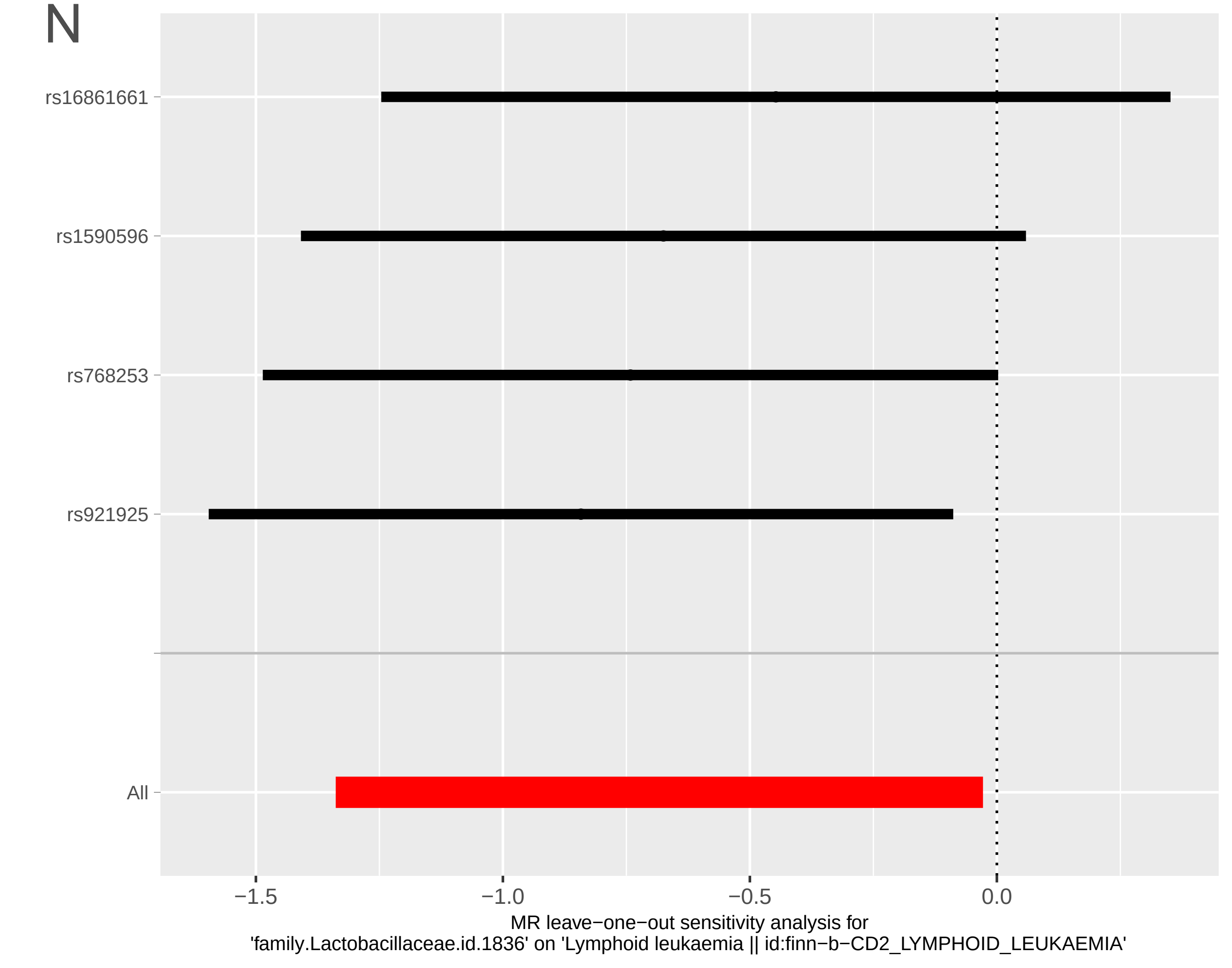
N
rs16861661
rs1590596
rs768253
rs921925
All
−1.5
−1.0
−0.5
0.0
MR leave−one−out sensitivity analysis for
'family.Lactobacillaceae.id.1836' on 'Lymphoid leukaemia || id:finn−b−CD2_LYMPHOID_LEUKAEMIA'

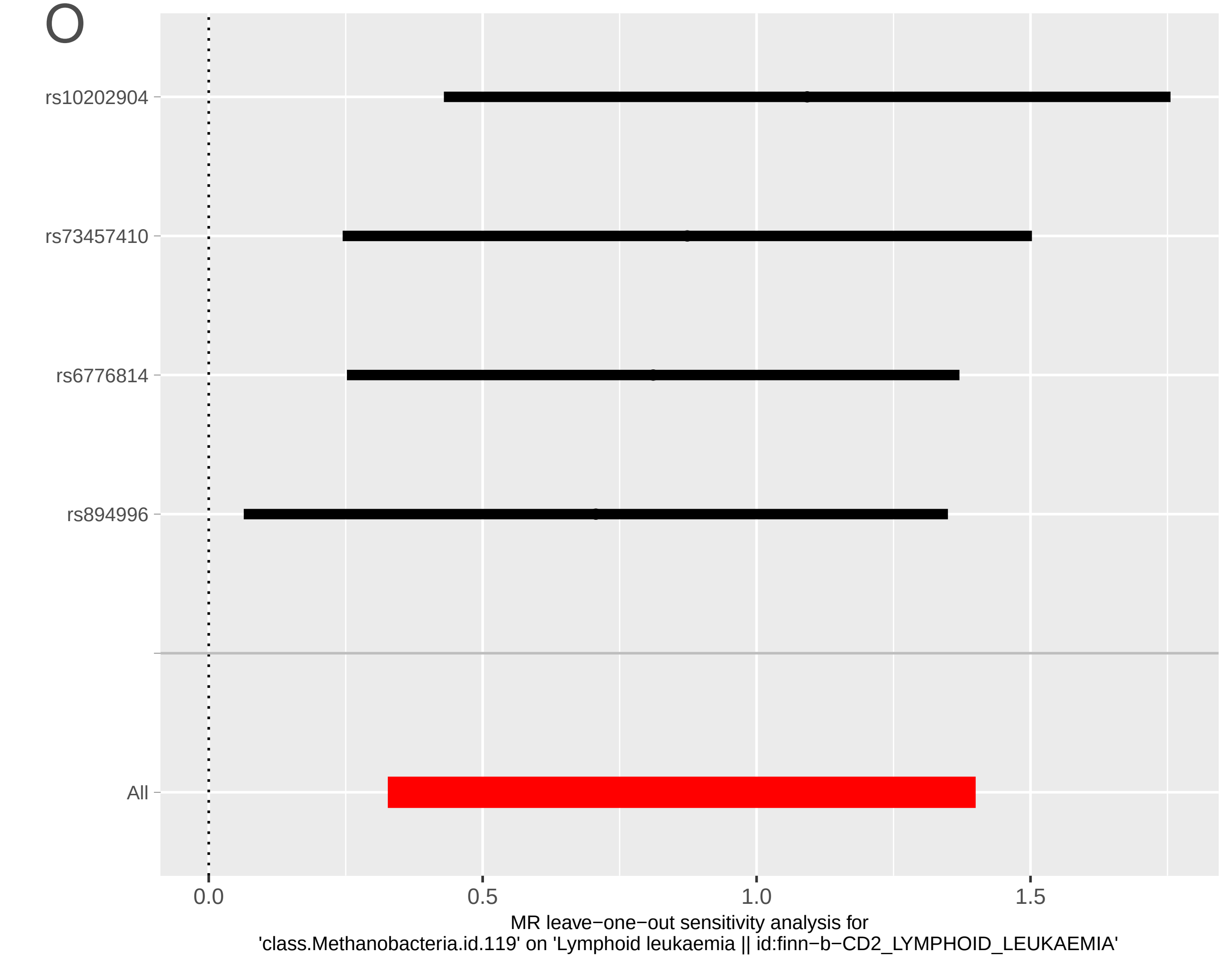
O
rs10202904
rs73457410
rs6776814
rs894996
All
0.0
0.5
1.0
1.5
MR leave−one−out sensitivity analysis for
'class.Methanobacteria.id.119' on 'Lymphoid leukaemia || id:finn−b−CD2_LYMPHOID_LEUKAEMIA'

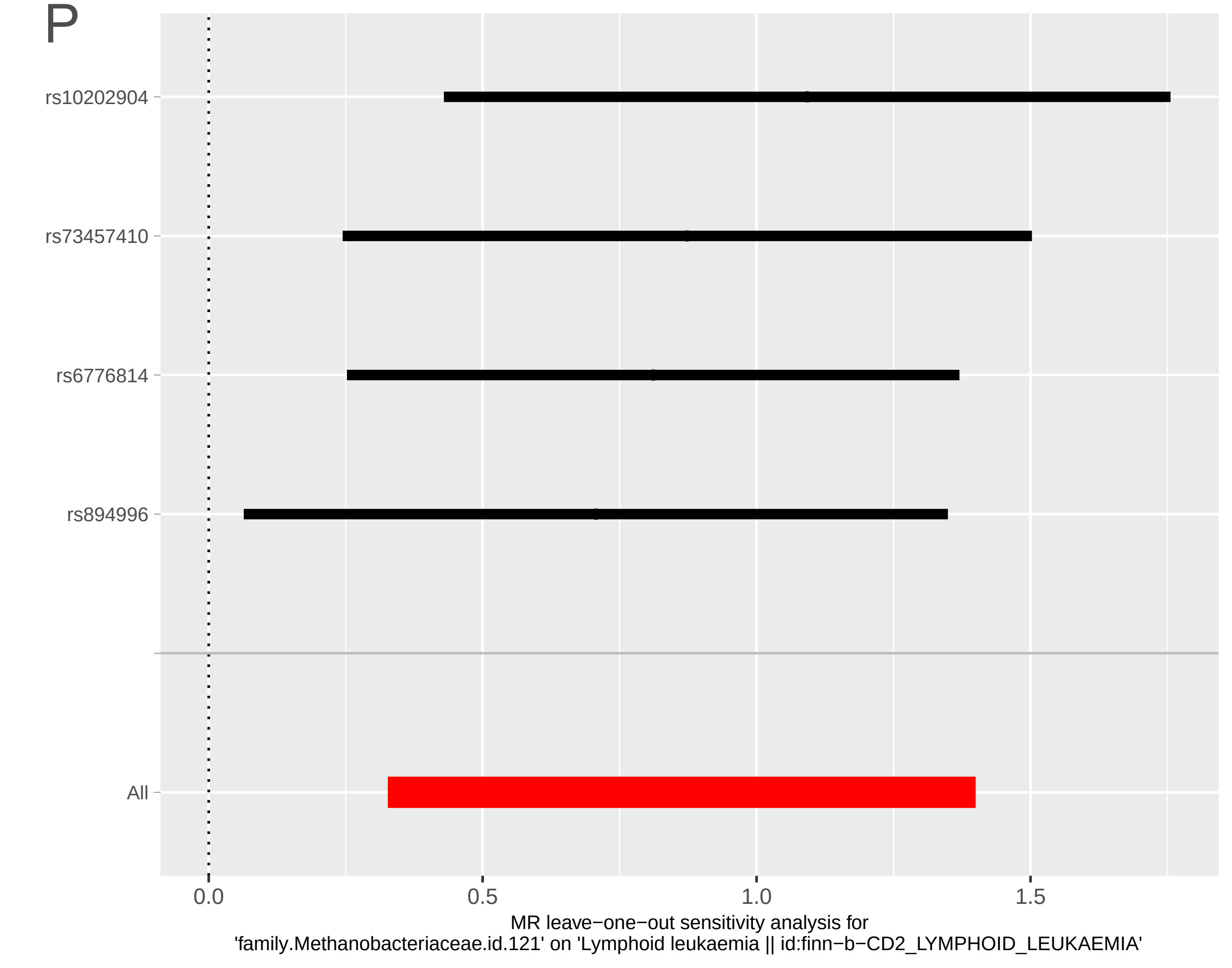
P
rs10202904
rs73457410
rs6776814
rs894996
All
0.0
0.5
1.0
1.5
MR leave−one−out sensitivity analysis for
'family.Methanobacteriaceae.id.121' on 'Lymphoid leukaemia || id:finn−b−CD2_LYMPHOID_LEUKAEMIA'

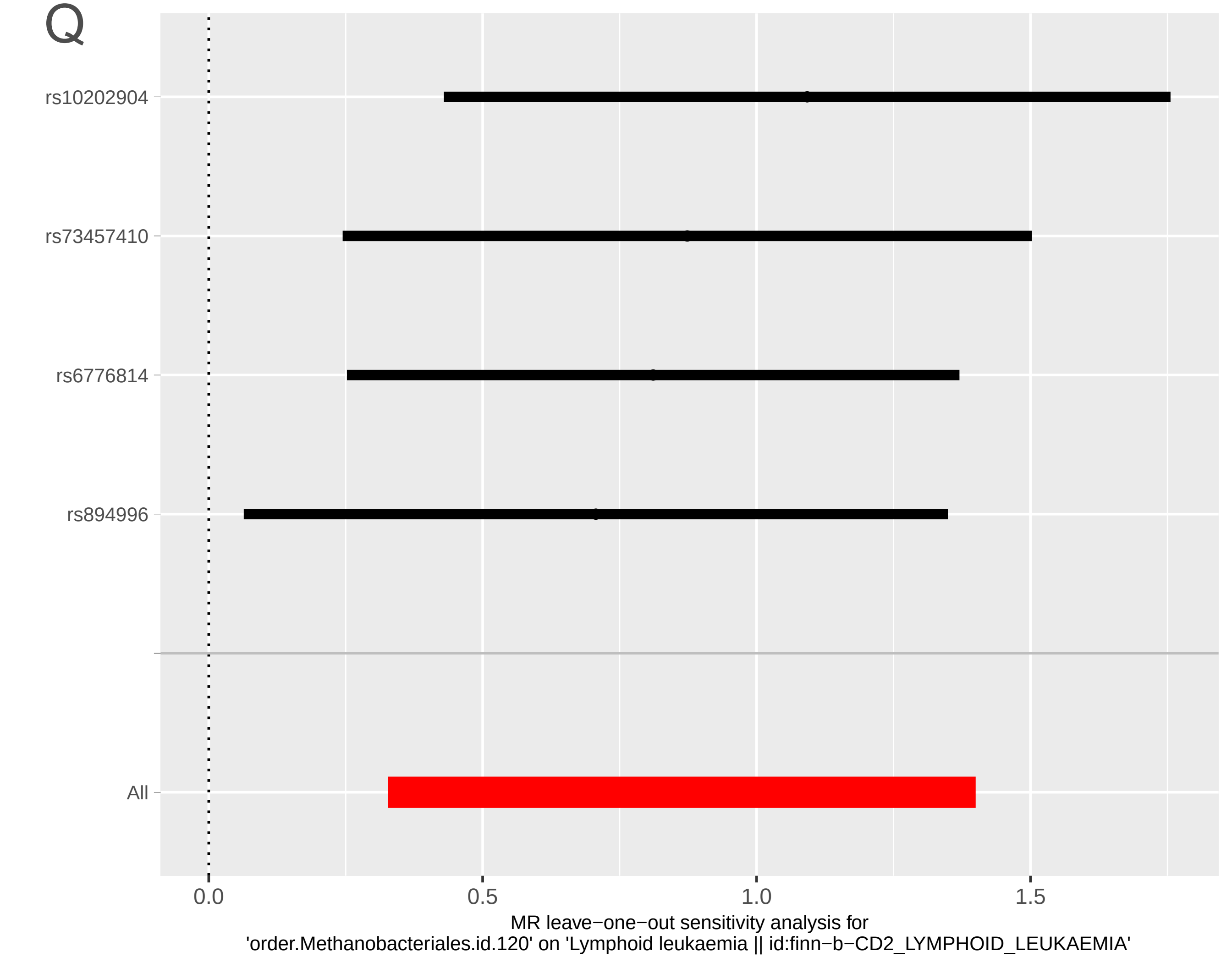
Q
rs10202904
rs73457410
rs6776814
rs894996
All
0.0
0.5
1.0
1.5
MR leave−one−out sensitivity analysis for
'order.Methanobacteriales.id.120' on 'Lymphoid leukaemia || id:finn−b−CD2_LYMPHOID_LEUKAEMIA'

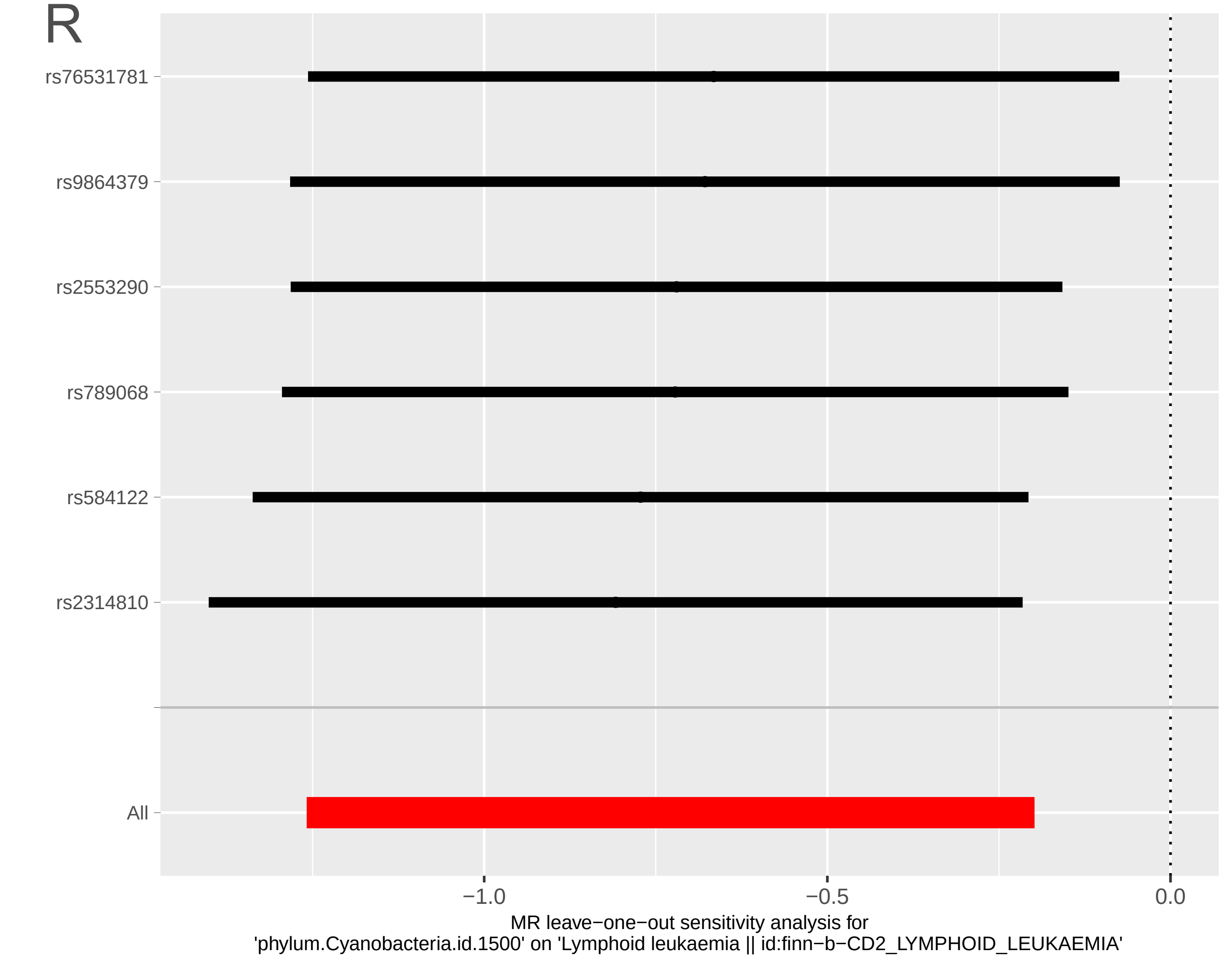
R
rs76531781
rs9864379
rs2553290
rs789068
rs584122
rs2314810
All
−1.0
−0.5
0.0
MR leave−one−out sensitivity analysis for
'phylum.Cyanobacteria.id.1500' on 'Lymphoid leukaemia || id:finn−b−CD2_LYMPHOID_LEUKAEMIA'

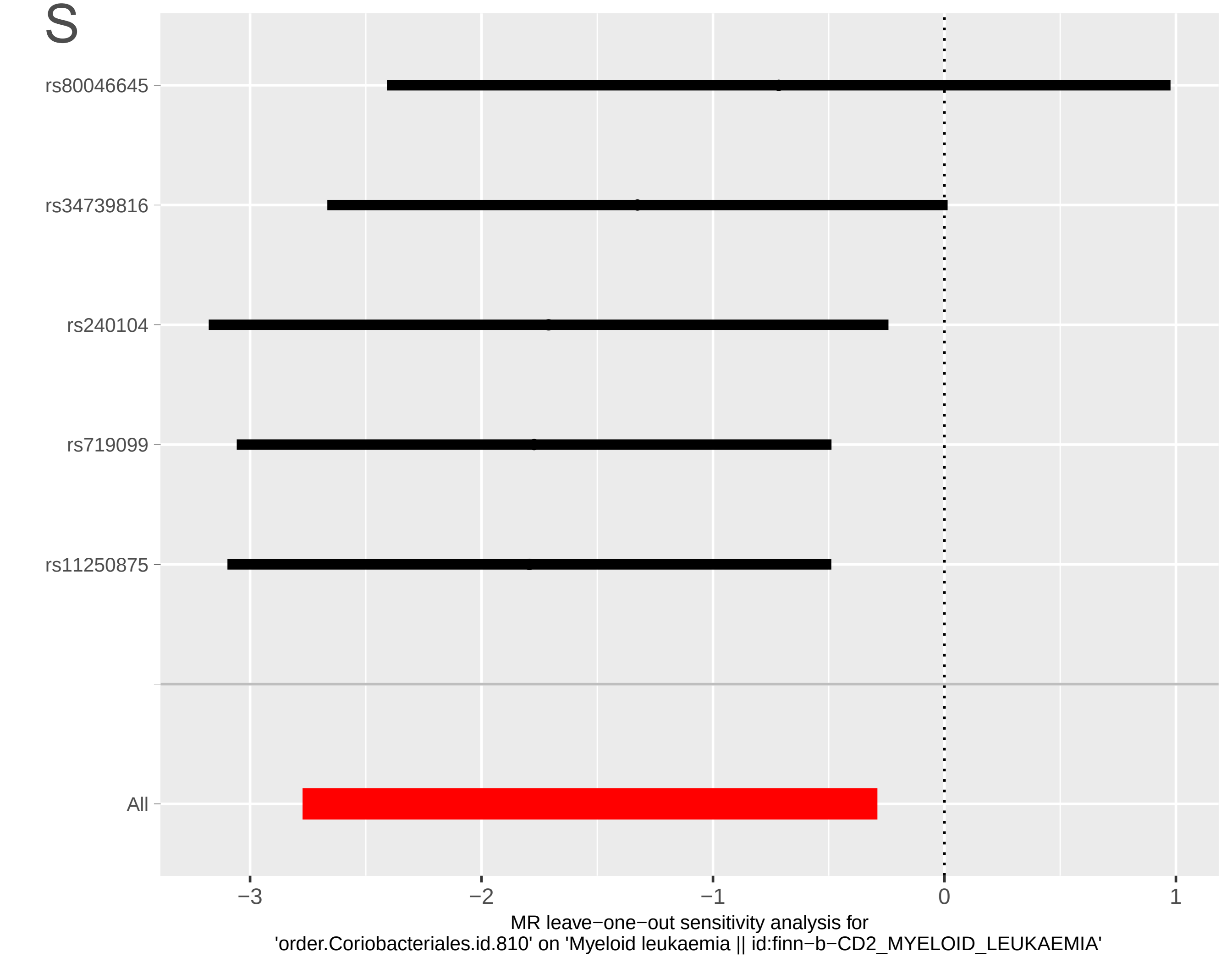
S
rs80046645
rs34739816
rs240104
rs719099
rs11250875
All
−3
−2
−1
0
1
MR leave−one−out sensitivity analysis for
'order.Coriobacteriales.id.810' on 'Myeloid leukaemia || id:finn−b−CD2_MYELOID_LEUKAEMIA'

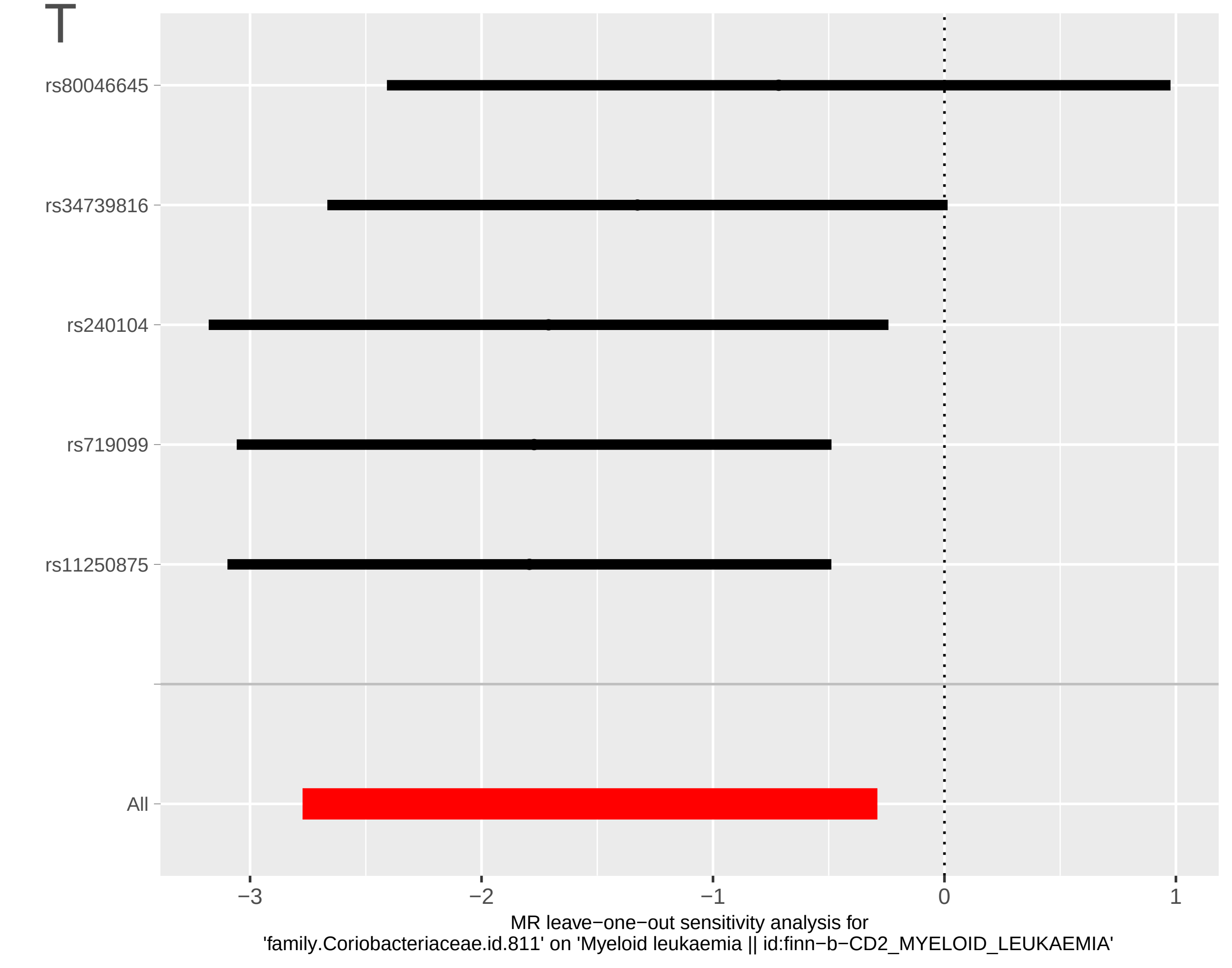
T
rs80046645
rs34739816
rs240104
rs719099
rs11250875
All
−3
−2
−1
0
1
MR leave−one−out sensitivity analysis for
'family.Coriobacteriaceae.id.811' on 'Myeloid leukaemia || id:finn−b−CD2_MYELOID_LEUKAEMIA'

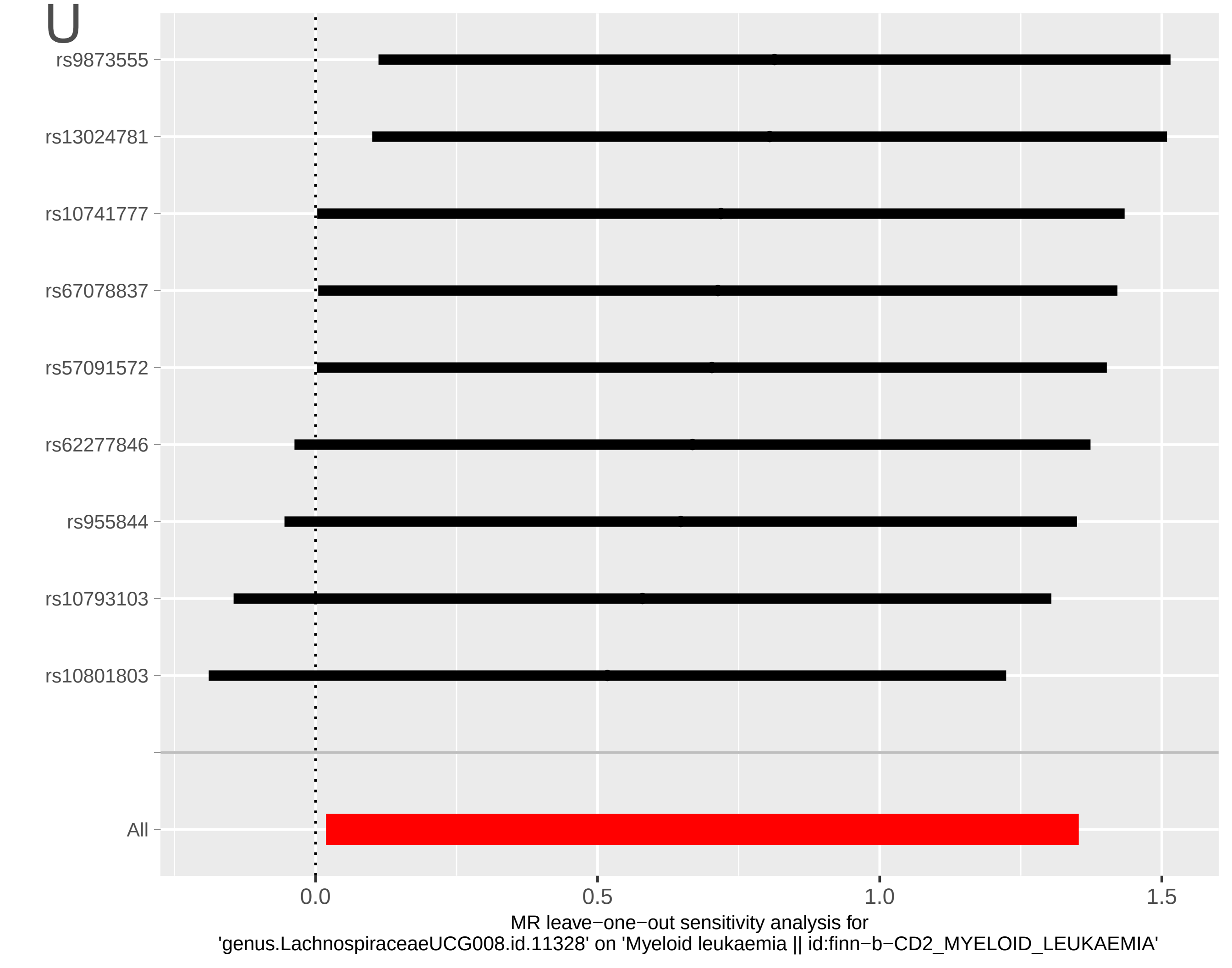
U
rs9873555
rs13024781
rs10741777
rs67078837
rs57091572
rs62277846
rs955844
rs10793103
rs10801803
All
0.0
0.5
1.0
1.5
MR leave−one−out sensitivity analysis for
'genus.LachnospiraceaeUCG008.id.11328' on 'Myeloid leukaemia || id:finn−b−CD2_MYELOID_LEUKAEMIA'

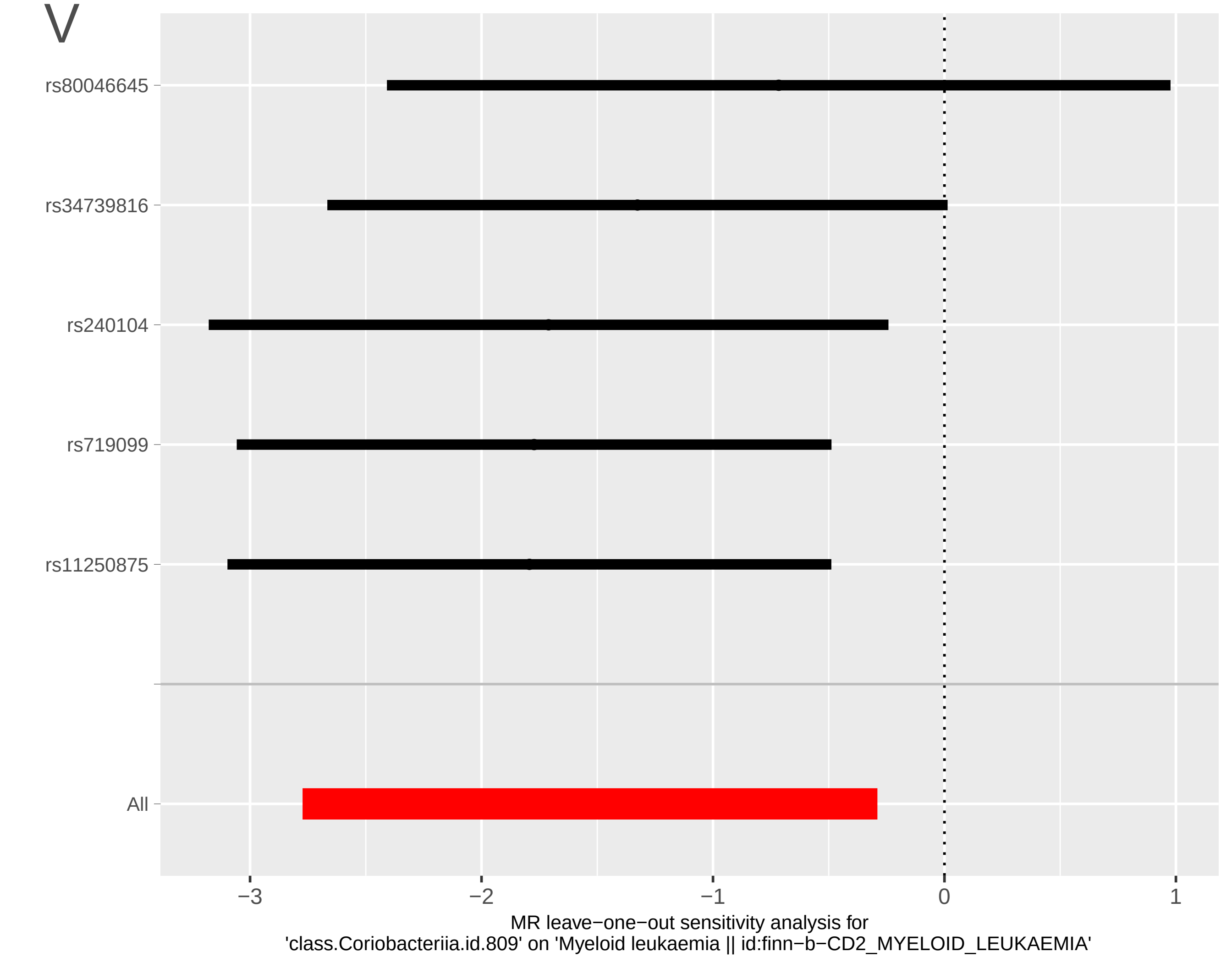
V
rs80046645
rs34739816
rs240104
rs719099
rs11250875
All
−3
−2
−1
0
1
MR leave−one−out sensitivity analysis for
'class.Coriobacteriia.id.809' on 'Myeloid leukaemia || id:finn−b−CD2_MYELOID_LEUKAEMIA'

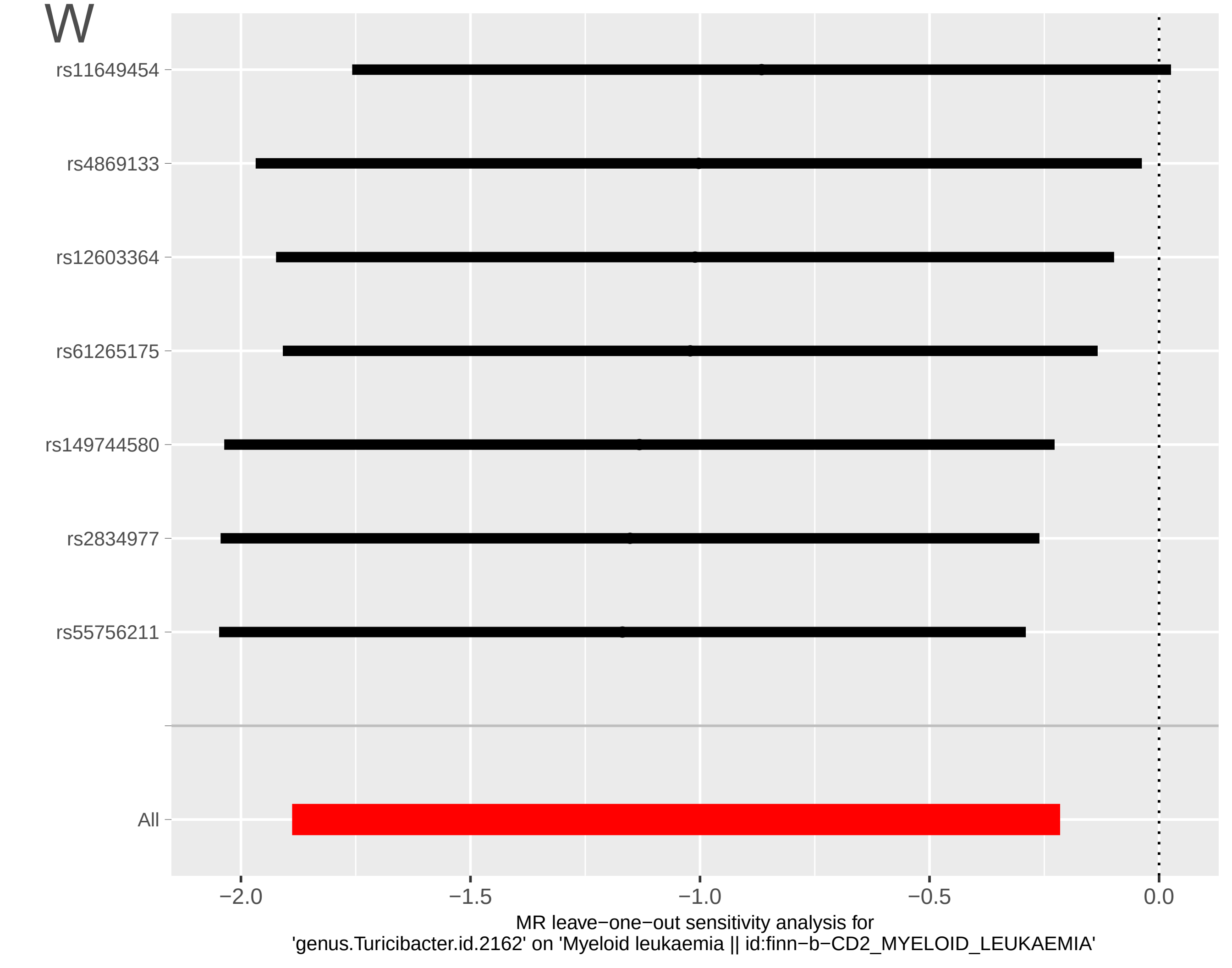
W
rs11649454
rs4869133
rs12603364
rs61265175
rs149744580
rs2834977
rs55756211
All
−2.0
−1.5
−1.0
−0.5
0.0
MR leave−one−out sensitivity analysis for
'genus.Turicibacter.id.2162' on 'Myeloid leukaemia || id:finn−b−CD2_MYELOID_LEUKAEMIA'

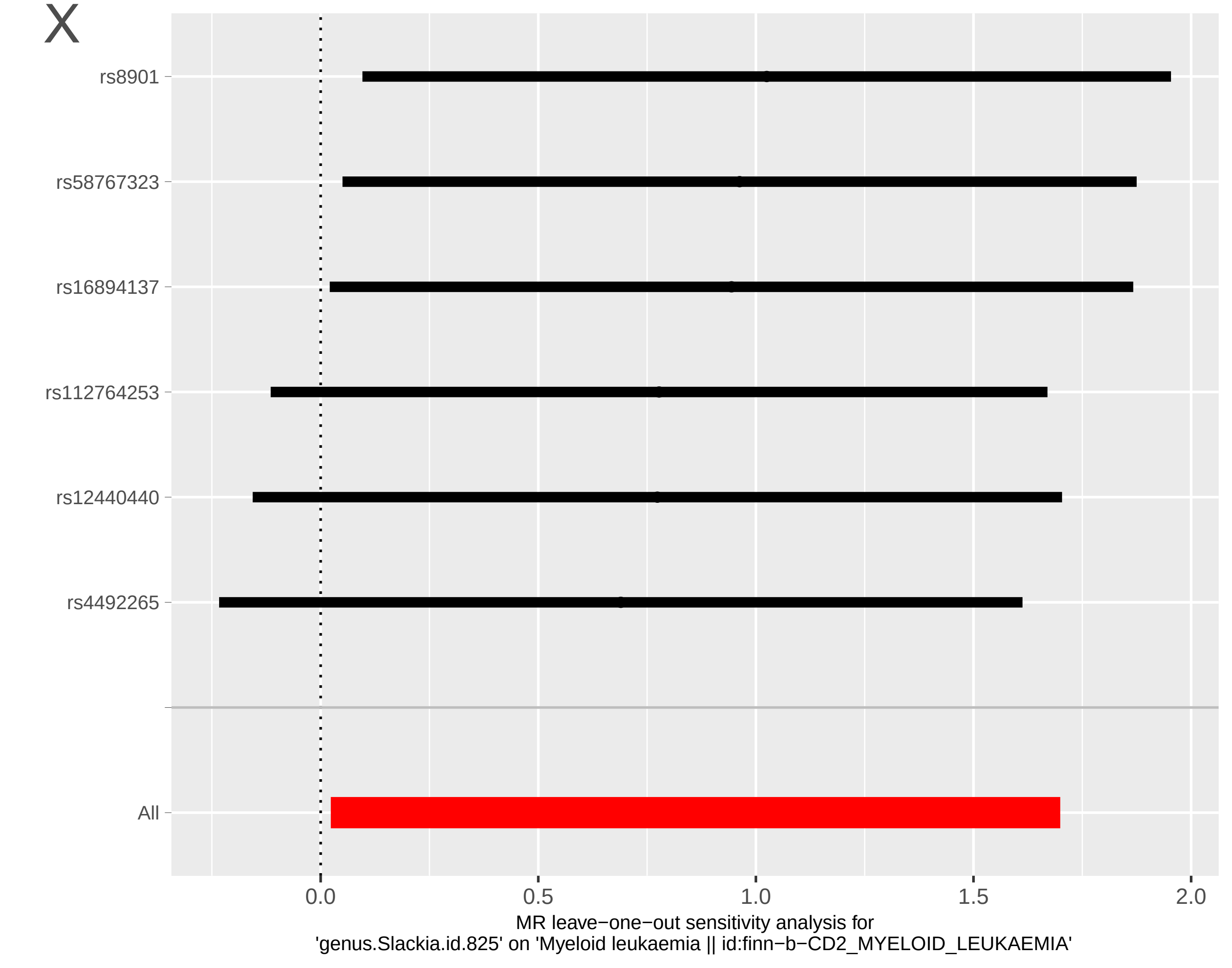
X
rs8901
rs58767323
rs16894137
rs112764253
rs12440440
rs4492265
All
0.0
0.5
1.0
1.5
2.0
MR leave−one−out sensitivity analysis for
'genus.Slackia.id.825' on 'Myeloid leukaemia || id:finn−b−CD2_MYELOID_LEUKAEMIA'

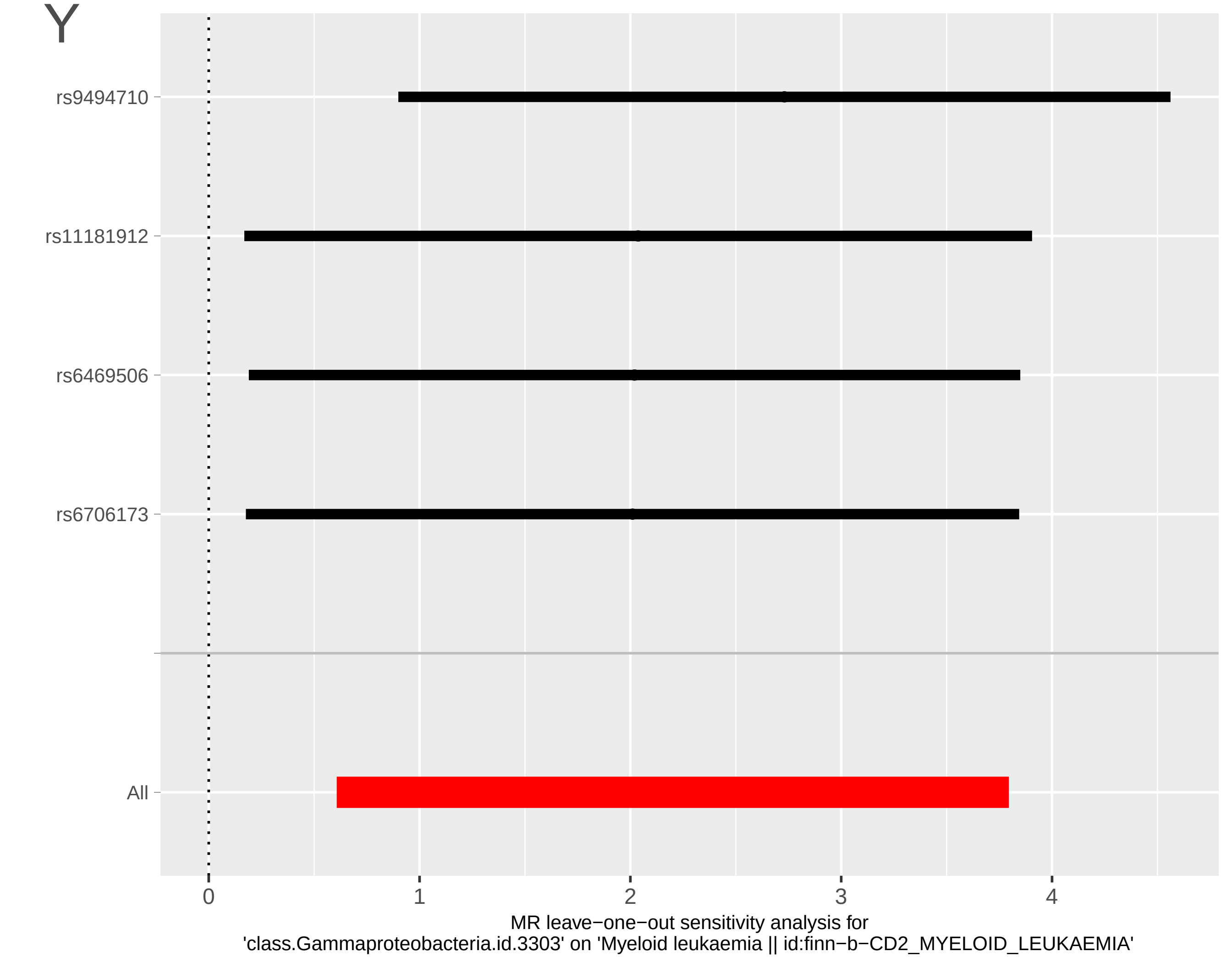

Y
rs9494710
rs11181912
rs6469506
rs6706173
All
0
1
2
3
4
MR leave−one−out sensitivity analysis for
'class.Gammaproteobacteria.id.3303' on 'Myeloid leukaemia || id:finn−b−CD2_MYELOID_LEUKAEMIA'

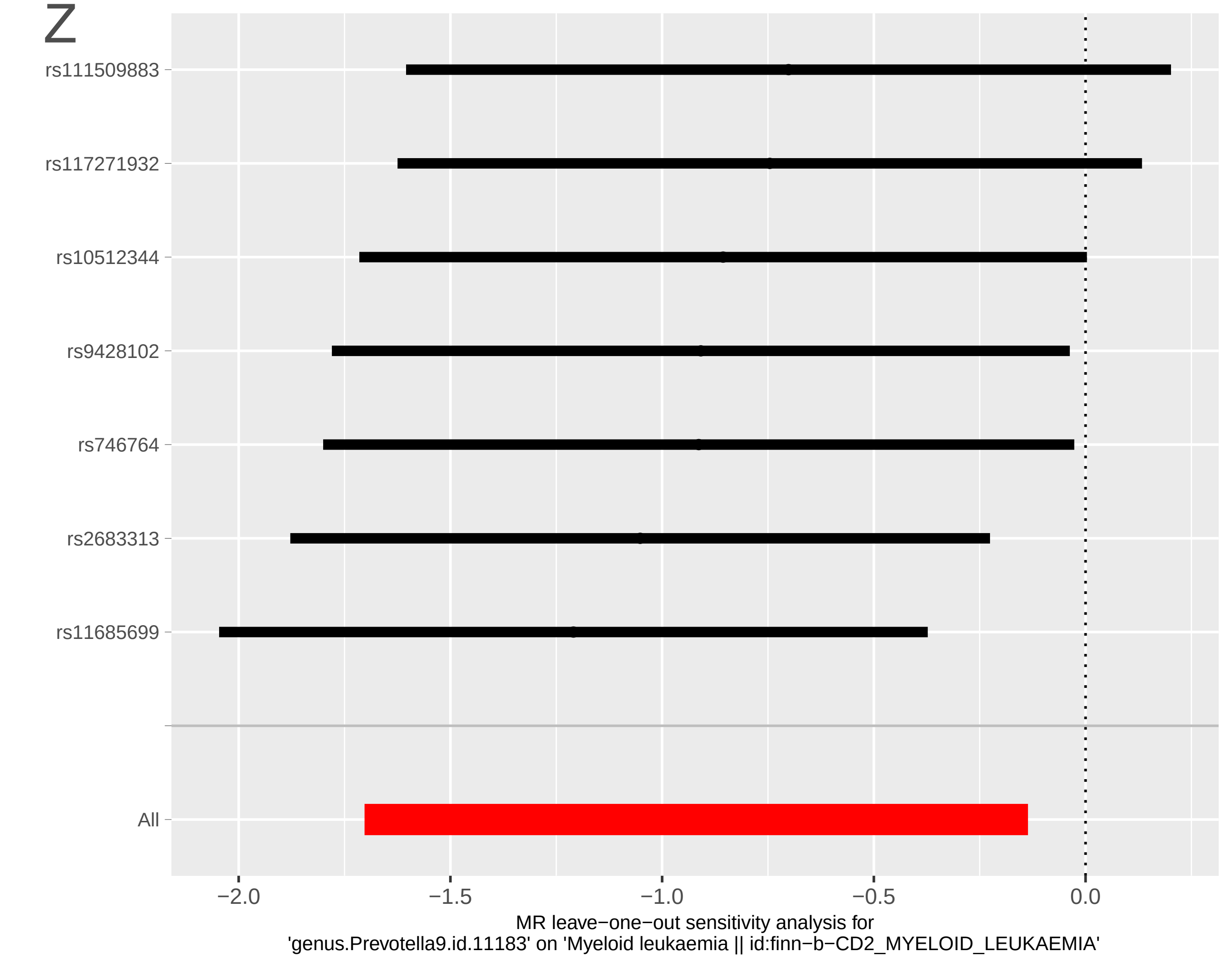
Z
rs111509883
rs117271932
rs10512344
rs9428102
rs746764
rs2683313
rs11685699
All
−2.0
−1.5
−1.0
−0.5
0.0
MR leave−one−out sensitivity analysis for
'genus.Prevotella9.id.11183' on 'Myeloid leukaemia || id:finn−b−CD2_MYELOID_LEUKAEMIA'

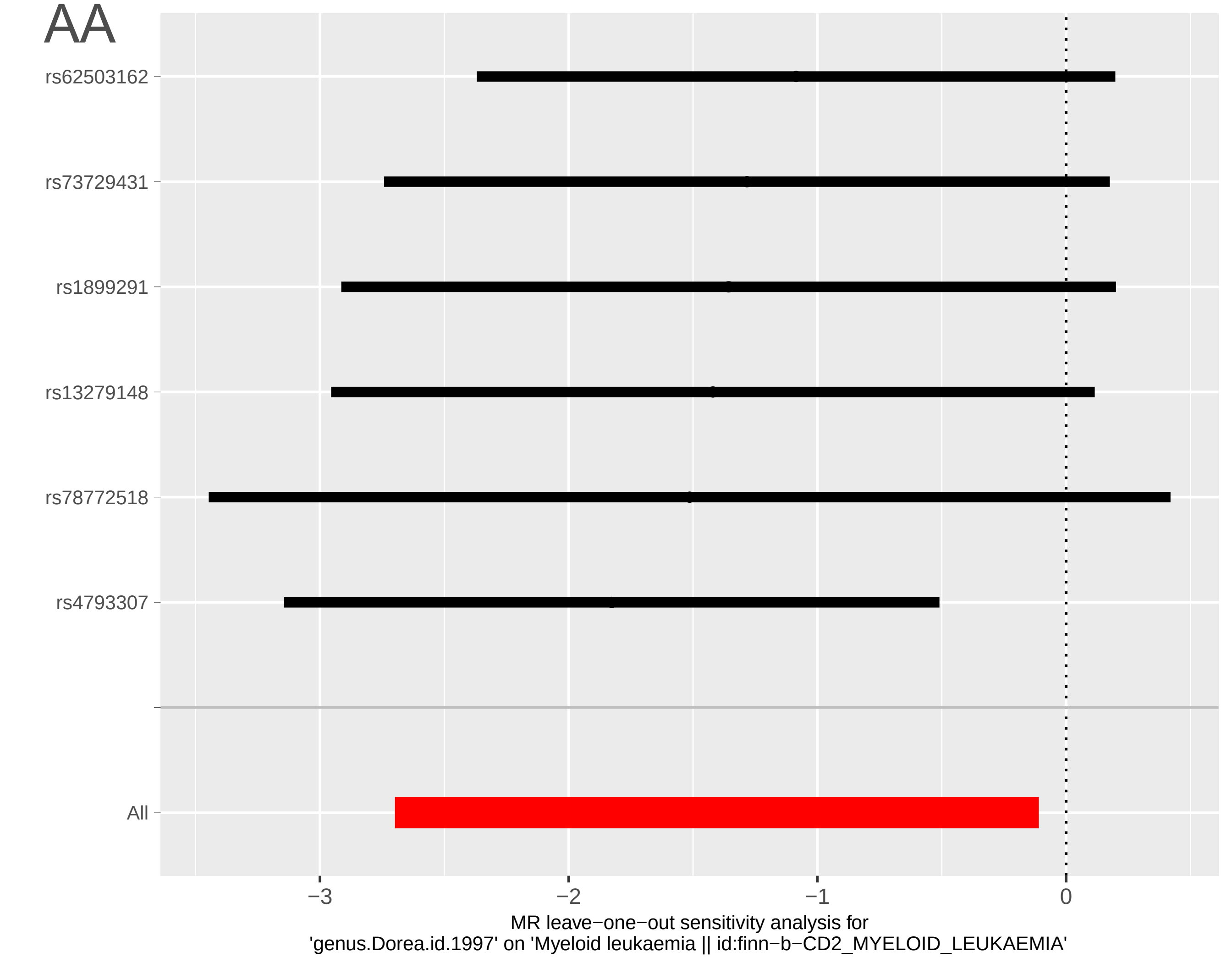

AA
rs62503162
rs73729431
rs1899291
rs13279148
rs78772518
rs4793307
All
−3
−2
−1
0
MR leave−one−out sensitivity analysis for
'genus.Dorea.id.1997' on 'Myeloid leukaemia || id:finn−b−CD2_MYELOID_LEUKAEMIA'

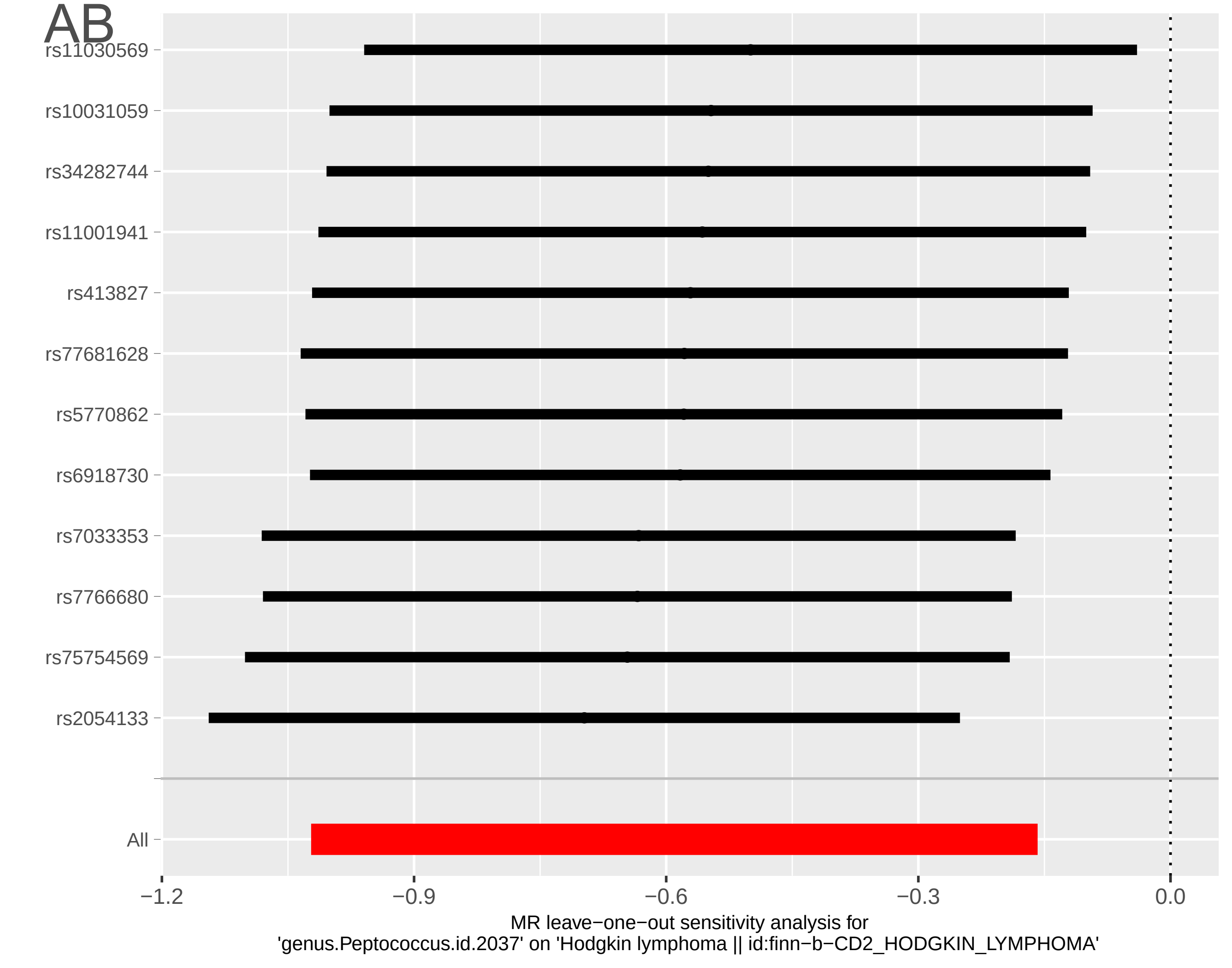
AB
rs11030569
rs10031059
rs34282744
rs11001941
rs413827
rs77681628
rs5770862
rs6918730
rs7033353
rs7766680
rs75754569
rs2054133
All
−1.2
−0.9
−0.6
−0.3
0.0
MR leave−one−out sensitivity analysis for
'genus.Peptococcus.id.2037' on 'Hodgkin lymphoma || id:finn−b−CD2_HODGKIN_LYMPHOMA'

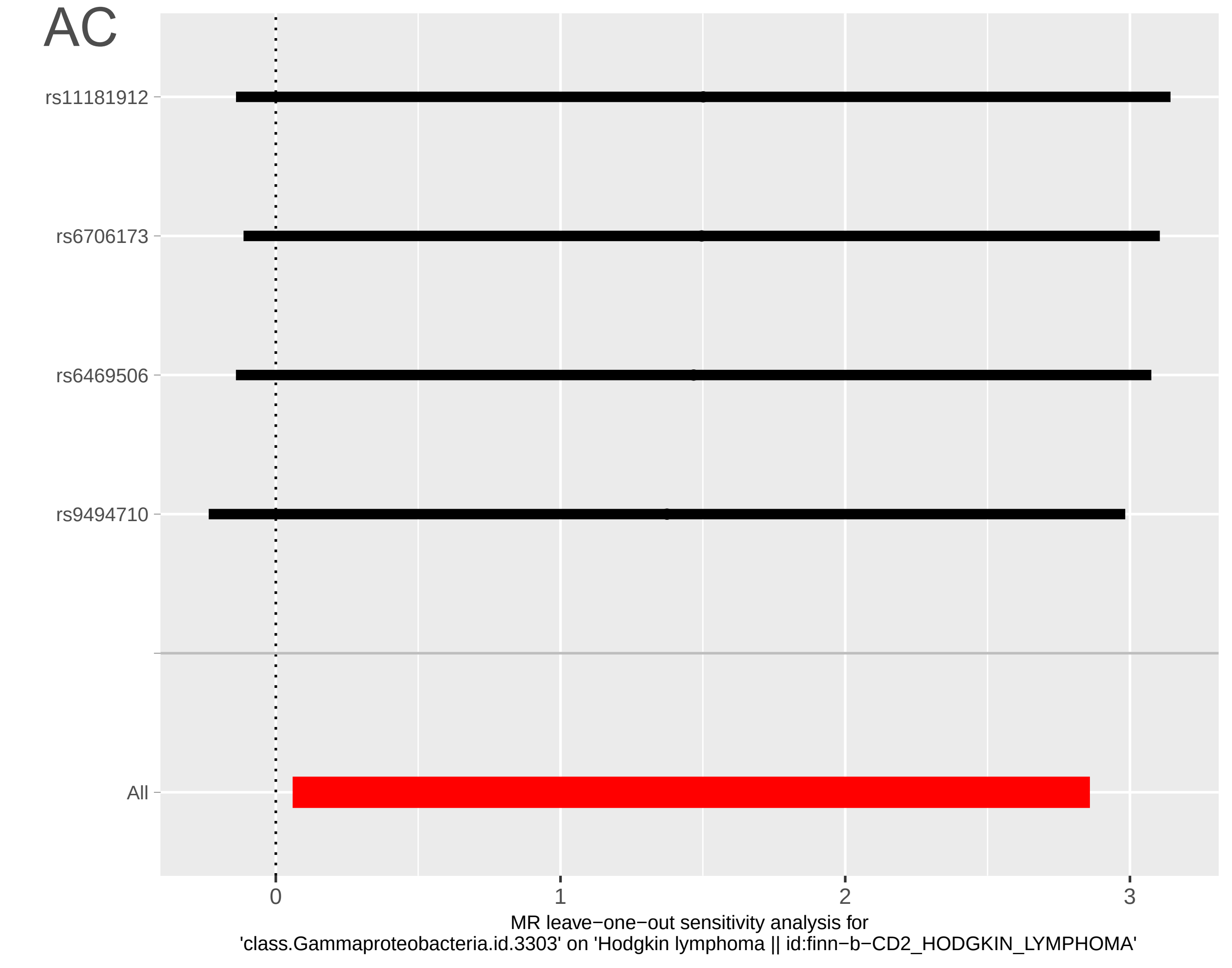
AC
rs11181912
rs6706173
rs6469506
rs9494710
All
0
1
2
3
MR leave−one−out sensitivity analysis for
'class.Gammaproteobacteria.id.3303' on 'Hodgkin lymphoma || id:finn−b−CD2_HODGKIN_LYMPHOMA'

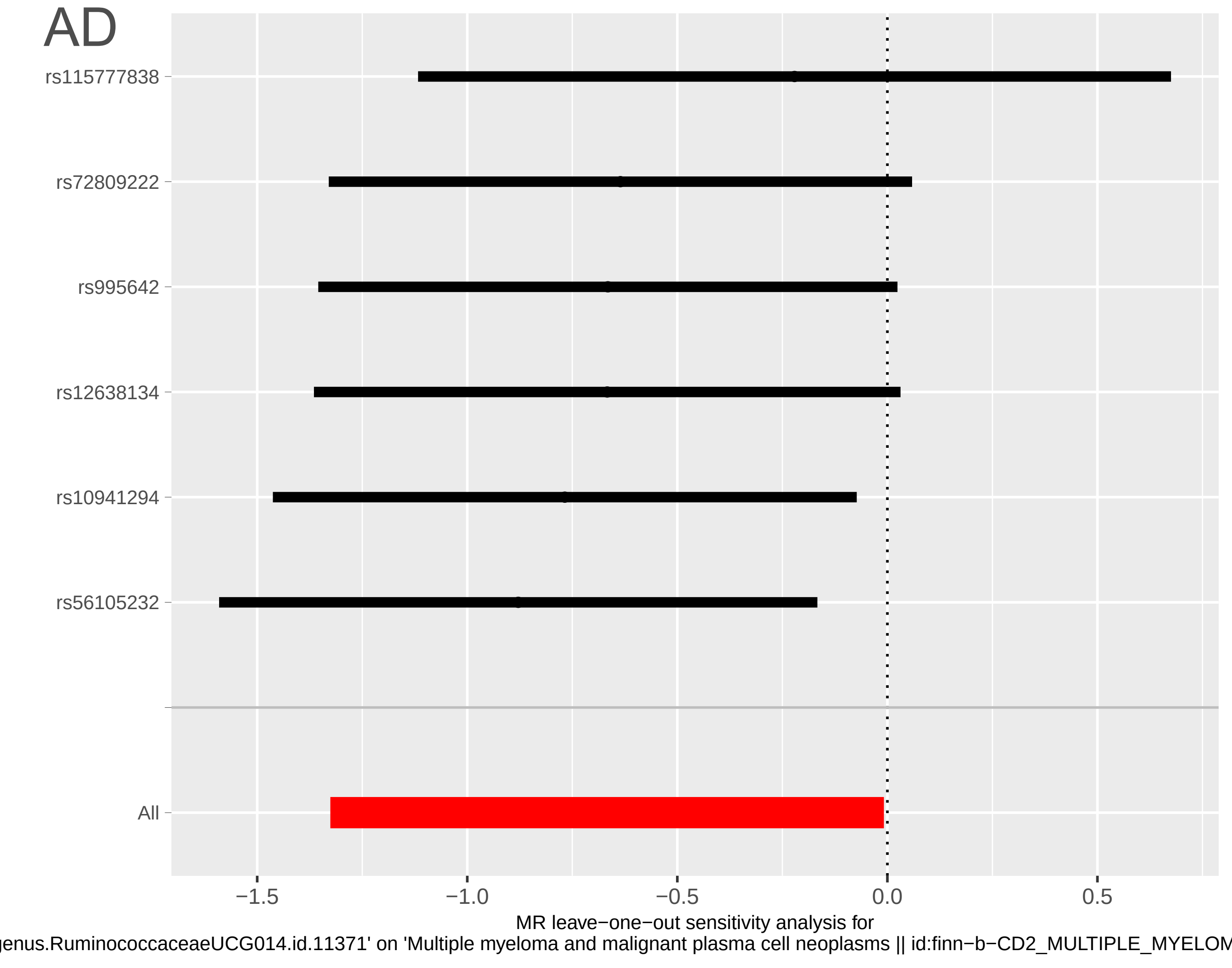
AD
rs115777838
rs72809222
rs995642
rs12638134
rs10941294
rs56105232
All
−1.5
−1.0
−0.5
0.0
0.5
MR leave−one−out sensitivity analysis for
genus.RuminococcaceaeUCG014.id.11371' on 'Multiple myeloma and malignant plasma cell neoplasms || id:finn−b−CD2_MULTIPLE_MYELOM

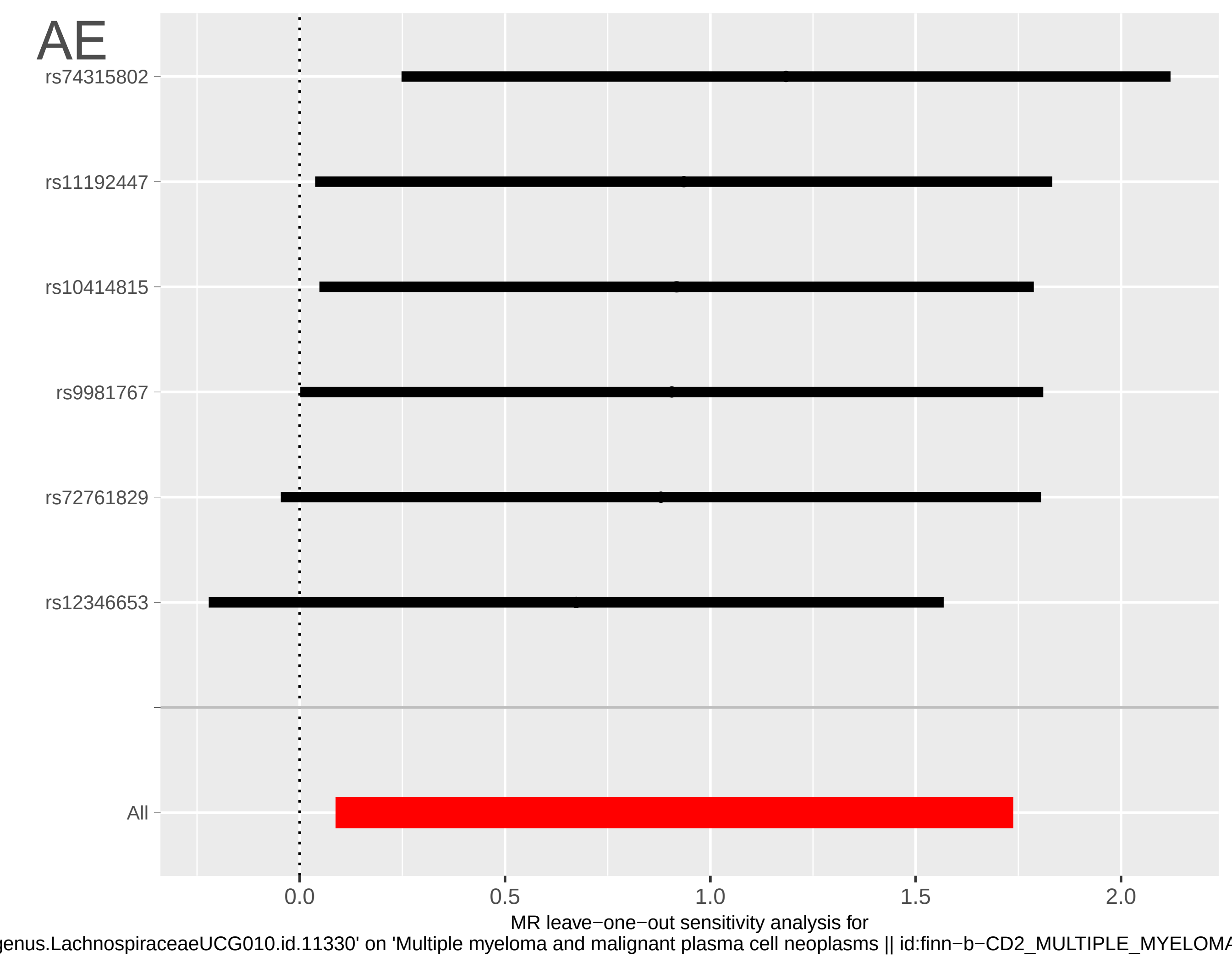
AE
rs74315802
rs11192447
rs10414815
rs9981767
rs72761829
rs12346653
All
0.0
0.5
1.0
1.5
2.0
MR leave−one−out sensitivity analysis for
genus.LachnospiraceaeUCG010.id.11330' on 'Multiple myeloma and malignant plasma cell neoplasms || id:finn−b−CD2_MULTIPLE_MYELOMA

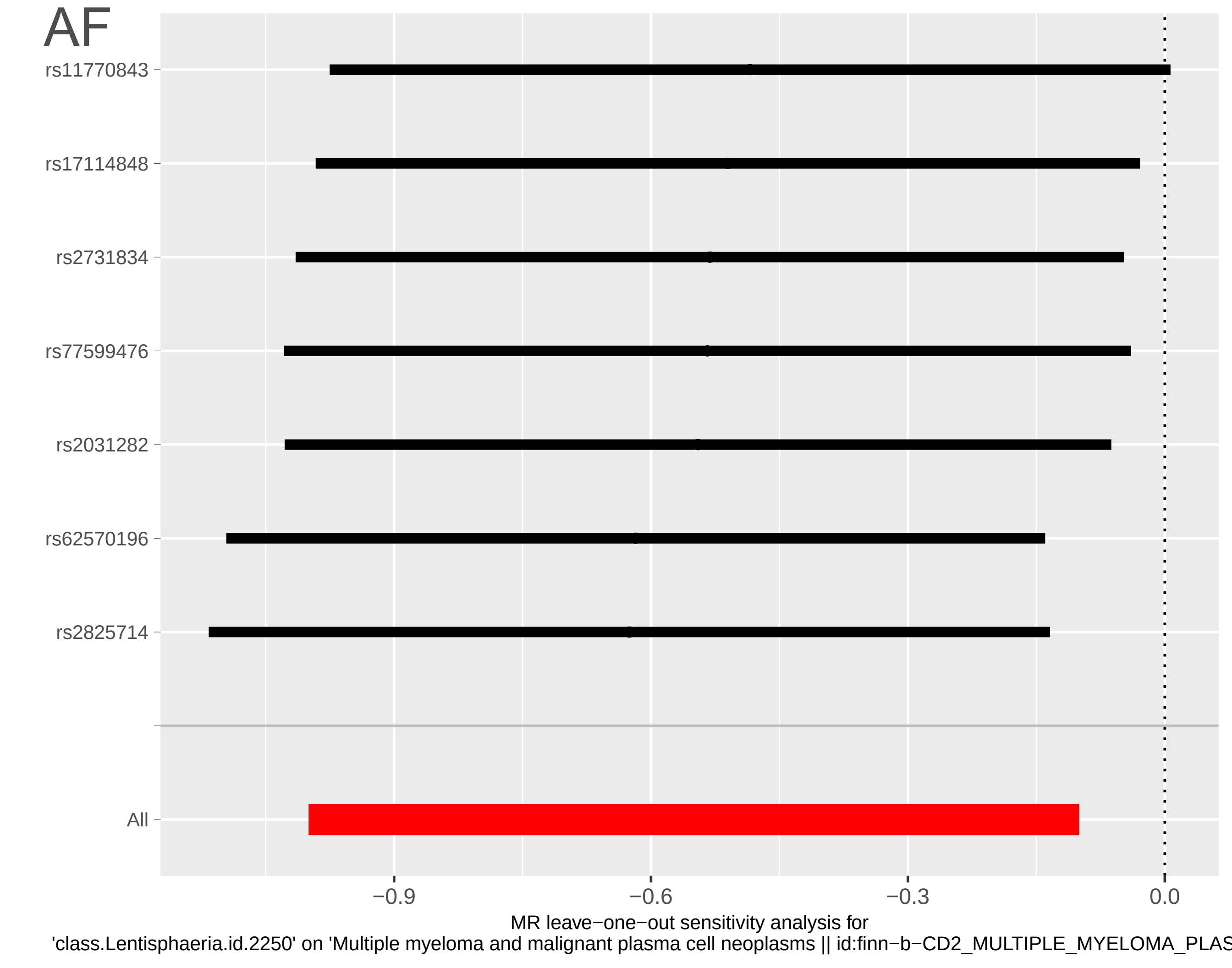
AF
rs11770843
rs17114848
rs2731834
rs77599476
rs2031282
rs62570196
rs2825714
All
−0.9
−0.6
−0.3
0.0
MR leave−one−out sensitivity analysis for
'class.Lentisphaeria.id.2250' on 'Multiple myeloma and malignant plasma cell neoplasms || id:finn−b−CD2_MULTIPLE_MYELOMA_PLAS

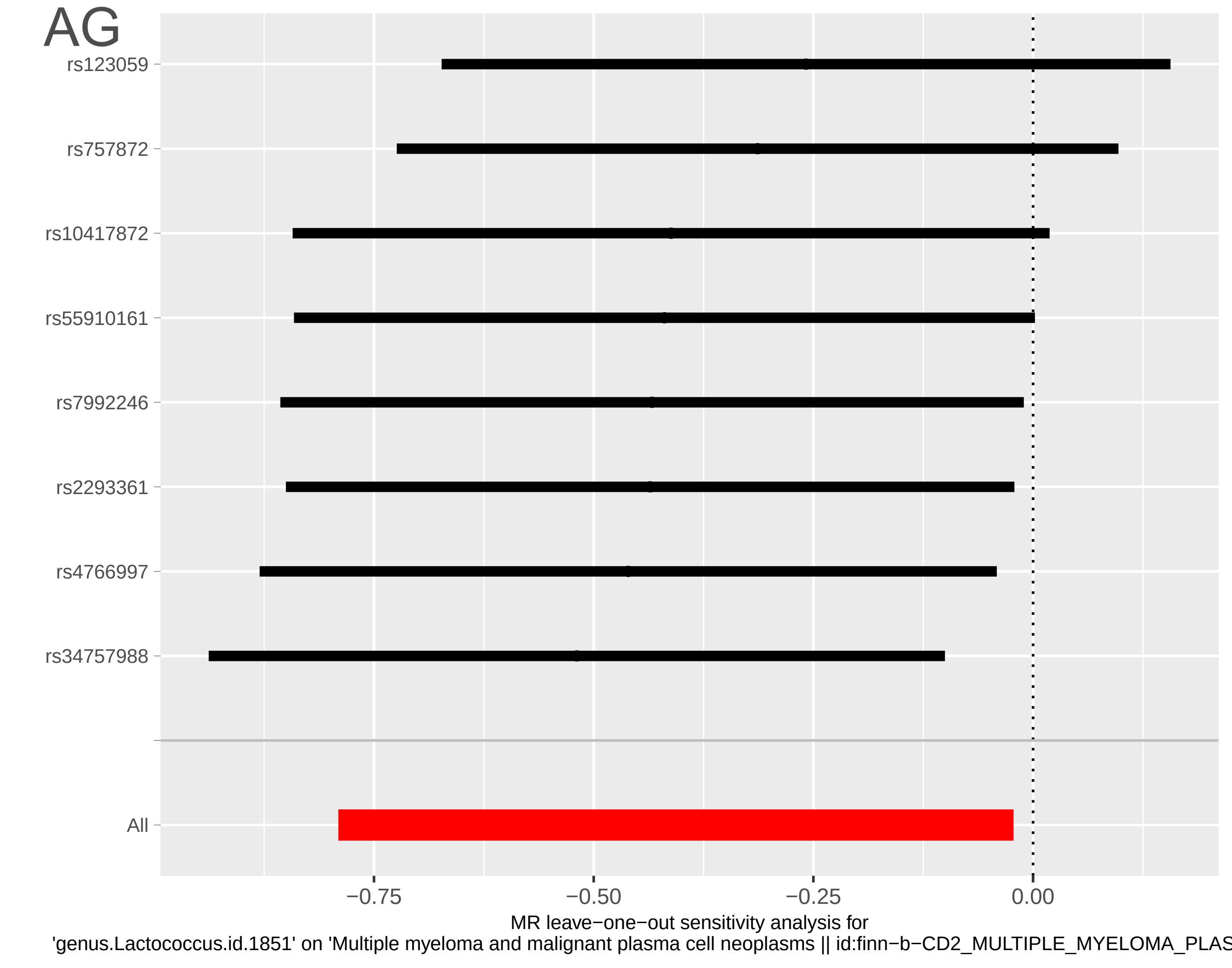
AG
rs123059
rs757872
rs10417872
rs55910161
rs7992246
rs2293361
rs4766997
rs34757988
All
−0.75
−0.50
−0.25
0.00
MR leave−one−out sensitivity analysis for
'genus.Lactococcus.id.1851' on 'Multiple myeloma and malignant plasma cell neoplasms || id:finn−b−CD2_MULTIPLE_MYELOMA_PLAS

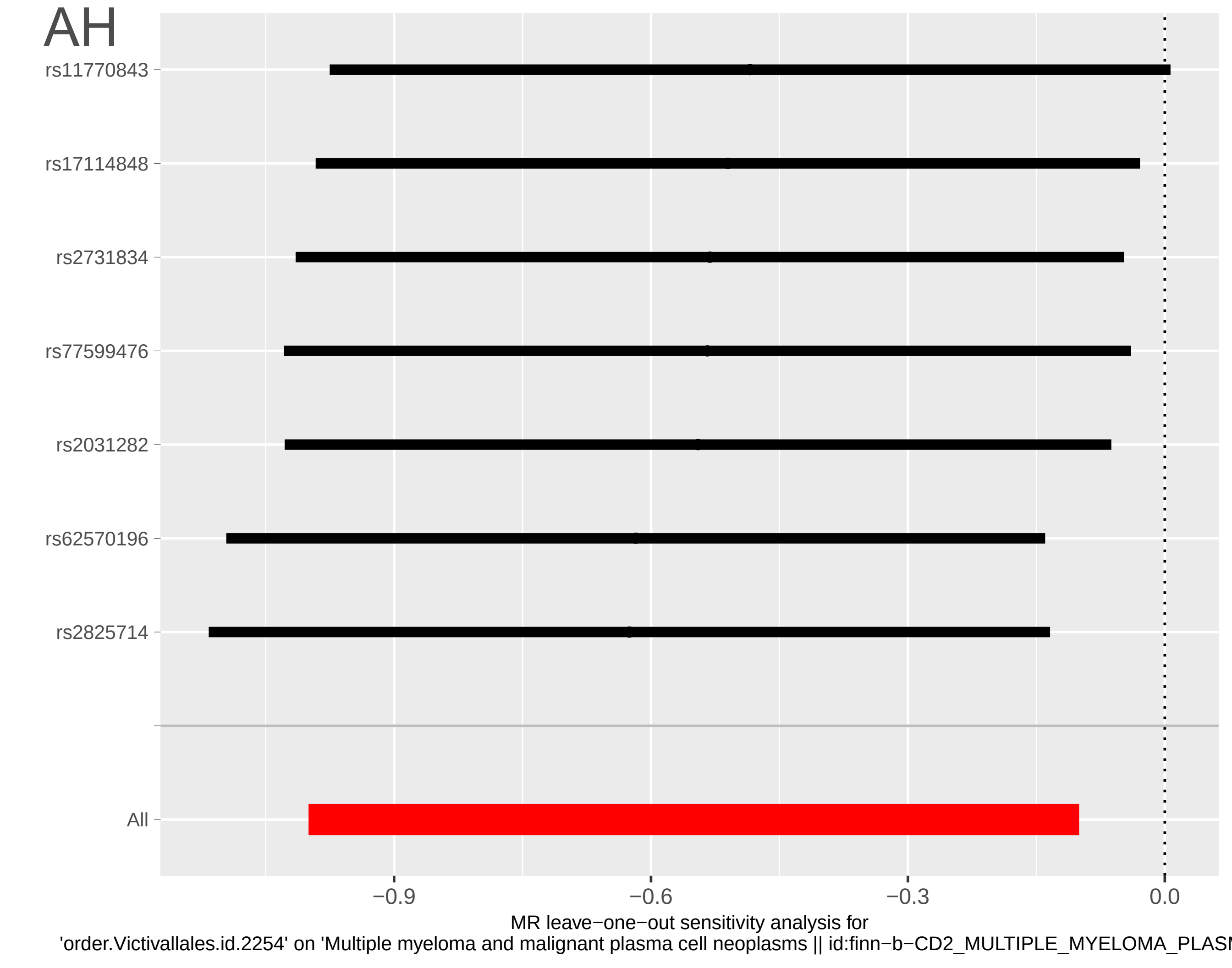
AH
rs11770843
rs17114848
rs2731834
rs77599476
rs2031282
rs62570196
rs2825714
All
−0.9
−0.6
−0.3
0.0
MR leave−one−out sensitivity analysis for
'order.Victivallales.id.2254' on 'Multiple myeloma and malignant plasma cell neoplasms || id:finn−b−CD2_MULTIPLE_MYELOMA_PLASM

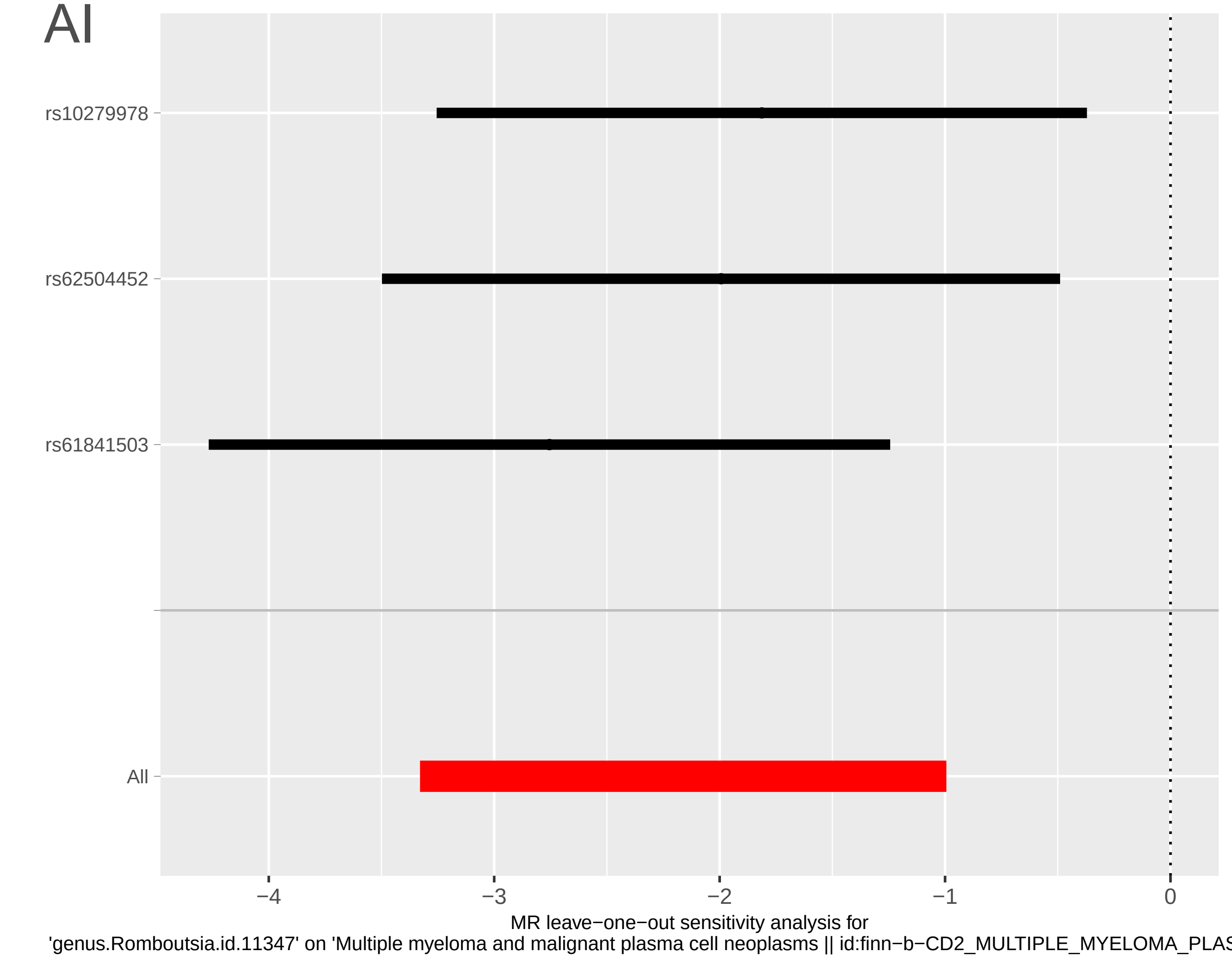
AI
rs10279978
rs62504452
rs61841503
All
−4
−3
−2
−1
0
MR leave−one−out sensitivity analysis for
'genus.Romboutsia.id.11347' on 'Multiple myeloma and malignant plasma cell neoplasms || id:finn−b−CD2_MULTIPLE_MYELOMA_PLAS

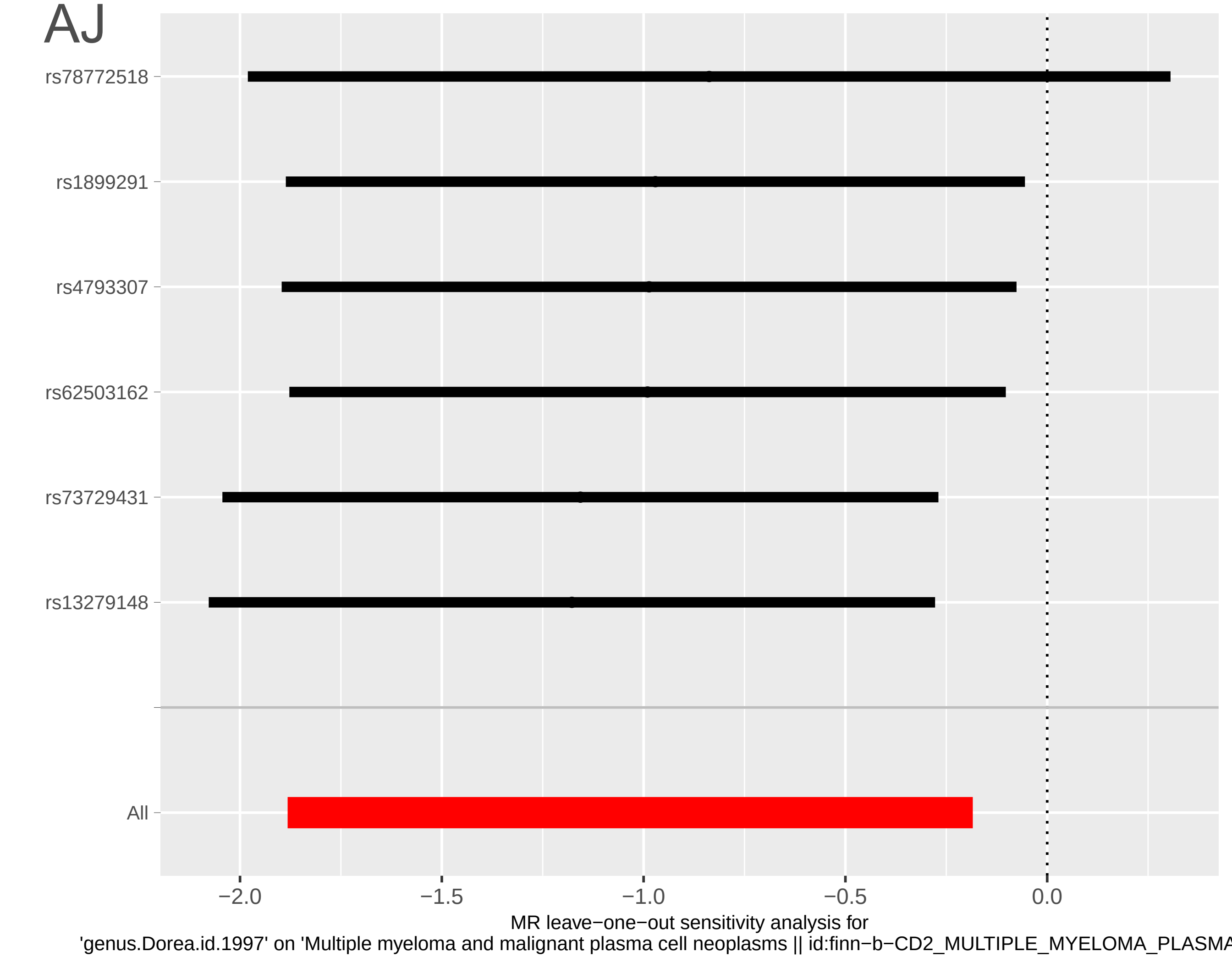
AJ
rs78772518
rs1899291
rs4793307
rs62503162
rs73729431
rs13279148
All
−2.0
−1.5
−1.0
−0.5
0.0
MR leave−one−out sensitivity analysis for
'genus.Dorea.id.1997' on 'Multiple myeloma and malignant plasma cell neoplasms || id:finn−b−CD2_MULTIPLE_MYELOMA_PLASMA

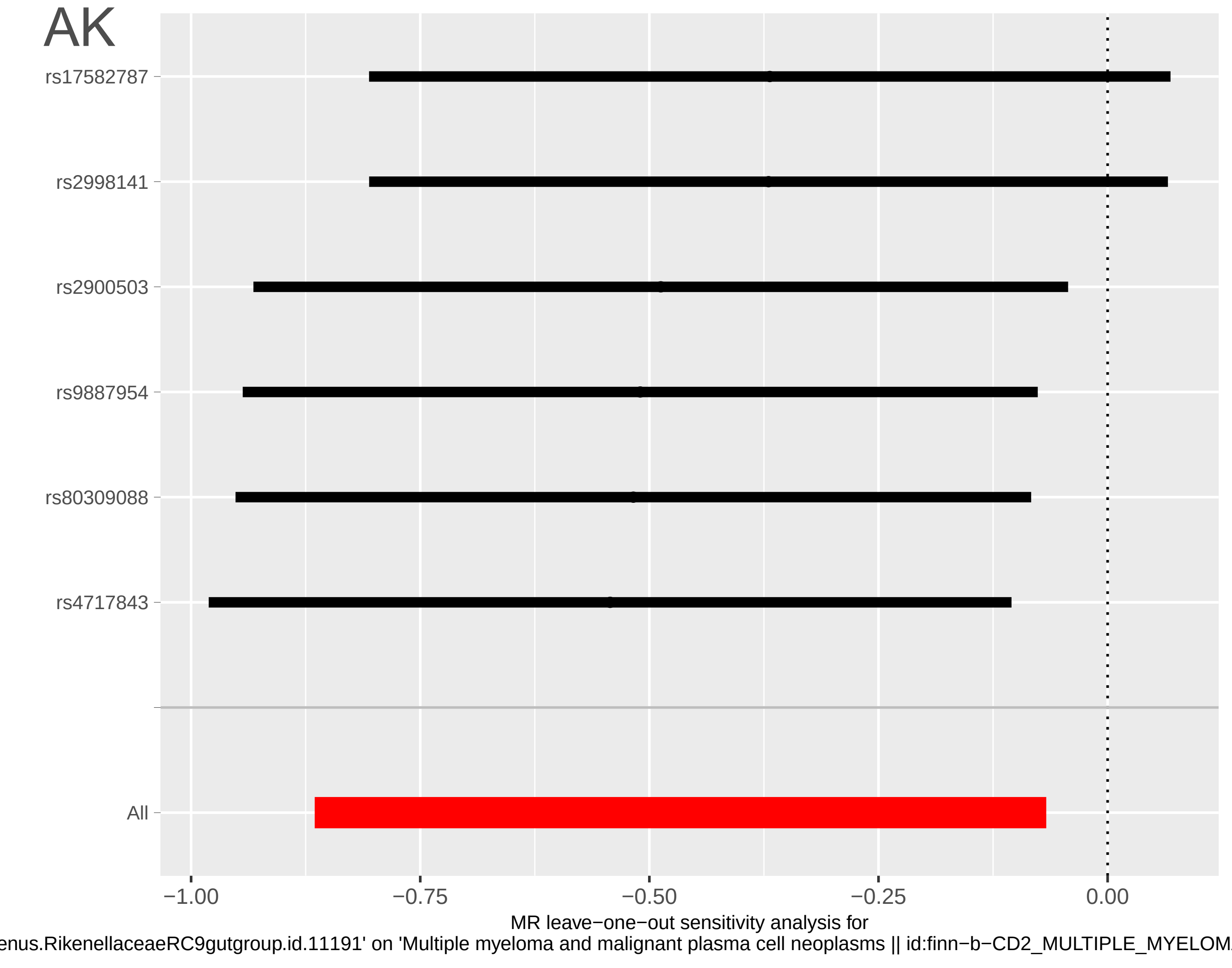
AK
rs17582787
rs2998141
rs2900503
rs9887954
rs80309088
rs4717843
All
−1.00
−0.75
−0.50
−0.25
0.00
MR leave−one−out sensitivity analysis for
enus.RikenellaceaeRC9gutgroup.id.11191' on 'Multiple myeloma and malignant plasma cell neoplasms || id:finn−b−CD2_MULTIPLE_MYELOM

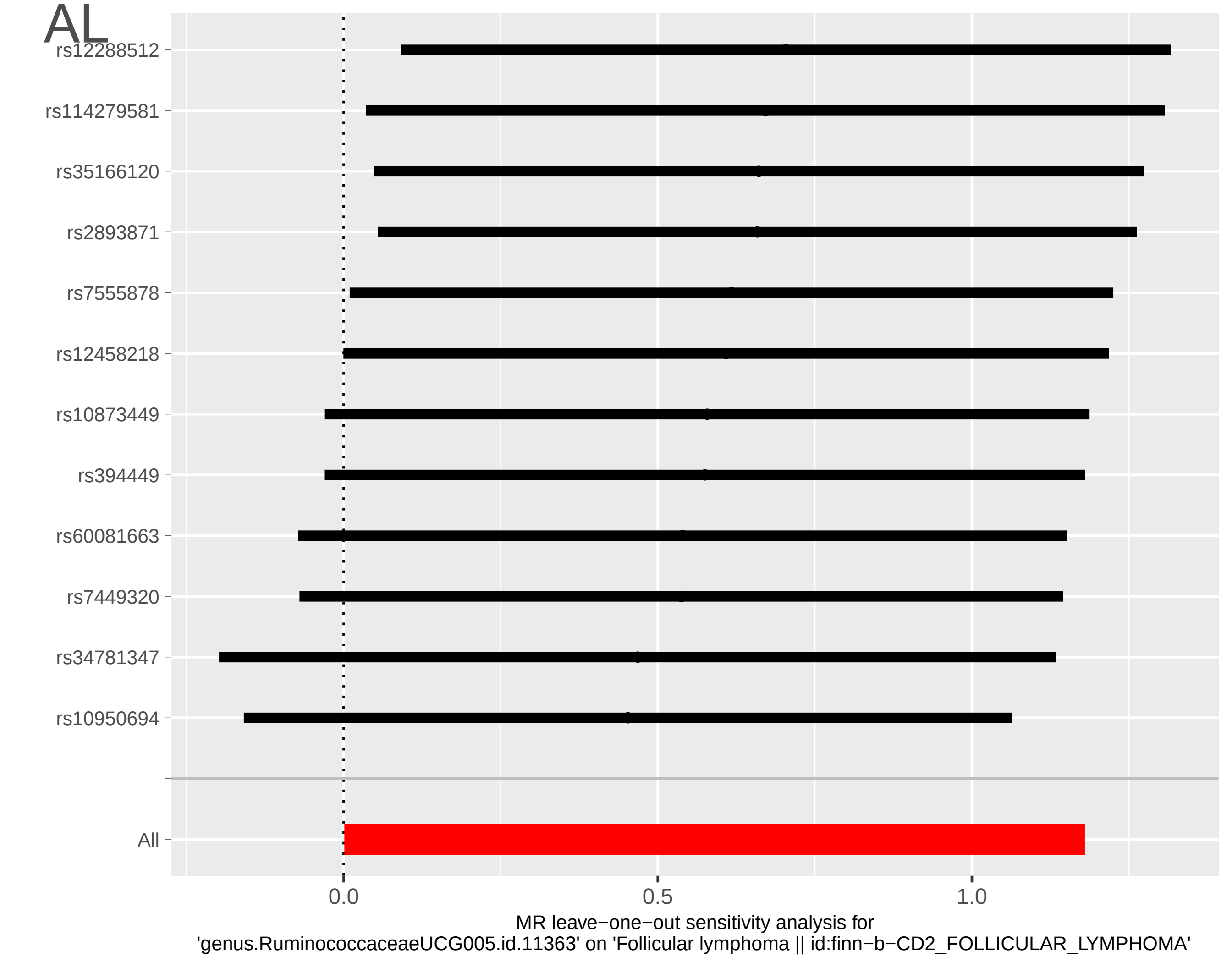
AL
rs12288512
rs114279581
rs35166120
rs2893871
rs7555878
rs12458218
rs10873449
rs394449
rs60081663
rs7449320
rs34781347
rs10950694
All
0.0
0.5
1.0
MR leave−one−out sensitivity analysis for
'genus.RuminococcaceaeUCG005.id.11363' on 'Follicular lymphoma || id:finn−b−CD2_FOLLICULAR_LYMPHOMA'

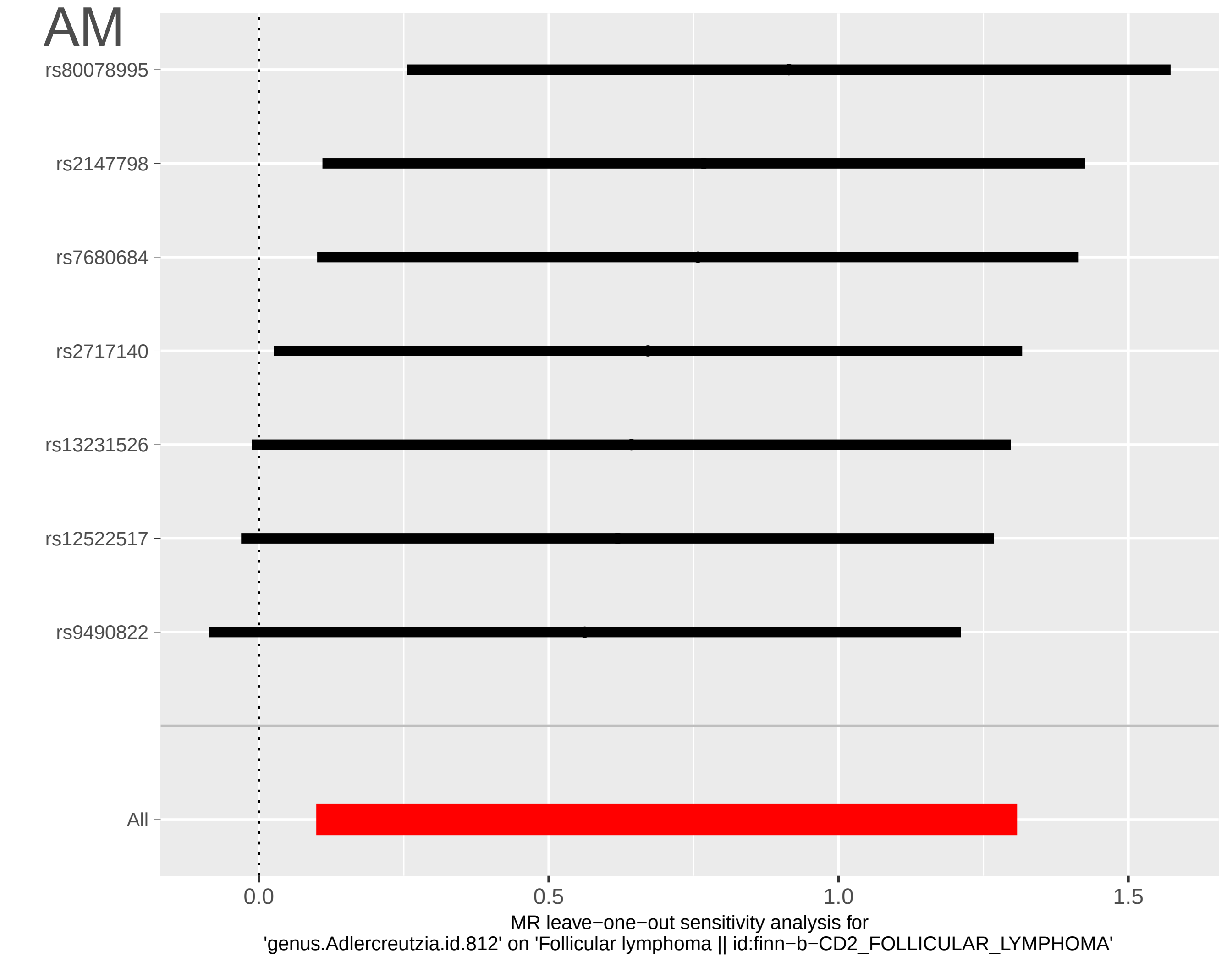
AM
rs80078995
rs2147798
rs7680684
rs2717140
rs13231526
rs12522517
rs9490822
All
0.0
0.5
1.0
1.5
MR leave−one−out sensitivity analysis for
'genus.Adlercreutzia.id.812' on 'Follicular lymphoma || id:finn−b−CD2_FOLLICULAR_LYMPHOMA'

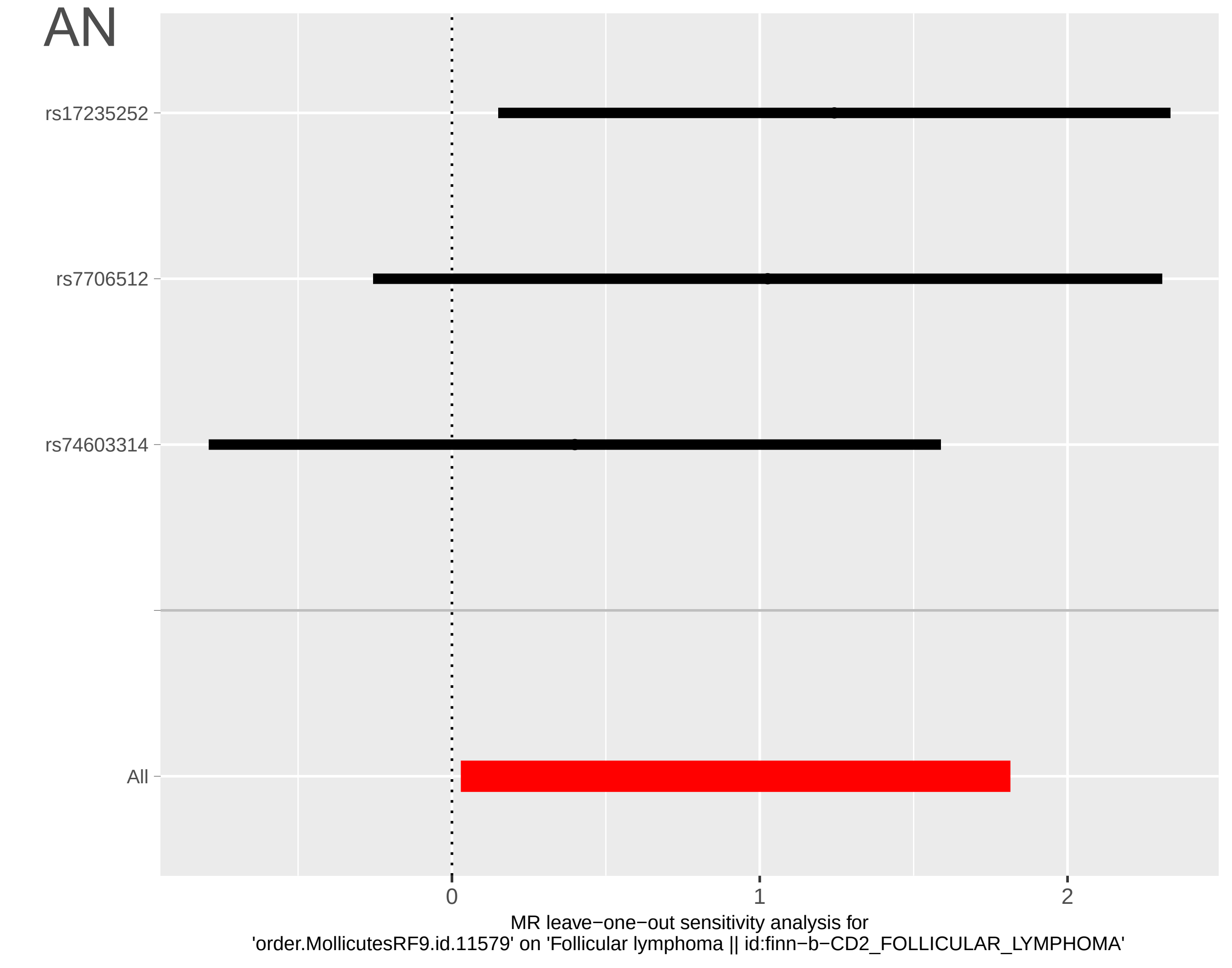
AN
rs17235252
rs7706512
rs74603314
All
0
1
2
MR leave−one−out sensitivity analysis for
'order.MollicutesRF9.id.11579' on 'Follicular lymphoma || id:finn−b−CD2_FOLLICULAR_LYMPHOMA'

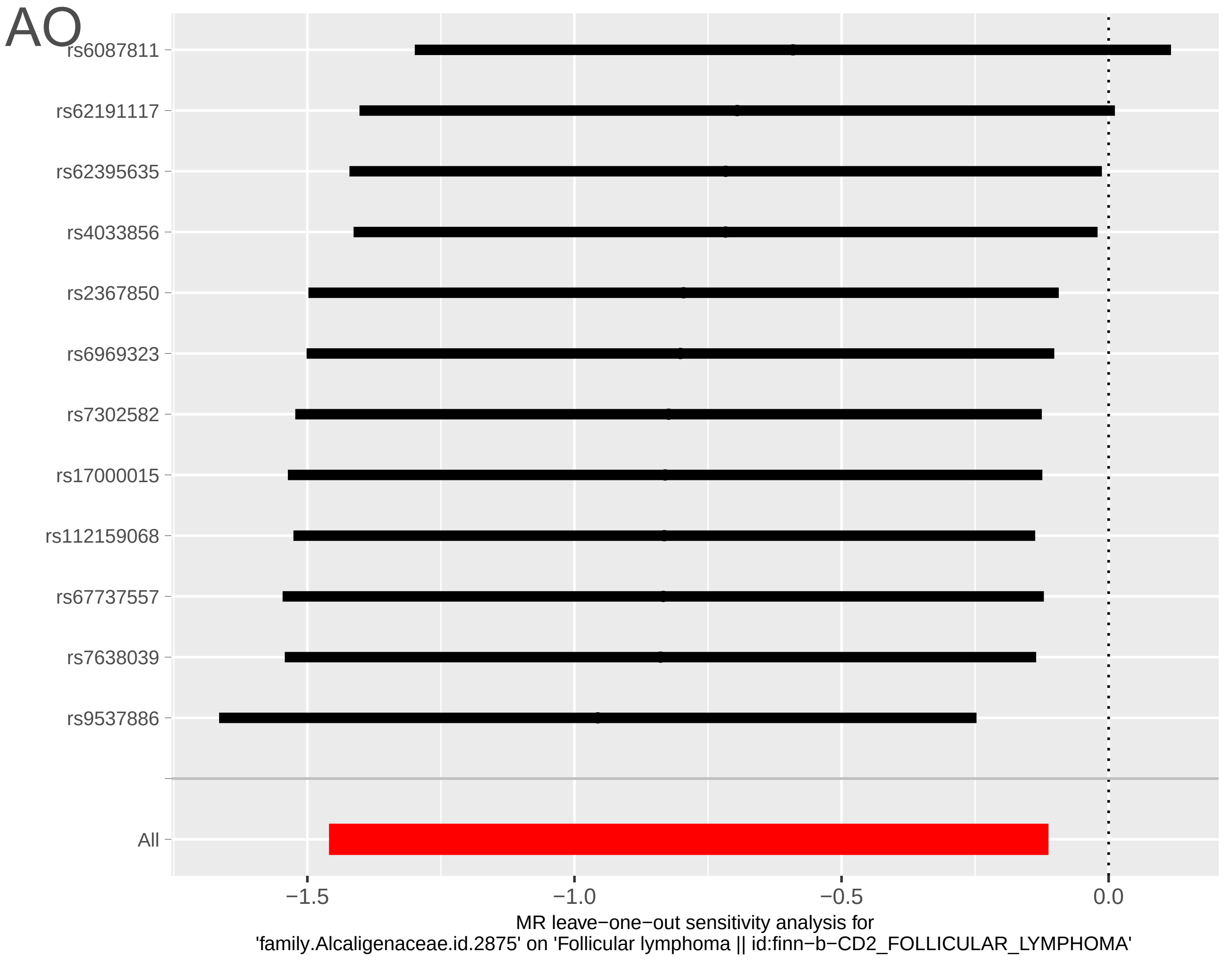
AO
rs6087811
rs62191117
rs62395635
rs4033856
rs2367850
rs6969323
rs7302582
rs17000015
rs112159068
rs67737557
rs7638039
rs9537886
All
−1.5
−1.0
−0.5
0.0
MR leave−one−out sensitivity analysis for
'family.Alcaligenaceae.id.2875' on 'Follicular lymphoma || id:finn−b−CD2_FOLLICULAR_LYMPHOMA'

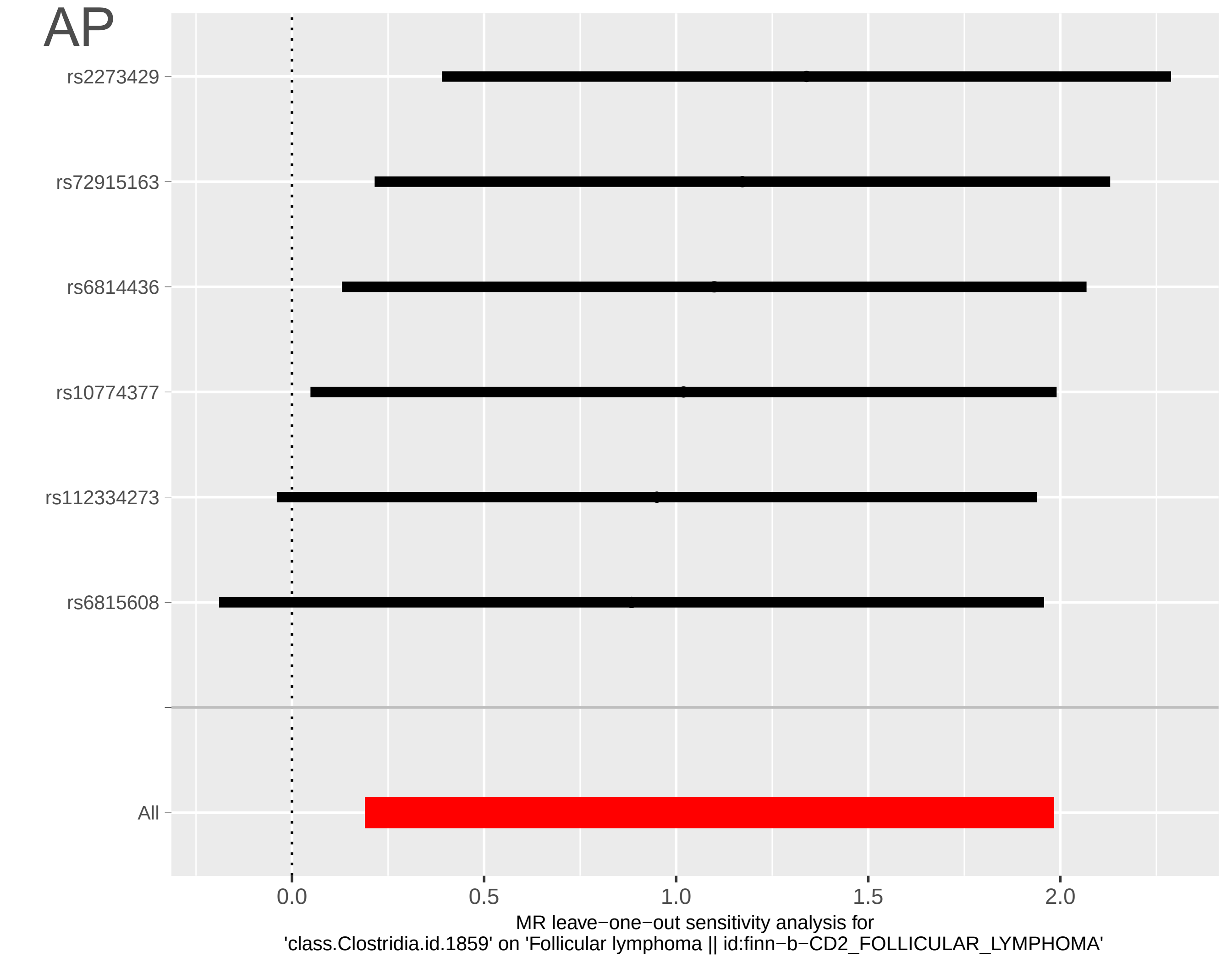
AP
rs2273429
rs72915163
rs6814436
rs10774377
rs112334273
rs6815608
All
0.0
0.5
1.0
1.5
2.0
MR leave−one−out sensitivity analysis for
'class.Clostridia.id.1859' on 'Follicular lymphoma || id:finn−b−CD2_FOLLICULAR_LYMPHOMA'

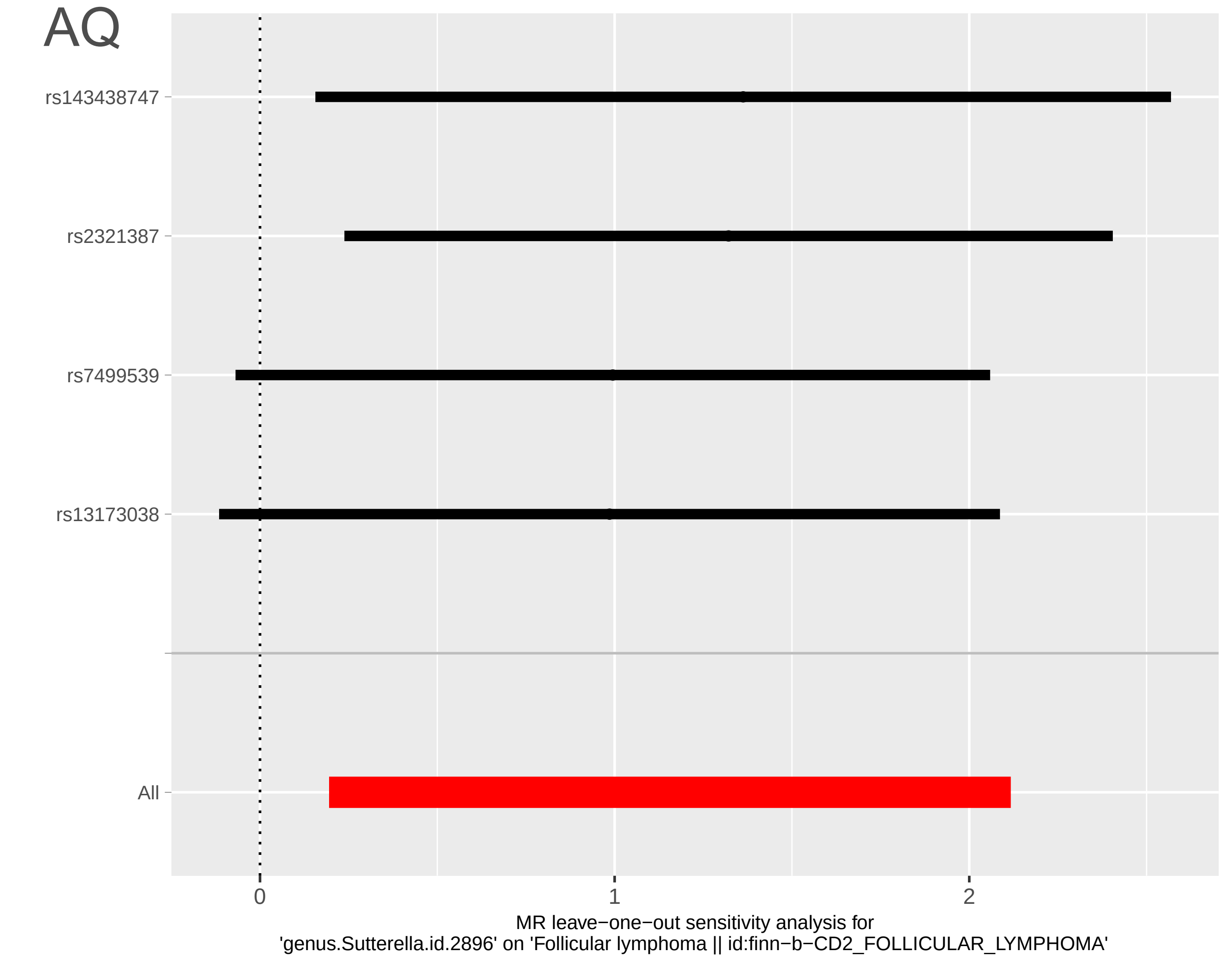
AQ
rs143438747
rs2321387
rs7499539
rs13173038
All
0
1
2
MR leave−one−out sensitivity analysis for
'genus.Sutterella.id.2896' on 'Follicular lymphoma || id:finn−b−CD2_FOLLICULAR_LYMPHOMA'

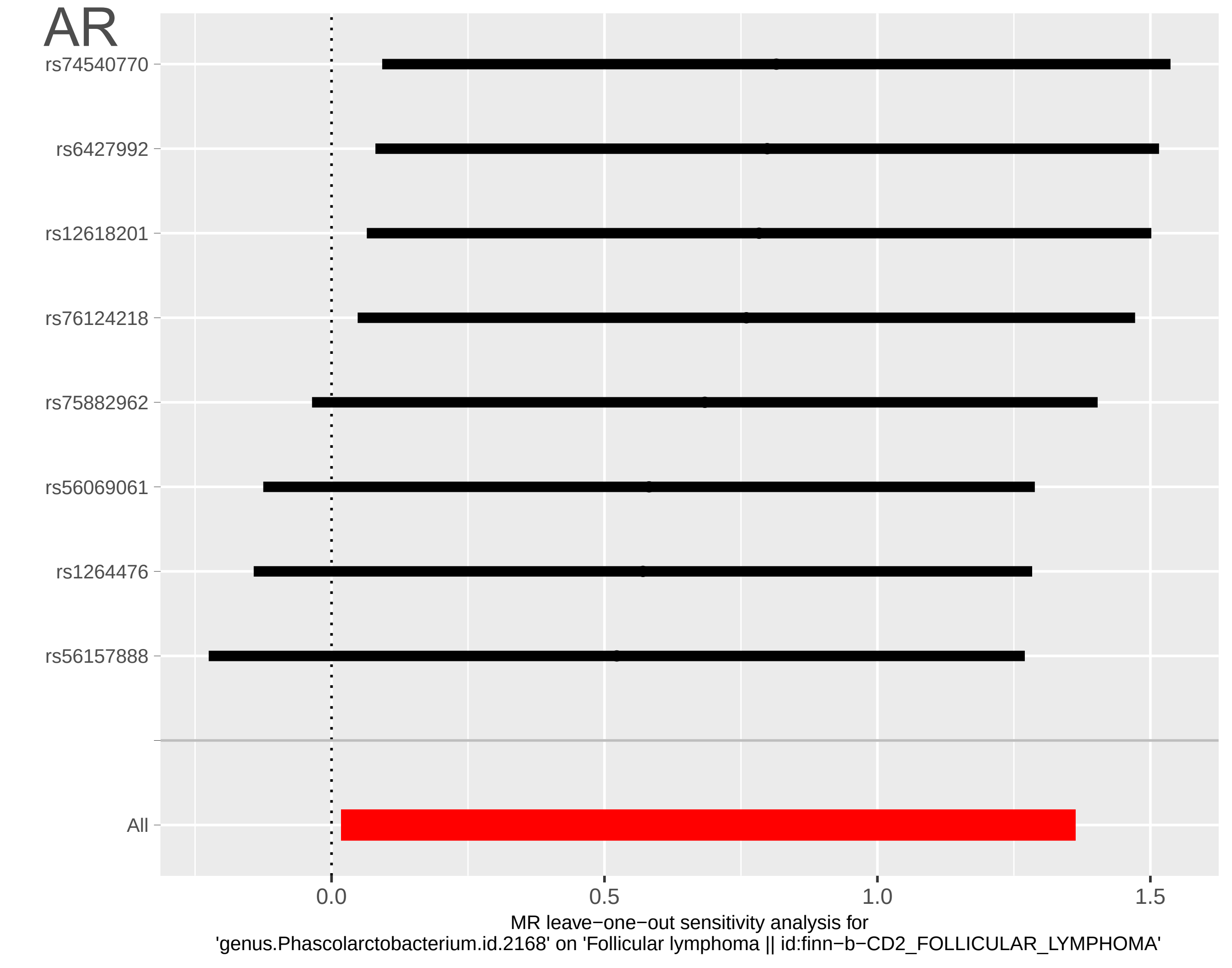
AR
rs74540770
rs6427992
rs12618201
rs76124218
rs75882962
rs56069061
rs1264476
rs56157888
All
0.0
0.5
1.0
1.5
MR leave−one−out sensitivity analysis for
'genus.Phascolarctobacterium.id.2168' on 'Follicular lymphoma || id:finn−b−CD2_FOLLICULAR_LYMPHOMA'

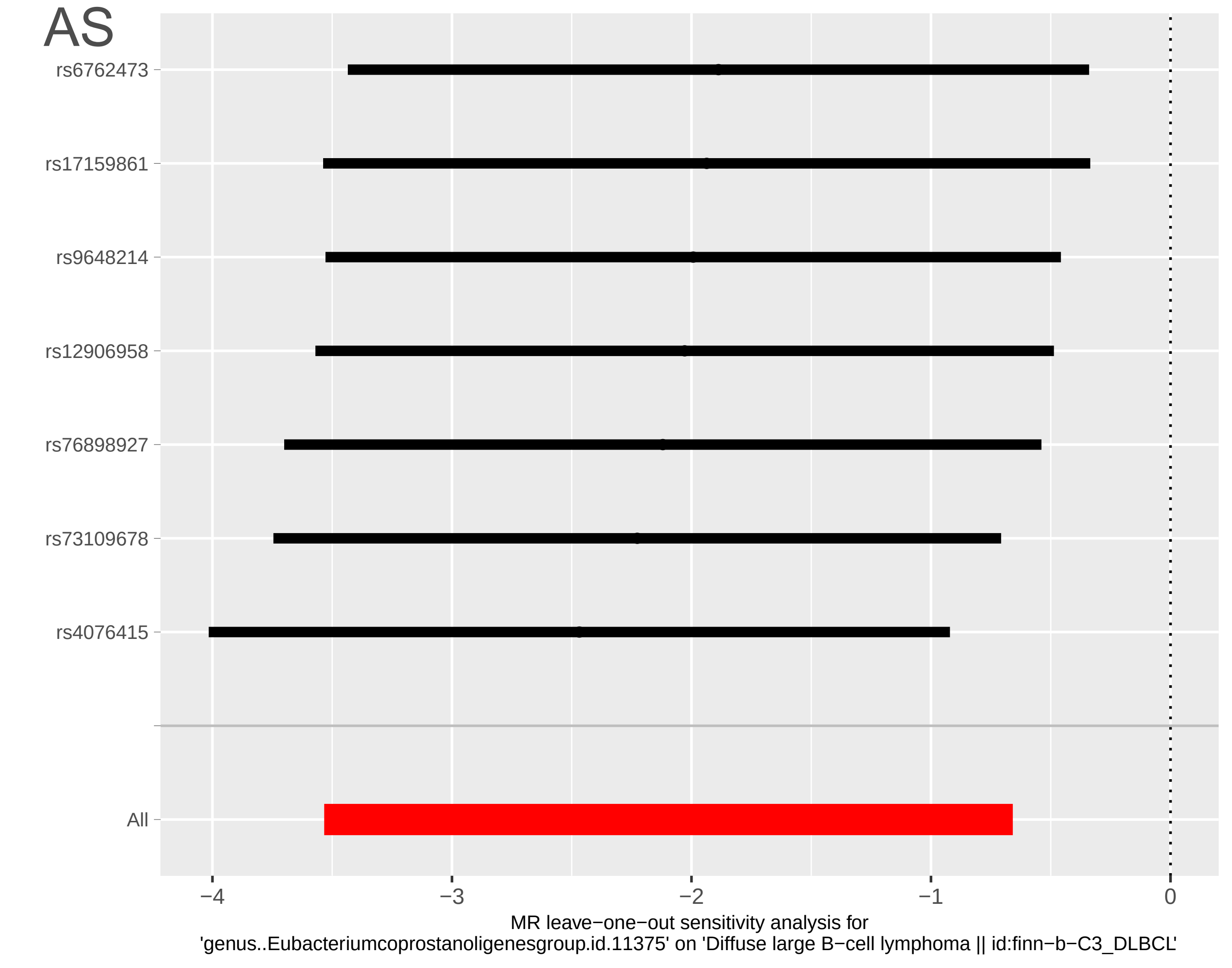
AS
rs6762473
rs17159861
rs9648214
rs12906958
rs76898927
rs73109678
rs4076415
All
−4
−3
−2
−1
0
MR leave−one−out sensitivity analysis for
'genus..Eubacteriumcoprostanoligenesgroup.id.11375' on 'Diffuse large B−cell lymphoma || id:finn−b−C3_DLBCL'

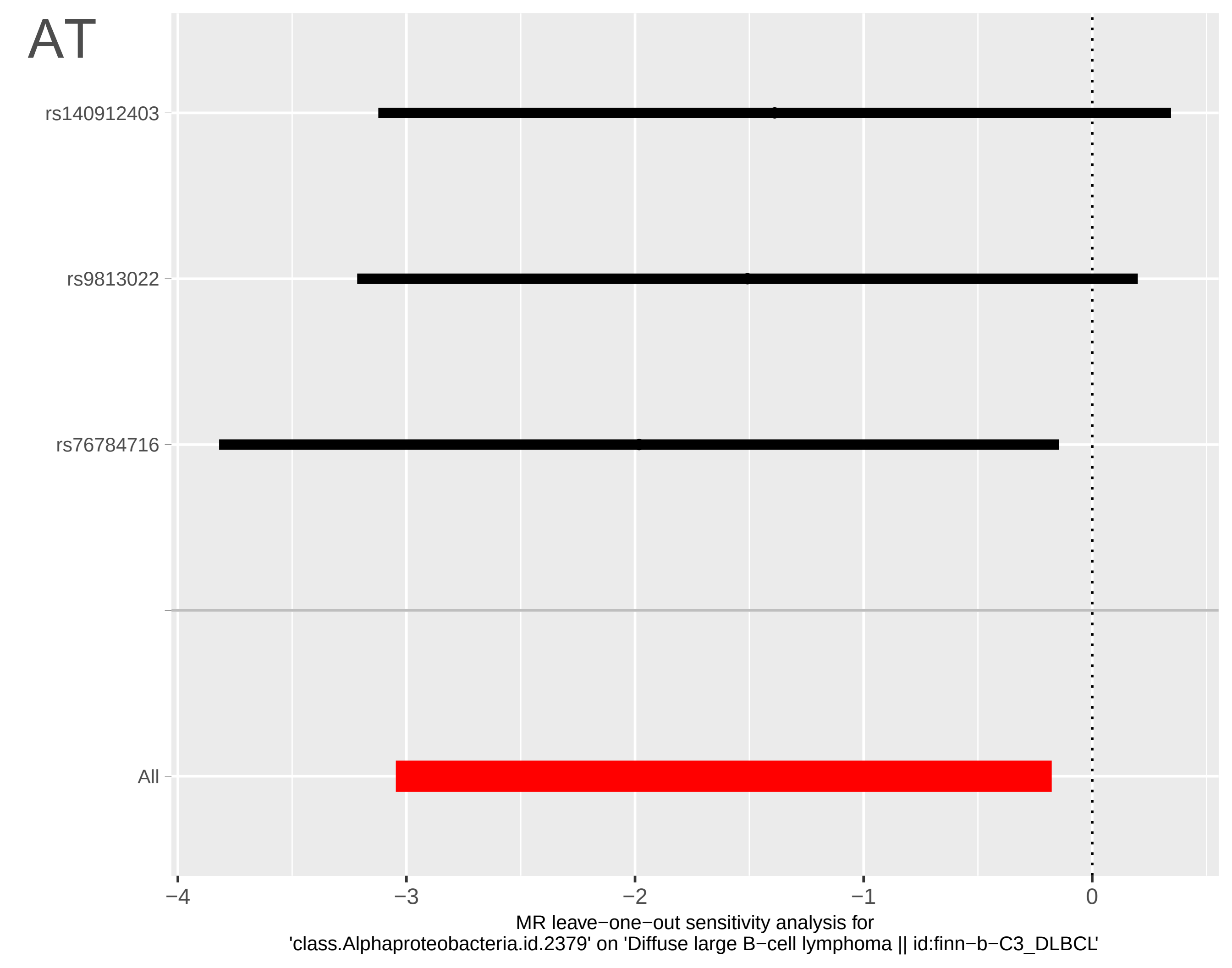

AT
rs140912403
rs9813022
rs76784716
All
−4
−3
−2
−1
0
MR leave−one−out sensitivity analysis for
'class.Alphaproteobacteria.id.2379' on 'Diffuse large B−cell lymphoma || id:finn−b−C3_DLBCL'

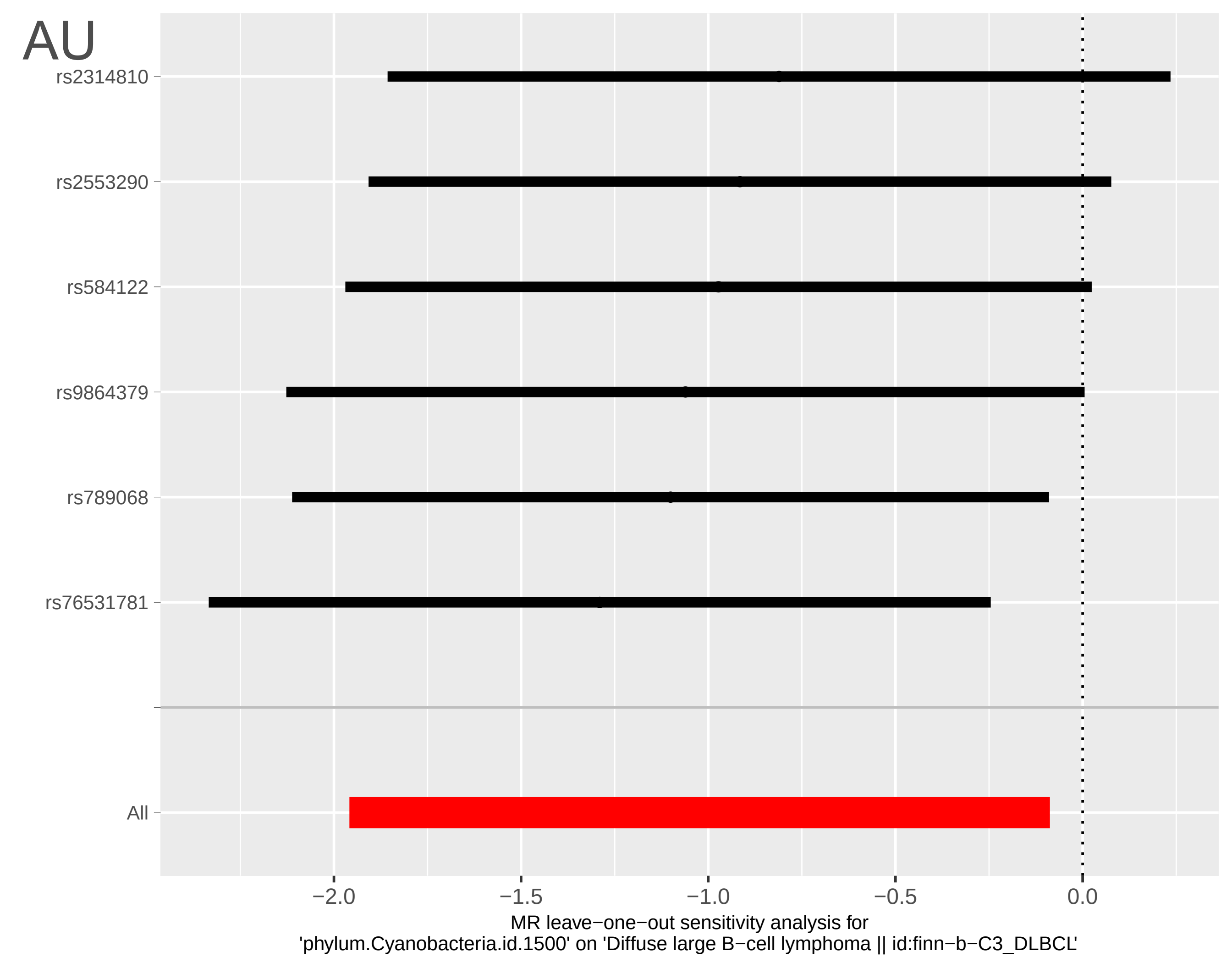
AU
rs2314810
rs2553290
rs584122
rs9864379
rs789068
rs76531781
All
−2.0
−1.5
−1.0
−0.5
0.0
MR leave−one−out sensitivity analysis for
'phylum.Cyanobacteria.id.1500' on 'Diffuse large B−cell lymphoma || id:finn−b−C3_DLBCL'

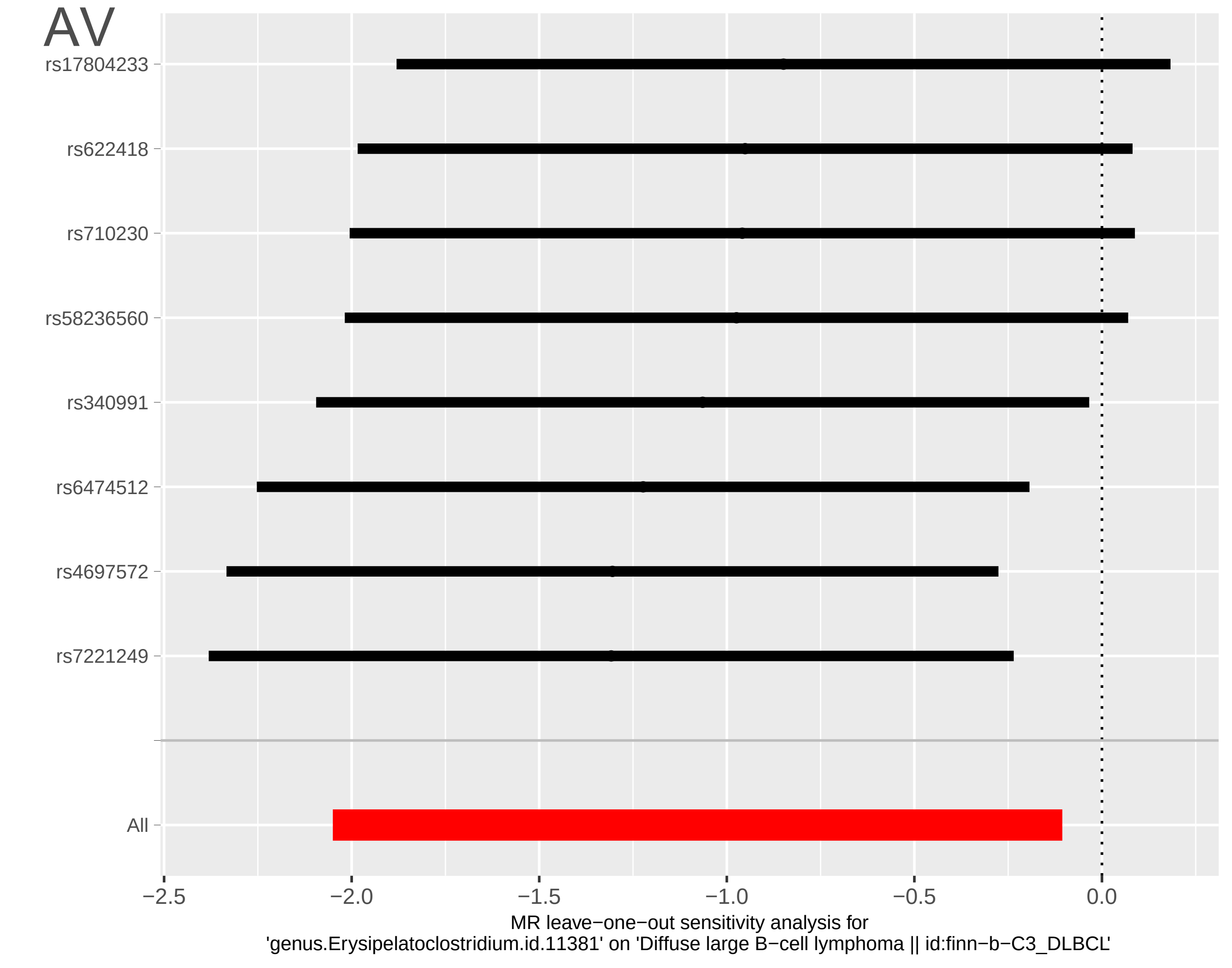

AV
rs17804233
rs622418
rs710230
rs58236560
rs340991
rs6474512
rs4697572
rs7221249
All
−2.5
−2.0
−1.5
−1.0
−0.5
0.0
MR leave−one−out sensitivity analysis for
'genus.Erysipelatoclostridium.id.11381' on 'Diffuse large B−cell lymphoma || id:finn−b−C3_DLBCL'

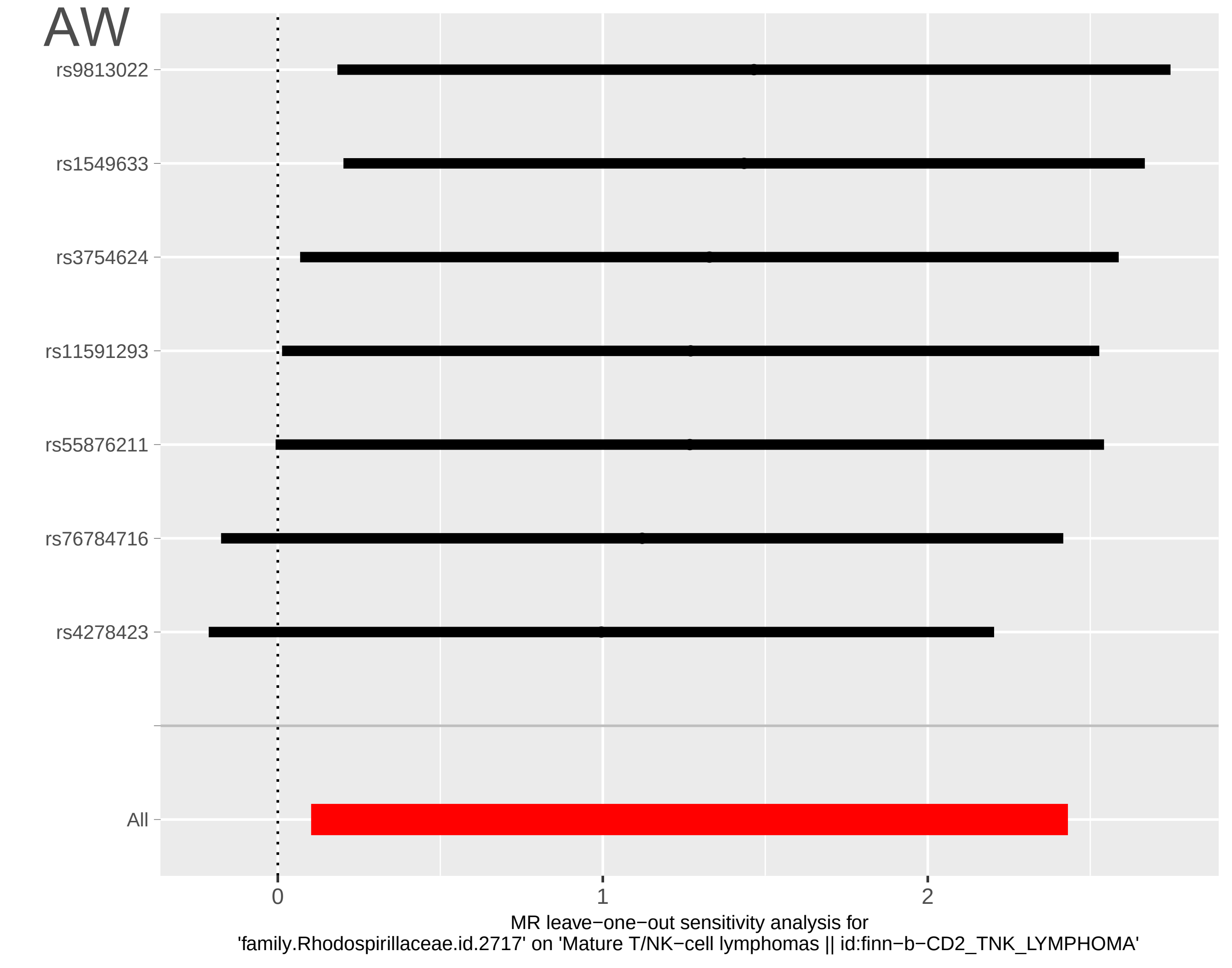
AW
rs9813022
rs1549633
rs3754624
rs11591293
rs55876211
rs76784716
rs4278423
All
0
1
2
MR leave−one−out sensitivity analysis for
'family.Rhodospirillaceae.id.2717' on 'Mature T/NK−cell lymphomas || id:finn−b−CD2_TNK_LYMPHOMA'

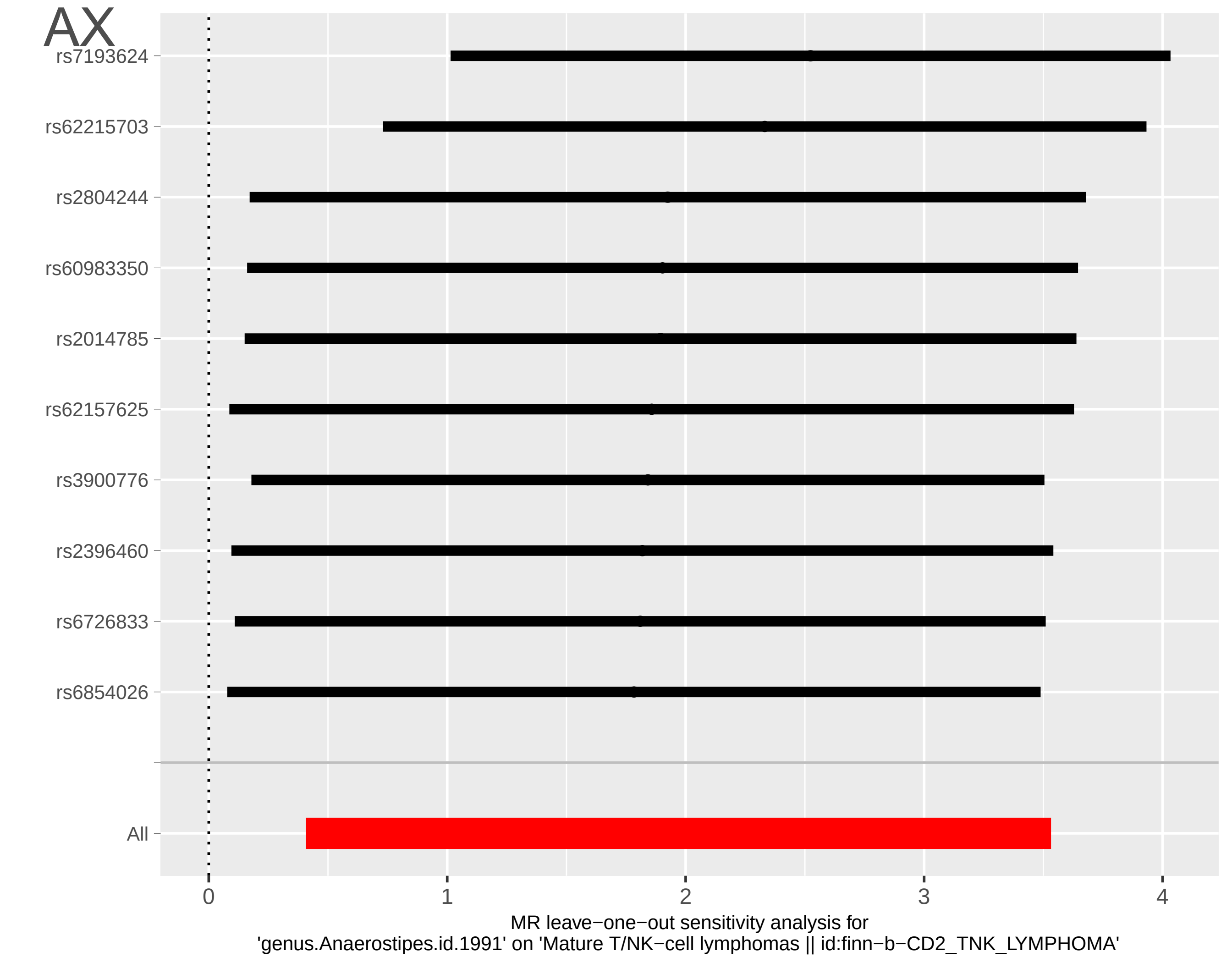
AX
rs7193624
rs62215703
rs2804244
rs60983350
rs2014785
rs62157625
rs3900776
rs2396460
rs6726833
rs6854026
All
0
1
2
3
4
MR leave−one−out sensitivity analysis for
'genus.Anaerostipes.id.1991' on 'Mature T/NK−cell lymphomas || id:finn−b−CD2_TNK_LYMPHOMA'

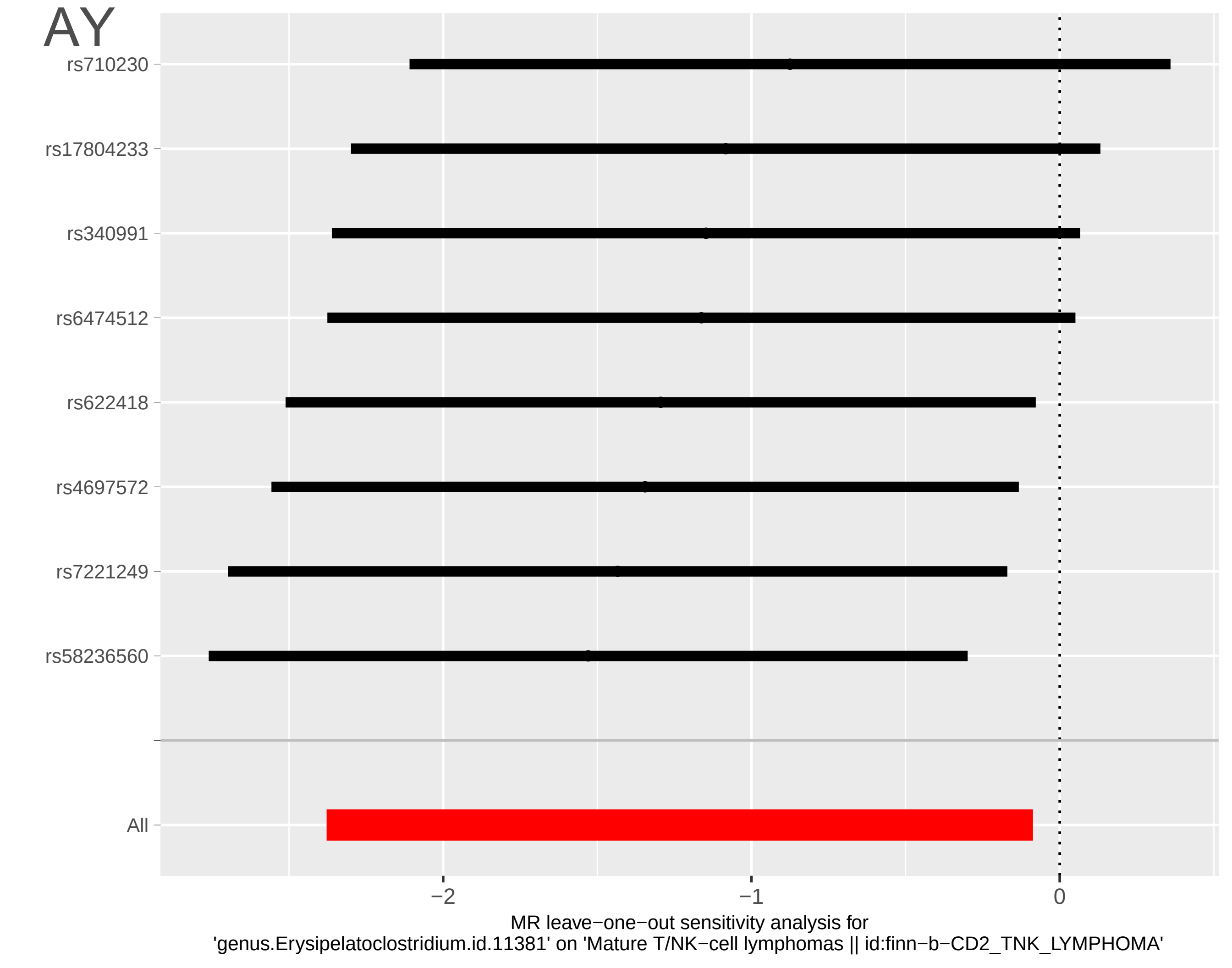
AY
rs710230
rs17804233
rs340991
rs6474512
rs622418
rs4697572
rs7221249
rs58236560
All
−2
−1
0
MR leave−one−out sensitivity analysis for
'genus.Erysipelatoclostridium.id.11381' on 'Mature T/NK−cell lymphomas || id:finn−b−CD2_TNK_LYMPHOMA'

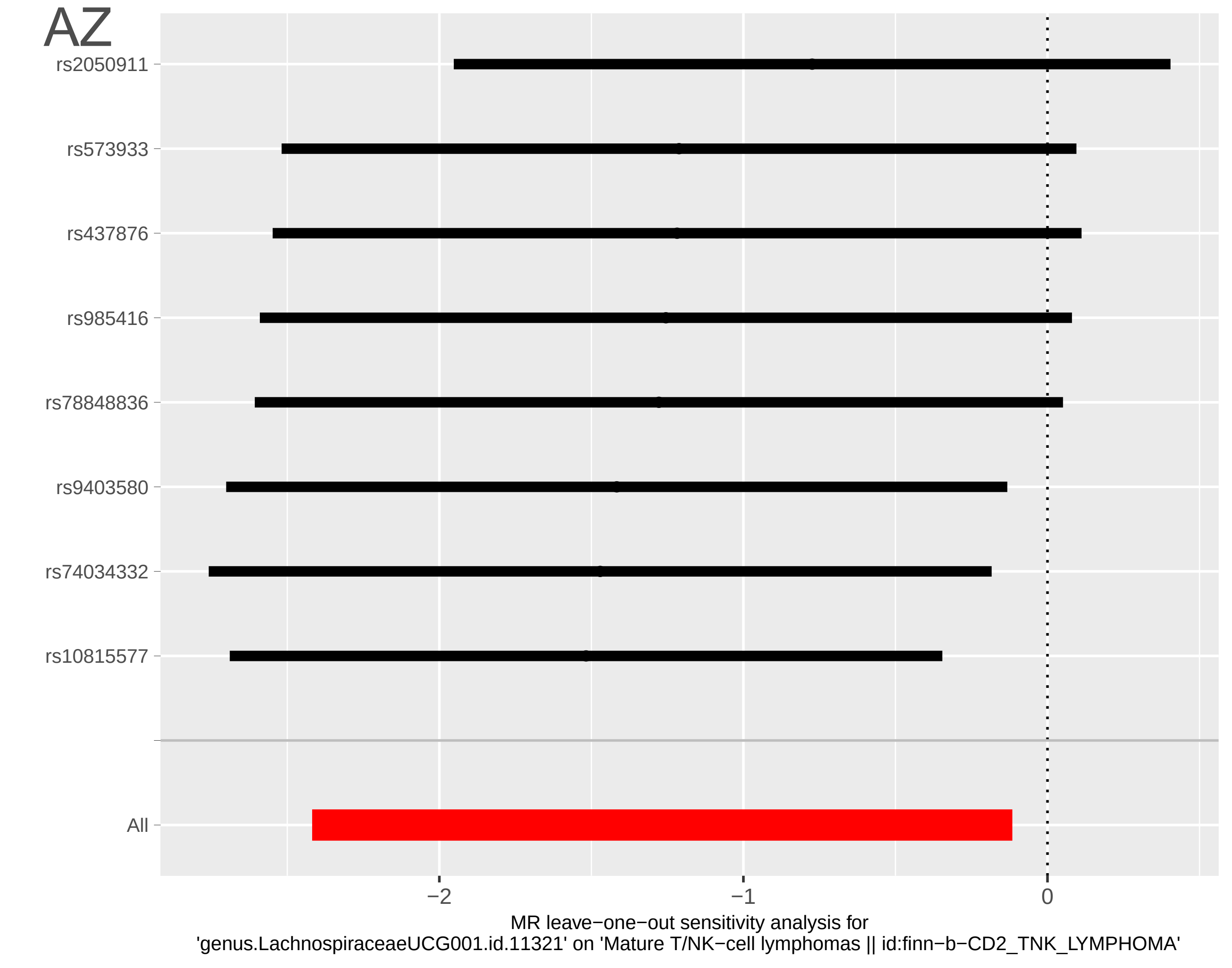

AZ
rs2050911
rs573933
rs437876
rs985416
rs78848836
rs9403580
rs74034332
rs10815577
All
−2
−1
0
MR leave−one−out sensitivity analysis for
'genus.LachnospiraceaeUCG001.id.11321' on 'Mature T/NK−cell lymphomas || id:finn−b−CD2_TNK_LYMPHOMA'

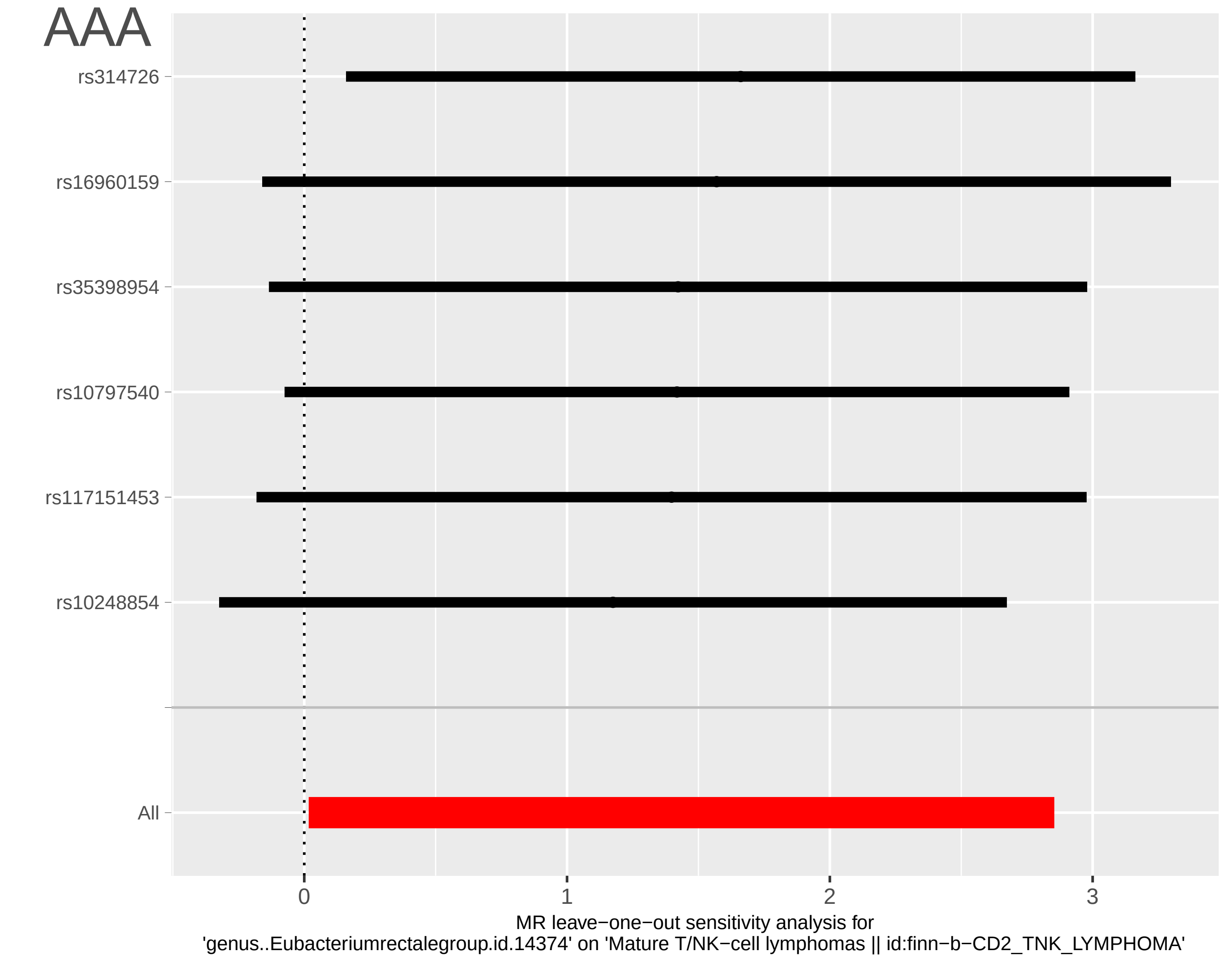
AAA
rs314726
rs16960159
rs35398954
rs10797540
rs117151453
rs10248854
All
0
1
2
3
MR leave−one−out sensitivity analysis for
'genus..Eubacteriumrectalegroup.id.14374' on 'Mature T/NK−cell lymphomas || id:finn−b−CD2_TNK_LYMPHOMA'

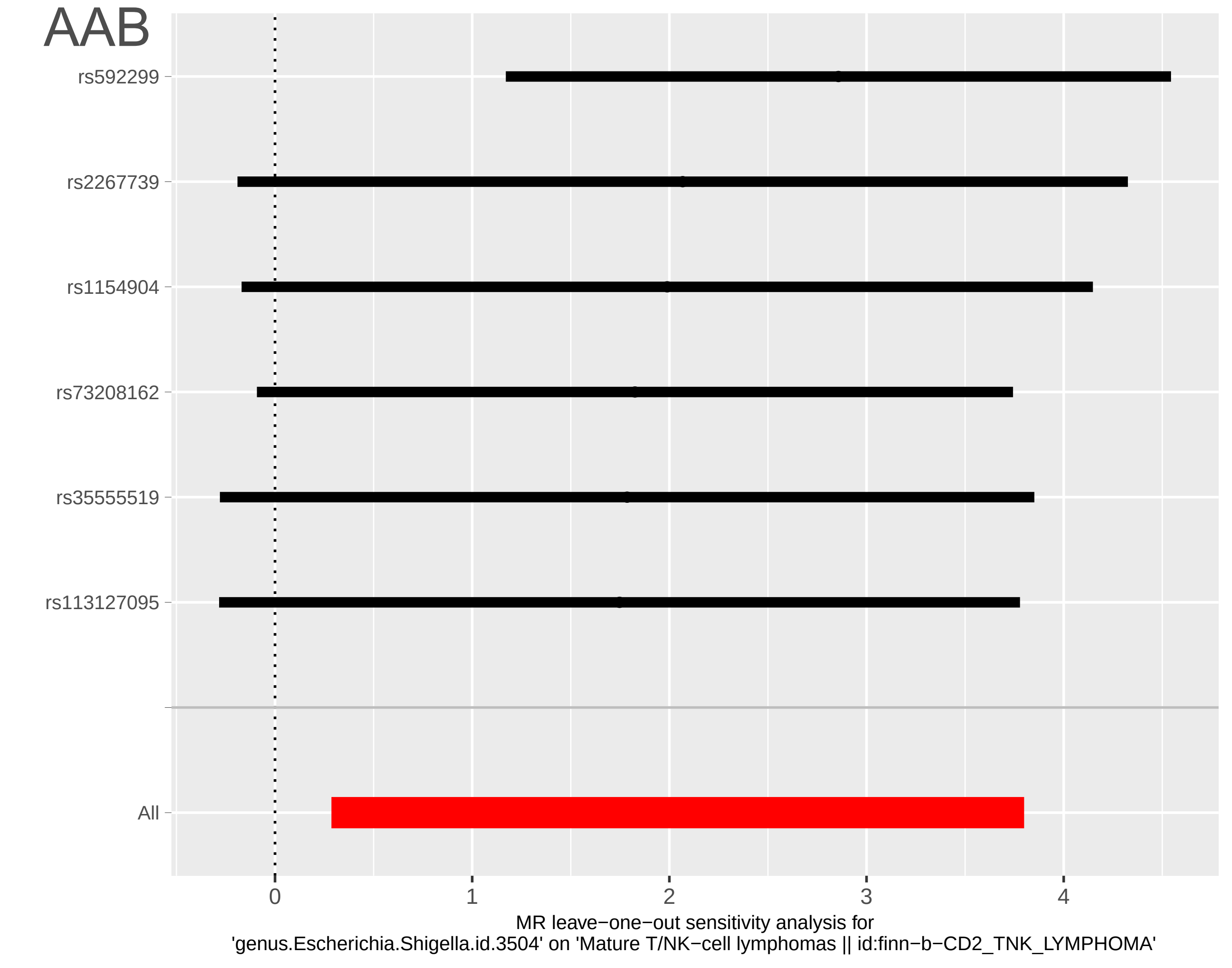
AAB
rs592299
rs2267739
rs1154904
rs73208162
rs35555519
rs113127095
All
0
1
2
3
4
MR leave−one−out sensitivity analysis for
'genus.Escherichia.Shigella.id.3504' on 'Mature T/NK−cell lymphomas || id:finn−b−CD2_TNK_LYMPHOMA'

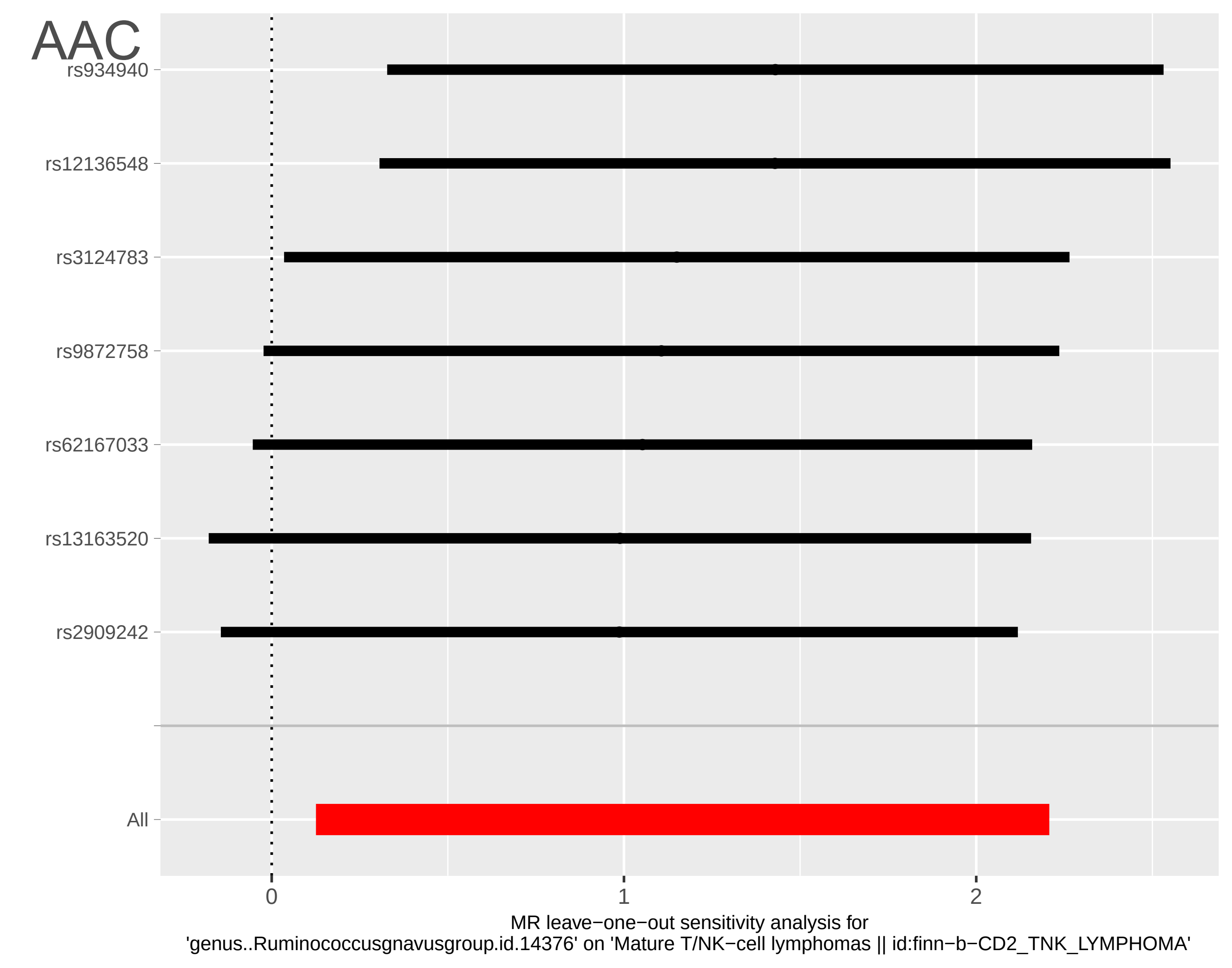

AAC
rs934940
rs12136548
rs3124783
rs9872758
rs62167033
rs13163520
rs2909242
All
0
1
2
MR leave−one−out sensitivity analysis for
'genus..Ruminococcusgnavusgroup.id.14376' on 'Mature T/NK−cell lymphomas || id:finn−b−CD2_TNK_LYMPHOMA'

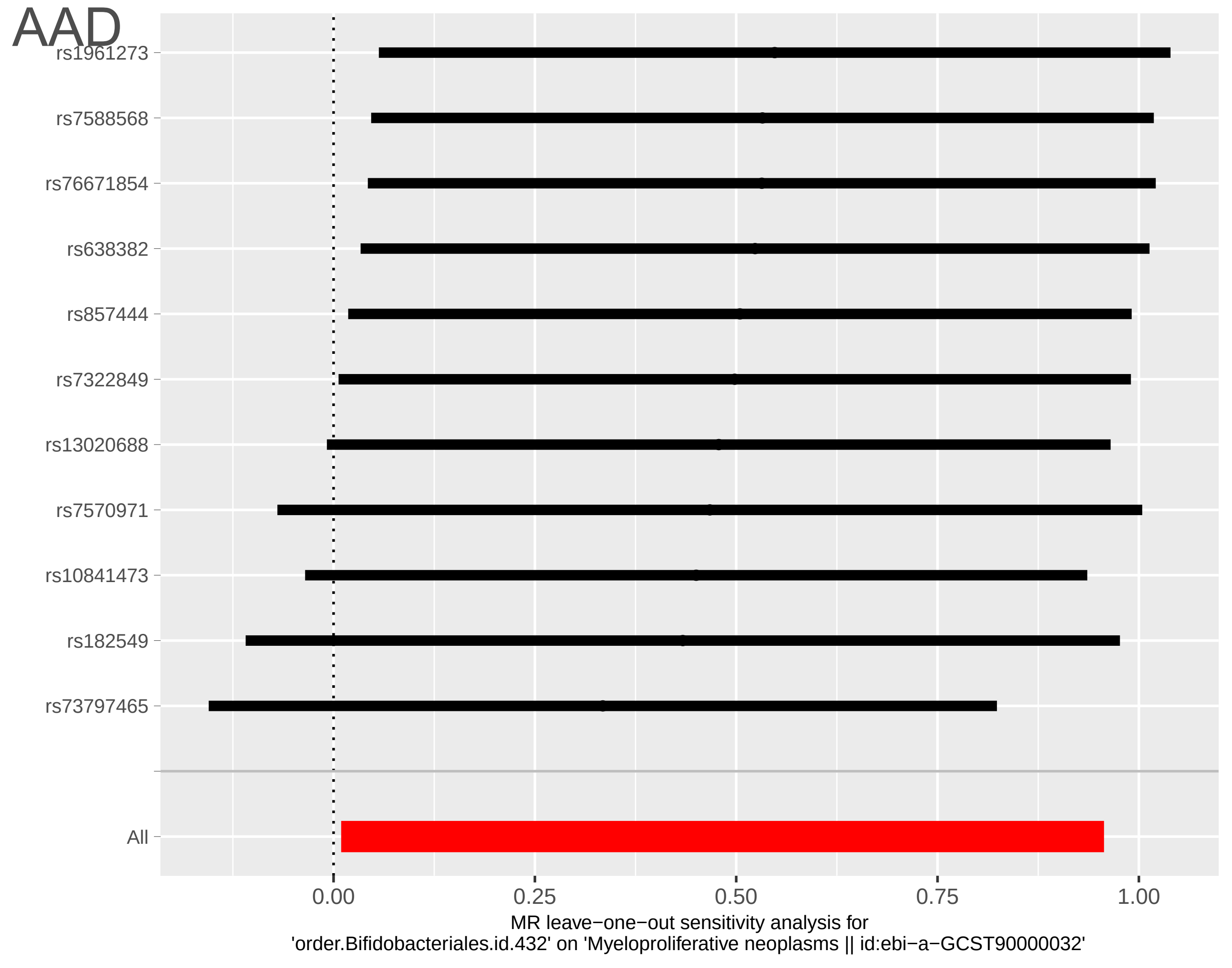
AAD
rs1961273
rs7588568
rs76671854
rs638382
rs857444
rs7322849
rs13020688
rs7570971
rs10841473
rs182549
rs73797465
All
0.00
0.25
0.50
0.75
1.00
MR leave−one−out sensitivity analysis for
'order.Bifidobacteriales.id.432' on 'Myeloproliferative neoplasms || id:ebi−a−GCST90000032'

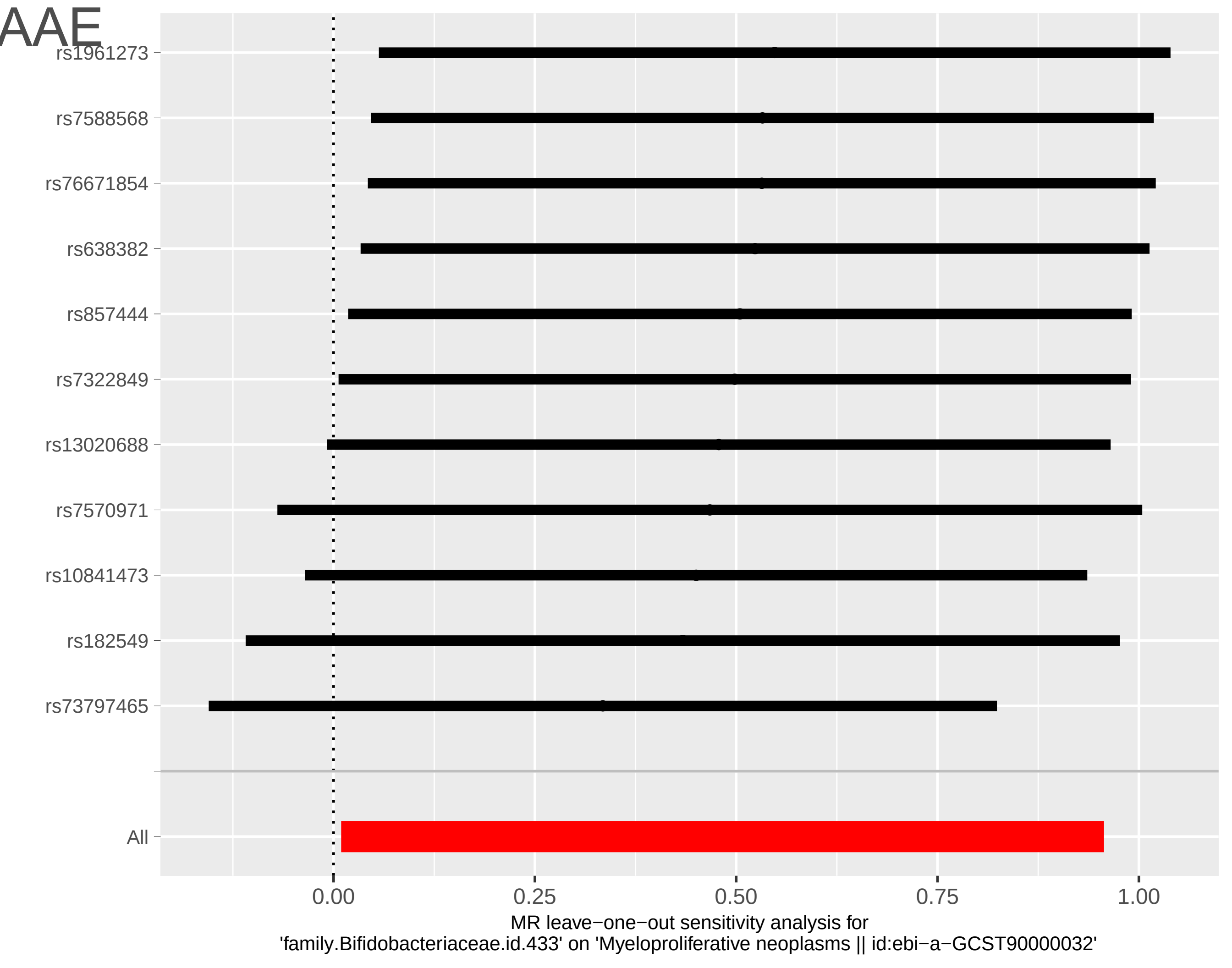
AAE
rs1961273
rs7588568
rs76671854
rs638382
rs857444
rs7322849
rs13020688
rs7570971
rs10841473
rs182549
rs73797465
All
0.00
0.25
0.50
0.75
1.00
MR leave−one−out sensitivity analysis for
'family.Bifidobacteriaceae.id.433' on 'Myeloproliferative neoplasms || id:ebi−a−GCST90000032'

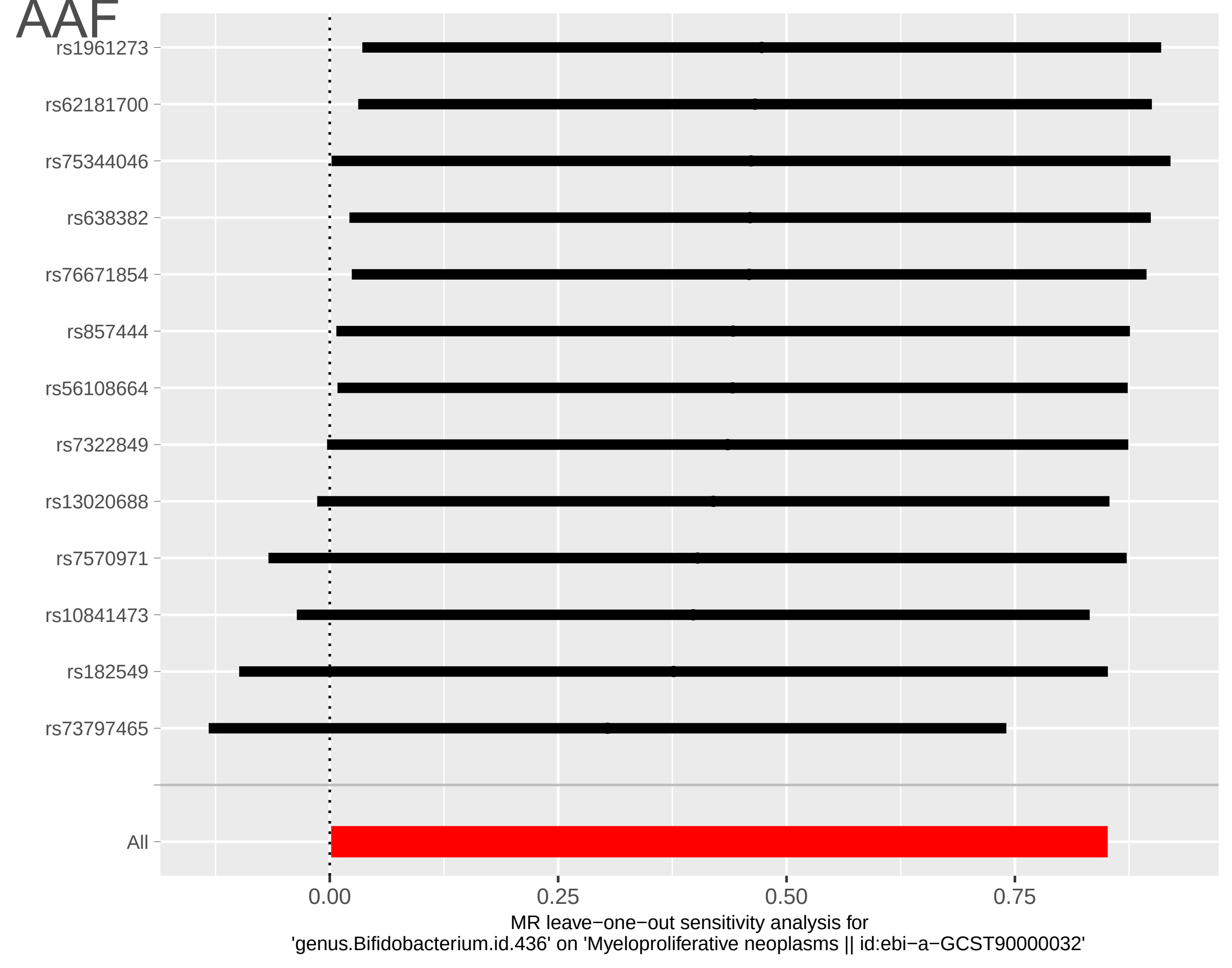
AAF
rs1961273
rs62181700
rs75344046
rs638382
rs76671854
rs857444
rs56108664
rs7322849
rs13020688
rs7570971
rs10841473
rs182549
rs73797465
All
0.00
0.25
0.50
0.75
MR leave−one−out sensitivity analysis for
'genus.Bifidobacterium.id.436' on 'Myeloproliferative neoplasms || id:ebi−a−GCST90000032'

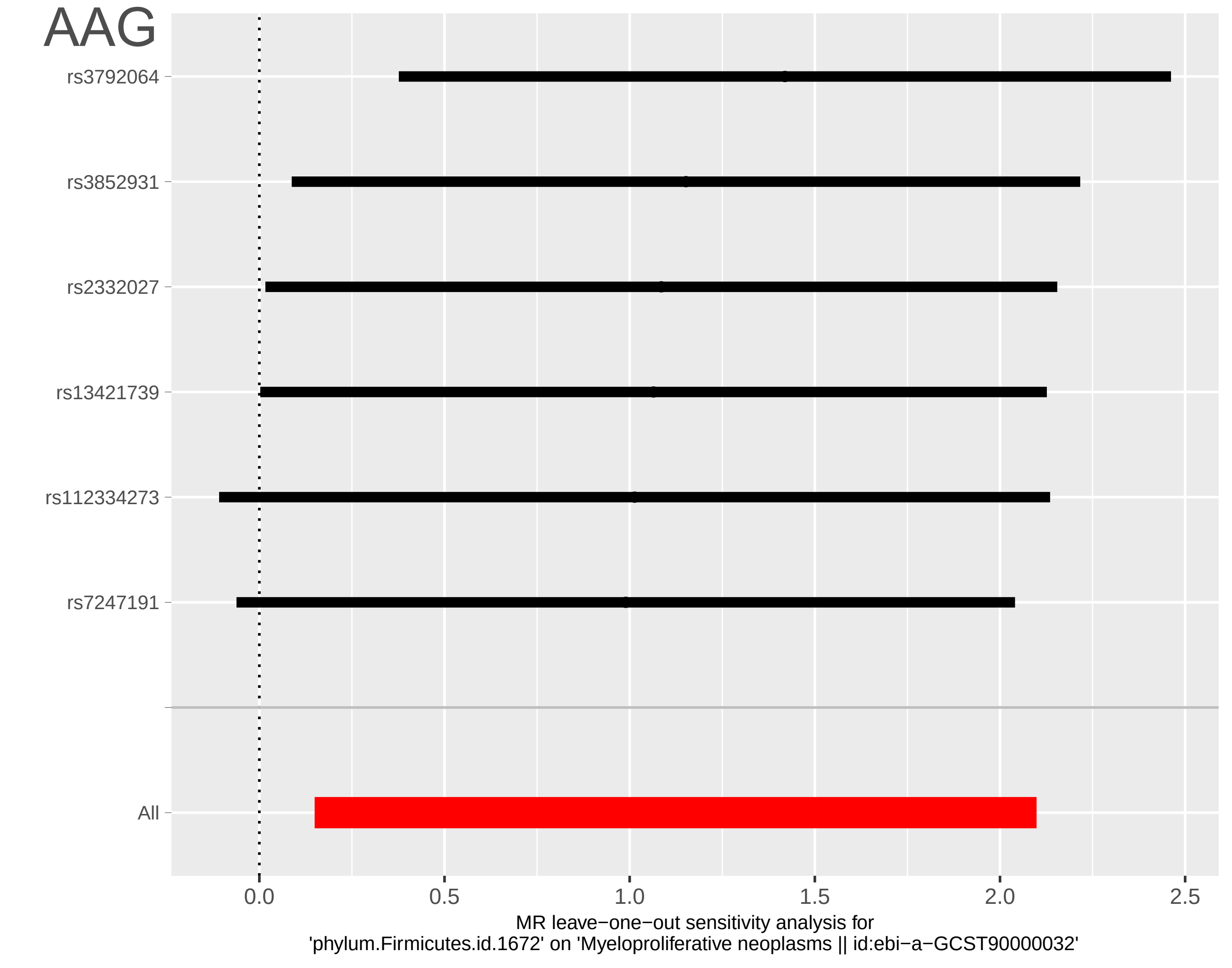
AAG
rs3792064
rs3852931
rs2332027
rs13421739
rs112334273
rs7247191
All
0.0
0.5
1.0
1.5
2.0
2.5
MR leave−one−out sensitivity analysis for
'phylum.Firmicutes.id.1672' on 'Myeloproliferative neoplasms || id:ebi−a−GCST90000032'

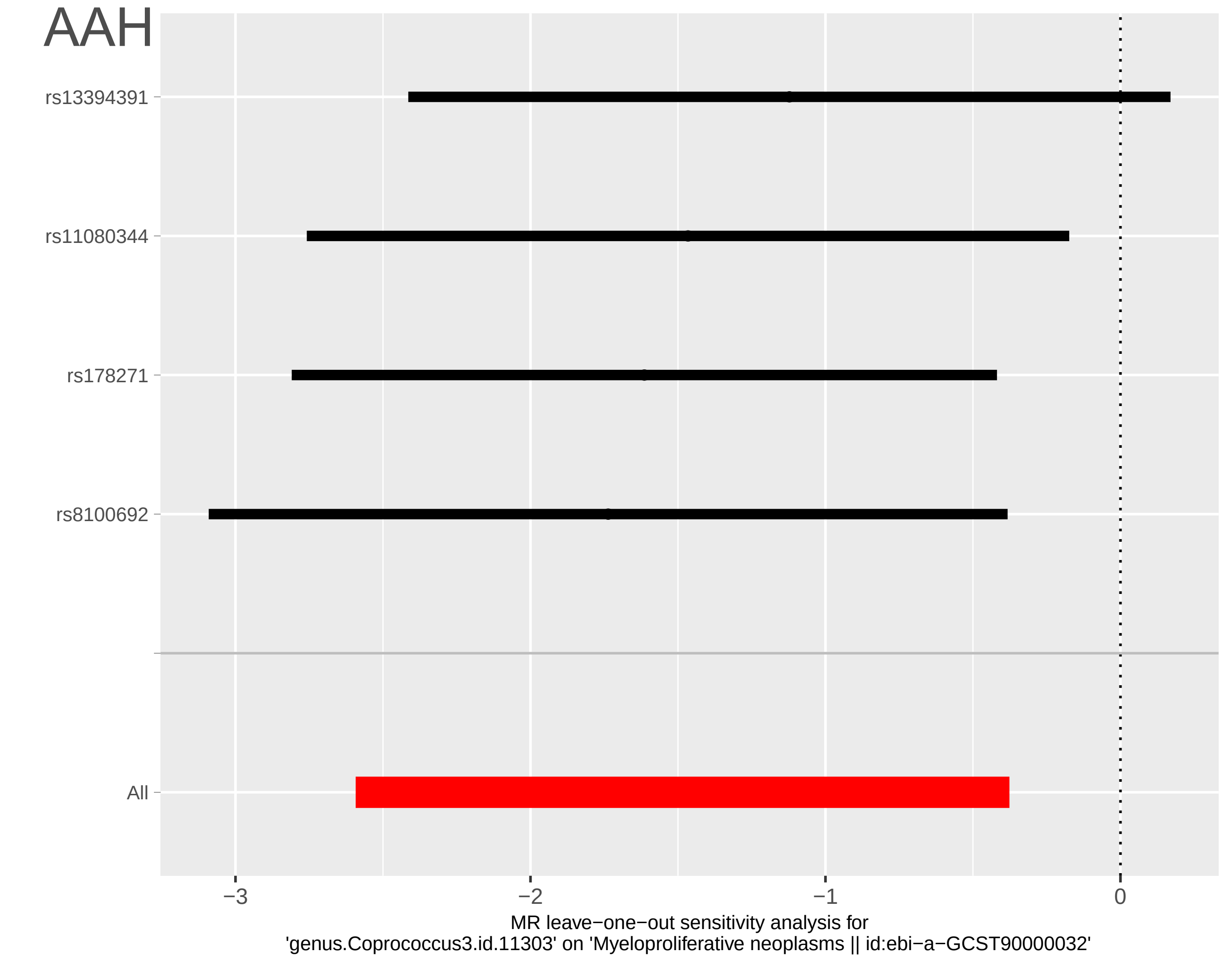
AAH
rs13394391
rs11080344
rs178271
rs8100692
All
−3
−2
−1
0
MR leave−one−out sensitivity analysis for
'genus.Coprococcus3.id.11303' on 'Myeloproliferative neoplasms || id:ebi−a−GCST90000032'

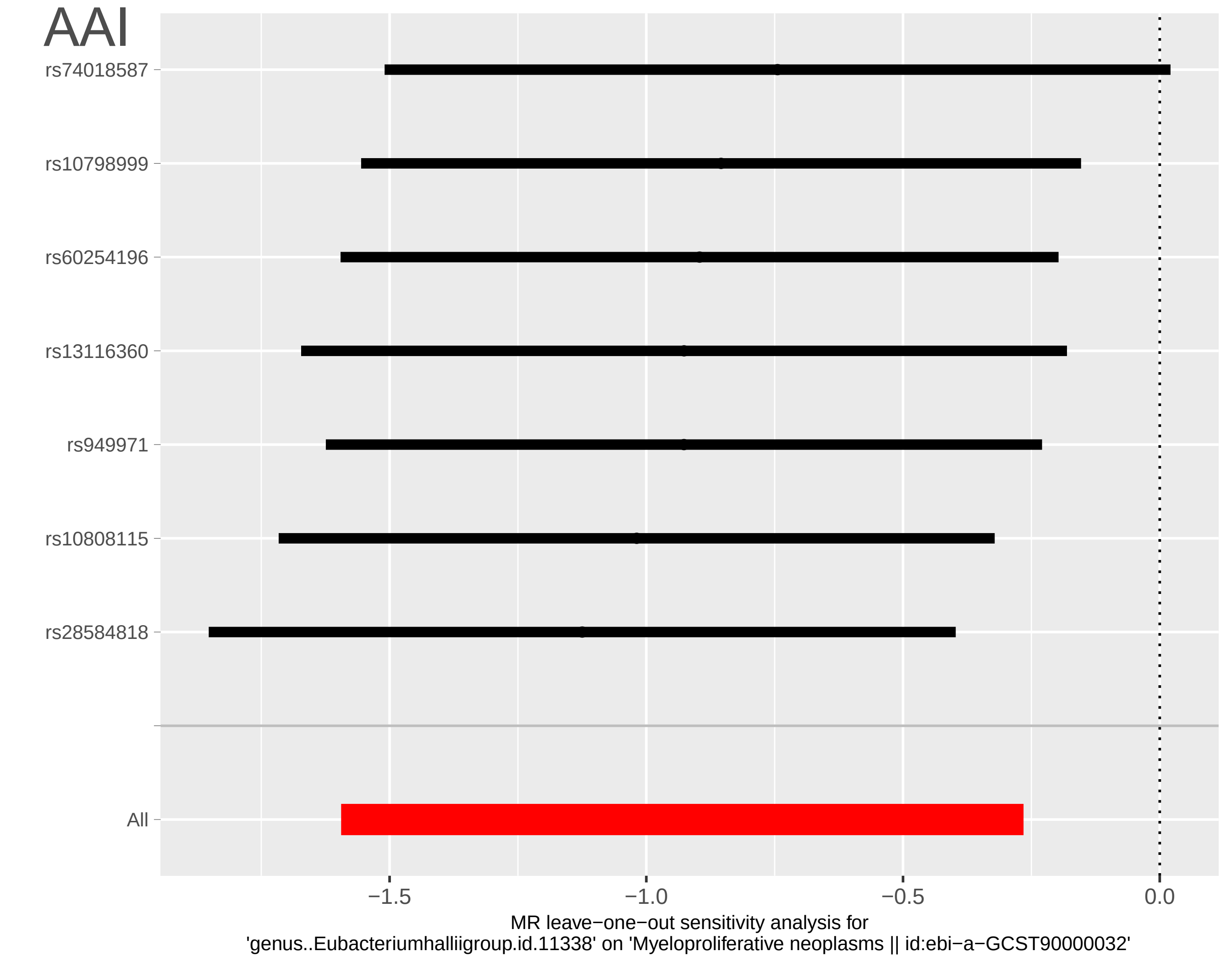
AAI
rs74018587
rs10798999
rs60254196
rs13116360
rs949971
rs10808115
rs28584818
All
−1.5
−1.0
−0.5
0.0
MR leave−one−out sensitivity analysis for
'genus..Eubacteriumhalliigroup.id.11338' on 'Myeloproliferative neoplasms || id:ebi−a−GCST90000032'

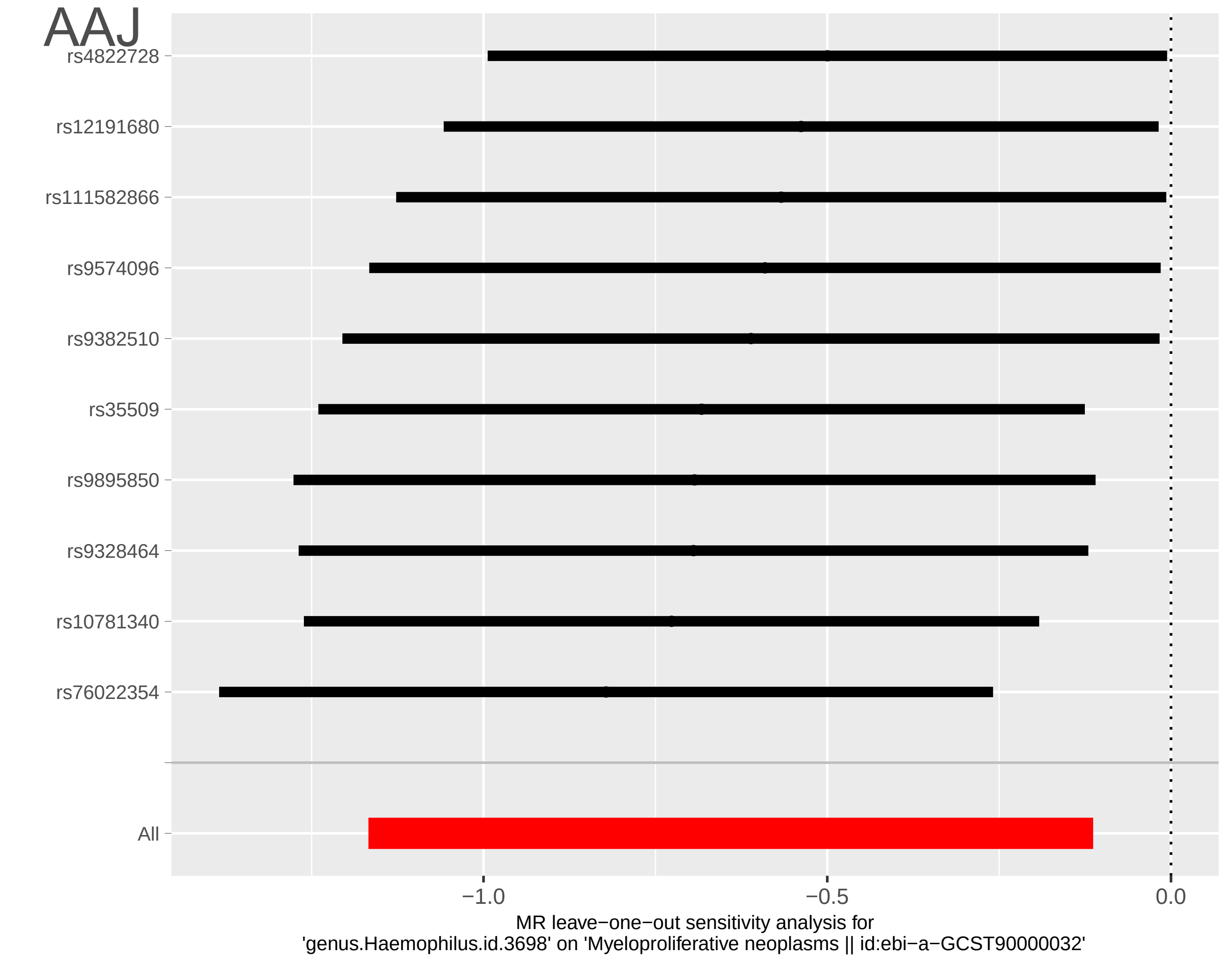
AAJ
rs4822728
rs12191680
rs111582866
rs9574096
rs9382510
rs35509
rs9895850
rs9328464
rs10781340
rs76022354
All
−1.0
−0.5
0.0
MR leave−one−out sensitivity analysis for
'genus.Haemophilus.id.3698' on 'Myeloproliferative neoplasms || id:ebi−a−GCST90000032'

Supplementary figure 3.Sensitivity analysis of the impact of gut microbiota on hematologic malignancies using MR. (leaveoneout plot)

A.Leaveoneout plot between genus.FamilyXIIIUCG001 and lymphoid leukemia

B.Leaveoneout plot between family.Peptococcaceae and lymphoid leukemia**.**

C.Leaveoneout plot between family.Desulfovibrionaceae and lymphoid leukemia

D.Leaveoneout plot between genus..Clostridiuminnocuumgroup and lymphoid leukemia.

E.Leaveoneout plot between genus.Holdemania and lymphoid leukemia.

F.Leaveoneout plot between family.Prevotellaceae and lymphoid leukemia.

G.Leaveoneout plot between genus.Ruminiclostridium9. and lymphoid leukemia.

H.Leaveoneout plot between genus.Methanobrevibacter and lymphoid leukemia.

I.Leaveoneout plot between order.Desulfovibrionales. and lymphoid leukemia.

J.Leaveoneout plot between genus.Coprococcus3 and lymphoid leukemia.

K.Leaveoneout plot between genus.Ruminiclostridium6 and lymphoid leukemia.

L.Leaveoneout plot between class.Negativicutes and lymphoid leukemia.

M.Leaveoneout plot between order.Selenomonadales. and lymphoid leukemia.

N.Leaveoneout plot between family.Lactobacillaceae. and lymphoid leukemia.

O.Leaveoneout plot between class.Methanobacteria and lymphoid leukemia.

P.Leaveoneout plot between family.Methanobacteriaceae and lymphoid leukemia.

Q.Leaveoneout plot between order.Methanobacteriales and lymphoid leukemia.

R.Leaveoneout plot between phylum.Cyanobacteria and lymphoid leukemia**.**

S.Leaveoneout plot between order.Coriobacteriales and myeloid leukemia**.**

T.Leaveoneout plot between family.Coriobacteriaceae. and myeloid leukemia**.**

U.Leaveoneout plot between genus.LachnospiraceaeUCG008 and myeloid leukemia.

V.Leaveoneout plot between class.Coriobacteriia. and myeloid leukemia**.**

W.Leaveoneout plot between genus.Turicibacter and myeloid leukemia**.**

X.Leaveoneout plot between genus.Slackia. and myeloid leukemia.

Y.Leaveoneout plot between class.Gammaproteobacteria and myeloid leukemia.

Z.Leaveoneout plot between genus.Prevotella9 and myeloid leukemia.

AA. Leaveoneout plot between genus.Dorea and myeloid leukemia.

AB.Leaveoneout plot between genus.Peptococcus. and Hodgkin lymphoma

AC.Leaveoneout plot between class.Gammaproteobacteria and Hodgkin lymphoma.

AD.Leaveoneout plot between genus.RuminococcaceaeUCG014 and malignant plasma cell tumor**.**

AE.Leaveoneout plot between genus.LachnospiraceaeUCG010. and malignant plasma cell tumor.

AF.Leaveoneout plot between class.Lentisphaeria and malignant plasma cell tumor.

AG.Leaveoneout plot between genus.Lactococcus. and malignant plasma cell tumor.

AH.Leaveoneout plot between order.Victivallales. and malignant plasma cell tumor.

AI.Leaveoneout plot between genus.Romboutsia and malignant plasma cell tumor.

AJ.Leaveoneout plot between genus.Dorea and malignant plasma cell tumor.

AK.Leaveoneout plot between genus.RikenellaceaeRC9gutgroup and malignant plasma cell tumor**.**

AL. Leaveoneout plot between genus.RuminococcaceaeUCG005 and follicular lymphoma.

AM.Leaveoneout plot between genus.Adlercreutzia and follicular lymphoma.

AN.Leaveoneout plot between order.MollicutesRF9 and follicular lymphoma.

AO.Leaveoneout plot between family.Alcaligenaceae and follicular lymphoma.

AP.Leaveoneout plot between class.Clostridia and follicular lymphoma.

AQ. Leaveoneout plot between genus.Sutterella and follicular lymphoma.

AR.Leaveoneout plot between genus.Phascolarctobacterium and follicular lymphoma.

AS.Leaveoneout plot between genus..Eubacteriumcoprostanoligenesgroup and diffuse large B-cell lymphoma.

AT.Leaveoneout plot between class.Alphaproteobacteria. and diffuse large B-cell lymphoma.

AU.Leaveoneout plot between phylum.Cyanobacter and diffuse large B-cell lymphoma.

AV.Leaveoneout plot between genus.Erysipelatoclostridium and diffuse large B-cell lymphoma.

AW.Leaveoneout plot between family.Rhodospirillaceae. and mature T/NK-cell lymphomas.

AX.Leaveoneout plot between genus.Anaerostipes and mature T/NK-cell lymphomas.

AY.Leaveoneout plot between genus.Erysipelatoclostridium. and mature T/NK-cell lymphomas.

AZ.Leaveoneout plot between genus.LachnospiraceaeUCG001.. and mature T/NK-cell lymphomas.

AAA.Leaveoneout plot between genus..Eubacteriumrectalegroup. and mature T/NK-cell lymphomas.

AAB. Leaveoneout plot between genus.Escherichia.Shigella. and mature T/NK-cell lymphomas.

AAC.Leaveoneout plot between genus..Ruminococcusgnavusgroup and mature T/NK-cell lymphomas.

AAD. Leaveoneout plot between order.Bifidobacteriales. and myeloproliferative neoplasms.

AAE. Leaveoneout plot between family.Bifidobacteriaceae.. and myeloproliferative neoplasms.

AAF. Leaveoneout plot between genus.Bifidobacterium. and myeloproliferative neoplasms.

AAG.Leaveoneout plot between phylum.Firmicutes. and myeloproliferative neoplasms.

AAH.Leaveoneout plot between genus.Coprococcus3. and myeloproliferative neoplasms.

AAI.Leaveoneout plot between genus..Eubacteriumhalliigroup. and myeloproliferative neoplasms.

AAJ.Leaveoneout plot between genus.Haemophilus. and myeloproliferative neoplasms

A

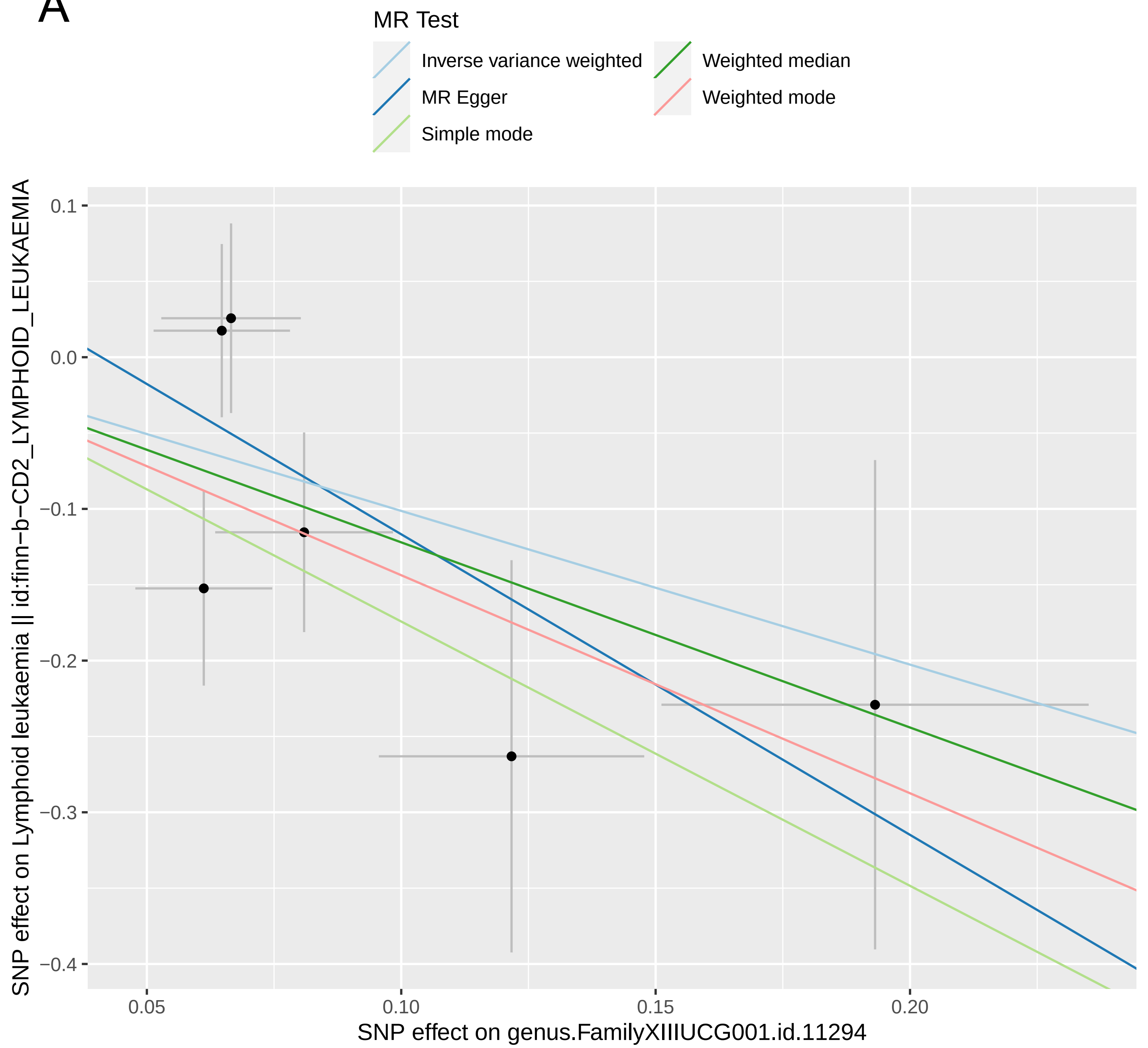

MR Test
Inverse variance weighted
MR Egger
Simple mode
Weighted median
Weighted mode
0.1
0.0
−0.1
−0.2
−0.3
−0.4
SNP effect on Lymphoid leukaemia || id:finn−b−CD2_LYMPHOID_LEUKAEMIA
0.05
0.10
0.15
0.20
SNP effect on genus.FamilyXIIIUCG001.id.11294

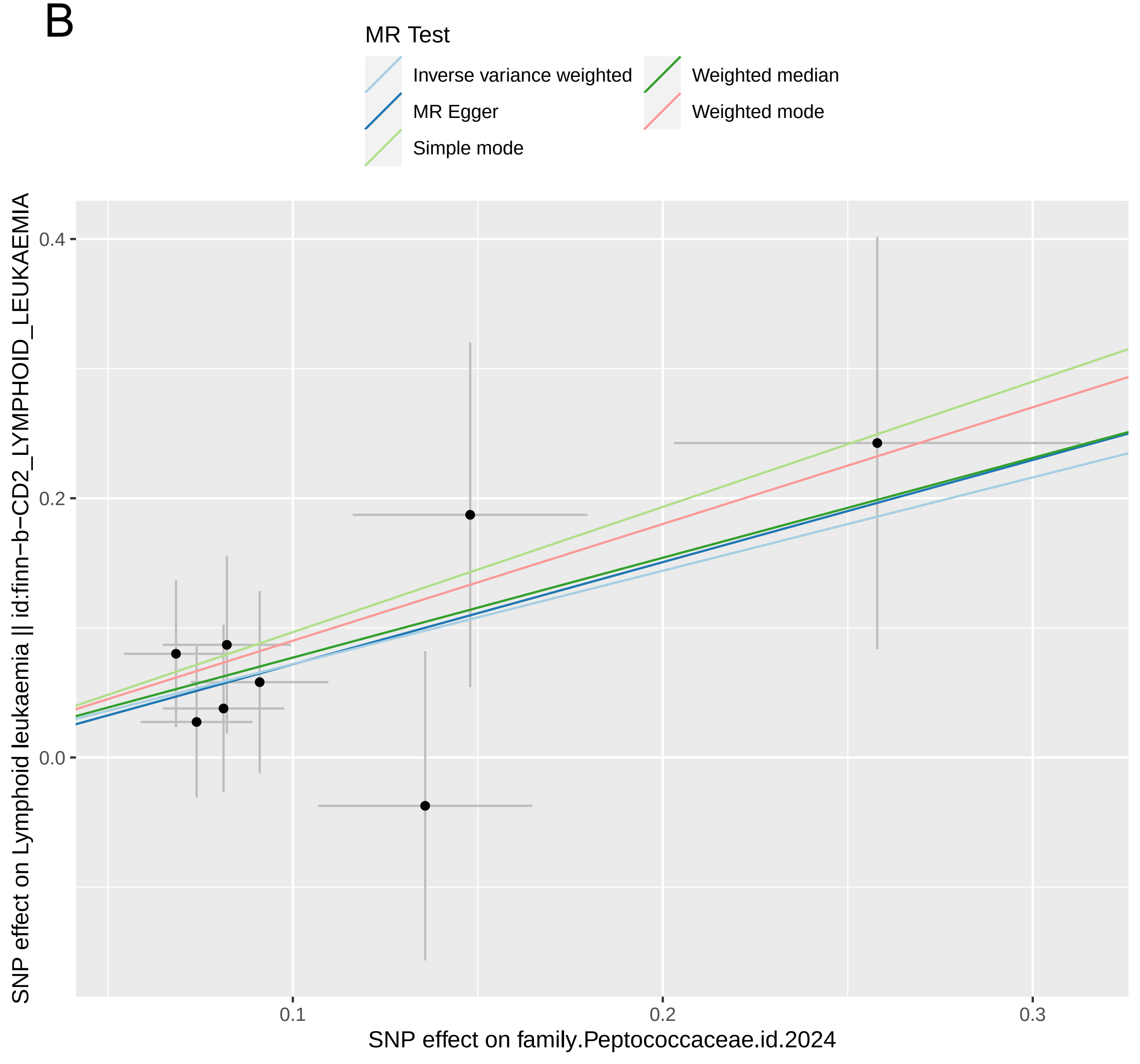

B
MR Test
Inverse variance weighted
MR Egger
Simple mode
Weighted median
Weighted mode
SNP effect on Lymphoid leukaemia || id:finn-b-CD2_LYMPHOID_LEUKAEMIA
0.4
0.2
0.0
0.1
0.2
0.3
SNP effect on family.Peptococcaceae.id.2024

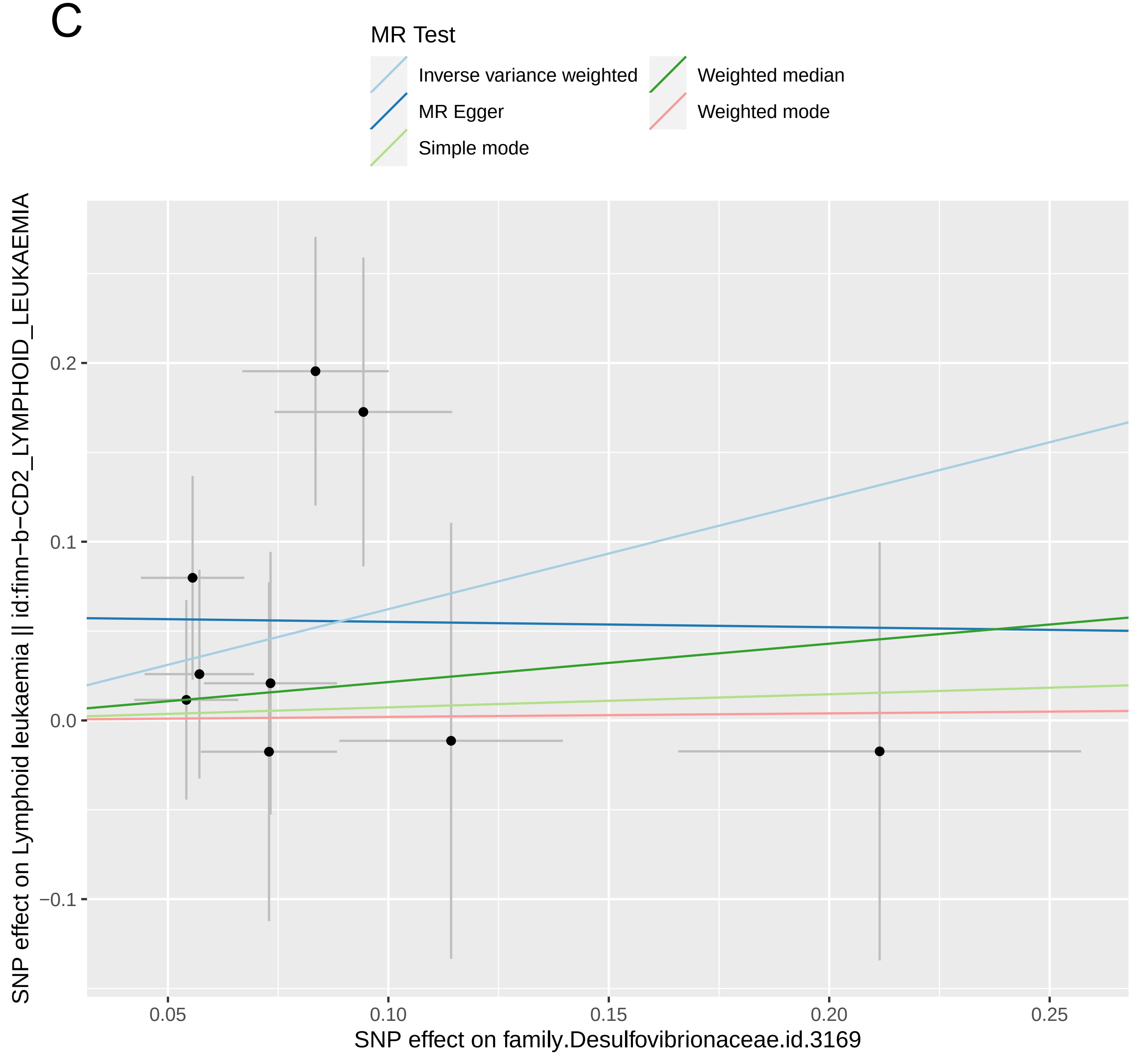

C
MR Test
Inverse variance weighted
MR Egger
Simple mode
Weighted median
Weighted mode
SNP effect on Lymphoid leukaemia || id:finn−b−CD2_LYMPHOID_LEUKAEMIA
0.2
0.1
0.0
−0.1
0.05
0.10
0.15
0.20
0.25
SNP effect on family.Desulfovibrionaceae.id.3169

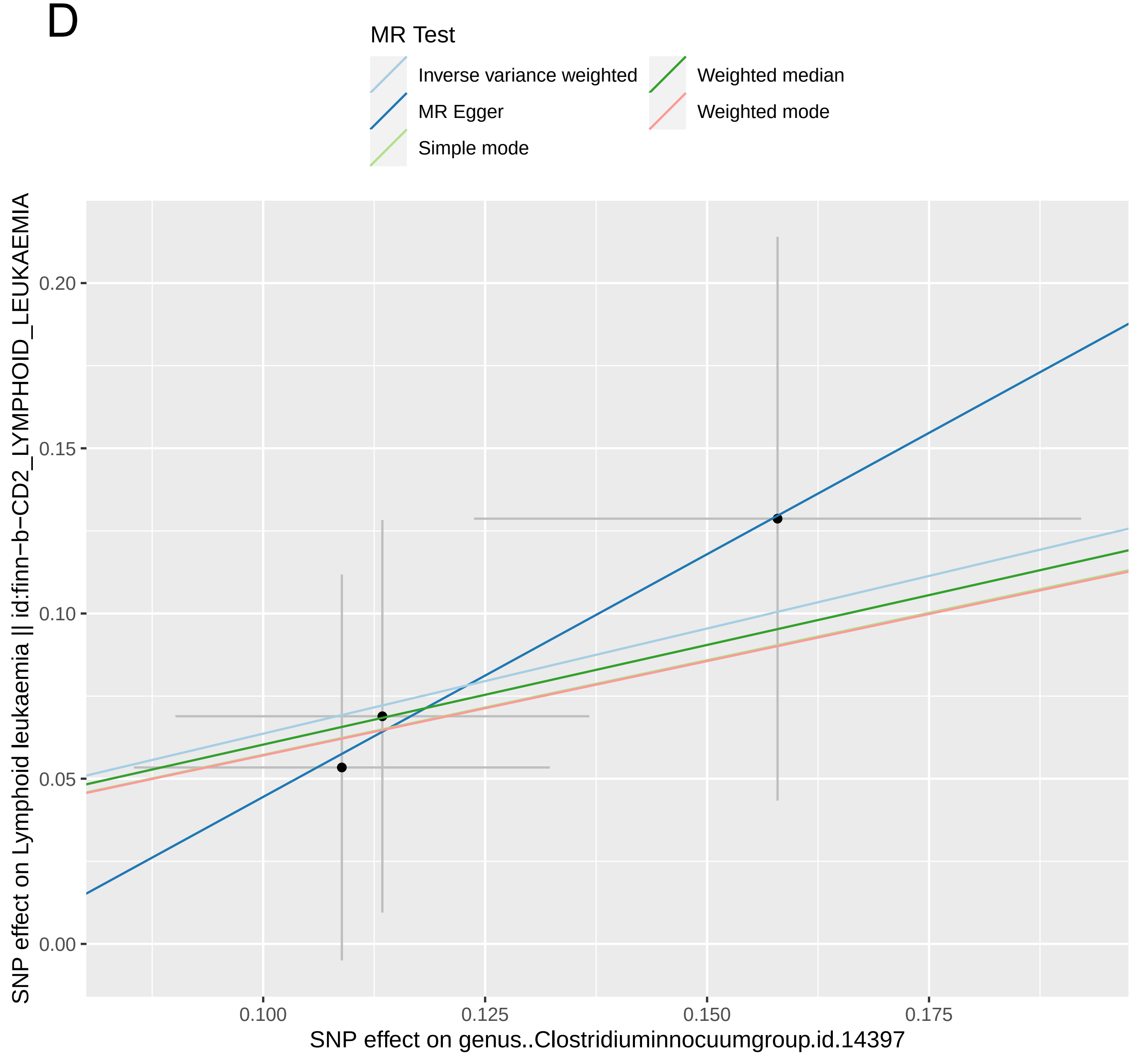
D
MR Test
Inverse variance weighted
MR Egger
Simple mode
Weighted median
Weighted mode
SNP effect on Lymphoid leukaemia || id:finn-b-CD2_LYMPHOID_LEUKAEMIA
0.00
0.05
0.10
0.15
0.20
0.100
0.125
0.150
0.175
SNP effect on genus..Clostridiuminnocuumgroup.id.14397

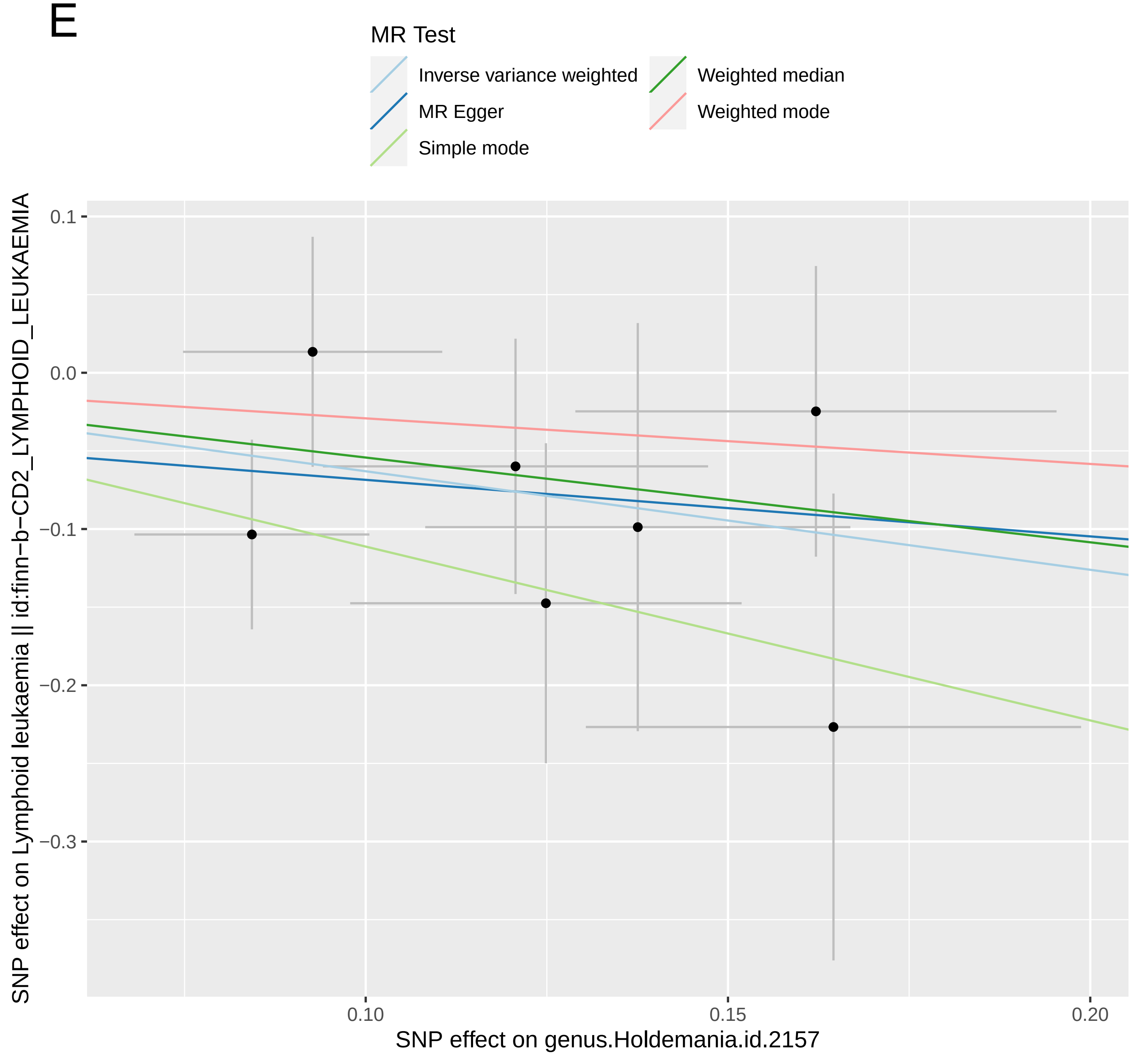
E
MR Test
Inverse variance weighted
MR Egger
Simple mode
Weighted median
Weighted mode
0.1
0.0
−0.1
−0.2
−0.3
SNP effect on Lymphoid leukaemia || id:finn−b−CD2_LYMPHOID_LEUKAEMIA
0.10
0.15
0.20
SNP effect on genus.Holdemania.id.2157

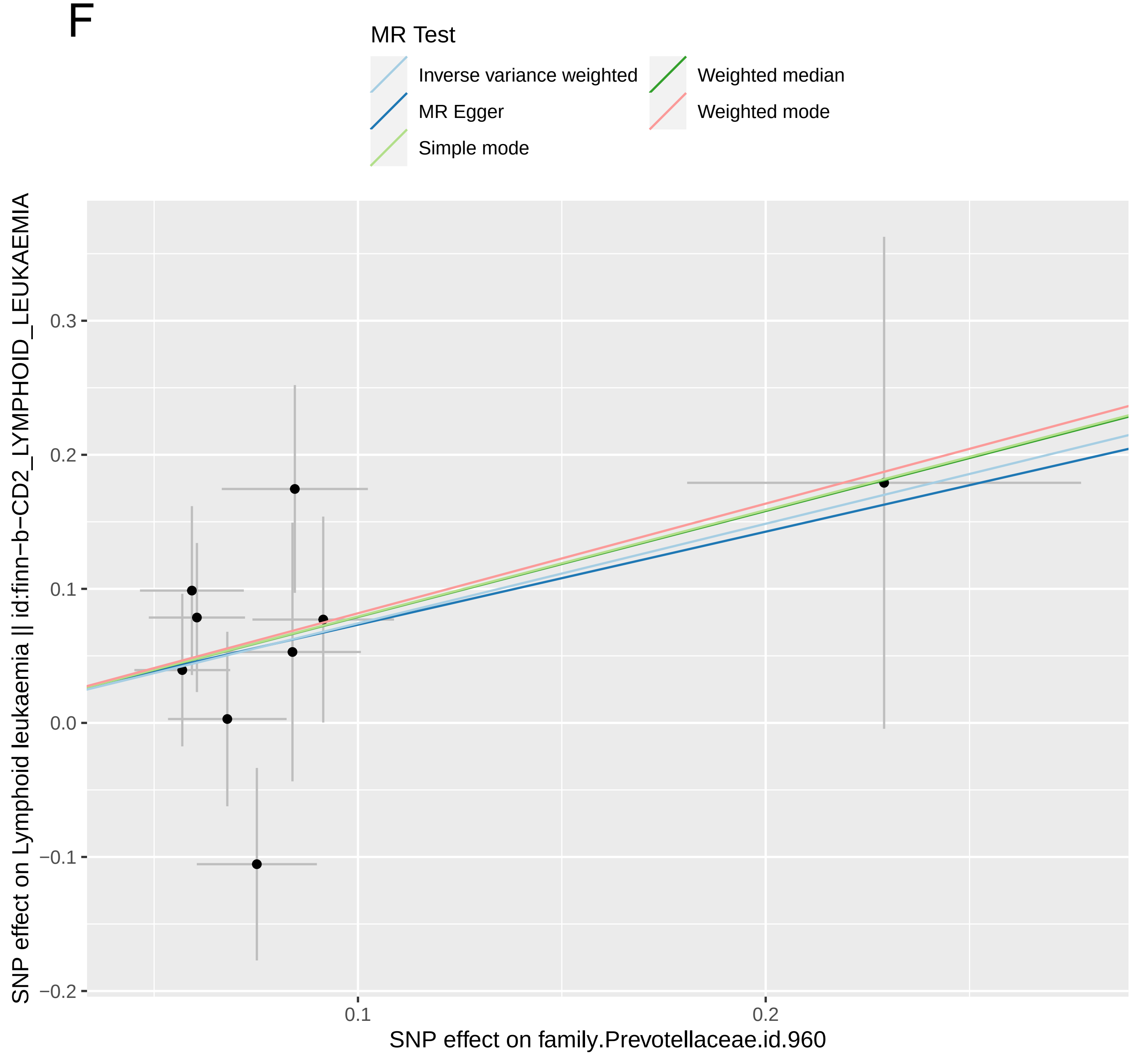

F
MR Test
Inverse variance weighted
MR Egger
Simple mode
Weighted median
Weighted mode
SNP effect on Lymphoid leukaemia || id:finn-b-CD2_LYMPHOID_LEUKAEMIA
0.3
0.2
0.1
0.0
−0.1
−0.2
0.1
0.2
SNP effect on family.Prevotellaceae.id.960

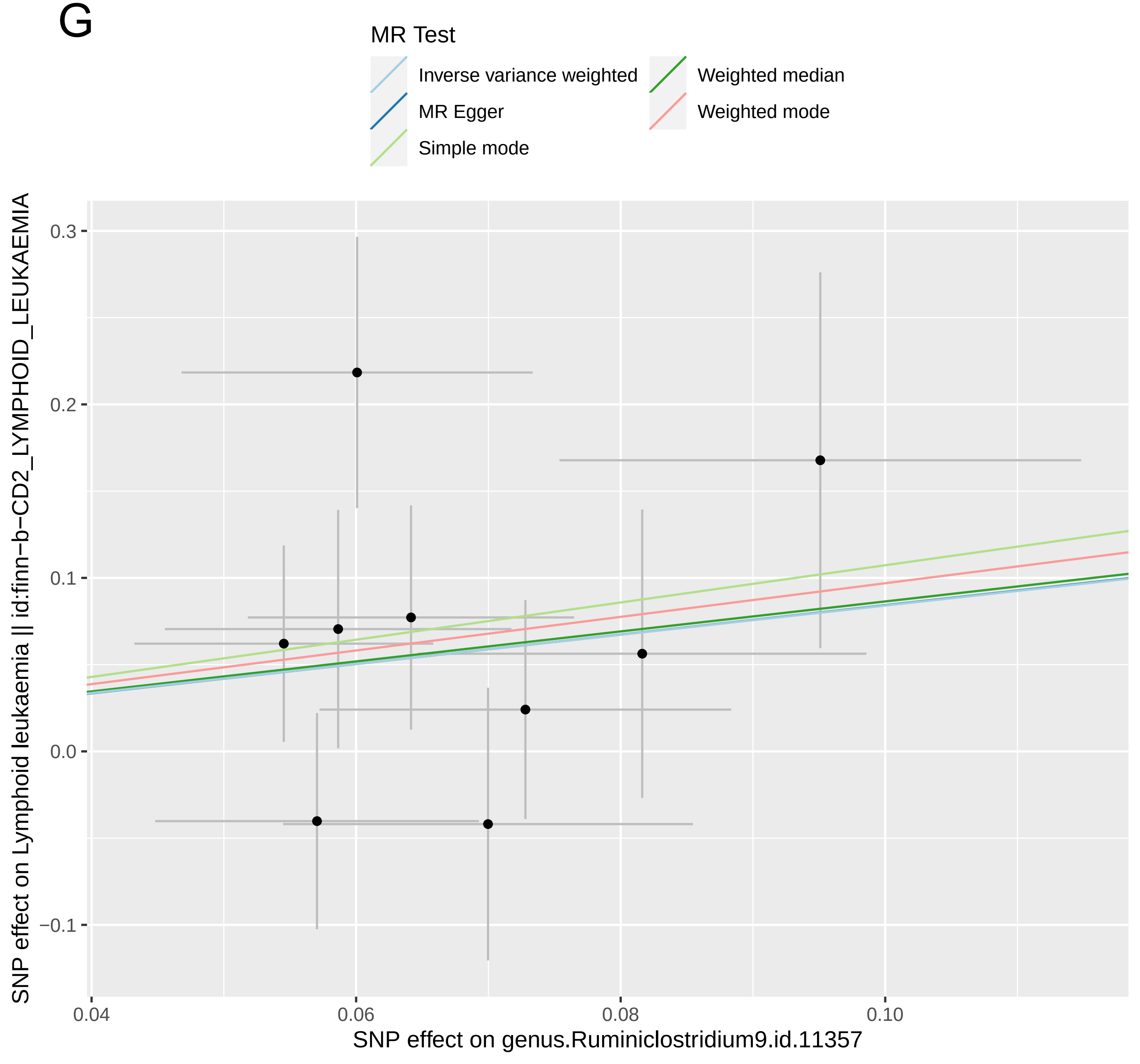
G
MR Test
Inverse variance weighted
MR Egger
Simple mode
Weighted median
Weighted mode
0.3
0.2
0.1
0.0
−0.1
0.04
0.06
0.08
0.10
SNP effect on Lymphoid leukaemia || id:finn−b−CD2_LYMPHOID_LEUKAEMIA
SNP effect on genus.Ruminiclostridium9.id.11357

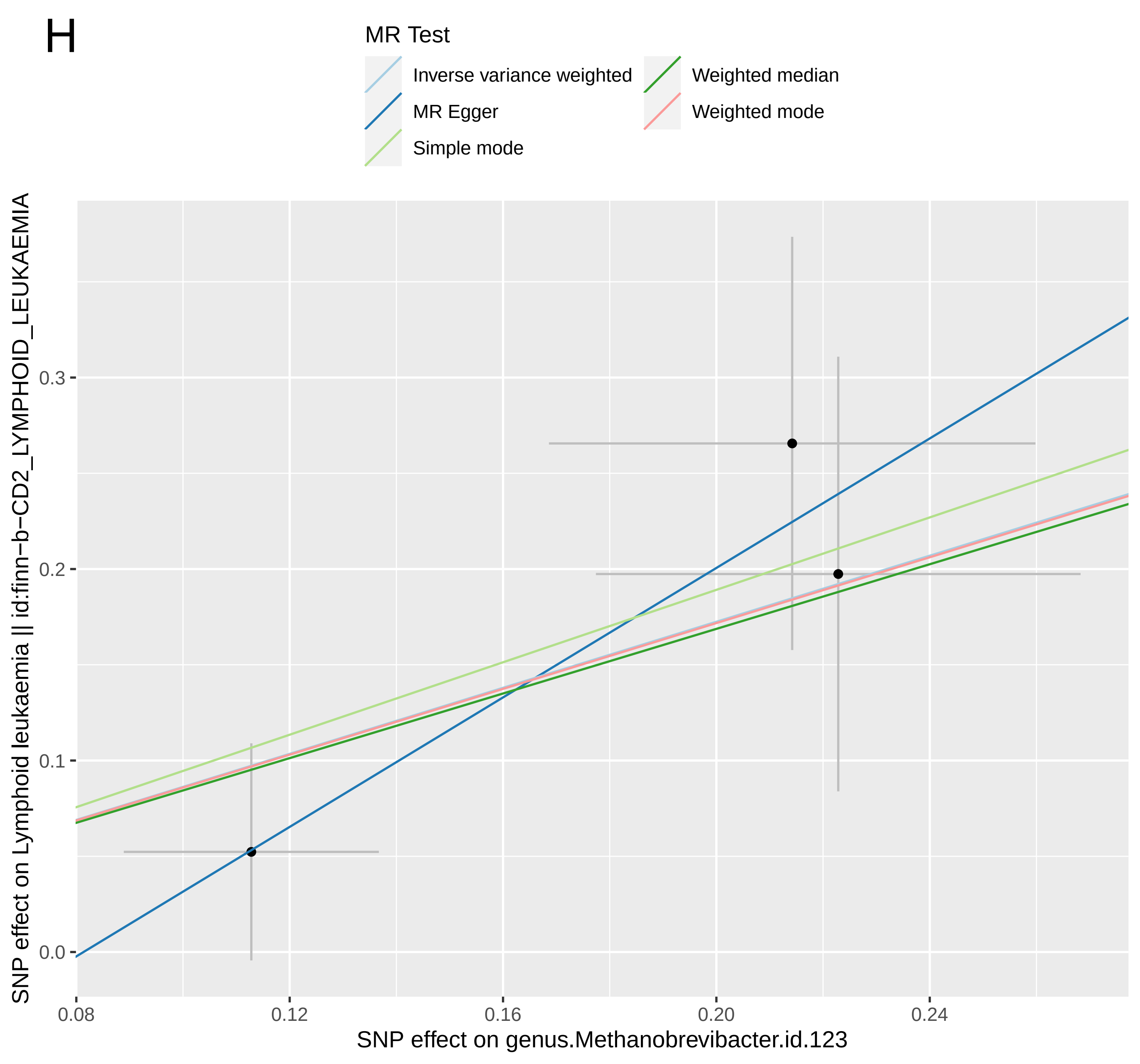
H
MR Test
Inverse variance weighted
MR Egger
Simple mode
Weighted median
Weighted mode
SNP effect on Lymphoid leukaemia || id:finn−b−CD2_LYMPHOID_LEUKAEMIA
0.0
0.1
0.2
0.3
0.08
0.12
0.16
0.20
0.24
SNP effect on genus.Methanobrevibacter.id.123

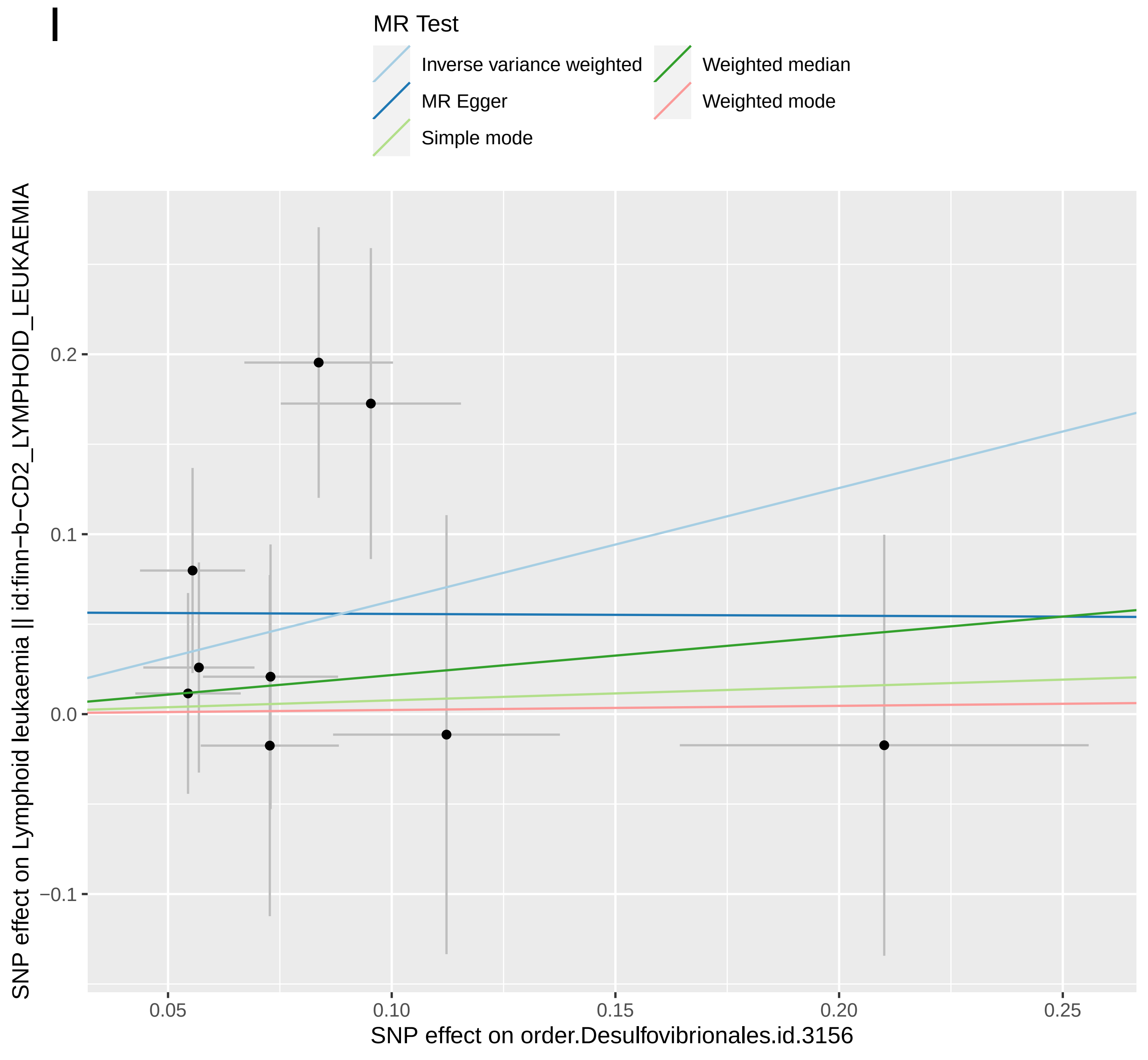
I
MR Test
Inverse variance weighted
MR Egger
Simple mode
Weighted median
Weighted mode
SNP effect on Lymphoid leukaemia || id:finn−b−CD2_LYMPHOID_LEUKAEMIA
0.2
0.1
0.0
−0.1
0.05
0.10
0.15
0.20
0.25
SNP effect on order.Desulfovibrionales.id.3156

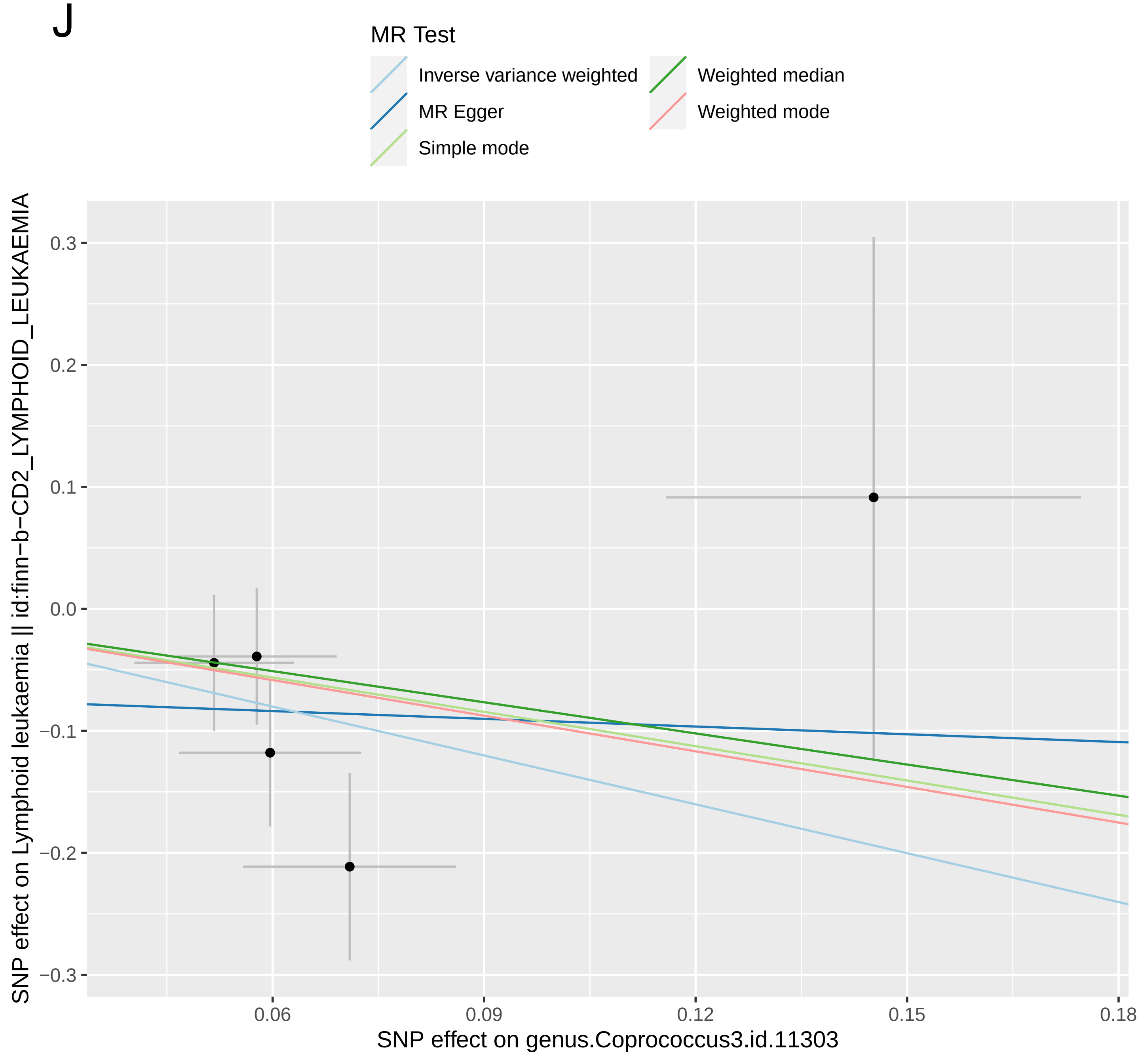
J
MR Test
Inverse variance weighted
MR Egger
Simple mode
Weighted median
Weighted mode
SNP effect on Lymphoid leukaemia || id:finn−b−CD2_LYMPHOID_LEUKAEMIA
0.3
0.2
0.1
0.0
−0.1
−0.2
−0.3
0.06
0.09
0.12
0.15
0.18
SNP effect on genus.Coprococcus3.id.11303

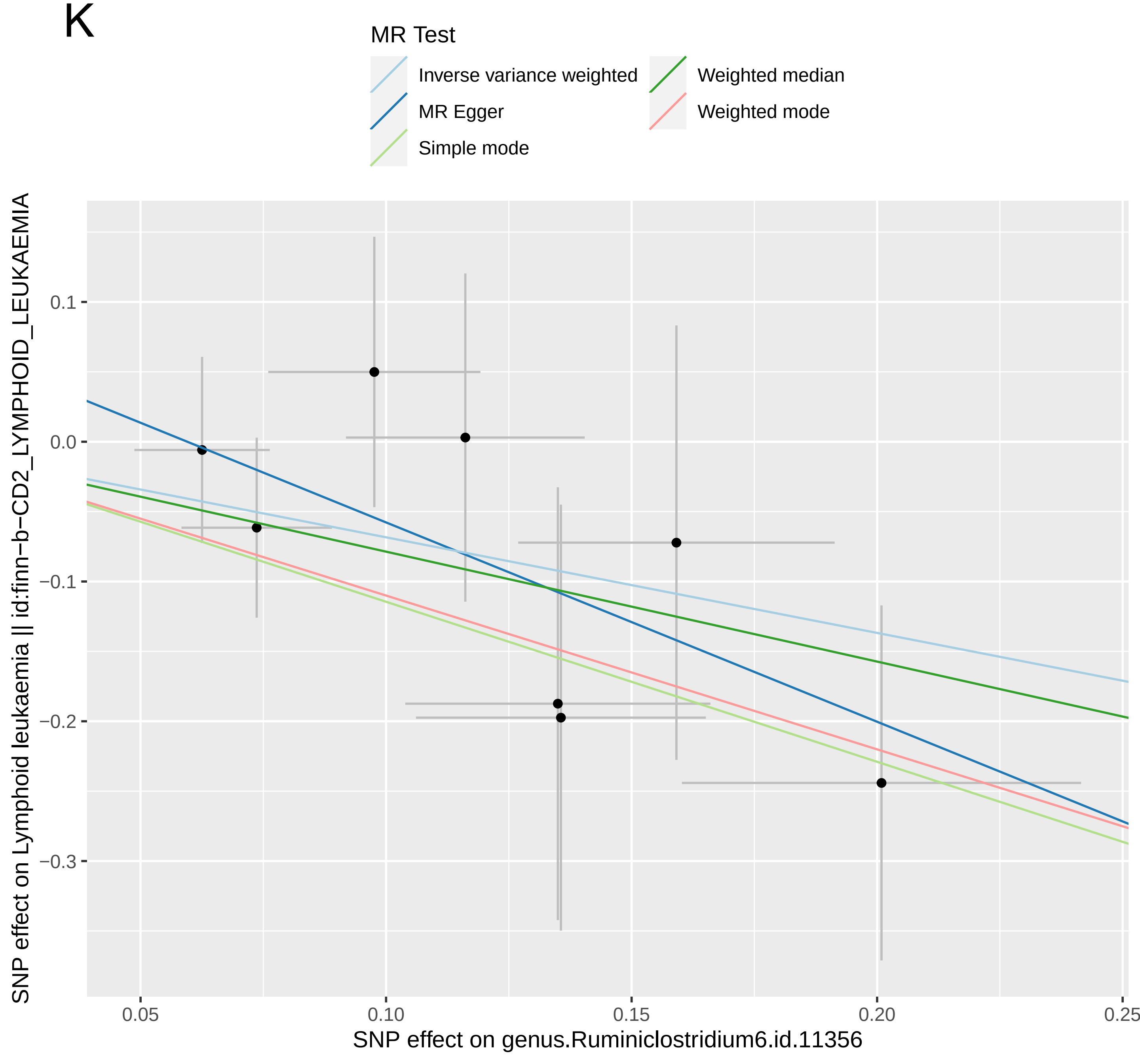

K
MR Test
Inverse variance weighted
MR Egger
Simple mode
Weighted median
Weighted mode
SNP effect on Lymphoid leukaemia || id:finn-b-CD2_LYMPHOID_LEUKAEMIA
0.1
0.0
−0.1
−0.2
−0.3
0.05
0.10
0.15
0.20
0.25
SNP effect on genus.Ruminiclostridium6.id.11356

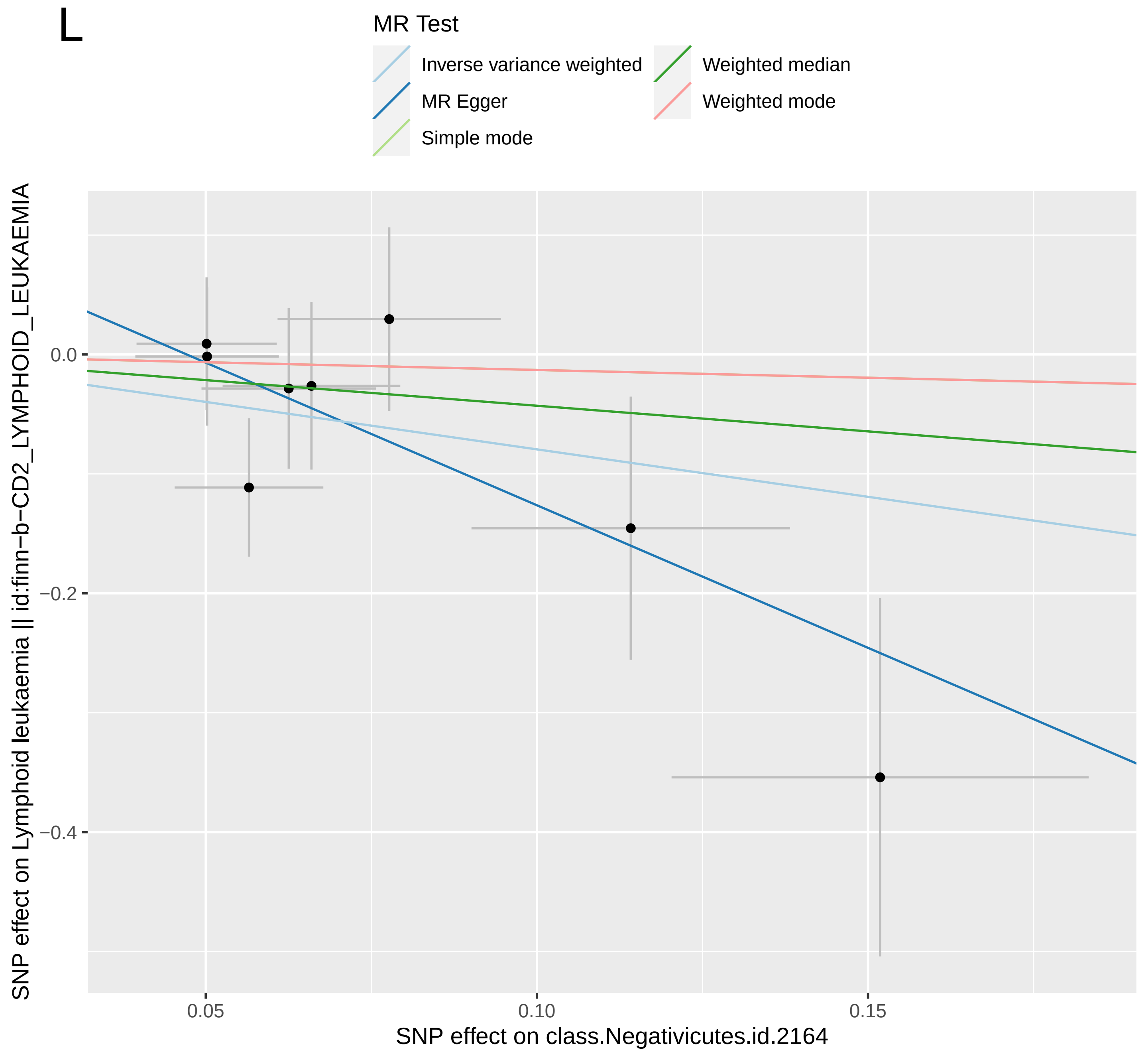
L
MR Test
Inverse variance weighted
MR Egger
Simple mode
Weighted median
Weighted mode
SNP effect on Lymphoid leukaemia || id:finn-b-CD2_LYMPHOID_LEUKAEMIA
0.0
−0.2
−0.4
0.05
0.10
0.15
SNP effect on class.Negativicutes.id.2164

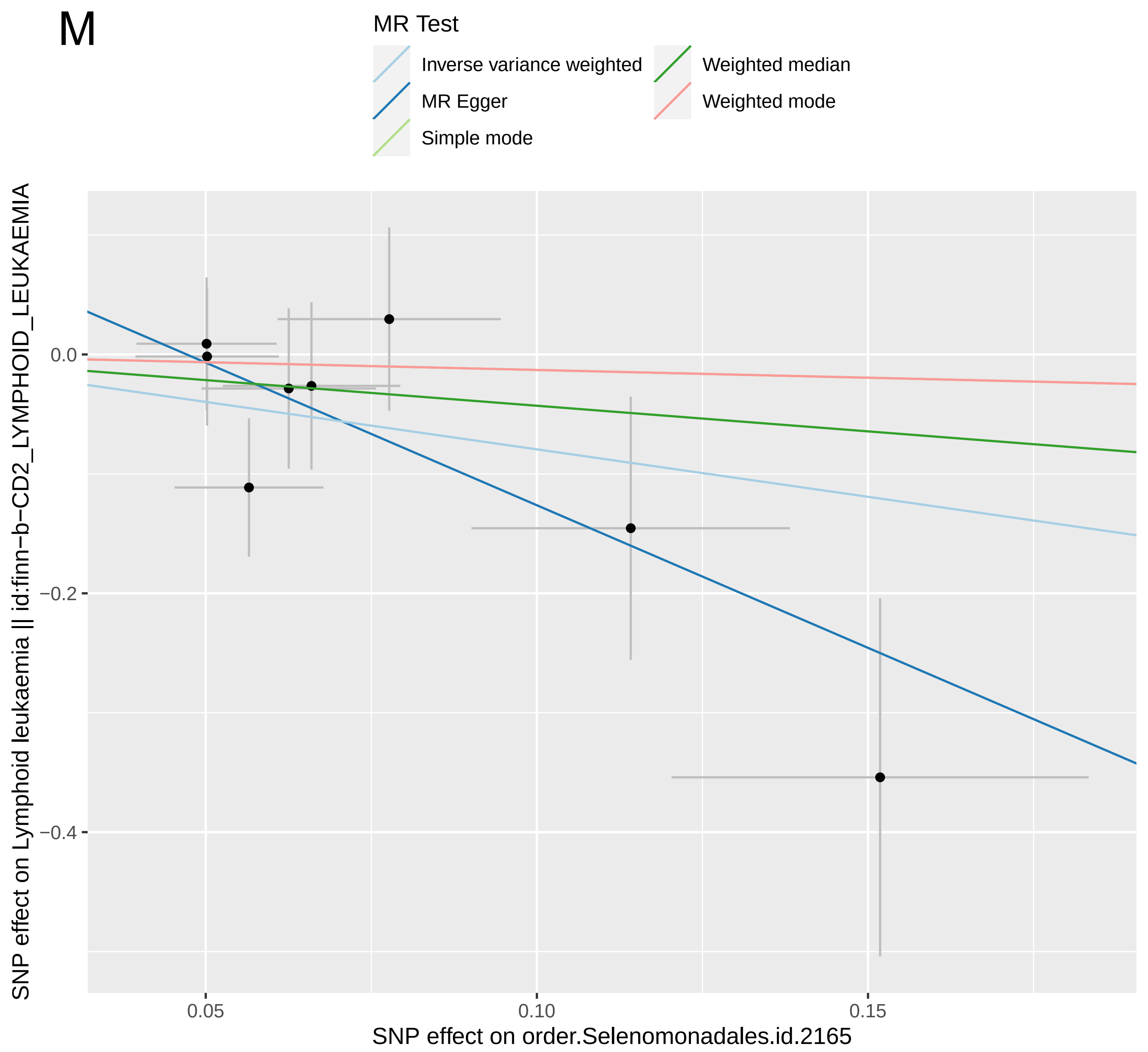
M
MR Test
Inverse variance weighted
MR Egger
Simple mode
Weighted median
Weighted mode
SNP effect on Lymphoid leukaemia || id:finn−b−CD2_LYMPHOID_LEUKAEMIA
0.0
−0.2
−0.4
0.05
0.10
0.15
SNP effect on order.Selenomonadales.id.2165

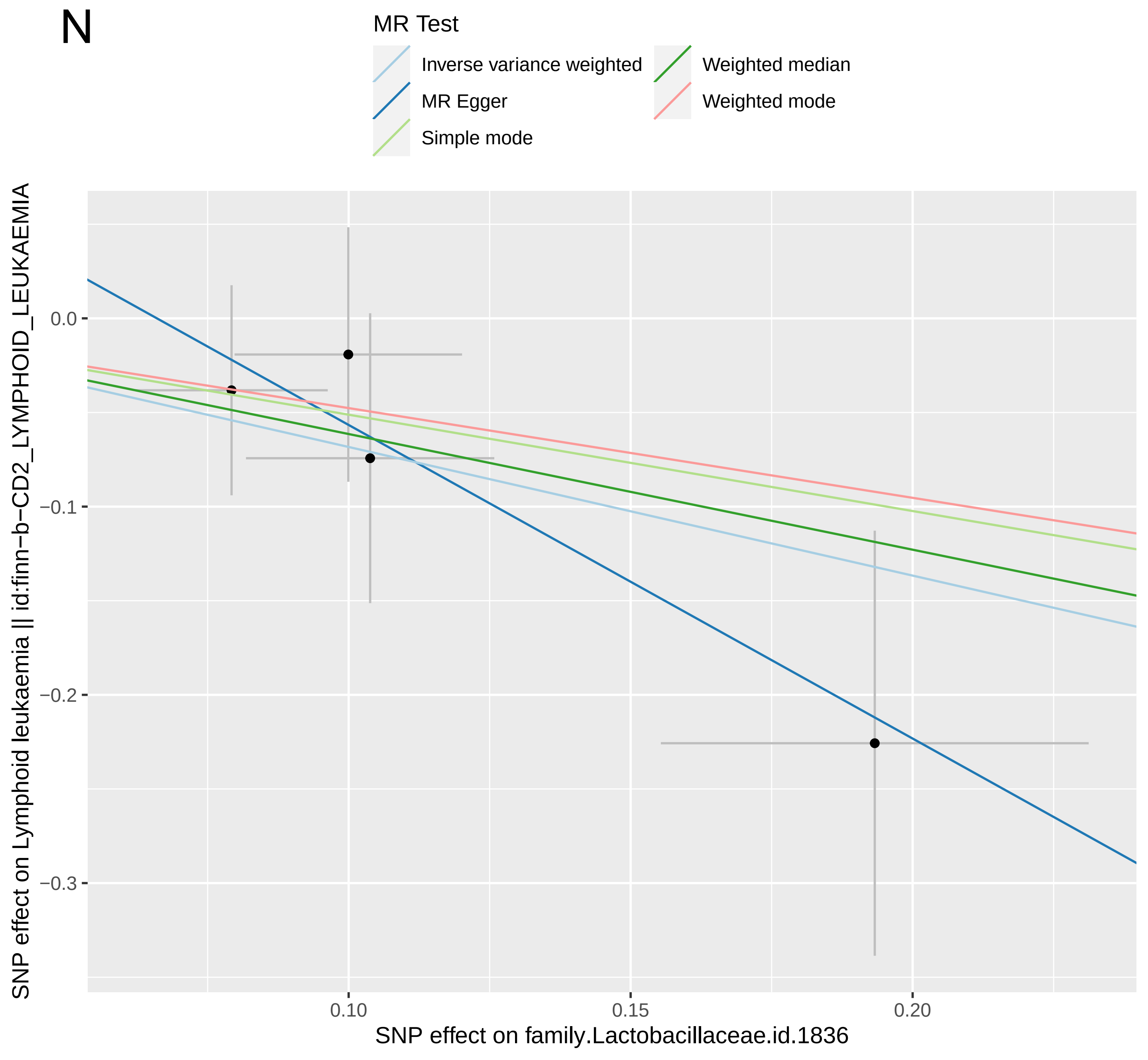
N
MR Test
Inverse variance weighted
MR Egger
Simple mode
Weighted median
Weighted mode
SNP effect on Lymphoid leukaemia || id:finn−b−CD2_LYMPHOID_LEUKAEMIA
0.0
−0.1
−0.2
−0.3
0.10
0.15
0.20
SNP effect on family.Lactobacillaceae.id.1836

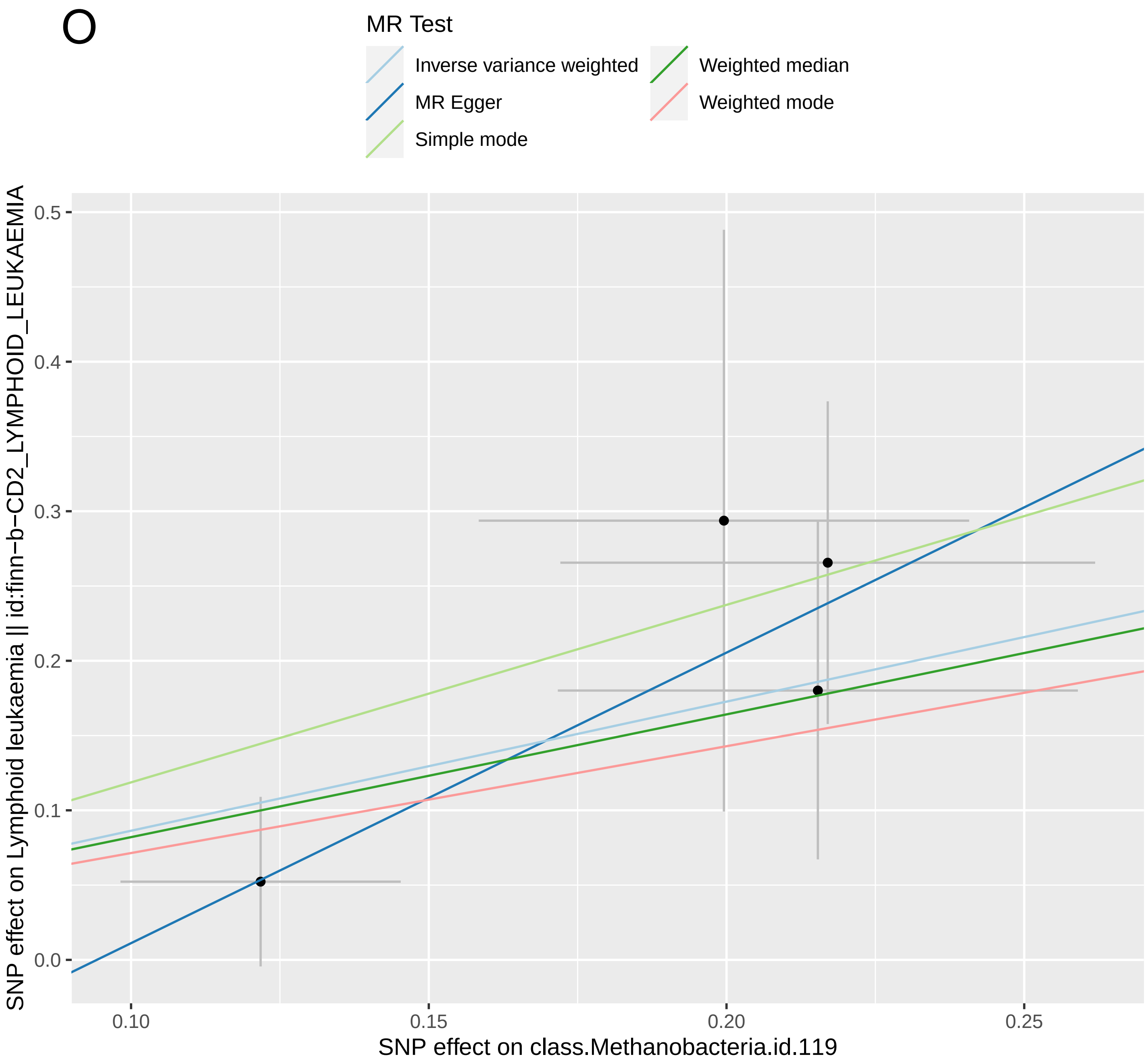
O
MR Test
Inverse variance weighted
MR Egger
Simple mode
Weighted median
Weighted mode
SNP effect on Lymphoid leukaemia || id:finn−b−CD2_LYMPHOID_LEUKAEMIA
SNP effect on class.Methanobacteria.id.119
0.0
0.1
0.2
0.3
0.4
0.5
0.10
0.15
0.20
0.25

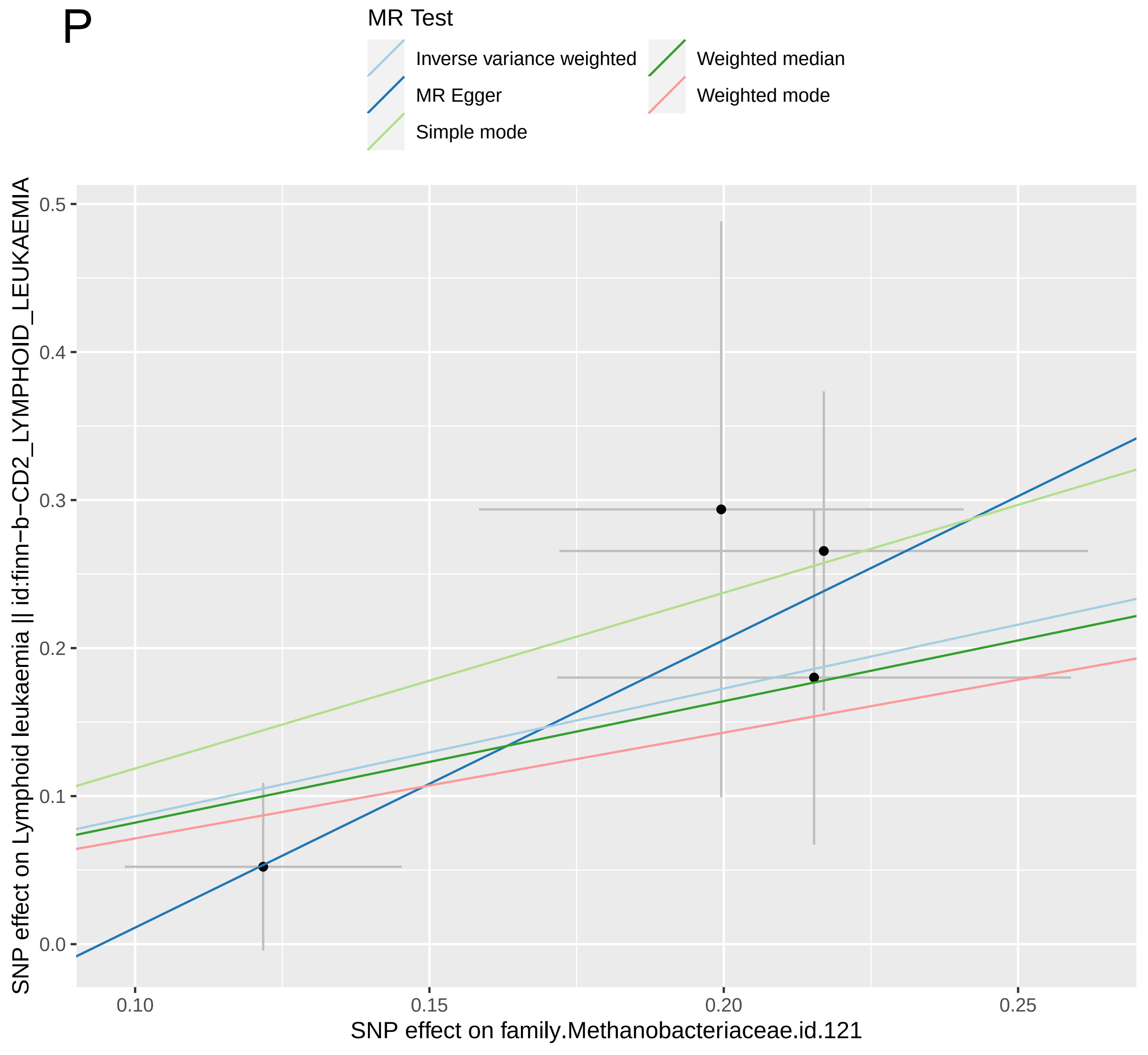
P
MR Test
Inverse variance weighted
MR Egger
Simple mode
Weighted median
Weighted mode
SNP effect on Lymphoid leukaemia || id:finn-b-CD2_LYMPHOID_LEUKAEMIA
0.0
0.1
0.2
0.3
0.4
0.5
0.10
0.15
0.20
0.25
SNP effect on family.Methanobacteriaceae.id.121

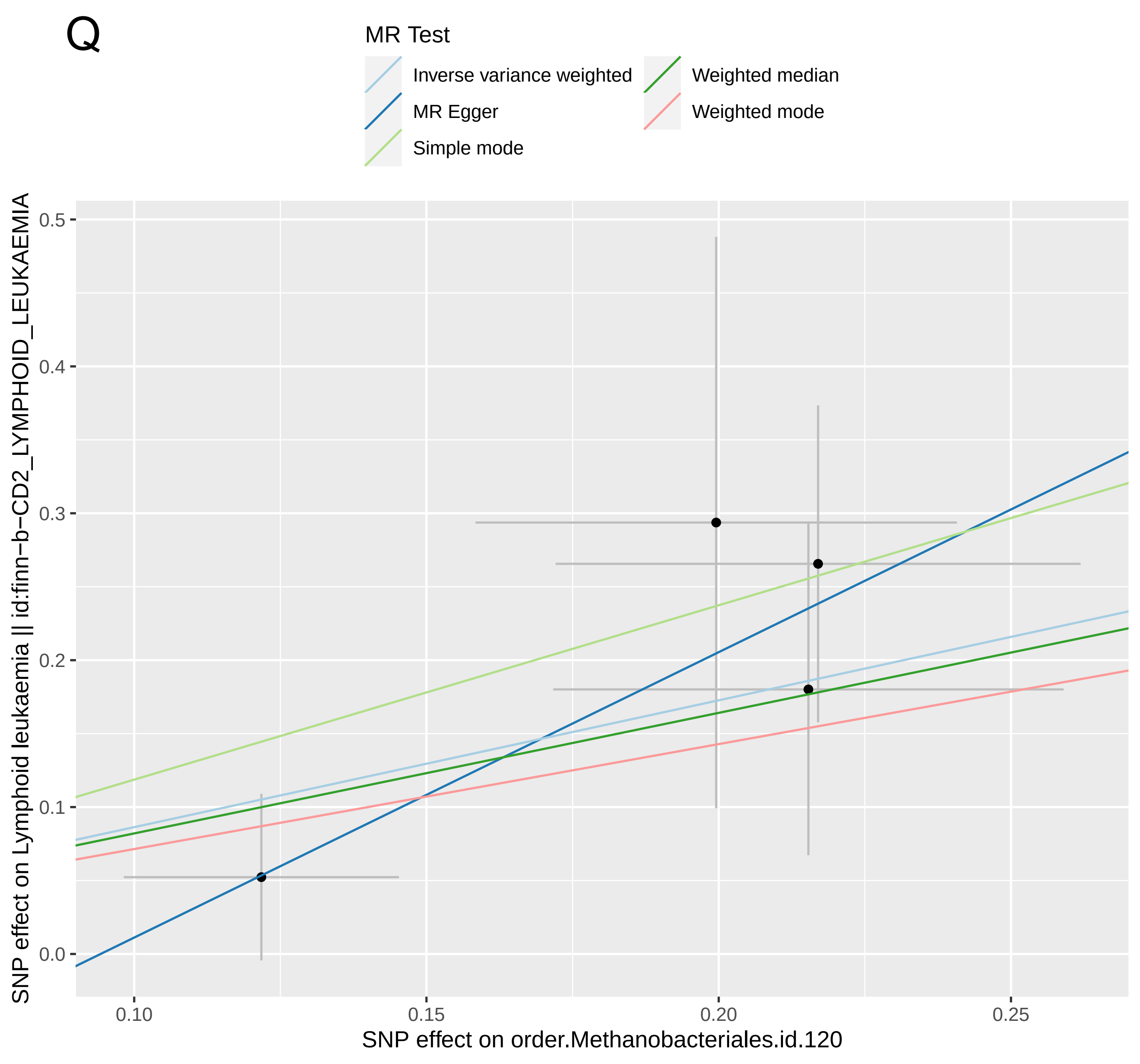
Q
MR Test
Inverse variance weighted
MR Egger
Simple mode
Weighted median
Weighted mode
SNP effect on Lymphoid leukaemia || id:finn-b-CD2_LYMPHOID_LEUKAEMIA
0.5
0.4
0.3
0.2
0.1
0.0
0.10
0.15
0.20
0.25
SNP effect on order.Methanobacteriales.id.120

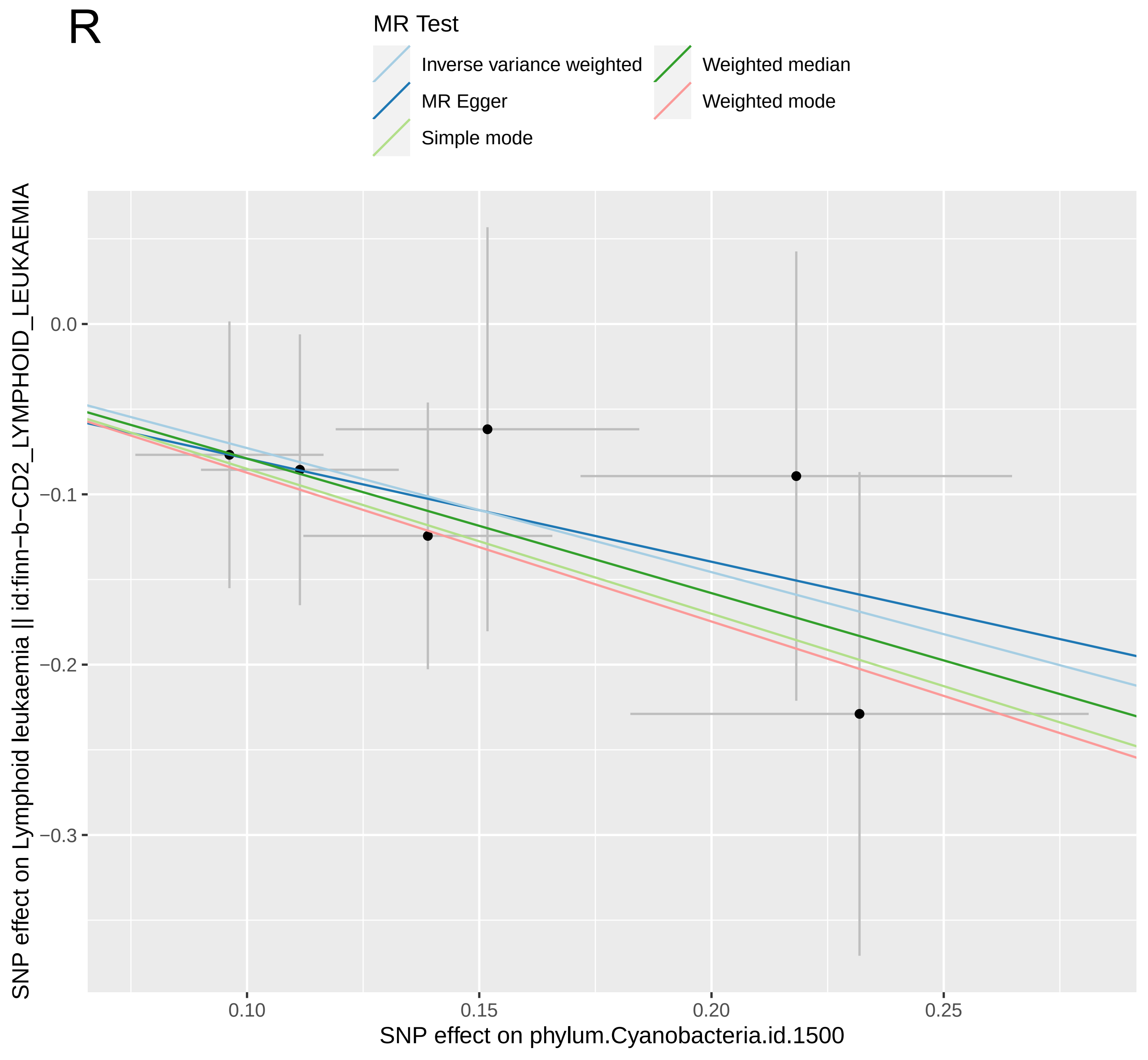
R
MR Test
Inverse variance weighted
MR Egger
Simple mode
Weighted median
Weighted mode
SNP effect on Lymphoid leukaemia || id:finn−b−CD2_LYMPHOID_LEUKAEMIA
0.0
−0.1
−0.2
−0.3
0.10
0.15
0.20
0.25
SNP effect on phylum.Cyanobacteria.id.1500

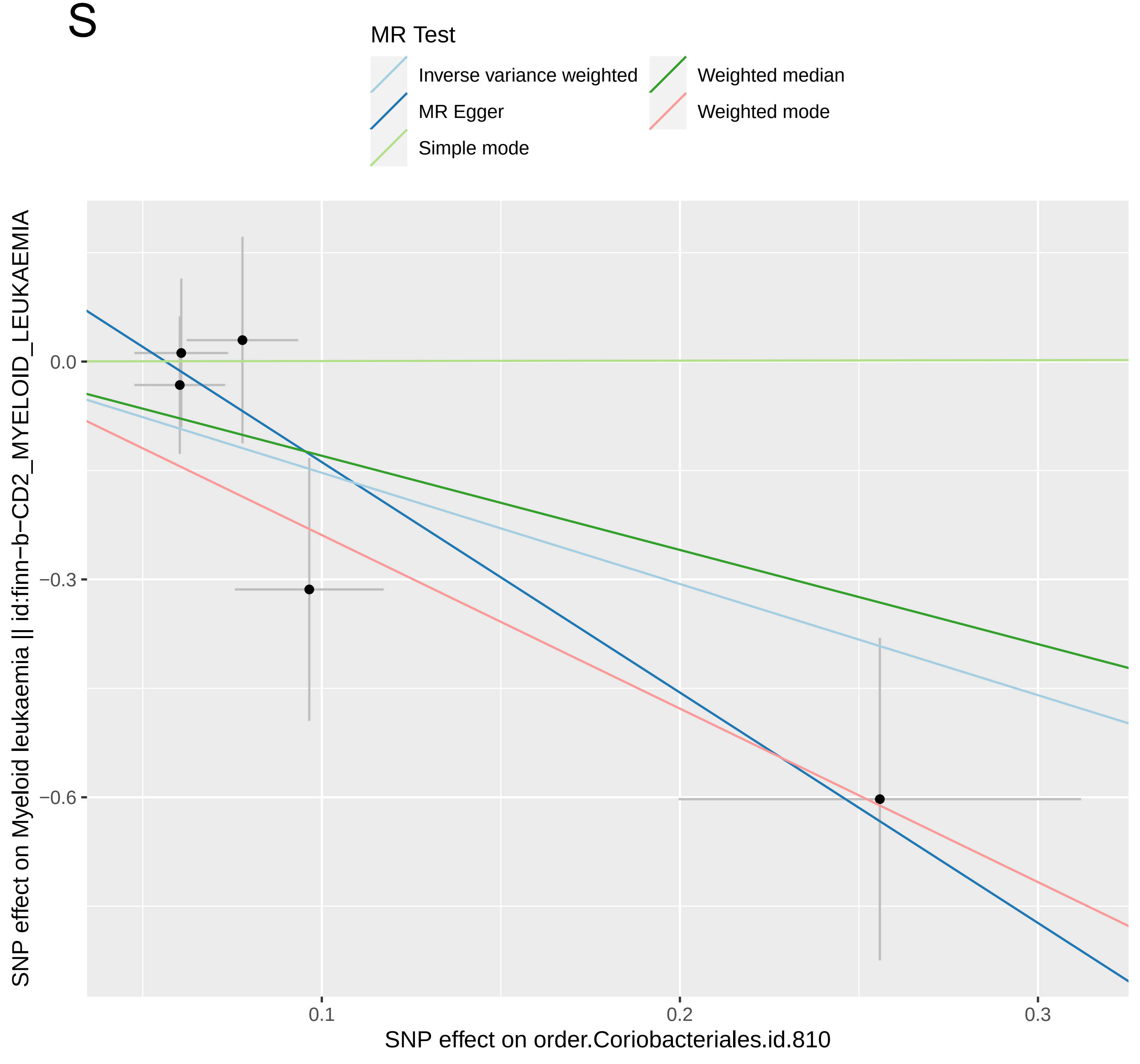
S
MR Test
Inverse variance weighted
MR Egger
Simple mode
Weighted median
Weighted mode
0.0
−0.3
−0.6
0.1
0.2
0.3
SNP effect on Myeloid leukaemia || id:finn−b−CD2_MYELOID_LEUKAEMIA
SNP effect on order.Coriobacteriales.id.810

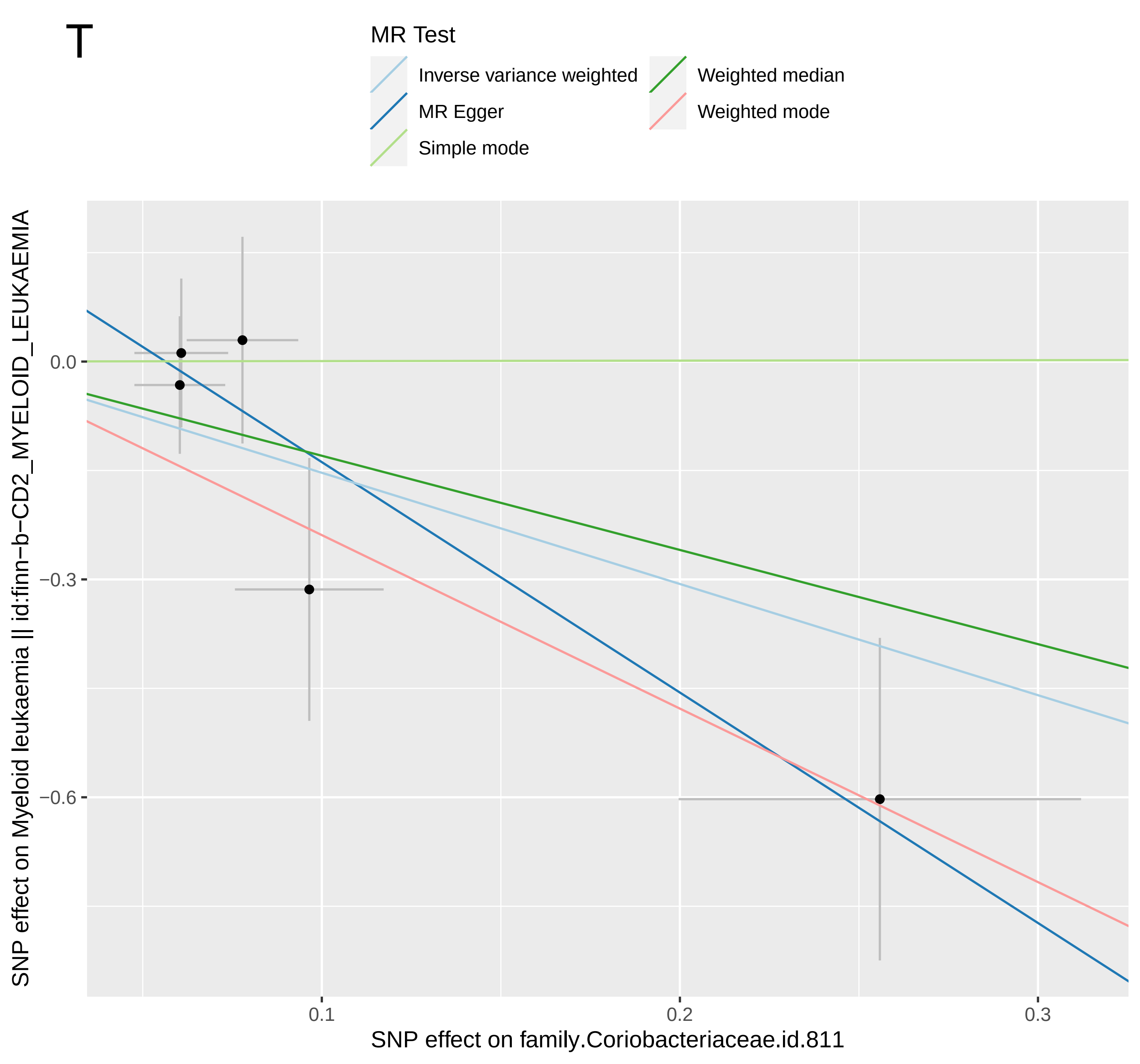
T
MR Test
Inverse variance weighted
MR Egger
Simple mode
Weighted median
Weighted mode
0.0
−0.3
−0.6
0.1
0.2
0.3
SNP effect on Myeloid leukaemia || id:finn−b−CD2_MYELOID_LEUKAEMIA
SNP effect on family.Coriobacteriaceae.id.811

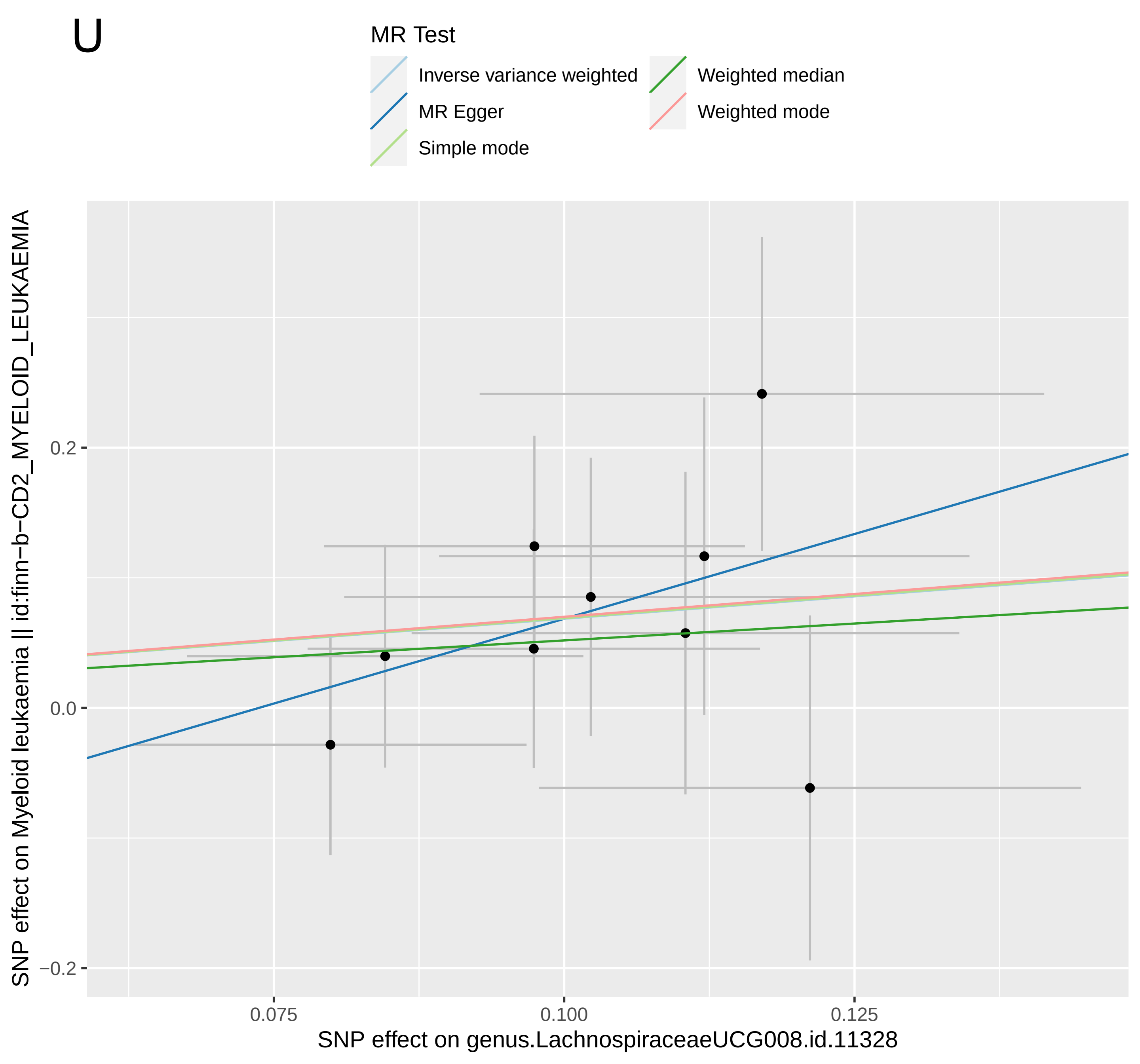

U
MR Test
Inverse variance weighted
MR Egger
Simple mode
Weighted median
Weighted mode
SNP effect on Myeloid leukaemia || id:finn−b−CD2_MYELOID_LEUKAEMIA
0.2
0.0
−0.2
0.075
0.100
0.125
SNP effect on genus.LachnospiraceaeUCG008.id.11328

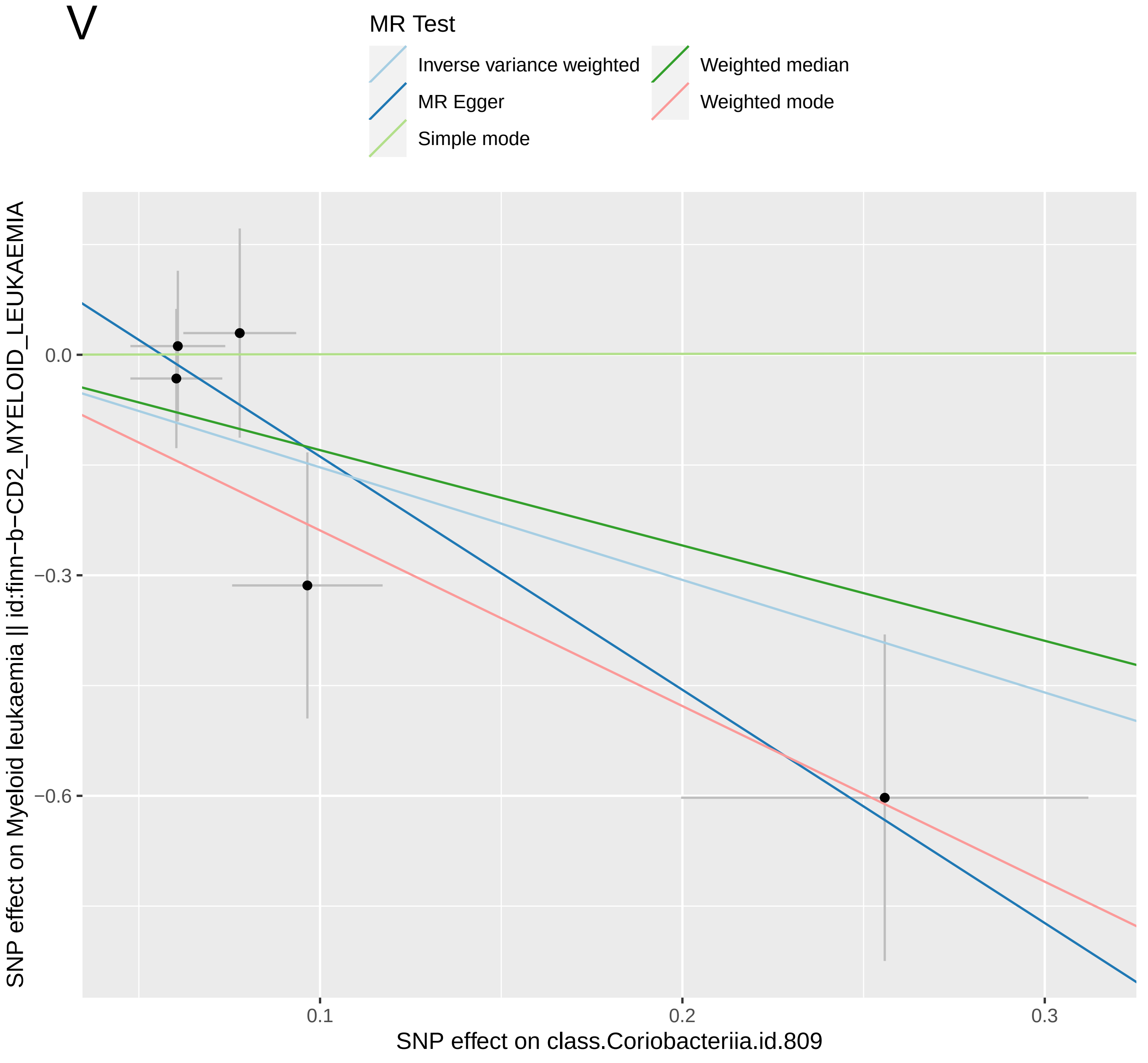

V
MR Test
Inverse variance weighted
MR Egger
Simple mode
Weighted median
Weighted mode
0.0
−0.3
−0.6
0.1
0.2
0.3
SNP effect on class.Coriobacteriia.id.809
SNP effect on Myeloid leukaemia || id:finn−b−CD2_MYELOID_LEUKAEMIA

W

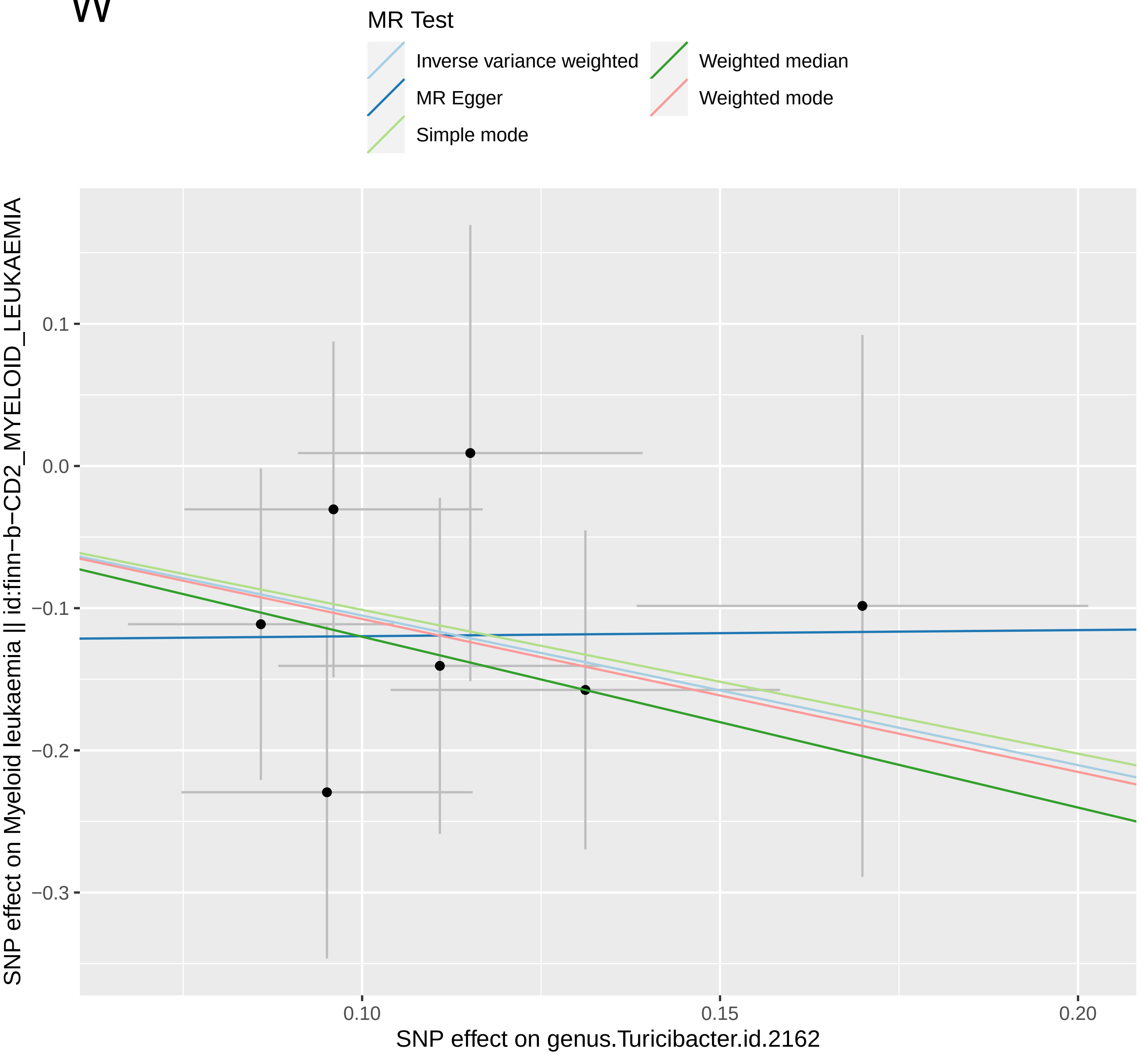
MR Test
Inverse variance weighted
MR Egger
Simple mode
Weighted median
Weighted mode
SNP effect on Myeloid leukaemia || id:finn−b−CD2_MYELOID_LEUKAEMIA
0.1
0.0
−0.1
−0.2
−0.3
0.10
0.15
0.20
SNP effect on genus.Turicibacter.id.2162

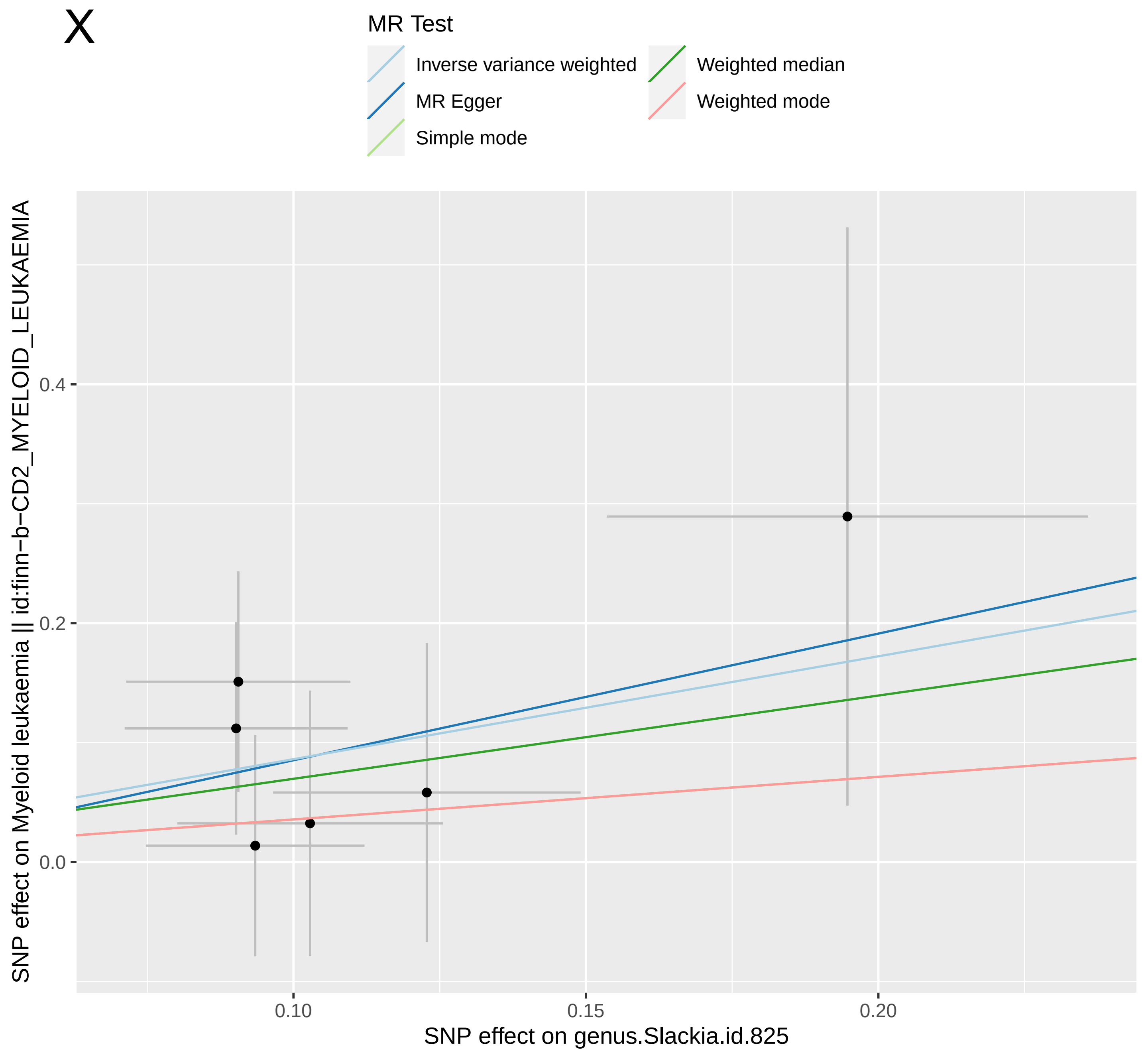

X
MR Test
Inverse variance weighted
MR Egger
Simple mode
Weighted median
Weighted mode
SNP effect on Myeloid leukaemia || id:finn−b−CD2_MYELOID_LEUKAEMIA
0.4
0.2
0.0
0.10
0.15
0.20
SNP effect on genus.Slackia.id.825

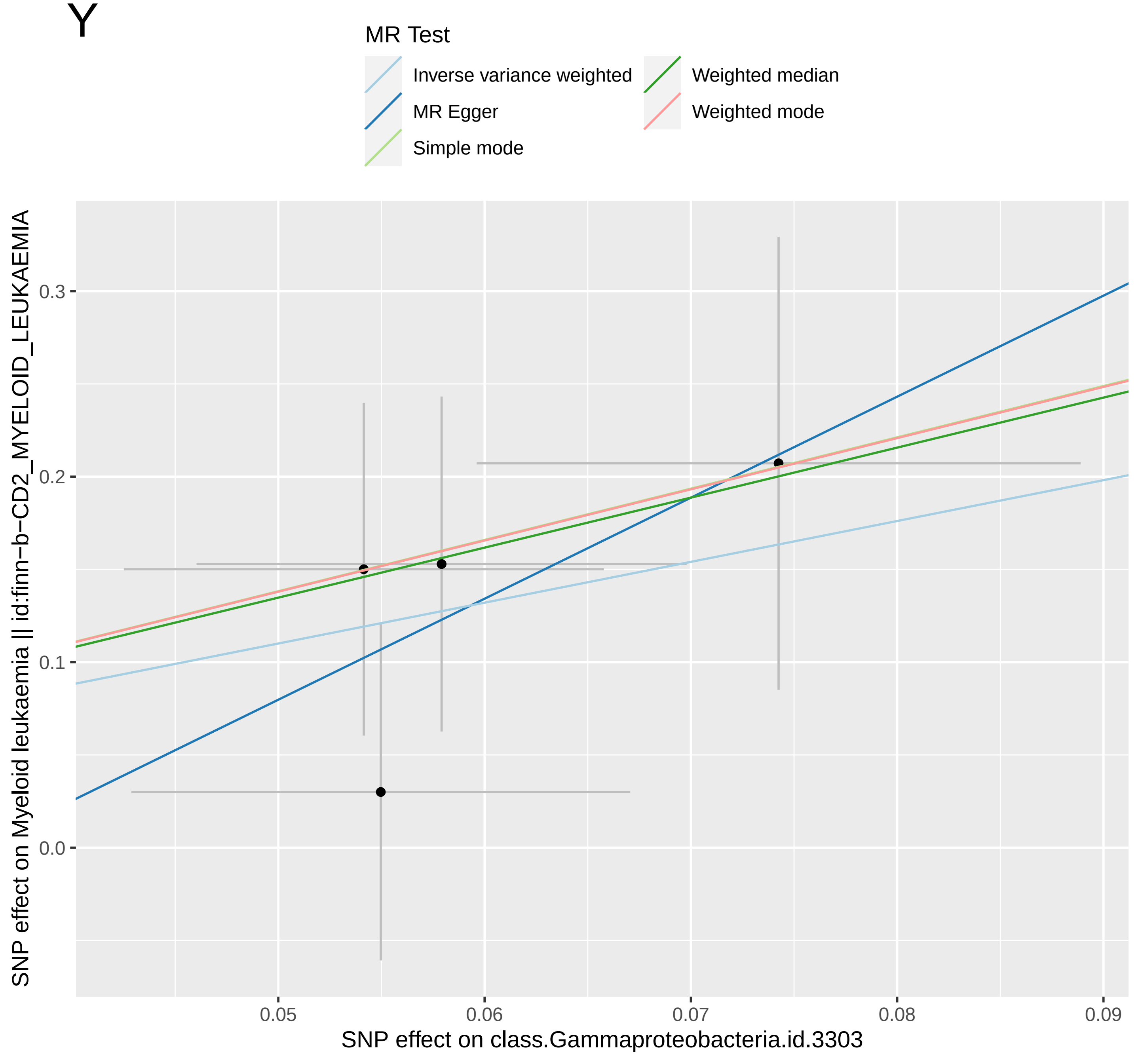
Y
MR Test
Inverse variance weighted
MR Egger
Simple mode
Weighted median
Weighted mode
SNP effect on Myeloid leukaemia || id:finn-b-CD2_MYELOID_LEUKAEMIA
SNP effect on class.Gammaproteobacteria.id.3303
0.0
0.1
0.2
0.3
0.05
0.06
0.07
0.08
0.09

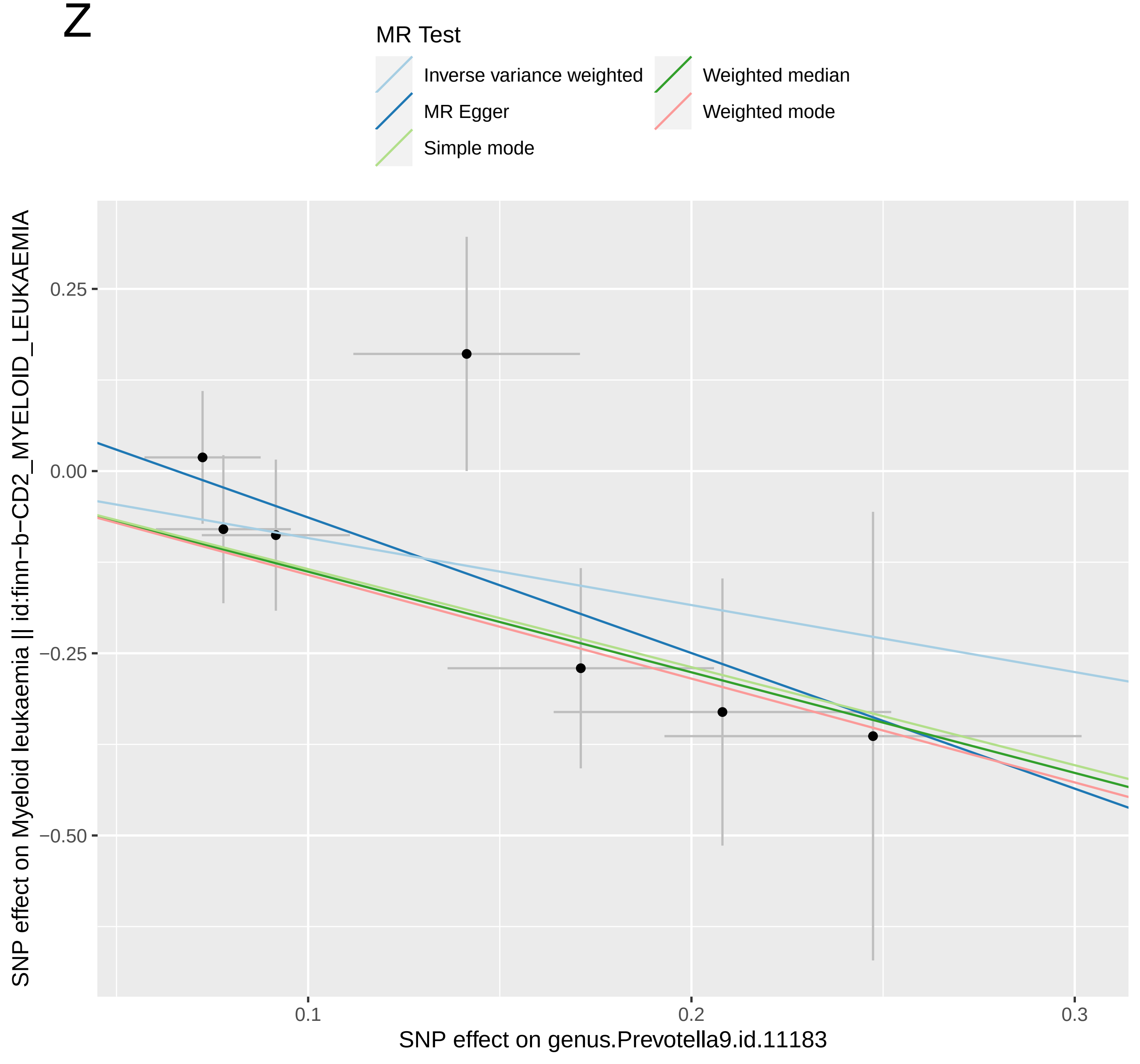

Z
MR Test
Inverse variance weighted
MR Egger
Simple mode
Weighted median
Weighted mode
SNP effect on Myeloid leukaemia || id:finn-b-CD2_MYELOID_LEUKAEMIA
0.25
0.00
−0.25
−0.50
0.1
0.2
0.3
SNP effect on genus.Prevotella9.id.11183

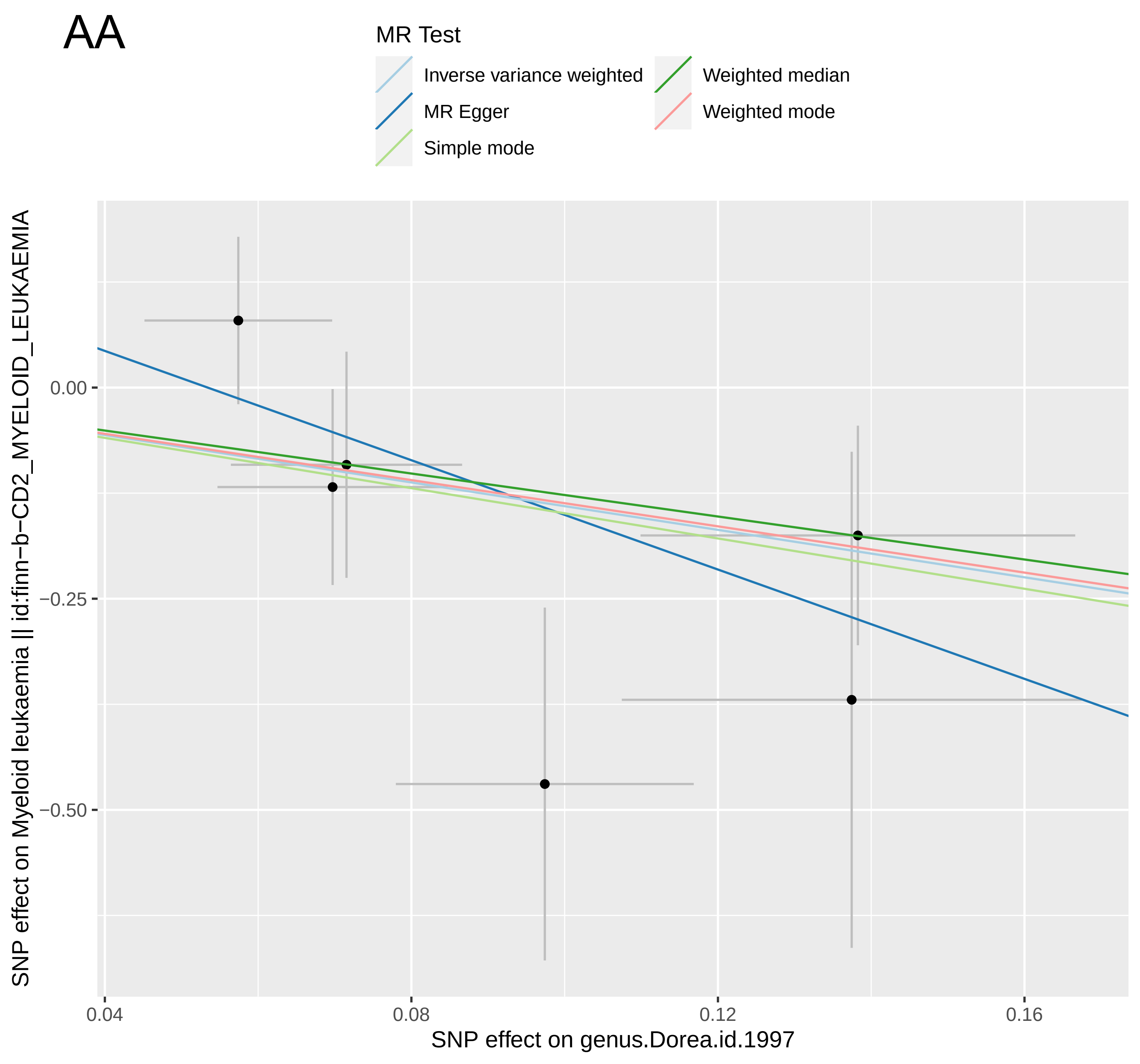

AA
MR Test
Inverse variance weighted
MR Egger
Simple mode
Weighted median
Weighted mode
SNP effect on Myeloid leukaemia || id:finn−b−CD2_MYELOID_LEUKAEMIA
0.00
−0.25
−0.50
0.04
0.08
0.12
0.16
SNP effect on genus.Dorea.id.1997

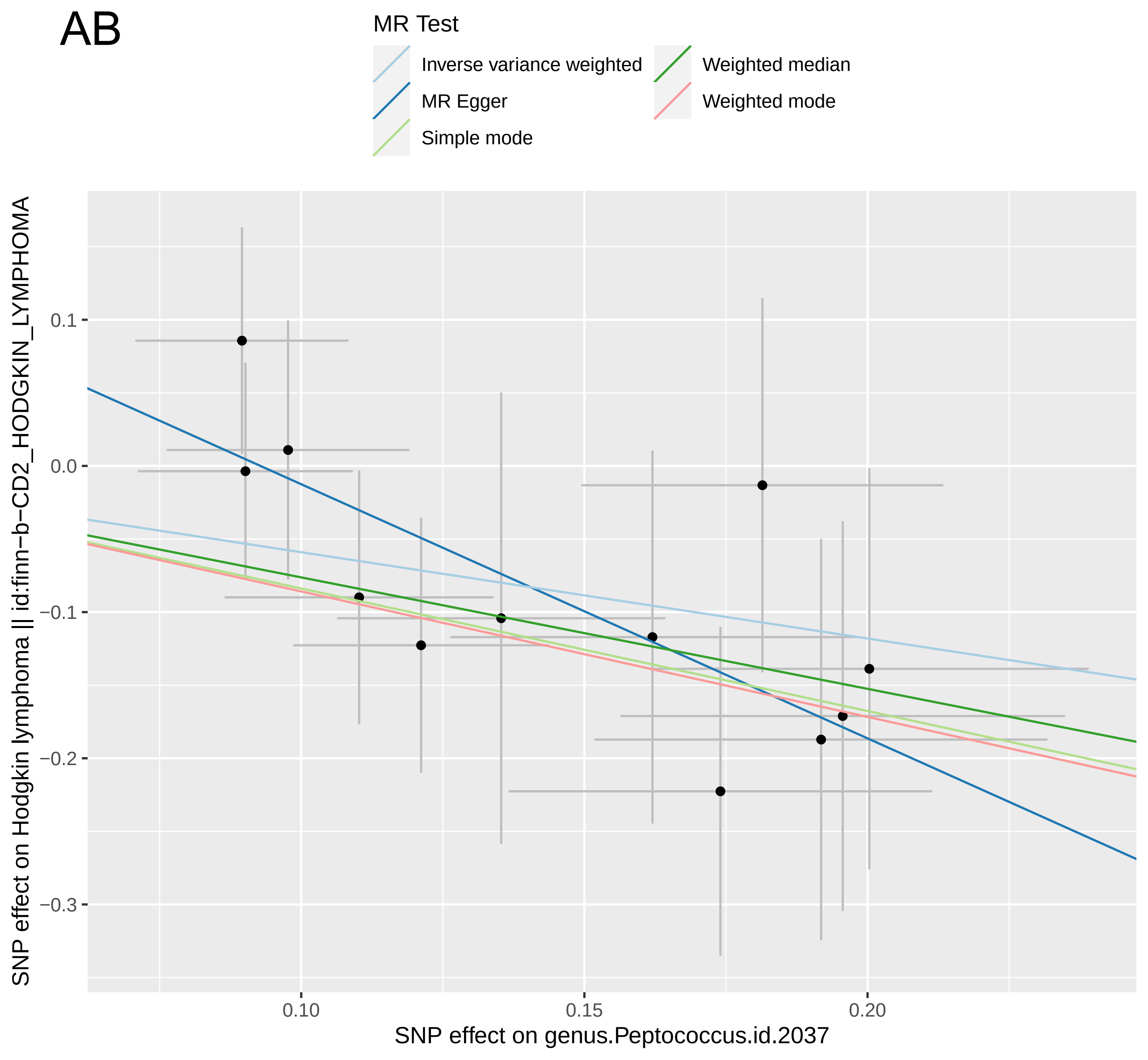

AB
MR Test
Inverse variance weighted
MR Egger
Simple mode
Weighted median
Weighted mode
SNP effect on Hodgkin lymphoma || id:finn−b−CD2_HODGKIN_LYMPHOMA
0.1
0.0
−0.1
−0.2
−0.3
0.10
0.15
0.20
SNP effect on genus.Peptococcus.id.2037

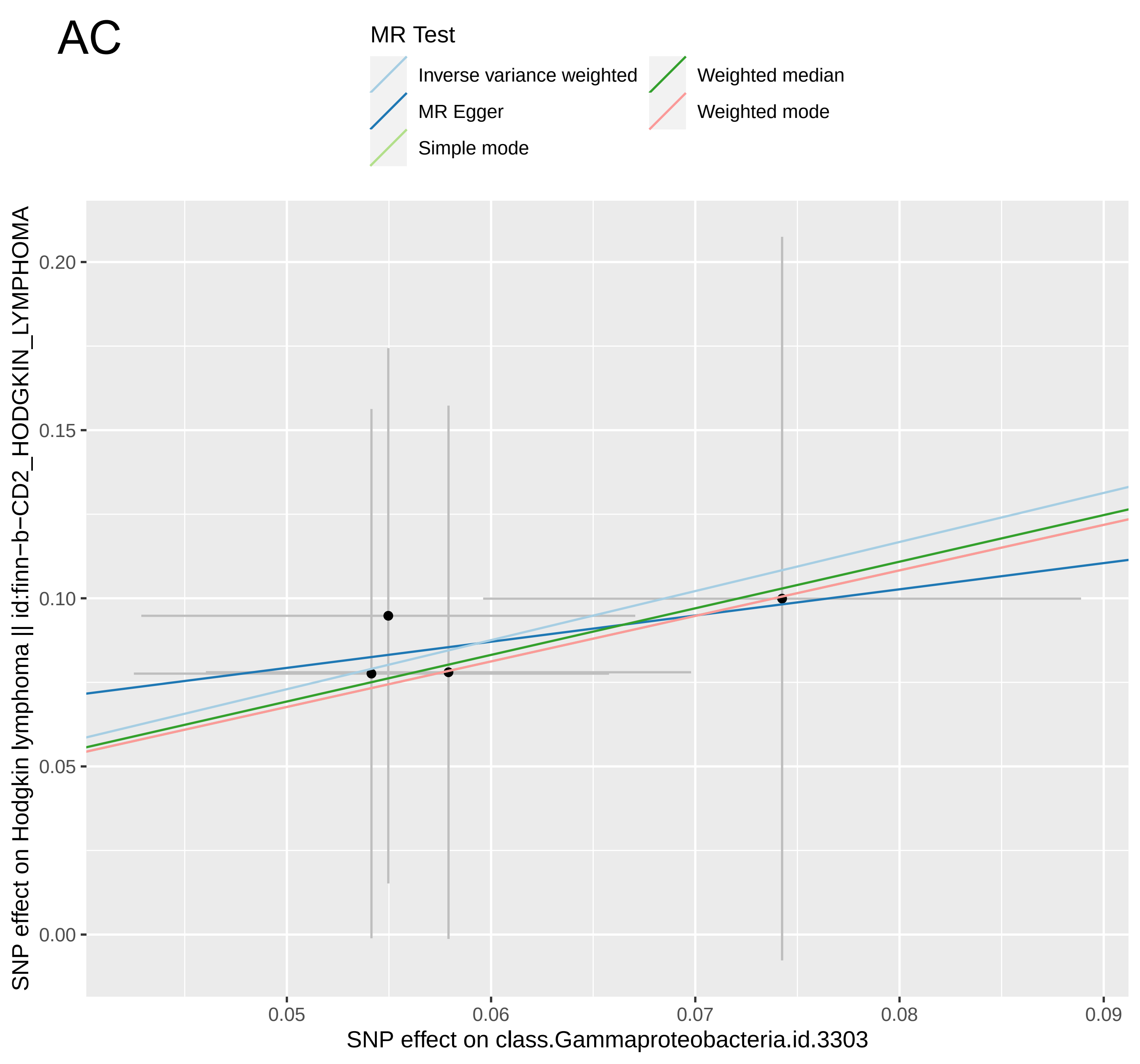
AC
MR Test
Inverse variance weighted
MR Egger
Simple mode
Weighted median
Weighted mode
SNP effect on Hodgkin lymphoma || id:finn-b-CD2_HODGKIN_LYMPHOMA
0.20
0.15
0.10
0.05
0.00
0.05
0.06
0.07
0.08
0.09
SNP effect on class.Gammaproteobacteria.id.3303

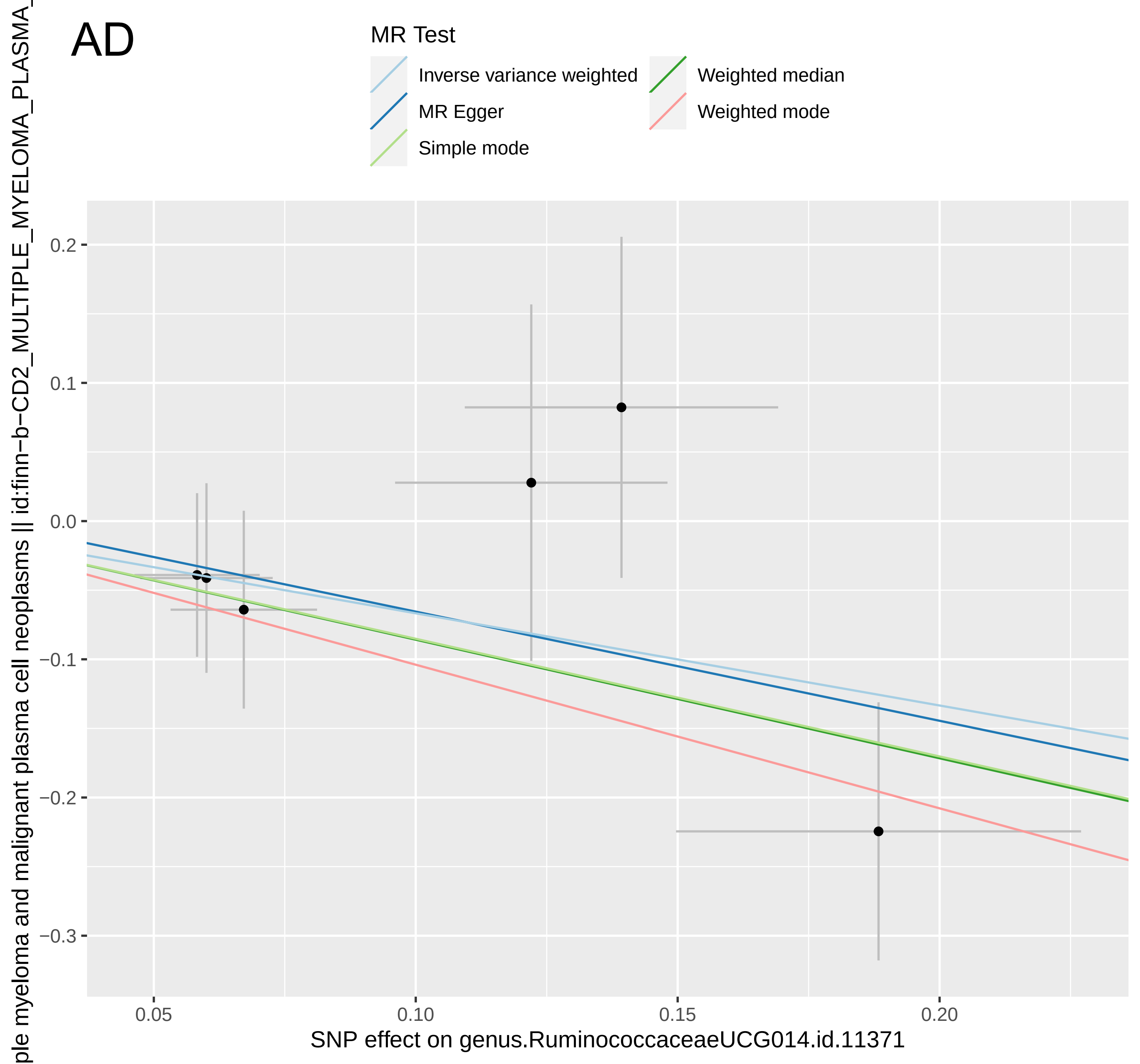

AD
MR Test
Inverse variance weighted
MR Egger
Simple mode
Weighted median
Weighted mode
0.2
0.1
0.0
−0.1
−0.2
−0.3
0.05
0.10
0.15
0.20
SNP effect on genus.RuminococcaceaeUCG014.id.11371
ple myeloma and malignant plasma cell neoplasms || id:finn−b−CD2_MULTIPLE_MYELOMA_PLASMA_

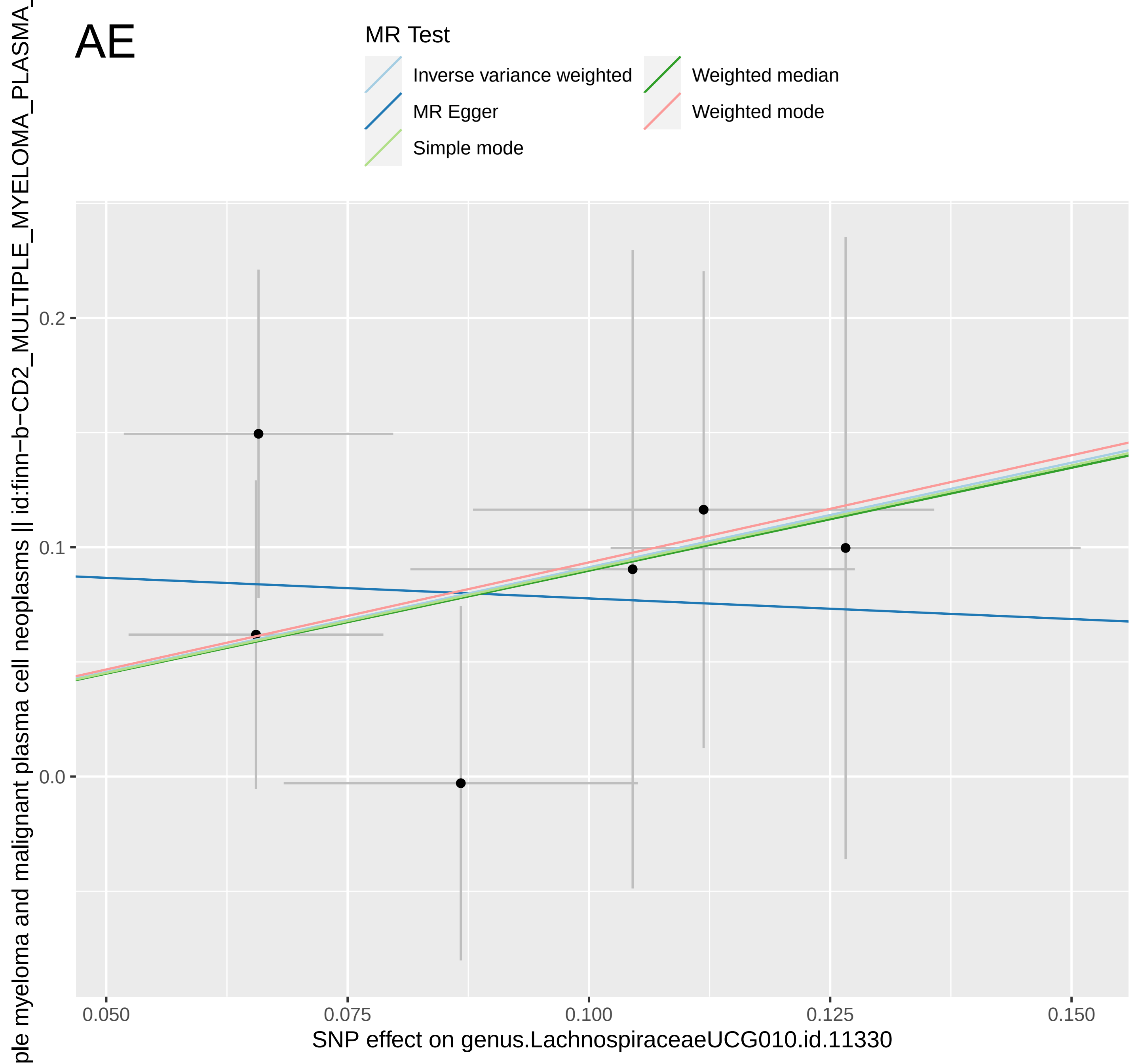

AE
MR Test
Inverse variance weighted
MR Egger
Simple mode
Weighted median
Weighted mode
0.2
0.1
0.0
0.050
0.075
0.100
0.125
0.150
SNP effect on genus.LachnospiraceaeUCG010.id.11330
ple myeloma and malignant plasma cell neoplasms || id:finn-b-CD2_MULTIPLE_MYELOMA_PLASMA_

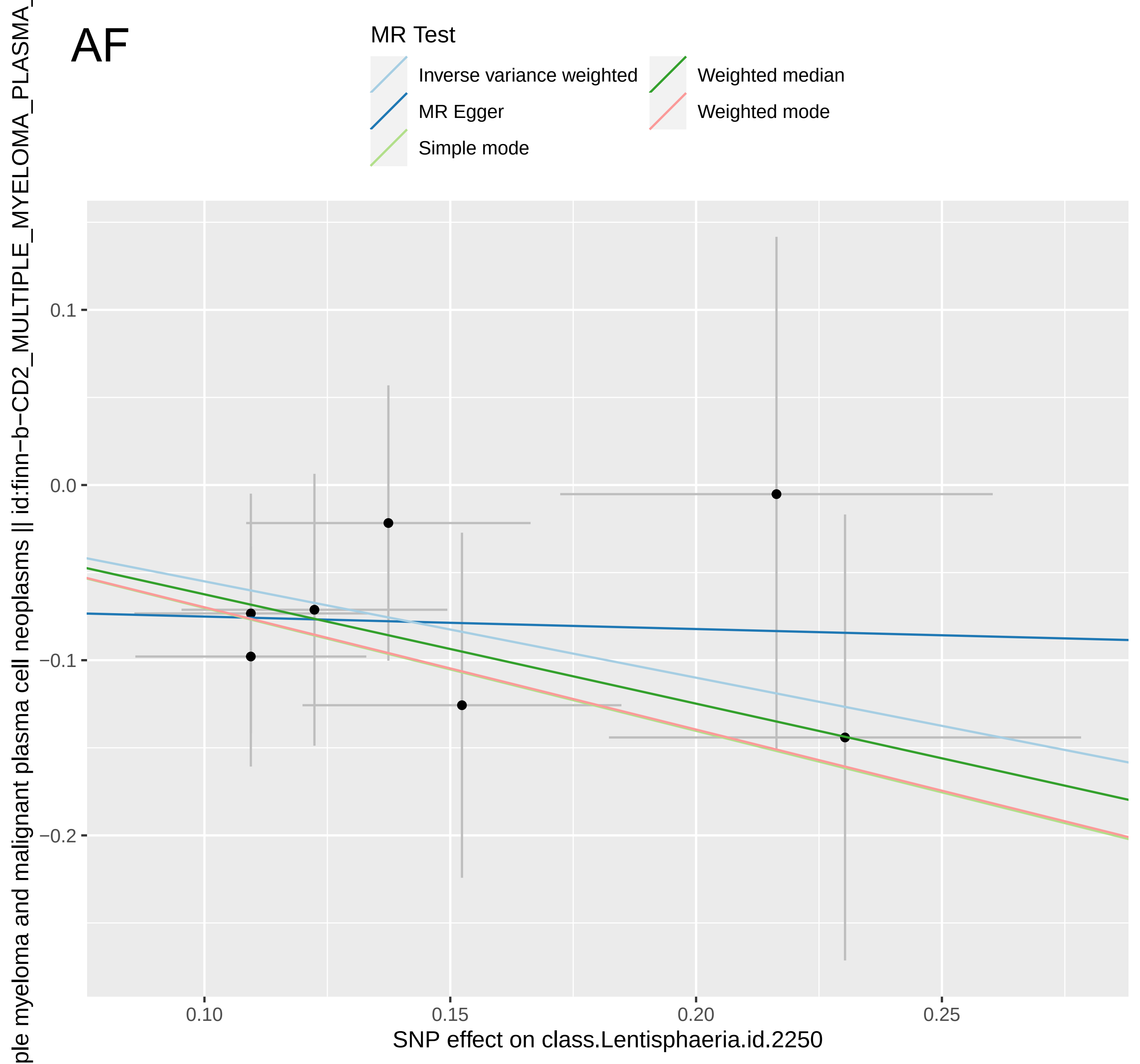
AF
MR Test
Inverse variance weighted
MR Egger
Simple mode
Weighted median
Weighted mode
0.1
0.0
−0.1
−0.2
0.10
0.15
0.20
0.25
SNP effect on class.Lentisphaeria.id.2250
ple myeloma and malignant plasma cell neoplasms || id:finn−b−CD2_MULTIPLE_MYELOMA_PLASMA_

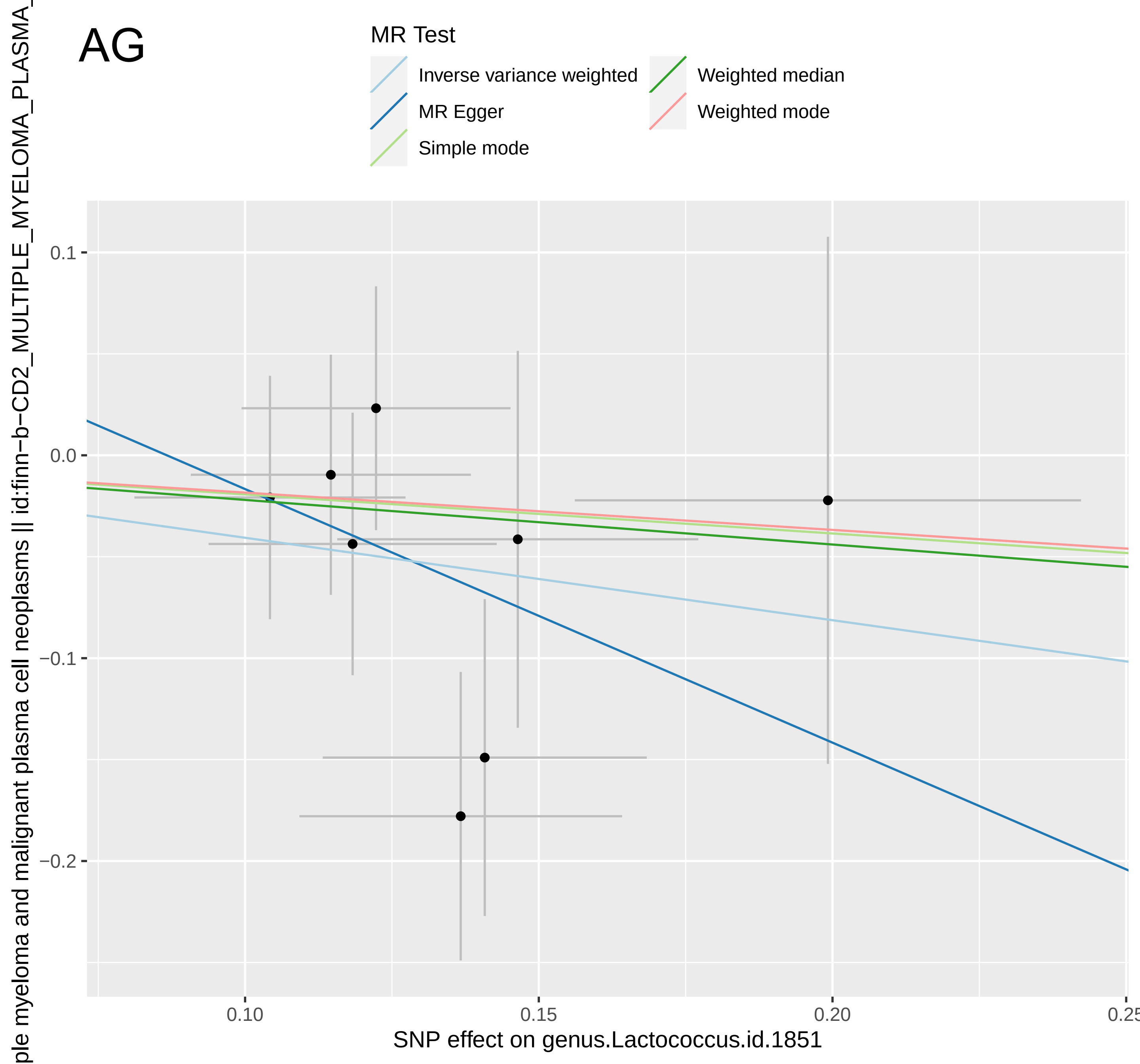

AG
MR Test
Inverse variance weighted
MR Egger
Simple mode
Weighted median
Weighted mode
0.1
0.0
−0.1
−0.2
0.10
0.15
0.20
0.25
SNP effect on genus.Lactococcus.id.1851
ple myeloma and malignant plasma cell neoplasms || id:finn−b−CD2_MULTIPLE_MYELOMA_PLASMA_

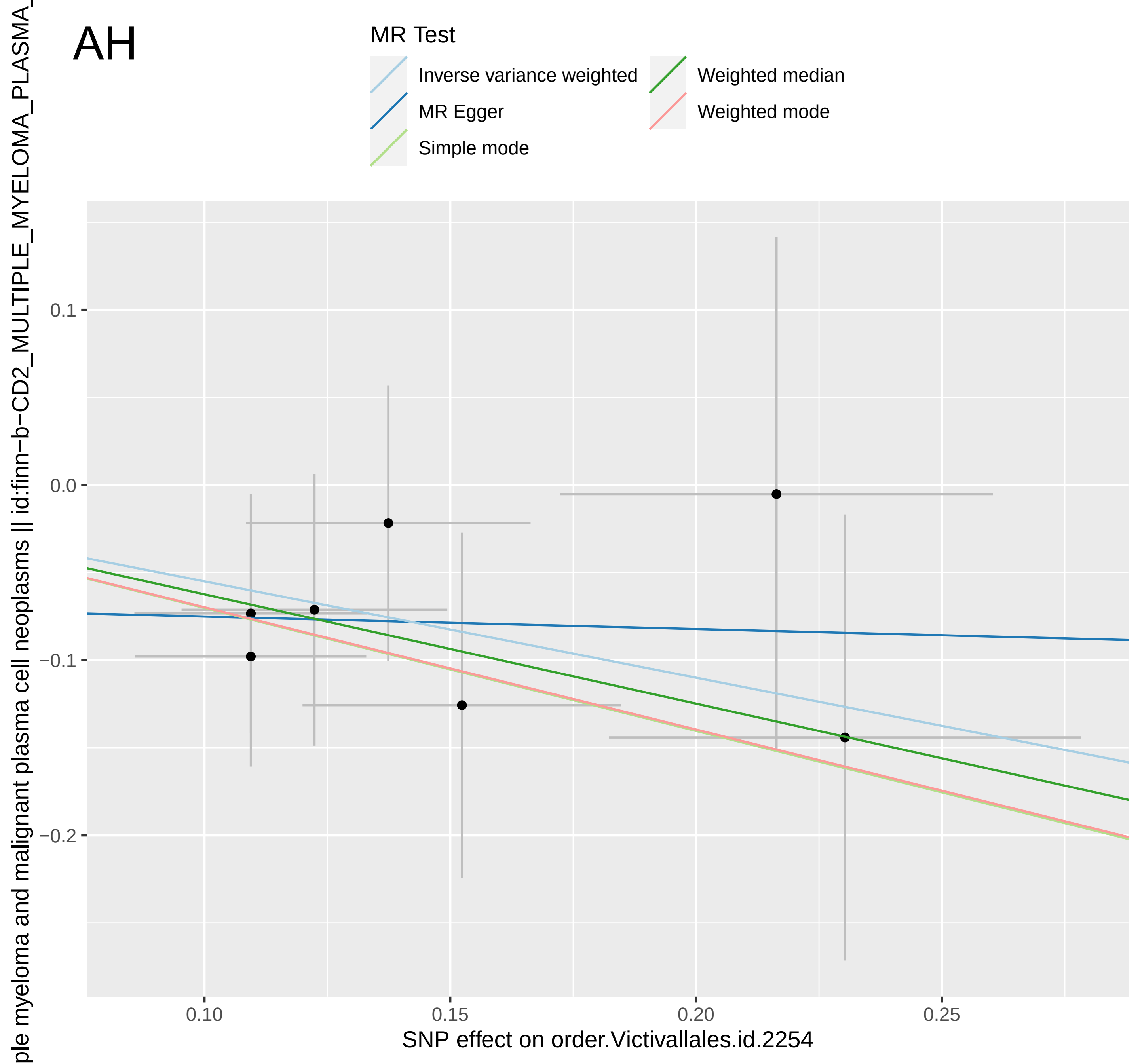

AH
MR Test
Inverse variance weighted
MR Egger
Simple mode
Weighted median
Weighted mode
ple myeloma and malignant plasma cell neoplasms || id:finn−b−CD2_MULTIPLE_MYELOMA_PLASMA_
0.1
0.0
−0.1
−0.2
0.10
0.15
0.20
0.25
SNP effect on order.Victivallales.id.2254

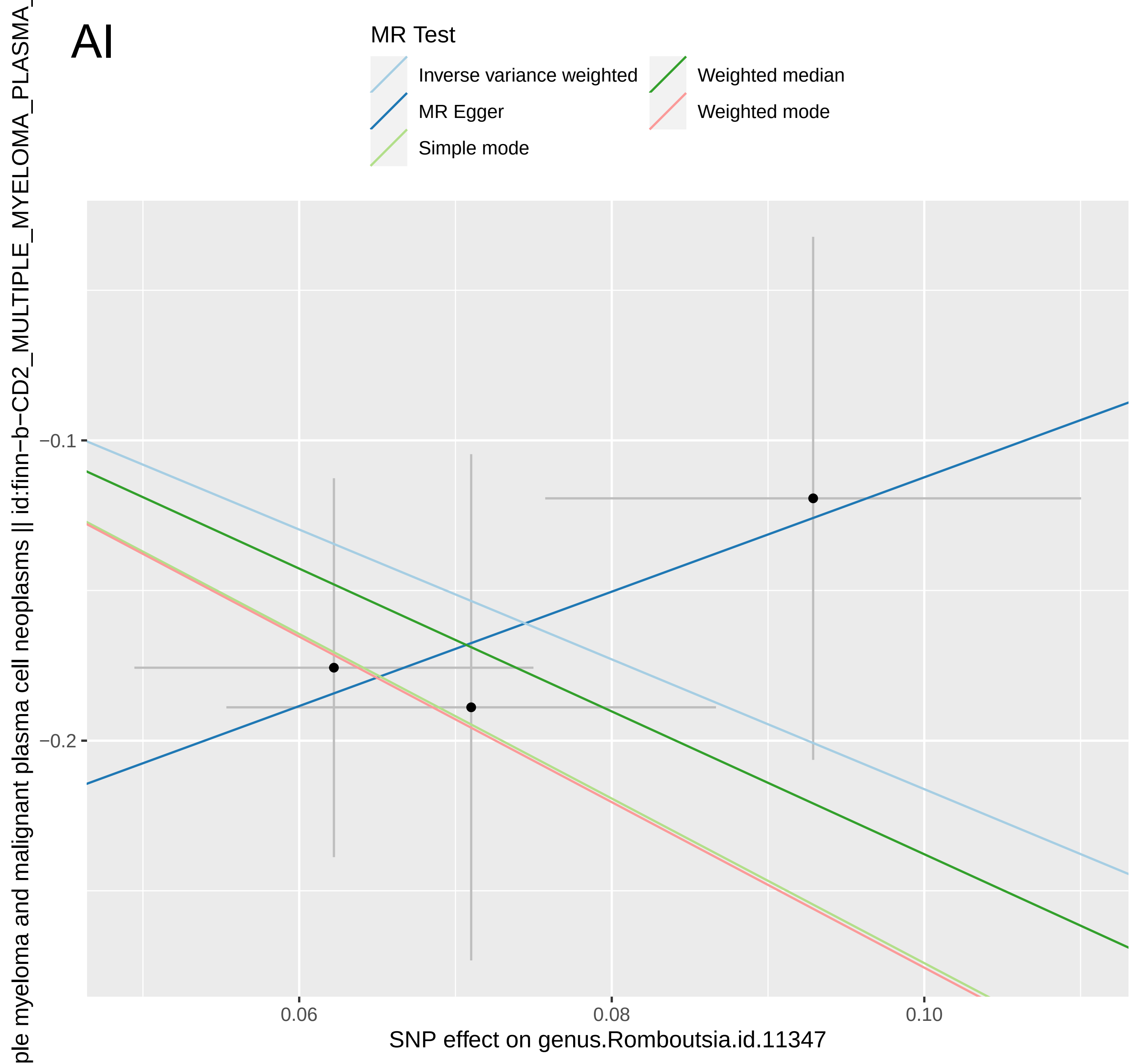
AI
MR Test
Inverse variance weighted
MR Egger
Simple mode
Weighted median
Weighted mode
−0.1
−0.2
0.06
0.08
0.10
SNP effect on genus.Romboutsia.id.11347
ple myeloma and malignant plasma cell neoplasms || id:finn−b−CD2_MULTIPLE_MYELOMA_PLASMA_

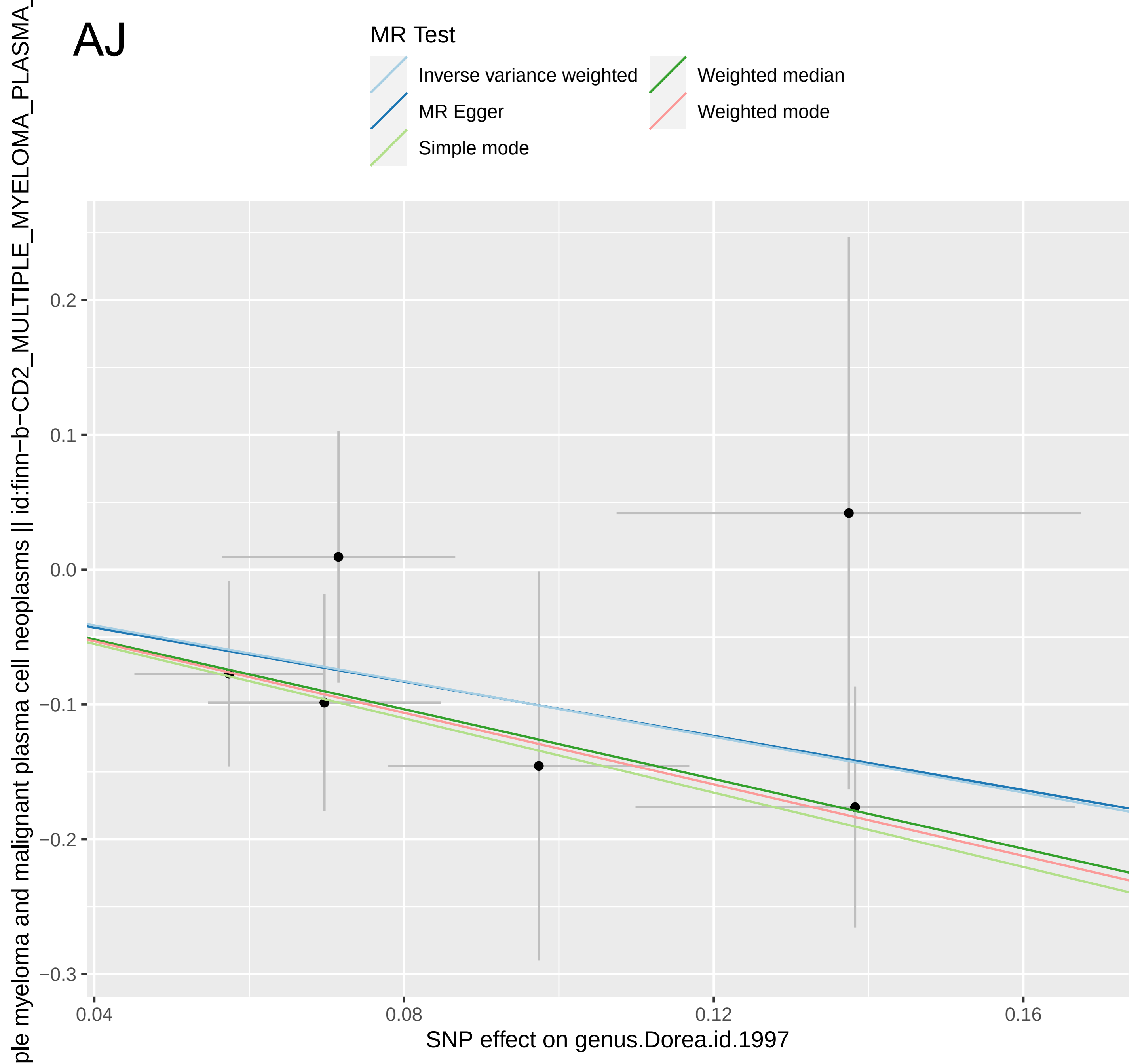
AJ
MR Test
Inverse variance weighted
MR Egger
Simple mode
Weighted median
Weighted mode
ple myeloma and malignant plasma cell neoplasms || id:finn-b-CD2_MULTIPLE_MYELOMA_PLASMA_
0.2
0.1
0.0
−0.1
−0.2
−0.3
0.04
0.08
0.12
0.16
SNP effect on genus.Dorea.id.1997

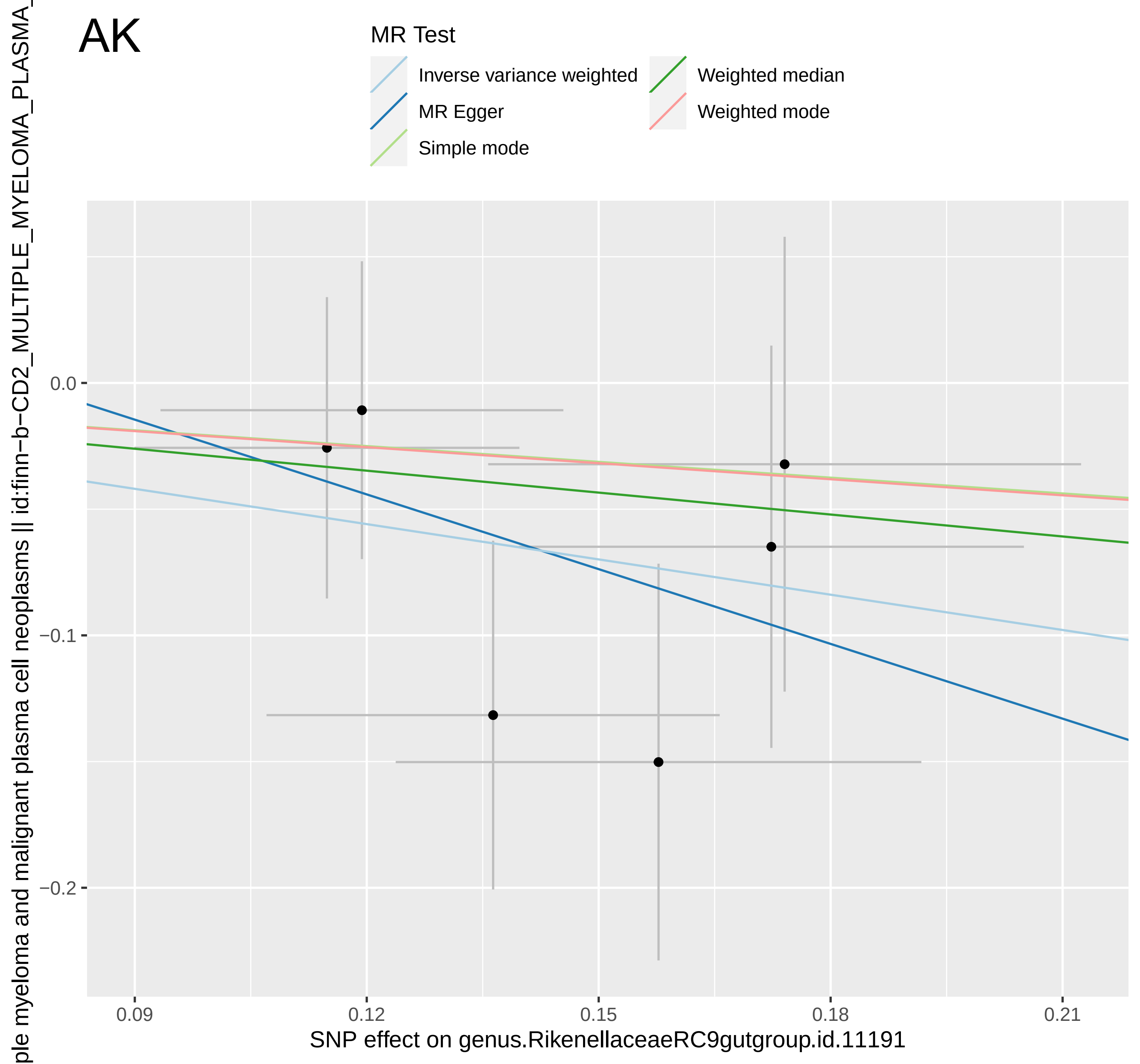
AK
MR Test
Inverse variance weighted
MR Egger
Simple mode
Weighted median
Weighted mode
0.0
−0.1
−0.2
0.09
0.12
0.15
0.18
0.21
SNP effect on genus.RikenellaceaeRC9gutgroup.id.11191
ple myeloma and malignant plasma cell neoplasms || id:finn−b−CD2_MULTIPLE_MYELOMA_PLASMA_

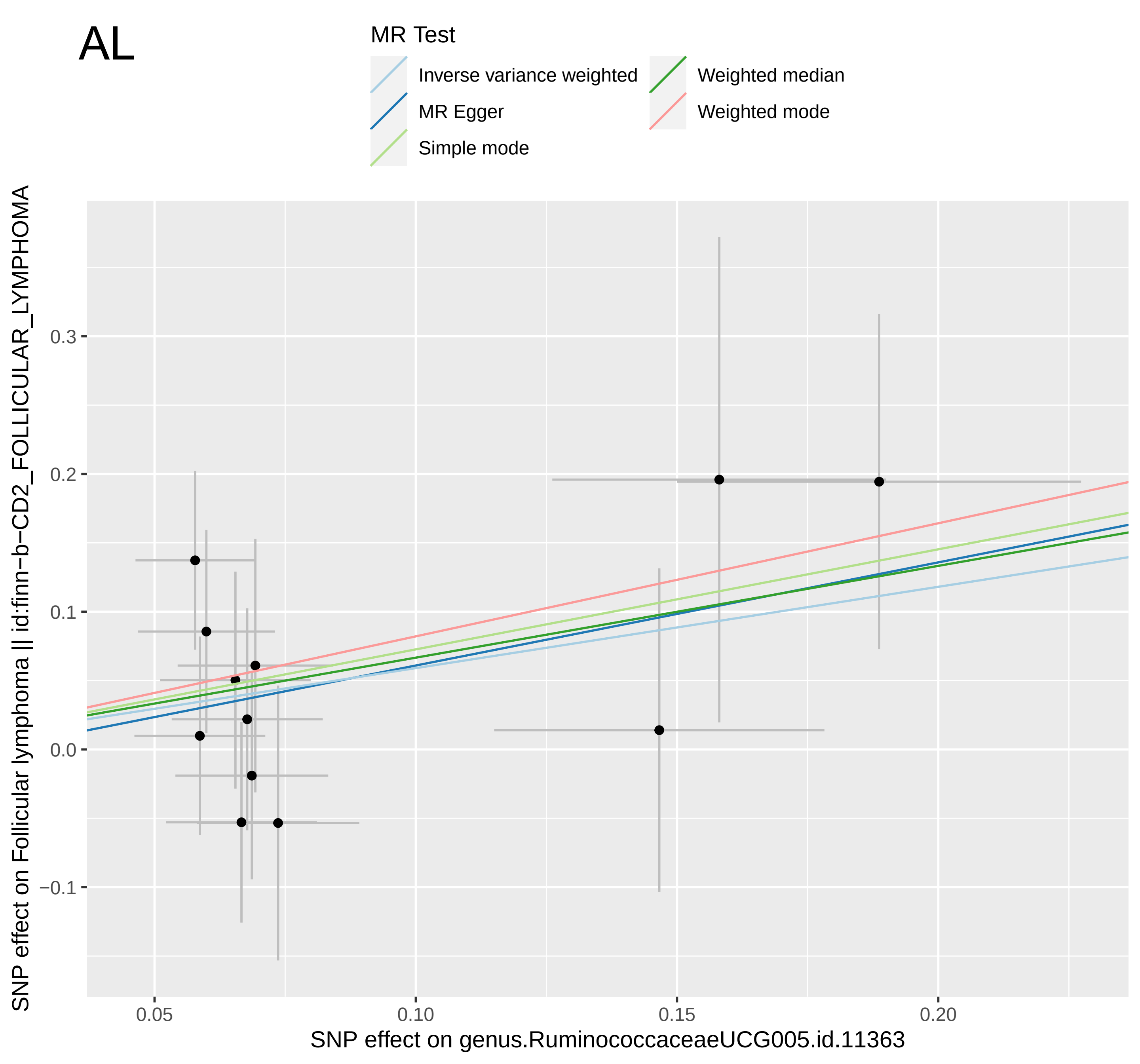
AL
MR Test
Inverse variance weighted
MR Egger
Simple mode
Weighted median
Weighted mode
SNP effect on Follicular lymphoma || id:finn-b-CD2_FOLLICULAR_LYMPHOMA
0.3
0.2
0.1
0.0
−0.1
0.05
0.10
0.15
0.20
SNP effect on genus.RuminococcaceaeUCG005.id.11363

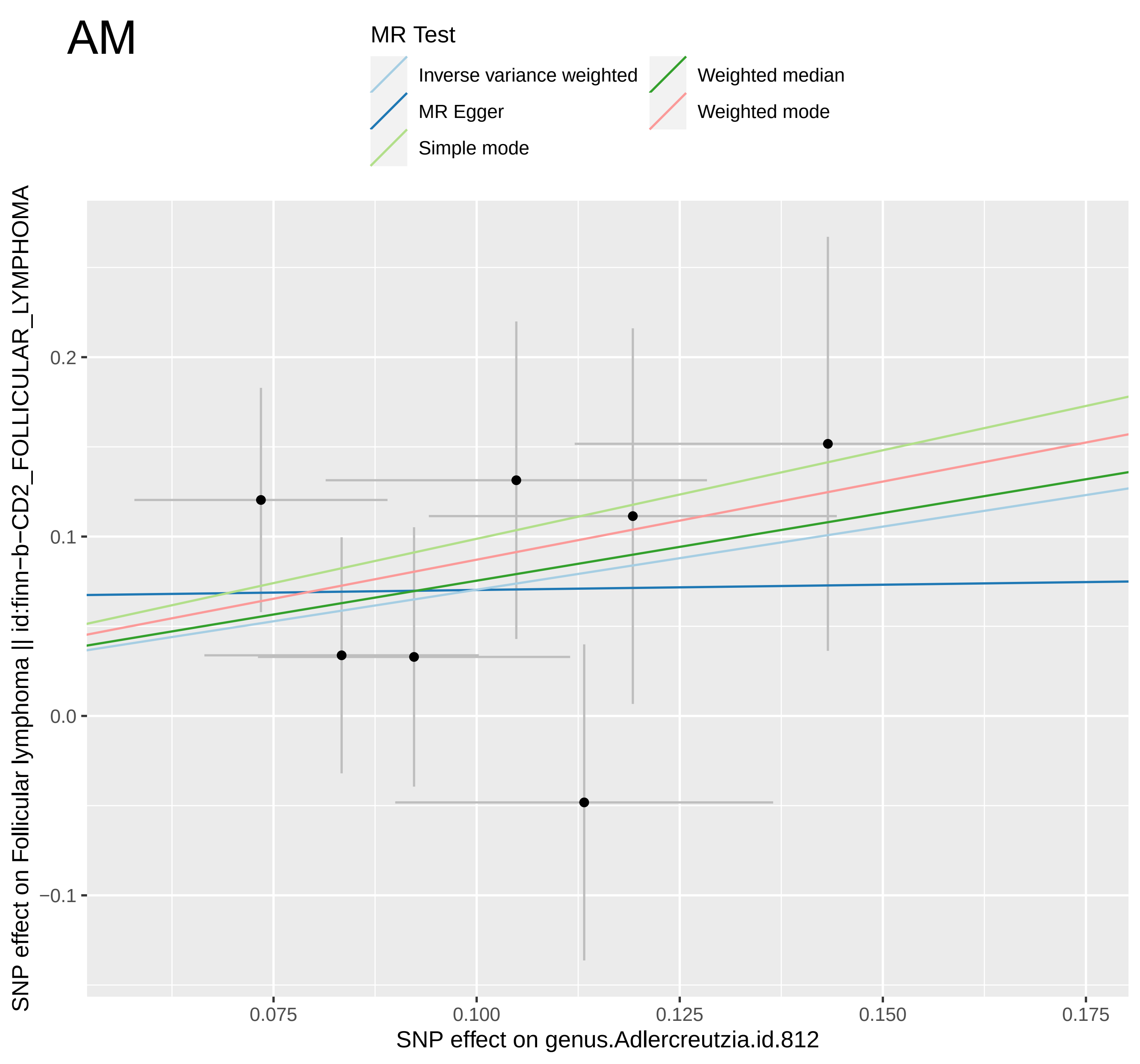

AM
MR Test
Inverse variance weighted
MR Egger
Simple mode
Weighted median
Weighted mode
SNP effect on Follicular lymphoma || id:finn−b−CD2_FOLLICULAR_LYMPHOMA
0.2
0.1
0.0
−0.1
0.075
0.100
0.125
0.150
0.175
SNP effect on genus.Adlercreutzia.id.812

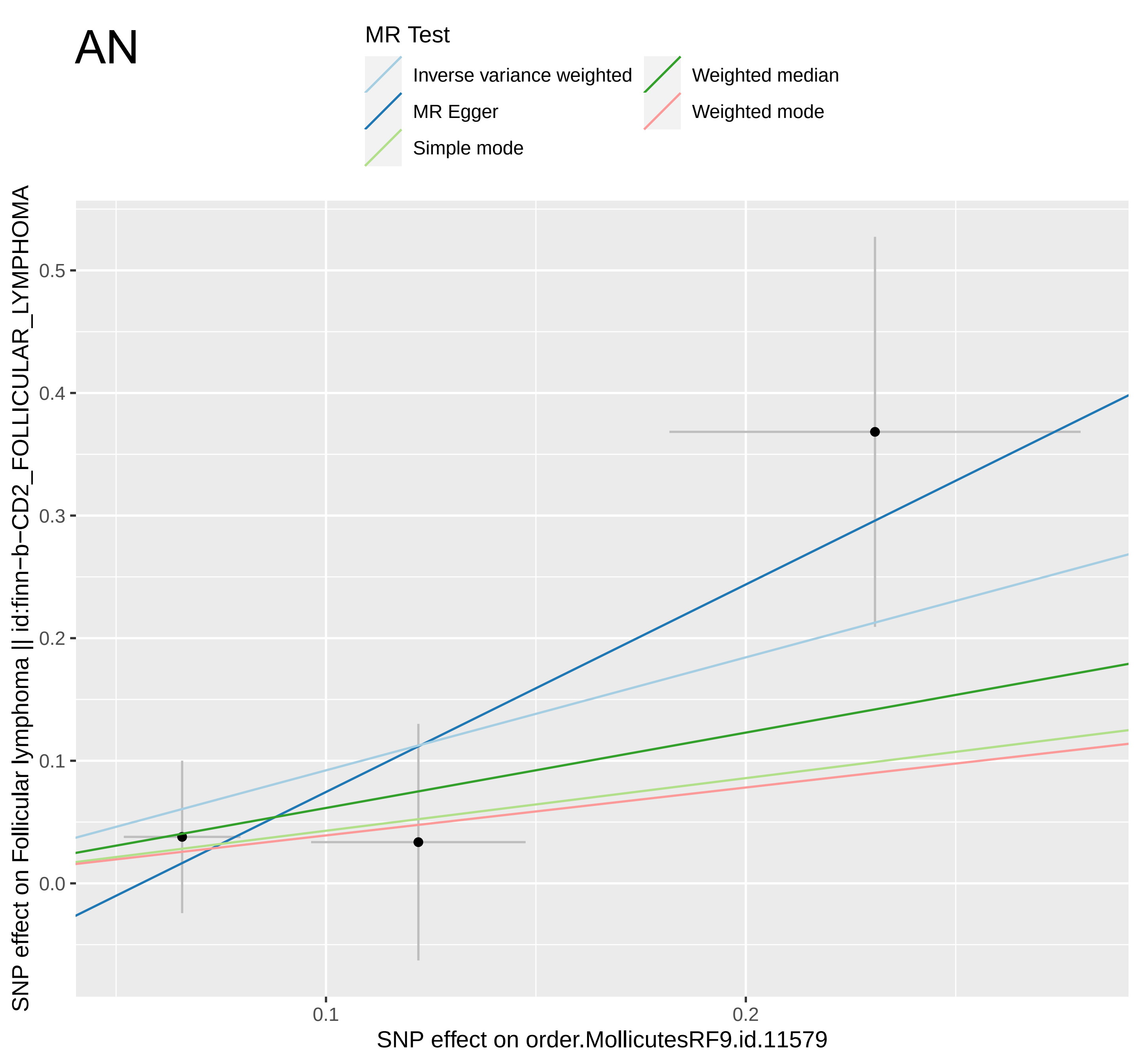

AN
MR Test
Inverse variance weighted
MR Egger
Simple mode
Weighted median
Weighted mode
SNP effect on Follicular lymphoma || id:finn-b-CD2_FOLLICULAR_LYMPHOMA
0.5
0.4
0.3
0.2
0.1
0.0
0.1
0.2
SNP effect on order.MollicutesRF9.id.11579

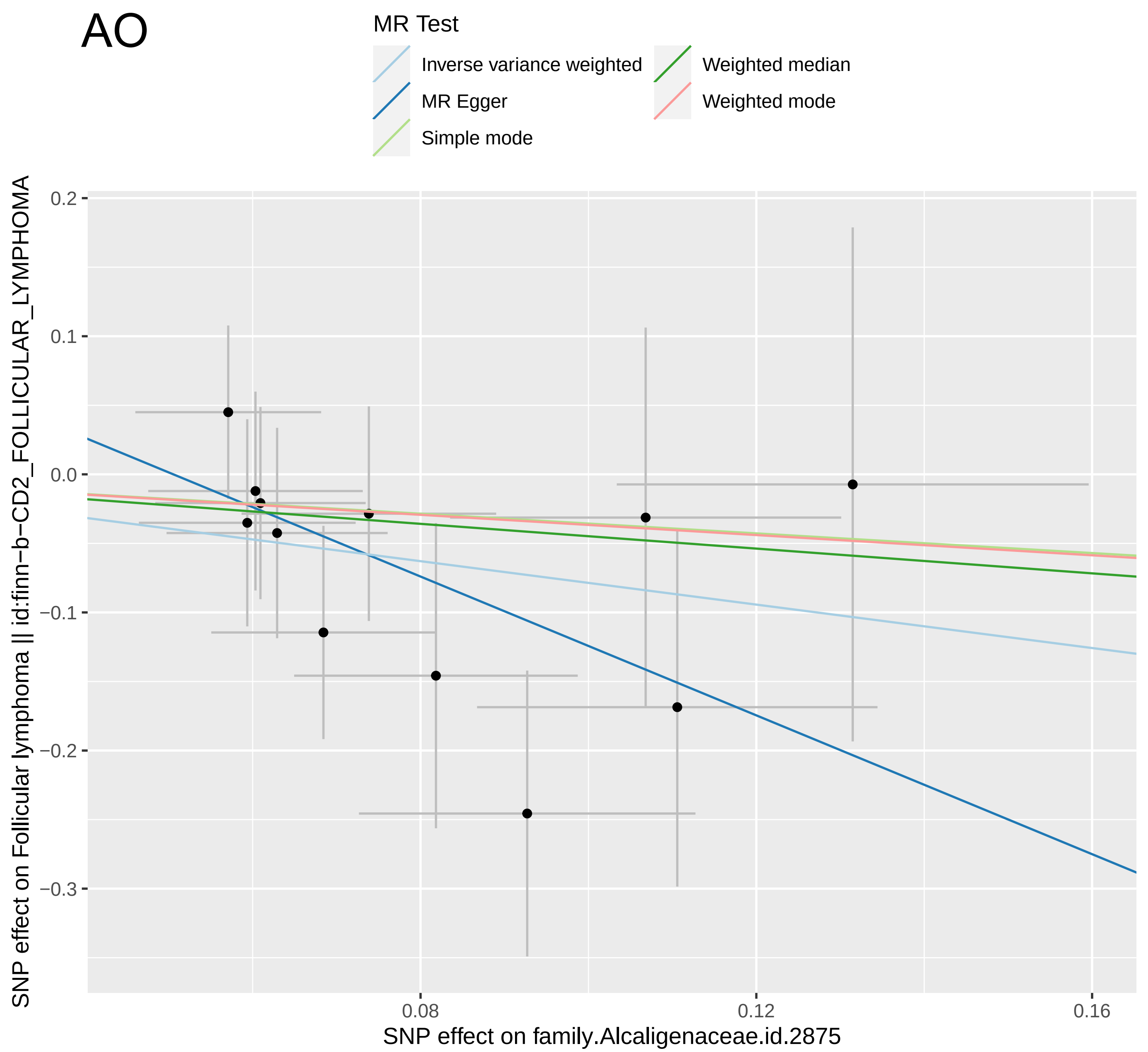

AO
MR Test
Inverse variance weighted
MR Egger
Simple mode
Weighted median
Weighted mode
0.2
0.1
0.0
−0.1
−0.2
−0.3
SNP effect on Follicular lymphoma || id:finn−b−CD2_FOLLICULAR_LYMPHOMA
0.08
0.12
0.16
SNP effect on family.Alcaligenaceae.id.2875

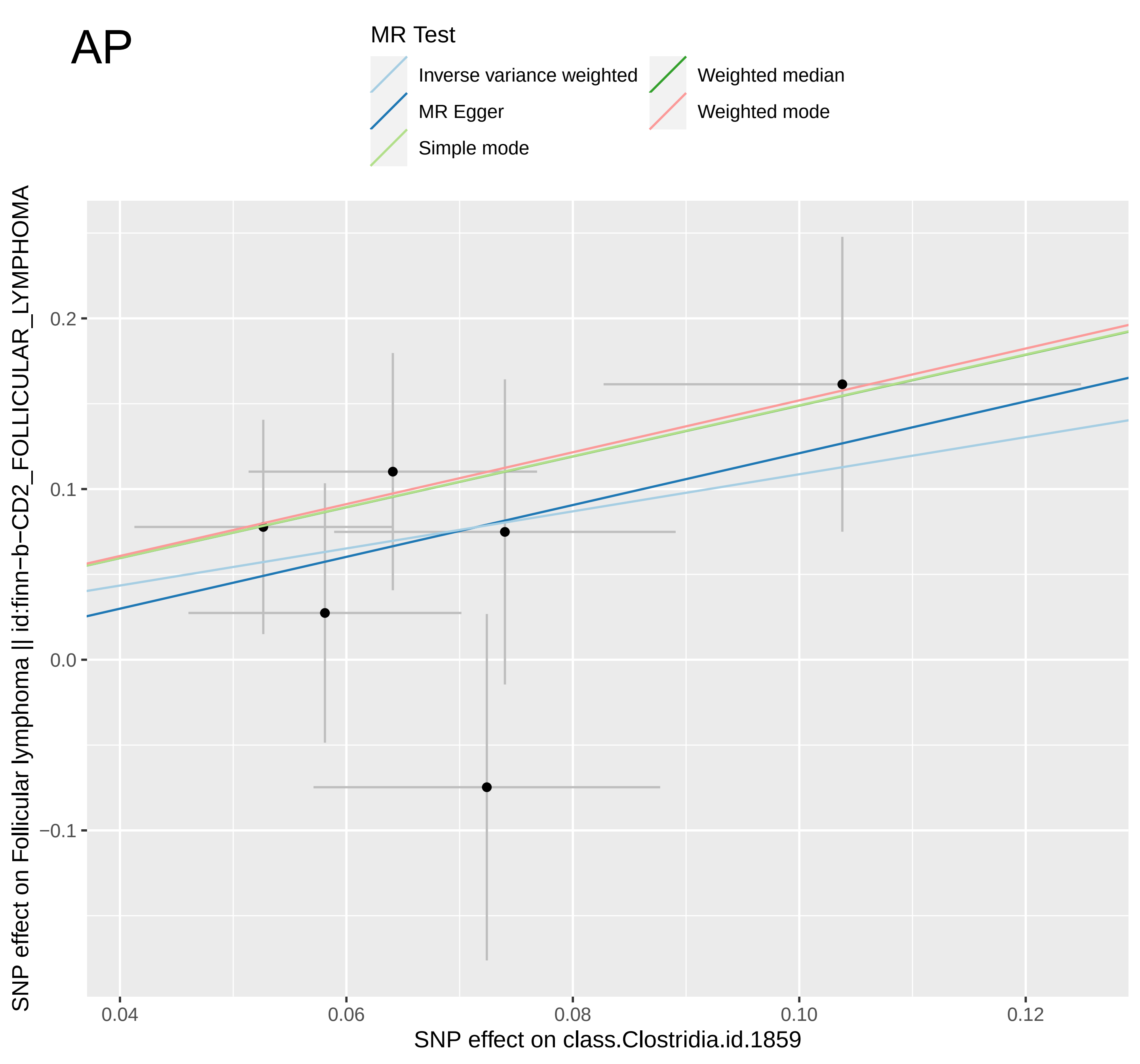
AP
MR Test
Inverse variance weighted
MR Egger
Simple mode
Weighted median
Weighted mode
SNP effect on Follicular lymphoma || id:finn-b-CD2_FOLLICULAR_LYMPHOMA
0.2
0.1
0.0
−0.1
0.04
0.06
0.08
0.10
0.12
SNP effect on class.Clostridia.id.1859

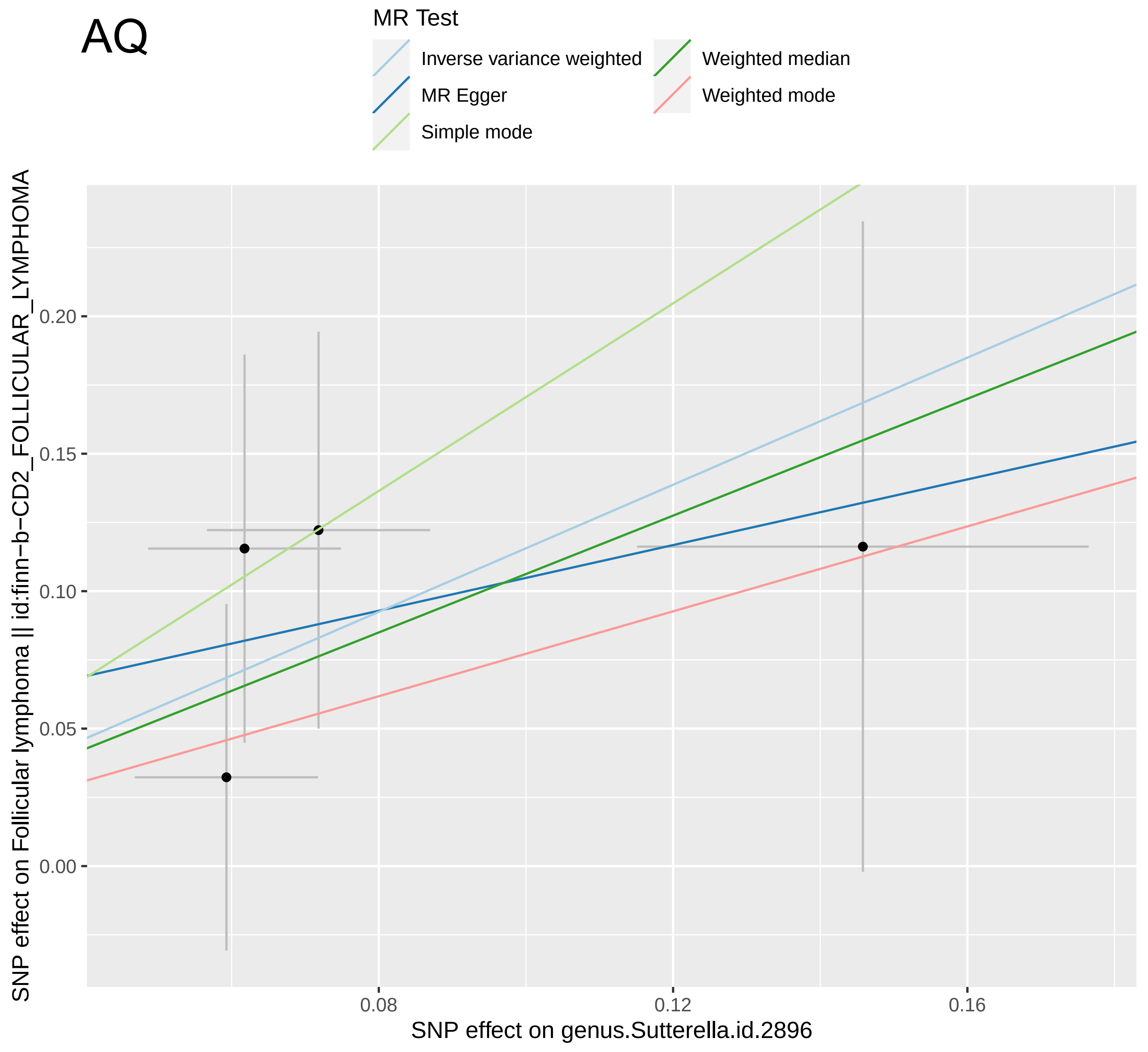
AQ
MR Test
Inverse variance weighted
MR Egger
Simple mode
Weighted median
Weighted mode
SNP effect on Follicular lymphoma || id:finn-b-CD2_FOLLICULAR_LYMPHOMA
0.20
0.15
0.10
0.05
0.00
0.08
0.12
0.16
SNP effect on genus.Sutterella.id.2896

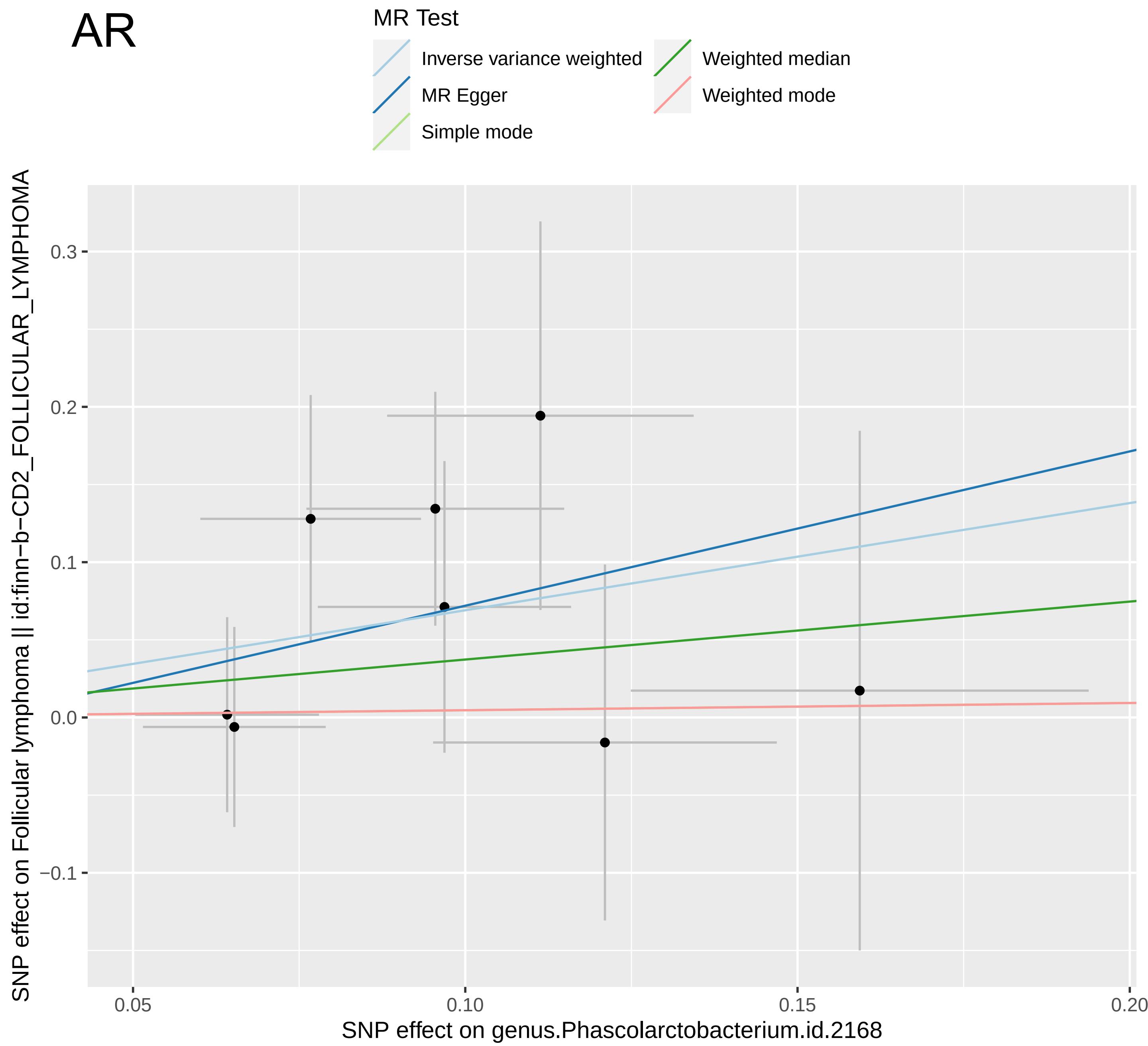
AR
MR Test
Inverse variance weighted
MR Egger
Simple mode
Weighted median
Weighted mode
SNP effect on Follicular lymphoma || id:finn-b-CD2_FOLLICULAR_LYMPHOMA
0.3
0.2
0.1
0.0
−0.1
0.05
0.10
0.15
0.20
SNP effect on genus.Phascolarctobacterium.id.2168

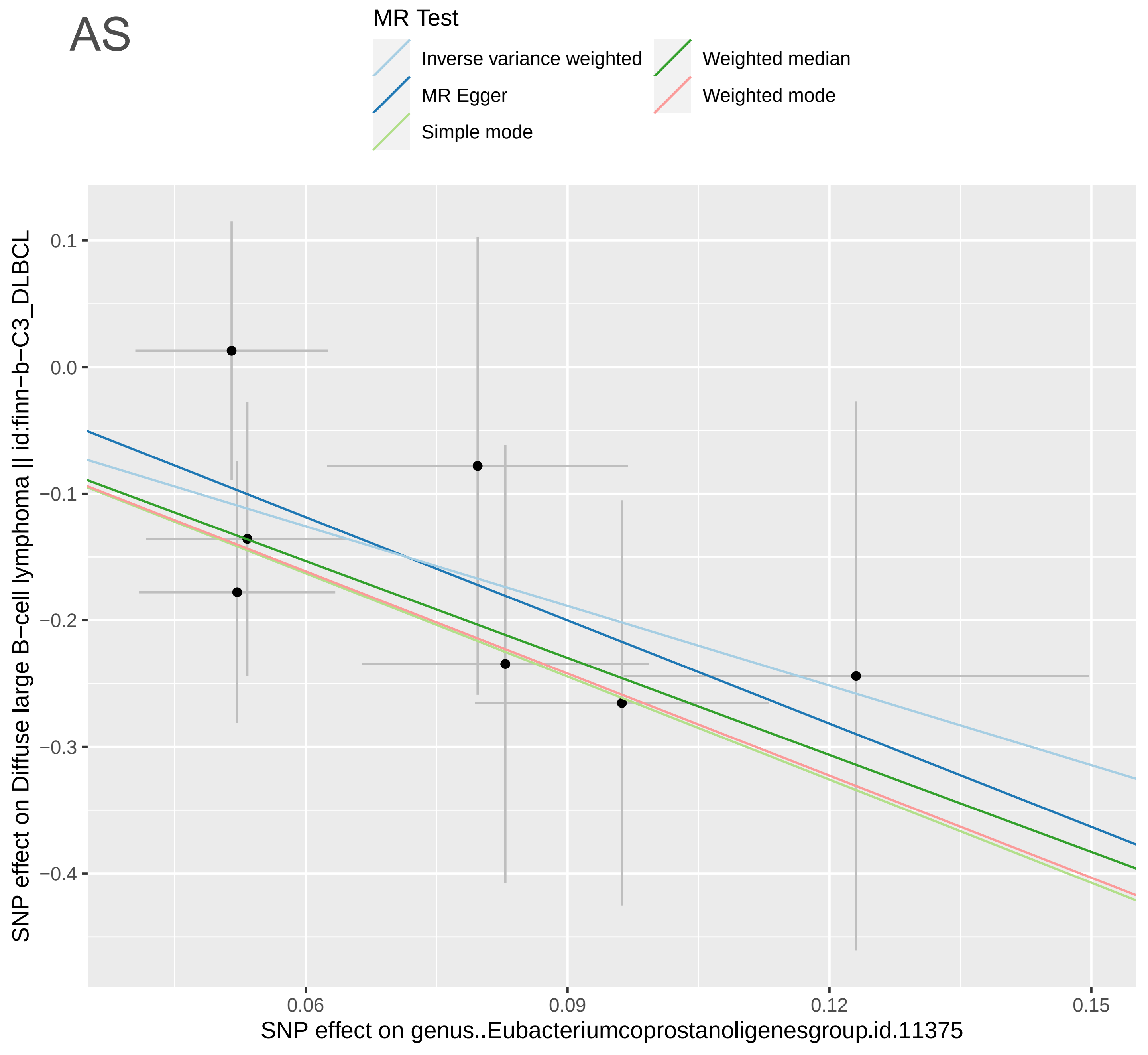
AS
MR Test
Inverse variance weighted
MR Egger
Simple mode
Weighted median
Weighted mode
0.1
0.0
−0.1
−0.2
−0.3
−0.4
SNP effect on Diffuse large B−cell lymphoma || id:finn−b−C3_DLBCL
0.06
0.09
0.12
0.15
SNP effect on genus..Eubacteriumcoprostanoligenesgroup.id.11375

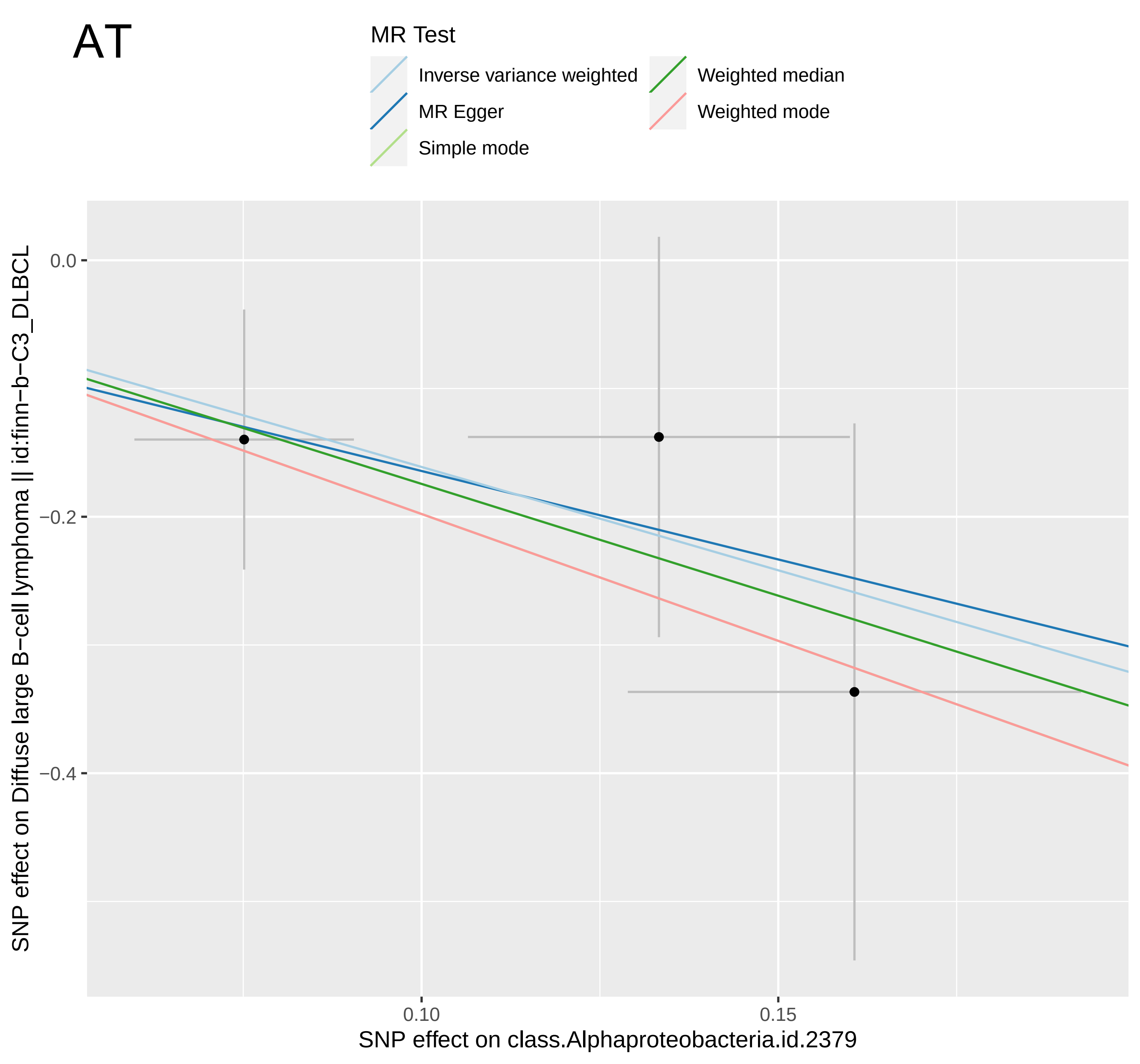
AT
MR Test
Inverse variance weighted
MR Egger
Simple mode
Weighted median
Weighted mode
0.0
−0.2
−0.4
0.10
0.15
SNP effect on Diffuse large B−cell lymphoma || id:finn−b−C3_DLBCL
SNP effect on class.Alphaproteobacteria.id.2379

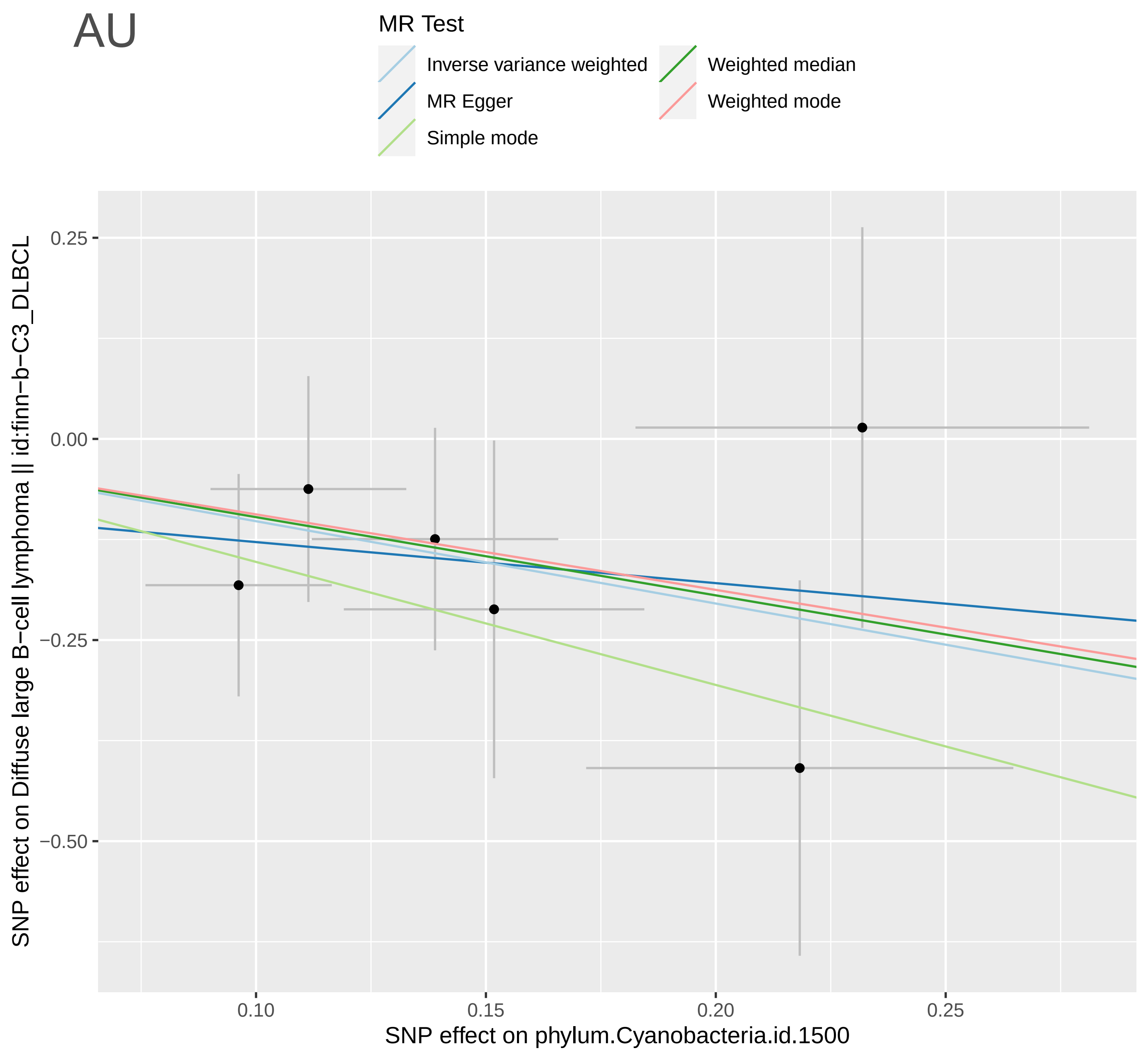
AU
MR Test
Inverse variance weighted
MR Egger
Simple mode
Weighted median
Weighted mode
SNP effect on Diffuse large B−cell lymphoma || id:finn−b−C3_DLBCL
0.25
0.00
−0.25
−0.50
0.10
0.15
0.20
0.25
SNP effect on phylum.Cyanobacteria.id.1500

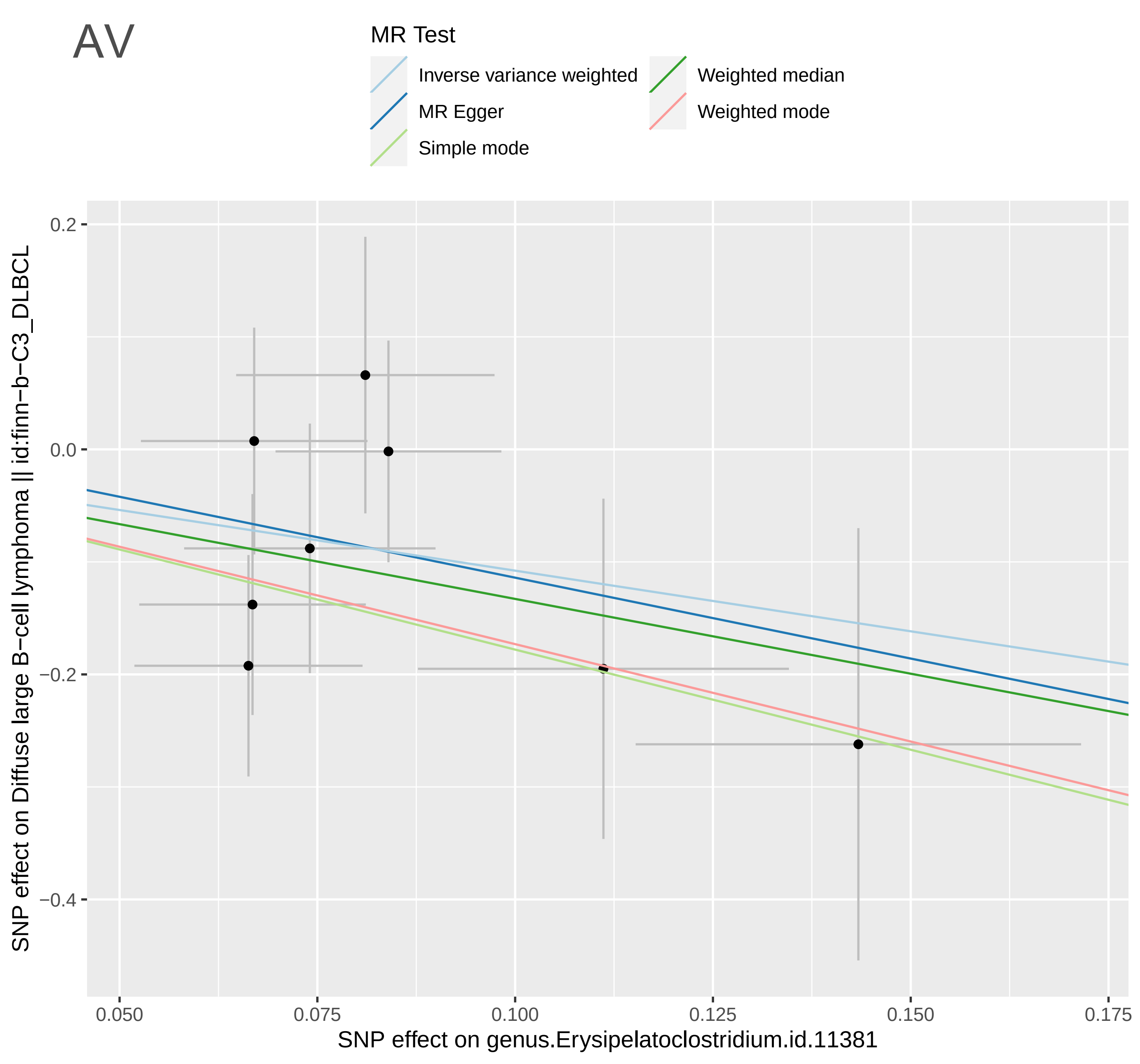
AV
MR Test
Inverse variance weighted
MR Egger
Simple mode
Weighted median
Weighted mode
SNP effect on Diffuse large B−cell lymphoma || id:finn−b−C3_DLBCL
0.2
0.0
−0.2
−0.4
0.050
0.075
0.100
0.125
0.150
0.175
SNP effect on genus.Erysipelatoclostridium.id.11381

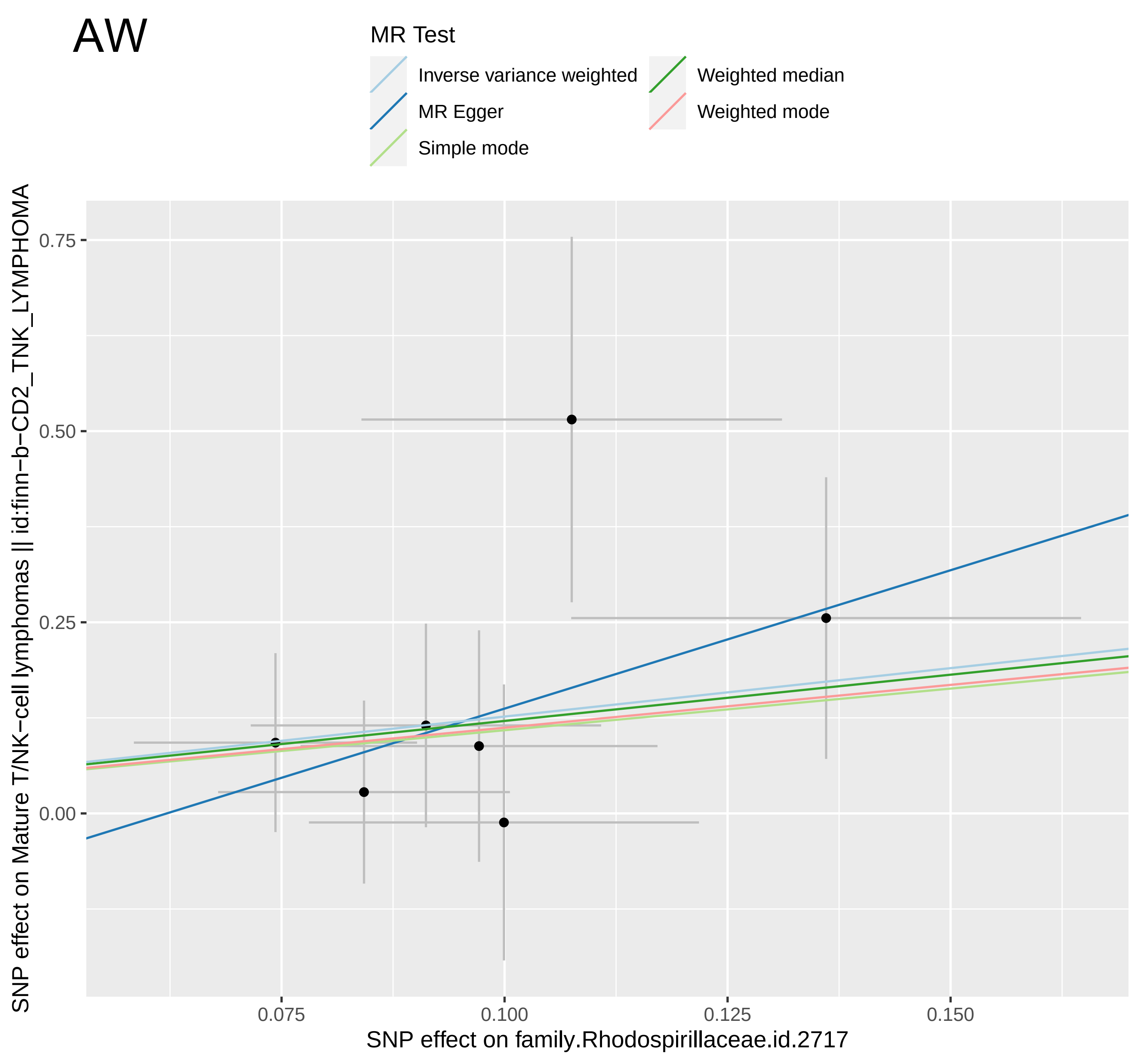
AW
MR Test
Inverse variance weighted
MR Egger
Simple mode
Weighted median
Weighted mode
SNP effect on Mature T/NK−cell lymphomas || id:finn−b−CD2_TNK_LYMPHOMA
0.75
0.50
0.25
0.00
0.075
0.100
0.125
0.150
SNP effect on family.Rhodospirillaceae.id.2717

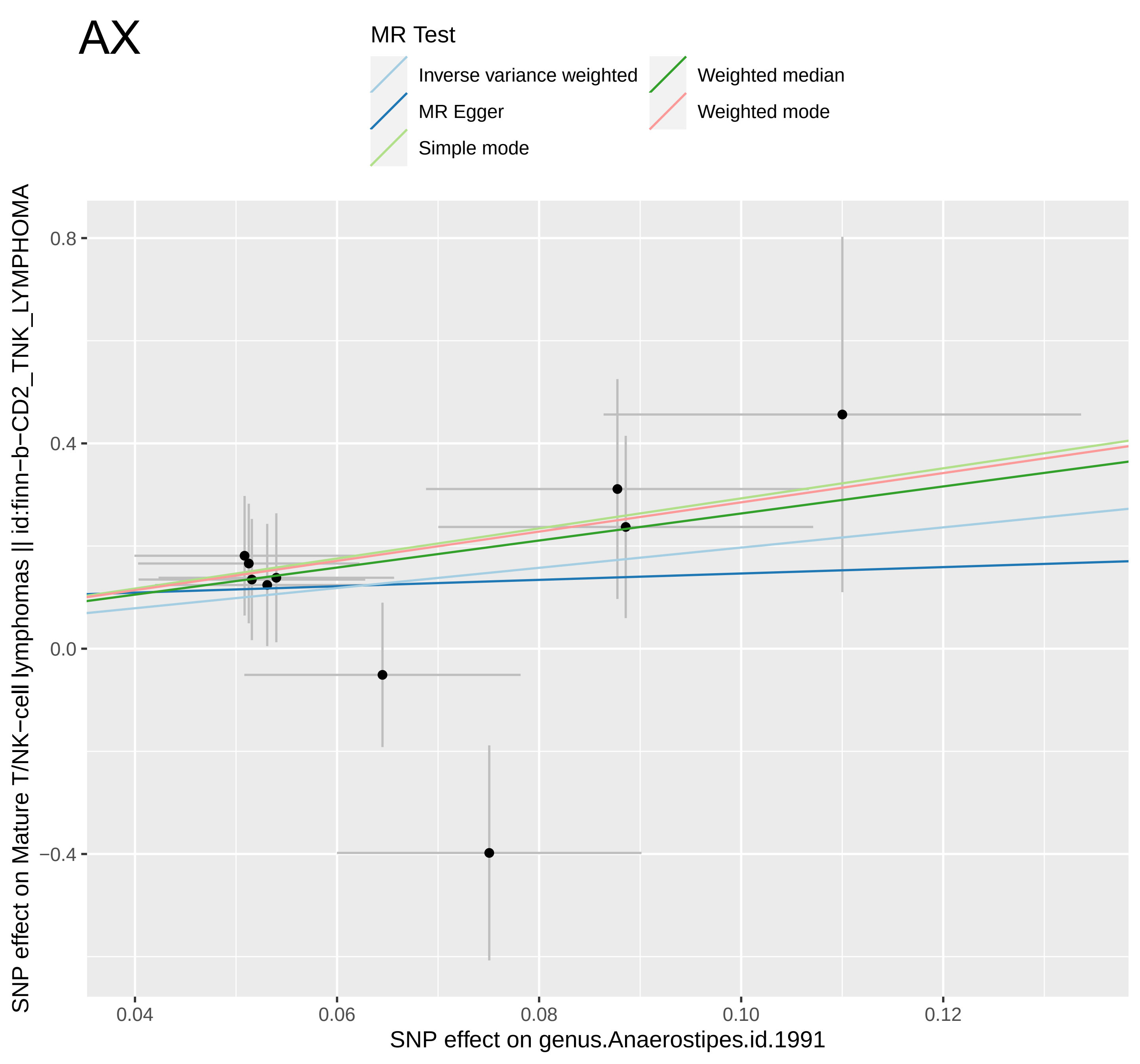
AX
MR Test
Inverse variance weighted
MR Egger
Simple mode
Weighted median
Weighted mode
0.8
0.4
0.0
−0.4
0.04
0.06
0.08
0.10
0.12
SNP effect on Mature T/NK−cell lymphomas || id:finn−b−CD2_TNK_LYMPHOMA
SNP effect on genus.Anaerostipes.id.1991

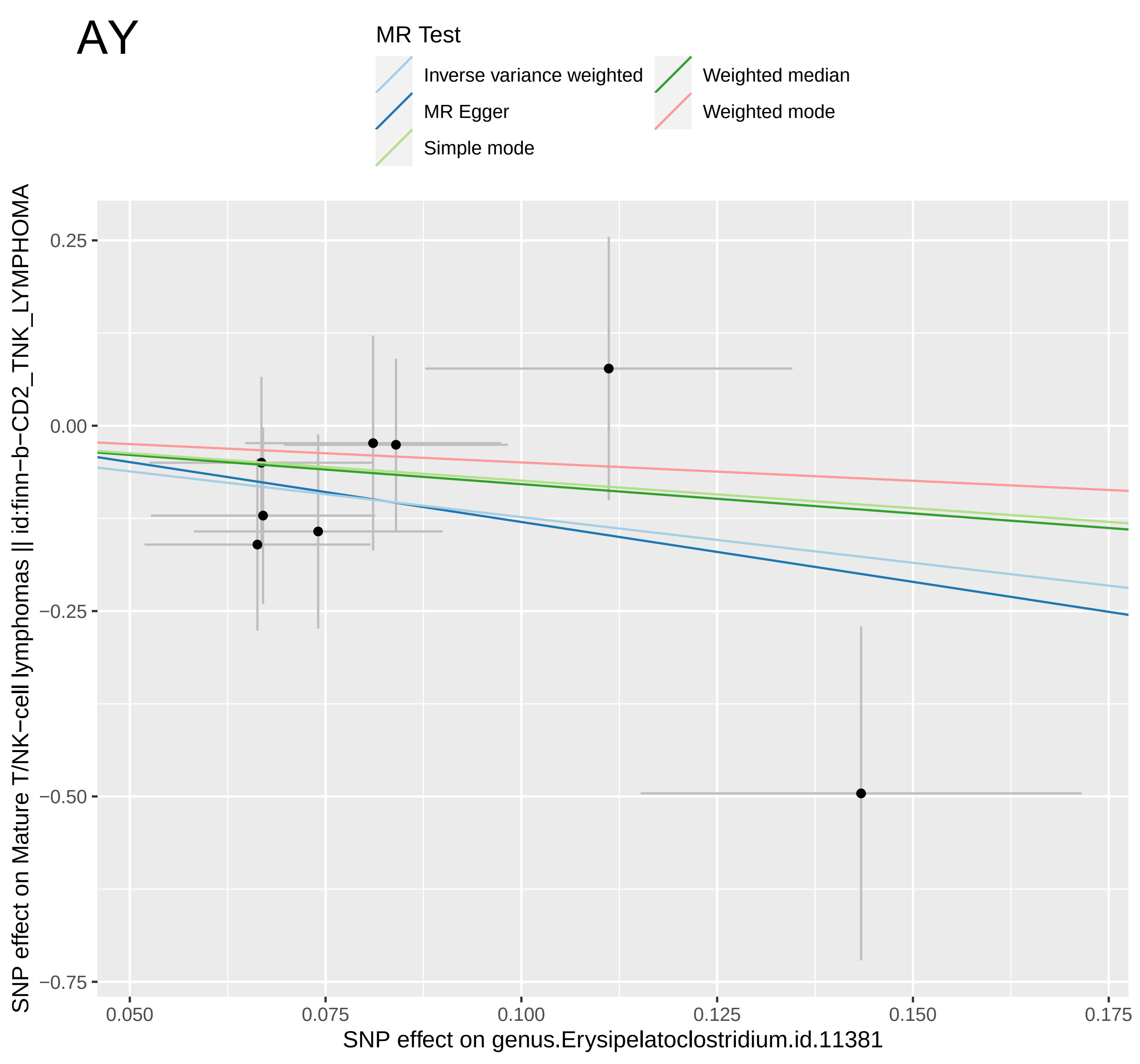
AY
MR Test
Inverse variance weighted
MR Egger
Simple mode
Weighted median
Weighted mode
SNP effect on Mature T/NK−cell lymphomas || id:finn−b−CD2_TNK_LYMPHOMA
0.25
0.00
−0.25
−0.50
−0.75
0.050
0.075
0.100
0.125
0.150
0.175
SNP effect on genus.Erysipelatoclostridium.id.11381

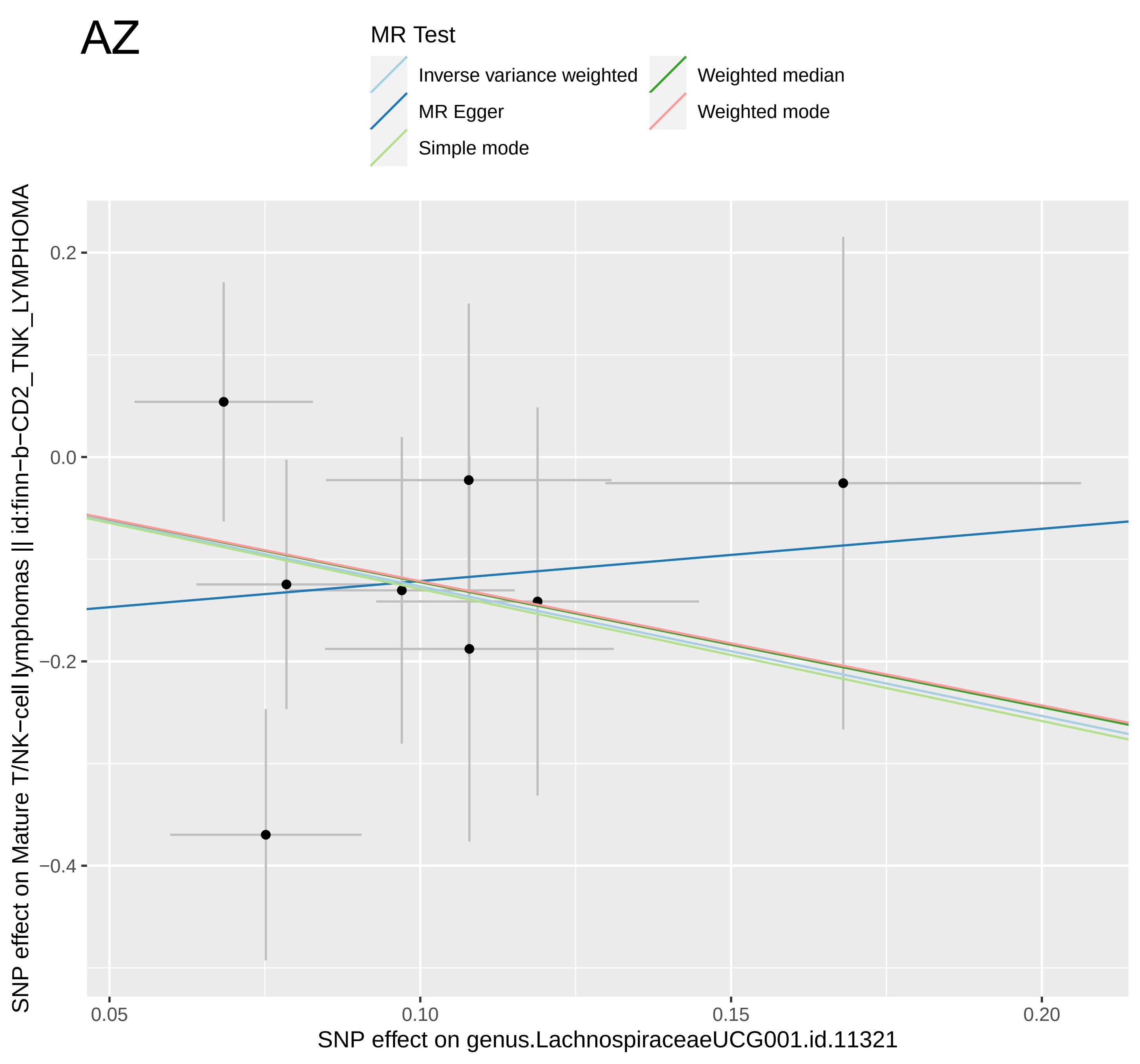

AZ
MR Test
Inverse variance weighted
Weighted median
MR Egger
Weighted mode
Simple mode
SNP effect on Mature T/NK−cell lymphomas || id:finn−b−CD2_TNK_LYMPHOMA
0.2
0.0
−0.2
−0.4
0.05
0.10
0.15
0.20
SNP effect on genus.LachnospiraceaeUCG001.id.11321

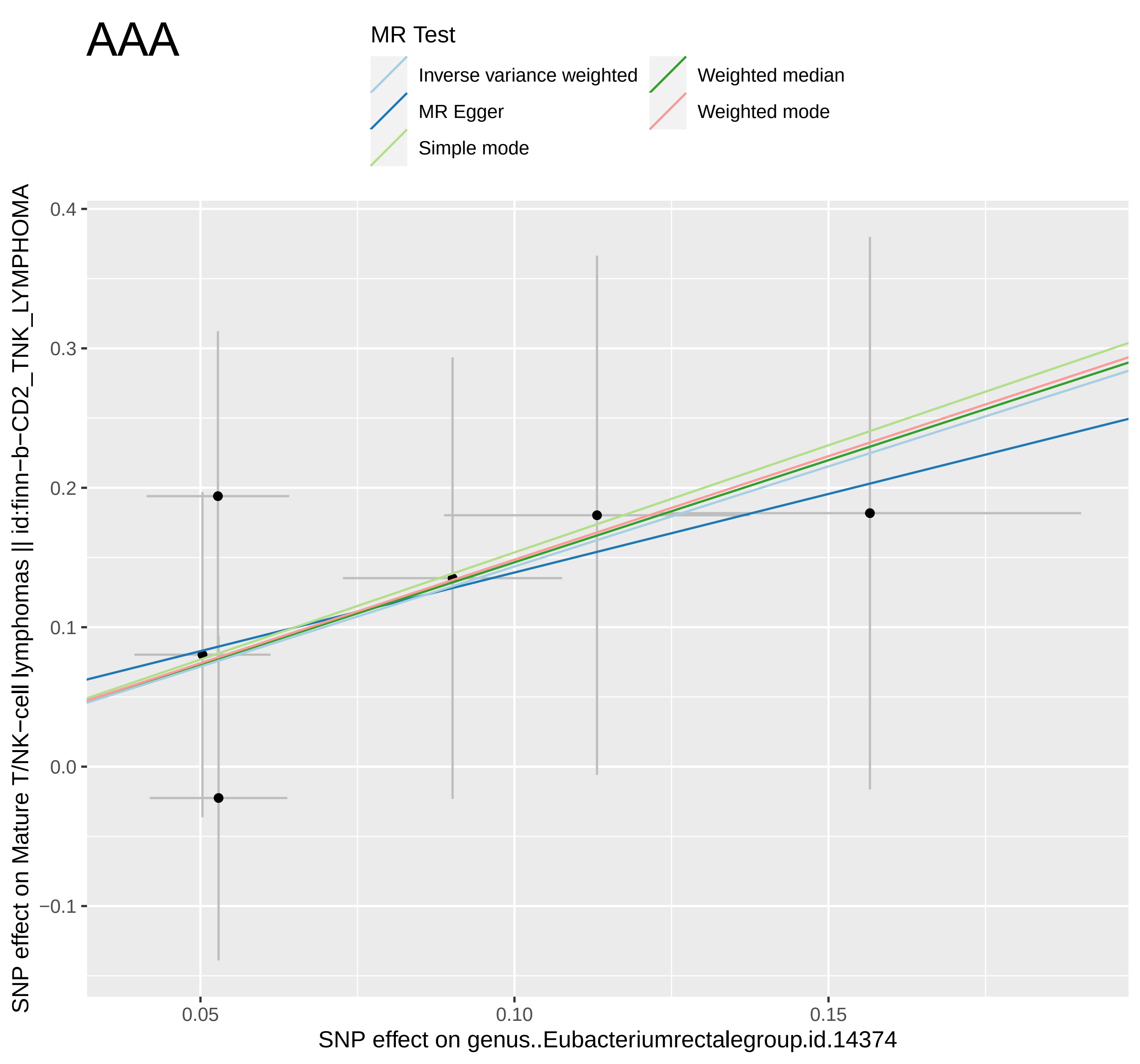

AAA
MR Test
Inverse variance weighted
MR Egger
Simple mode
Weighted median
Weighted mode
SNP effect on Mature T/NK−cell lymphomas || id:finn-b-CD2_TNK_LYMPHOMA
SNP effect on genus..Eubacteriumrectalegroup.id.14374
0.4
0.3
0.2
0.1
0.0
−0.1
0.05
0.10
0.15

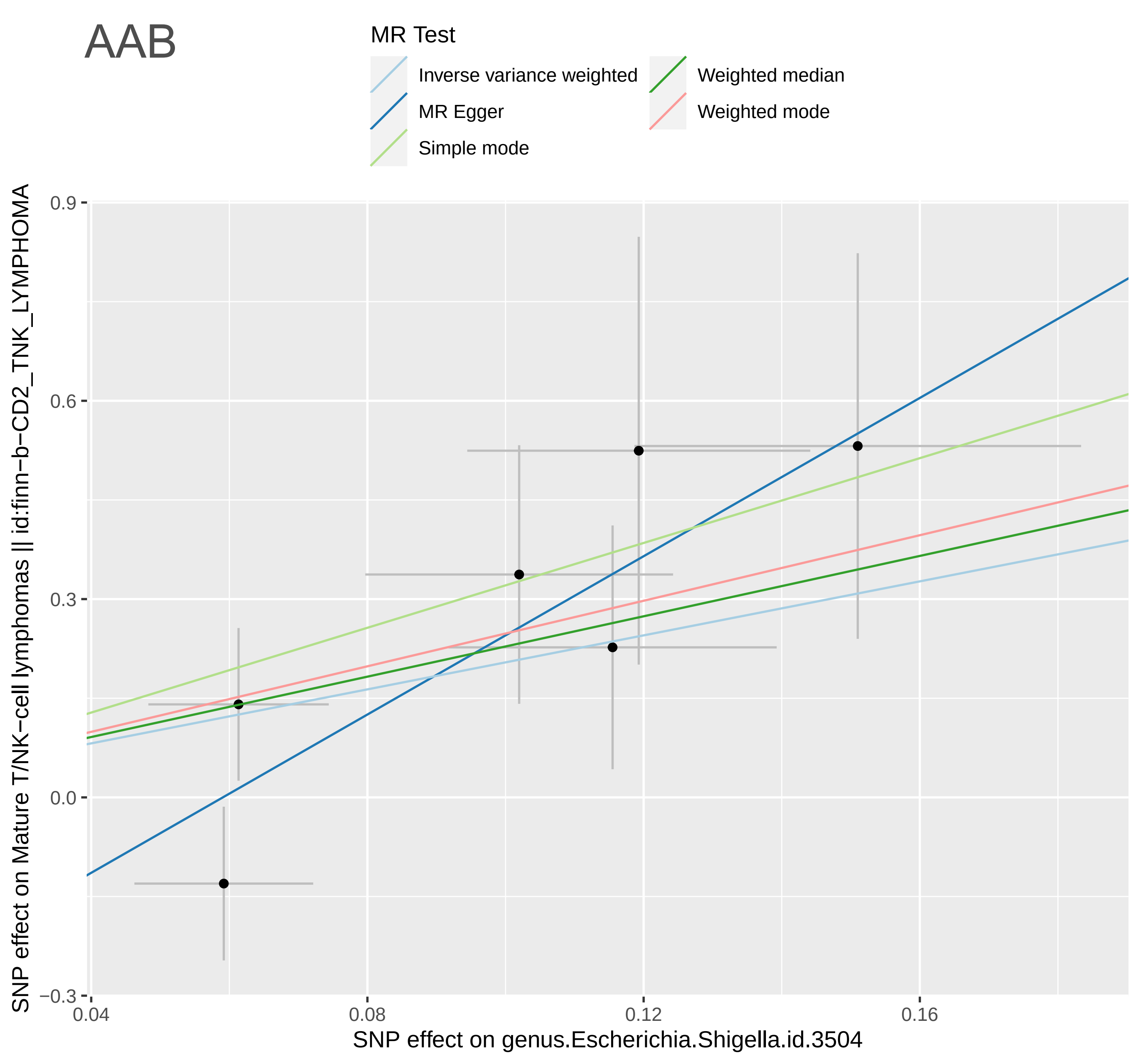

AAB
MR Test
Inverse variance weighted
MR Egger
Simple mode
Weighted median
Weighted mode
SNP effect on Mature T/NK−cell lymphomas || id:finn−b−CD2_TNK_LYMPHOMA
SNP effect on genus.Escherichia.Shigella.id.3504
0.9
0.6
0.3
0.0
−0.3
0.04
0.08
0.12
0.16

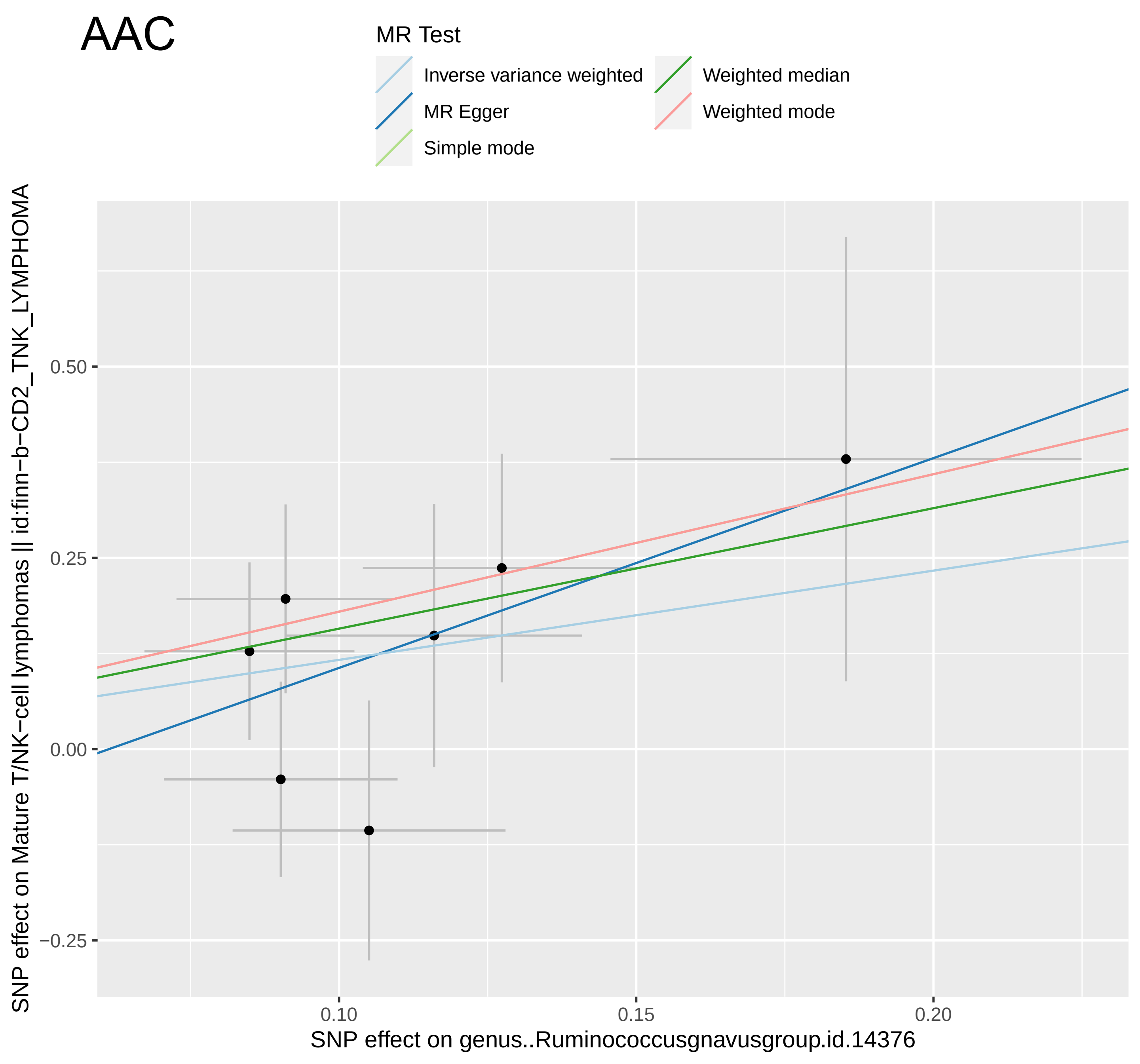

AAC
MR Test
Inverse variance weighted
MR Egger
Simple mode
Weighted median
Weighted mode
SNP effect on Mature T/NK−cell lymphomas || id:finn−b−CD2_TNK_LYMPHOMA
0.50
0.25
0.00
−0.25
0.10
0.15
0.20
SNP effect on genus..Ruminococcusgnavusgroup.id.14376

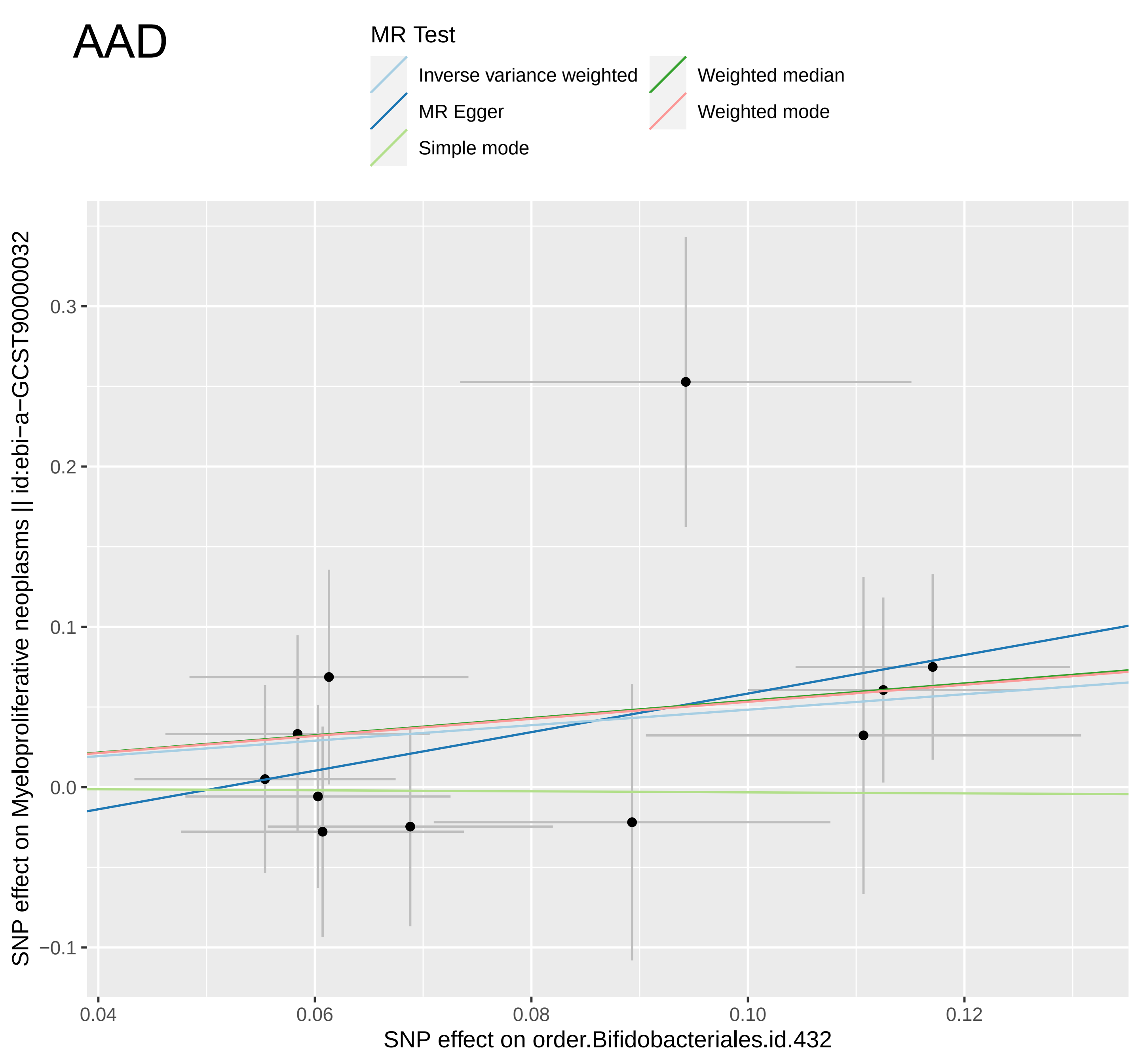

AAD
MR Test
Inverse variance weighted
MR Egger
Simple mode
Weighted median
Weighted mode
SNP effect on Myeloproliferative neoplasms || id:ebi−a−GCST90000032
0.3
0.2
0.1
0.0
−0.1
0.04
0.06
0.08
0.10
0.12
SNP effect on order.Bifidobacteriales.id.432

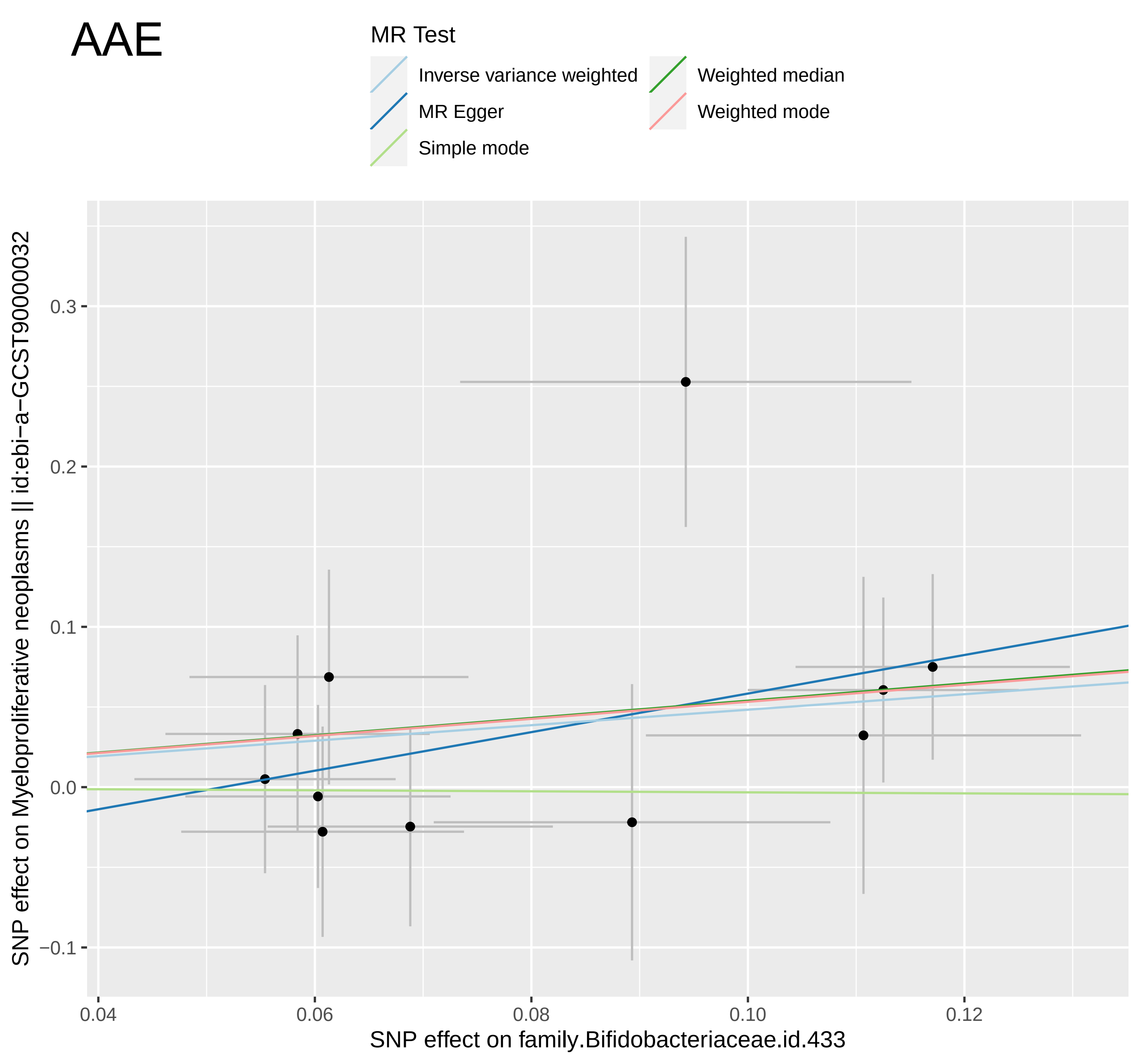
AAE
MR Test
Inverse variance weighted
MR Egger
Simple mode
Weighted median
Weighted mode
SNP effect on Myeloproliferative neoplasms || id:ebi−a−GCST90000032
0.3
0.2
0.1
0.0
−0.1
0.04
0.06
0.08
0.10
0.12
SNP effect on family.Bifidobacteriaceae.id.433

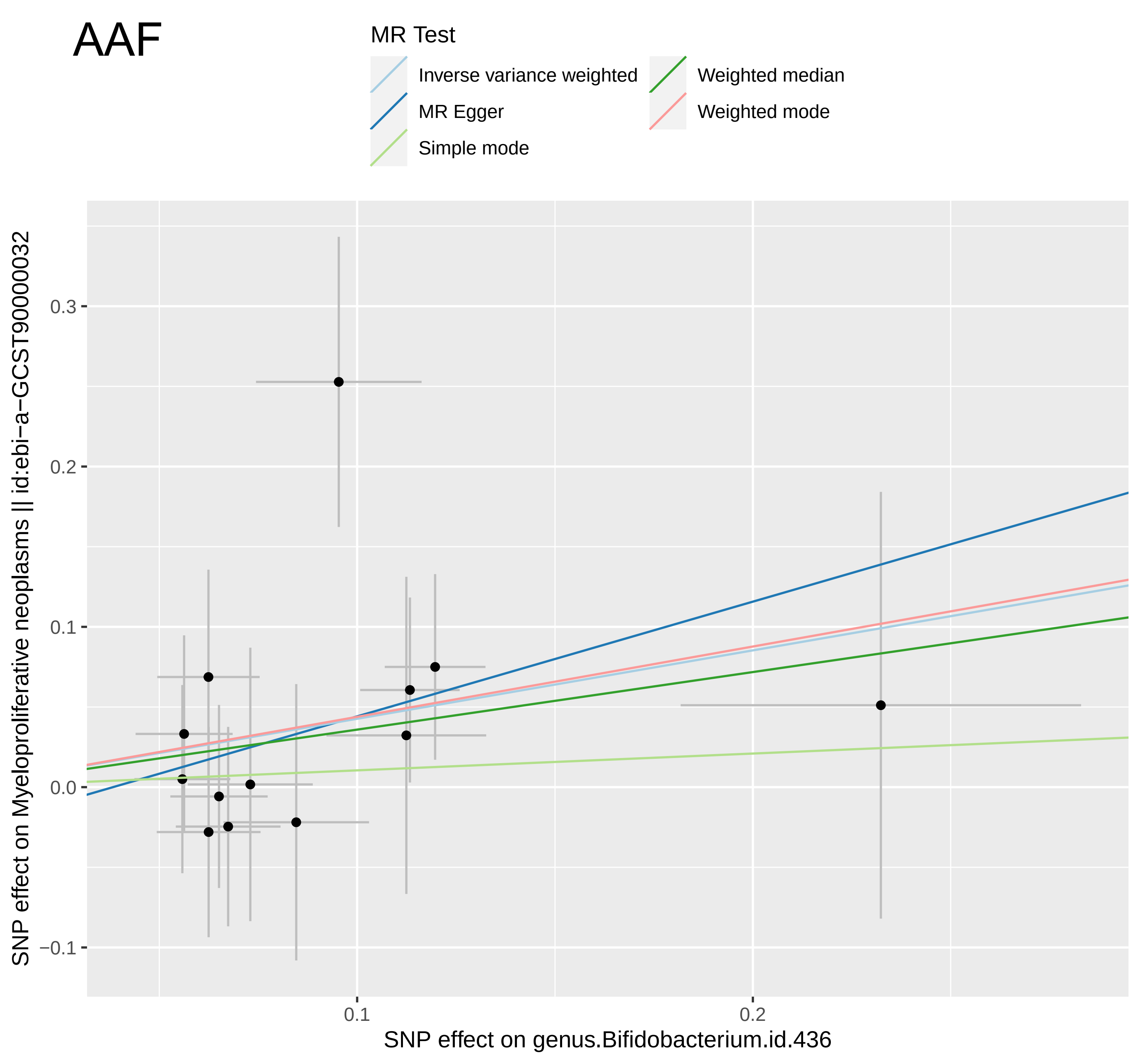
AAF
MR Test
Inverse variance weighted
MR Egger
Simple mode
Weighted median
Weighted mode
0.3
0.2
0.1
0.0
−0.1
0.1
0.2
SNP effect on Myeloproliferative neoplasms || id:ebi−a−GCST90000032
SNP effect on genus.Bifidobacterium.id.436

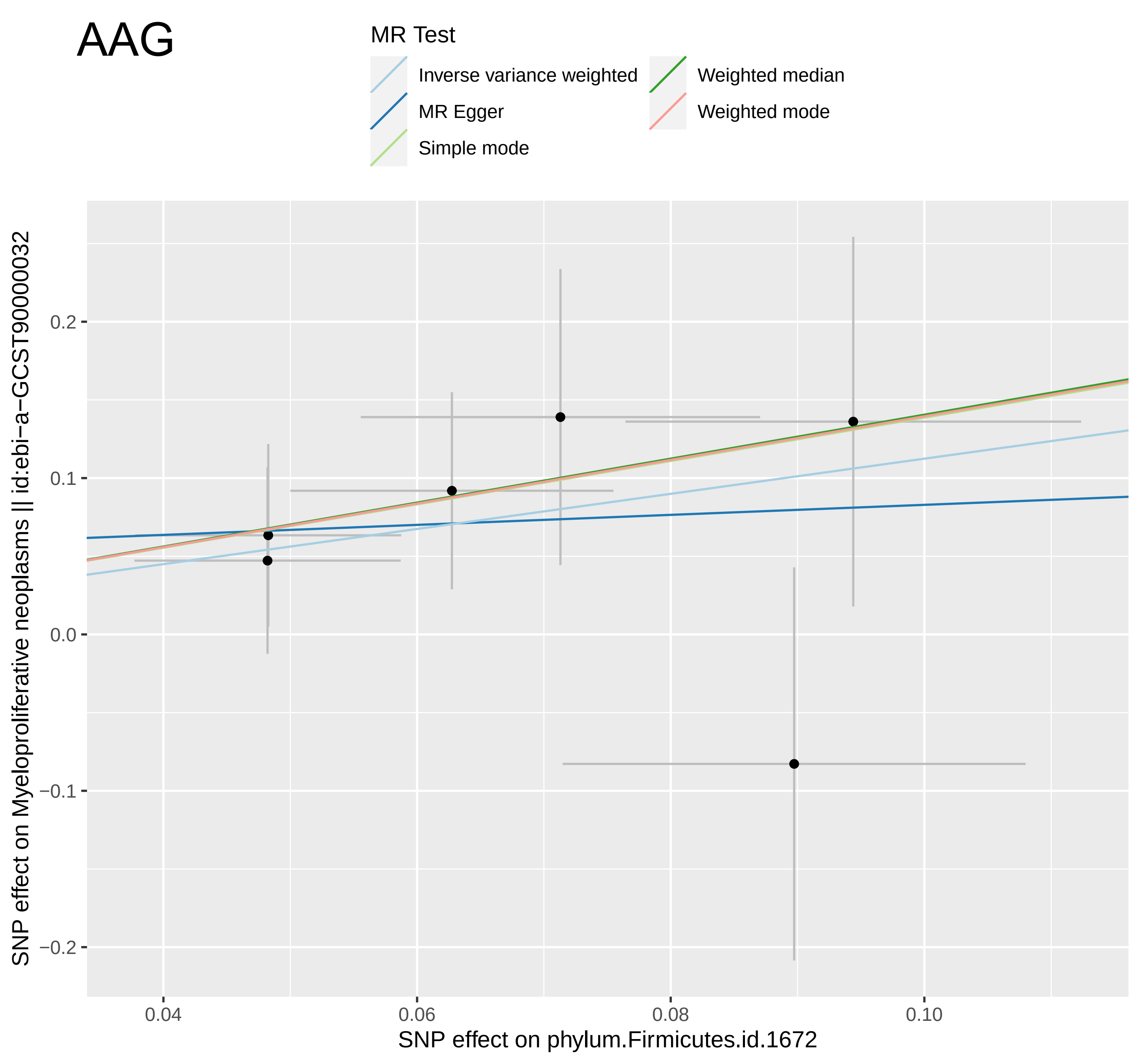
AAG
MR Test
Inverse variance weighted
MR Egger
Simple mode
Weighted median
Weighted mode
SNP effect on Myeloproliferative neoplasms || id:ebi−a−GCST90000032
0.2
0.1
0.0
−0.1
−0.2
0.04
0.06
0.08
0.10
SNP effect on phylum.Firmicutes.id.1672

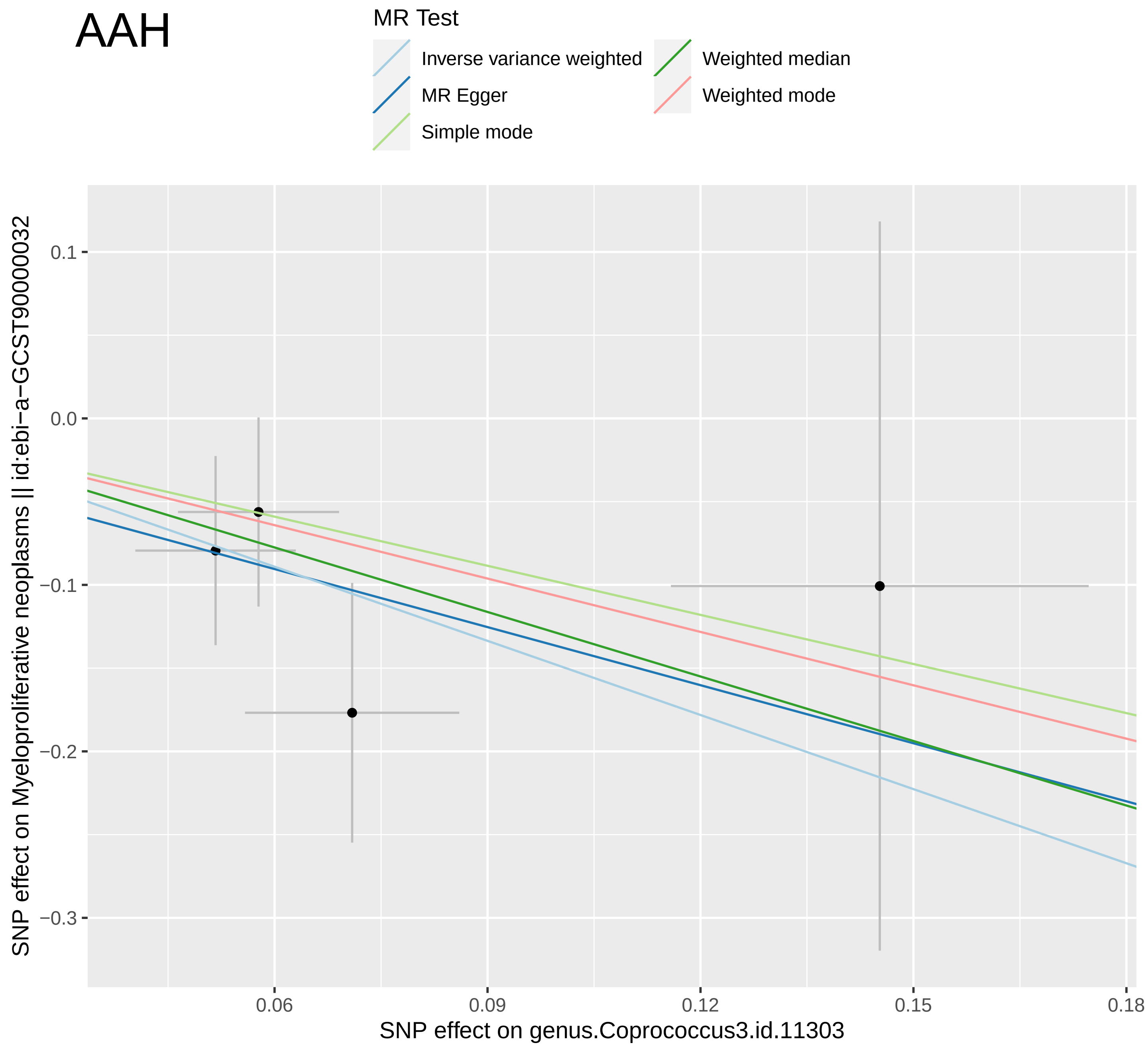
AAH
MR Test
Inverse variance weighted
MR Egger
Simple mode
Weighted median
Weighted mode
SNP effect on Myeloproliferative neoplasms || id:ebi−a−GCST90000032
0.1
0.0
−0.1
−0.2
−0.3
0.06
0.09
0.12
0.15
0.18
SNP effect on genus.Coprococcus3.id.11303

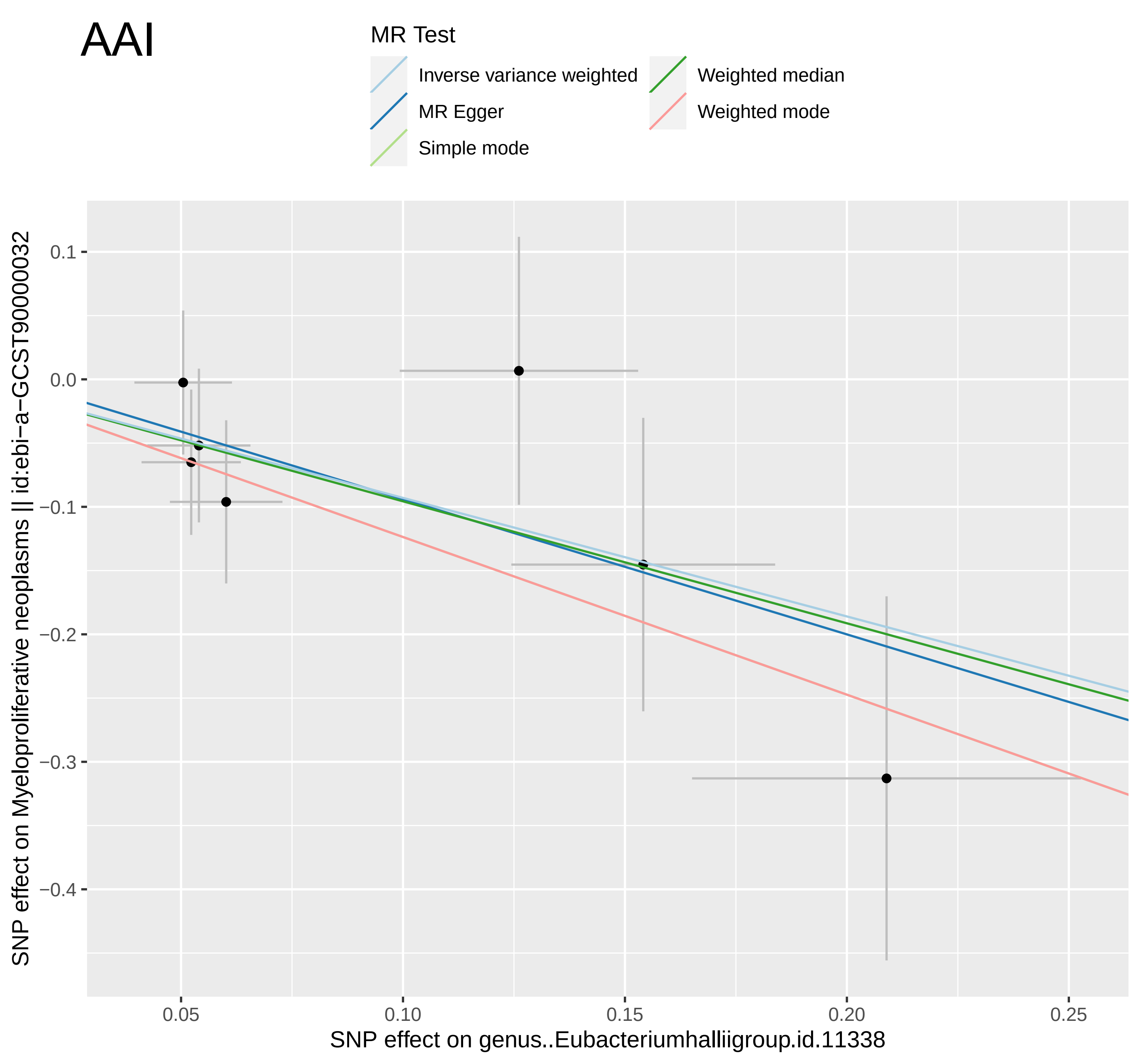

AAI
MR Test
Inverse variance weighted
MR Egger
Simple mode
Weighted median
Weighted mode
SNP effect on Myeloproliferative neoplasms || id:ebi−a−GCST90000032
0.1
0.0
−0.1
−0.2
−0.3
−0.4
0.05
0.10
0.15
0.20
0.25
SNP effect on genus..Eubacteriumhalliigroup.id.11338

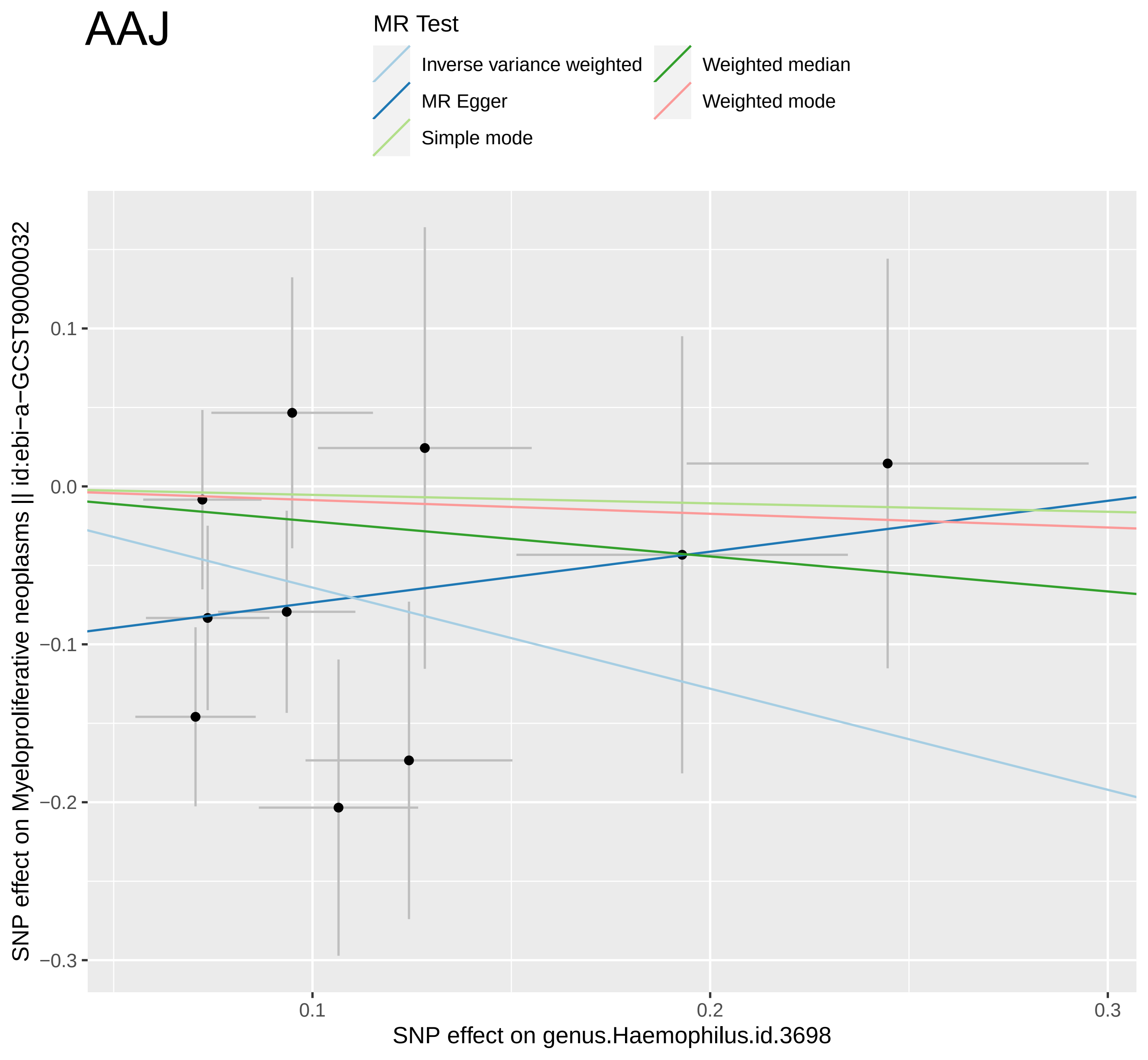

AAJ
MR Test
Inverse variance weighted
MR Egger
Simple mode
Weighted median
Weighted mode
0.1
0.0
−0.1
−0.2
−0.3
0.1
0.2
0.3
SNP effect on Myeloproliferative neoplasms || id:ebi−a−GCST90000032
SNP effect on genus.Haemophilus.id.3698

Supplementary figure 4.Sensitivity analysis of the impact of gut microbiota on hematologic malignancies using MR. (scatter plot)

A.Scatter plot between genus.FamilyXIIIUCG001 and lymphoid leukemia

B.Scatter plot between family.Peptococcaceae and lymphoid leukemia**.**

C.Scatter plot between family.Desulfovibrionaceae and lymphoid leukemia

D.Scatter plot between genus..Clostridiuminnocuumgroup and lymphoid leukemia.

E.Scatter plot between genus.Holdemania and lymphoid leukemia.

F.Scatter plot between family.Prevotellaceae and lymphoid leukemia.

G.Scatter plot between genus.Ruminiclostridium9. and lymphoid leukemia.

H.Scatter plot between genus.Methanobrevibacter and lymphoid leukemia.

I.Scatter plot between order.Desulfovibrionales. and lymphoid leukemia.

J.Scatter plot between genus.Coprococcus3 and lymphoid leukemia.

K.Scatter plot between genus.Ruminiclostridium6 and lymphoid leukemia.

L.Scatter plot between class.Negativicutes and lymphoid leukemia.

M.Scatter plot between order.Selenomonadales. and lymphoid leukemia.

N.Scatter plot between family.Lactobacillaceae. and lymphoid leukemia.

O.Scatter plot between class.Methanobacteria and lymphoid leukemia.

P.Scatter plot between family.Methanobacteriaceae and lymphoid leukemia.

Q.Scatter plot between order.Methanobacteriales and lymphoid leukemia.

R.Scatter plot between phylum.Cyanobacteria and lymphoid leukemia**.**

S.Scatter plot between order.Coriobacteriales and myeloid leukemia**.**

T.Scatter plot between family.Coriobacteriaceae. and myeloid leukemia**.**

U.Scatter plot between genus.LachnospiraceaeUCG008 and myeloid leukemia.

V.Scatter plot between class.Coriobacteriia. and myeloid leukemia**.**

W.Scatter plot between genus.Turicibacter and myeloid leukemia**.**

X.Scatter plot between genus.Slackia. and myeloid leukemia.

Y.Scatter plot between class.Gammaproteobacteria and myeloid leukemia.

Z.Scatter plot between genus.Prevotella9 and myeloid leukemia.

AA. Scatter plot between genus.Dorea and myeloid leukemia.

AB.Scatter plot between genus.Peptococcus. and Hodgkin lymphoma

AC.Scatter plot between class.Gammaproteobacteria and Hodgkin lymphoma.

AD.Scatter plot between genus.RuminococcaceaeUCG014 and malignant plasma cell tumor**.**

AE.Scatter plot between genus.LachnospiraceaeUCG010. and malignant plasma cell tumor.

AF.Scatter plot between class.Lentisphaeria and malignant plasma cell tumor.

AG.Scatter plot between genus.Lactococcus. and malignant plasma cell tumor.

AH.Scatter plot between order.Victivallales. and malignant plasma cell tumor.

AI.Scatter plot between genus.Romboutsia and malignant plasma cell tumor.

AJ.Scatter plot between genus.Dorea and malignant plasma cell tumor.

AK.Scatter plot between genus.RikenellaceaeRC9gutgroup and malignant plasma cell tumor**.**

AL. Scatter plot between genus.RuminococcaceaeUCG005 and follicular lymphoma.

AM.Scatter plot between genus.Adlercreutzia and follicular lymphoma.

AN.Scatter plot between order.MollicutesRF9 and follicular lymphoma.

AO.Scatter plot between family.Alcaligenaceae and follicular lymphoma.

AP.Scatter plot between class.Clostridia and follicular lymphoma.

AQ. Scatter plot between genus.Sutterella and follicular lymphoma.

AR.Scatter plot between genus.Phascolarctobacterium and follicular lymphoma.

AS.Scatter plot between genus..Eubacteriumcoprostanoligenesgroup and diffuse large B-cell lymphoma.

AT.Scatter plot between class.Alphaproteobacteria. and diffuse large B-cell lymphoma.

AU.Scatter plot between phylum.Cyanobacter and diffuse large B-cell lymphoma.

AV.Scatter plot between genus.Erysipelatoclostridium and diffuse large B-cell lymphoma.

AW.Scatter plot between family.Rhodospirillaceae. and mature T/NK-cell lymphomas.

AX.Scatter plot between genus.Anaerostipes and mature T/NK-cell lymphomas.

AY.Scatter plot between genus.Erysipelatoclostridium. and mature T/NK-cell lymphomas.

AZ.Scatter plot between genus.LachnospiraceaeUCG001.. and mature T/NK-cell lymphomas.

AAA.Scatter plot between genus..Eubacteriumrectalegroup. and mature T/NK-cell lymphomas.

AAB. Scatter plot between genus.Escherichia.Shigella. and mature T/NK-cell lymphomas.

AAC.Scatter plot between genus..Ruminococcusgnavusgroup and mature T/NK-cell lymphomas.

AAD. Scatter plot between order.Bifidobacteriales. and myeloproliferative neoplasms.

AAE. Scatter plot between family.Bifidobacteriaceae.. and myeloproliferative neoplasms.

AAF. Scatter plot between genus.Bifidobacterium. and myeloproliferative neoplasms.

AAG.Scatter plot between phylum.Firmicutes. and myeloproliferative neoplasms.

AAH.Scatter plot between genus.Coprococcus3. and myeloproliferative neoplasms.

AAI.Scatter plot between genus..Eubacteriumhalliigroup. and myeloproliferative neoplasms.

AAJ.Scatter plot between genus.Haemophilus. and myeloproliferative neoplasms

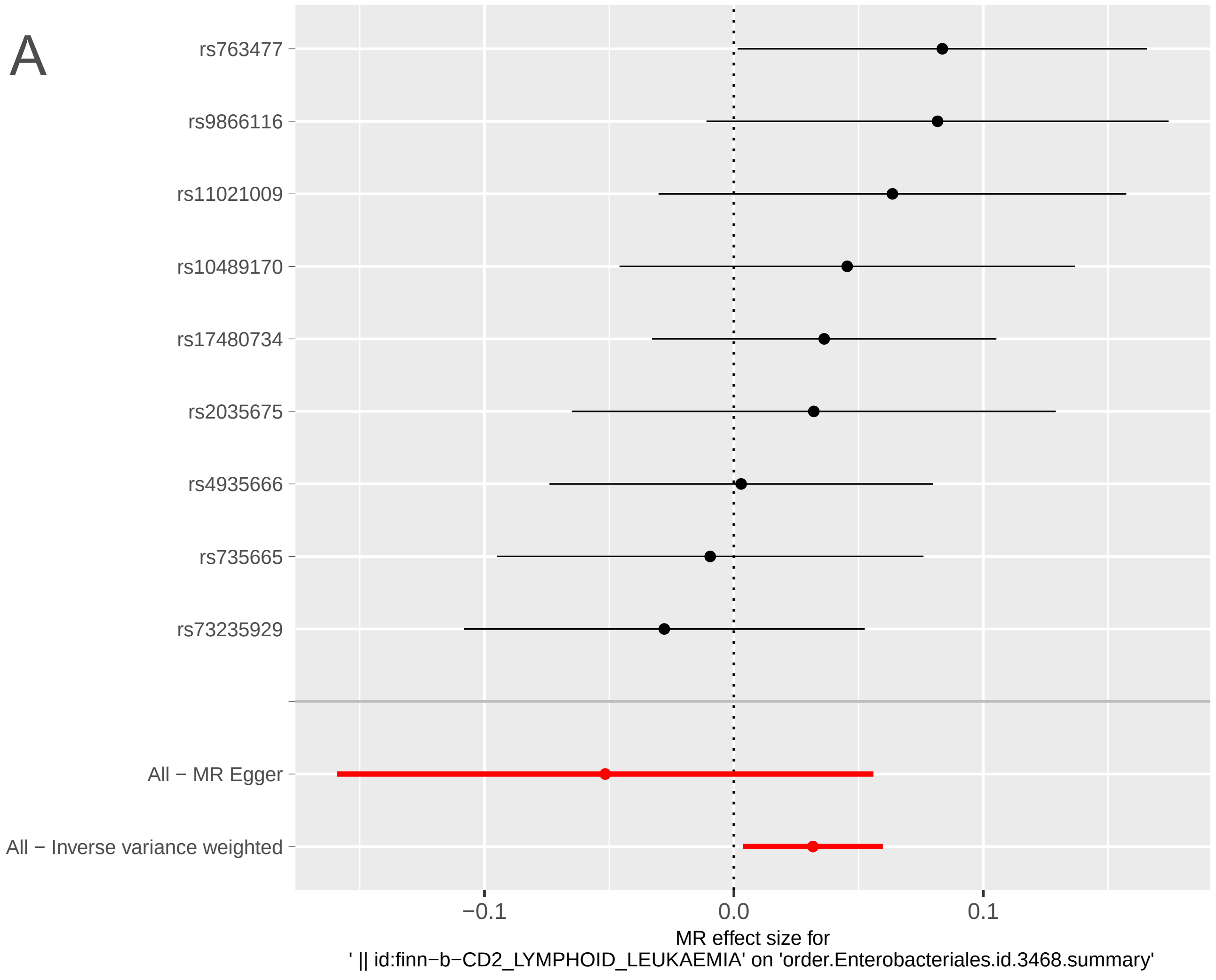
A
rs763477
rs9866116
rs11021009
rs10489170
rs17480734
rs2035675
rs4935666
rs735665
rs73235929
All − MR Egger
All − Inverse variance weighted
−0.1
0.0
0.1
MR effect size for
' || id:finn−b−CD2_LYMPHOID_LEUKAEMIA' on 'order.Enterobacteriales.id.3468.summary'

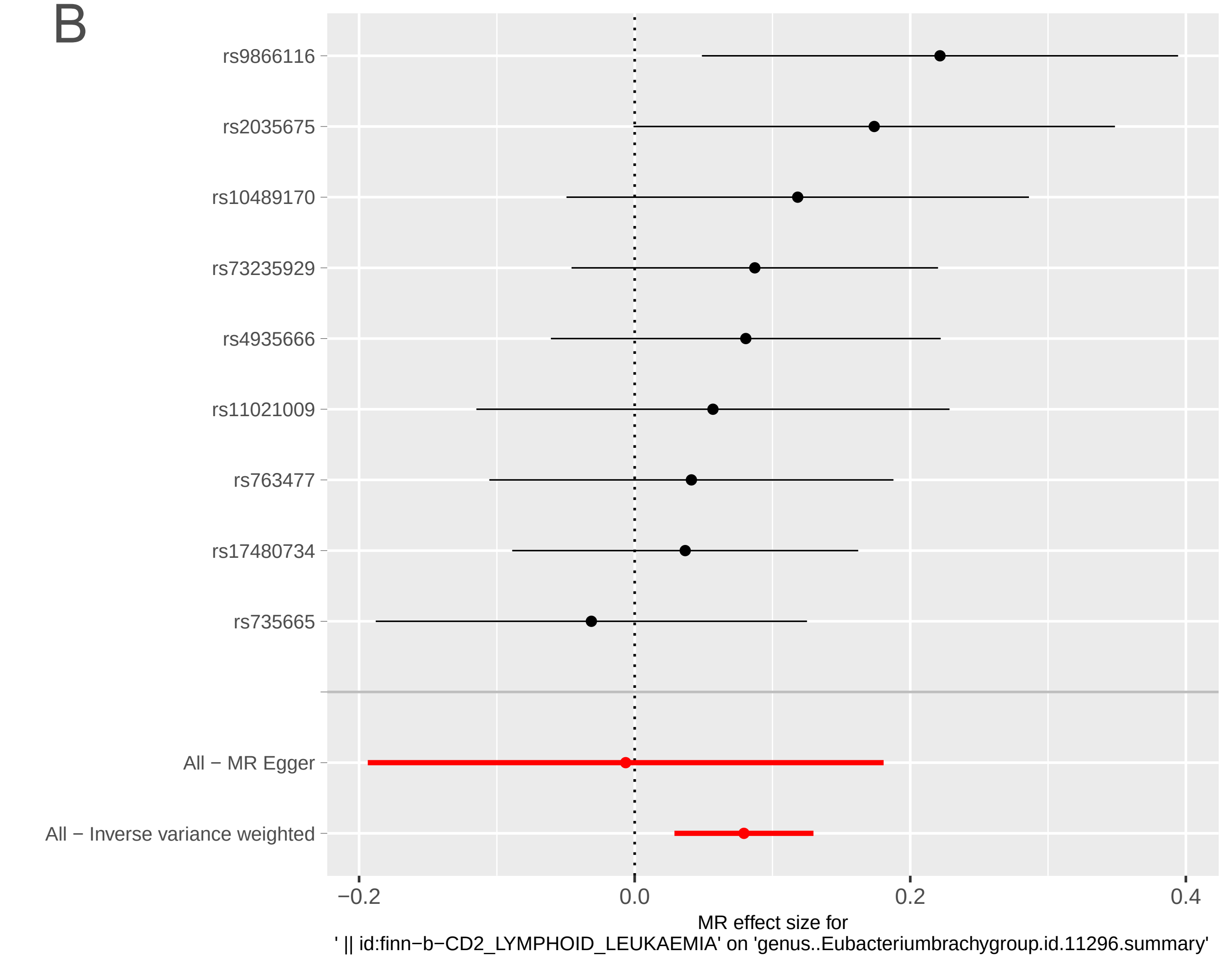
B
rs9866116
rs2035675
rs10489170
rs73235929
rs4935666
rs11021009
rs763477
rs17480734
rs735665
All − MR Egger
All − Inverse variance weighted
−0.2
0.0
0.2
0.4
MR effect size for
' || id:finn−b−CD2_LYMPHOID_LEUKAEMIA' on 'genus..Eubacteriumbrachygroup.id.11296.summary'

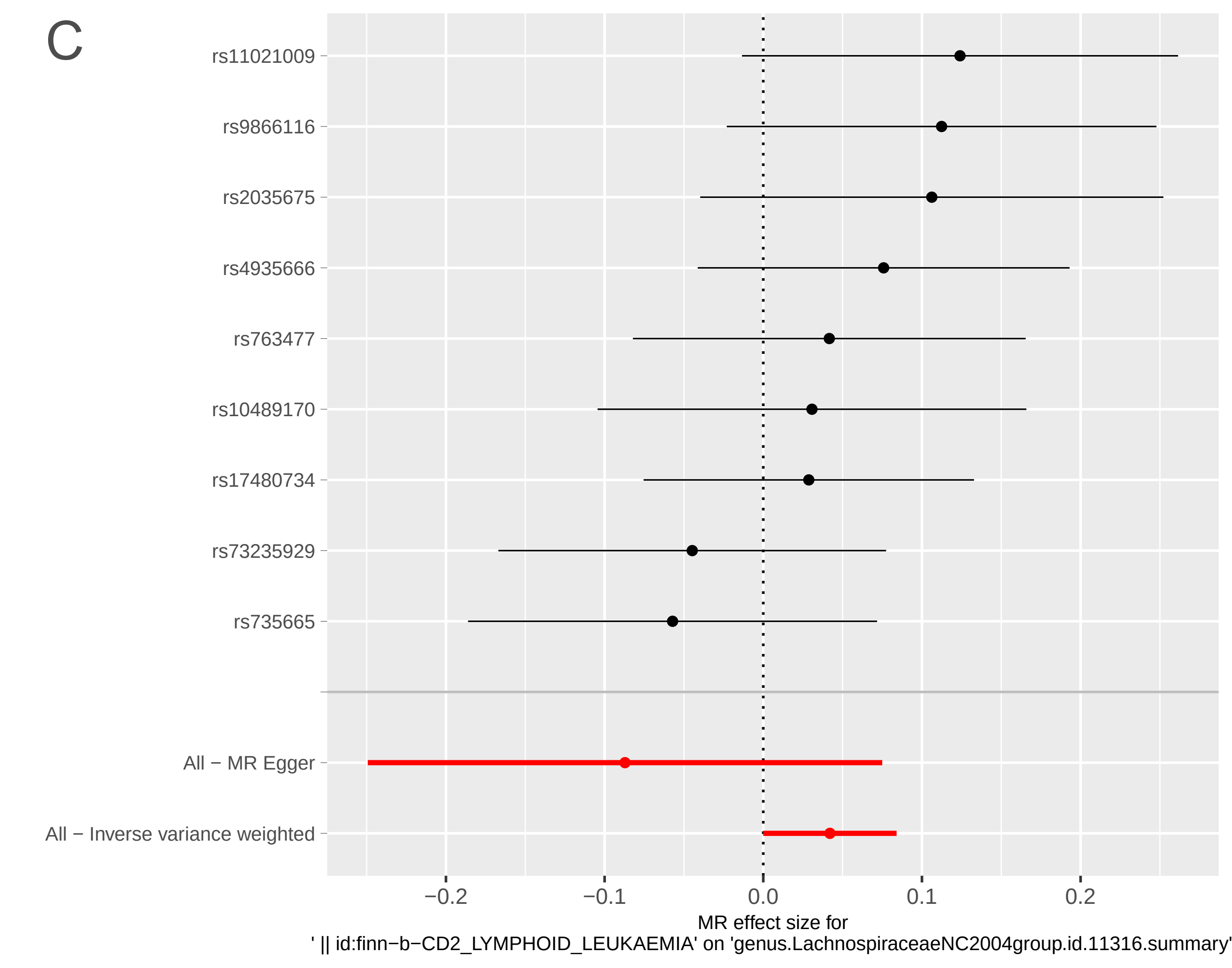
C
rs11021009
rs9866116
rs2035675
rs4935666
rs763477
rs10489170
rs17480734
rs73235929
rs735665
All − MR Egger
All − Inverse variance weighted
−0.2
−0.1
0.0
0.1
0.2
MR effect size for
' || id:finn−b−CD2_LYMPHOID_LEUKAEMIA' on 'genus.LachnospiraceaeNC2004group.id.11316.summary'

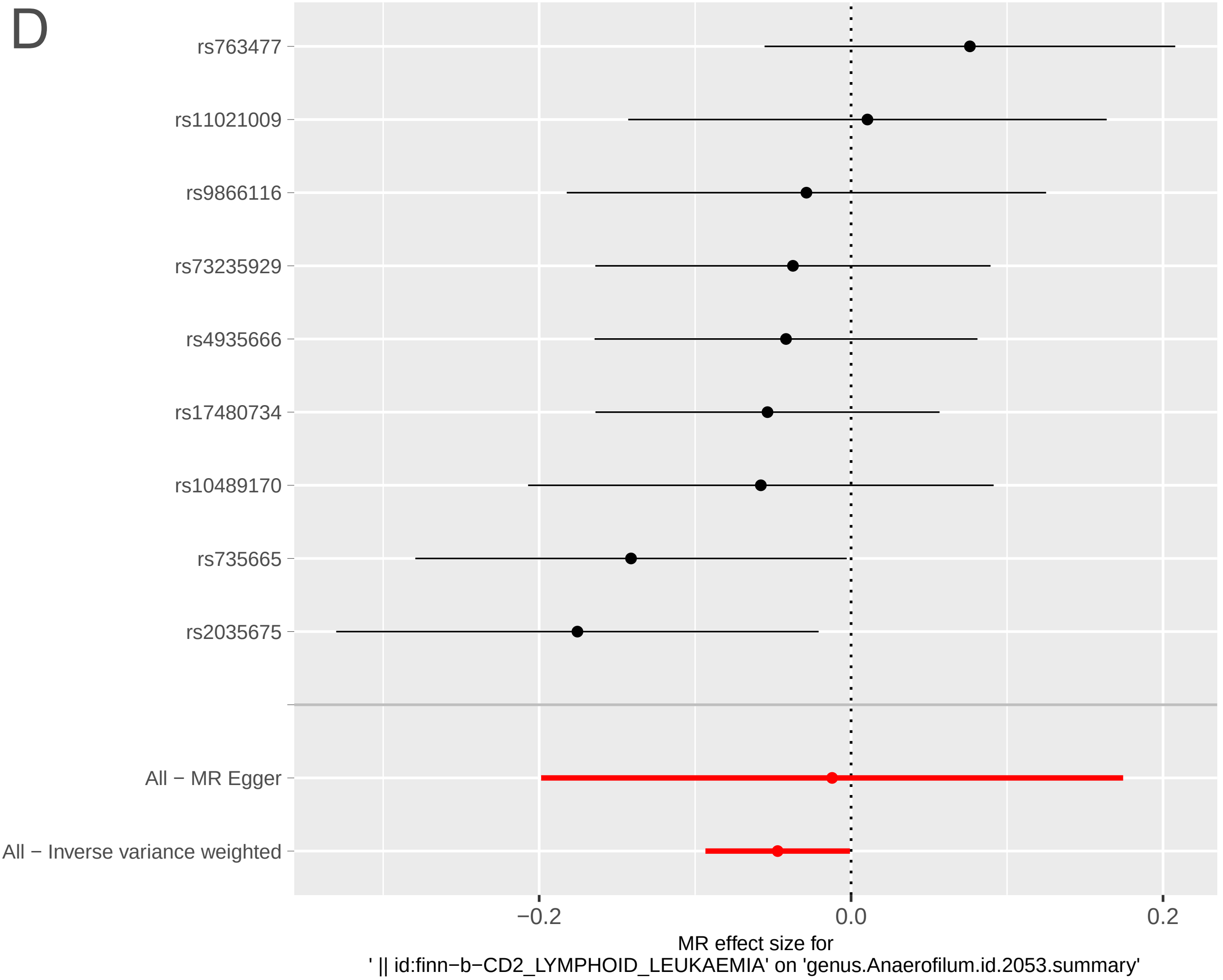
D
rs763477
rs11021009
rs9866116
rs73235929
rs4935666
rs17480734
rs10489170
rs735665
rs2035675
All − MR Egger
All − Inverse variance weighted
−0.2
0.0
0.2
MR effect size for
' || id:finn−b−CD2_LYMPHOID_LEUKAEMIA' on 'genus.Anaerofilum.id.2053.summary'

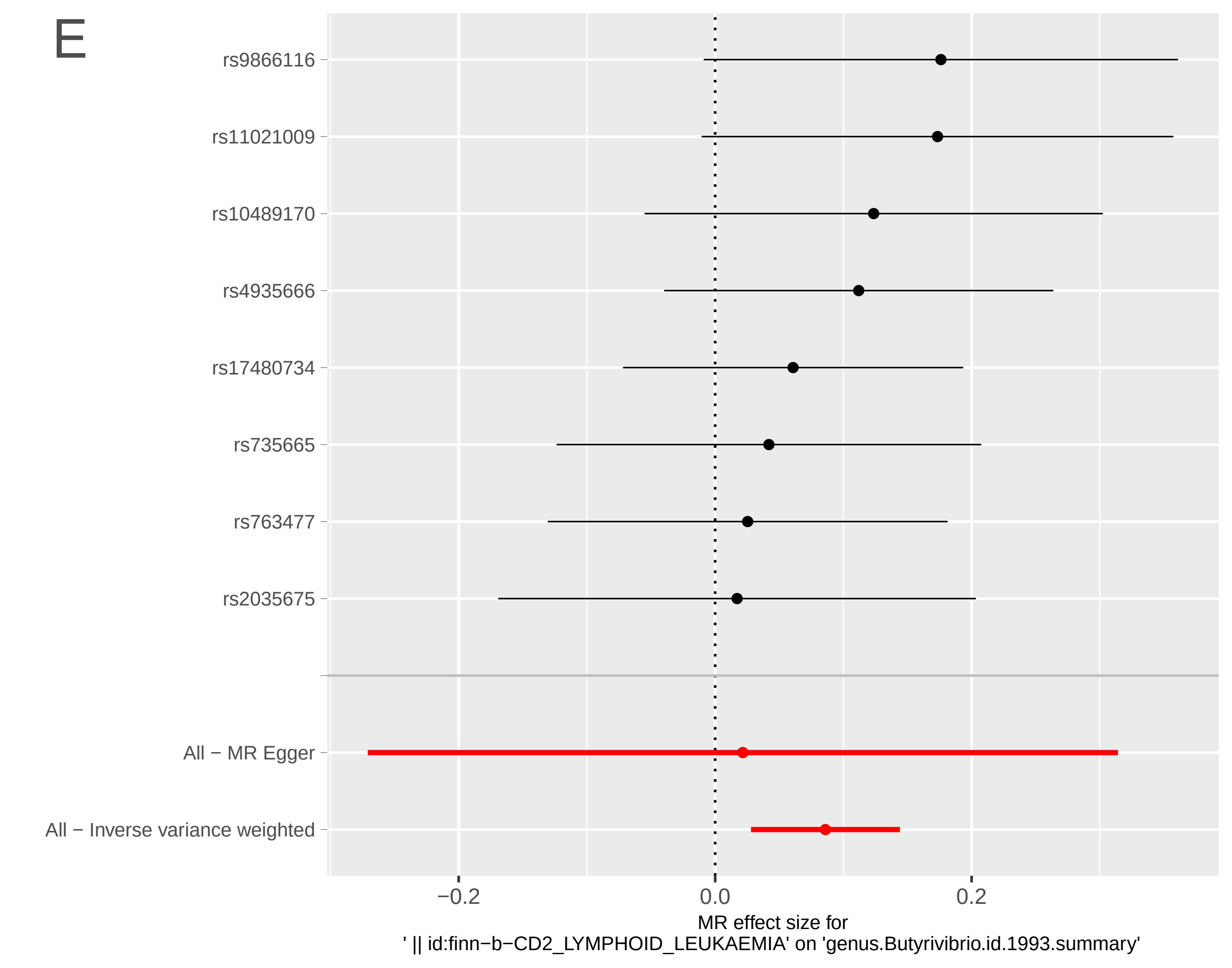
E
rs9866116
rs11021009
rs10489170
rs4935666
rs17480734
rs735665
rs763477
rs2035675
All − MR Egger
All − Inverse variance weighted
−0.2
0.0
0.2
MR effect size for
' || id:finn−b−CD2_LYMPHOID_LEUKAEMIA' on 'genus.Butyrivibrio.id.1993.summary'

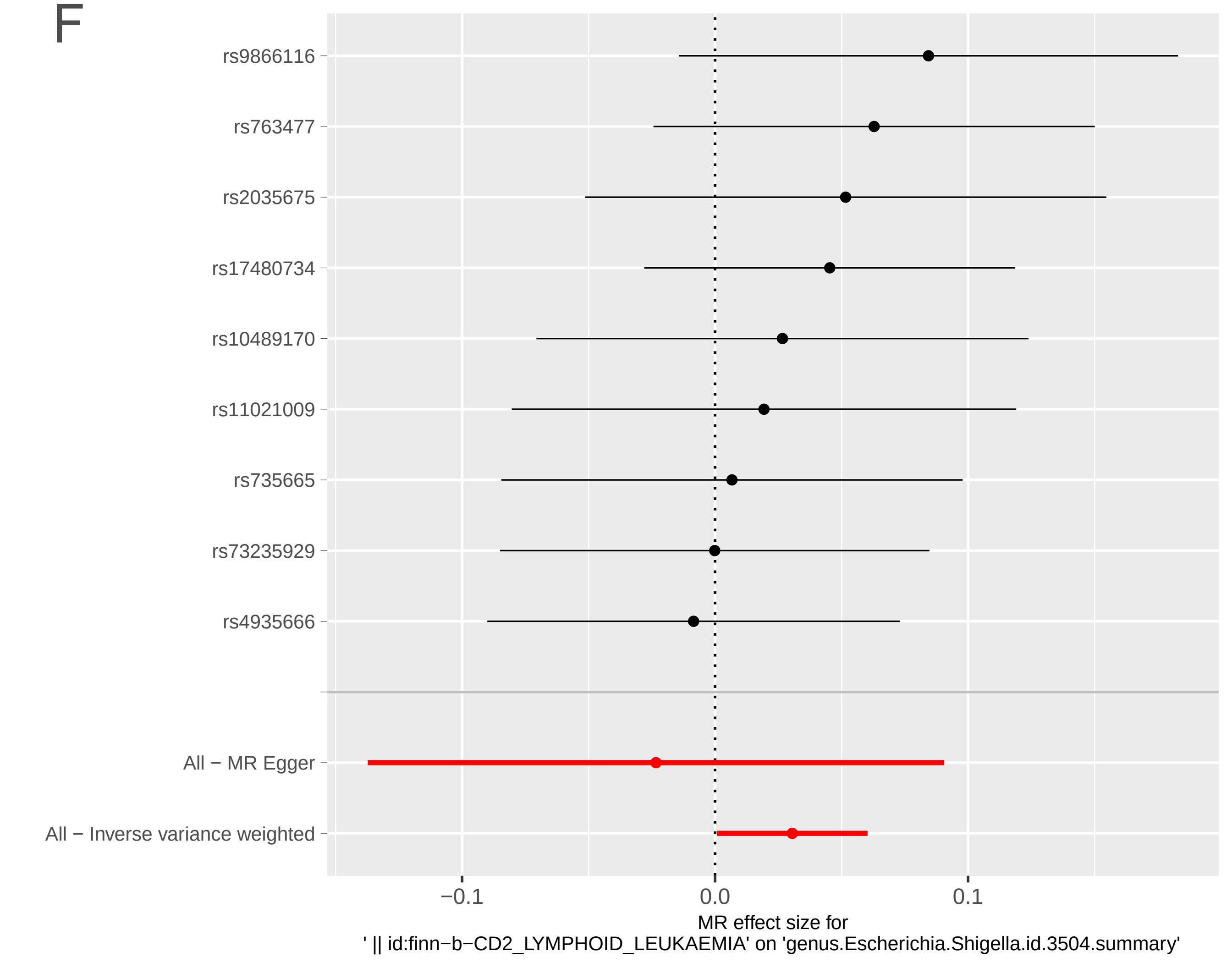
F
rs9866116
rs763477
rs2035675
rs17480734
rs10489170
rs11021009
rs735665
rs73235929
rs4935666
All − MR Egger
All − Inverse variance weighted
−0.1
0.0
0.1
MR effect size for
' || id:finn−b−CD2_LYMPHOID_LEUKAEMIA' on 'genus.Escherichia.Shigella.id.3504.summary'

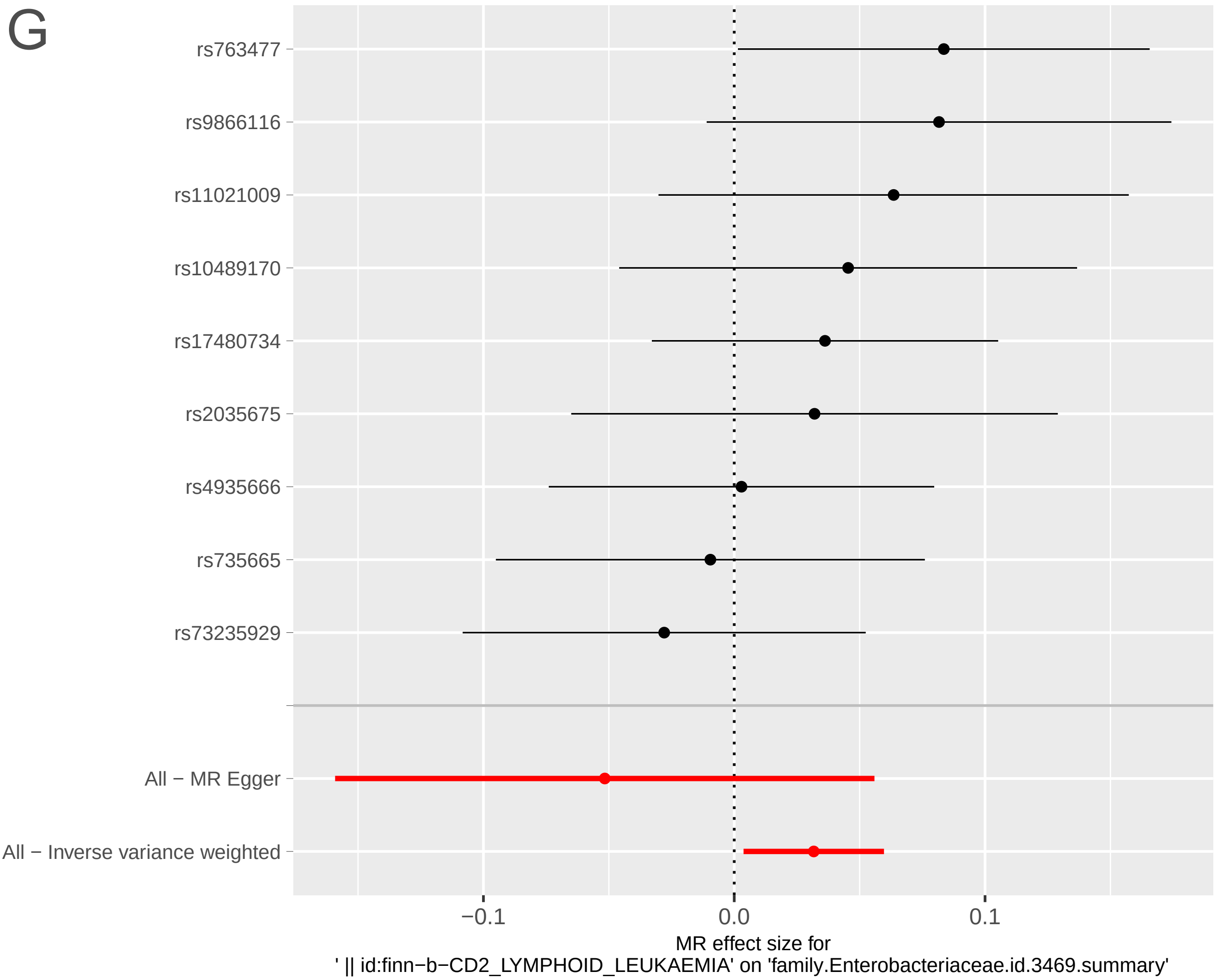
G
rs763477
rs9866116
rs11021009
rs10489170
rs17480734
rs2035675
rs4935666
rs735665
rs73235929
All − MR Egger
All − Inverse variance weighted
−0.1
0.0
0.1
MR effect size for
' || id:finn−b−CD2_LYMPHOID_LEUKAEMIA' on 'family.Enterobacteriaceae.id.3469.summary'

H

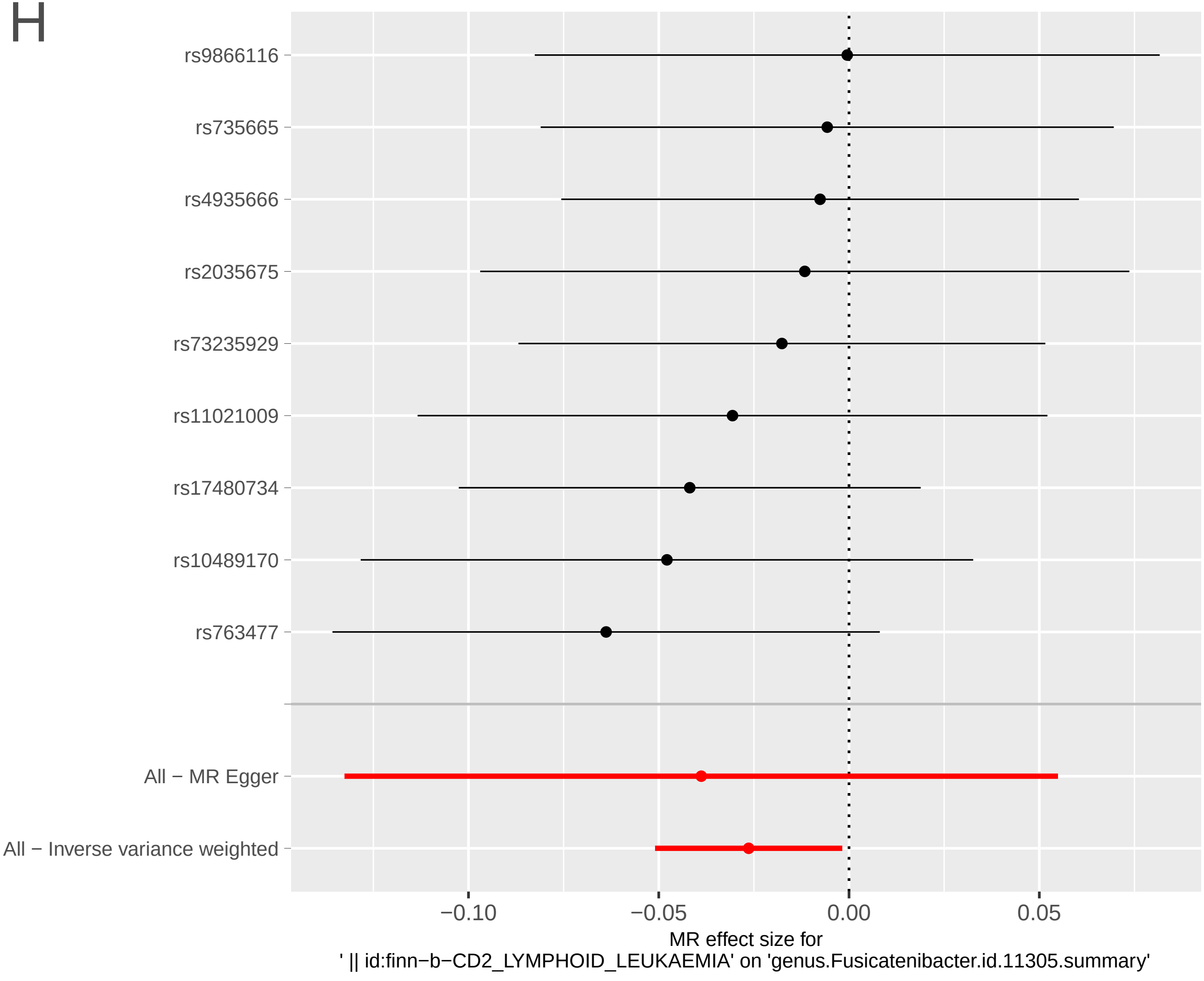
rs9866116
rs735665
rs4935666
rs2035675
rs73235929
rs11021009
rs17480734
rs10489170
rs763477
All − MR Egger
All − Inverse variance weighted
−0.10
−0.05
0.00
0.05
MR effect size for
' || id:finn−b−CD2_LYMPHOID_LEUKAEMIA' on 'genus.Fusicatenibacter.id.11305.summary'

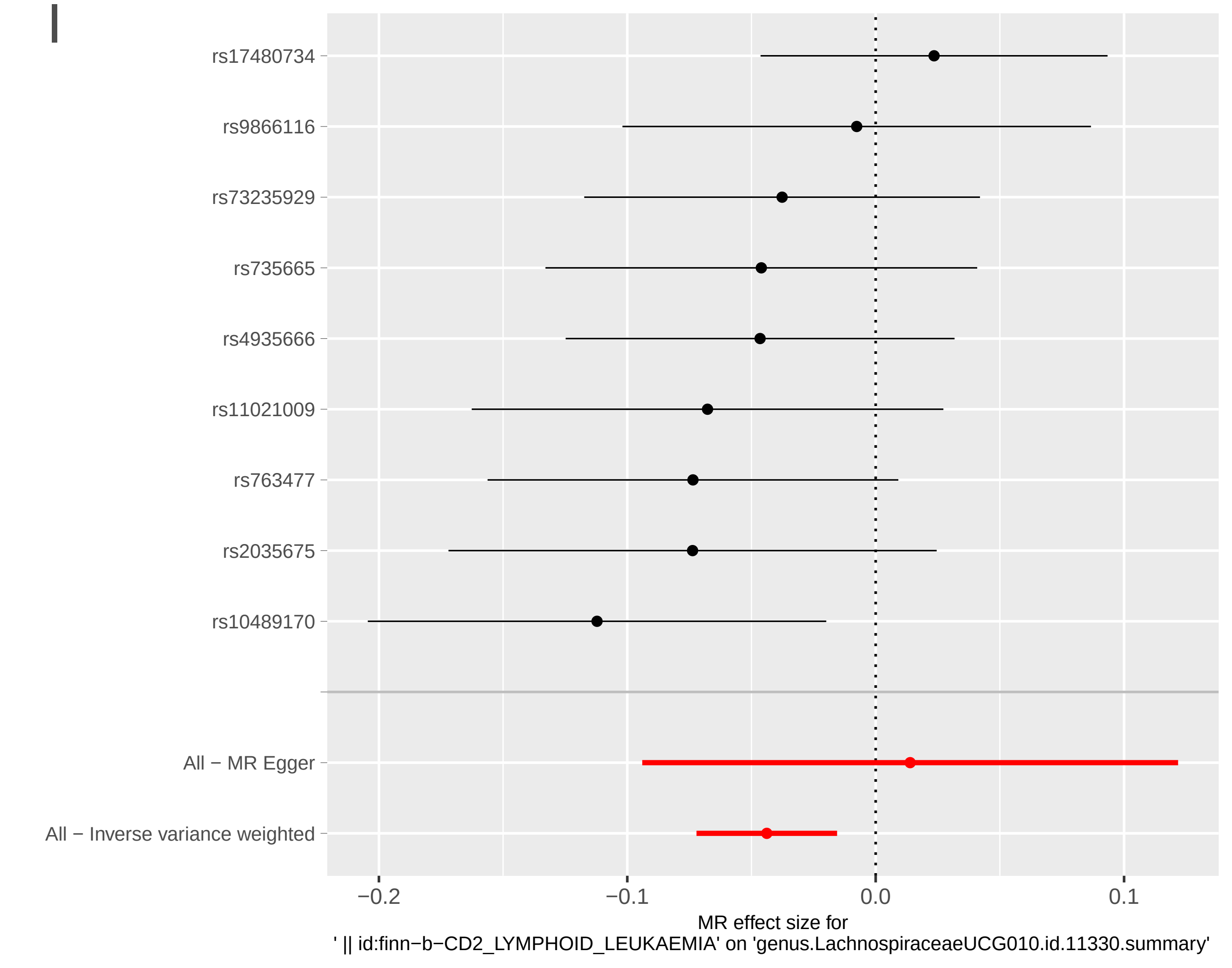
rs17480734
rs9866116
rs73235929
rs735665
rs4935666
rs11021009
rs763477
rs2035675
rs10489170
All − MR Egger
All − Inverse variance weighted
−0.2
−0.1
0.0
0.1
MR effect size for
' || id:finn−b−CD2_LYMPHOID_LEUKAEMIA' on 'genus.LachnospiraceaeUCG010.id.11330.summary'

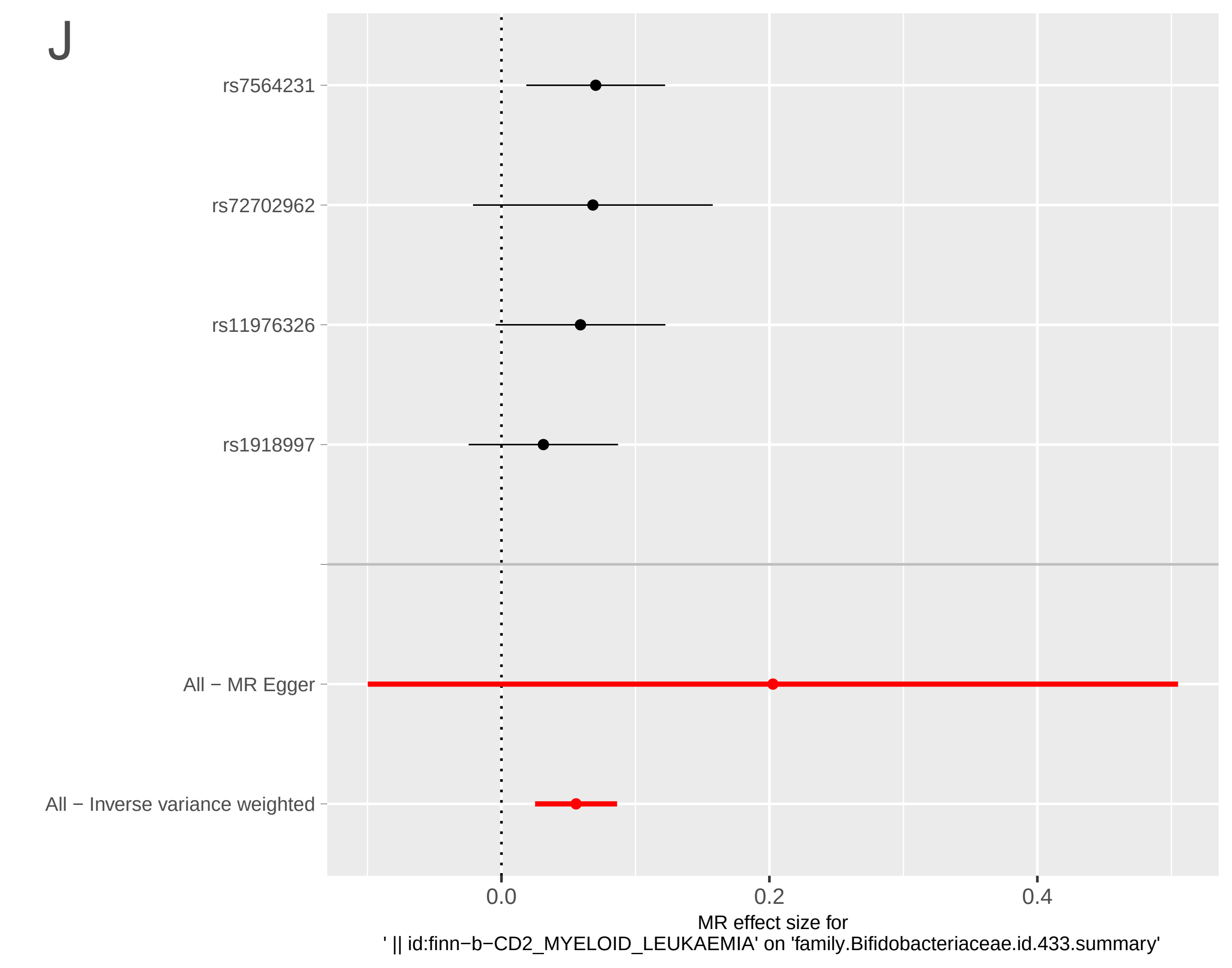
J
rs7564231
rs72702962
rs11976326
rs1918997
All − MR Egger
All − Inverse variance weighted
0.0
0.2
0.4
MR effect size for
' || id:finn−b−CD2_MYELOID_LEUKAEMIA' on 'family.Bifidobacteriaceae.id.433.summary'

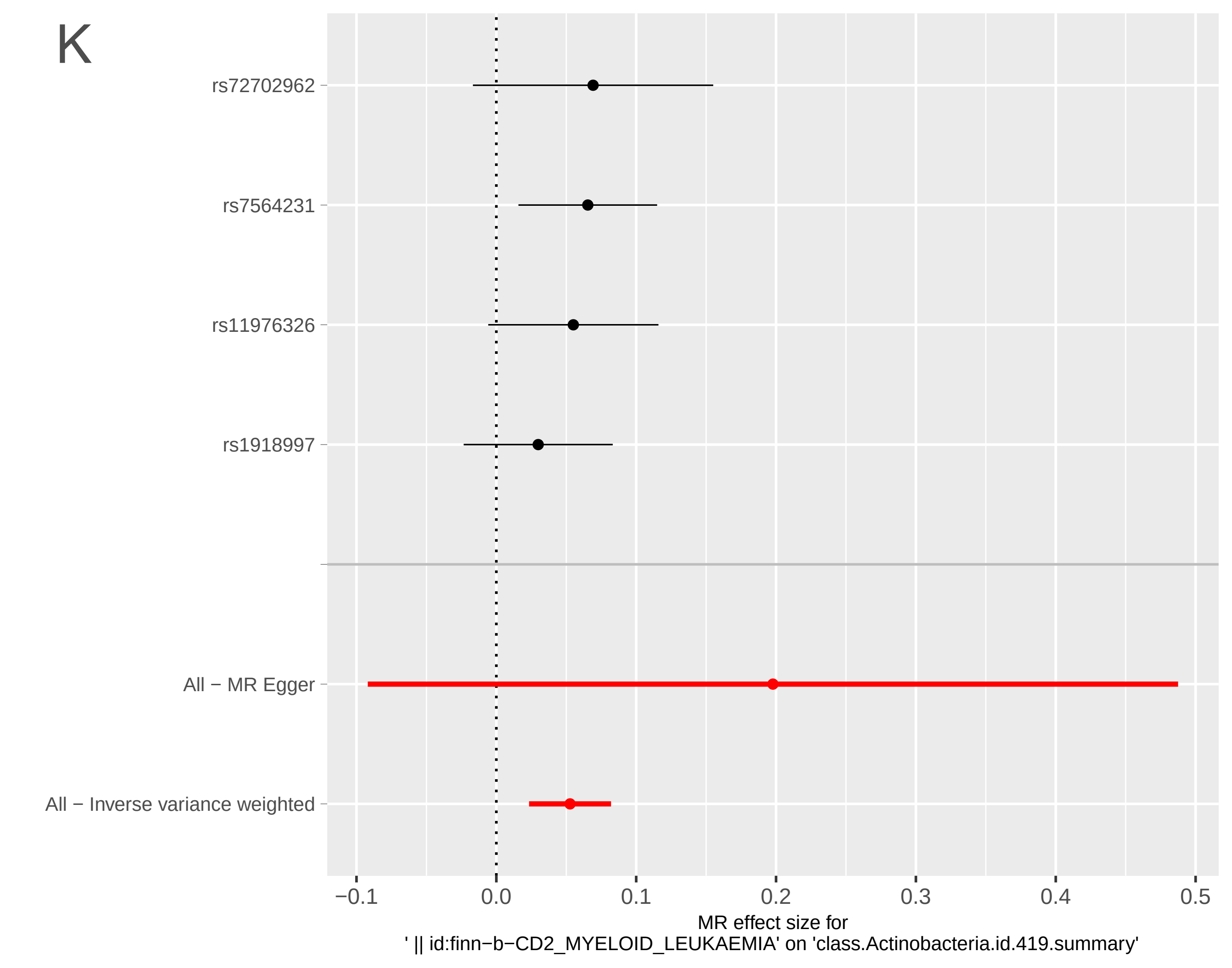
K
rs72702962
rs7564231
rs11976326
rs1918997
All − MR Egger
All − Inverse variance weighted
−0.1
0.0
0.1
0.2
0.3
0.4
0.5
MR effect size for
' || id:finn−b−CD2_MYELOID_LEUKAEMIA' on 'class.Actinobacteria.id.419.summary'

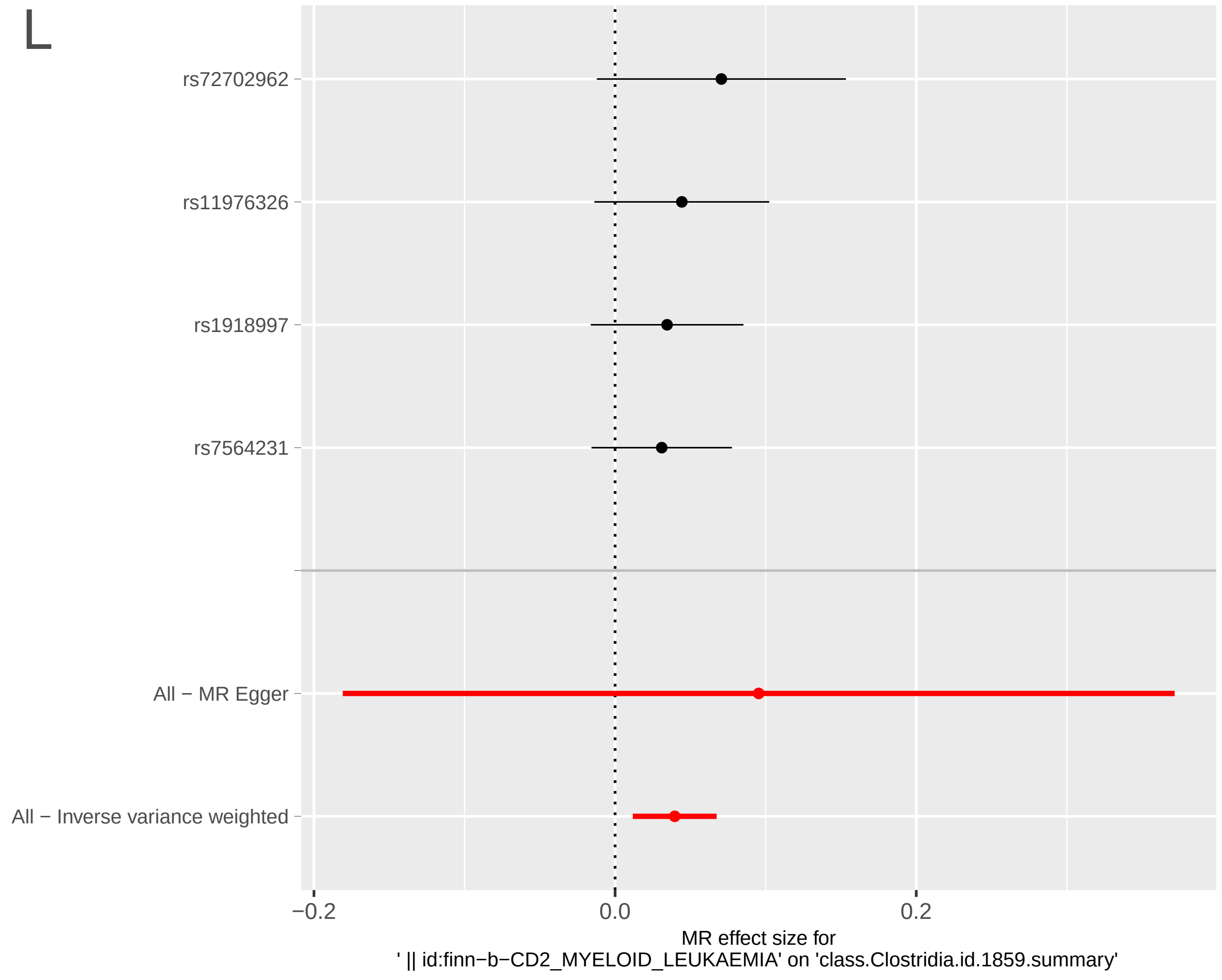
L
rs72702962
rs11976326
rs1918997
rs7564231
All − MR Egger
All − Inverse variance weighted
−0.2
0.0
0.2
MR effect size for
' || id:finn−b−CD2_MYELOID_LEUKAEMIA' on 'class.Clostridia.id.1859.summary'

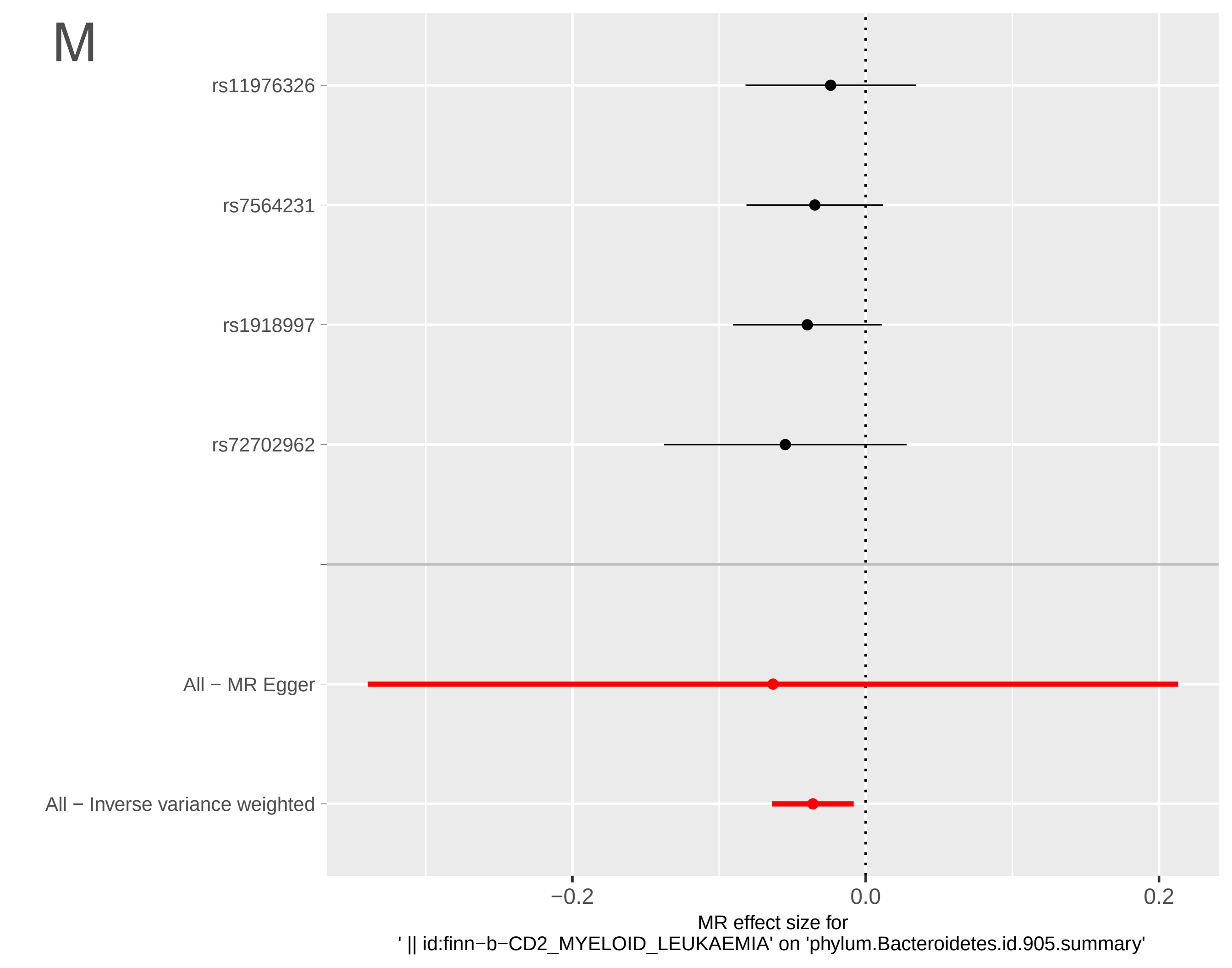
M
rs11976326
rs7564231
rs1918997
rs72702962
All − MR Egger
All − Inverse variance weighted
−0.2
0.0
0.2
MR effect size for
' || id:finn−b−CD2_MYELOID_LEUKAEMIA' on 'phylum.Bacteroidetes.id.905.summary'

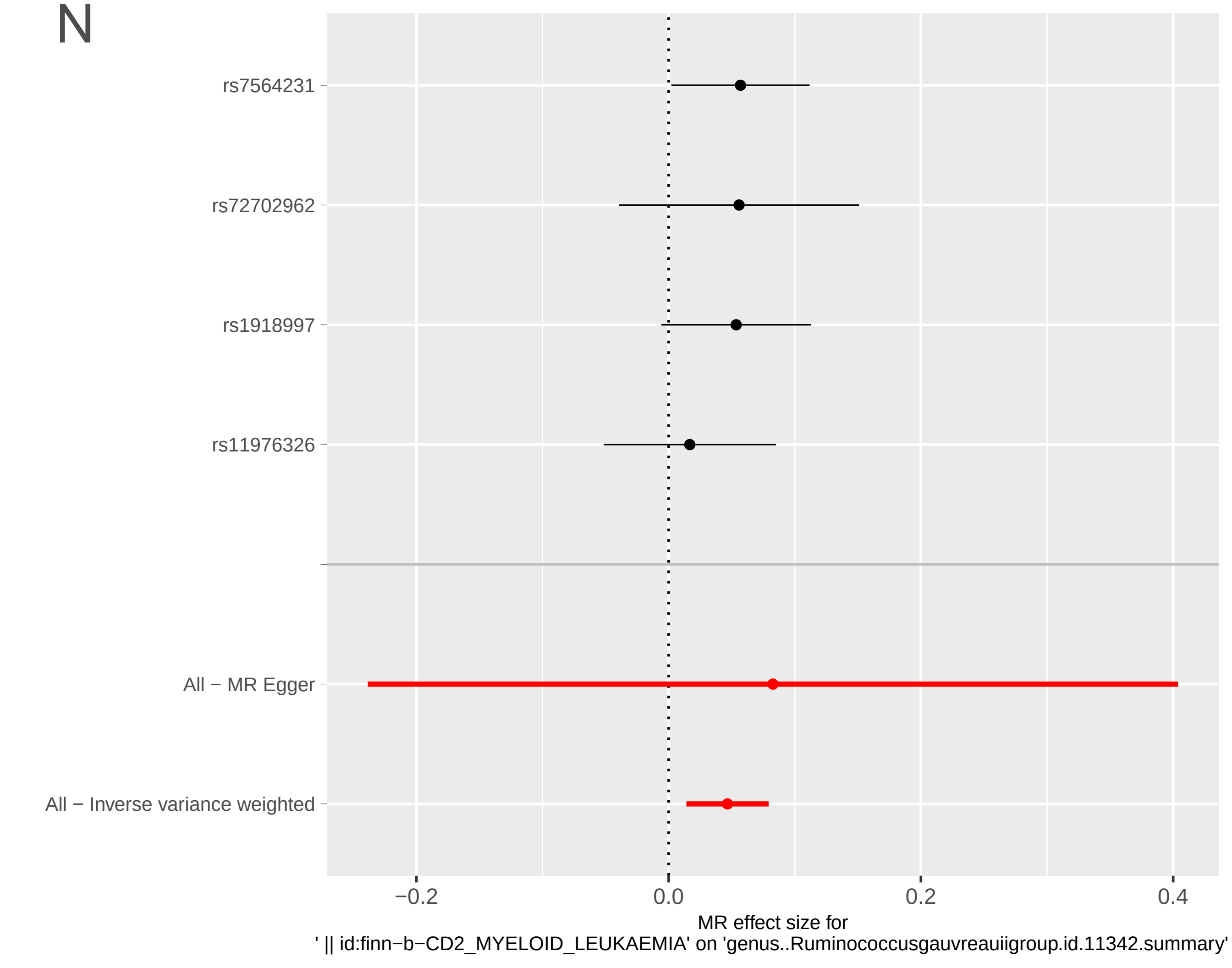

N
rs7564231
rs72702962
rs1918997
rs11976326
All − MR Egger
All − Inverse variance weighted
−0.2
0.0
0.2
0.4
MR effect size for
' || id:finn−b−CD2_MYELOID_LEUKAEMIA' on 'genus..Ruminococcusgauvreauiigroup.id.11342.summary'

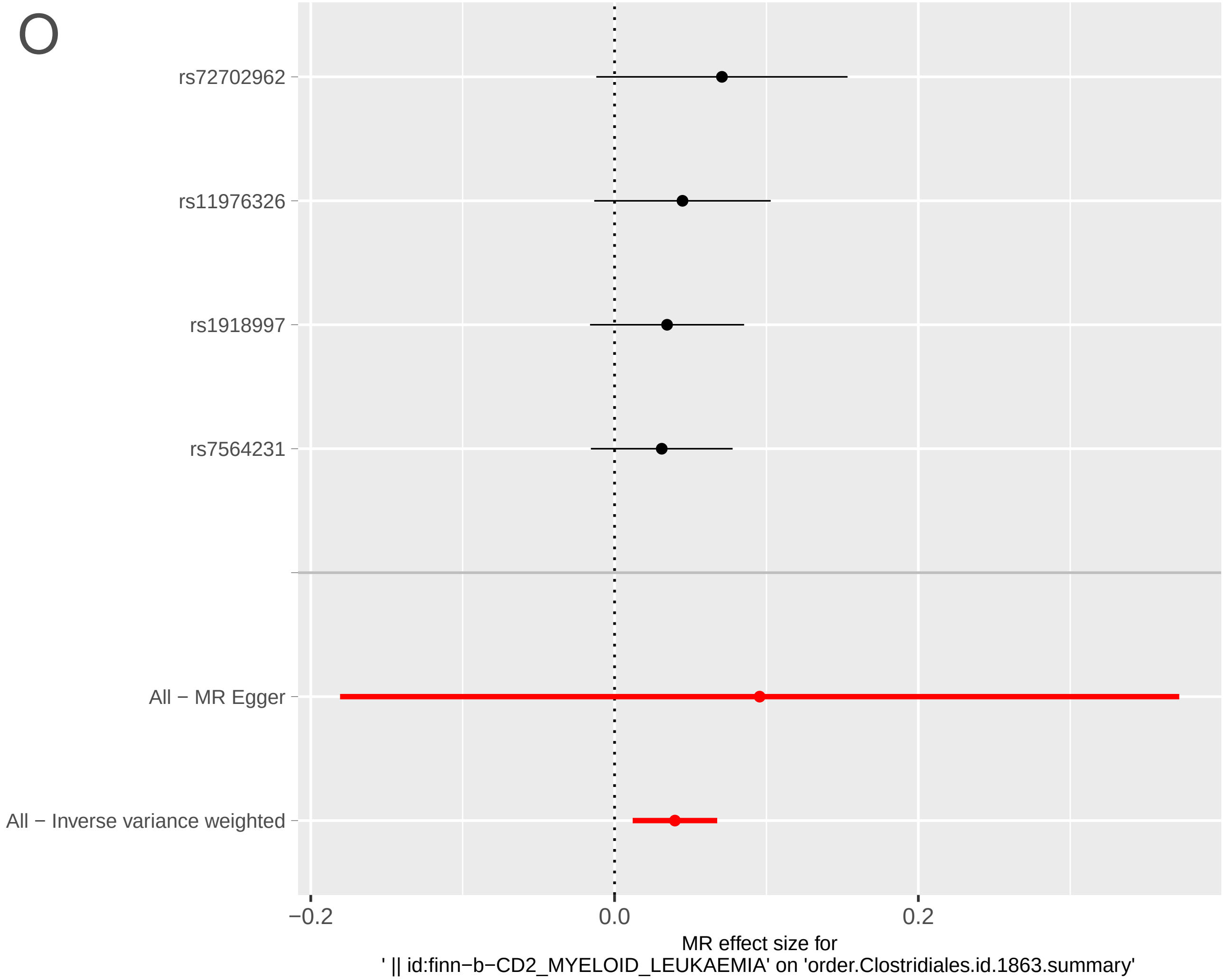
O
rs72702962
rs11976326
rs1918997
rs7564231
All − MR Egger
All − Inverse variance weighted
−0.2
0.0
0.2
MR effect size for
' || id:finn−b−CD2_MYELOID_LEUKAEMIA' on 'order.Clostridiales.id.1863.summary'

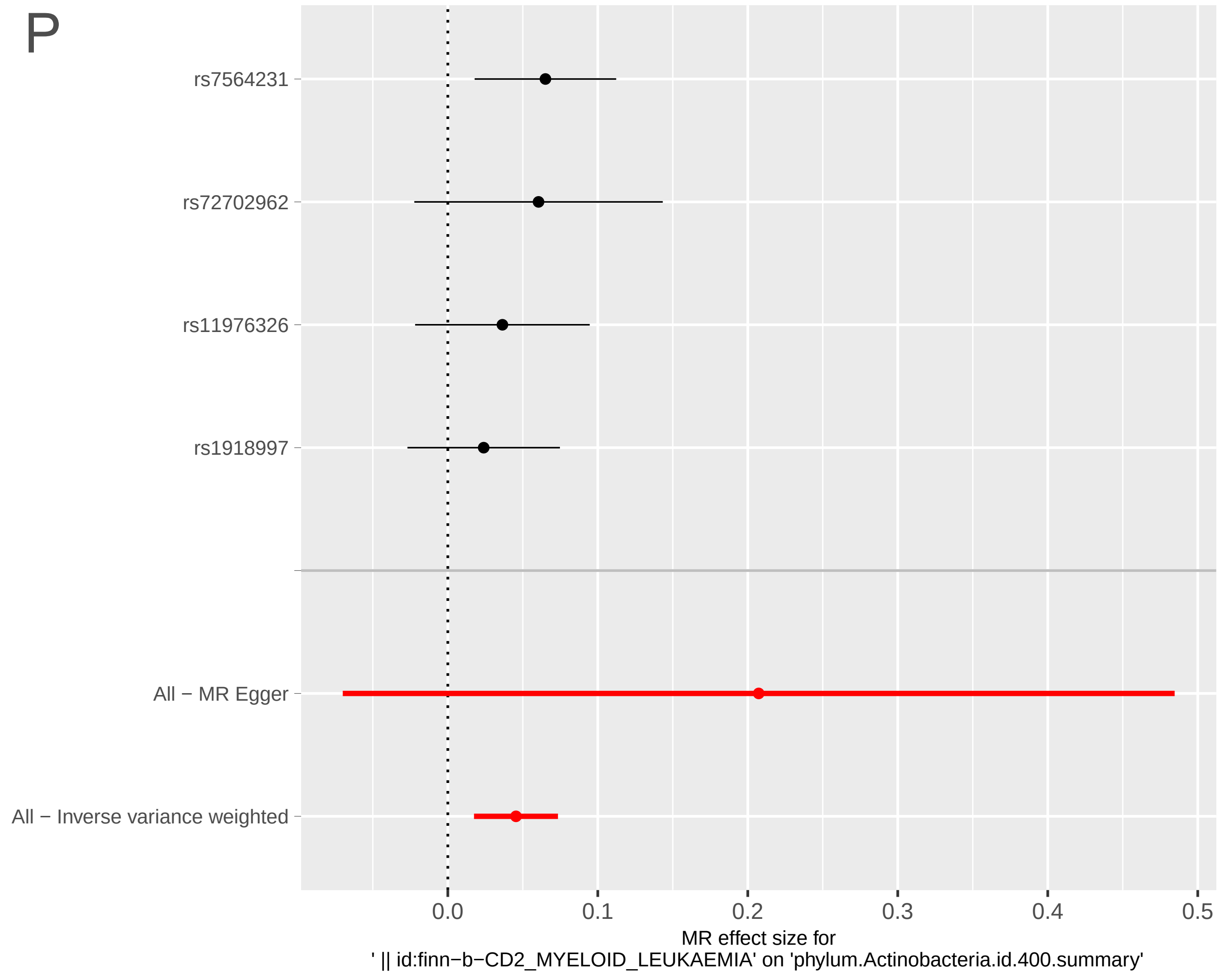
P
rs7564231
rs72702962
rs11976326
rs1918997
All − MR Egger
All − Inverse variance weighted
0.0
0.1
0.2
0.3
0.4
0.5
MR effect size for
' || id:finn−b−CD2_MYELOID_LEUKAEMIA' on 'phylum.Actinobacteria.id.400.summary'

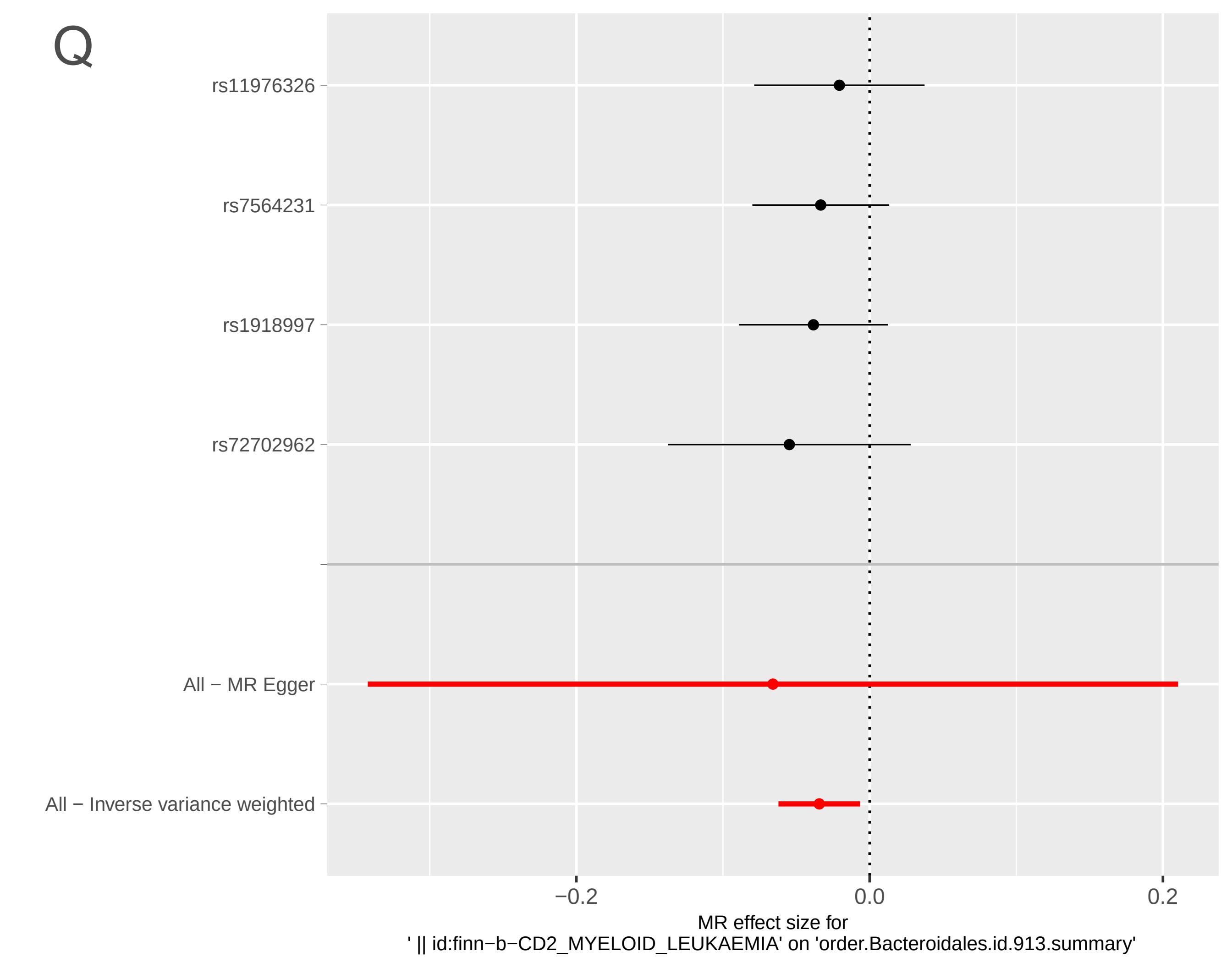
Q
rs11976326
rs7564231
rs1918997
rs72702962
All − MR Egger
All − Inverse variance weighted
−0.2
0.0
0.2
MR effect size for
' || id:finn−b−CD2_MYELOID_LEUKAEMIA' on 'order.Bacteroidales.id.913.summary'

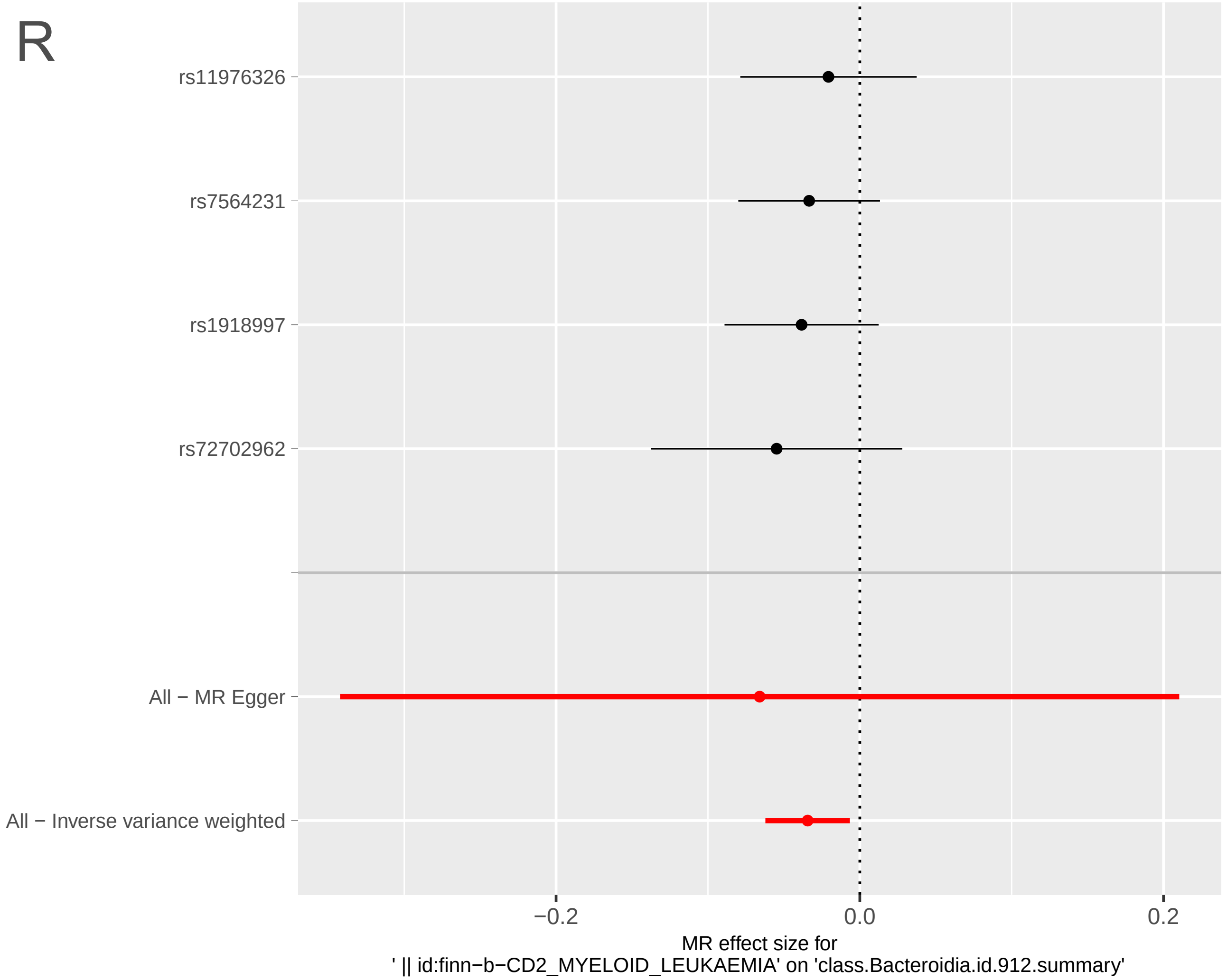
R
rs11976326
rs7564231
rs1918997
rs72702962
All − MR Egger
All − Inverse variance weighted
−0.2
0.0
0.2
MR effect size for
' || id:finn−b−CD2_MYELOID_LEUKAEMIA' on 'class.Bacteroidia.id.912.summary'

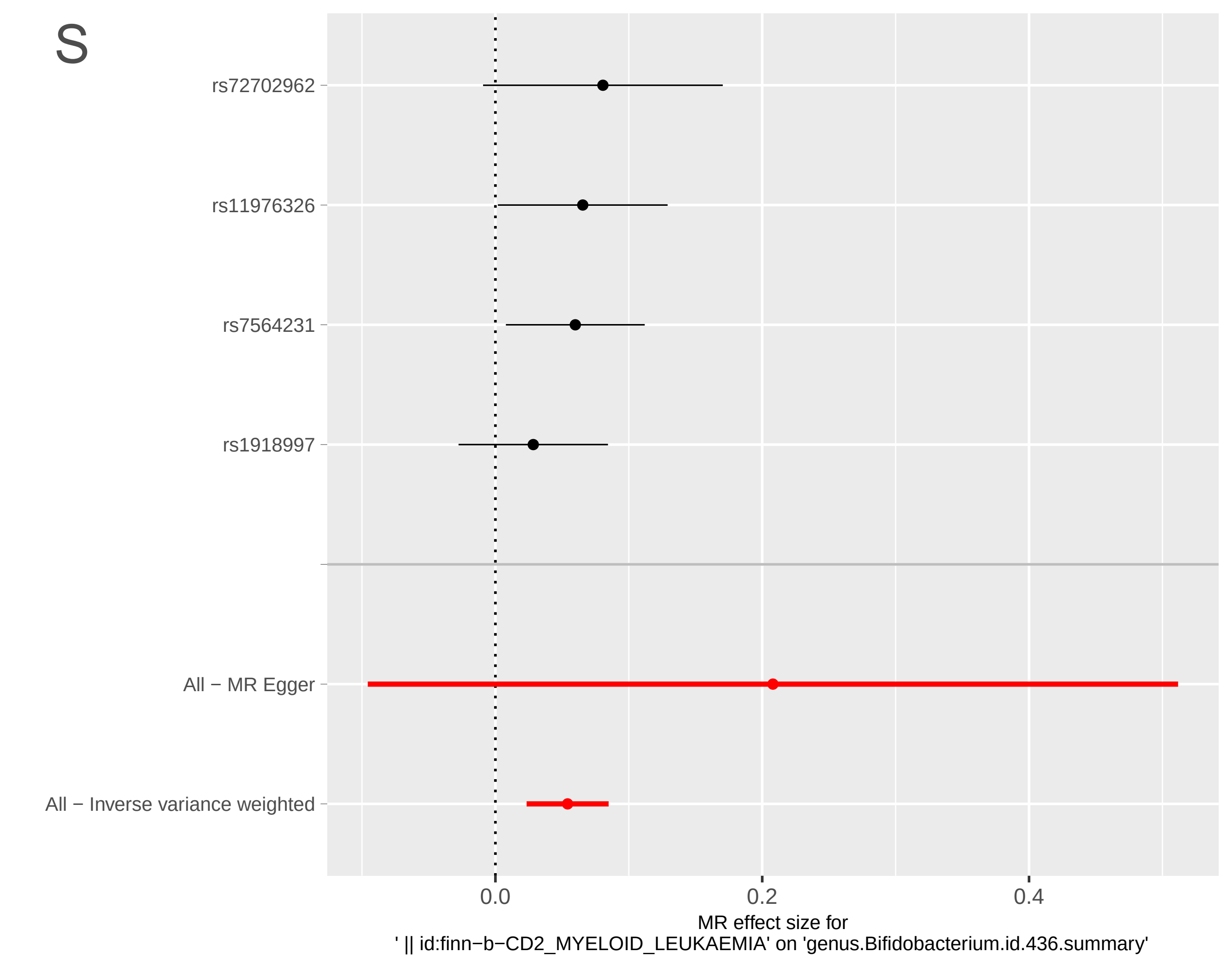
S
rs72702962
rs11976326
rs7564231
rs1918997
All − MR Egger
All − Inverse variance weighted
0.0
0.2
0.4
MR effect size for
' || id:finn−b−CD2_MYELOID_LEUKAEMIA' on 'genus.Bifidobacterium.id.436.summary'

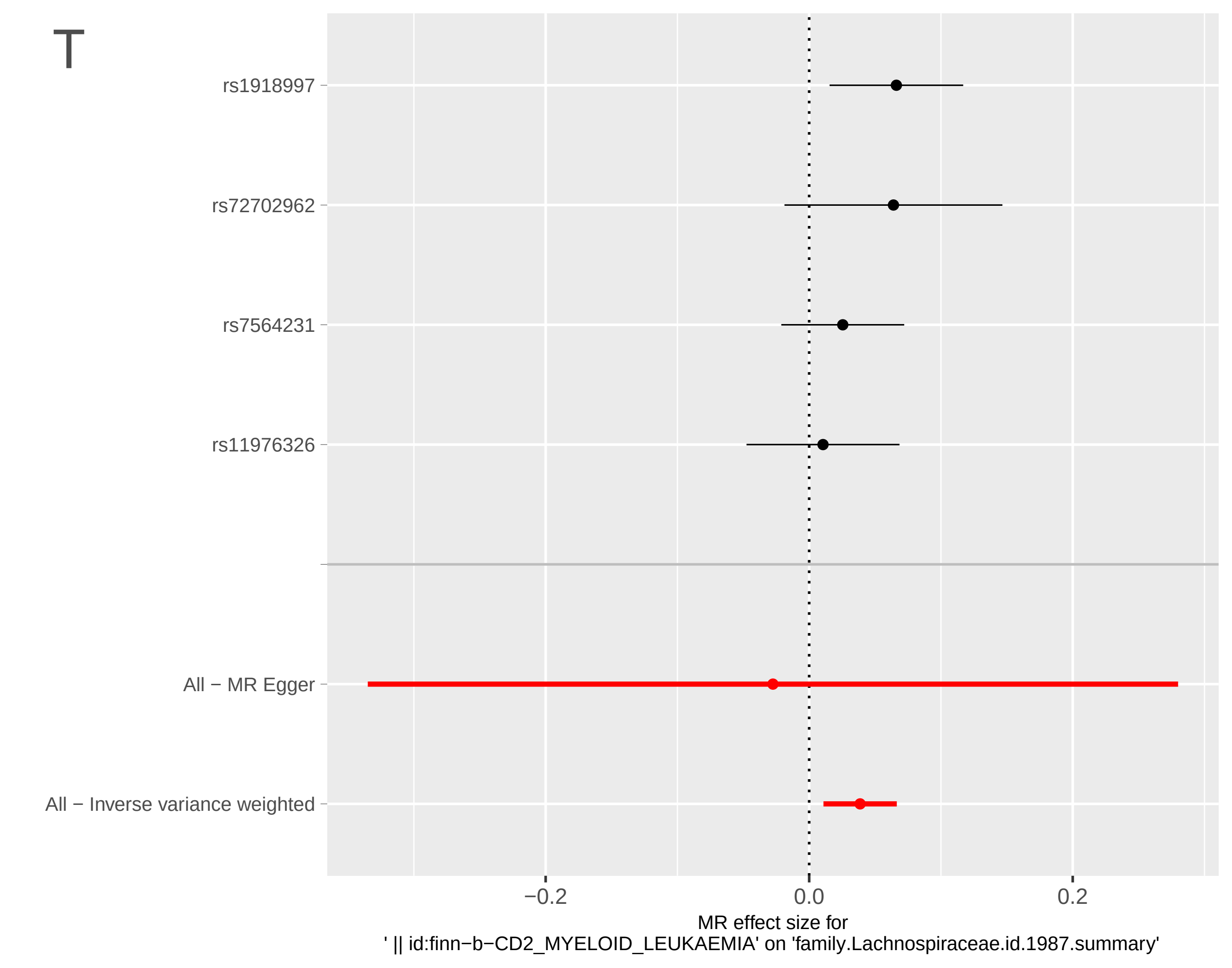
T
rs1918997
rs72702962
rs7564231
rs11976326
All − MR Egger
All − Inverse variance weighted
−0.2
0.0
0.2
MR effect size for
' || id:finn−b−CD2_MYELOID_LEUKAEMIA' on 'family.Lachnospiraceae.id.1987.summary'

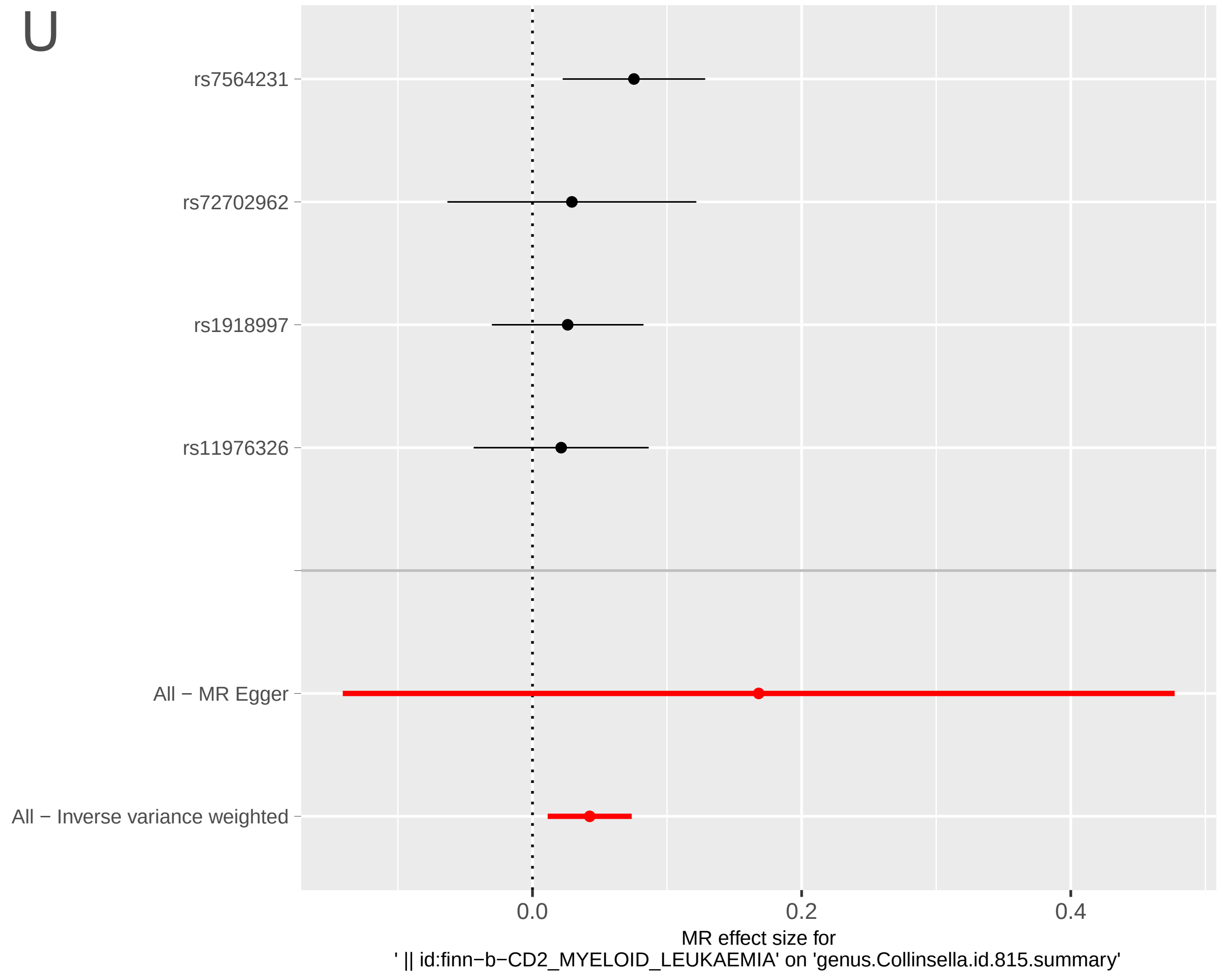
U
rs7564231
rs72702962
rs1918997
rs11976326
All − MR Egger
All − Inverse variance weighted
0.0
0.2
0.4
MR effect size for
' || id:finn−b−CD2_MYELOID_LEUKAEMIA' on 'genus.Collinsella.id.815.summary'

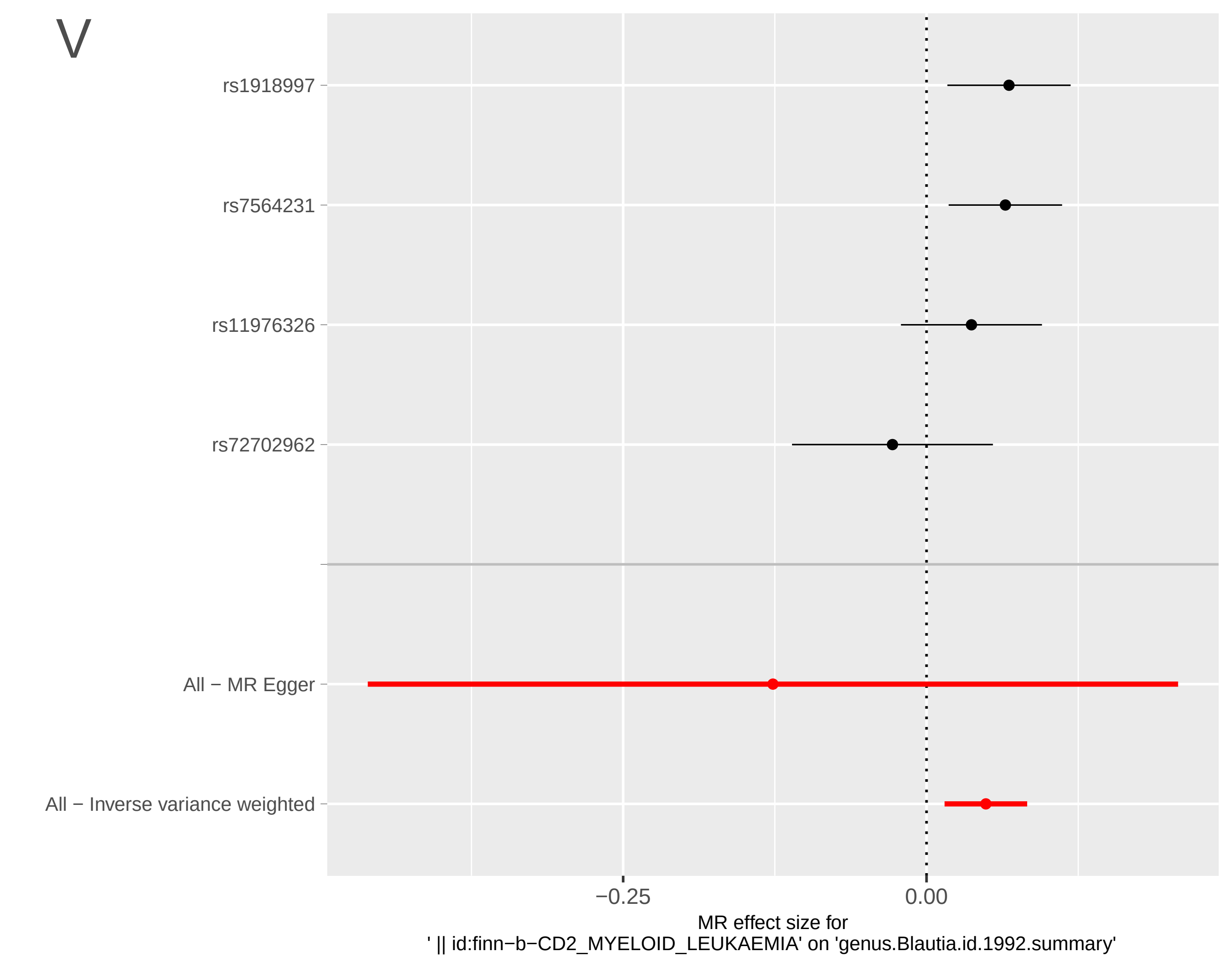
V
rs1918997
rs7564231
rs11976326
rs72702962
All − MR Egger
All − Inverse variance weighted
−0.25
0.00
MR effect size for
' || id:finn−b−CD2_MYELOID_LEUKAEMIA' on 'genus.Blautia.id.1992.summary'

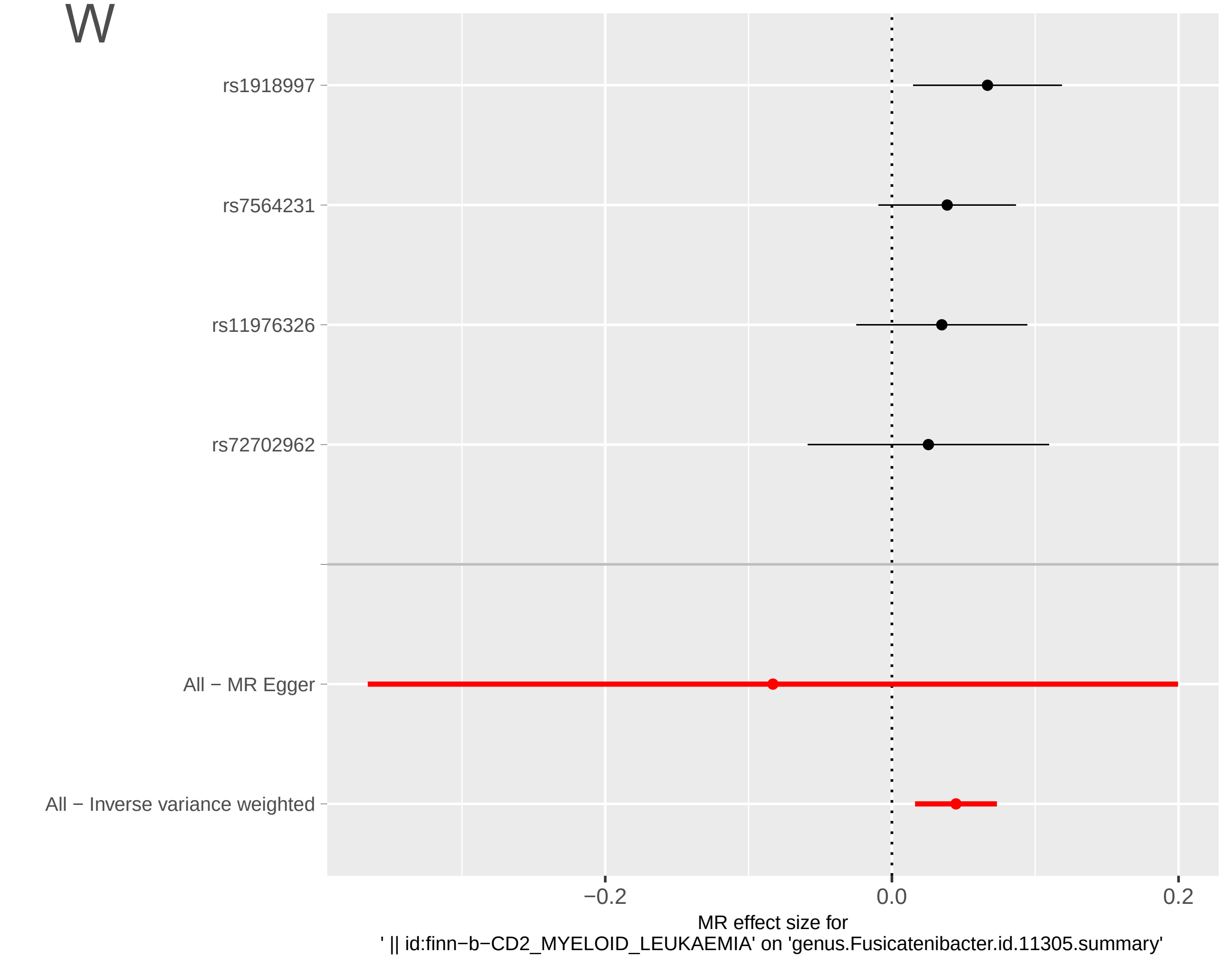
W
rs1918997
rs7564231
rs11976326
rs72702962
All − MR Egger
All − Inverse variance weighted
−0.2
0.0
0.2
MR effect size for
' || id:finn−b−CD2_MYELOID_LEUKAEMIA' on 'genus.Fusicatenibacter.id.11305.summary'

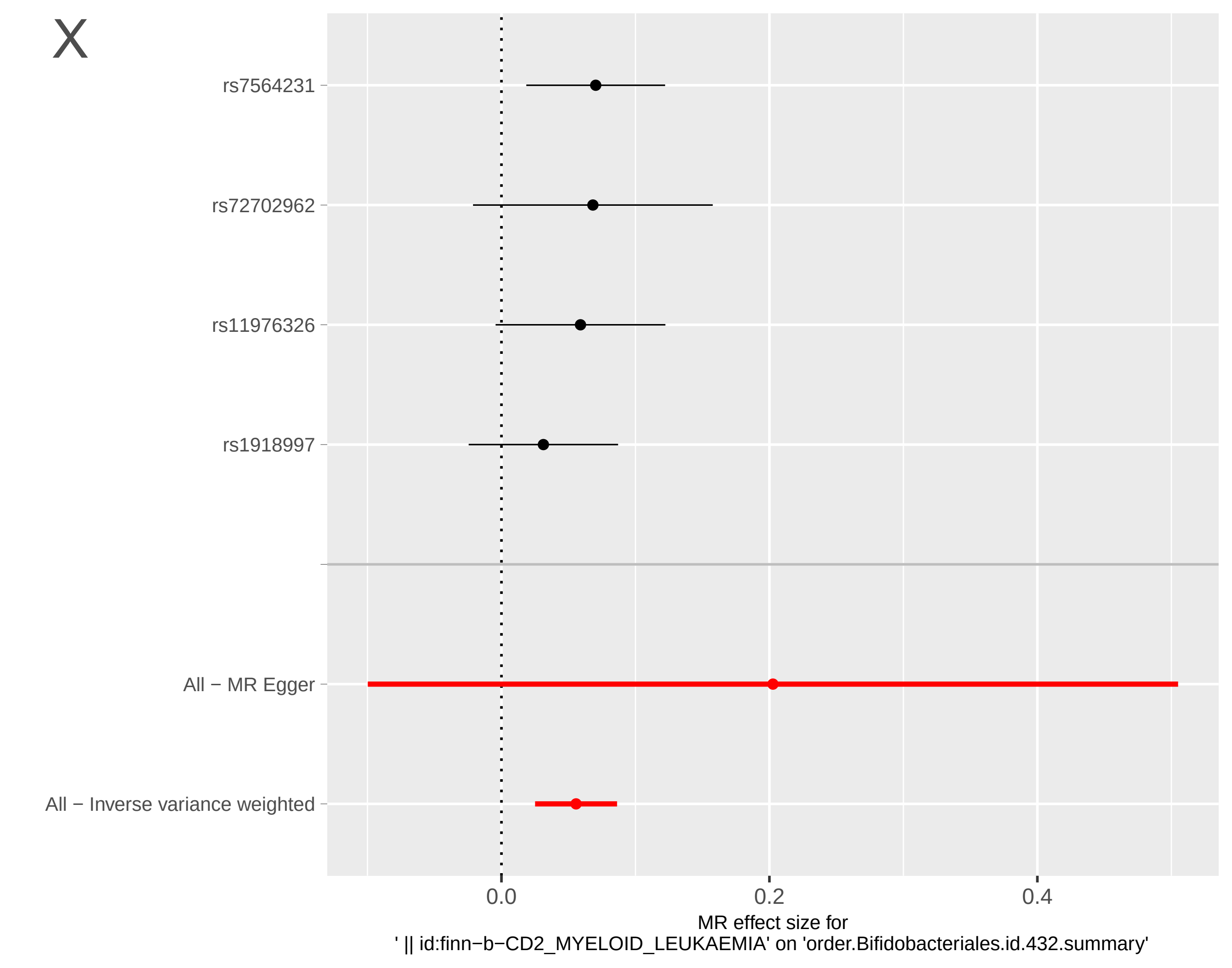
X
rs7564231
rs72702962
rs11976326
rs1918997
All − MR Egger
All − Inverse variance weighted
0.0
0.2
0.4
MR effect size for
' || id:finn−b−CD2_MYELOID_LEUKAEMIA' on 'order.Bifidobacteriales.id.432.summary'

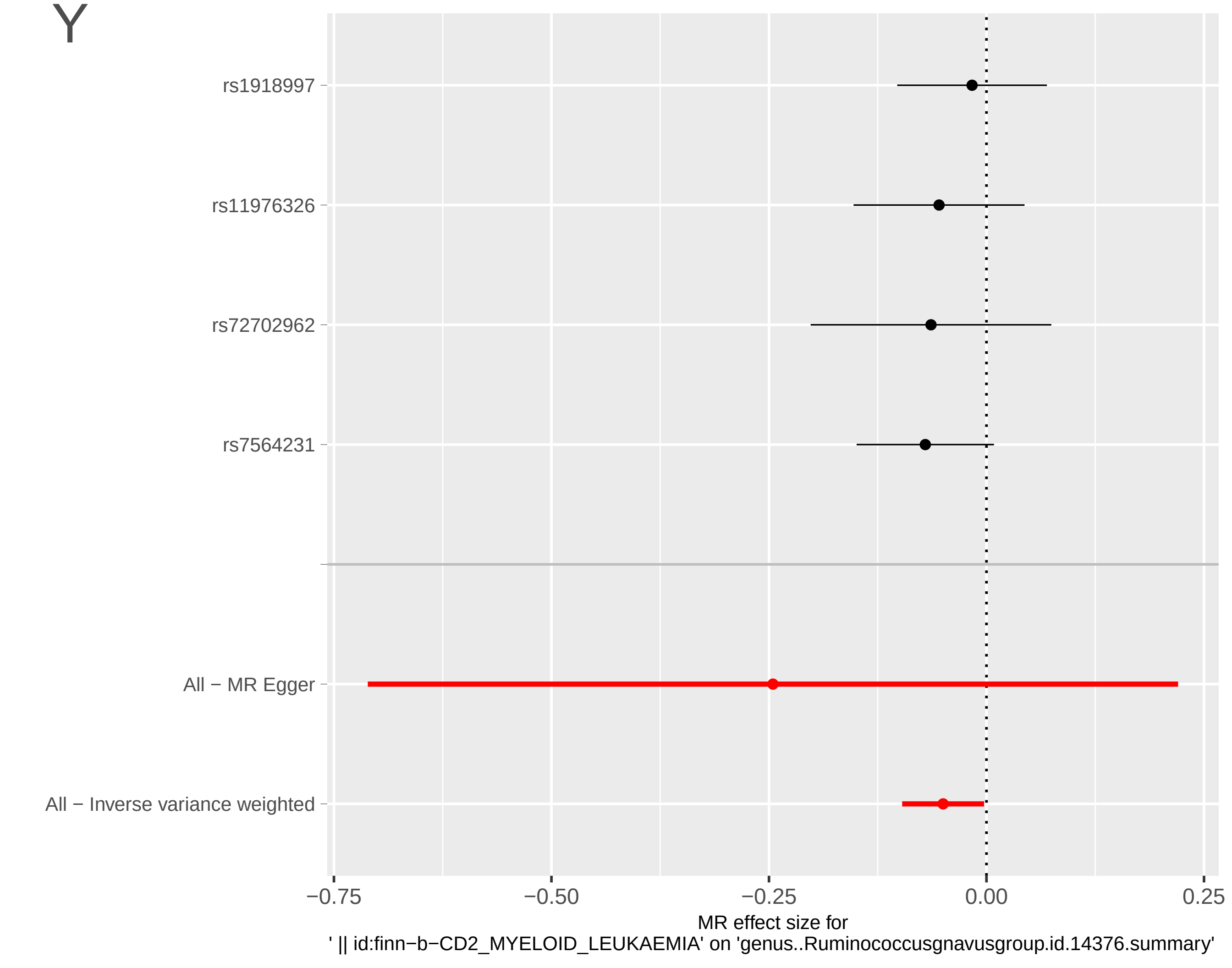
Y
rs1918997
rs11976326
rs72702962
rs7564231
All − MR Egger
All − Inverse variance weighted
−0.75
−0.50
−0.25
0.00
0.25
MR effect size for
' || id:finn−b−CD2_MYELOID_LEUKAEMIA' on 'genus..Ruminococcusgnavusgroup.id.14376.summary'

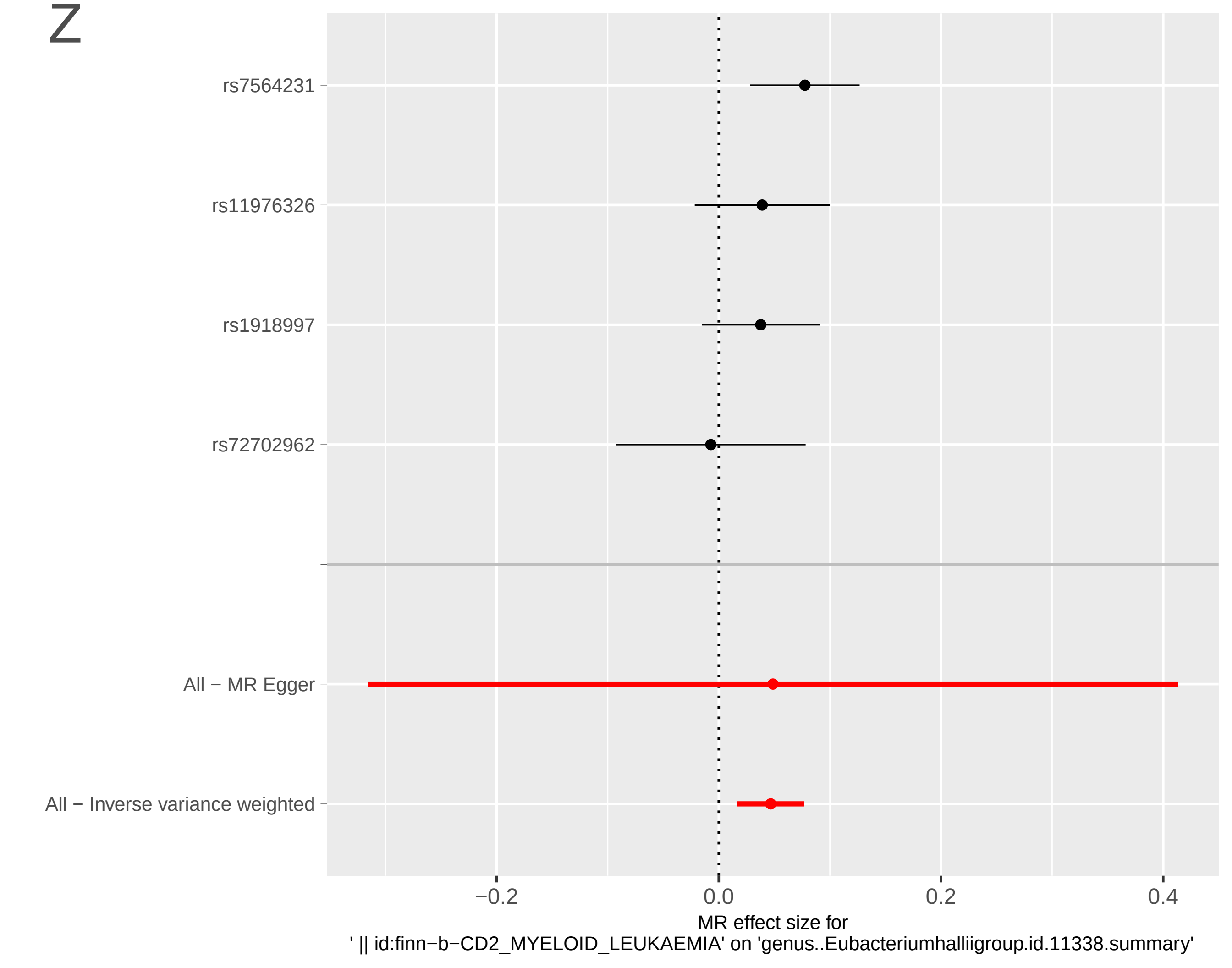
Z
rs7564231
rs11976326
rs1918997
rs72702962
All − MR Egger
All − Inverse variance weighted
−0.2
0.0
0.2
0.4
MR effect size for
' || id:finn−b−CD2_MYELOID_LEUKAEMIA' on 'genus..Eubacteriumhalliigroup.id.11338.summary'

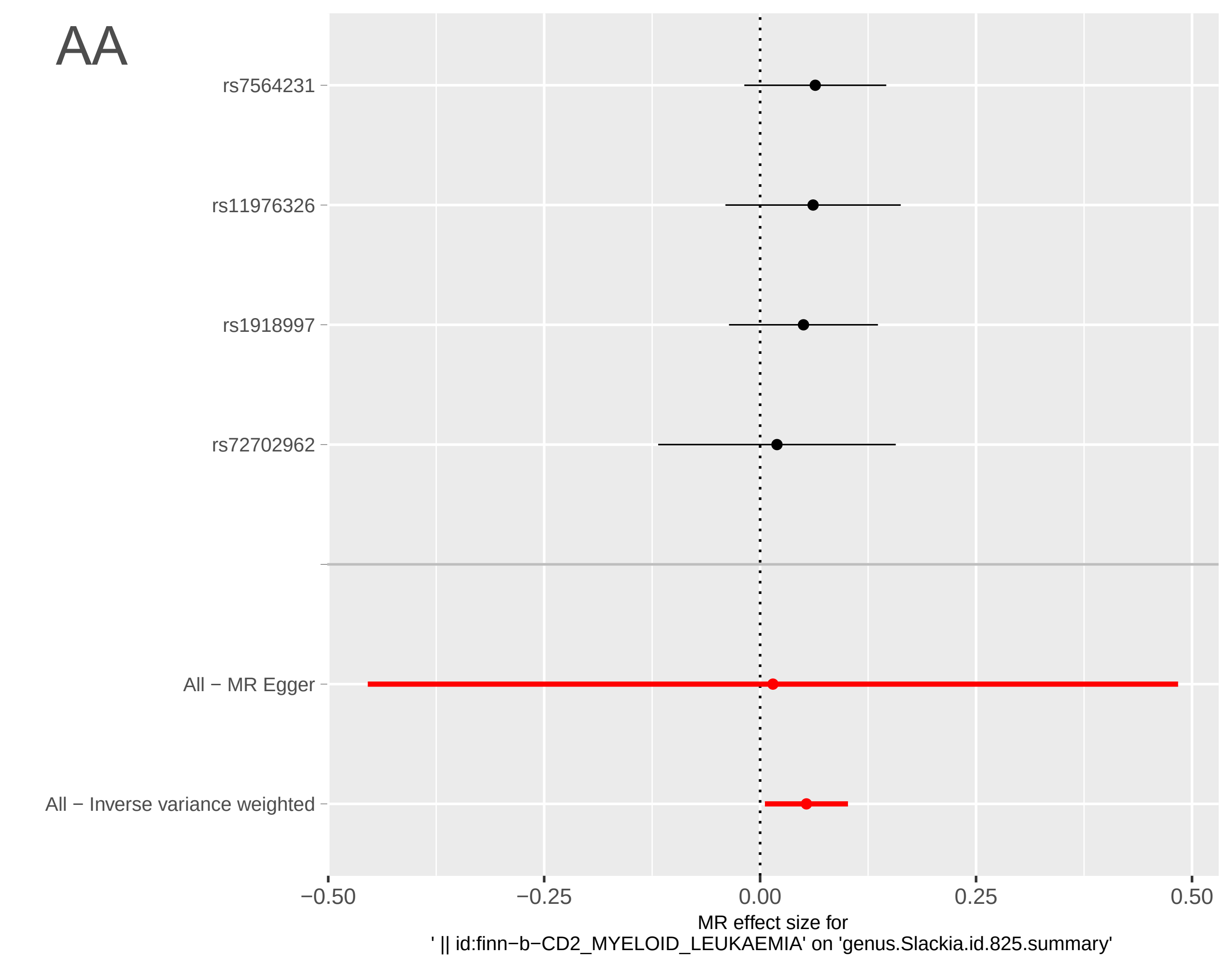
AA
rs7564231
rs11976326
rs1918997
rs72702962
All − MR Egger
All − Inverse variance weighted
−0.50
−0.25
0.00
0.25
0.50
MR effect size for
' || id:finn−b−CD2_MYELOID_LEUKAEMIA' on 'genus.Slackia.id.825.summary'

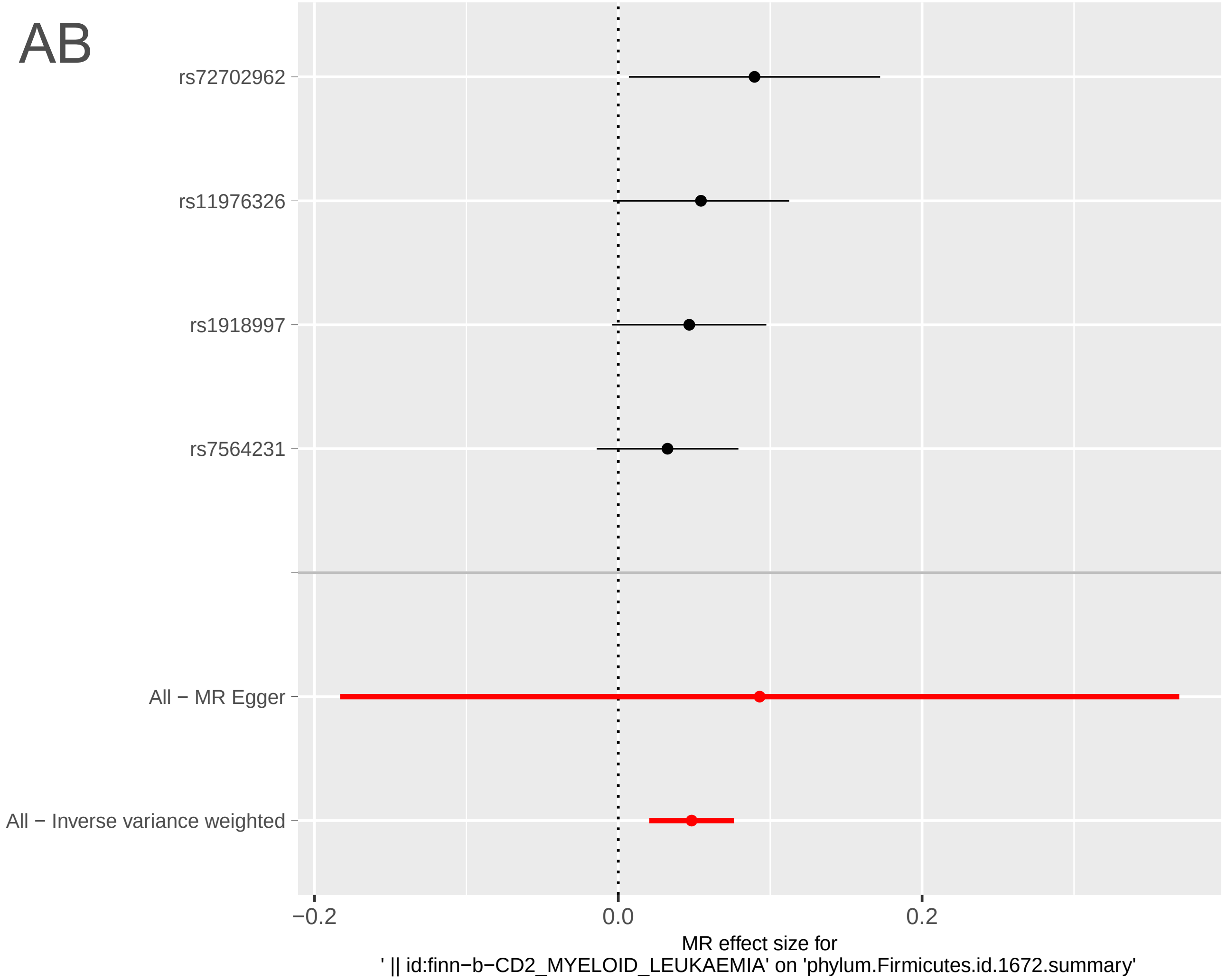
AB
rs72702962
rs11976326
rs1918997
rs7564231
All − MR Egger
All − Inverse variance weighted
−0.2
0.0
0.2
MR effect size for
' || id:finn−b−CD2_MYELOID_LEUKAEMIA' on 'phylum.Firmicutes.id.1672.summary'

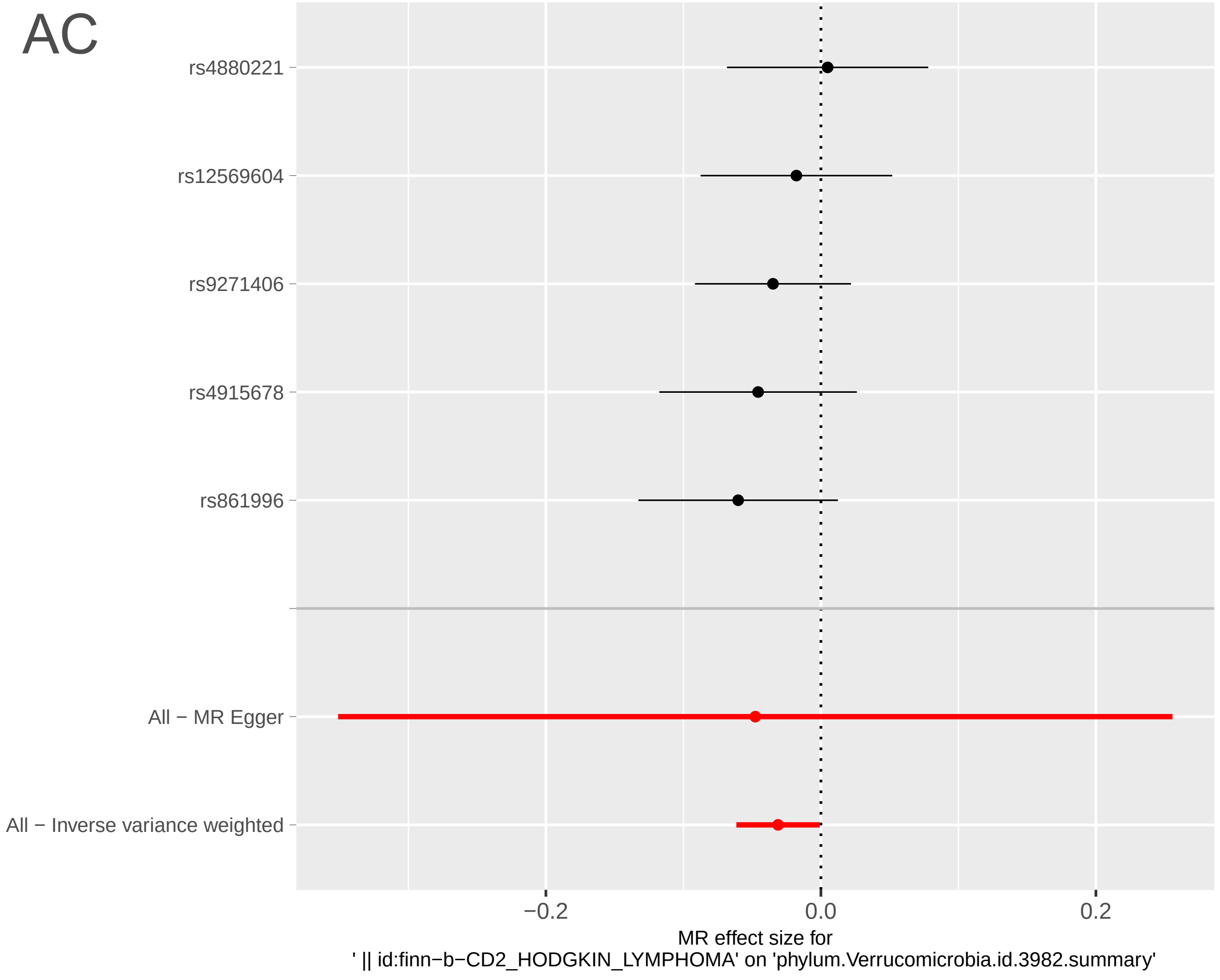
AC
rs4880221
rs12569604
rs9271406
rs4915678
rs861996
All − MR Egger
All − Inverse variance weighted
−0.2
0.0
0.2
MR effect size for
' || id:finn−b−CD2_HODGKIN_LYMPHOMA' on 'phylum.Verrucomicrobia.id.3982.summary'

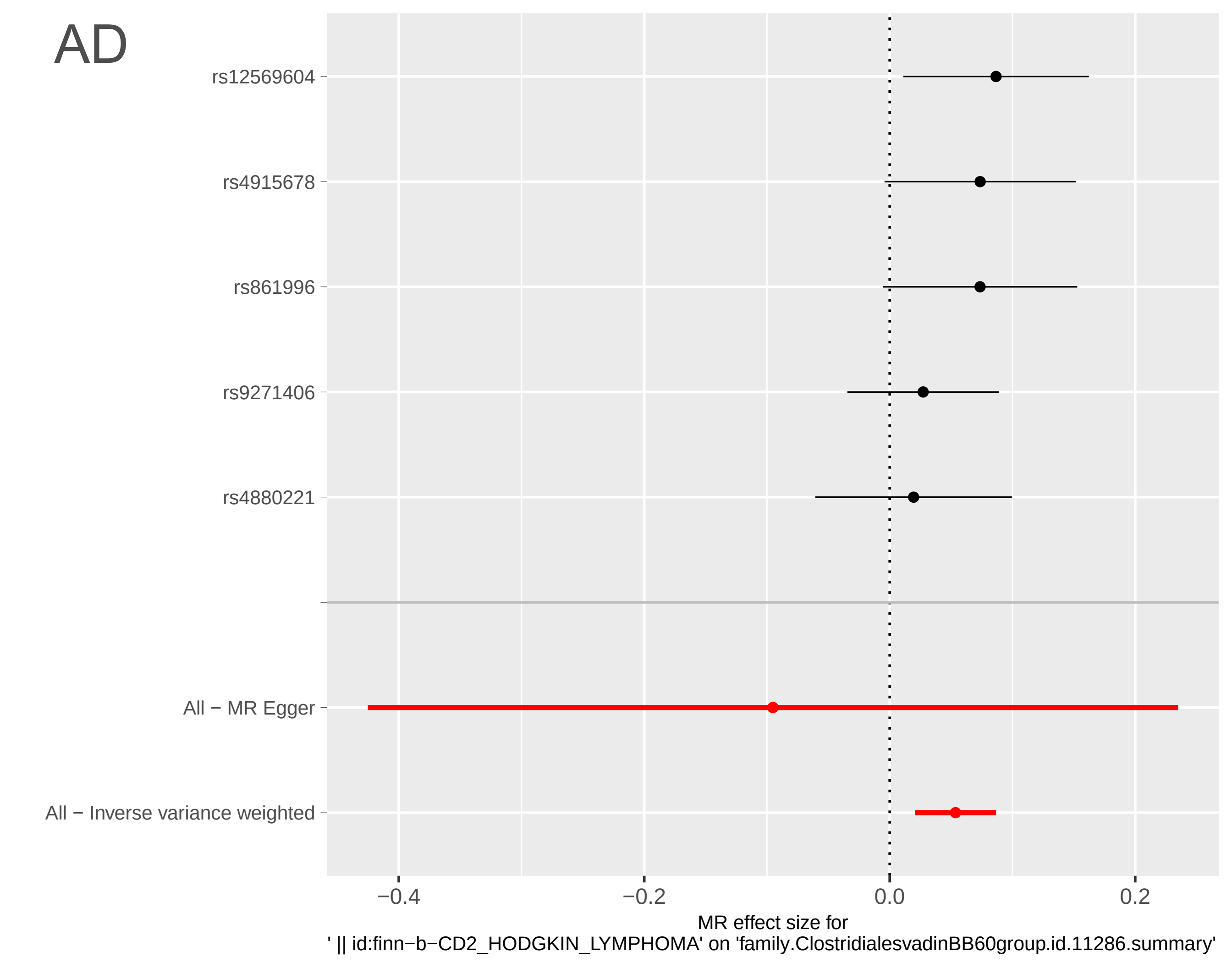
AD
rs12569604
rs4915678
rs861996
rs9271406
rs4880221
All − MR Egger
All − Inverse variance weighted
−0.4
−0.2
0.0
0.2
MR effect size for
' || id:finn−b−CD2_HODGKIN_LYMPHOMA' on 'family.ClostridialesvadinBB60group.id.11286.summary'

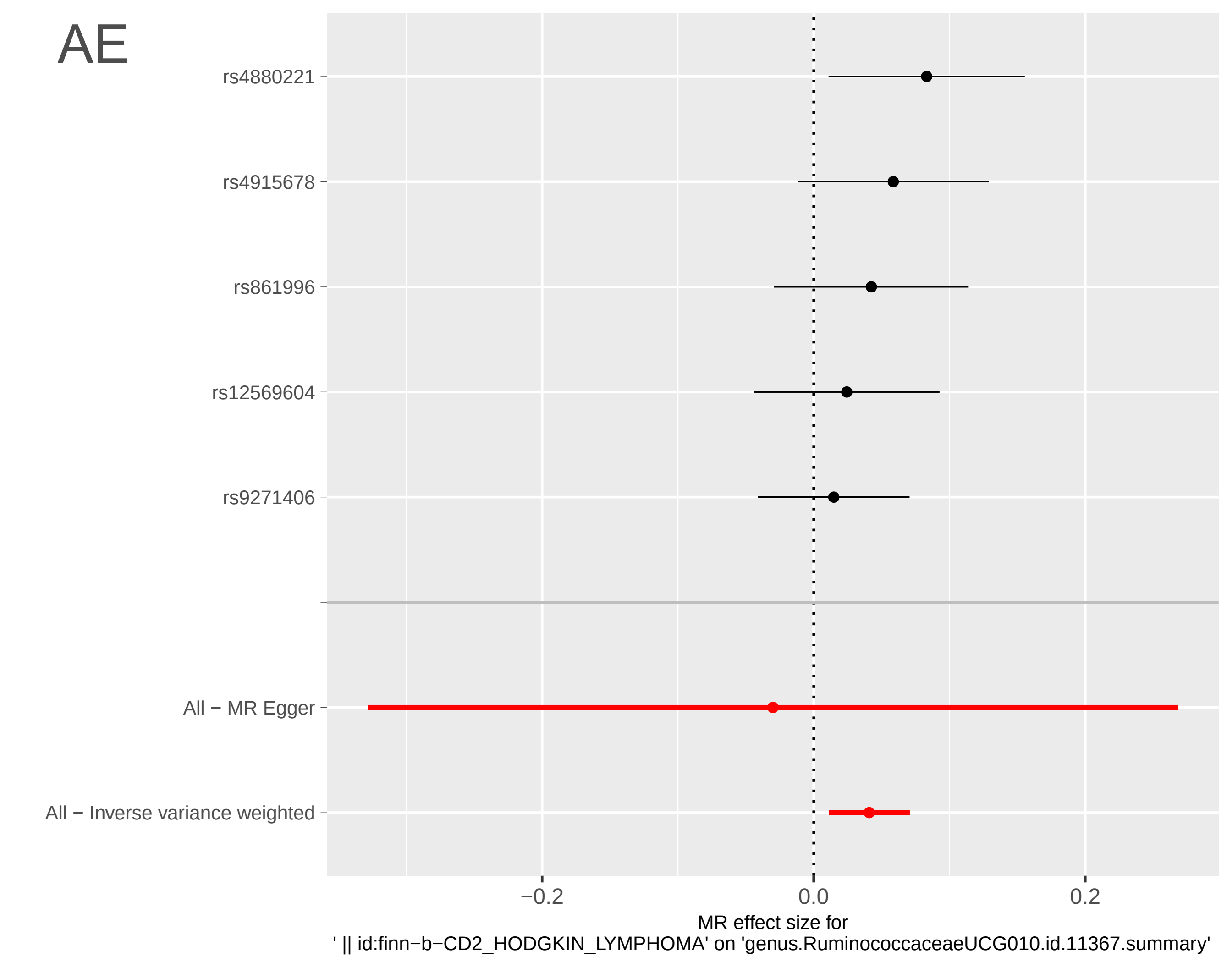
AE
rs4880221
rs4915678
rs861996
rs12569604
rs9271406
All − MR Egger
All − Inverse variance weighted
−0.2
0.0
0.2
MR effect size for
' || id:finn−b−CD2_HODGKIN_LYMPHOMA' on 'genus.RuminococcaceaeUCG010.id.11367.summary'

AF

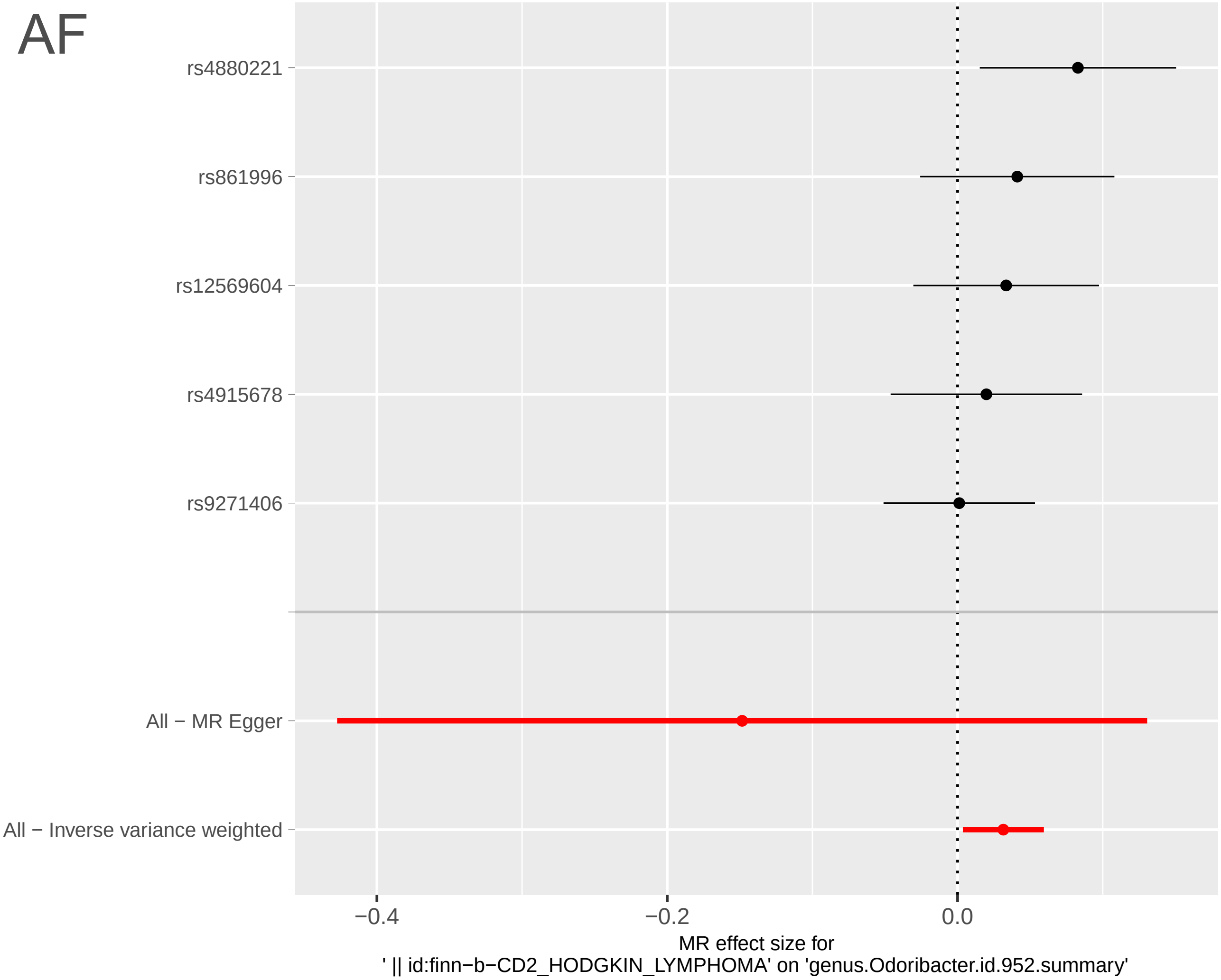

rs4880221
rs861996
rs12569604
rs4915678
rs9271406
All − MR Egger
All − Inverse variance weighted
−0.4
−0.2
0.0
MR effect size for
' || id:finn−b−CD2_HODGKIN_LYMPHOMA' on 'genus.Odoribacter.id.952.summary'

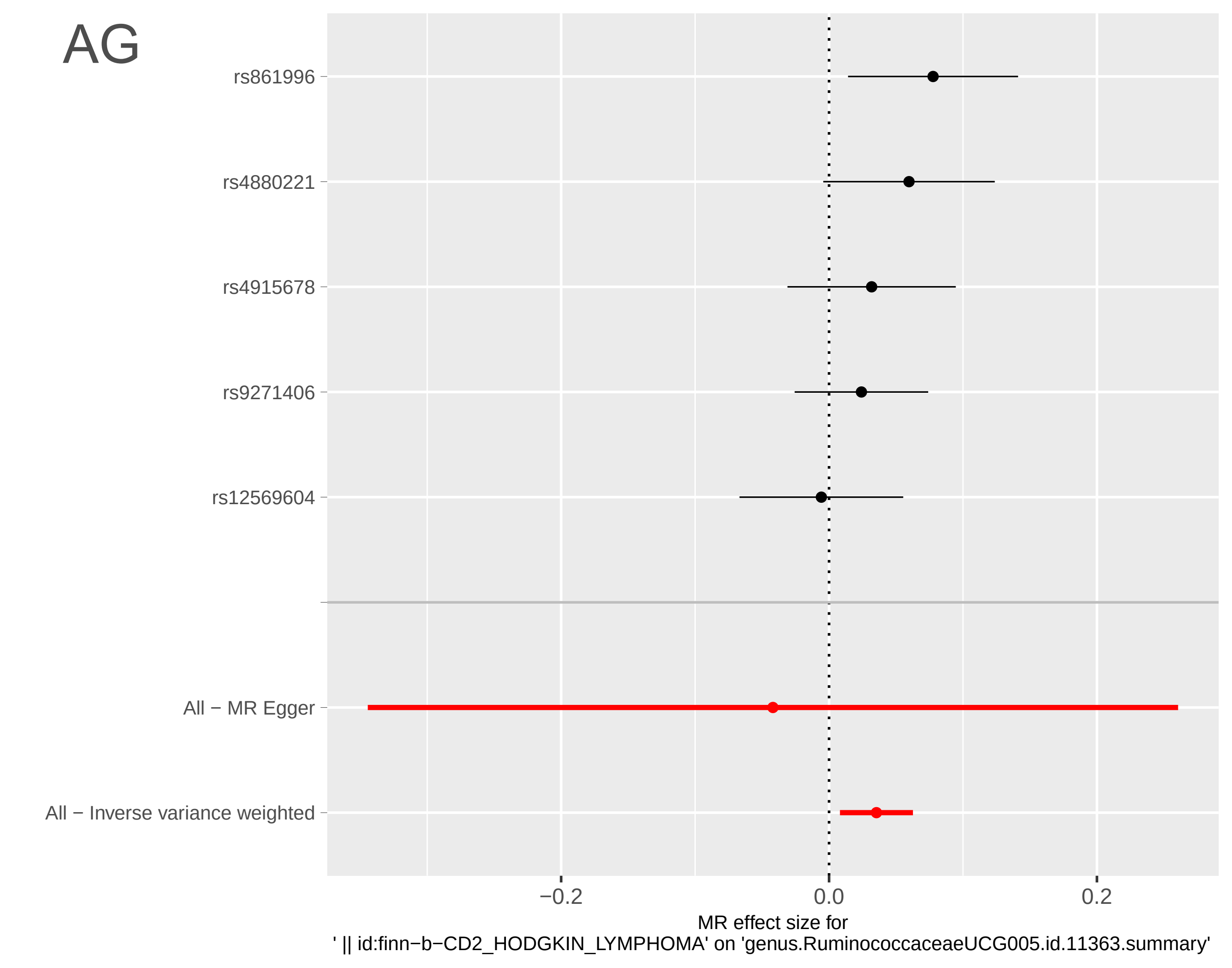
AG
rs861996
rs4880221
rs4915678
rs9271406
rs12569604
All − MR Egger
All − Inverse variance weighted
−0.2
0.0
0.2
MR effect size for
' || id:finn−b−CD2_HODGKIN_LYMPHOMA' on 'genus.RuminococcaceaeUCG005.id.11363.summary'

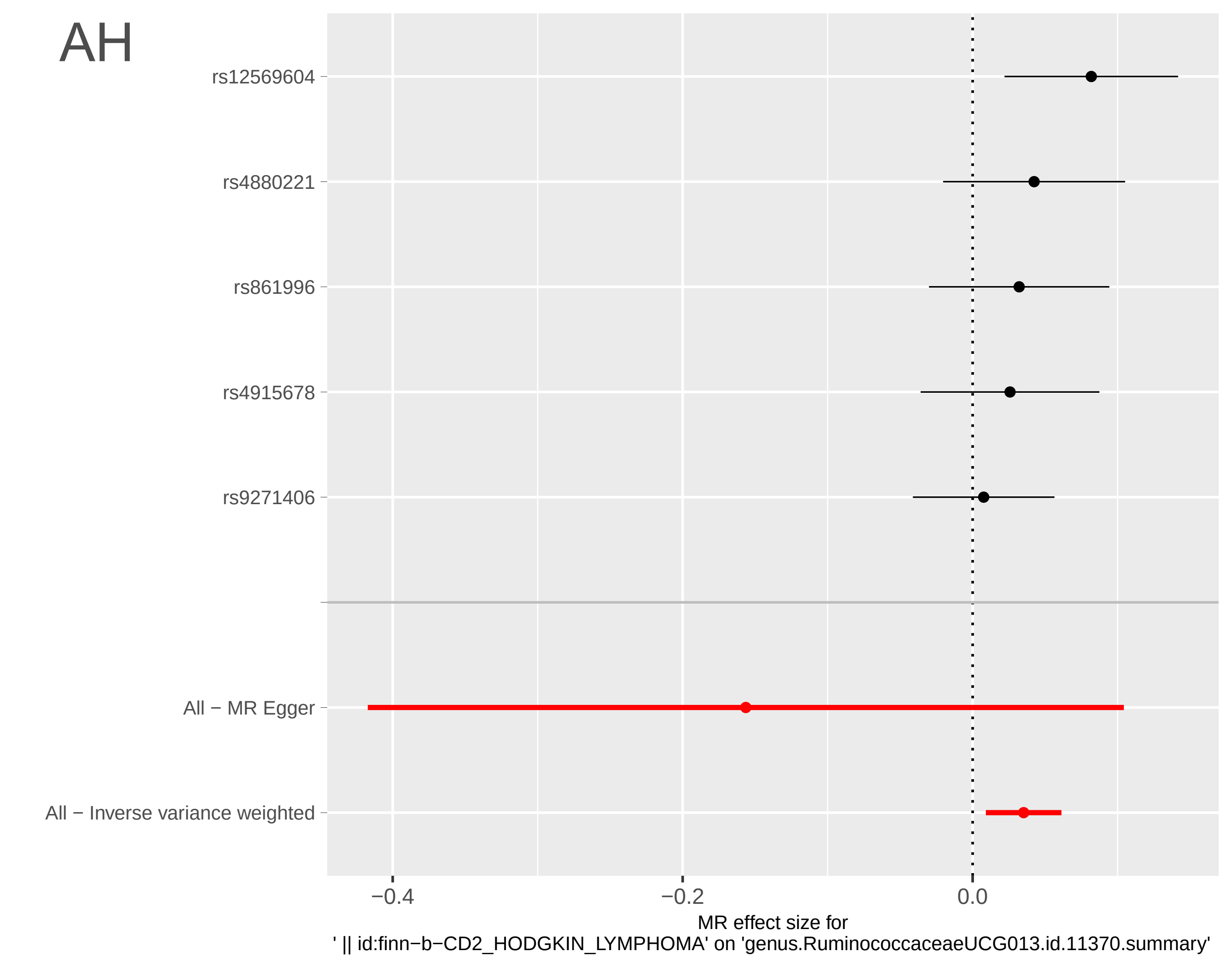
AH
rs12569604
rs4880221
rs861996
rs4915678
rs9271406
All − MR Egger
All − Inverse variance weighted
−0.4
−0.2
0.0
MR effect size for
' || id:finn−b−CD2_HODGKIN_LYMPHOMA' on 'genus.RuminococcaceaeUCG013.id.11370.summary'

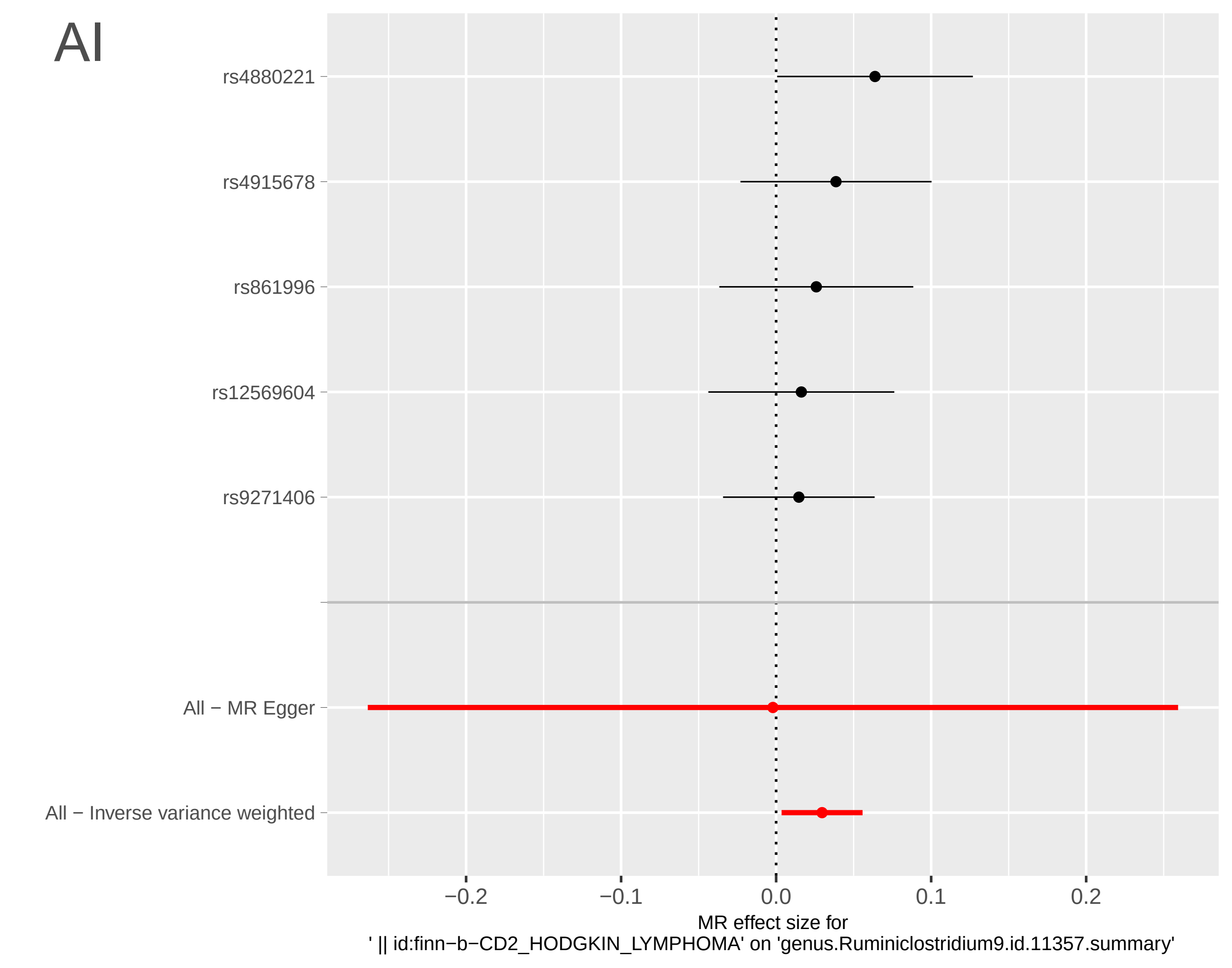
AI
rs4880221
rs4915678
rs861996
rs12569604
rs9271406
All − MR Egger
All − Inverse variance weighted
−0.2
−0.1
0.0
0.1
0.2
MR effect size for
' || id:finn−b−CD2_HODGKIN_LYMPHOMA' on 'genus.Ruminiclostridium9.id.11357.summary'

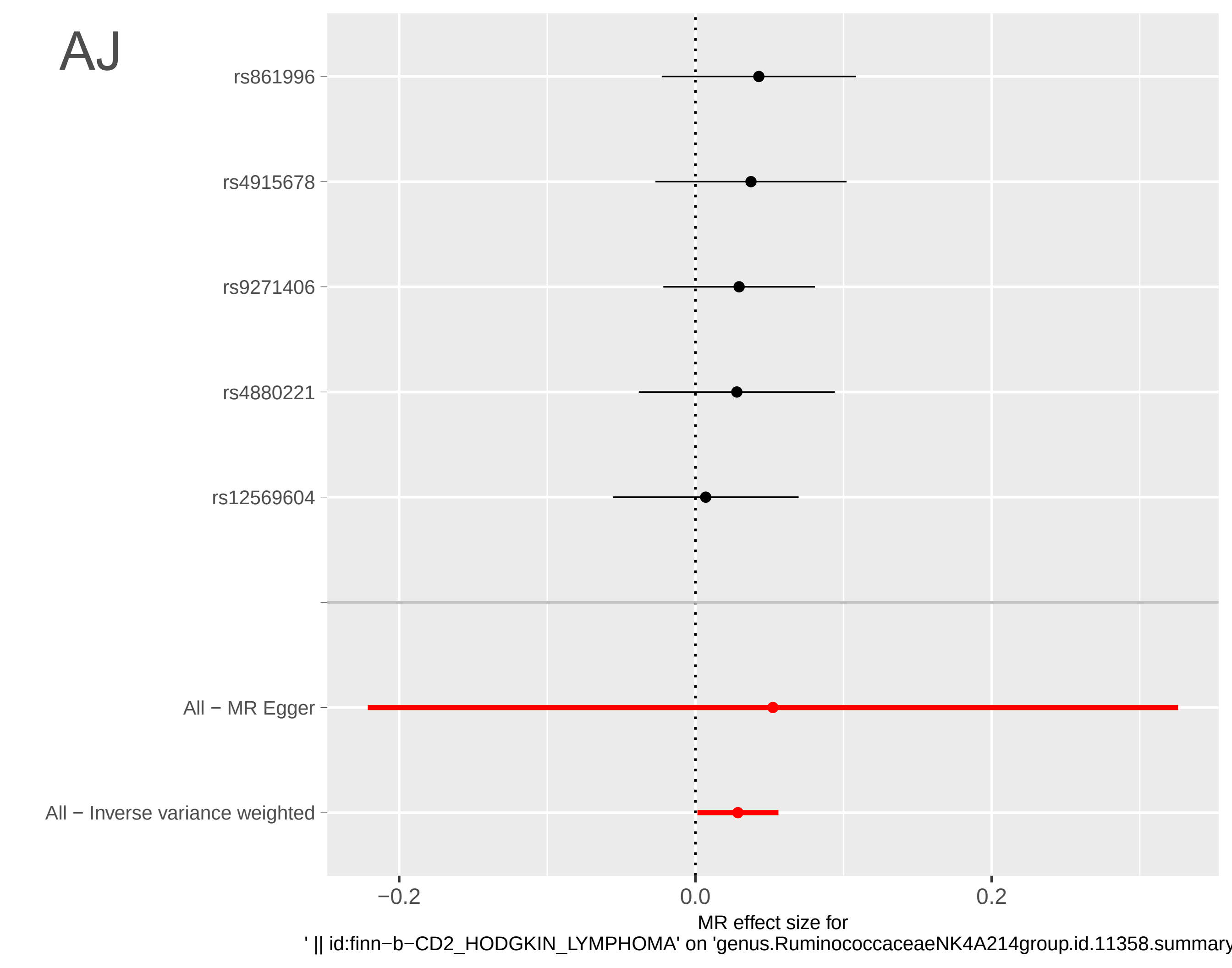
AJ
rs861996
rs4915678
rs9271406
rs4880221
rs12569604
All − MR Egger
All − Inverse variance weighted
−0.2
0.0
0.2
MR effect size for
' || id:finn−b−CD2_HODGKIN_LYMPHOMA' on 'genus.RuminococcaceaeNK4A214group.id.11358.summary

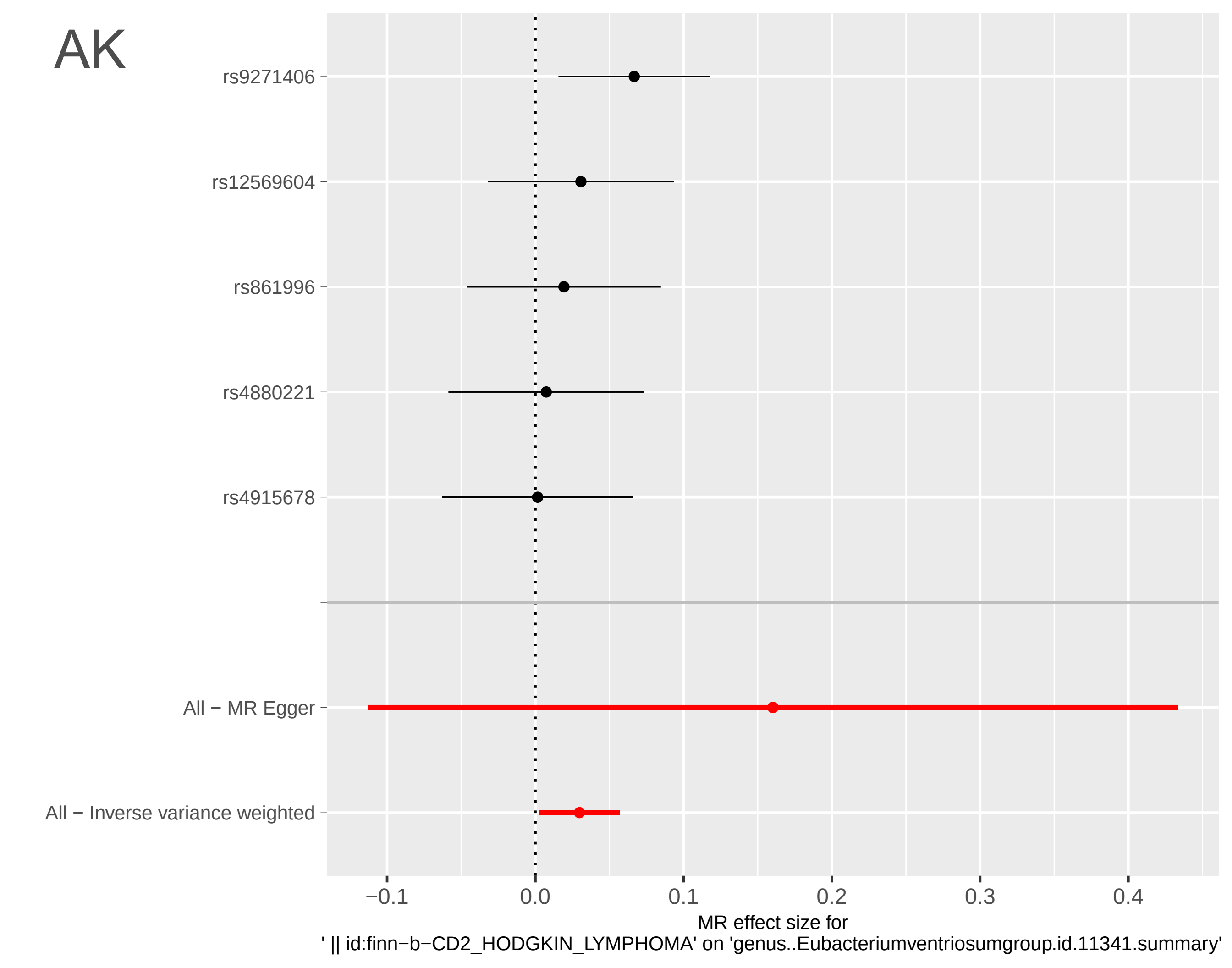
AK
rs9271406
rs12569604
rs861996
rs4880221
rs4915678
All − MR Egger
All − Inverse variance weighted
−0.1
0.0
0.1
0.2
0.3
0.4
MR effect size for
' || id:finn−b−CD2_HODGKIN_LYMPHOMA' on 'genus..Eubacteriumventriosumgroup.id.11341.summary'

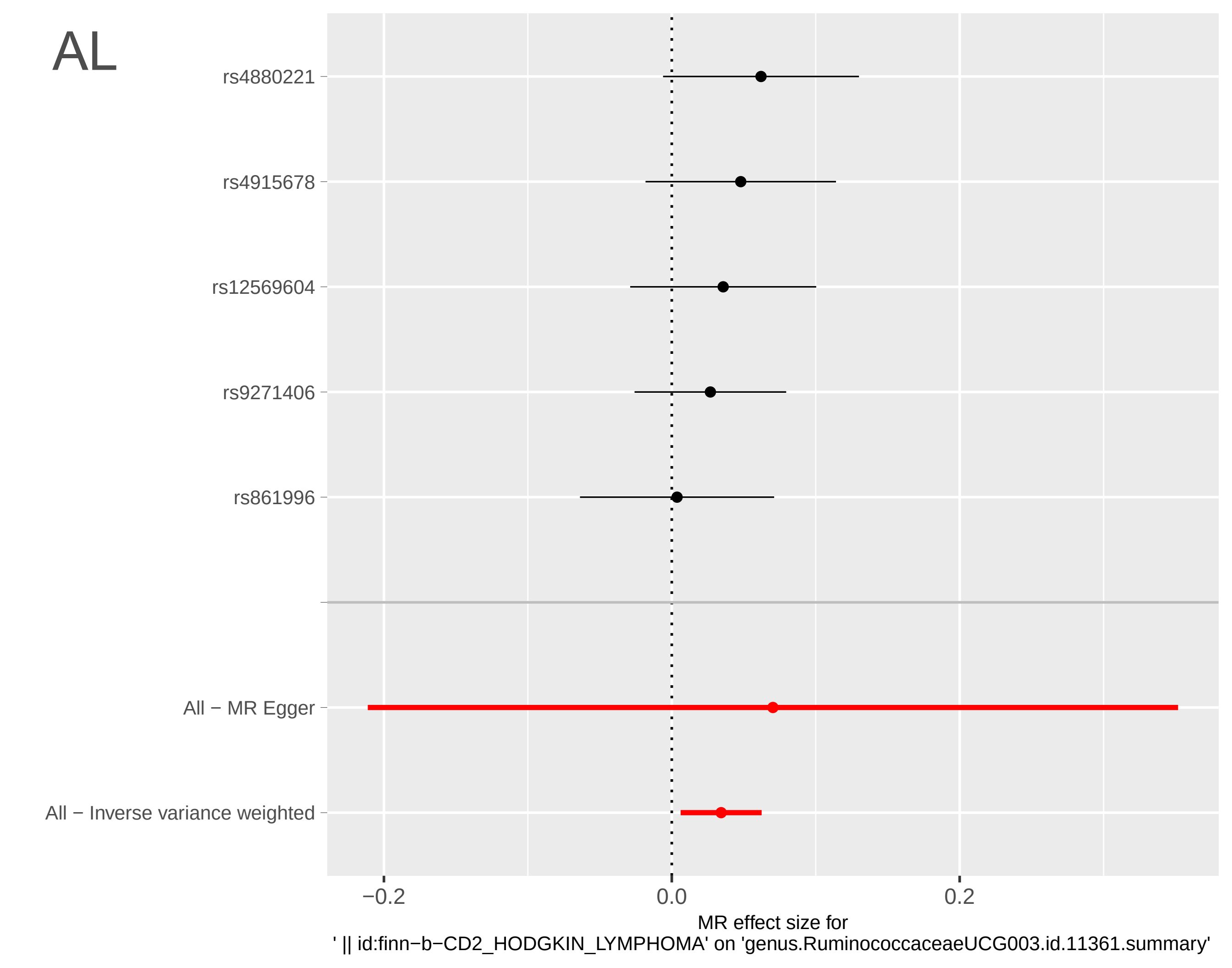
AL
rs4880221
rs4915678
rs12569604
rs9271406
rs861996
All − MR Egger
All − Inverse variance weighted
−0.2
0.0
0.2
MR effect size for
' || id:finn−b−CD2_HODGKIN_LYMPHOMA' on 'genus.RuminococcaceaeUCG003.id.11361.summary'

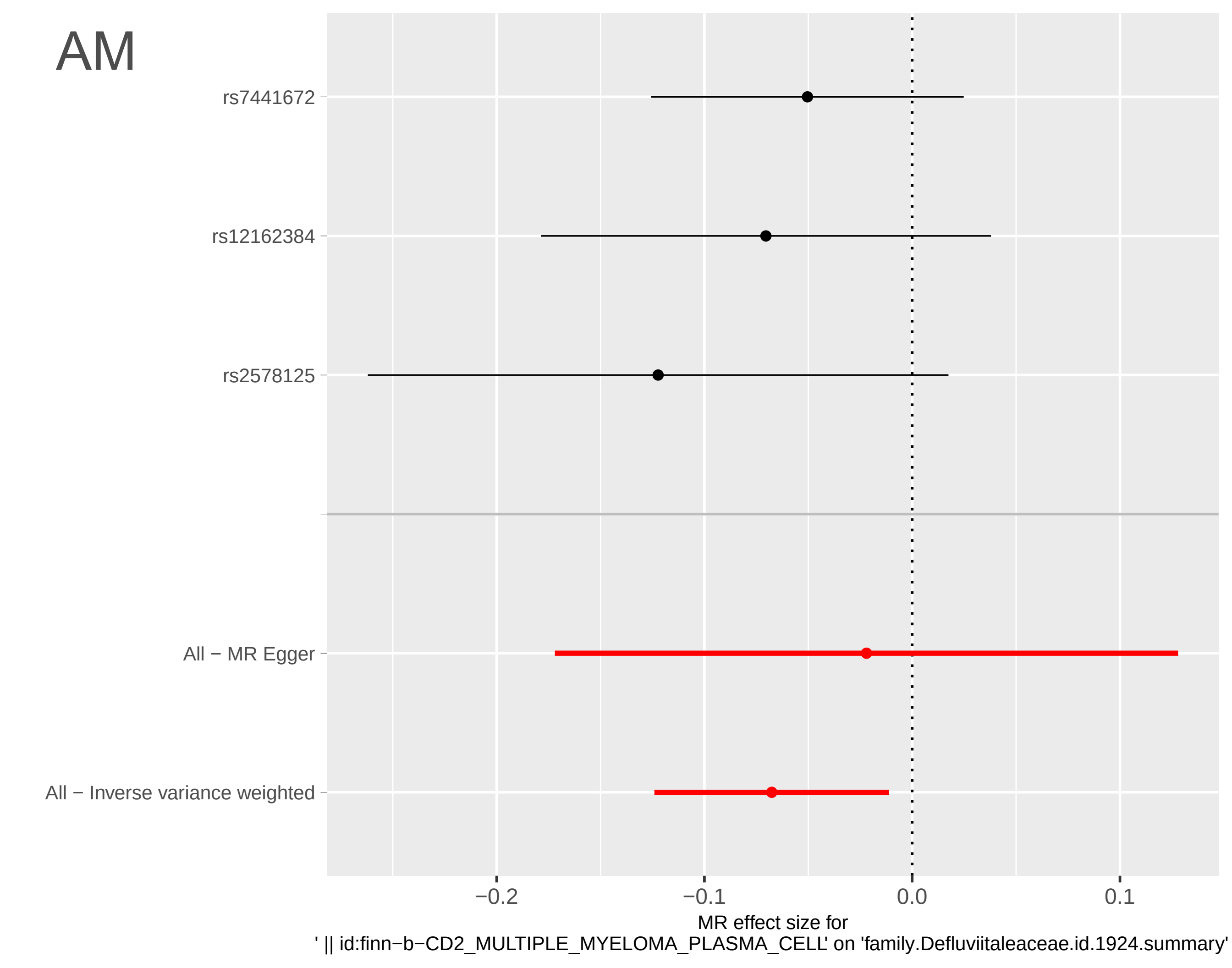
AM
rs7441672
rs12162384
rs2578125
All − MR Egger
All − Inverse variance weighted
−0.2
−0.1
0.0
0.1
MR effect size for
' || id:finn−b−CD2_MULTIPLE_MYELOMA_PLASMA_CELL' on 'family.Defluviitaleaceae.id.1924.summary'

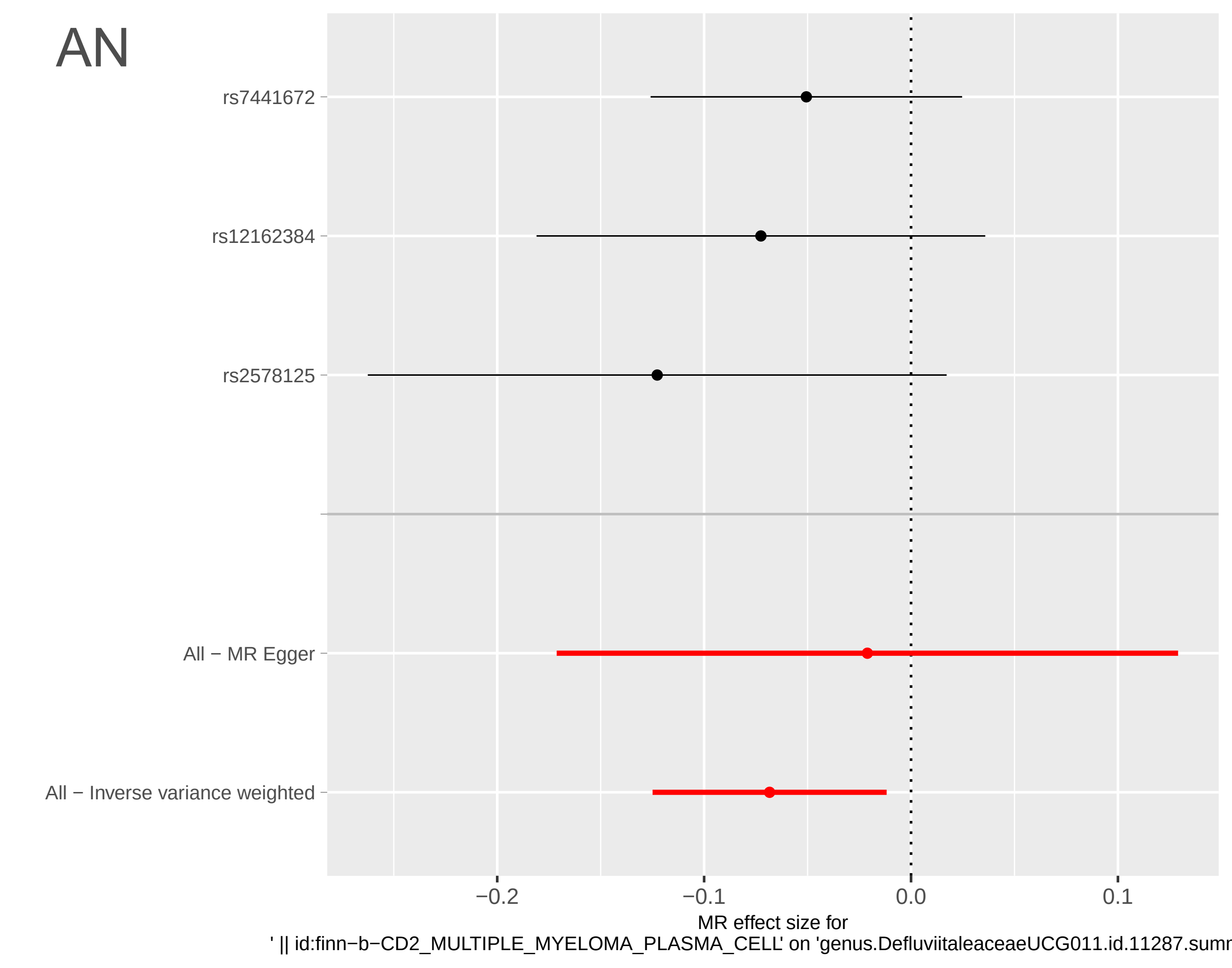
AN
rs7441672
rs12162384
rs2578125
All − MR Egger
All − Inverse variance weighted
−0.2
−0.1
0.0
0.1
MR effect size for
' || id:finn−b−CD2_MULTIPLE_MYELOMA_PLASMA_CELL' on 'genus.DefluviitaleaceaeUCG011.id.11287.sumr

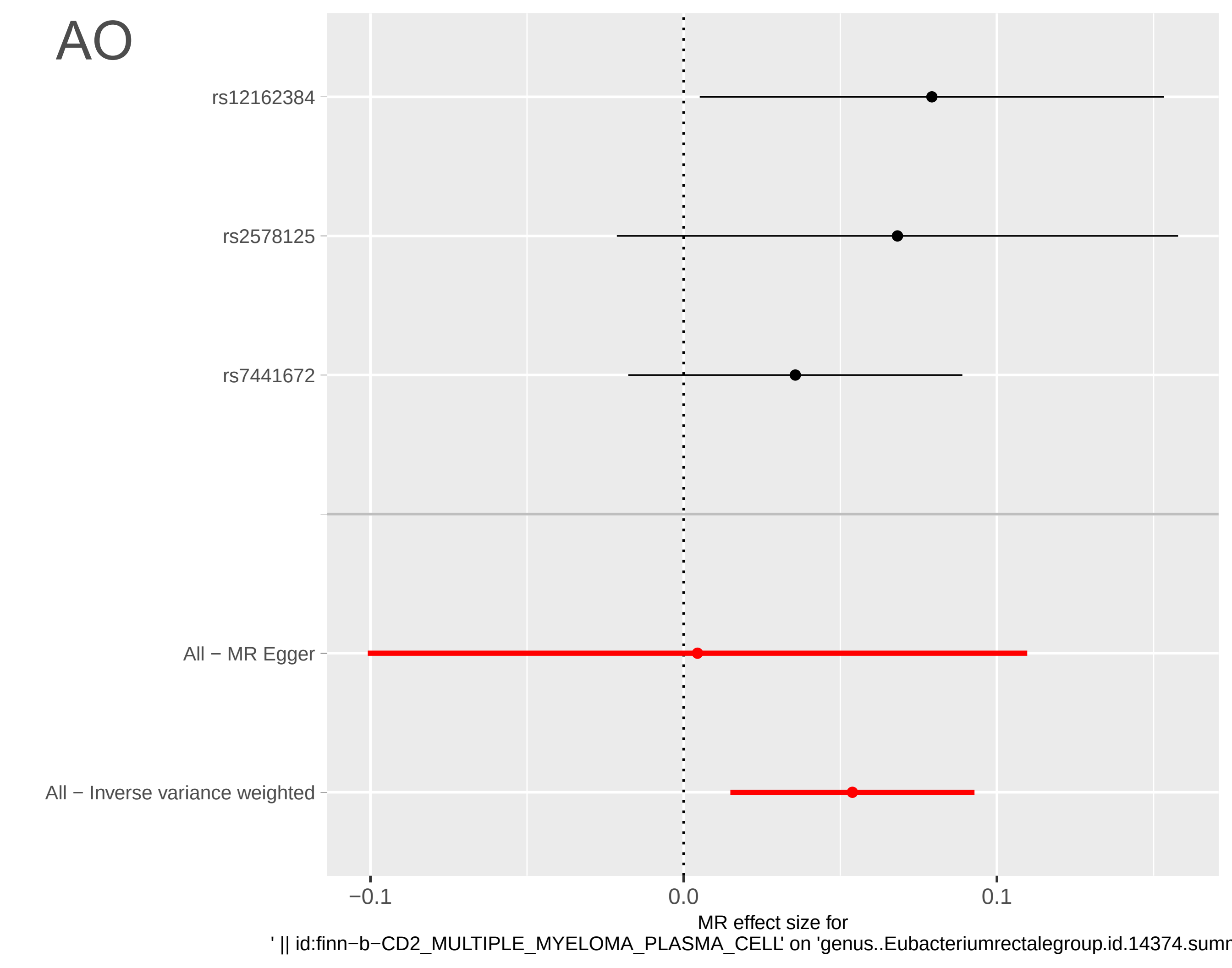
AO
rs12162384
rs2578125
rs7441672
All − MR Egger
All − Inverse variance weighted
−0.1
0.0
0.1
MR effect size for
' || id:finn−b−CD2_MULTIPLE_MYELOMA_PLASMA_CELL' on 'genus..Eubacteriumrectalegroup.id.14374.summ

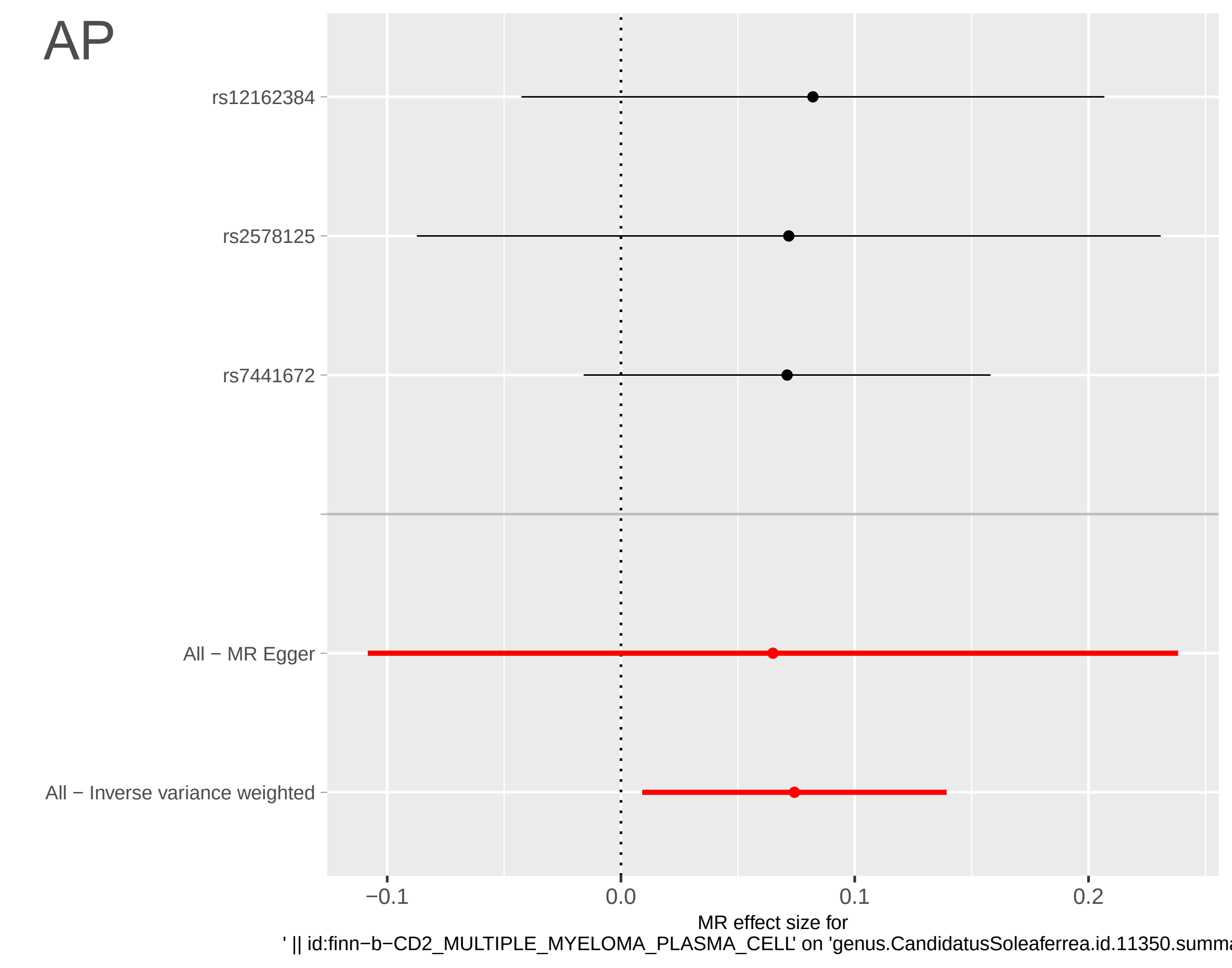
AP
rs12162384
rs2578125
rs7441672
All − MR Egger
All − Inverse variance weighted
−0.1
0.0
0.1
0.2
MR effect size for
' || id:finn−b−CD2_MULTIPLE_MYELOMA_PLASMA_CELL' on 'genus.CandidatusSoleaferrea.id.11350.summa

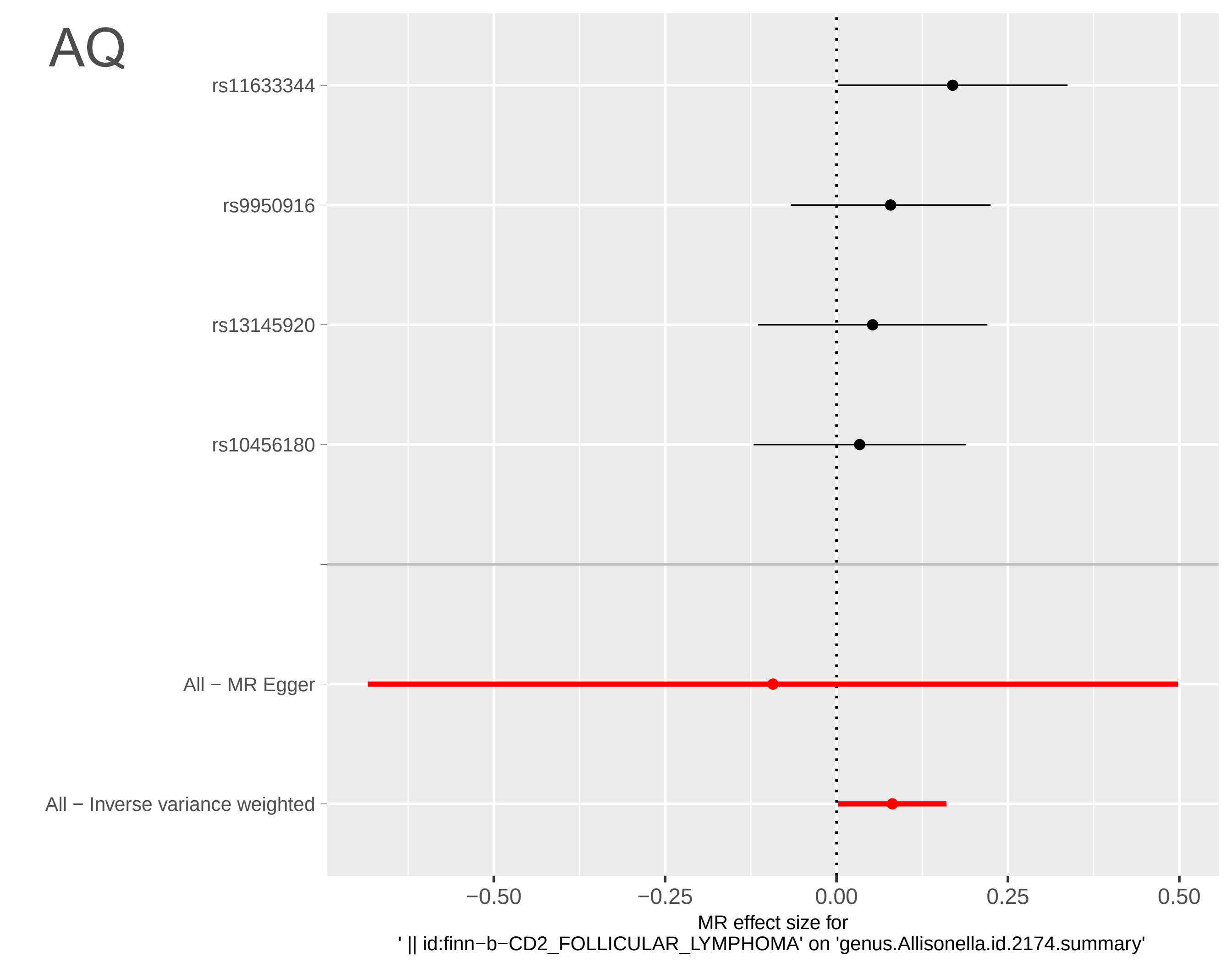
AQ
rs11633344
rs9950916
rs13145920
rs10456180
All − MR Egger
All − Inverse variance weighted
−0.50
−0.25
0.00
0.25
0.50
MR effect size for
' || id:finn−b−CD2_FOLLICULAR_LYMPHOMA' on 'genus.Allisonella.id.2174.summary'

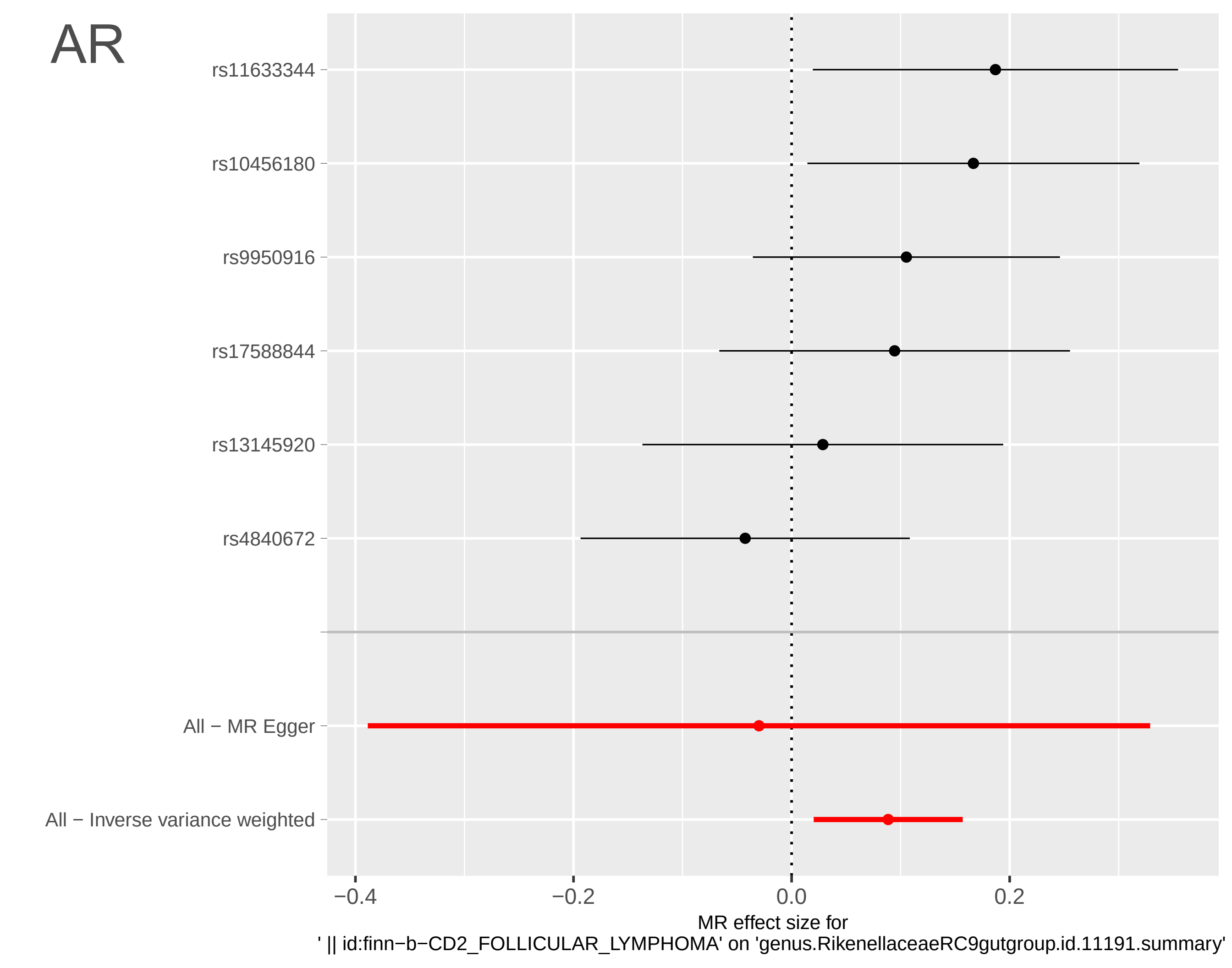
AR
rs11633344
rs10456180
rs9950916
rs17588844
rs13145920
rs4840672
All − MR Egger
All − Inverse variance weighted
−0.4
−0.2
0.0
0.2
MR effect size for
' || id:finn−b−CD2_FOLLICULAR_LYMPHOMA' on 'genus.RikenellaceaeRC9gutgroup.id.11191.summary'

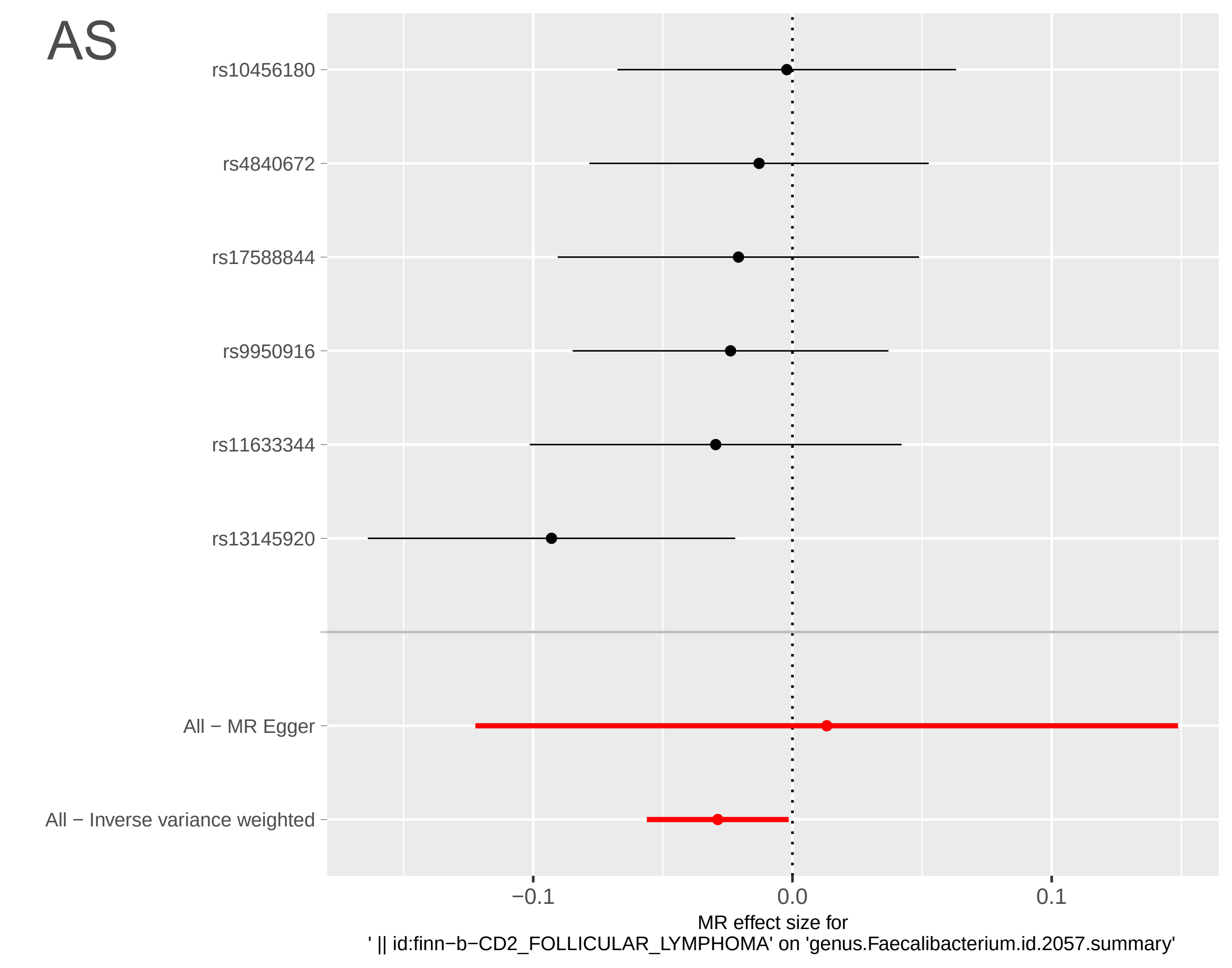
AS
rs10456180
rs4840672
rs17588844
rs9950916
rs11633344
rs13145920
All − MR Egger
All − Inverse variance weighted
−0.1
0.0
0.1
MR effect size for
' || id:finn−b−CD2_FOLLICULAR_LYMPHOMA' on 'genus.Faecalibacterium.id.2057.summary'

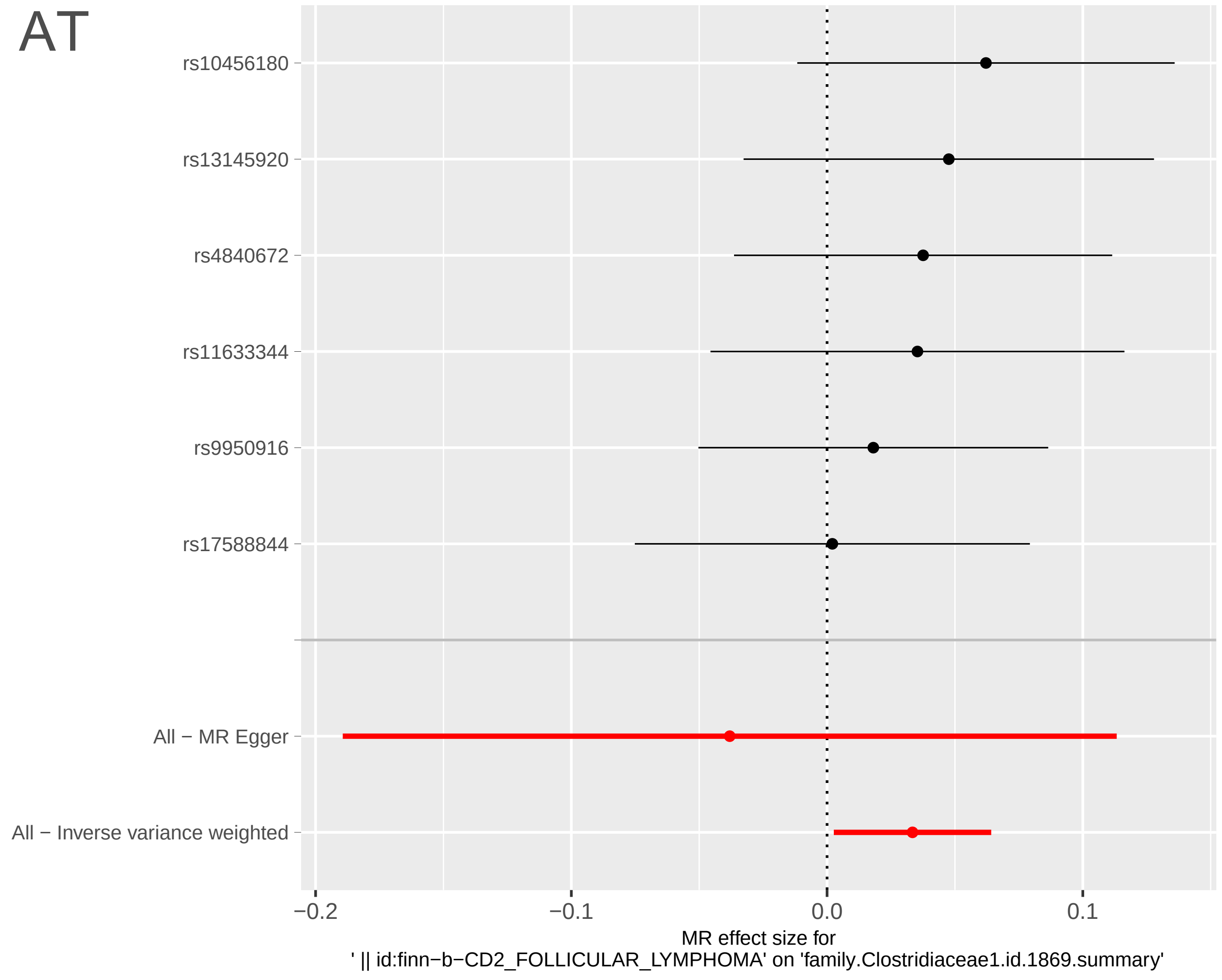
AT
rs10456180
rs13145920
rs4840672
rs11633344
rs9950916
rs17588844
All − MR Egger
All − Inverse variance weighted
−0.2
−0.1
0.0
0.1
MR effect size for
' || id:finn−b−CD2_FOLLICULAR_LYMPHOMA' on 'family.Clostridiaceae1.id.1869.summary'

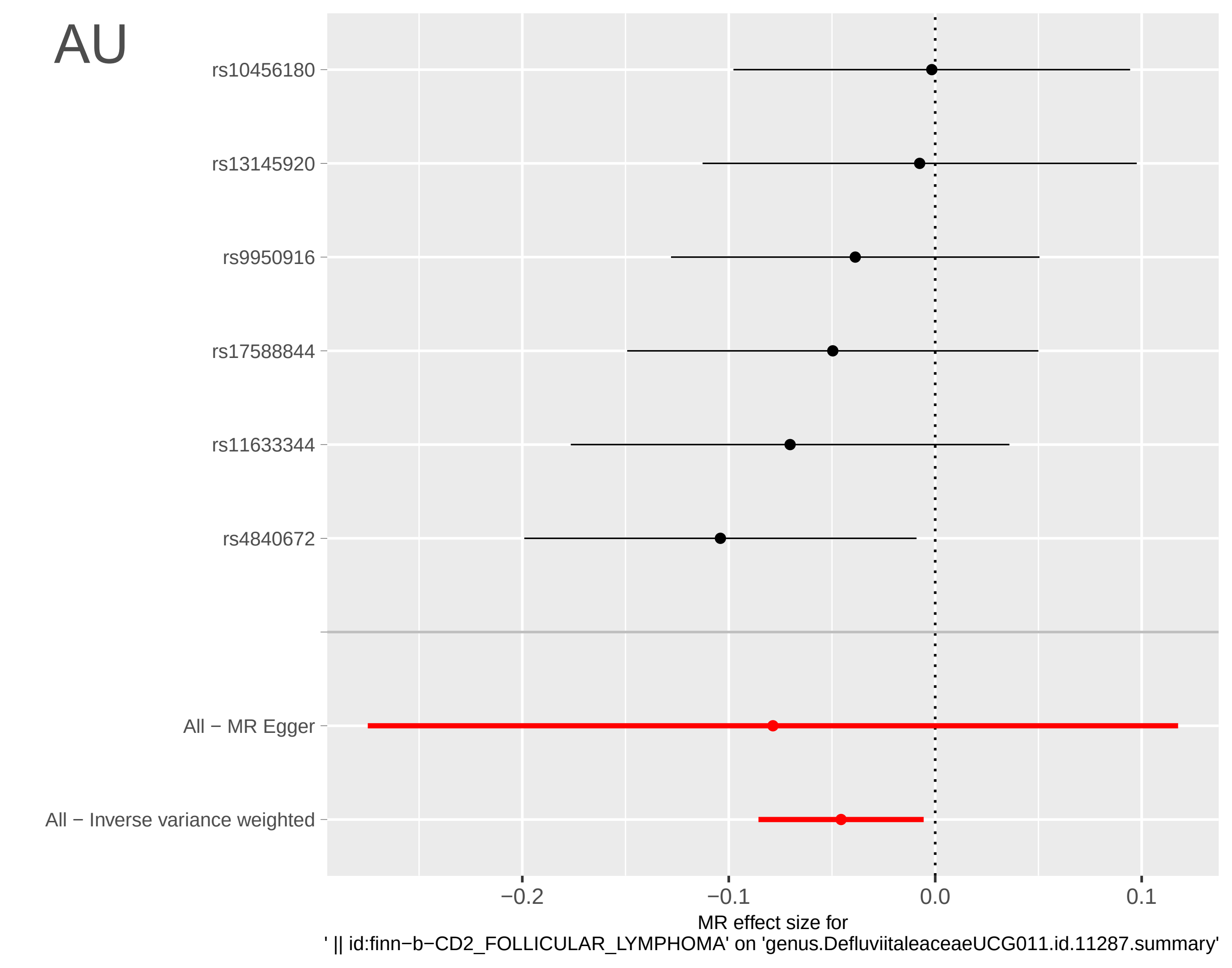
AU
rs10456180
rs13145920
rs9950916
rs17588844
rs11633344
rs4840672
All − MR Egger
All − Inverse variance weighted
−0.2
−0.1
0.0
0.1
MR effect size for
' || id:finn−b−CD2_FOLLICULAR_LYMPHOMA' on 'genus.DefluviitaleaceaeUCG011.id.11287.summary'

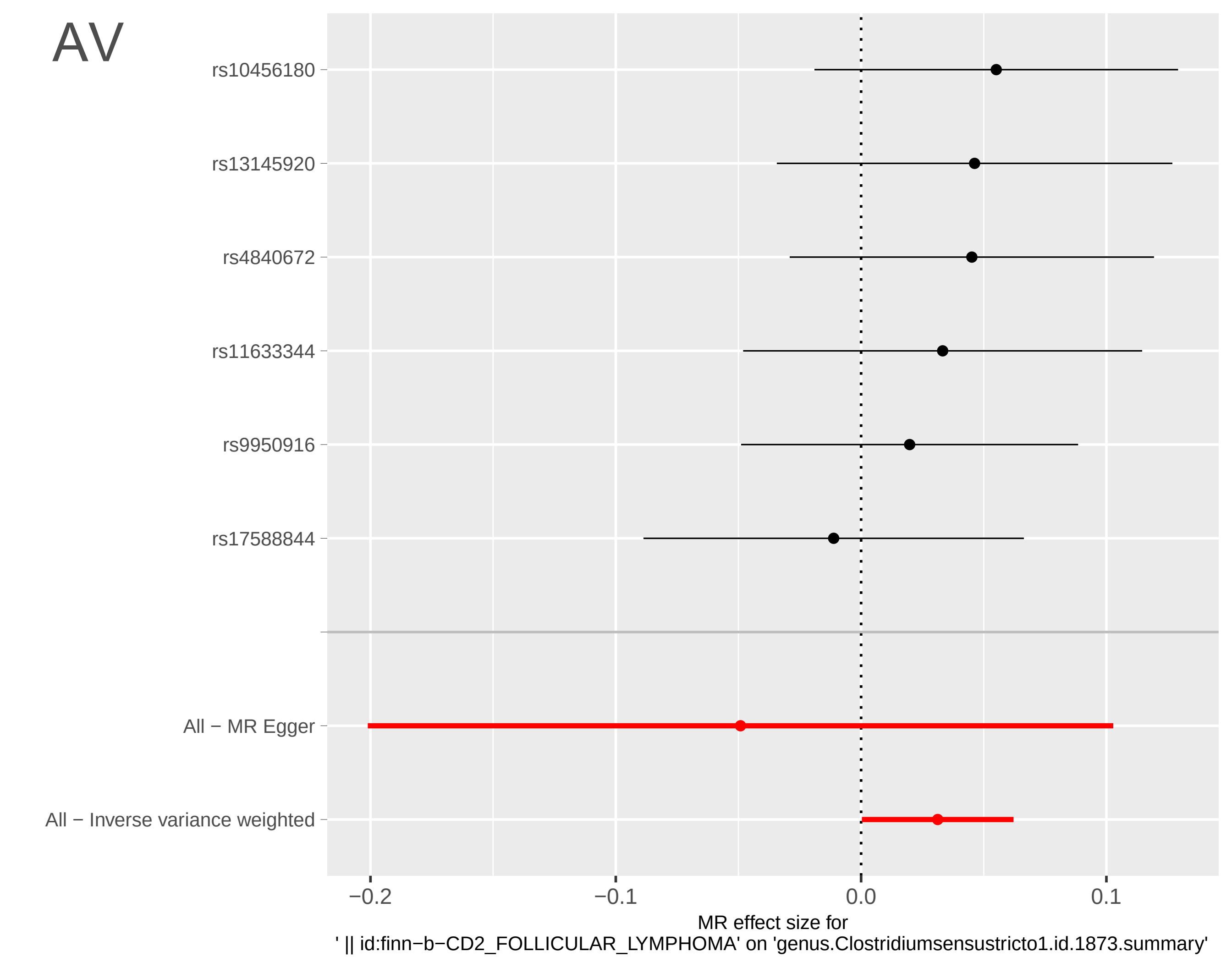
AV
rs10456180
rs13145920
rs4840672
rs11633344
rs9950916
rs17588844
All − MR Egger
All − Inverse variance weighted
−0.2
−0.1
0.0
0.1
MR effect size for
' || id:finn−b−CD2_FOLLICULAR_LYMPHOMA' on 'genus.Clostridiumsensustricto1.id.1873.summary'

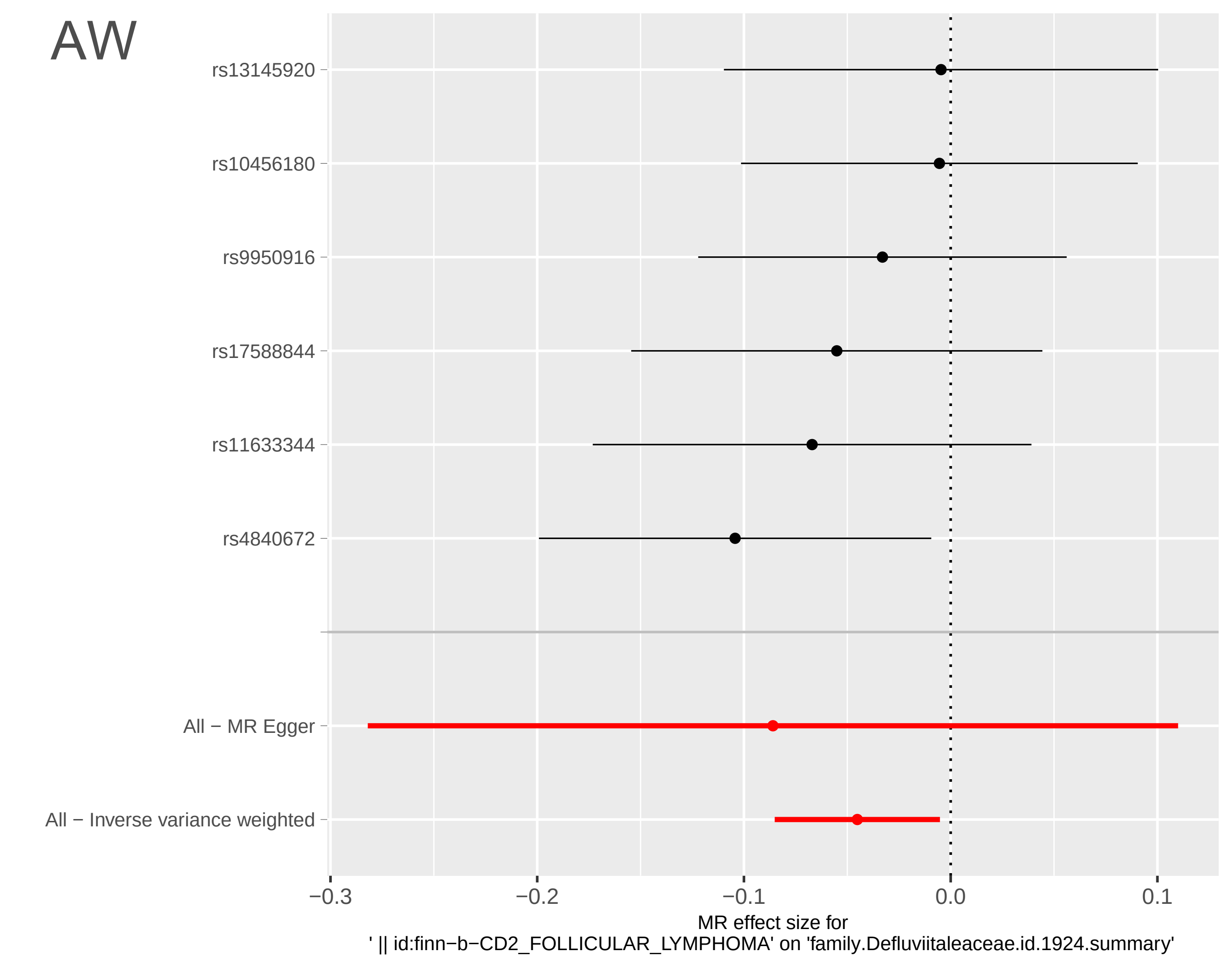
AW
rs13145920
rs10456180
rs9950916
rs17588844
rs11633344
rs4840672
All − MR Egger
All − Inverse variance weighted
−0.3
−0.2
−0.1
0.0
0.1
MR effect size for
' || id:finn−b−CD2_FOLLICULAR_LYMPHOMA' on 'family.Defluviitaleaceae.id.1924.summary'

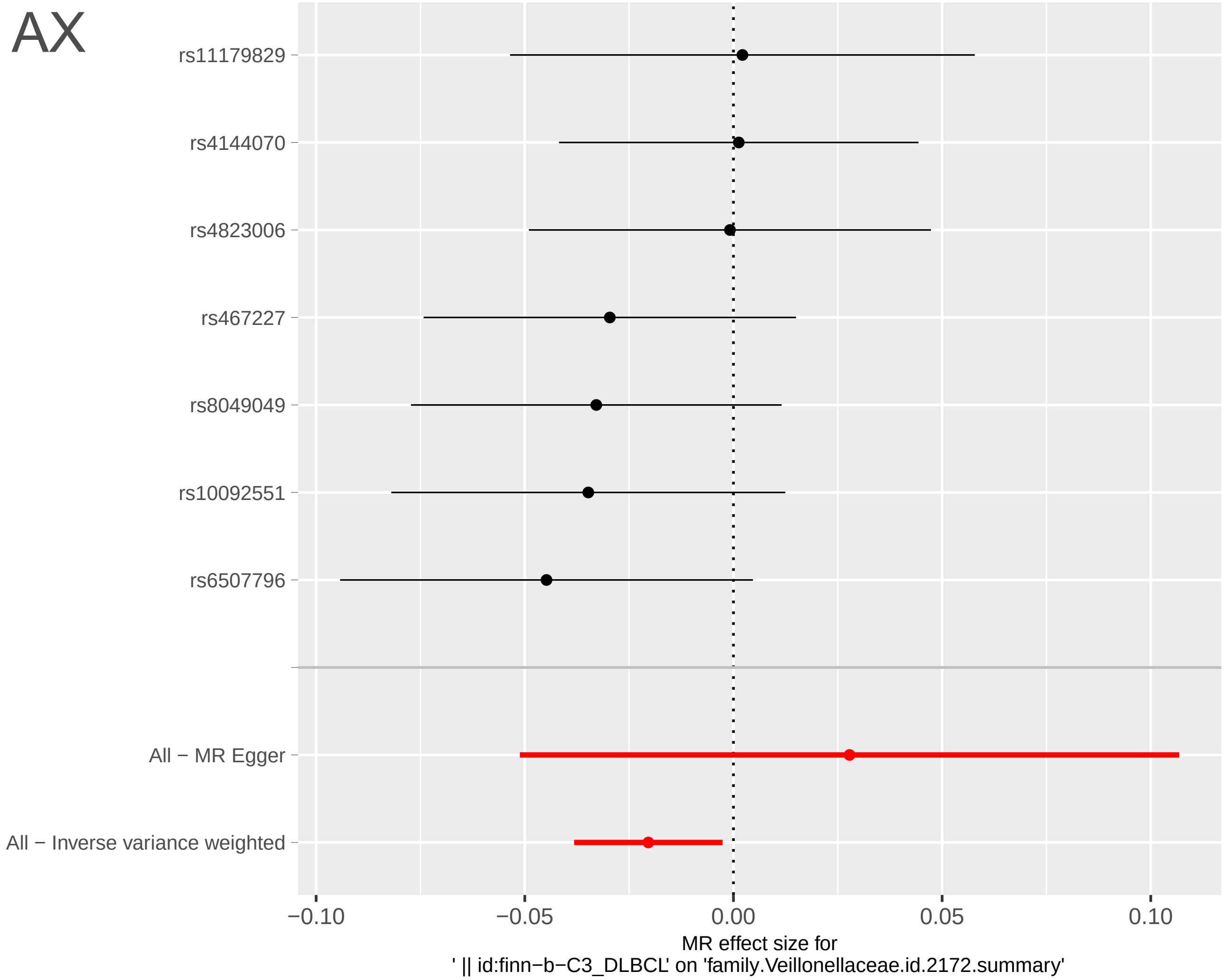
AX
rs11179829
rs4144070
rs4823006
rs467227
rs8049049
rs10092551
rs6507796
All − MR Egger
All − Inverse variance weighted
−0.10
−0.05
0.00
0.05
0.10
MR effect size for
' || id:finn−b−C3_DLBCL' on 'family.Veillonellaceae.id.2172.summary'

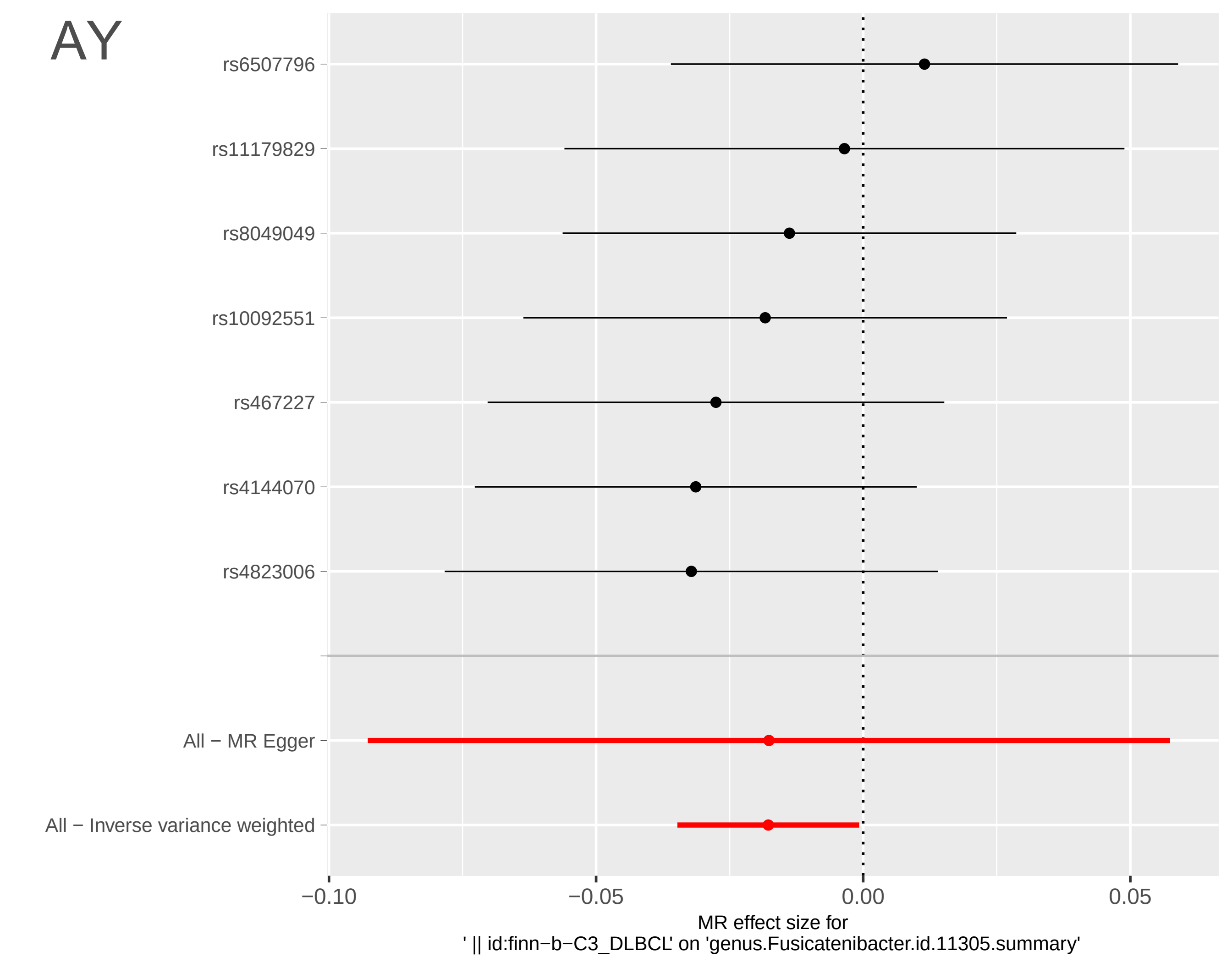
AY
rs6507796
rs11179829
rs8049049
rs10092551
rs467227
rs4144070
rs4823006
All − MR Egger
All − Inverse variance weighted
−0.10
−0.05
0.00
0.05
MR effect size for
' || id:finn−b−C3_DLBCL' on 'genus.Fusicatenibacter.id.11305.summary'

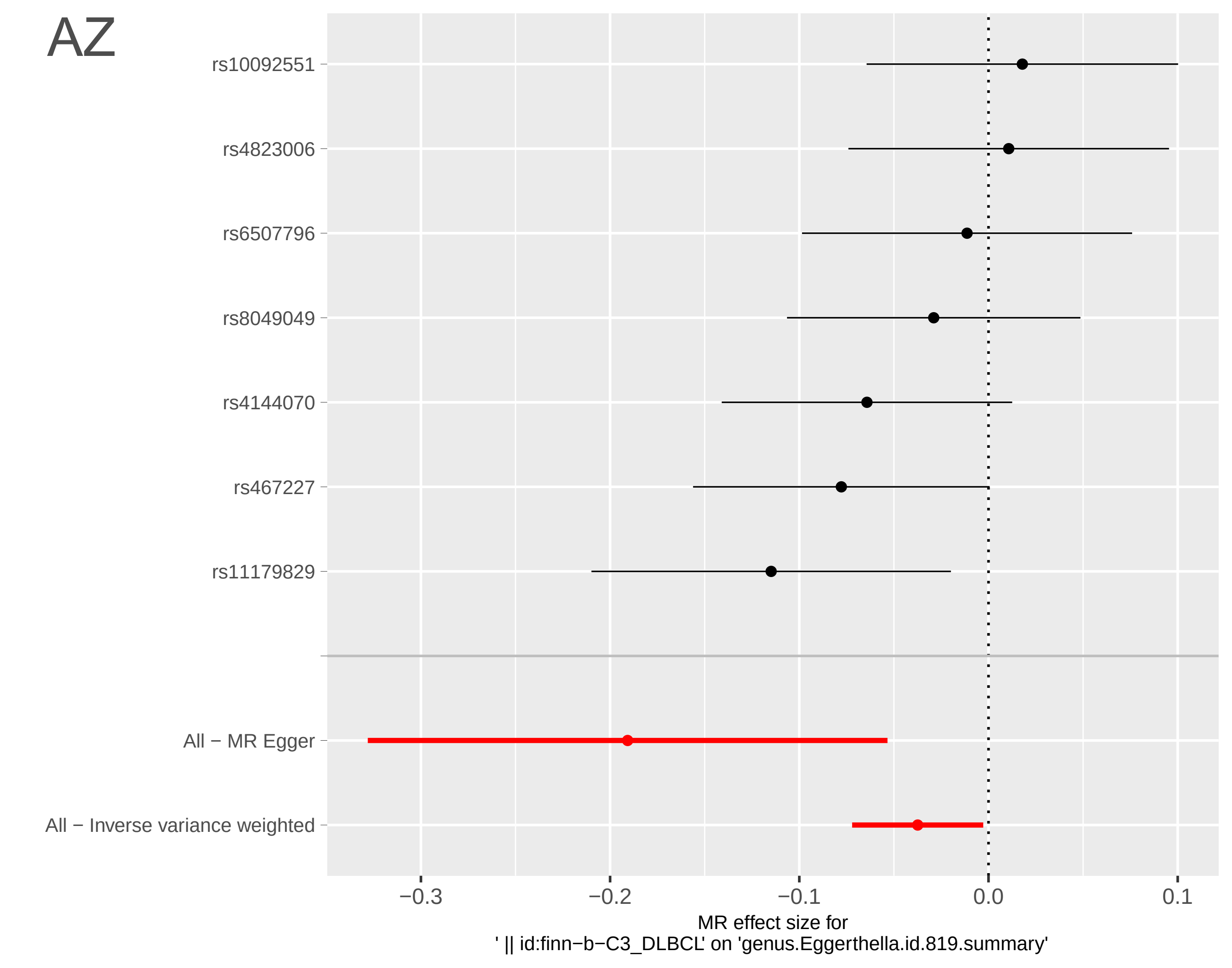
AZ
rs10092551
rs4823006
rs6507796
rs8049049
rs4144070
rs467227
rs11179829
All − MR Egger
All − Inverse variance weighted
−0.3
−0.2
−0.1
0.0
0.1
MR effect size for
' || id:finn−b−C3_DLBCL' on 'genus.Eggerthella.id.819.summary'

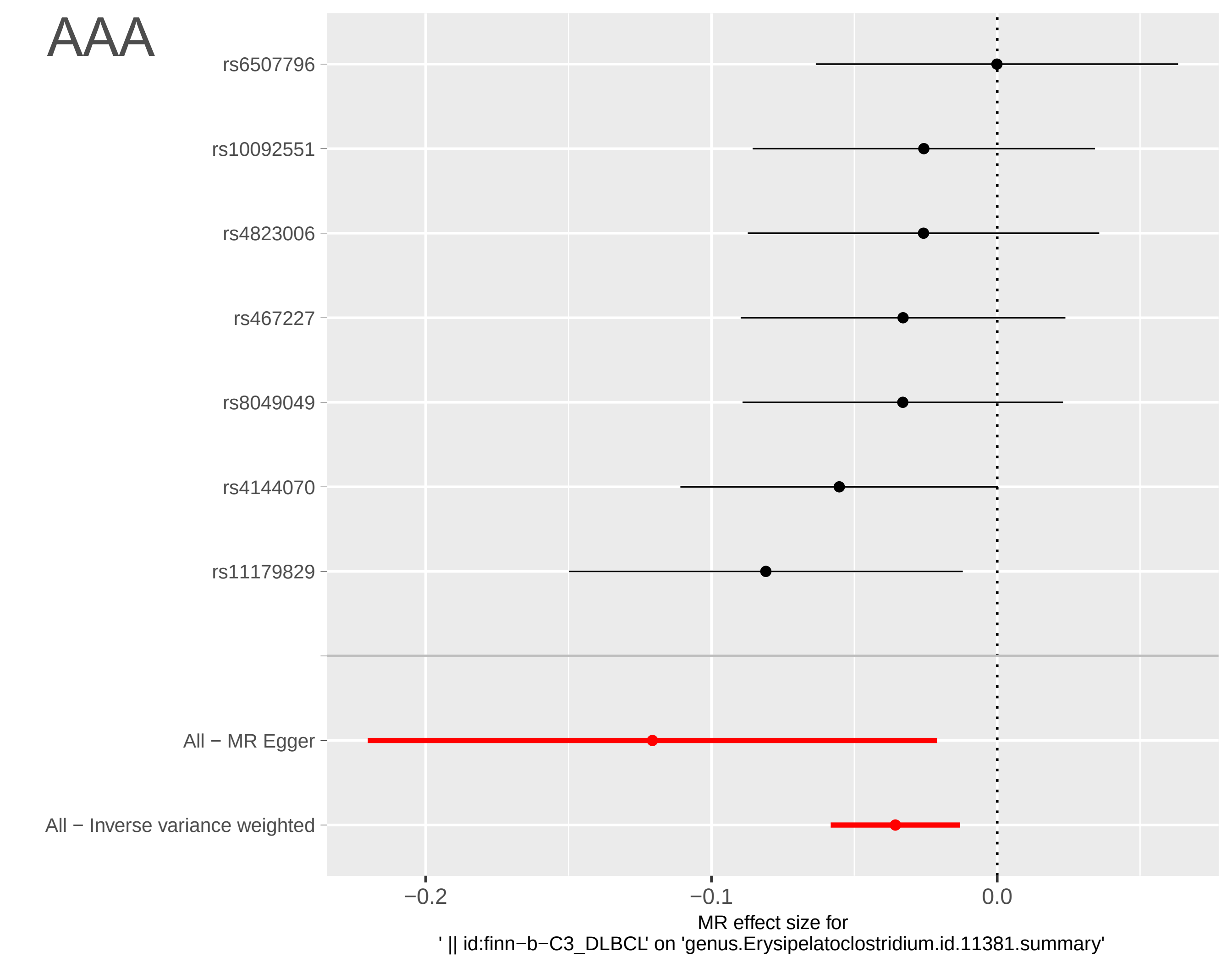

AAA
rs6507796
rs10092551
rs4823006
rs467227
rs8049049
rs4144070
rs11179829
All − MR Egger
All − Inverse variance weighted
−0.2
−0.1
0.0
MR effect size for
' || id:finn−b−C3_DLBCL' on 'genus.Erysipelatoclostridium.id.11381.summary'

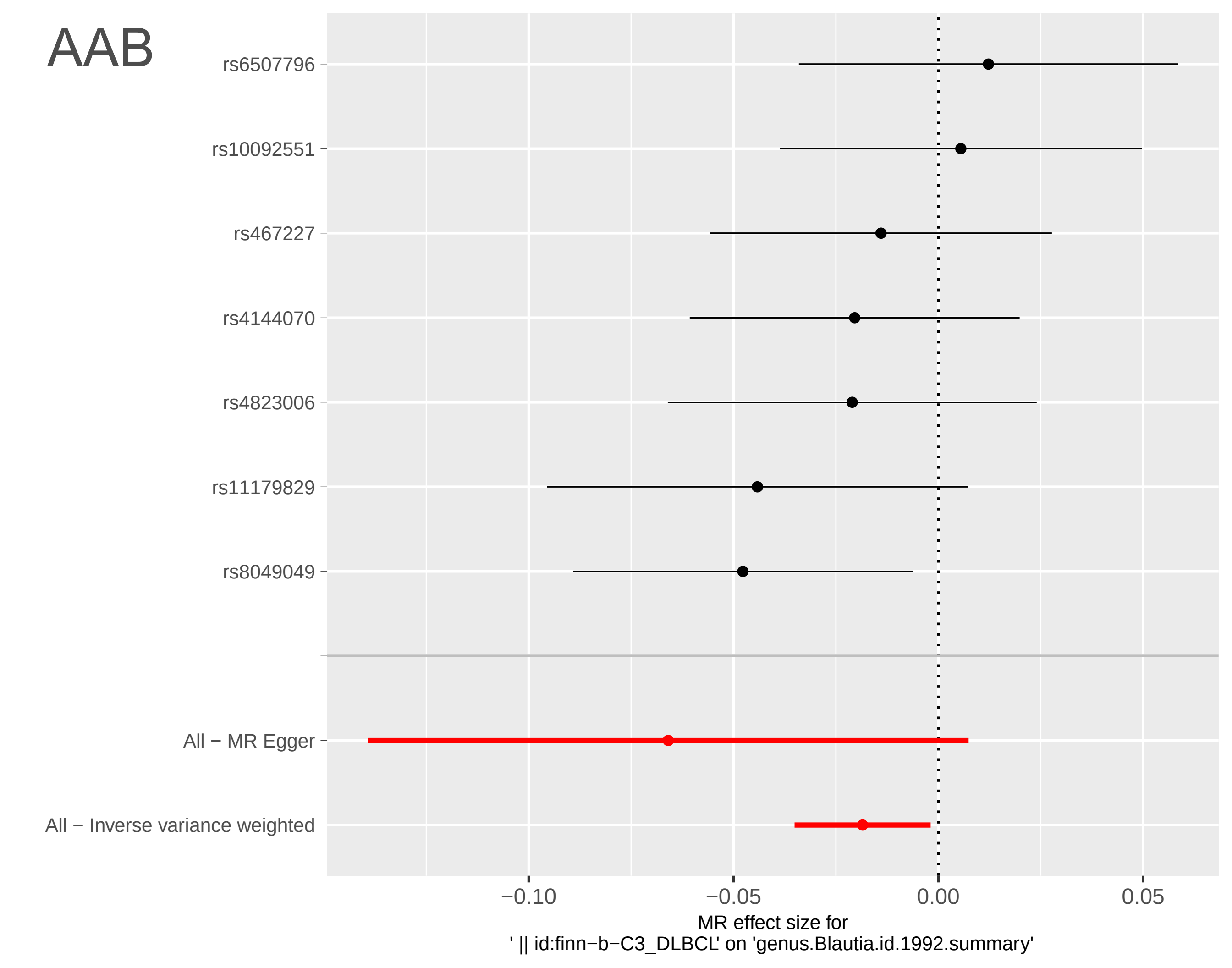
AAB
rs6507796
rs10092551
rs467227
rs4144070
rs4823006
rs11179829
rs8049049
All − MR Egger
All − Inverse variance weighted
−0.10
−0.05
0.00
0.05
MR effect size for
' || id:finn−b−C3_DLBCL' on 'genus.Blautia.id.1992.summary'

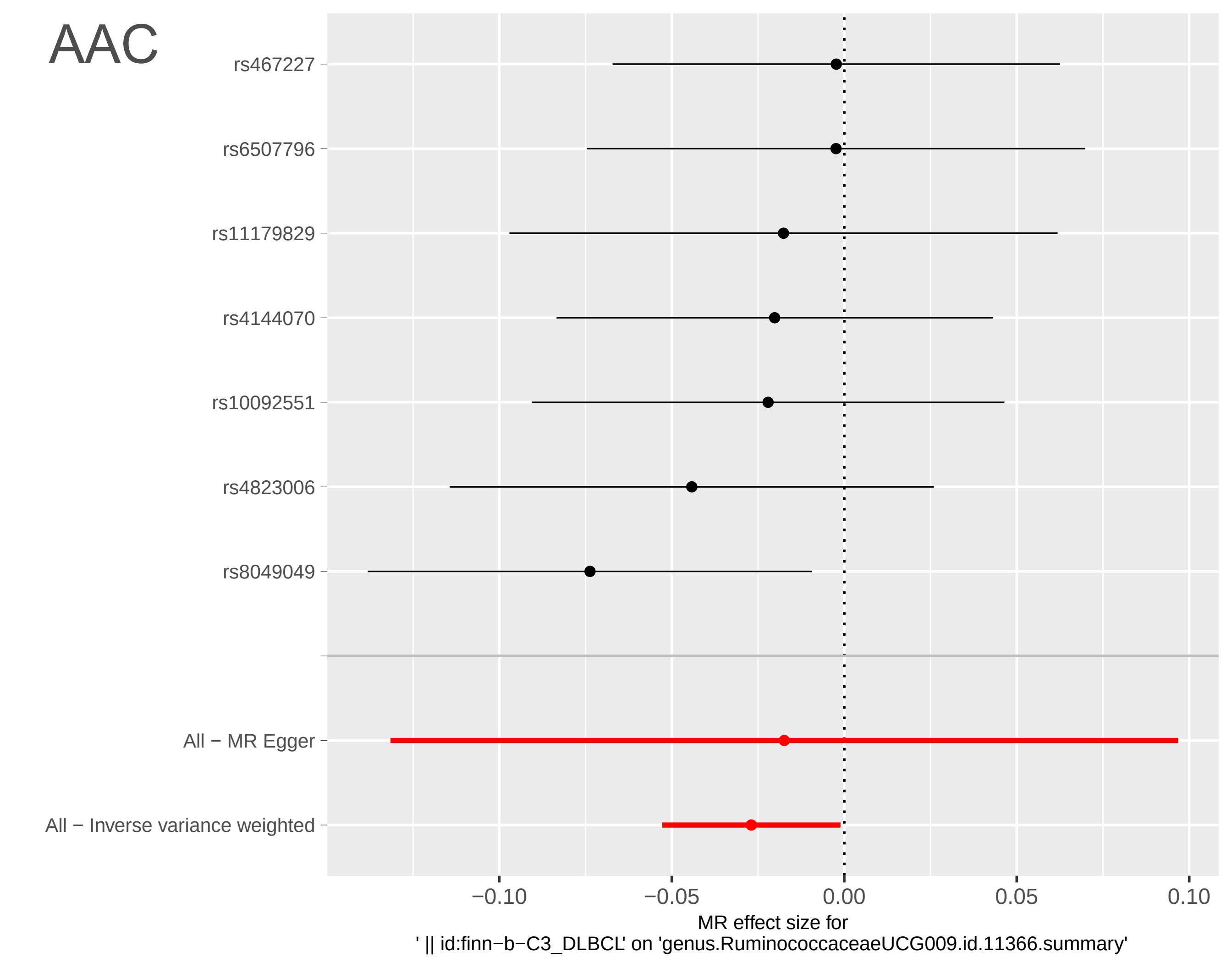
AAC
rs467227
rs6507796
rs11179829
rs4144070
rs10092551
rs4823006
rs8049049
All − MR Egger
All − Inverse variance weighted
−0.10
−0.05
0.00
0.05
0.10
MR effect size for
' || id:finn−b−C3_DLBCL' on 'genus.RuminococcaceaeUCG009.id.11366.summary'

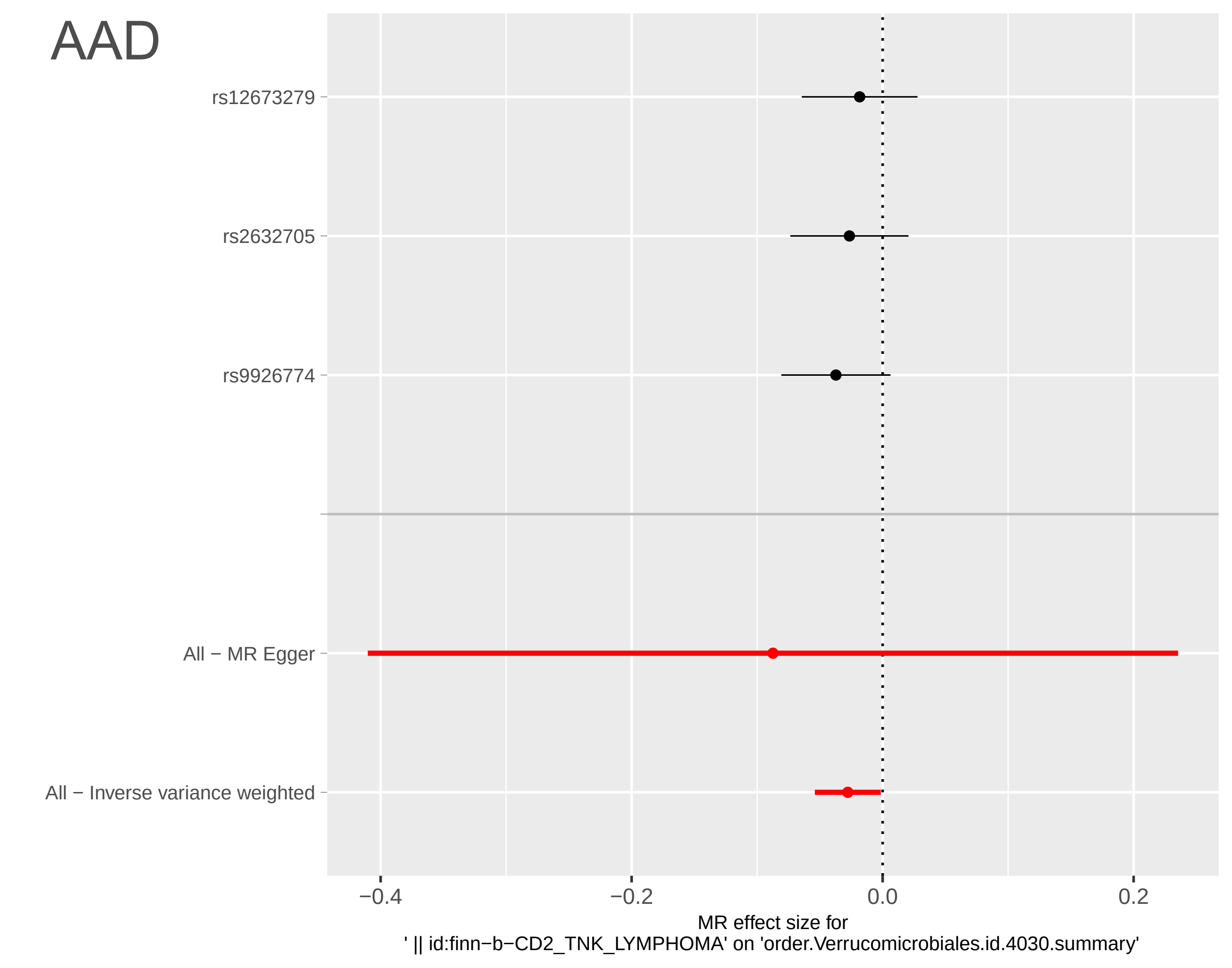
AAD
rs12673279
rs2632705
rs9926774
All − MR Egger
All − Inverse variance weighted
−0.4
−0.2
0.0
0.2
MR effect size for
' || id:finn−b−CD2_TNK_LYMPHOMA' on 'order.Verrucomicrobiales.id.4030.summary'

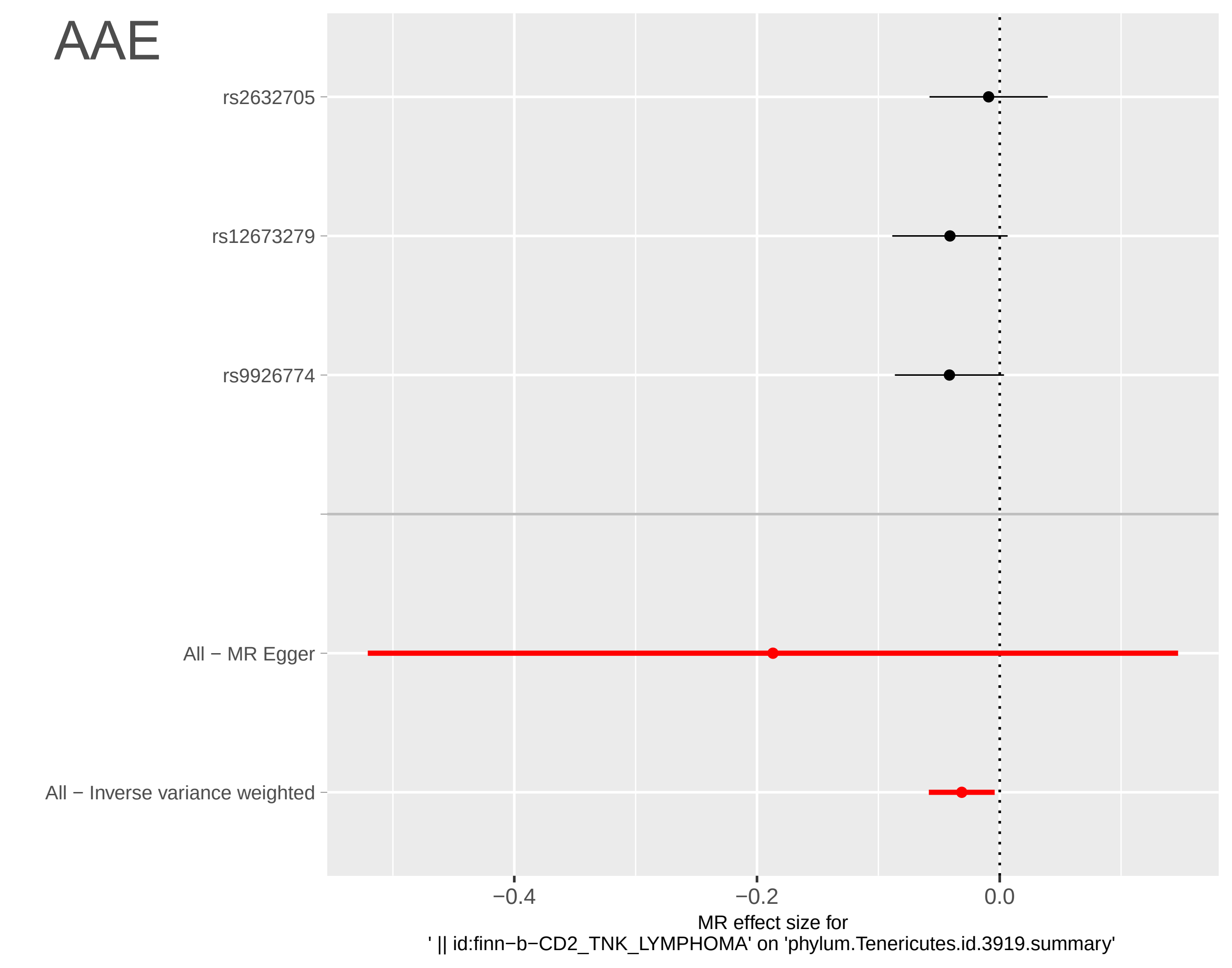
AAE
rs2632705
rs12673279
rs9926774
All − MR Egger
All − Inverse variance weighted
−0.4
−0.2
0.0
MR effect size for
' || id:finn−b−CD2_TNK_LYMPHOMA' on 'phylum.Tenericutes.id.3919.summary'

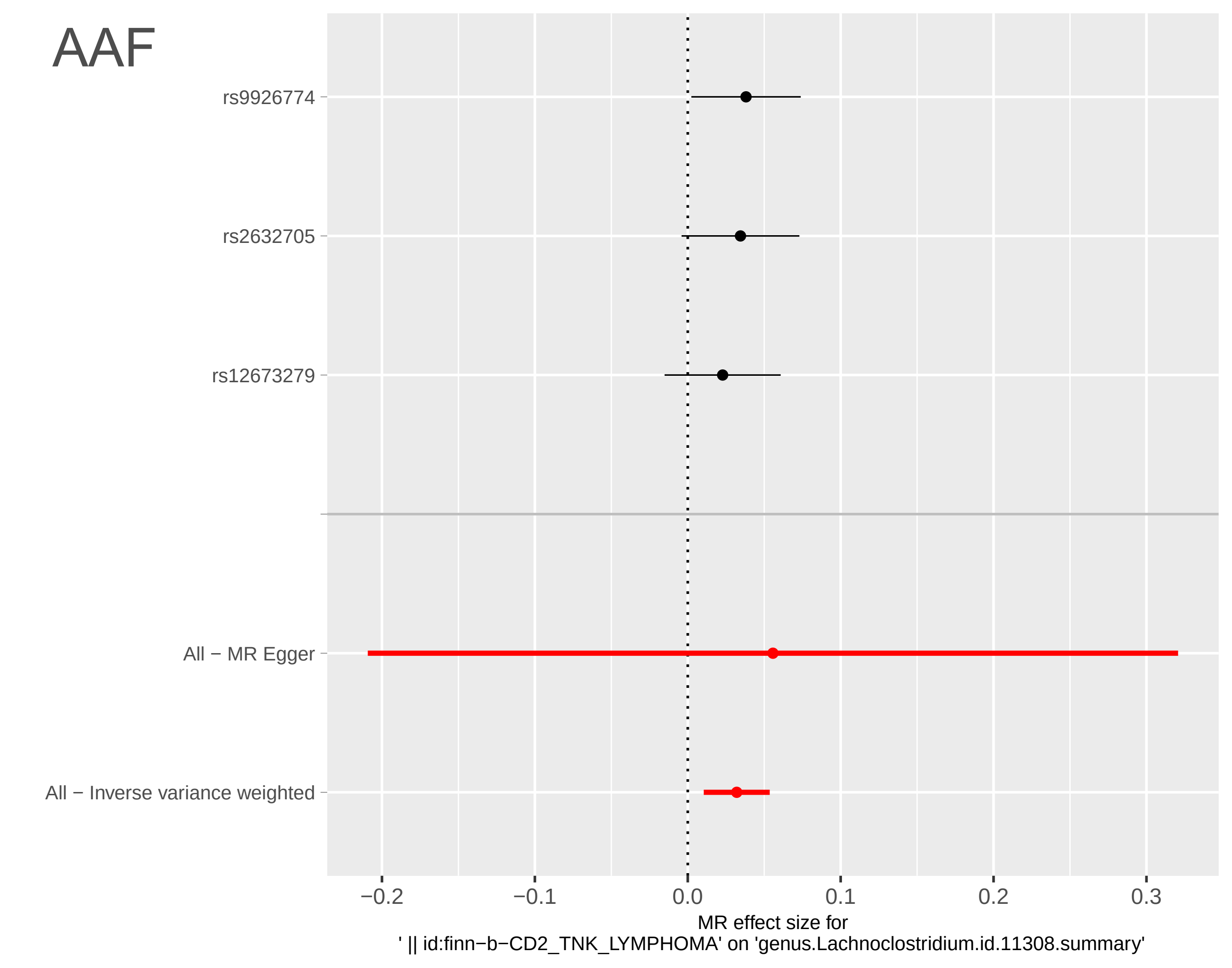

AAF
rs9926774
rs2632705
rs12673279
All − MR Egger
All − Inverse variance weighted
−0.2
−0.1
0.0
0.1
0.2
0.3
MR effect size for
' || id:finn−b−CD2_TNK_LYMPHOMA' on 'genus.Lachnoclostridium.id.11308.summary'

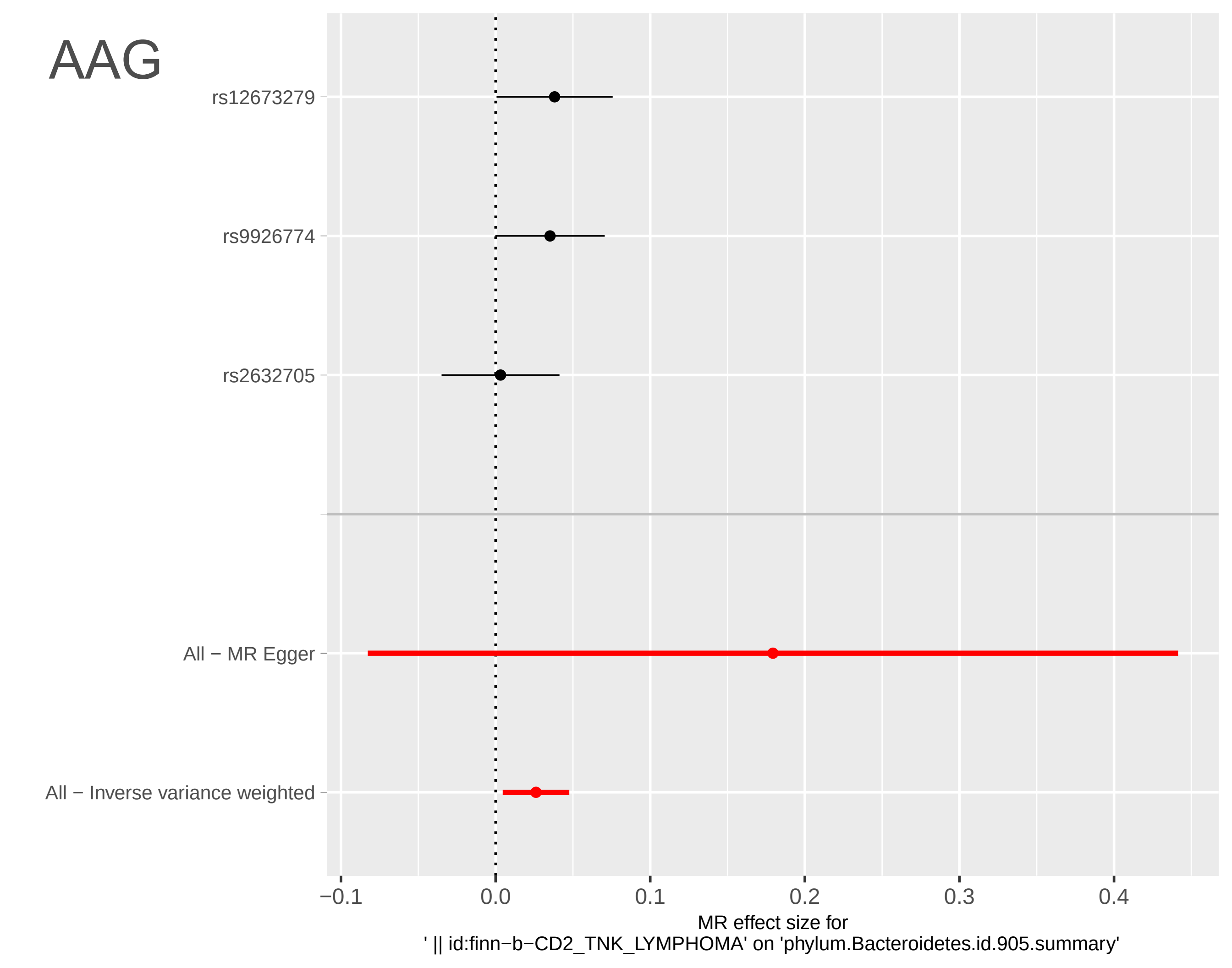

AAG
rs12673279
rs9926774
rs2632705
All − MR Egger
All − Inverse variance weighted
−0.1
0.0
0.1
0.2
0.3
0.4
MR effect size for
' || id:finn−b−CD2_TNK_LYMPHOMA' on 'phylum.Bacteroidetes.id.905.summary'

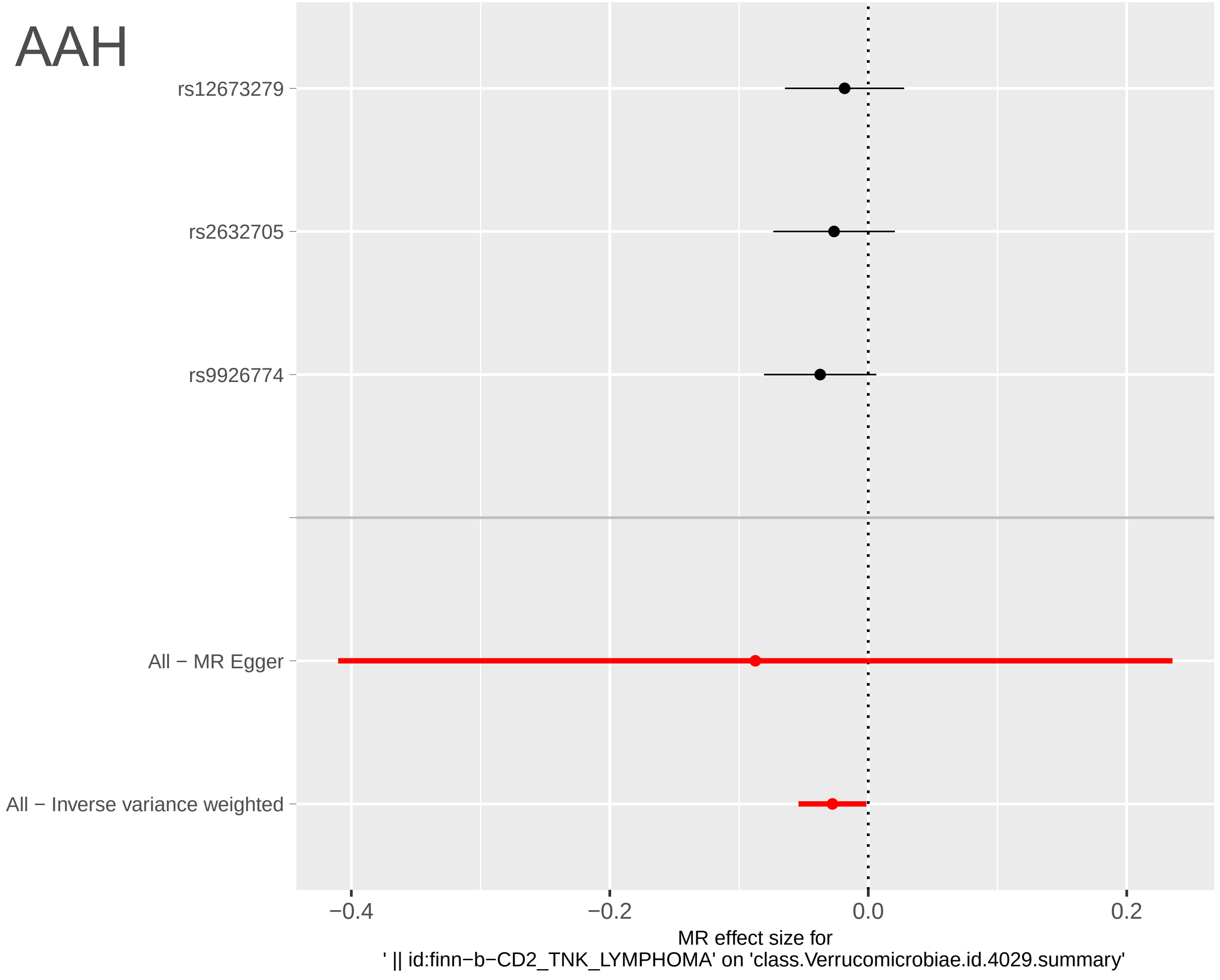
AAH
rs12673279
rs2632705
rs9926774
All − MR Egger
All − Inverse variance weighted
−0.4
−0.2
0.0
0.2
MR effect size for
' || id:finn−b−CD2_TNK_LYMPHOMA' on 'class.Verrucomicrobiae.id.4029.summary'

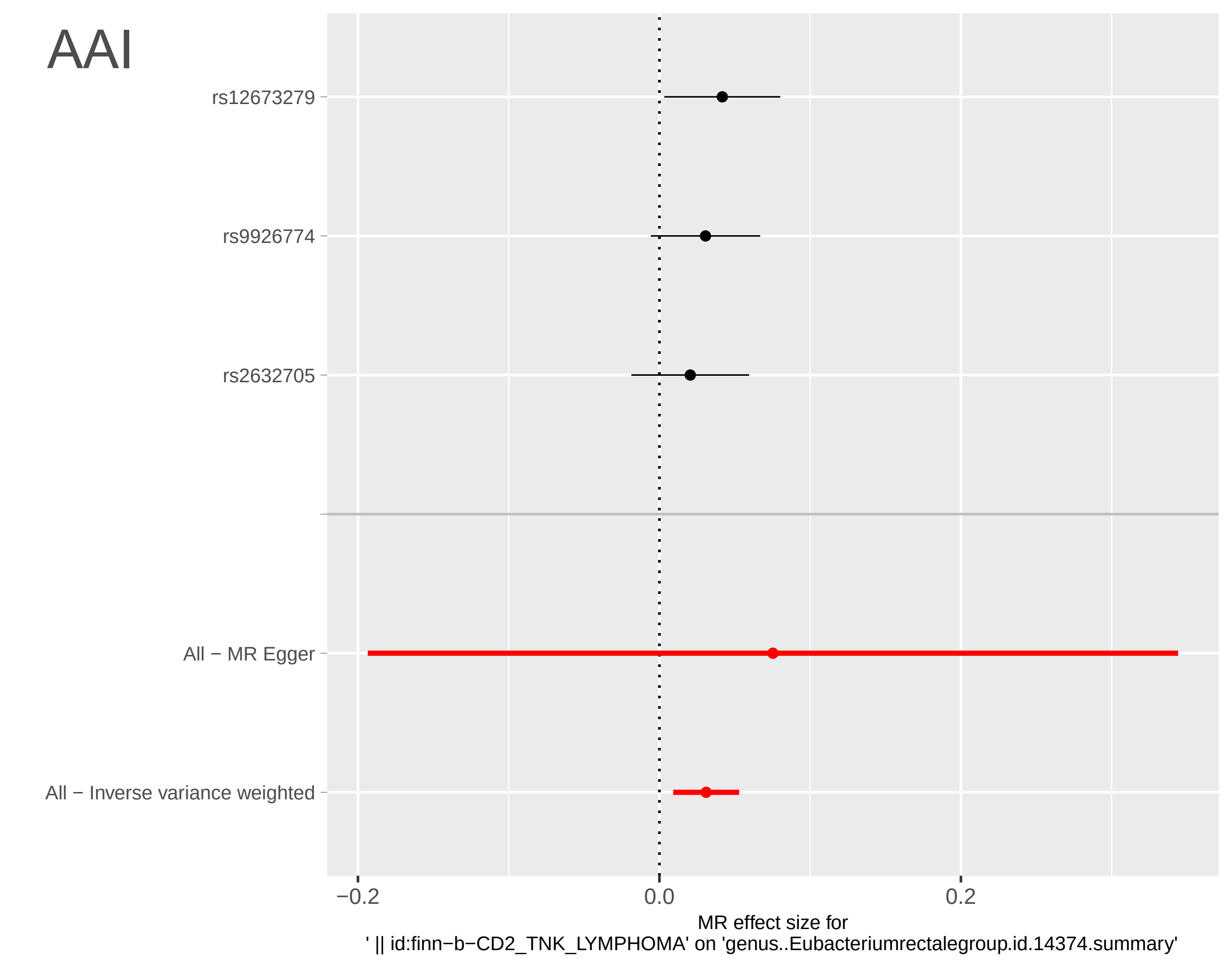
AAI
rs12673279
rs9926774
rs2632705
All − MR Egger
All − Inverse variance weighted
−0.2
0.0
0.2
MR effect size for
' || id:finn−b−CD2_TNK_LYMPHOMA' on 'genus..Eubacteriumrectalegroup.id.14374.summary'

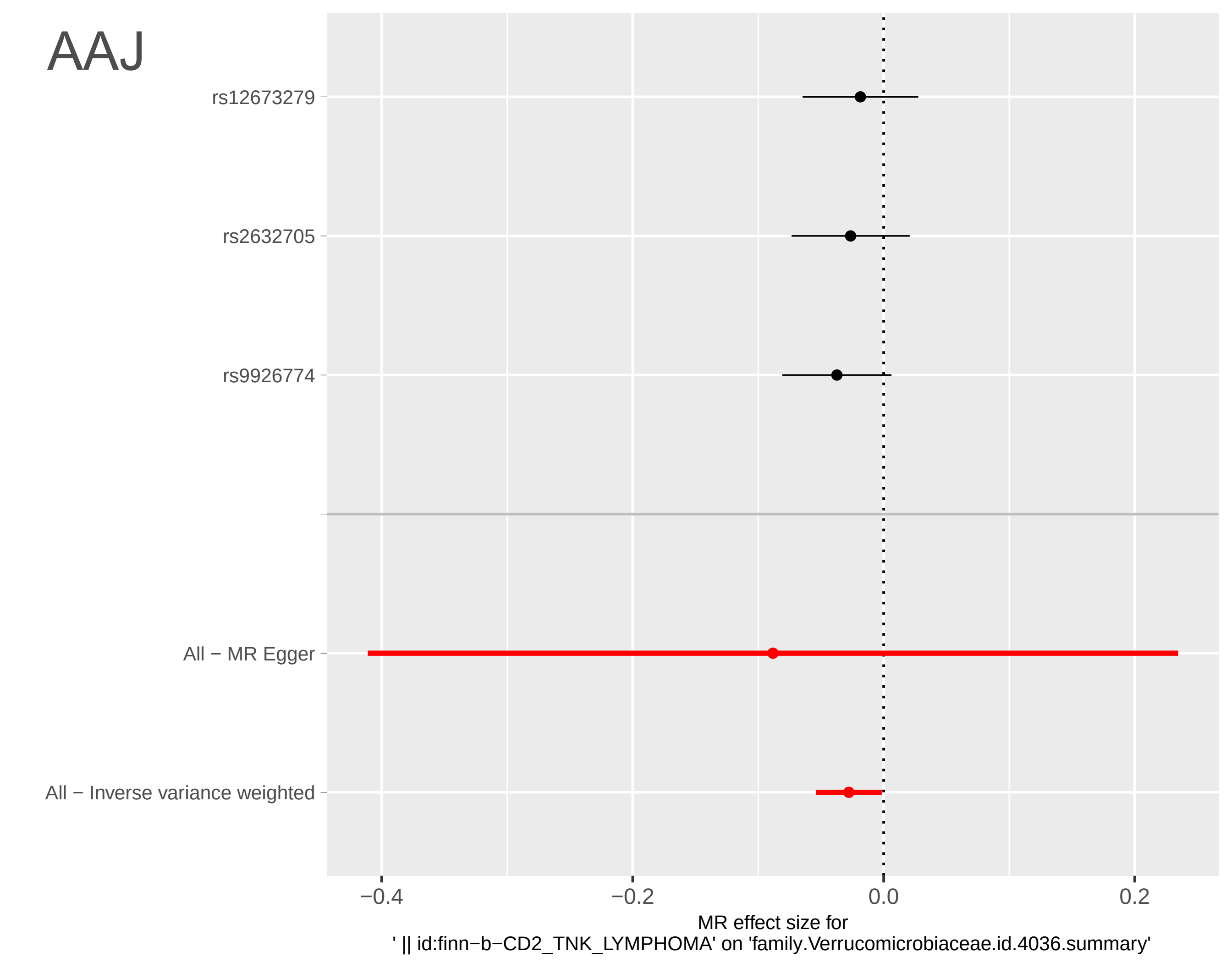
AAJ
rs12673279
rs2632705
rs9926774
All − MR Egger
All − Inverse variance weighted
−0.4
−0.2
0.0
0.2
MR effect size for
' || id:finn−b−CD2_TNK_LYMPHOMA' on 'family.Verrucomicrobiaceae.id.4036.summary'

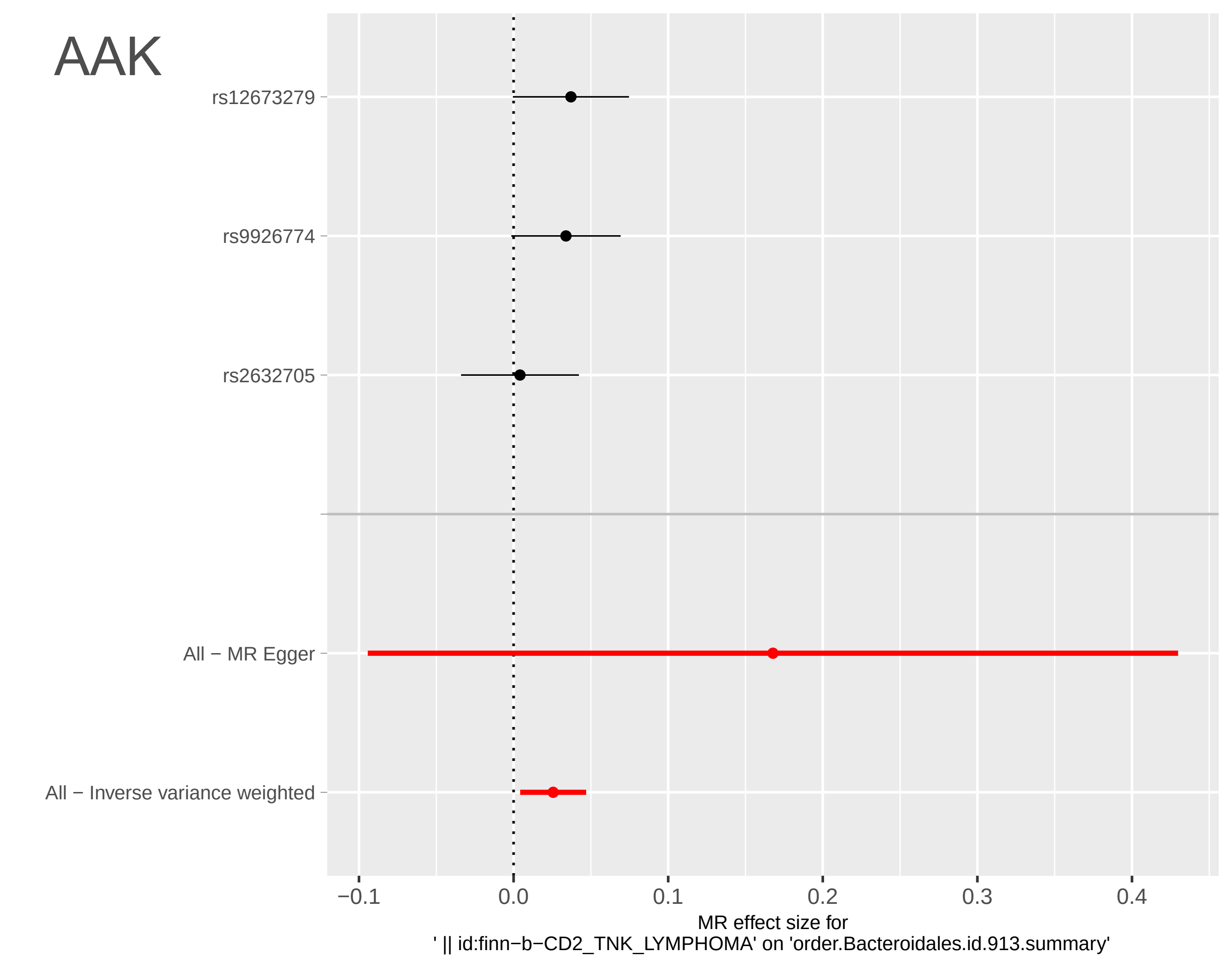
AAK
rs12673279
rs9926774
rs2632705
All − MR Egger
All − Inverse variance weighted
−0.1
0.0
0.1
0.2
0.3
0.4
MR effect size for
' || id:finn−b−CD2_TNK_LYMPHOMA' on 'order.Bacteroidales.id.913.summary'

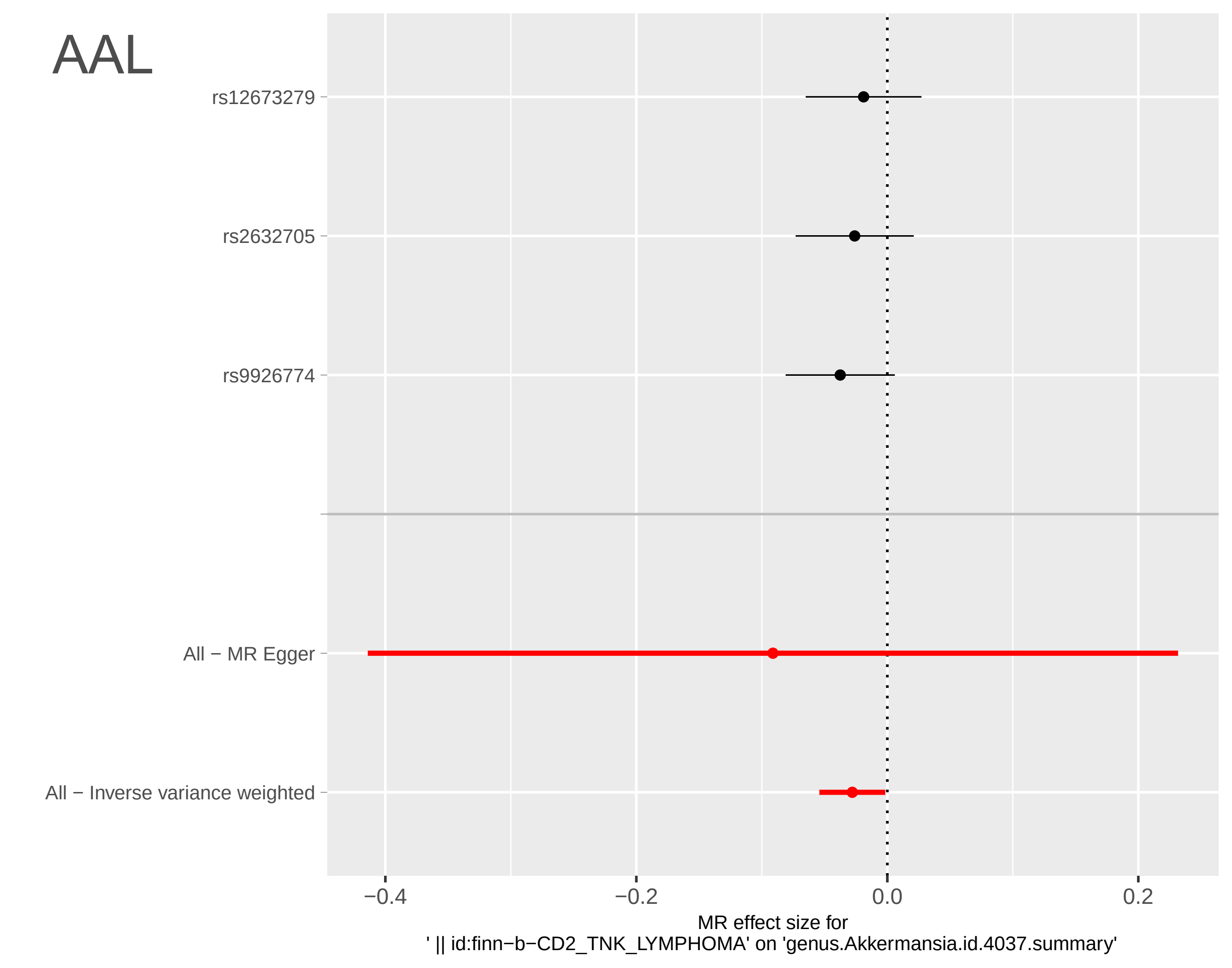
AAL
rs12673279
rs2632705
rs9926774
All − MR Egger
All − Inverse variance weighted
−0.4
−0.2
0.0
0.2
MR effect size for
' || id:finn−b−CD2_TNK_LYMPHOMA' on 'genus.Akkermansia.id.4037.summary'

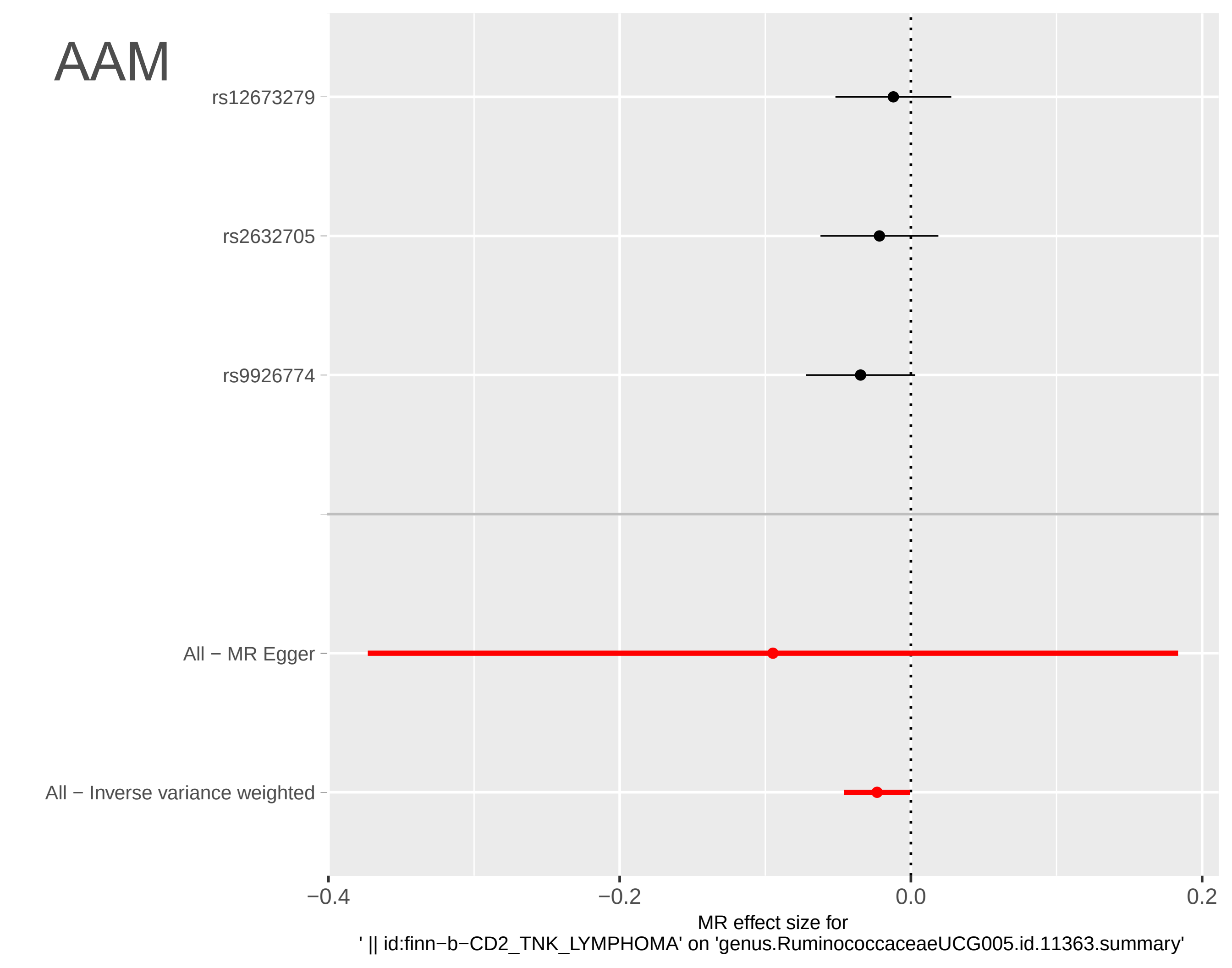

AAM
rs12673279
rs2632705
rs9926774
All − MR Egger
All − Inverse variance weighted
−0.4
−0.2
0.0
0.2
MR effect size for
' || id:finn−b−CD2_TNK_LYMPHOMA' on 'genus.RuminococcaceaeUCG005.id.11363.summary'

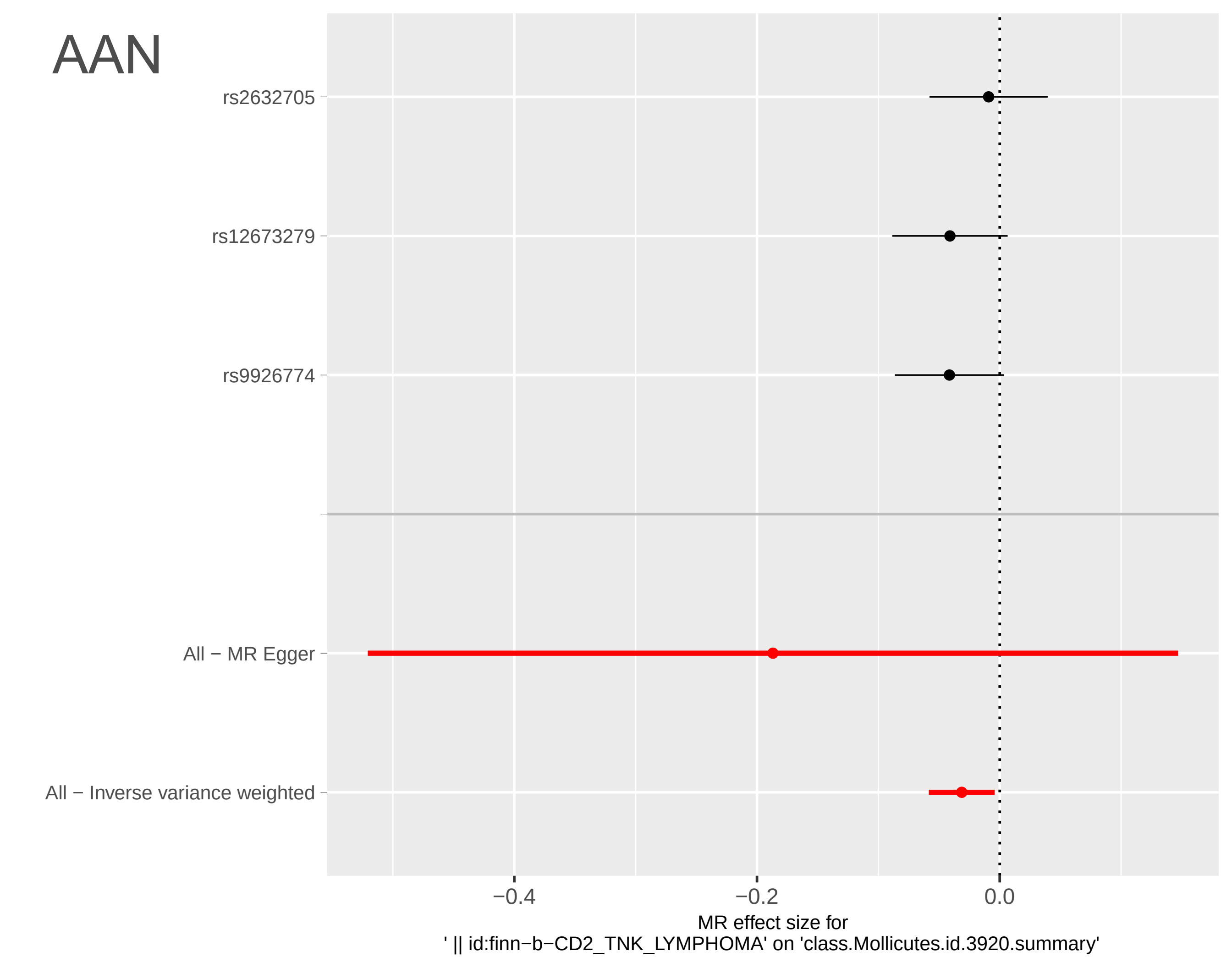
AAN
rs2632705
rs12673279
rs9926774
All − MR Egger
All − Inverse variance weighted
−0.4
−0.2
0.0
MR effect size for
' || id:finn−b−CD2_TNK_LYMPHOMA' on 'class.Mollicutes.id.3920.summary'

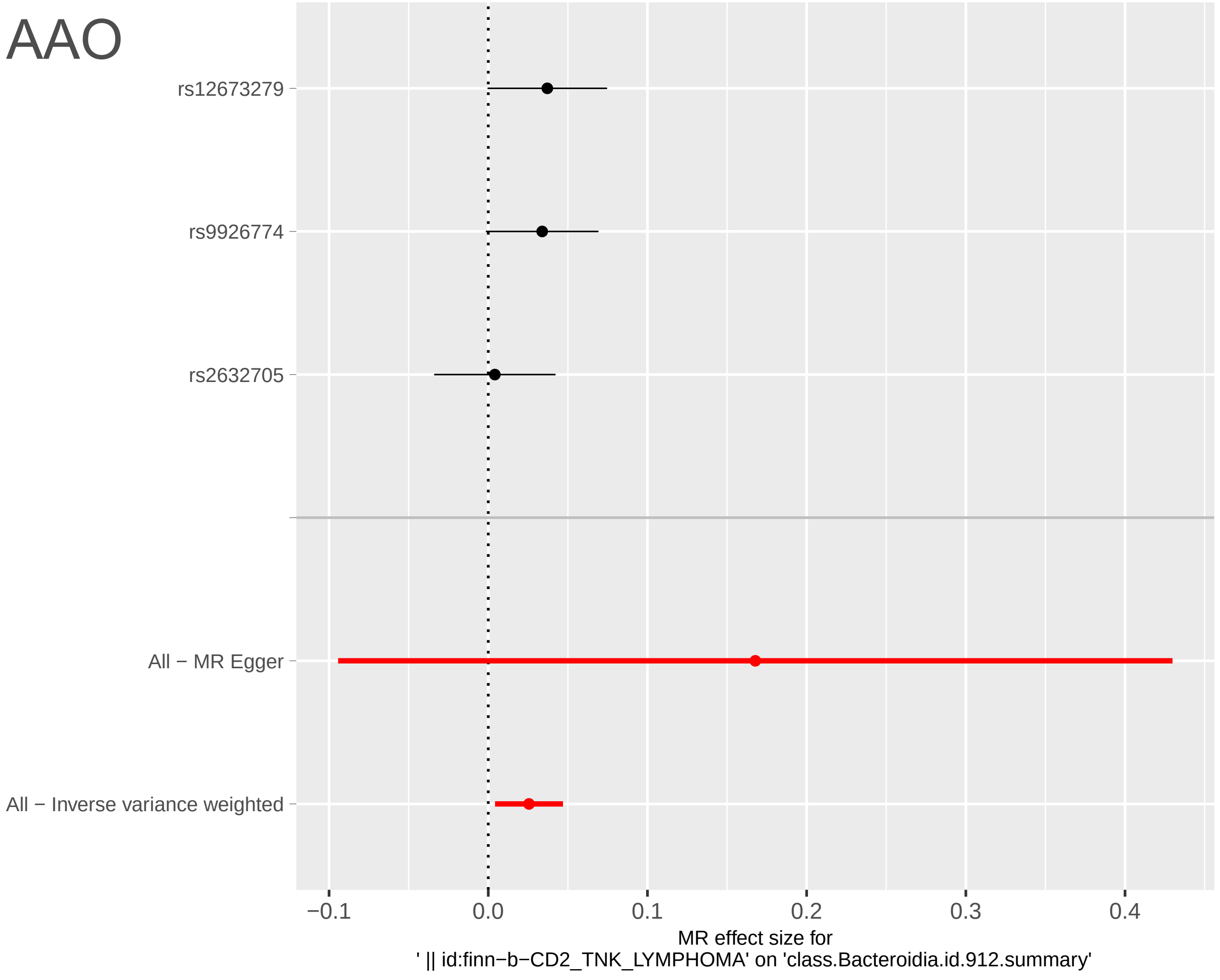
AAO
rs12673279
rs9926774
rs2632705
All − MR Egger
All − Inverse variance weighted
−0.1
0.0
0.1
0.2
0.3
0.4
MR effect size for
' || id:finn−b−CD2_TNK_LYMPHOMA' on 'class.Bacteroidia.id.912.summary'

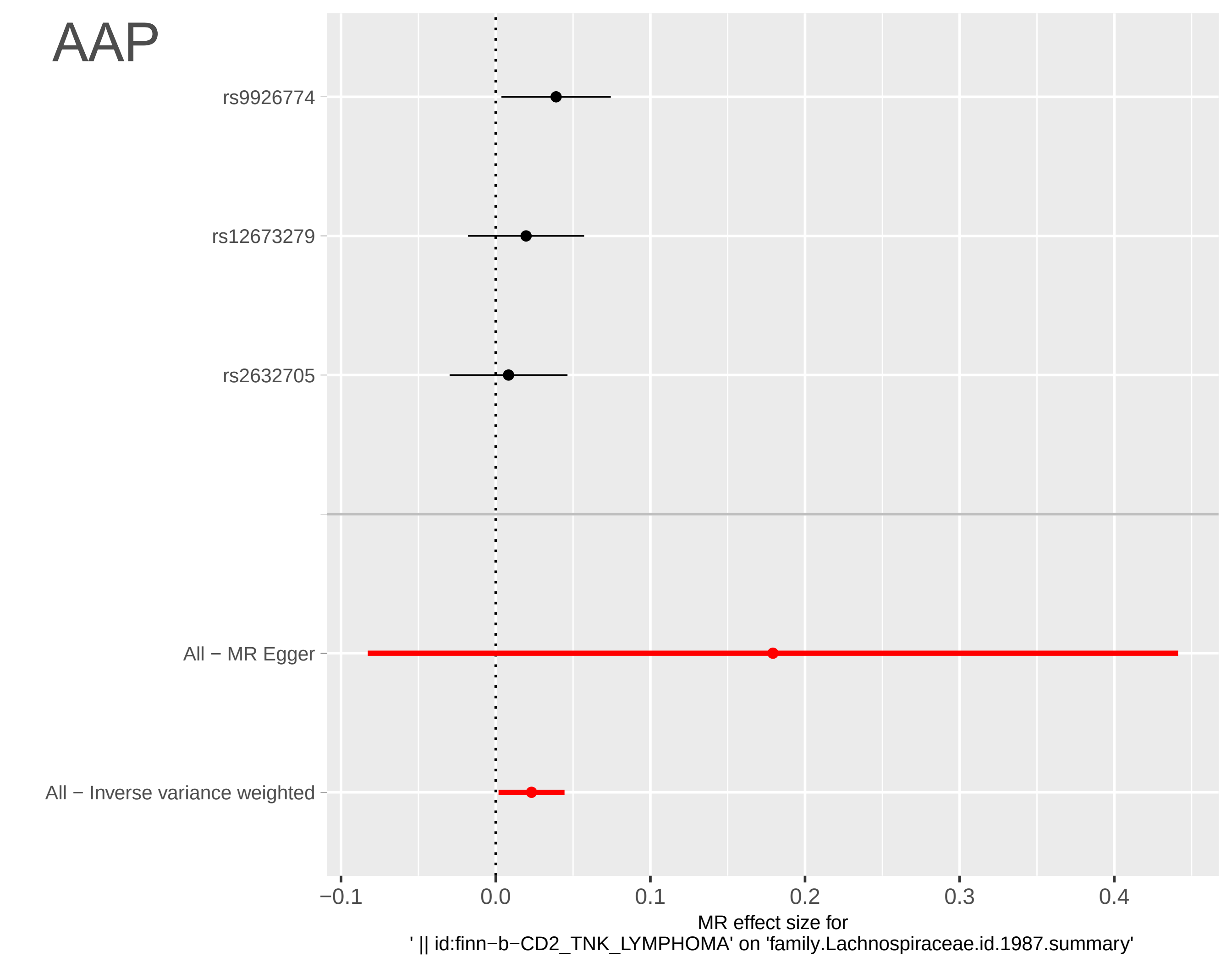

AAP
rs9926774
rs12673279
rs2632705
All − MR Egger
All − Inverse variance weighted
−0.1
0.0
0.1
0.2
0.3
0.4
MR effect size for
' || id:finn−b−CD2_TNK_LYMPHOMA' on 'family.Lachnospiraceae.id.1987.summary'

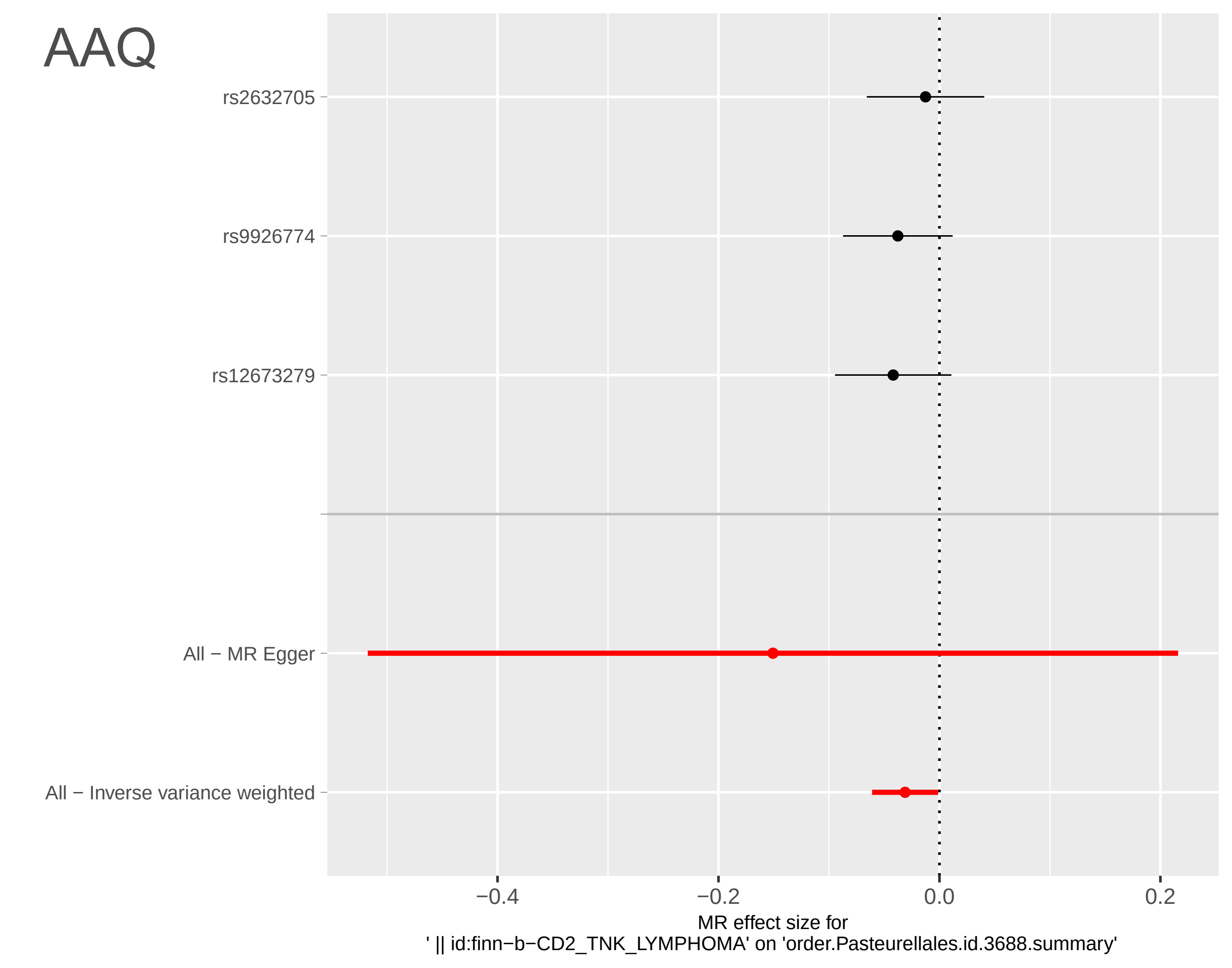
AAQ
rs2632705
rs9926774
rs12673279
All − MR Egger
All − Inverse variance weighted
−0.4
−0.2
0.0
0.2
MR effect size for
' || id:finn−b−CD2_TNK_LYMPHOMA' on 'order.Pasteurellales.id.3688.summary'

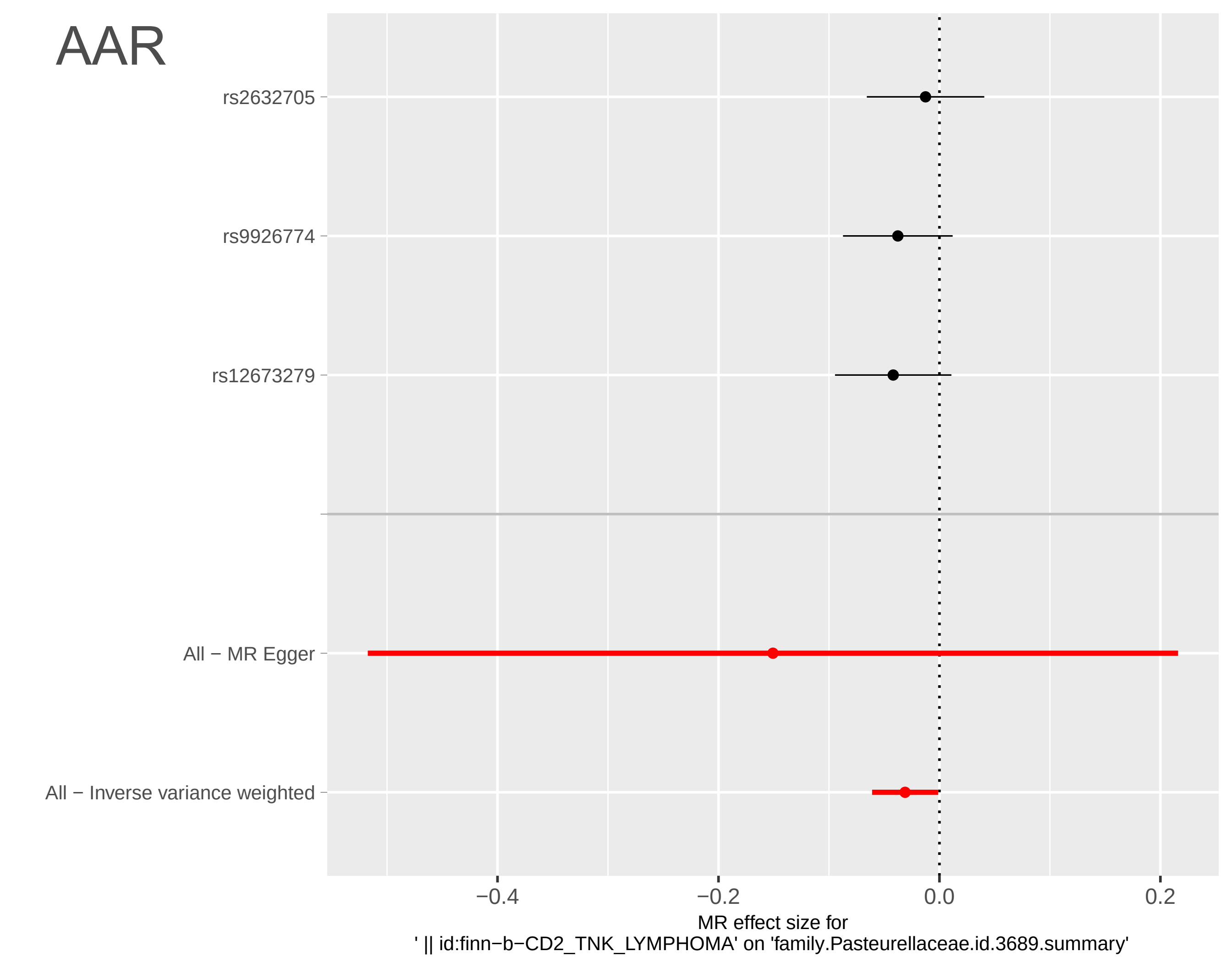
AAR
rs2632705
rs9926774
rs12673279
All − MR Egger
All − Inverse variance weighted
−0.4
−0.2
0.0
0.2
MR effect size for
' || id:finn−b−CD2_TNK_LYMPHOMA' on 'family.Pasteurellaceae.id.3689.summary'

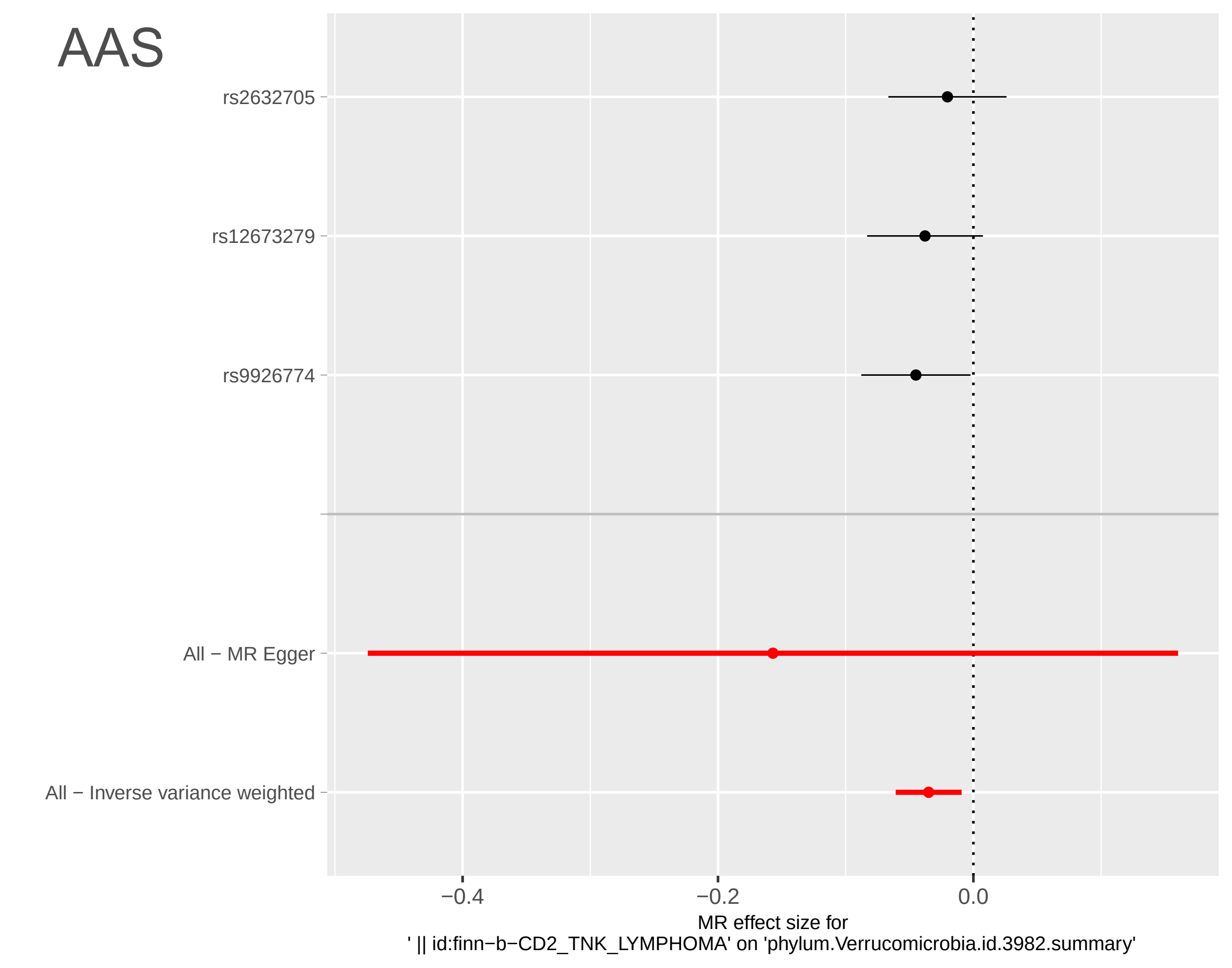

AAS
rs2632705
rs12673279
rs9926774
All − MR Egger
All − Inverse variance weighted
−0.4
−0.2
0.0
MR effect size for
' || id:finn−b−CD2_TNK_LYMPHOMA' on 'phylum.Verrucomicrobia.id.3982.summary'

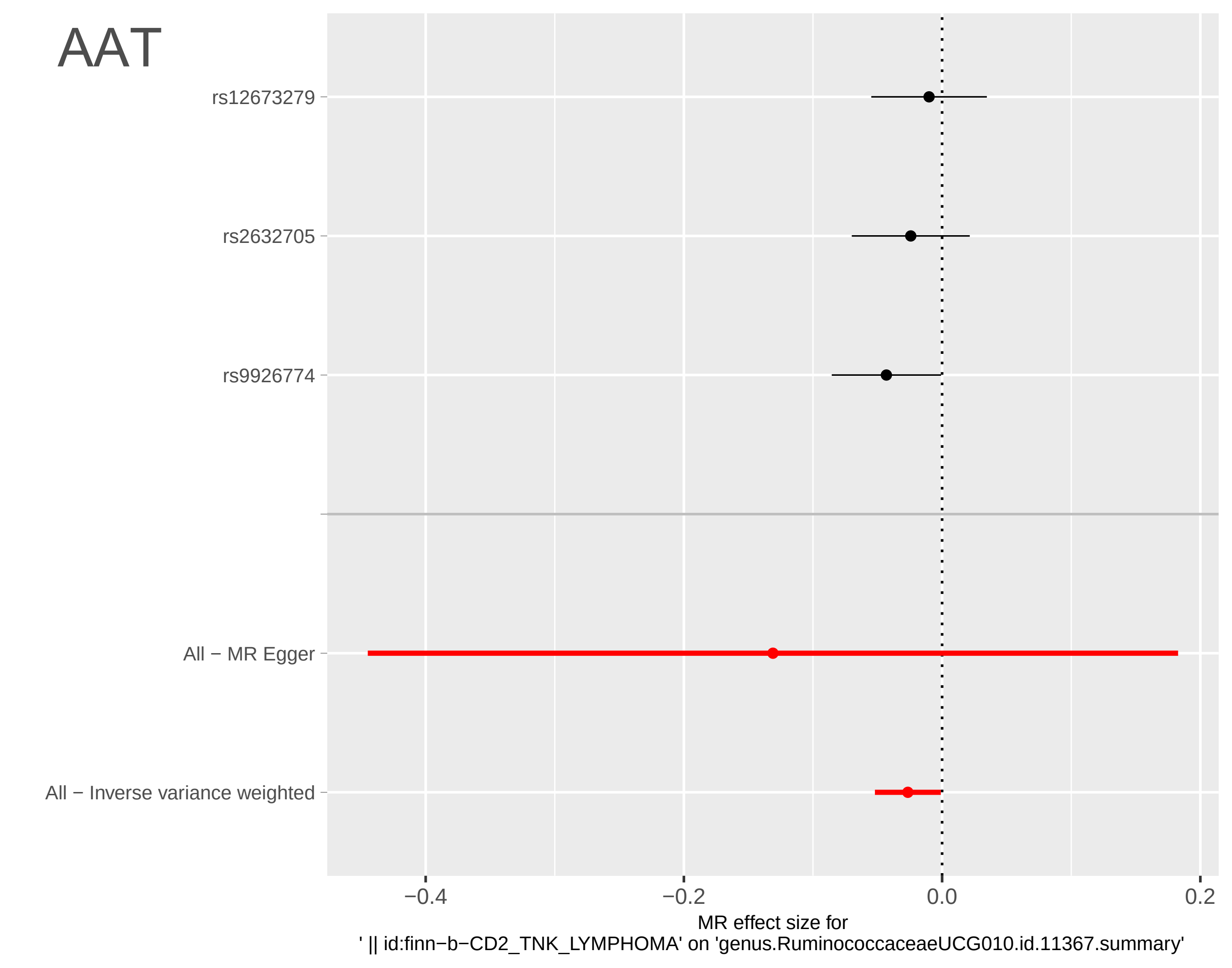
AAT
rs12673279
rs2632705
rs9926774
All − MR Egger
All − Inverse variance weighted
−0.4
−0.2
0.0
0.2
MR effect size for
' || id:finn−b−CD2_TNK_LYMPHOMA' on 'genus.RuminococcaceaeUCG010.id.11367.summary'

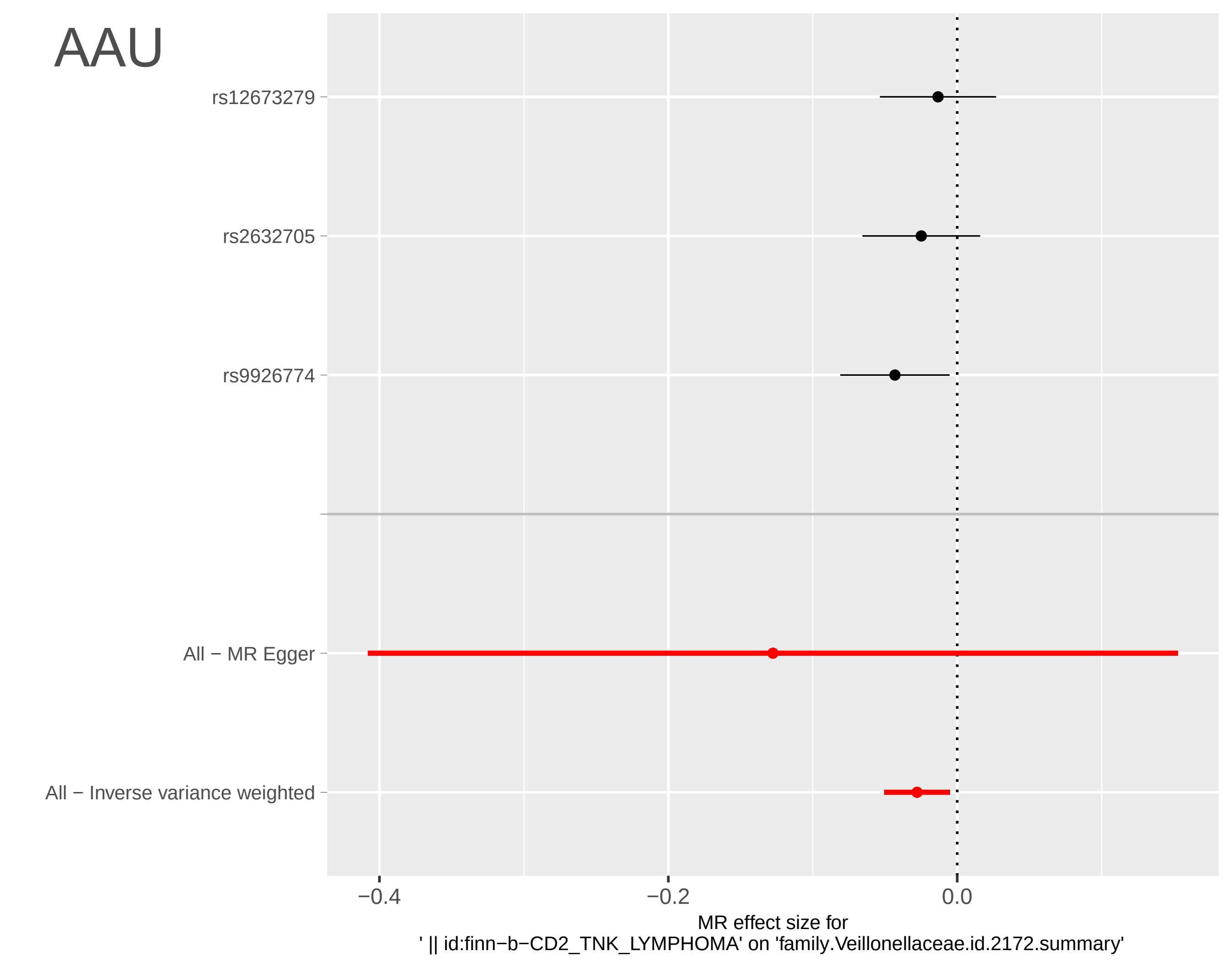
AAU
rs12673279
rs2632705
rs9926774
All − MR Egger
All − Inverse variance weighted
−0.4
−0.2
0.0
MR effect size for
' || id:finn−b−CD2_TNK_LYMPHOMA' on 'family.Veillonellaceae.id.2172.summary'

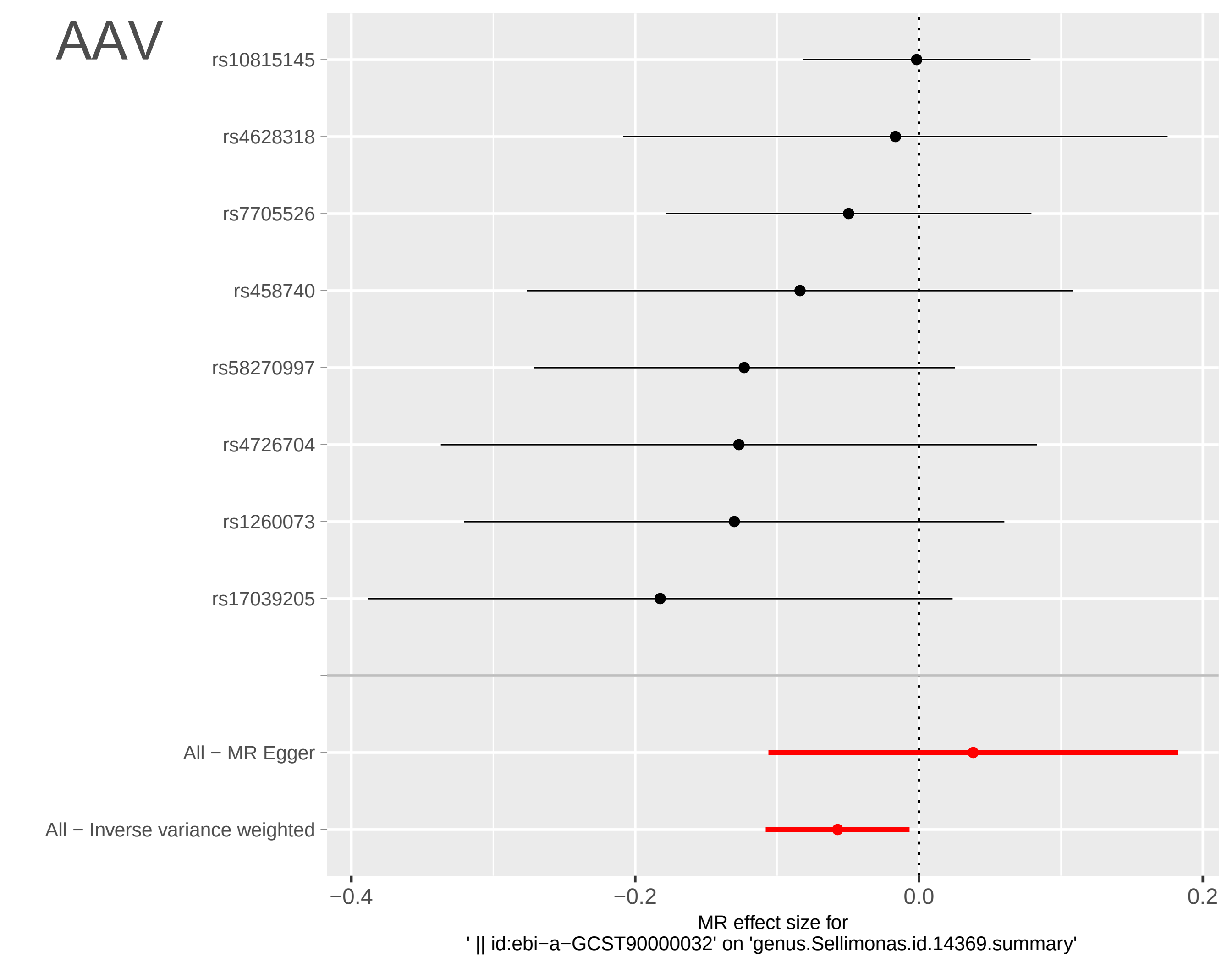

AAV
rs10815145
rs4628318
rs7705526
rs458740
rs58270997
rs4726704
rs1260073
rs17039205
All − MR Egger
All − Inverse variance weighted
−0.4
−0.2
0.0
0.2
MR effect size for
' || id:ebi−a−GCST90000032' on 'genus.Sellimonas.id.14369.summary'

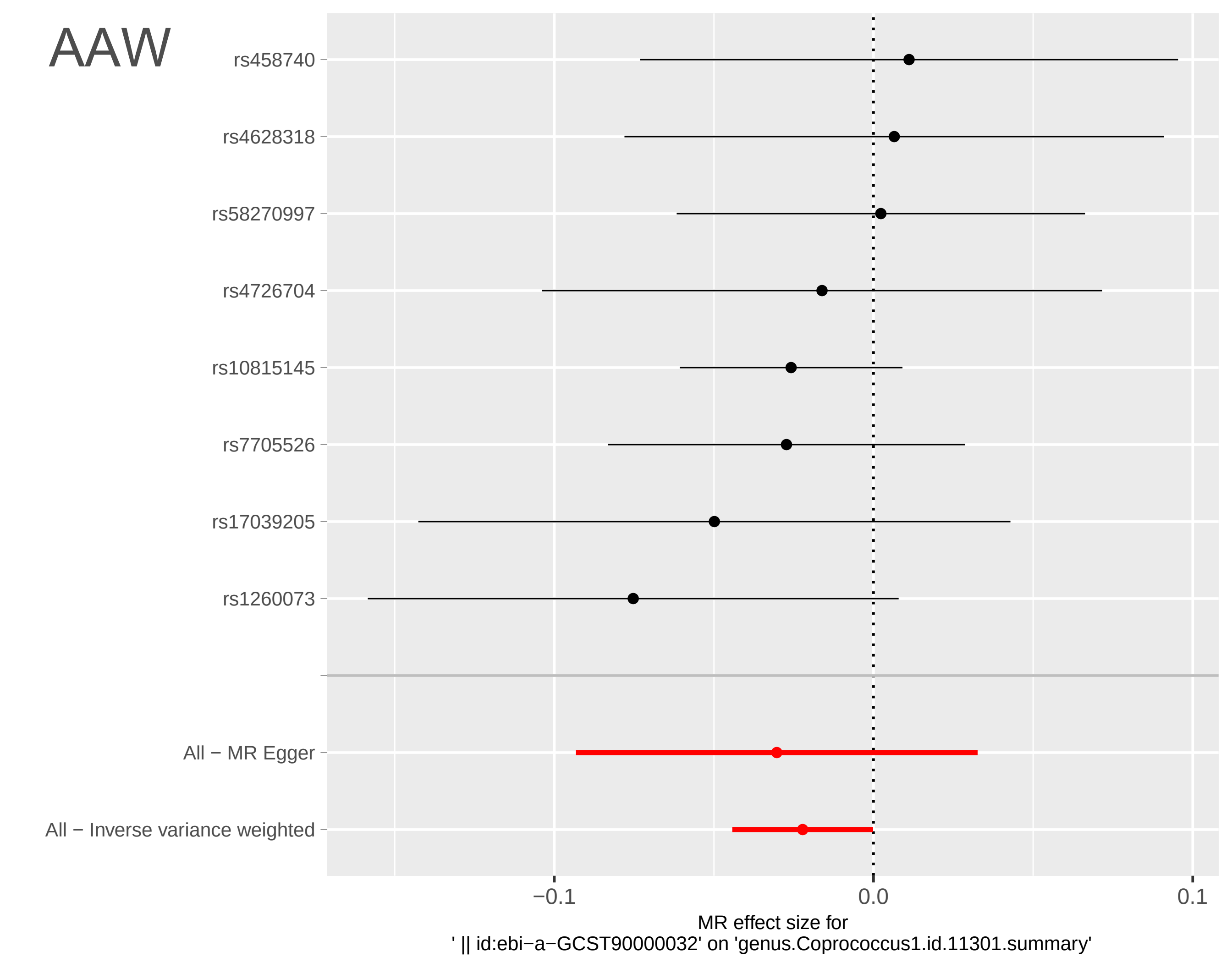
AAW
rs458740
rs4628318
rs58270997
rs4726704
rs10815145
rs7705526
rs17039205
rs1260073
All − MR Egger
All − Inverse variance weighted
−0.1
0.0
0.1
MR effect size for
' || id:ebi−a−GCST90000032' on 'genus.Coprococcus1.id.11301.summary'

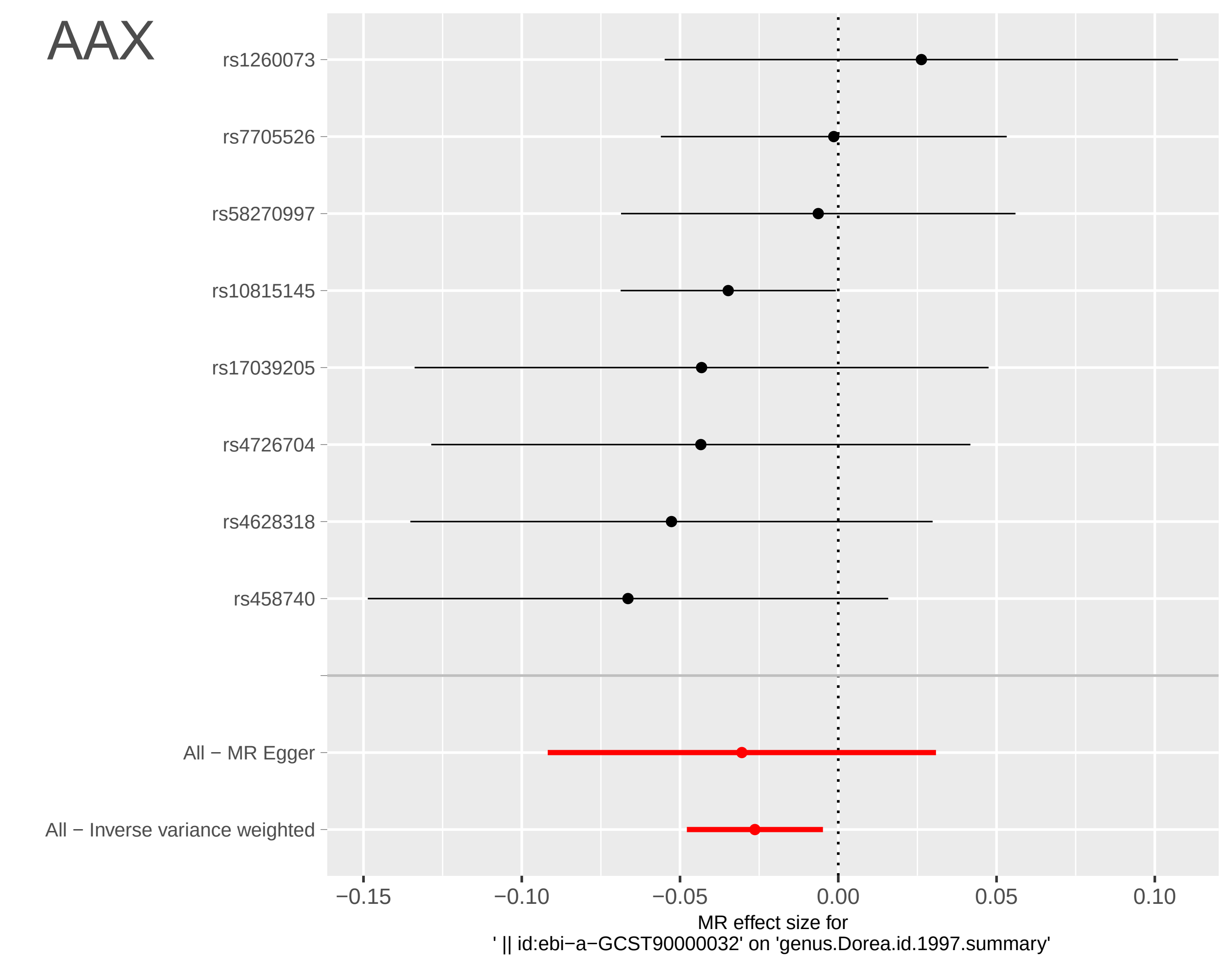
AAX
rs1260073
rs7705526
rs58270997
rs10815145
rs17039205
rs4726704
rs4628318
rs458740
All − MR Egger
All − Inverse variance weighted
−0.15
−0.10
−0.05
0.00
0.05
0.10
MR effect size for
' || id:ebi−a−GCST90000032' on 'genus.Dorea.id.1997.summary'

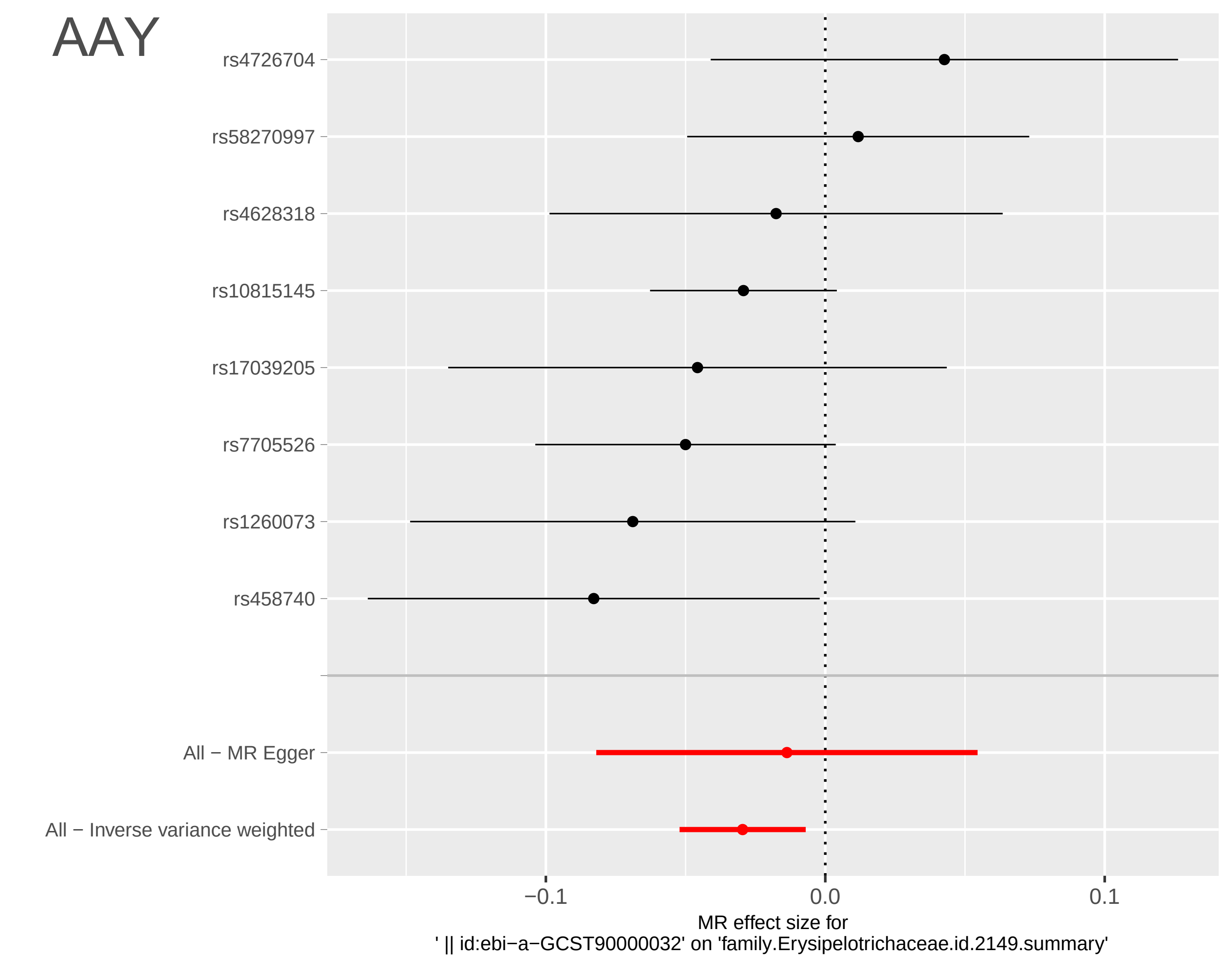
AAY
rs4726704
rs58270997
rs4628318
rs10815145
rs17039205
rs7705526
rs1260073
rs458740
All − MR Egger
All − Inverse variance weighted
−0.1
0.0
0.1
MR effect size for
' || id:ebi−a−GCST90000032' on 'family.Erysipelotrichaceae.id.2149.summary'

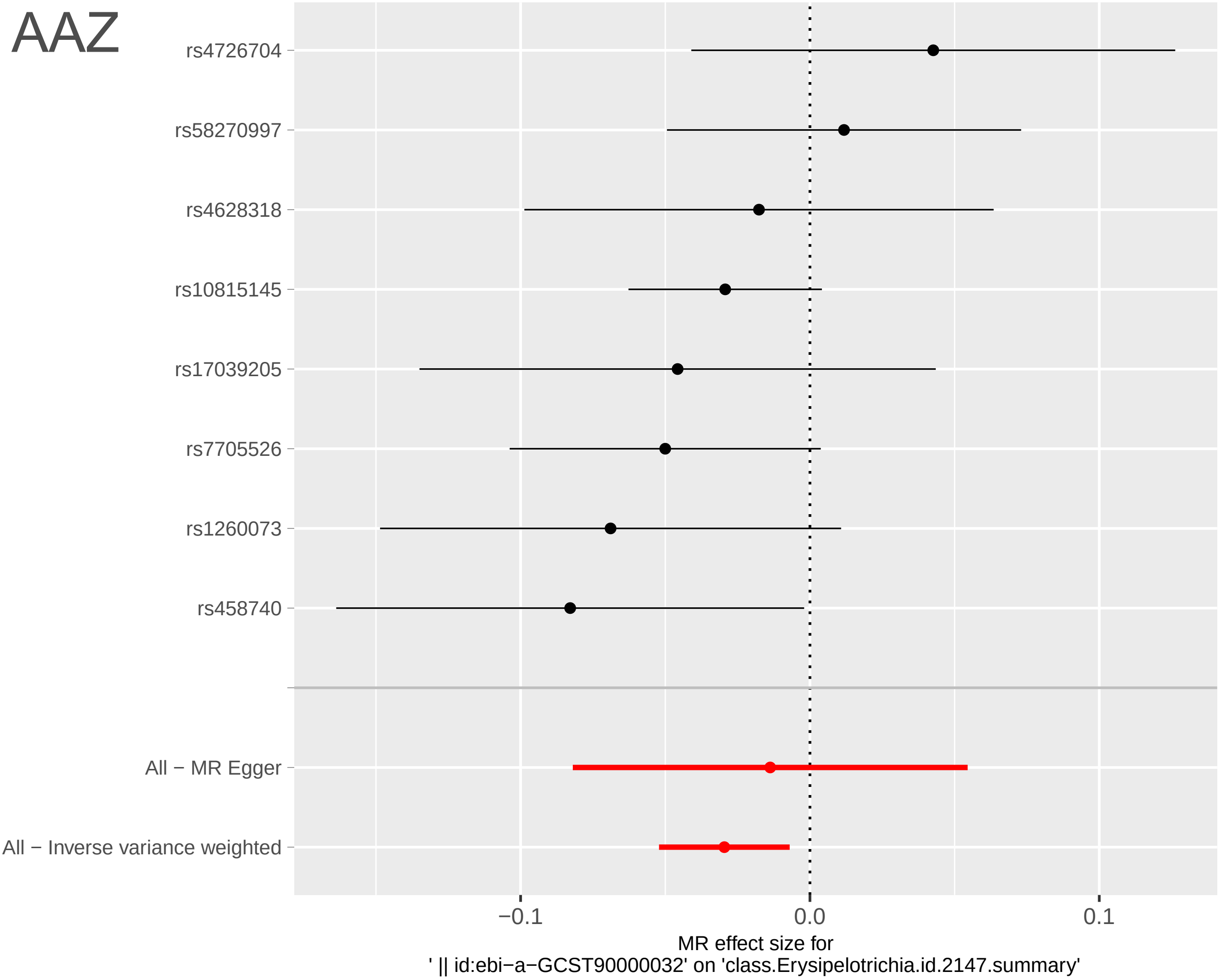
AAZ
rs4726704
rs58270997
rs4628318
rs10815145
rs17039205
rs7705526
rs1260073
rs458740
All − MR Egger
All − Inverse variance weighted
−0.1
0.0
0.1
MR effect size for
' || id:ebi−a−GCST90000032' on 'class.Erysipelotrichia.id.2147.summary'

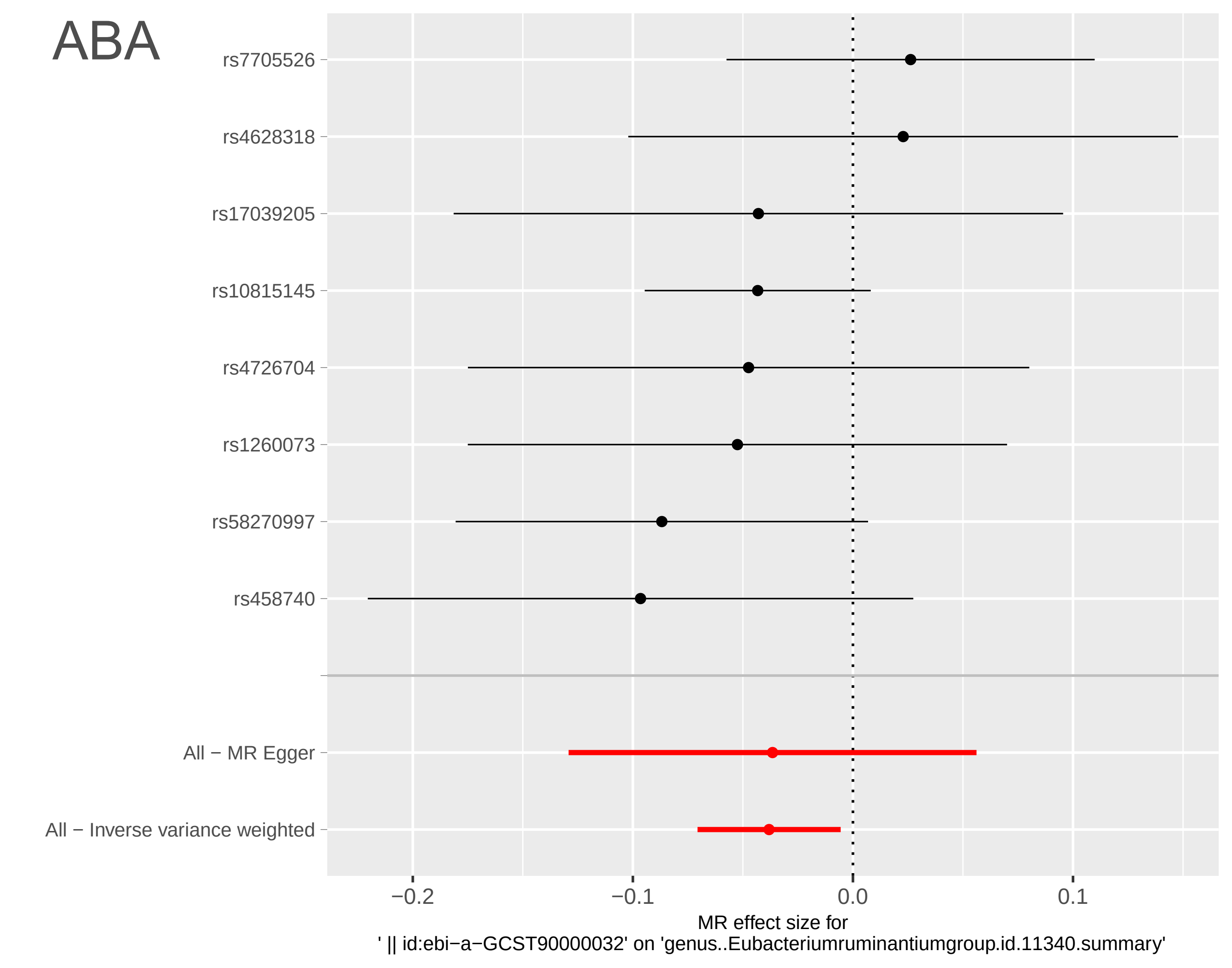
ABA
rs7705526
rs4628318
rs17039205
rs10815145
rs4726704
rs1260073
rs58270997
rs458740
All − MR Egger
All − Inverse variance weighted
−0.2
−0.1
0.0
0.1
MR effect size for
' || id:ebi−a−GCST90000032' on 'genus..Eubacteriumruminantiumgroup.id.11340.summary'

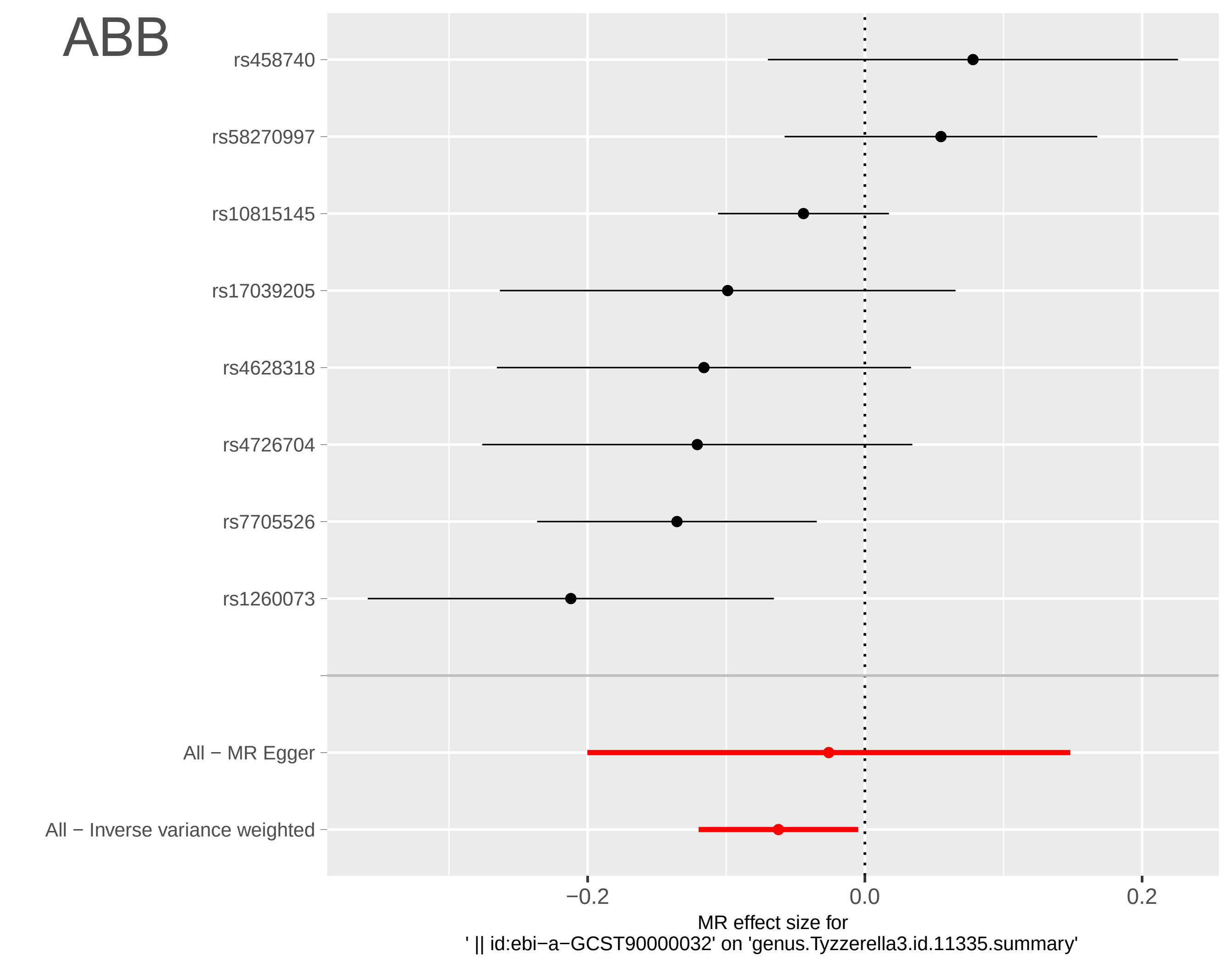
ABB
rs458740
rs58270997
rs10815145
rs17039205
rs4628318
rs4726704
rs7705526
rs1260073
All − MR Egger
All − Inverse variance weighted
−0.2
0.0
0.2
MR effect size for
' || id:ebi−a−GCST90000032' on 'genus.Tyzzerella3.id.11335.summary'

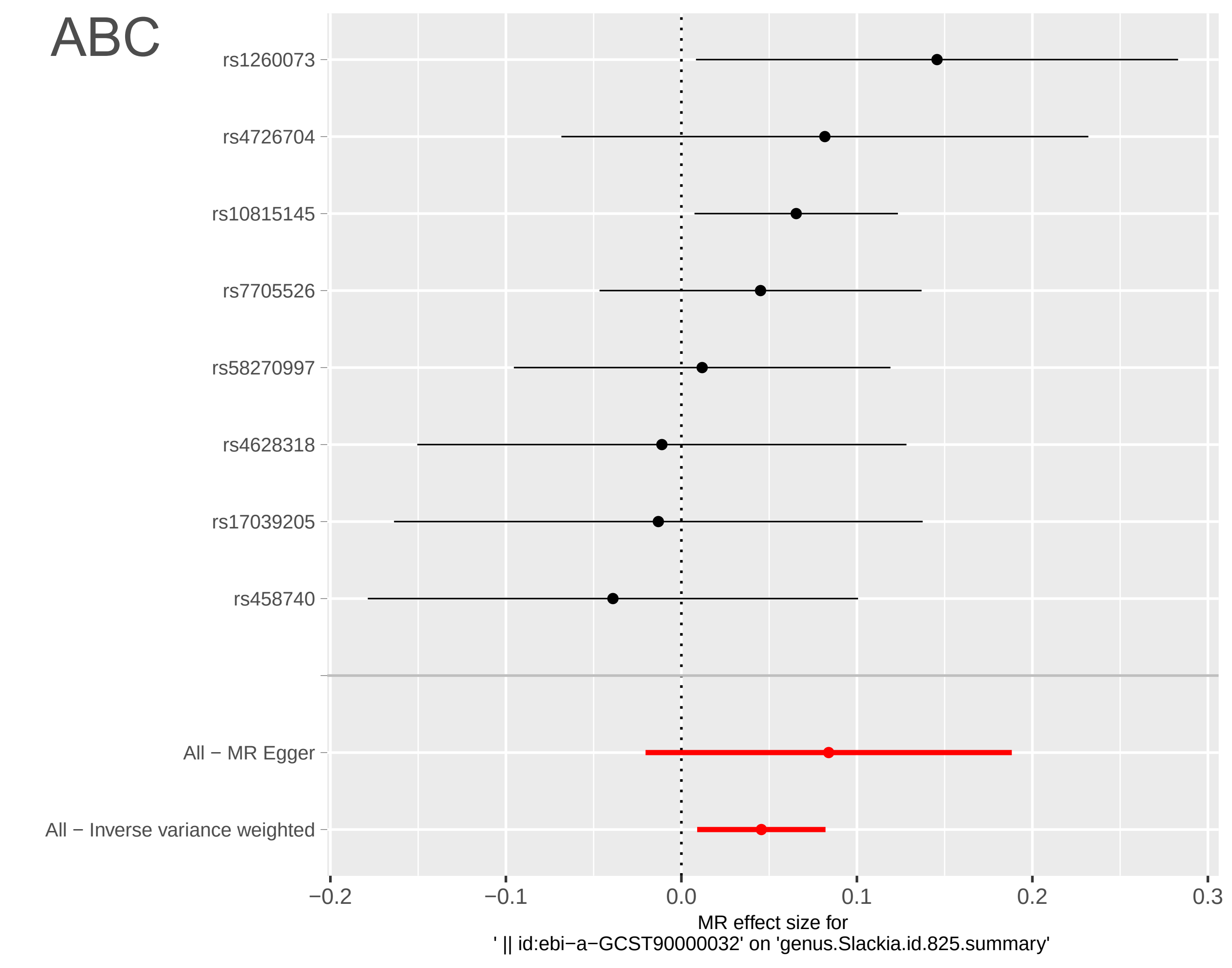
ABC
rs1260073
rs4726704
rs10815145
rs7705526
rs58270997
rs4628318
rs17039205
rs458740
All − MR Egger
All − Inverse variance weighted
−0.2
−0.1
0.0
0.1
0.2
0.3
MR effect size for
' || id:ebi−a−GCST90000032' on 'genus.Slackia.id.825.summary'

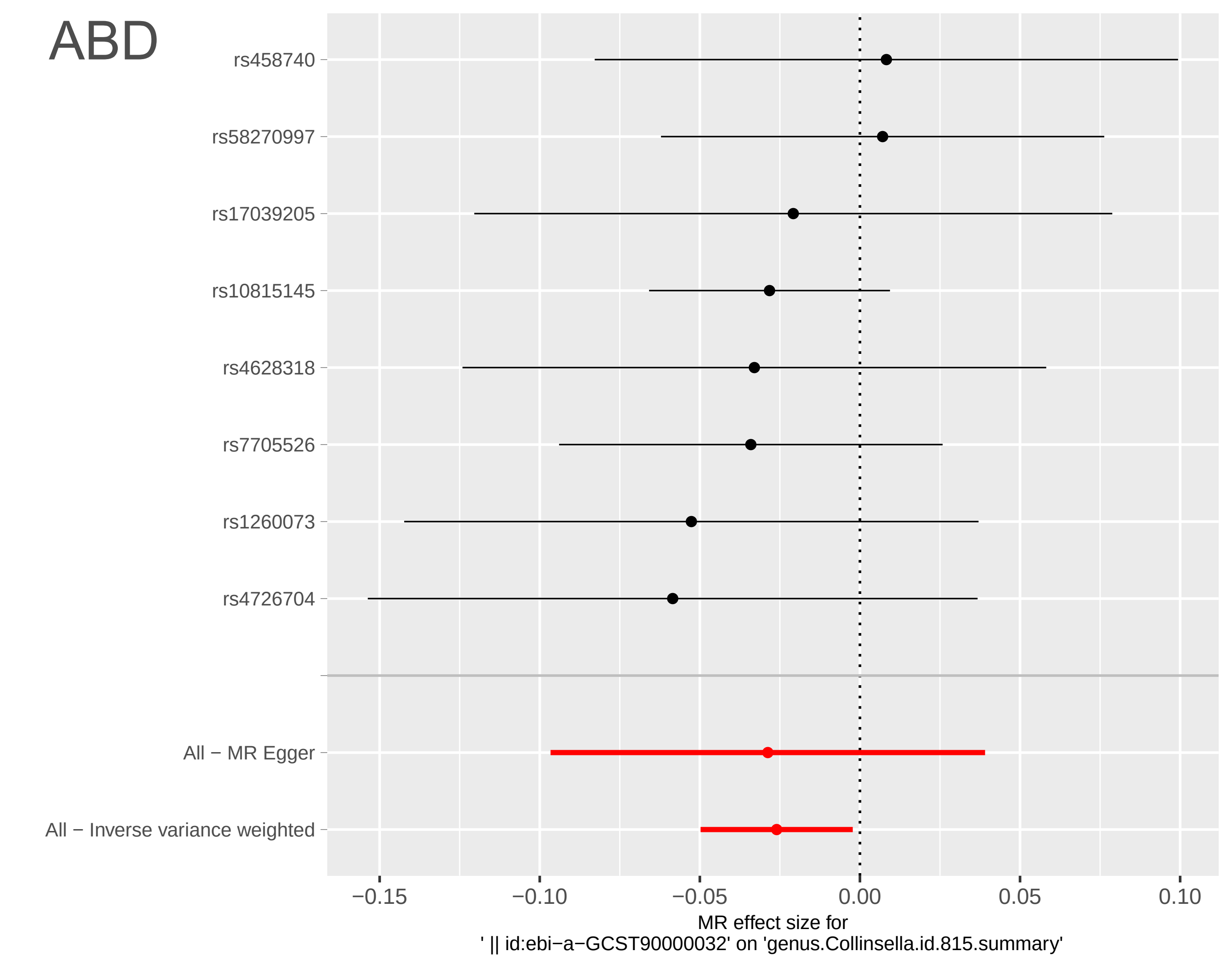
ABD
rs458740
rs58270997
rs17039205
rs10815145
rs4628318
rs7705526
rs1260073
rs4726704
All − MR Egger
All − Inverse variance weighted
−0.15
−0.10
−0.05
0.00
0.05
0.10
MR effect size for
' || id:ebi−a−GCST90000032' on 'genus.Collinsella.id.815.summary'

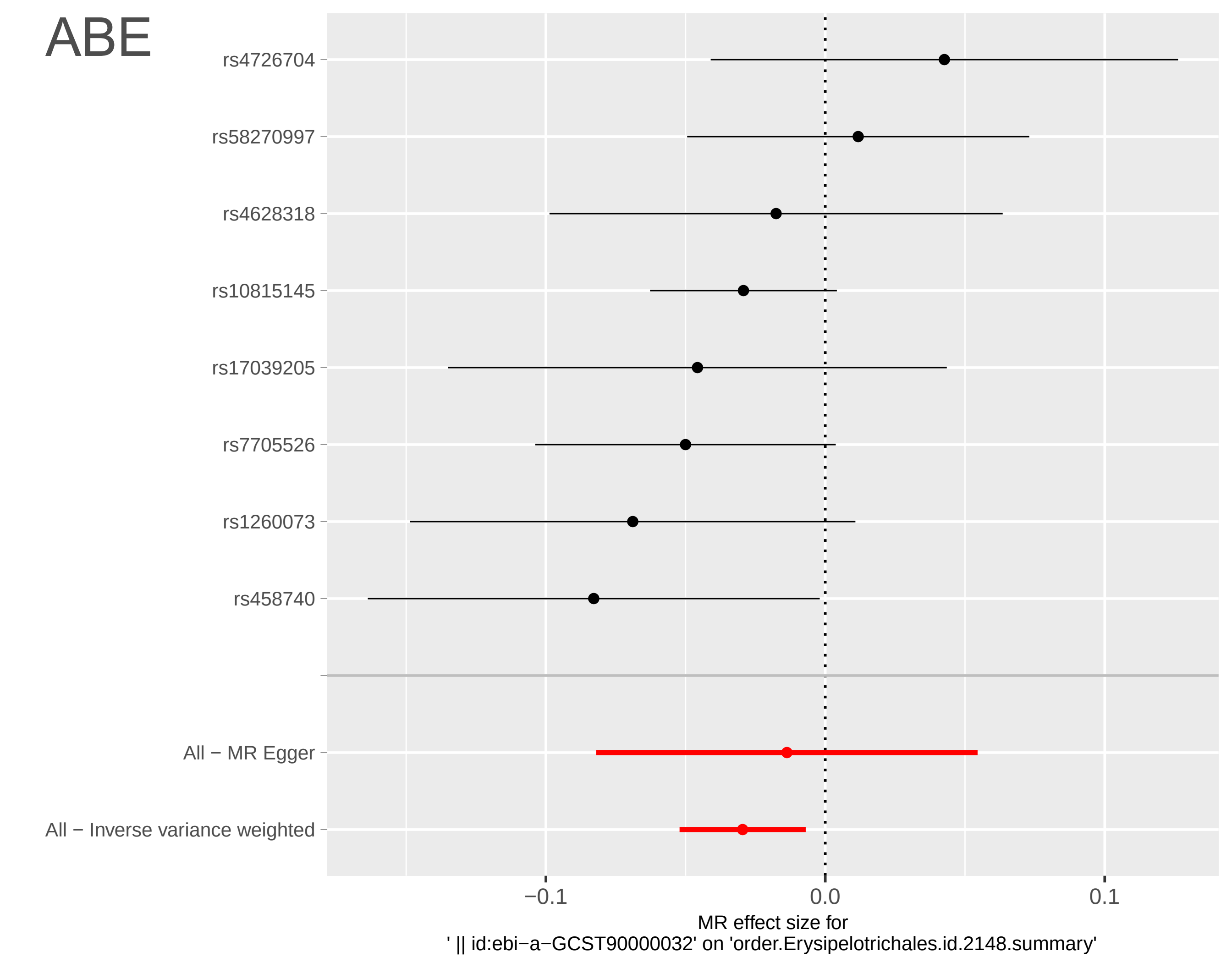
ABE
rs4726704
rs58270997
rs4628318
rs10815145
rs17039205
rs7705526
rs1260073
rs458740
All − MR Egger
All − Inverse variance weighted
−0.1
0.0
0.1
MR effect size for
' || id:ebi−a−GCST90000032' on 'order.Erysipelotrichales.id.2148.summary'

Supplementary figure 5.Sensitivity analysis of the impact of hematologic malignancies on gut microbiota using MR. (forest plot)

A.Forest plot between lymphoid leukemia and order.Enterobacteriales

B.Forest plot between lymphoid leukemia and genus Eubacteriumbrachygroup

C.Forest plot between lymphoid leukemia and genus.LachnospiraceaeNC2004group

D.Forest plot between lymphoid leukemia and genus.Anaerofilum.

E.Forest plot between lymphoid leukemia and genus.Butyrivibrio

F.Forest plot between lymphoid leukemia and genus.Escherichia.Shigella

G.Forest plot between lymphoid leukemia and family.Enterobacteriaceae

H.Forest plot between lymphoid leukemia and genus.Fusicatenibacter

I.Forest plot between lymphoid leukemia and genus.LachnospiraceaeUCG010

J.Forest plot between myeloid leukemia and family.Bifidobacteriaceae

K.Forest plot between myeloid leukemia and class.Actinobacteria

L.Forest plot between myeloid leukemia and class.Clostridia

M.Forest plot between myeloid leukemia and phylum.Bacteroidetes

N.Forest plot between myeloid leukemia and genus..Ruminococcusgauvreauiigroup

O.Forest plot between myeloid leukemia and order.Clostridiales

P.Forest plot between myeloid leukemia and phylum.Actinobacteria.

Q.Forest plot between myeloid leukemia and order.Bacteroidales

R.Forest plot between myeloid leukemia and class.Bacteroidia

S.Forest plot between myeloid leukemia and genus.Bifidobacterium

T.Forest plot between myeloid leukemia and family.Lachnospiraceae

U.Forest plot between myeloid leukemia and genus.Collinsella

V.Forest plot between myeloid leukemia and genus.Blautia

W.Forest plot between myeloid leukemia and genus.Fusicatenibacter

X.Forest plot between myeloid leukemia and order.Bifidobacteriales

Y.Forest plot between myeloid leukemia and genus..Ruminococcusgnavusgroup

Z.Forest plot between myeloid leukemia and genus..Eubacteriumhalliigroup

AA..Forest plot between myeloid leukemia and genus.Slackia.

AB.Forest plot between myeloid leukemia and phylum.Firmicutes

AC.Forest plot between Hodgkin lymphoma and phylum.Verrucomicrobia

AD.Forest plot between Hodgkin lymphoma and family.ClostridialesvadinBB60group

AE.Forest plot between Hodgkin lymphoma and genus.RuminococcaceaeUCG010

AF.Forest plot between Hodgkin lymphoma and genus.Odoribacter

AG.Forest plot between Hodgkin lymphoma and genus.RuminococcaceaeUCG005

AH.Forest plot between Hodgkin lymphoma and genus.RuminococcaceaeUCG013

AI. Forest plot between Hodgkin lymphoma and genus.Ruminiclostridium9

AJ.Forest plot between Hodgkin lymphoma and genus.RuminococcaceaeNK4A214group

AK.Forest plot between Hodgkin lymphoma and genus..Eubacteriumventriosumgroup

AL.Forest plot between Hodgkin lymphoma and genus.RuminococcaceaeUCG003

AM.Forest plot between malignant plasma cell tumor and family.Defluviitaleaceae

AN. Forest plot between malignant plasma cell tumor and genus.DefluviitaleaceaeUCG011

AO.Forest plot between malignant plasma cell tumor and genus..Eubacteriumrectalegroup

AP.Forest plot between malignant plasma cell tumor and genus.CandidatusSoleaferrea

AQ. Forest plot between follicular lymphoma and genus.Allisonella

AR.Forest plot between follicular lymphoma and genus.RikenellaceaeRC9gutgroup

AS.Forest plot between follicular lymphoma and genus.Faecalibacterium

AT.Forest plot between follicular lymphoma and family.Clostridiaceae

AU.Forest plot between follicular lymphoma and genus.DefluviitaleaceaeUCG011.

AV.Forest plot between follicular lymphoma and genus.Clostridiumsensustricto

AW.Forest plot between follicular lymphoma and family.Defluviitaleaceae

AX.Forest plot between diffuse large B-cell lymphomaand family.Veillonellaceae

AY.Forest plot between diffuse large B-cell lymphomaand genus.Fusicatenibacter

AZ.Forest plot between diffuse large B-cell lymphomaand genus.Eggerthella

AAA.Forest plot between diffuse large B-cell lymphomaand genus.Erysipelatoclostridium

AAB.Forest plot between diffuse large B-cell lymphomaand genus.Blautia

AAC.Forest plot between diffuse large B-cell lymphomaand genus.RuminococcaceaeUCG009

AAD.Forest plot between mature T/NK-cell lymphomas and order.Verrucomicrobiales

AAE.Forest plot between mature T/NK-cell lymphomas and phylum.Tenericutes

AAF.Forest plot between mature T/NK-cell lymphomas and genus.Lachnoclostridium

AAG.Forest plot between mature T/NK-cell lymphomas and phylum.Bacteroidetes

AAH.Forest plot between mature T/NK-cell lymphomas and class.Verrucomicrobiae

AAI.Forest plot between mature T/NK-cell lymphomas and genus..Eubacteriumrectalegroup

AAJ.Forest plot between mature T/NK-cell lymphomas and family.Verrucomicrobiaceae

AAK.Forest plot between mature T/NK-cell lymphomas and order.Bacteroidales

AAL. Forest plot between mature T/NK-cell lymphomas and genus.Akkermansia

AAM. Forest plot between mature T/NK-cell lymphomas and genus.RuminococcaceaeUCG005

AAN.Forest plot between mature T/NK-cell lymphomas and class.Mollicutes.

AAO. Forest plot between mature T/NK-cell lymphomas and class.Bacteroidia

AAP.Forest plot between mature T/NK-cell lymphomas and family.Lachnospiraceae

AAQ.Forest plot between mature T/NK-cell lymphomas and order.Pasteurellales

AAR. Forest plot between mature T/NK-cell lymphomas and family.Pasteurellaceae.

AAS. Forest plot between mature T/NK-cell lymphomas and phylum.Verrucomicrobia

AAT.Forest plot between mature T/NK-cell lymphomas and genus.RuminococcaceaeUCG010

AAU.Forest plot between mature T/NK-cell lymphomas and family.Veillonellaceae.

AAV.Forest plot between myeloproliferative neoplasms and genus.Sellimonas

AAW.Forest plot between myeloproliferative neoplasms and genus.Coprococcus1

AAX.Forest plot between myeloproliferative neoplasms and genus.Dorea

AAY.Forest plot between myeloproliferative neoplasms and family.Erysipelotrichaceae

AAZ.Forest plot between myeloproliferative neoplasms and class.Erysipelotrichia

ABA.Forest plot between myeloproliferative neoplasms and genus..Eubacteriumruminantiumgroup

ABB.Forest plot between myeloproliferative neoplasms and genus.Tyzzerella3

ABC.Forest plot between myeloproliferative neoplasms and genus.Slackia

ABD.Forest plot between myeloproliferative neoplasms and genus.Collinsella

ABE.Forest plot between myeloproliferative neoplasms and order.Erysipelotrichales

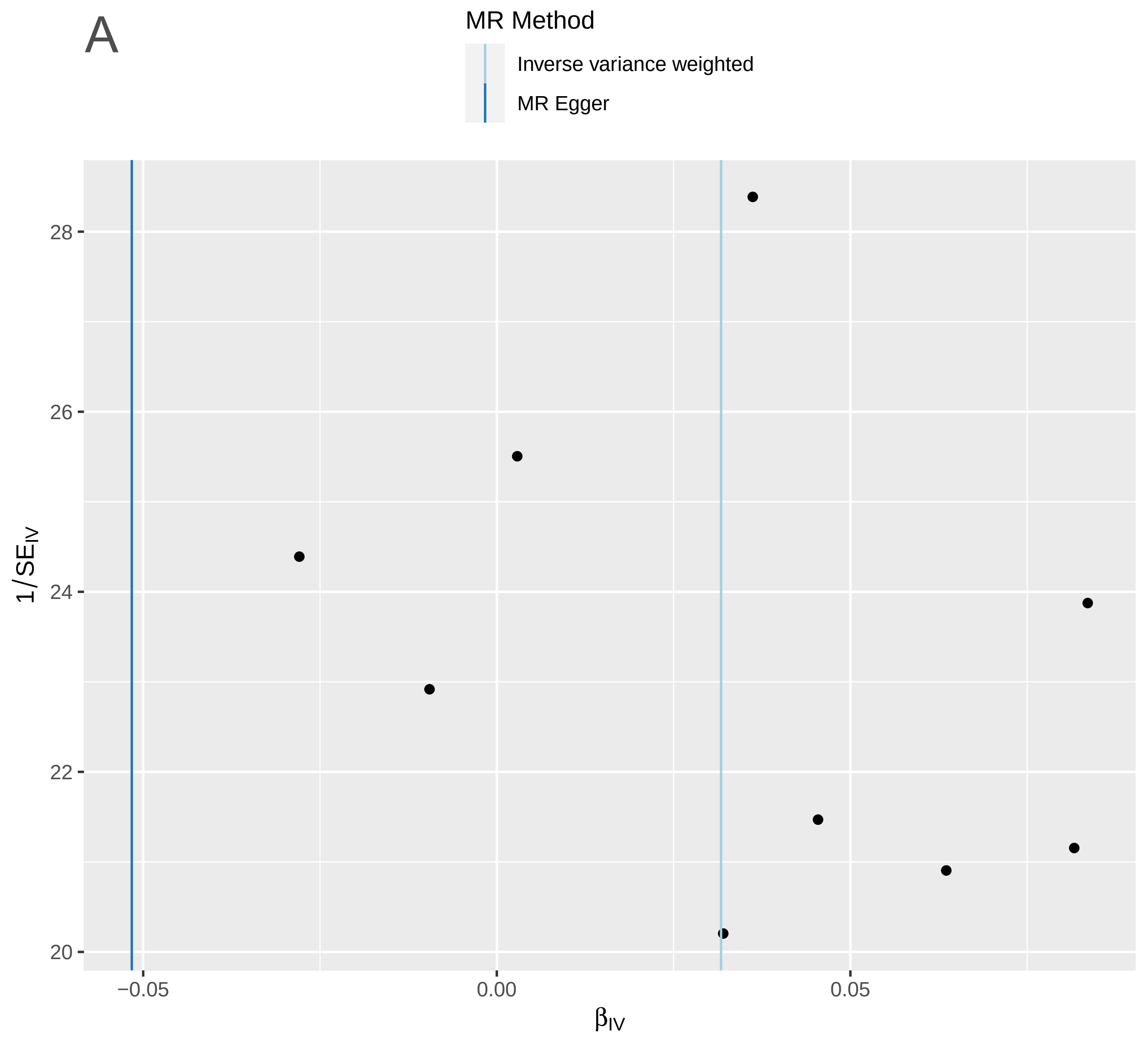
A
MR Method
Inverse variance weighted
MR Egger
28
26
24
22
20
1/SE$_{IV}$
−0.05
0.00
0.05
β$_{IV}$

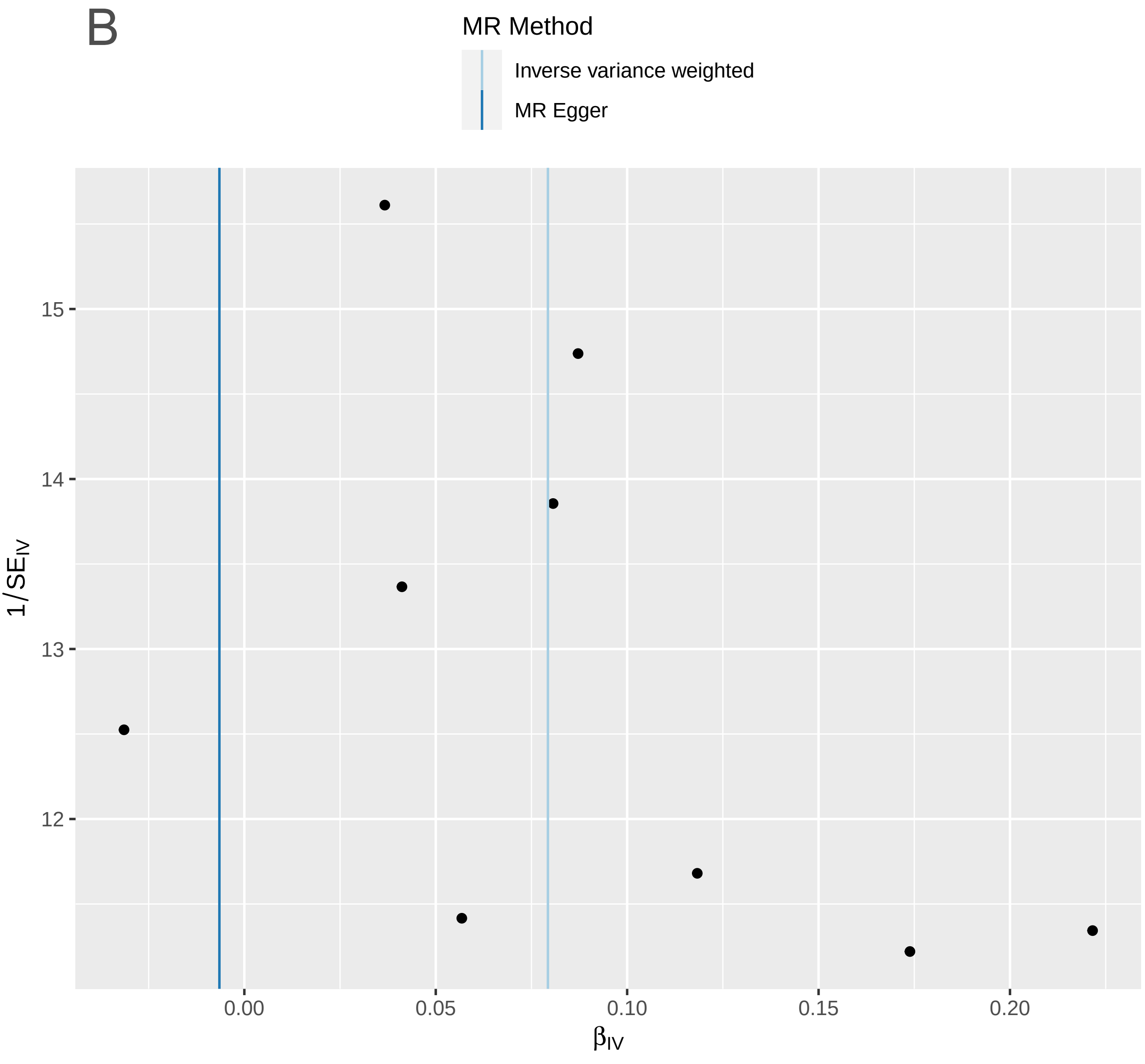
B
MR Method
Inverse variance weighted
MR Egger
15
14
13
12
1/SE_IV
0.00
0.05
0.10
0.15
0.20
β_IV

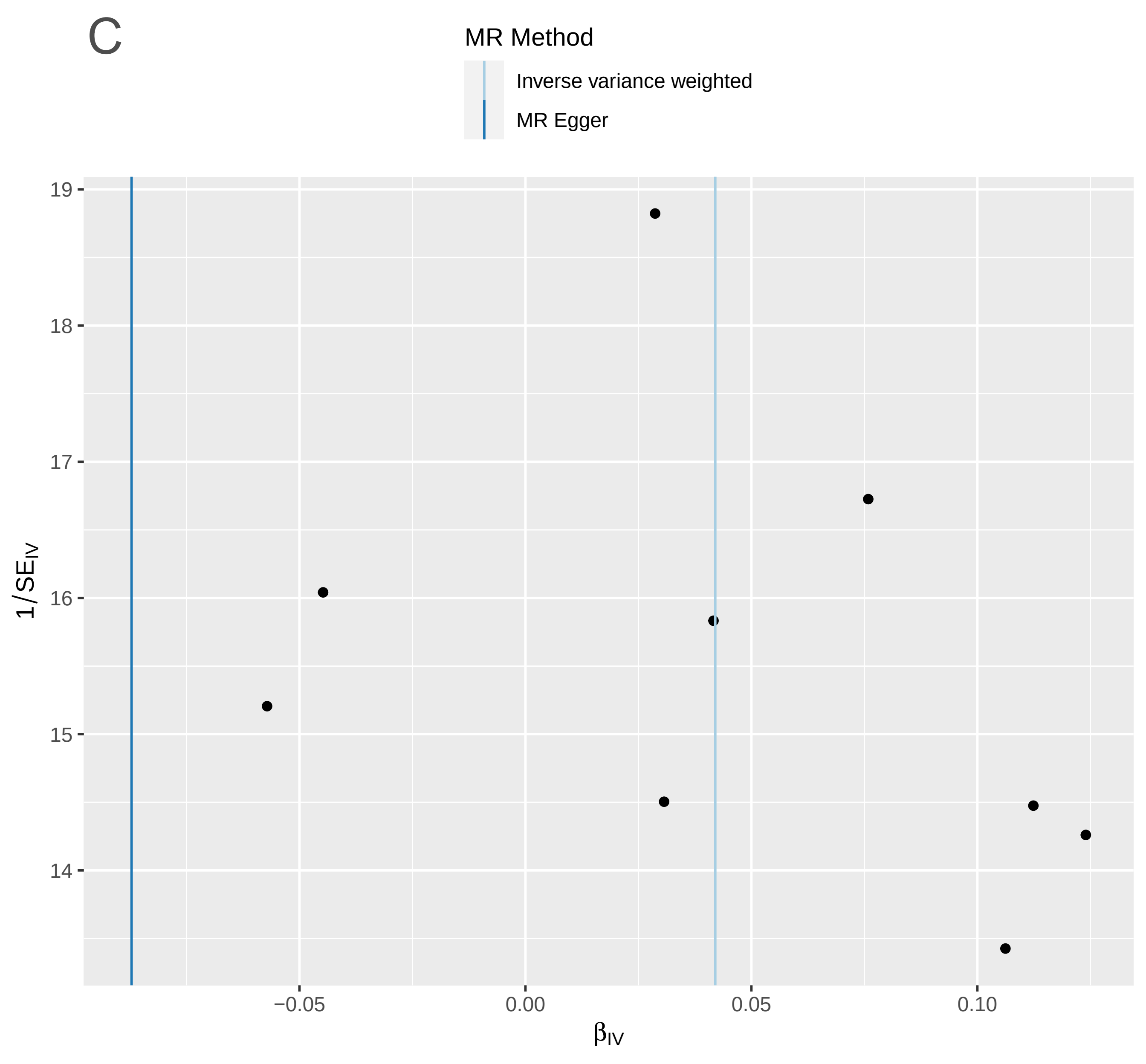
C
MR Method
Inverse variance weighted
MR Egger
19
18
17
16
15
14
−0.05
0.00
0.05
0.10
1/SE$_{IV}$
β$_{IV}$

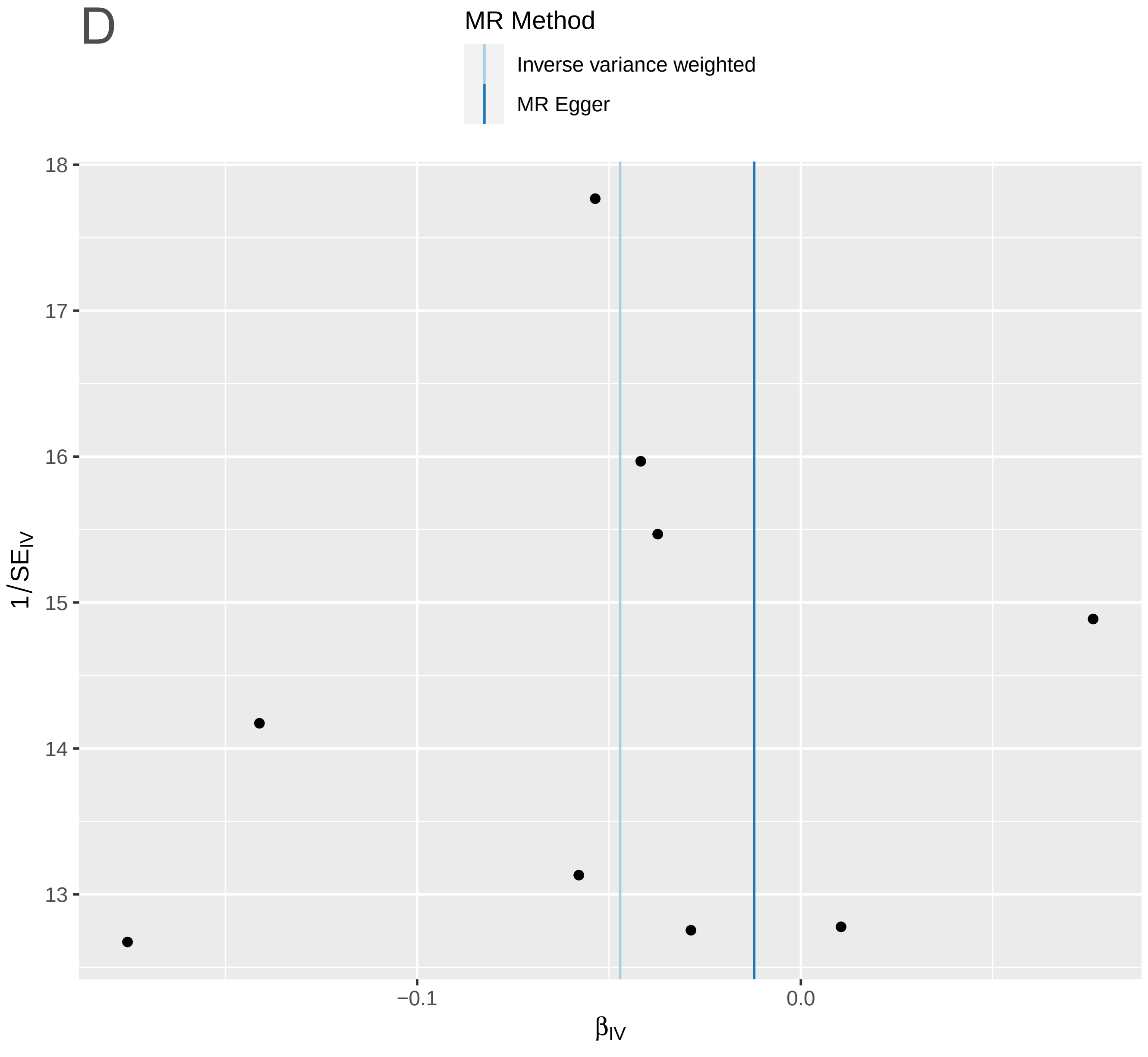

D
MR Method
Inverse variance weighted
MR Egger
18
17
16
15
14
13
−0.1
0.0
1/SE_IV
β_IV

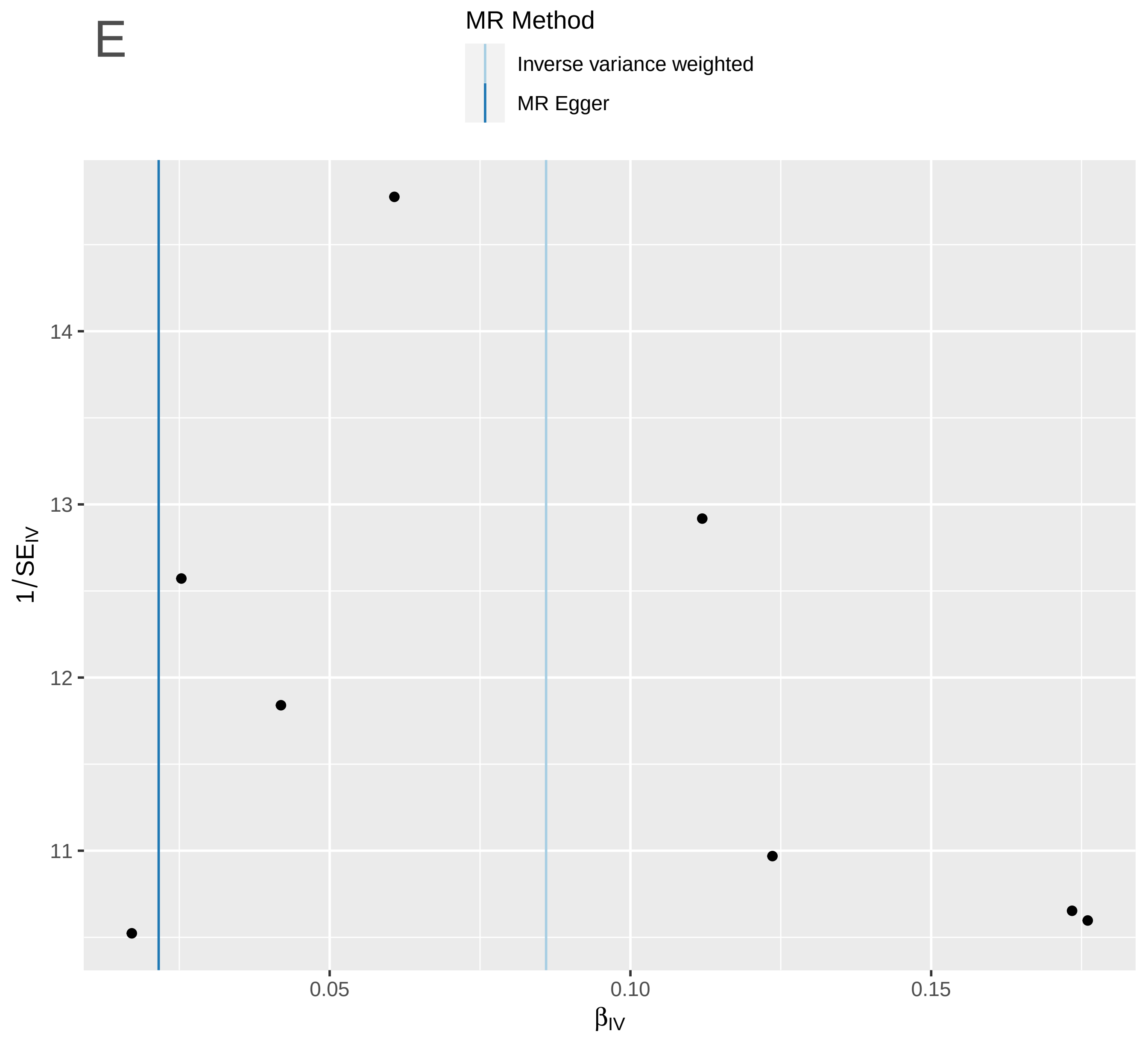

E
MR Method
Inverse variance weighted
MR Egger
14
13
12
11
1/SE_{IV}
0.05
0.10
0.15
β_{IV}

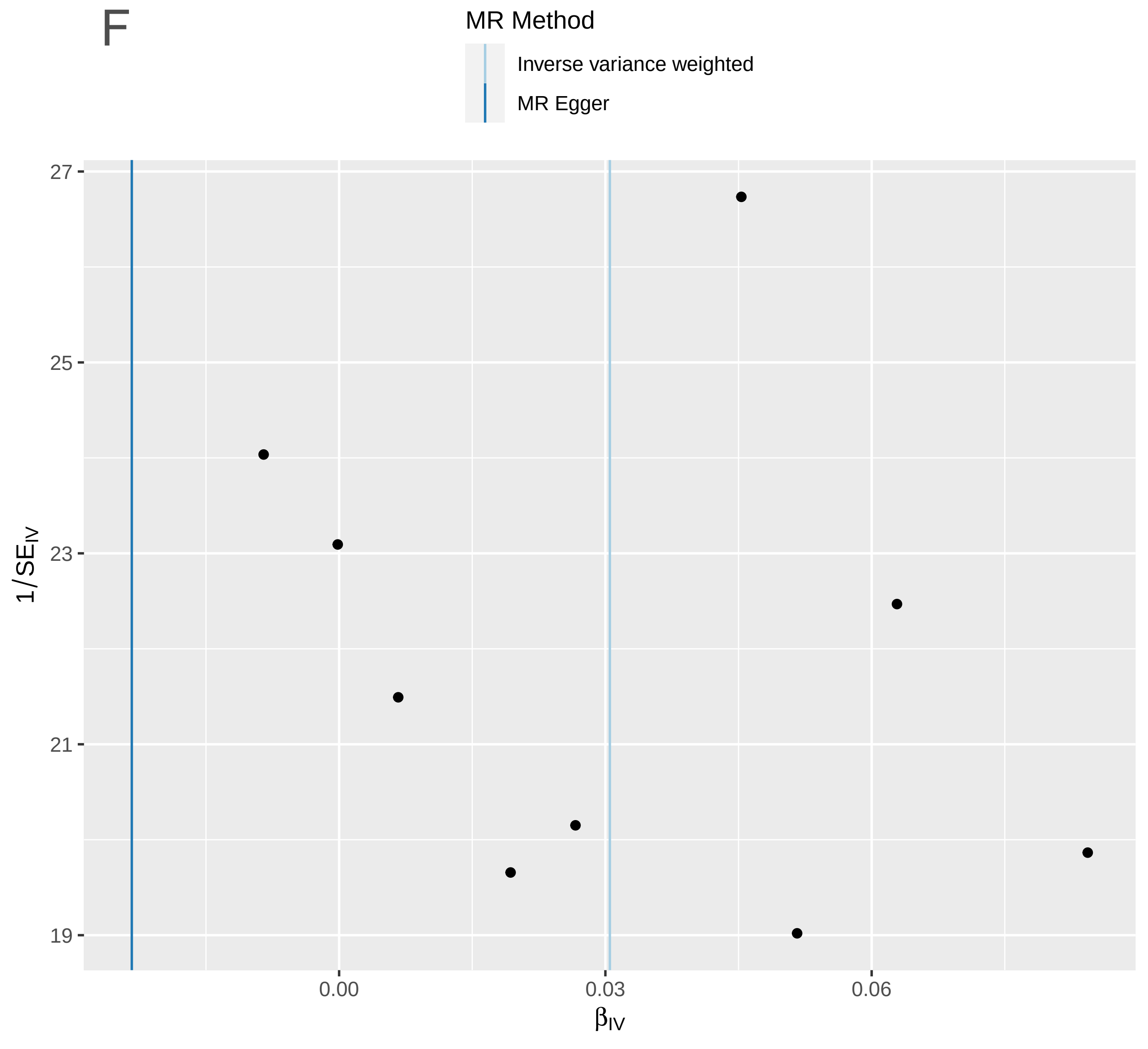
F
MR Method
Inverse variance weighted
MR Egger
27
25
23
21
19
1/SE$_{IV}$
0.00
0.03
0.06
β$_{IV}$

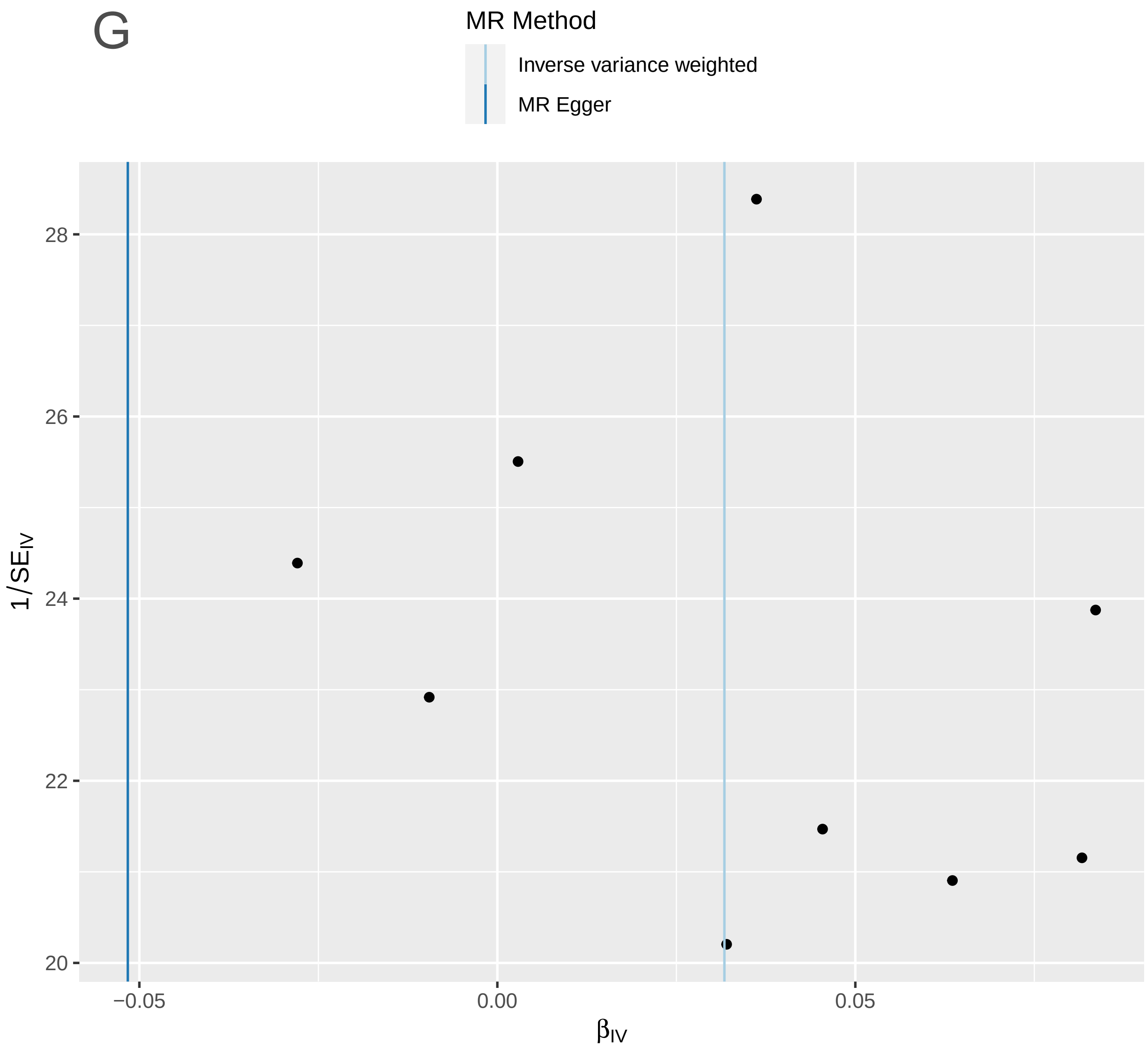
G
MR Method
Inverse variance weighted
MR Egger
28
26
24
22
20
$1/SE_{IV}$
−0.05
0.00
0.05
β_{IV}

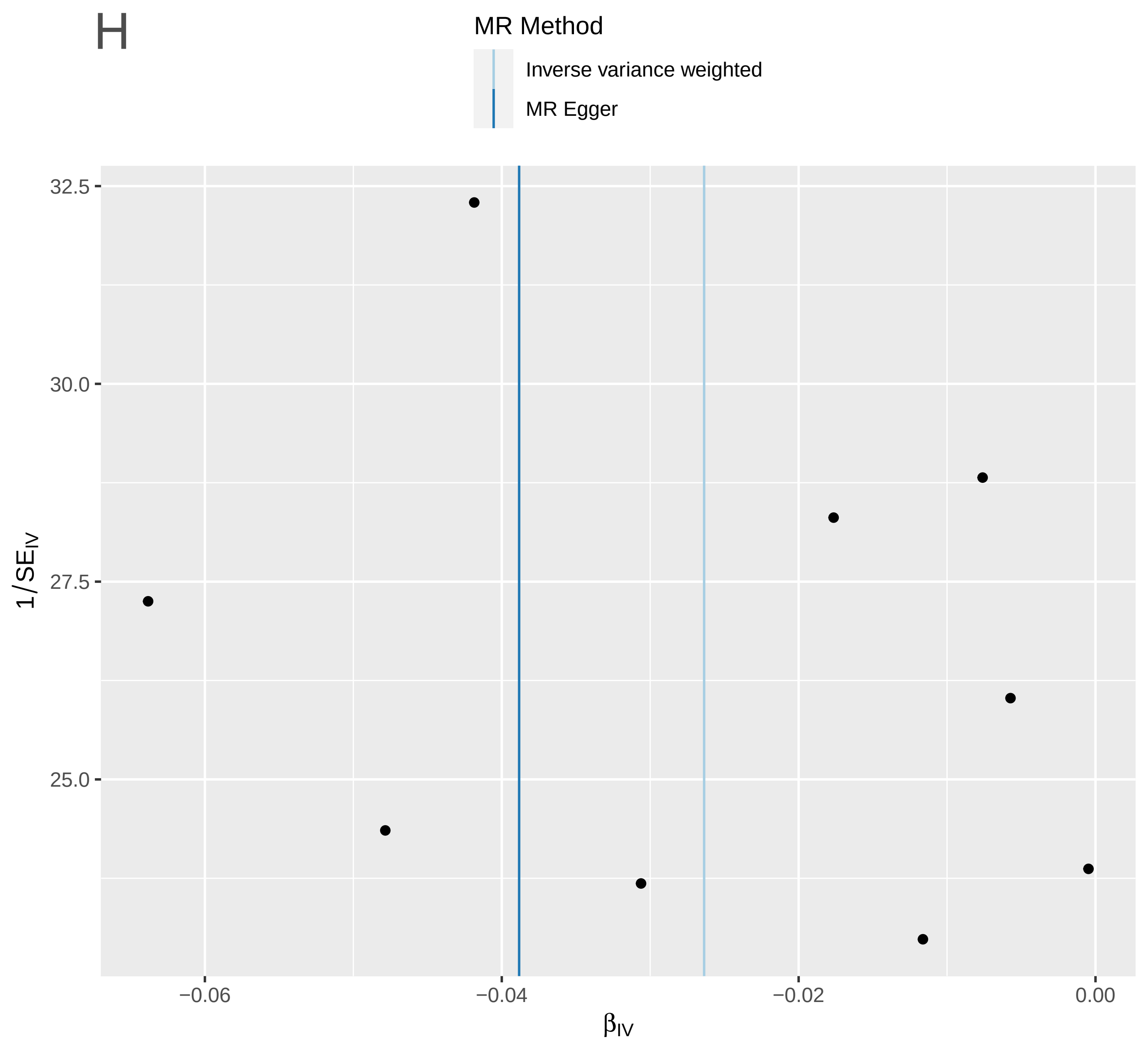
H
MR Method
Inverse variance weighted
MR Egger
32.5
30.0
27.5
25.0
1/SE$_{IV}$
−0.06
−0.04
−0.02
0.00
β$_{IV}$

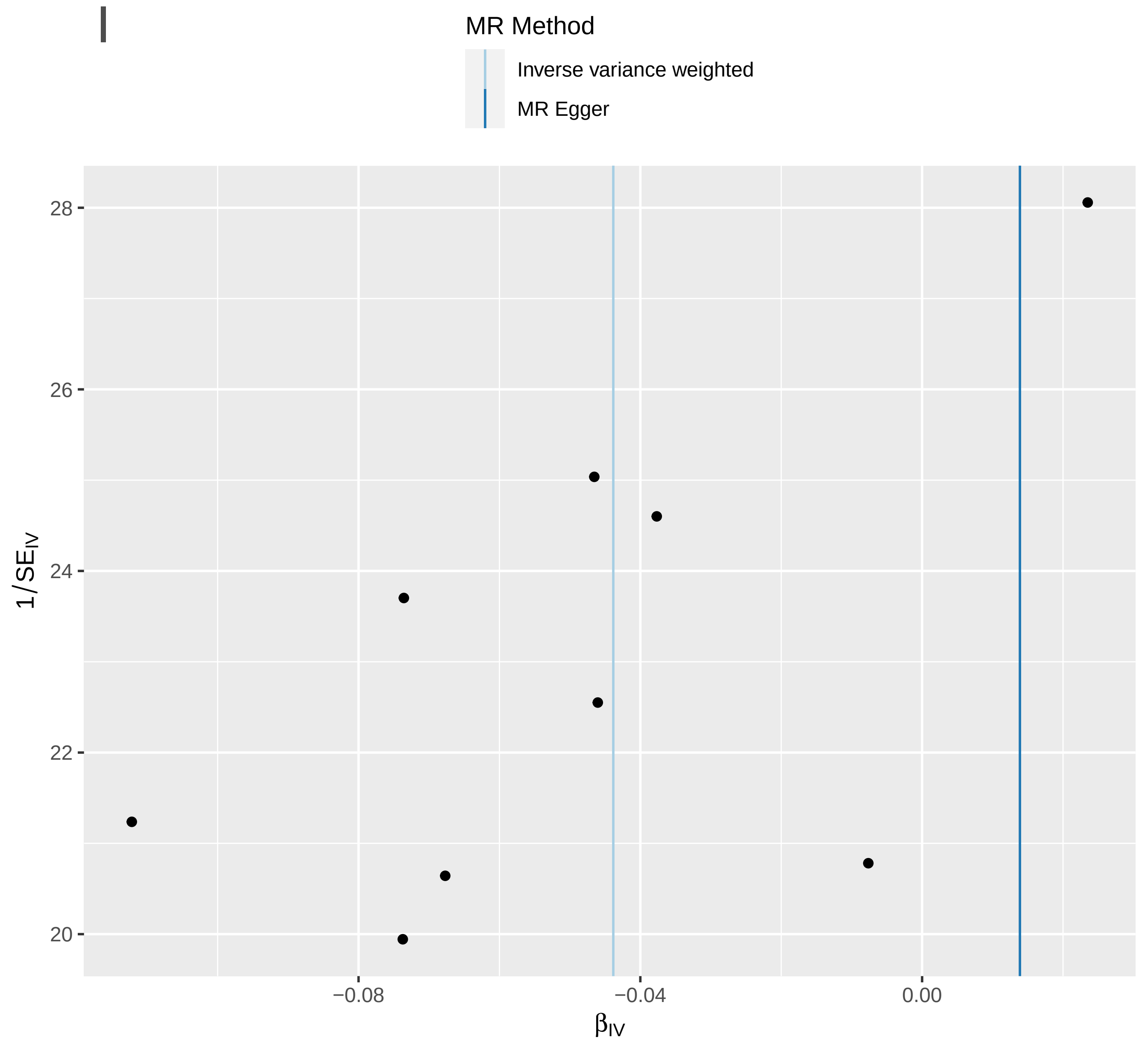

I
MR Method
Inverse variance weighted
MR Egger
28
26
24
22
20
1/SE$_{IV}$
−0.08
−0.04
0.00
β$_{IV}$

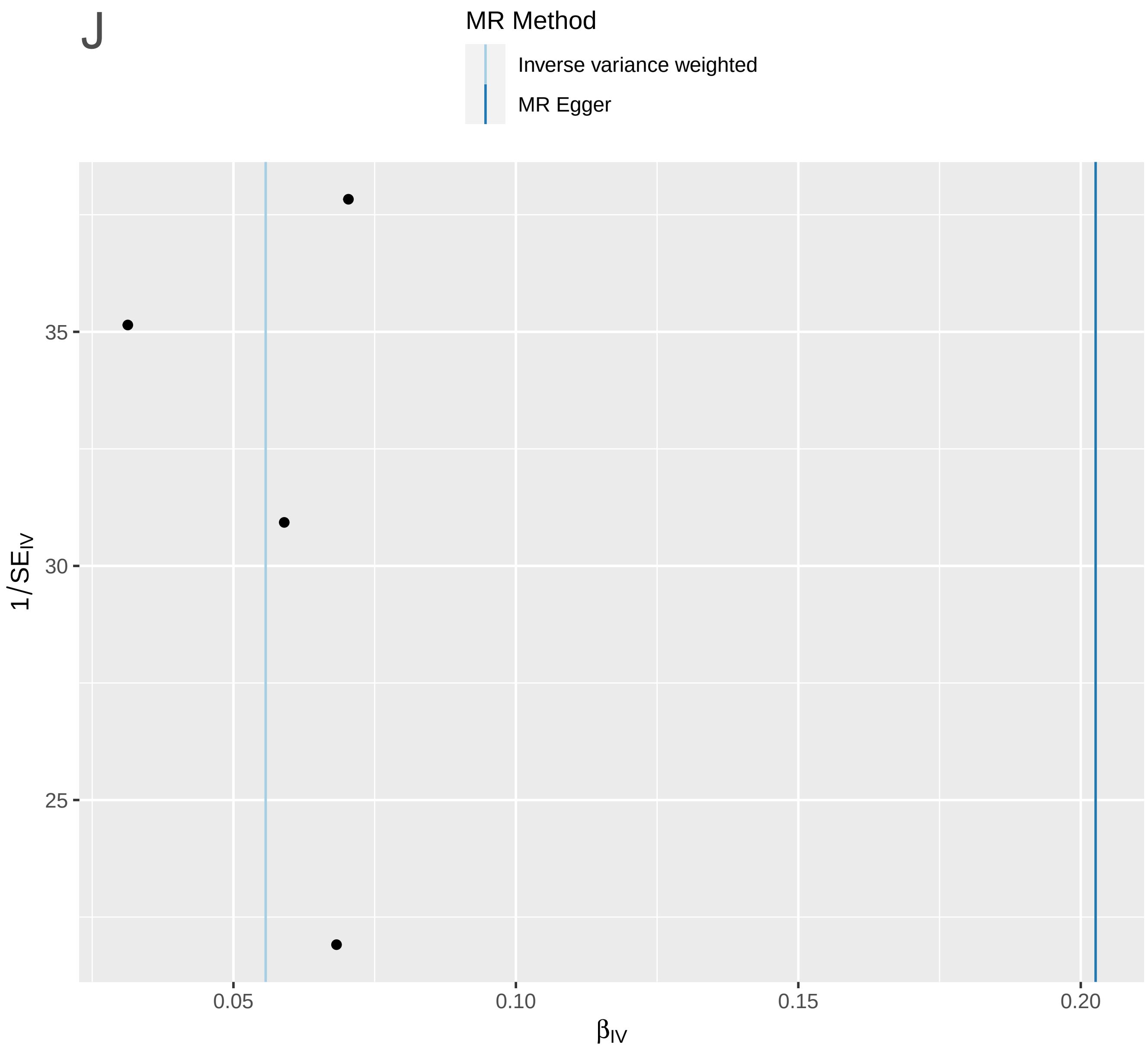
J
MR Method
Inverse variance weighted
MR Egger
35
30
25
$1/SE_{IV}$
0.05
0.10
0.15
0.20
β_{IV}

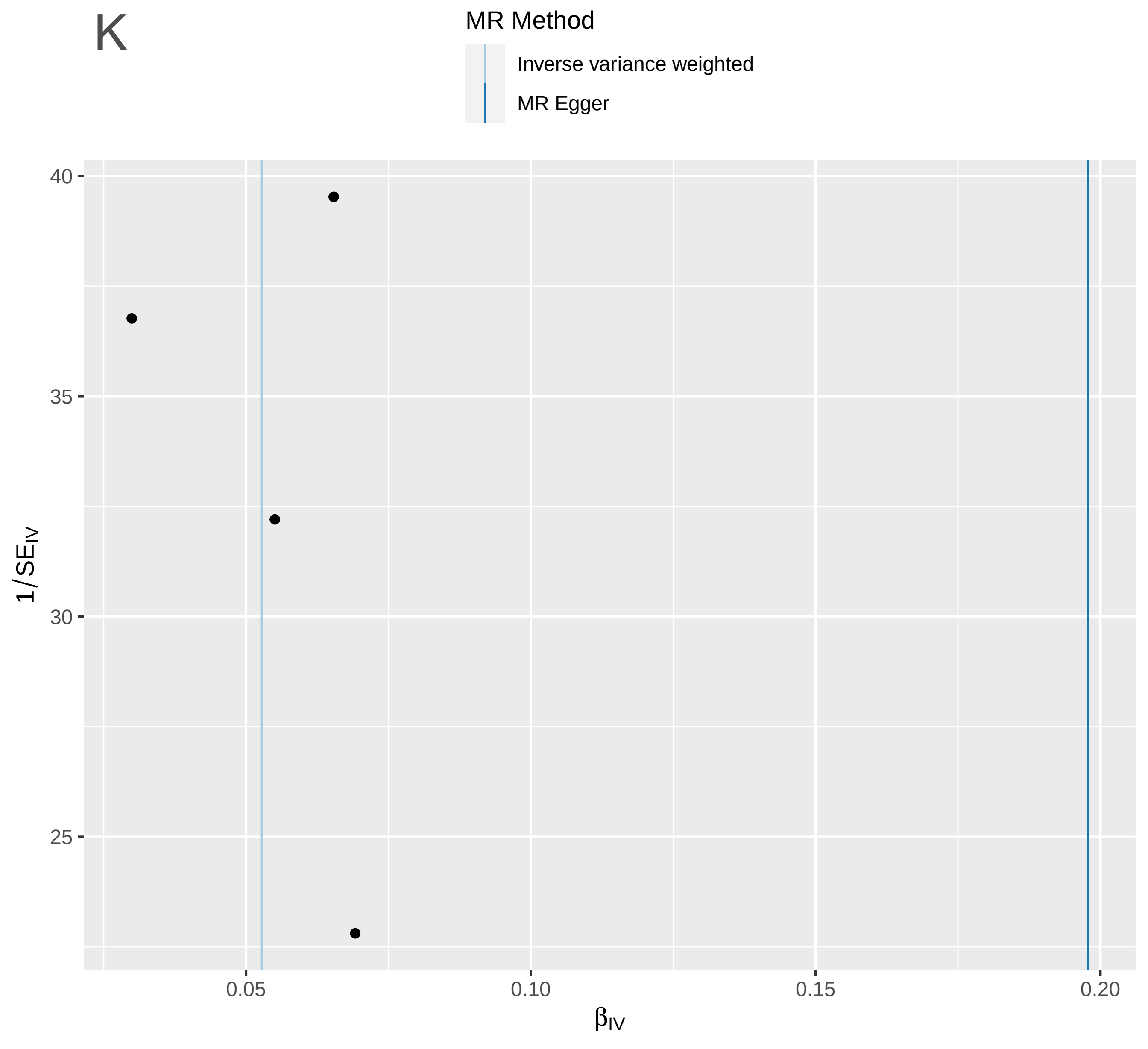

K
MR Method
Inverse variance weighted
MR Egger
40
35
30
25
1/SE$_{IV}$
0.05
0.10
0.15
0.20
β_{IV}

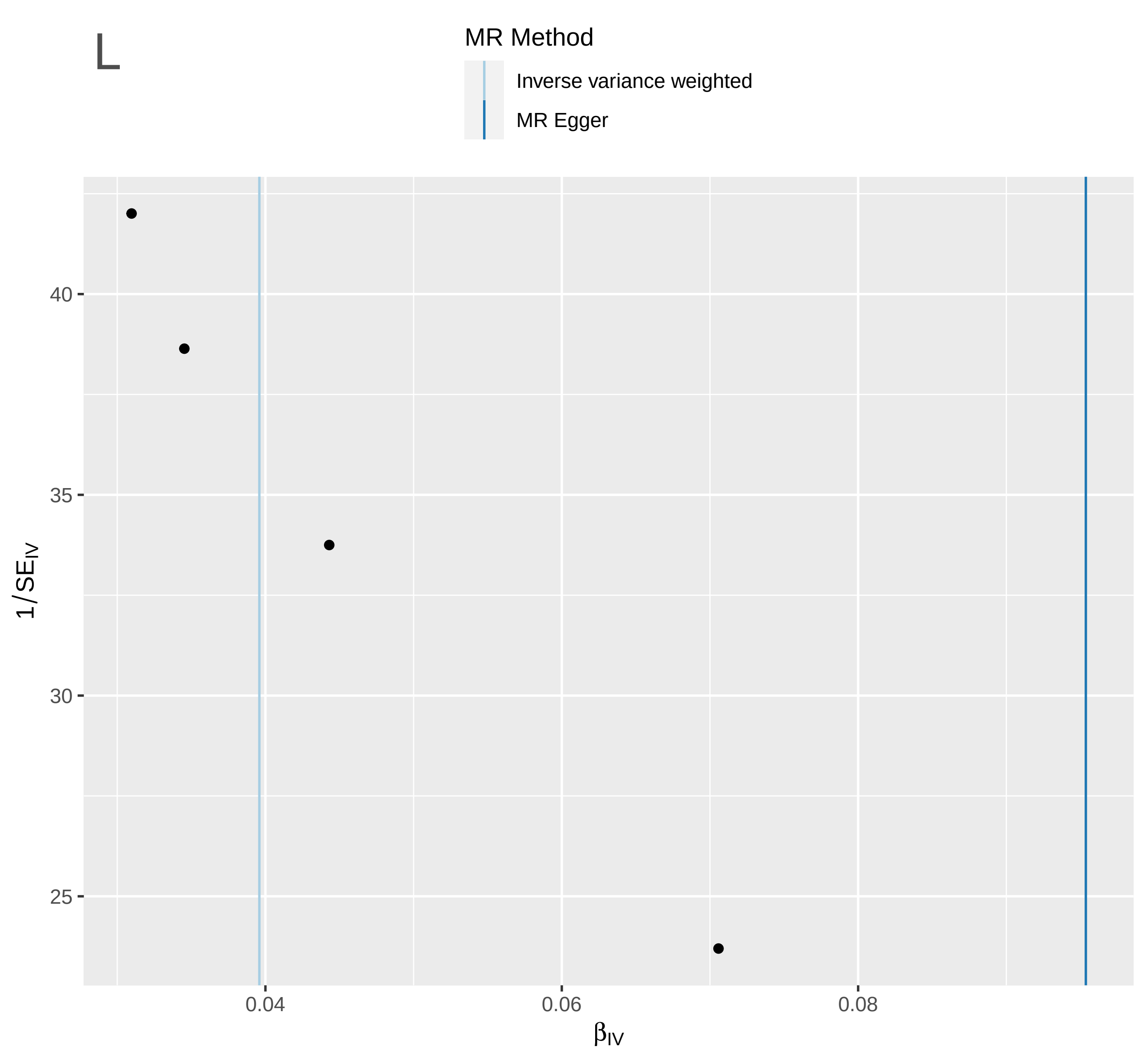
L
MR Method
Inverse variance weighted
MR Egger
40
35
30
25
1/SE_IV
0.04
0.06
0.08
β_IV

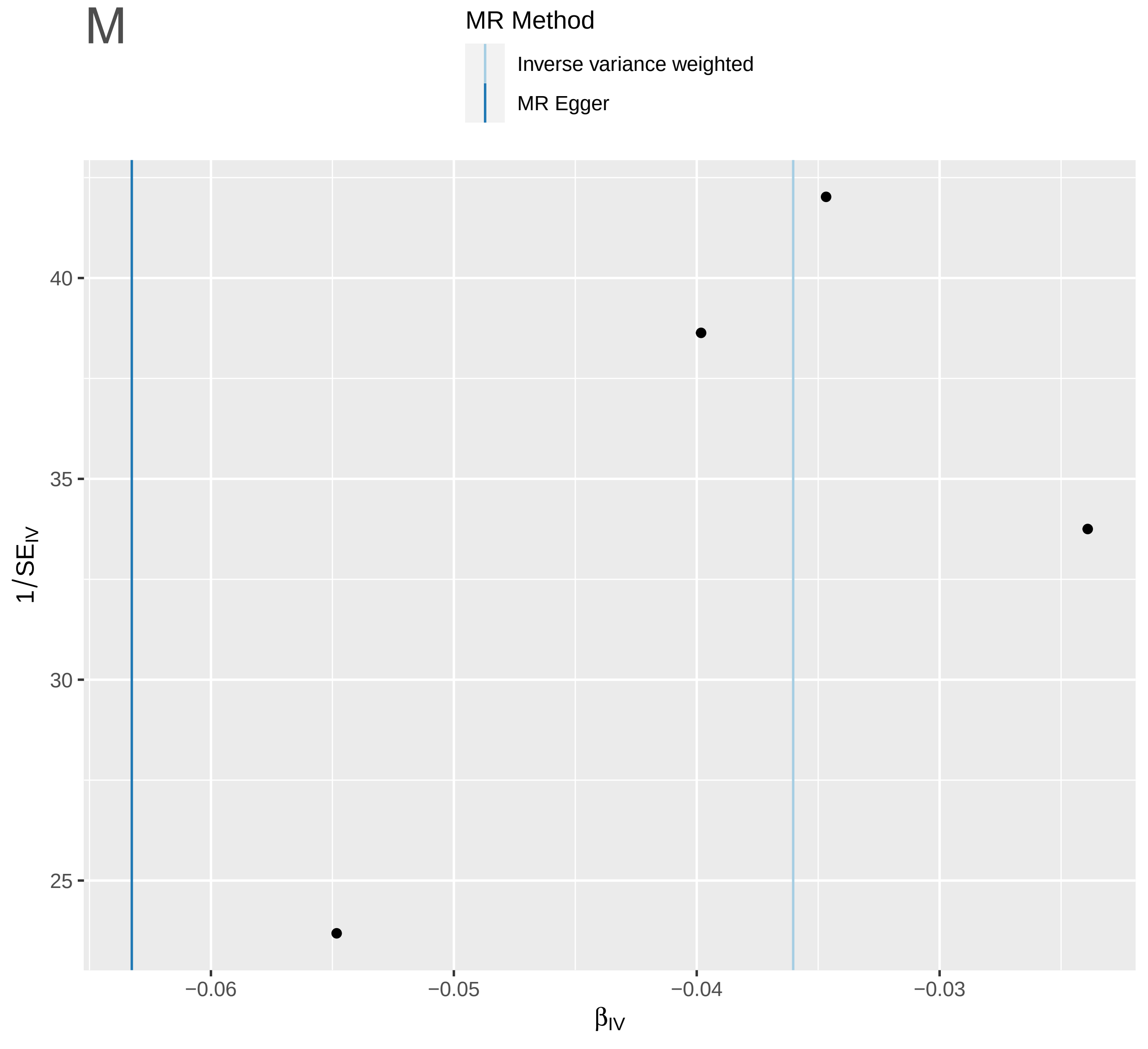
M
MR Method
Inverse variance weighted
MR Egger
40
35
30
25
1/SE_IV
−0.06
−0.05
−0.04
−0.03
β_IV

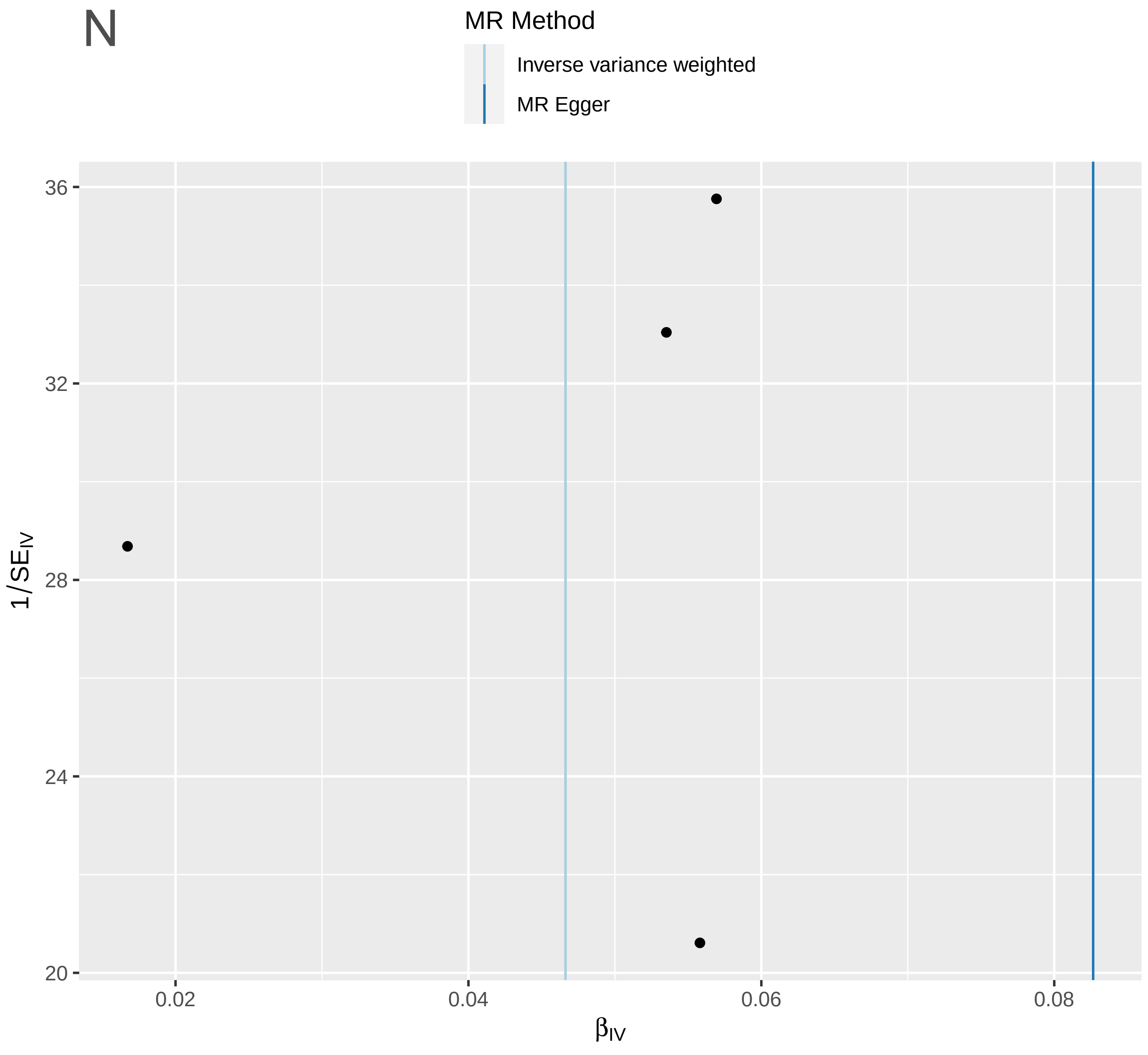
N
MR Method
Inverse variance weighted
MR Egger
36
32
28
24
20
$1/SE_{IV}$
0.02
0.04
0.06
0.08
β_{IV}

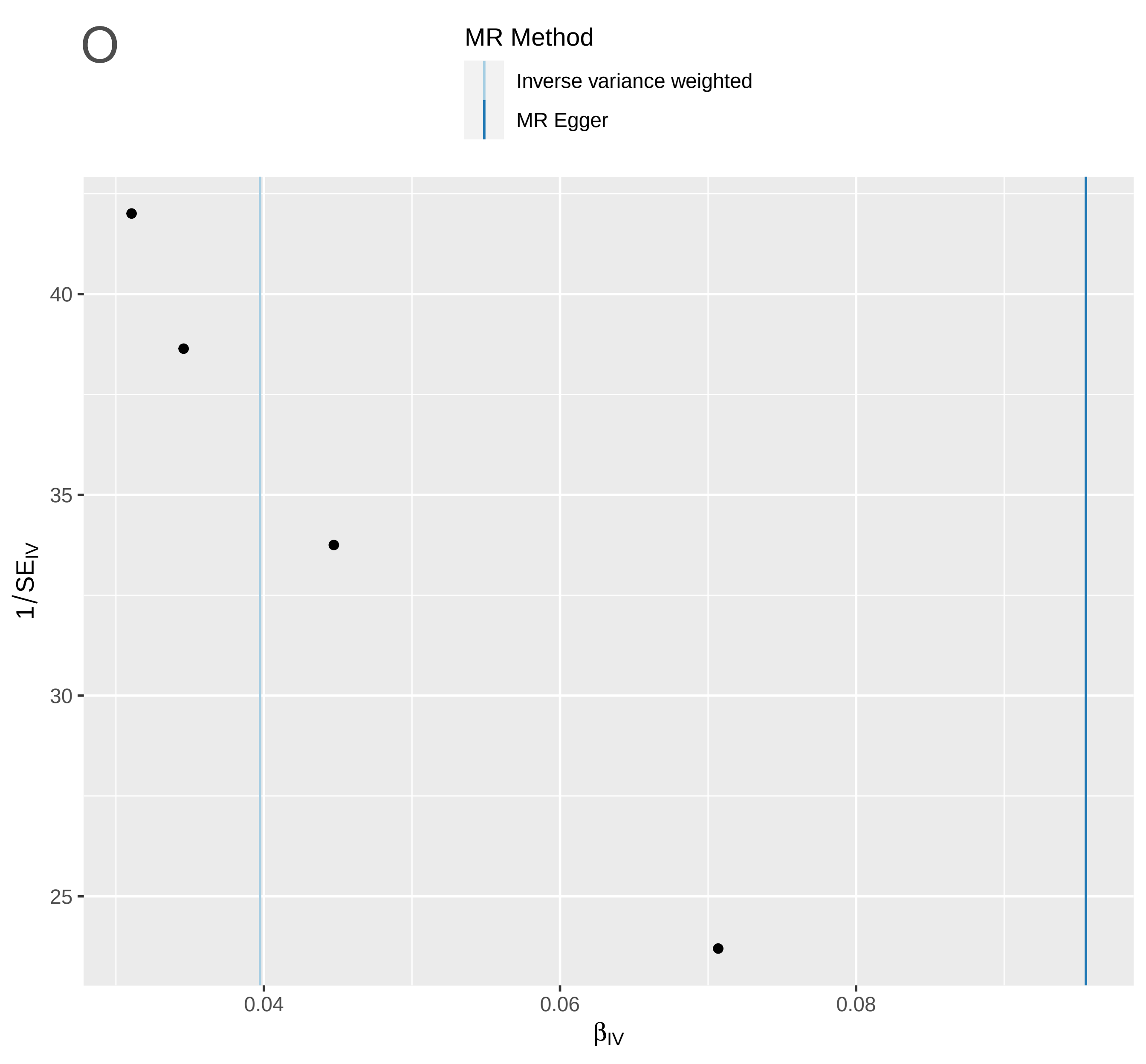

O
MR Method
Inverse variance weighted
MR Egger
40
35
30
25
1/SE_IV
0.04
0.06
0.08
β_IV

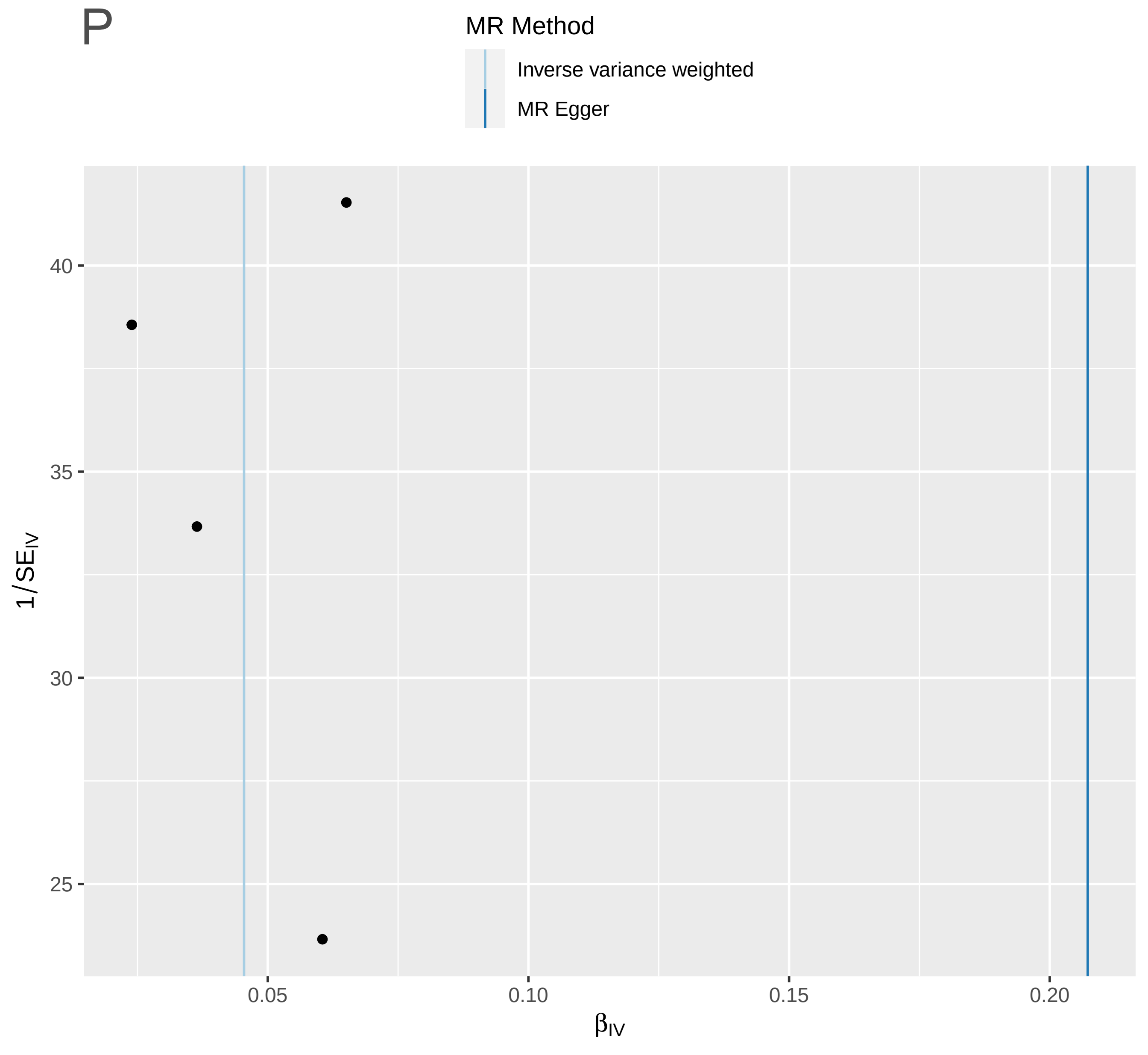
P
MR Method
Inverse variance weighted
MR Egger
40
35
30
25
0.05
0.10
0.15
0.20
1/SE_IV
β_IV

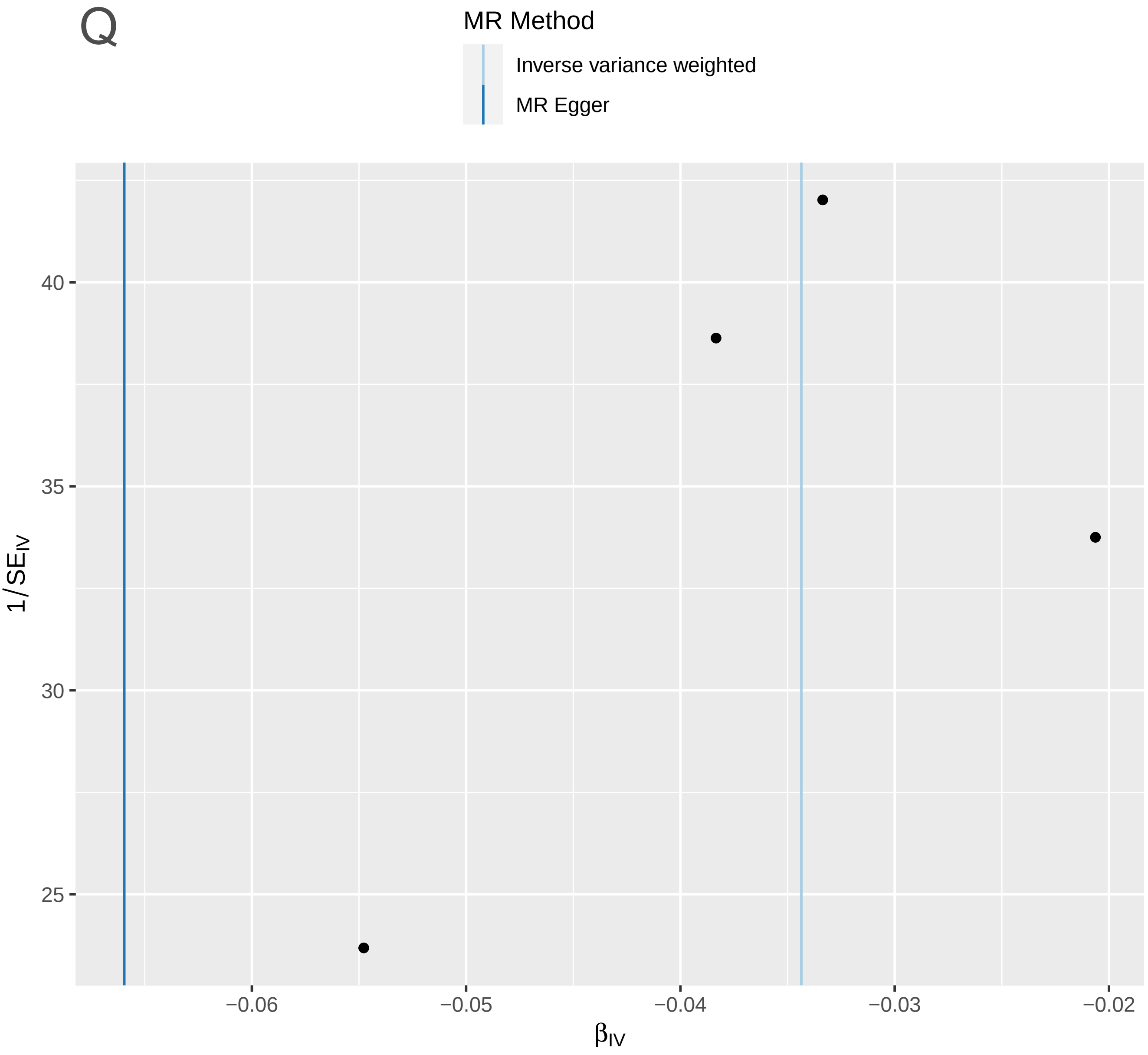
Q
MR Method
Inverse variance weighted
MR Egger
40
35
30
25
1/SE_IV
−0.06
−0.05
−0.04
−0.03
−0.02
β_IV

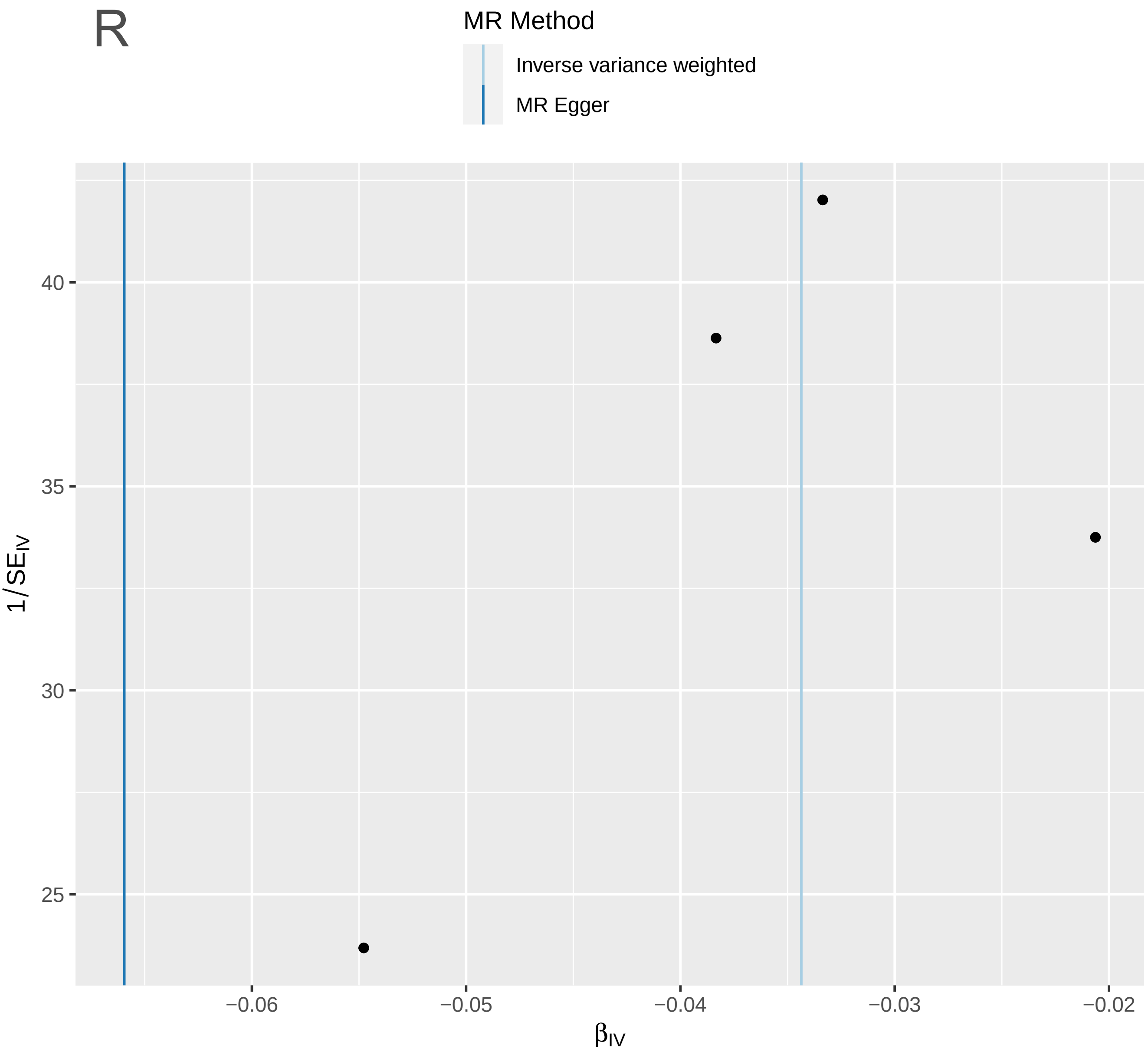
R
MR Method
Inverse variance weighted
MR Egger
40
35
30
25
1/SE_IV
−0.06
−0.05
−0.04
−0.03
−0.02
β_IV

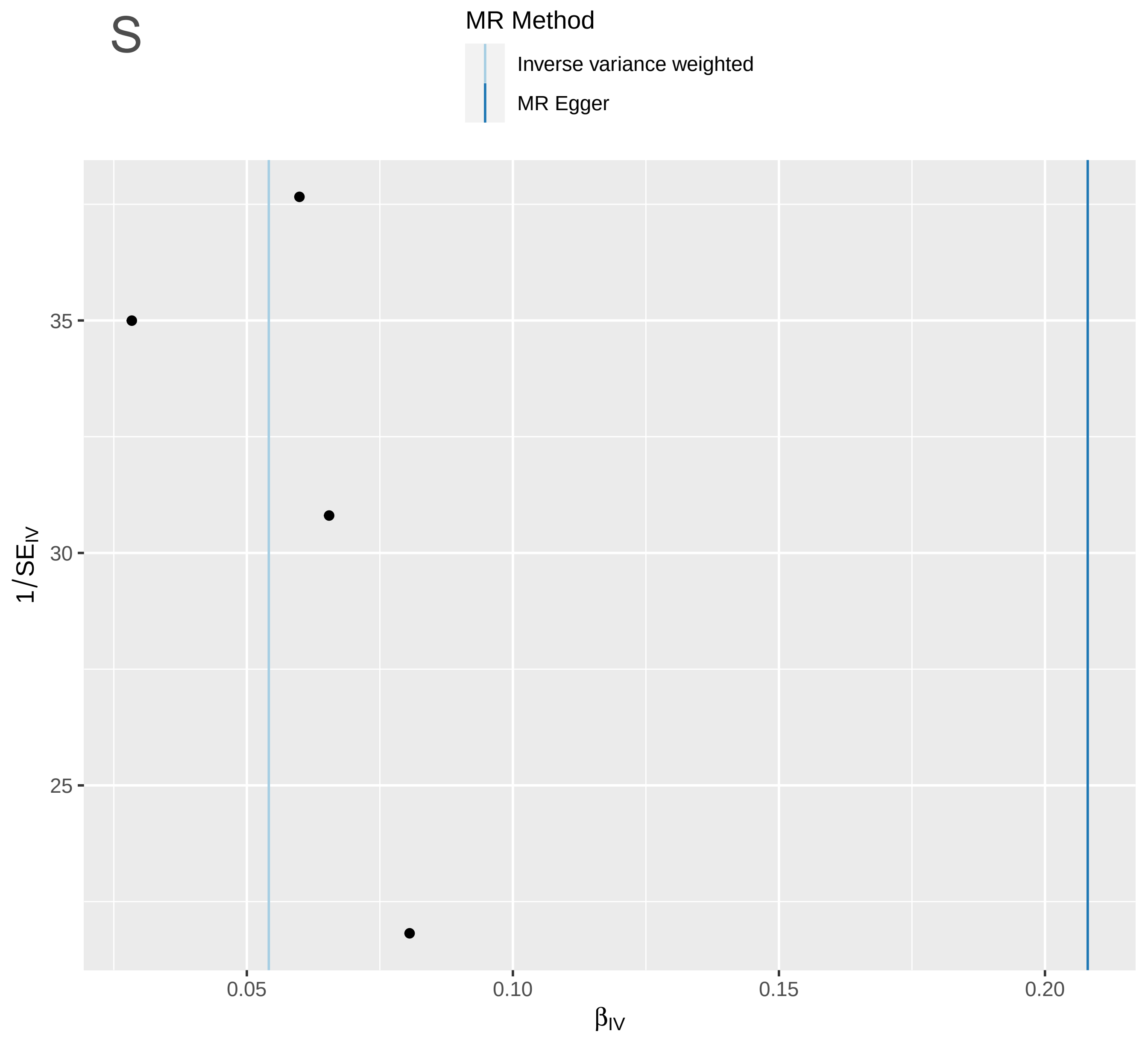
S
MR Method
Inverse variance weighted
MR Egger
35
30
25
$1/SE_{IV}$
0.05
0.10
0.15
0.20
β_{IV}

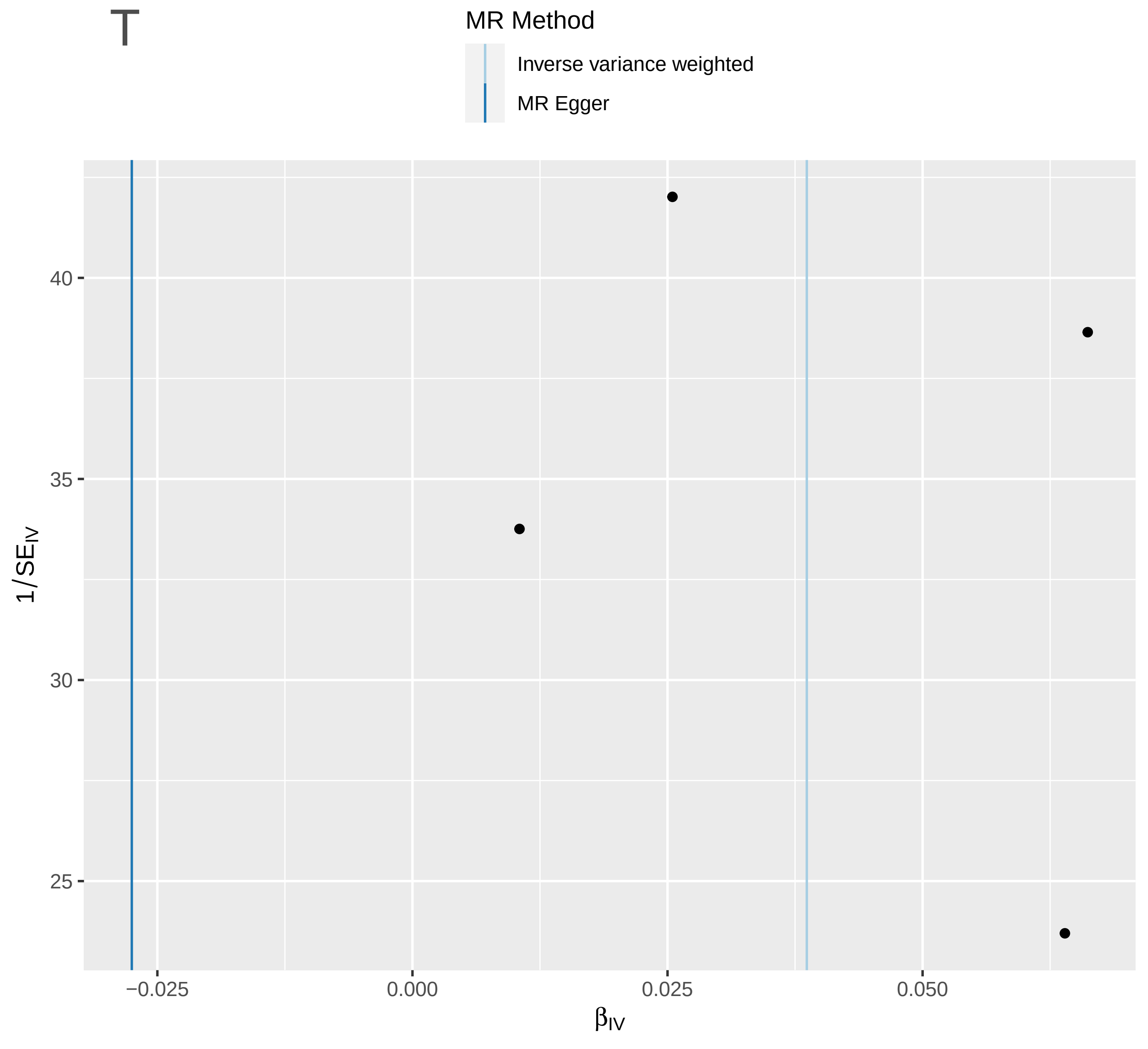
T
MR Method
Inverse variance weighted
MR Egger
40
35
30
25
1/SE_IV
−0.025
0.000
0.025
0.050
β_IV

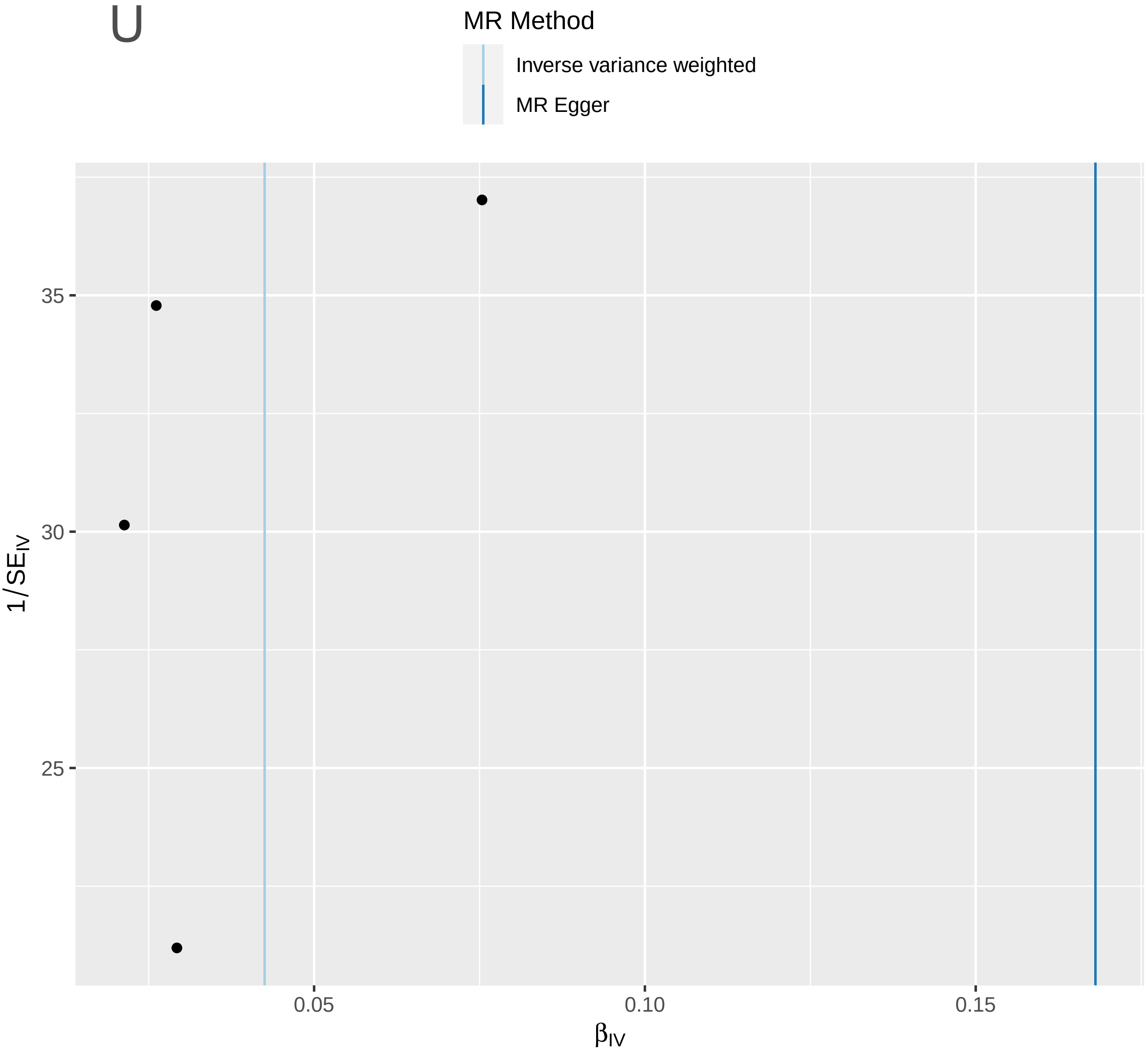
U
MR Method
Inverse variance weighted
MR Egger
35
30
25
0.05
0.10
0.15
$1/SE_{IV}$
β_{IV}

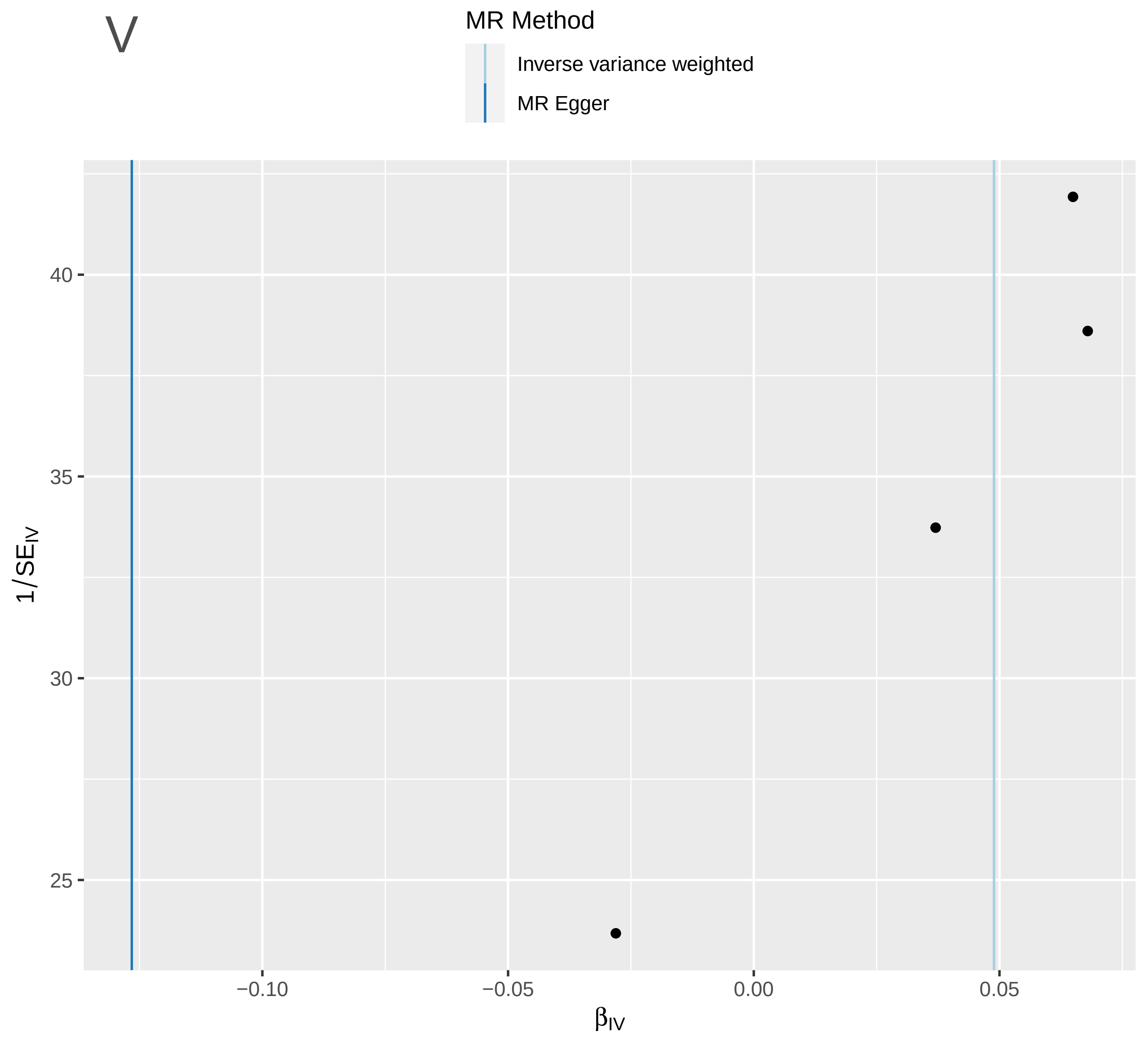
V
MR Method
Inverse variance weighted
MR Egger
40
35
30
25
1/SE_IV
−0.10
−0.05
0.00
0.05
β_IV

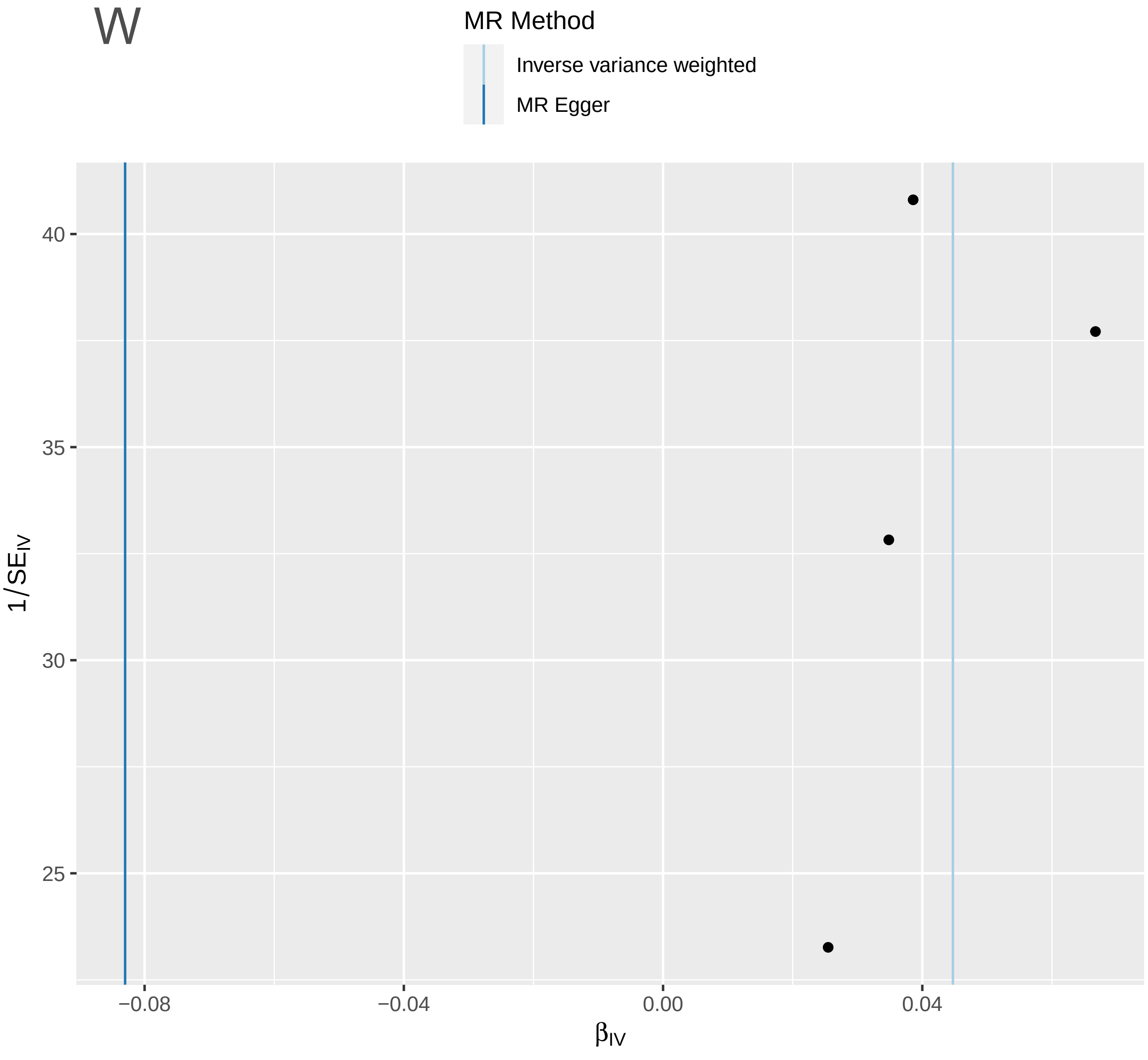
W
MR Method
Inverse variance weighted
MR Egger
40
35
30
25
1/SE_{IV}
−0.08
−0.04
0.00
0.04
β_{IV}

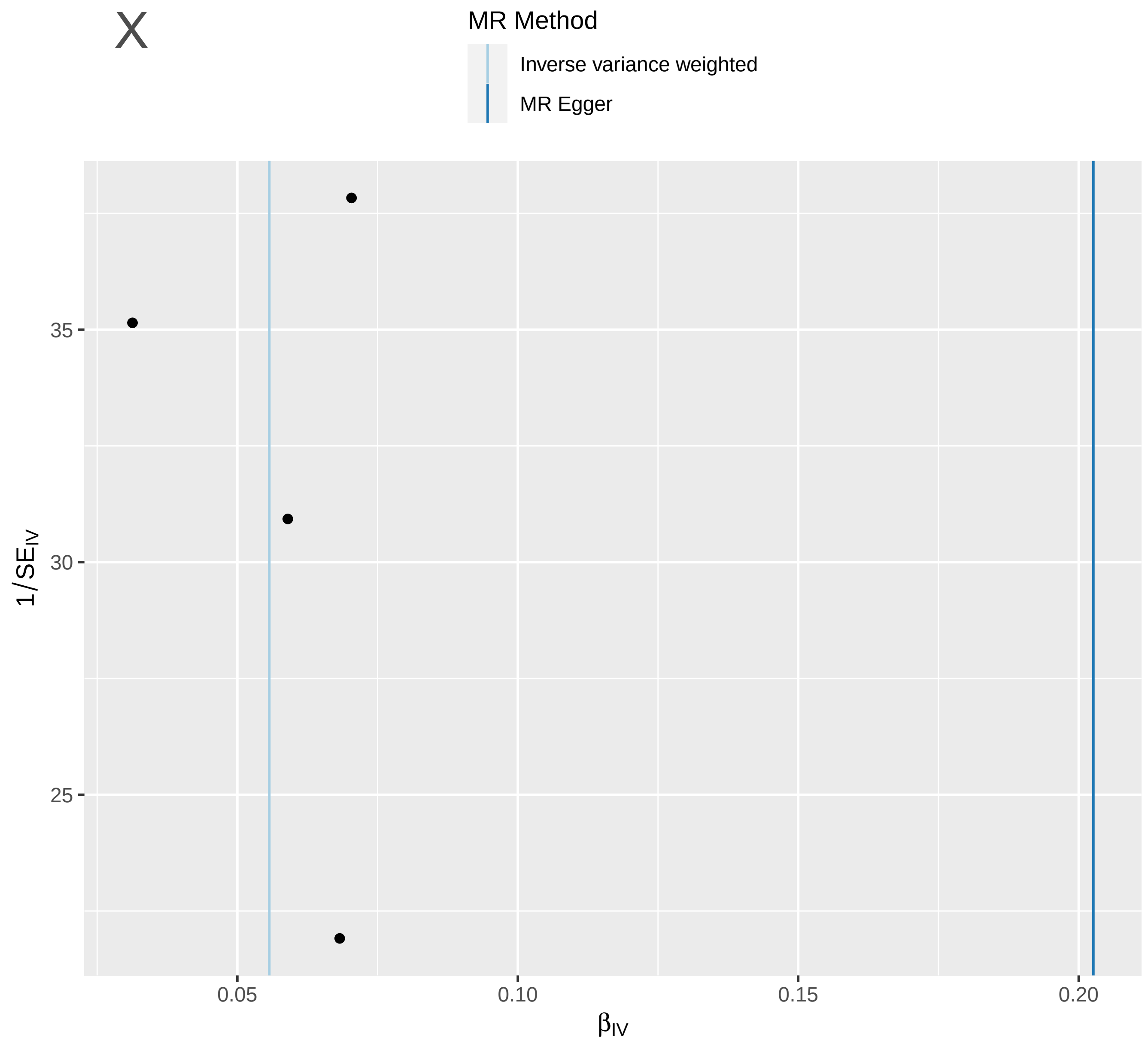
X
MR Method
Inverse variance weighted
MR Egger
35
30
25
0.05
0.10
0.15
0.20
1/SE_IV
β_IV

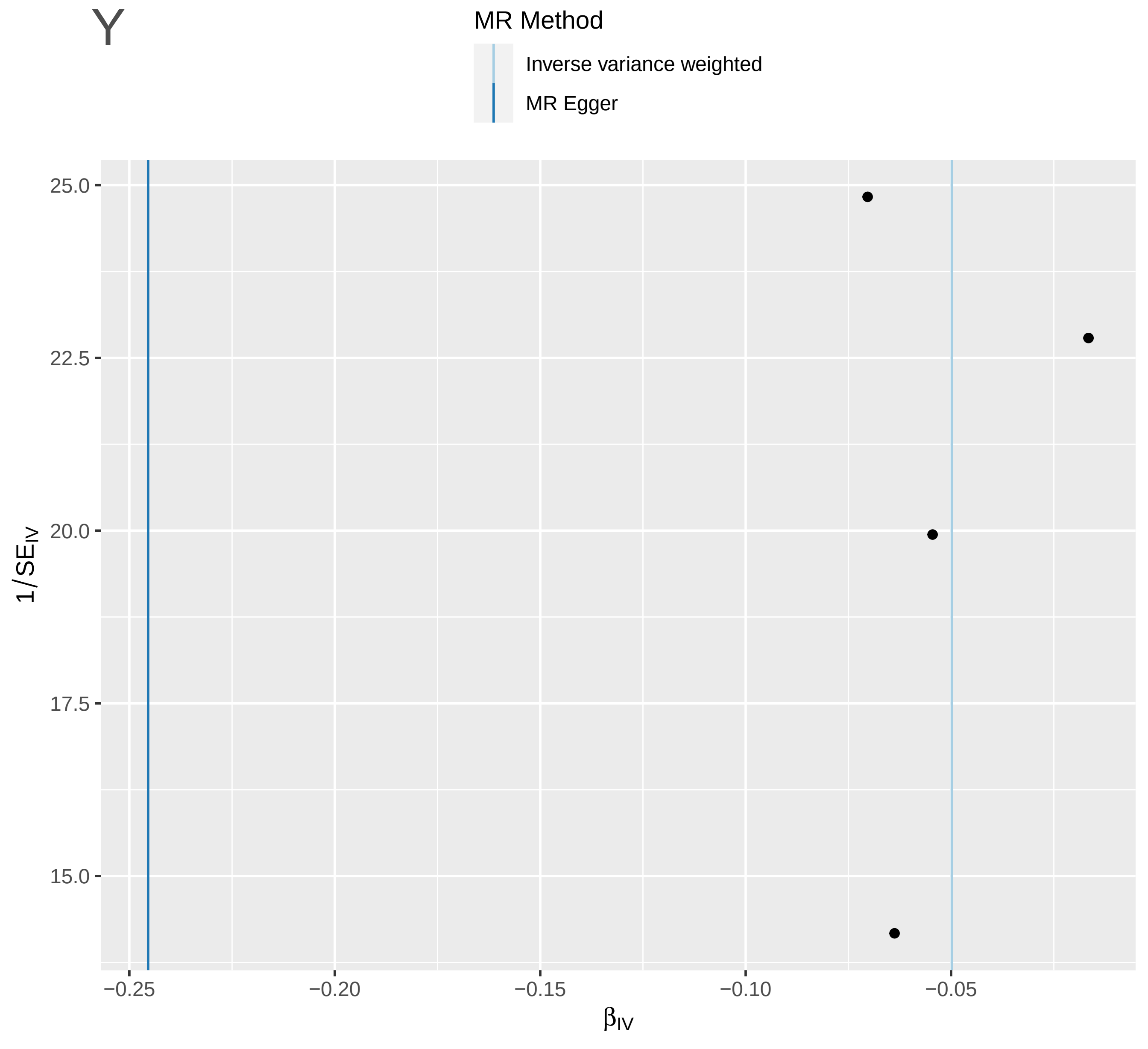

Y
MR Method
Inverse variance weighted
MR Egger
25.0
22.5
20.0
17.5
15.0
1/SE_IV
−0.25
−0.20
−0.15
−0.10
−0.05
β_IV

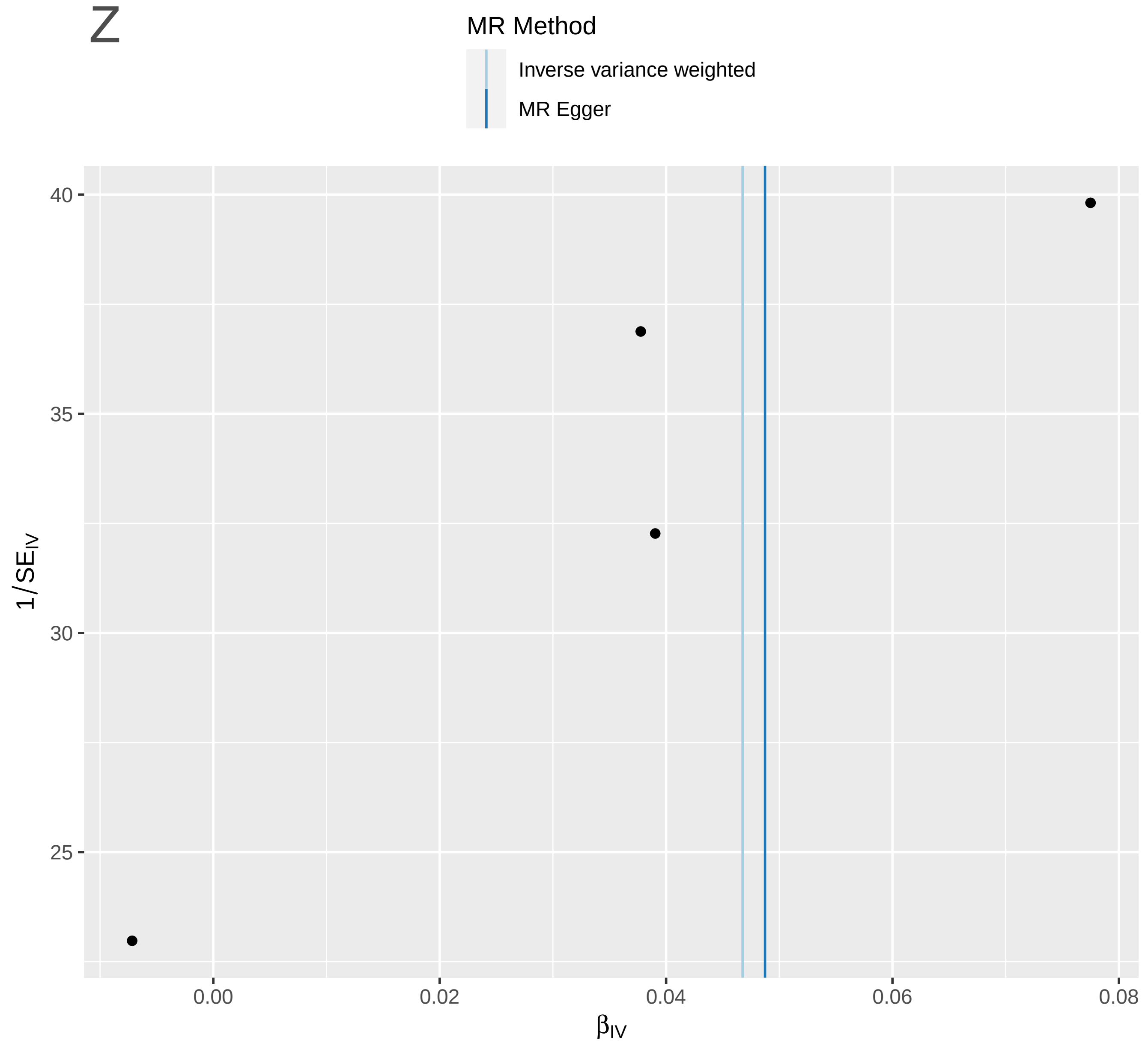

Z
MR Method
Inverse variance weighted
MR Egger
40
35
30
25
0.00
0.02
0.04
0.06
0.08
$1/SE_{IV}$
β_{IV}

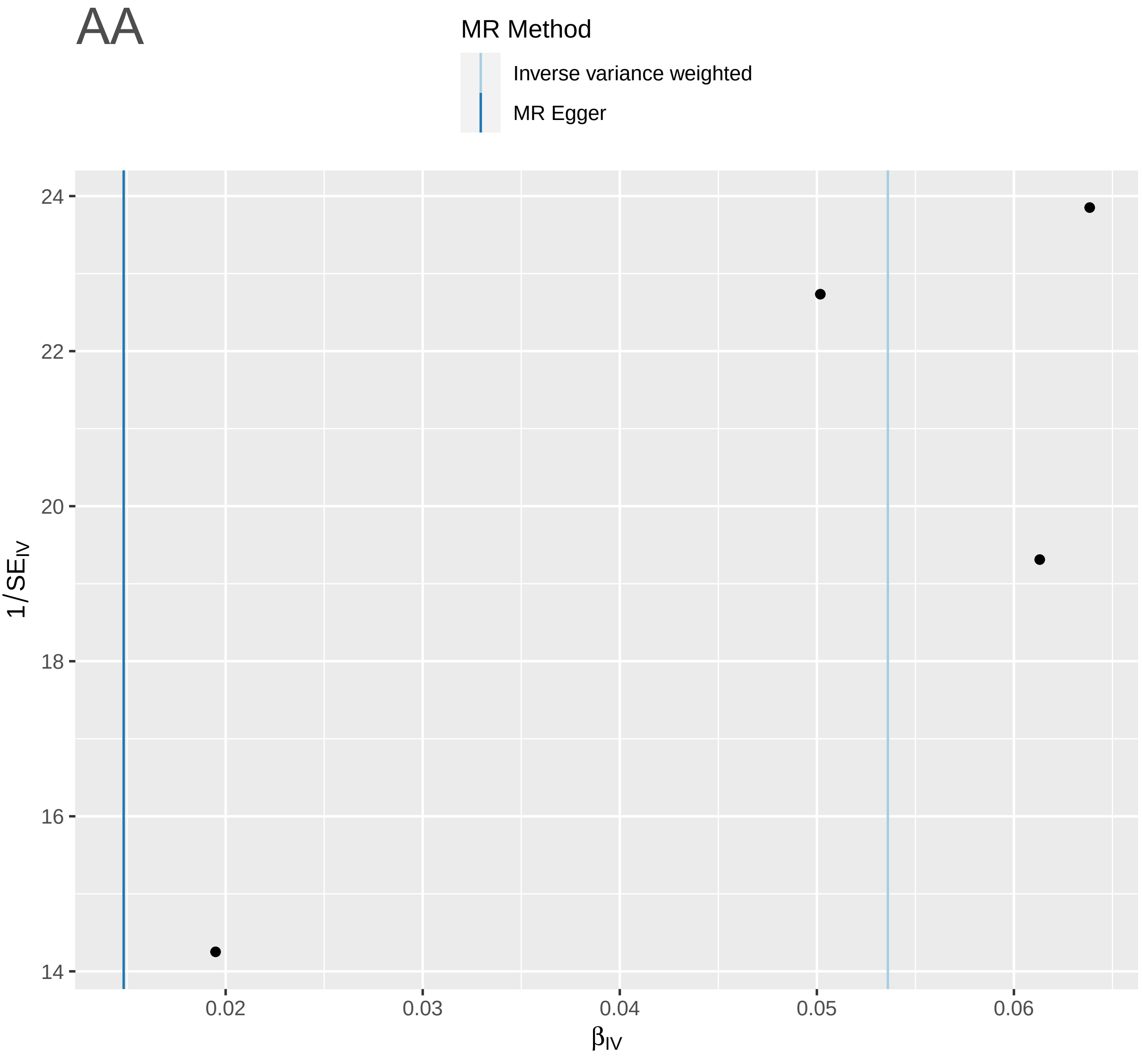

AA
MR Method
Inverse variance weighted
MR Egger
24
22
20
18
16
14
$1/SE_{IV}$
0.02
0.03
0.04
0.05
0.06
β_{IV}

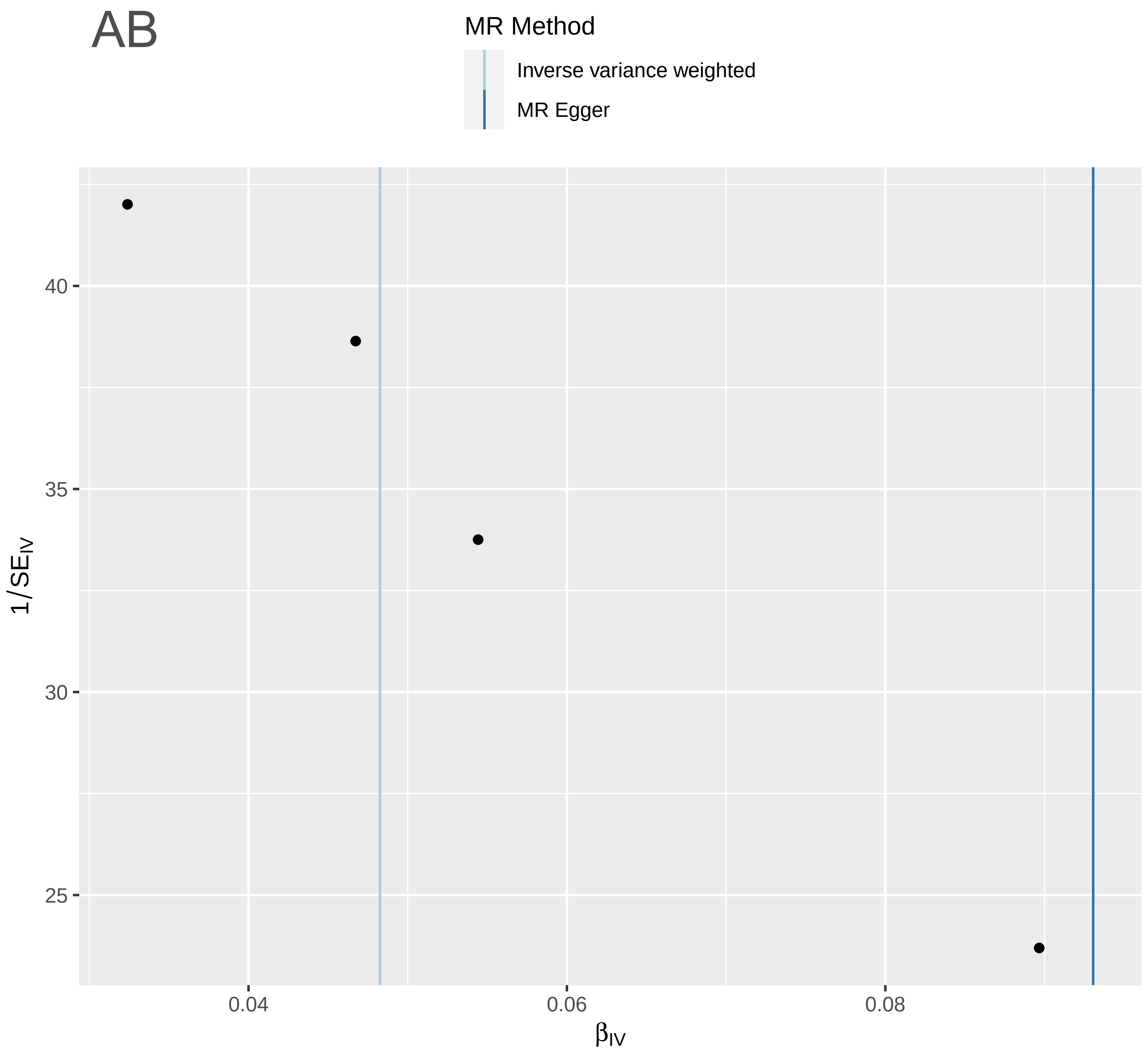

AB
MR Method
Inverse variance weighted
MR Egger
40
35
30
25
1/SE_IV
0.04
0.06
0.08
β_IV

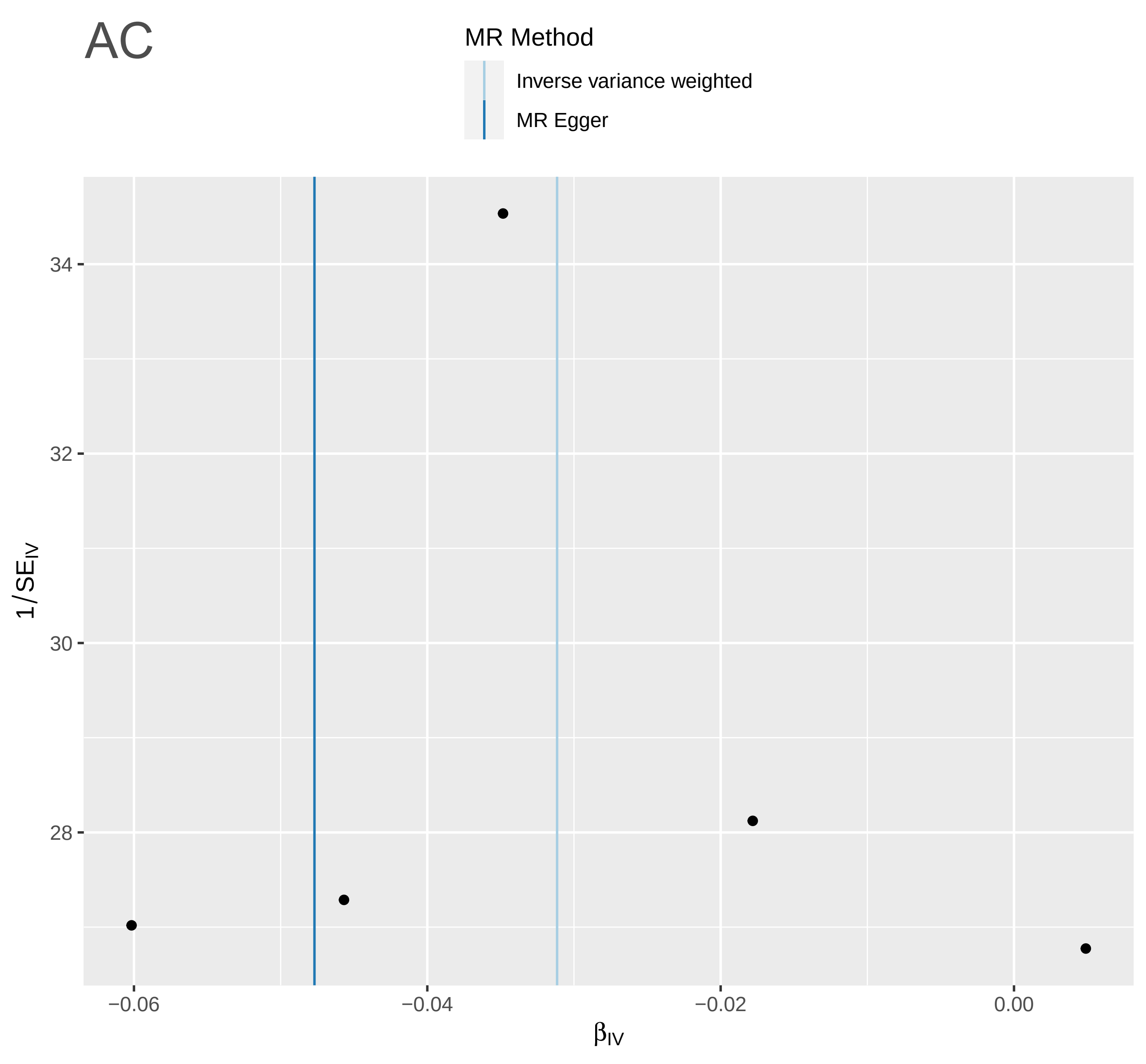
AC
MR Method
Inverse variance weighted
MR Egger
34
32
30
28
1/SE_IV
−0.06
−0.04
−0.02
0.00
β_IV

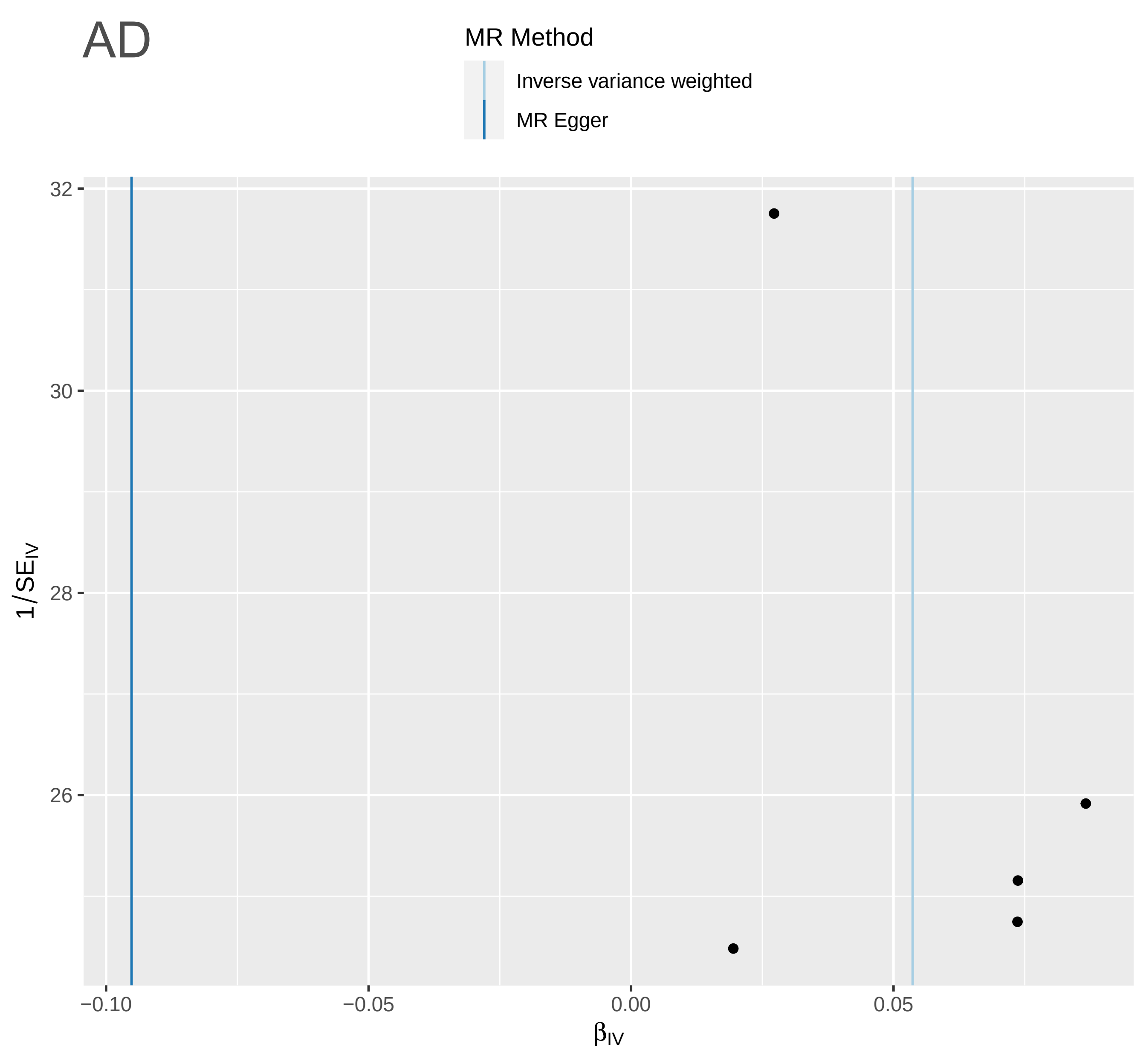

AD
MR Method
Inverse variance weighted
MR Egger
32
30
28
26
1/SE$_{IV}$
−0.10
−0.05
0.00
0.05
β$_{IV}$

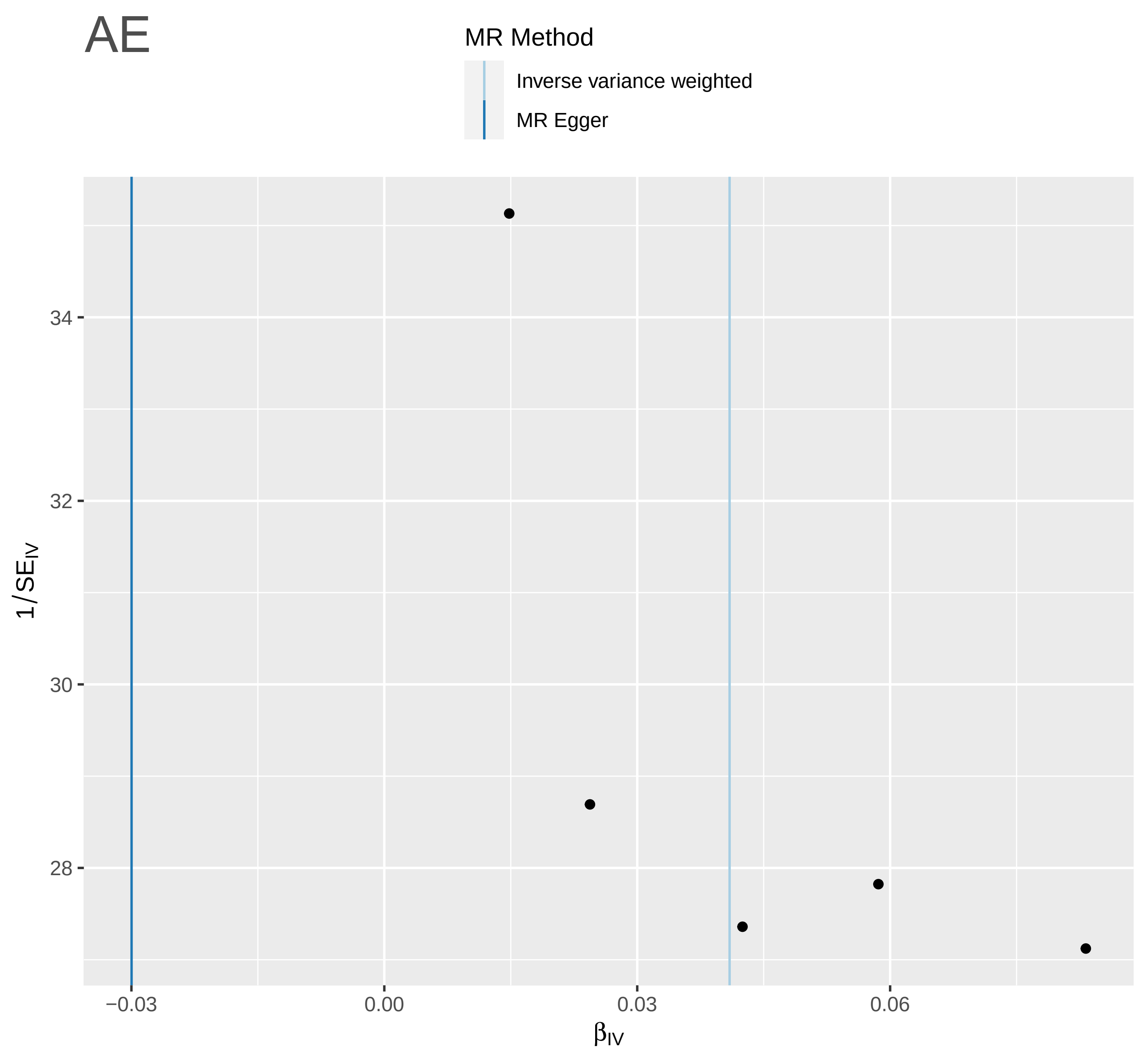
AE
MR Method
Inverse variance weighted
MR Egger
34
32
30
28
1/SE$_{IV}$
−0.03
0.00
0.03
0.06
β$_{IV}$

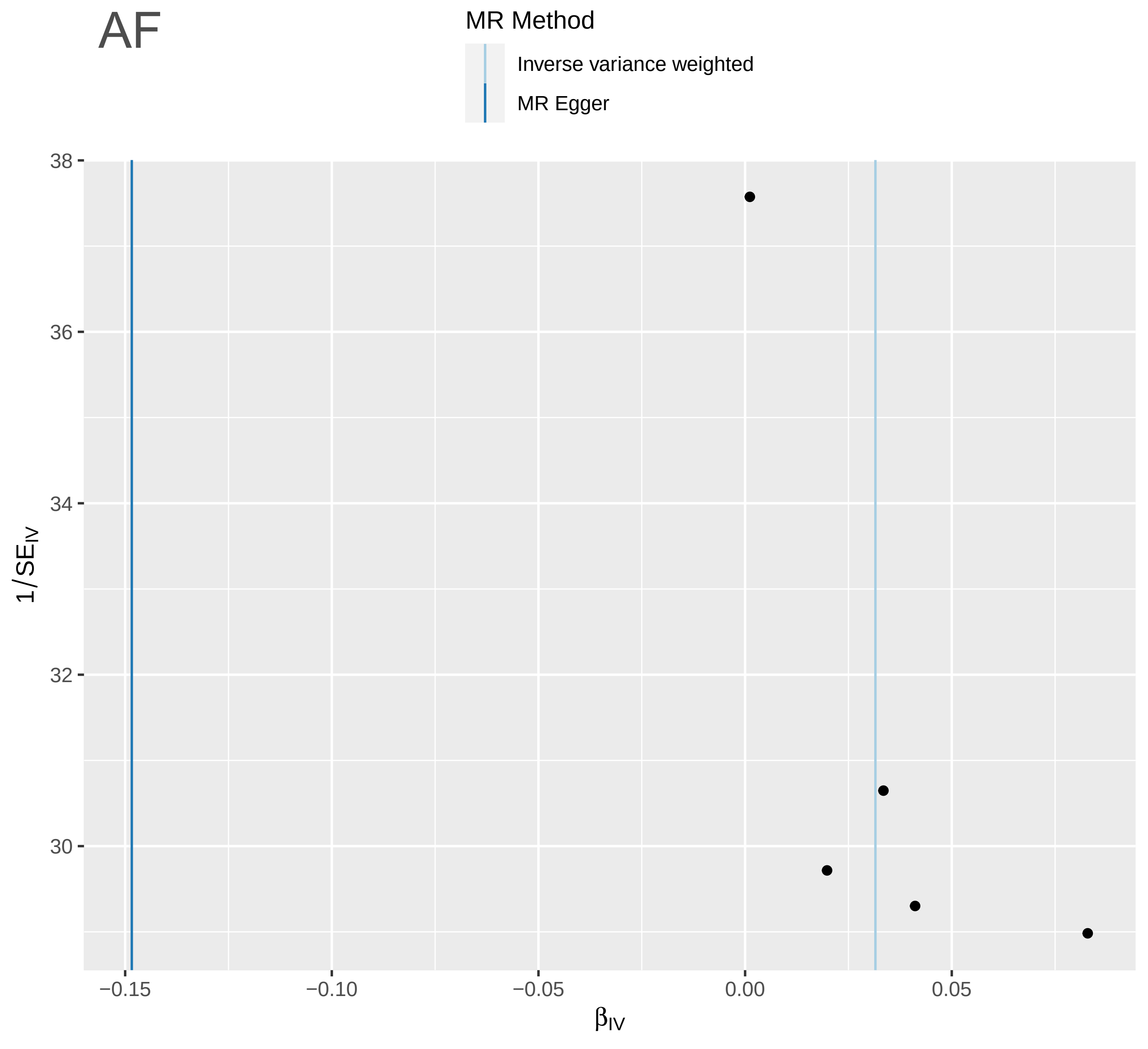

AF
MR Method
Inverse variance weighted
MR Egger
38
36
34
32
30
1/SE_IV
−0.15
−0.10
−0.05
0.00
0.05
β_IV

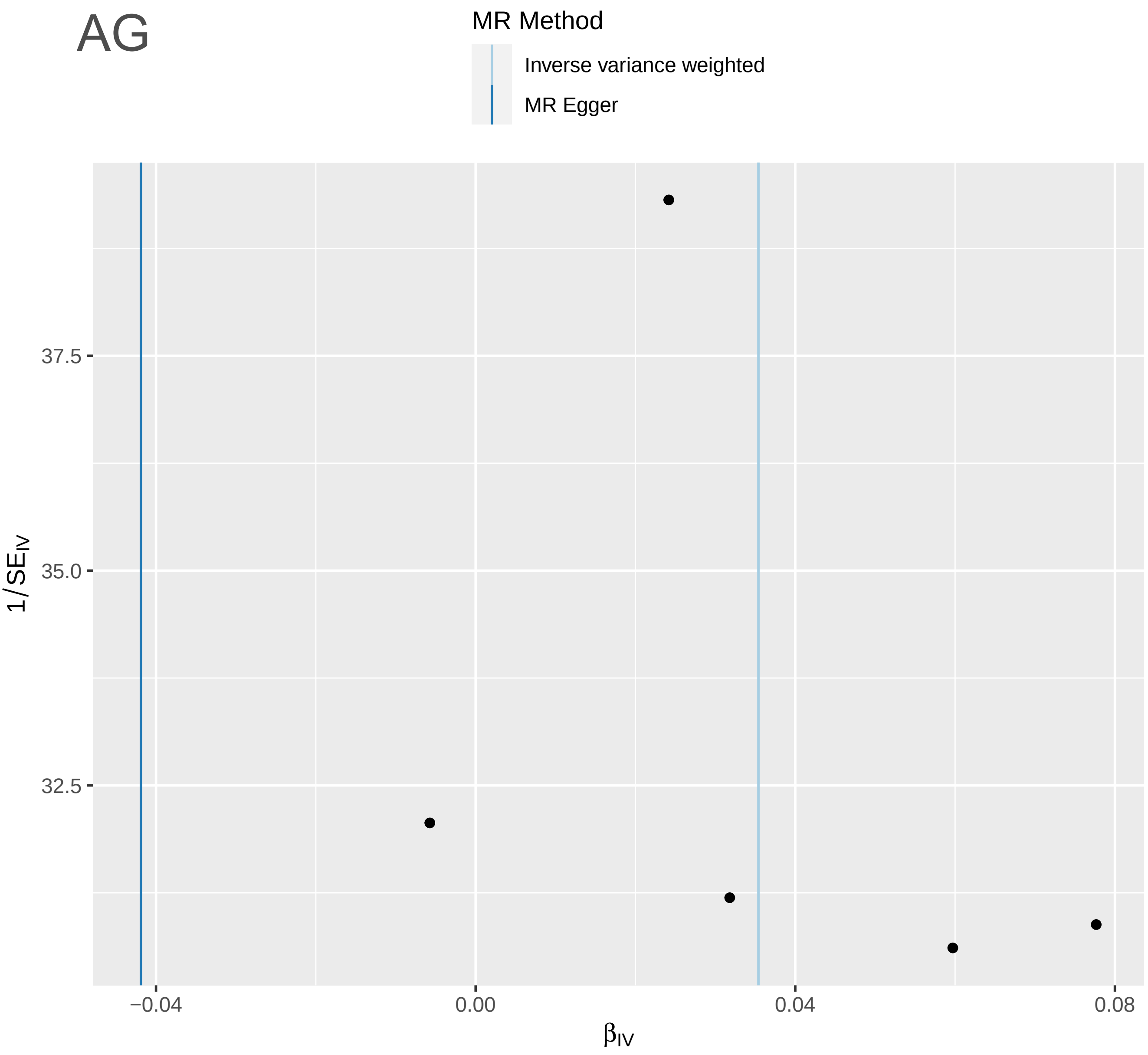

AG
MR Method
Inverse variance weighted
MR Egger
37.5
35.0
32.5
1/SE$_{IV}$
−0.04
0.00
0.04
0.08
β$_{IV}$

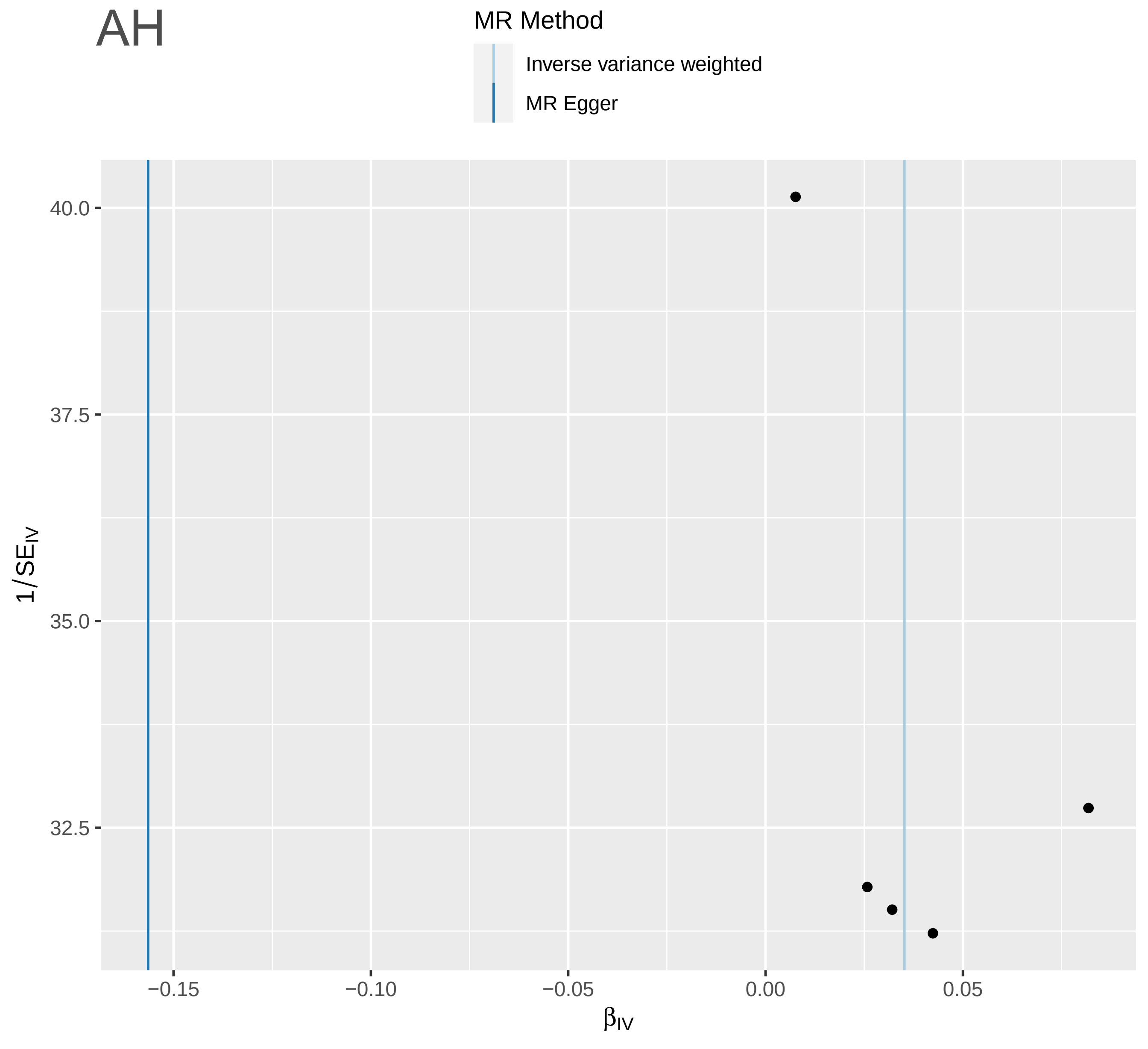
AH
MR Method
Inverse variance weighted
MR Egger
40.0
37.5
35.0
32.5
1/SE_IV
−0.15
−0.10
−0.05
0.00
0.05
β_IV

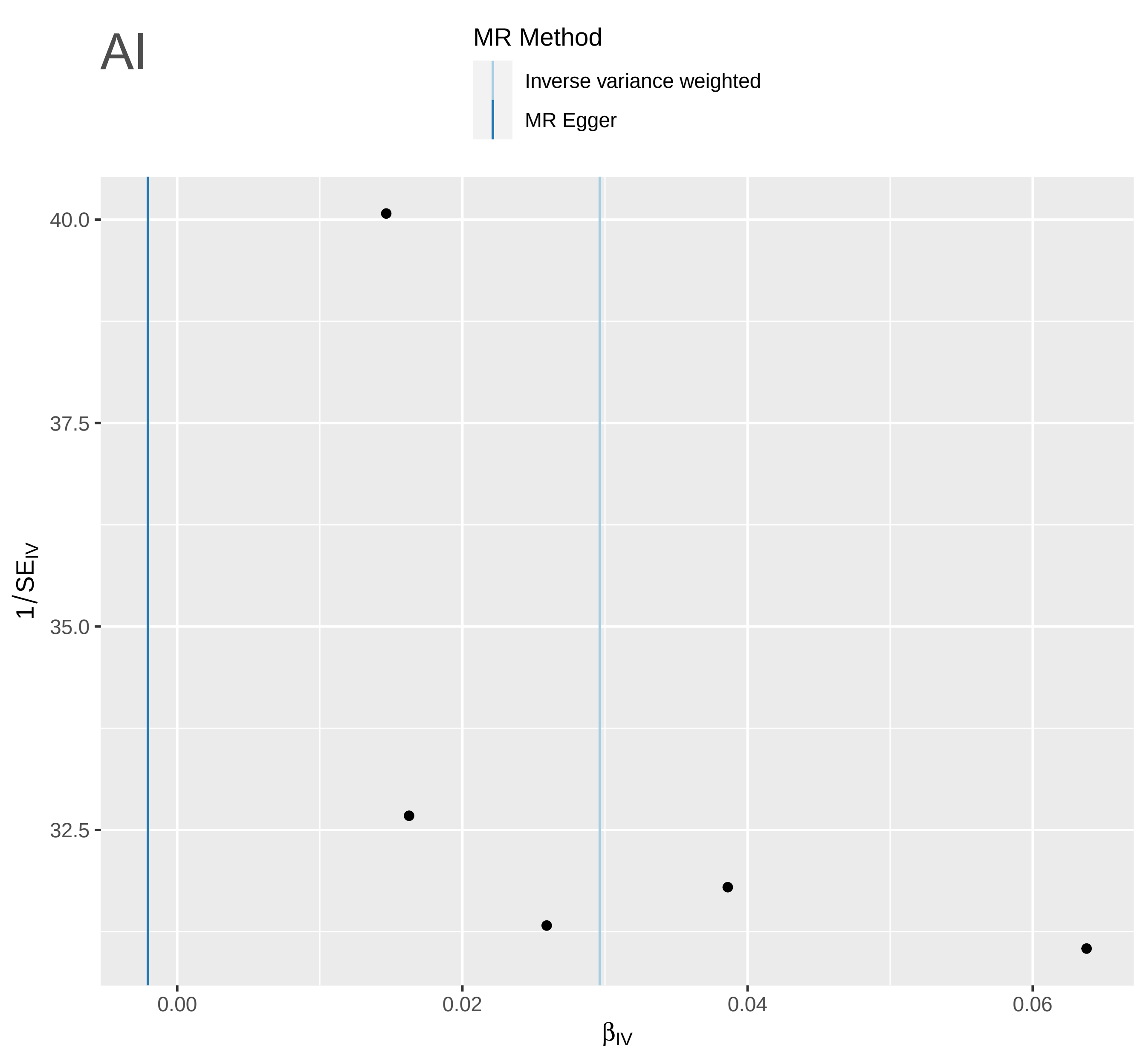

AI
MR Method
Inverse variance weighted
MR Egger
40.0
37.5
35.0
32.5
0.00
0.02
0.04
0.06
1/SE_IV
β_IV

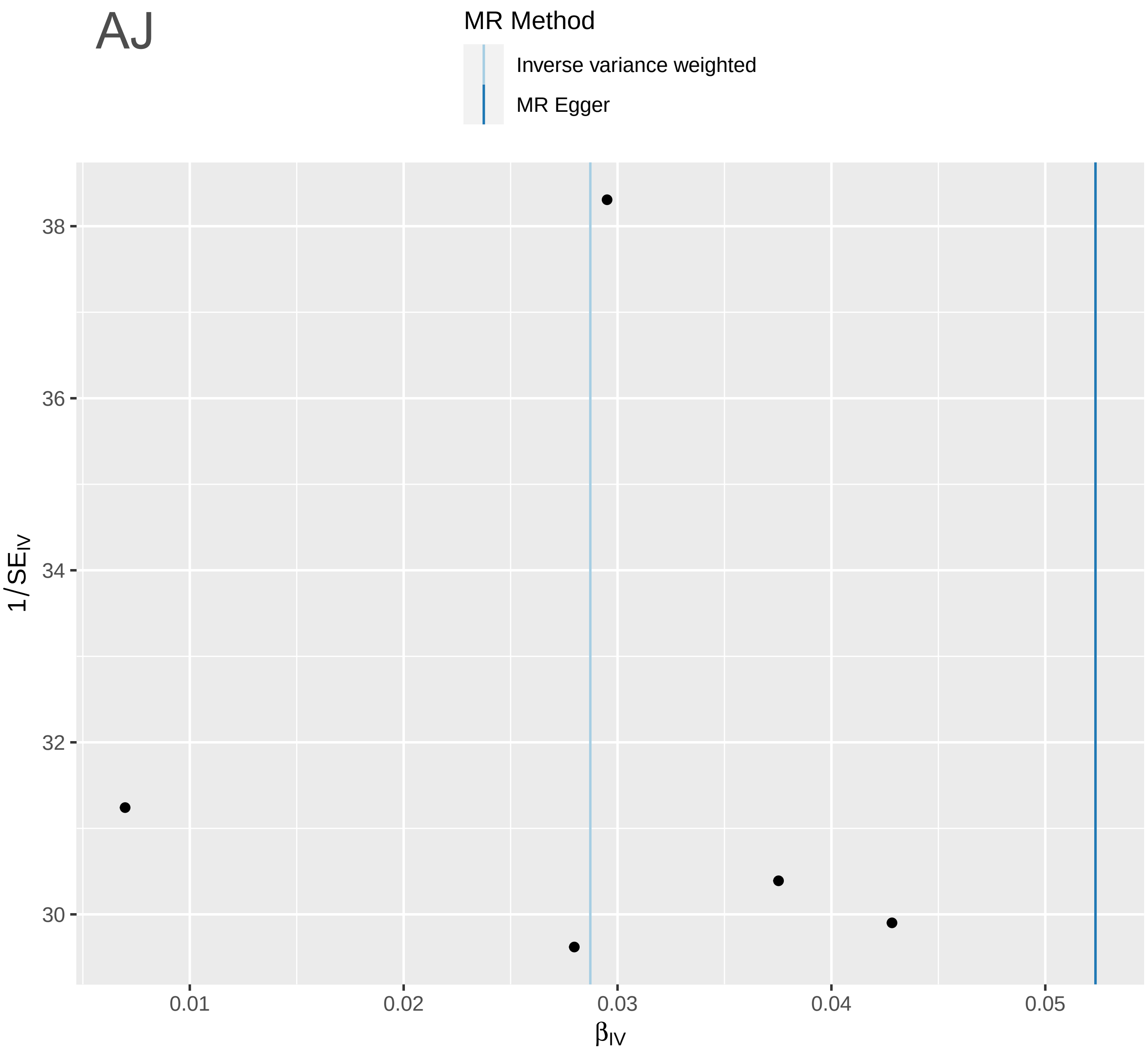
AJ
MR Method
Inverse variance weighted
MR Egger
38
36
34
32
30
1/SE$_{IV}$
0.01
0.02
0.03
0.04
0.05
β_{IV}

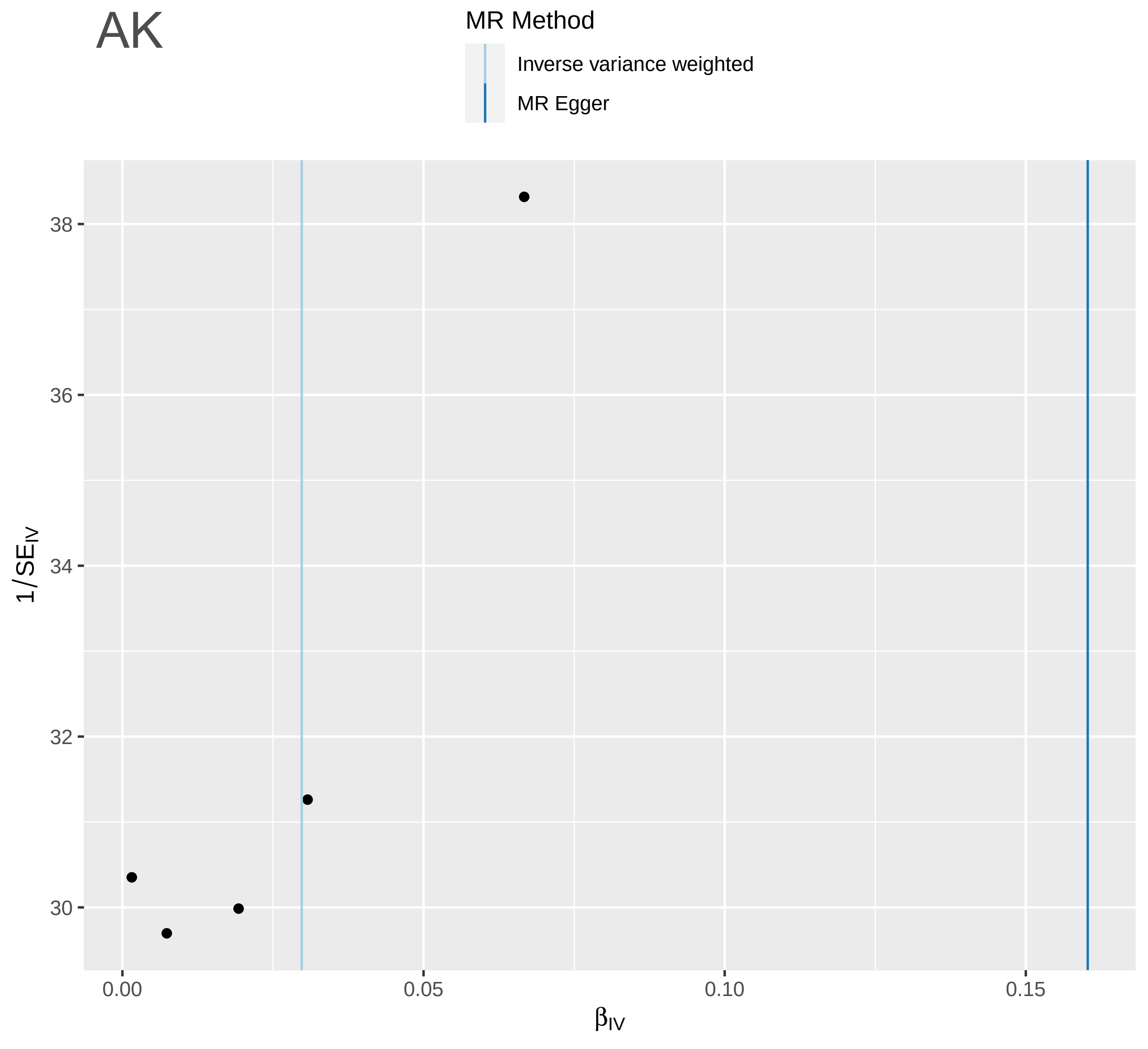
AK
MR Method
Inverse variance weighted
MR Egger
38
36
34
32
30
1/SE$_{IV}$
0.00
0.05
0.10
0.15
β_{IV}

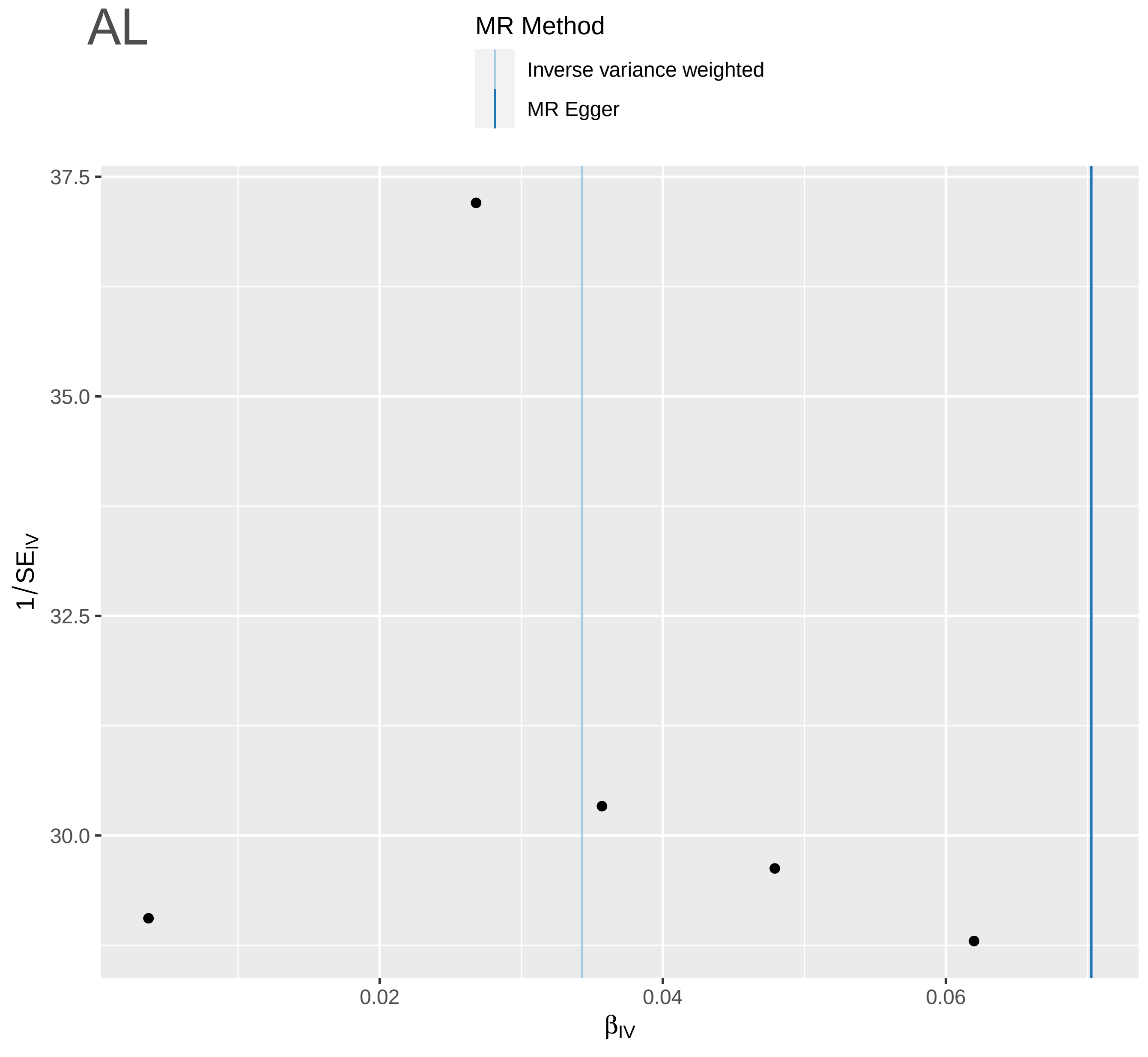
AL
MR Method
Inverse variance weighted
MR Egger
37.5
35.0
32.5
30.0
1/SE_{IV}
0.02
0.04
0.06
β_{IV}

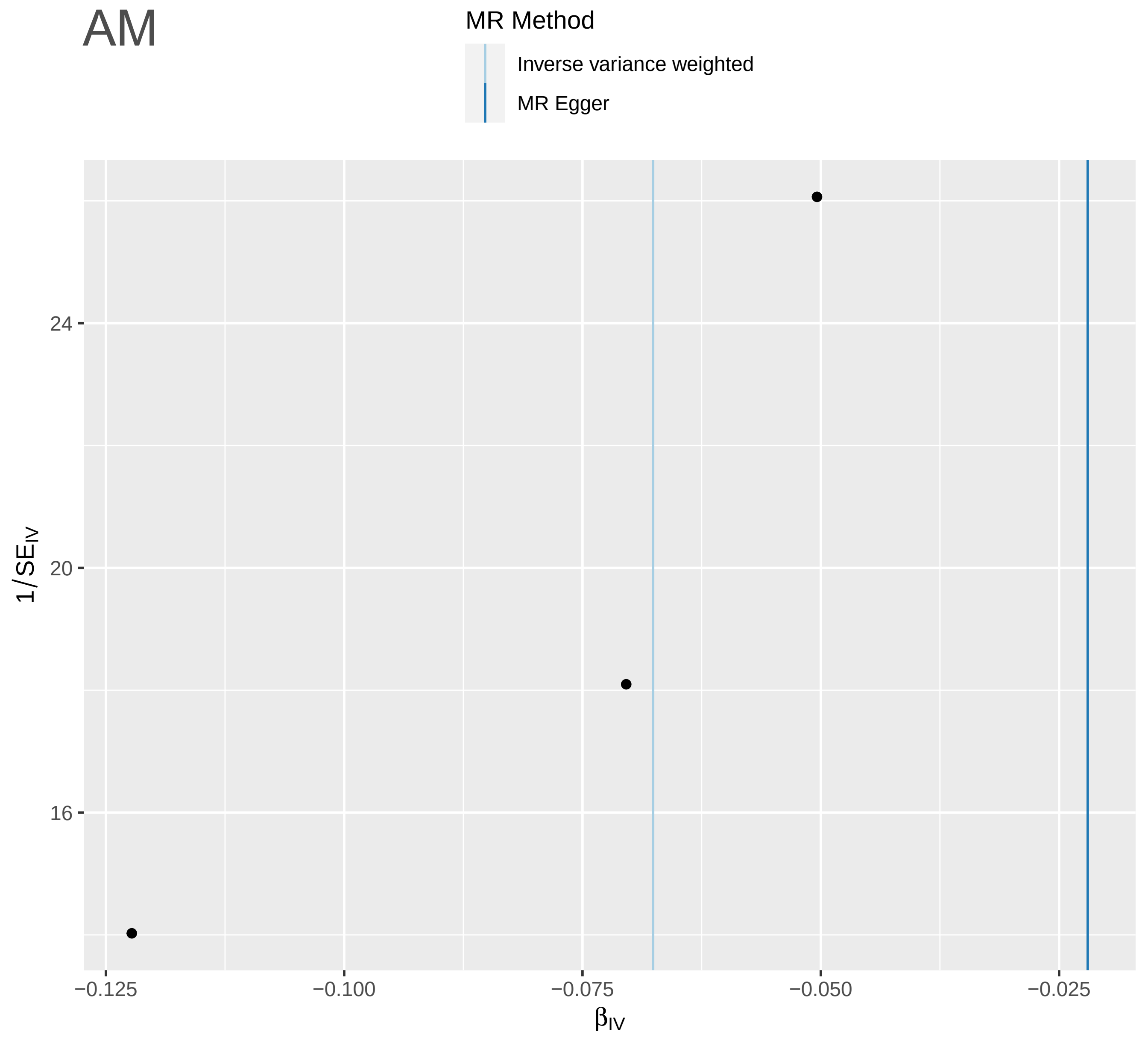
AM
MR Method
Inverse variance weighted
MR Egger
24
20
16
1/SE_IV
−0.125
−0.100
−0.075
−0.050
−0.025
β_IV

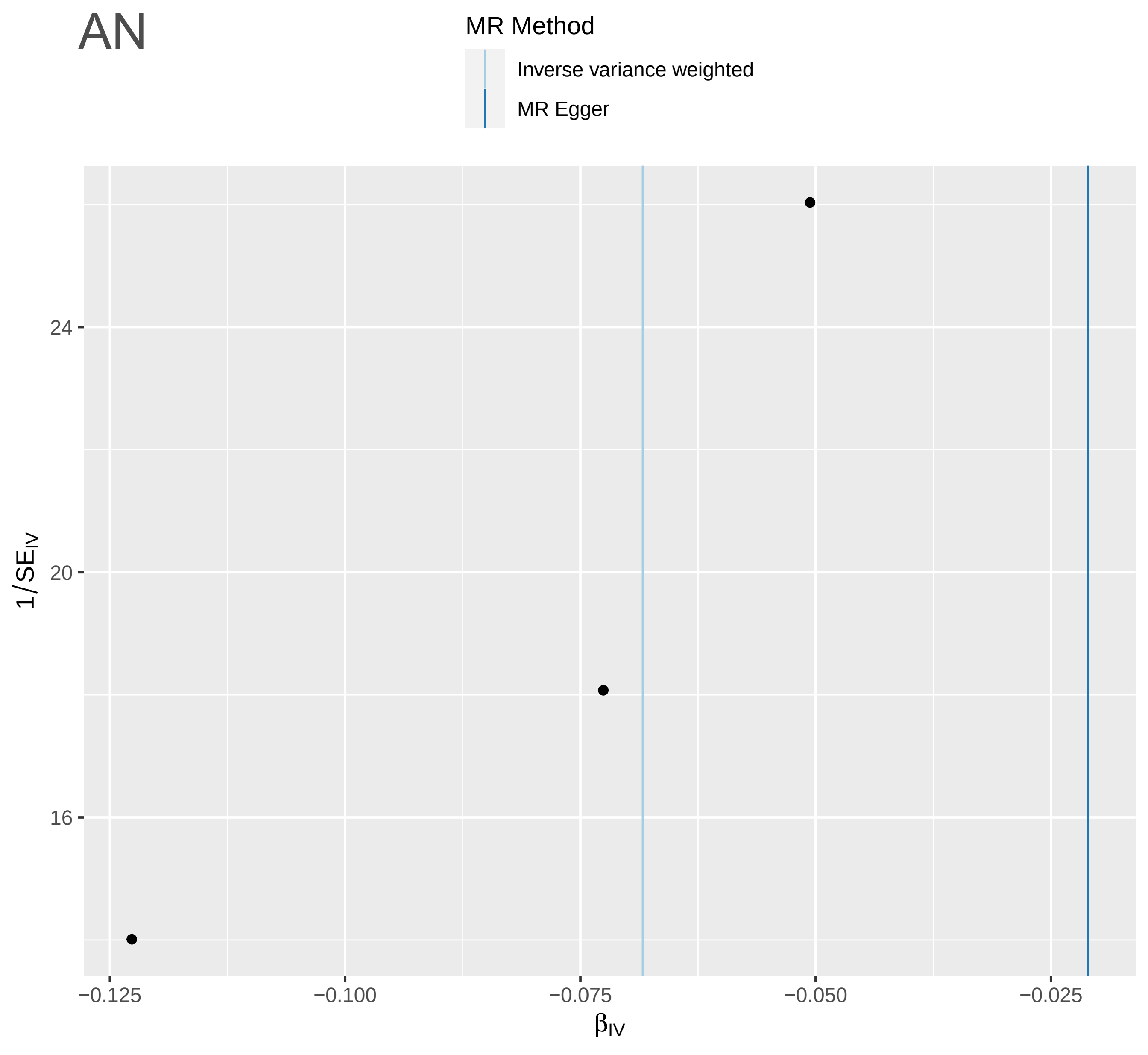
AN
MR Method
Inverse variance weighted
MR Egger
24
20
16
1/SE$_{IV}$
−0.125
−0.100
−0.075
−0.050
−0.025
β$_{IV}$

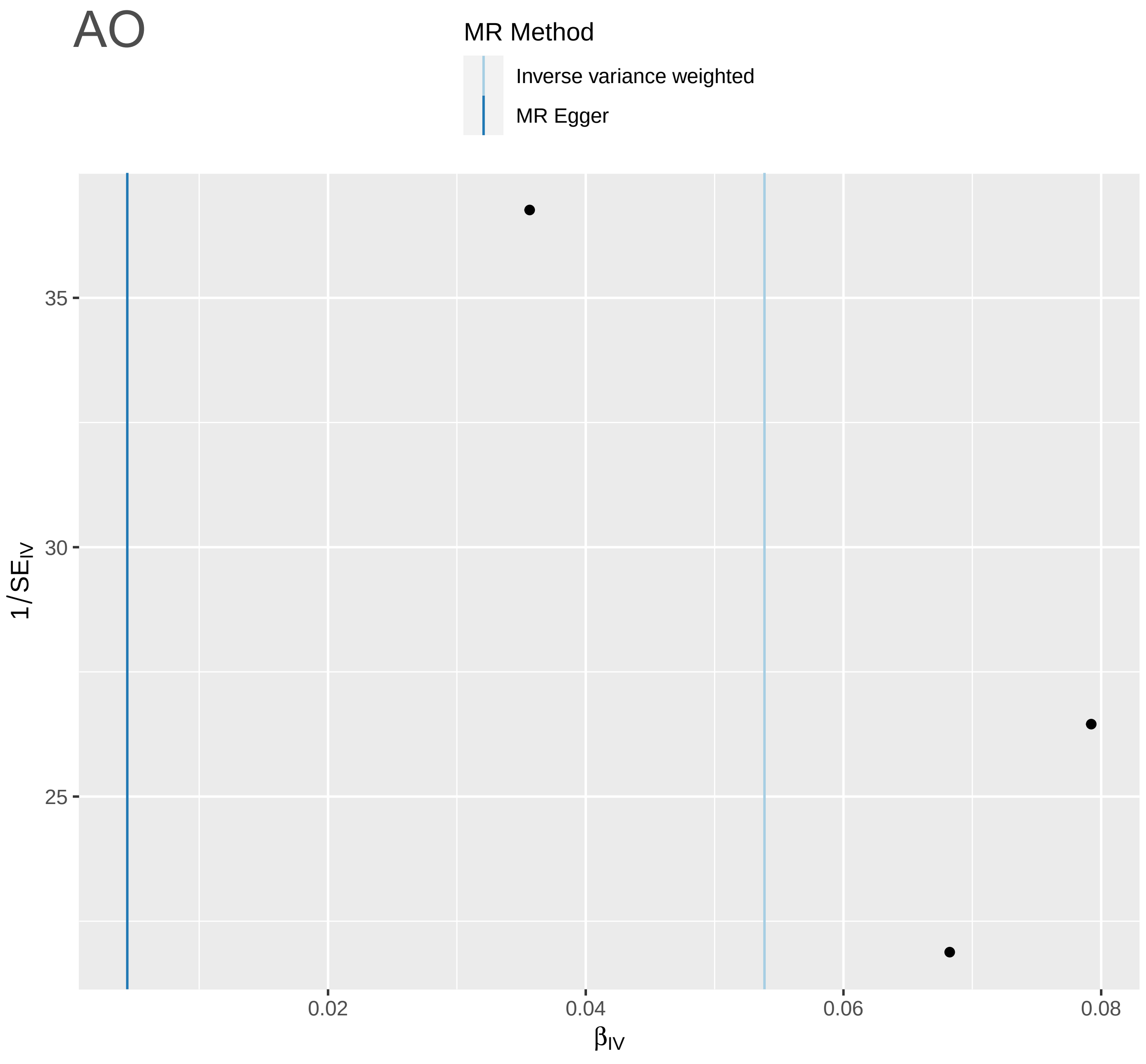
AO
MR Method
Inverse variance weighted
MR Egger
35
30
25
1/SE$_{IV}$
0.02
0.04
0.06
0.08
β_{IV}

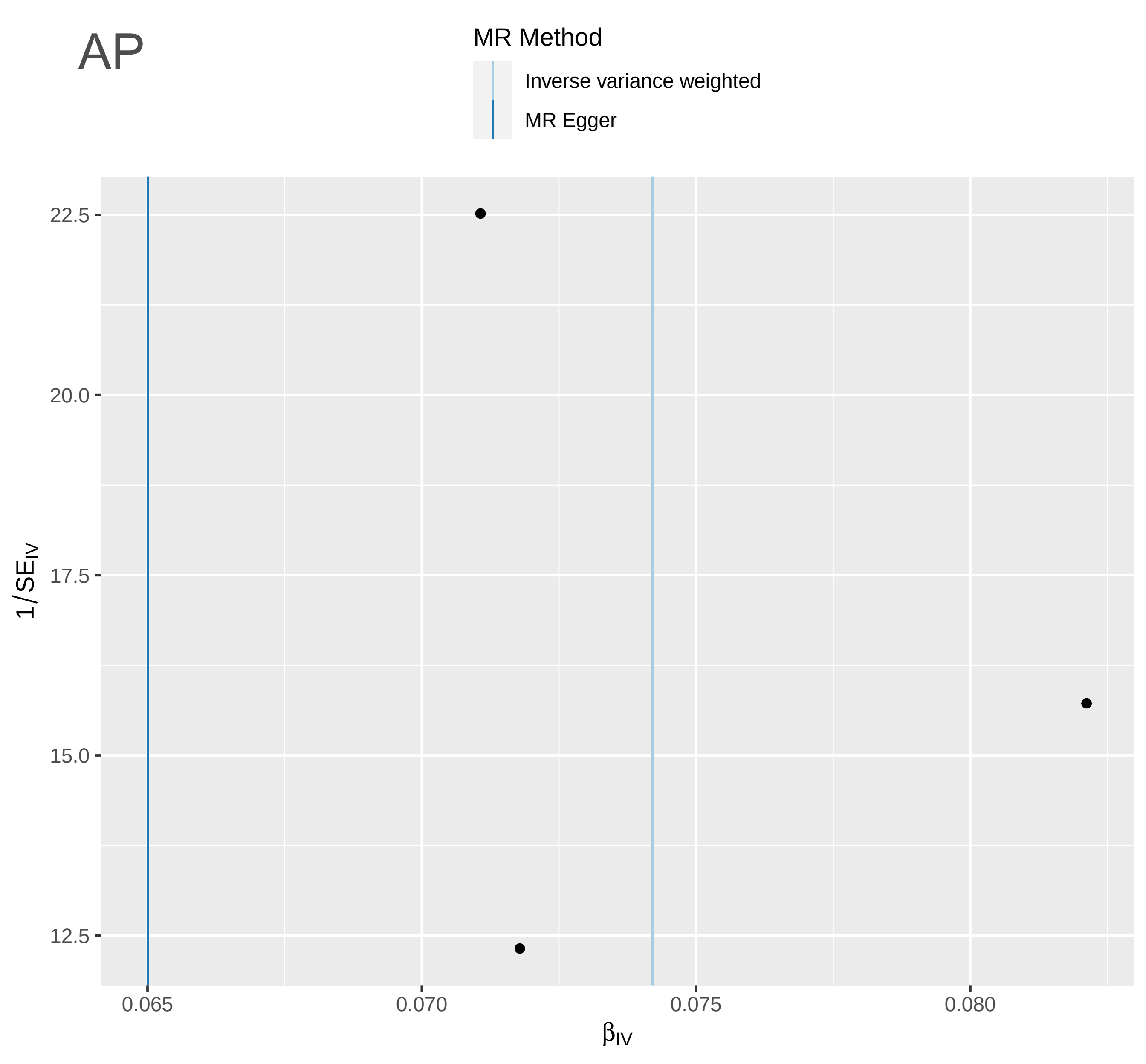

AP
MR Method
Inverse variance weighted
MR Egger
22.5
20.0
17.5
15.0
12.5
1/SE_IV
0.065
0.070
0.075
0.080
β_IV

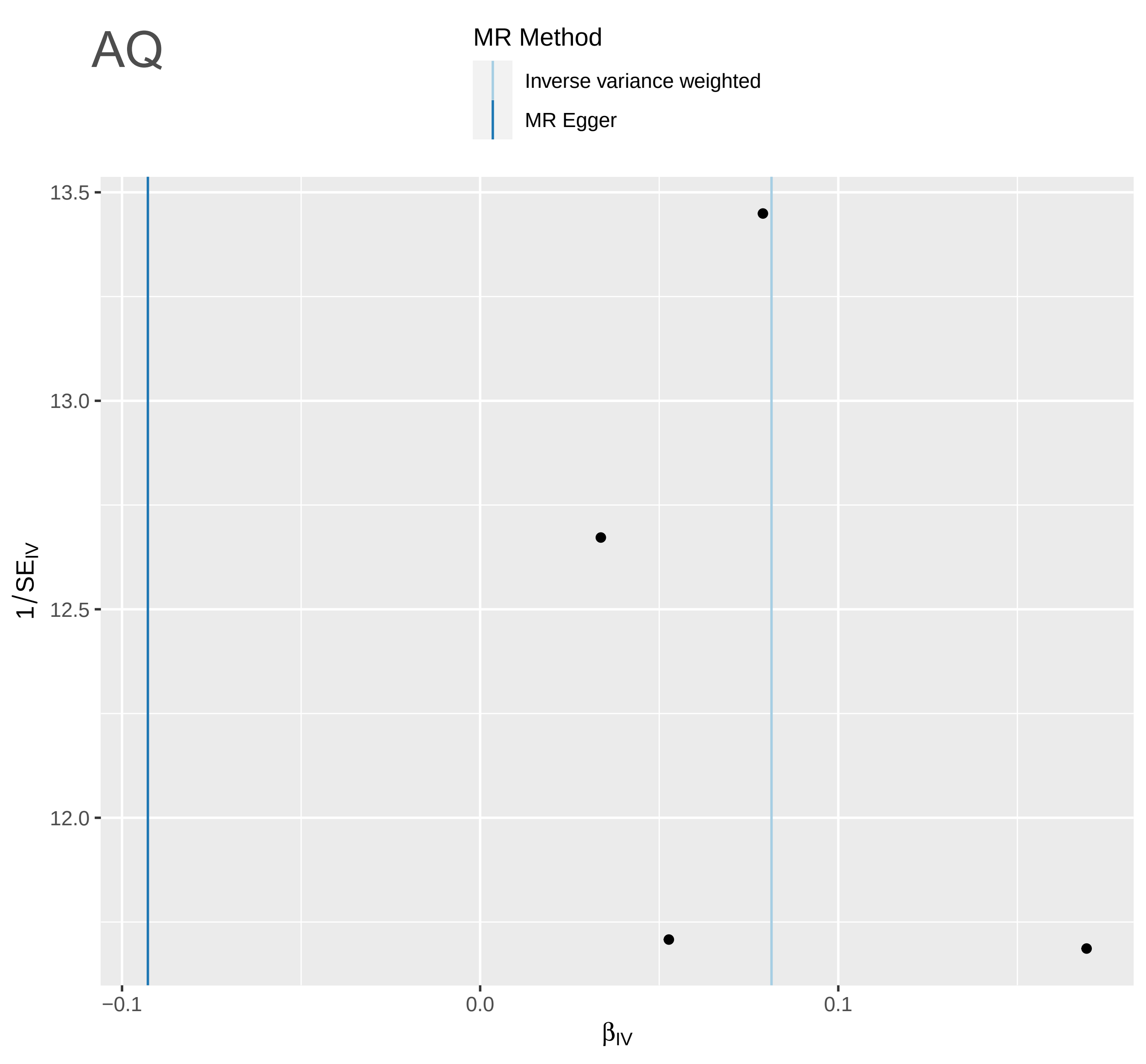
AQ
MR Method
Inverse variance weighted
MR Egger
13.5
13.0
12.5
12.0
1/SE_IV
−0.1
0.0
0.1
β_IV

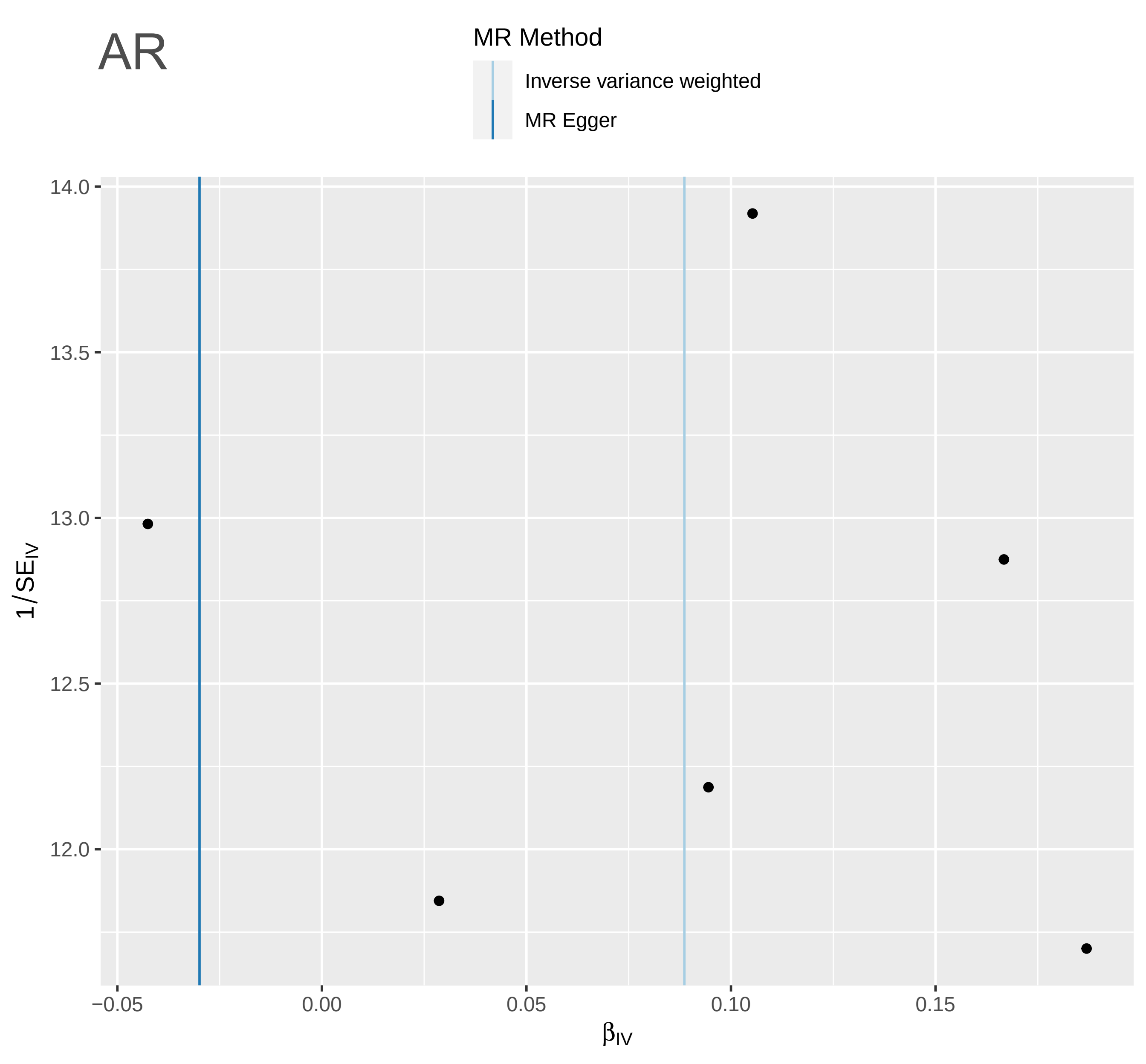
AR
MR Method
Inverse variance weighted
MR Egger
14.0
13.5
13.0
12.5
12.0
$1/SE_{IV}$
−0.05
0.00
0.05
0.10
0.15
β_{IV}

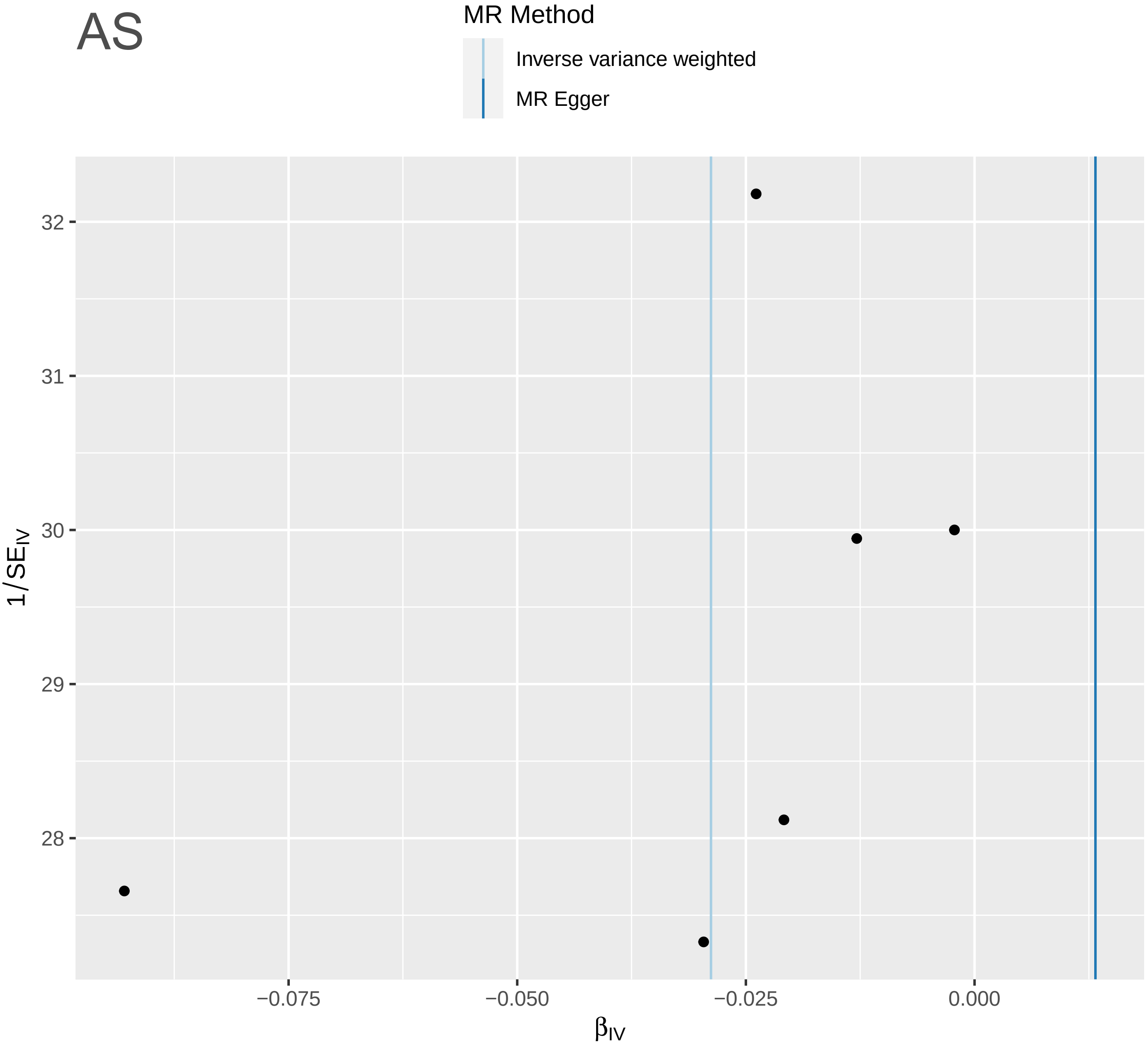

AS
MR Method
Inverse variance weighted
MR Egger
32
31
30
29
28
1/SE_IV
−0.075
−0.050
−0.025
0.000
β_IV

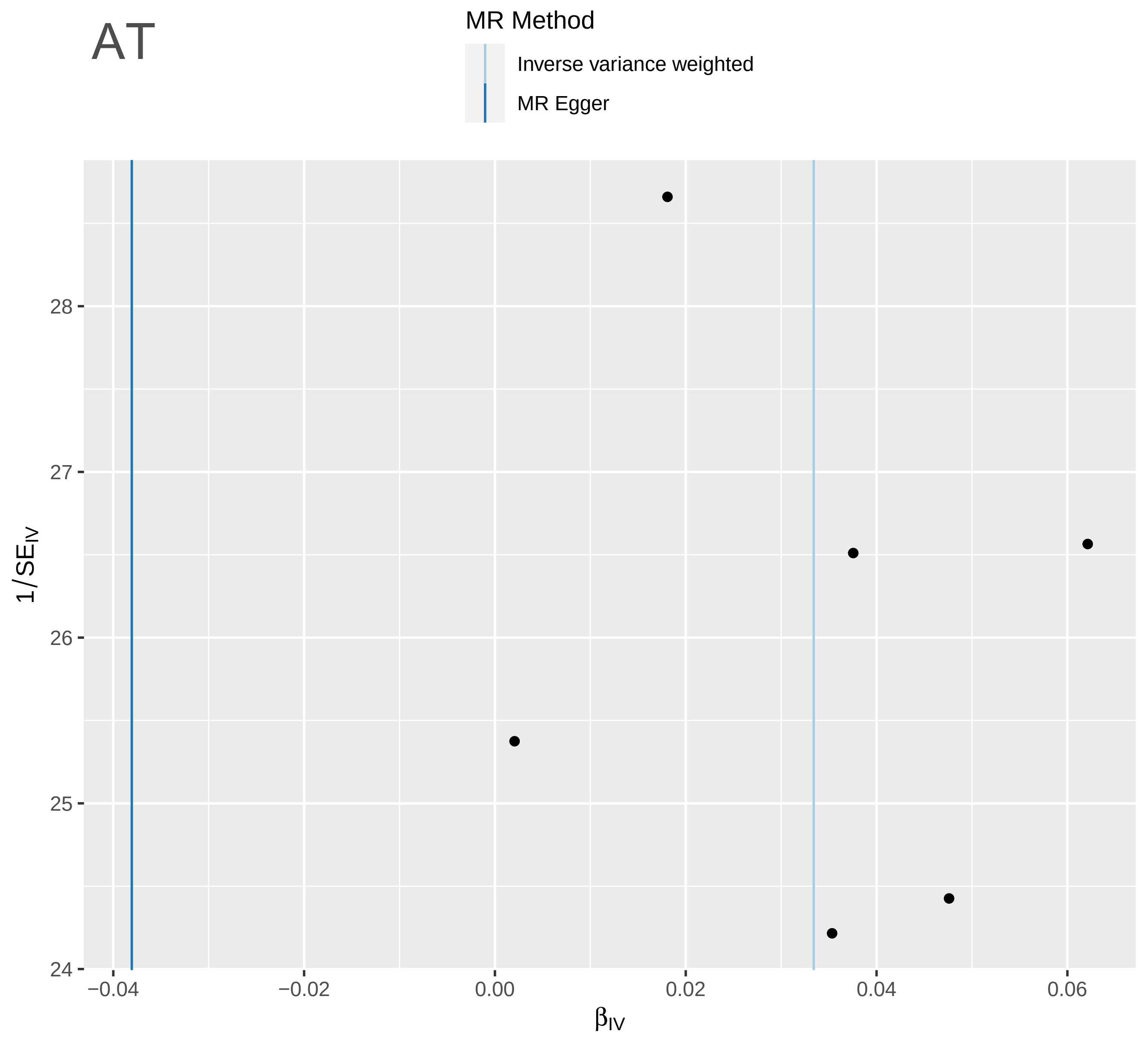

AT
MR Method
Inverse variance weighted
MR Egger
28
27
26
25
24
1/SE_IV
−0.04
−0.02
0.00
0.02
0.04
0.06
β_IV

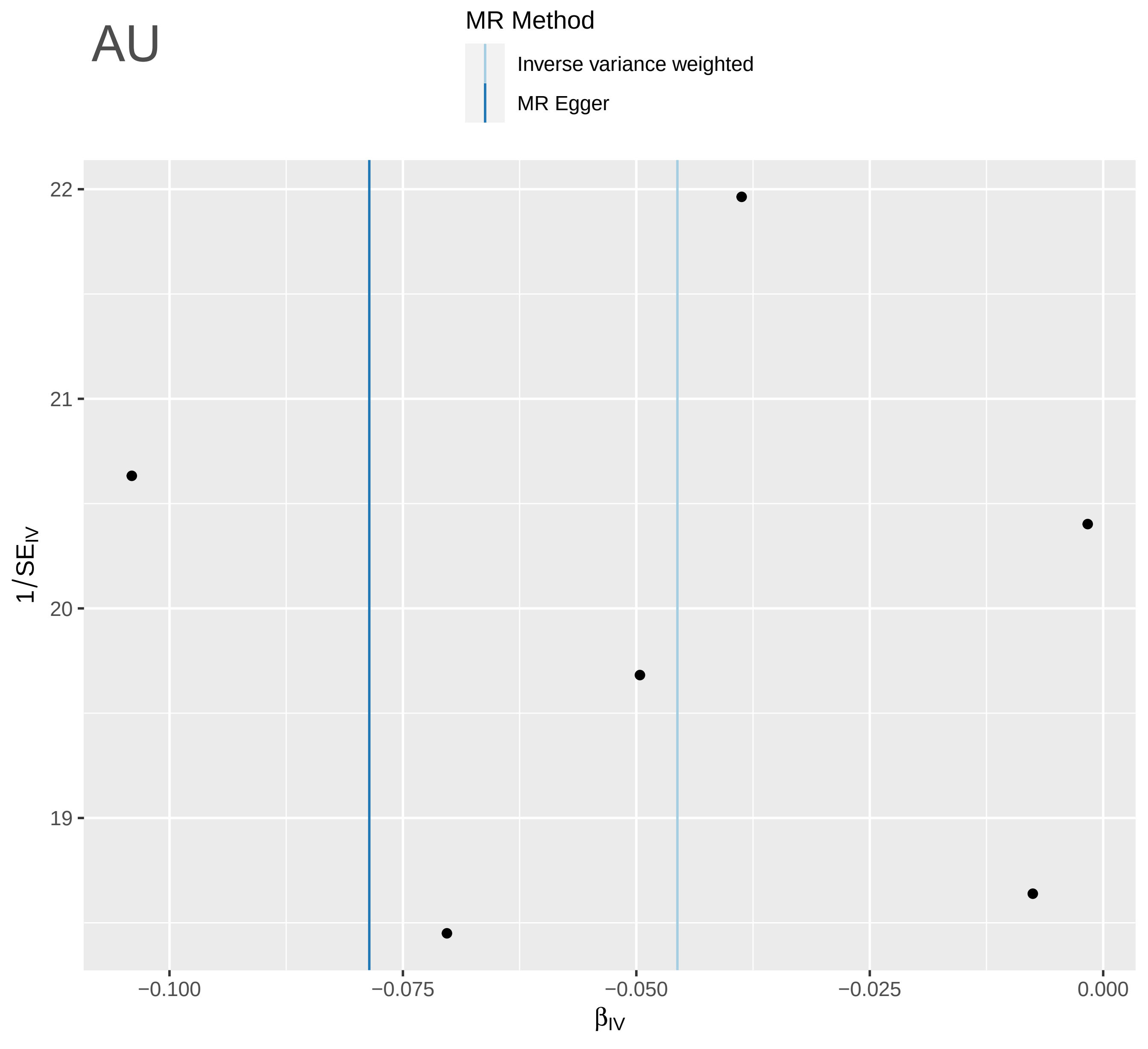
AU
MR Method
Inverse variance weighted
MR Egger
22
21
20
19
1/SE_{IV}
−0.100
−0.075
−0.050
−0.025
0.000
β_{IV}

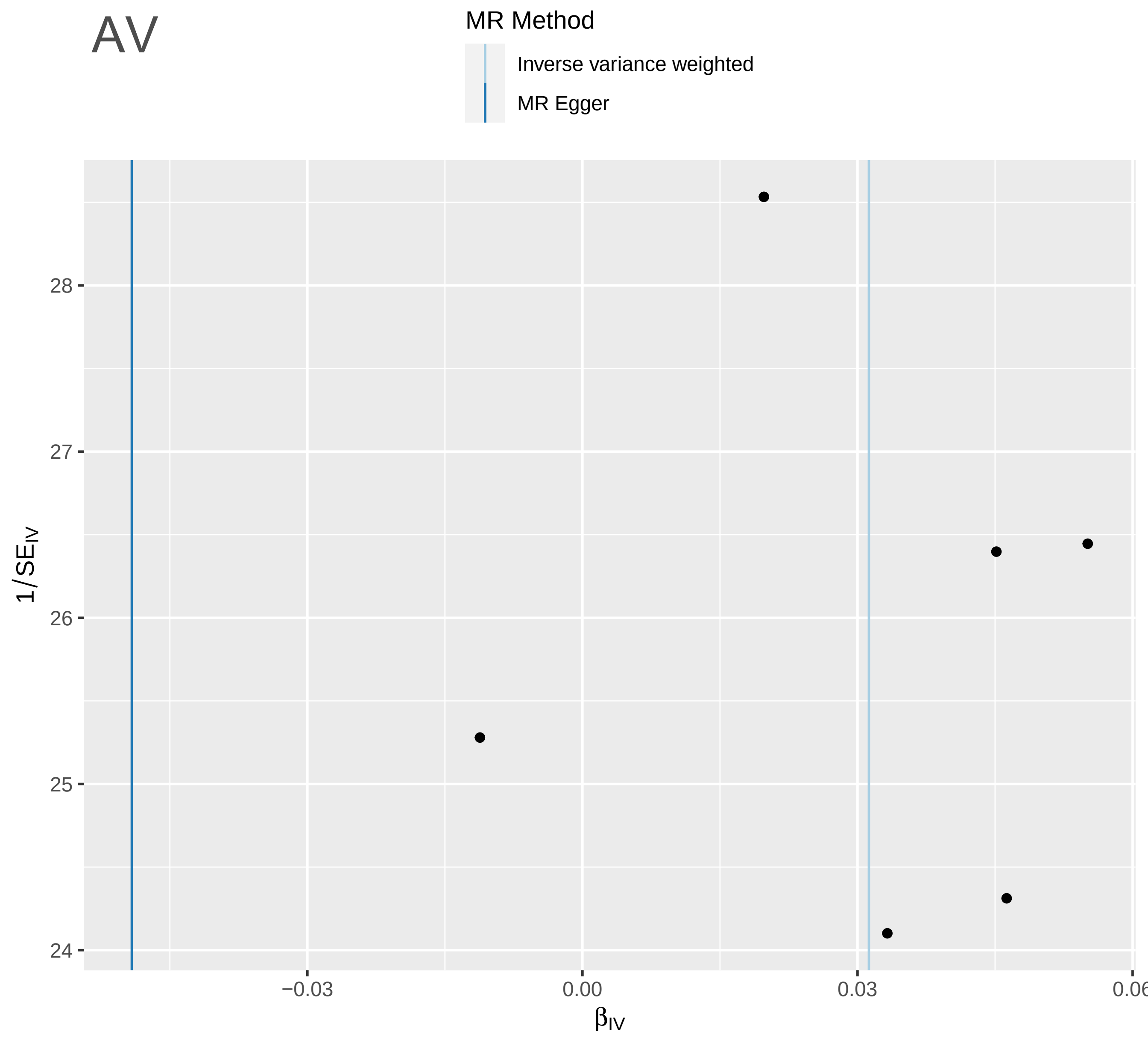

AV
MR Method
Inverse variance weighted
MR Egger
28
27
26
25
24
1/SE_IV
−0.03
0.00
0.03
0.06
β_IV

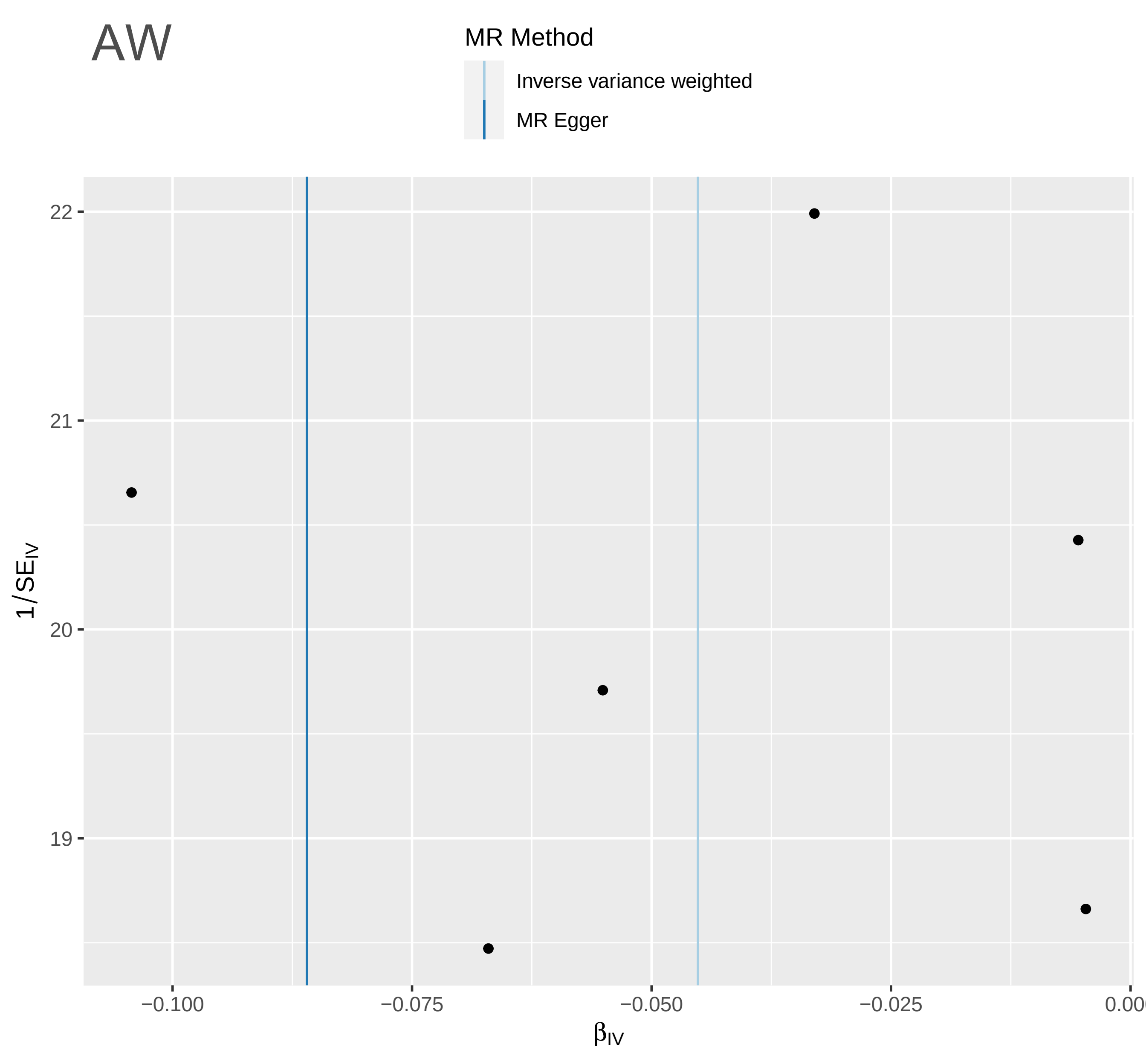
AW
MR Method
Inverse variance weighted
MR Egger
22
21
20
19
1/SE$_{IV}$
−0.100
−0.075
−0.050
−0.025
β$_{IV}$

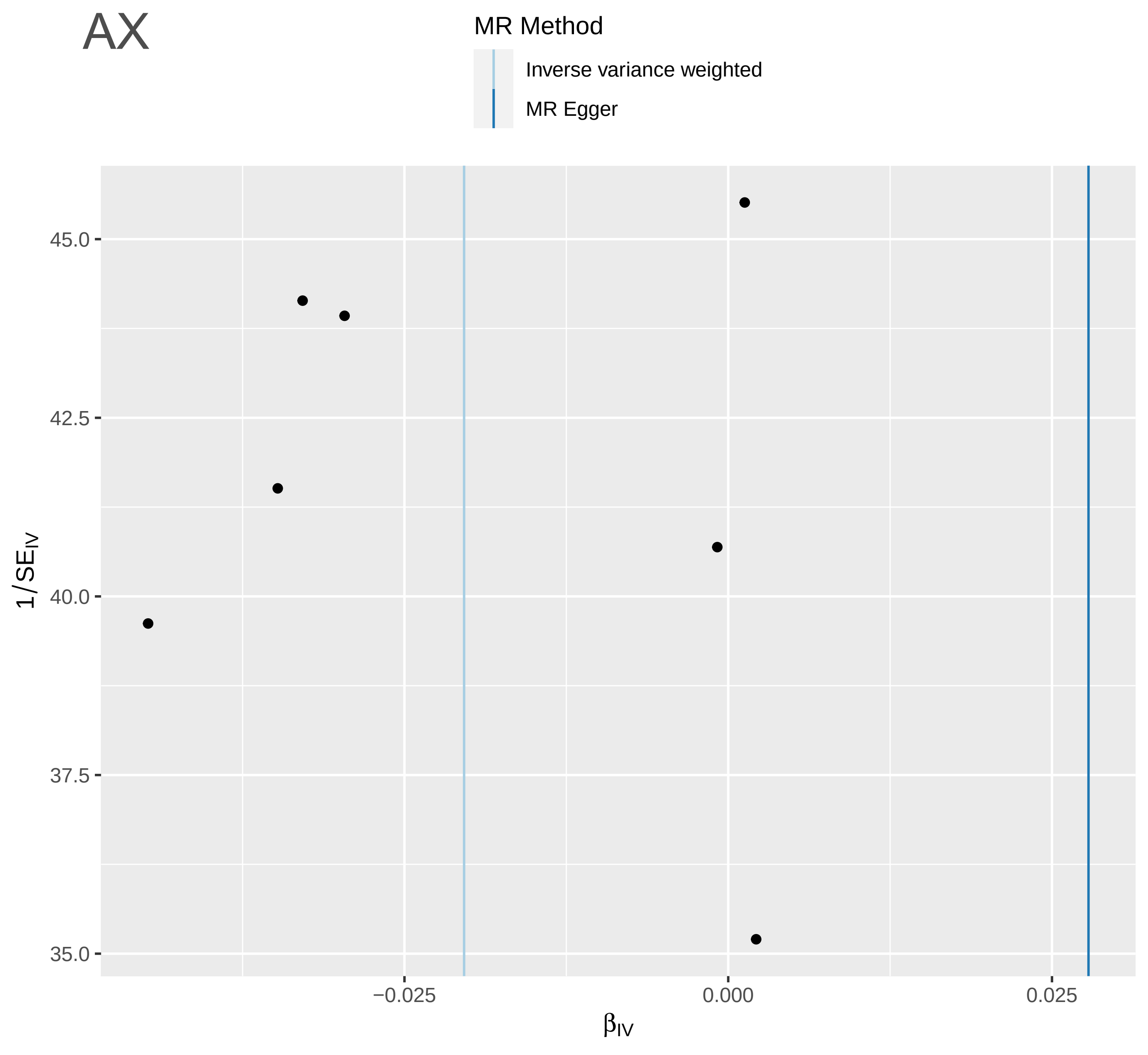
AX
MR Method
Inverse variance weighted
MR Egger
45.0
42.5
40.0
37.5
35.0
1/SE$_{IV}$
−0.025
0.000
0.025
β$_{IV}$

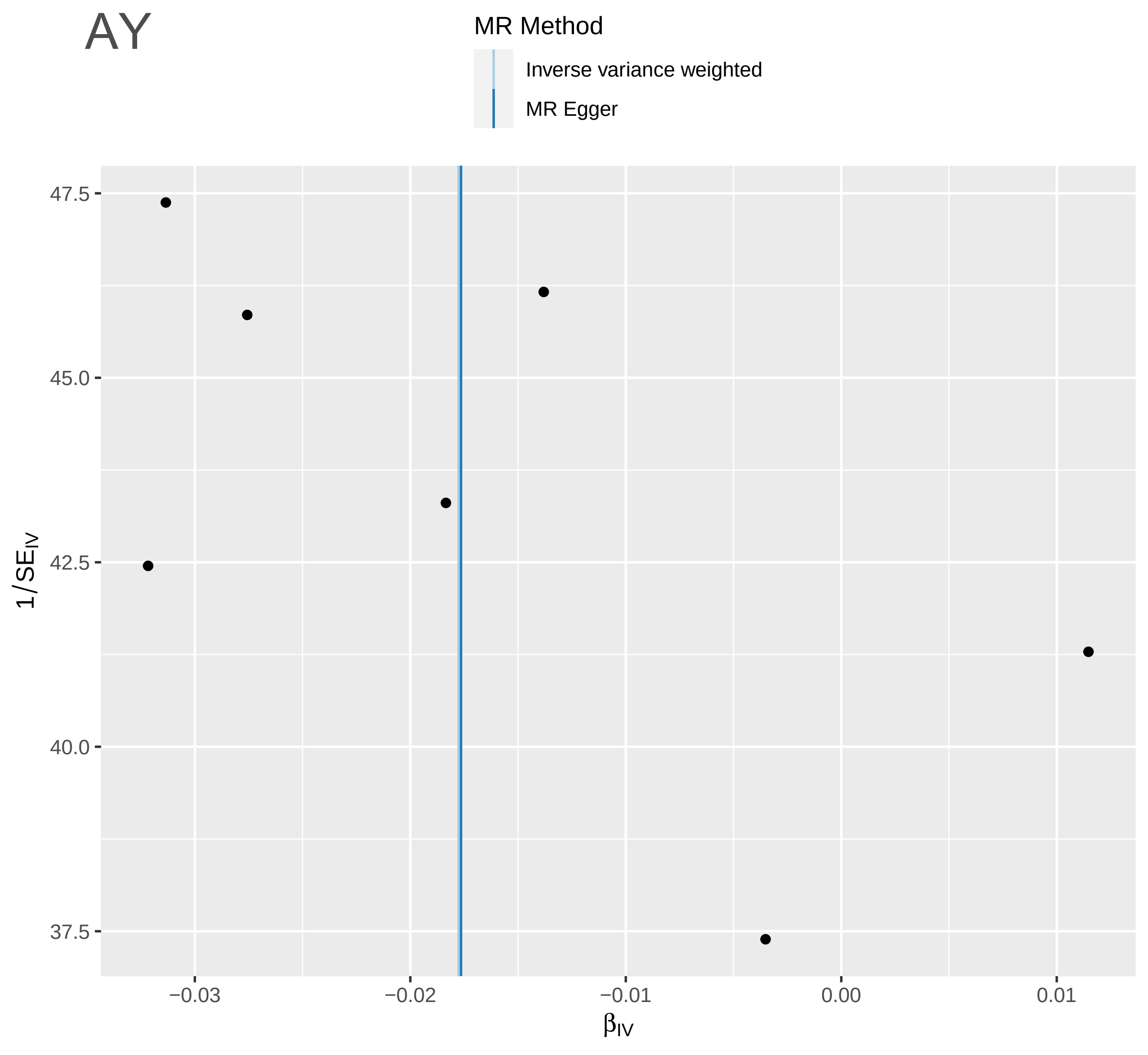
AY
MR Method
Inverse variance weighted
MR Egger
47.5
45.0
42.5
40.0
37.5
$1/SE_{IV}$
−0.03
−0.02
−0.01
0.00
0.01
β_{IV}

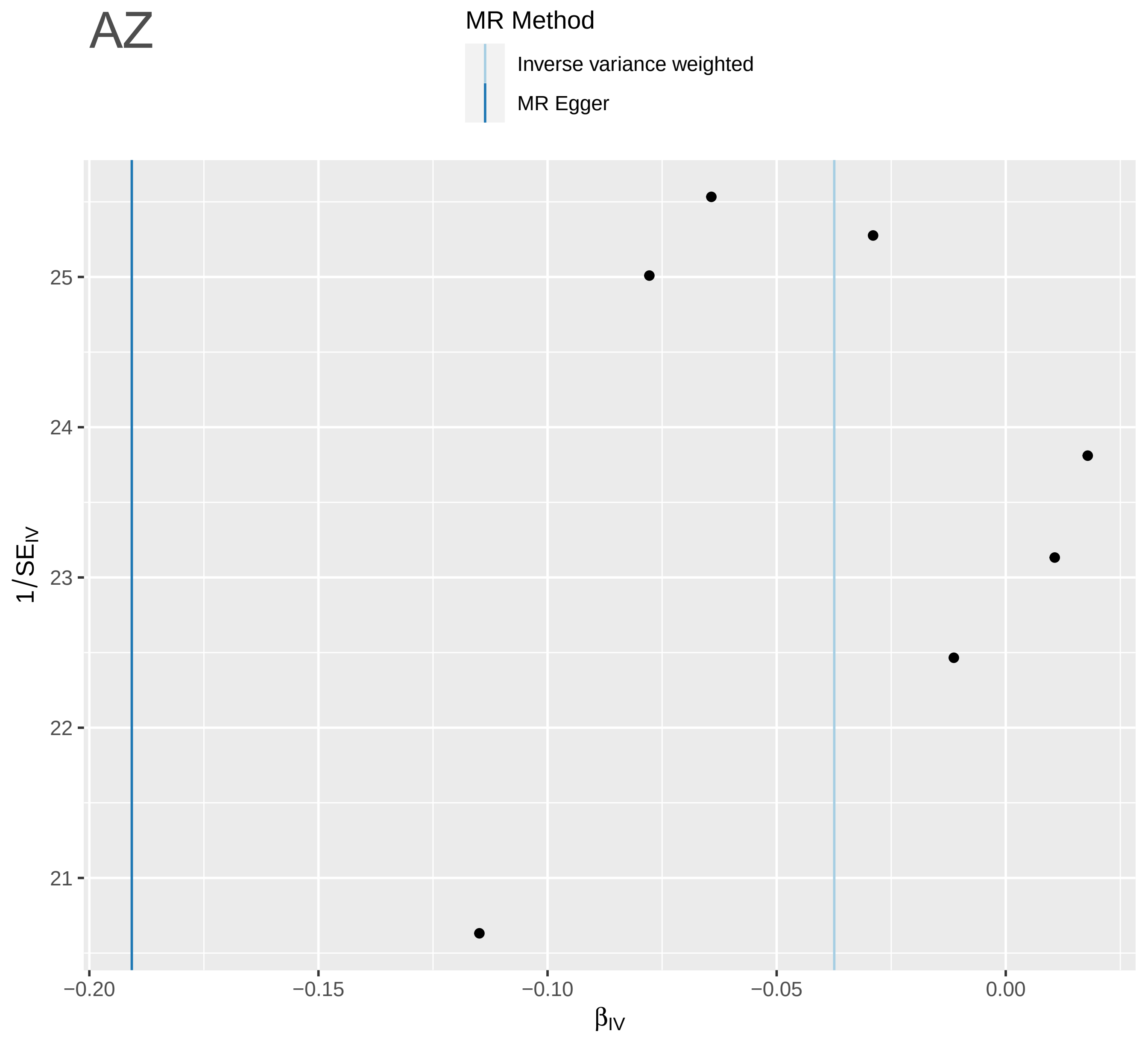
AZ
MR Method
Inverse variance weighted
MR Egger
25
24
23
22
21
1/SE_IV
−0.20
−0.15
−0.10
−0.05
0.00
β_IV

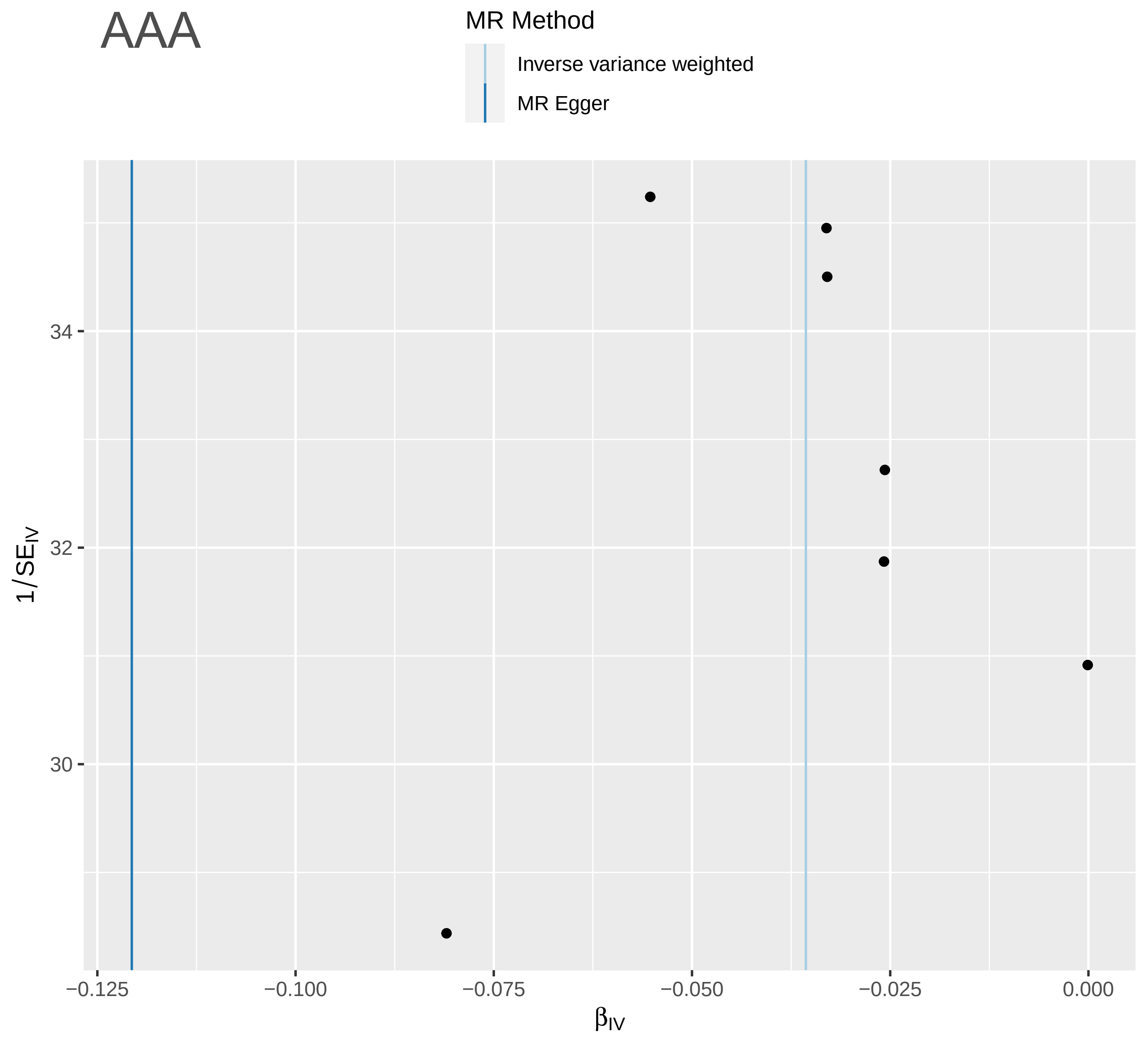
AAA
MR Method
Inverse variance weighted
MR Egger
34
32
30
$1/SE_{IV}$
−0.125
−0.100
−0.075
−0.050
−0.025
0.000
β_{IV}

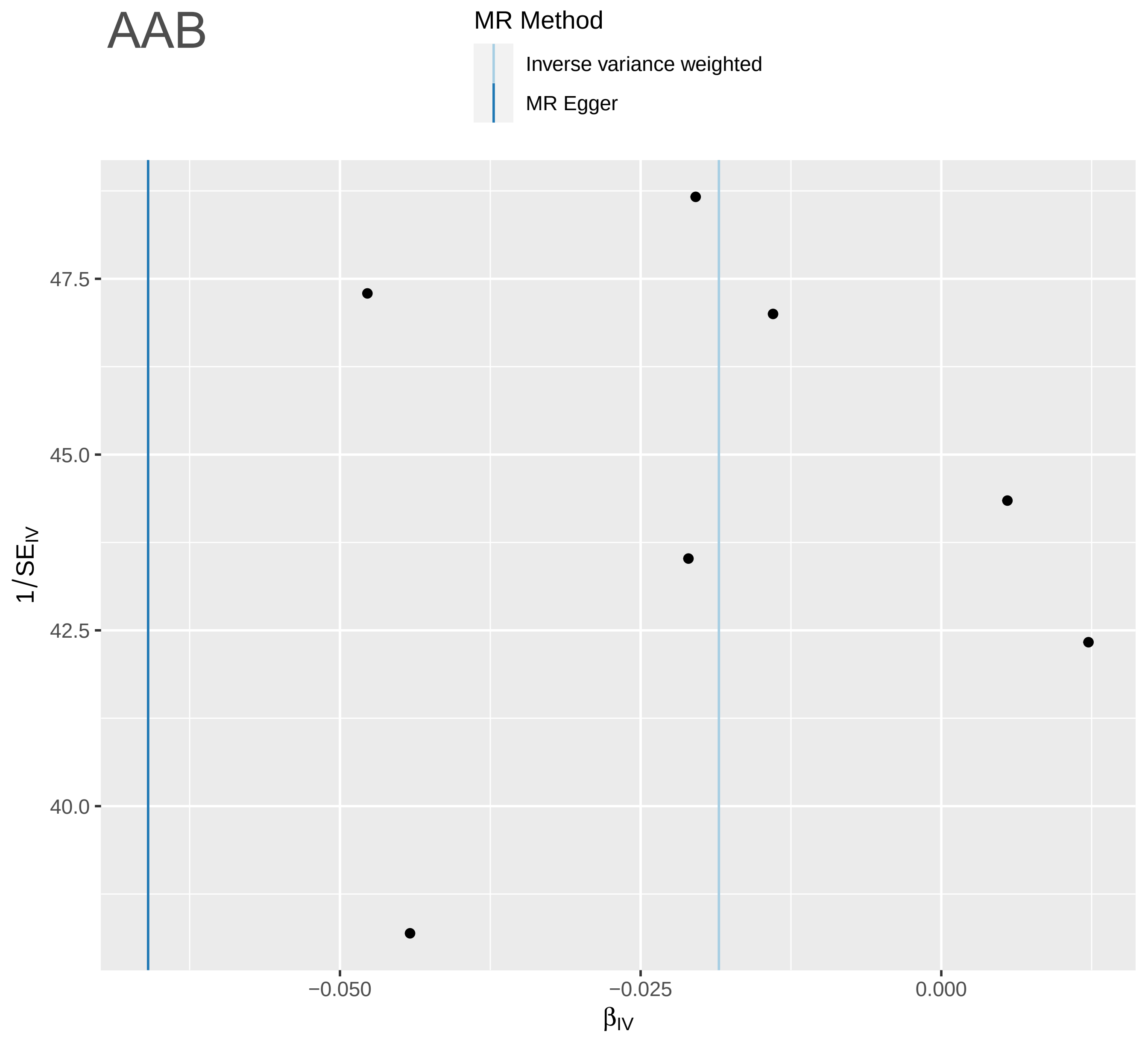

AAB
MR Method
Inverse variance weighted
MR Egger
47.5
45.0
42.5
40.0
1/SE_IV
−0.050
−0.025
0.000
β_IV

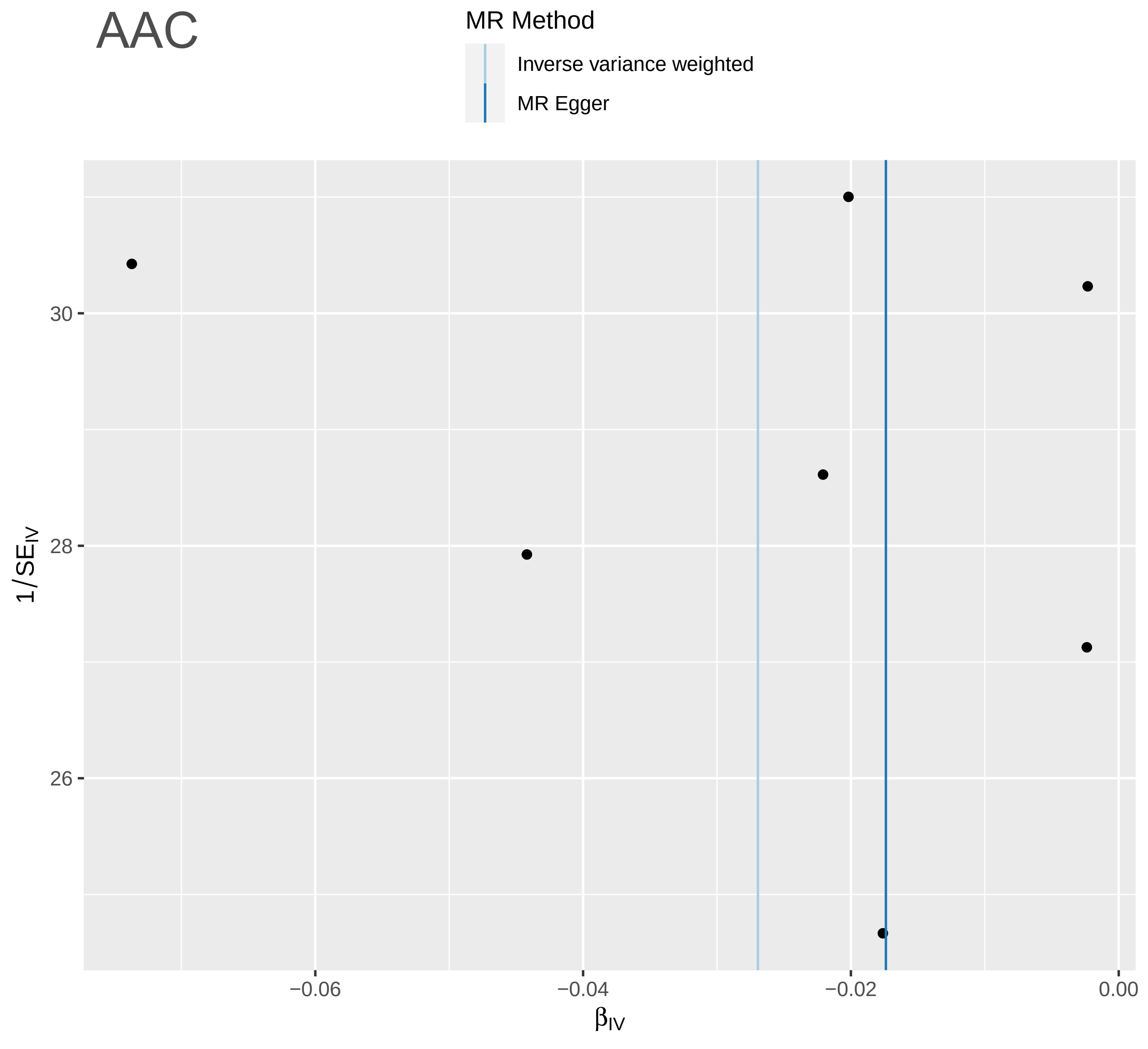

AAC
MR Method
Inverse variance weighted
MR Egger
30
28
26
1/SE_IV
−0.06
−0.04
−0.02
0.00
β_IV

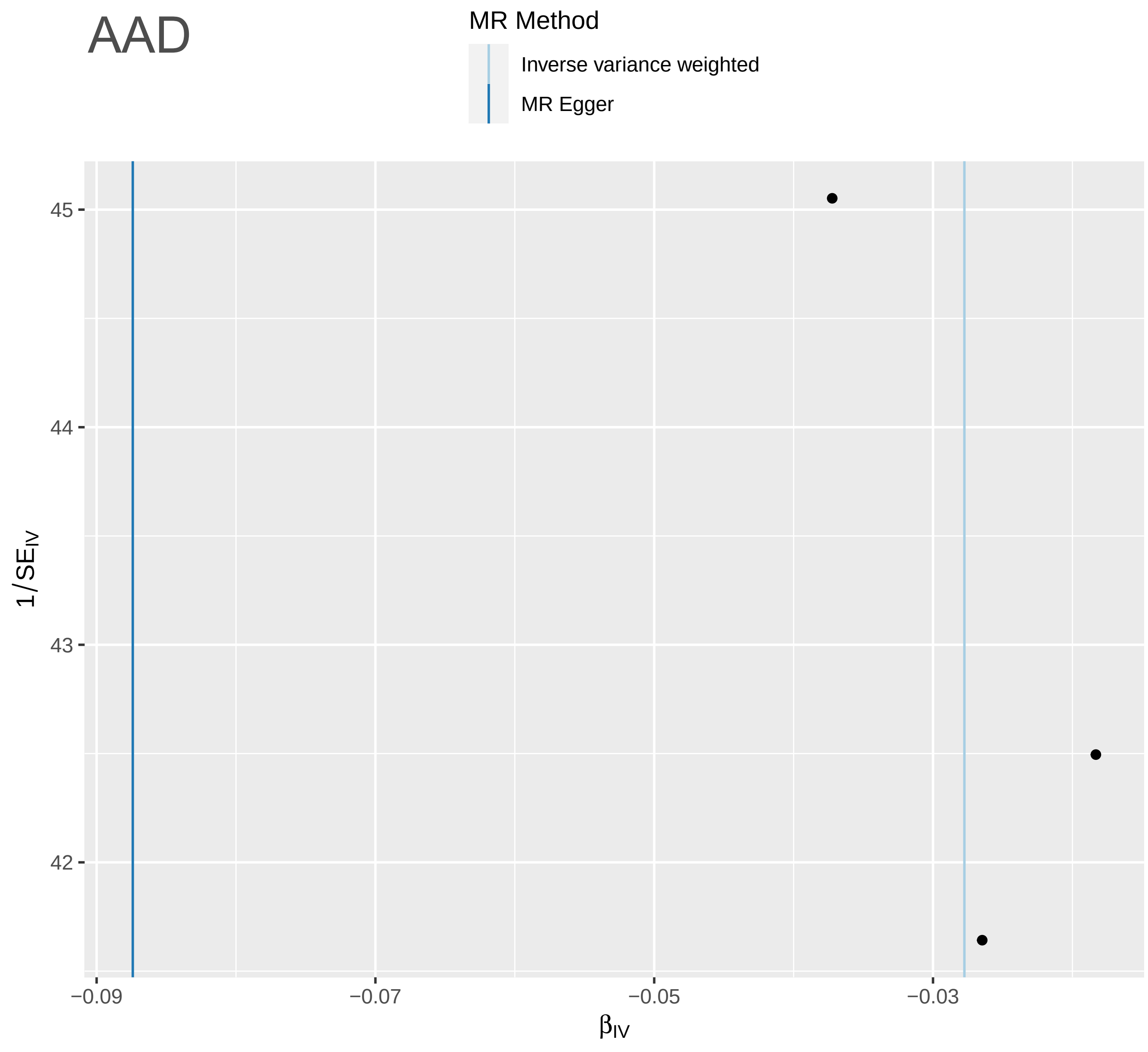
AAD
MR Method
Inverse variance weighted
MR Egger
45
44
43
42
1/SE_IV
−0.09
−0.07
−0.05
−0.03
β_IV

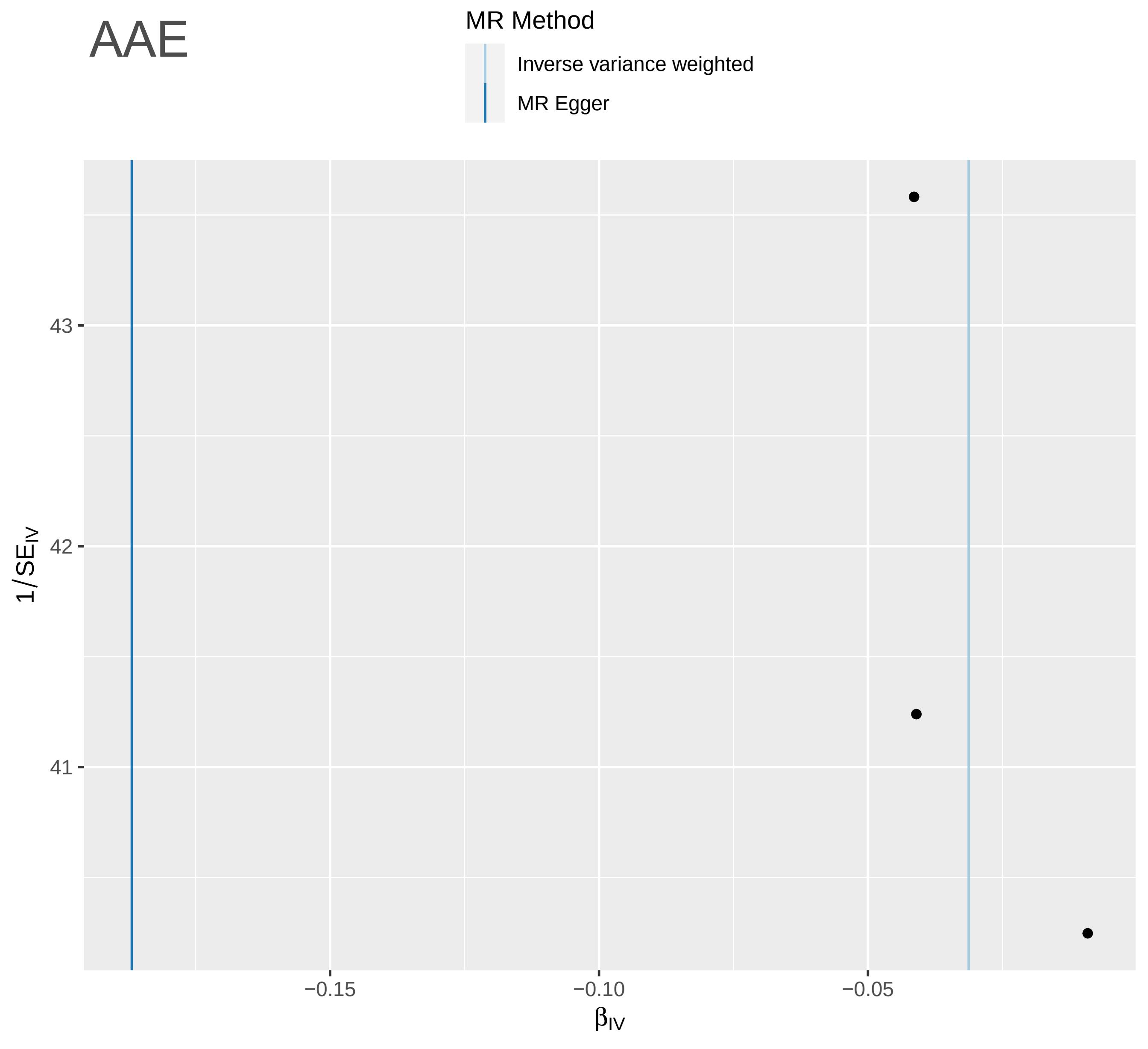
AAE
MR Method
Inverse variance weighted
MR Egger
43
42
41
1/SE_IV
−0.15
−0.10
−0.05
β_IV

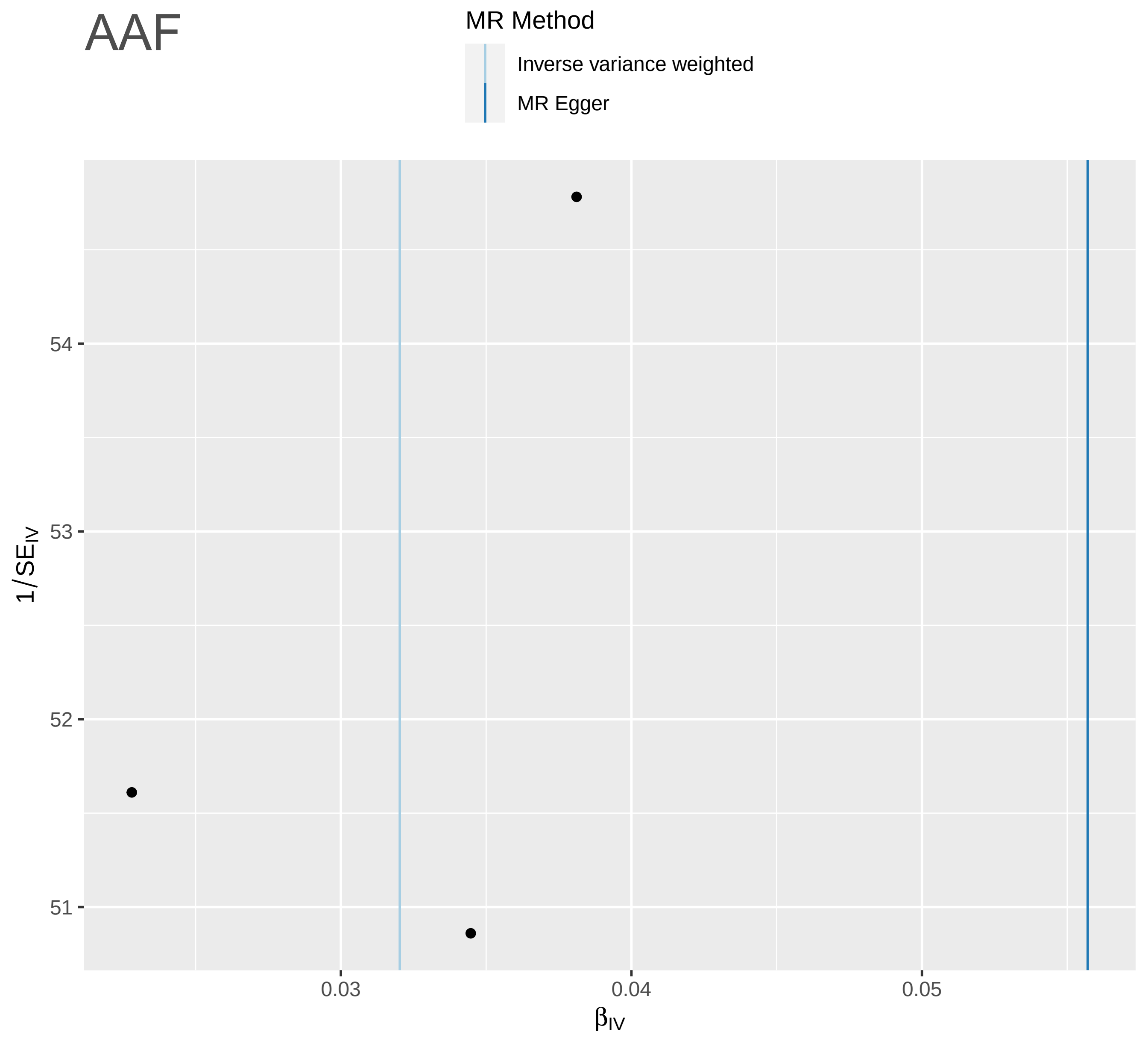
AAF
MR Method
Inverse variance weighted
MR Egger
54
53
52
51
0.03
0.04
0.05
$1/SE_{IV}$
β_{IV}

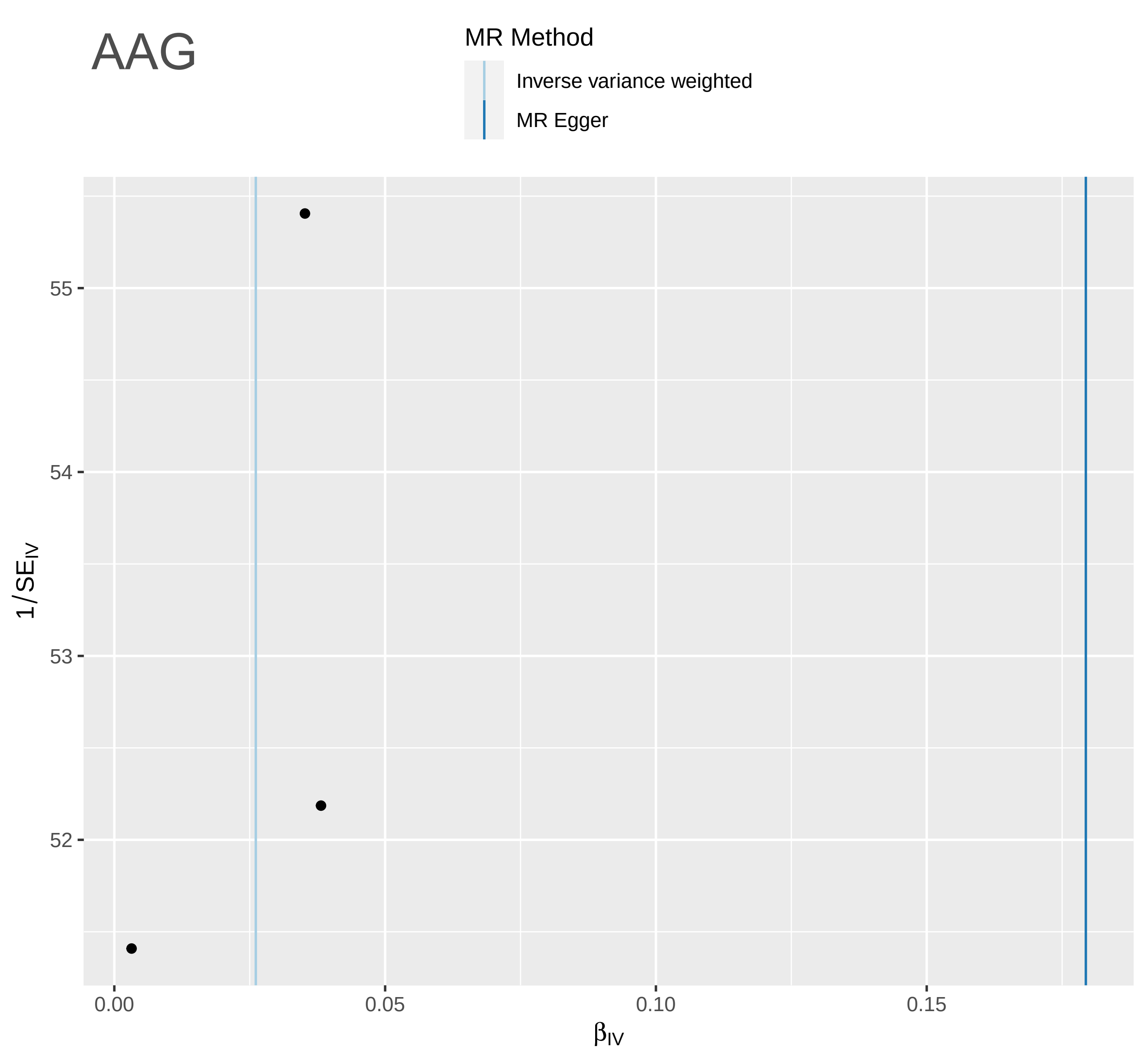

AAG
MR Method
Inverse variance weighted
MR Egger
55
54
53
52
1/SE_IV
0.00
0.05
0.10
0.15
β_IV

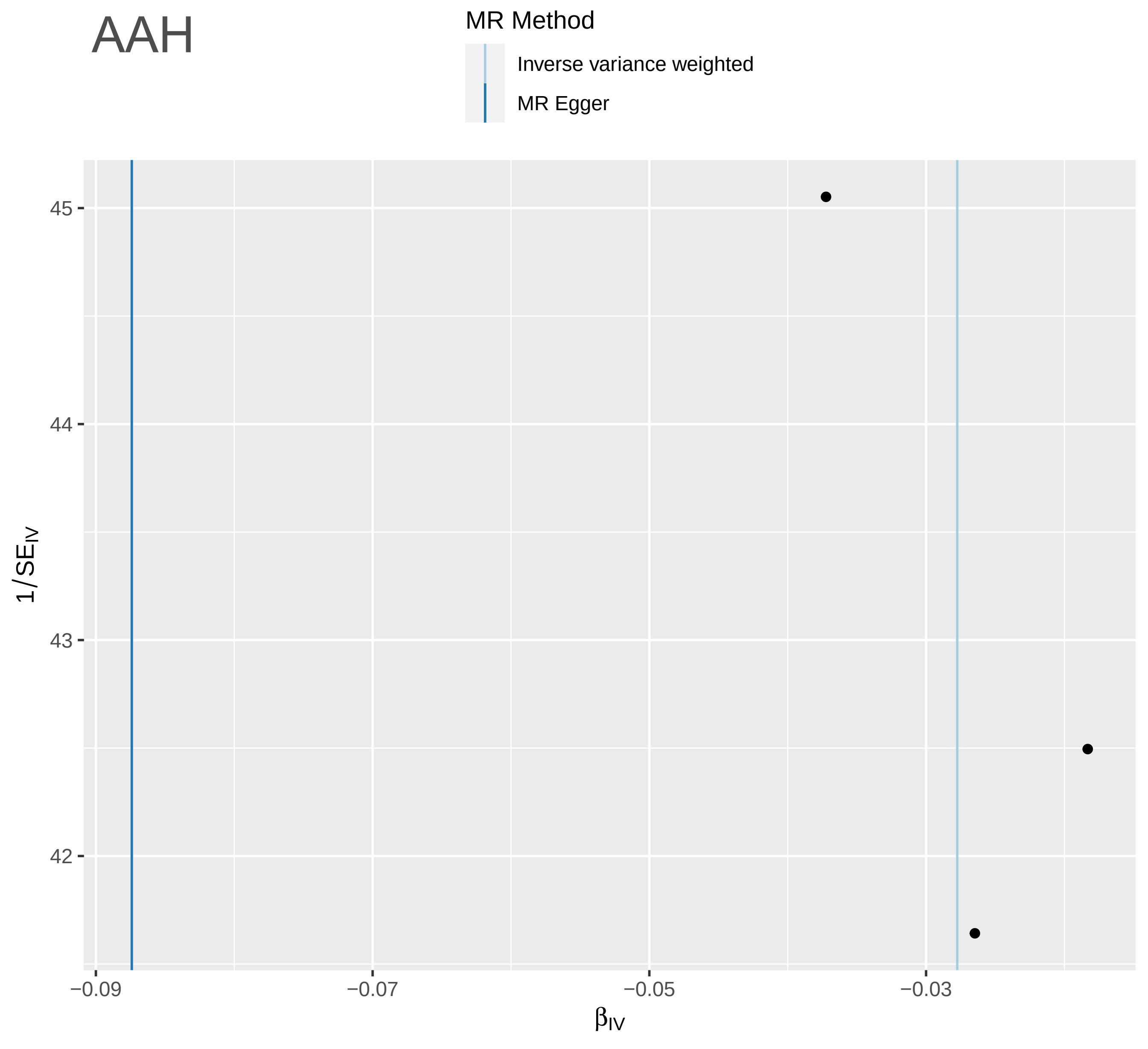
AAH
MR Method
Inverse variance weighted
MR Egger
45
44
43
42
1/SE_IV
−0.09
−0.07
−0.05
−0.03
β_IV

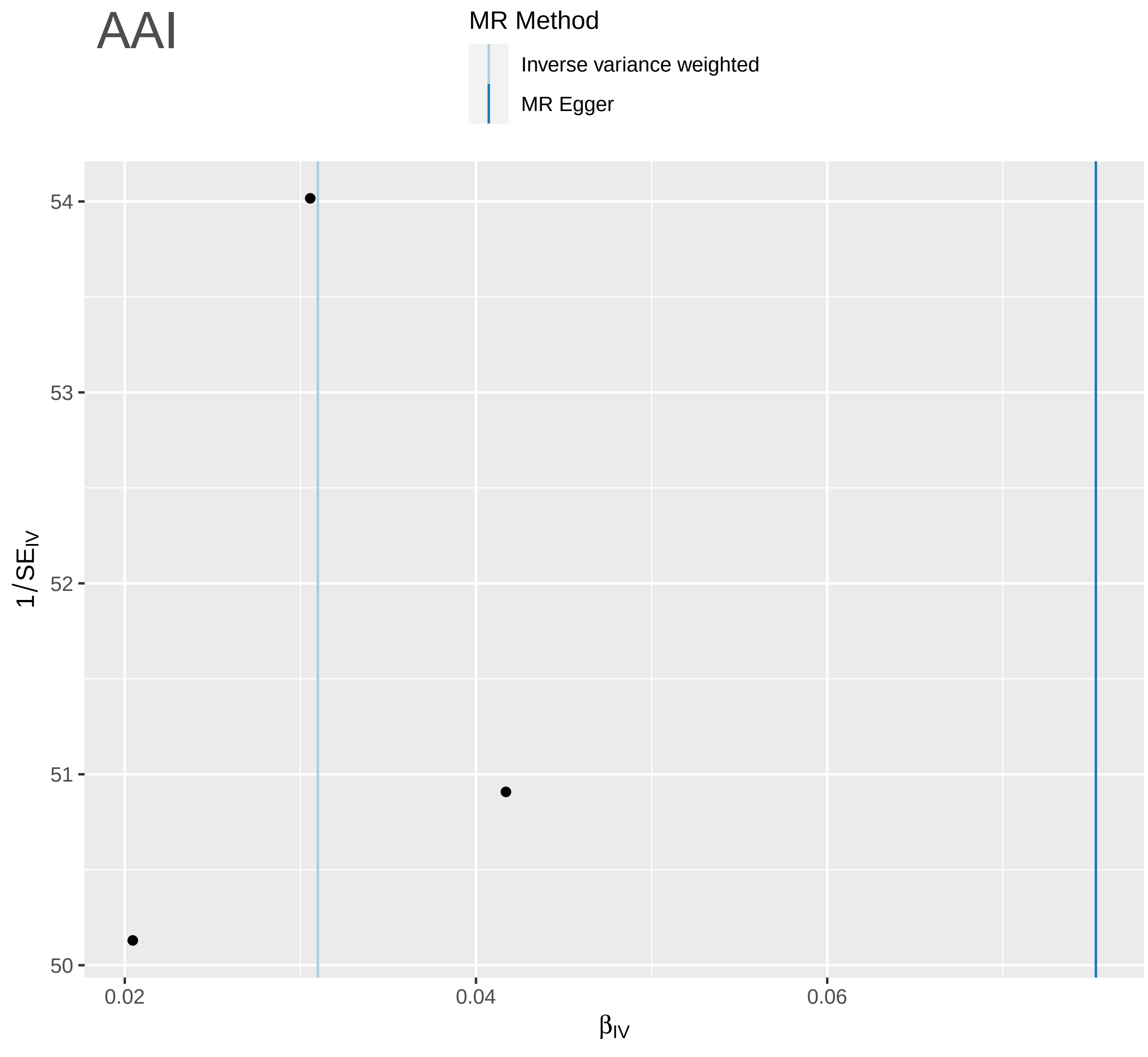

AAI
MR Method
Inverse variance weighted
MR Egger
54
53
52
51
50
$1/SE_{IV}$
0.02
0.04
0.06
β_{IV}

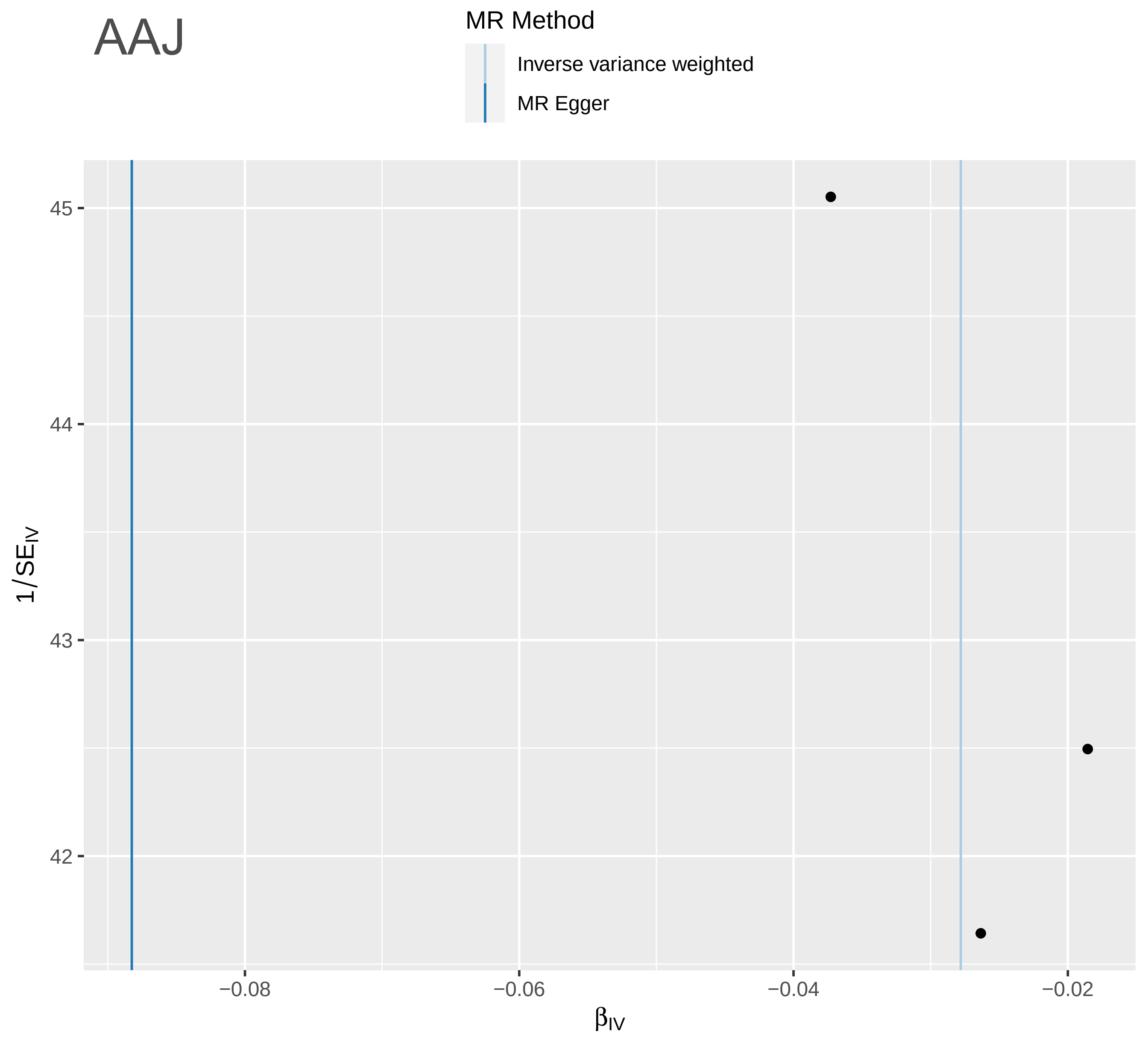
AAJ
MR Method
Inverse variance weighted
MR Egger
45
44
43
42
$1/SE_{IV}$
−0.08
−0.06
−0.04
−0.02
β_{IV}

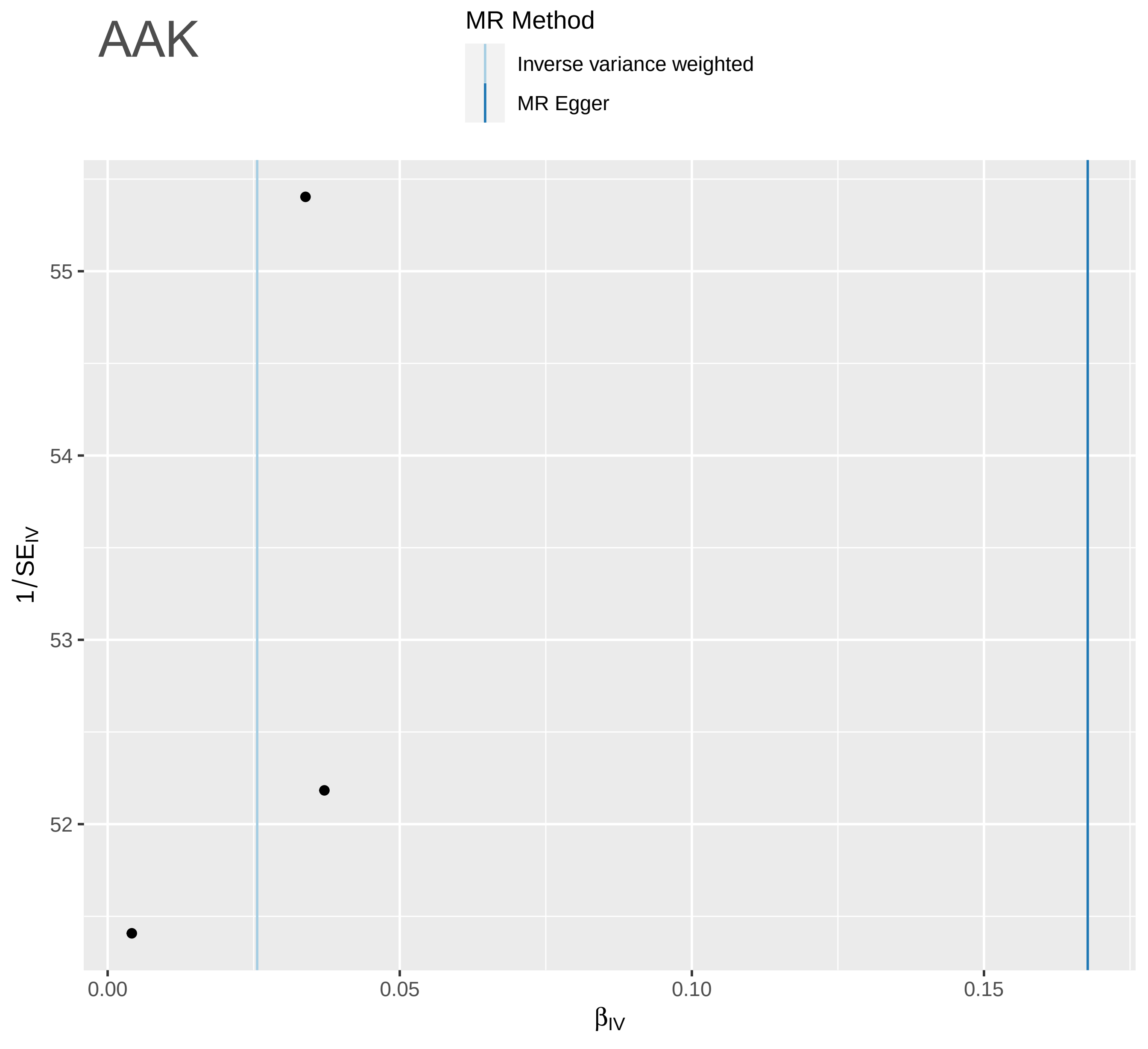
AAK
MR Method
Inverse variance weighted
MR Egger
55
54
53
52
1/SE_IV
0.00
0.05
0.10
0.15
β_IV

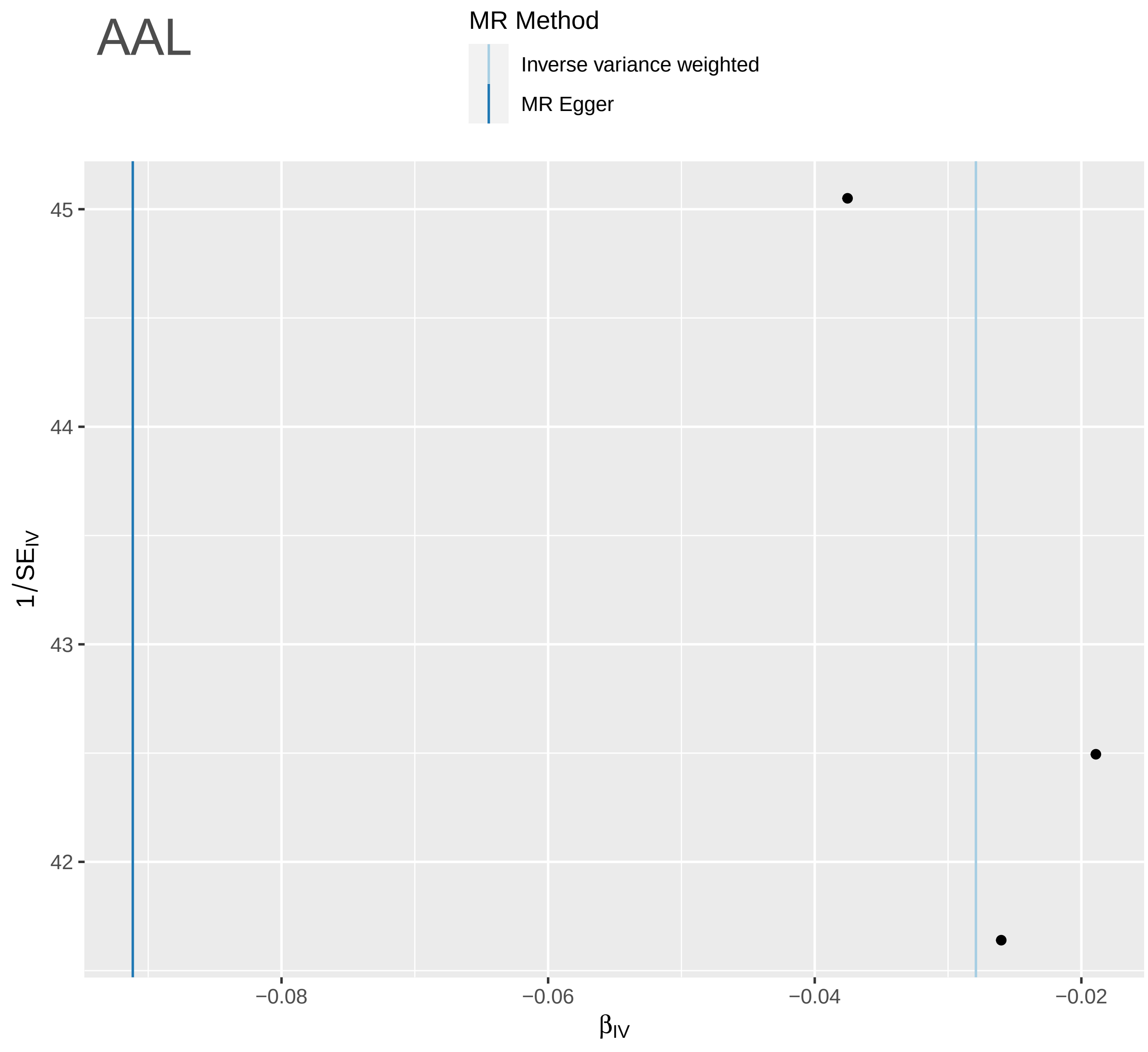
AAL
MR Method
Inverse variance weighted
MR Egger
45
44
43
42
1/SE$_{IV}$
−0.08
−0.06
−0.04
−0.02
β$_{IV}$

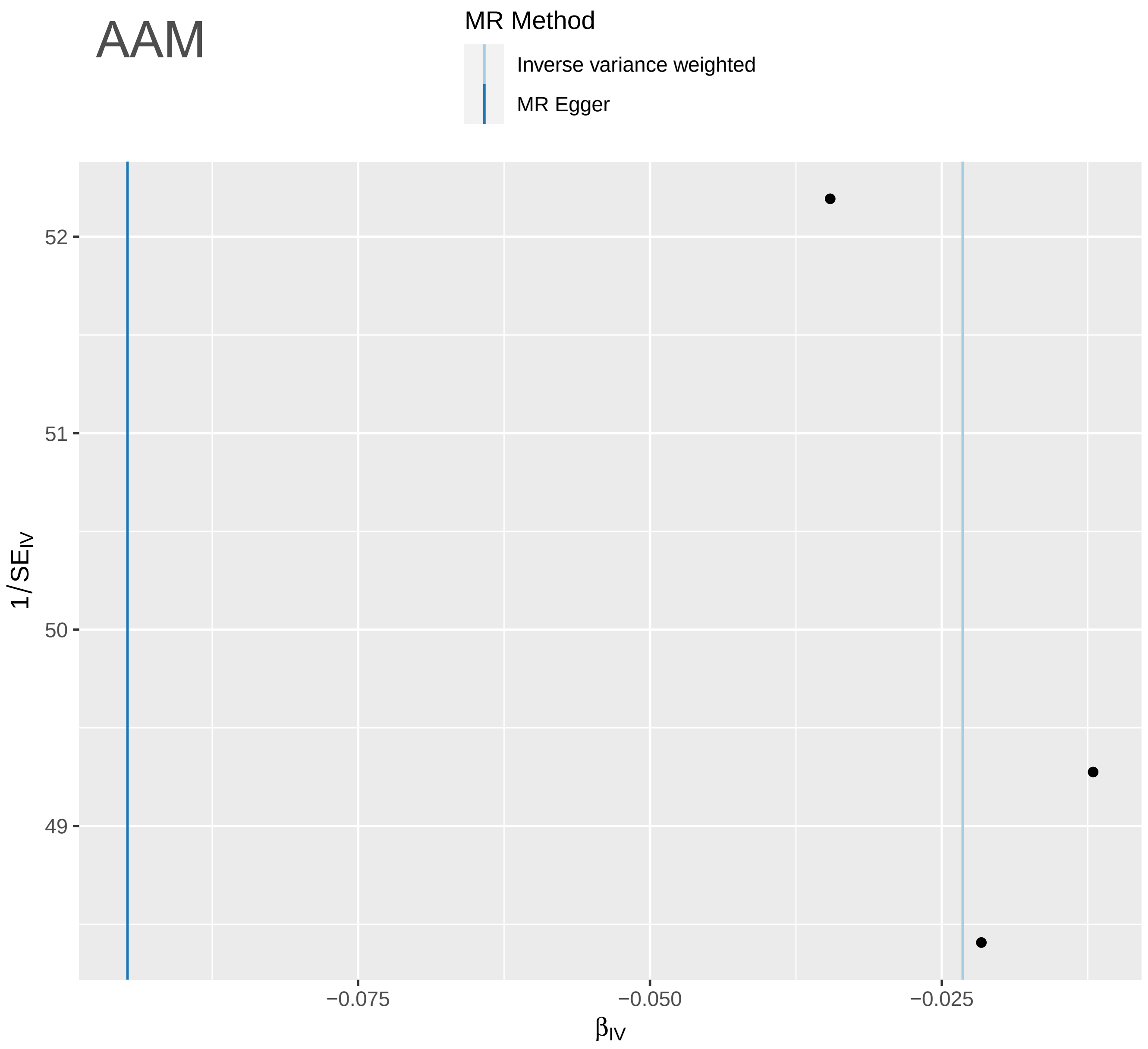
AAM
MR Method
Inverse variance weighted
MR Egger
52
51
50
49
1/SE_IV
−0.075
−0.050
−0.025
β_IV

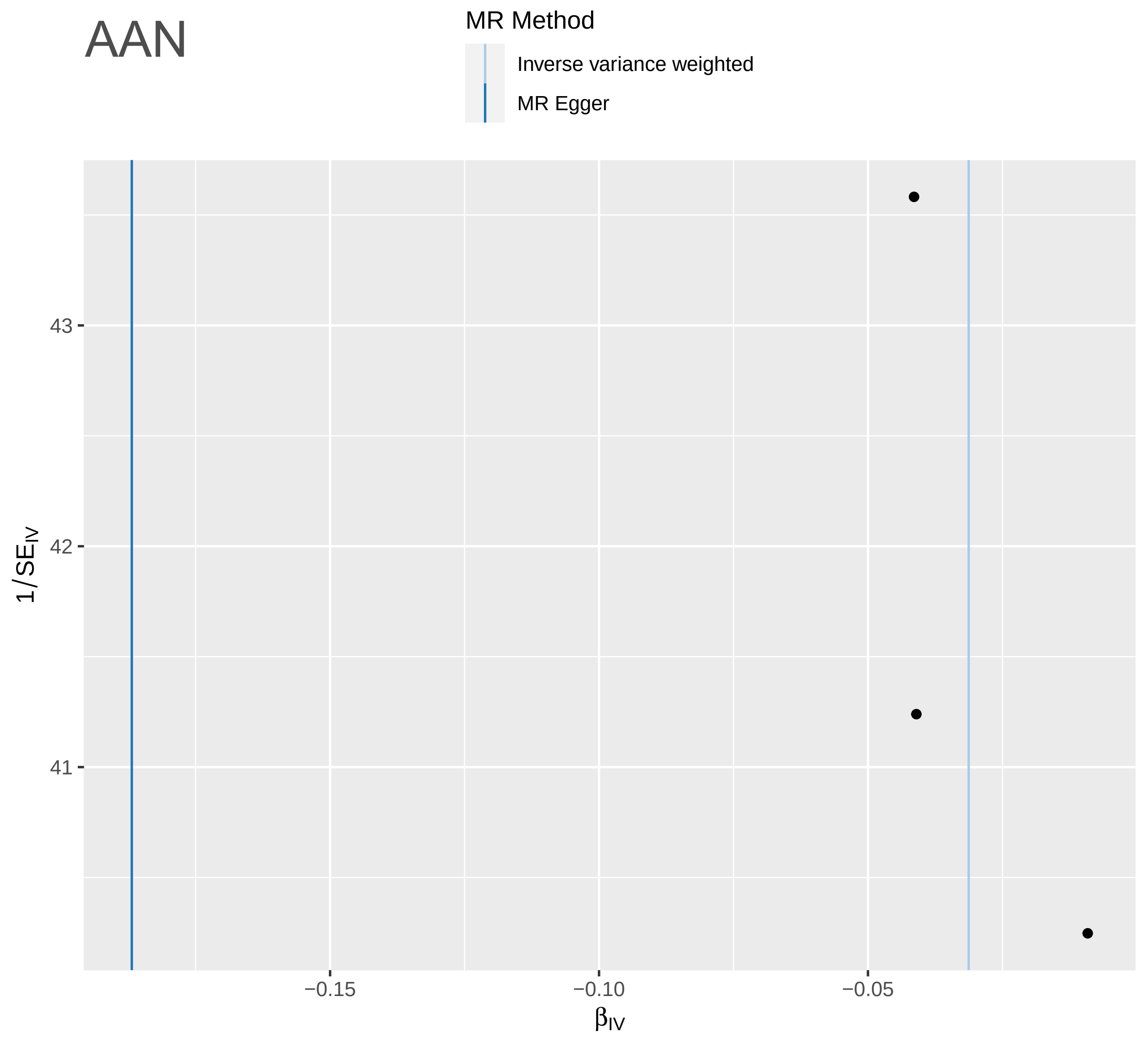
AAN
MR Method
Inverse variance weighted
MR Egger
43
42
41
1/SE_IV
−0.15
−0.10
−0.05
β_IV

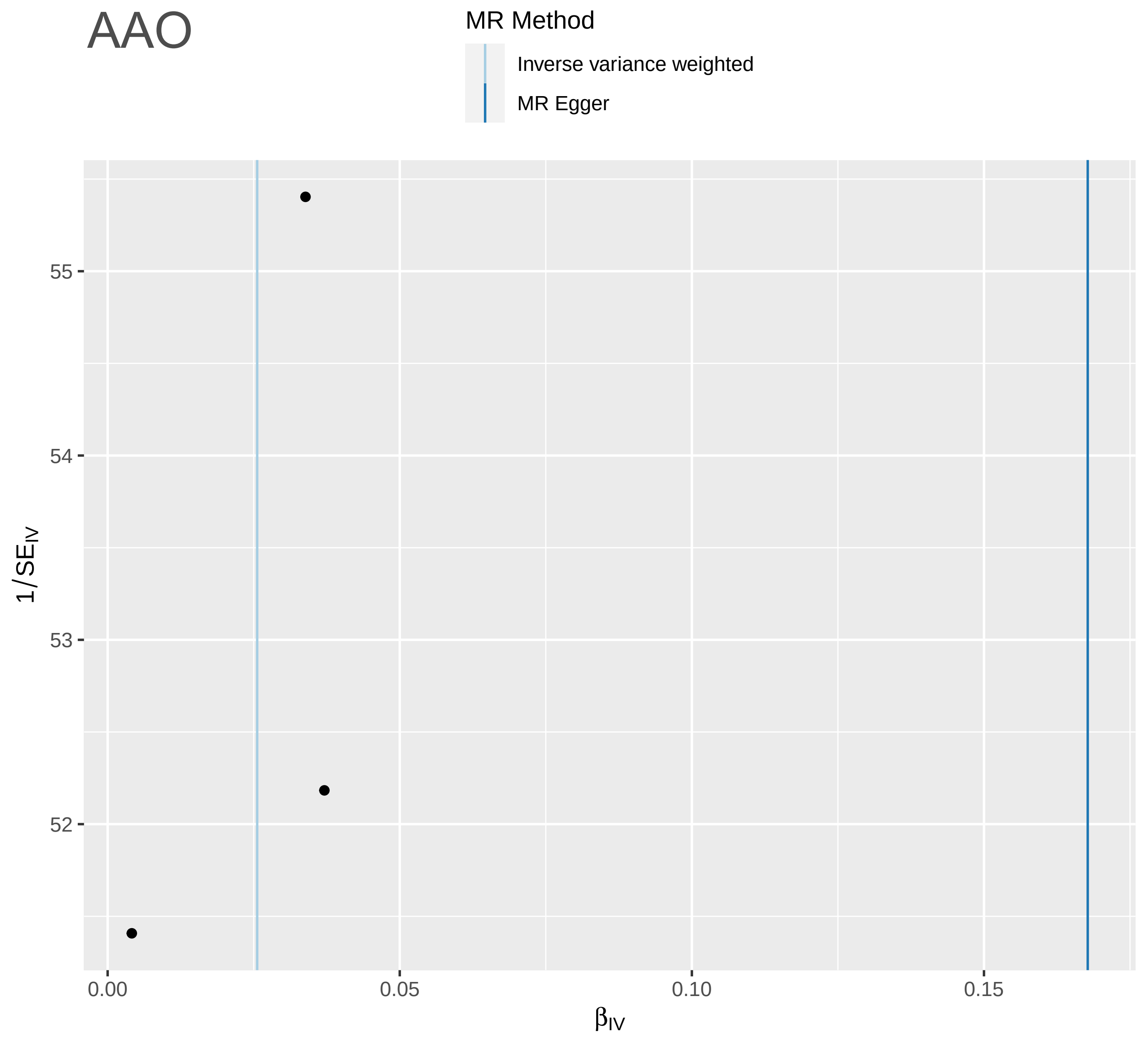
AAO
MR Method
Inverse variance weighted
MR Egger
55
54
53
52
1/SE_IV
0.00
0.05
0.10
0.15
β_IV

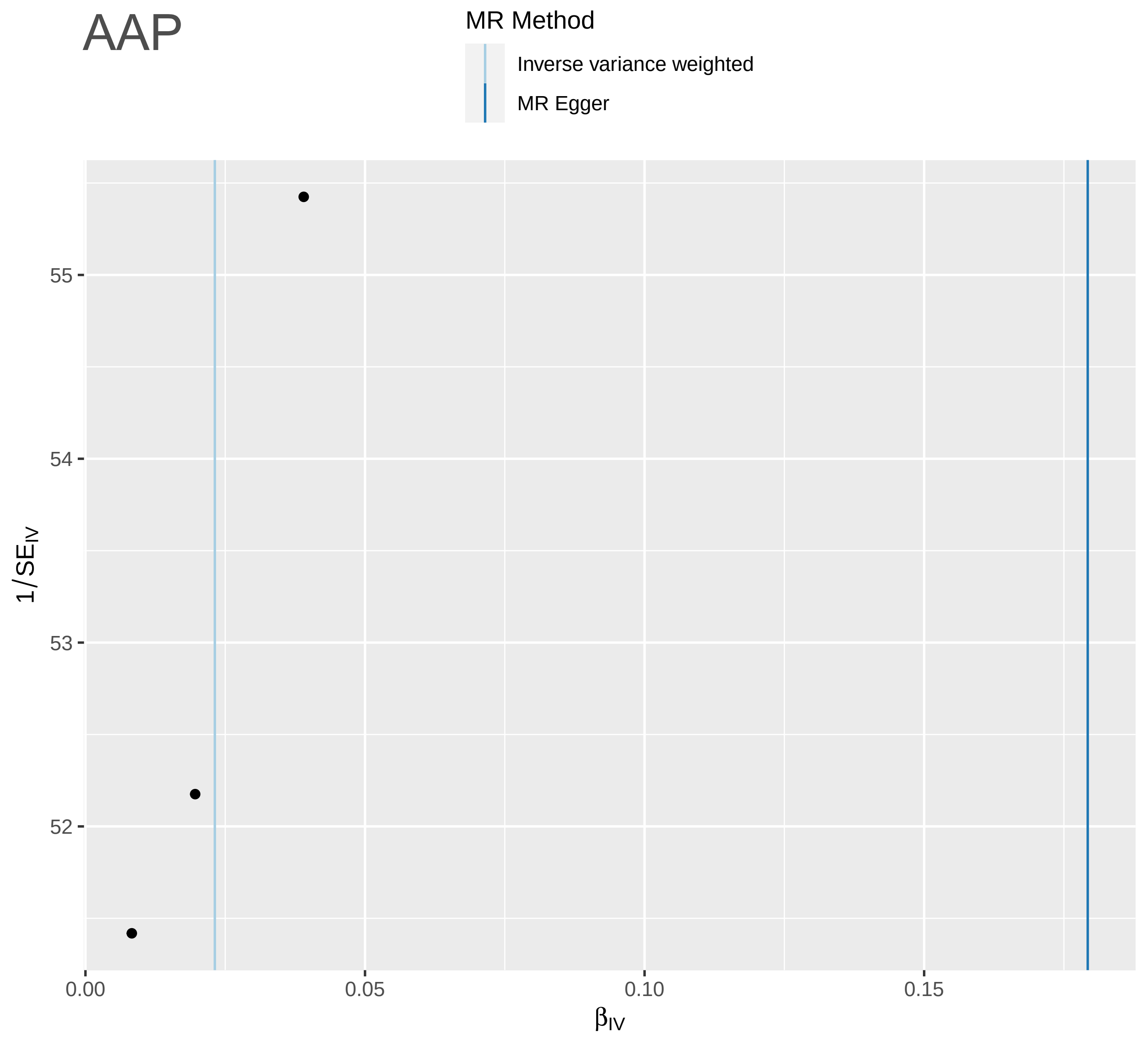
AAP
MR Method
Inverse variance weighted
MR Egger
55
54
53
52
$1/SE_{IV}$
0.00
0.05
0.10
0.15
β_{IV}

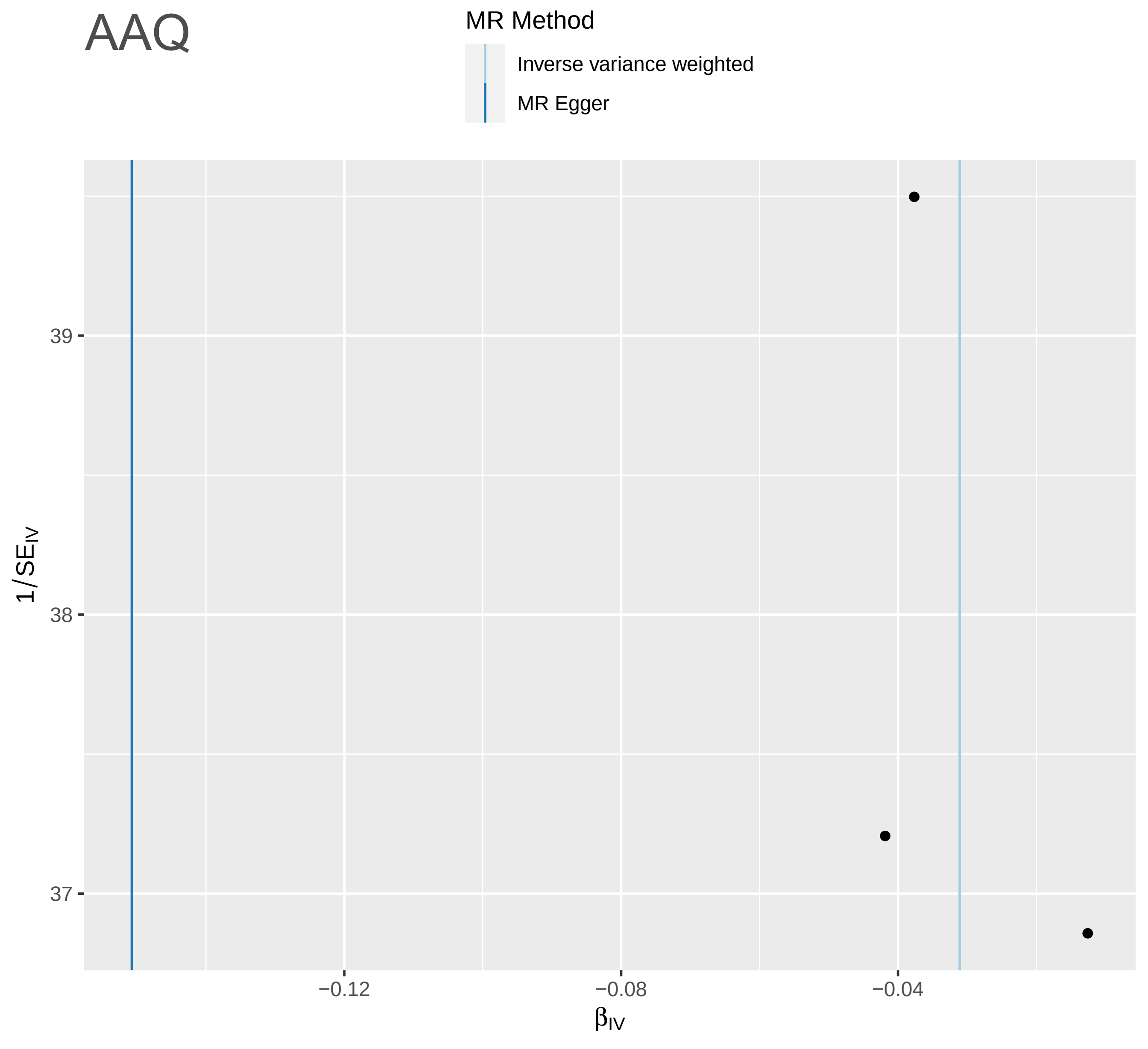
AAQ
MR Method
Inverse variance weighted
MR Egger
39
38
37
−0.12
−0.08
−0.04
$1/SE_{IV}$
β_{IV}

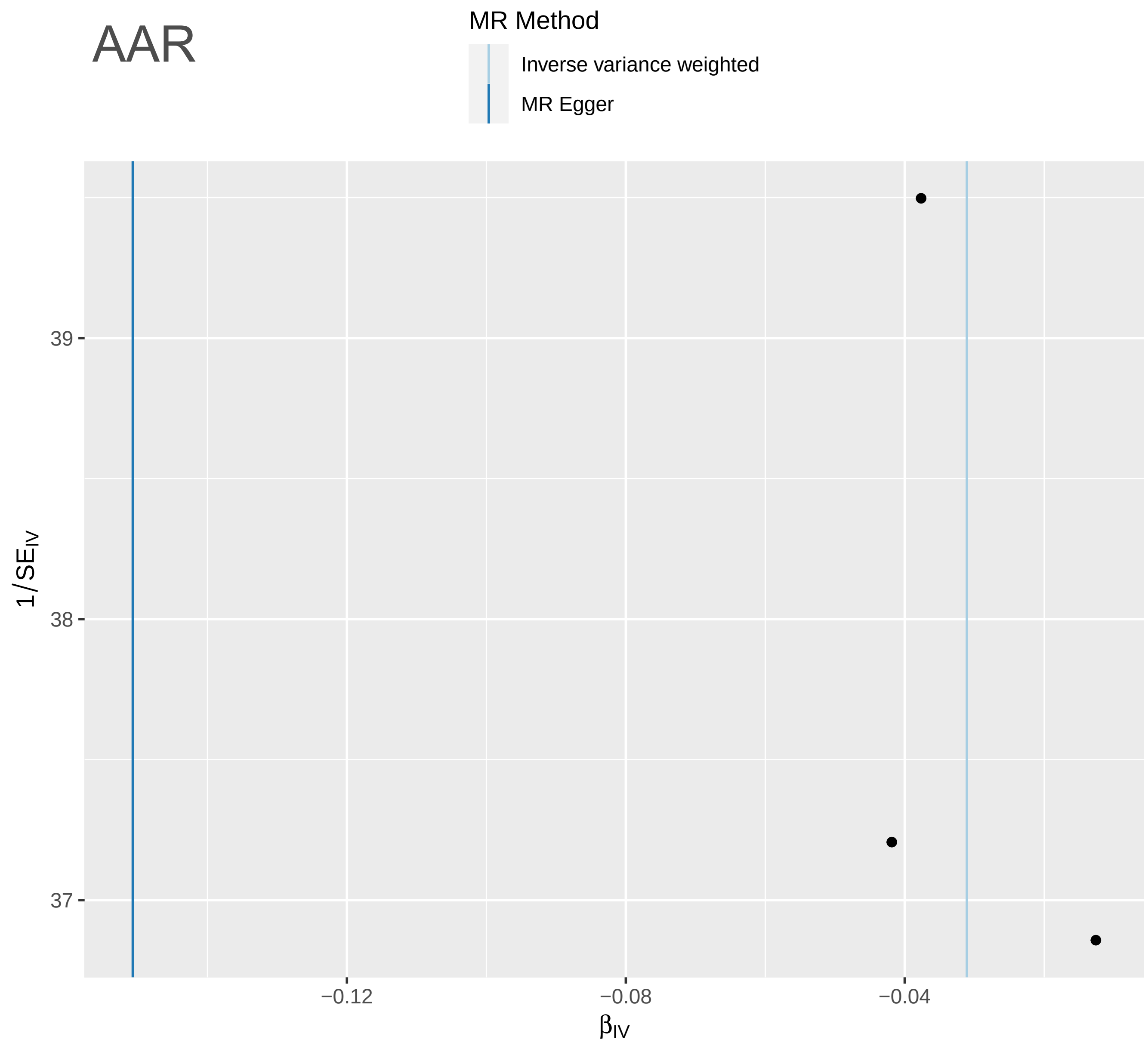
AAR
MR Method
Inverse variance weighted
MR Egger
39
38
37
1/SE_IV
−0.12
−0.08
−0.04
β_IV

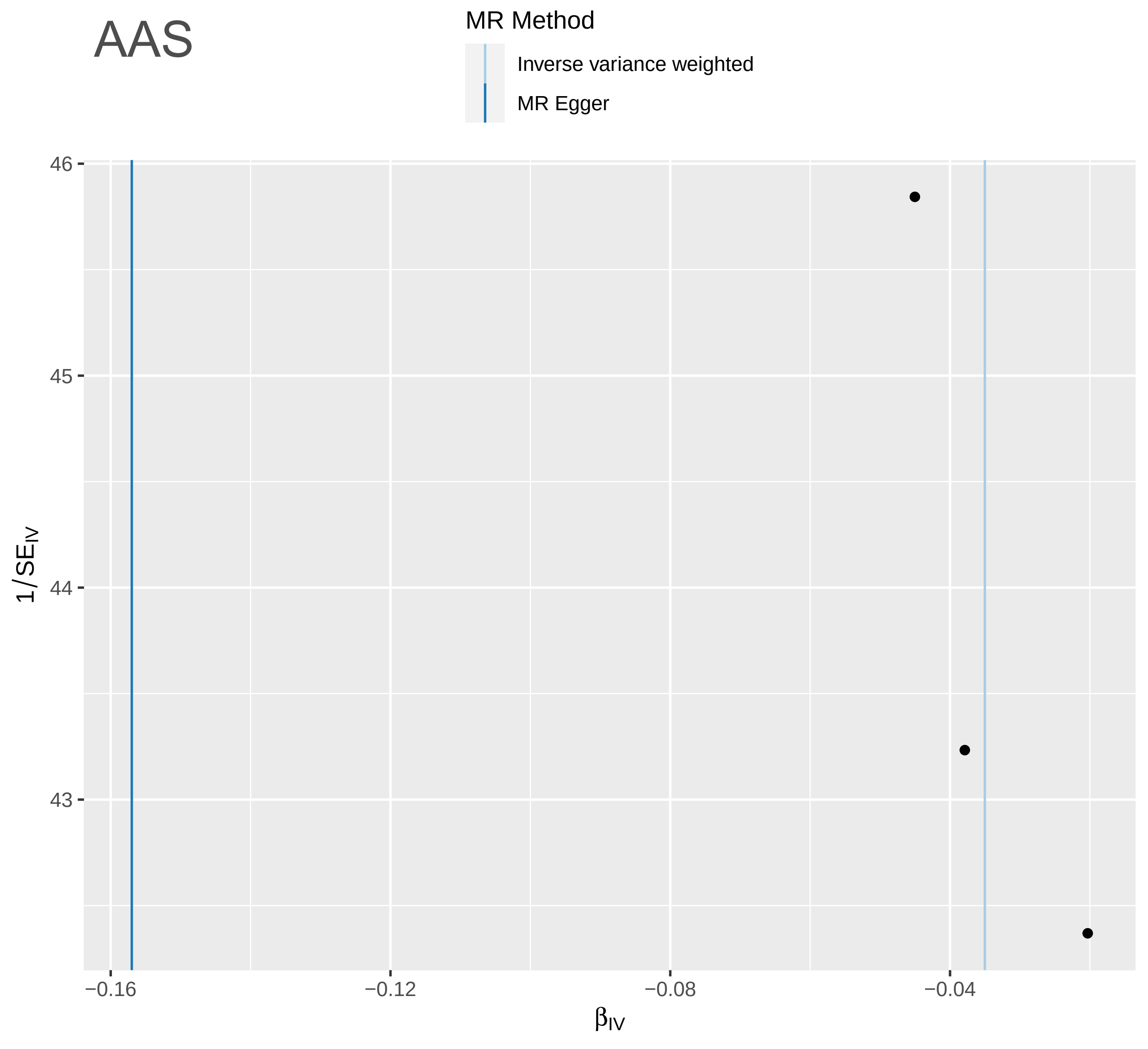
AAS
MR Method
Inverse variance weighted
MR Egger
46
45
44
43
$1/SE_{IV}$
−0.16
−0.12
−0.08
−0.04
β_{IV}

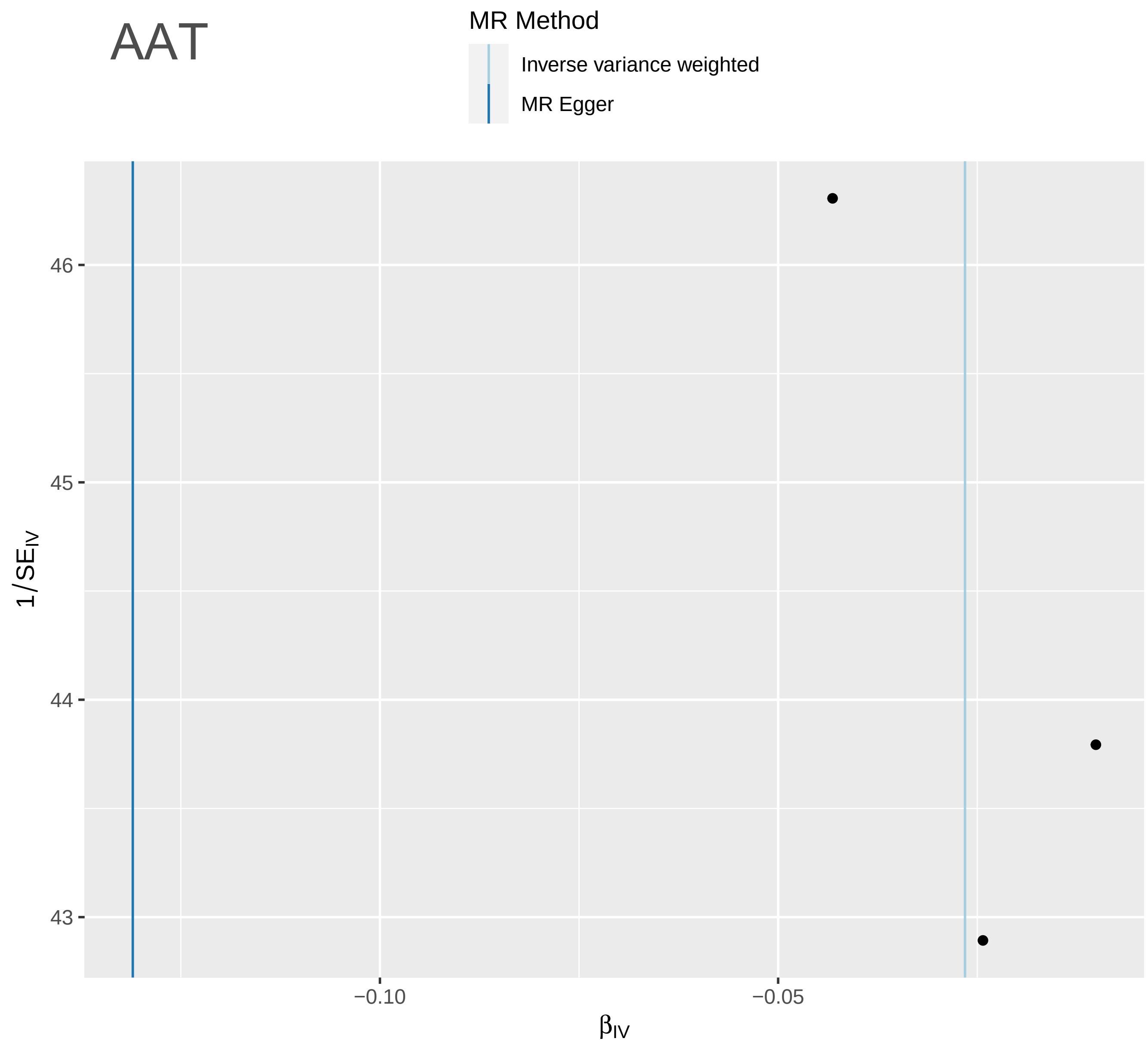

AAT
MR Method
Inverse variance weighted
MR Egger
46
45
44
43
$1/SE_{IV}$
−0.10
−0.05
β_{IV}

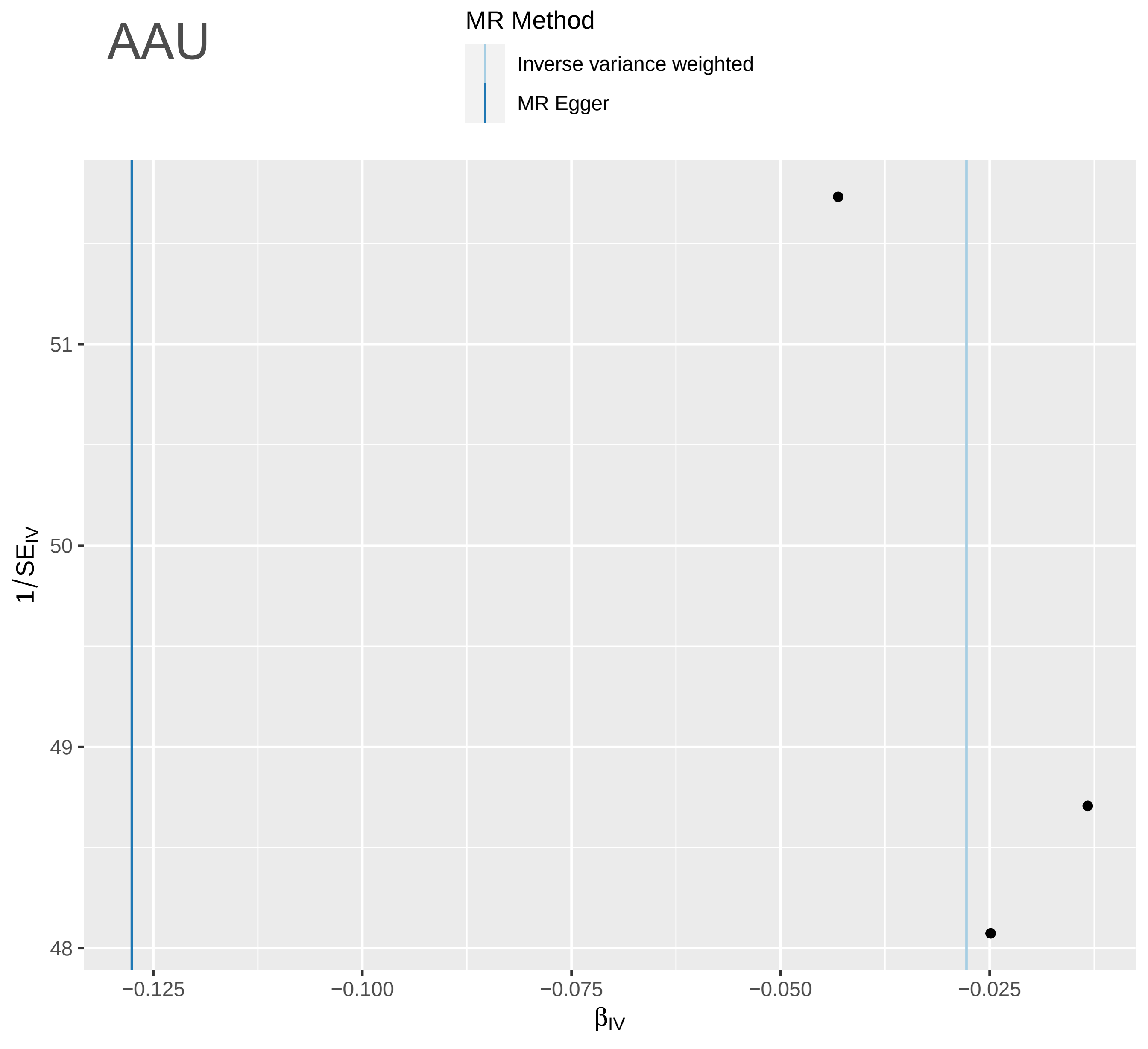

AAU
MR Method
Inverse variance weighted
MR Egger
51
50
49
48
1/SE$_{IV}$
−0.125
−0.100
−0.075
−0.050
−0.025
β$_{IV}$

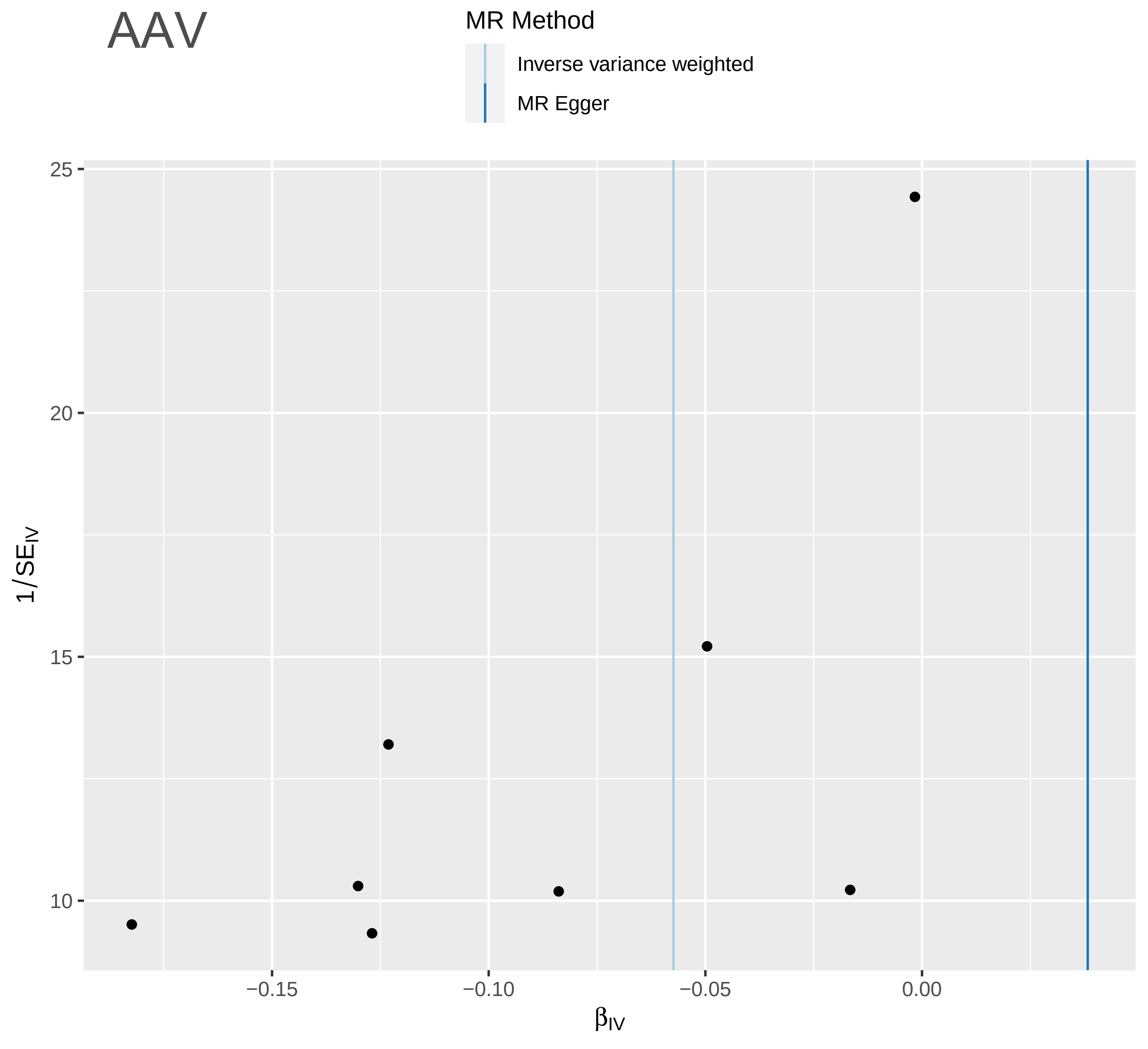
AAV
MR Method
Inverse variance weighted
MR Egger
25
20
15
10
1/SE_IV
−0.15
−0.10
−0.05
0.00
β_IV

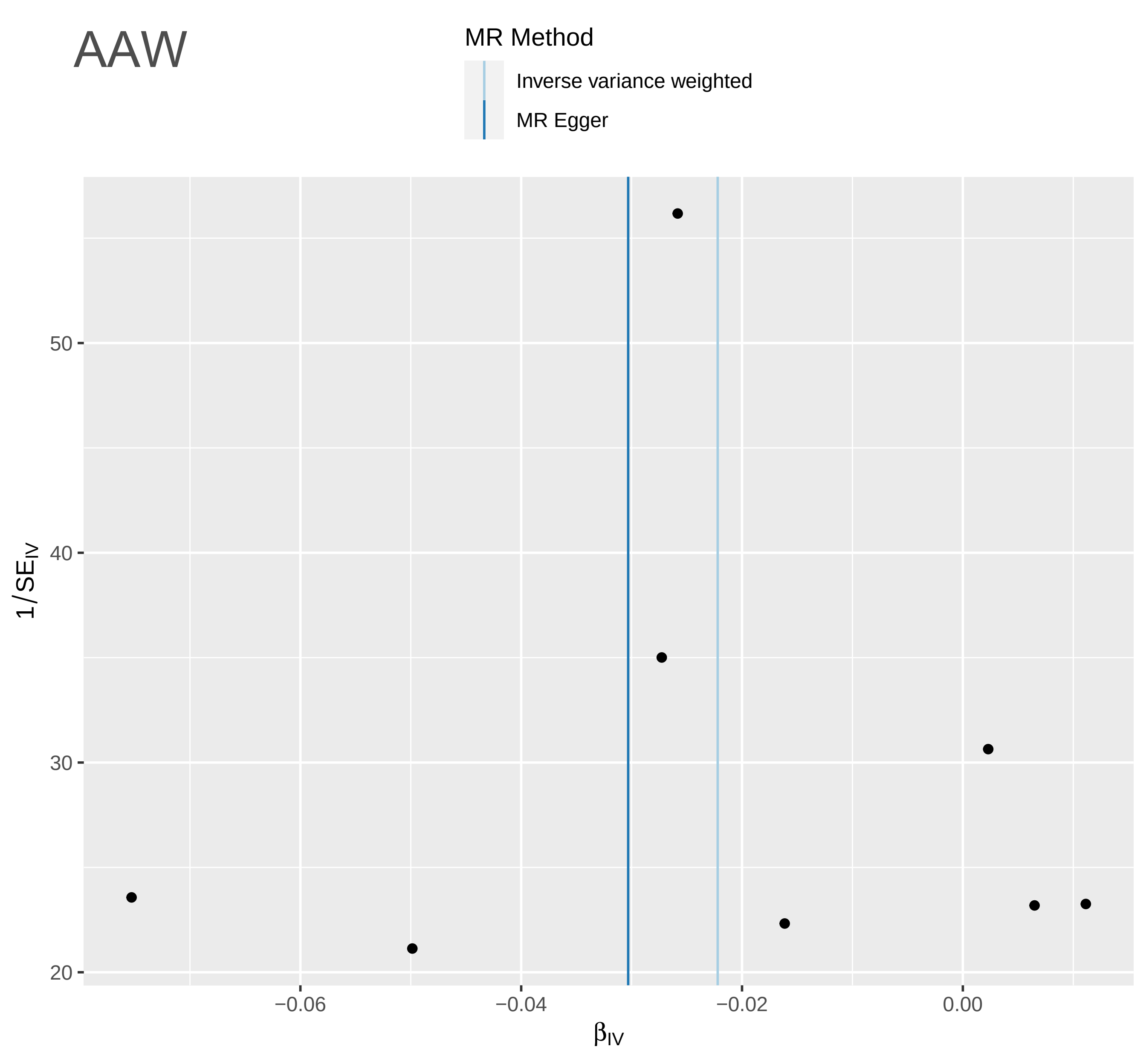
AAW
MR Method
Inverse variance weighted
MR Egger
50
40
30
20
1/SE_{IV}
−0.06
−0.04
−0.02
0.00
β_{IV}

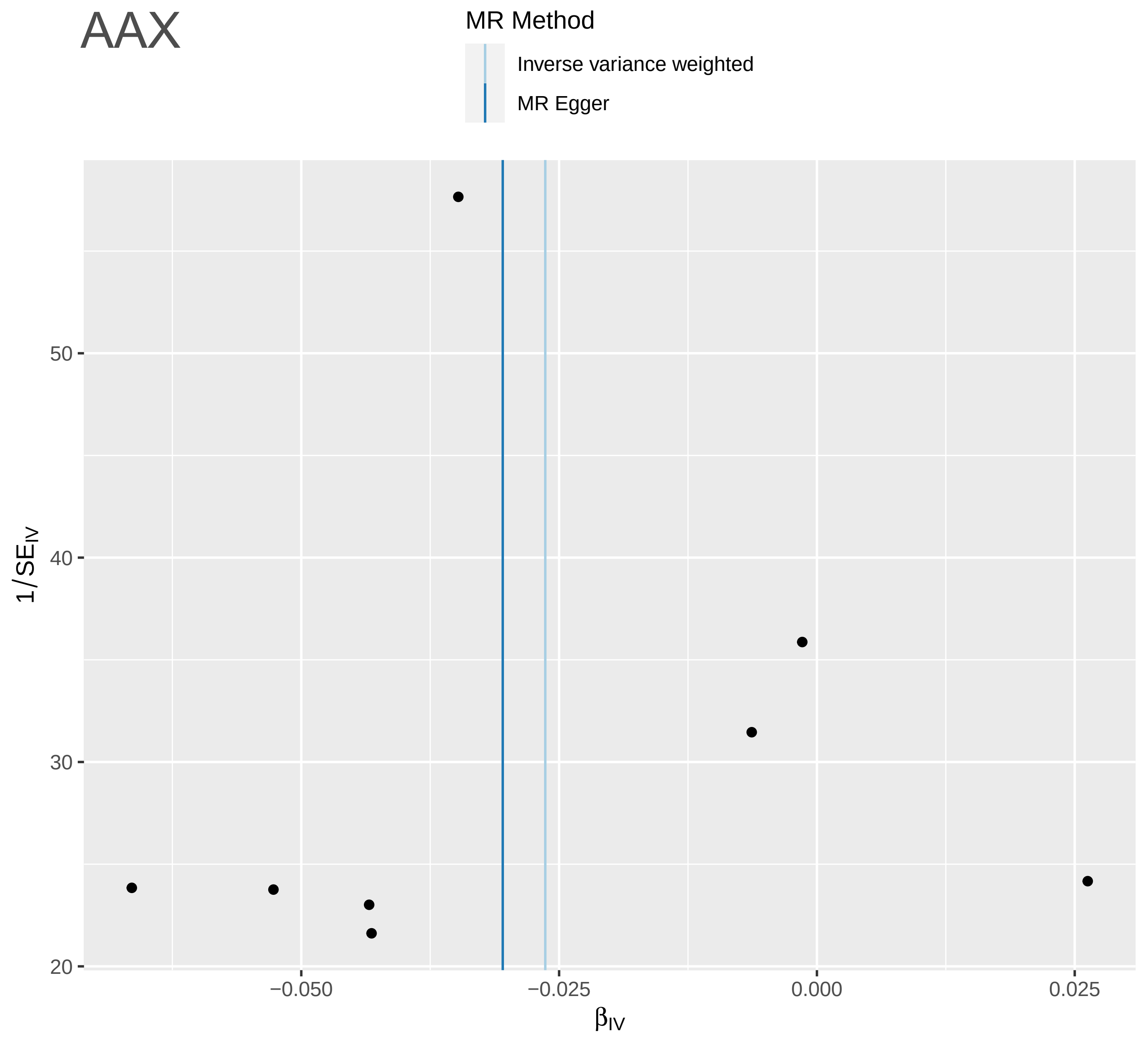
AAX
MR Method
Inverse variance weighted
MR Egger
1/SE_IV
50
40
30
20
−0.050
−0.025
0.000
0.025
β_IV

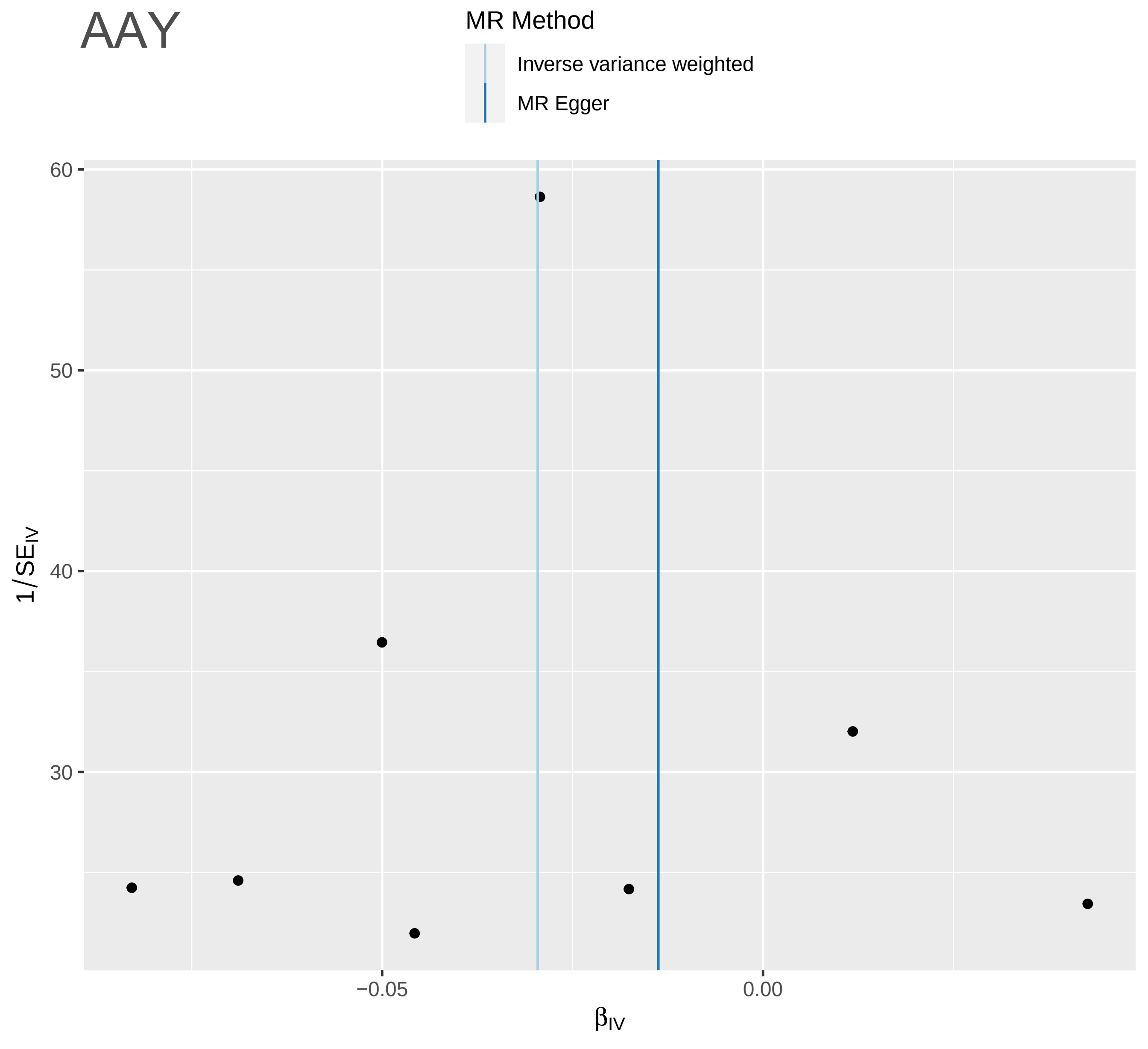

AAY
MR Method
Inverse variance weighted
MR Egger
60
50
40
30
1/SE_IV
−0.05
0.00
β_IV

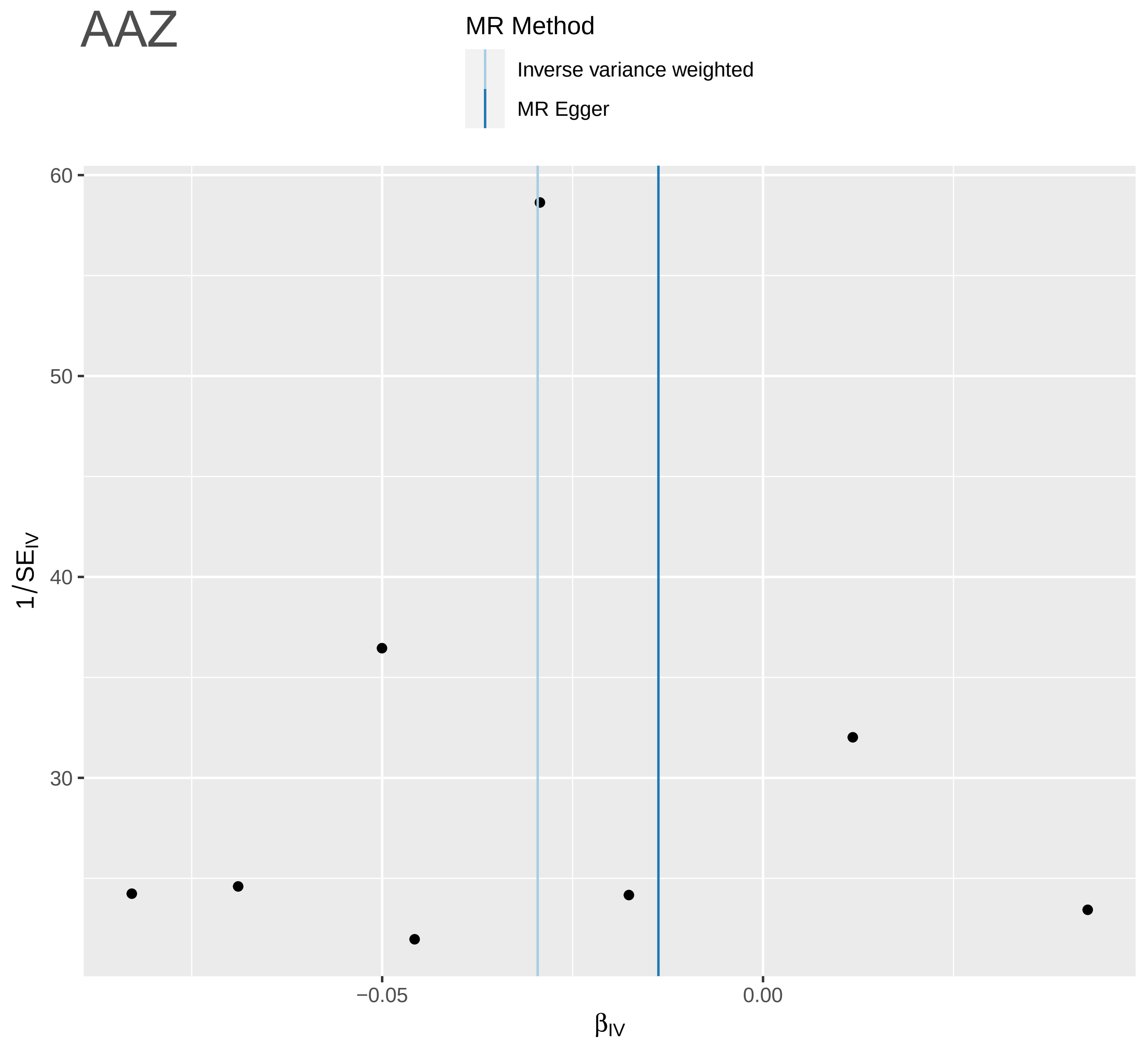
AAZ
MR Method
Inverse variance weighted
MR Egger
60
50
40
30
1/SE_IV
−0.05
0.00
β_IV

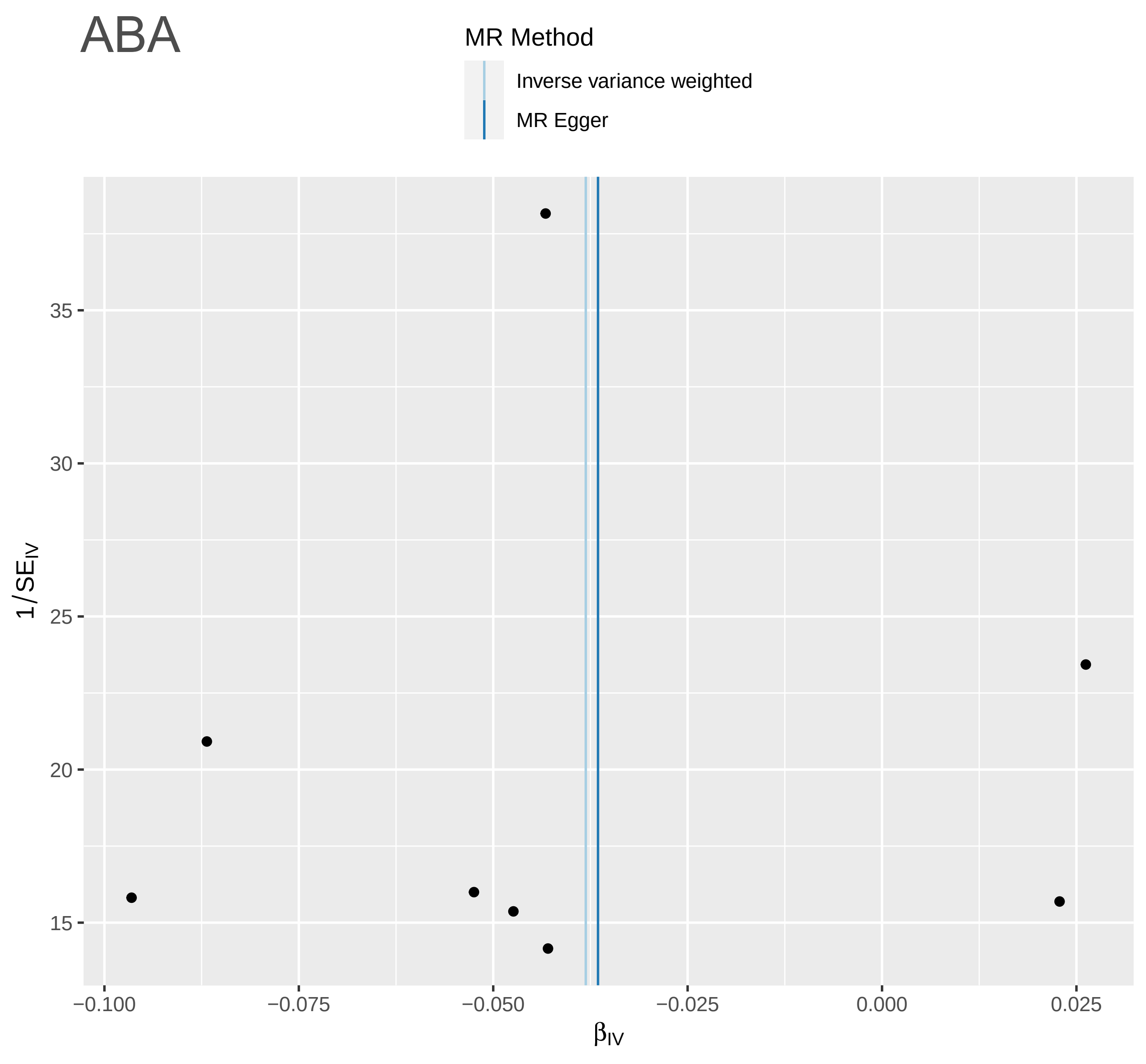

ABA
MR Method
Inverse variance weighted
MR Egger
35
30
25
20
15
1/SE_IV
−0.100
−0.075
−0.050
−0.025
0.000
0.025
β_IV

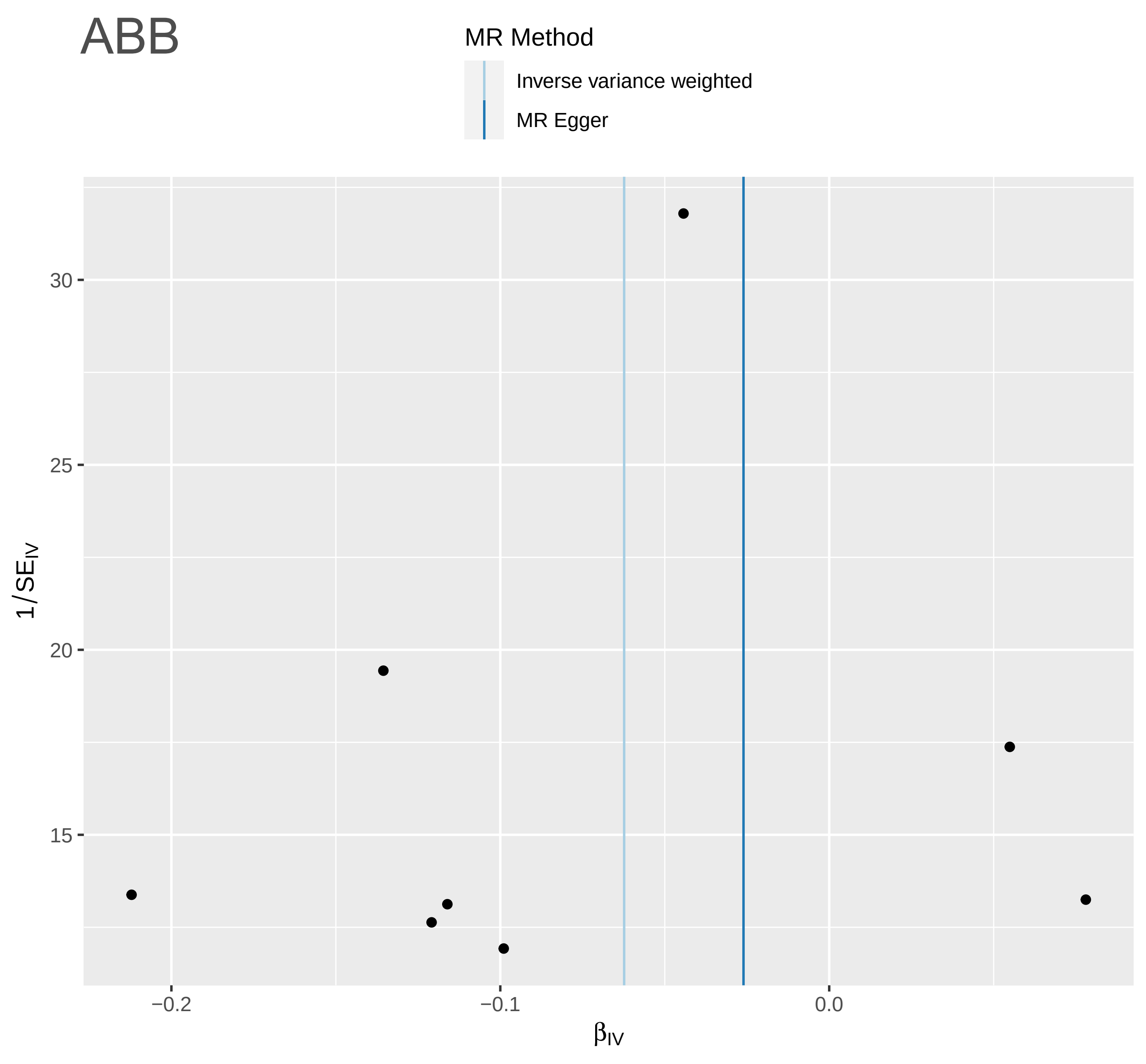

ABB
MR Method
Inverse variance weighted
MR Egger
30
25
20
15
1/SE_IV
−0.2
−0.1
0.0
β_IV

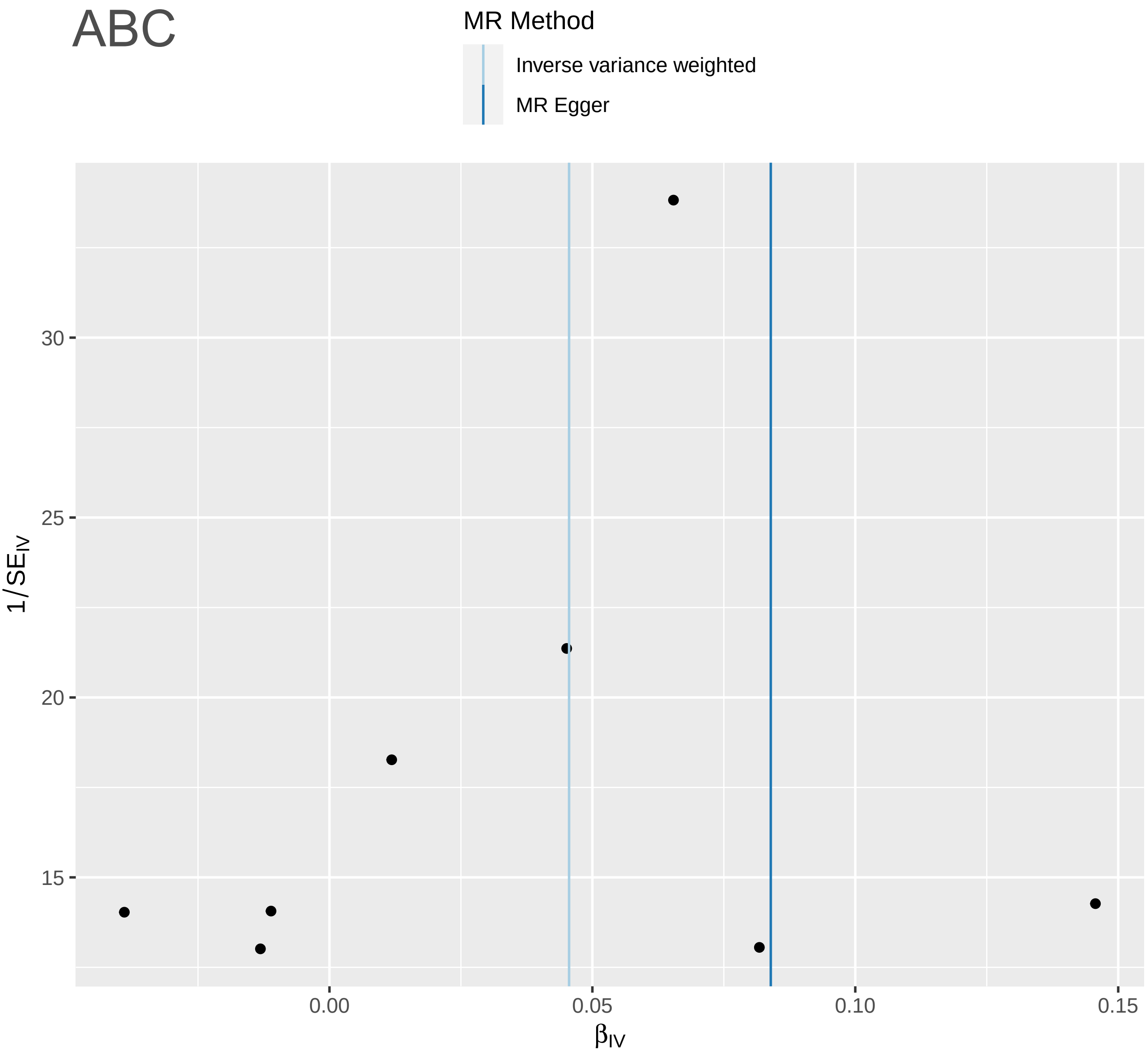
ABC
MR Method
Inverse variance weighted
MR Egger
30
25
20
15
0.00
0.05
0.10
0.15
β_{IV}
$1/SE_{IV}$

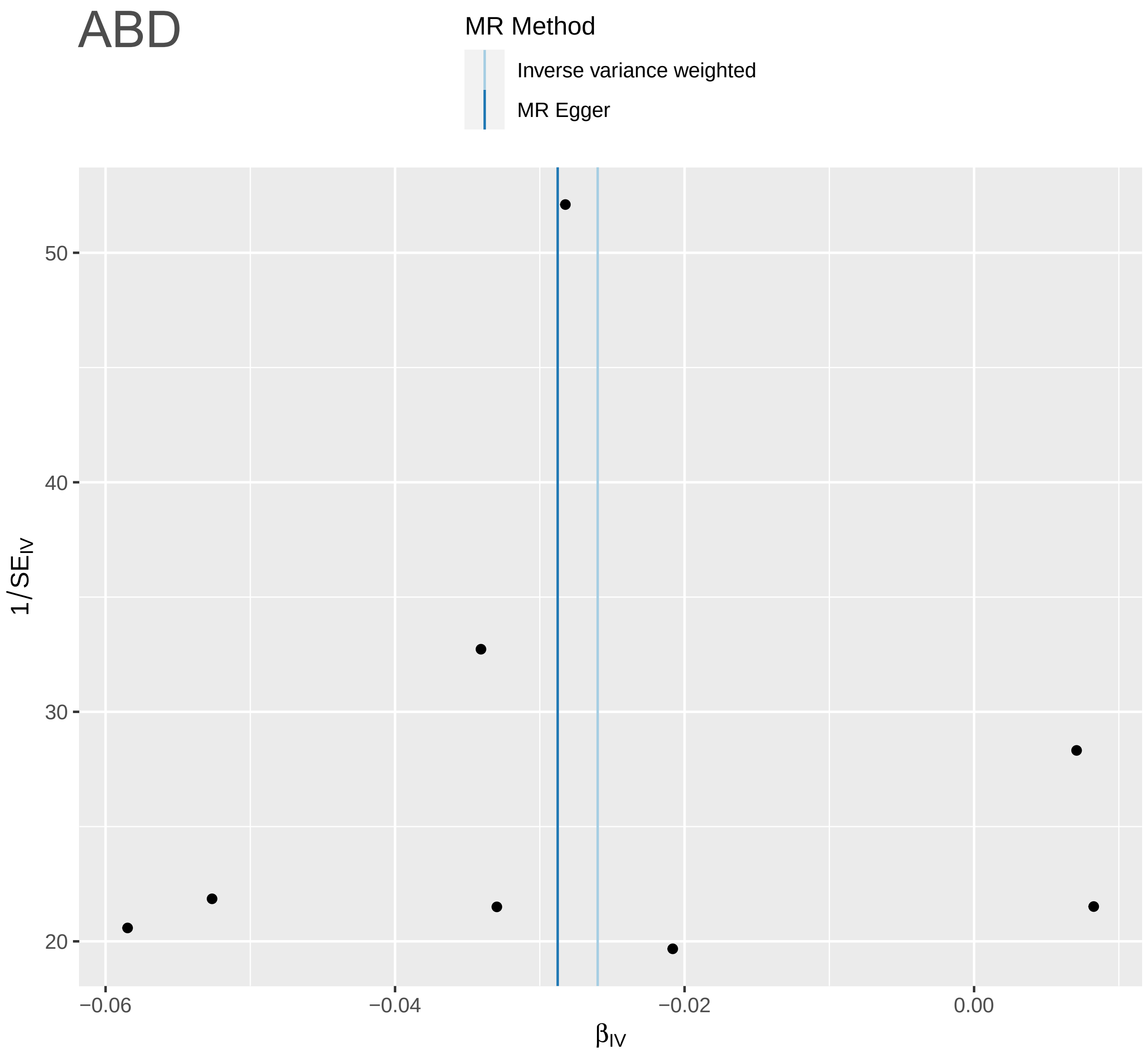
ABD
MR Method
Inverse variance weighted
MR Egger
50
40
30
20
$1/SE_{IV}$
−0.06
−0.04
−0.02
0.00
β_{IV}

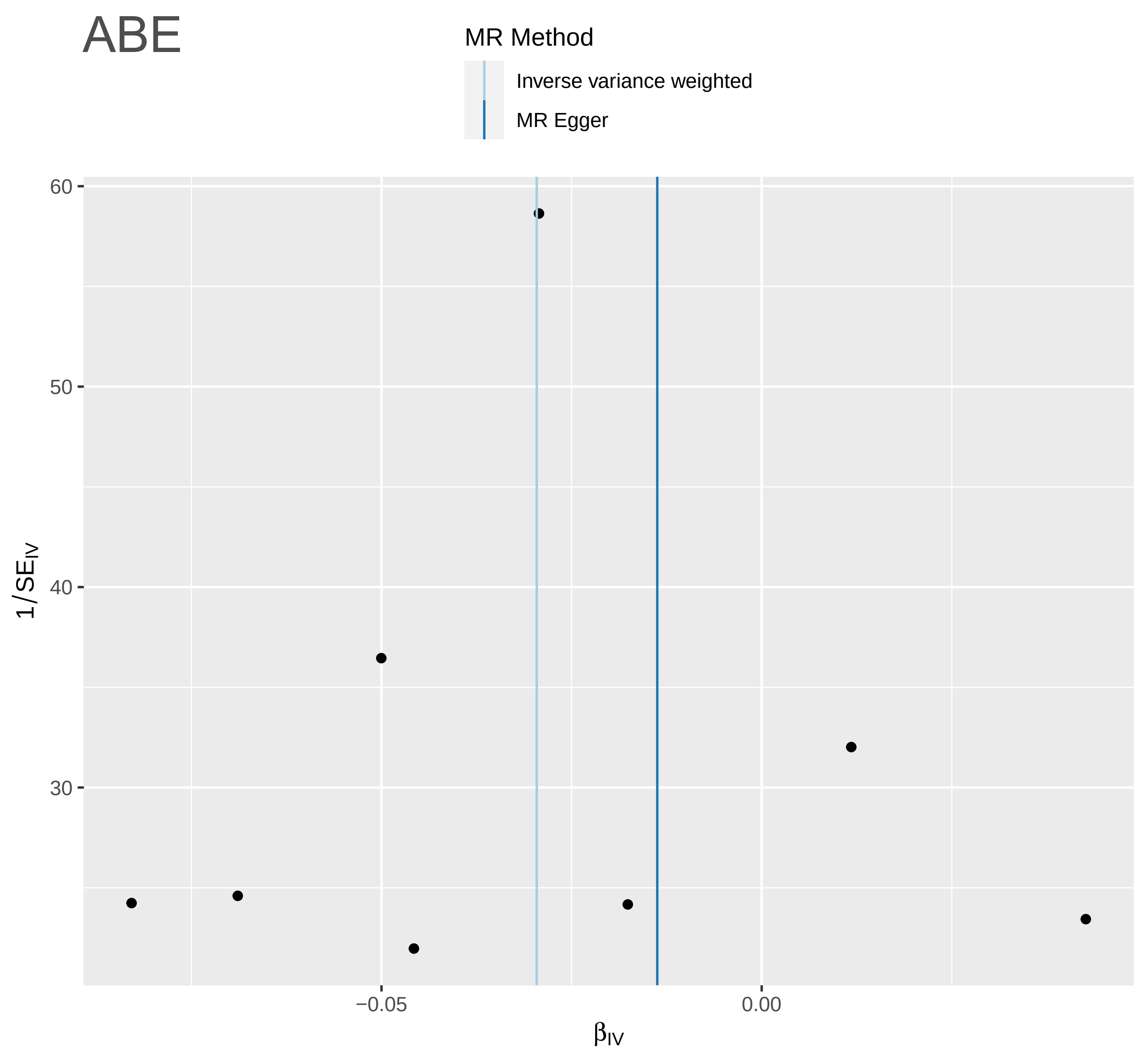

ABE
MR Method
Inverse variance weighted
MR Egger
60
50
40
30
1/SE_IV
−0.05
0.00
β_IV

Supplementary figure 6.Sensitivity analysis of the impact of hematologic malignancies on gut microbiota using MR. (funnel plot)

A.Funnel plot between lymphoid leukemia and order.Enterobacteriales

B.Funnel plot between lymphoid leukemia and genus Eubacteriumbrachygroup

C.Funnel plot between lymphoid leukemia and genus.LachnospiraceaeNC2004group

D.Funnel plot between lymphoid leukemia and genus.Anaerofilum.

E.Funnel plot between lymphoid leukemia and genus.Butyrivibrio

F.Funnel plot between lymphoid leukemia and genus.Escherichia.Shigella

G.Funnel plot between lymphoid leukemia and family.Enterobacteriaceae

H.Funnel plot between lymphoid leukemia and genus.Fusicatenibacter

I.Funnel plot between lymphoid leukemia and genus.LachnospiraceaeUCG010

J.Funnel plot between myeloid leukemia and family.Bifidobacteriaceae

K.Funnel plot between myeloid leukemia and class.Actinobacteria

L.Funnel plot between myeloid leukemia and class.Clostridia

M.Funnel plot between myeloid leukemia and phylum.Bacteroidetes

N.Funnel plot between myeloid leukemia and genus..Ruminococcusgauvreauiigroup

O.Funnel plot between myeloid leukemia and order.Clostridiales

P.Funnel plot between myeloid leukemia and phylum.Actinobacteria.

Q.Funnel plot between myeloid leukemia and order.Bacteroidales

R.Funnel plot between myeloid leukemia and class.Bacteroidia

S.Funnel plot between myeloid leukemia and genus.Bifidobacterium

T.Funnel plot between myeloid leukemia and family.Lachnospiraceae

U.Funnel plot between myeloid leukemia and genus.Collinsella

V.Funnel plot between myeloid leukemia and genus.Blautia

W.Funnel plot between myeloid leukemia and genus.Fusicatenibacter

X.Funnel plot between myeloid leukemia and order.Bifidobacteriales

Y.Funnel plot between myeloid leukemia and genus..Ruminococcusgnavusgroup

Z.Funnel plot between myeloid leukemia and genus..Eubacteriumhalliigroup

AA..Funnel plot between myeloid leukemia and genus.Slackia.

AB.Funnel plot between myeloid leukemia and phylum.Firmicutes

AC.Funnel plot between Hodgkin lymphoma and phylum.Verrucomicrobia

AD.Funnel plot between Hodgkin lymphoma and family.ClostridialesvadinBB60group

AE.Funnel plot between Hodgkin lymphoma and genus.RuminococcaceaeUCG010

AF.Funnel plot between Hodgkin lymphoma and genus.Odoribacter

AG.Funnel plot between Hodgkin lymphoma and genus.RuminococcaceaeUCG005

AH.Funnel plot between Hodgkin lymphoma and genus.RuminococcaceaeUCG013

AI. Funnel plot between Hodgkin lymphoma and genus.Ruminiclostridium9

AJ.Funnel plot between Hodgkin lymphoma and genus.RuminococcaceaeNK4A214group

AK.Funnel plot between Hodgkin lymphoma and genus..Eubacteriumventriosumgroup

AL.Funnel plot between Hodgkin lymphoma and genus.RuminococcaceaeUCG003

AM.Funnel plot between malignant plasma cell tumor and family.Defluviitaleaceae

AN. Funnel plot between malignant plasma cell tumor and genus.DefluviitaleaceaeUCG011

AO.Funnel plot between malignant plasma cell tumor and genus..Eubacteriumrectalegroup

AP.Funnel plot between malignant plasma cell tumor and genus.CandidatusSoleaferrea

AQ. Funnel plot between follicular lymphoma and genus.Allisonella

AR.Funnel plot between follicular lymphoma and genus.RikenellaceaeRC9gutgroup

AS.Funnel plot between follicular lymphoma and genus.Faecalibacterium

AT.Funnel plot between follicular lymphoma and family.Clostridiaceae

AU.Funnel plot between follicular lymphoma and genus.DefluviitaleaceaeUCG011.

AV.Funnel plot between follicular lymphoma and genus.Clostridiumsensustricto

AW.Funnel plot between follicular lymphoma and family.Defluviitaleaceae

AX.Funnel plot between diffuse large B-cell lymphomaand family.Veillonellaceae

AY.Funnel plot between diffuse large B-cell lymphomaand genus.Fusicatenibacter

AZ.Funnel plot between diffuse large B-cell lymphomaand genus.Eggerthella

AAA.Funnel plot between diffuse large B-cell lymphomaand genus.Erysipelatoclostridium

AAB.Funnel plot between diffuse large B-cell lymphomaand genus.Blautia

AAC.Funnel plot between diffuse large B-cell lymphomaand genus.RuminococcaceaeUCG009

AAD.Funnel plot between mature T/NK-cell lymphomas and order.Verrucomicrobiales

AAE.Funnel plot between mature T/NK-cell lymphomas and phylum.Tenericutes

AAF.Funnel plot between mature T/NK-cell lymphomas and genus.Lachnoclostridium

AAG.Funnel plot between mature T/NK-cell lymphomas and phylum.Bacteroidetes

AAH.Funnel plot between mature T/NK-cell lymphomas and class.Verrucomicrobiae

AAI.Funnel plot between mature T/NK-cell lymphomas and genus..Eubacteriumrectalegroup

AAJ.Funnel plot between mature T/NK-cell lymphomas and family.Verrucomicrobiaceae

AAK.Funnel plot between mature T/NK-cell lymphomas and order.Bacteroidales

AAL. Funnel plot between mature T/NK-cell lymphomas and genus.Akkermansia

AAM. Funnel plot between mature T/NK-cell lymphomas and genus.RuminococcaceaeUCG005

AAN.Funnel plot between mature T/NK-cell lymphomas and class.Mollicutes.

AAO. Funnel plot between mature T/NK-cell lymphomas and class.Bacteroidia

AAP.Funnel plot between mature T/NK-cell lymphomas and family.Lachnospiraceae

AAQ.Funnel plot between mature T/NK-cell lymphomas and order.Pasteurellales

AAR. Funnel plot between mature T/NK-cell lymphomas and family.Pasteurellaceae.

AAS. Funnel plot between mature T/NK-cell lymphomas and phylum.Verrucomicrobia

AAT.Funnel plot between mature T/NK-cell lymphomas and genus.RuminococcaceaeUCG010

AAU.Funnel plot between mature T/NK-cell lymphomas and family.Veillonellaceae.

AAV.Funnel plot between myeloproliferative neoplasms and genus.Sellimonas

AAW.Funnel plot between myeloproliferative neoplasms and genus.Coprococcus1

AAX.Funnel plot between myeloproliferative neoplasms and genus.Dorea

AAY.Funnel plot between myeloproliferative neoplasms and family.Erysipelotrichaceae

AAZ.Funnel plot between myeloproliferative neoplasms and class.Erysipelotrichia

ABA.Funnel plot between myeloproliferative neoplasms and genus..Eubacteriumruminantiumgroup

ABB.Funnel plot between myeloproliferative neoplasms and genus.Tyzzerella3

ABC.Funnel plot between myeloproliferative neoplasms and genus.Slackia

ABD.Funnel plot between myeloproliferative neoplasms and genus.Collinsella

ABE.Funnel plot between myeloproliferative neoplasms and order.Erysipelotrichales

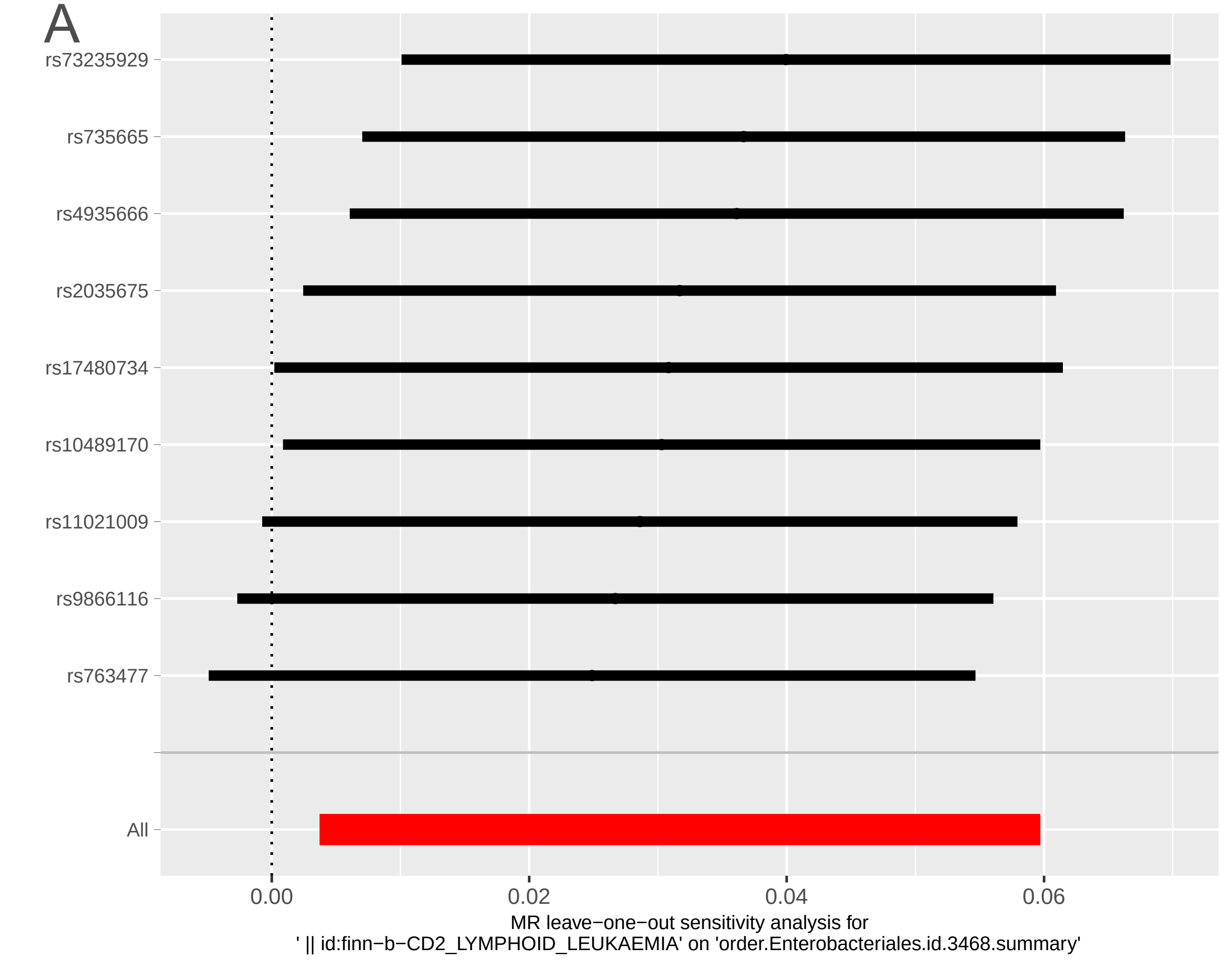

A
rs73235929
rs735665
rs4935666
rs2035675
rs17480734
rs10489170
rs11021009
rs9866116
rs763477
All
0.00
0.02
0.04
0.06
MR leave−one−out sensitivity analysis for
' || id:finn−b−CD2_LYMPHOID_LEUKAEMIA' on 'order.Enterobacteriales.id.3468.summary'

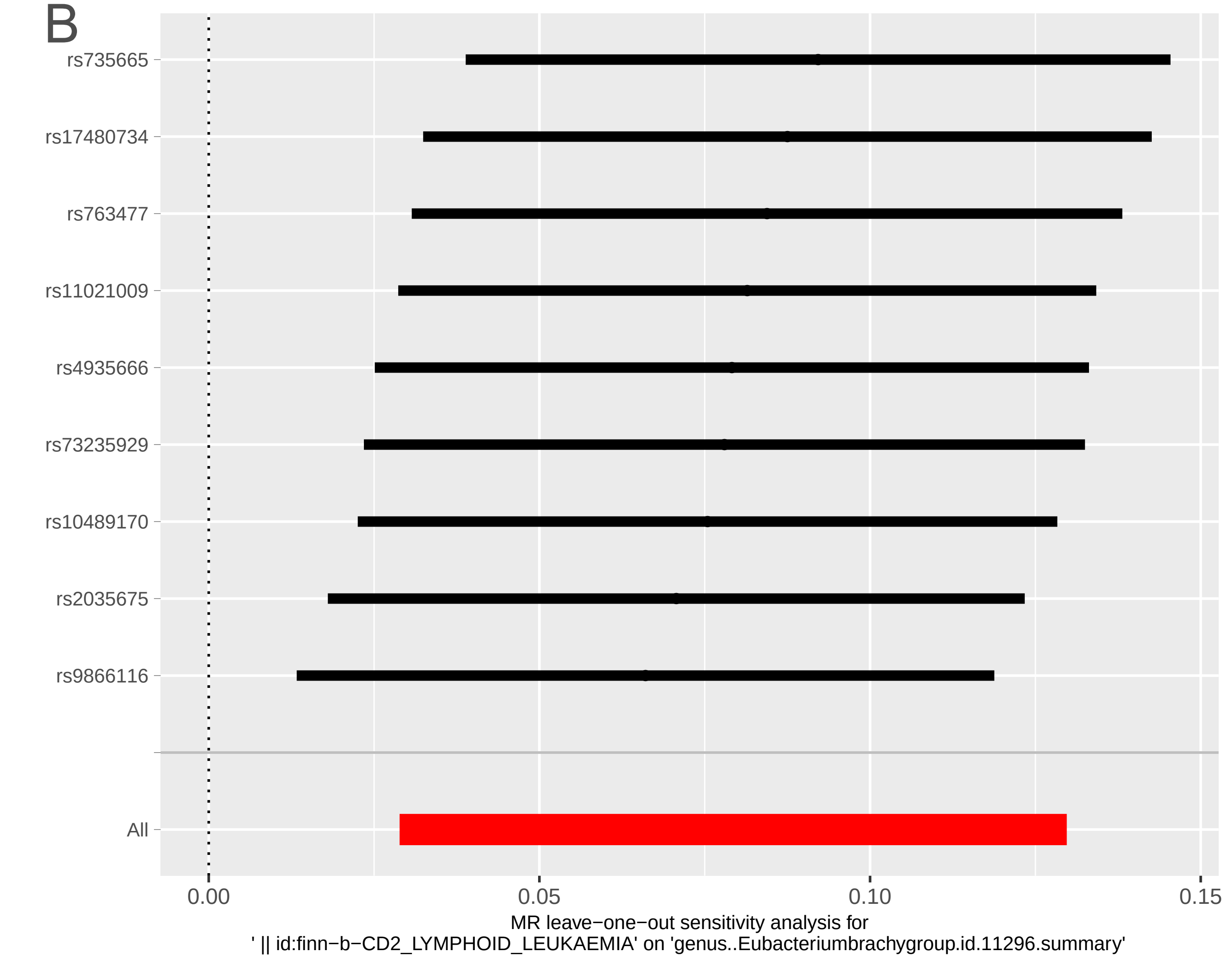

B
rs735665
rs17480734
rs763477
rs11021009
rs4935666
rs73235929
rs10489170
rs2035675
rs9866116
All
0.00
0.05
0.10
0.15
MR leave−one−out sensitivity analysis for
' || id:finn−b−CD2_LYMPHOID_LEUKAEMIA' on 'genus..Eubacteriumbrachygroup.id.11296.summary'

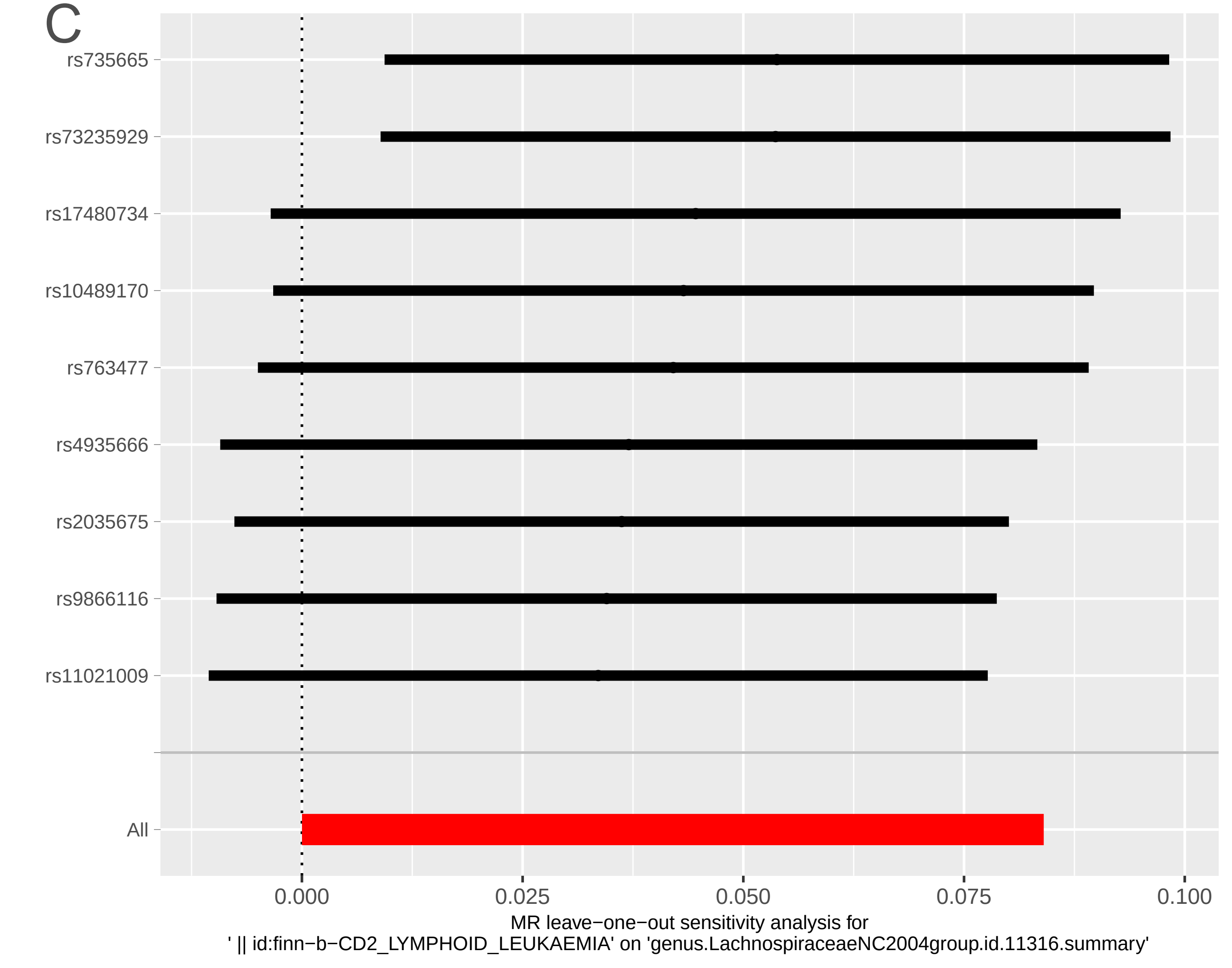
C
rs735665
rs73235929
rs17480734
rs10489170
rs763477
rs4935666
rs2035675
rs9866116
rs11021009
All
0.000
0.025
0.050
0.075
0.100
MR leave−one−out sensitivity analysis for
' || id:finn−b−CD2_LYMPHOID_LEUKAEMIA' on 'genus.LachnospiraceaeNC2004group.id.11316.summary'

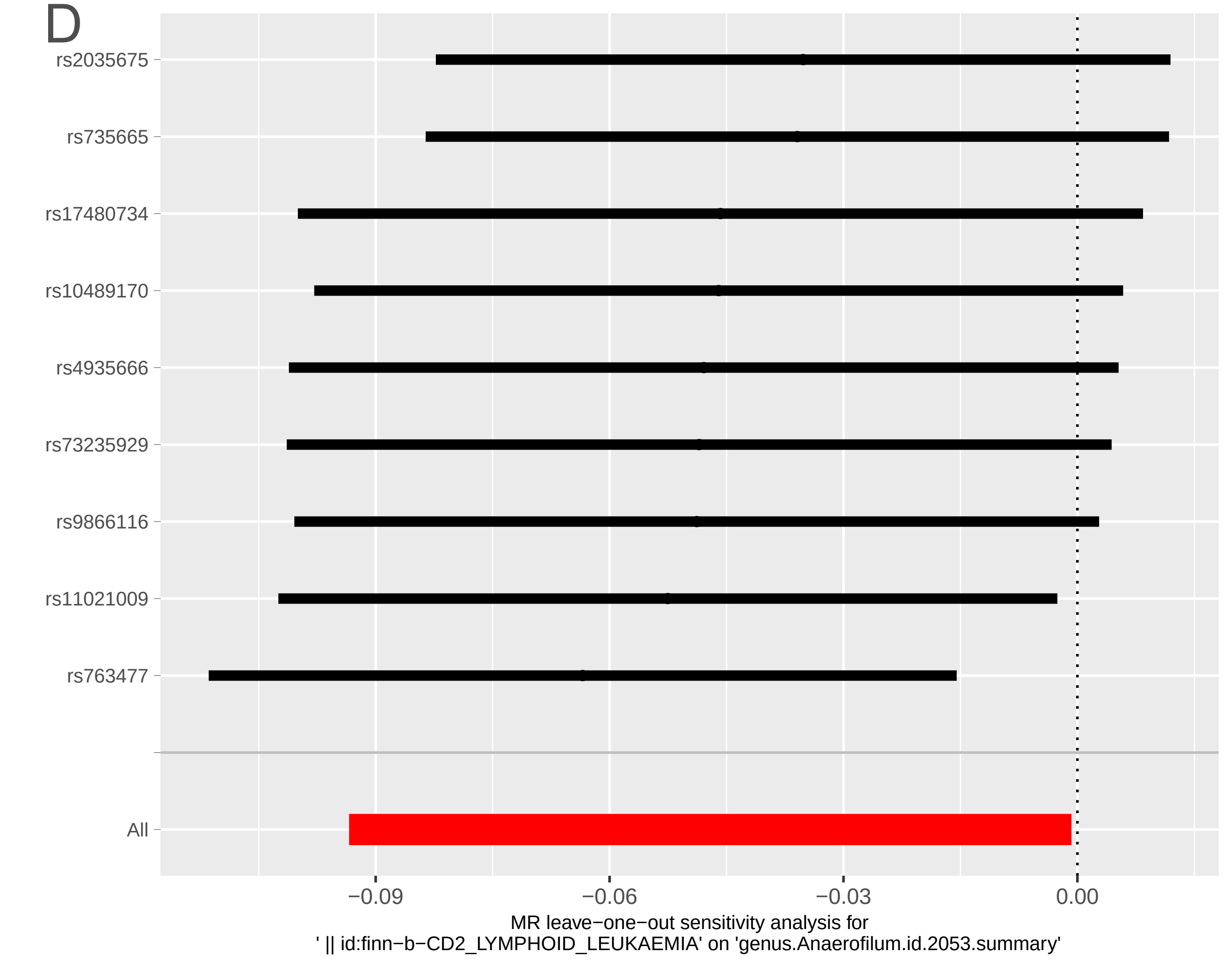
D
rs2035675
rs735665
rs17480734
rs10489170
rs4935666
rs73235929
rs9866116
rs11021009
rs763477
All
−0.09
−0.06
−0.03
0.00
MR leave−one−out sensitivity analysis for
' || id:finn−b−CD2_LYMPHOID_LEUKAEMIA' on 'genus.Anaerofilum.id.2053.summary'

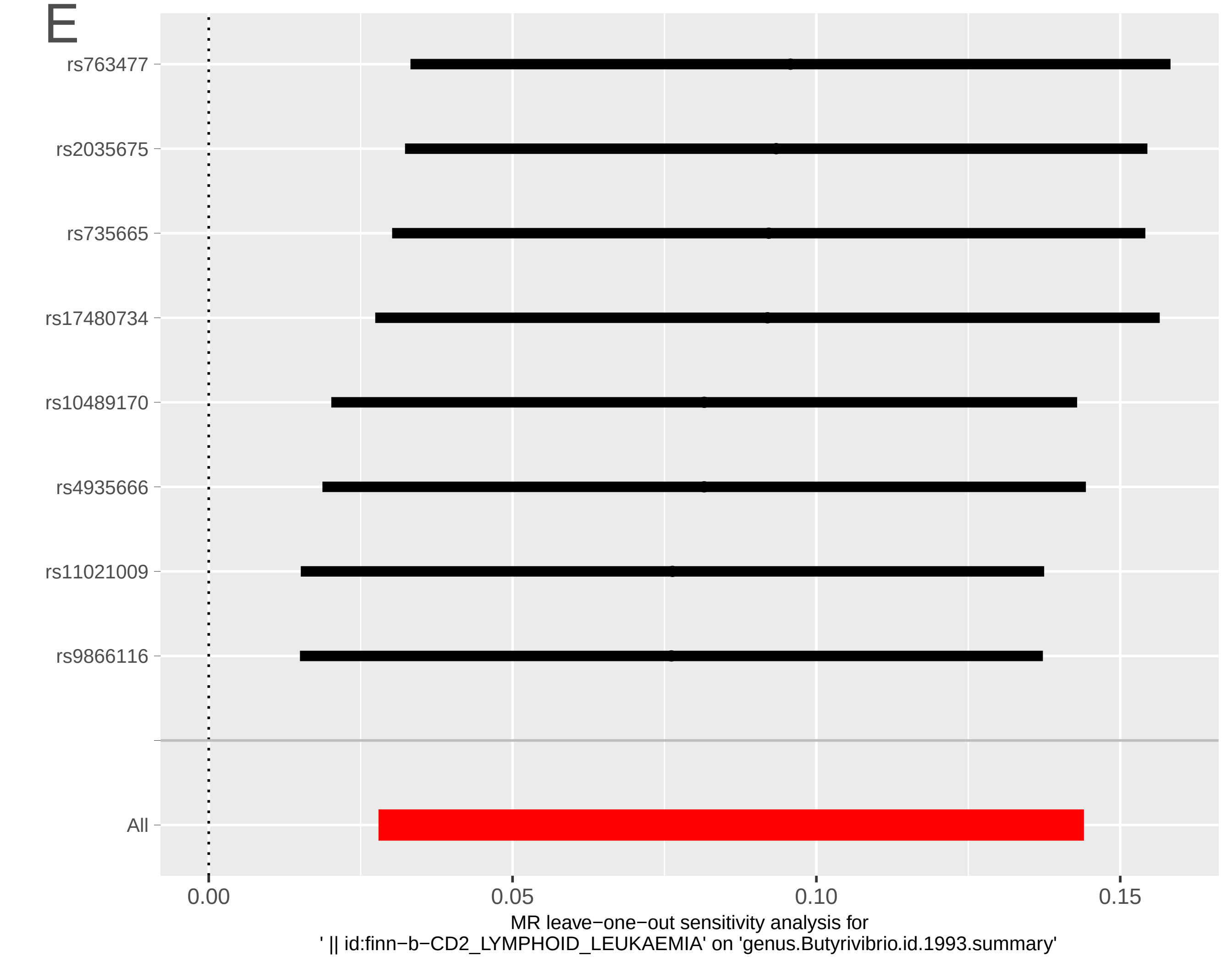
E
rs763477
rs2035675
rs735665
rs17480734
rs10489170
rs4935666
rs11021009
rs9866116
All
0.00
0.05
0.10
0.15
MR leave−one−out sensitivity analysis for
' || id:finn−b−CD2_LYMPHOID_LEUKAEMIA' on 'genus.Butyrivibrio.id.1993.summary'

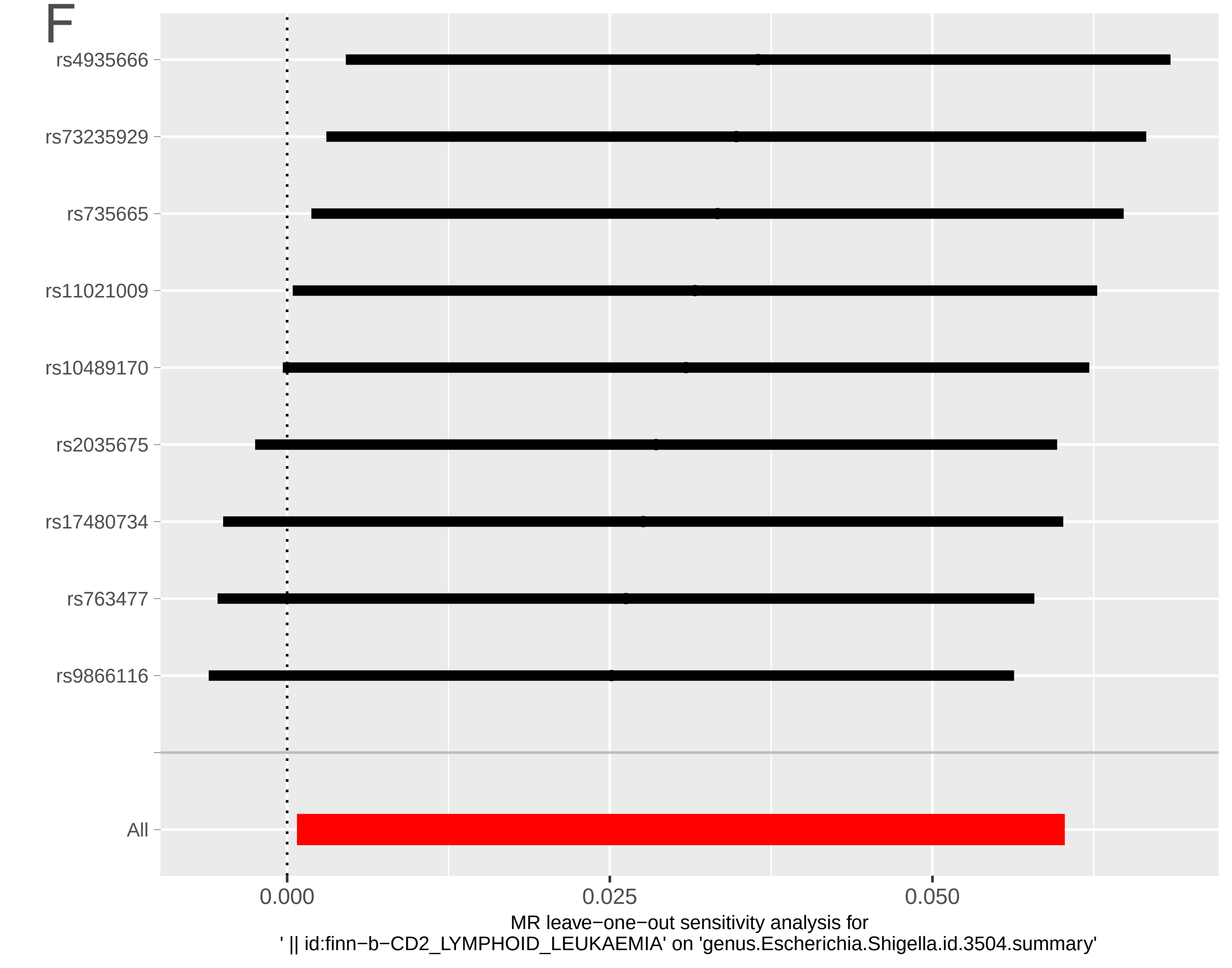
F
rs4935666
rs73235929
rs735665
rs11021009
rs10489170
rs2035675
rs17480734
rs763477
rs9866116
All
0.000
0.025
0.050
MR leave−one−out sensitivity analysis for
' || id:finn−b−CD2_LYMPHOID_LEUKAEMIA' on 'genus.Escherichia.Shigella.id.3504.summary'

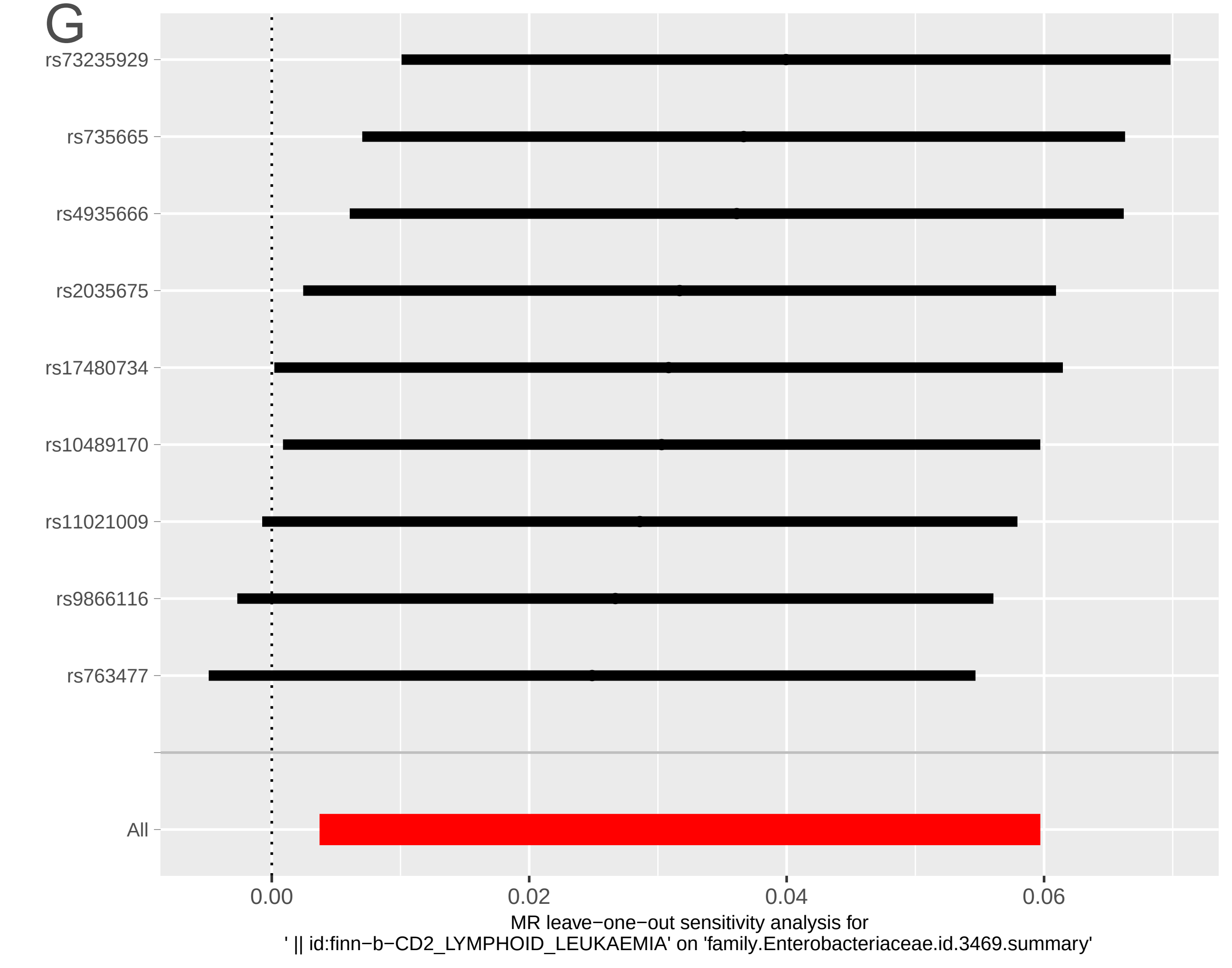
G
rs73235929
rs735665
rs4935666
rs2035675
rs17480734
rs10489170
rs11021009
rs9866116
rs763477
All
0.00
0.02
0.04
0.06
MR leave−one−out sensitivity analysis for
' || id:finn−b−CD2_LYMPHOID_LEUKAEMIA' on 'family.Enterobacteriaceae.id.3469.summary'

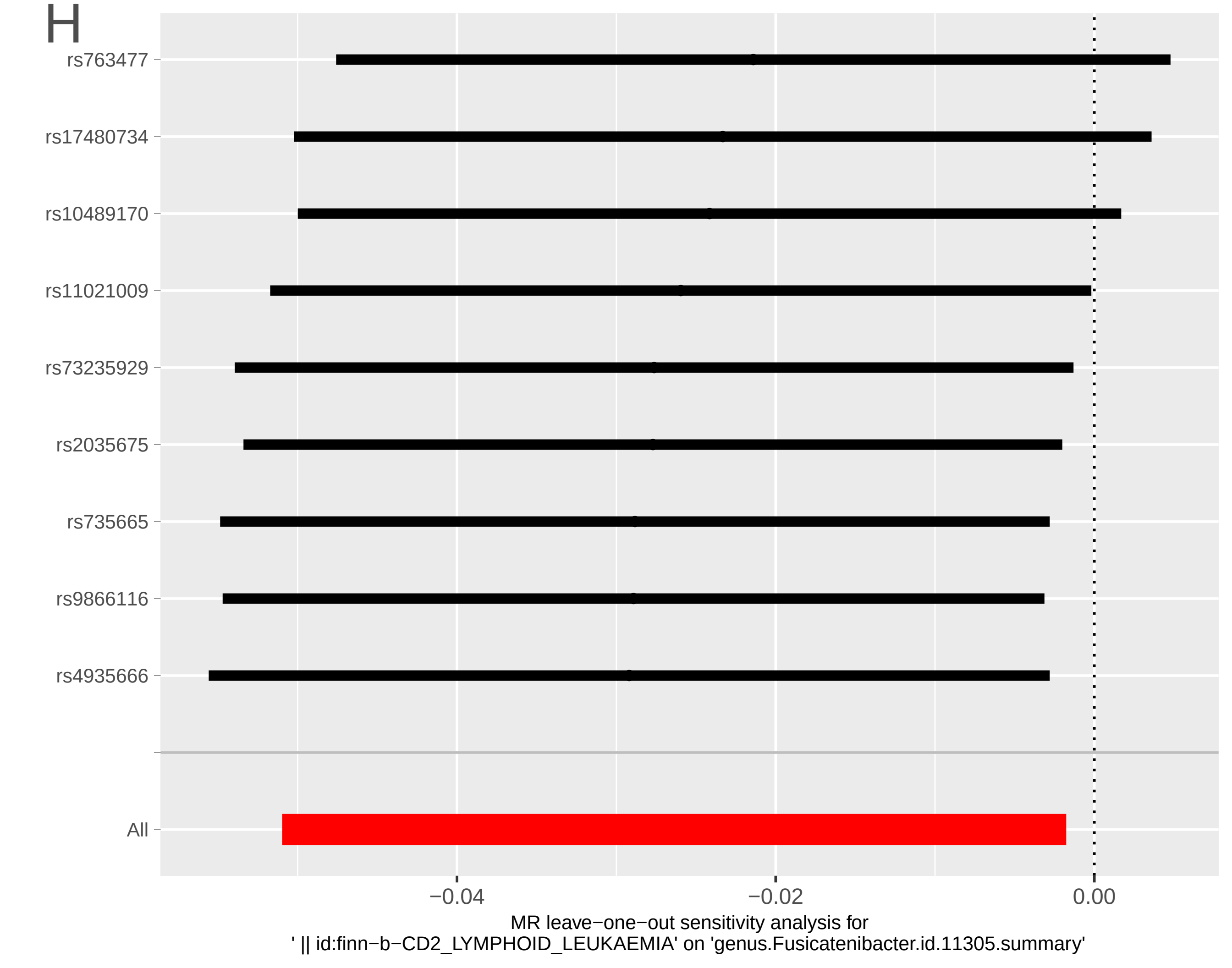
H
rs763477
rs17480734
rs10489170
rs11021009
rs73235929
rs2035675
rs735665
rs9866116
rs4935666
All
−0.04
−0.02
0.00
MR leave−one−out sensitivity analysis for
' || id:finn−b−CD2_LYMPHOID_LEUKAEMIA' on 'genus.Fusicatenibacter.id.11305.summary'

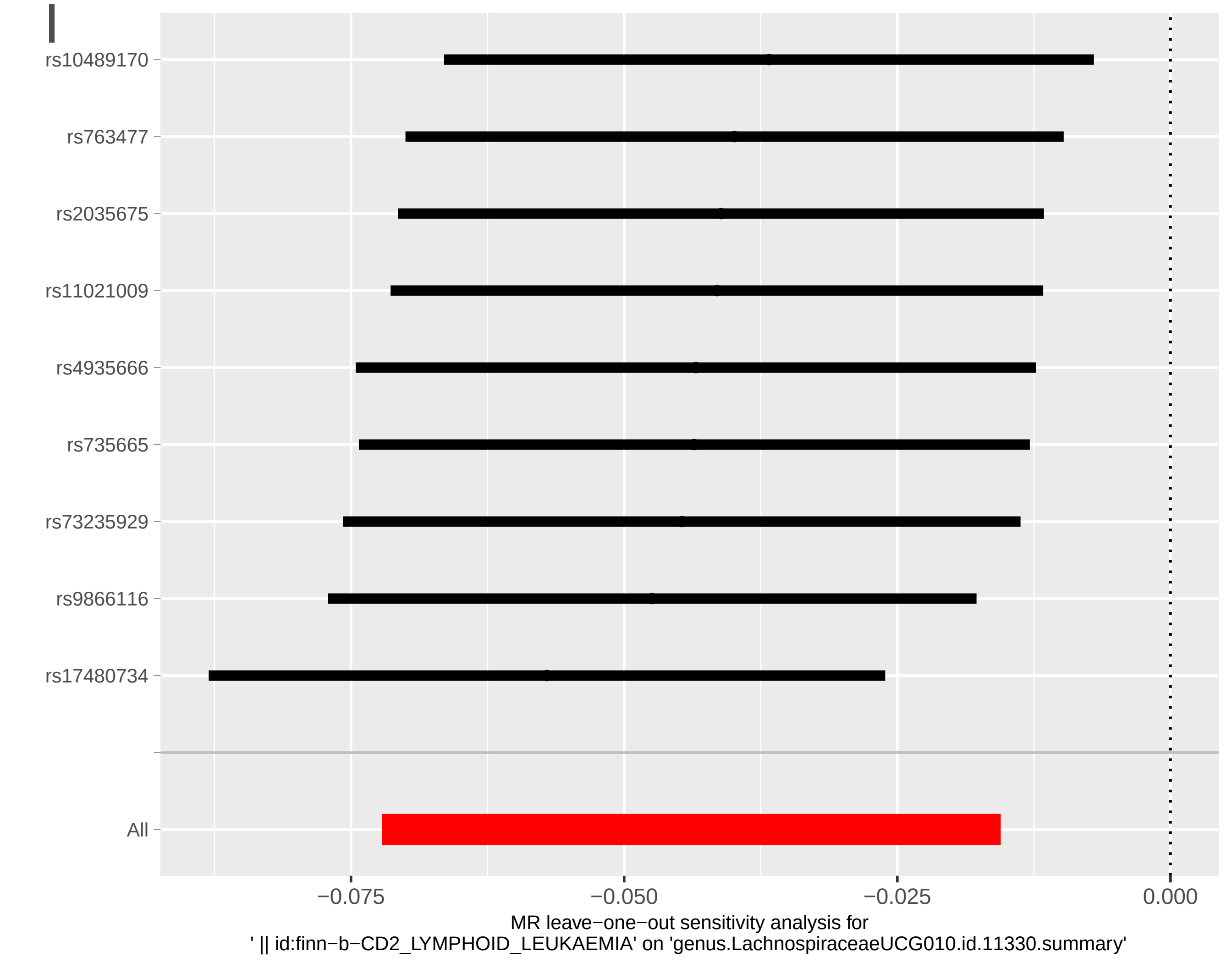
rs10489170
rs763477
rs2035675
rs11021009
rs4935666
rs735665
rs73235929
rs9866116
rs17480734
All
−0.075
−0.050
−0.025
0.000
MR leave−one−out sensitivity analysis for
' || id:finn−b−CD2_LYMPHOID_LEUKAEMIA' on 'genus.LachnospiraceaeUCG010.id.11330.summary'

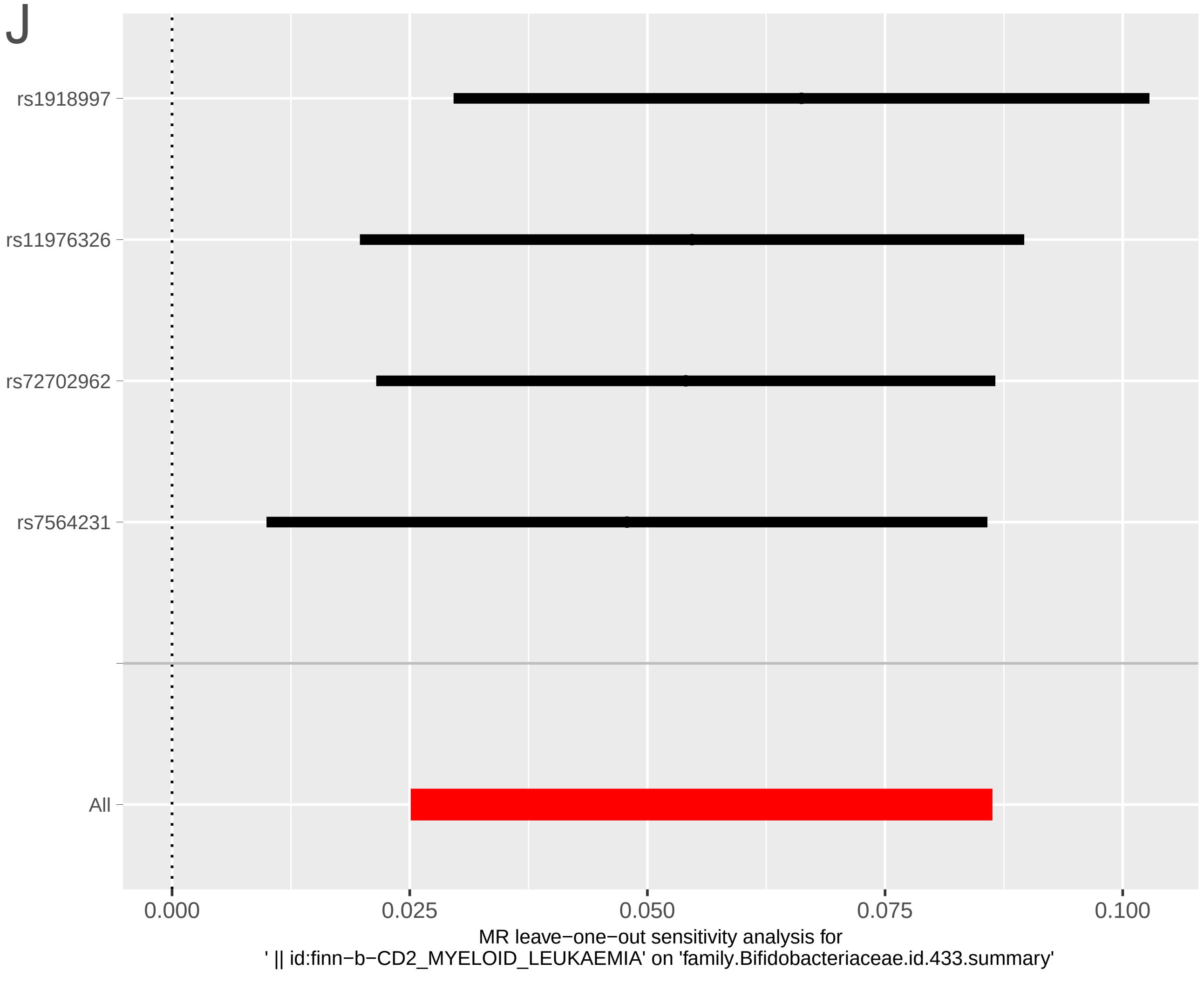
J
rs1918997
rs11976326
rs72702962
rs7564231
All
0.000
0.025
0.050
0.075
0.100
MR leave−one−out sensitivity analysis for
' || id:finn−b−CD2_MYELOID_LEUKAEMIA' on 'family.Bifidobacteriaceae.id.433.summary'

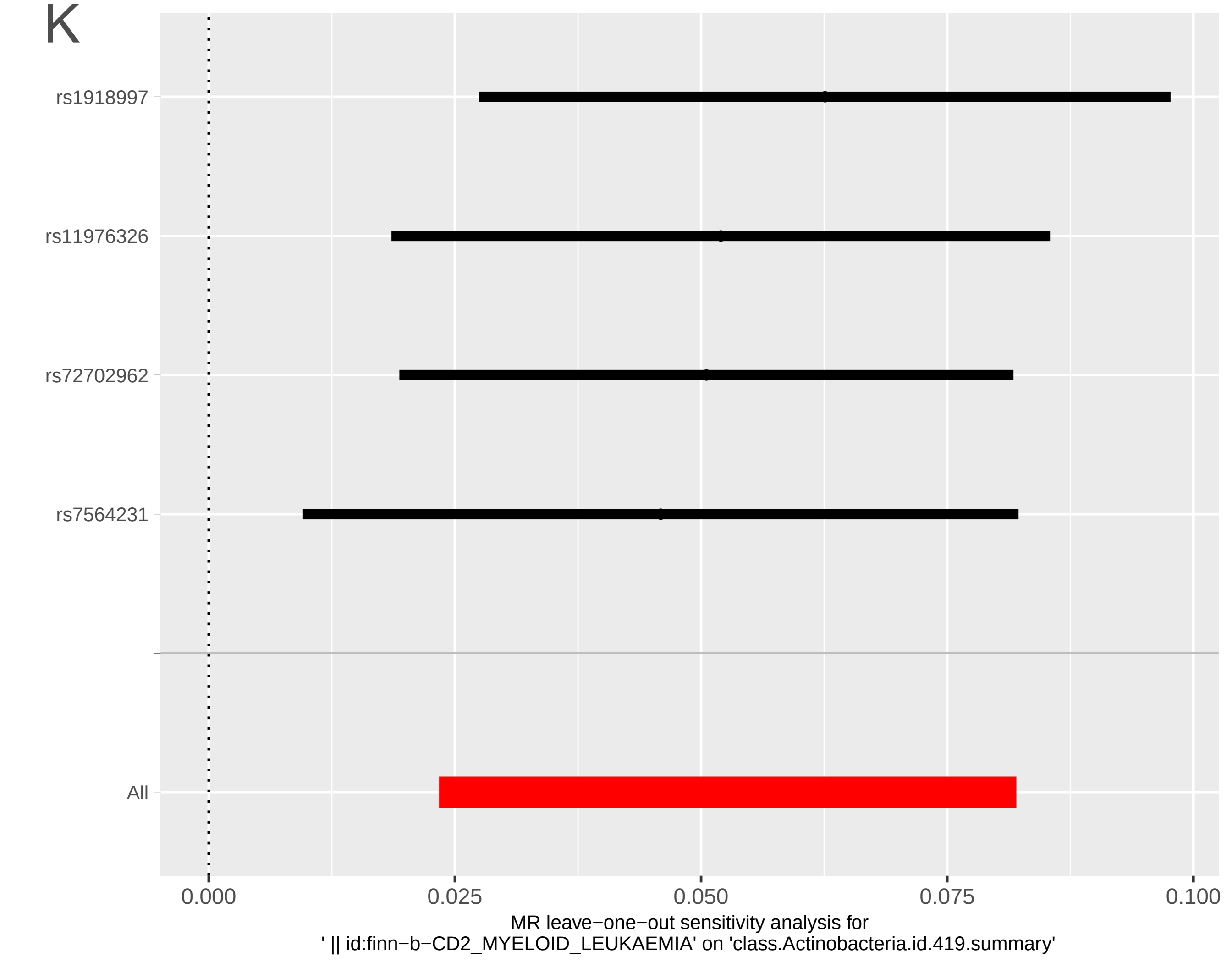
K
rs1918997
rs11976326
rs72702962
rs7564231
All
0.000
0.025
0.050
0.075
0.100
MR leave−one−out sensitivity analysis for
' || id:finn−b−CD2_MYELOID_LEUKAEMIA' on 'class.Actinobacteria.id.419.summary'

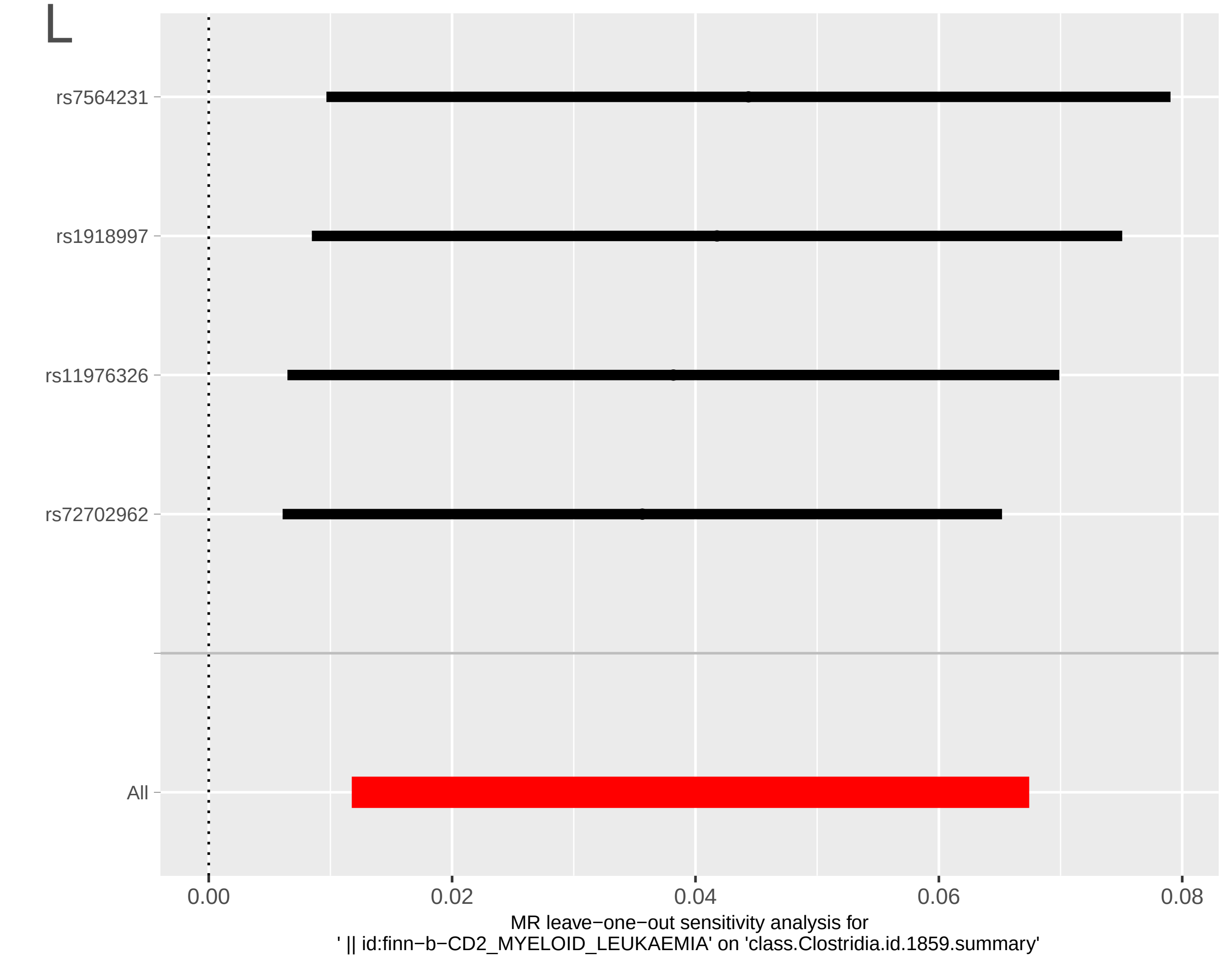
L
rs7564231
rs1918997
rs11976326
rs72702962
All
0.00
0.02
0.04
0.06
0.08
MR leave−one−out sensitivity analysis for
' || id:finn−b−CD2_MYELOID_LEUKAEMIA' on 'class.Clostridia.id.1859.summary'

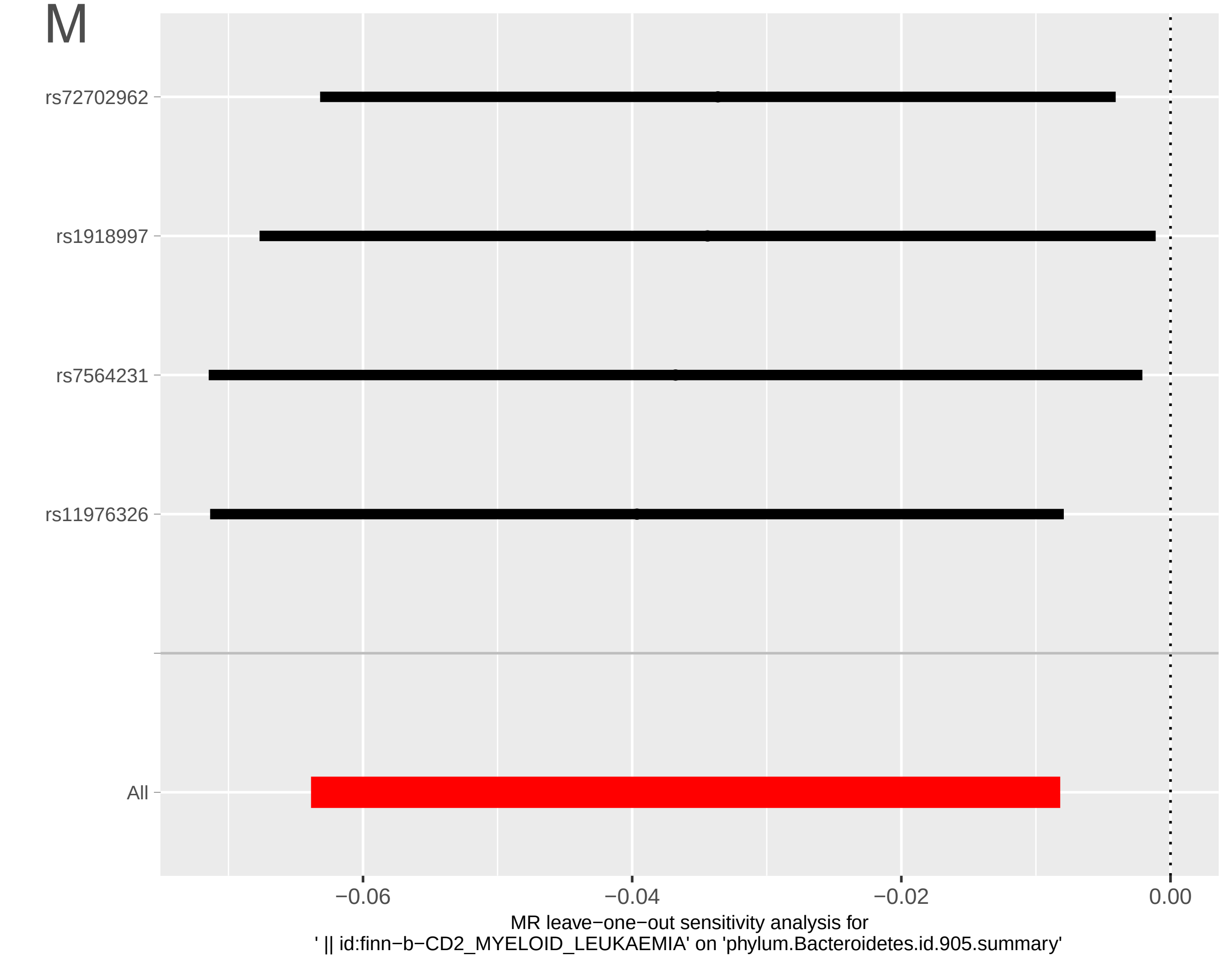
M
rs72702962
rs1918997
rs7564231
rs11976326
All
−0.06
−0.04
−0.02
0.00
MR leave−one−out sensitivity analysis for
' || id:finn−b−CD2_MYELOID_LEUKAEMIA' on 'phylum.Bacteroidetes.id.905.summary'

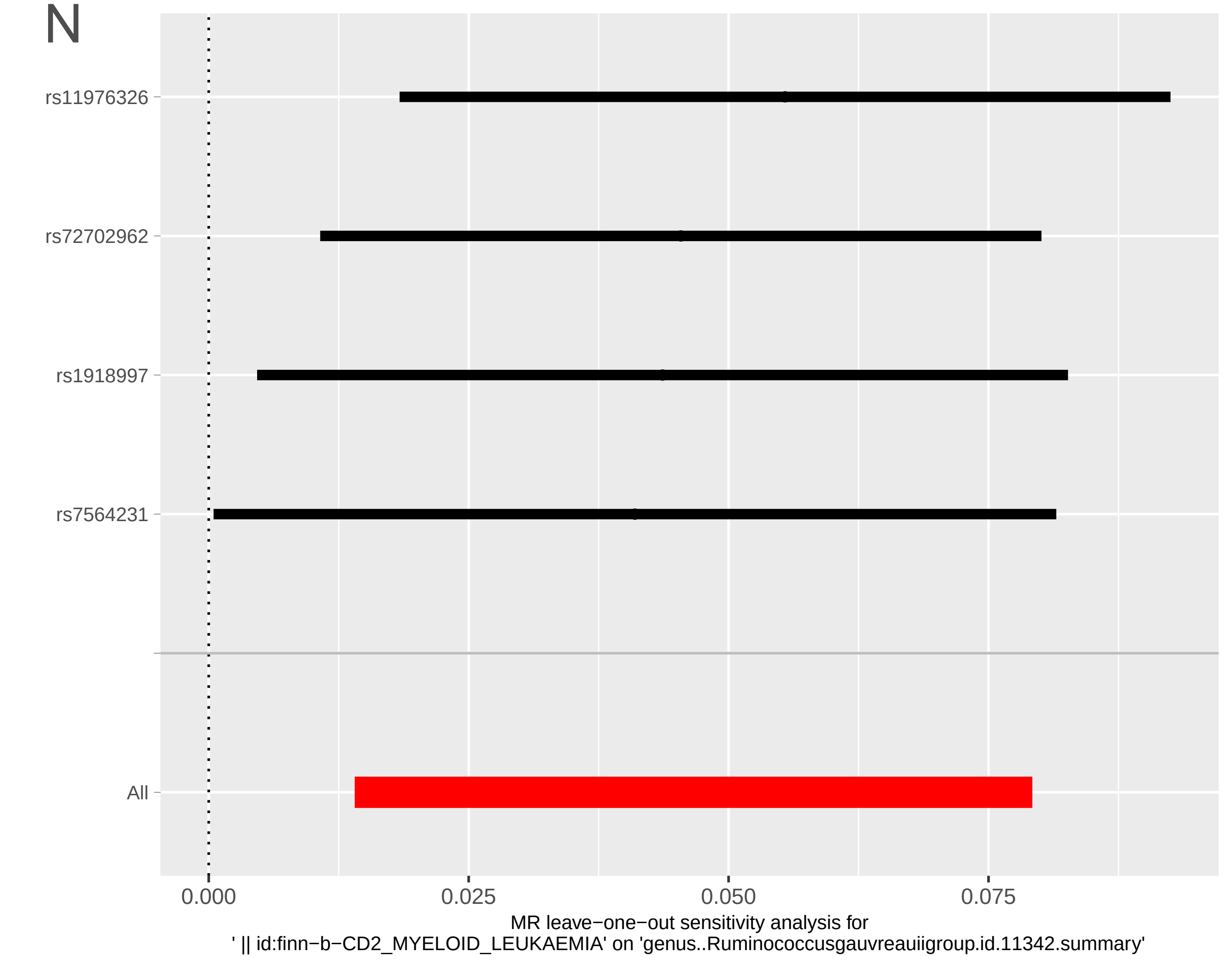
N
rs11976326
rs72702962
rs1918997
rs7564231
All
0.000
0.025
0.050
0.075
MR leave−one−out sensitivity analysis for
' || id:finn−b−CD2_MYELOID_LEUKAEMIA' on 'genus..Ruminococcusgauvreauiigroup.id.11342.summary'

O

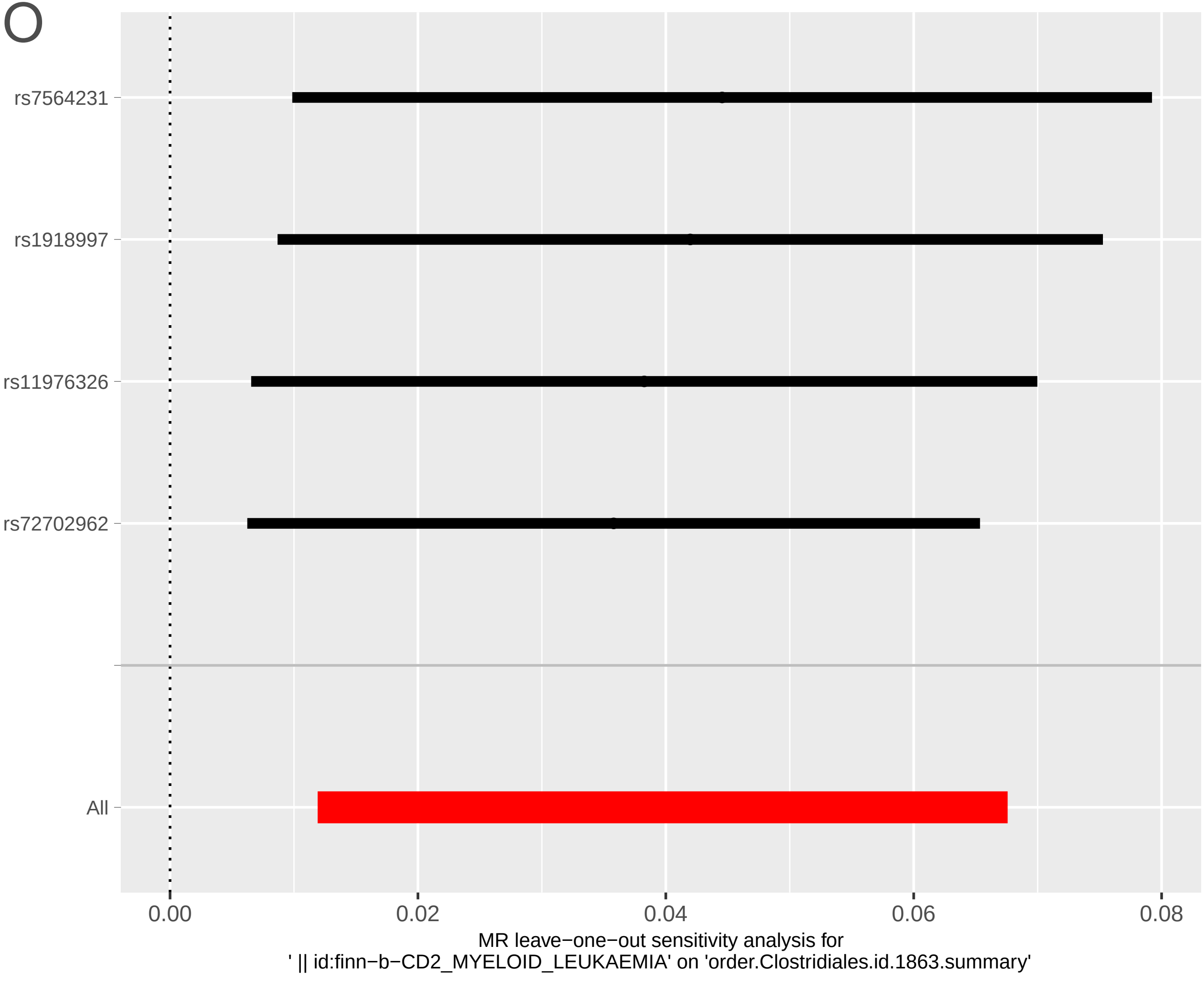

rs7564231
rs1918997
rs11976326
rs72702962
All
0.00
0.02
0.04
0.06
0.08
MR leave−one−out sensitivity analysis for
' || id:finn−b−CD2_MYELOID_LEUKAEMIA' on 'order.Clostridiales.id.1863.summary'

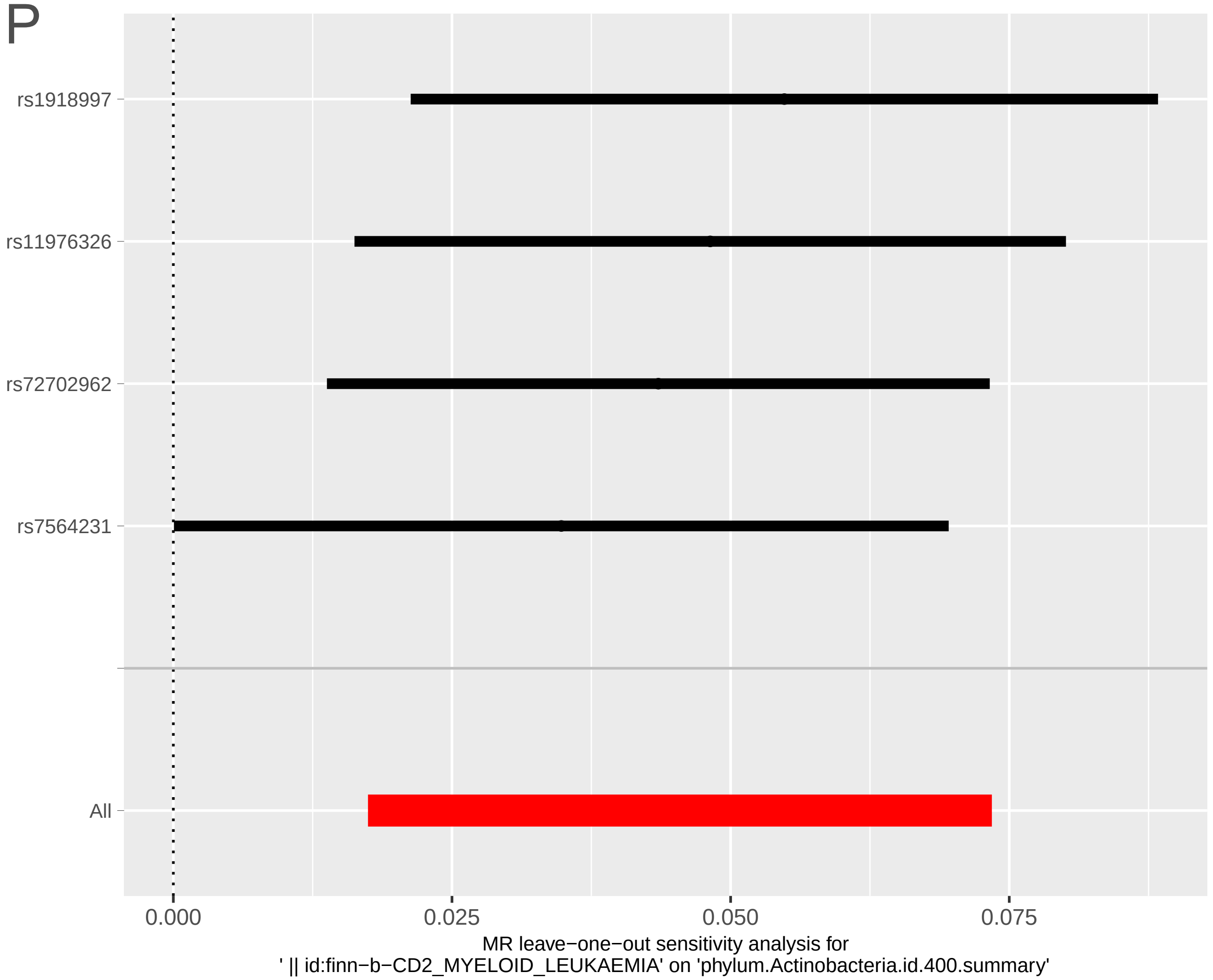
P
rs1918997
rs11976326
rs72702962
rs7564231
All
0.000
0.025
0.050
0.075
MR leave−one−out sensitivity analysis for
' || id:finn−b−CD2_MYELOID_LEUKAEMIA' on 'phylum.Actinobacteria.id.400.summary'

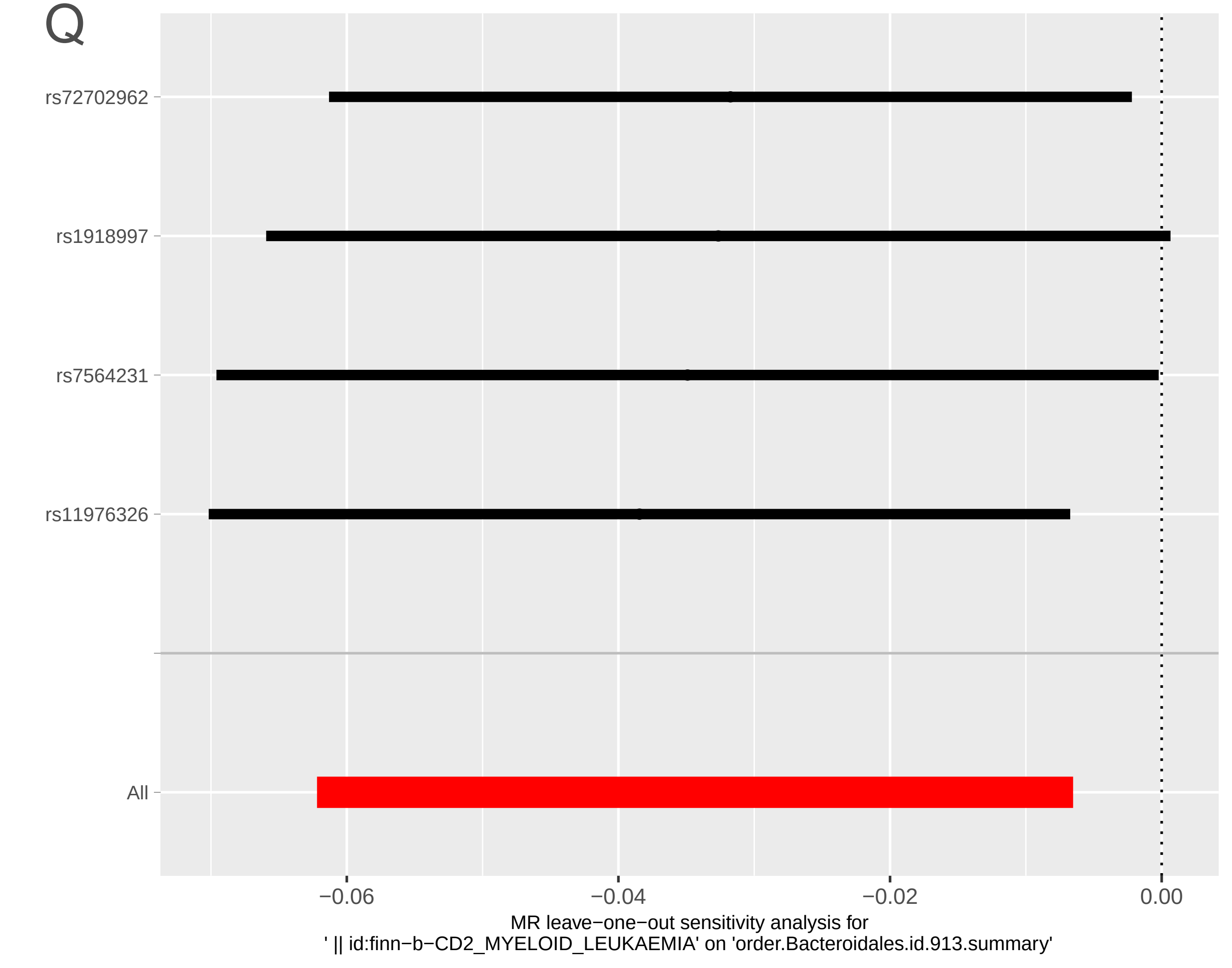
Q
rs72702962
rs1918997
rs7564231
rs11976326
All
−0.06
−0.04
−0.02
0.00
MR leave−one−out sensitivity analysis for
' || id:finn−b−CD2_MYELOID_LEUKAEMIA' on 'order.Bacteroidales.id.913.summary'

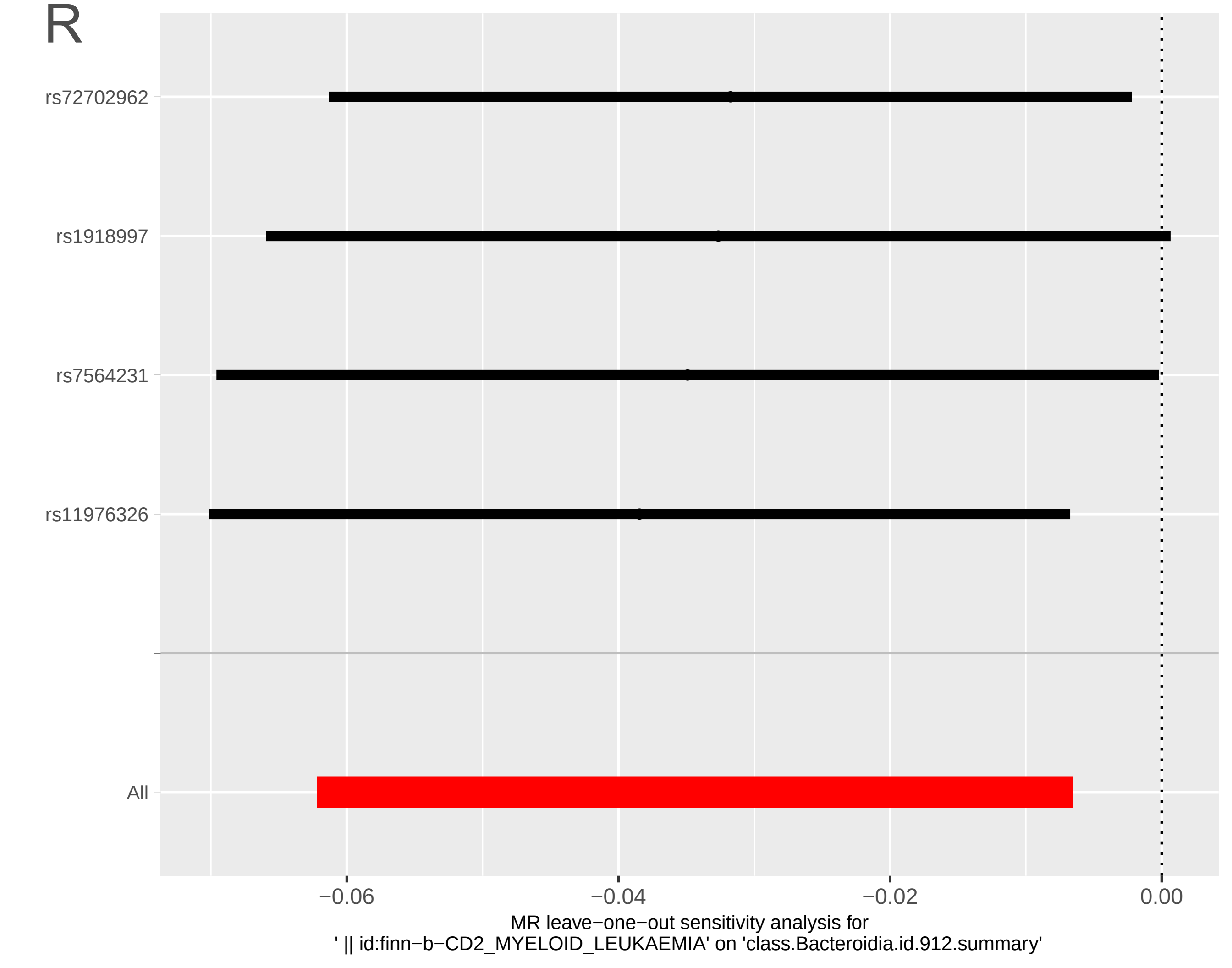
R
rs72702962
rs1918997
rs7564231
rs11976326
All
−0.06
−0.04
−0.02
0.00
MR leave−one−out sensitivity analysis for
' || id:finn−b−CD2_MYELOID_LEUKAEMIA' on 'class.Bacteroidia.id.912.summary'

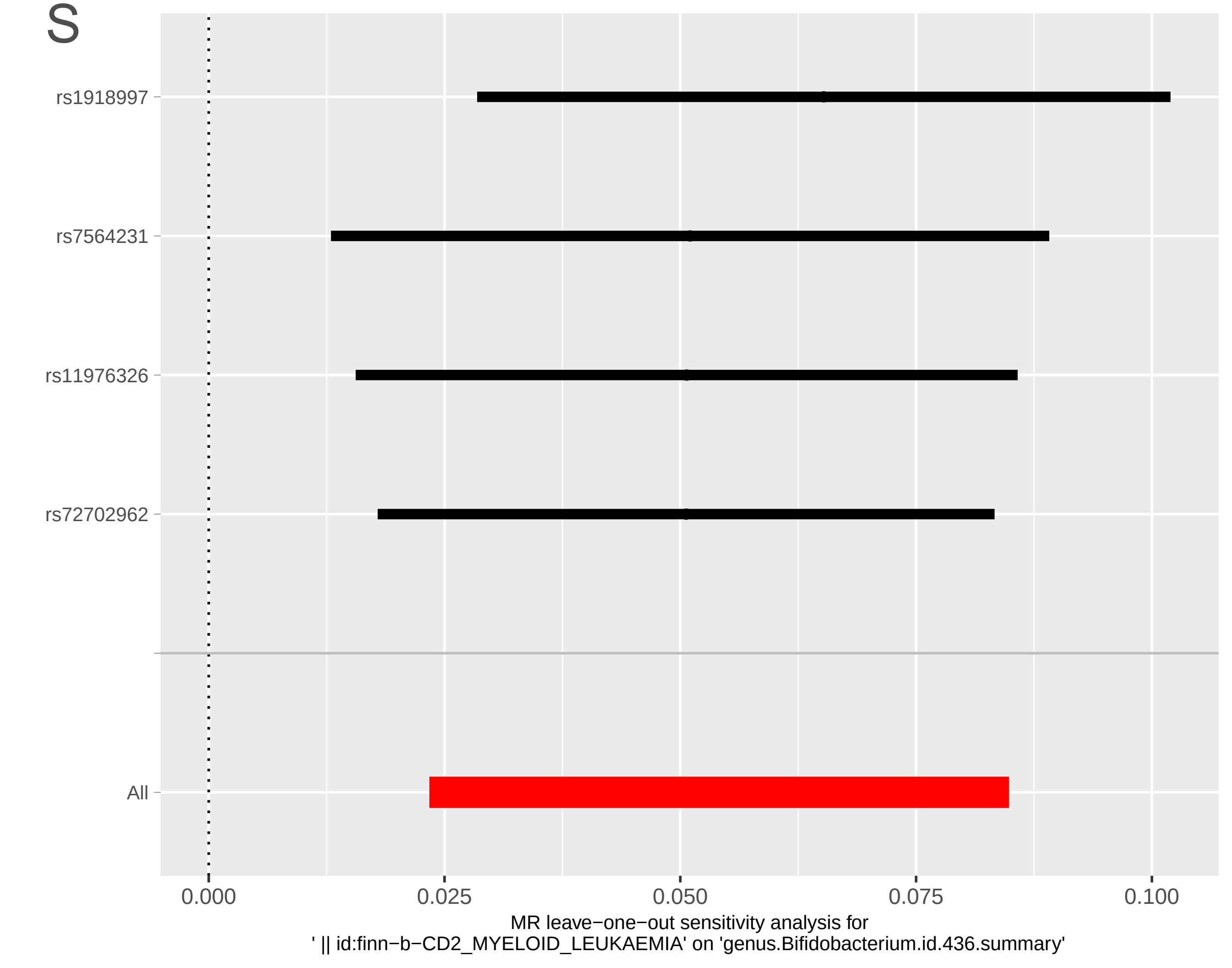
S
rs1918997
rs7564231
rs11976326
rs72702962
All
0.000
0.025
0.050
0.075
0.100
MR leave−one−out sensitivity analysis for
' || id:finn−b−CD2_MYELOID_LEUKAEMIA' on 'genus.Bifidobacterium.id.436.summary'

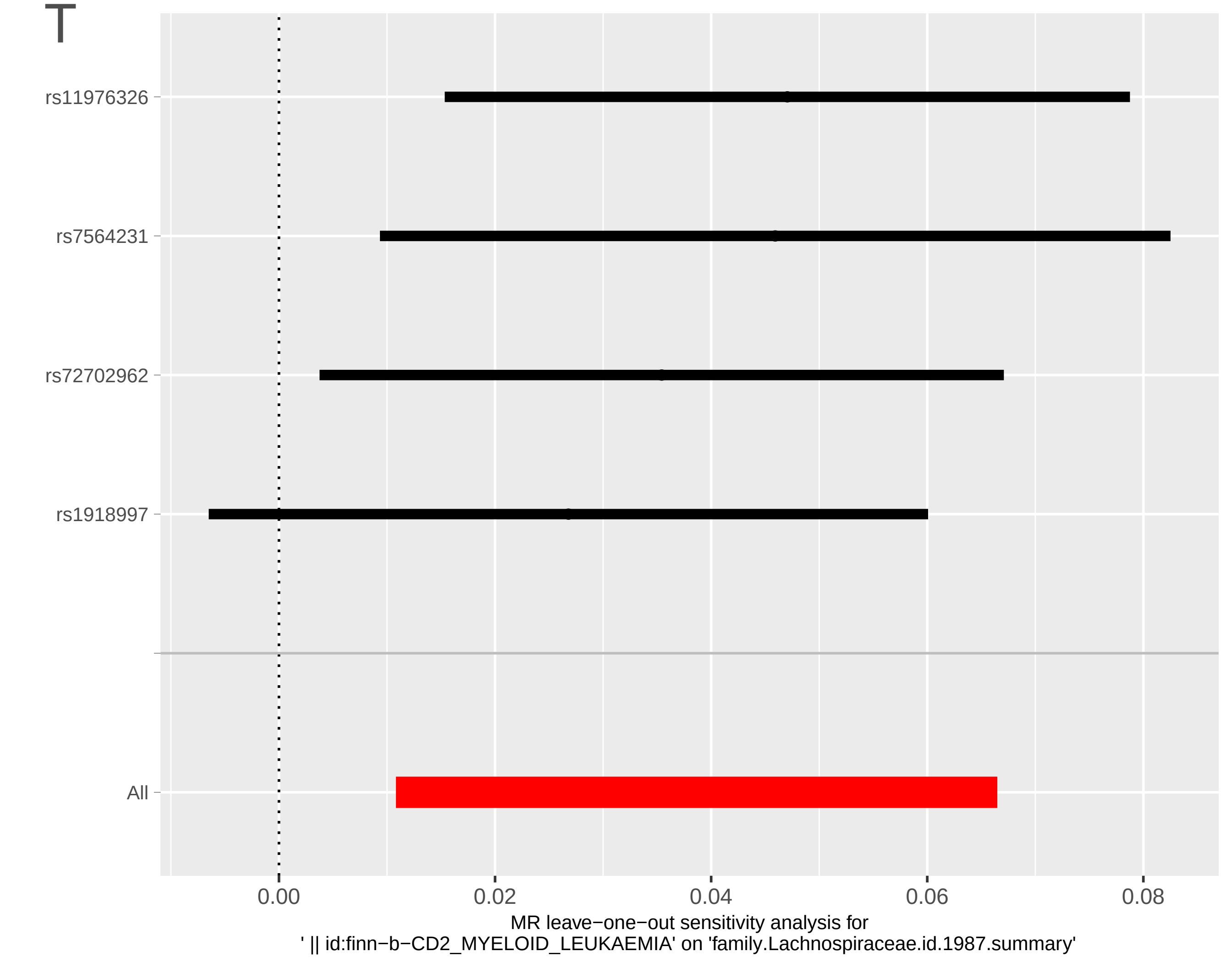
T
rs11976326
rs7564231
rs72702962
rs1918997
All
0.00
0.02
0.04
0.06
0.08
MR leave−one−out sensitivity analysis for
' || id:finn−b−CD2_MYELOID_LEUKAEMIA' on 'family.Lachnospiraceae.id.1987.summary'

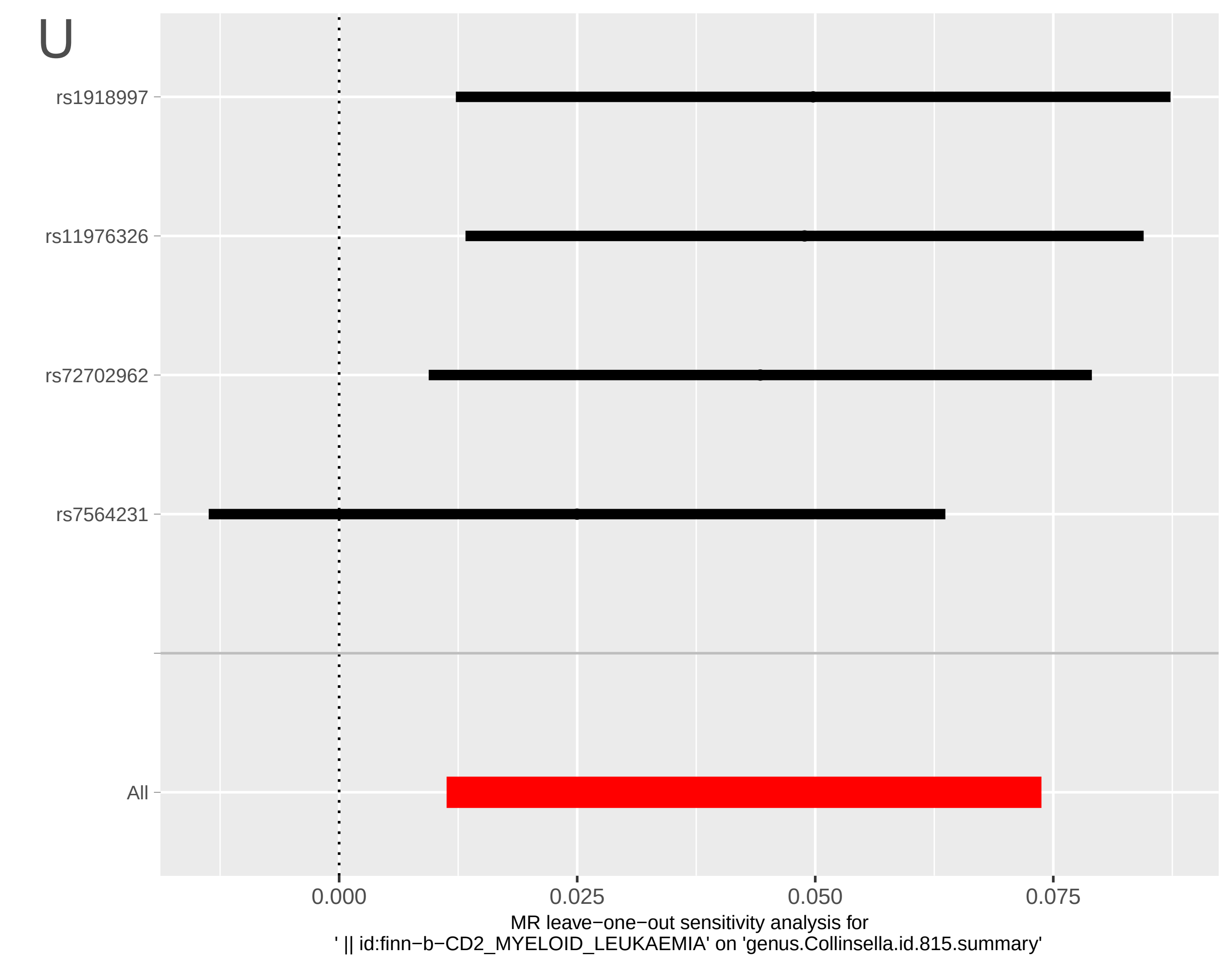
U
rs1918997
rs11976326
rs72702962
rs7564231
All
0.000
0.025
0.050
0.075
MR leave−one−out sensitivity analysis for
' || id:finn−b−CD2_MYELOID_LEUKAEMIA' on 'genus.Collinsella.id.815.summary'

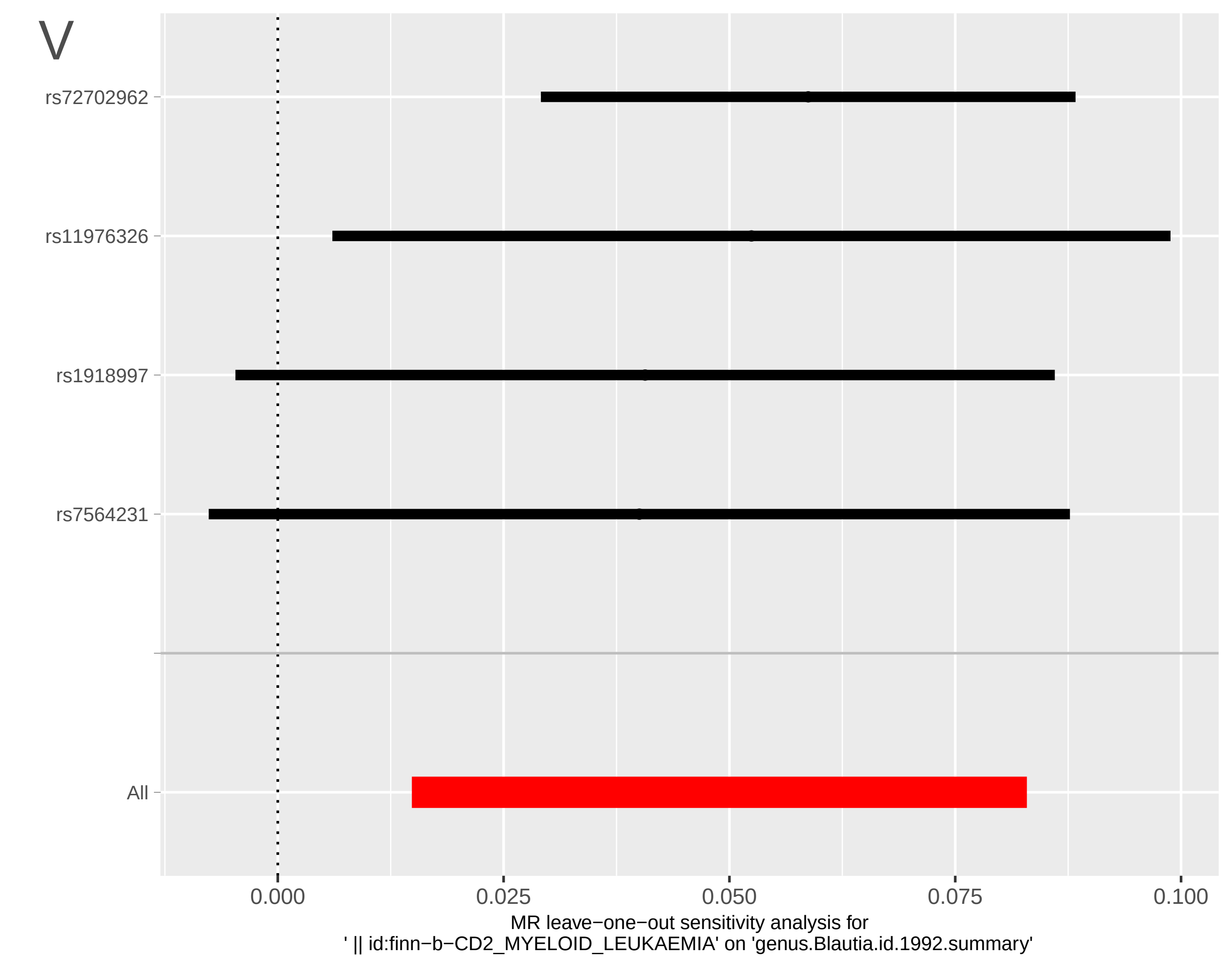
V
rs72702962
rs11976326
rs1918997
rs7564231
All
0.000
0.025
0.050
0.075
0.100
MR leave−one−out sensitivity analysis for
' || id:finn−b−CD2_MYELOID_LEUKAEMIA' on 'genus.Blautia.id.1992.summary'

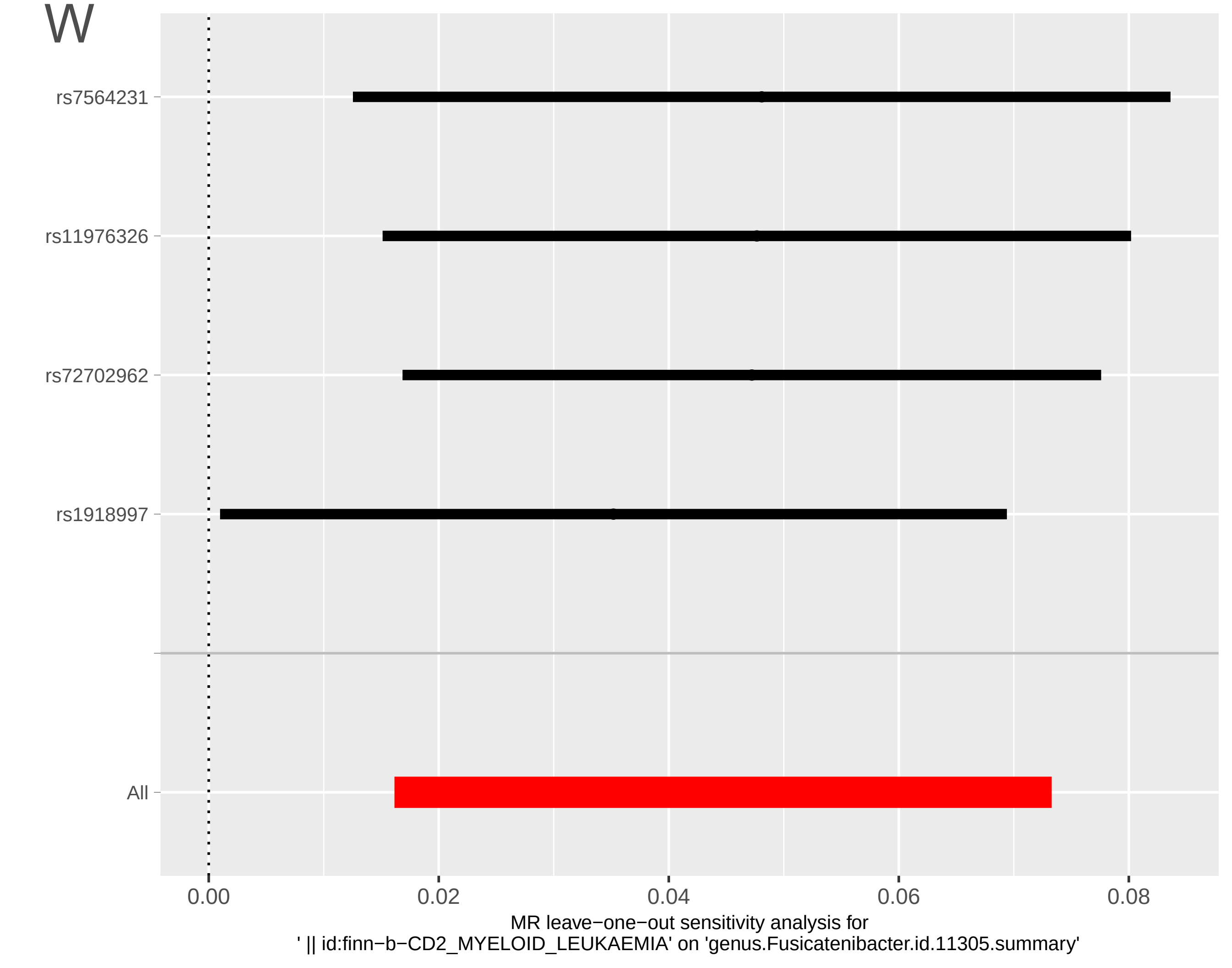
W
rs7564231
rs11976326
rs72702962
rs1918997
All
0.00
0.02
0.04
0.06
0.08
MR leave−one−out sensitivity analysis for
' || id:finn−b−CD2_MYELOID_LEUKAEMIA' on 'genus.Fusicatenibacter.id.11305.summary'

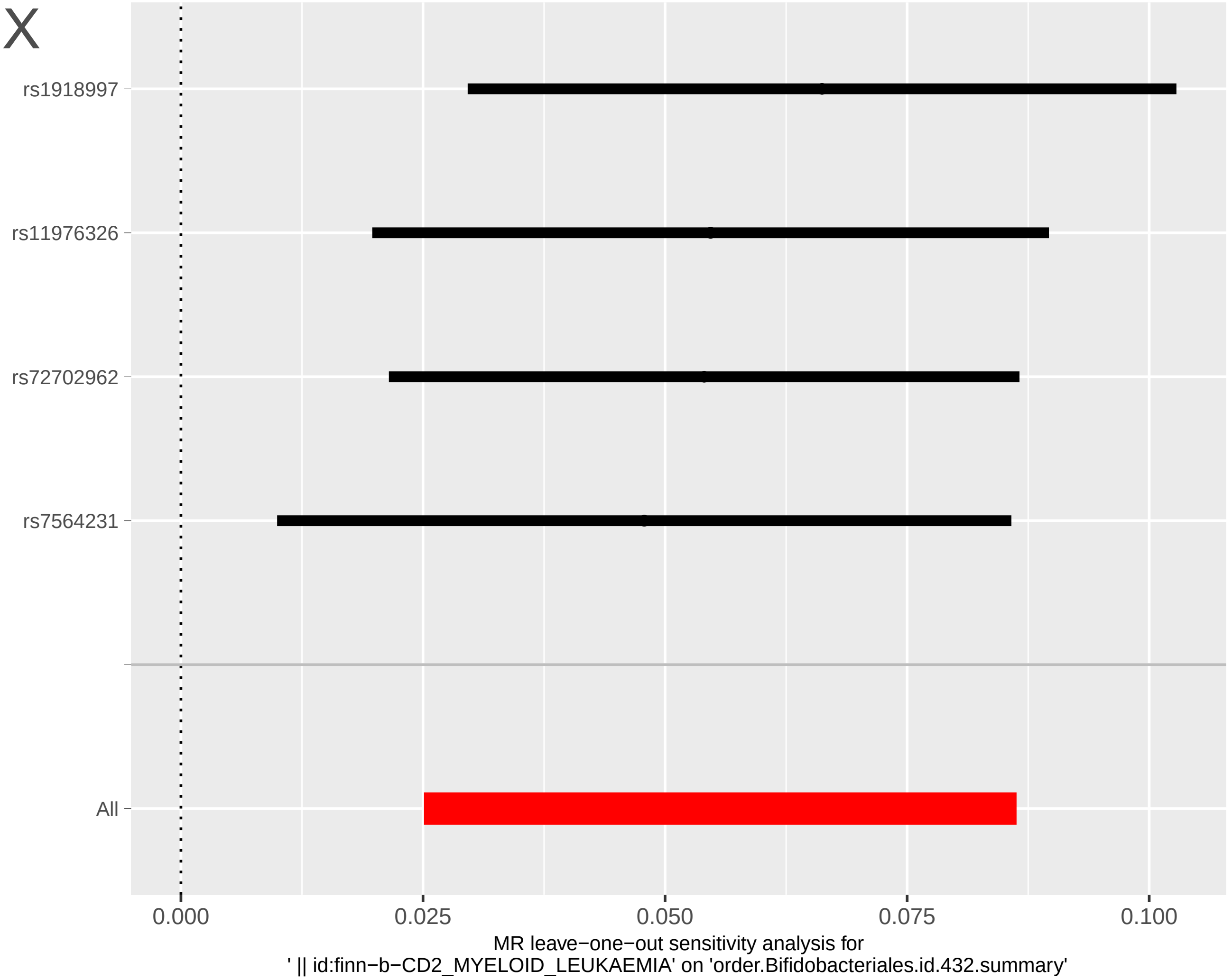
X
rs1918997
rs11976326
rs72702962
rs7564231
All
0.000
0.025
0.050
0.075
0.100
MR leave−one−out sensitivity analysis for
' || id:finn−b−CD2_MYELOID_LEUKAEMIA' on 'order.Bifidobacteriales.id.432.summary'

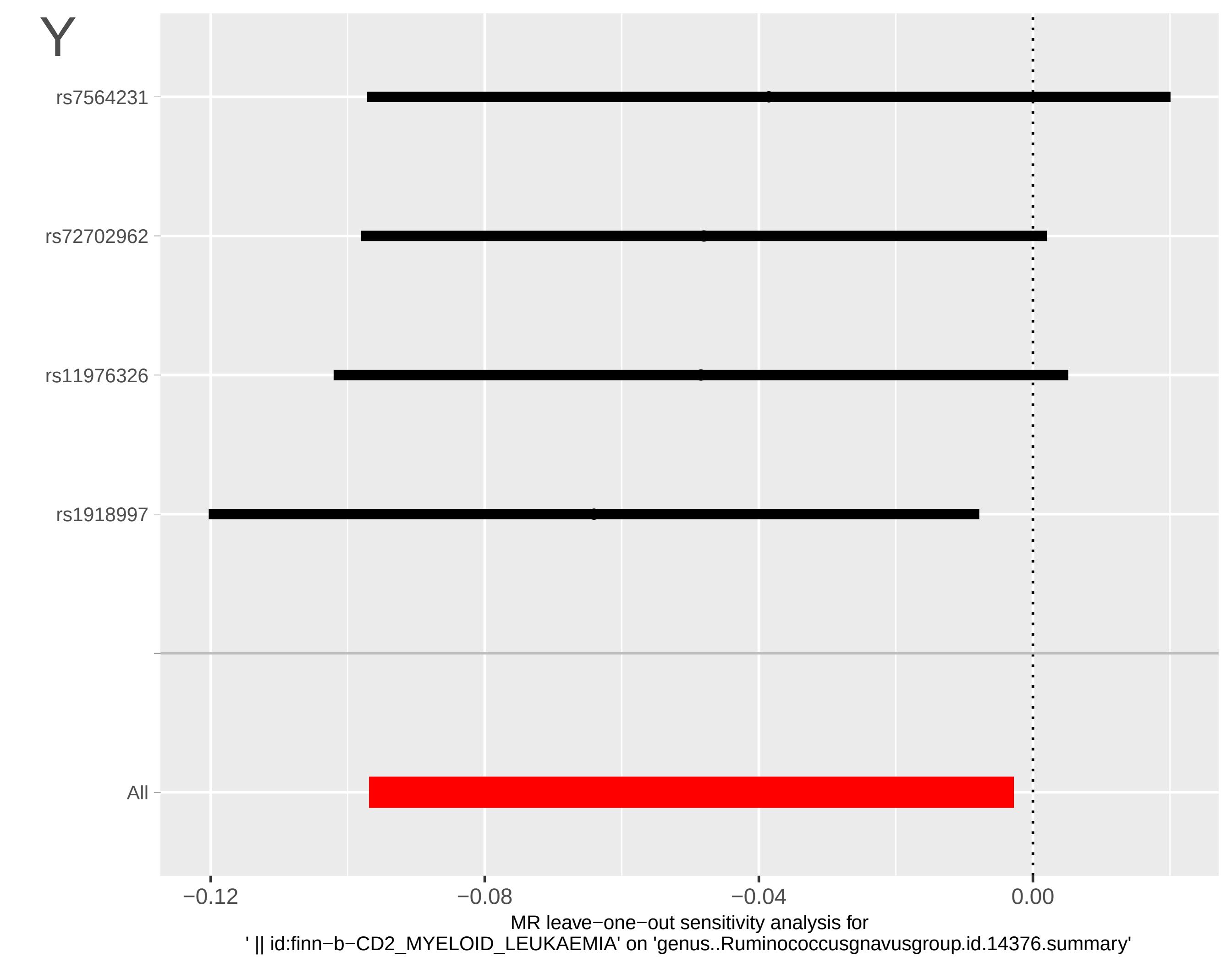

Y
rs7564231
rs72702962
rs11976326
rs1918997
All
−0.12
−0.08
−0.04
0.00
MR leave−one−out sensitivity analysis for
' || id:finn−b−CD2_MYELOID_LEUKAEMIA' on 'genus..Ruminococcusgnavusgroup.id.14376.summary'

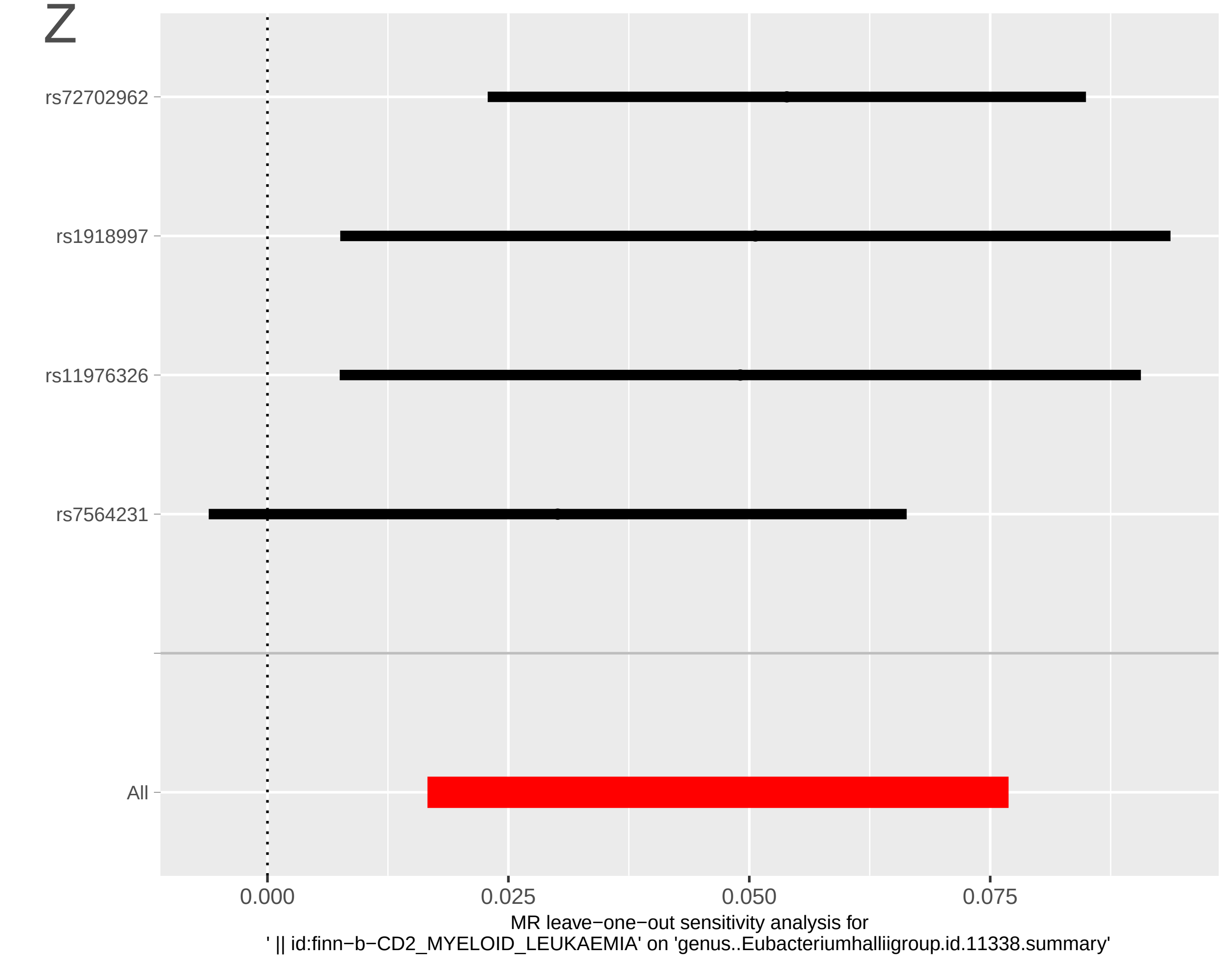

Z
rs72702962
rs1918997
rs11976326
rs7564231
All
0.000
0.025
0.050
0.075
MR leave−one−out sensitivity analysis for
' || id:finn−b−CD2_MYELOID_LEUKAEMIA' on 'genus..Eubacteriumhalliigroup.id.11338.summary'

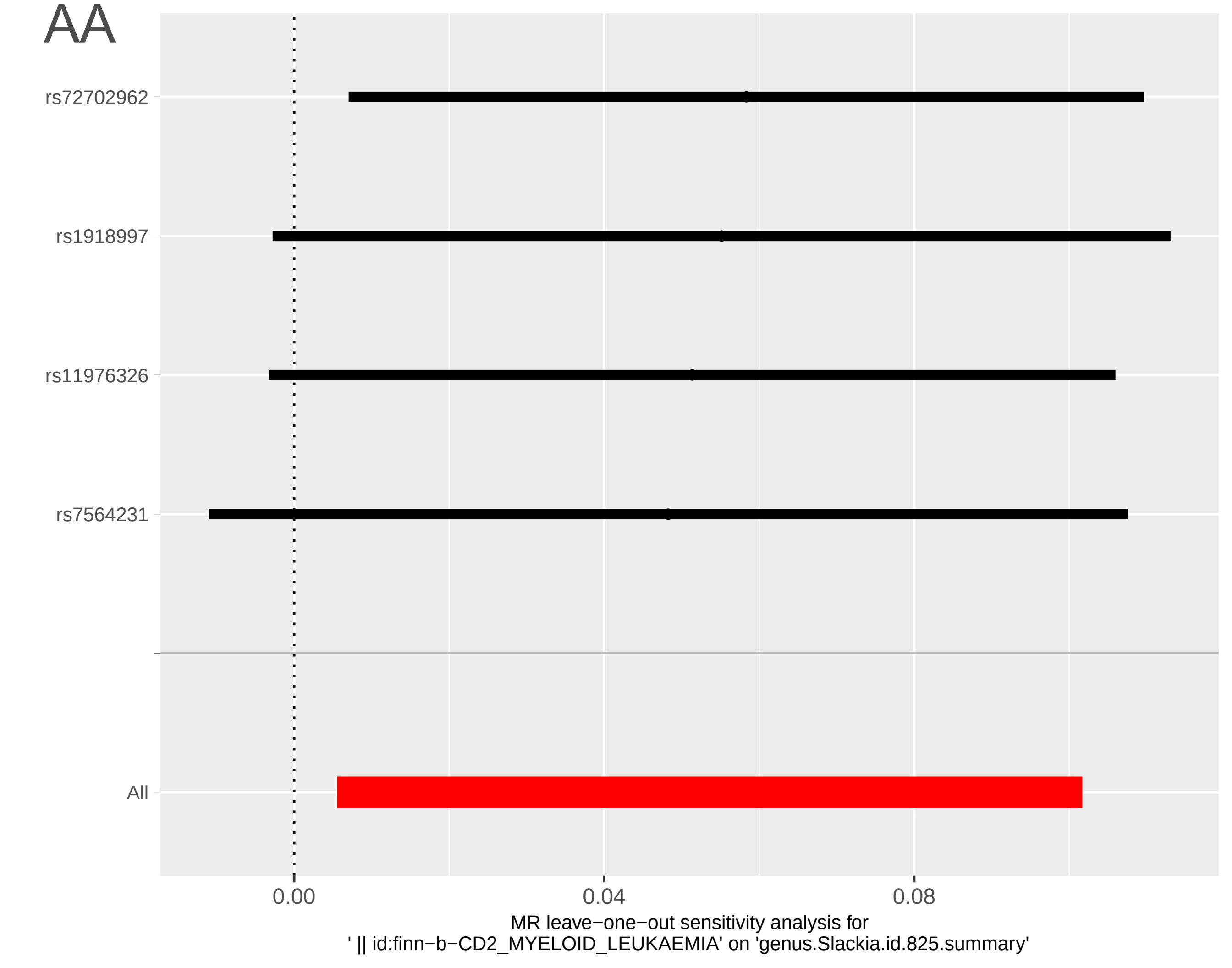
AA
rs72702962
rs1918997
rs11976326
rs7564231
All
0.00
0.04
0.08
MR leave−one−out sensitivity analysis for
' || id:finn−b−CD2_MYELOID_LEUKAEMIA' on 'genus.Slackia.id.825.summary'

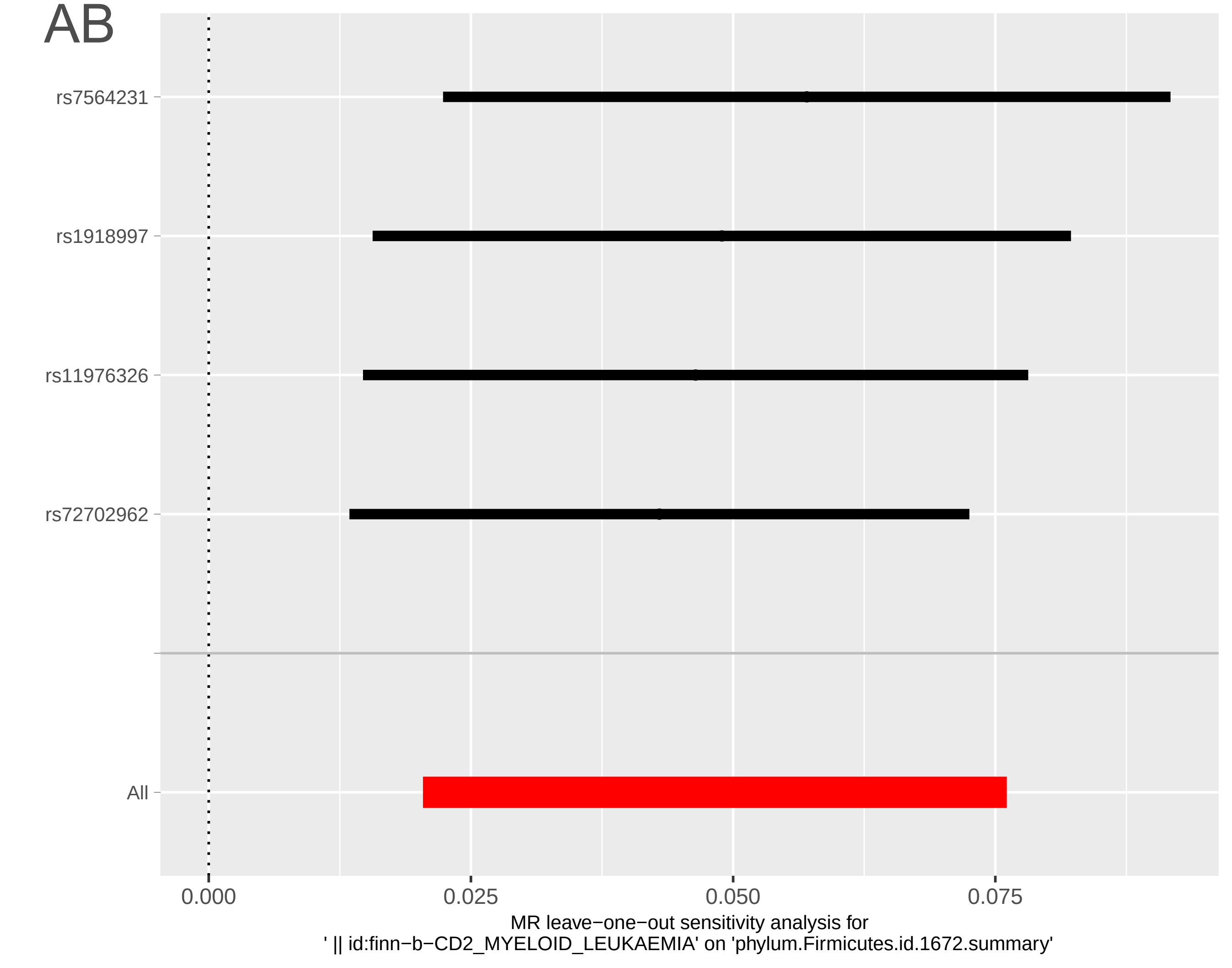

AB
rs7564231
rs1918997
rs11976326
rs72702962
All
0.000
0.025
0.050
0.075
MR leave−one−out sensitivity analysis for
' || id:finn−b−CD2_MYELOID_LEUKAEMIA' on 'phylum.Firmicutes.id.1672.summary'

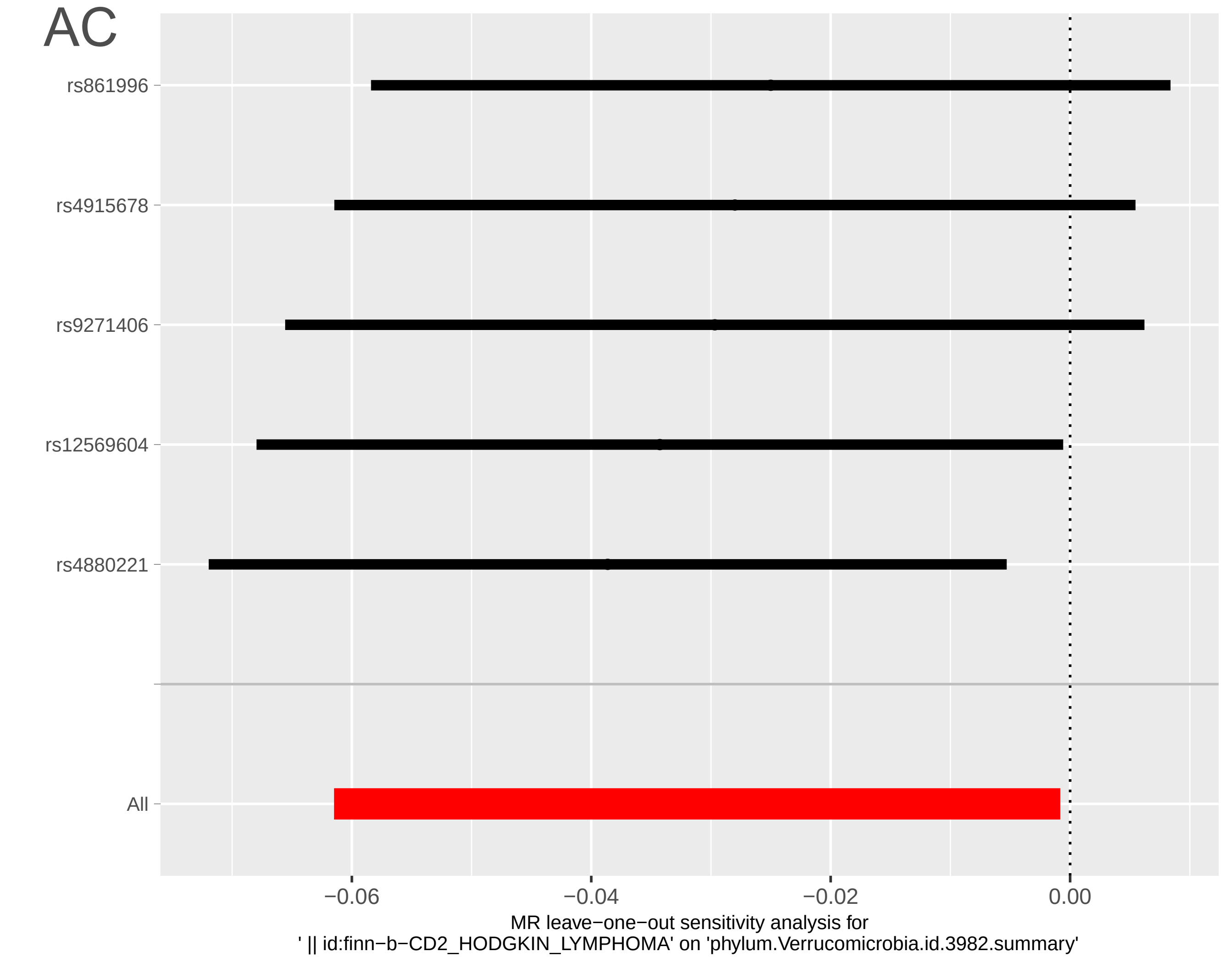
AC
rs861996
rs4915678
rs9271406
rs12569604
rs4880221
All
−0.06
−0.04
−0.02
0.00
MR leave−one−out sensitivity analysis for
' || id:finn−b−CD2_HODGKIN_LYMPHOMA' on 'phylum.Verrucomicrobia.id.3982.summary'

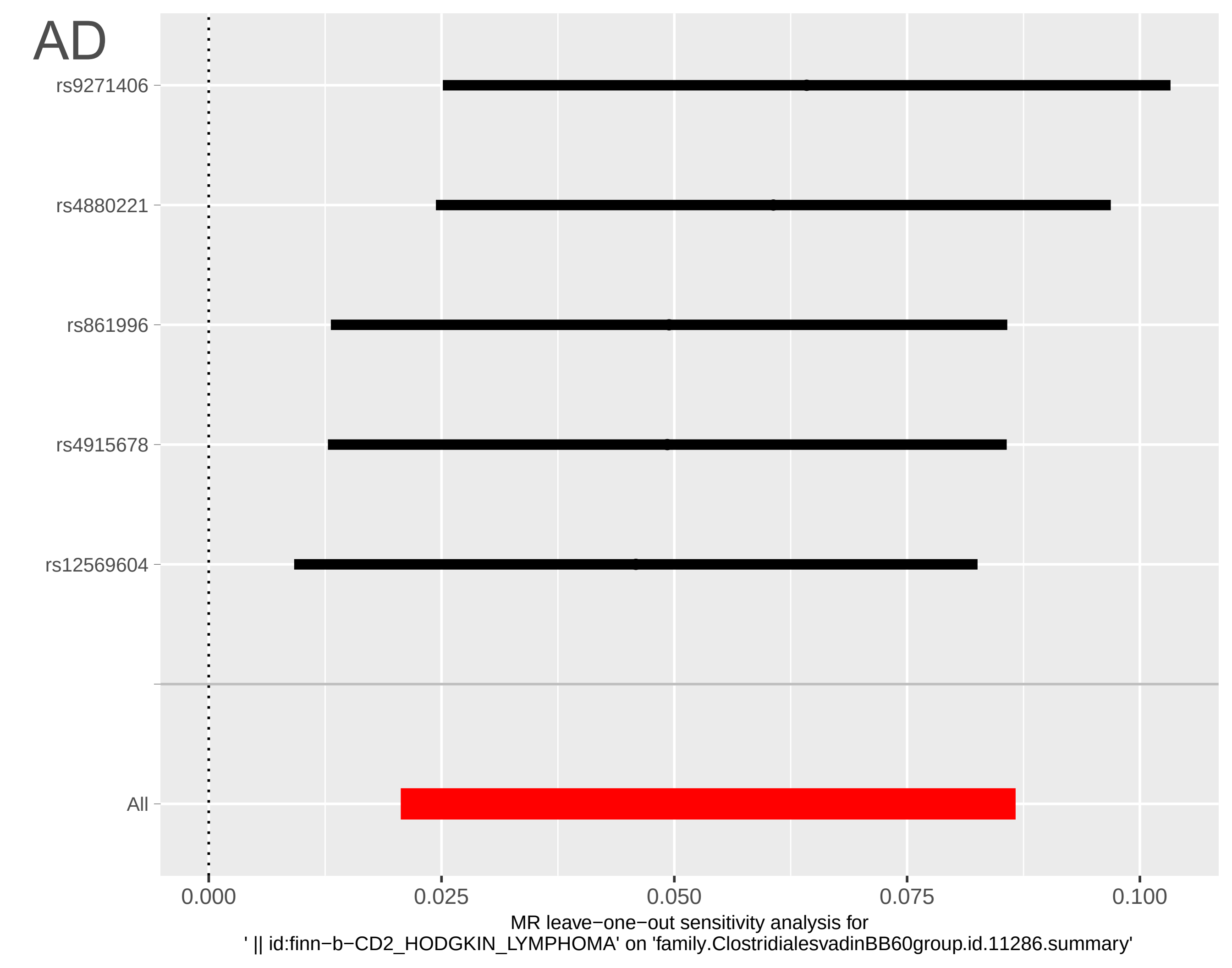
AD
rs9271406
rs4880221
rs861996
rs4915678
rs12569604
All
0.000
0.025
0.050
0.075
0.100
MR leave−one−out sensitivity analysis for
' || id:finn−b−CD2_HODGKIN_LYMPHOMA' on 'family.ClostridialesvadinBB60group.id.11286.summary'

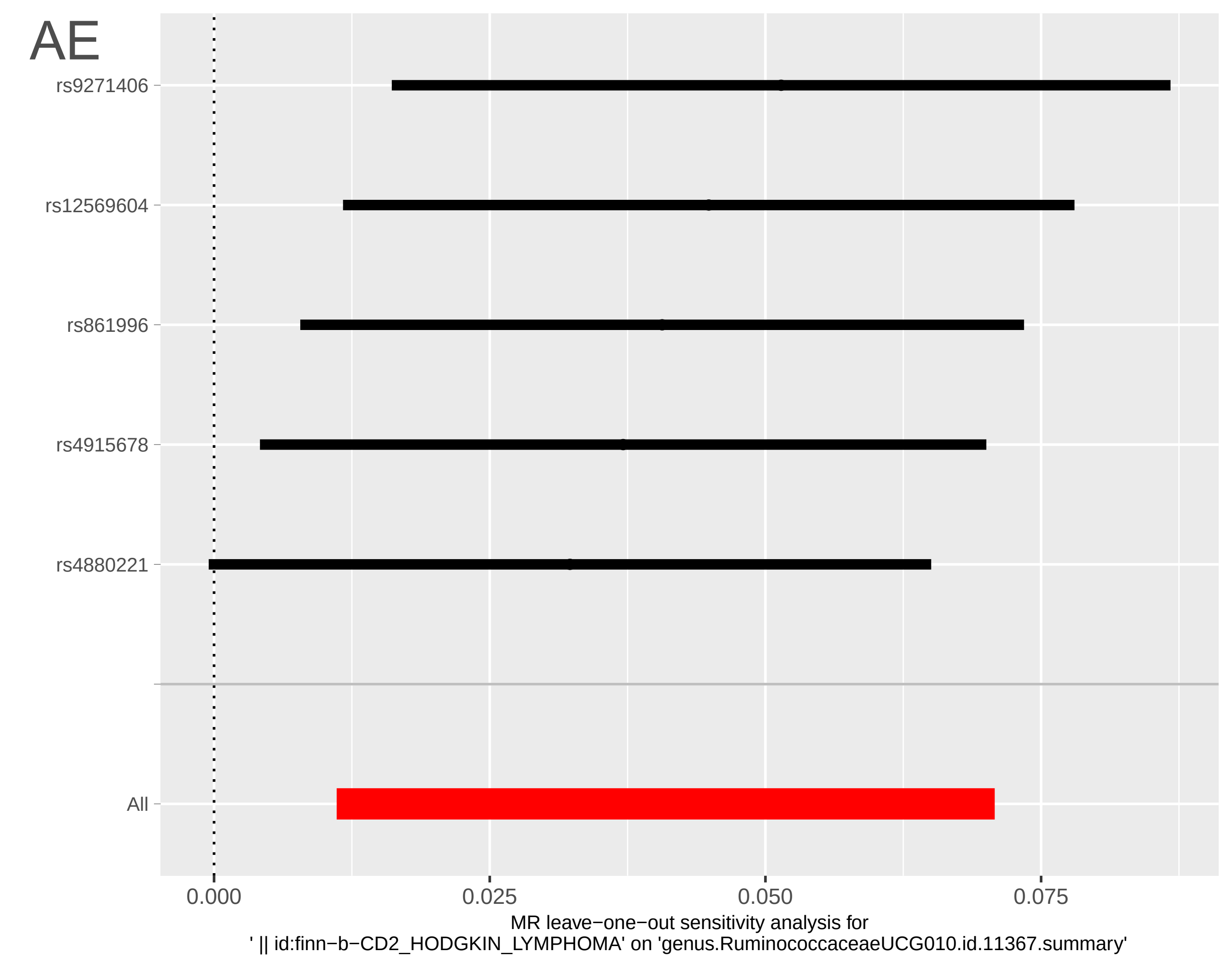
AE
rs9271406
rs12569604
rs861996
rs4915678
rs4880221
All
0.000
0.025
0.050
0.075
MR leave−one−out sensitivity analysis for
' || id:finn−b−CD2_HODGKIN_LYMPHOMA' on 'genus.RuminococcaceaeUCG010.id.11367.summary'

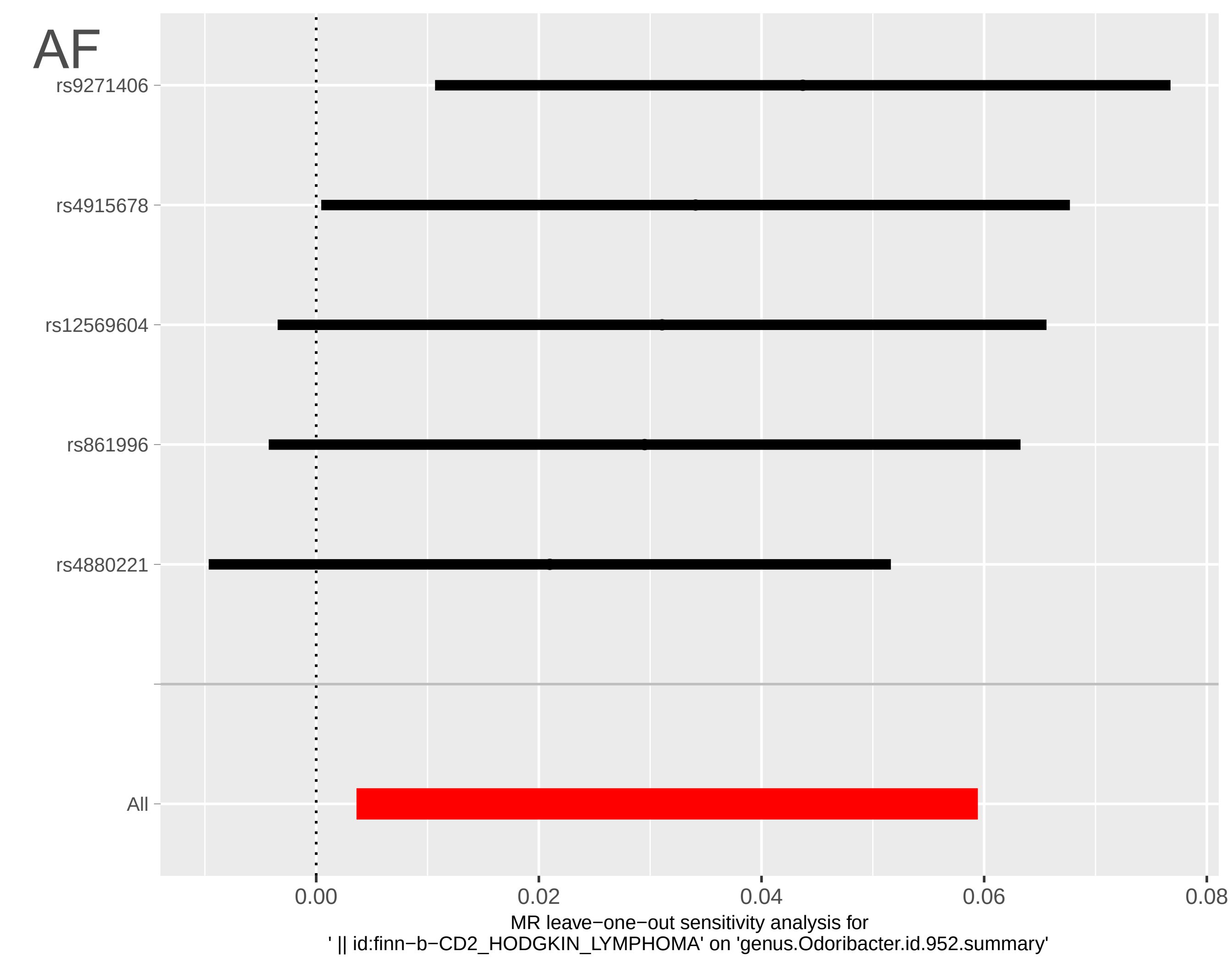
AF
rs9271406
rs4915678
rs12569604
rs861996
rs4880221
All
0.00
0.02
0.04
0.06
0.08
MR leave−one−out sensitivity analysis for
' || id:finn−b−CD2_HODGKIN_LYMPHOMA' on 'genus.Odoribacter.id.952.summary'

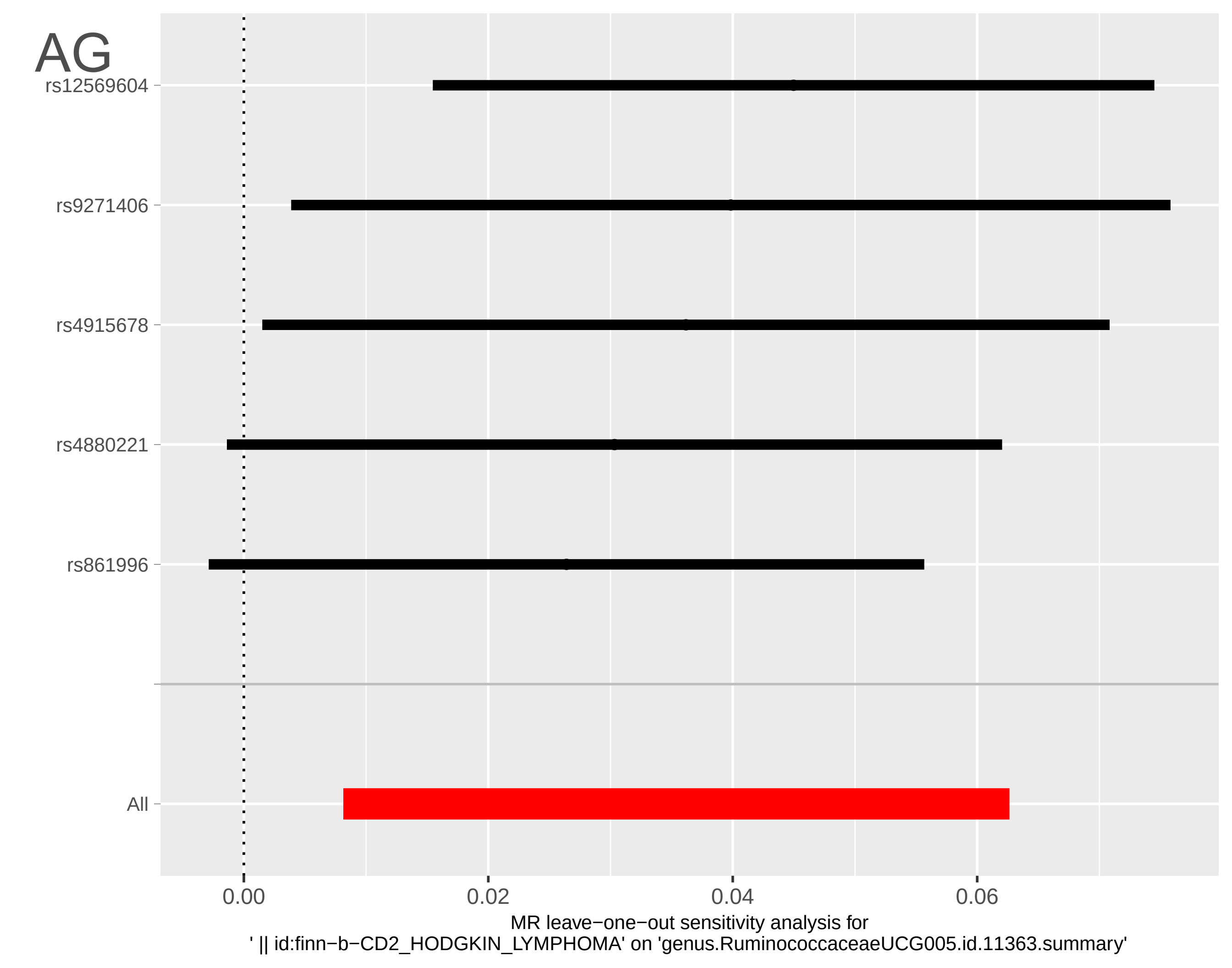
AG
rs12569604
rs9271406
rs4915678
rs4880221
rs861996
All
0.00
0.02
0.04
0.06
MR leave−one−out sensitivity analysis for
' || id:finn−b−CD2_HODGKIN_LYMPHOMA' on 'genus.RuminococcaceaeUCG005.id.11363.summary'

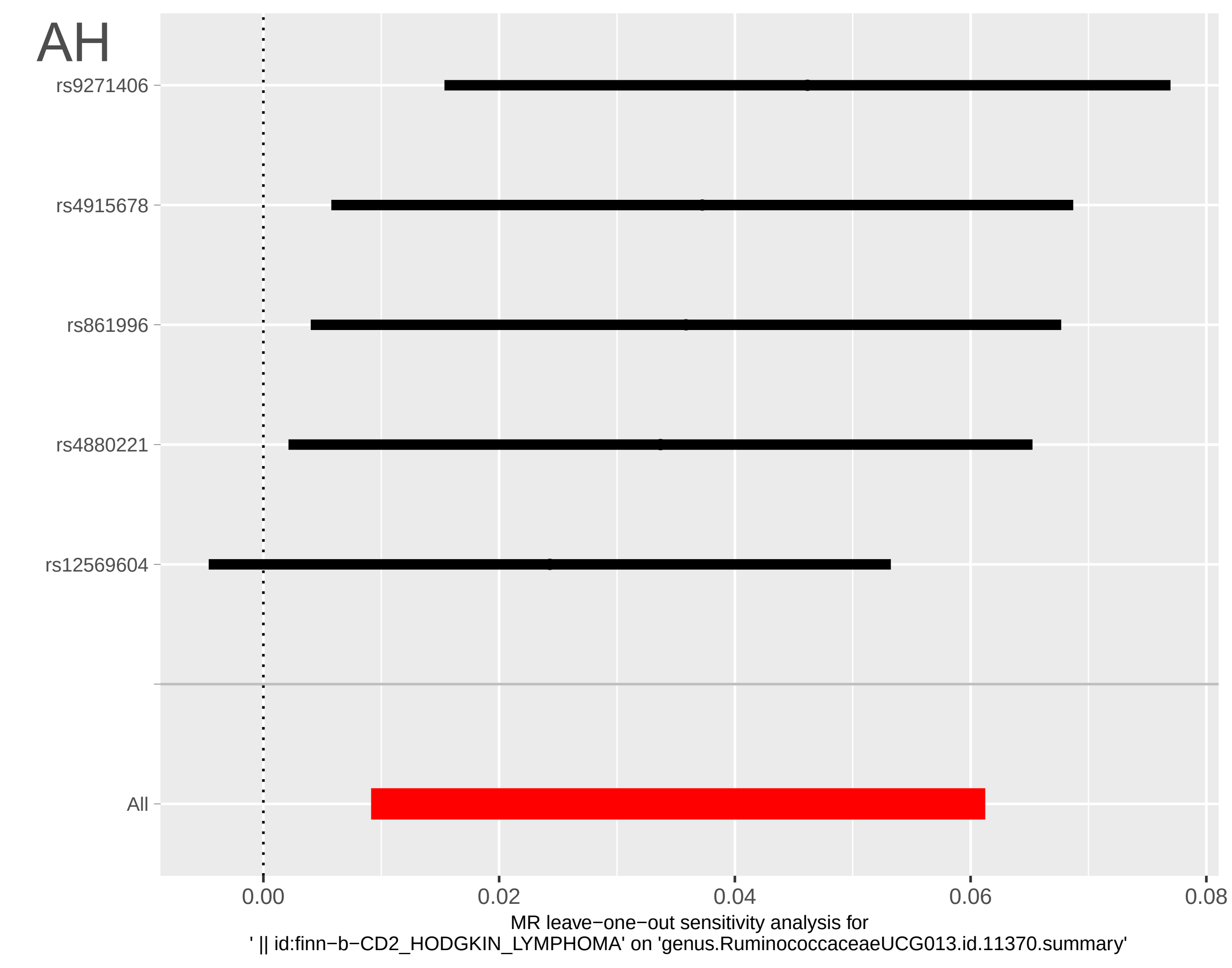
AH
rs9271406
rs4915678
rs861996
rs4880221
rs12569604
All
0.00
0.02
0.04
0.06
0.08
MR leave−one−out sensitivity analysis for
' || id:finn−b−CD2_HODGKIN_LYMPHOMA' on 'genus.RuminococcaceaeUCG013.id.11370.summary'

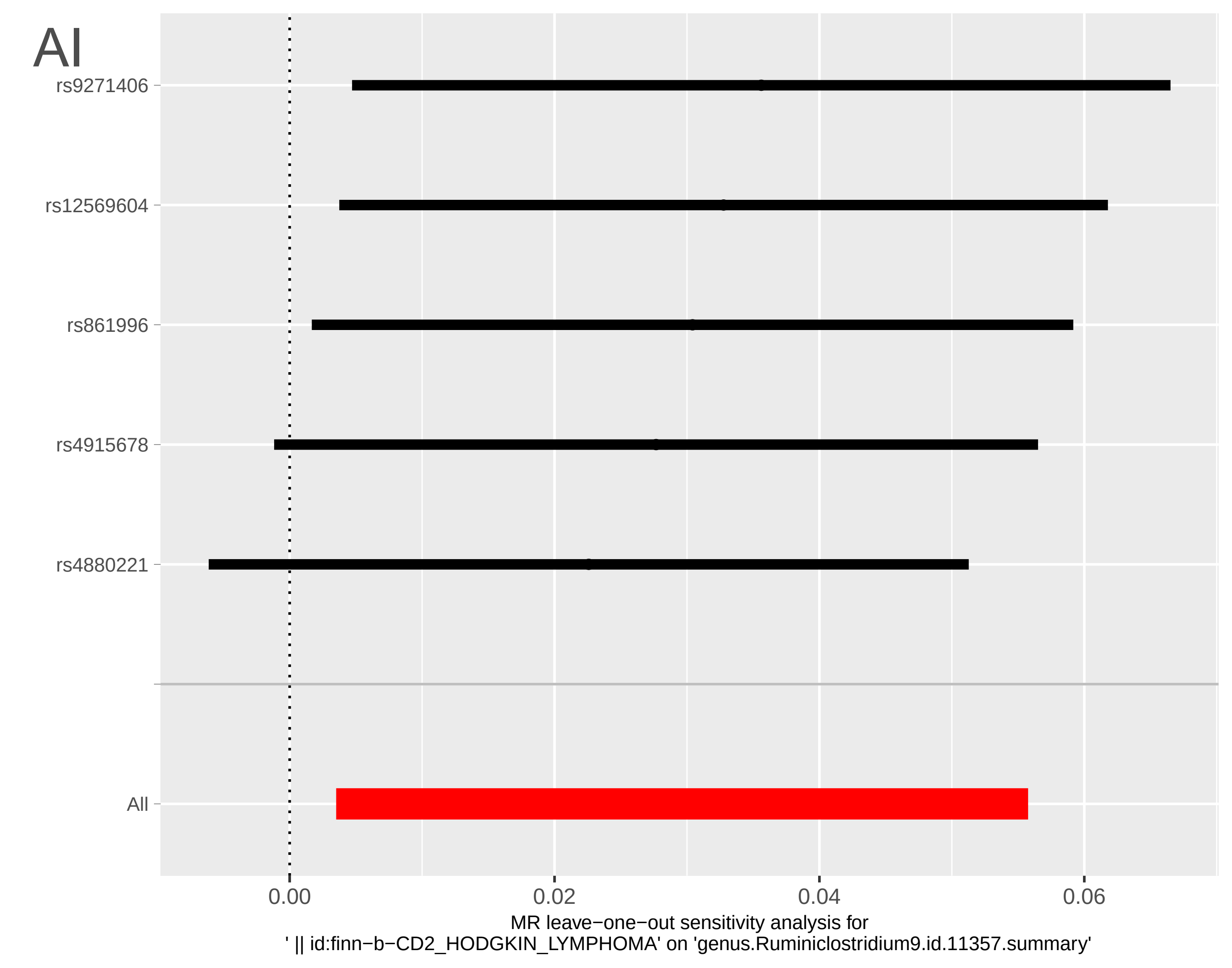
AI
rs9271406
rs12569604
rs861996
rs4915678
rs4880221
All
0.00
0.02
0.04
0.06
MR leave−one−out sensitivity analysis for
' || id:finn−b−CD2_HODGKIN_LYMPHOMA' on 'genus.Ruminiclostridium9.id.11357.summary'

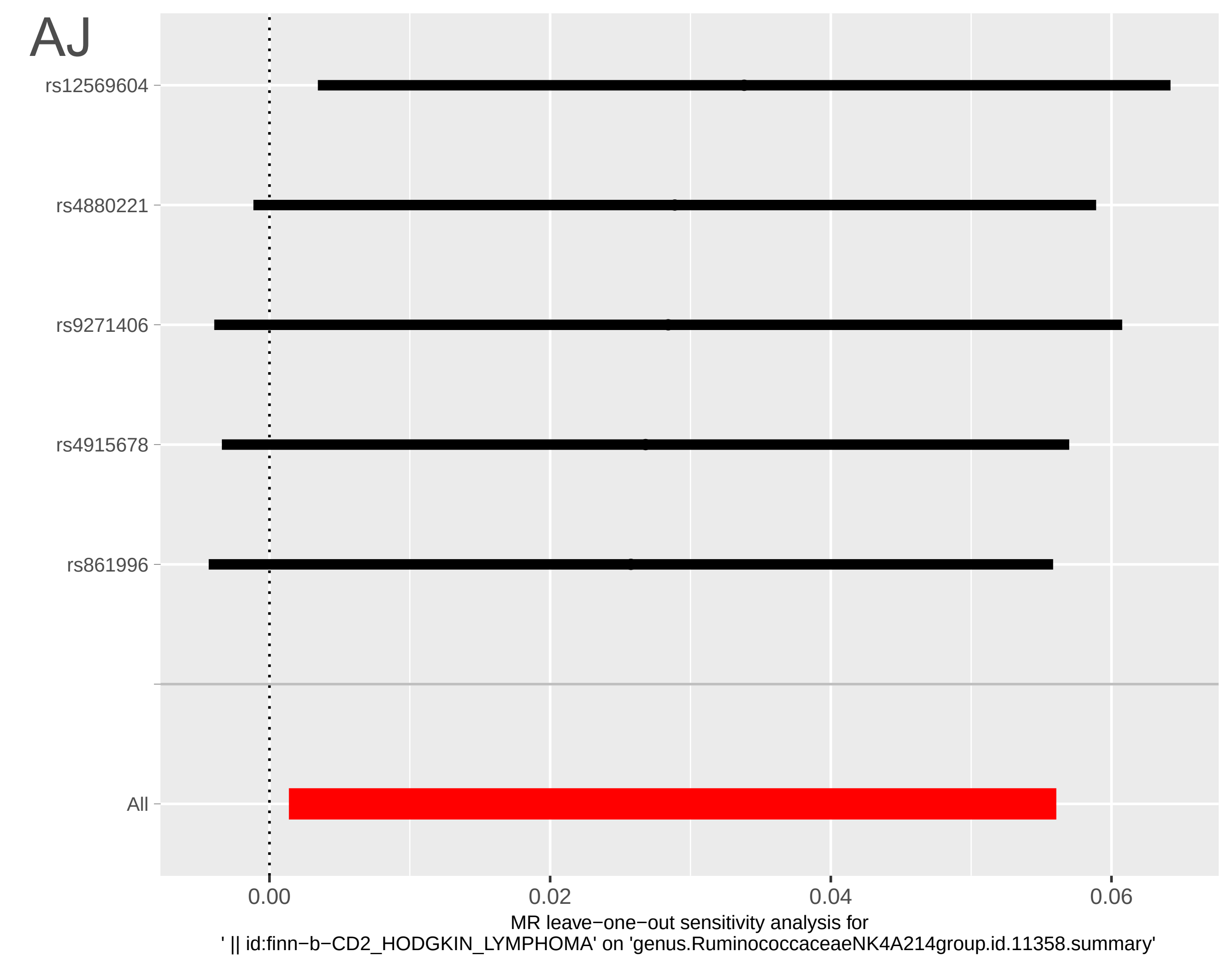
AJ
rs12569604
rs4880221
rs9271406
rs4915678
rs861996
All
0.00
0.02
0.04
0.06
MR leave−one−out sensitivity analysis for
' || id:finn−b−CD2_HODGKIN_LYMPHOMA' on 'genus.RuminococcaceaeNK4A214group.id.11358.summary'

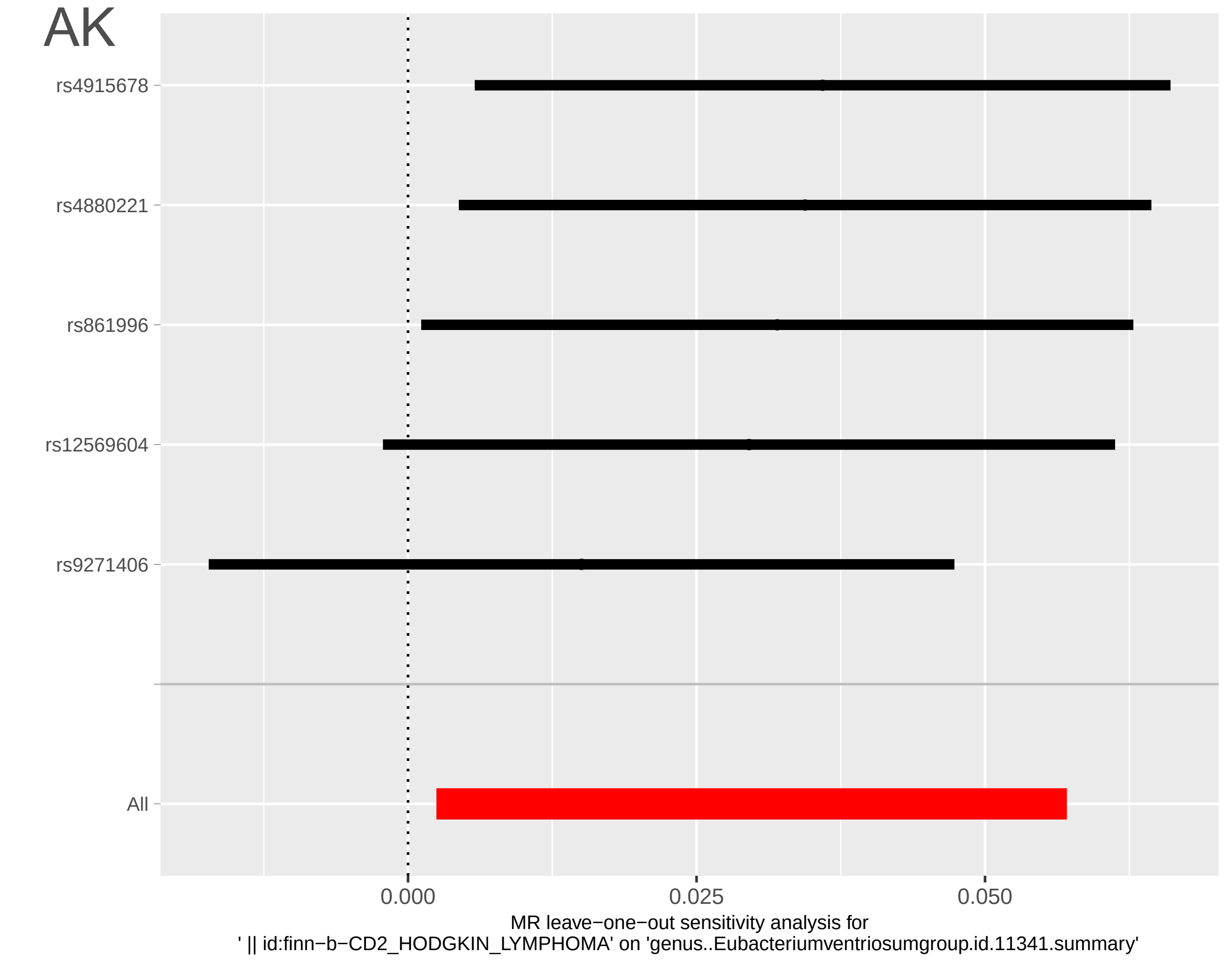
AK
rs4915678
rs4880221
rs861996
rs12569604
rs9271406
All
0.000
0.025
0.050
MR leave−one−out sensitivity analysis for
' || id:finn−b−CD2_HODGKIN_LYMPHOMA' on 'genus..Eubacteriumventriosumgroup.id.11341.summary'

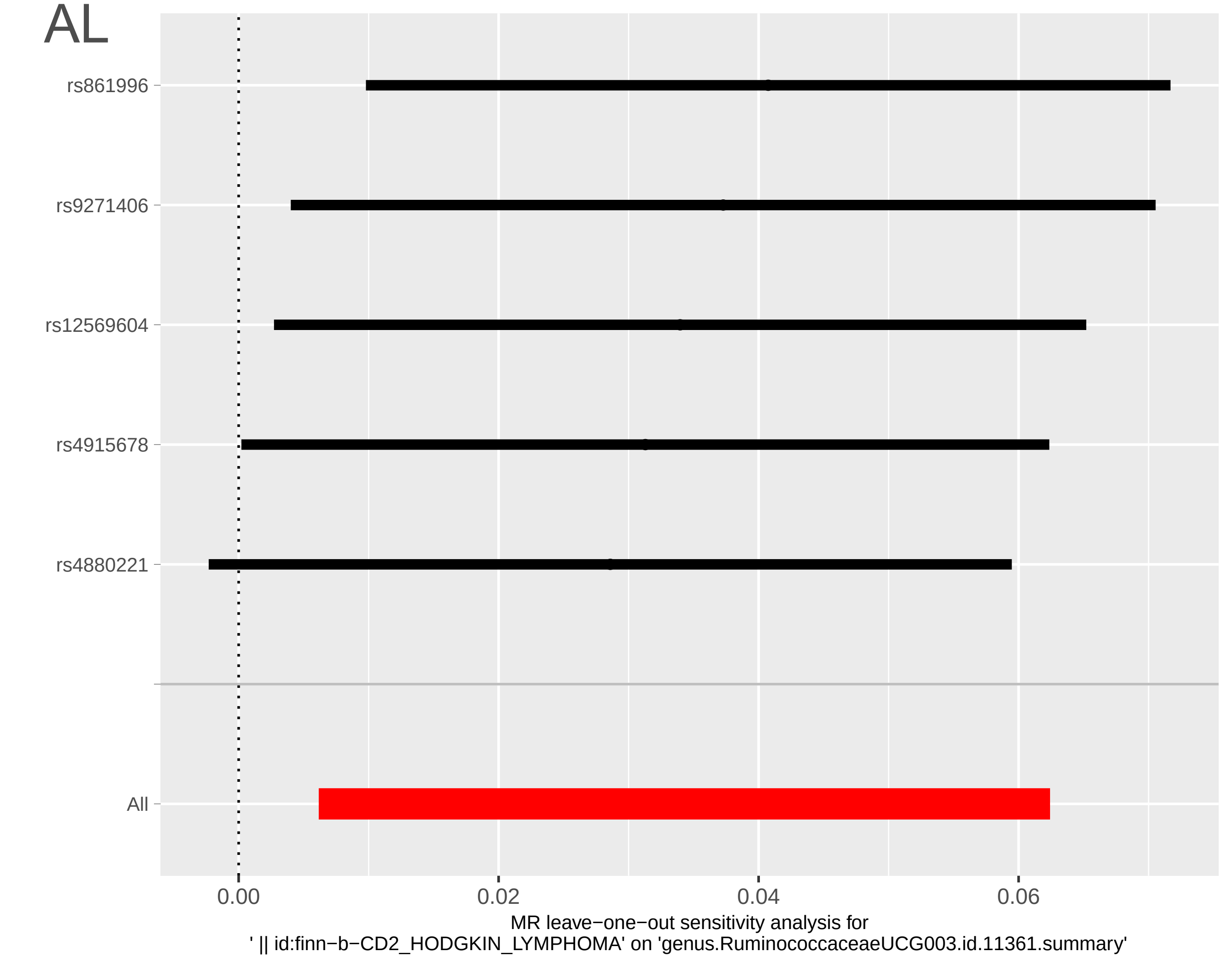
AL
rs861996
rs9271406
rs12569604
rs4915678
rs4880221
All
0.00
0.02
0.04
0.06
MR leave−one−out sensitivity analysis for
' || id:finn−b−CD2_HODGKIN_LYMPHOMA' on 'genus.RuminococcaceaeUCG003.id.11361.summary'

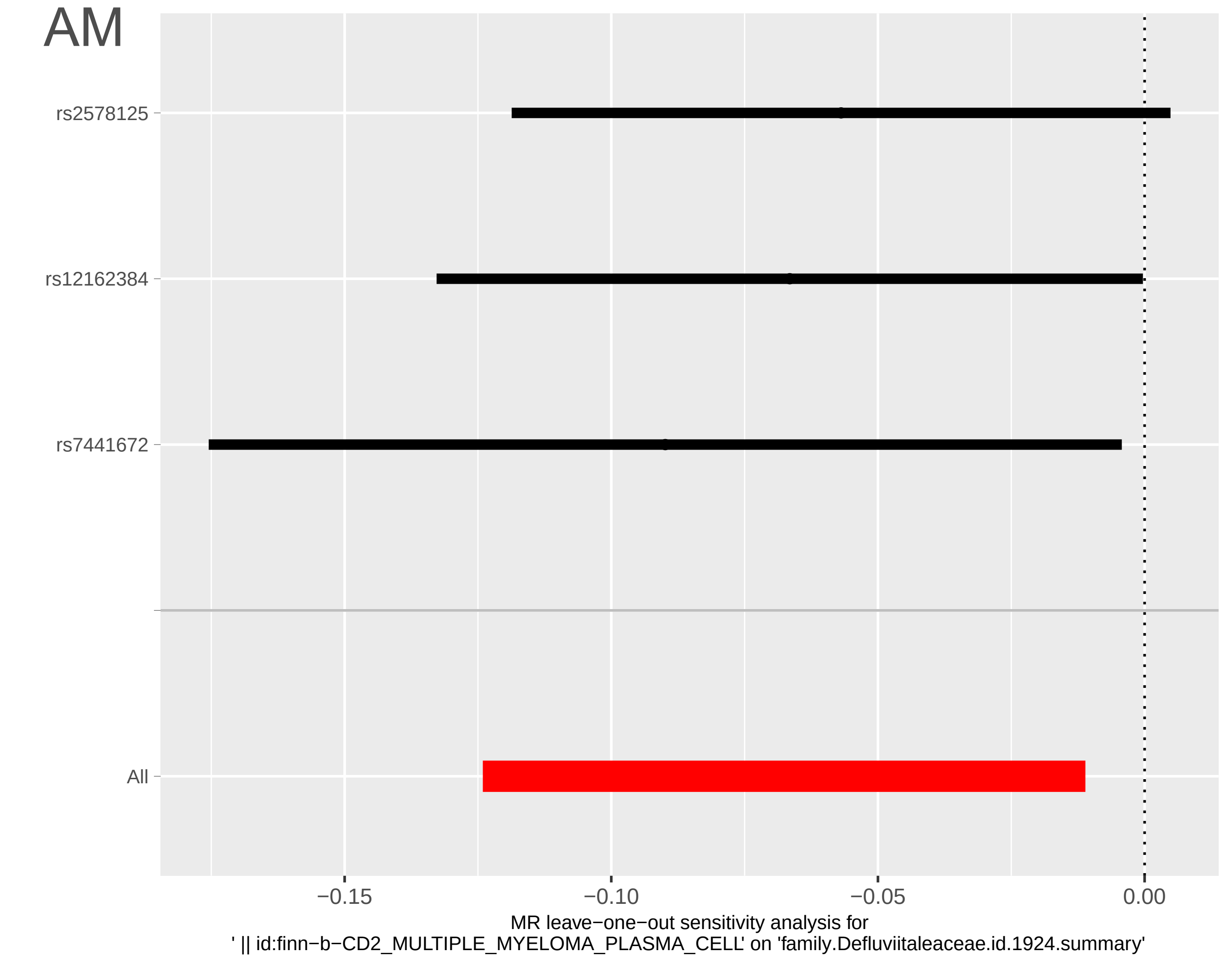

AM
rs2578125
rs12162384
rs7441672
All
−0.15
−0.10
−0.05
0.00
MR leave−one−out sensitivity analysis for
' || id:finn−b−CD2_MULTIPLE_MYELOMA_PLASMA_CELL' on 'family.Defluviitaleaceae.id.1924.summary'

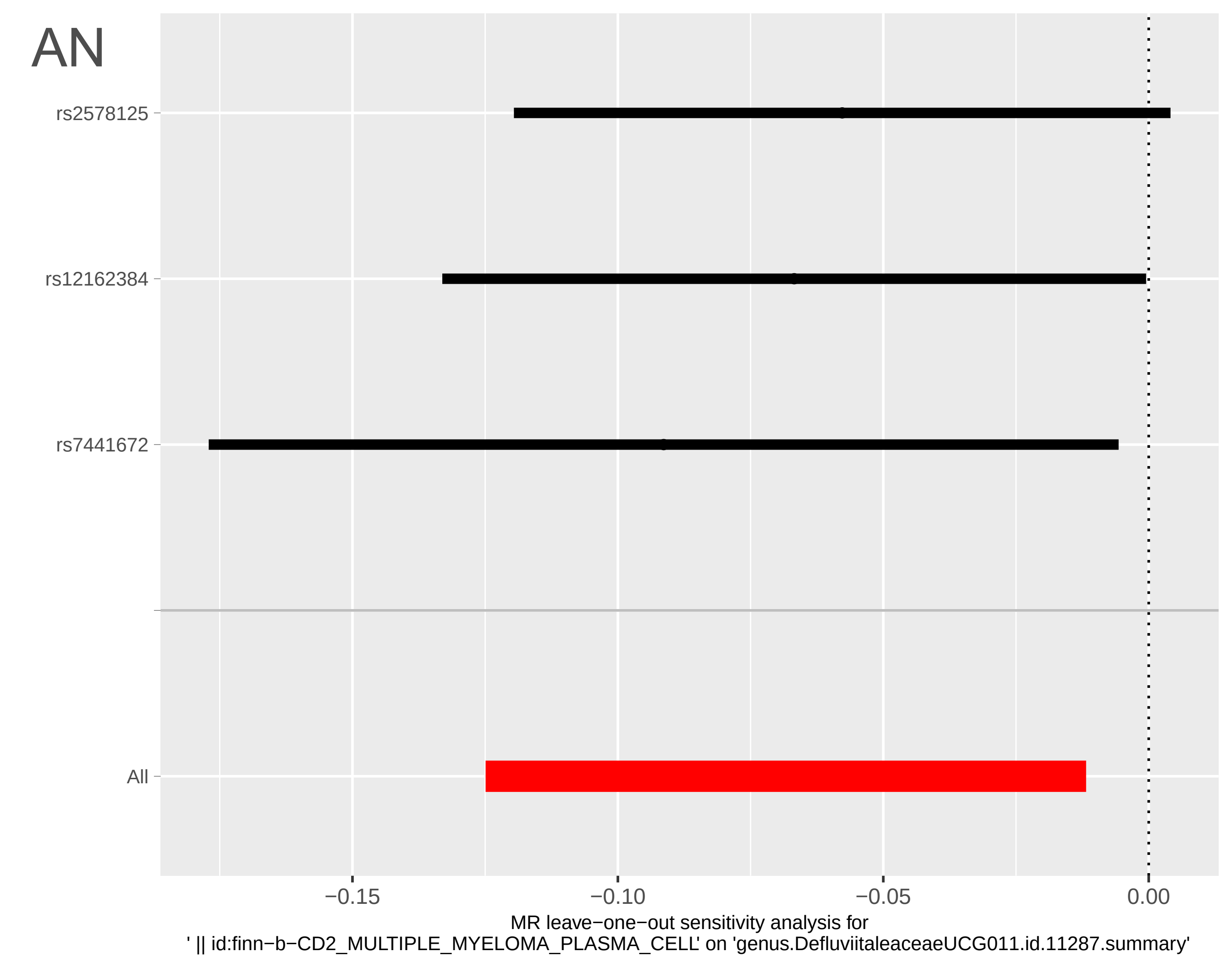
AN
rs2578125
rs12162384
rs7441672
All
−0.15
−0.10
−0.05
0.00
MR leave−one−out sensitivity analysis for
' || id:finn−b−CD2_MULTIPLE_MYELOMA_PLASMA_CELL' on 'genus.DefluviitaleaceaeUCG011.id.11287.summary'

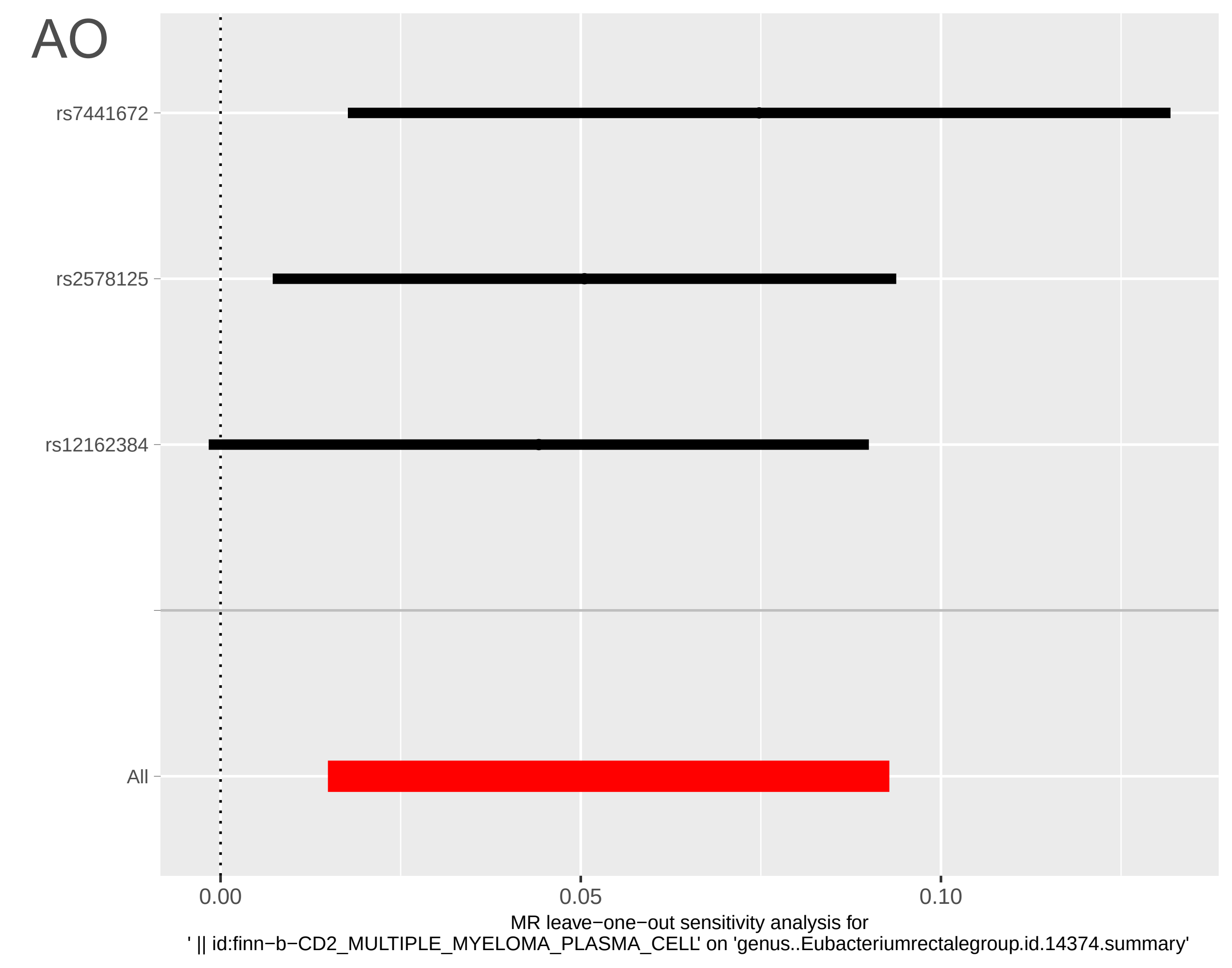
AO
rs7441672
rs2578125
rs12162384
All
0.00
0.05
0.10
MR leave−one−out sensitivity analysis for
' || id:finn−b−CD2_MULTIPLE_MYELOMA_PLASMA_CELL' on 'genus..Eubacteriumrectalegroup.id.14374.summary'

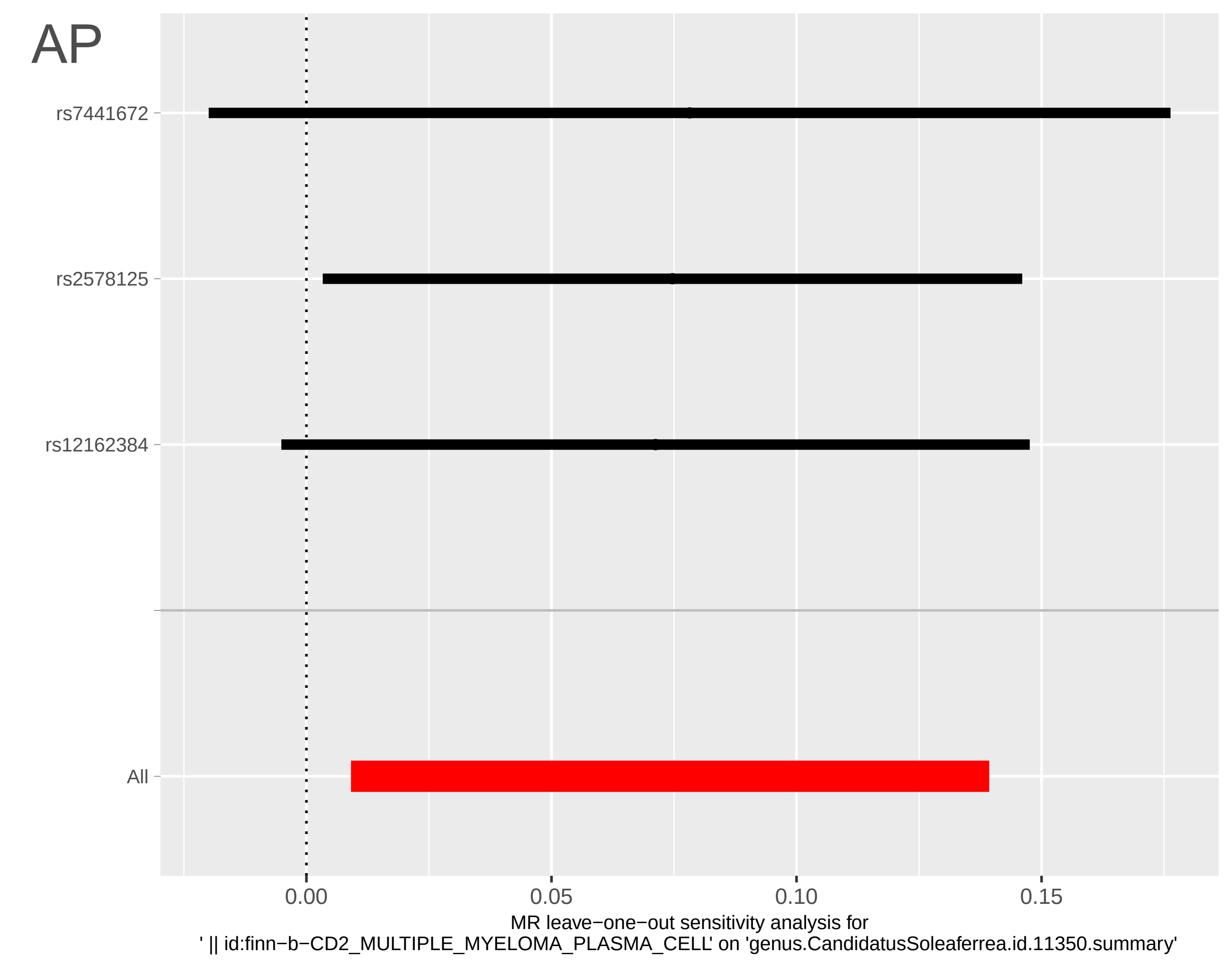
AP
rs7441672
rs2578125
rs12162384
All
0.00
0.05
0.10
0.15
MR leave−one−out sensitivity analysis for
' || id:finn−b−CD2_MULTIPLE_MYELOMA_PLASMA_CELL' on 'genus.CandidatusSoleaferrea.id.11350.summary'

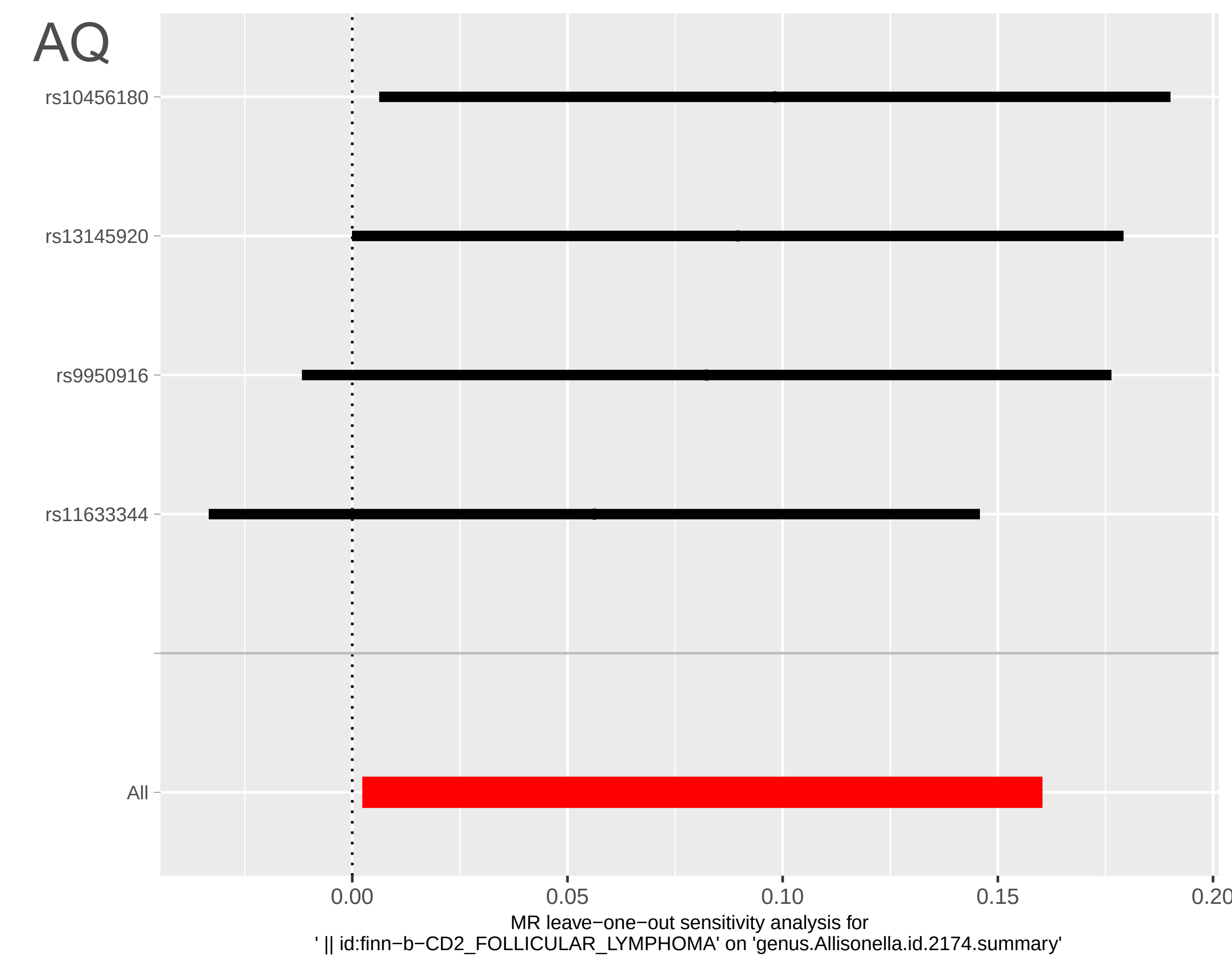
AQ
rs10456180
rs13145920
rs9950916
rs11633344
All
0.00
0.05
0.10
0.15
0.20
MR leave−one−out sensitivity analysis for
' || id:finn−b−CD2_FOLLICULAR_LYMPHOMA' on 'genus.Allisonella.id.2174.summary'

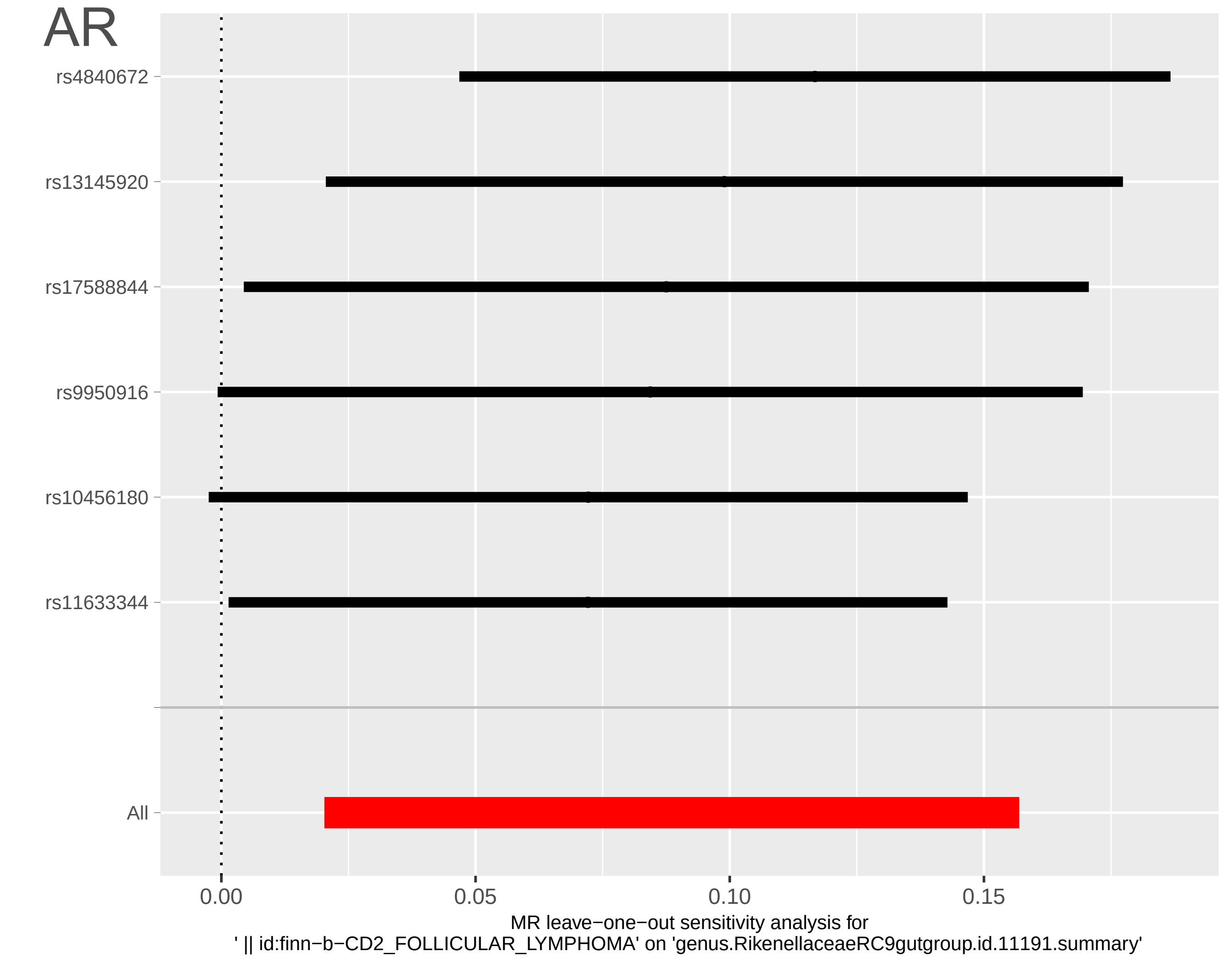
AR
rs4840672
rs13145920
rs17588844
rs9950916
rs10456180
rs11633344
All
0.00
0.05
0.10
0.15
MR leave−one−out sensitivity analysis for
' || id:finn−b−CD2_FOLLICULAR_LYMPHOMA' on 'genus.RikenellaceaeRC9gutgroup.id.11191.summary'

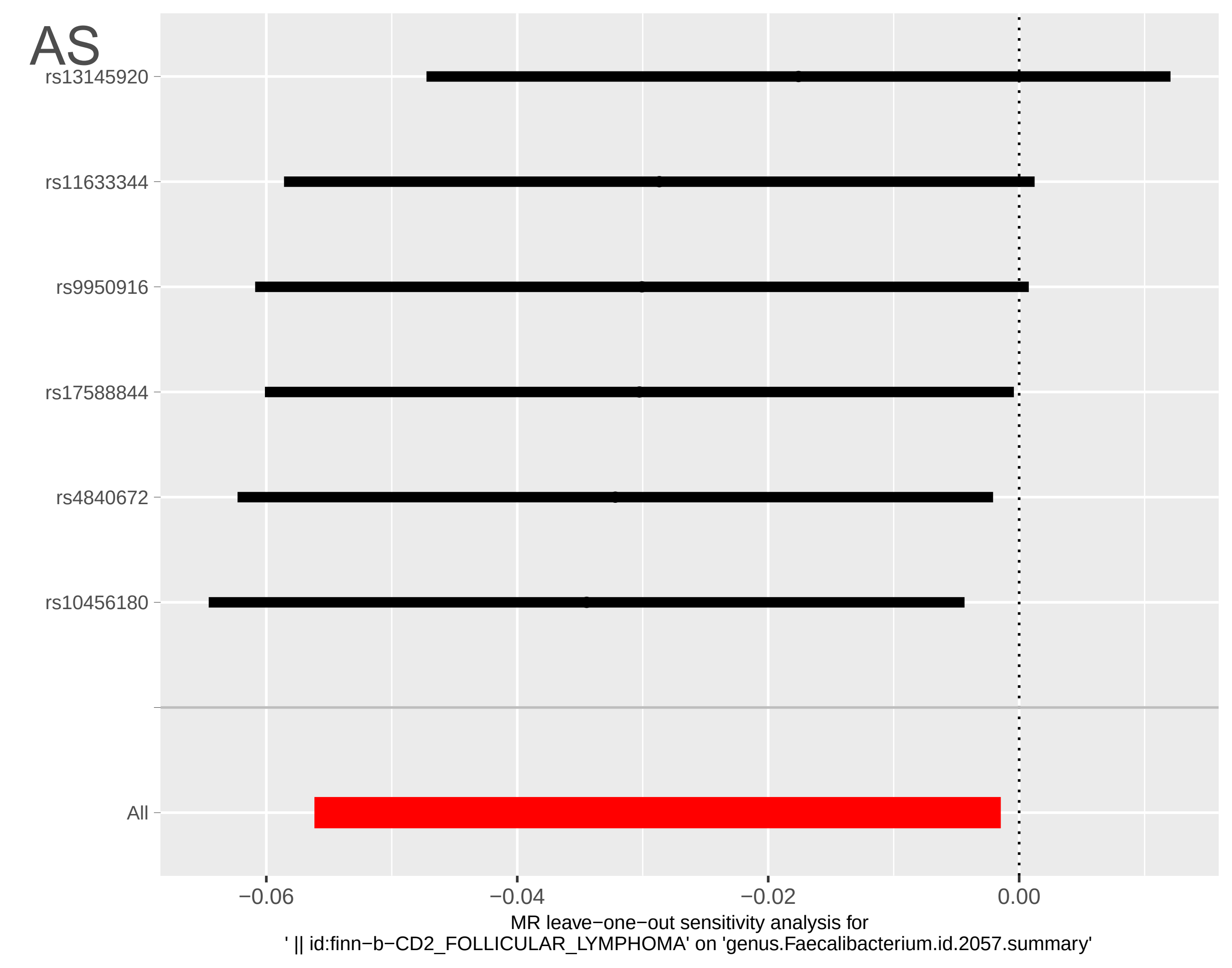
AS
rs13145920
rs11633344
rs9950916
rs17588844
rs4840672
rs10456180
All
−0.06
−0.04
−0.02
0.00
MR leave−one−out sensitivity analysis for
' || id:finn−b−CD2_FOLLICULAR_LYMPHOMA' on 'genus.Faecalibacterium.id.2057.summary'

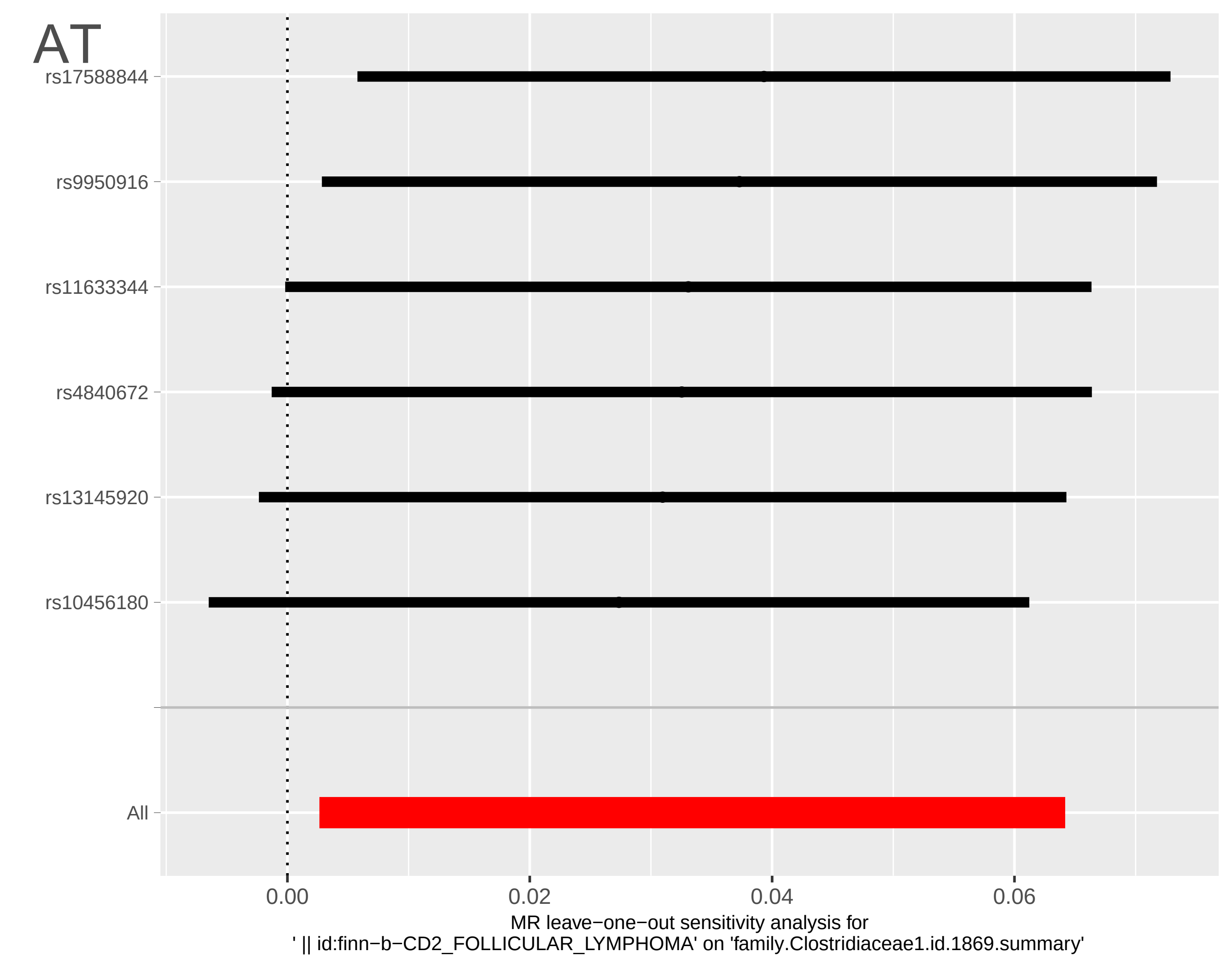
AT
rs17588844
rs9950916
rs11633344
rs4840672
rs13145920
rs10456180
All
0.00
0.02
0.04
0.06
MR leave−one−out sensitivity analysis for
' || id:finn−b−CD2_FOLLICULAR_LYMPHOMA' on 'family.Clostridiaceae1.id.1869.summary'

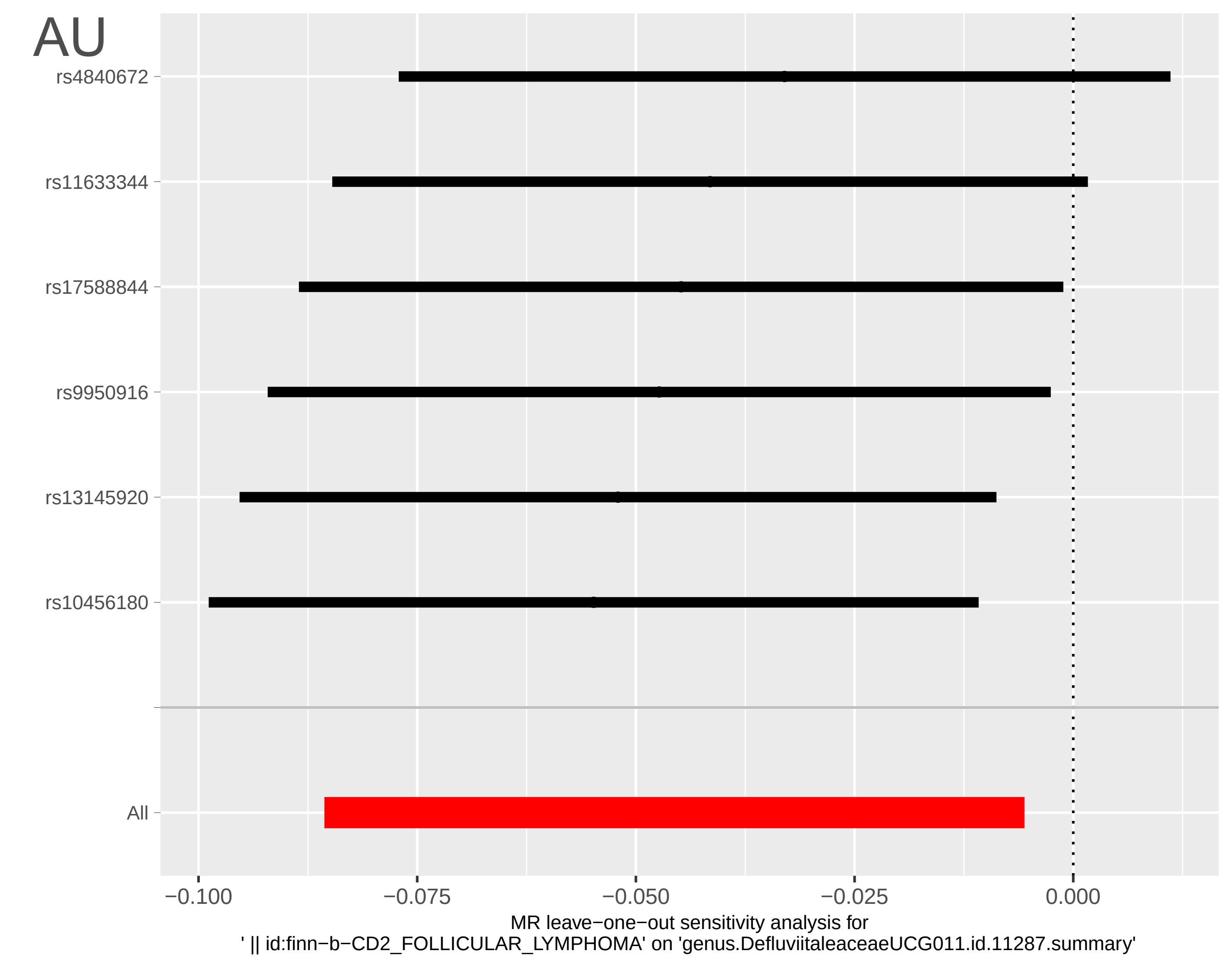

AU
rs4840672
rs11633344
rs17588844
rs9950916
rs13145920
rs10456180
All
−0.100
−0.075
−0.050
−0.025
0.000
MR leave−one−out sensitivity analysis for
' || id:finn−b−CD2_FOLLICULAR_LYMPHOMA' on 'genus.DefluviitaleaceaeUCG011.id.11287.summary'

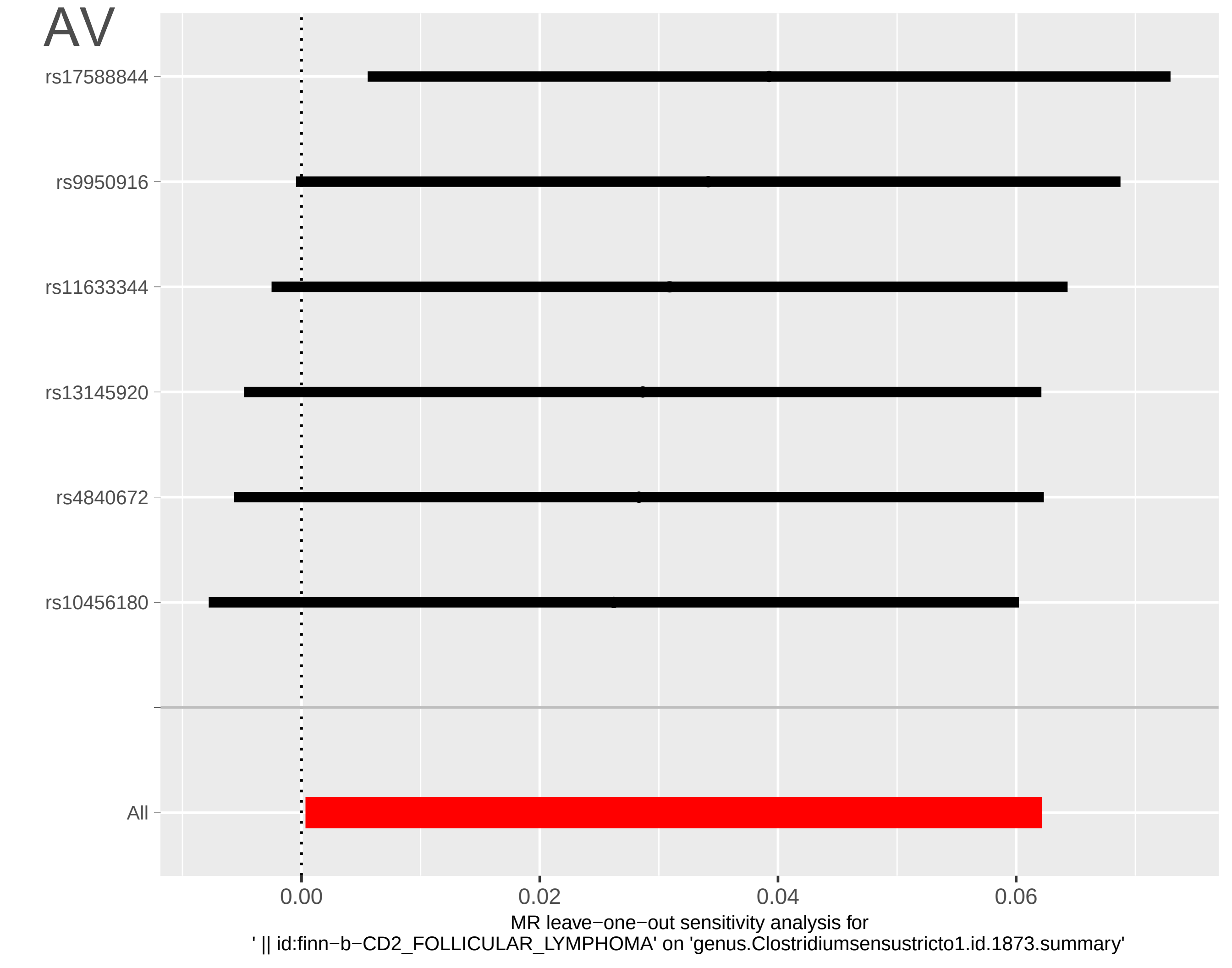
AV
rs17588844
rs9950916
rs11633344
rs13145920
rs4840672
rs10456180
All
0.00
0.02
0.04
0.06
MR leave−one−out sensitivity analysis for
' || id:finn−b−CD2_FOLLICULAR_LYMPHOMA' on 'genus.Clostridiumsensustricto1.id.1873.summary'

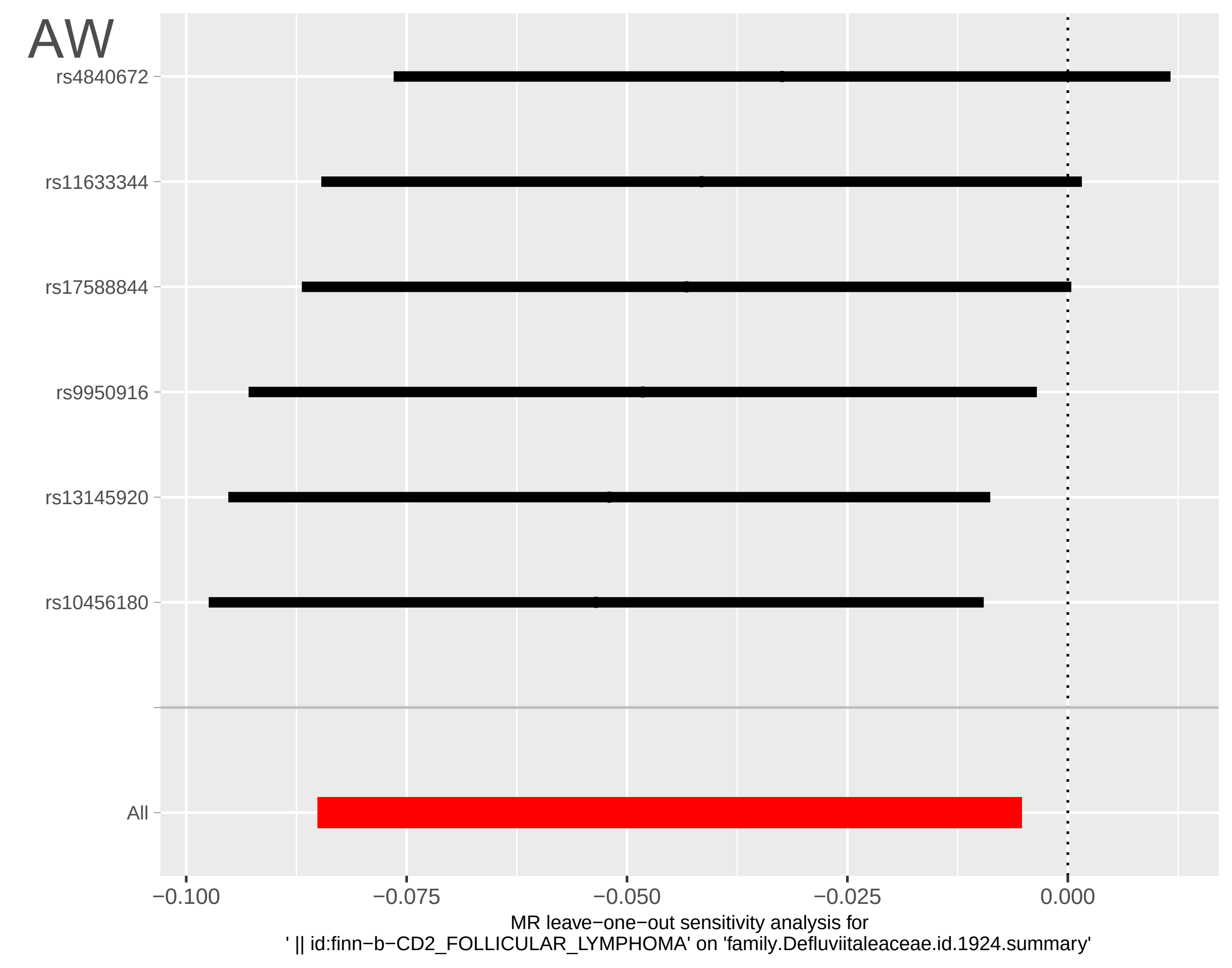
AW
rs4840672
rs11633344
rs17588844
rs9950916
rs13145920
rs10456180
All
−0.100
−0.075
−0.050
−0.025
0.000
MR leave−one−out sensitivity analysis for
' || id:finn−b−CD2_FOLLICULAR_LYMPHOMA' on 'family.Defluviitaleaceae.id.1924.summary'

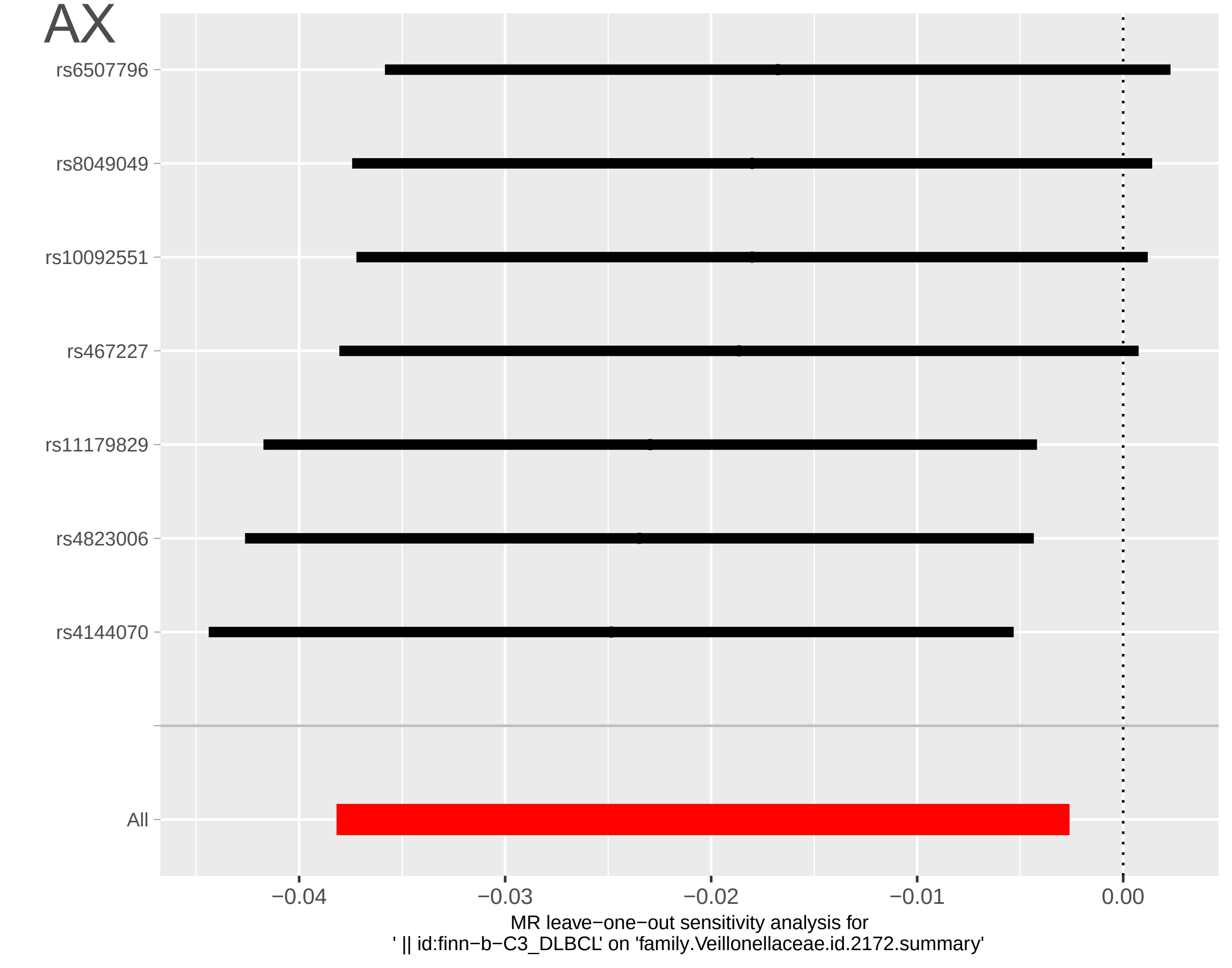
AX
rs6507796
rs8049049
rs10092551
rs467227
rs11179829
rs4823006
rs4144070
All
−0.04
−0.03
−0.02
−0.01
0.00
MR leave−one−out sensitivity analysis for
' || id:finn−b−C3_DLBCL' on 'family.Veillonellaceae.id.2172.summary'

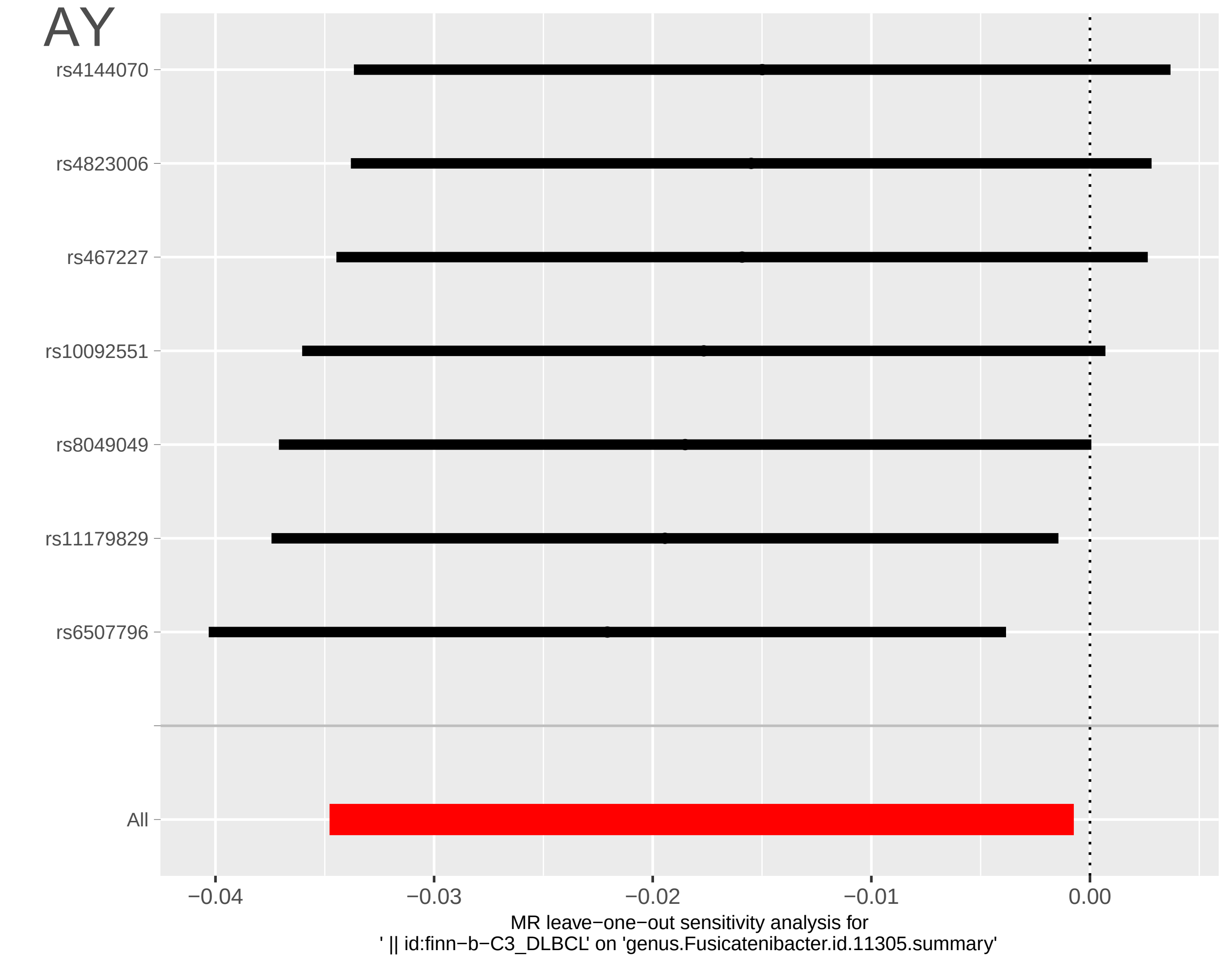
AY
rs4144070
rs4823006
rs467227
rs10092551
rs8049049
rs11179829
rs6507796
All
−0.04
−0.03
−0.02
−0.01
0.00
MR leave−one−out sensitivity analysis for
' || id:finn−b−C3_DLBCL' on 'genus.Fusicatenibacter.id.11305.summary'

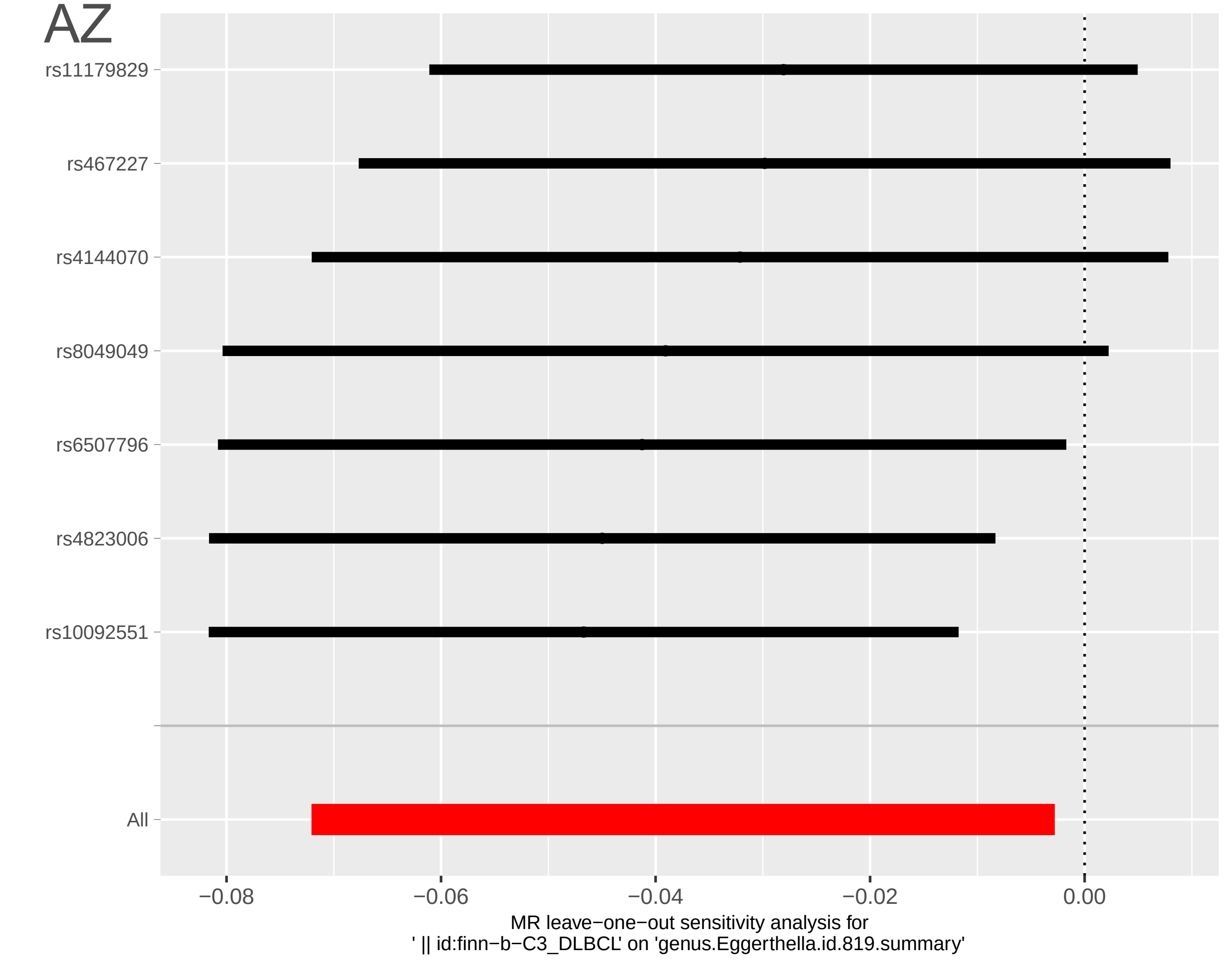
AZ
rs11179829
rs467227
rs4144070
rs8049049
rs6507796
rs4823006
rs10092551
All
−0.08
−0.06
−0.04
−0.02
0.00
MR leave−one−out sensitivity analysis for
' || id:finn−b−C3_DLBCL' on 'genus.Eggerthella.id.819.summary'

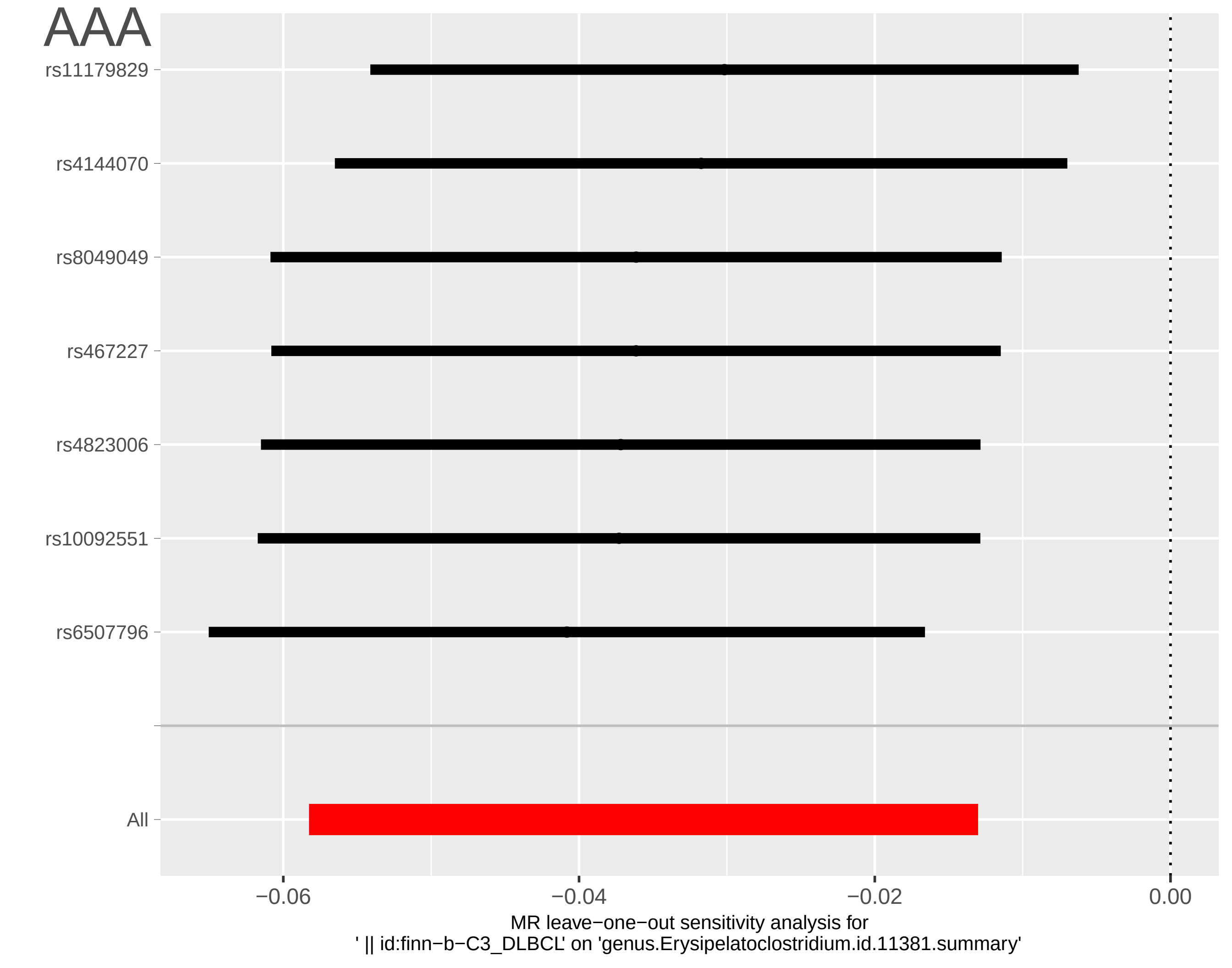
AAA
rs11179829
rs4144070
rs8049049
rs467227
rs4823006
rs10092551
rs6507796
All
−0.06
−0.04
−0.02
0.00
MR leave−one−out sensitivity analysis for
' || id:finn−b−C3_DLBCL' on 'genus.Erysipelatoclostridium.id.11381.summary'

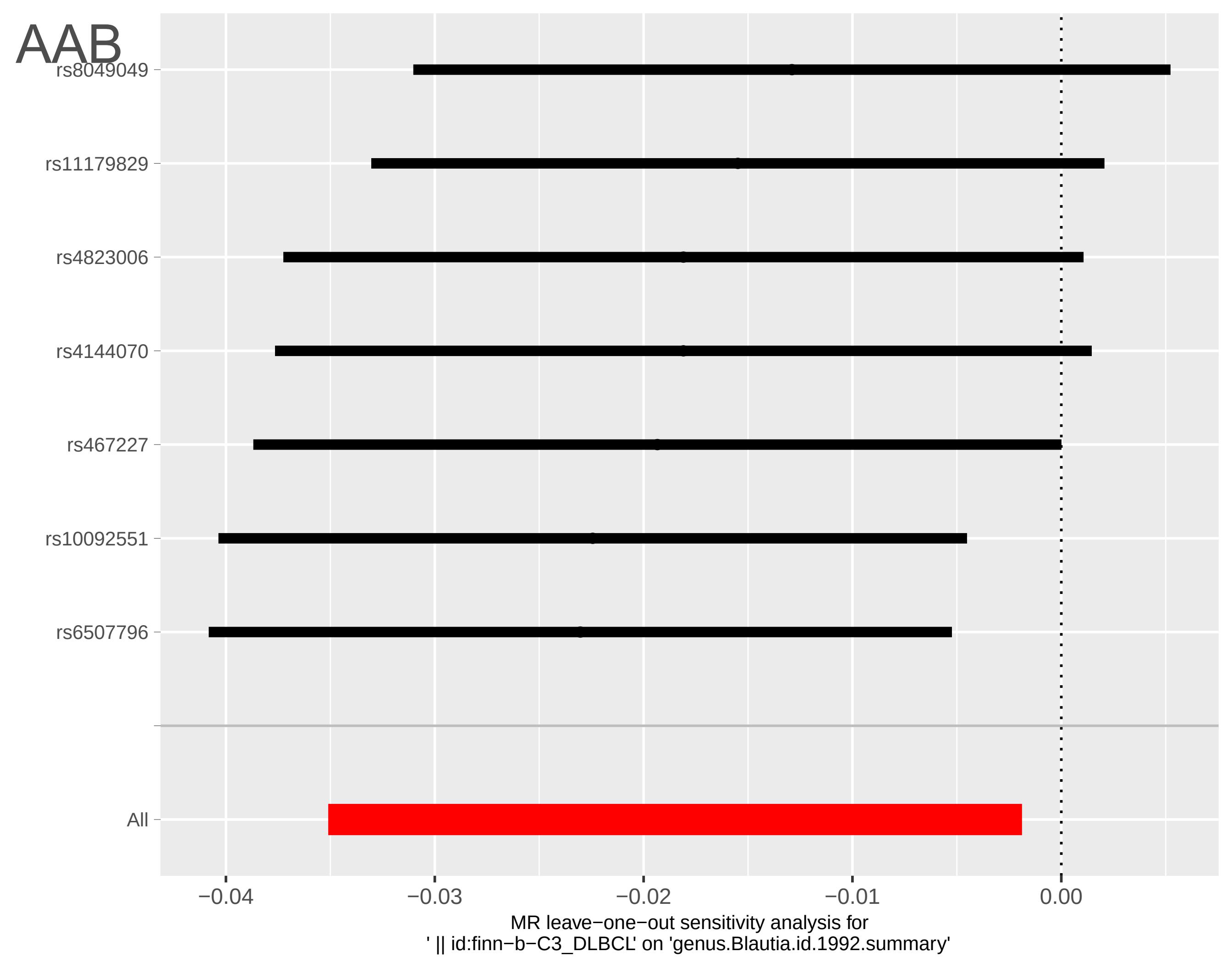
AAB
rs8049049
rs11179829
rs4823006
rs4144070
rs467227
rs10092551
rs6507796
All
−0.04
−0.03
−0.02
−0.01
0.00
MR leave−one−out sensitivity analysis for
' || id:finn−b−C3_DLBCL' on 'genus.Blautia.id.1992.summary'

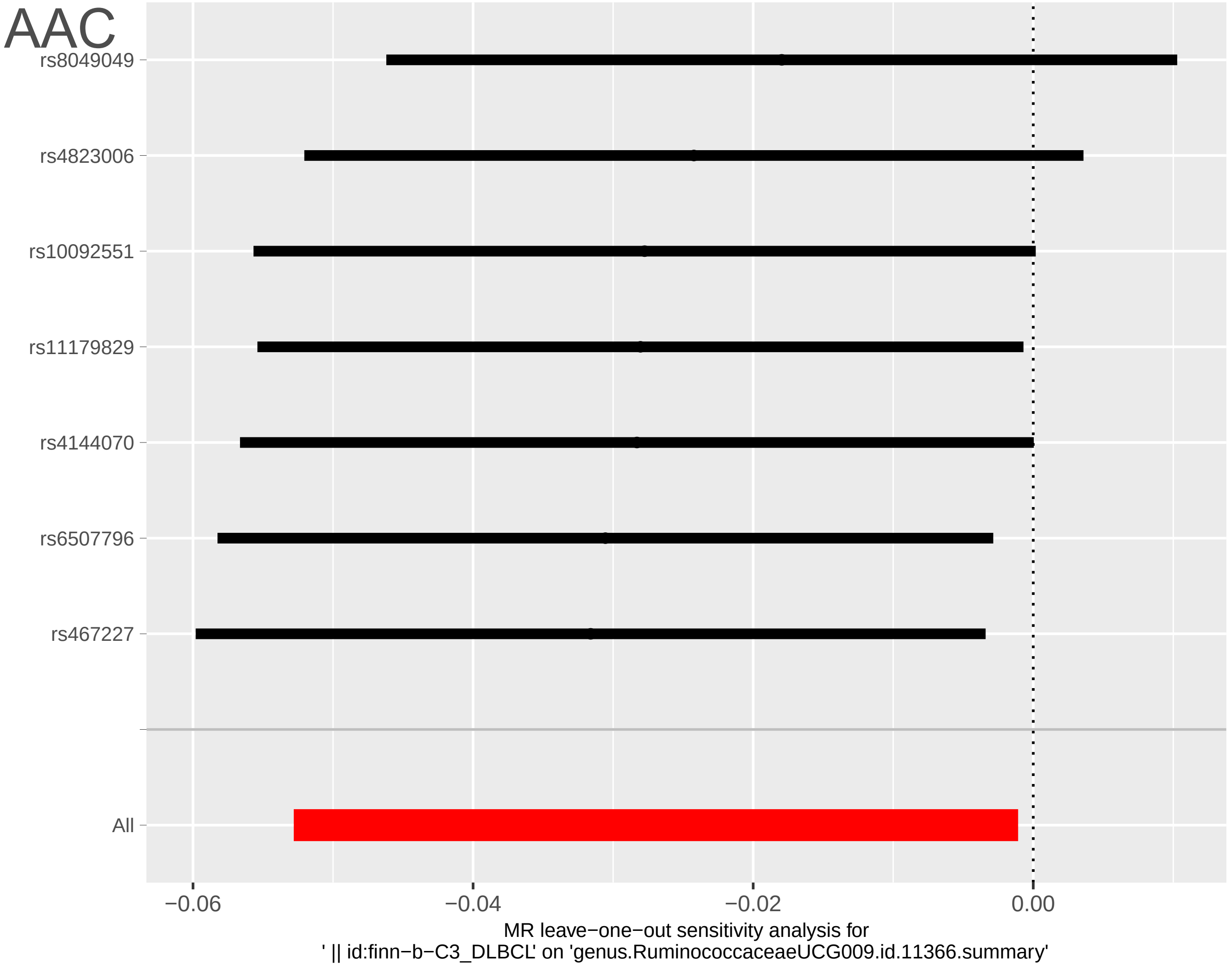
AAC
rs8049049
rs4823006
rs10092551
rs11179829
rs4144070
rs6507796
rs467227
All
−0.06
−0.04
−0.02
0.00
MR leave−one−out sensitivity analysis for
' || id:finn−b−C3_DLBCL' on 'genus.RuminococcaceaeUCG009.id.11366.summary'

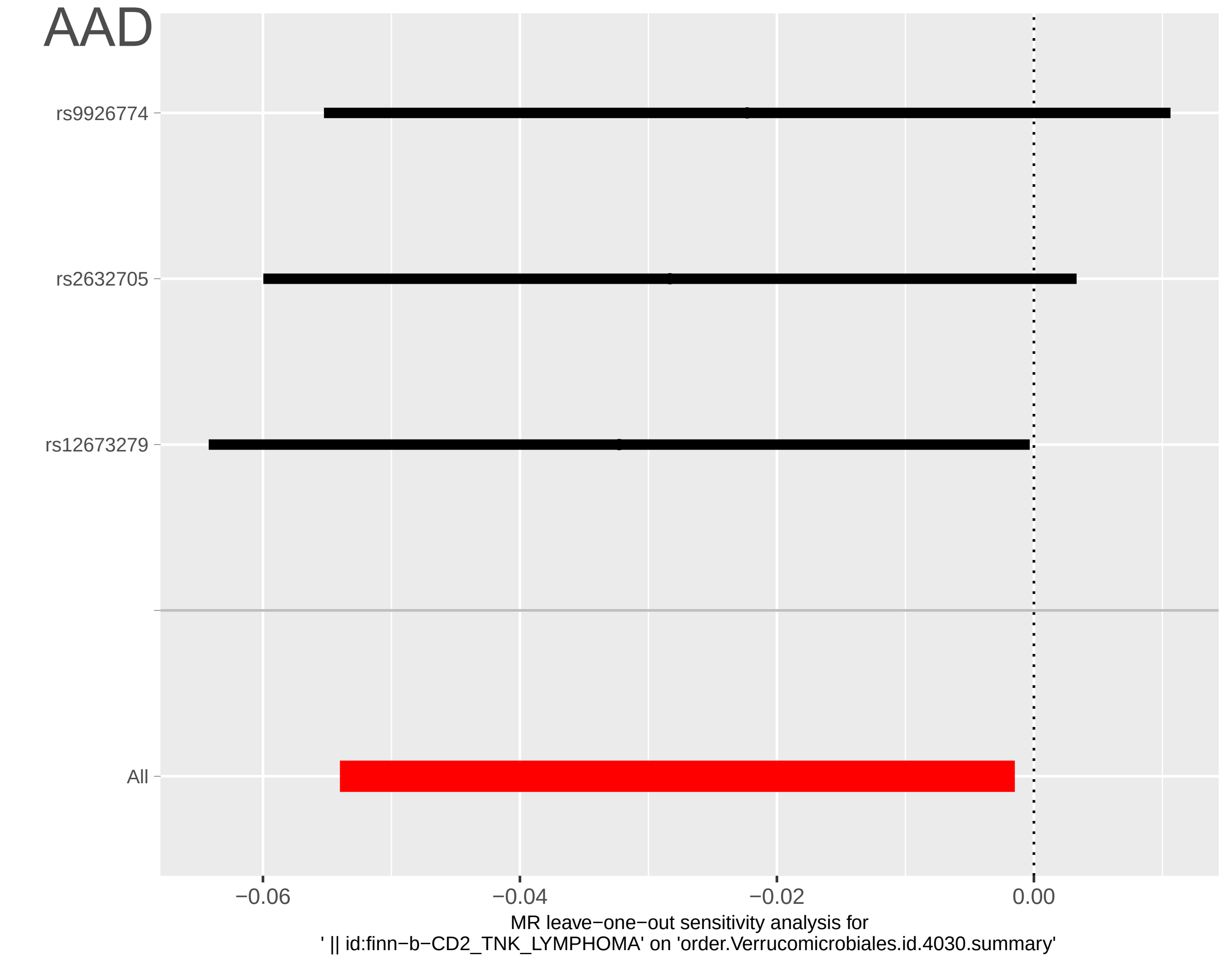
AAD
rs9926774
rs2632705
rs12673279
All
−0.06
−0.04
−0.02
0.00
MR leave−one−out sensitivity analysis for
' || id:finn−b−CD2_TNK_LYMPHOMA' on 'order.Verrucomicrobiales.id.4030.summary'

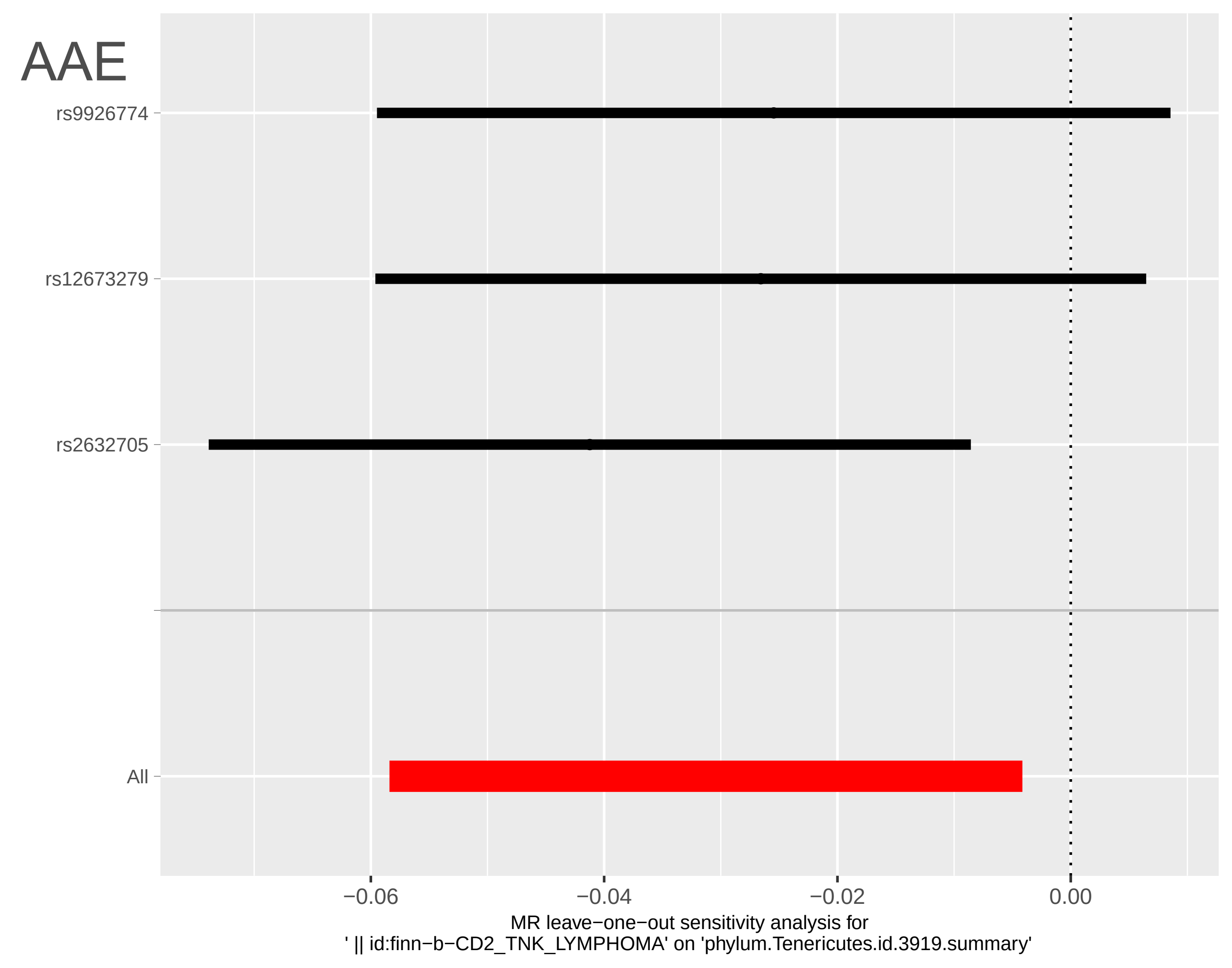
AAE
rs9926774
rs12673279
rs2632705
All
−0.06
−0.04
−0.02
0.00
MR leave−one−out sensitivity analysis for
' || id:finn−b−CD2_TNK_LYMPHOMA' on 'phylum.Tenericutes.id.3919.summary'

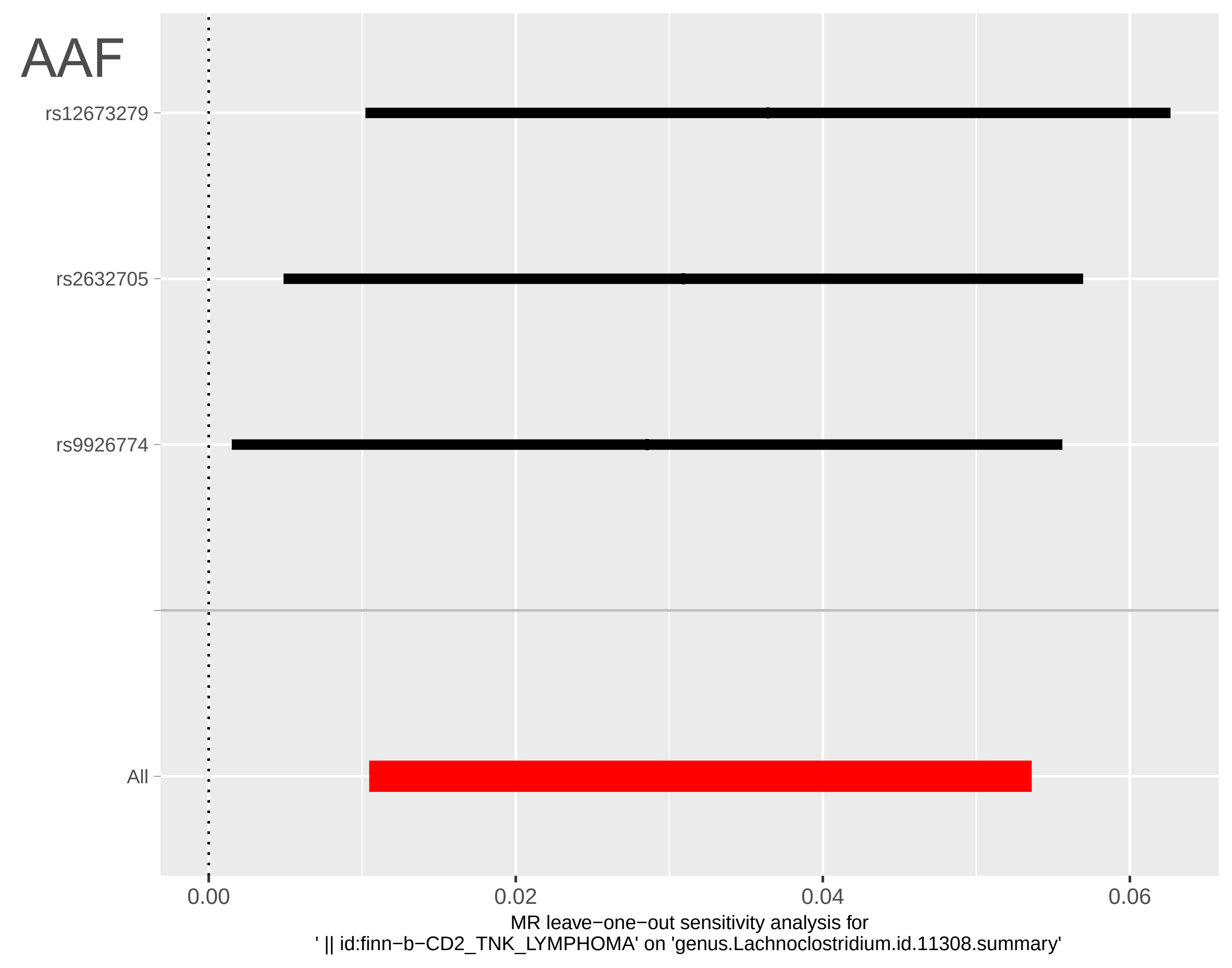
AAF
rs12673279
rs2632705
rs9926774
All
0.00
0.02
0.04
0.06
MR leave−one−out sensitivity analysis for
' || id:finn−b−CD2_TNK_LYMPHOMA' on 'genus.Lachnoclostridium.id.11308.summary'

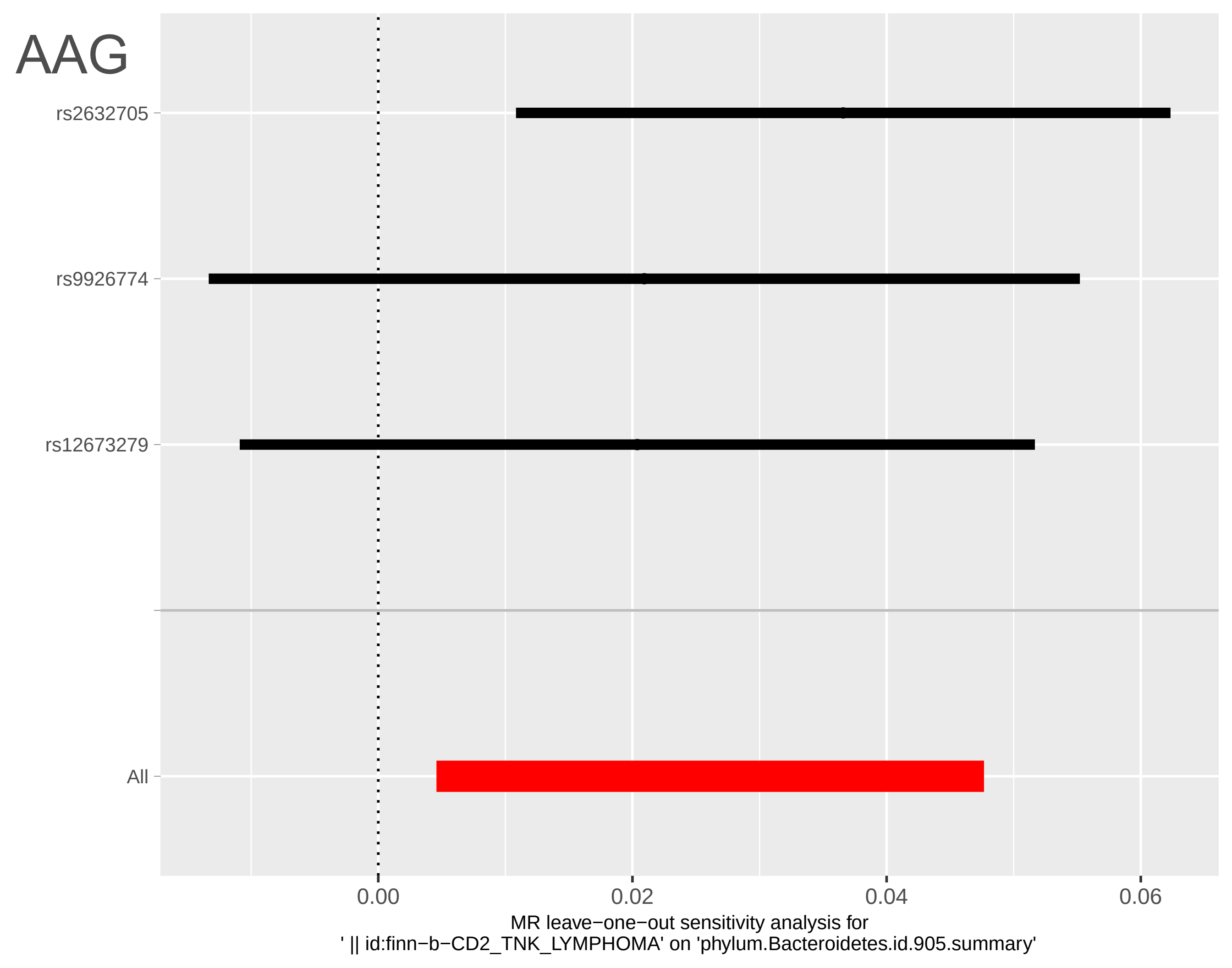
AAG
rs2632705
rs9926774
rs12673279
All
0.00
0.02
0.04
0.06
MR leave−one−out sensitivity analysis for
' || id:finn−b−CD2_TNK_LYMPHOMA' on 'phylum.Bacteroidetes.id.905.summary'

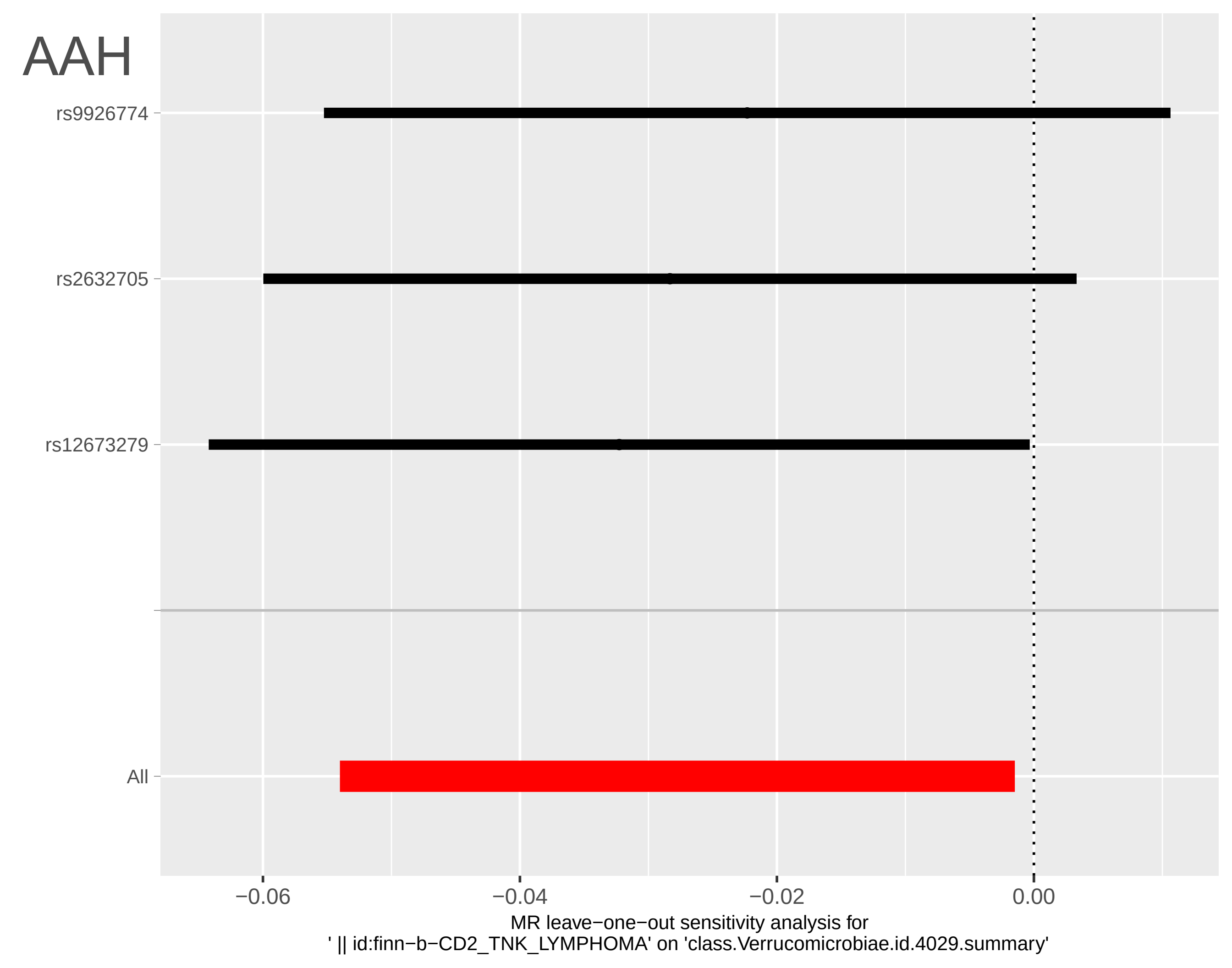
AAH
rs9926774
rs2632705
rs12673279
All
−0.06
−0.04
−0.02
0.00
MR leave−one−out sensitivity analysis for
' || id:finn−b−CD2_TNK_LYMPHOMA' on 'class.Verrucomicrobiae.id.4029.summary'

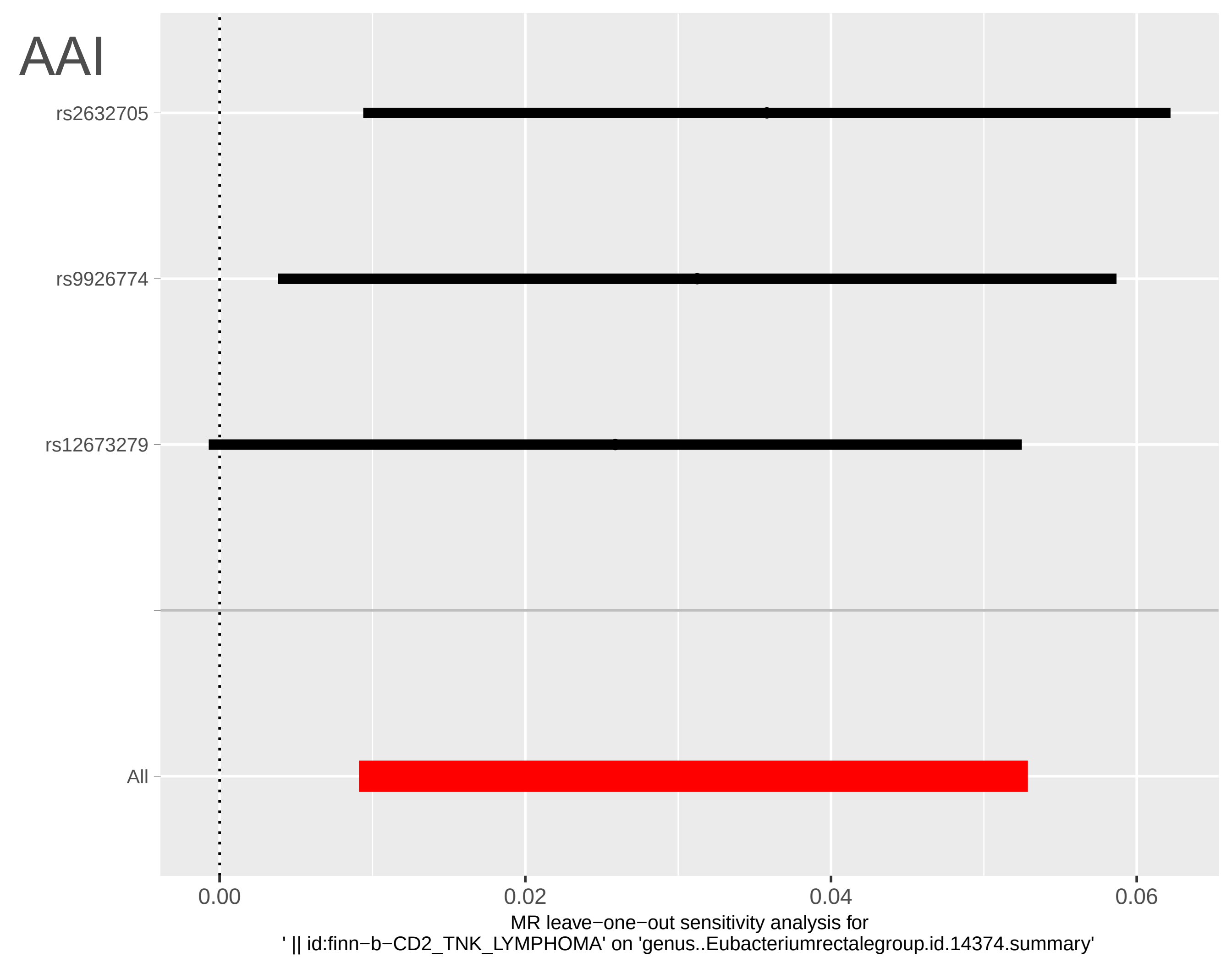
AAI
rs2632705
rs9926774
rs12673279
All
0.00
0.02
0.04
0.06
MR leave−one−out sensitivity analysis for
' || id:finn−b−CD2_TNK_LYMPHOMA' on 'genus..Eubacteriumrectalegroup.id.14374.summary'

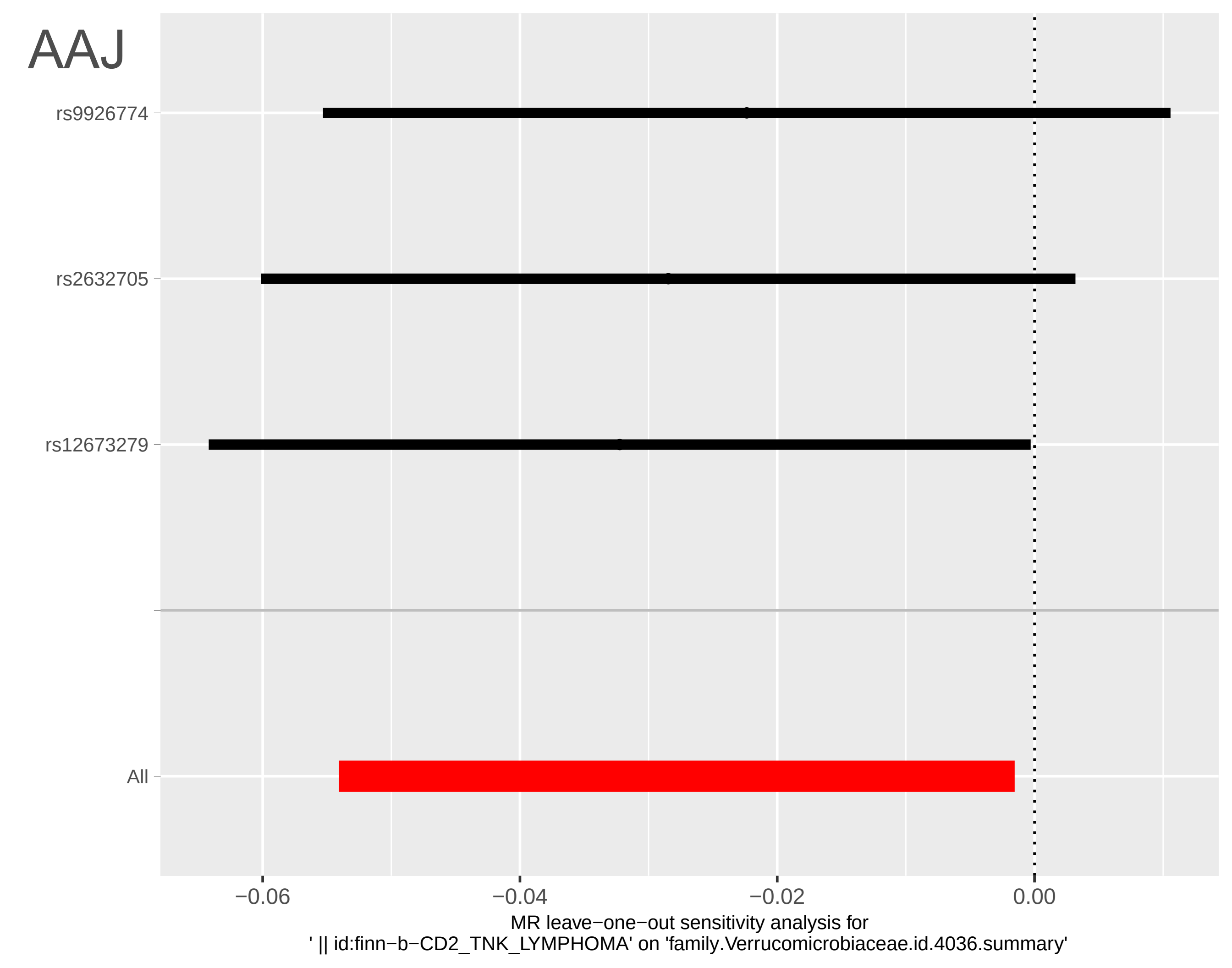
AAJ
rs9926774
rs2632705
rs12673279
All
−0.06
−0.04
−0.02
0.00
MR leave−one−out sensitivity analysis for
' || id:finn−b−CD2_TNK_LYMPHOMA' on 'family.Verrucomicrobiaceae.id.4036.summary'

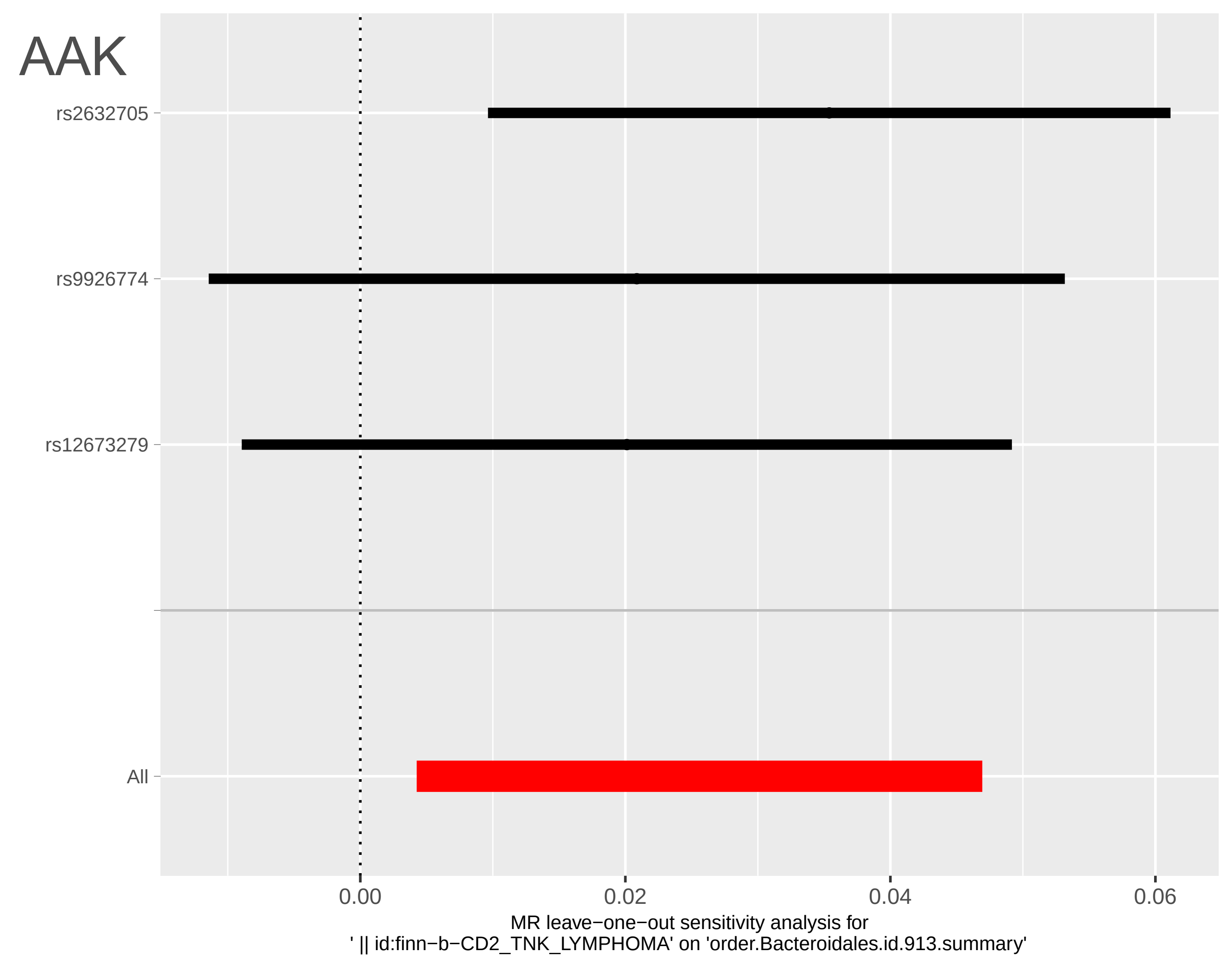

AAK
rs2632705
rs9926774
rs12673279
All
0.00
0.02
0.04
0.06
MR leave−one−out sensitivity analysis for
' || id:finn−b−CD2_TNK_LYMPHOMA' on 'order.Bacteroidales.id.913.summary'

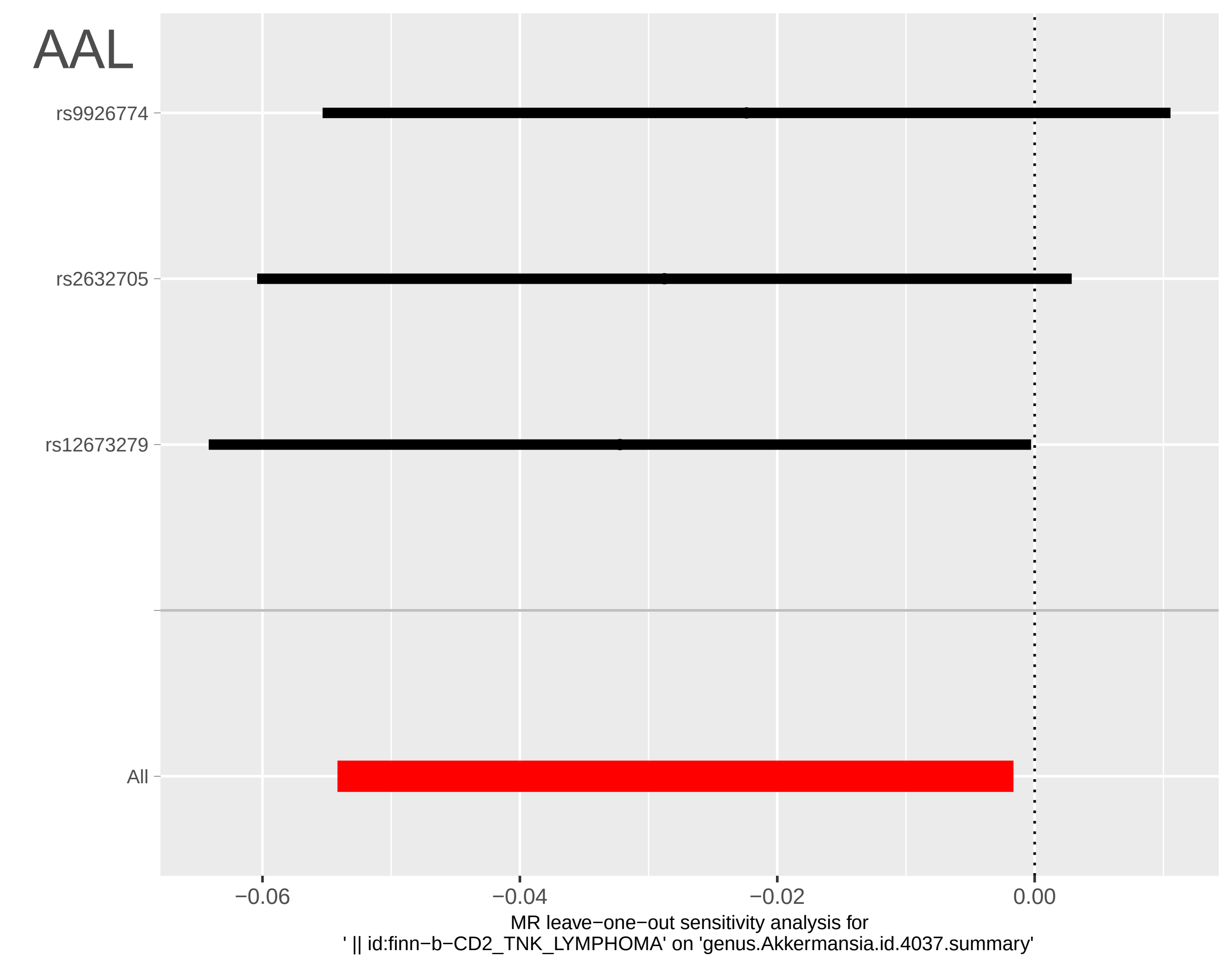
AAL
rs9926774
rs2632705
rs12673279
All
−0.06
−0.04
−0.02
0.00
MR leave−one−out sensitivity analysis for
' || id:finn−b−CD2_TNK_LYMPHOMA' on 'genus.Akkermansia.id.4037.summary'

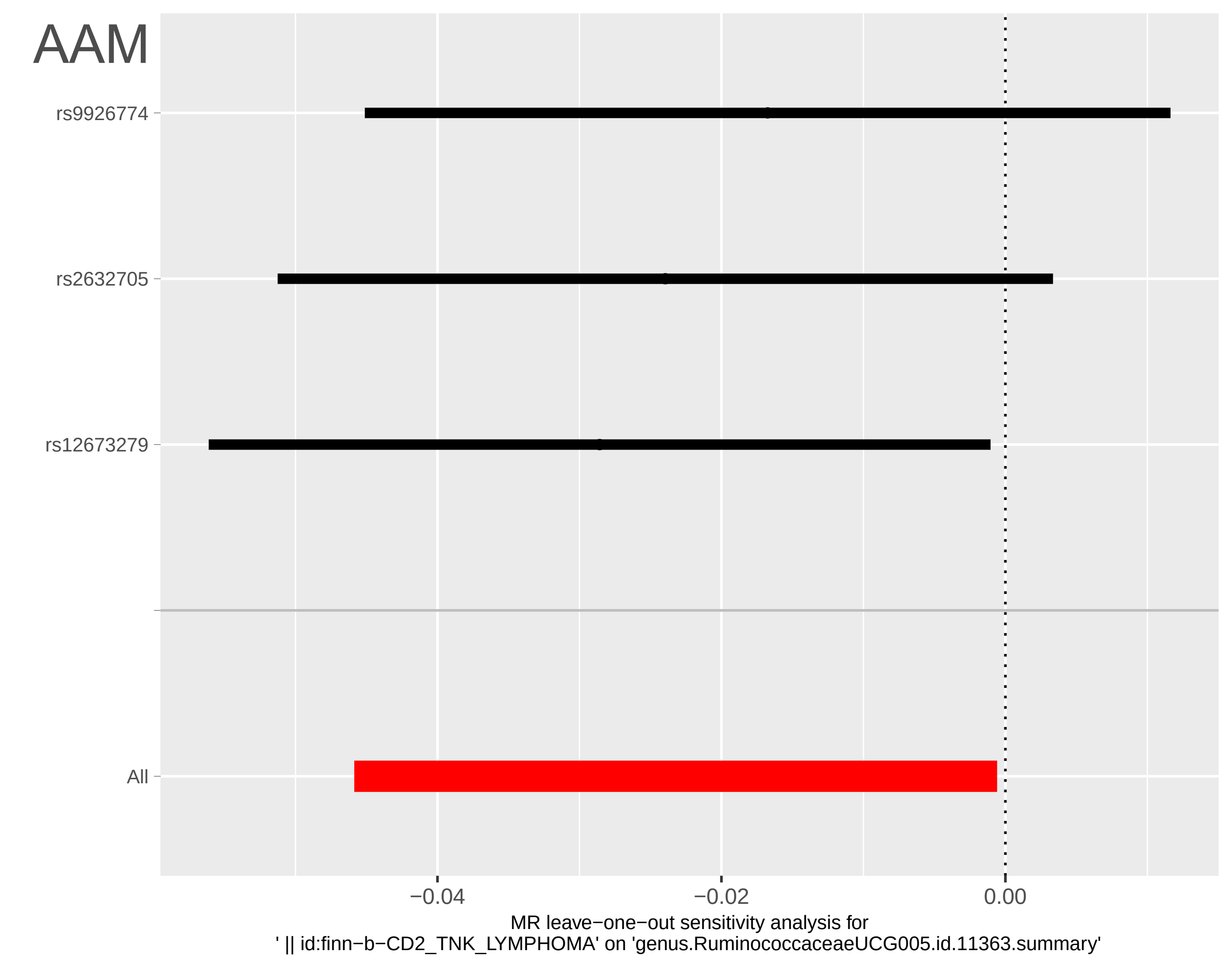
AAM
rs9926774
rs2632705
rs12673279
All
−0.04
−0.02
0.00
MR leave−one−out sensitivity analysis for
' || id:finn−b−CD2_TNK_LYMPHOMA' on 'genus.RuminococcaceaeUCG005.id.11363.summary'

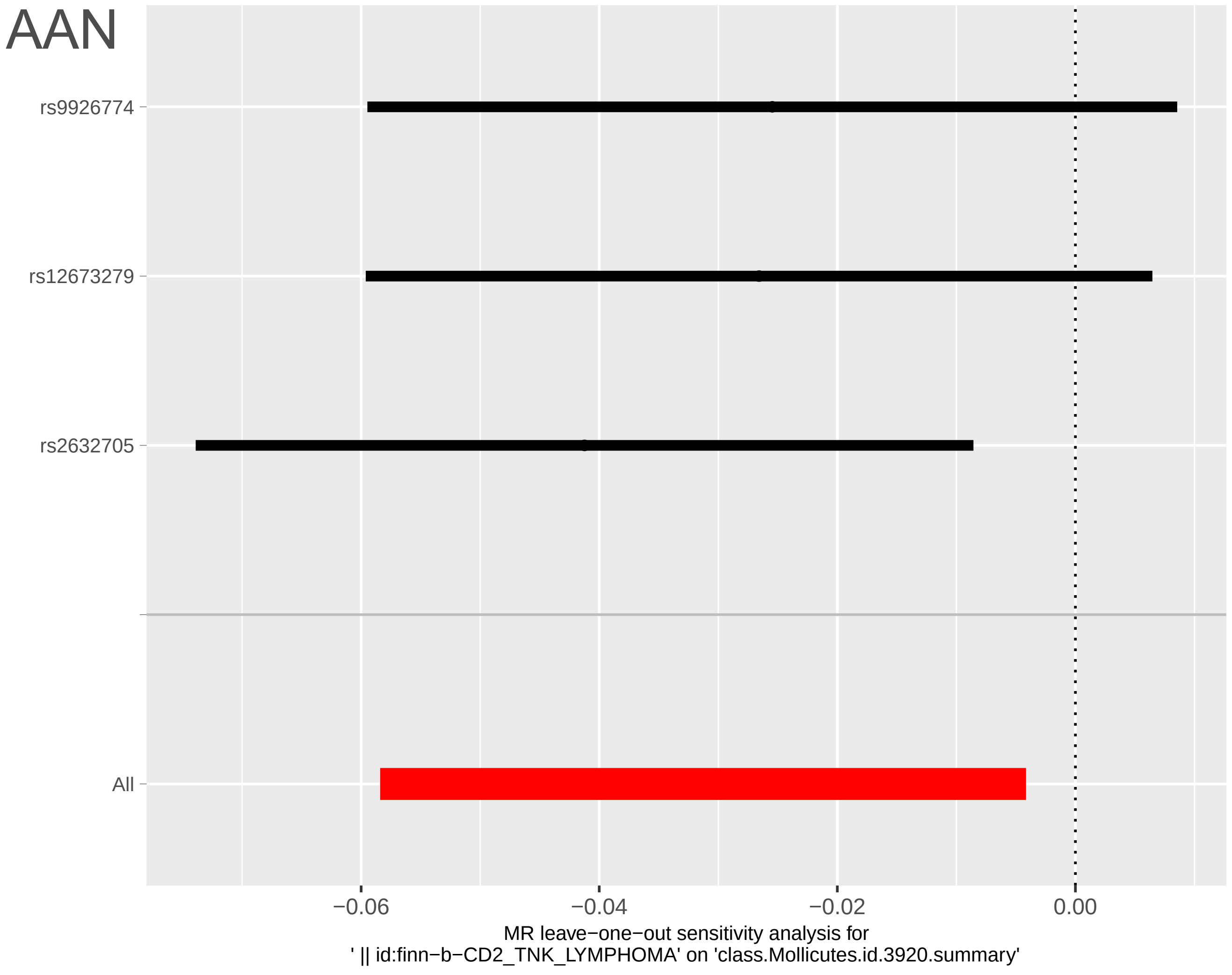

AAN
rs9926774
rs12673279
rs2632705
All
−0.06
−0.04
−0.02
0.00
MR leave−one−out sensitivity analysis for
' || id:finn−b−CD2_TNK_LYMPHOMA' on 'class.Mollicutes.id.3920.summary'

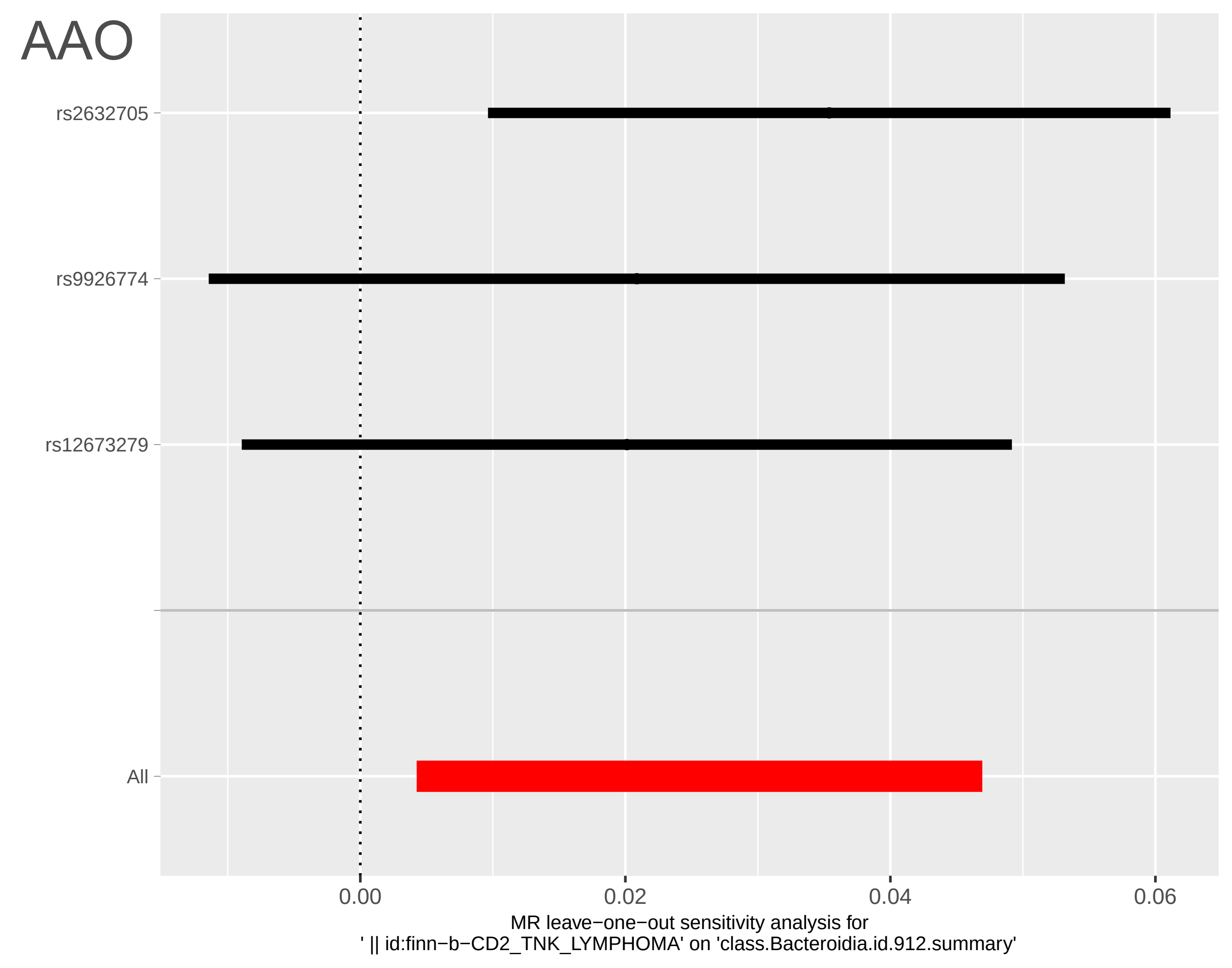
AAO
rs2632705
rs9926774
rs12673279
All
0.00
0.02
0.04
0.06
MR leave−one−out sensitivity analysis for
' || id:finn−b−CD2_TNK_LYMPHOMA' on 'class.Bacteroidia.id.912.summary'

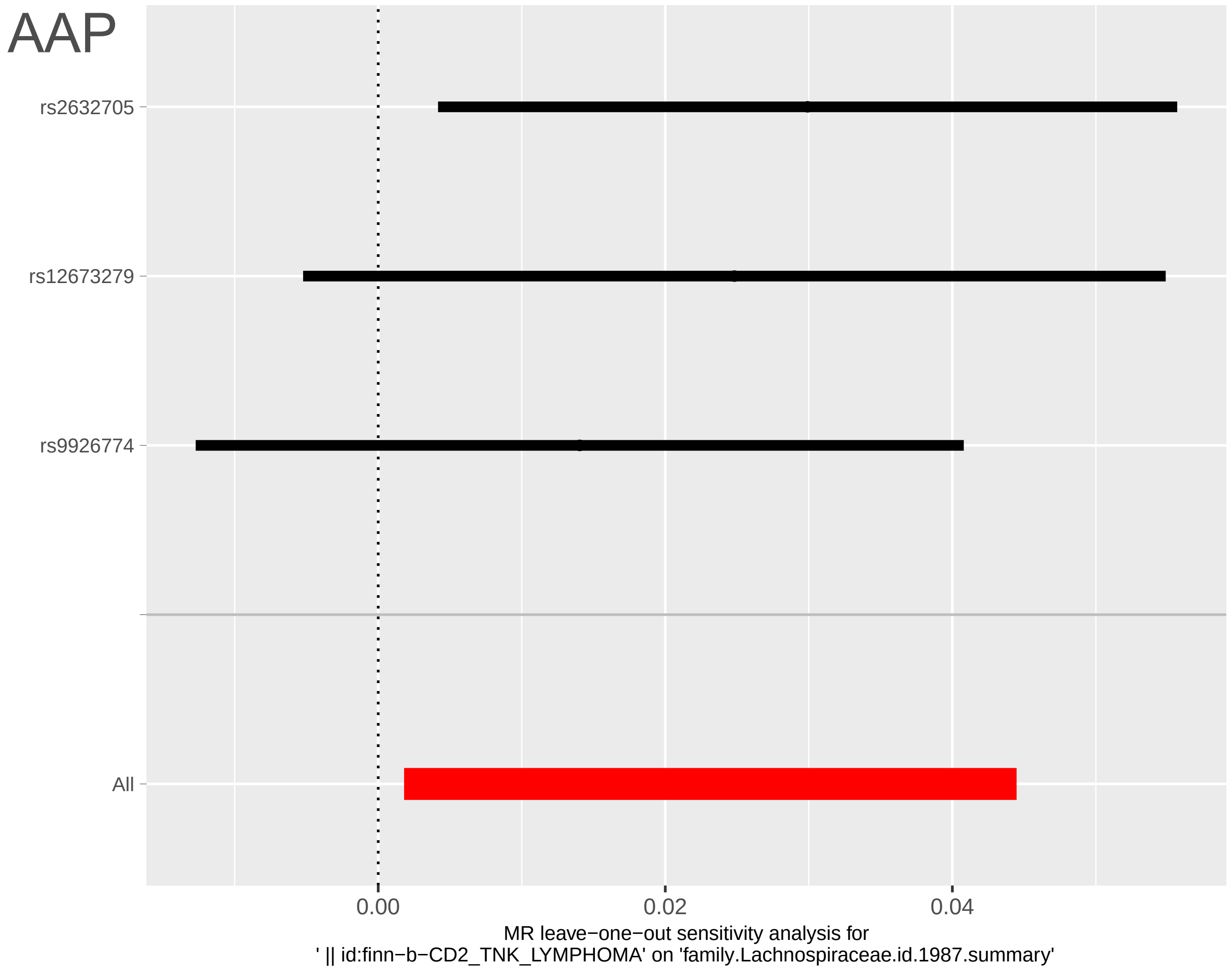
AAP
rs2632705
rs12673279
rs9926774
All
0.00
0.02
0.04
MR leave−one−out sensitivity analysis for
' || id:finn−b−CD2_TNK_LYMPHOMA' on 'family.Lachnospiraceae.id.1987.summary'

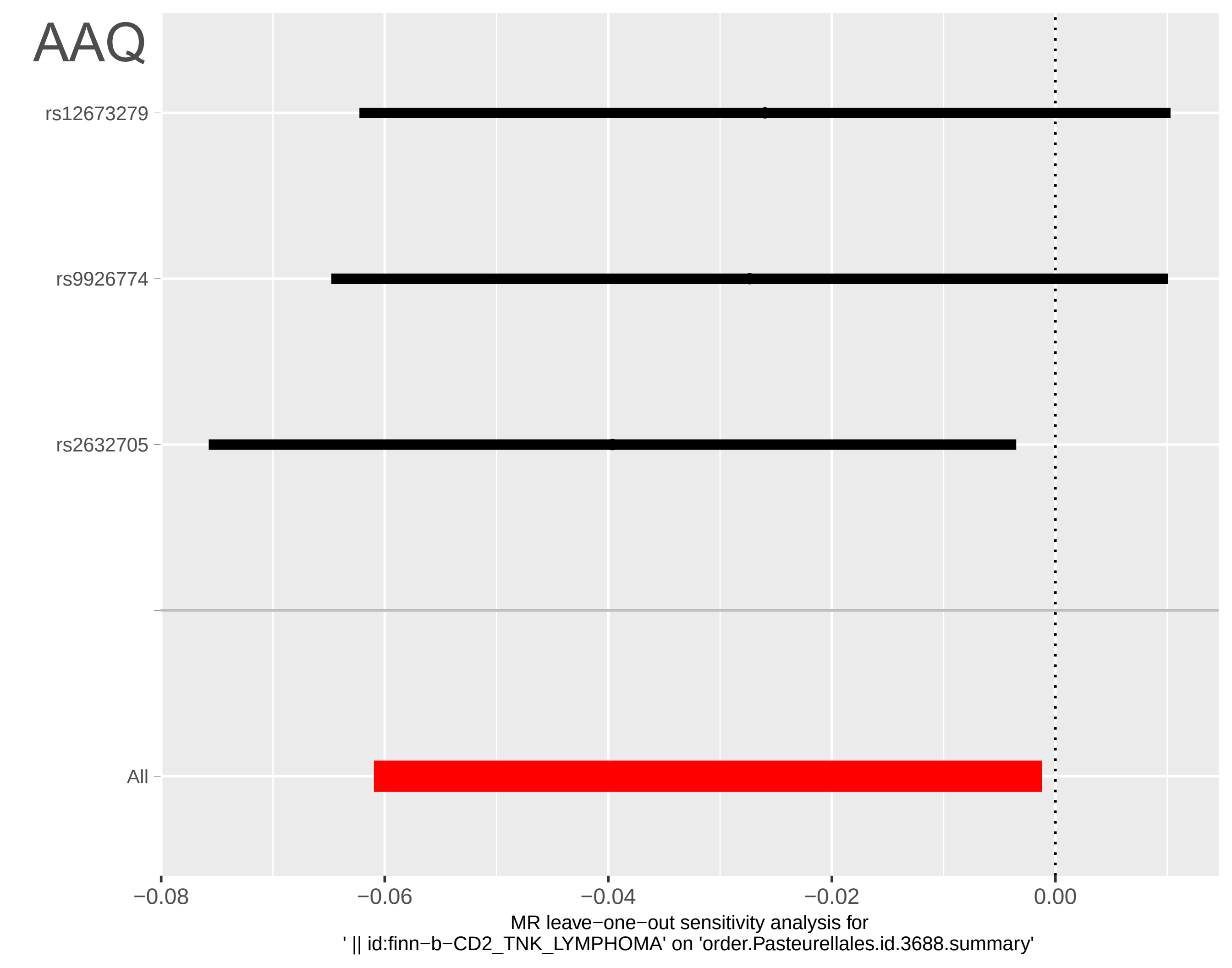
AAQ
rs12673279
rs9926774
rs2632705
All
−0.08
−0.06
−0.04
−0.02
0.00
MR leave−one−out sensitivity analysis for
' || id:finn−b−CD2_TNK_LYMPHOMA' on 'order.Pasteurellales.id.3688.summary'

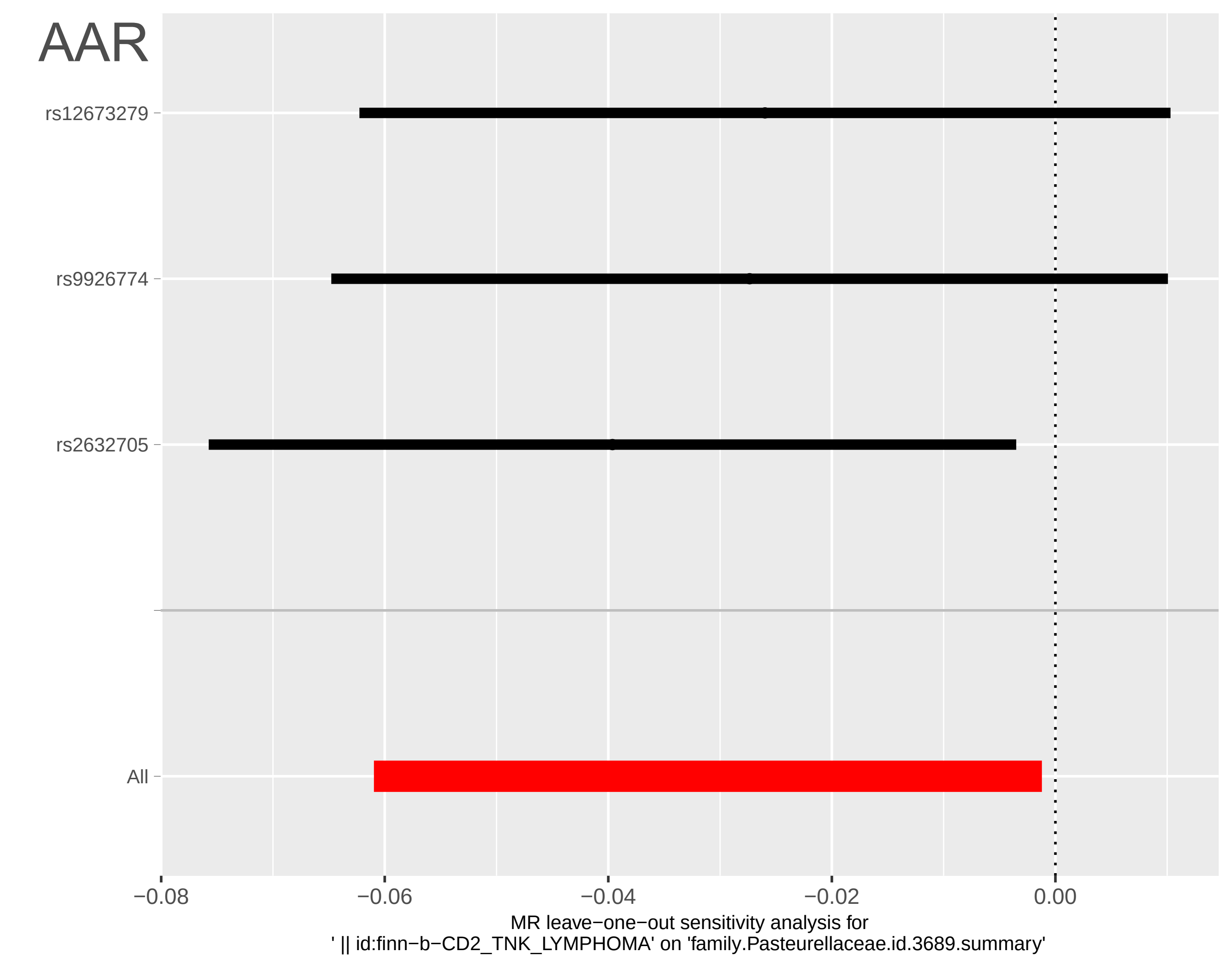
AAR
rs12673279
rs9926774
rs2632705
All
−0.08
−0.06
−0.04
−0.02
0.00
MR leave−one−out sensitivity analysis for
' || id:finn−b−CD2_TNK_LYMPHOMA' on 'family.Pasteurellaceae.id.3689.summary'

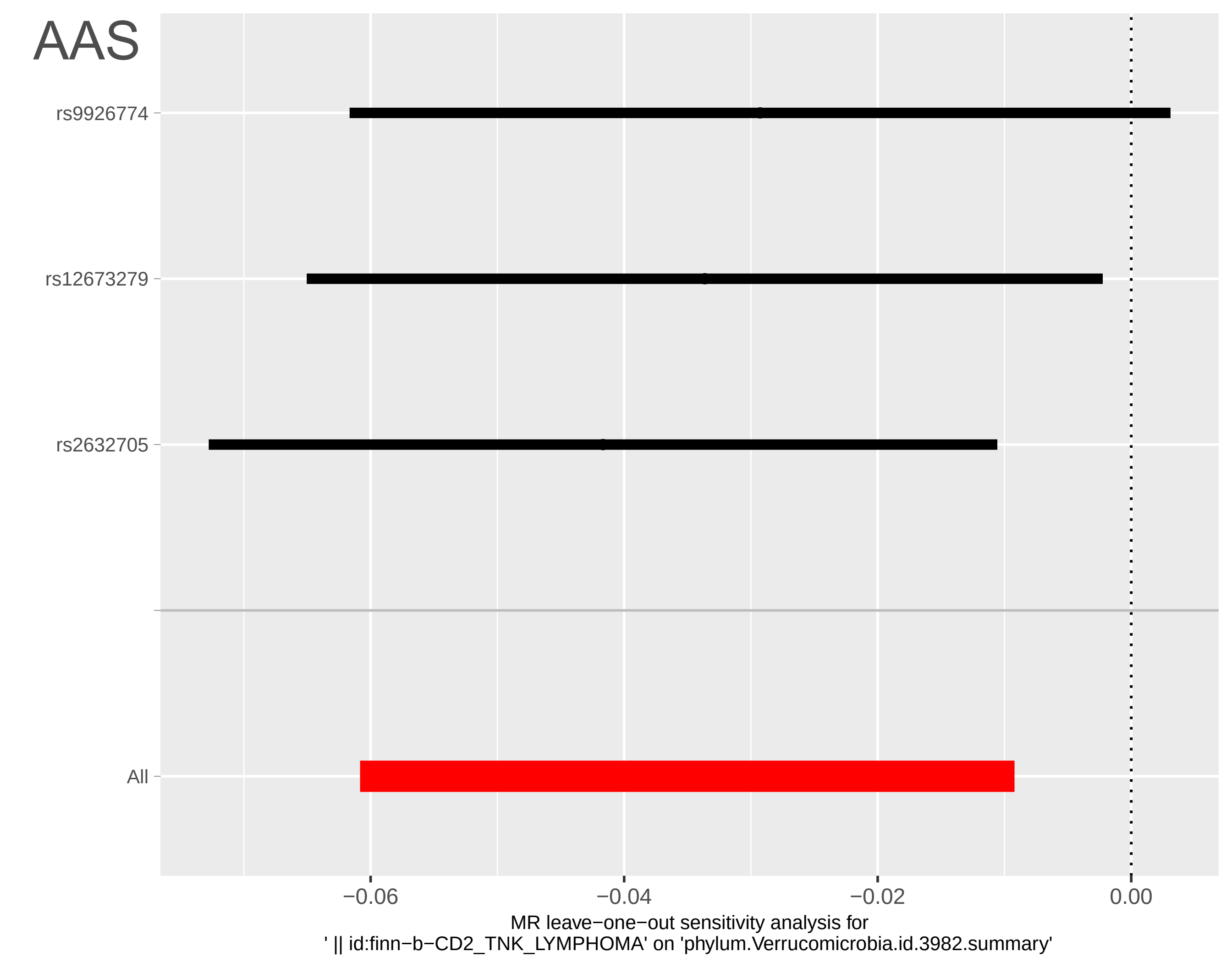
AAS
rs9926774
rs12673279
rs2632705
All
−0.06
−0.04
−0.02
0.00
MR leave−one−out sensitivity analysis for
' || id:finn−b−CD2_TNK_LYMPHOMA' on 'phylum.Verrucomicrobia.id.3982.summary'

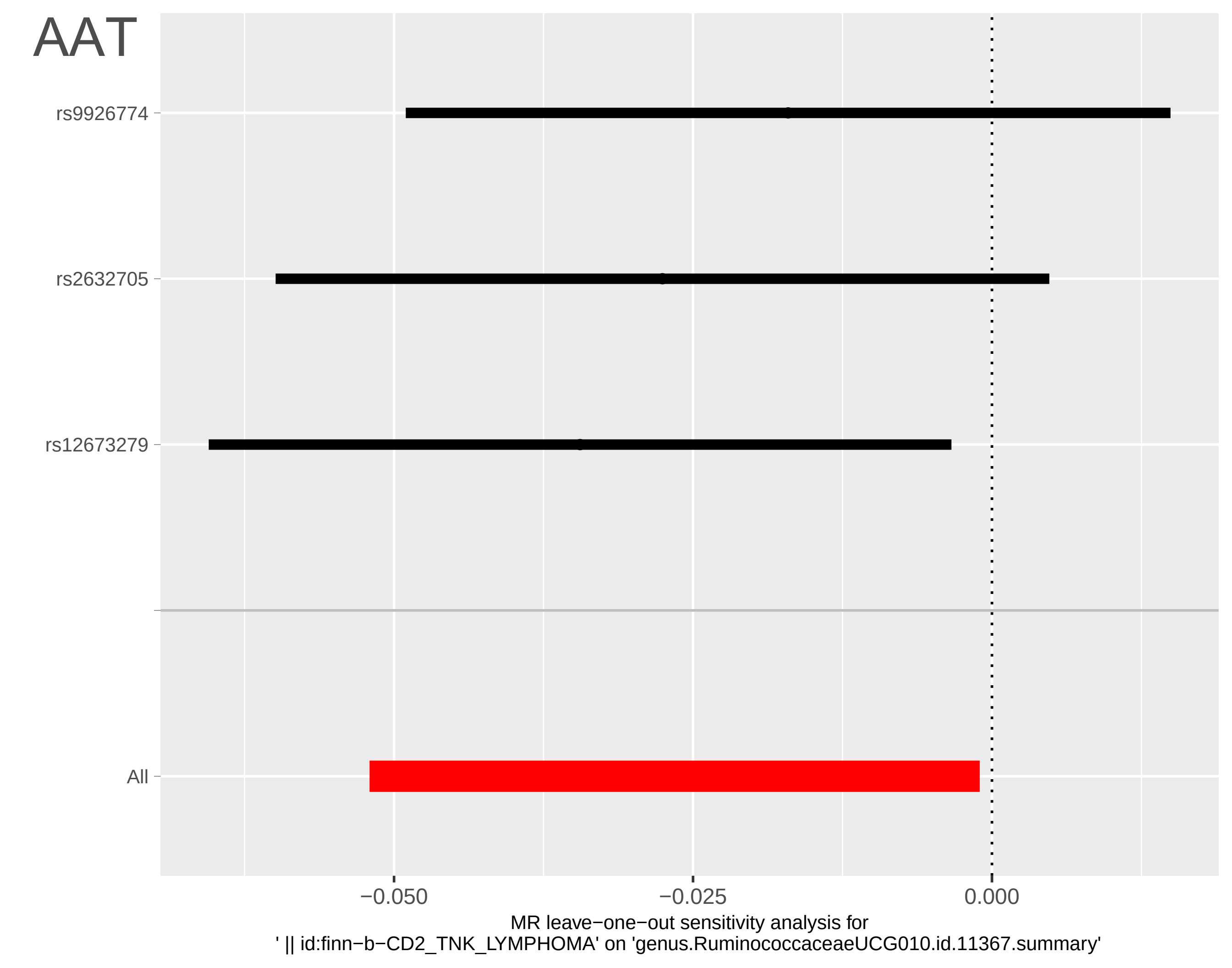
AAT
rs9926774
rs2632705
rs12673279
All
−0.050
−0.025
0.000
MR leave−one−out sensitivity analysis for
' || id:finn−b−CD2_TNK_LYMPHOMA' on 'genus.RuminococcaceaeUCG010.id.11367.summary'

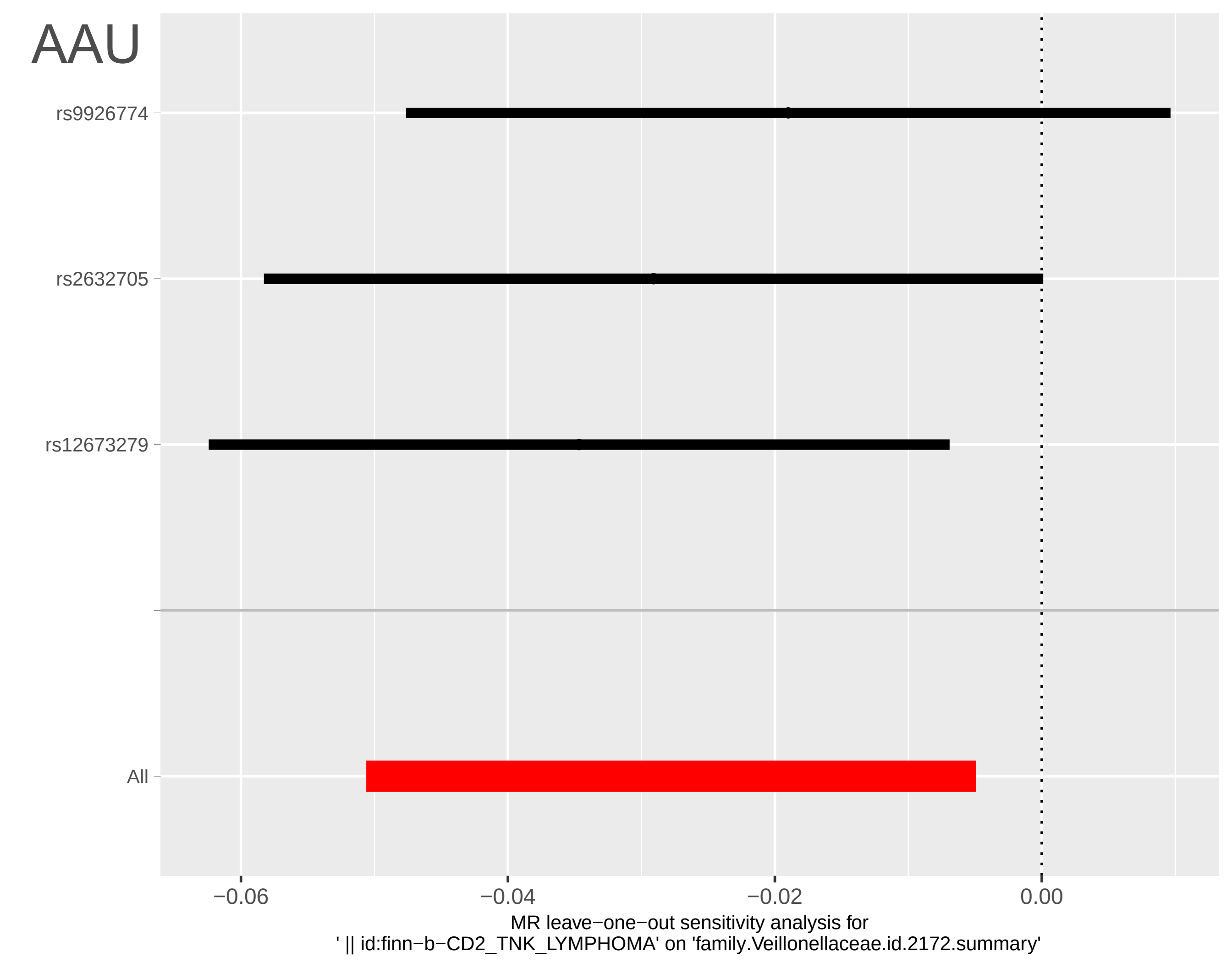
AAU
rs9926774
rs2632705
rs12673279
All
−0.06
−0.04
−0.02
0.00
MR leave−one−out sensitivity analysis for
' || id:finn−b−CD2_TNK_LYMPHOMA' on 'family.Veillonellaceae.id.2172.summary'

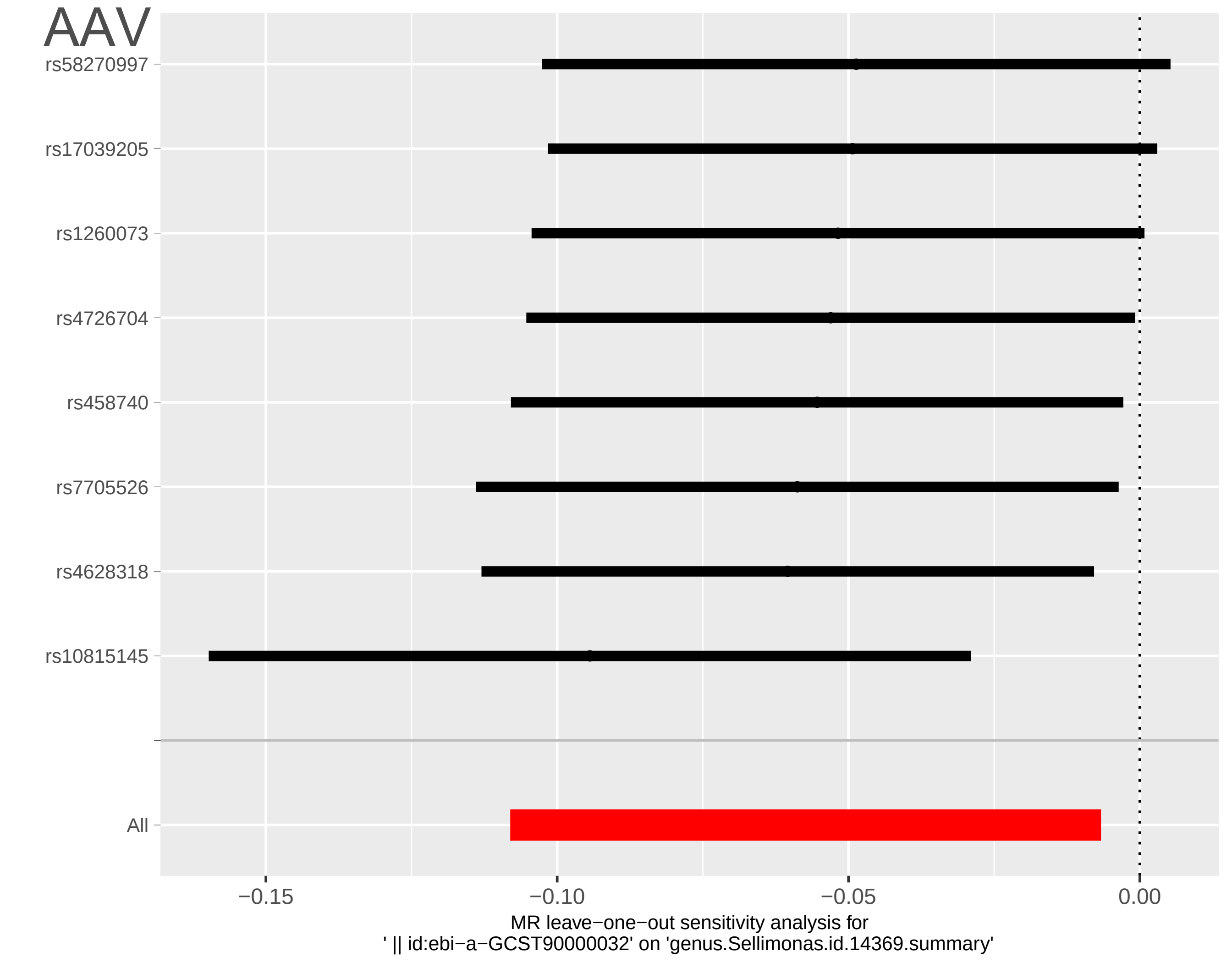
AAV
rs58270997
rs17039205
rs1260073
rs4726704
rs458740
rs7705526
rs4628318
rs10815145
All
−0.15
−0.10
−0.05
0.00
MR leave−one−out sensitivity analysis for
' || id:ebi−a−GCST90000032' on 'genus.Sellimonas.id.14369.summary'

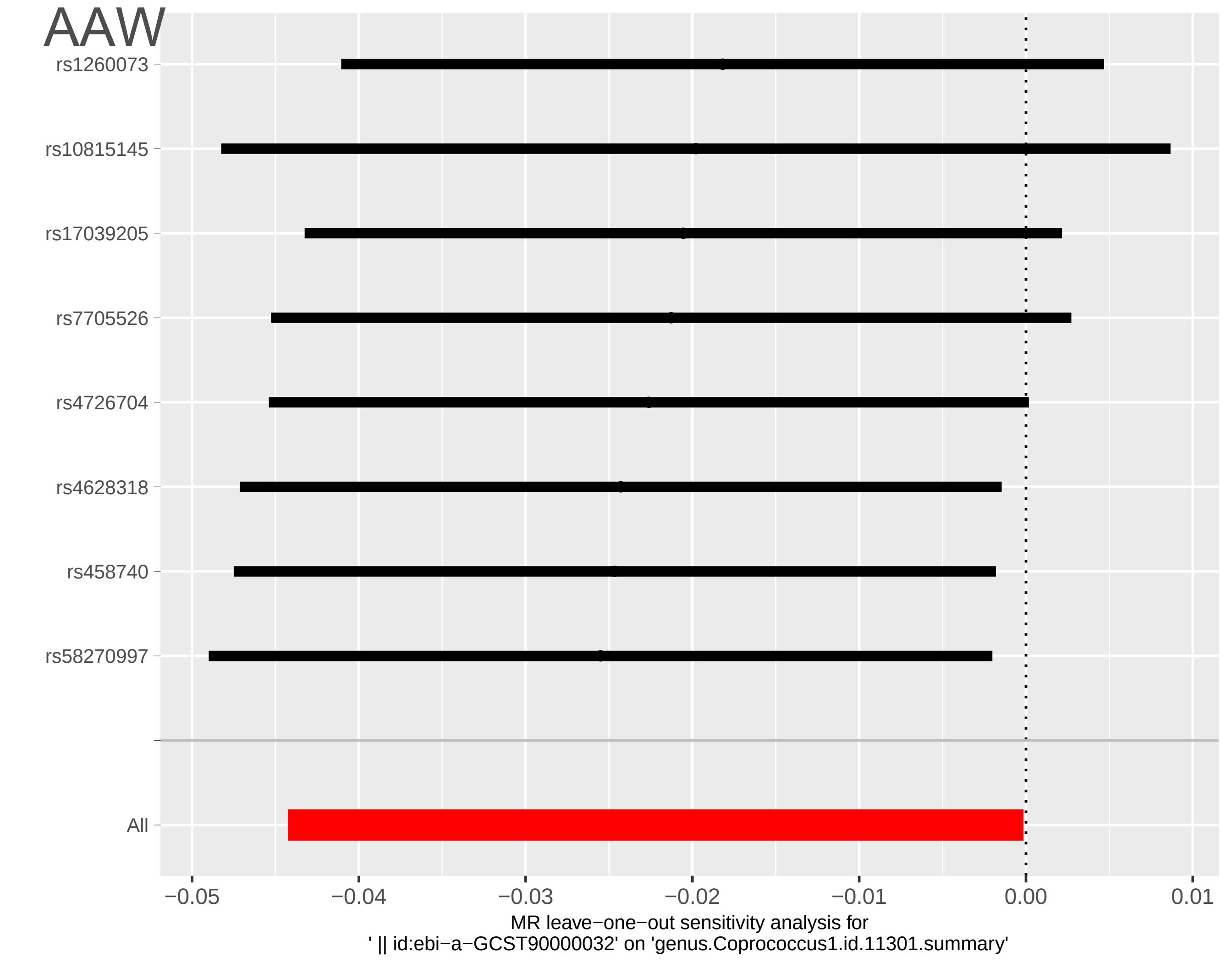
AAW
rs1260073
rs10815145
rs17039205
rs7705526
rs4726704
rs4628318
rs458740
rs58270997
All
−0.05
−0.04
−0.03
−0.02
−0.01
0.00
0.01
MR leave−one−out sensitivity analysis for
' || id:ebi−a−GCST90000032' on 'genus.Coprococcus1.id.11301.summary'

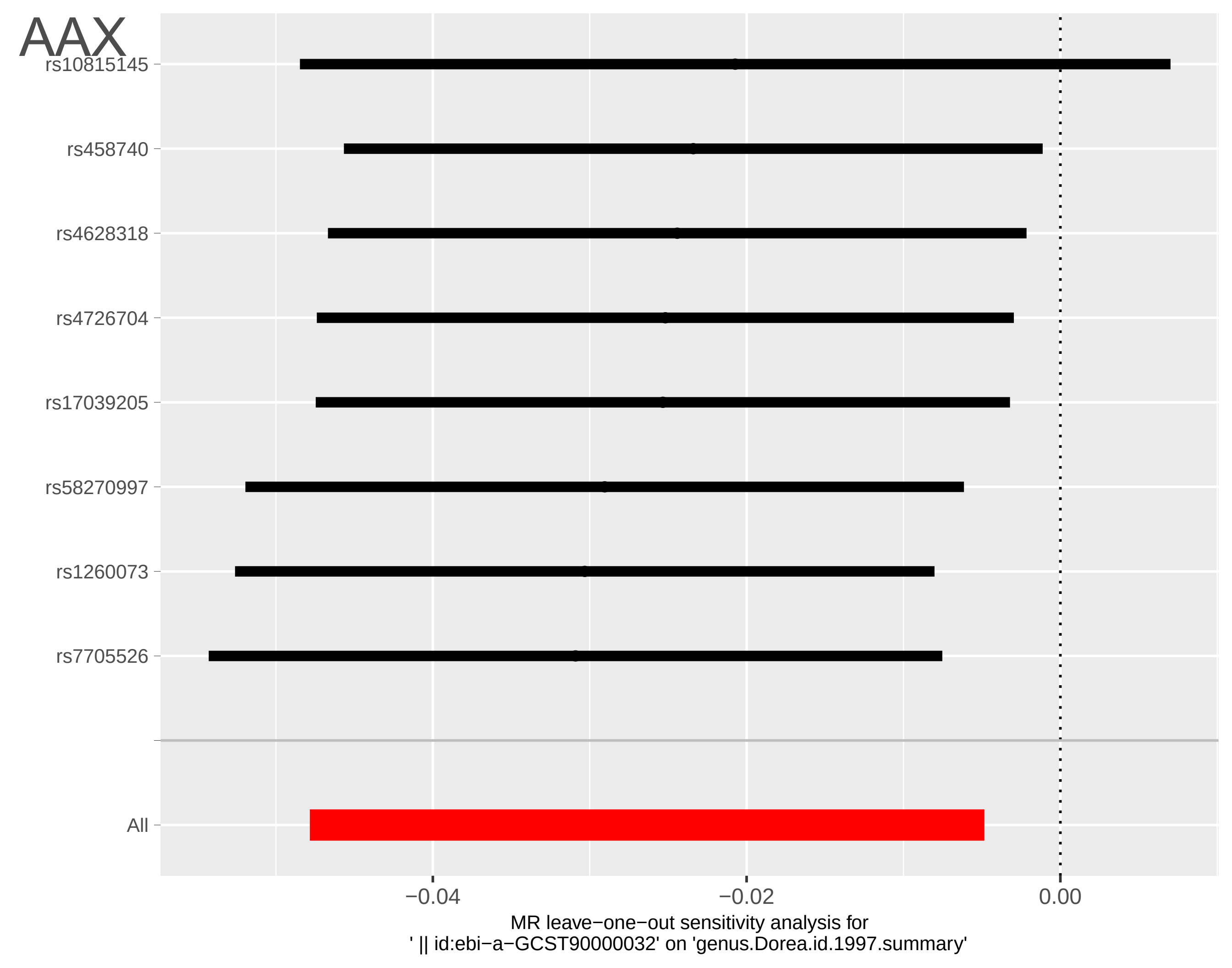
AAX
rs10815145
rs458740
rs4628318
rs4726704
rs17039205
rs58270997
rs1260073
rs7705526
All
−0.04
−0.02
0.00
MR leave−one−out sensitivity analysis for
' || id:ebi−a−GCST90000032' on 'genus.Dorea.id.1997.summary'

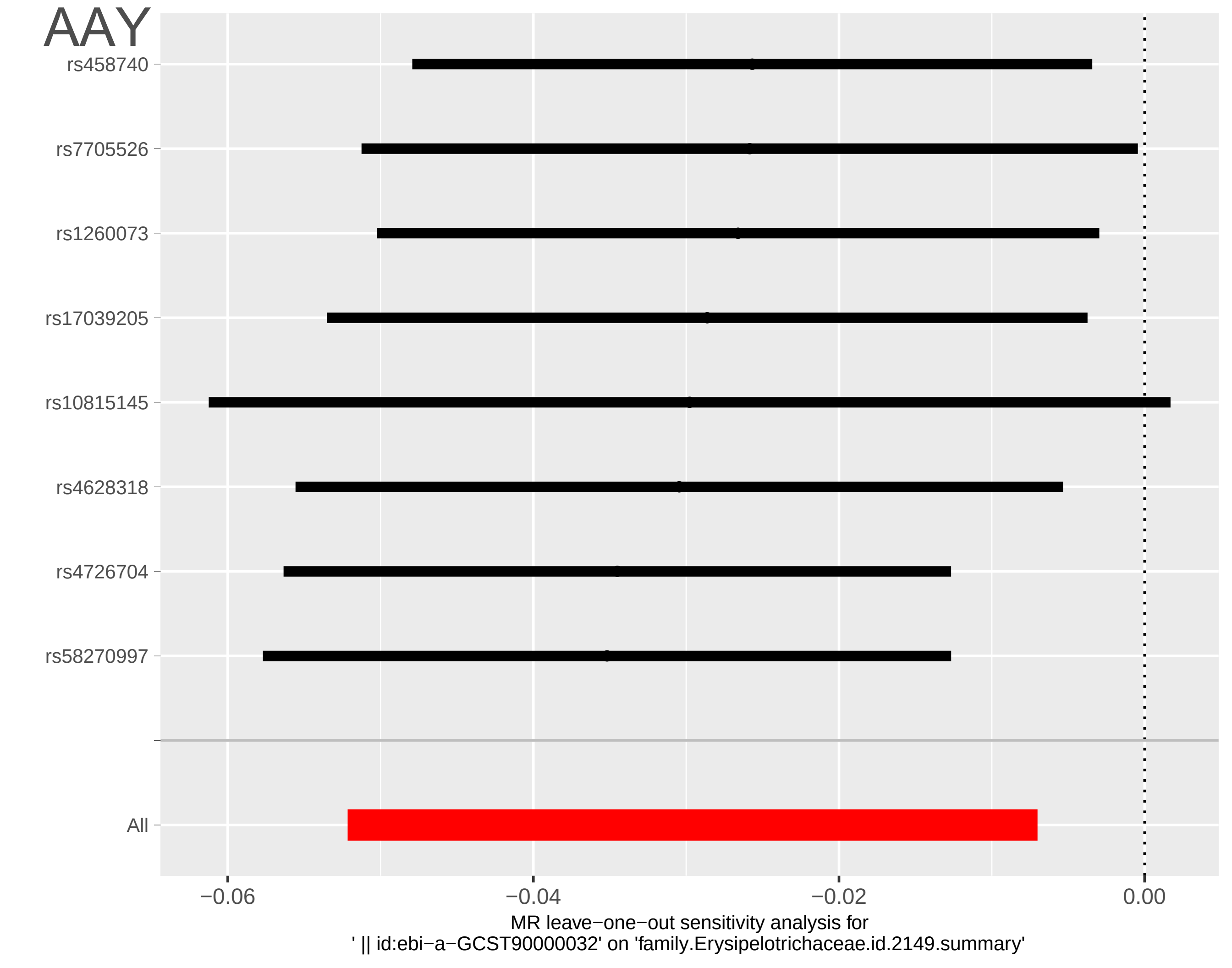
AAY
rs458740
rs7705526
rs1260073
rs17039205
rs10815145
rs4628318
rs4726704
rs58270997
All
−0.06
−0.04
−0.02
0.00
MR leave−one−out sensitivity analysis for
' || id:ebi−a−GCST90000032' on 'family.Erysipelotrichaceae.id.2149.summary'

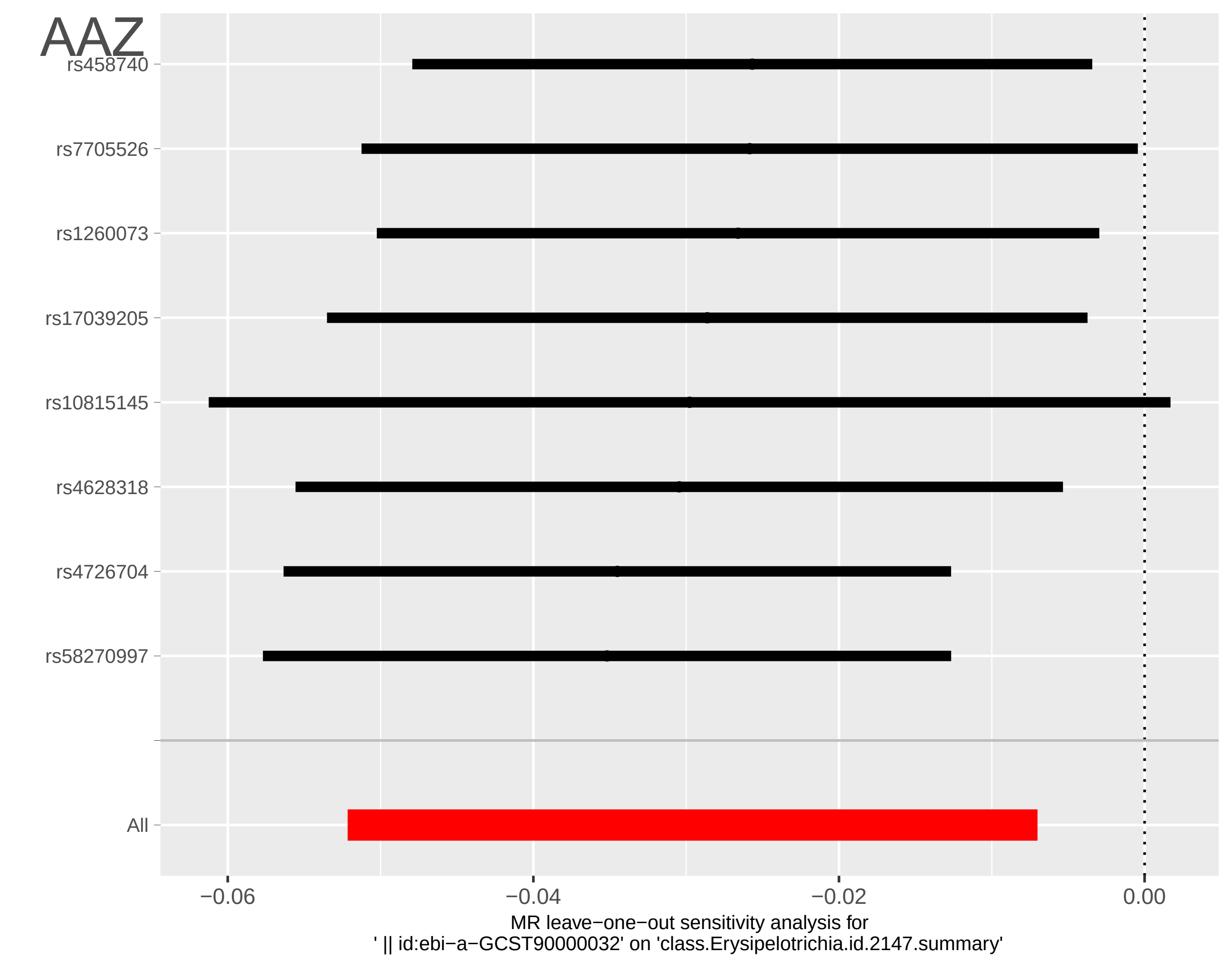

AAZ
rs458740
rs7705526
rs1260073
rs17039205
rs10815145
rs4628318
rs4726704
rs58270997
All
−0.06
−0.04
−0.02
0.00
MR leave−one−out sensitivity analysis for
' || id:ebi−a−GCST90000032' on 'class.Erysipelotrichia.id.2147.summary'

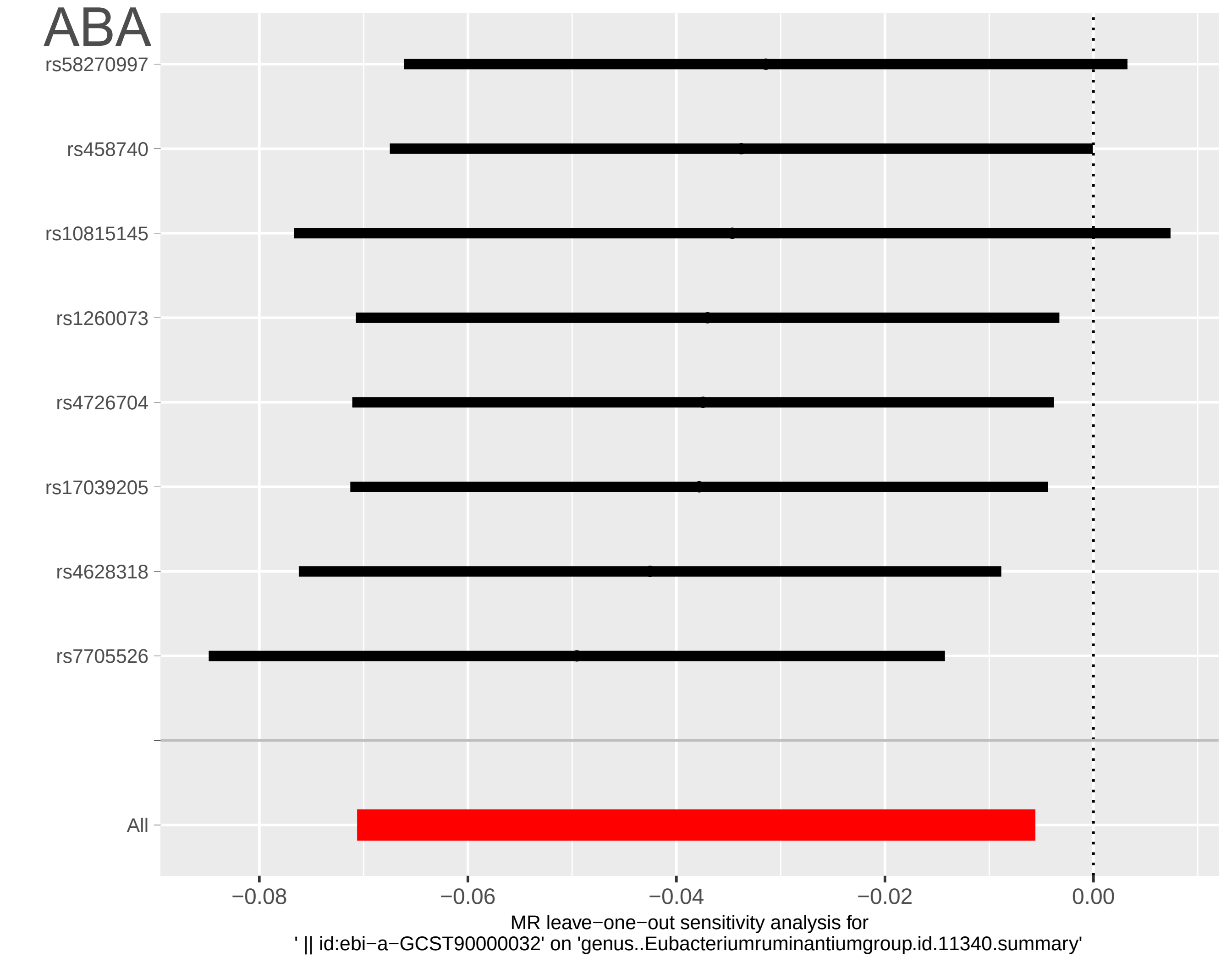
ABA
rs58270997
rs458740
rs10815145
rs1260073
rs4726704
rs17039205
rs4628318
rs7705526
All
−0.08
−0.06
−0.04
−0.02
0.00
MR leave−one−out sensitivity analysis for
' || id:ebi−a−GCST90000032' on 'genus..Eubacteriumruminantiumgroup.id.11340.summary'

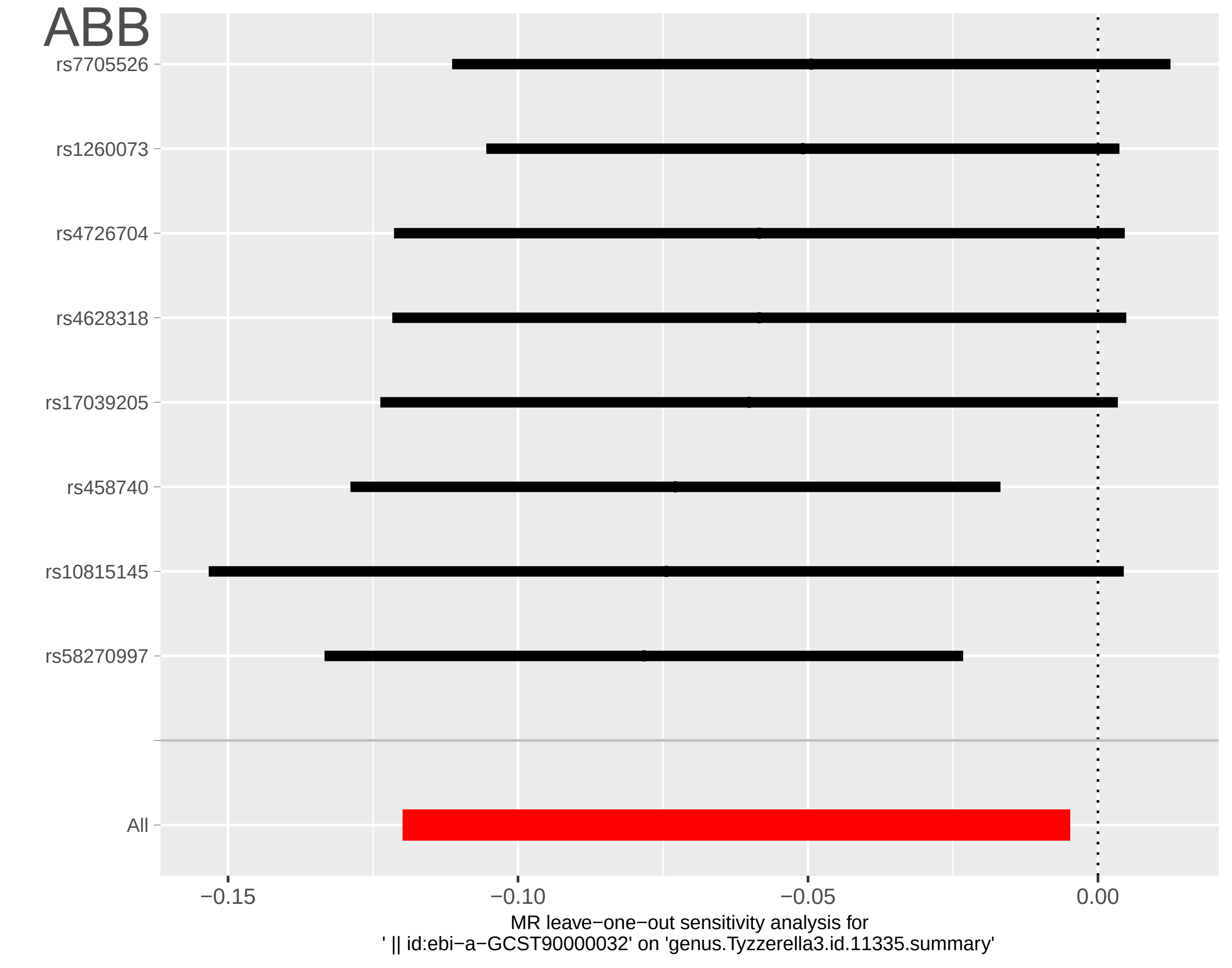

ABB
rs7705526
rs1260073
rs4726704
rs4628318
rs17039205
rs458740
rs10815145
rs58270997
All
−0.15
−0.10
−0.05
0.00
MR leave−one−out sensitivity analysis for
' || id:ebi−a−GCST90000032' on 'genus.Tyzzerella3.id.11335.summary'

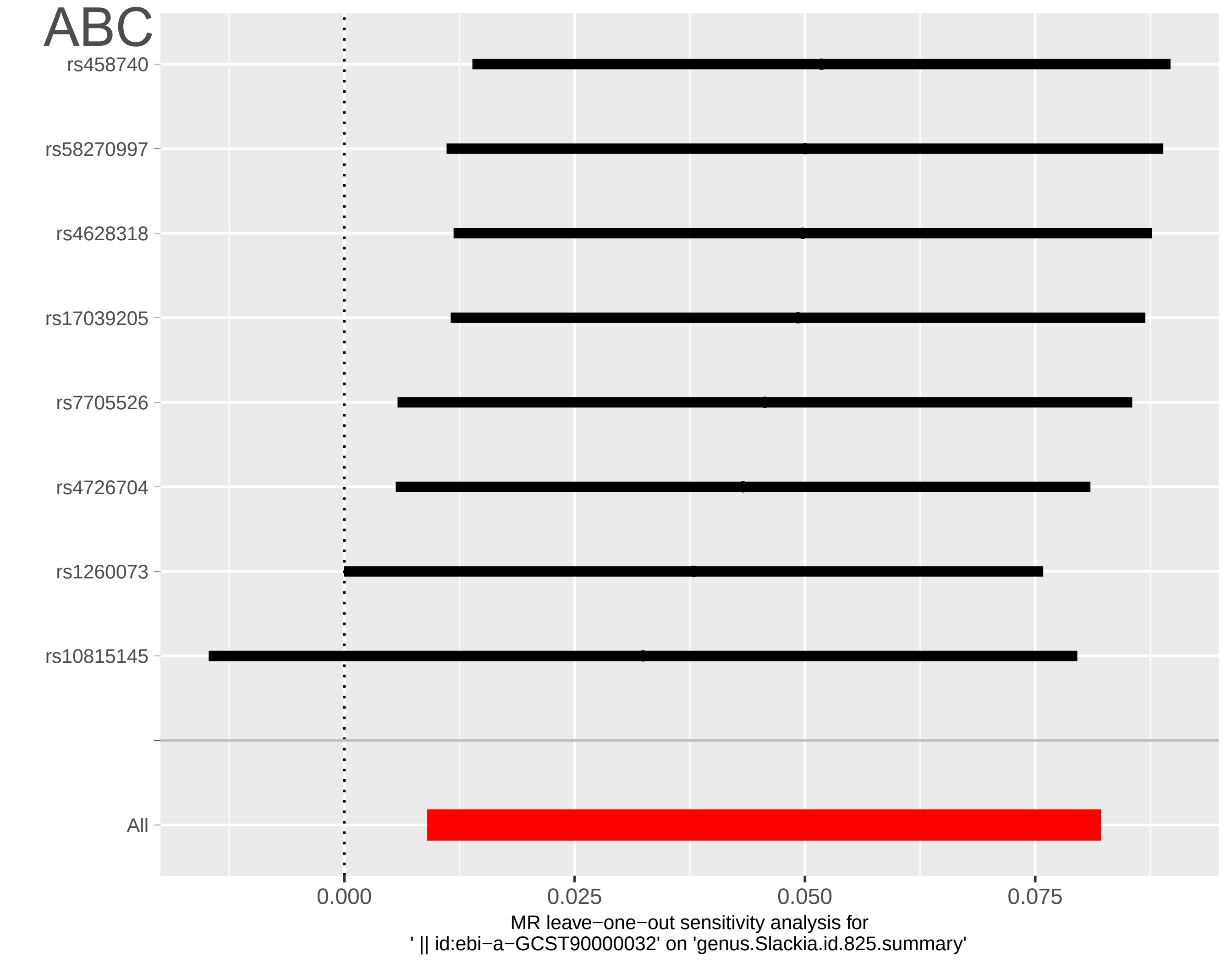
ABC
rs458740
rs58270997
rs4628318
rs17039205
rs7705526
rs4726704
rs1260073
rs10815145
All
0.000
0.025
0.050
0.075
MR leave−one−out sensitivity analysis for
' || id:ebi−a−GCST90000032' on 'genus.Slackia.id.825.summary'

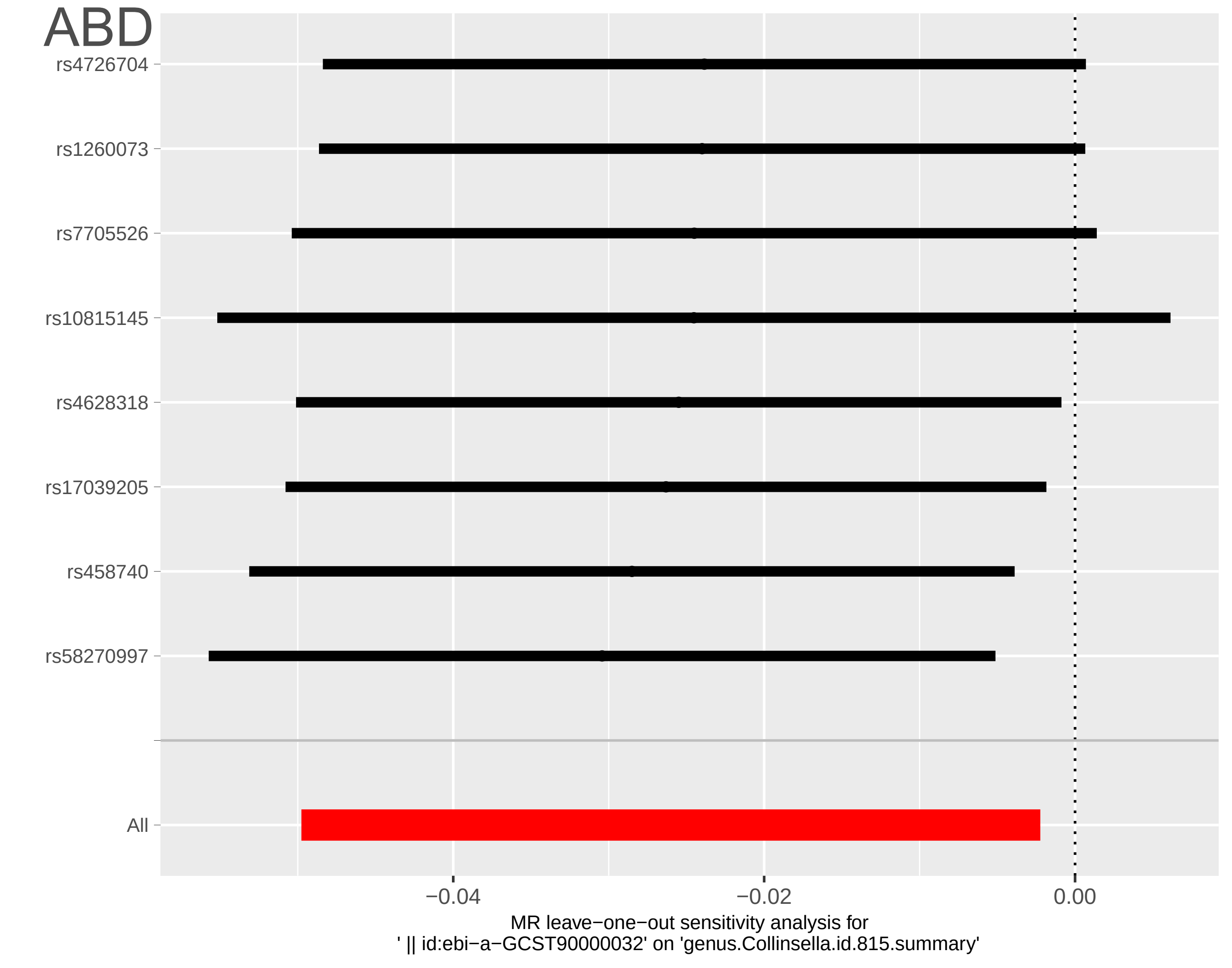
ABD
rs4726704
rs1260073
rs7705526
rs10815145
rs4628318
rs17039205
rs458740
rs58270997
All
−0.04
−0.02
0.00
MR leave−one−out sensitivity analysis for
' || id:ebi−a−GCST90000032' on 'genus.Collinsella.id.815.summary'

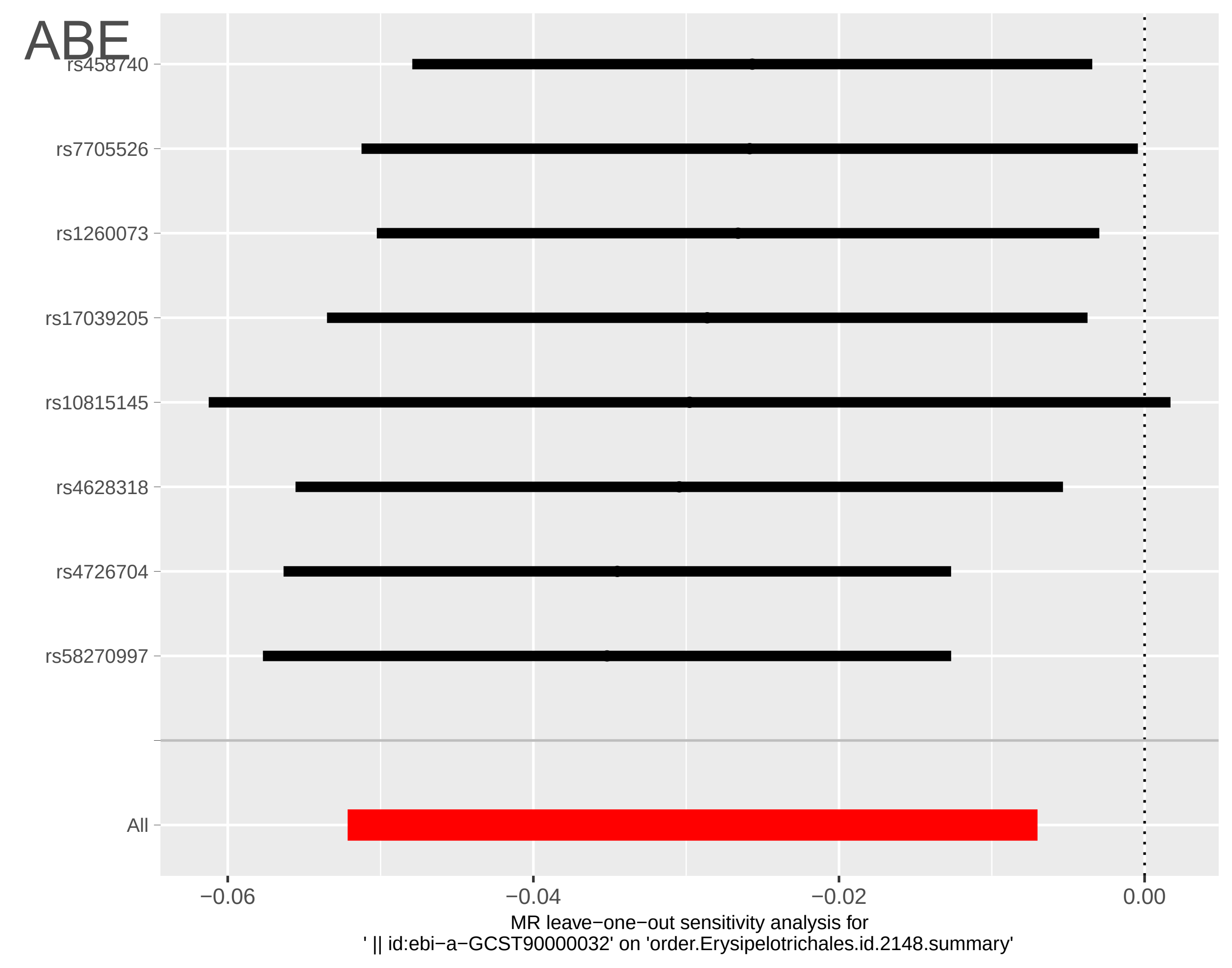
ABE
rs458740
rs7705526
rs1260073
rs17039205
rs10815145
rs4628318
rs4726704
rs58270997
All
−0.06
−0.04
−0.02
0.00
MR leave−one−out sensitivity analysis for
' || id:ebi−a−GCST90000032' on 'order.Erysipelotrichales.id.2148.summary'

Supplementary figure 7.Sensitivity analysis of the impact of hematologic malignancies on gut microbiota using MR. (leaveoutone plot)

A.Leaveoutone plot between lymphoid leukemia and order.Enterobacteriales

B.Leaveoutone plot between lymphoid leukemia and genus Eubacteriumbrachygroup

C.Leaveoutone plot between lymphoid leukemia and genus.LachnospiraceaeNC2004group

D.Leaveoutone plot between lymphoid leukemia and genus.Anaerofilum.

E.Leaveoutone plot between lymphoid leukemia and genus.Butyrivibrio

F.Leaveoutone plot between lymphoid leukemia and genus.Escherichia.Shigella

G.Leaveoutone plot between lymphoid leukemia and family.Enterobacteriaceae

H.Leaveoutone plot between lymphoid leukemia and genus.Fusicatenibacter

I.Leaveoutone plot between lymphoid leukemia and genus.LachnospiraceaeUCG010

J.Leaveoutone plot between myeloid leukemia and family.Bifidobacteriaceae

K.Leaveoutone plot between myeloid leukemia and class.Actinobacteria

L.Leaveoutone plot between myeloid leukemia and class.Clostridia

M.Leaveoutone plot between myeloid leukemia and phylum.Bacteroidetes

N.Leaveoutone plot between myeloid leukemia and genus..Ruminococcusgauvreauiigroup

O.Leaveoutone plot between myeloid leukemia and order.Clostridiales

P.Leaveoutone plot between myeloid leukemia and phylum.Actinobacteria.

Q.Leaveoutone plot between myeloid leukemia and order.Bacteroidales

R.Leaveoutone plot between myeloid leukemia and class.Bacteroidia

S.Leaveoutone plot between myeloid leukemia and genus.Bifidobacterium

T.Leaveoutone plot between myeloid leukemia and family.Lachnospiraceae

U.Leaveoutone plot between myeloid leukemia and genus.Collinsella

V.Leaveoutone plot between myeloid leukemia and genus.Blautia

W.Leaveoutone plot between myeloid leukemia and genus.Fusicatenibacter

X.Leaveoutone plot between myeloid leukemia and order.Bifidobacteriales

Y.Leaveoutone plot between myeloid leukemia and genus..Ruminococcusgnavusgroup

Z.Leaveoutone plot between myeloid leukemia and genus..Eubacteriumhalliigroup

AA..Leaveoutone plot between myeloid leukemia and genus.Slackia.

AB.Leaveoutone plot between myeloid leukemia and phylum.Firmicutes

AC.Leaveoutone plot between Hodgkin lymphoma and phylum.Verrucomicrobia

AD.Leaveoutone plot between Hodgkin lymphoma and family.ClostridialesvadinBB60group

AE.Leaveoutone plot between Hodgkin lymphoma and genus.RuminococcaceaeUCG010

AF.Leaveoutone plot between Hodgkin lymphoma and genus.Odoribacter

AG.Leaveoutone plot between Hodgkin lymphoma and genus.RuminococcaceaeUCG005

AH.Leaveoutone plot between Hodgkin lymphoma and genus.RuminococcaceaeUCG013

AI. Leaveoutone plot between Hodgkin lymphoma and genus.Ruminiclostridium9

AJ.Leaveoutone plot between Hodgkin lymphoma and genus.RuminococcaceaeNK4A214group

AK.Leaveoutone plot between Hodgkin lymphoma and genus..Eubacteriumventriosumgroup

AL.Leaveoutone plot between Hodgkin lymphoma and genus.RuminococcaceaeUCG003

AM.Leaveoutone plot between malignant plasma cell tumor and family.Defluviitaleaceae

AN. Leaveoutone plot between malignant plasma cell tumor and genus.DefluviitaleaceaeUCG011

AO.Leaveoutone plot between malignant plasma cell tumor and genus..Eubacteriumrectalegroup

AP.Leaveoutone plot between malignant plasma cell tumor and genus.CandidatusSoleaferrea

AQ. Leaveoutone plot between follicular lymphoma and genus.Allisonella

AR.Leaveoutone plot between follicular lymphoma and genus.RikenellaceaeRC9gutgroup

AS.Leaveoutone plot between follicular lymphoma and genus.Faecalibacterium

AT.Leaveoutone plot between follicular lymphoma and family.Clostridiaceae

AU.Leaveoutone plot between follicular lymphoma and genus.DefluviitaleaceaeUCG011.

AV.Leaveoutone plot between follicular lymphoma and genus.Clostridiumsensustricto

AW.Leaveoutone plot between follicular lymphoma and family.Defluviitaleaceae

AX.Leaveoutone plot between diffuse large B-cell lymphomaand family.Veillonellaceae

AY.Leaveoutone plot between diffuse large B-cell lymphomaand genus.Fusicatenibacter

AZ.Leaveoutone plot between diffuse large B-cell lymphomaand genus.Eggerthella

AAA.Leaveoutone plot between diffuse large B-cell lymphomaand genus.Erysipelatoclostridium

AAB.Leaveoutone plot between diffuse large B-cell lymphomaand genus.Blautia

AAC.Leaveoutone plot between diffuse large B-cell lymphomaand genus.RuminococcaceaeUCG009

AAD.Leaveoutone plot between mature T/NK-cell lymphomas and order.Verrucomicrobiales

AAE.Leaveoutone plot between mature T/NK-cell lymphomas and phylum.Tenericutes

AAF.Leaveoutone plot between mature T/NK-cell lymphomas and genus.Lachnoclostridium

AAG.Leaveoutone plot between mature T/NK-cell lymphomas and phylum.Bacteroidetes

AAH.Leaveoutone plot between mature T/NK-cell lymphomas and class.Verrucomicrobiae

AAI.Leaveoutone plot between mature T/NK-cell lymphomas and genus..Eubacteriumrectalegroup

AAJ.Leaveoutone plot between mature T/NK-cell lymphomas and family.Verrucomicrobiaceae

AAK.Leaveoutone plot between mature T/NK-cell lymphomas and order.Bacteroidales

AAL. Leaveoutone plot between mature T/NK-cell lymphomas and genus.Akkermansia

AAM. Leaveoutone plot between mature T/NK-cell lymphomas and genus.RuminococcaceaeUCG005

AAN.Leaveoutone plot between mature T/NK-cell lymphomas and class.Mollicutes.

AAO. Leaveoutone plot between mature T/NK-cell lymphomas and class.Bacteroidia

AAP.Leaveoutone plot between mature T/NK-cell lymphomas and family.Lachnospiraceae

AAQ.Leaveoutone plot between mature T/NK-cell lymphomas and order.Pasteurellales

AAR. Leaveoutone plot between mature T/NK-cell lymphomas and family.Pasteurellaceae.

AAS. Leaveoutone plot between mature T/NK-cell lymphomas and phylum.Verrucomicrobia

AAT.Leaveoutone plot between mature T/NK-cell lymphomas and genus.RuminococcaceaeUCG010

AAU.Leaveoutone plot between mature T/NK-cell lymphomas and family.Veillonellaceae.

AAV.Leaveoutone plot between myeloproliferative neoplasms and genus.Sellimonas

AAW.Leaveoutone plot between myeloproliferative neoplasms and genus.Coprococcus1

AAX.Leaveoutone plot between myeloproliferative neoplasms and genus.Dorea

AAY.Leaveoutone plot between myeloproliferative neoplasms and family.Erysipelotrichaceae

AAZ.Leaveoutone plot between myeloproliferative neoplasms and class.Erysipelotrichia

ABA.Leaveoutone plot between myeloproliferative neoplasms and genus..Eubacteriumruminantiumgroup

ABB.Leaveoutone plot between myeloproliferative neoplasms and genus.Tyzzerella3

ABC.Leaveoutone plot between myeloproliferative neoplasms and genus.Slackia

ABD.Leaveoutone plot between myeloproliferative neoplasms and genus.Collinsella

ABE.Leaveoutone plot between myeloproliferative neoplasms and order.Erysipelotrichales

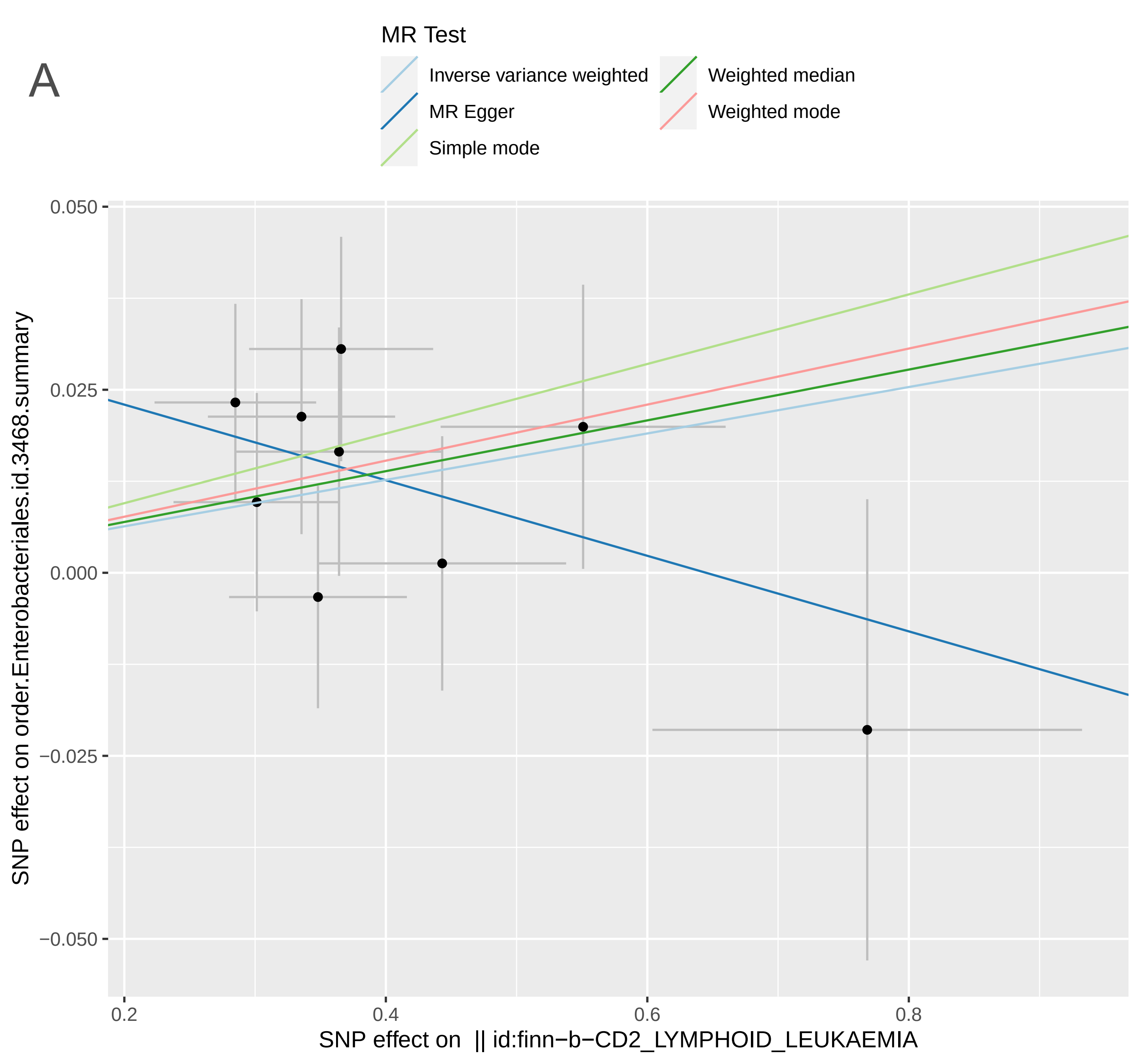

A
MR Test
Inverse variance weighted
MR Egger
Simple mode
Weighted median
Weighted mode
0.050
0.025
0.000
−0.025
−0.050
0.2
0.4
0.6
0.8
SNP effect on order.Enterobacteriales.id.3468.summary
SNP effect on || id:finn−b−CD2_LYMPHOID_LEUKAEMIA

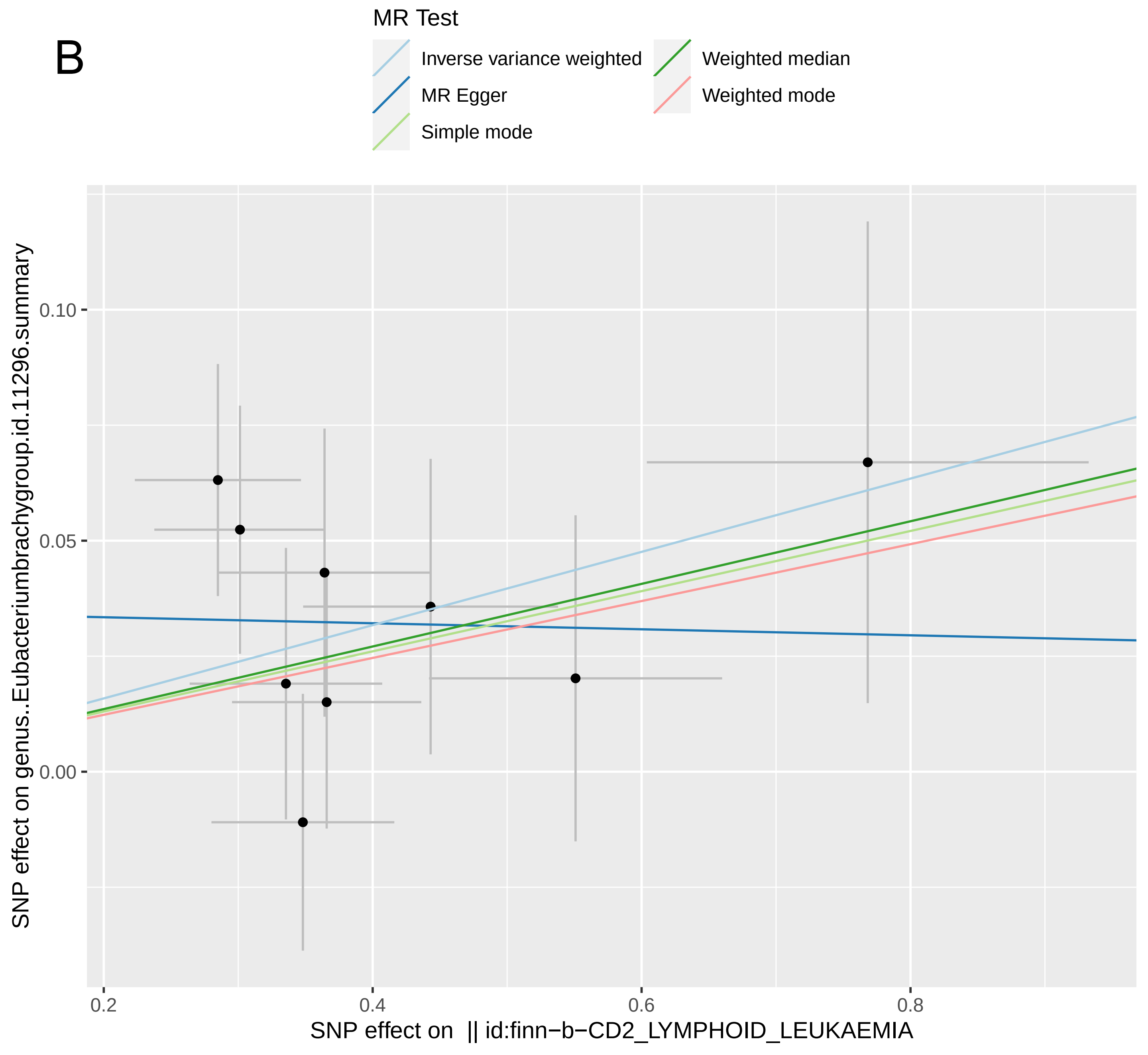

B
MR Test
Inverse variance weighted
MR Egger
Simple mode
Weighted median
Weighted mode
0.10
0.05
0.00
0.2
0.4
0.6
0.8
SNP effect on genus..Eubacteriumbrachygroup.id.11296.summary
SNP effect on || id:finn−b−CD2_LYMPHOID_LEUKAEMIA

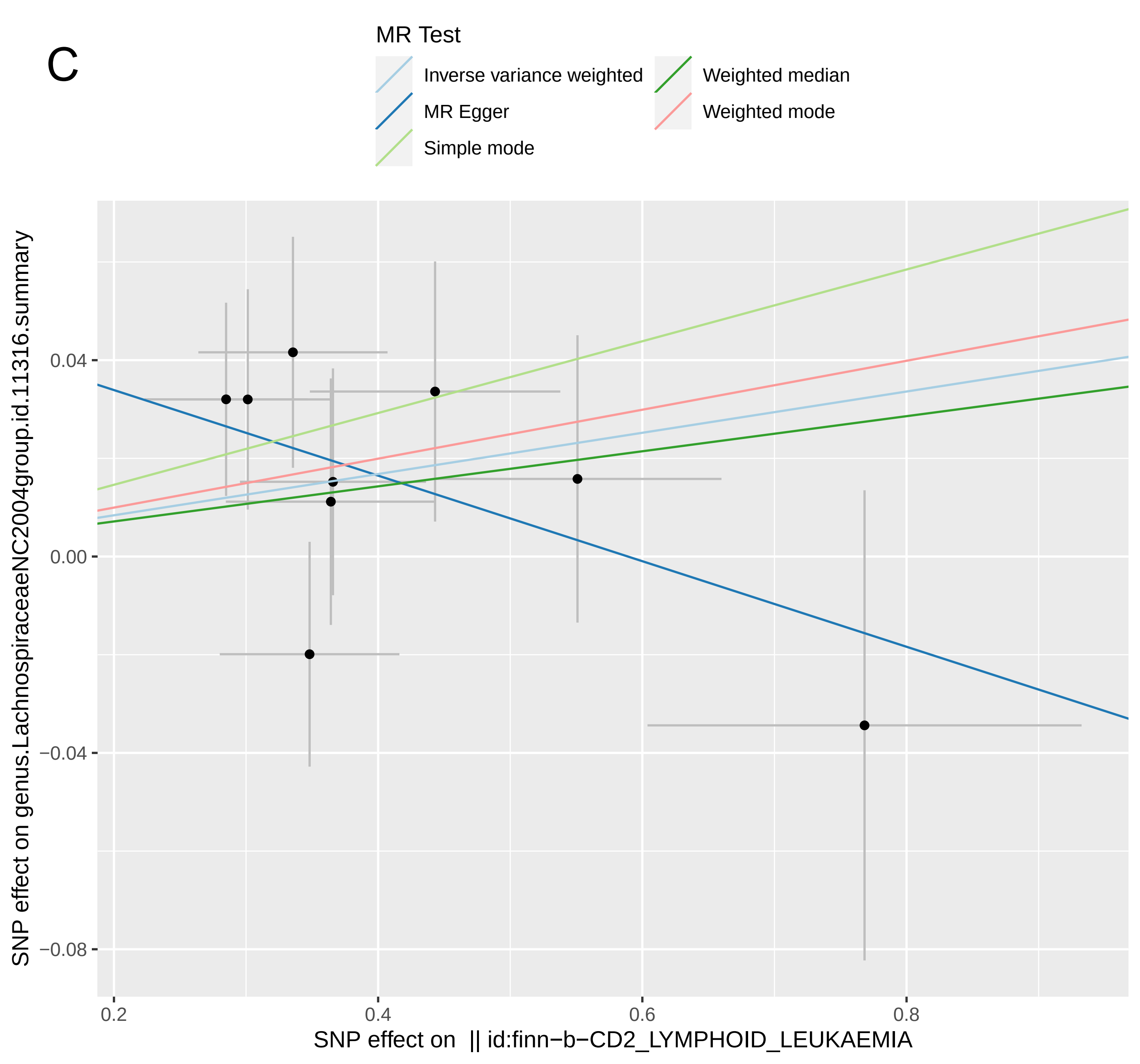

C
MR Test
Inverse variance weighted
MR Egger
Simple mode
Weighted median
Weighted mode
SNP effect on genus.LachnospiraceaeNC2004group.id.11316.summary
0.04
0.00
−0.04
−0.08
0.2
0.4
0.6
0.8
SNP effect on || id:finn−b−CD2_LYMPHOID_LEUKAEMIA

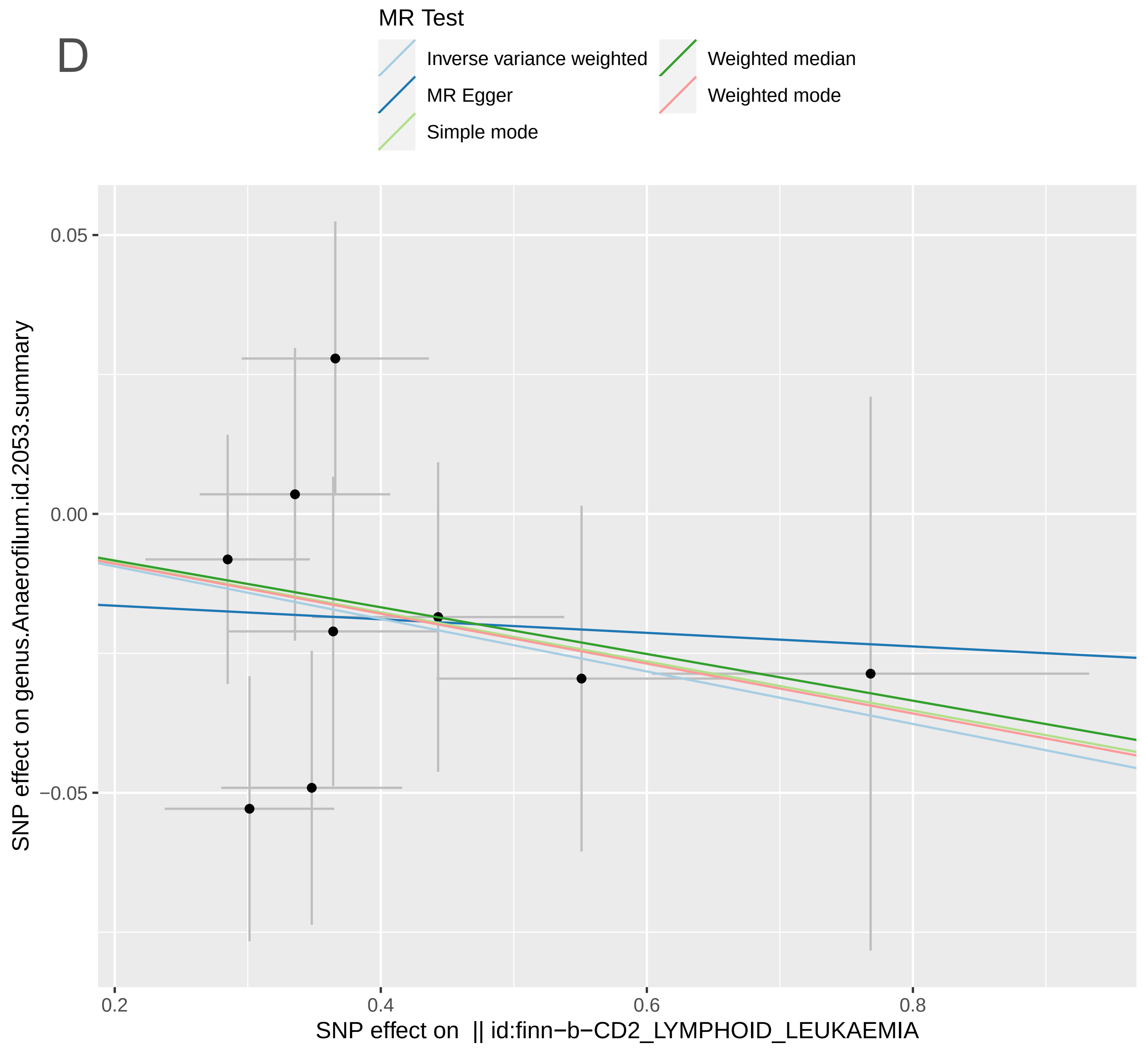
D
MR Test
Inverse variance weighted
MR Egger
Simple mode
Weighted median
Weighted mode
0.05
0.00
−0.05
SNP effect on genus.Anaerofilum.id.2053.summary
0.2
0.4
0.6
0.8
SNP effect on || id:finn−b−CD2_LYMPHOID_LEUKAEMIA

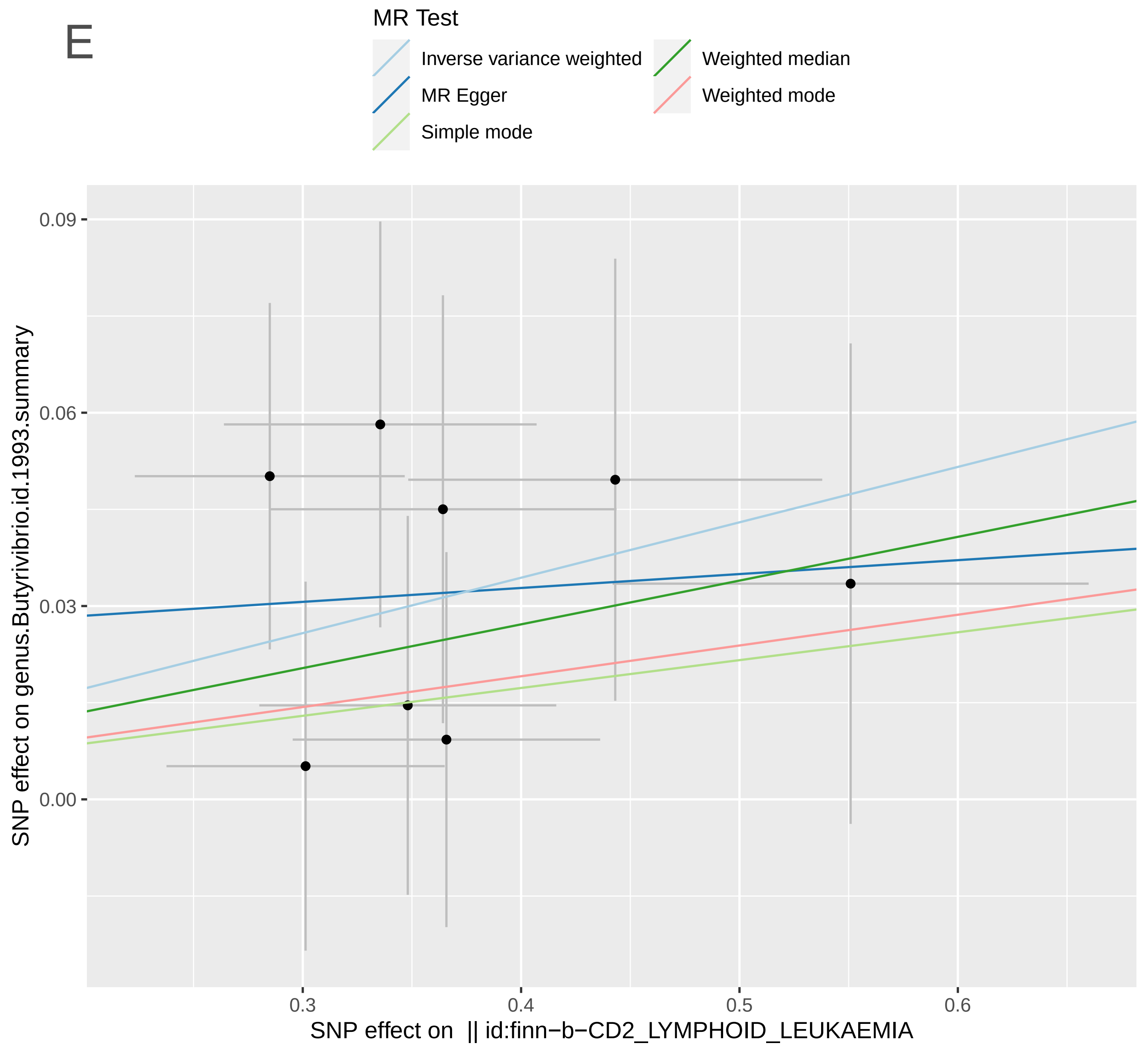
E
MR Test
Inverse variance weighted
MR Egger
Simple mode
Weighted median
Weighted mode
0.09
0.06
0.03
0.00
0.3
0.4
0.5
0.6
SNP effect on genus.Butyrivibrio.id.1993.summary
SNP effect on || id:finn−b−CD2_LYMPHOID_LEUKAEMIA

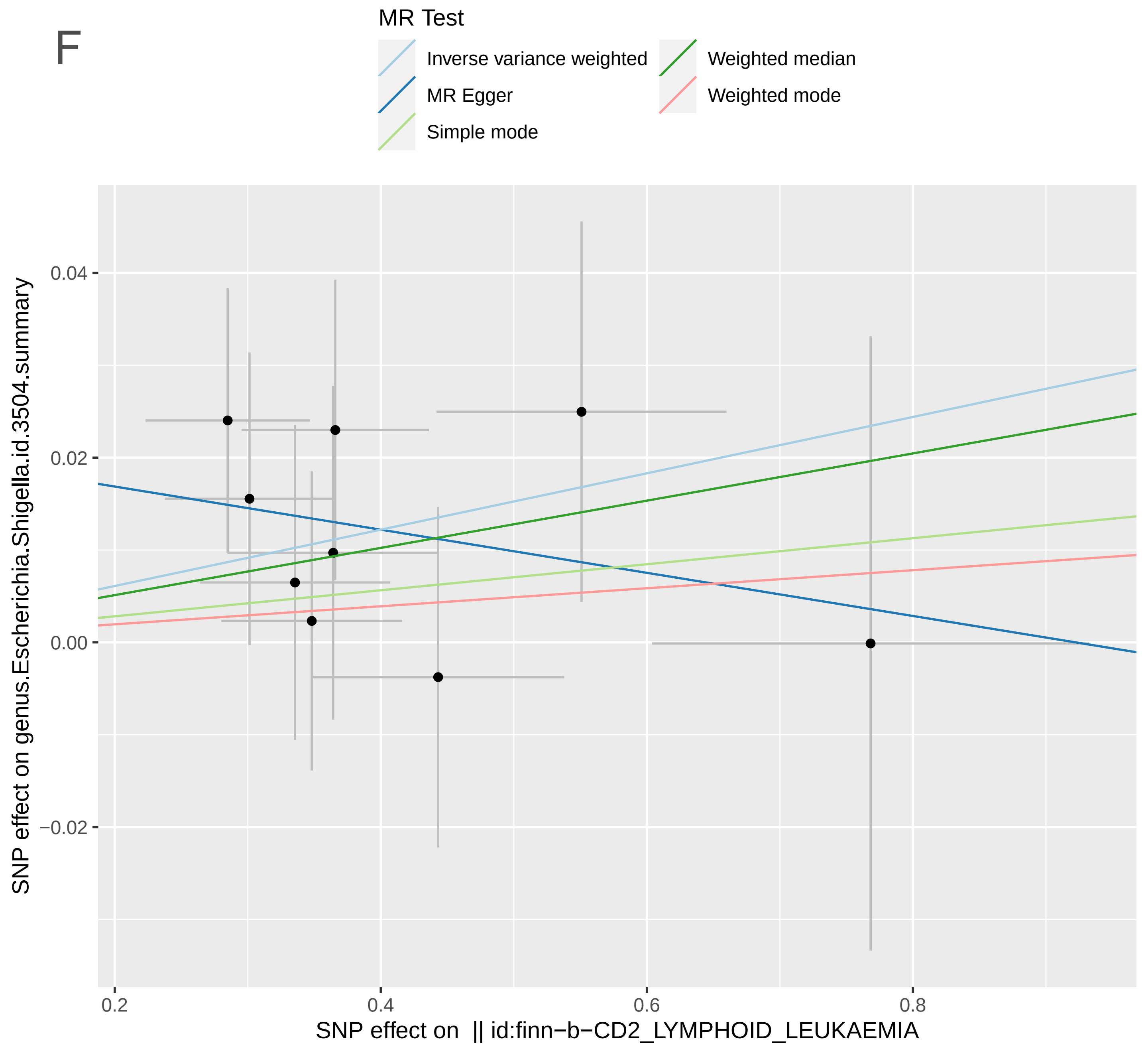
F
MR Test
Inverse variance weighted
MR Egger
Simple mode
Weighted median
Weighted mode
0.04
0.02
0.00
−0.02
0.2
0.4
0.6
0.8
SNP effect on genus.Escherichia.Shigella.id.3504.summary
SNP effect on || id:finn−b−CD2_LYMPHOID_LEUKAEMIA

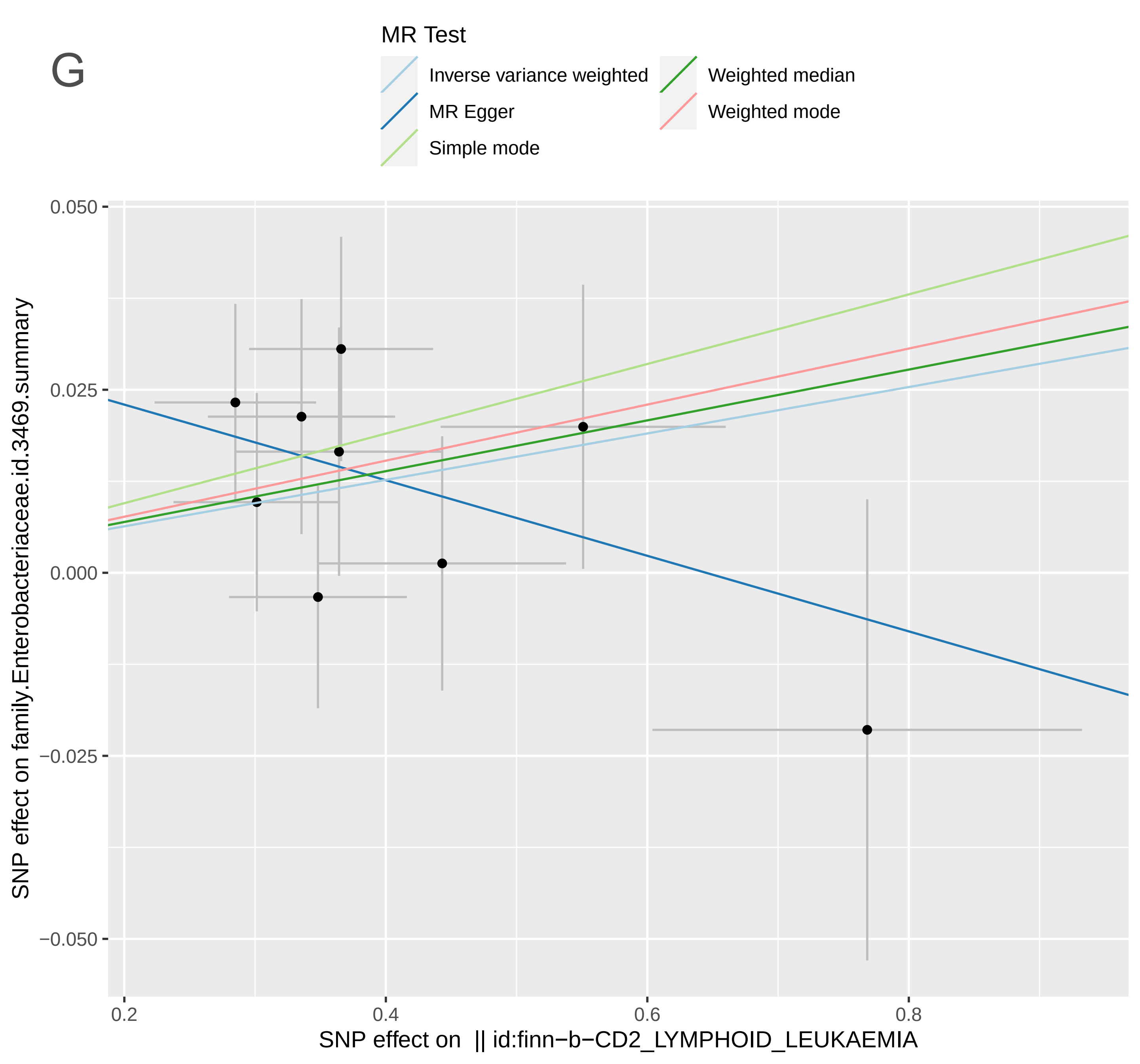

G
MR Test
Inverse variance weighted
MR Egger
Simple mode
Weighted median
Weighted mode
0.050
0.025
0.000
−0.025
−0.050
SNP effect on family.Enterobacteriaceae.id.3469.summary
0.2
0.4
0.6
0.8
SNP effect on || id:finn−b−CD2_LYMPHOID_LEUKAEMIA

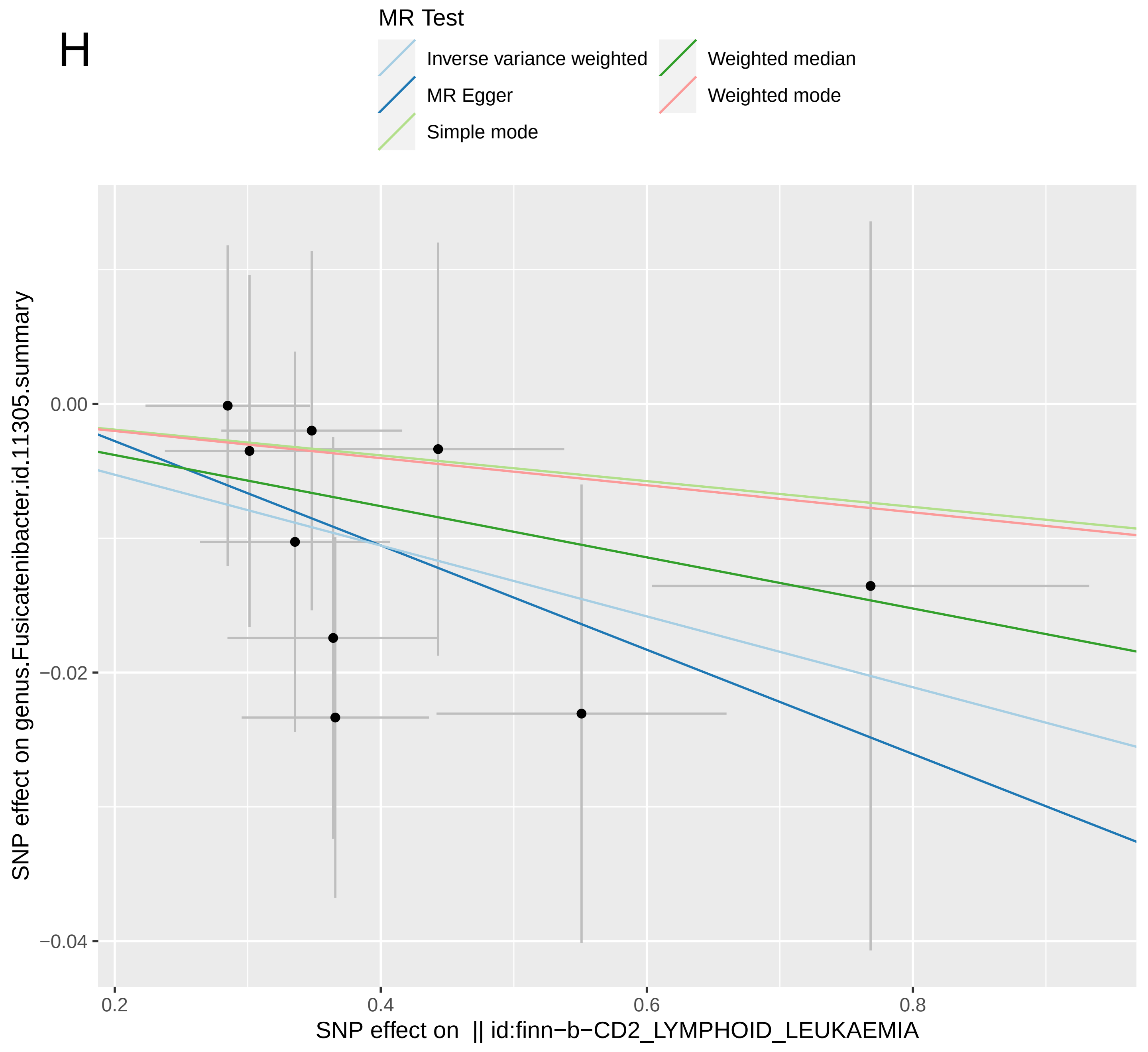

H
MR Test
Inverse variance weighted
MR Egger
Simple mode
Weighted median
Weighted mode
SNP effect on genus.Fusicatenibacter.id.11305.summary
0.00
−0.02
−0.04
0.2
0.4
0.6
0.8
SNP effect on || id:finn−b−CD2_LYMPHOID_LEUKAEMIA

I

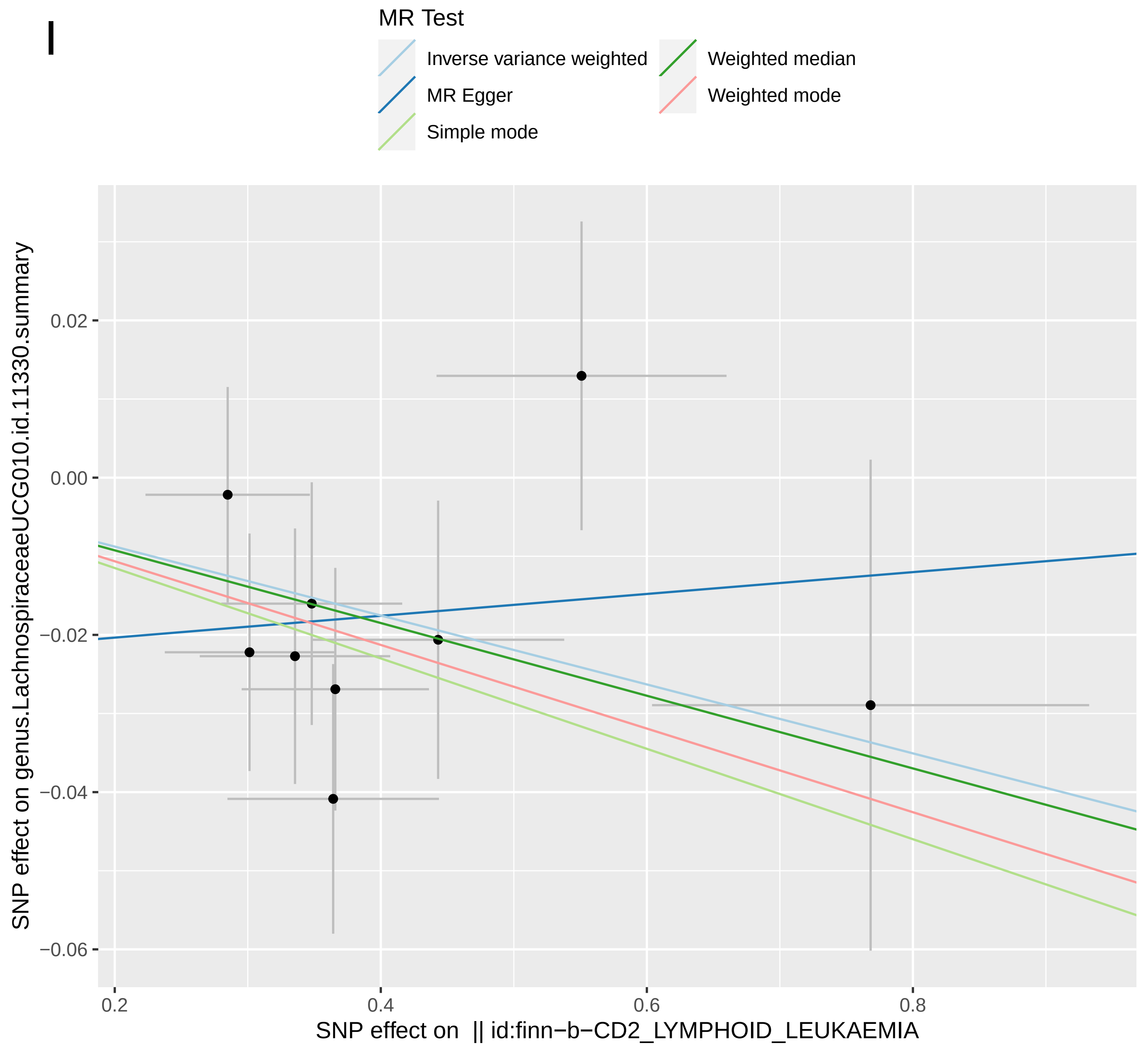
MR Test
Inverse variance weighted
MR Egger
Simple mode
Weighted median
Weighted mode
0.02
0.00
−0.02
−0.04
−0.06
0.2
0.4
0.6
0.8
SNP effect on genus.LachnospiraceaeUCG010.id.11330.summary
SNP effect on || id:finn−b−CD2_LYMPHOID_LEUKAEMIA

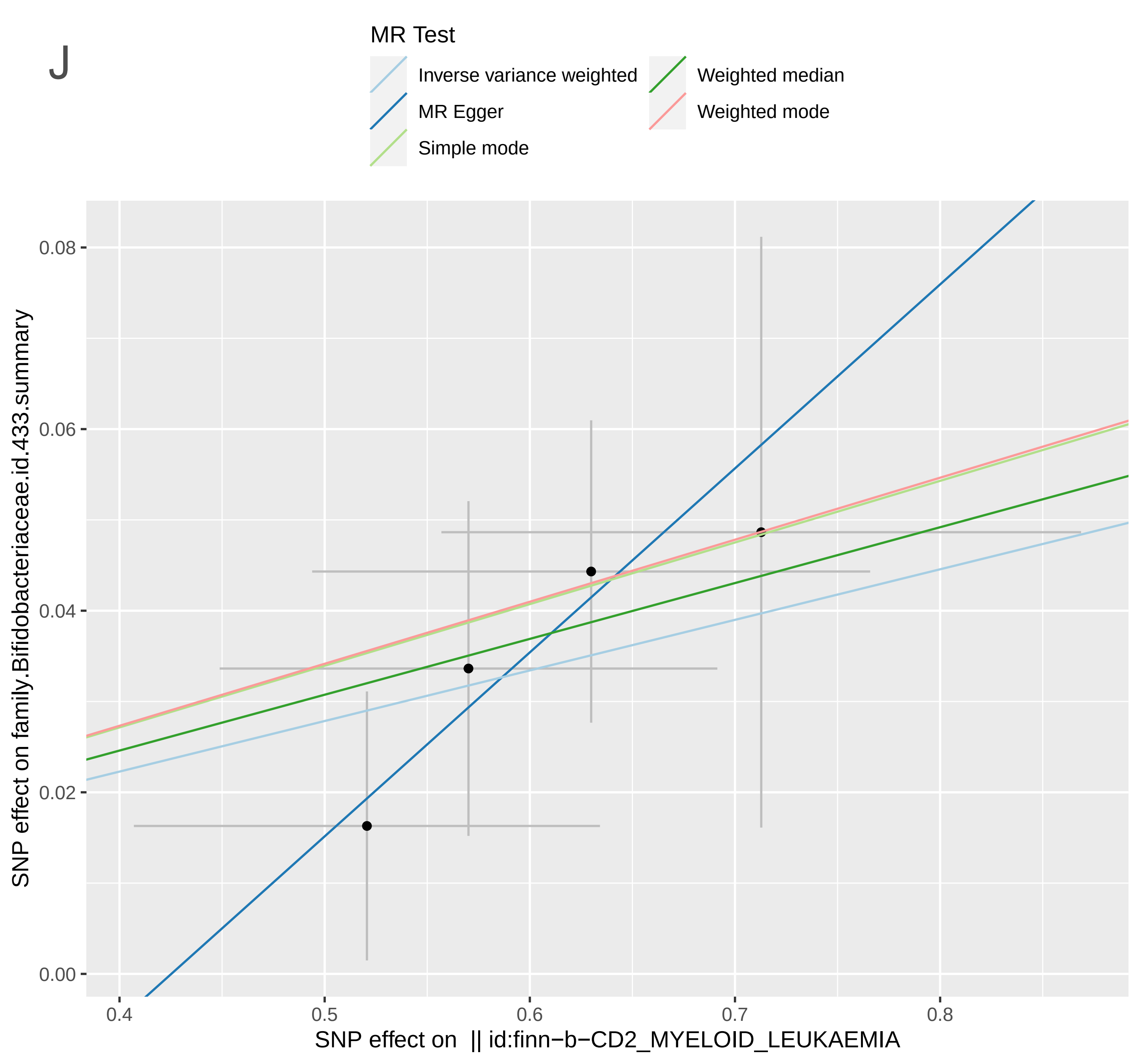

J
MR Test
Inverse variance weighted
MR Egger
Simple mode
Weighted median
Weighted mode
0.08
0.06
0.04
0.02
0.00
SNP effect on family.Bifidobacteriaceae.id.433.summary
0.4
0.5
0.6
0.7
0.8
SNP effect on || id:finn−b−CD2_MYELOID_LEUKAEMIA

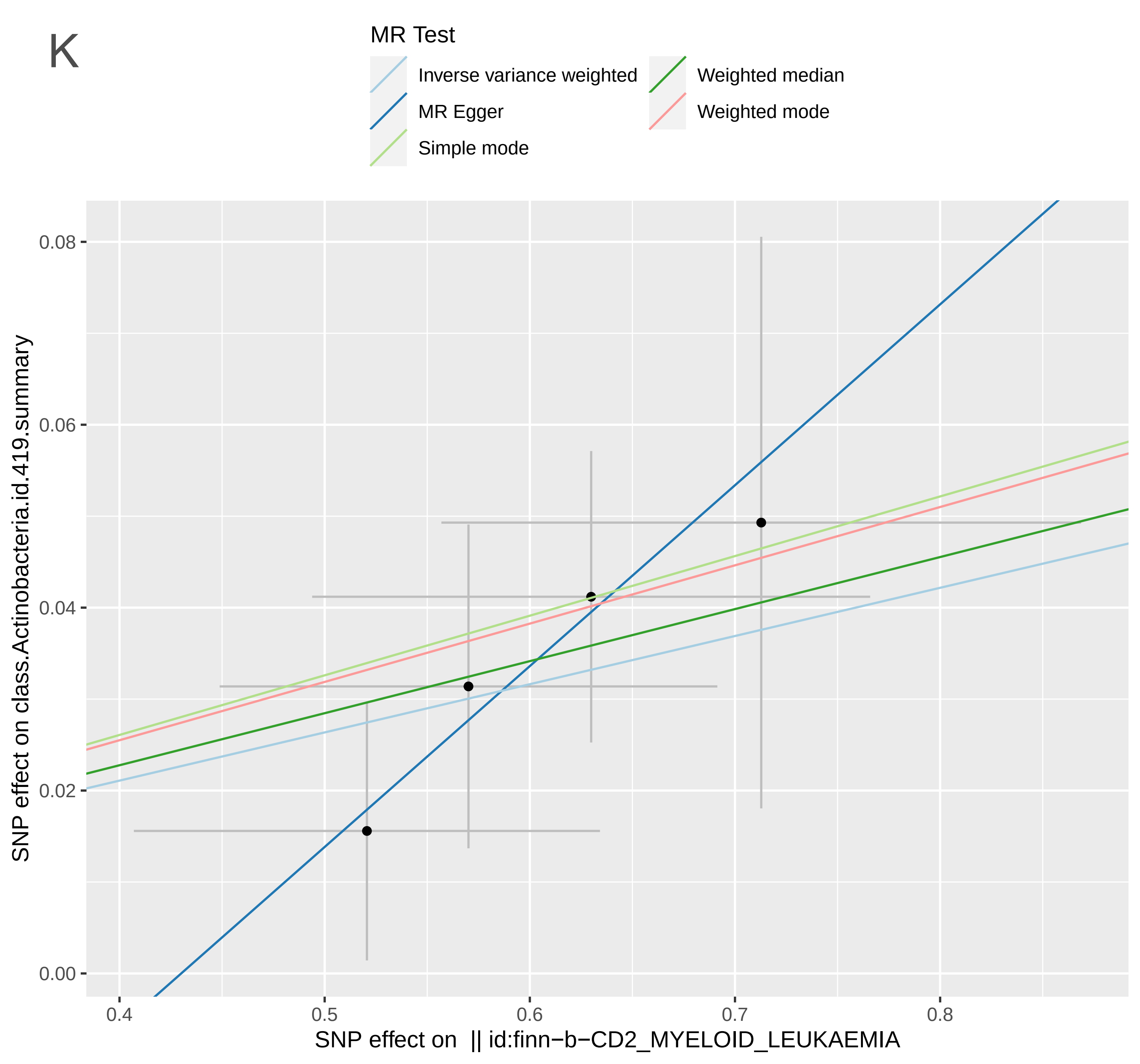
K
MR Test
Inverse variance weighted
MR Egger
Simple mode
Weighted median
Weighted mode
0.08
0.06
0.04
0.02
0.00
SNP effect on class.Actinobacteria.id.419.summary
0.4
0.5
0.6
0.7
0.8
SNP effect on || id:finn−b−CD2_MYELOID_LEUKAEMIA

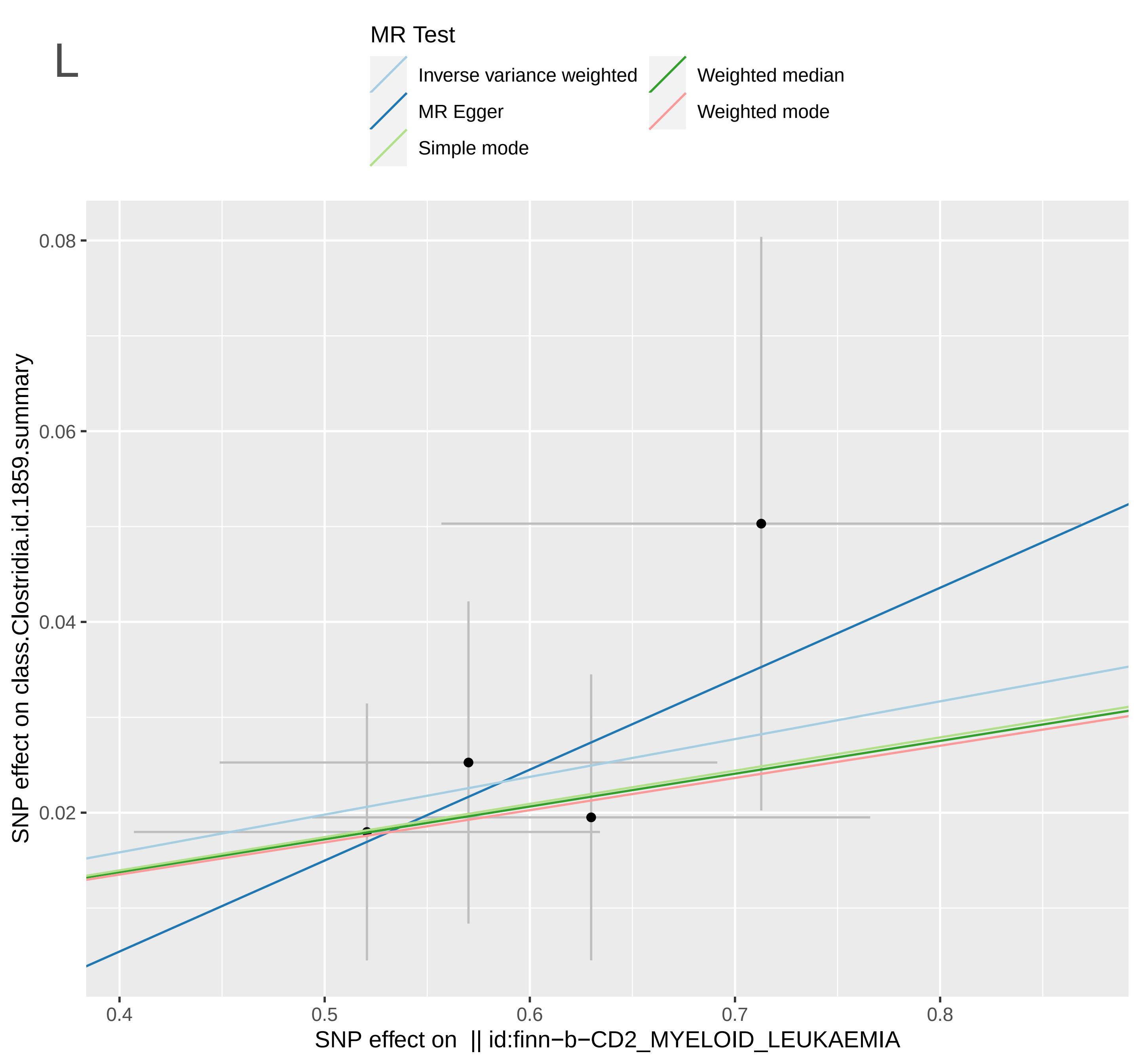

L
MR Test
Inverse variance weighted
MR Egger
Simple mode
Weighted median
Weighted mode
0.08
0.06
0.04
0.02
SNP effect on class.Clostridia.id.1859.summary
0.4
0.5
0.6
0.7
0.8
SNP effect on || id:finn−b−CD2_MYELOID_LEUKAEMIA

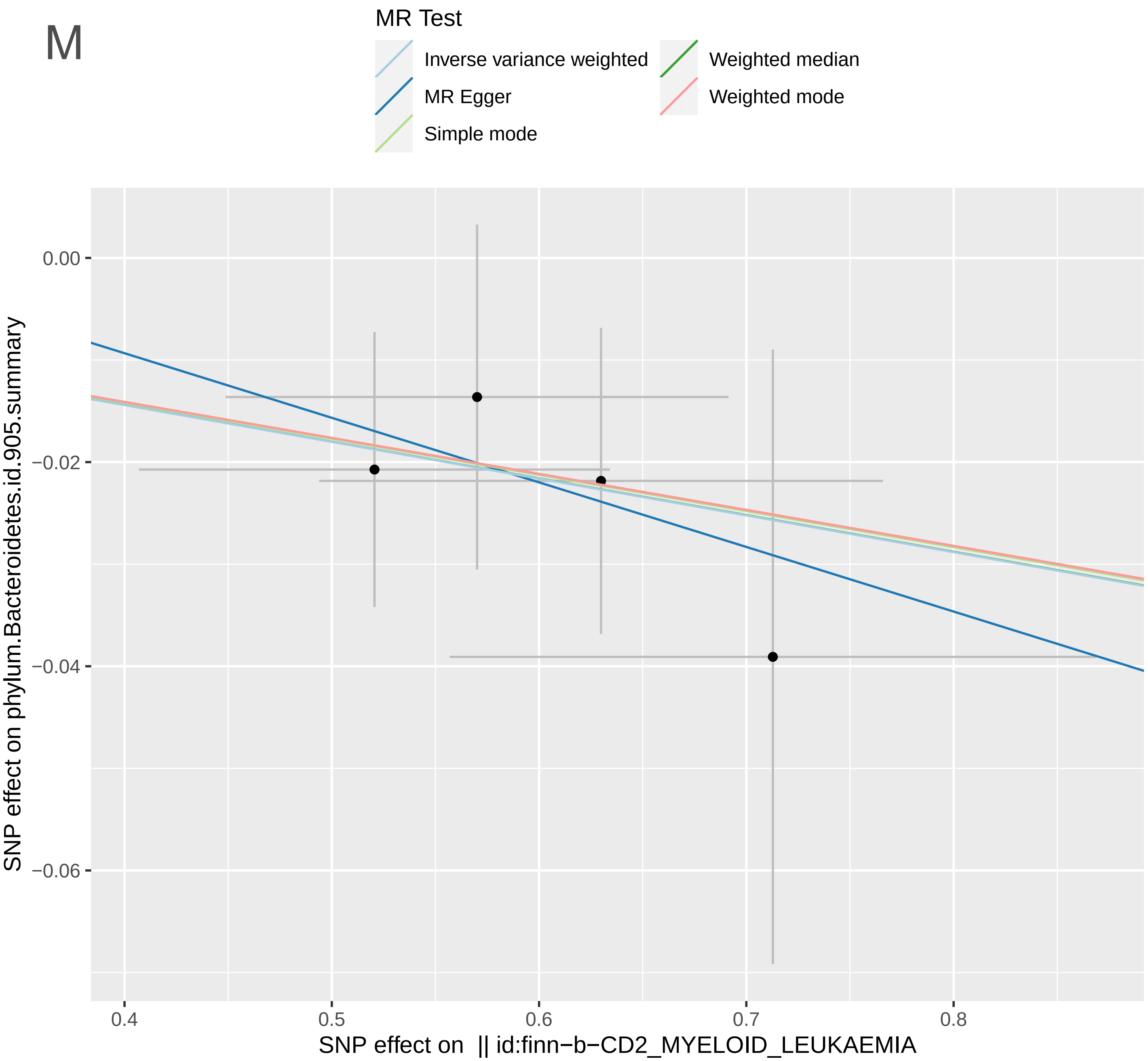
M
MR Test
Inverse variance weighted
MR Egger
Simple mode
Weighted median
Weighted mode
0.00
−0.02
−0.04
−0.06
0.4
0.5
0.6
0.7
0.8
SNP effect on phylum.Bacteroidetes.id.905.summary
SNP effect on || id:finn−b−CD2_MYELOID_LEUKAEMIA

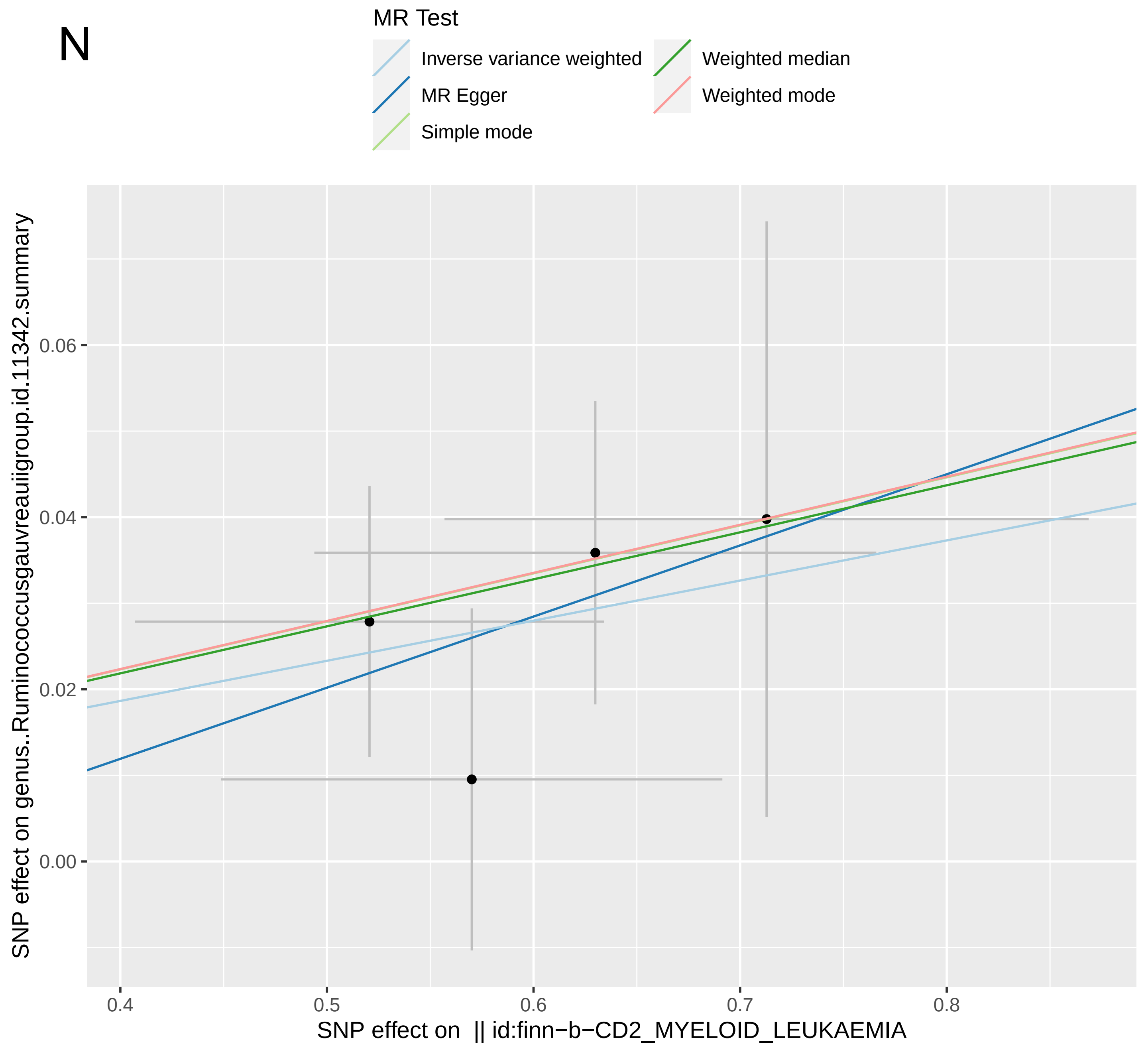
N
MR Test
Inverse variance weighted
MR Egger
Simple mode
Weighted median
Weighted mode
SNP effect on genus..Ruminococcusgauvreauiigroup.id.11342.summary
0.06
0.04
0.02
0.00
0.4
0.5
0.6
0.7
0.8
SNP effect on || id:finn−b−CD2_MYELOID_LEUKAEMIA

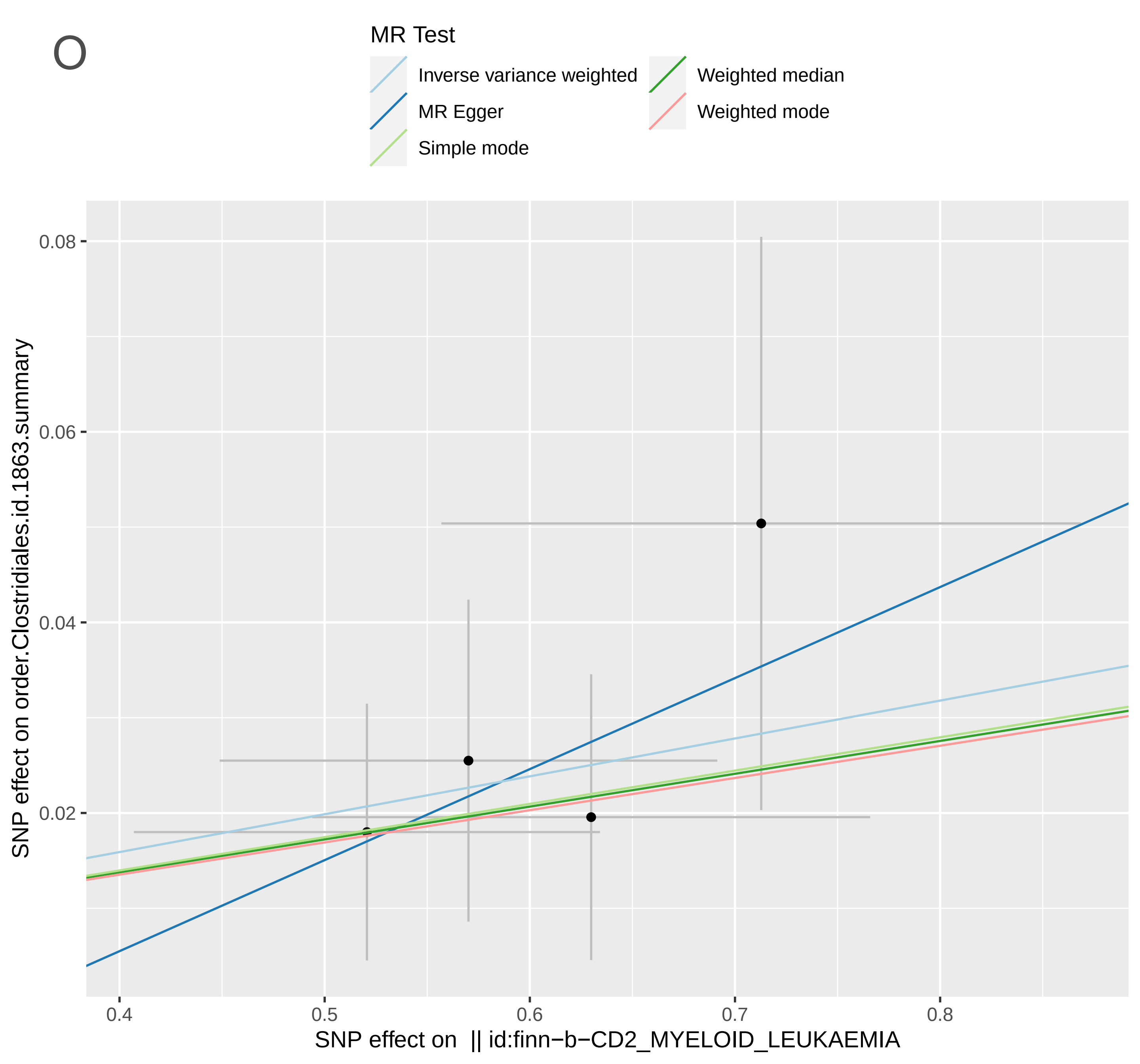

O
MR Test
Inverse variance weighted
MR Egger
Simple mode
Weighted median
Weighted mode
0.08
0.06
0.04
0.02
0.4
0.5
0.6
0.7
0.8
SNP effect on order.Clostridiales.id.1863.summary
SNP effect on || id:finn−b−CD2_MYELOID_LEUKAEMIA

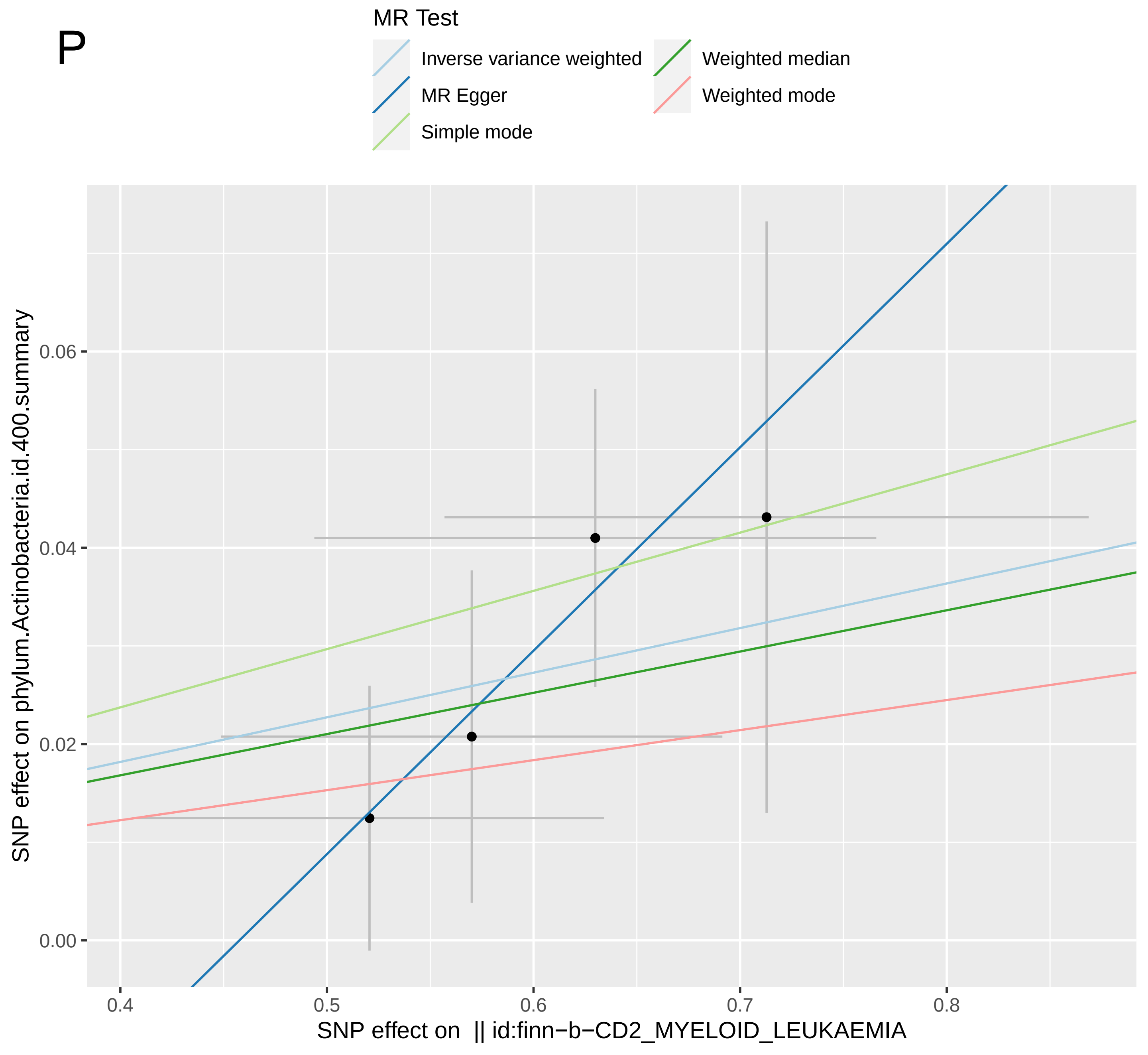
P
MR Test
Inverse variance weighted
MR Egger
Simple mode
Weighted median
Weighted mode
SNP effect on phylum.Actinobacteria.id.400.summary
0.06
0.04
0.02
0.00
0.4
0.5
0.6
0.7
0.8
SNP effect on || id:finn−b−CD2_MYELOID_LEUKAEMIA

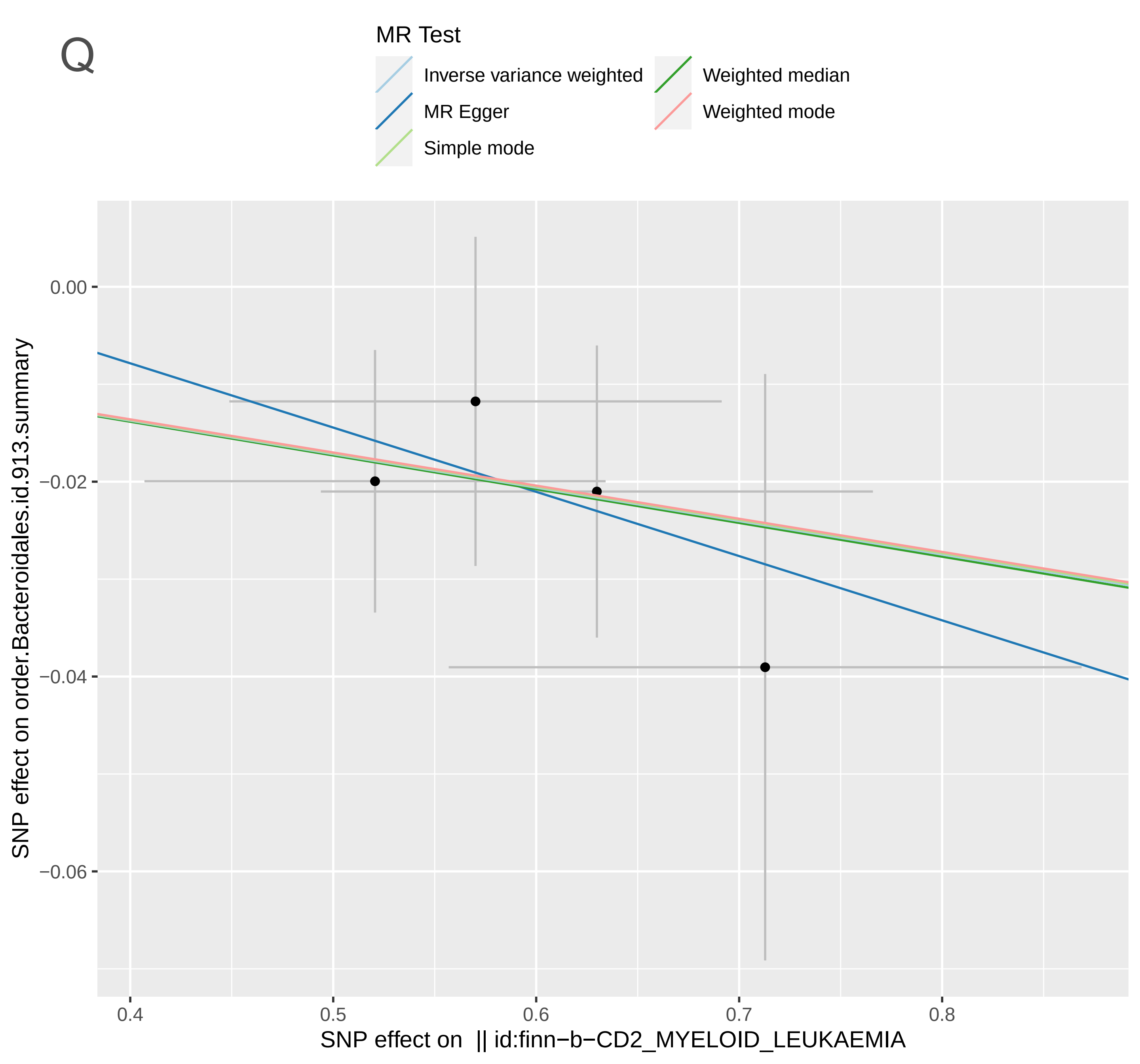
Q
MR Test
Inverse variance weighted
MR Egger
Simple mode
Weighted median
Weighted mode
0.00
−0.02
−0.04
−0.06
SNP effect on order.Bacteroidales.id.913.summary
0.4
0.5
0.6
0.7
0.8
SNP effect on || id:finn−b−CD2_MYELOID_LEUKAEMIA

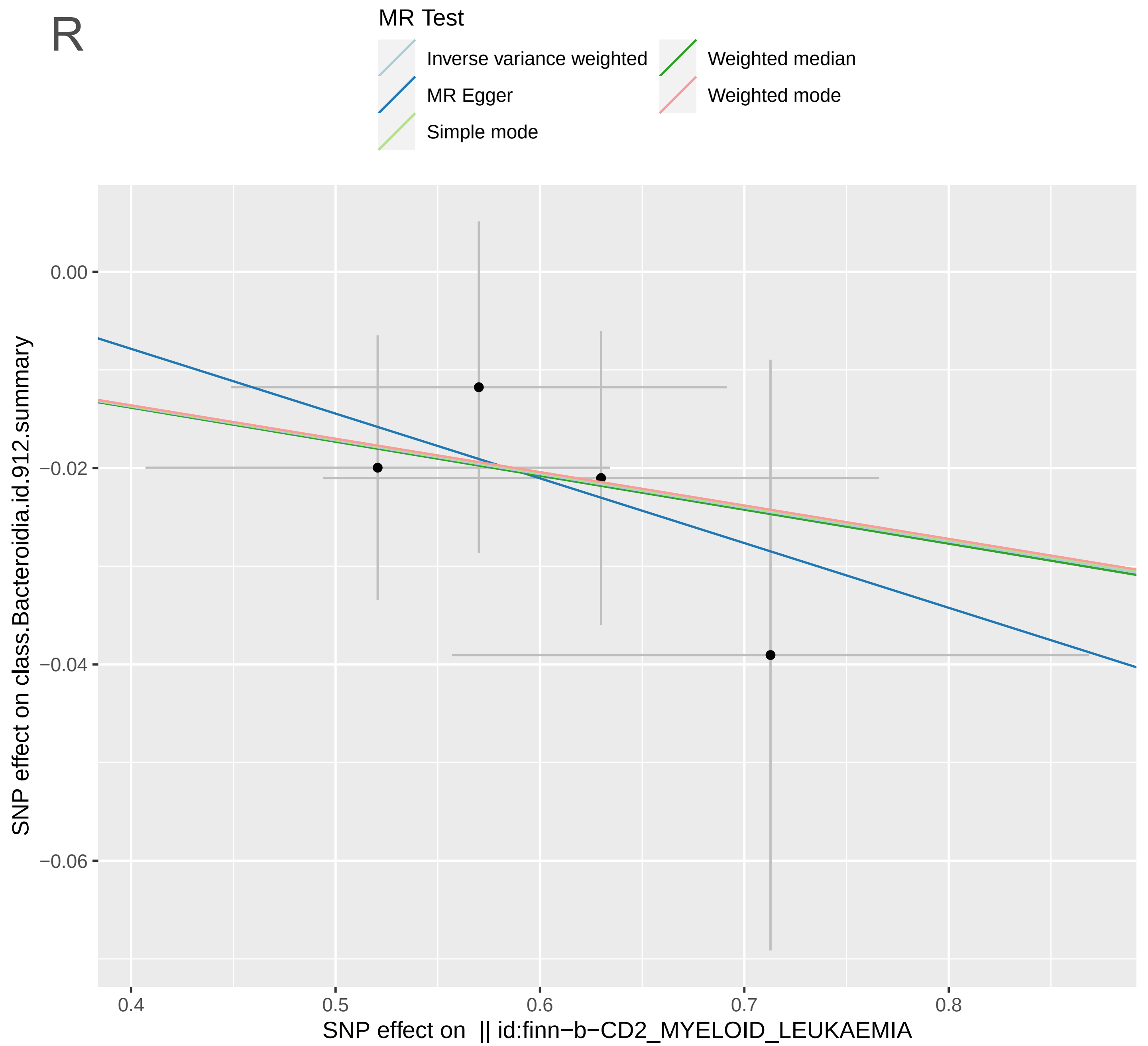
R
MR Test
Inverse variance weighted
MR Egger
Simple mode
Weighted median
Weighted mode
0.00
−0.02
−0.04
−0.06
SNP effect on class.Bacteroidia.id.912.summary
0.4
0.5
0.6
0.7
0.8
SNP effect on || id:finn−b−CD2_MYELOID_LEUKAEMIA

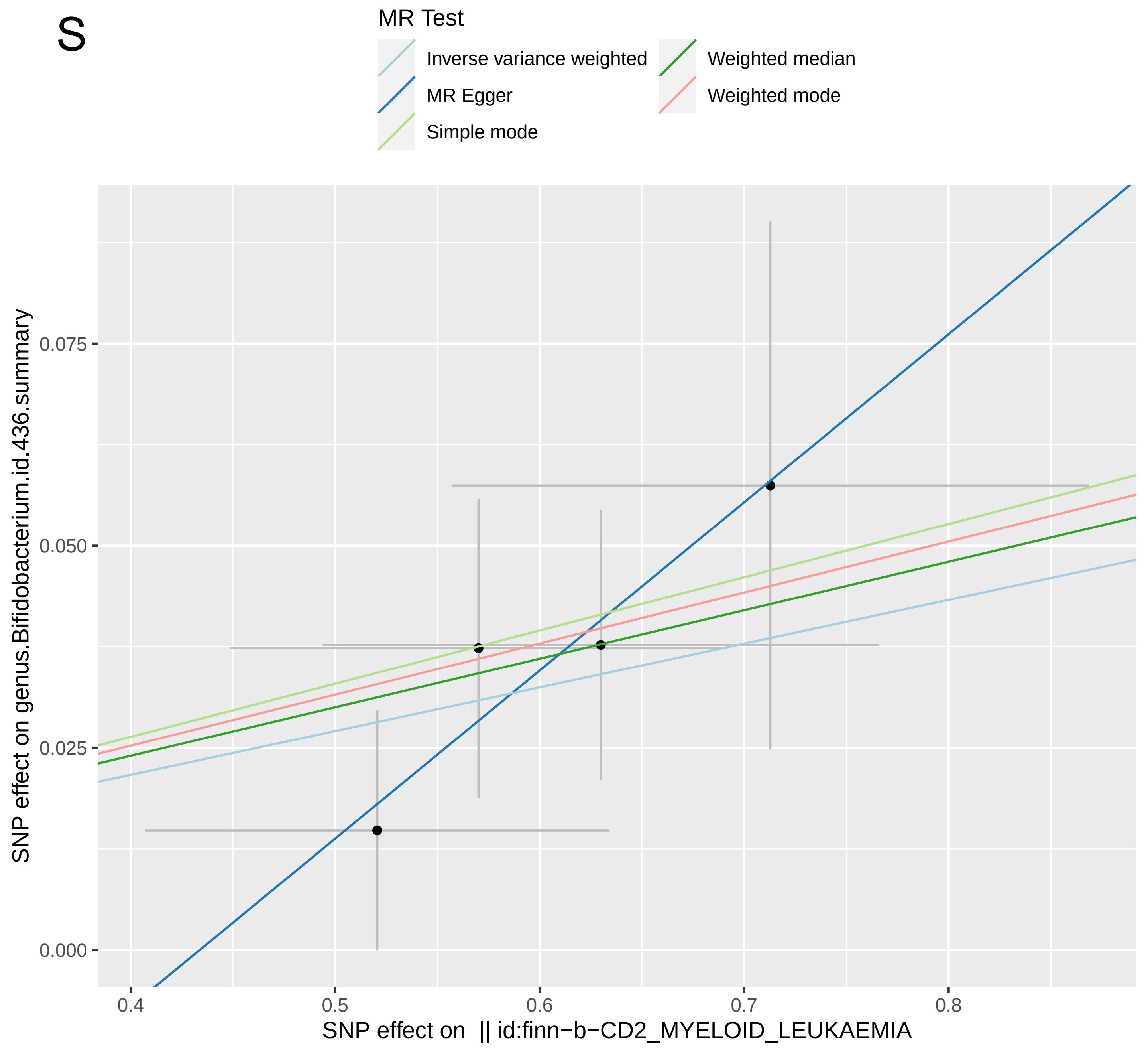
S
MR Test
Inverse variance weighted
MR Egger
Simple mode
Weighted median
Weighted mode
0.000
0.025
0.050
0.075
SNP effect on genus.Bifidobacterium.id.436.summary
0.4
0.5
0.6
0.7
0.8
SNP effect on || id:finn−b−CD2_MYELOID_LEUKAEMIA

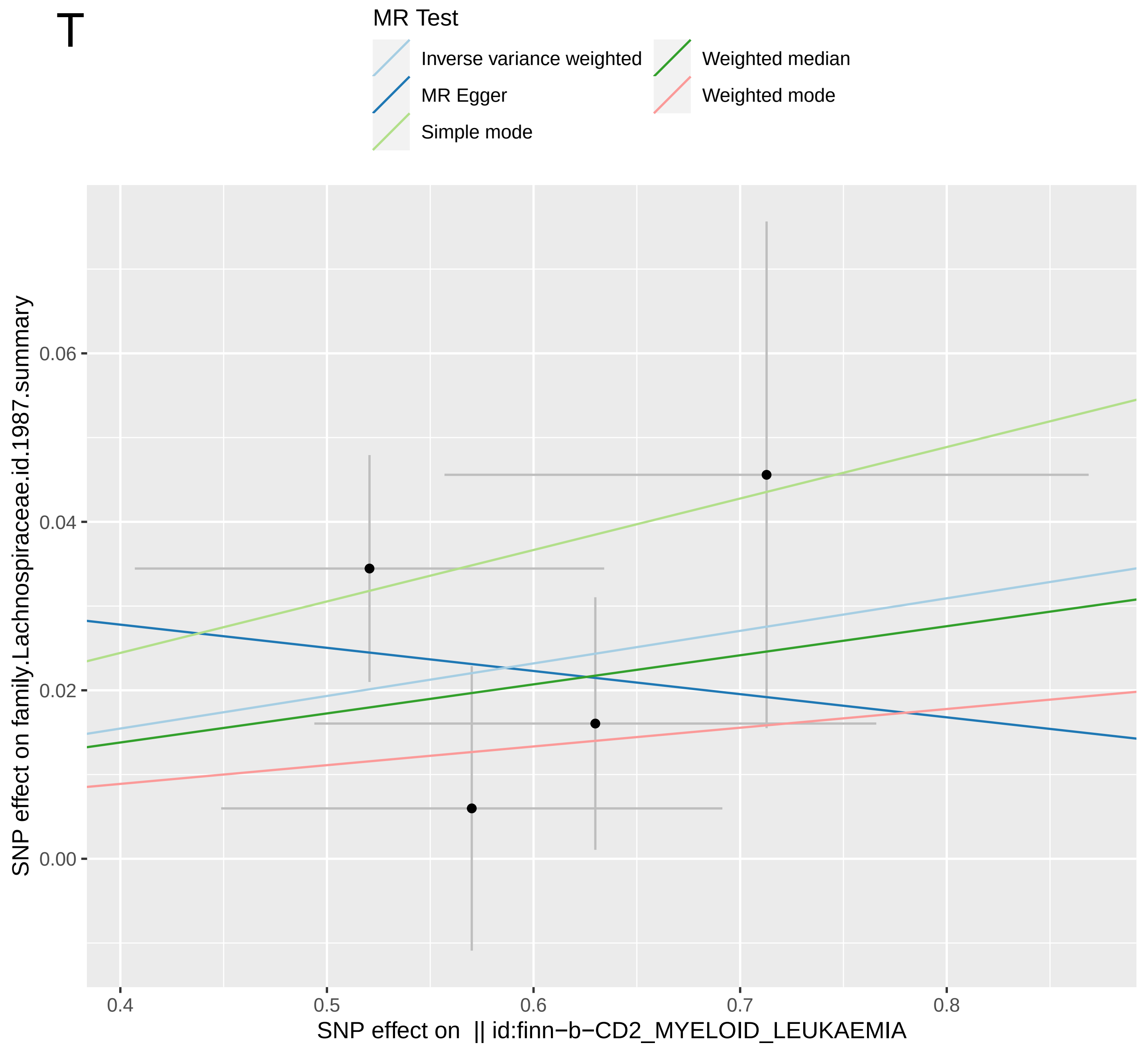
T
MR Test
Inverse variance weighted
MR Egger
Simple mode
Weighted median
Weighted mode
0.06
0.04
0.02
0.00
0.4
0.5
0.6
0.7
0.8
SNP effect on family.Lachnospiraceae.id.1987.summary
SNP effect on || id:finn−b−CD2_MYELOID_LEUKAEMIA

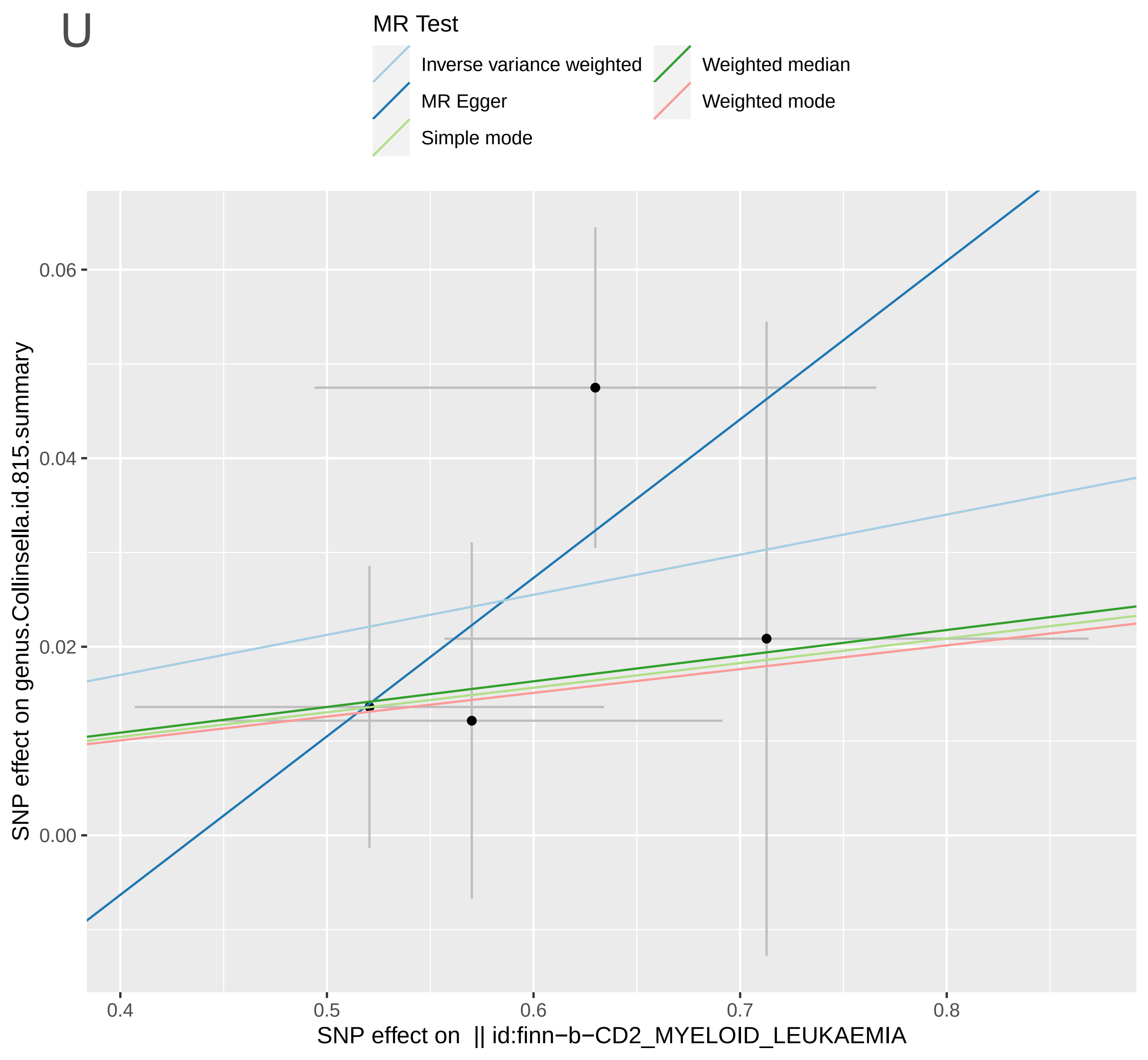

U
MR Test
Inverse variance weighted
MR Egger
Simple mode
Weighted median
Weighted mode
0.06
0.04
0.02
0.00
SNP effect on genus.Collinsella.id.815.summary
0.4
0.5
0.6
0.7
0.8
SNP effect on || id:finn−b−CD2_MYELOID_LEUKAEMIA

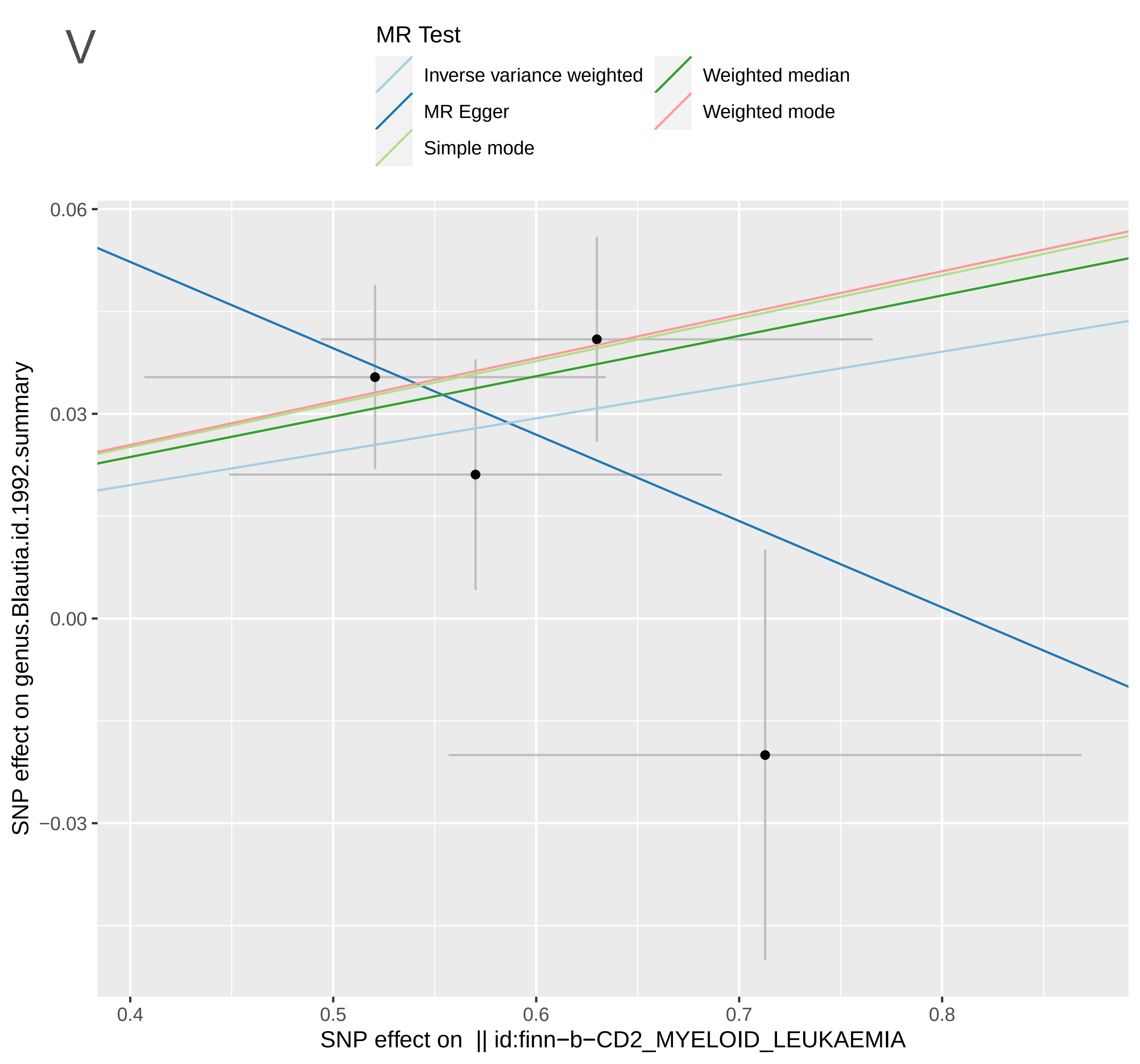
V
MR Test
Inverse variance weighted
MR Egger
Simple mode
Weighted median
Weighted mode
SNP effect on genus.Blautia.id.1992.summary
0.06
0.03
0.00
−0.03
0.4
0.5
0.6
0.7
0.8
SNP effect on || id:finn−b−CD2_MYELOID_LEUKAEMIA

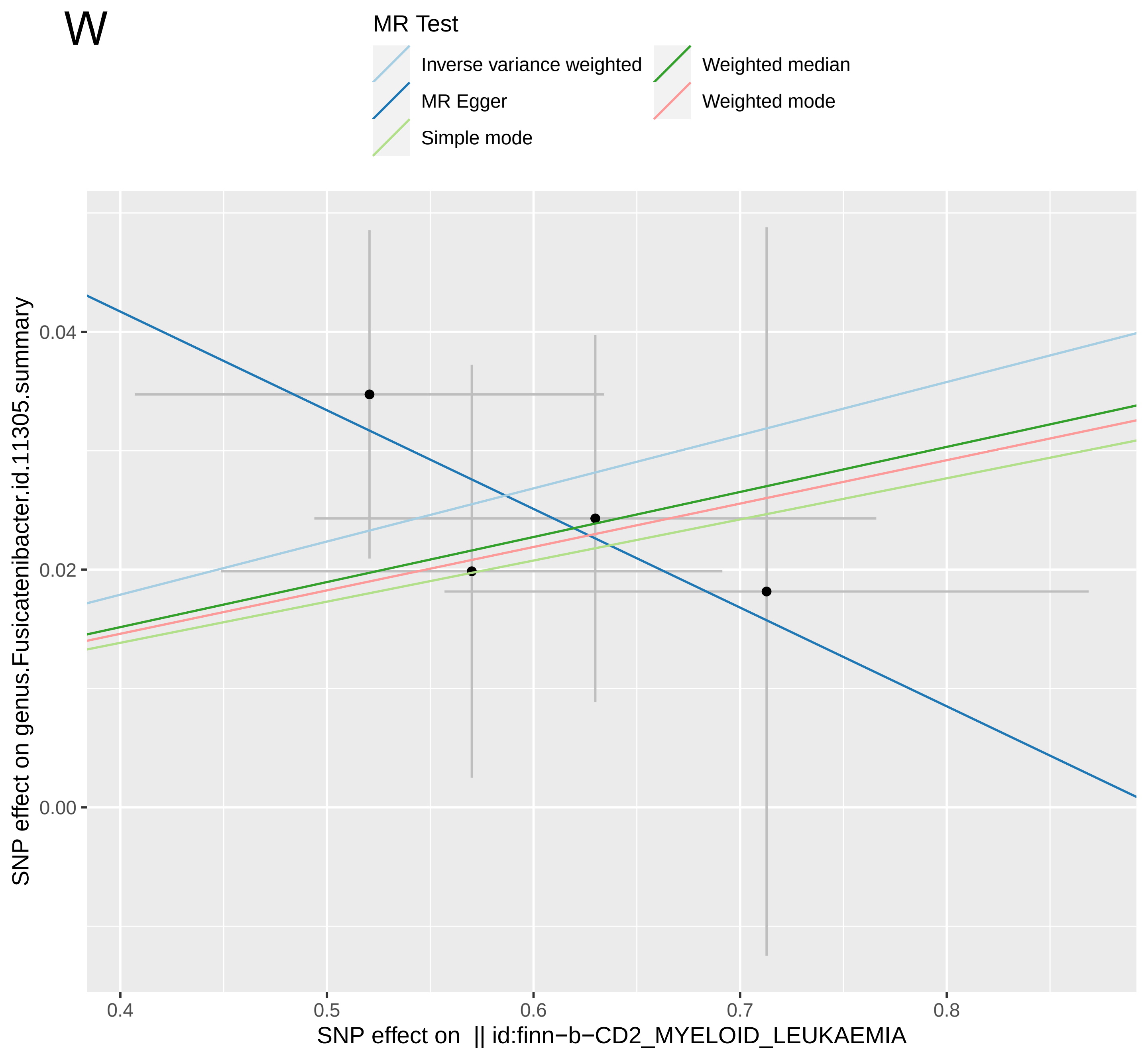
W
MR Test
Inverse variance weighted
MR Egger
Simple mode
Weighted median
Weighted mode
0.04
0.02
0.00
0.4
0.5
0.6
0.7
0.8
SNP effect on genus.Fusicatenibacter.id.11305.summary
SNP effect on || id:finn−b−CD2_MYELOID_LEUKAEMIA

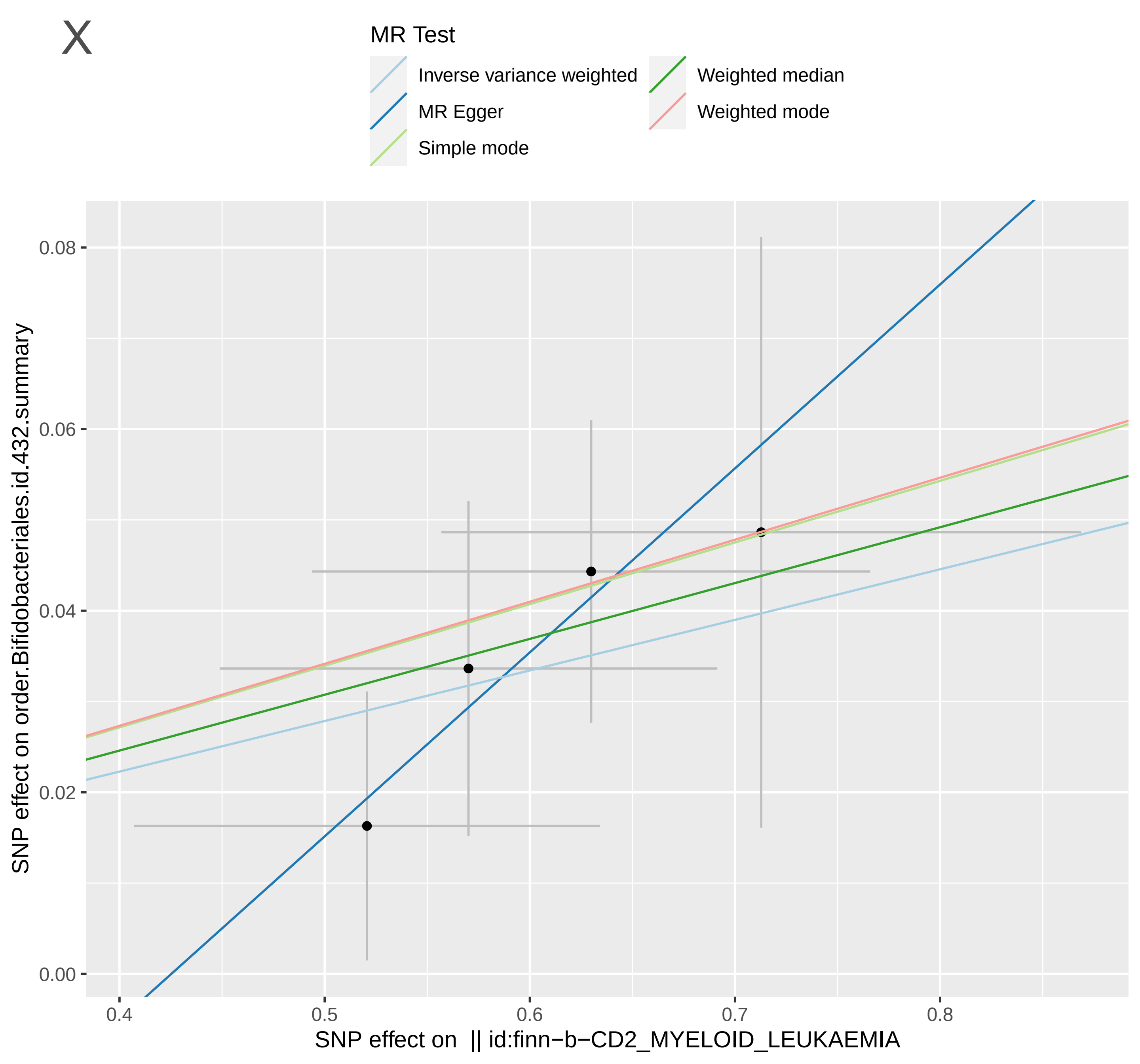
X
MR Test
Inverse variance weighted
MR Egger
Simple mode
Weighted median
Weighted mode
0.08
0.06
0.04
0.02
0.00
0.4
0.5
0.6
0.7
0.8
SNP effect on order.Bifidobacteriales.id.432.summary
SNP effect on || id:finn−b−CD2_MYELOID_LEUKAEMIA

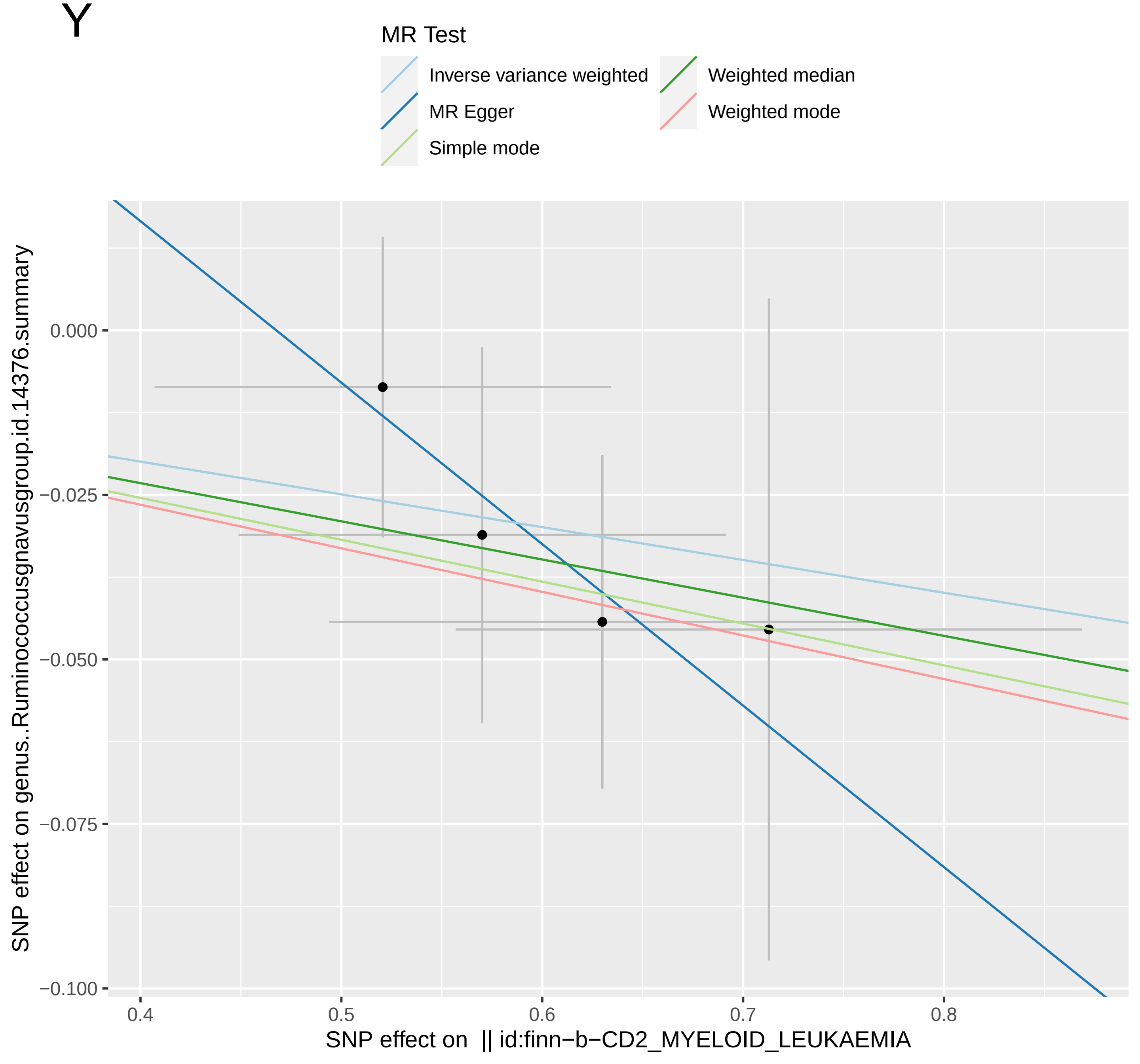

Y
MR Test
Inverse variance weighted
MR Egger
Simple mode
Weighted median
Weighted mode
0.000
−0.025
−0.050
−0.075
−0.100
0.4
0.5
0.6
0.7
0.8
SNP effect on genus..Ruminococcusgnavusgroup.id.14376.summary
SNP effect on || id:finn−b−CD2_MYELOID_LEUKAEMIA

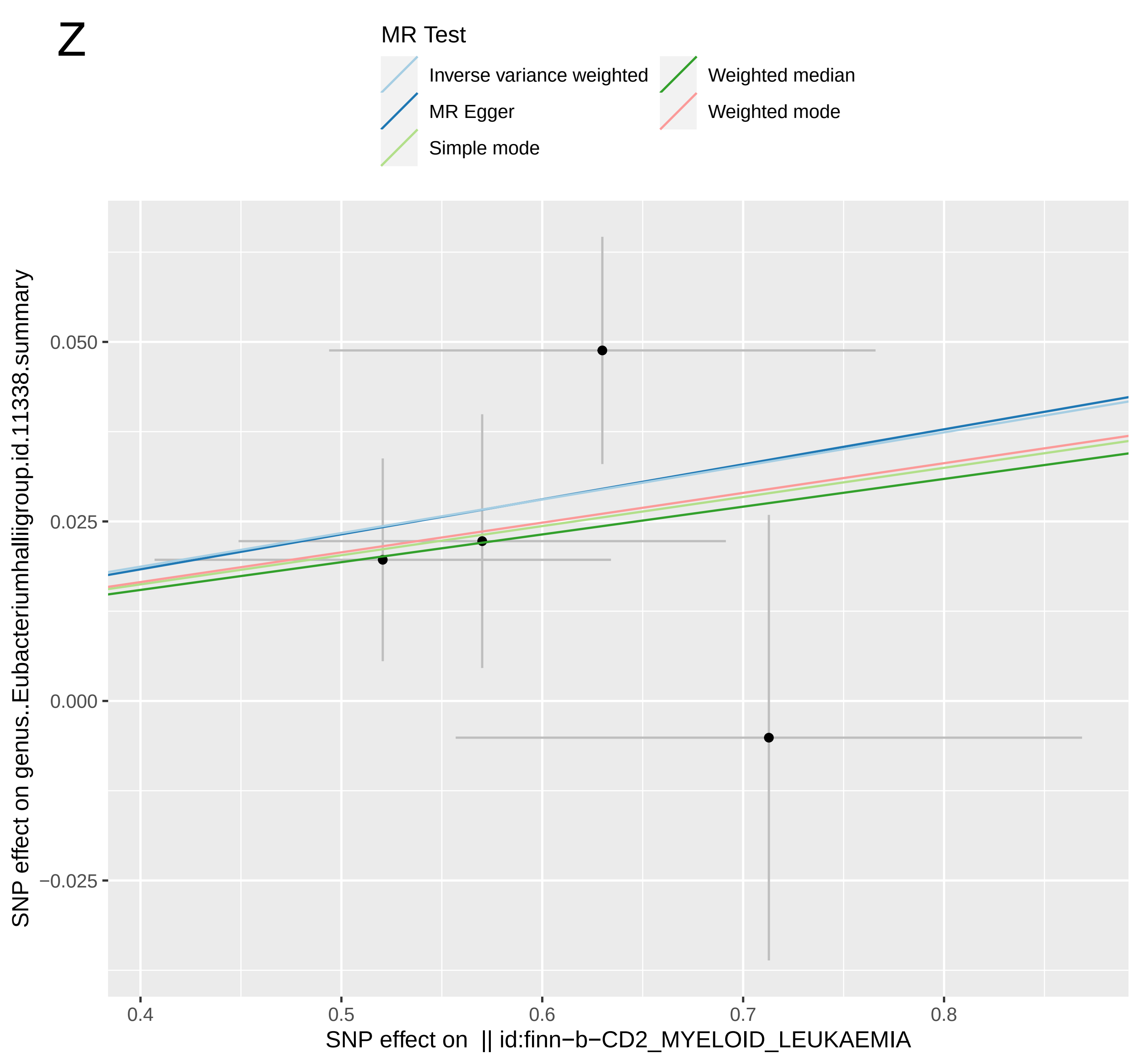

Z
MR Test
Inverse variance weighted
MR Egger
Simple mode
Weighted median
Weighted mode
0.050
0.025
0.000
−0.025
0.4
0.5
0.6
0.7
0.8
SNP effect on genus..Eubacteriumhalliigroup.id.11338.summary
SNP effect on || id:finn−b−CD2_MYELOID_LEUKAEMIA

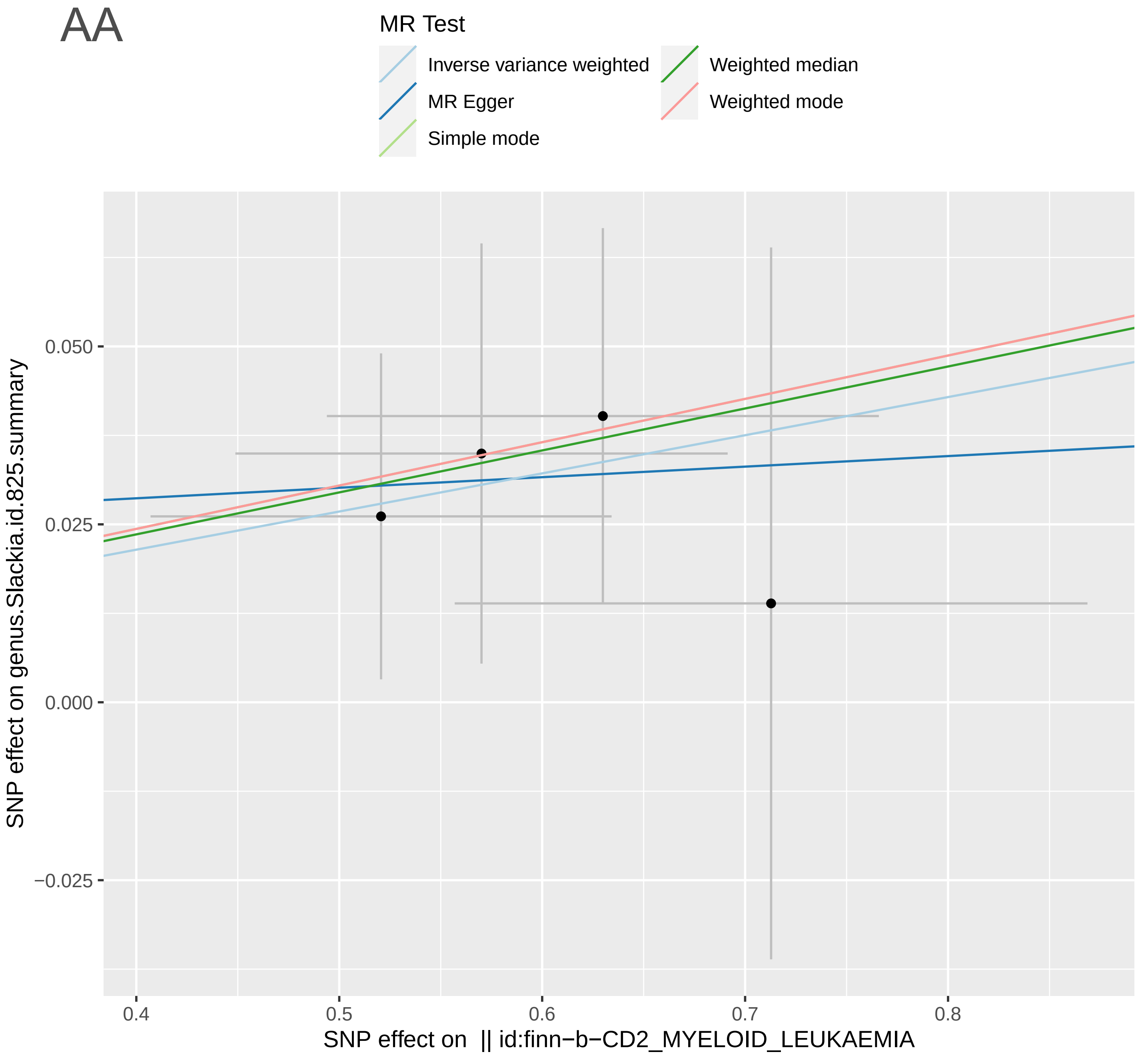
AA
MR Test
Inverse variance weighted
MR Egger
Simple mode
Weighted median
Weighted mode
SNP effect on genus.Slackia.id.825.summary
0.050
0.025
0.000
−0.025
0.4
0.5
0.6
0.7
0.8
SNP effect on || id:finn−b−CD2_MYELOID_LEUKAEMIA

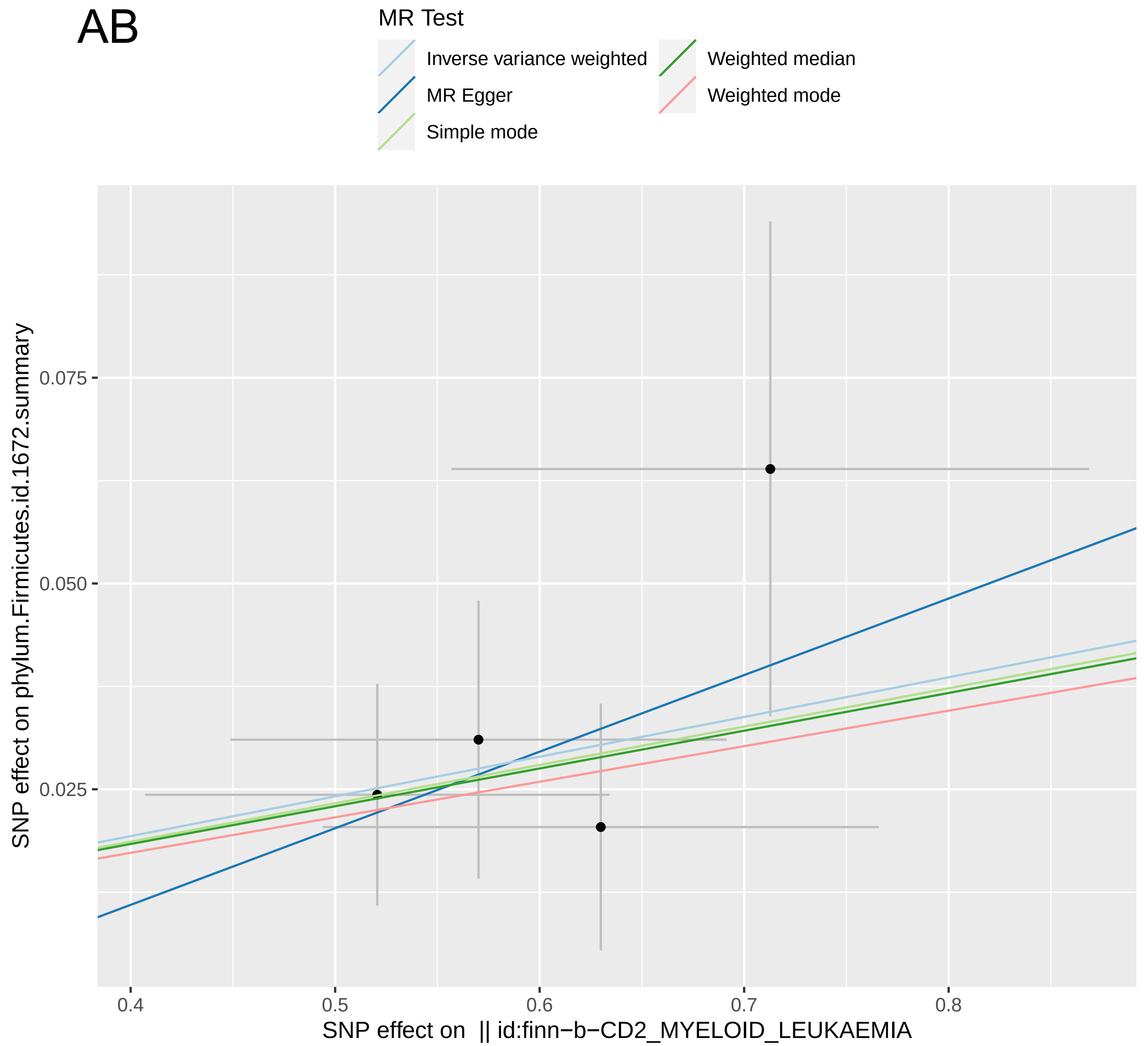
AB
MR Test
Inverse variance weighted
MR Egger
Simple mode
Weighted median
Weighted mode
SNP effect on phylum.Firmicutes.id.1672.summary
0.075
0.050
0.025
0.4
0.5
0.6
0.7
0.8
SNP effect on || id:finn−b−CD2_MYELOID_LEUKAEMIA

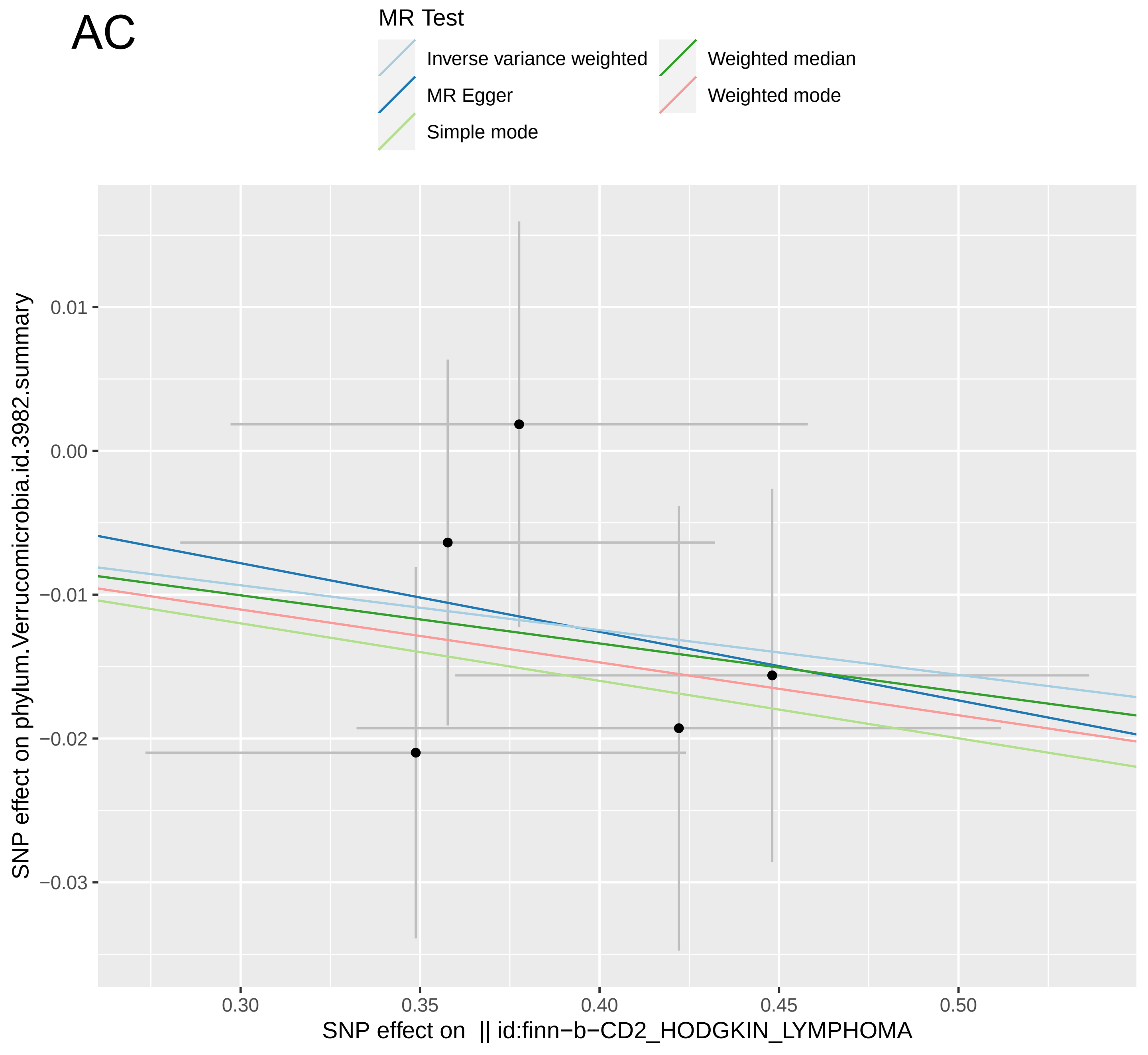

AC
MR Test
Inverse variance weighted
MR Egger
Simple mode
Weighted median
Weighted mode
0.01
0.00
−0.01
−0.02
−0.03
0.30
0.35
0.40
0.45
0.50
SNP effect on phylum.Verrucomicrobia.id.3982.summary
SNP effect on || id:finn−b−CD2_HODGKIN_LYMPHOMA

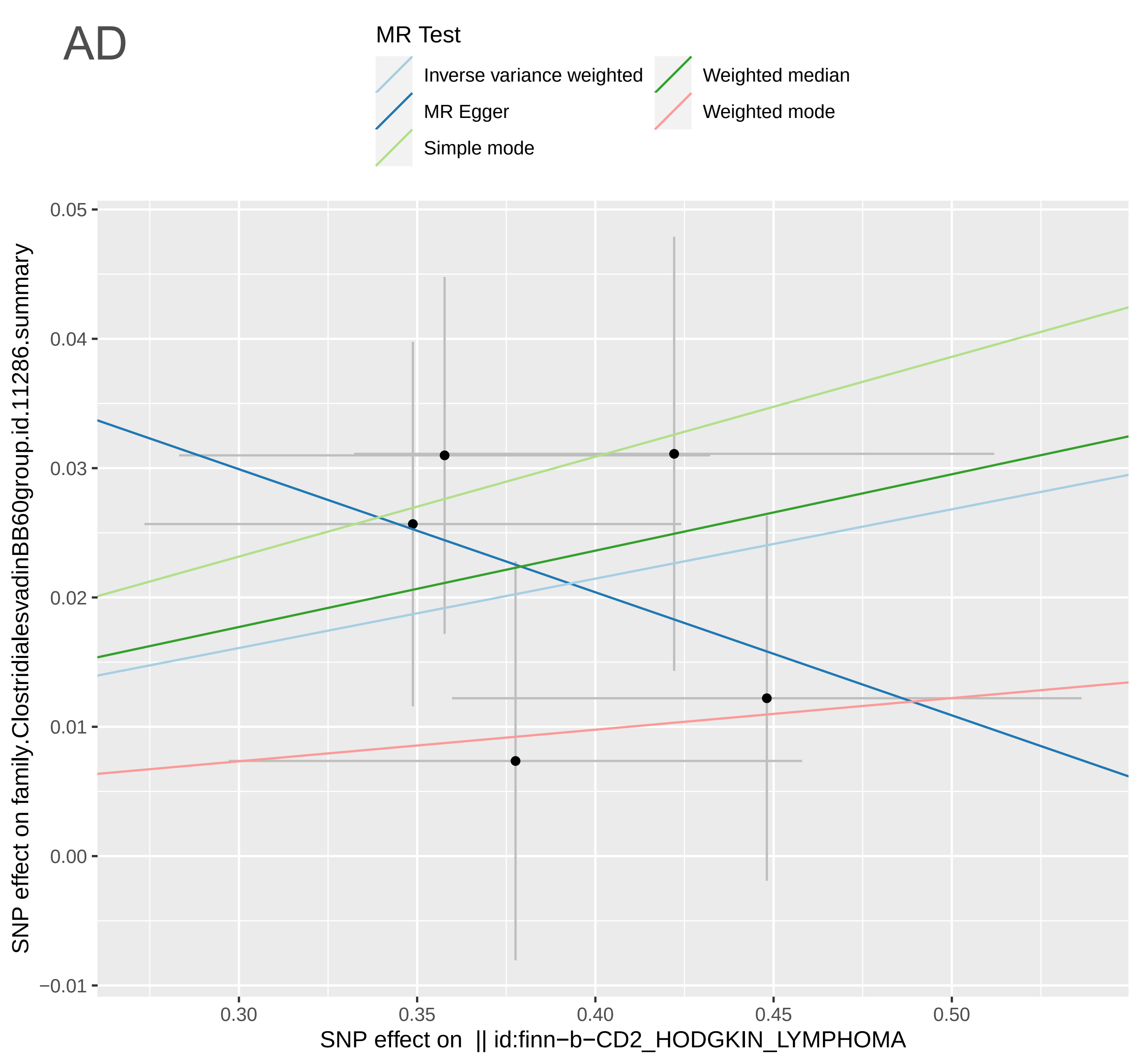
AD
MR Test
Inverse variance weighted
MR Egger
Simple mode
Weighted median
Weighted mode
0.05
0.04
0.03
0.02
0.01
0.00
−0.01
SNP effect on family.ClostridialesvadinBB60group.id.11286.summary
0.30
0.35
0.40
0.45
0.50
SNP effect on || id:finn−b−CD2_HODGKIN_LYMPHOMA

AE

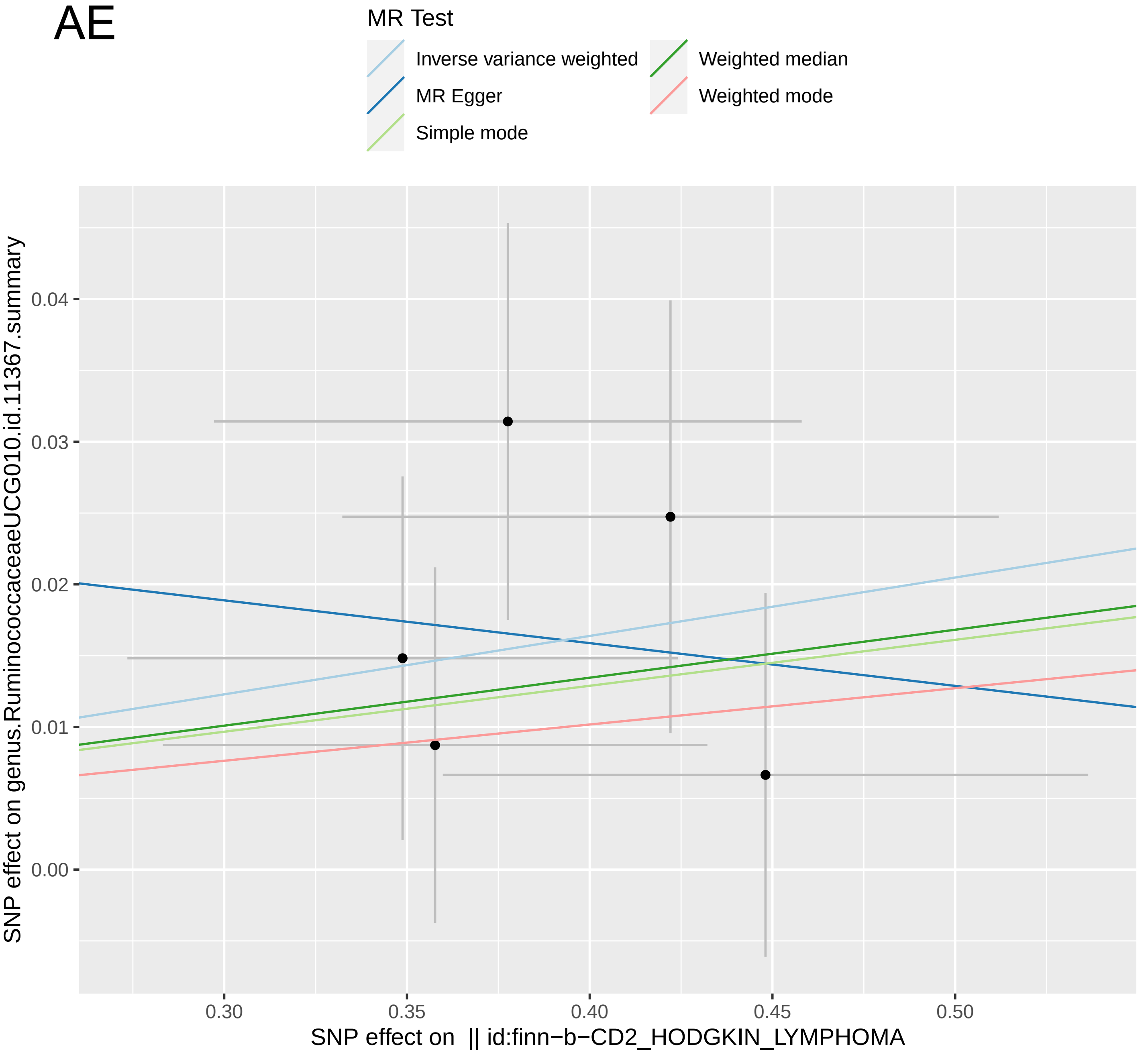

MR Test
Inverse variance weighted
MR Egger
Simple mode
Weighted median
Weighted mode
SNP effect on genus.RuminococcaceaeUCG010.id.11367.summary
0.04
0.03
0.02
0.01
0.00
0.30
0.35
0.40
0.45
0.50
SNP effect on || id:finn−b−CD2_HODGKIN_LYMPHOMA

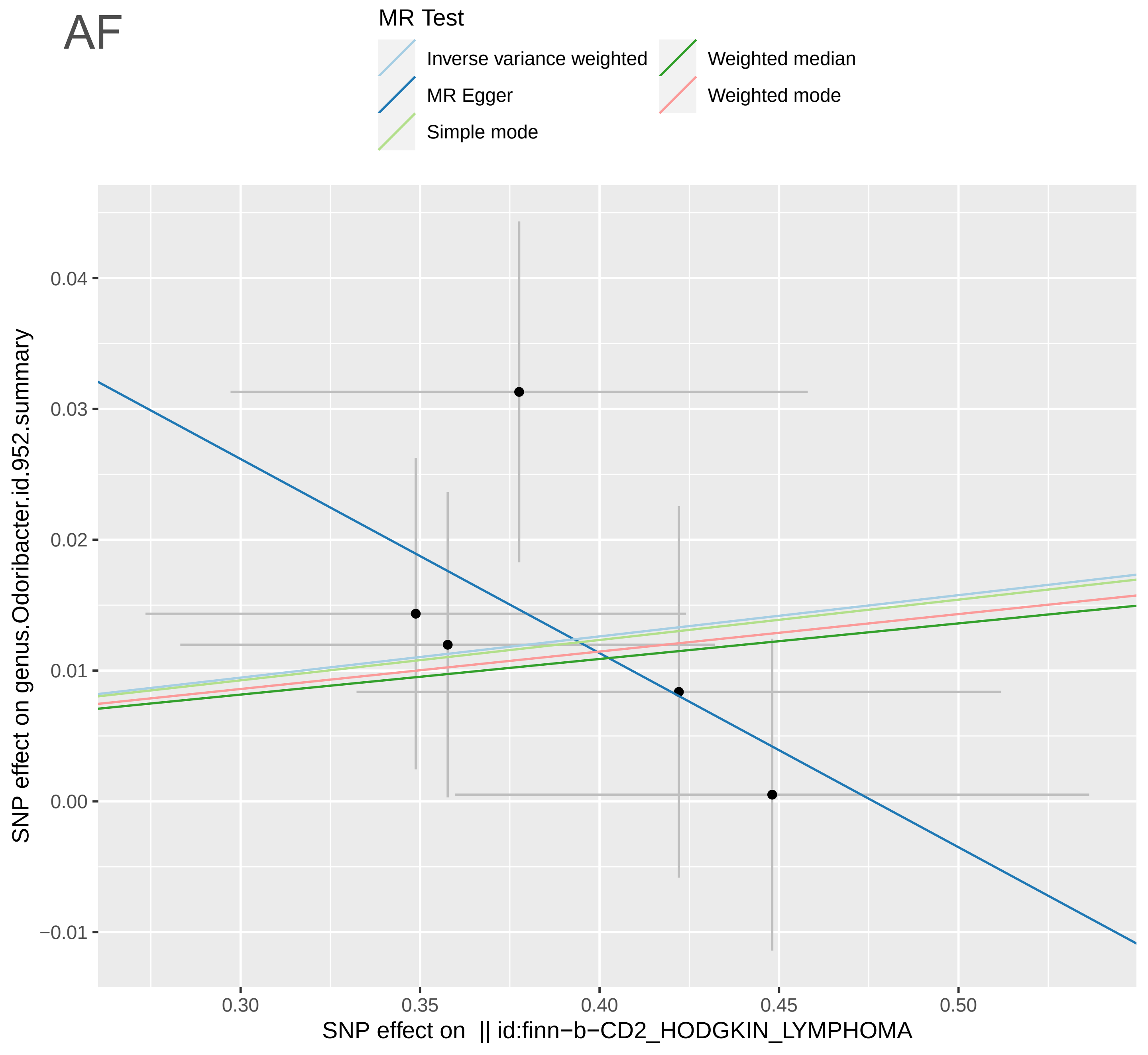

AF
MR Test
Inverse variance weighted
MR Egger
Simple mode
Weighted median
Weighted mode
SNP effect on genus.Odoribacter.id.952.summary
0.04
0.03
0.02
0.01
0.00
−0.01
0.30
0.35
0.40
0.45
0.50
SNP effect on || id:finn−b−CD2_HODGKIN_LYMPHOMA

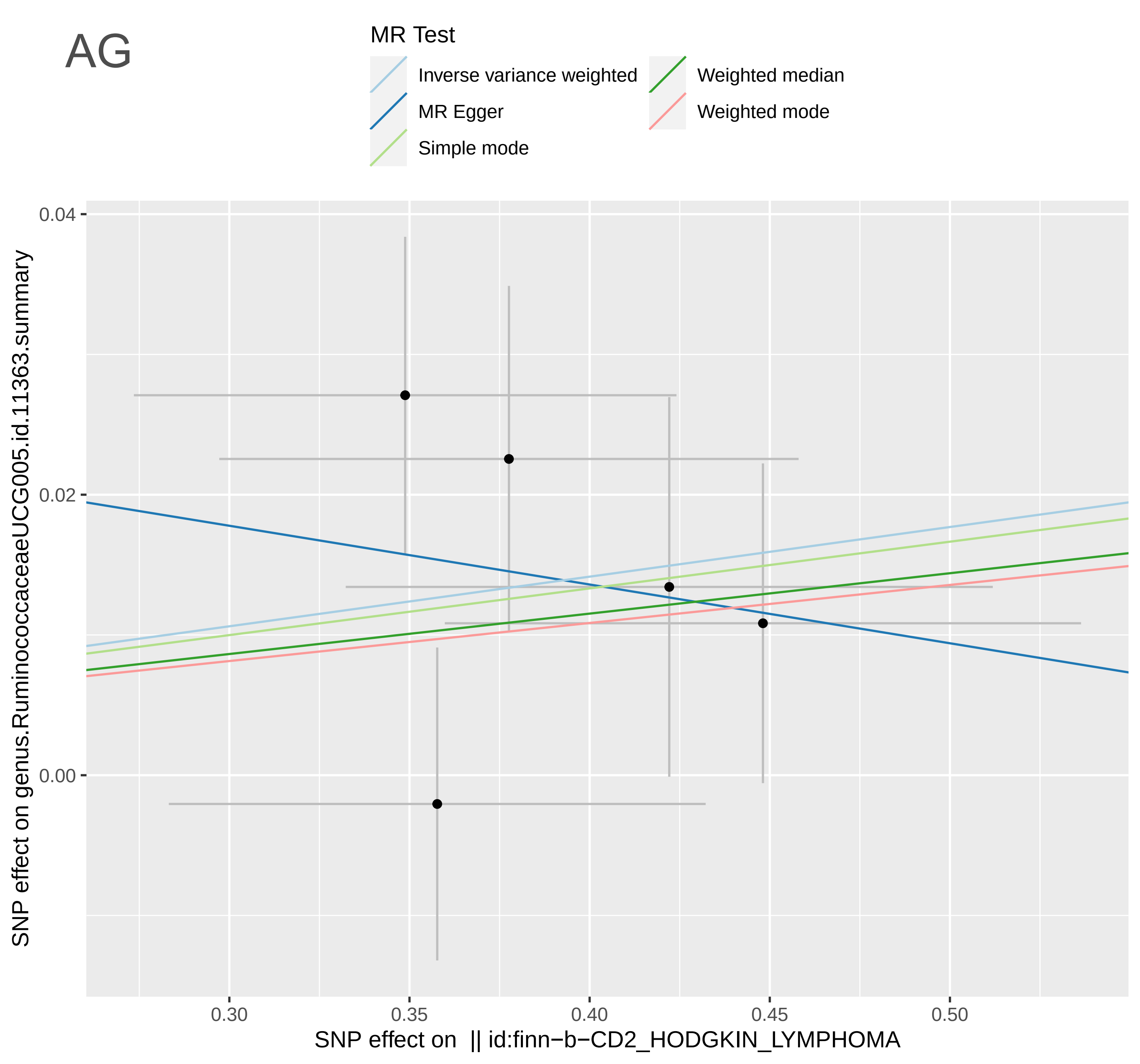
AG
MR Test
Inverse variance weighted
MR Egger
Simple mode
Weighted median
Weighted mode
0.04
0.02
0.00
0.30
0.35
0.40
0.45
0.50
SNP effect on genus.RuminococcaceaeUCG005.id.11363.summary
SNP effect on || id:finn−b−CD2_HODGKIN_LYMPHOMA

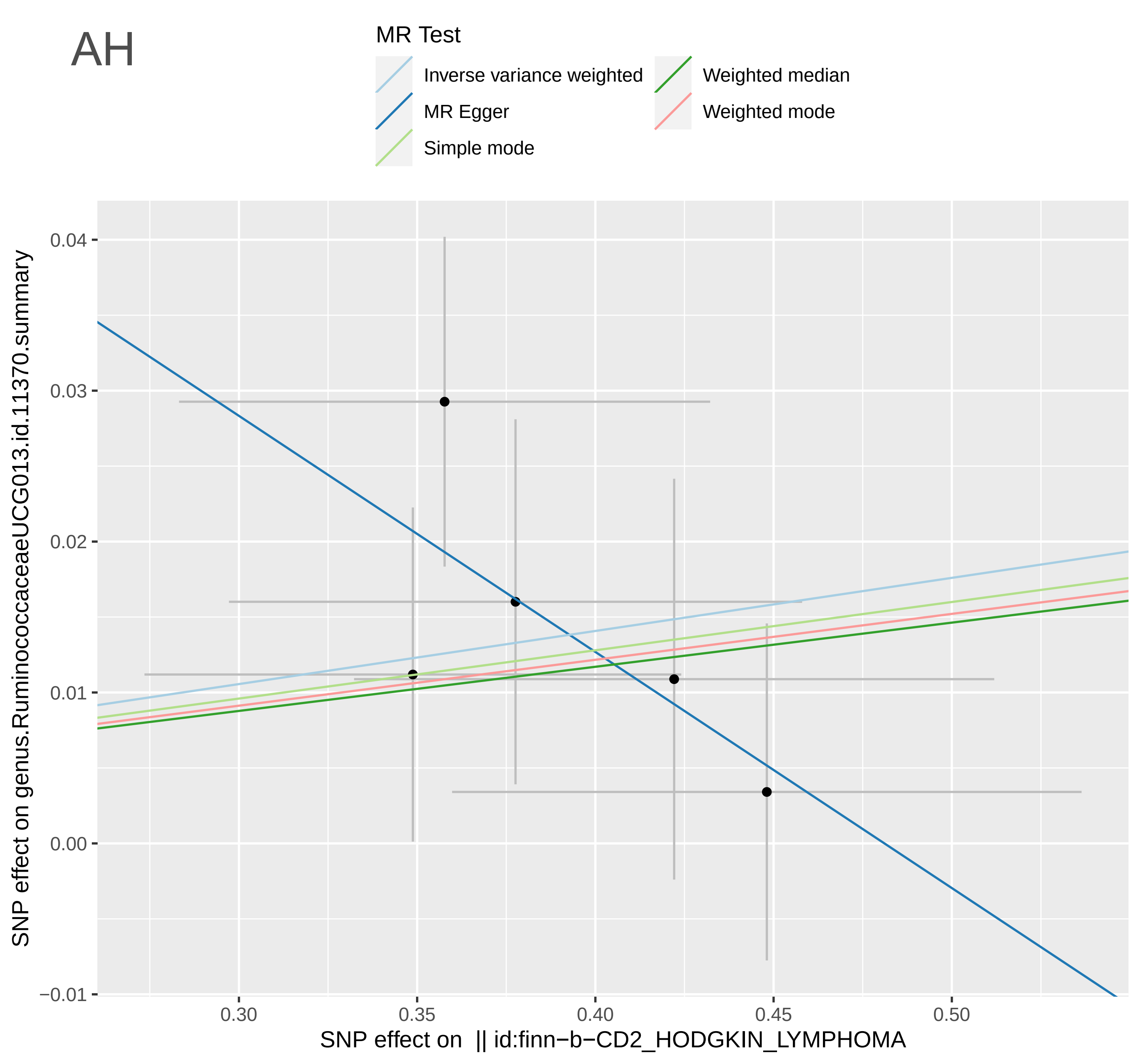

AH
MR Test
Inverse variance weighted
MR Egger
Simple mode
Weighted median
Weighted mode
SNP effect on genus.RuminococcaceaeUCG013.id.11370.summary
0.04
0.03
0.02
0.01
0.00
−0.01
0.30
0.35
0.40
0.45
0.50
SNP effect on || id:finn−b−CD2_HODGKIN_LYMPHOMA

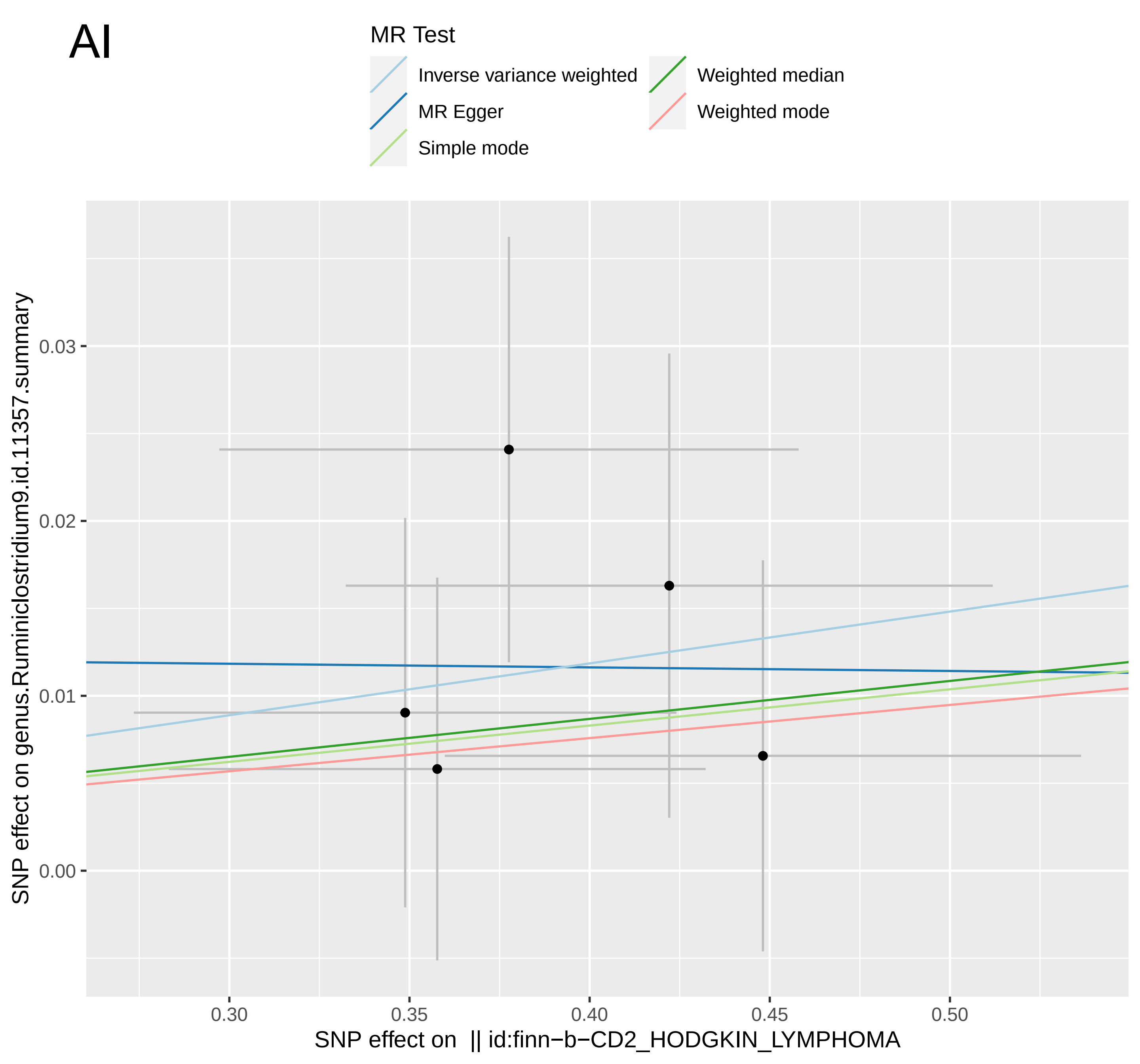
AI
MR Test
Inverse variance weighted
MR Egger
Simple mode
Weighted median
Weighted mode
SNP effect on genus.Ruminiclostridium9.id.11357.summary
0.03
0.02
0.01
0.00
0.30
0.35
0.40
0.45
0.50
SNP effect on || id:finn−b−CD2_HODGKIN_LYMPHOMA

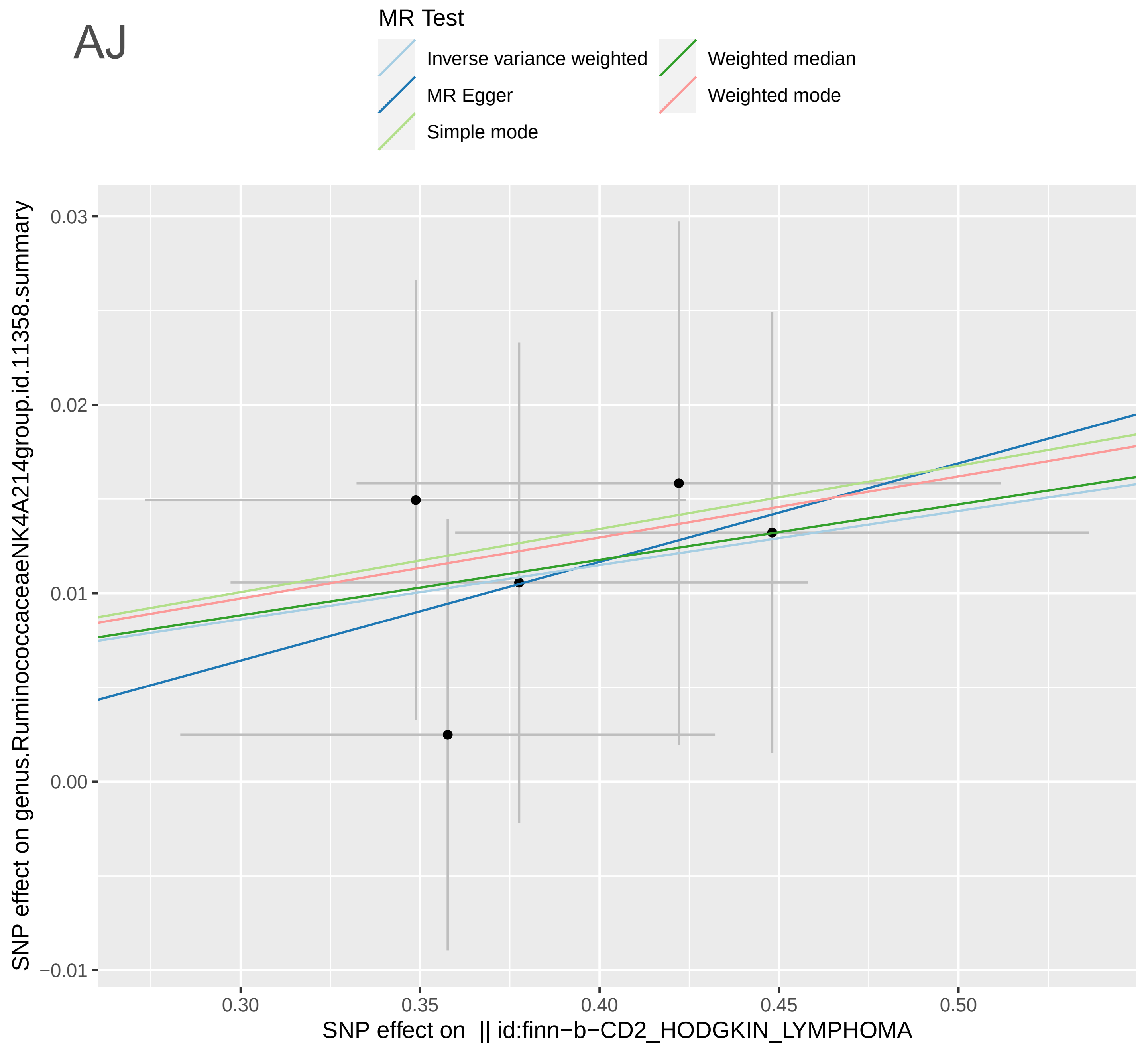

AJ
MR Test
Inverse variance weighted
MR Egger
Simple mode
Weighted median
Weighted mode
SNP effect on genus.RuminococcaceaeNK4A214group.id.11358.summary
0.03
0.02
0.01
0.00
−0.01
0.30
0.35
0.40
0.45
0.50
SNP effect on || id:finn−b−CD2_HODGKIN_LYMPHOMA

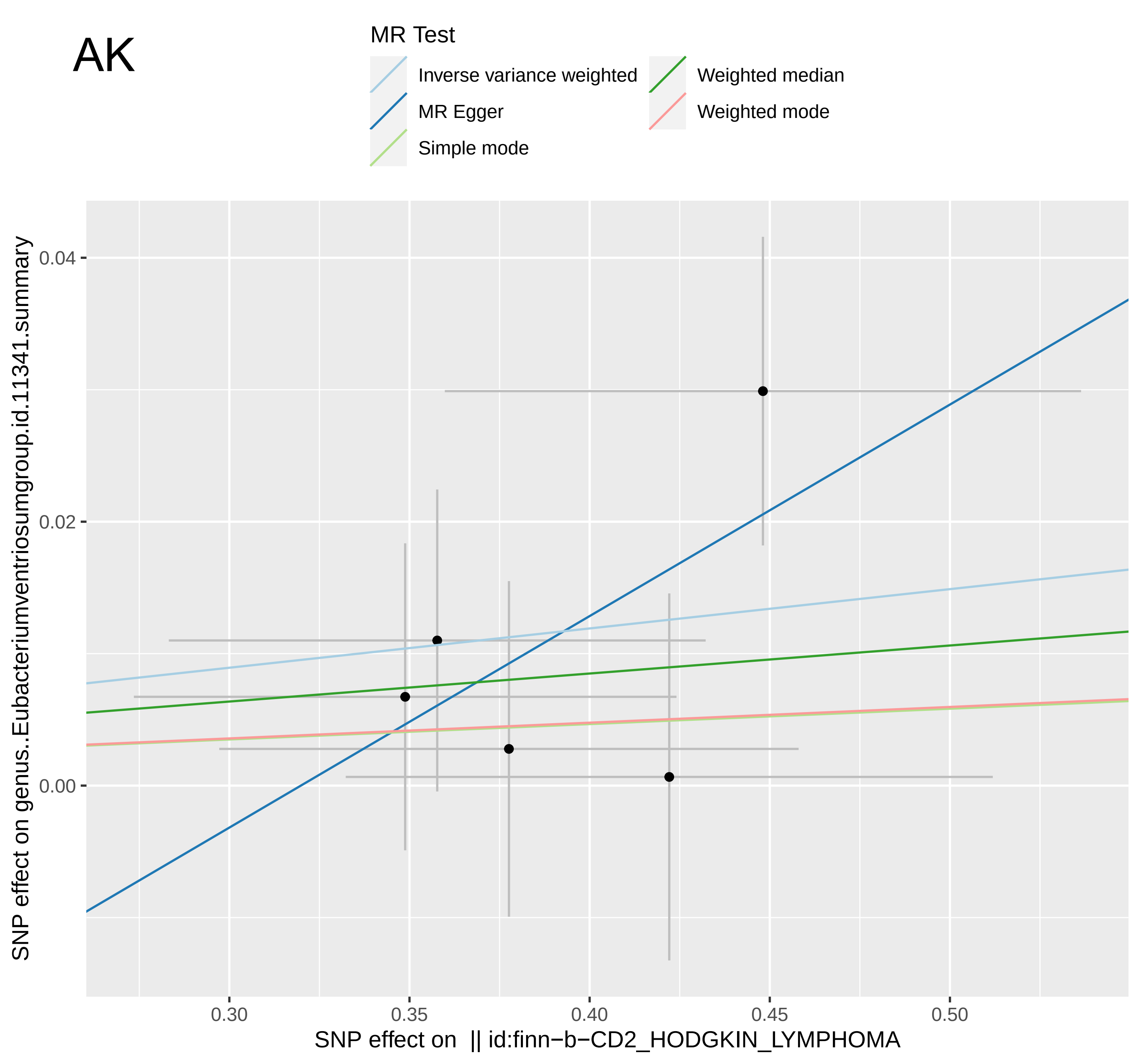

AK
MR Test
Inverse variance weighted
MR Egger
Simple mode
Weighted median
Weighted mode
SNP effect on genus..Eubacteriumventriosumgroup.id.11341.summary
0.04
0.02
0.00
0.30
0.35
0.40
0.45
0.50
SNP effect on || id:finn−b−CD2_HODGKIN_LYMPHOMA

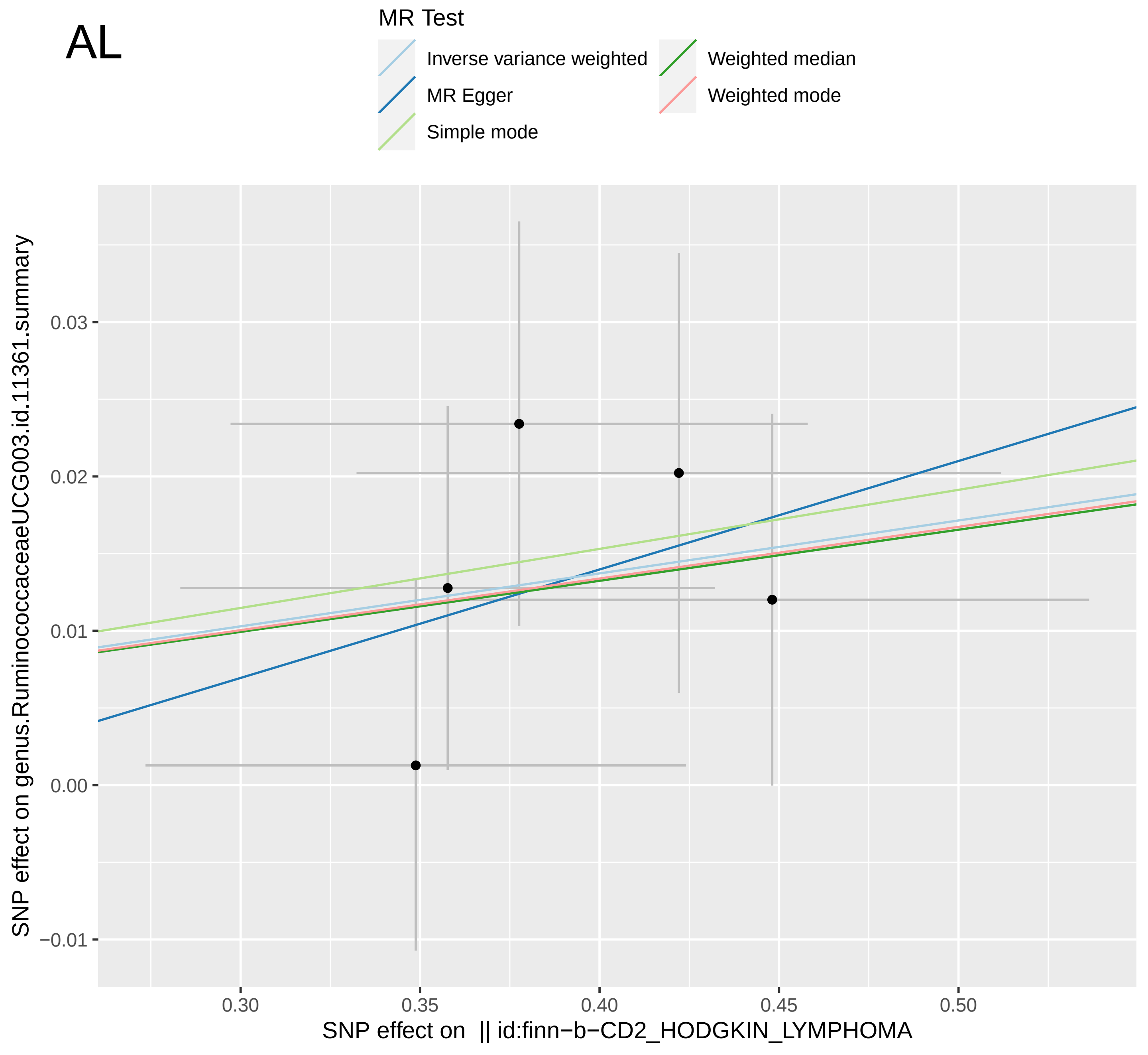
AL
MR Test
Inverse variance weighted
MR Egger
Simple mode
Weighted median
Weighted mode
SNP effect on genus.RuminococcaceaeUCG003.id.11361.summary
0.03
0.02
0.01
0.00
−0.01
0.30
0.35
0.40
0.45
0.50
SNP effect on || id:finn−b−CD2_HODGKIN_LYMPHOMA

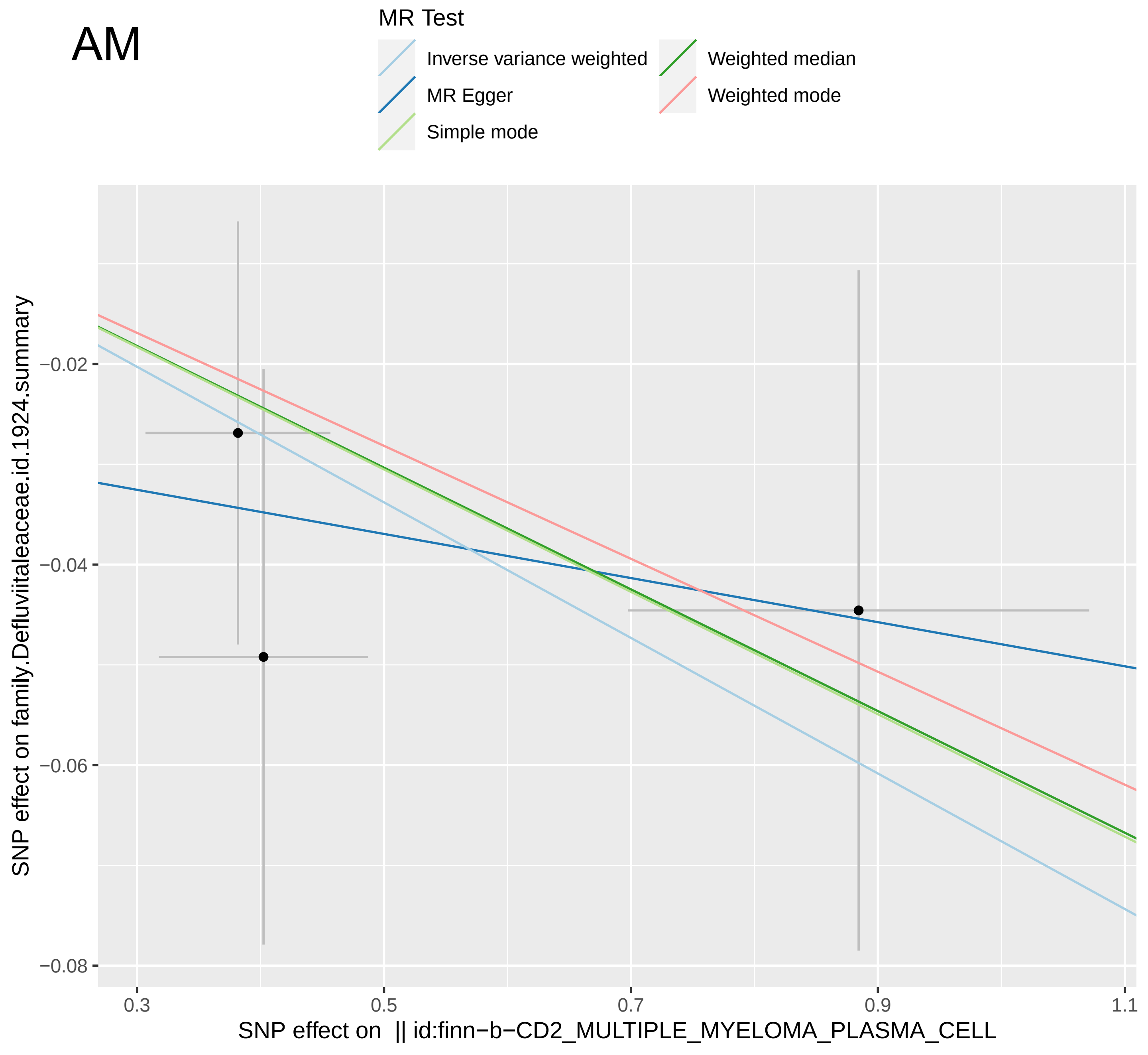

AM
MR Test
Inverse variance weighted
MR Egger
Simple mode
Weighted median
Weighted mode
−0.02
−0.04
−0.06
−0.08
0.3
0.5
0.7
0.9
1.1
SNP effect on family.Defluviitaleaceae.id.1924.summary
SNP effect on || id:finn−b−CD2_MULTIPLE_MYELOMA_PLASMA_CELL

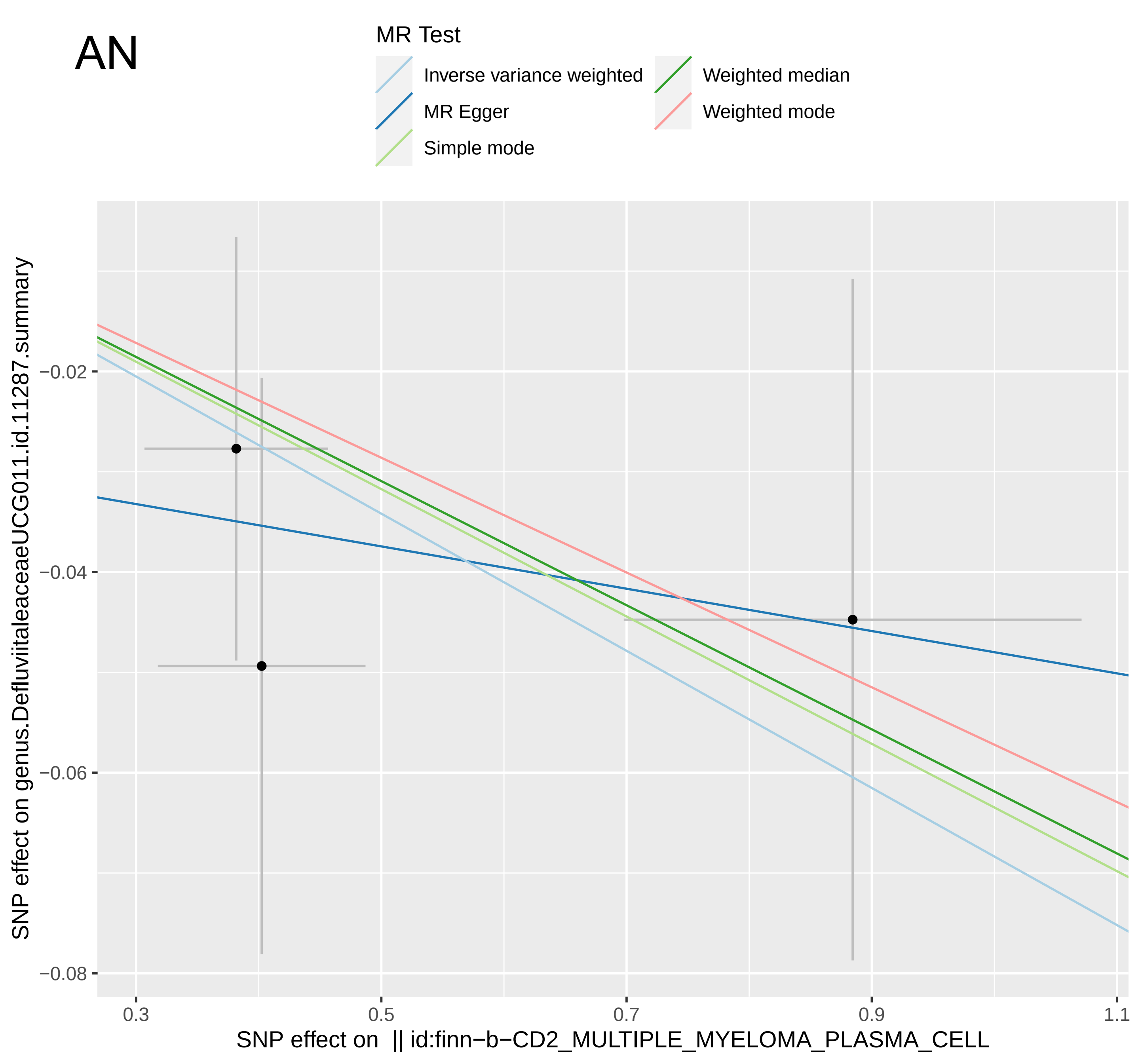
AN
MR Test
Inverse variance weighted
MR Egger
Simple mode
Weighted median
Weighted mode
SNP effect on genus.DefluviitaleaceaeUCG011.id.11287.summary
−0.02
−0.04
−0.06
−0.08
0.3
0.5
0.7
0.9
1.1
SNP effect on || id:finn−b−CD2_MULTIPLE_MYELOMA_PLASMA_CELL

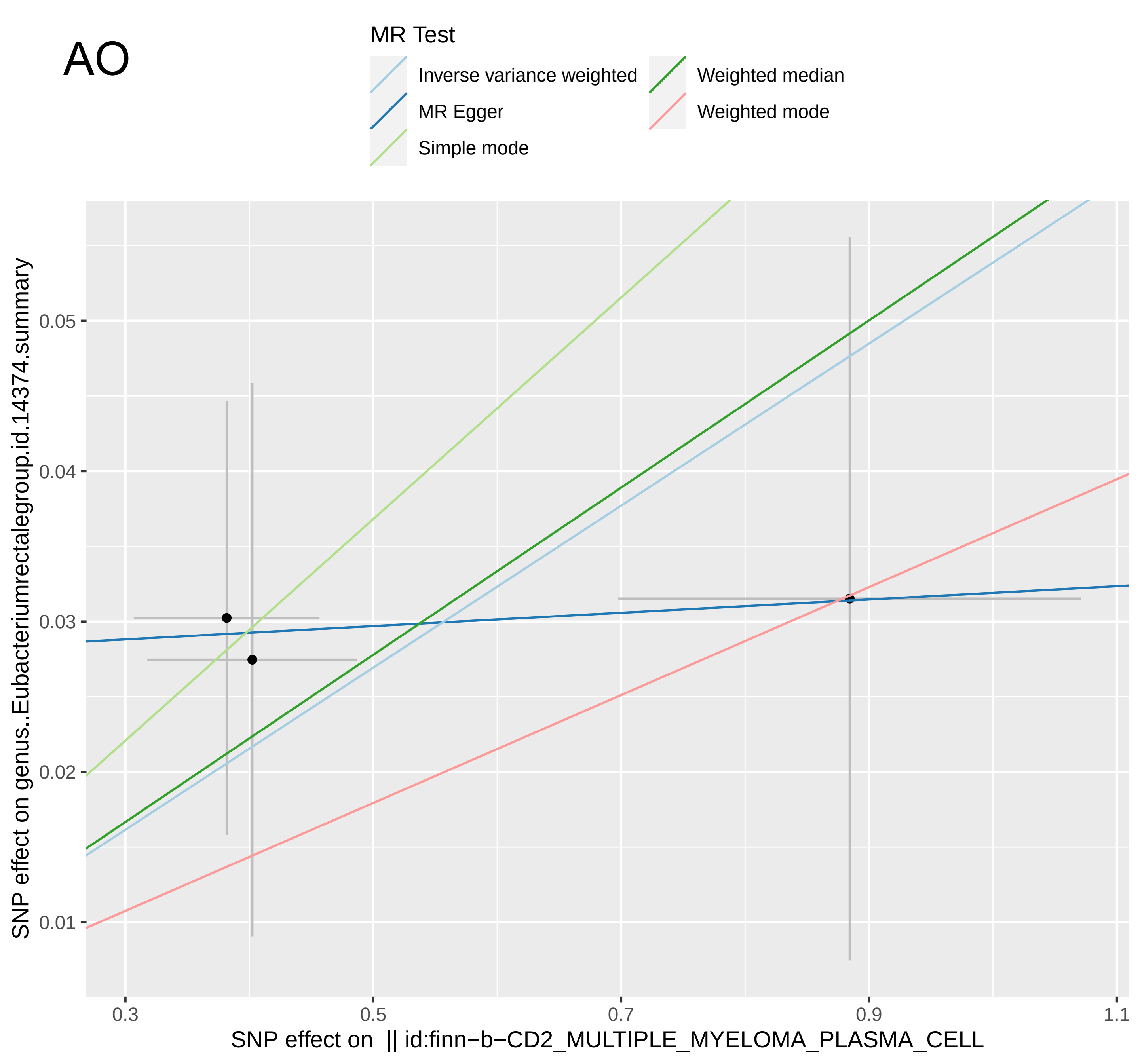
AO
MR Test
Inverse variance weighted
MR Egger
Simple mode
Weighted median
Weighted mode
SNP effect on genus..Eubacteriumrectalegroup.id.14374.summary
0.05
0.04
0.03
0.02
0.01
0.3
0.5
0.7
0.9
1.1
SNP effect on || id:finn−b−CD2_MULTIPLE_MYELOMA_PLASMA_CELL

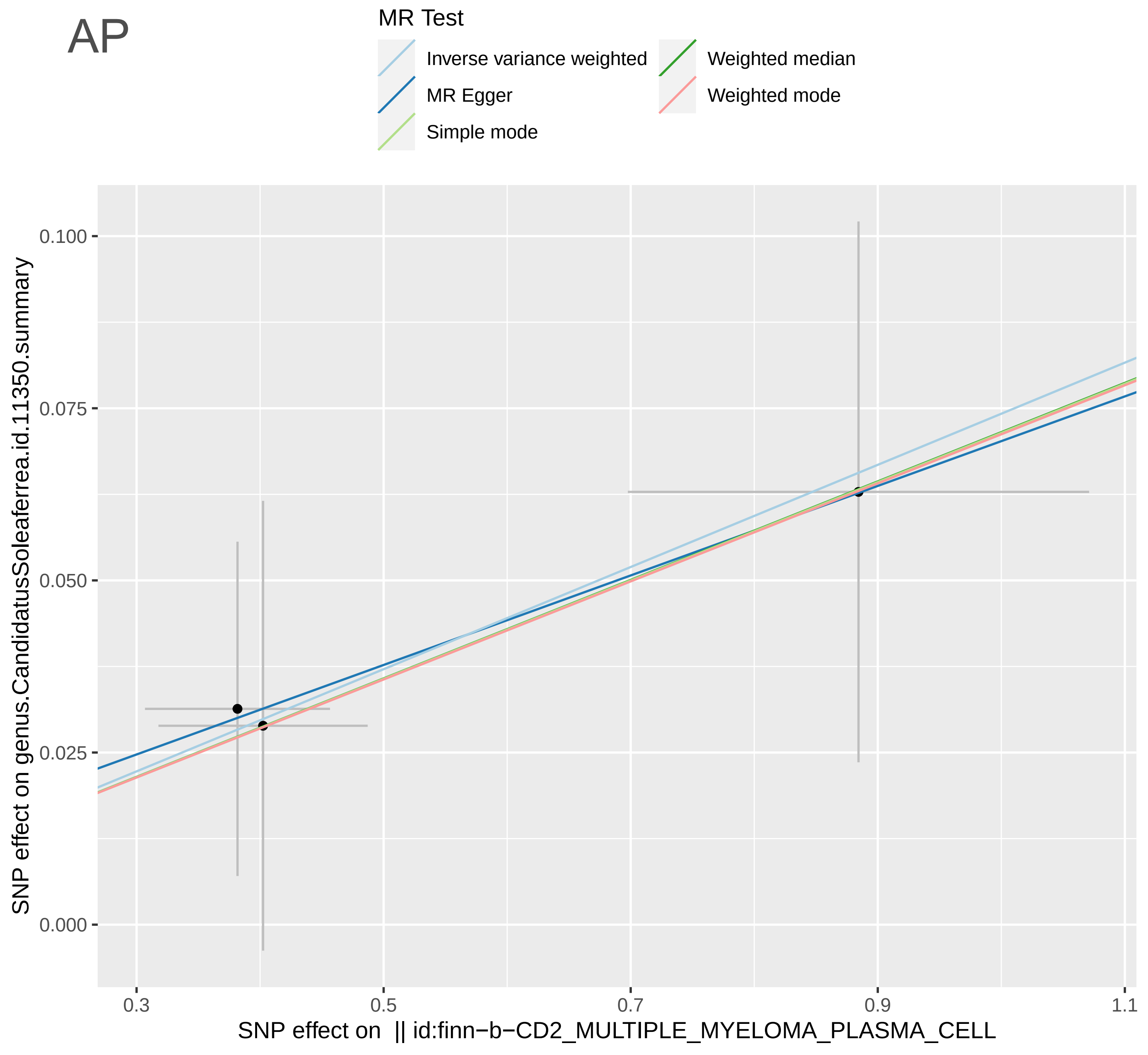
AP
MR Test
Inverse variance weighted
MR Egger
Simple mode
Weighted median
Weighted mode
0.100
0.075
0.050
0.025
0.000
0.3
0.5
0.7
0.9
1.1
SNP effect on genus.CandidatusSoleaferrea.id.11350.summary
SNP effect on || id:finn−b−CD2_MULTIPLE_MYELOMA_PLASMA_CELL

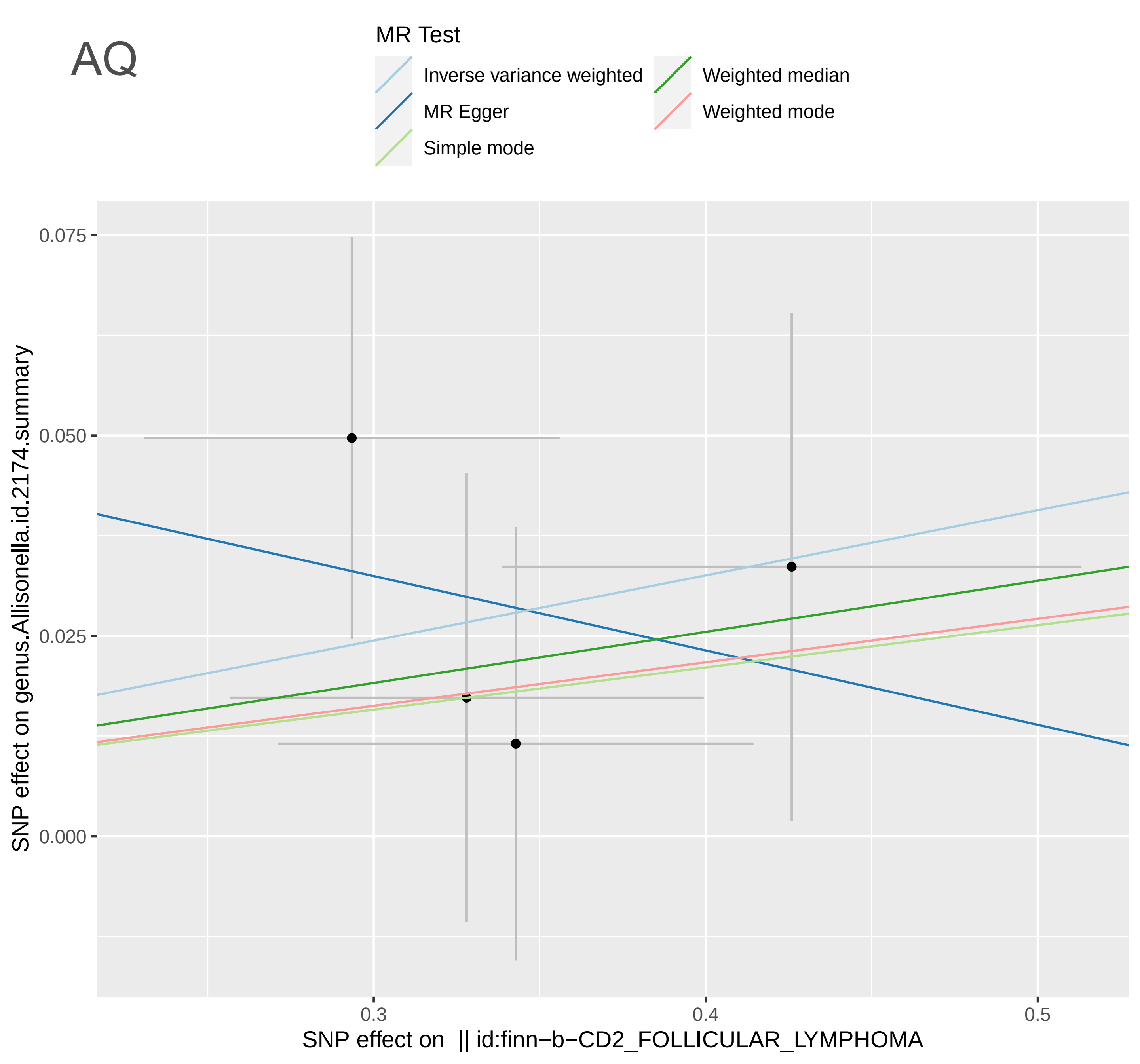

AQ
MR Test
Inverse variance weighted
MR Egger
Simple mode
Weighted median
Weighted mode
0.075
0.050
0.025
0.000
0.3
0.4
0.5
SNP effect on genus.Allisonella.id.2174.summary
SNP effect on || id:finn−b−CD2_FOLLICULAR_LYMPHOMA

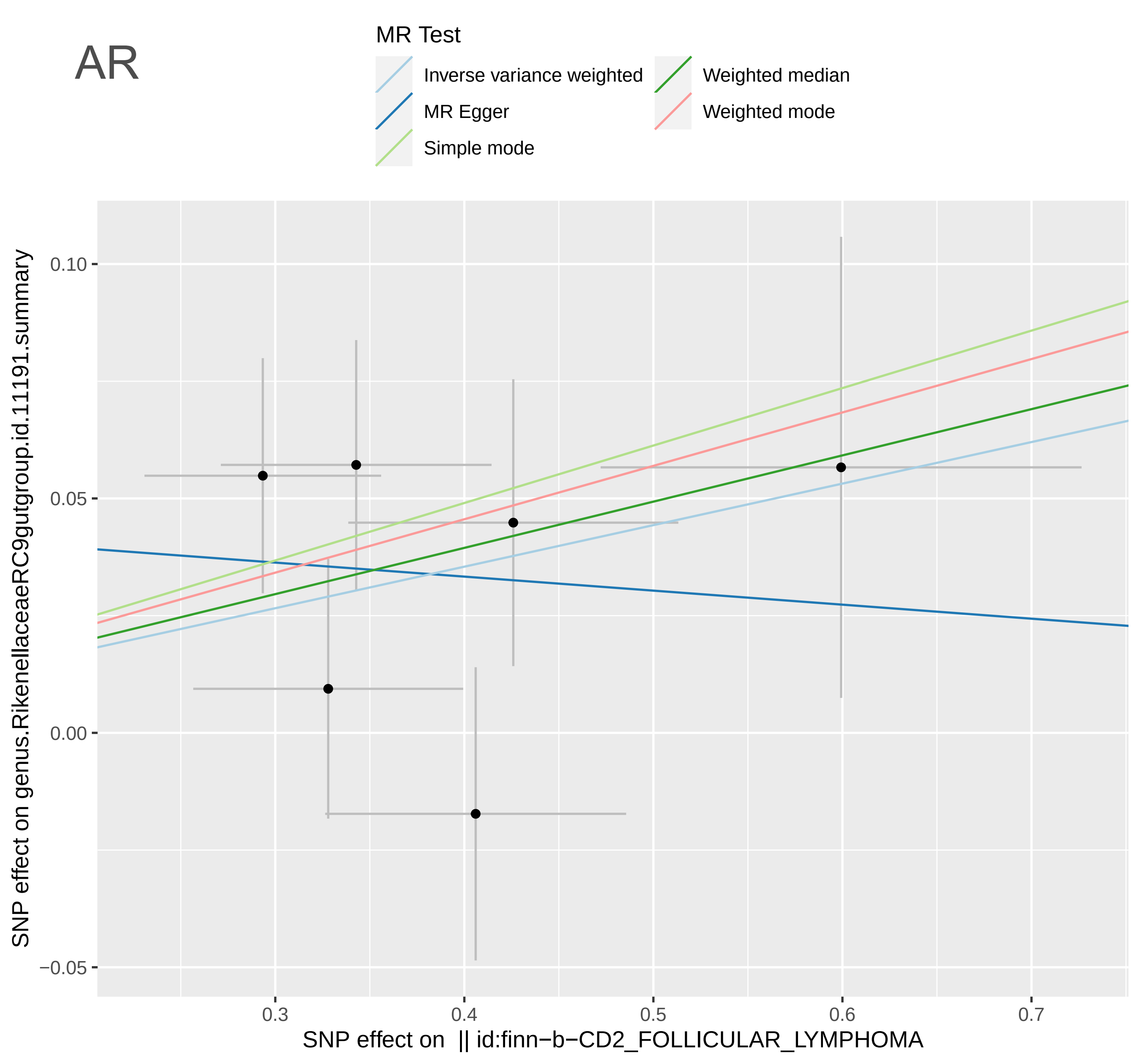
AR
MR Test
Inverse variance weighted
MR Egger
Simple mode
Weighted median
Weighted mode
0.10
0.05
0.00
−0.05
0.3
0.4
0.5
0.6
0.7
SNP effect on genus.RikenellaceaeRC9gutgroup.id.11191.summary
SNP effect on || id:finn−b−CD2_FOLLICULAR_LYMPHOMA

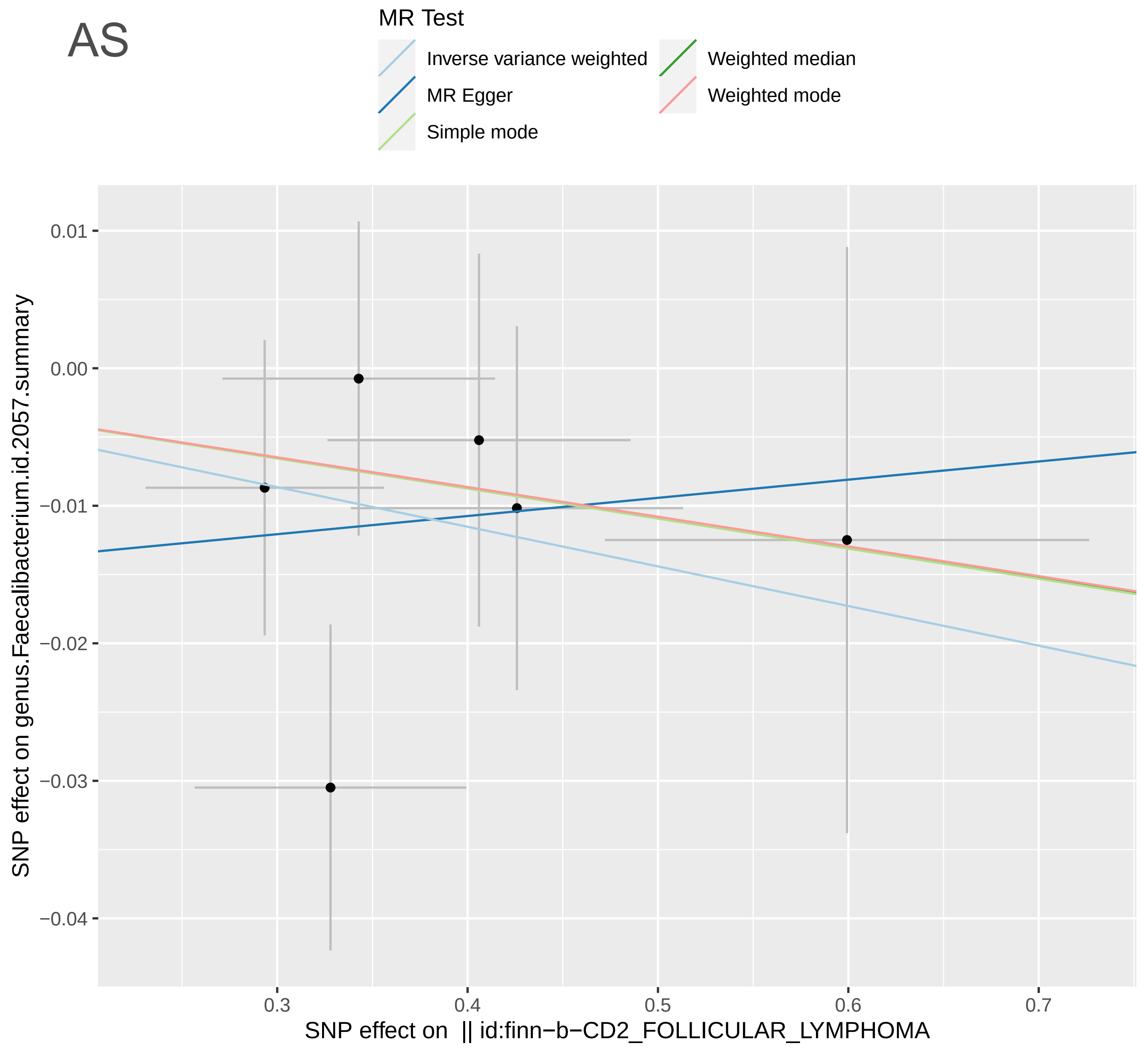
AS
MR Test
Inverse variance weighted
MR Egger
Simple mode
Weighted median
Weighted mode
0.01
0.00
−0.01
−0.02
−0.03
−0.04
SNP effect on genus.Faecalibacterium.id.2057.summary
0.3
0.4
0.5
0.6
0.7
SNP effect on || id:finn−b−CD2_FOLLICULAR_LYMPHOMA

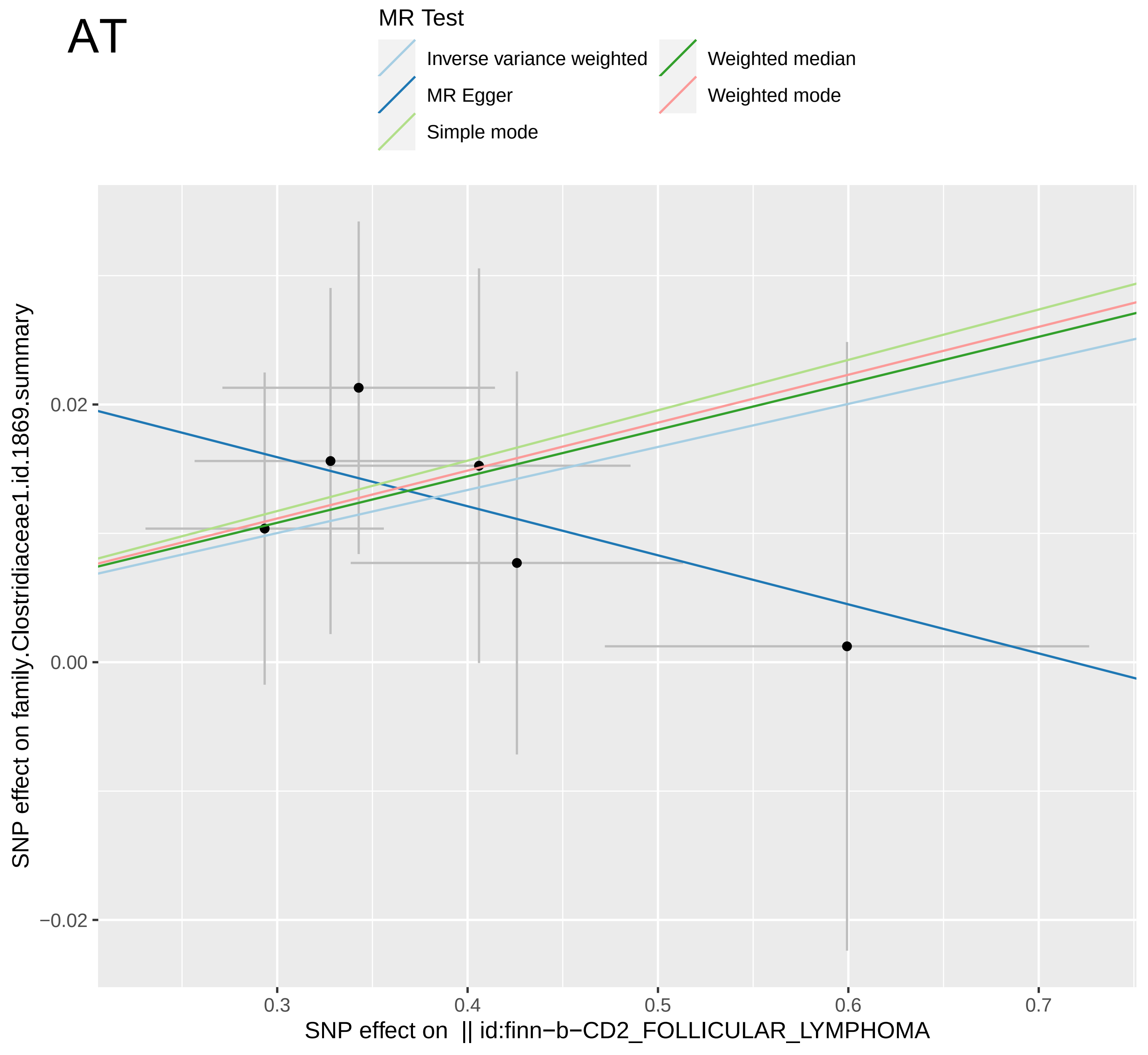
AT
MR Test
Inverse variance weighted
MR Egger
Simple mode
Weighted median
Weighted mode
SNP effect on family.Clostridiaceae1.id.1869.summary
0.02
0.00
−0.02
0.3
0.4
0.5
0.6
0.7
SNP effect on || id:finn−b−CD2_FOLLICULAR_LYMPHOMA

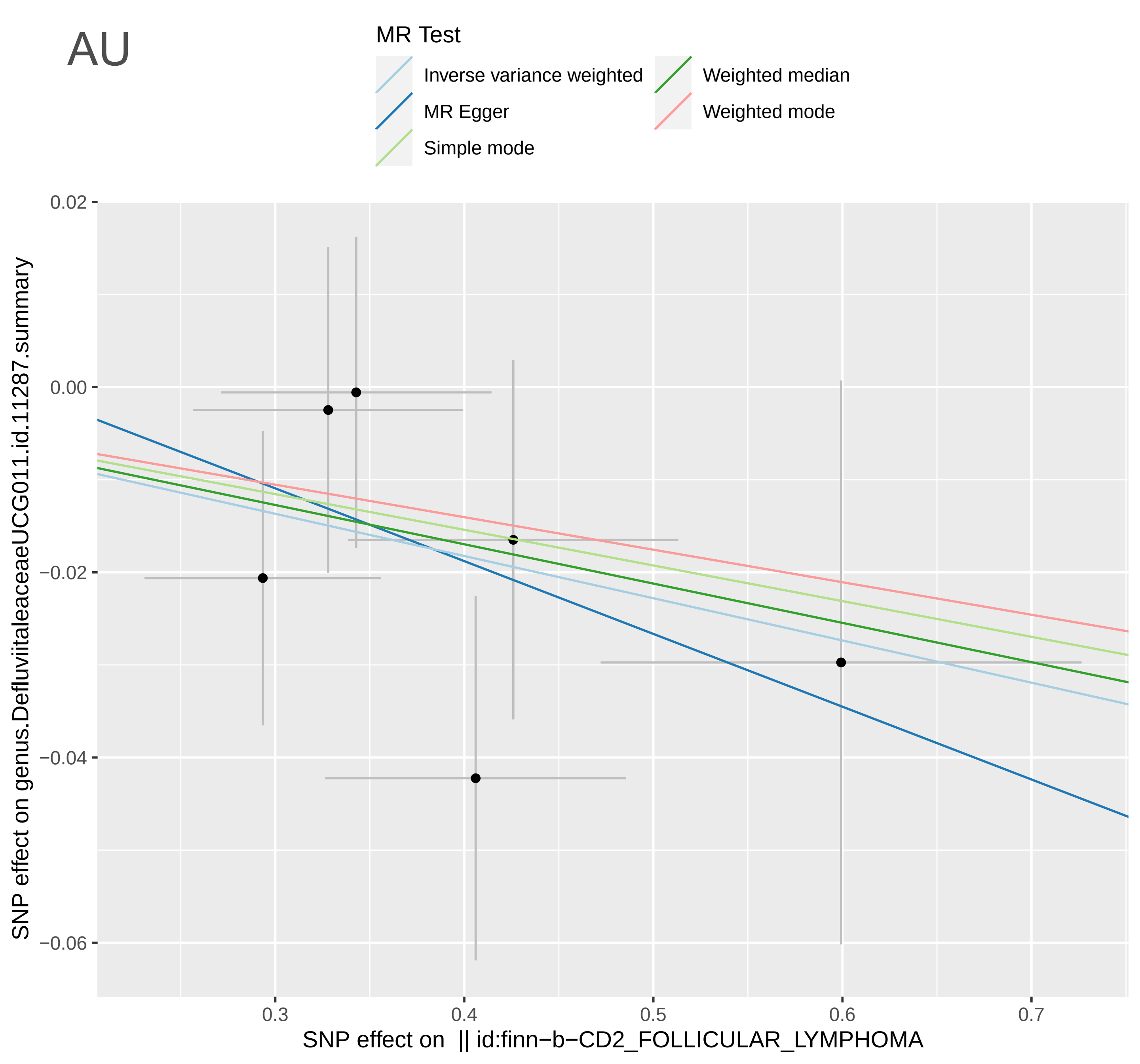
AU
MR Test
Inverse variance weighted
MR Egger
Simple mode
Weighted median
Weighted mode
0.02
0.00
−0.02
−0.04
−0.06
0.3
0.4
0.5
0.6
0.7
SNP effect on genus.DefluviitaleaceaeUCG011.id.11287.summary
SNP effect on || id:finn−b−CD2_FOLLICULAR_LYMPHOMA

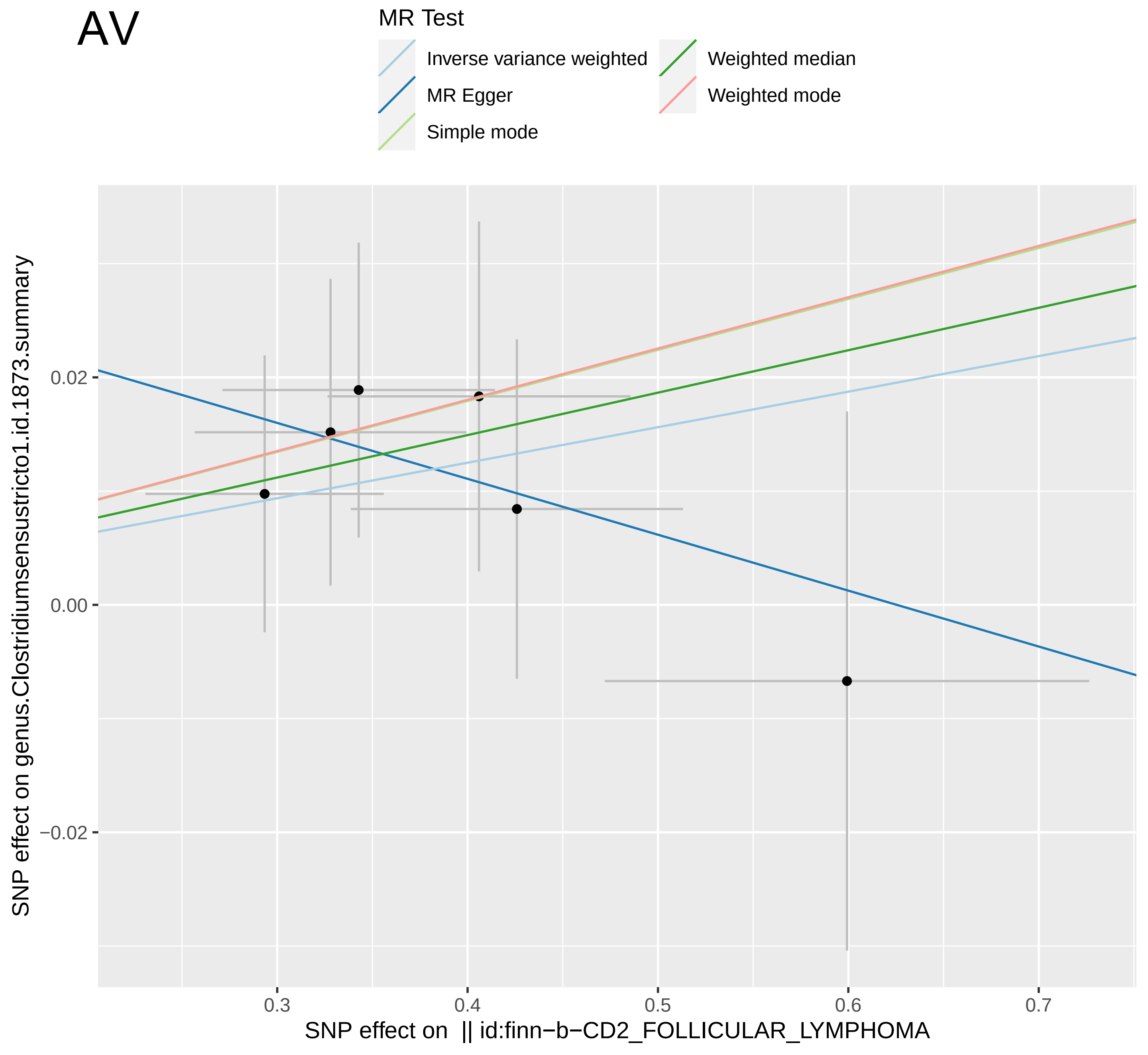

AV
MR Test
Inverse variance weighted
MR Egger
Simple mode
Weighted median
Weighted mode
SNP effect on genus.Clostridiumsensustricto1.id.1873.summary
0.02
0.00
−0.02
0.3
0.4
0.5
0.6
0.7
SNP effect on || id:finn−b−CD2_FOLLICULAR_LYMPHOMA

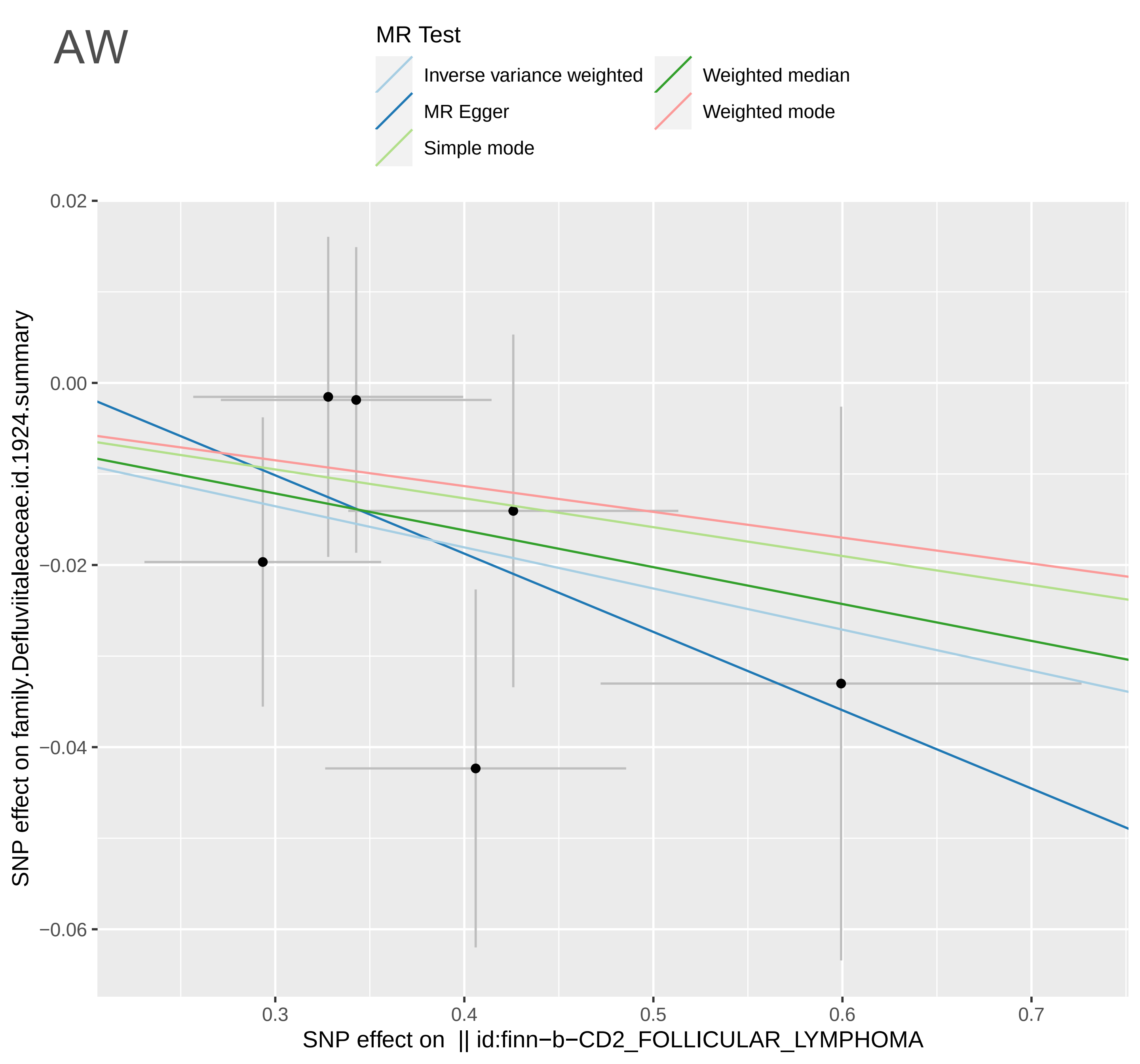
AW
MR Test
Inverse variance weighted
MR Egger
Simple mode
Weighted median
Weighted mode
0.02
0.00
−0.02
−0.04
−0.06
0.3
0.4
0.5
0.6
0.7
SNP effect on family.Defluviitaleaceae.id.1924.summary
SNP effect on || id:finn−b−CD2_FOLLICULAR_LYMPHOMA

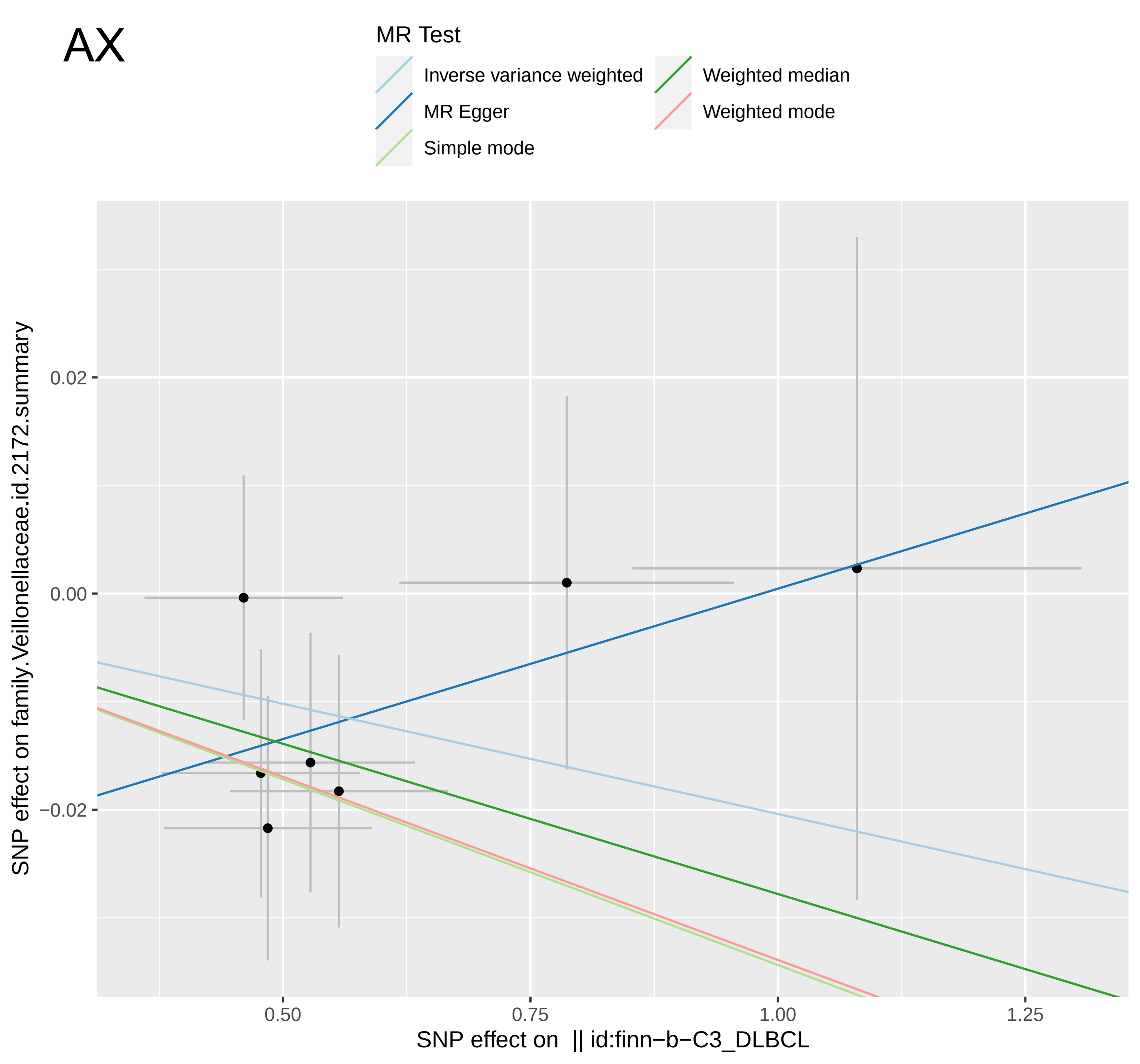

AX
MR Test
Inverse variance weighted
MR Egger
Simple mode
Weighted median
Weighted mode
SNP effect on family.Veillonellaceae.id.2172.summary
0.02
0.00
−0.02
0.50
0.75
1.00
1.25
SNP effect on || id:finn−b−C3_DLBCL

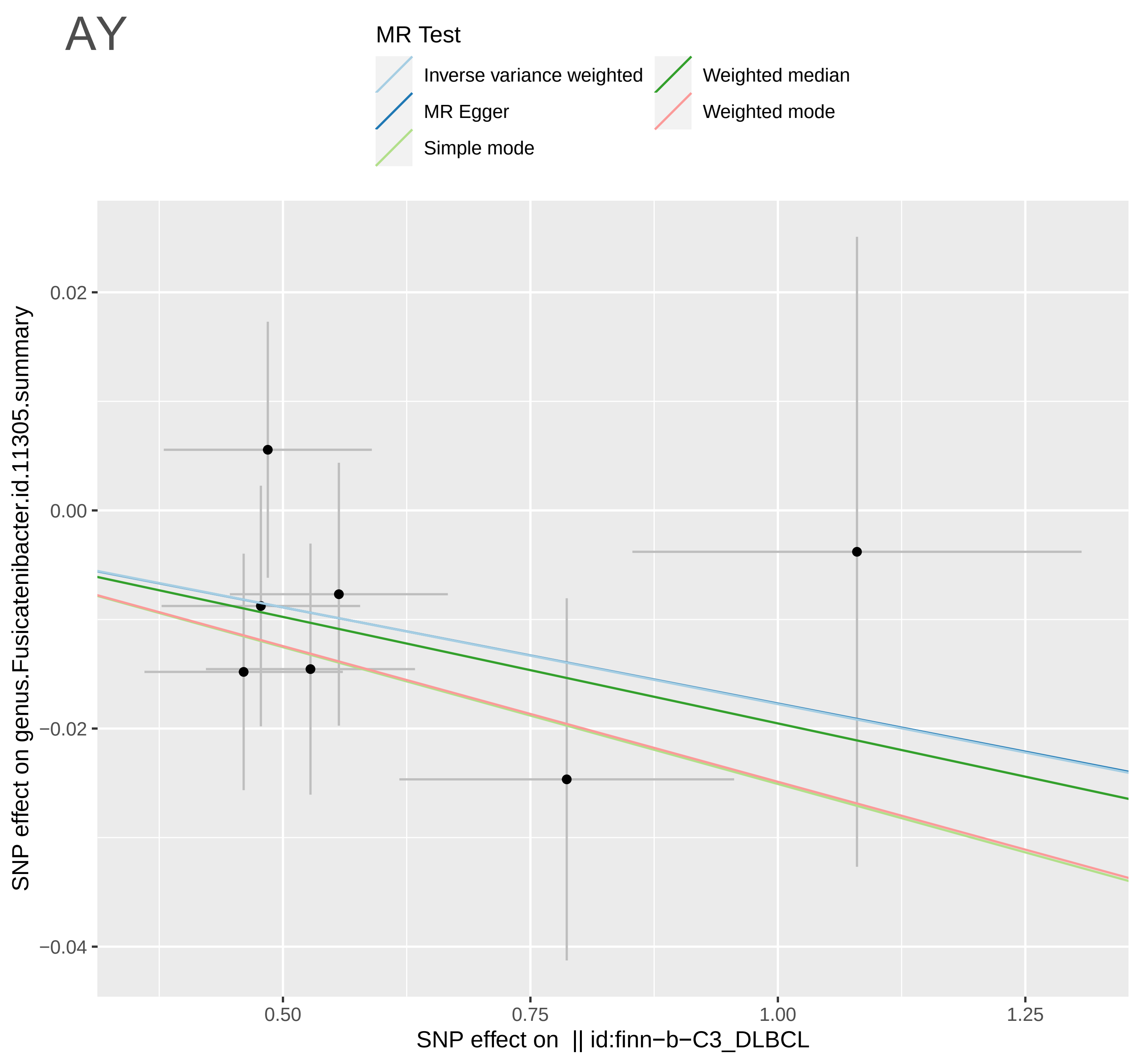

AY
MR Test
Inverse variance weighted
MR Egger
Simple mode
Weighted median
Weighted mode
0.02
0.00
−0.02
−0.04
0.50
0.75
1.00
1.25
SNP effect on genus.Fusicatenibacter.id.11305.summary
SNP effect on || id:finn−b−C3_DLBCL

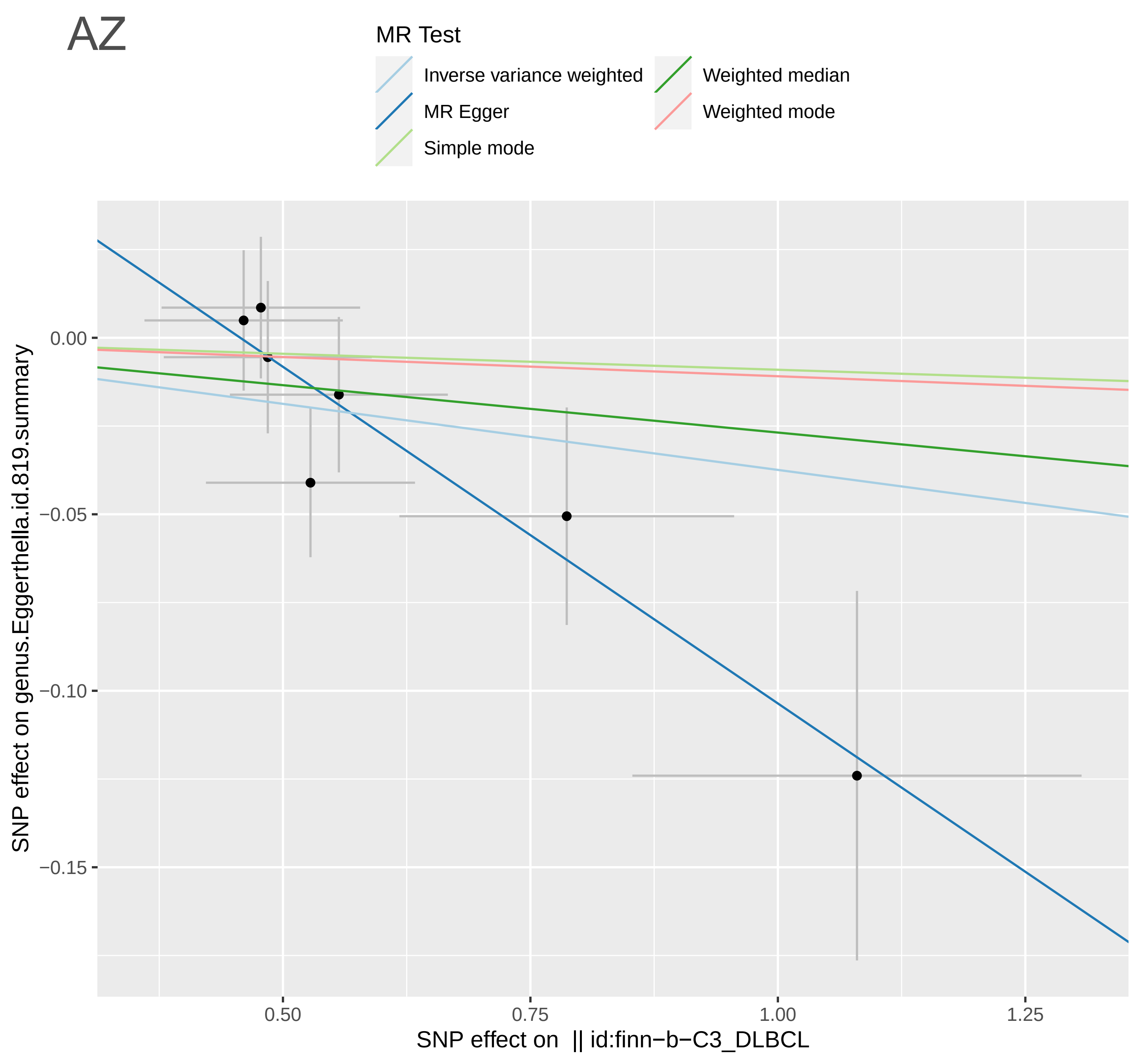

AZ
MR Test
Inverse variance weighted
MR Egger
Simple mode
Weighted median
Weighted mode
0.00
−0.05
−0.10
−0.15
0.50
0.75
1.00
1.25
SNP effect on genus.Eggerthella.id.819.summary
SNP effect on || id:finn−b−C3_DLBCL

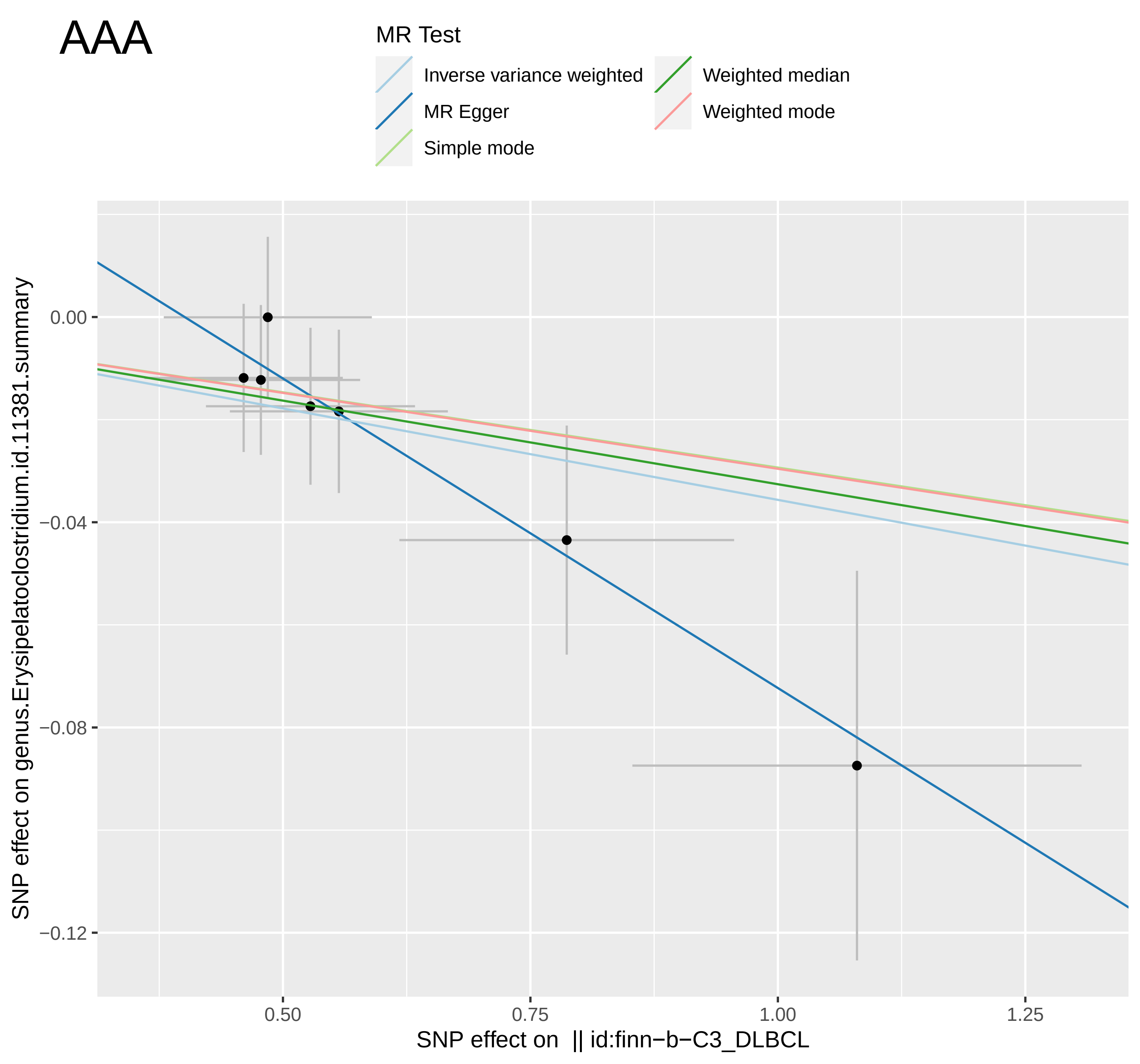
AAA
MR Test
Inverse variance weighted
MR Egger
Simple mode
Weighted median
Weighted mode
SNP effect on genus.Erysipelatoclostridium.id.11381.summary
0.00
−0.04
−0.08
−0.12
0.50
0.75
1.00
1.25
SNP effect on || id:finn−b−C3_DLBCL

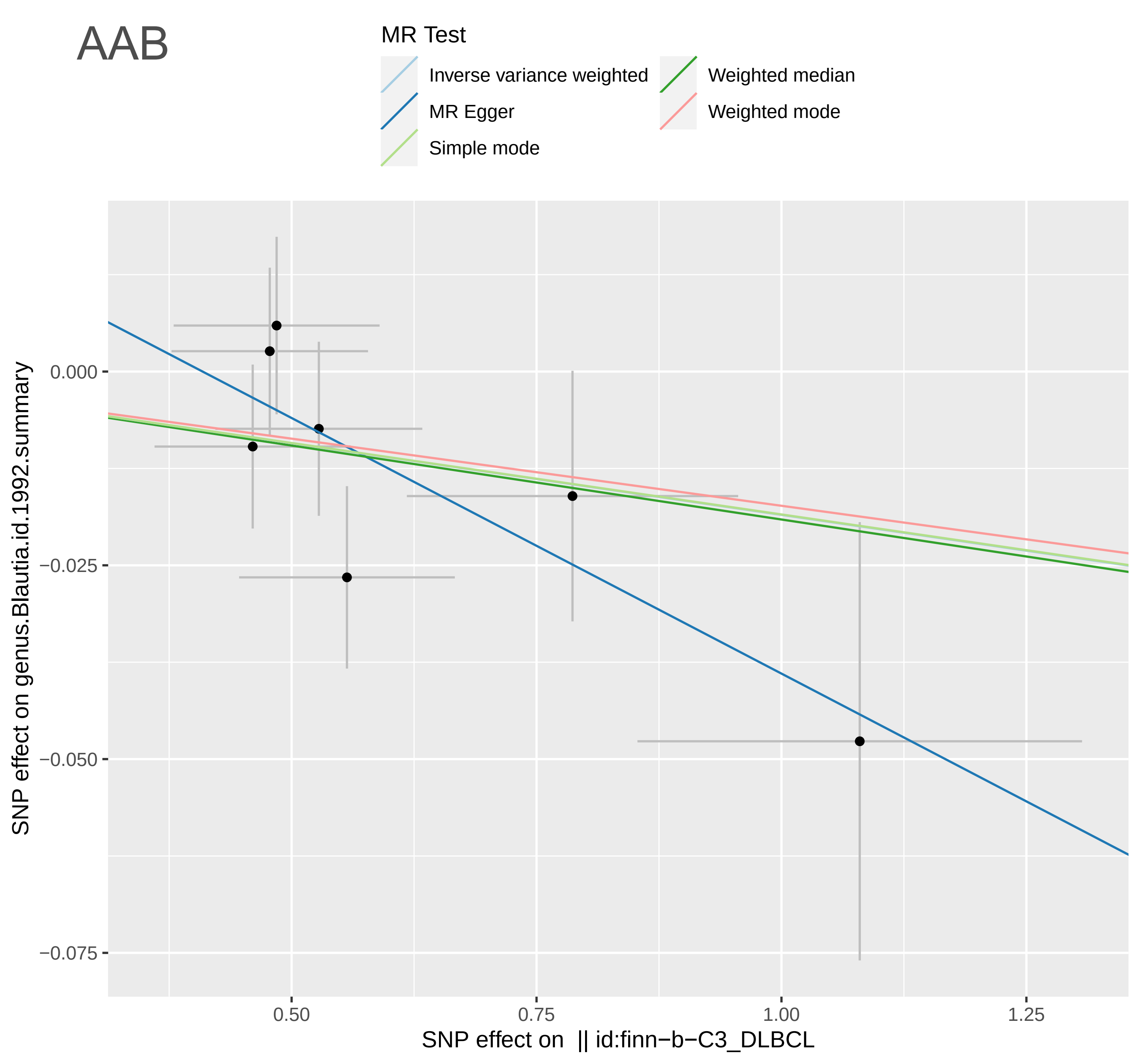

AAB
MR Test
Inverse variance weighted
MR Egger
Simple mode
Weighted median
Weighted mode
0.000
−0.025
−0.050
−0.075
0.50
0.75
1.00
1.25
SNP effect on genus.Blautia.id.1992.summary
SNP effect on || id:finn−b−C3_DLBCL

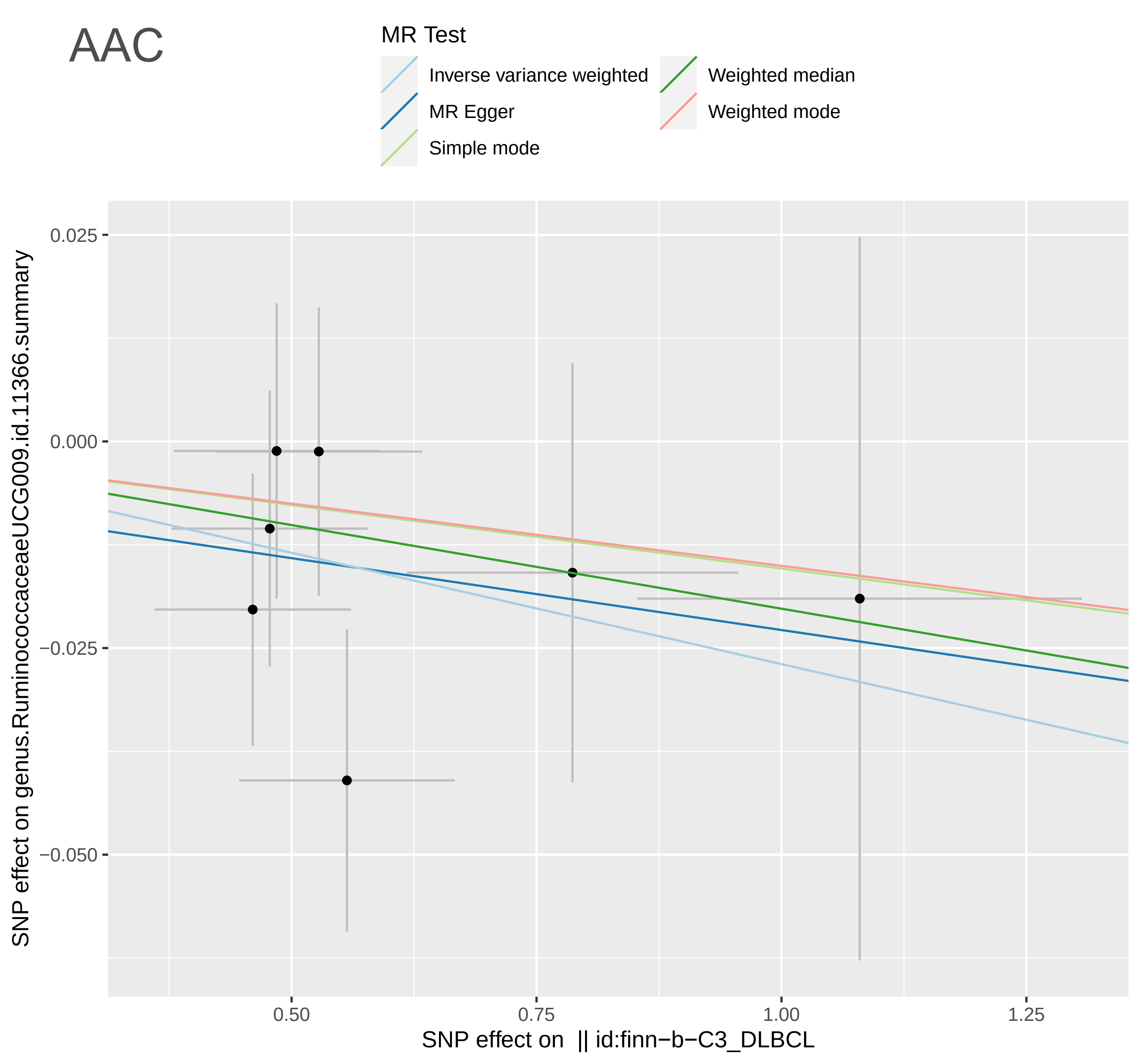
AAC
MR Test
Inverse variance weighted
MR Egger
Simple mode
Weighted median
Weighted mode
0.025
0.000
−0.025
−0.050
0.50
0.75
1.00
1.25
SNP effect on genus.RuminococcaceaeUCG009.id.11366.summary
SNP effect on || id:finn−b−C3_DLBCL

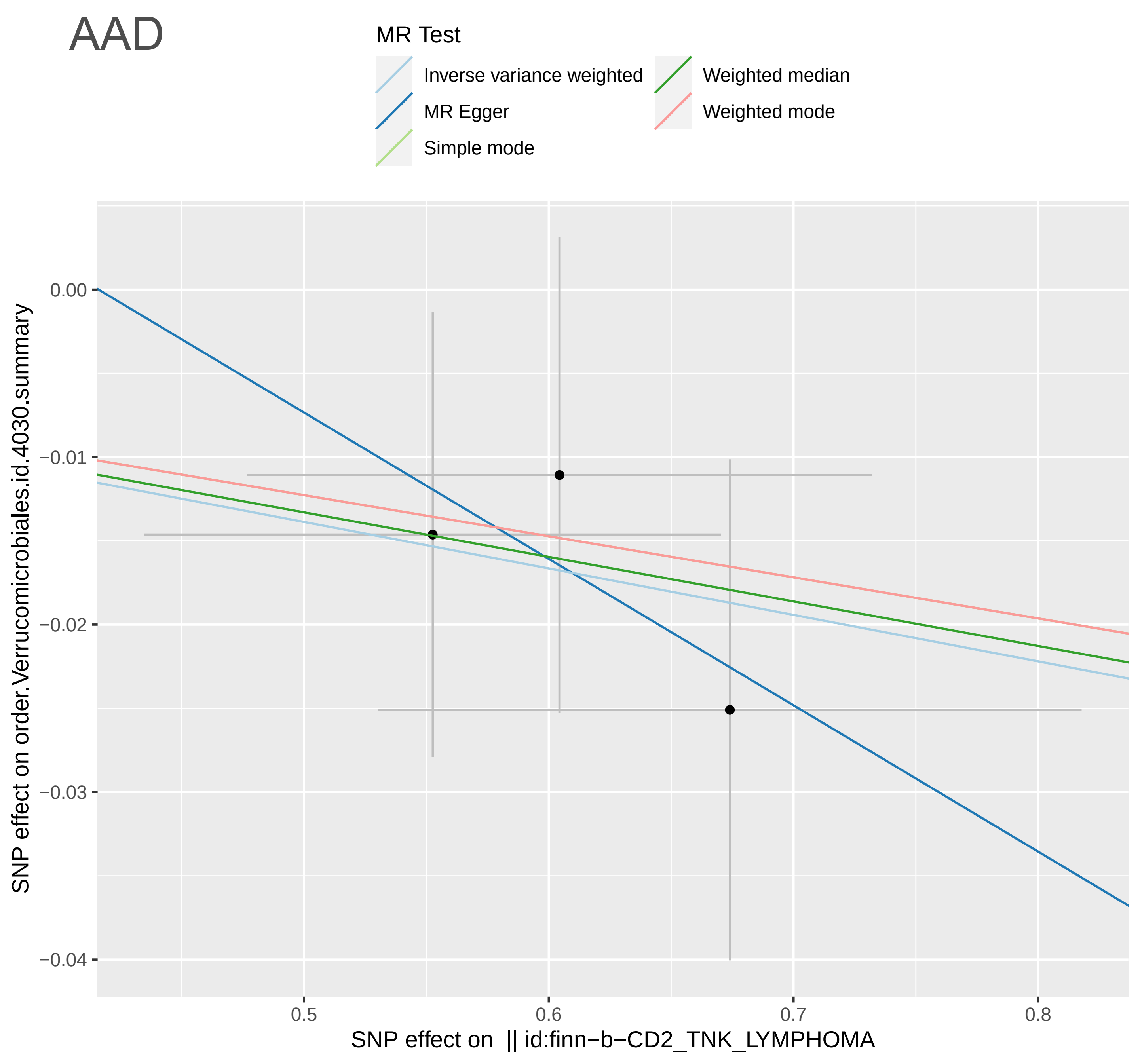
AAD
MR Test
Inverse variance weighted
MR Egger
Simple mode
Weighted median
Weighted mode
0.00
−0.01
−0.02
−0.03
−0.04
SNP effect on order.Verrucomicrobiales.id.4030.summary
0.5
0.6
0.7
0.8
SNP effect on || id:finn−b−CD2_TNK_LYMPHOMA

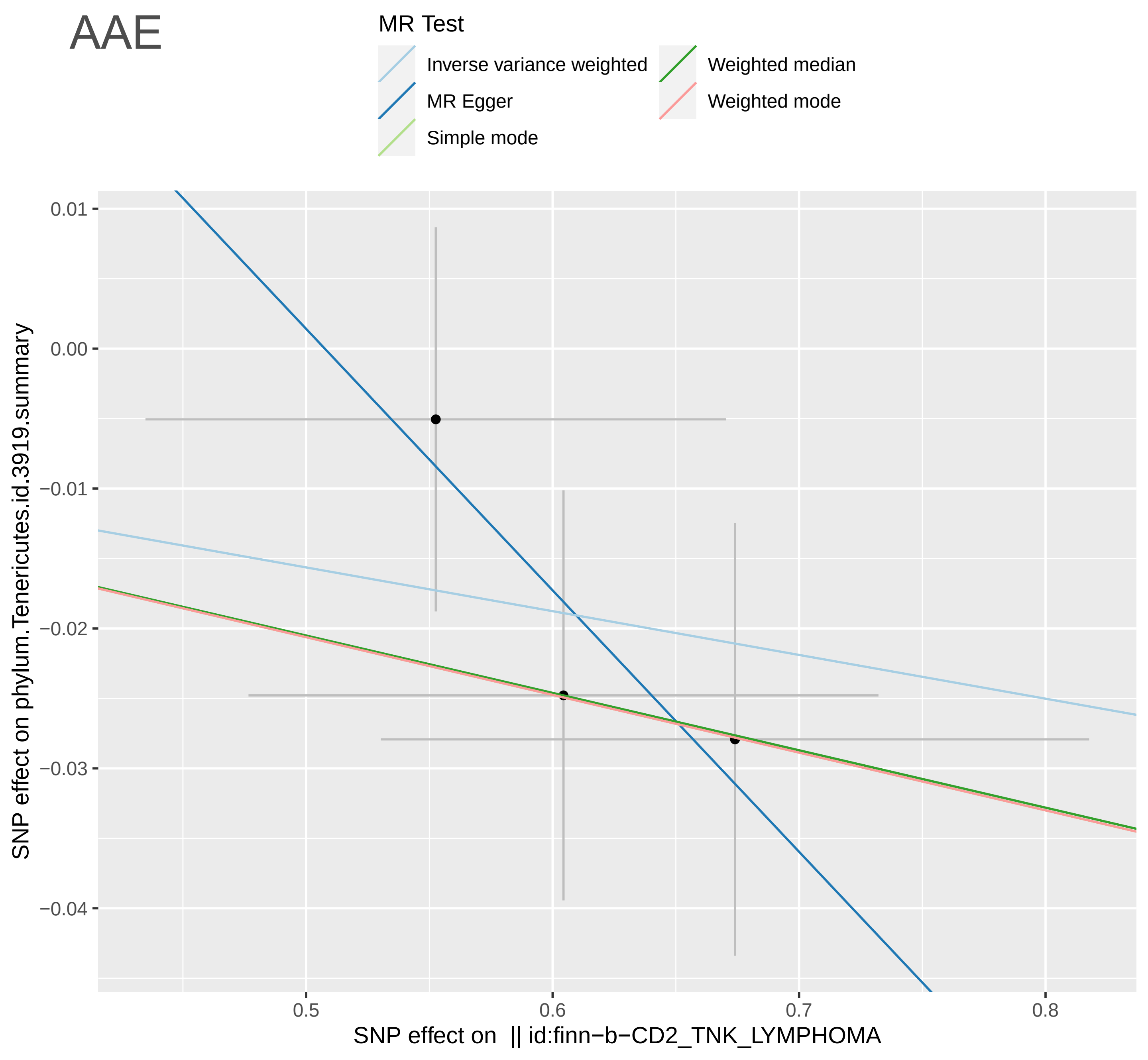
AAE
MR Test
Inverse variance weighted
MR Egger
Simple mode
Weighted median
Weighted mode
0.01
0.00
−0.01
−0.02
−0.03
−0.04
0.5
0.6
0.7
0.8
SNP effect on phylum.Tenericutes.id.3919.summary
SNP effect on || id:finn−b−CD2_TNK_LYMPHOMA

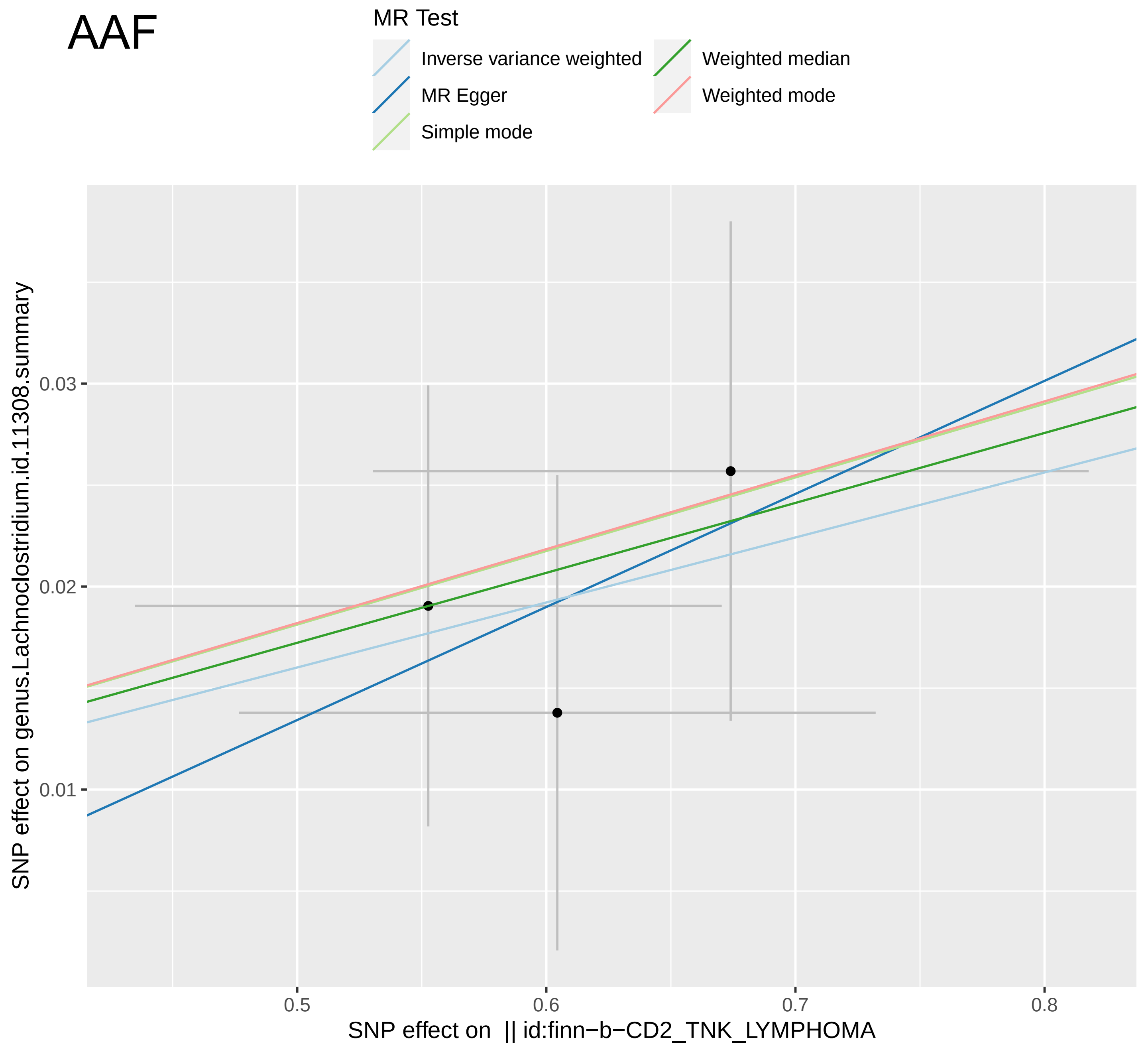

AAF
MR Test
Inverse variance weighted
MR Egger
Simple mode
Weighted median
Weighted mode
0.03
0.02
0.01
0.5
0.6
0.7
0.8
SNP effect on genus.Lachnoclostridium.id.11308.summary
SNP effect on || id:finn−b−CD2_TNK_LYMPHOMA

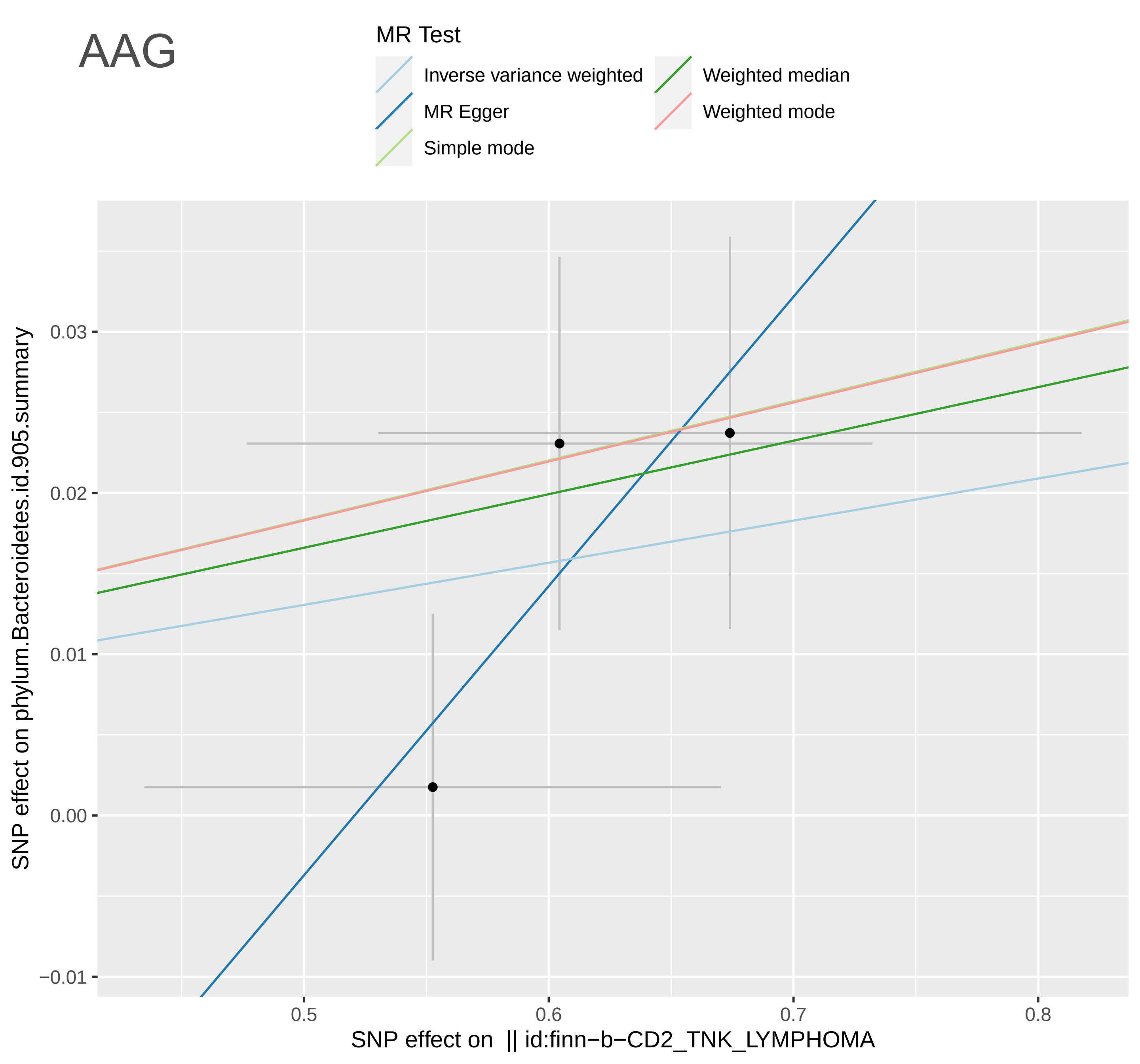

AAG
MR Test
Inverse variance weighted
MR Egger
Simple mode
Weighted median
Weighted mode
0.03
0.02
0.01
0.00
−0.01
0.5
0.6
0.7
0.8
SNP effect on phylum.Bacteroidetes.id.905.summary
SNP effect on || id:finn−b−CD2_TNK_LYMPHOMA

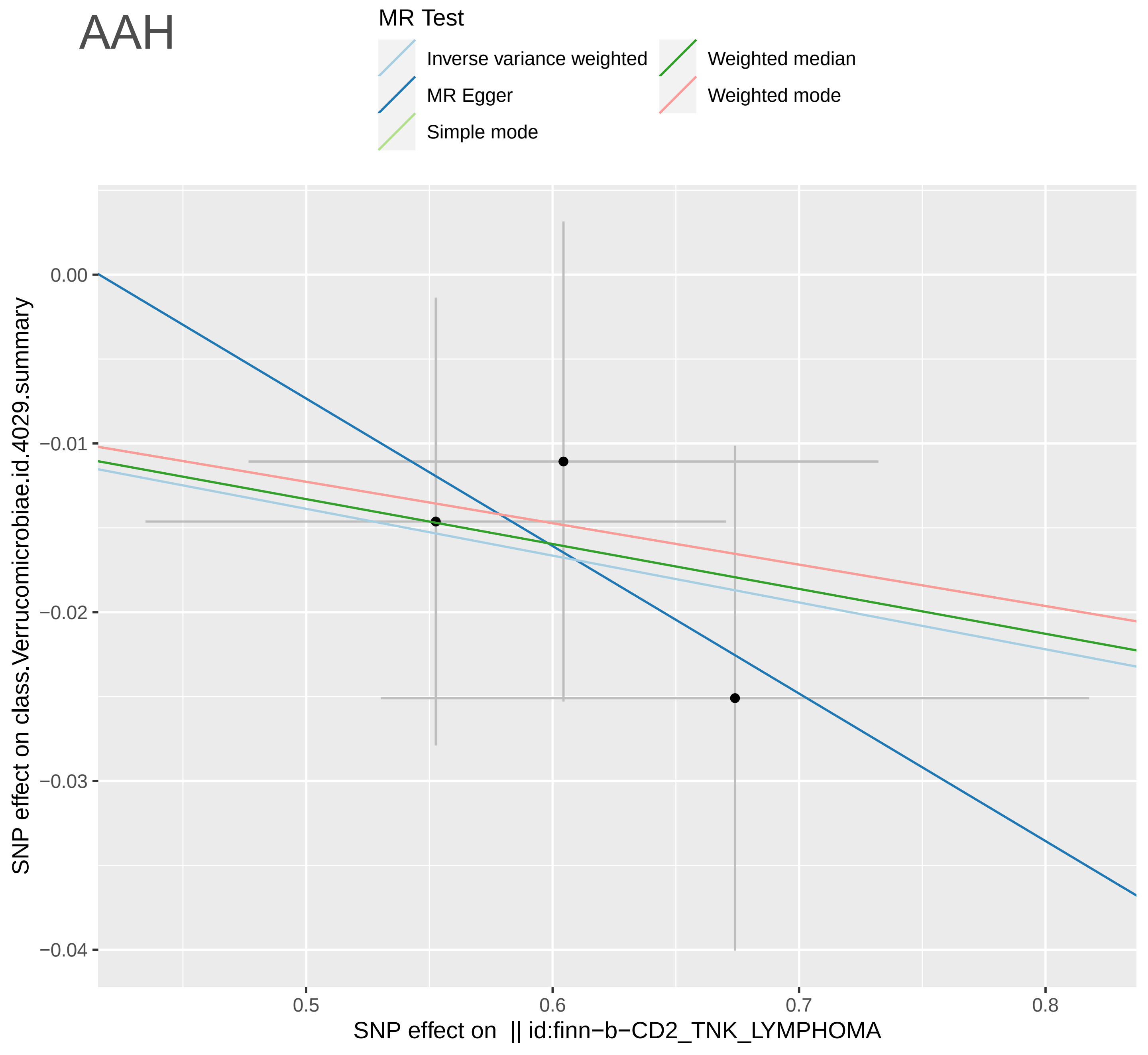

AAH
MR Test
Inverse variance weighted
MR Egger
Simple mode
Weighted median
Weighted mode
0.00
−0.01
−0.02
−0.03
−0.04
0.5
0.6
0.7
0.8
SNP effect on class.Verrucomicrobiae.id.4029.summary
SNP effect on || id:finn−b−CD2_TNK_LYMPHOMA

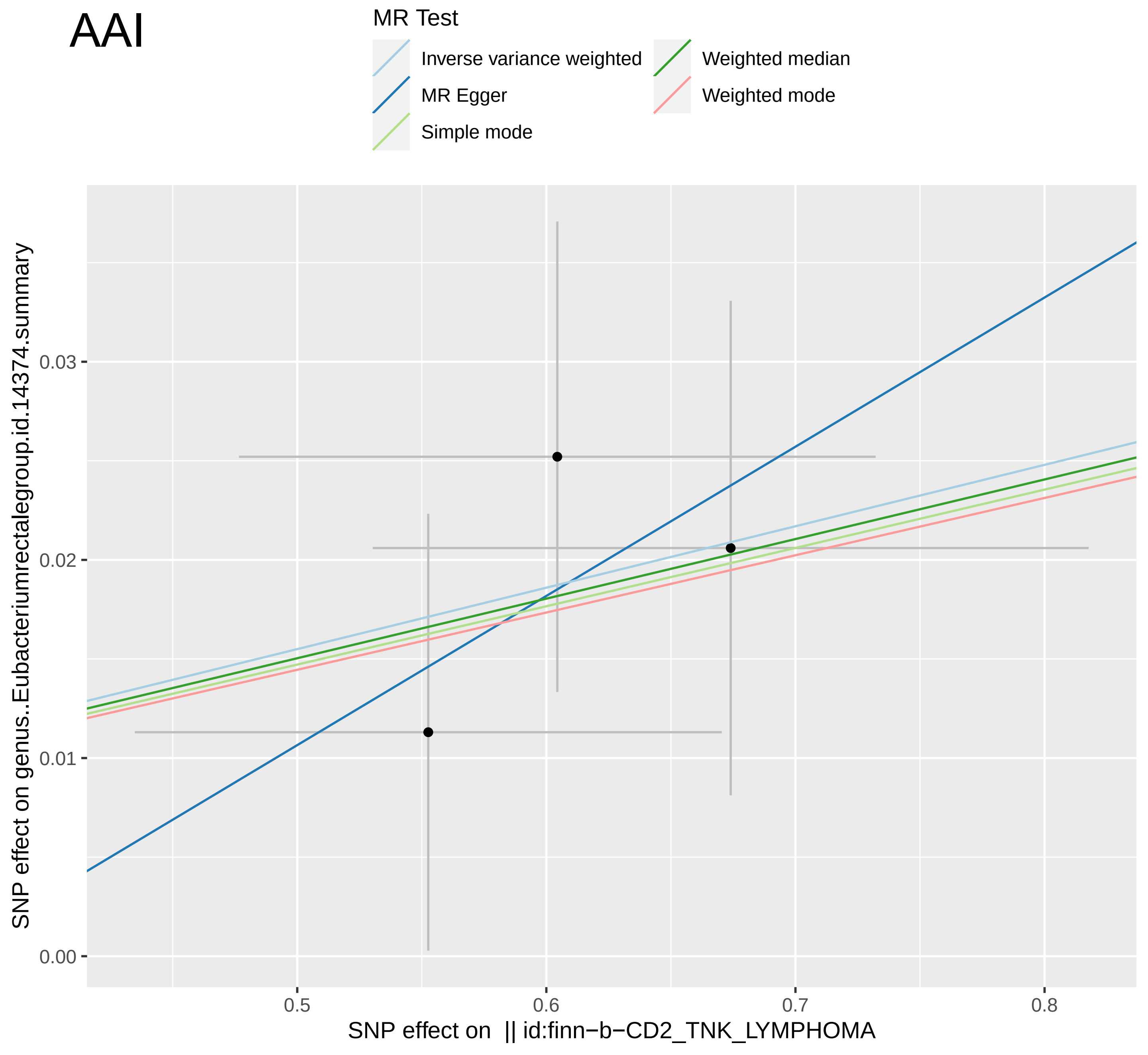

AAI
MR Test
Inverse variance weighted
MR Egger
Simple mode
Weighted median
Weighted mode
0.00
0.01
0.02
0.03
0.5
0.6
0.7
0.8
SNP effect on genus..Eubacteriumrectalegroup.id.14374.summary
SNP effect on || id:finn−b−CD2_TNK_LYMPHOMA

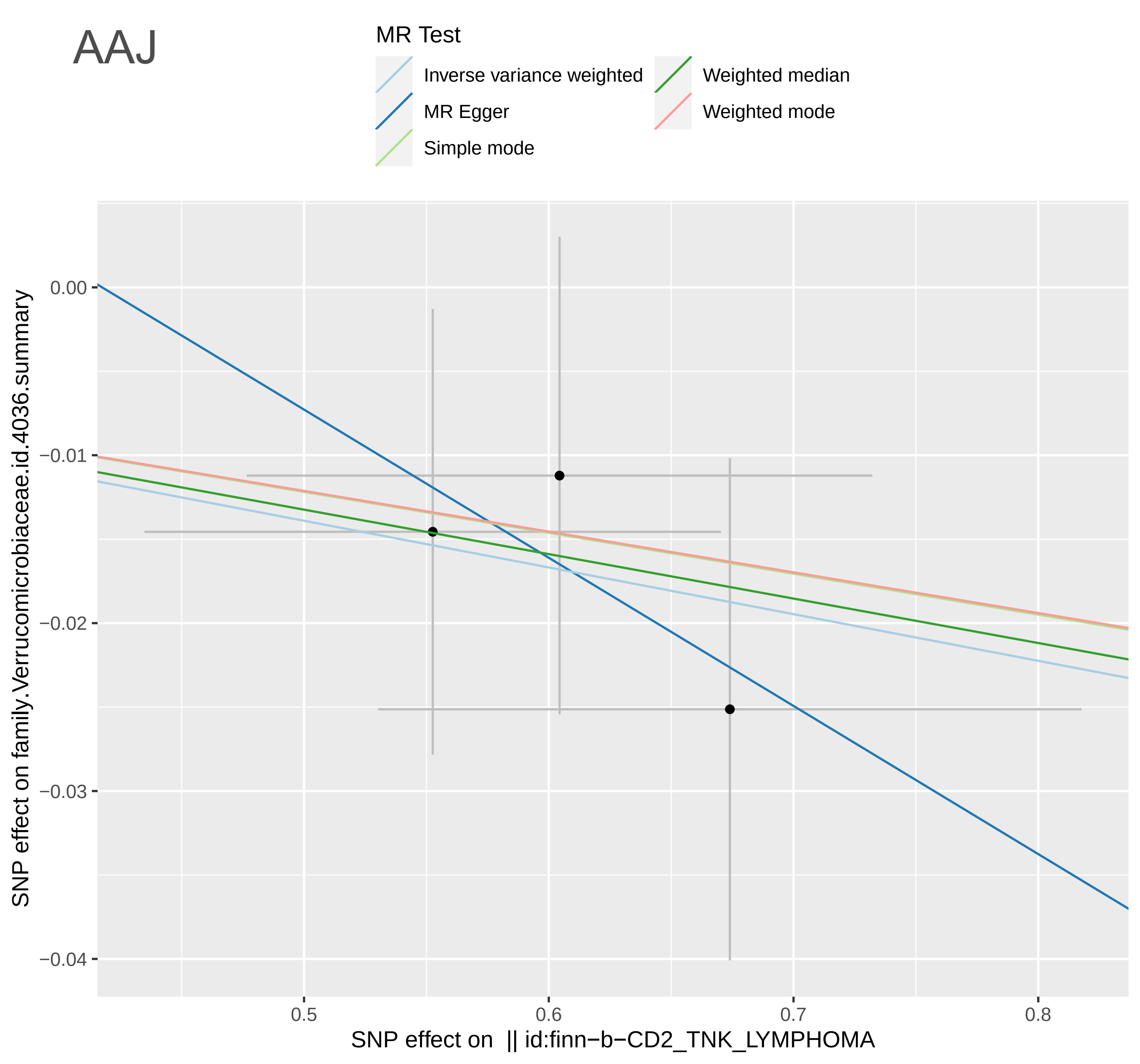

AAJ
MR Test
Inverse variance weighted
MR Egger
Simple mode
Weighted median
Weighted mode
0.00
−0.01
−0.02
−0.03
−0.04
0.5
0.6
0.7
0.8
SNP effect on family.Verrucomicrobiaceae.id.4036.summary
SNP effect on || id:finn−b−CD2_TNK_LYMPHOMA

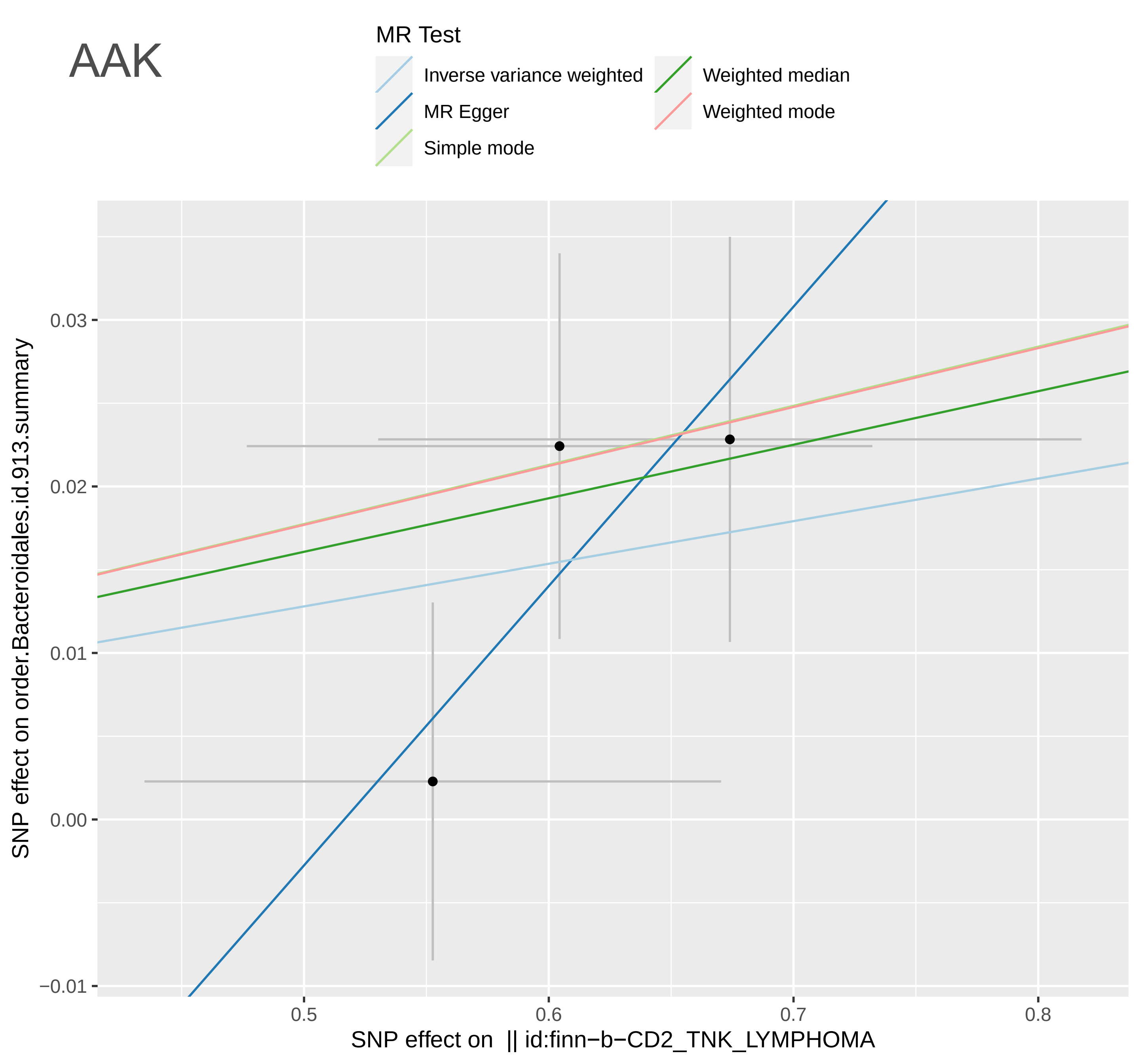

AAK
MR Test
Inverse variance weighted
MR Egger
Simple mode
Weighted median
Weighted mode
0.03
0.02
0.01
0.00
−0.01
0.5
0.6
0.7
0.8
SNP effect on order.Bacteroidales.id.913.summary
SNP effect on || id:finn−b−CD2_TNK_LYMPHOMA

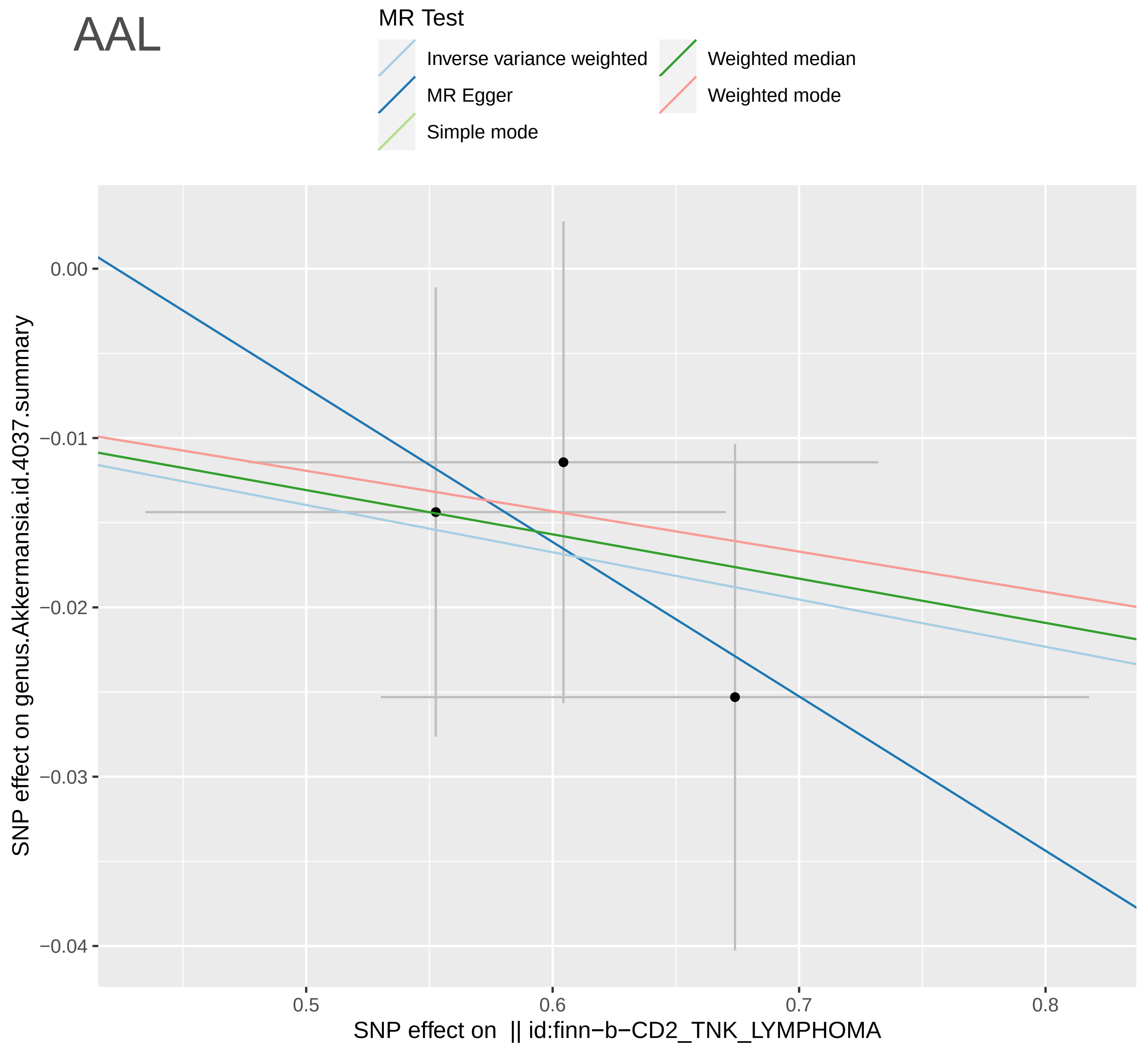

AAL
MR Test
Inverse variance weighted
MR Egger
Simple mode
Weighted median
Weighted mode
0.00
−0.01
−0.02
−0.03
−0.04
SNP effect on genus.Akkermansia.id.4037.summary
0.5
0.6
0.7
0.8
SNP effect on || id:finn−b−CD2_TNK_LYMPHOMA

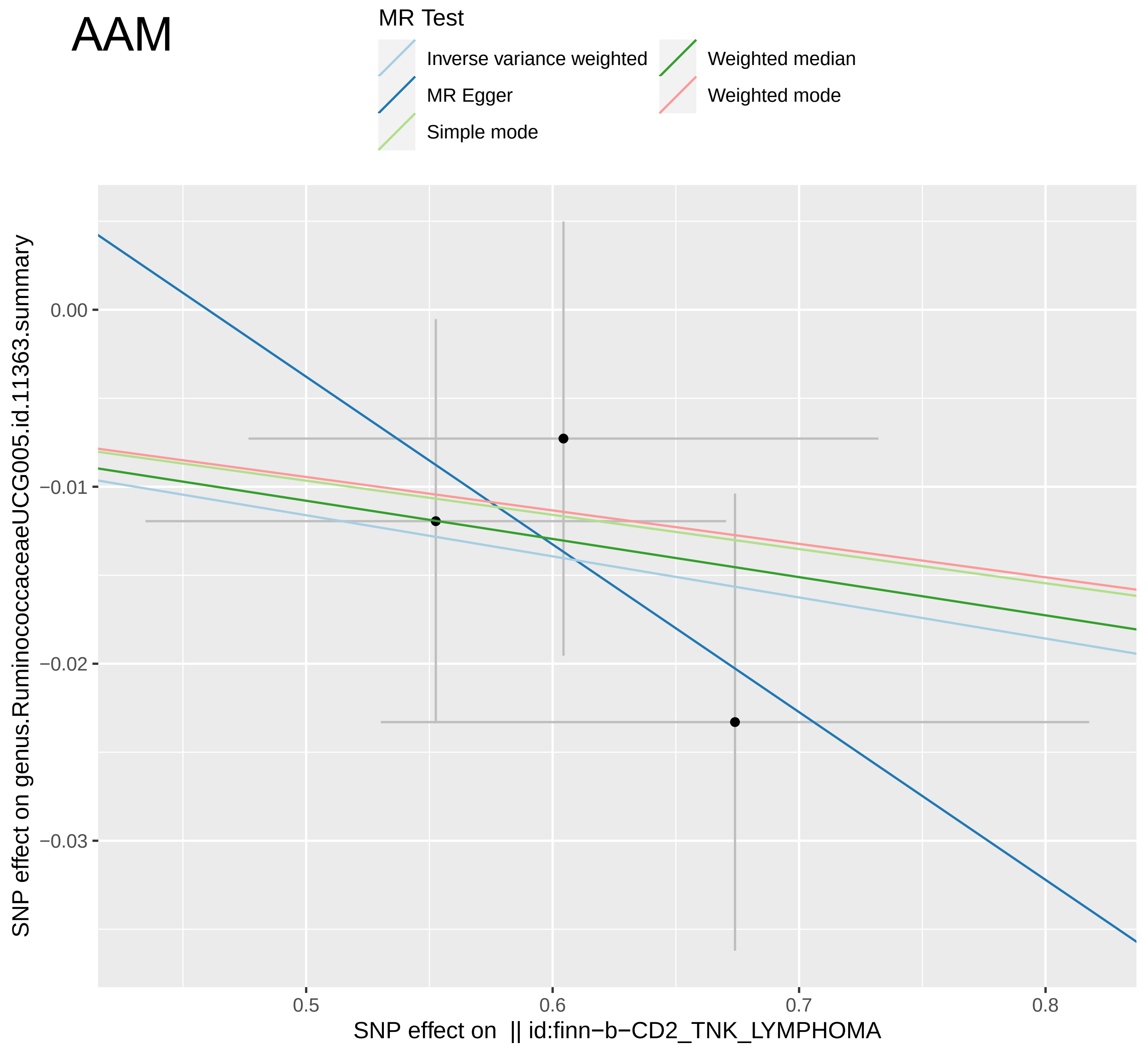
AAM
MR Test
Inverse variance weighted
MR Egger
Simple mode
Weighted median
Weighted mode
0.00
−0.01
−0.02
−0.03
0.5
0.6
0.7
0.8
SNP effect on genus.RuminococcaceaeUCG005.id.11363.summary
SNP effect on || id:finn−b−CD2_TNK_LYMPHOMA

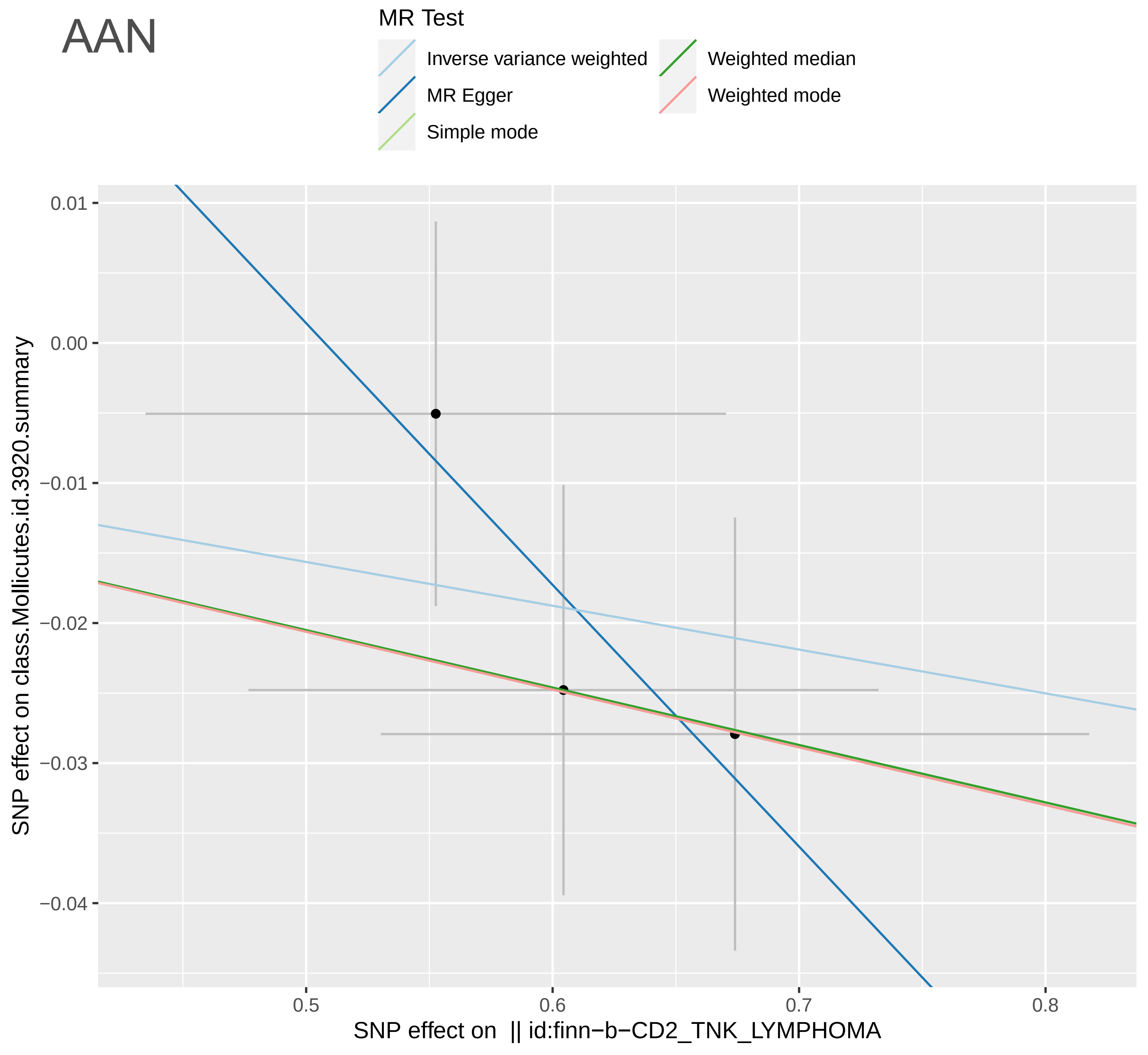

AAN
MR Test
Inverse variance weighted
MR Egger
Simple mode
Weighted median
Weighted mode
0.01
0.00
−0.01
−0.02
−0.03
−0.04
0.5
0.6
0.7
0.8
SNP effect on class.Mollicutes.id.3920.summary
SNP effect on || id:finn−b−CD2_TNK_LYMPHOMA

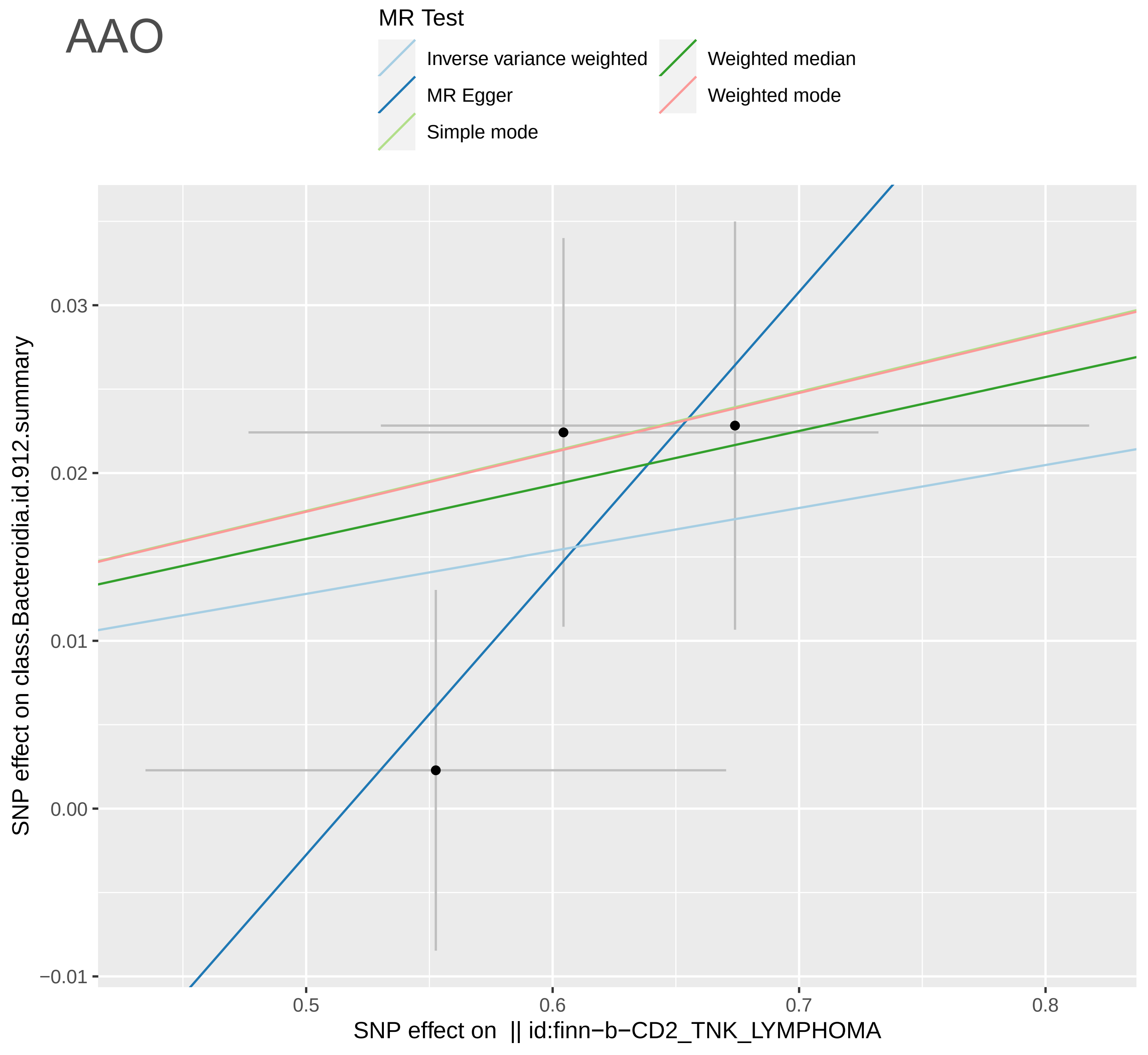

AAO
MR Test
Inverse variance weighted
MR Egger
Simple mode
Weighted median
Weighted mode
0.03
0.02
0.01
0.00
−0.01
SNP effect on class.Bacteroidia.id.912.summary
0.5
0.6
0.7
0.8
SNP effect on || id:finn−b−CD2_TNK_LYMPHOMA

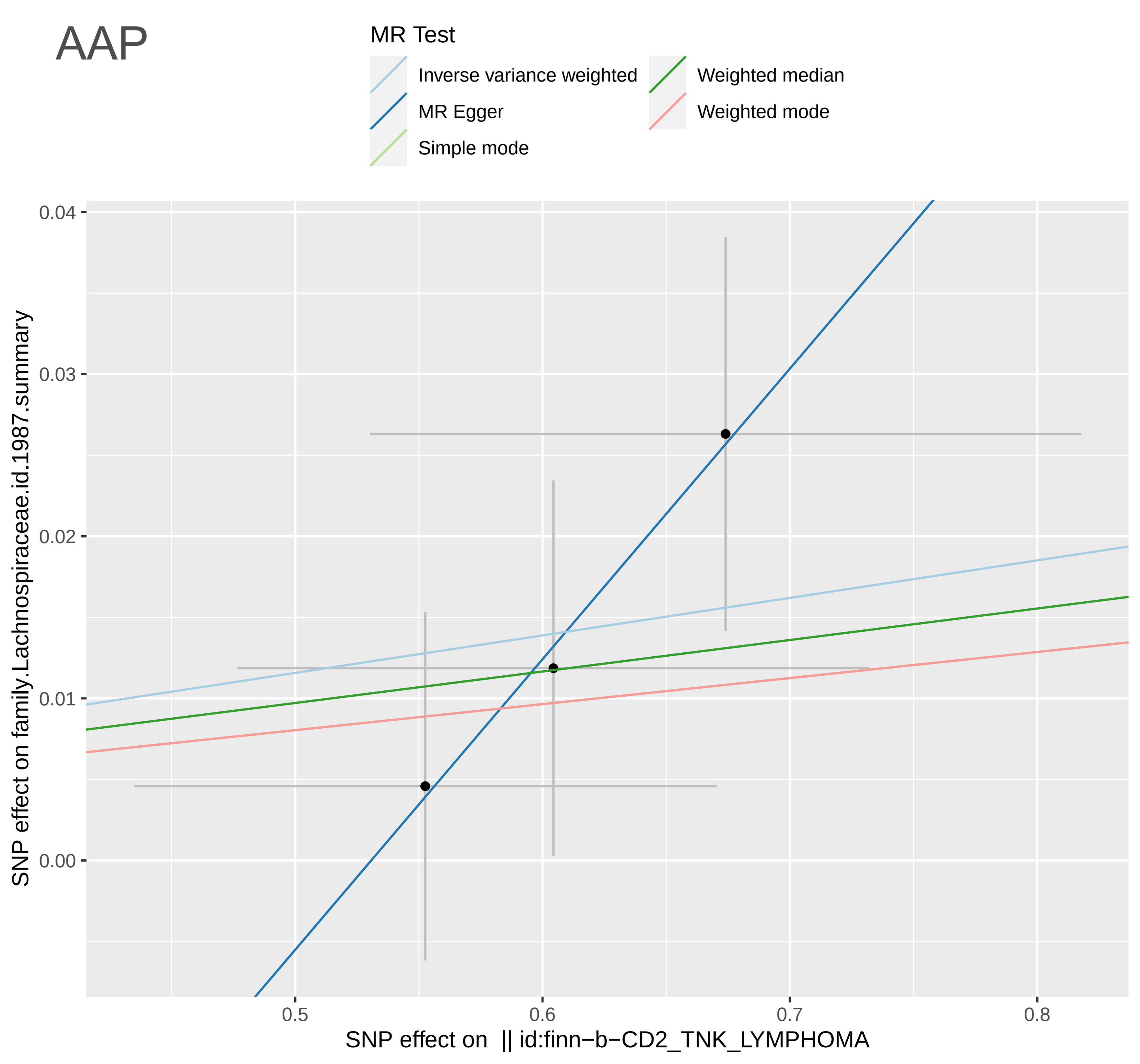

AAP
MR Test
Inverse variance weighted
MR Egger
Simple mode
Weighted median
Weighted mode
0.04
0.03
0.02
0.01
0.00
0.5
0.6
0.7
0.8
SNP effect on family.Lachnospiraceae.id.1987.summary
SNP effect on || id:finn−b−CD2_TNK_LYMPHOMA

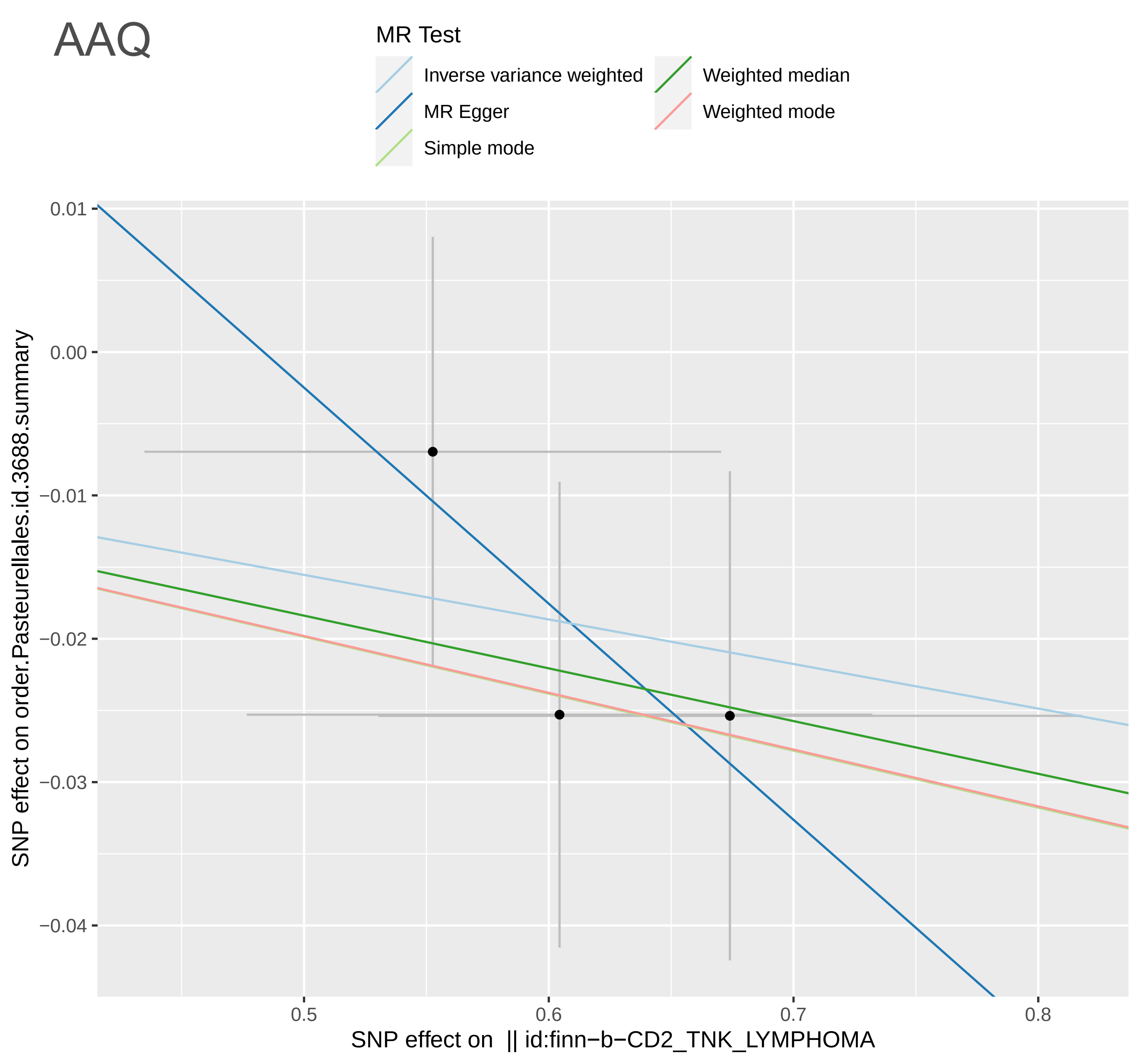
AAQ
MR Test
Inverse variance weighted
MR Egger
Simple mode
Weighted median
Weighted mode
0.01
0.00
−0.01
−0.02
−0.03
−0.04
0.5
0.6
0.7
0.8
SNP effect on order.Pasteurellales.id.3688.summary
SNP effect on || id:finn−b−CD2_TNK_LYMPHOMA

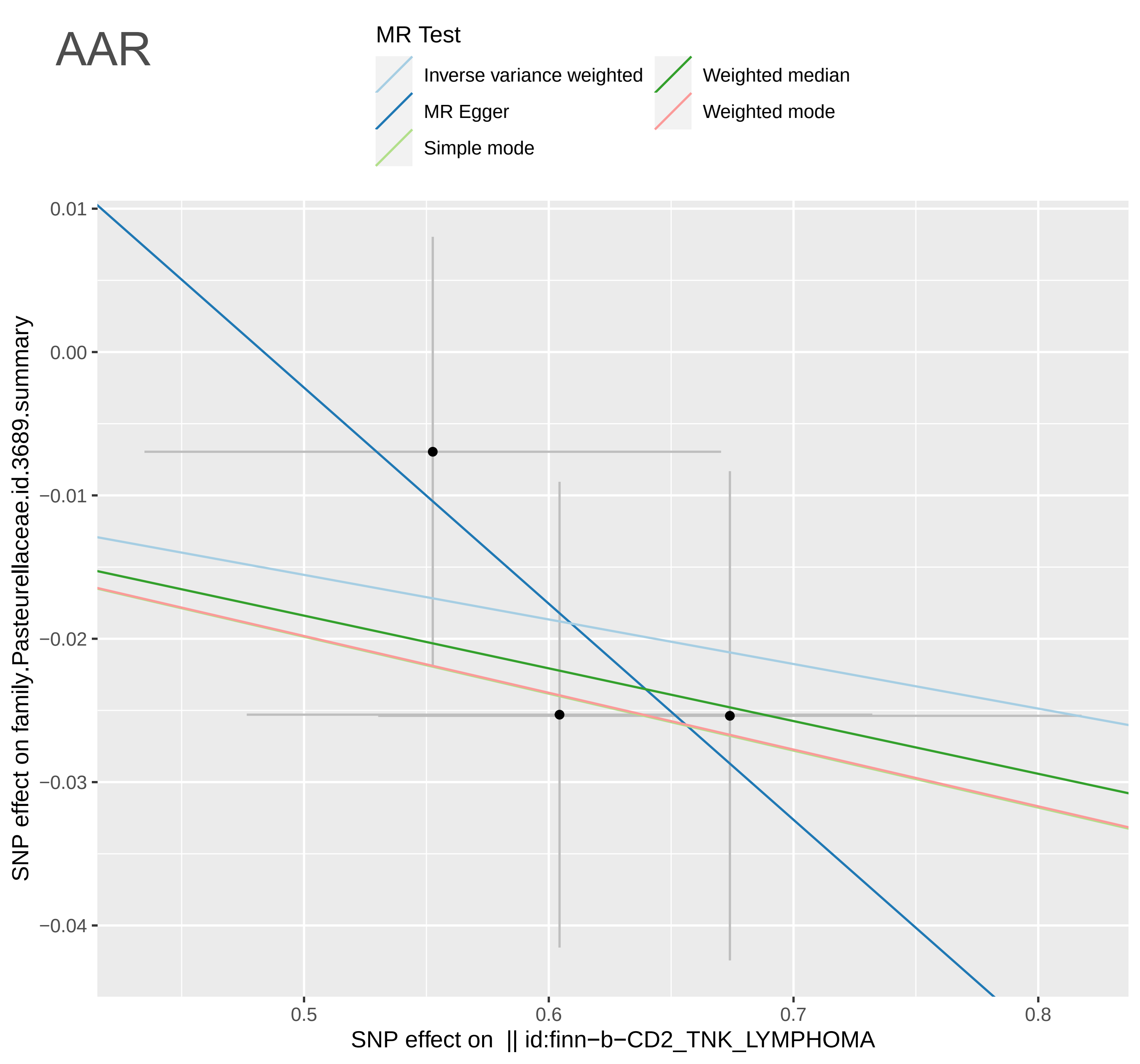

AAR
MR Test
Inverse variance weighted
MR Egger
Simple mode
Weighted median
Weighted mode
0.01
0.00
−0.01
−0.02
−0.03
−0.04
0.5
0.6
0.7
0.8
SNP effect on family.Pasteurellaceae.id.3689.summary
SNP effect on || id:finn−b−CD2_TNK_LYMPHOMA

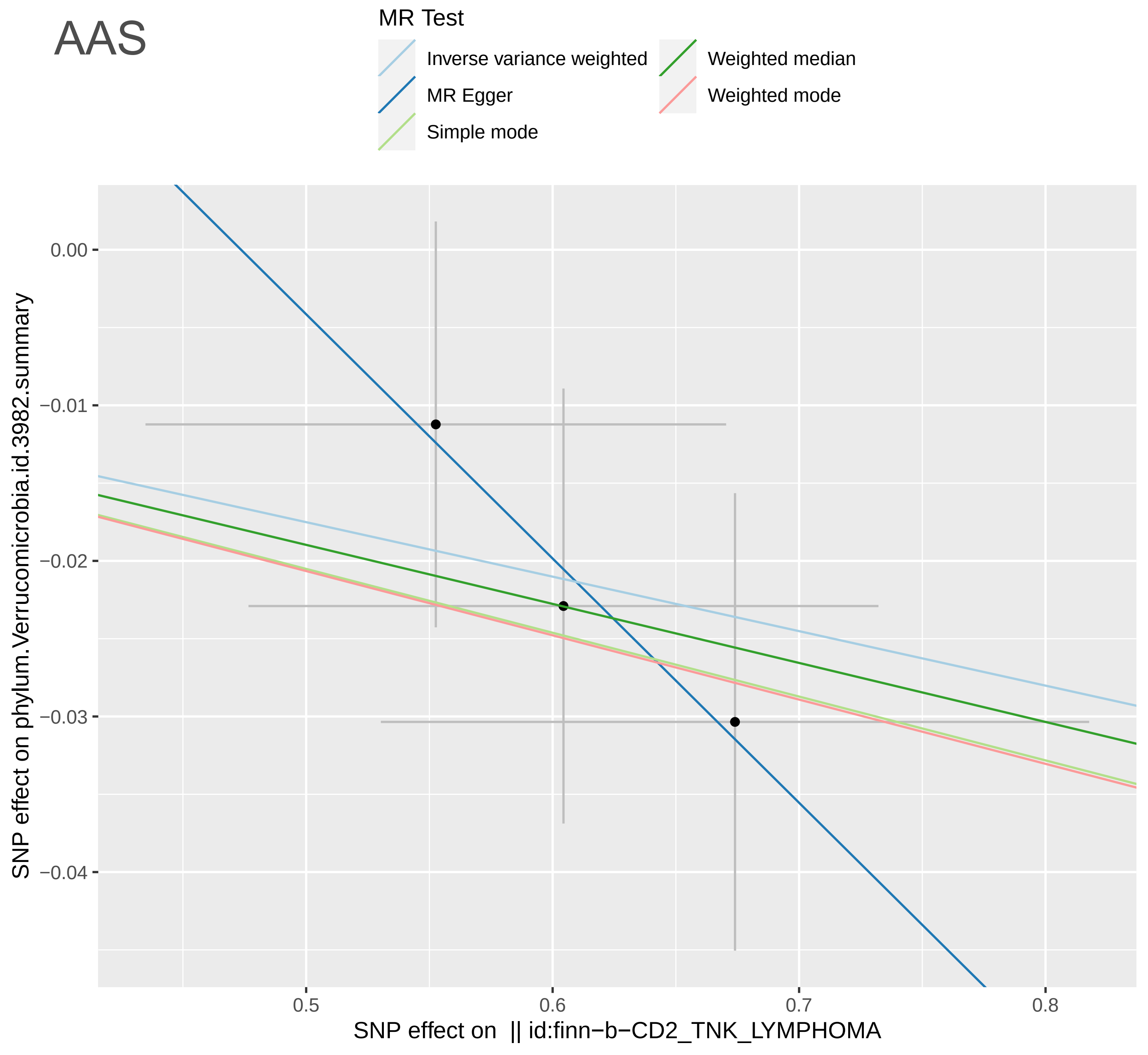

AAS
MR Test
Inverse variance weighted
MR Egger
Simple mode
Weighted median
Weighted mode
0.00
−0.01
−0.02
−0.03
−0.04
0.5
0.6
0.7
0.8
SNP effect on phylum.Verrucomicrobia.id.3982.summary
SNP effect on || id:finn−b−CD2_TNK_LYMPHOMA

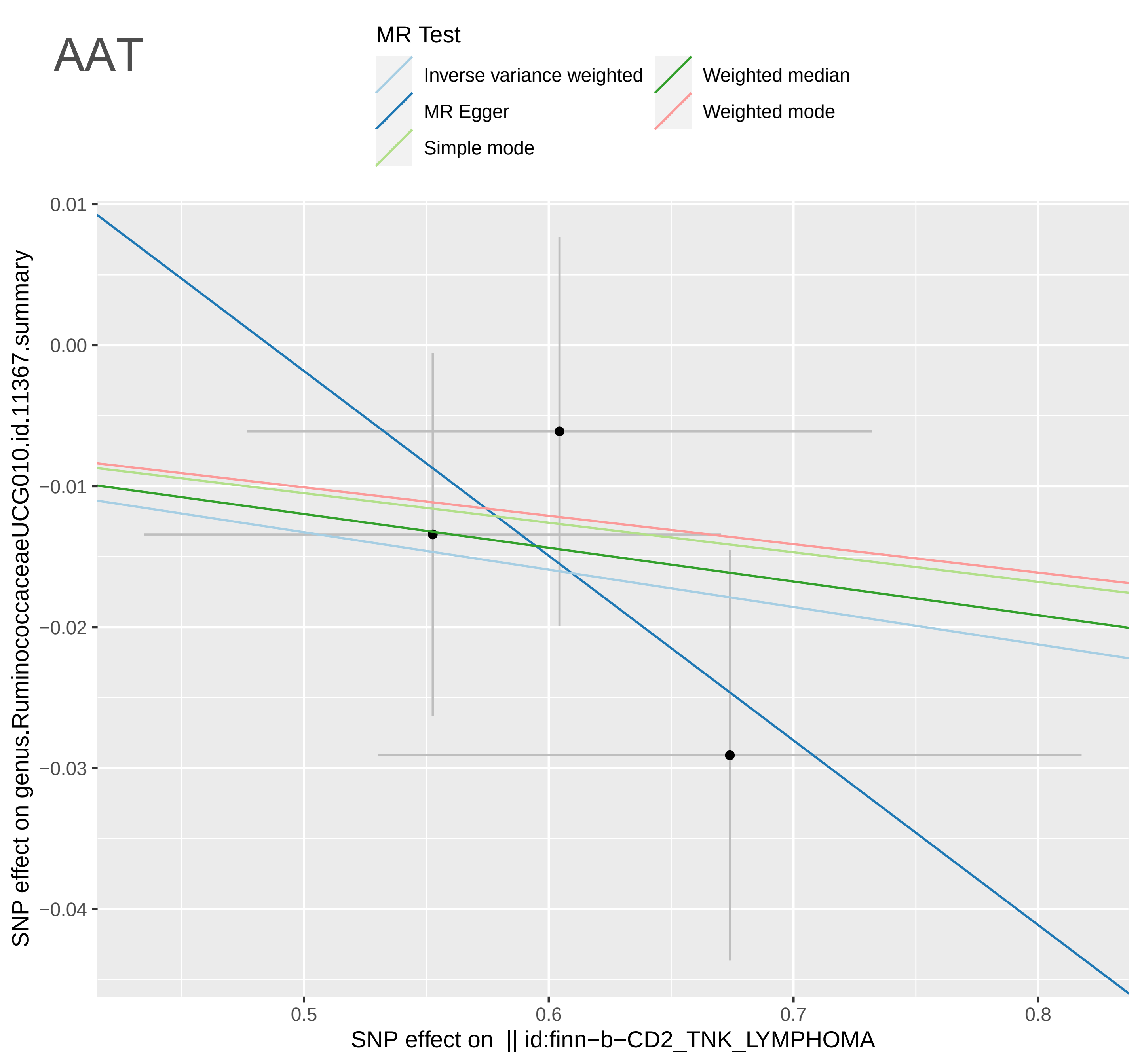
AAT
MR Test
Inverse variance weighted
MR Egger
Simple mode
Weighted median
Weighted mode
0.01
0.00
−0.01
−0.02
−0.03
−0.04
0.5
0.6
0.7
0.8
SNP effect on genus.RuminococcaceaeUCG010.id.11367.summary
SNP effect on || id:finn−b−CD2_TNK_LYMPHOMA

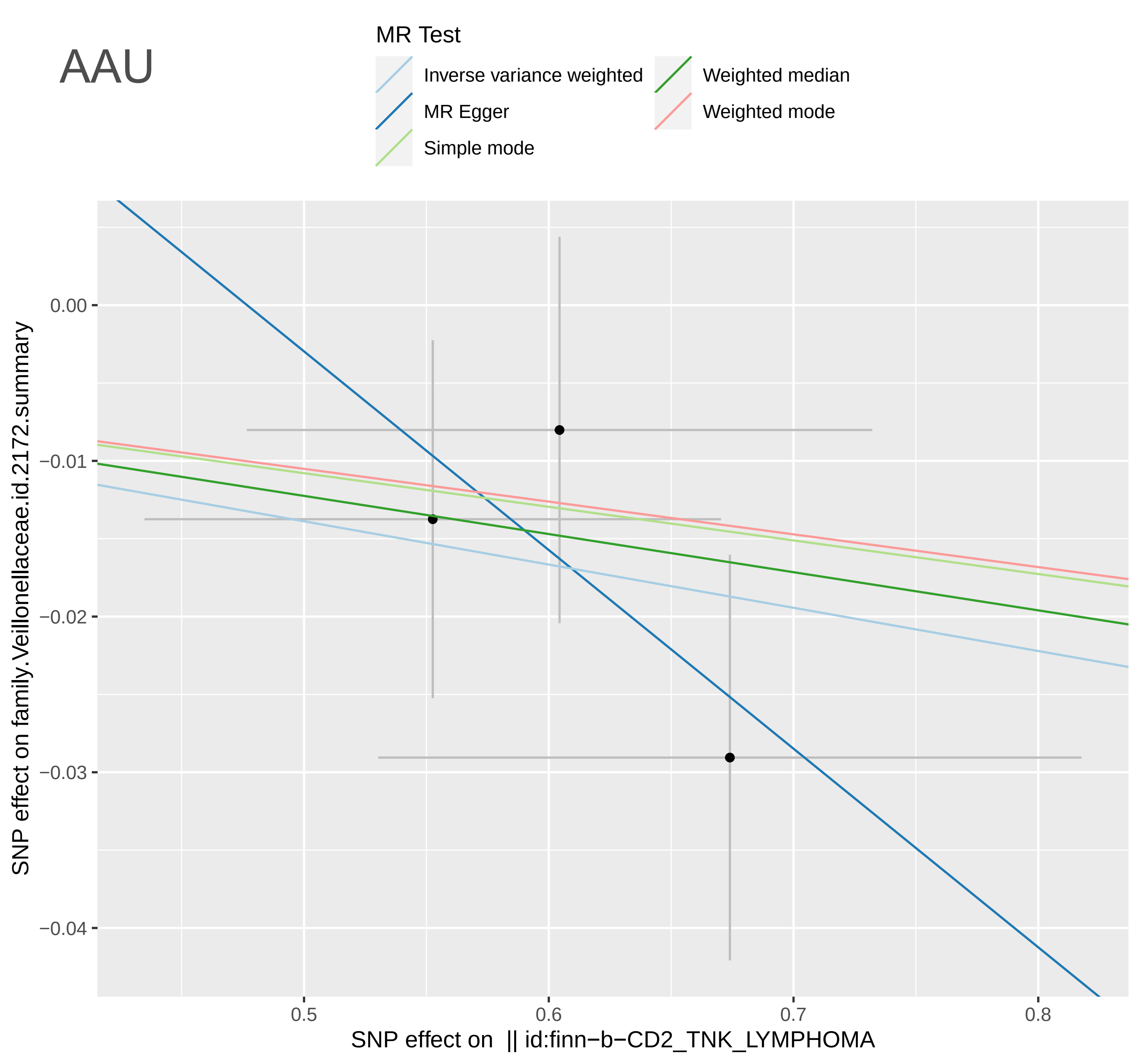
AAU
MR Test
Inverse variance weighted
MR Egger
Simple mode
Weighted median
Weighted mode
0.00
−0.01
−0.02
−0.03
−0.04
0.5
0.6
0.7
0.8
SNP effect on family.Veillonellaceae.id.2172.summary
SNP effect on || id:finn−b−CD2_TNK_LYMPHOMA

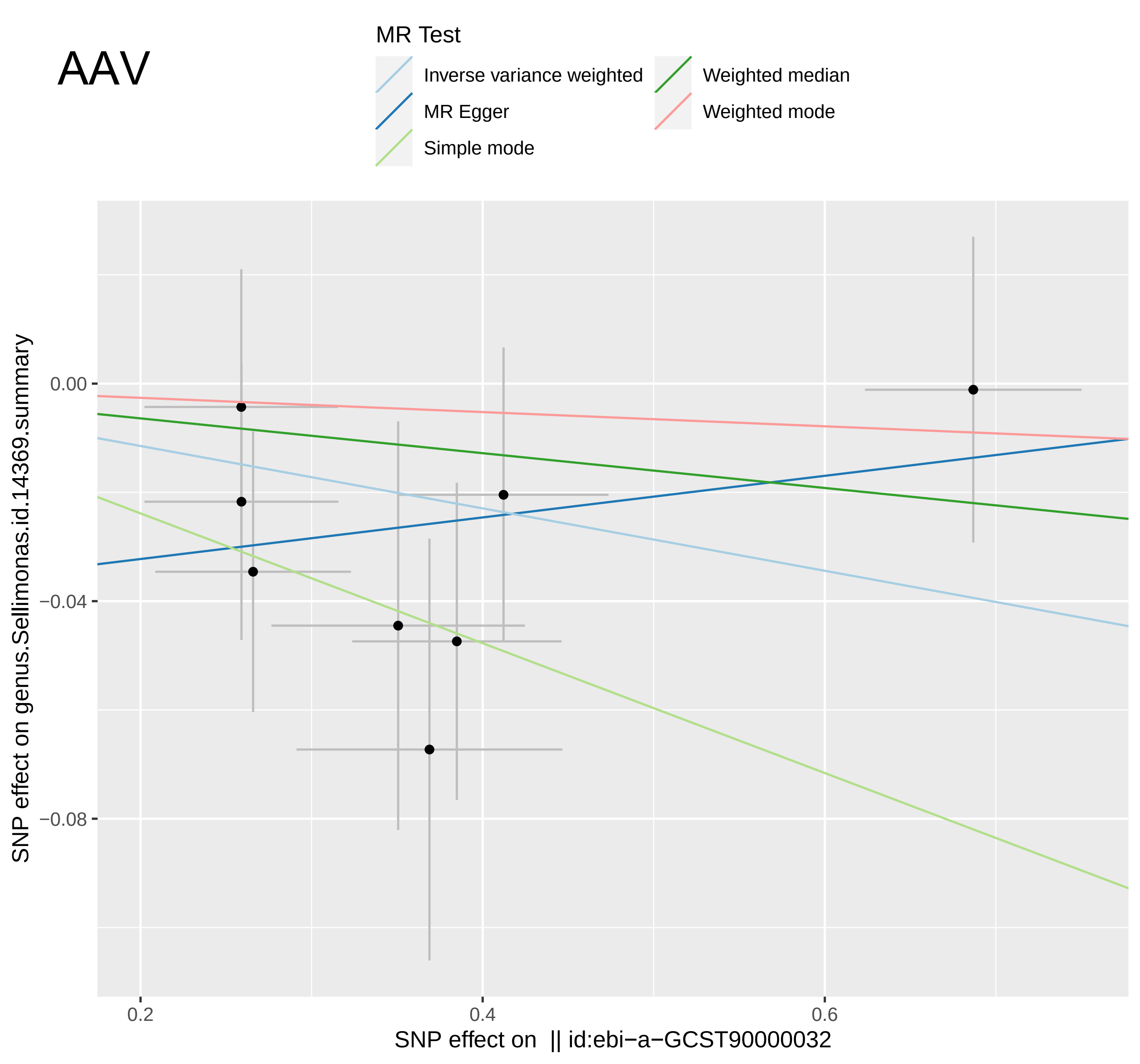

AAV
MR Test
Inverse variance weighted
MR Egger
Simple mode
Weighted median
Weighted mode
SNP effect on genus.Sellimonas.id.14369.summary
0.00
−0.04
−0.08
0.2
0.4
0.6
SNP effect on || id:ebi−a−GCST90000032

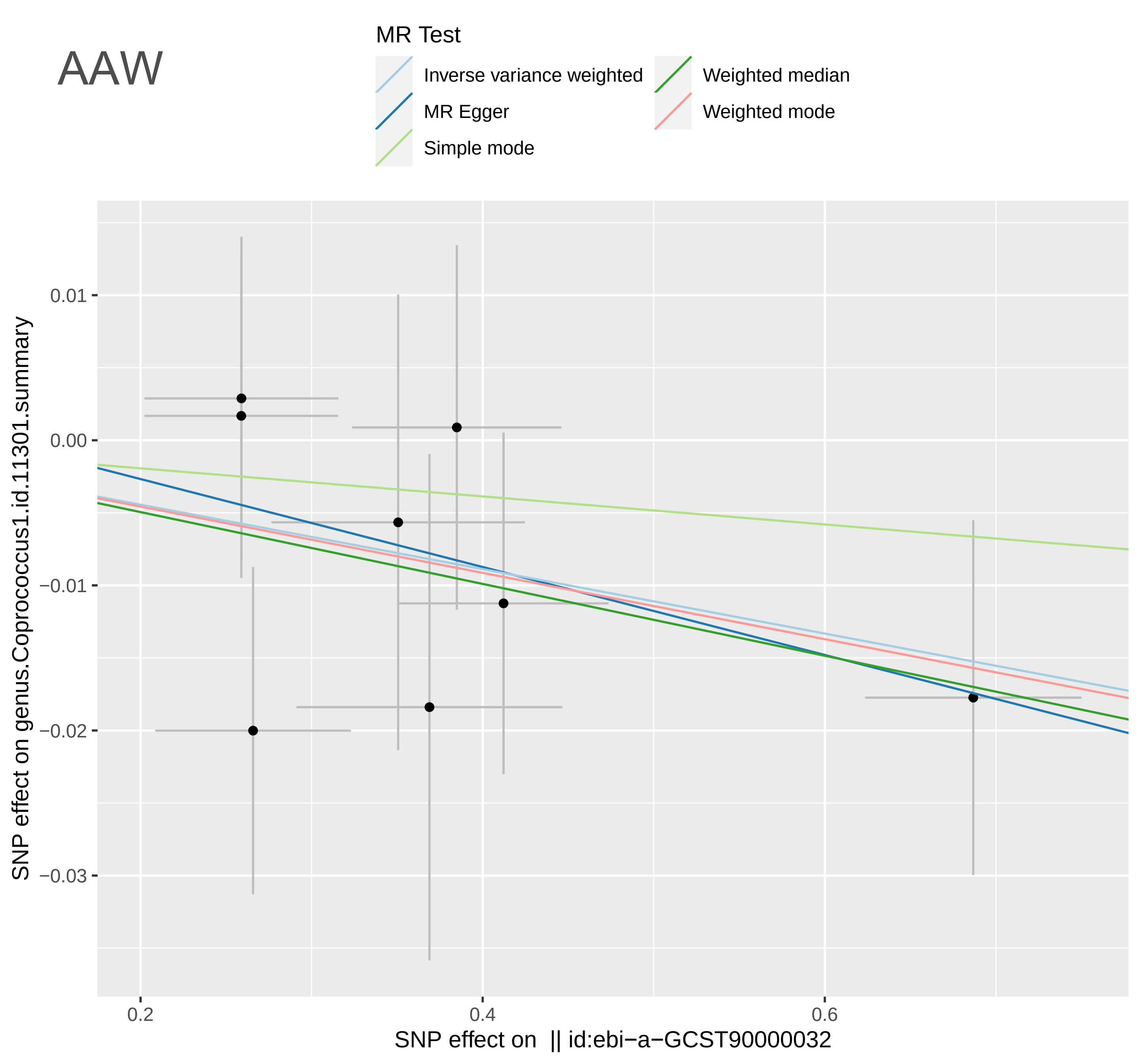

AAW
MR Test
Inverse variance weighted
MR Egger
Simple mode
Weighted median
Weighted mode
0.01
0.00
−0.01
−0.02
−0.03
0.2
0.4
0.6
SNP effect on genus.Coprococcus1.id.11301.summary
SNP effect on || id:ebi−a−GCST90000032

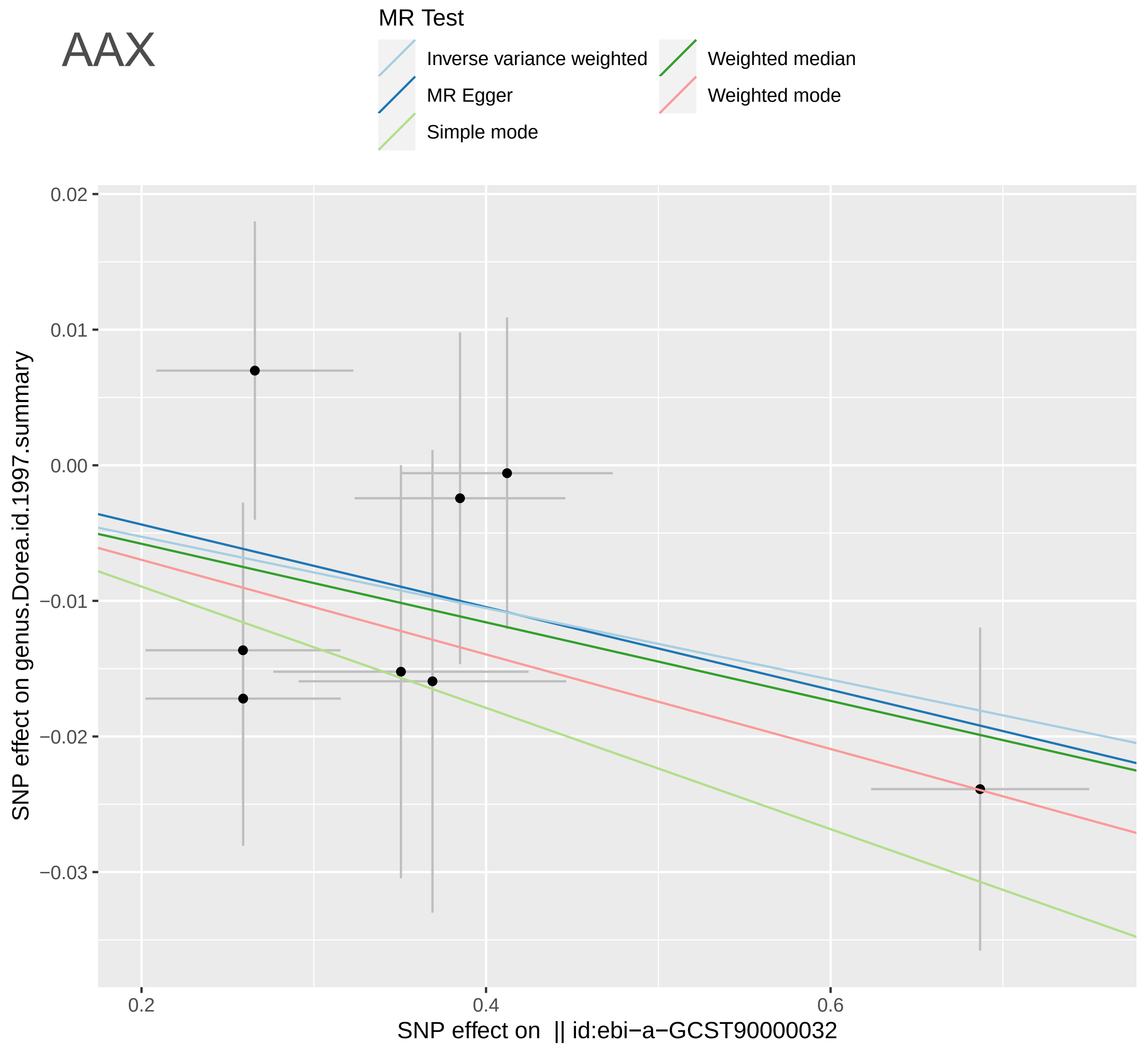

AAX
MR Test
Inverse variance weighted
MR Egger
Simple mode
Weighted median
Weighted mode
0.02
0.01
0.00
−0.01
−0.02
−0.03
SNP effect on genus.Dorea.id.1997.summary
0.2
0.4
0.6
SNP effect on || id:ebi−a−GCST90000032

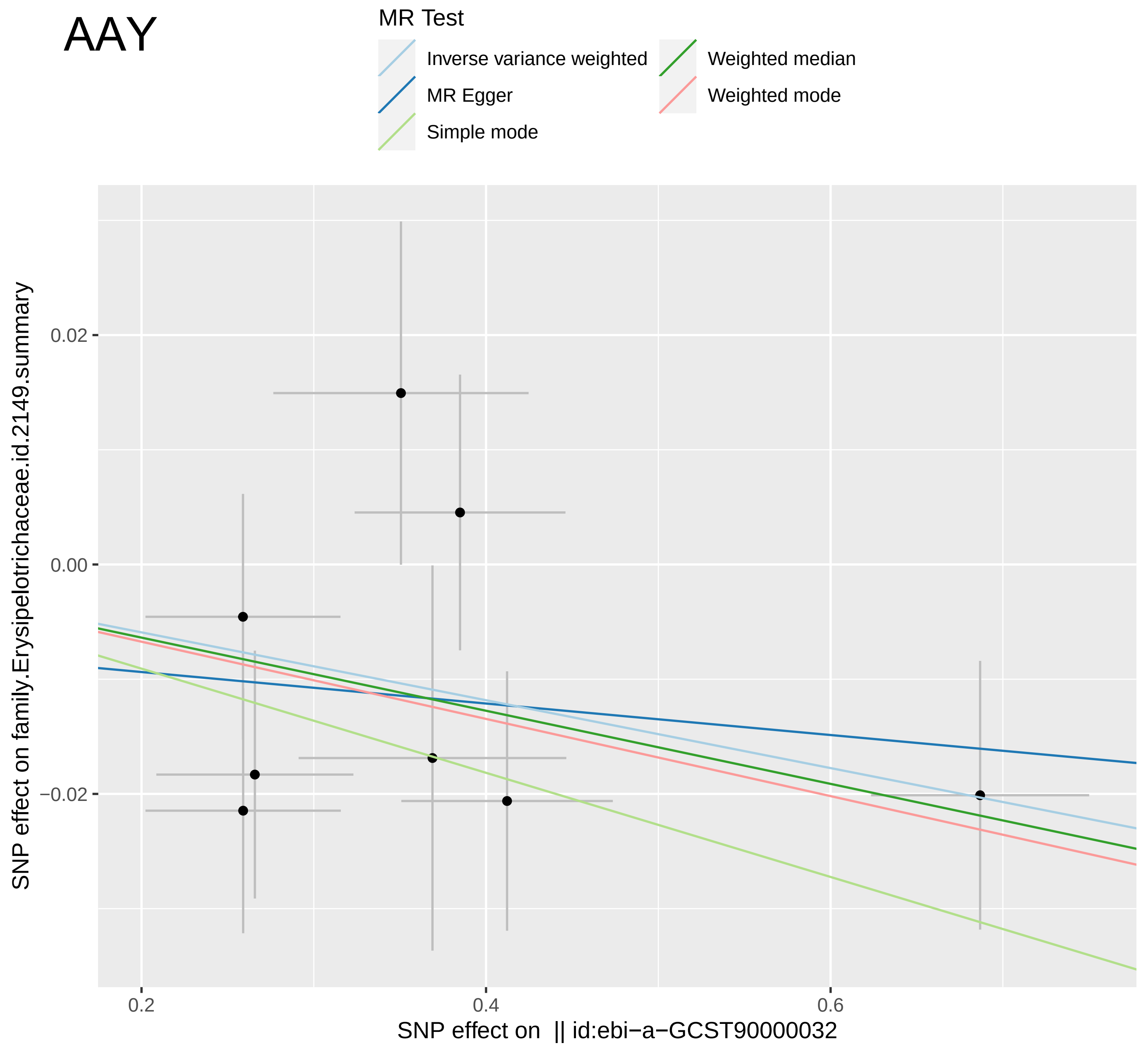

AAY
MR Test
Inverse variance weighted
MR Egger
Simple mode
Weighted median
Weighted mode
0.02
0.00
−0.02
0.2
0.4
0.6
SNP effect on family.Erysipelotrichaceae.id.2149.summary
SNP effect on || id:ebi−a−GCST90000032

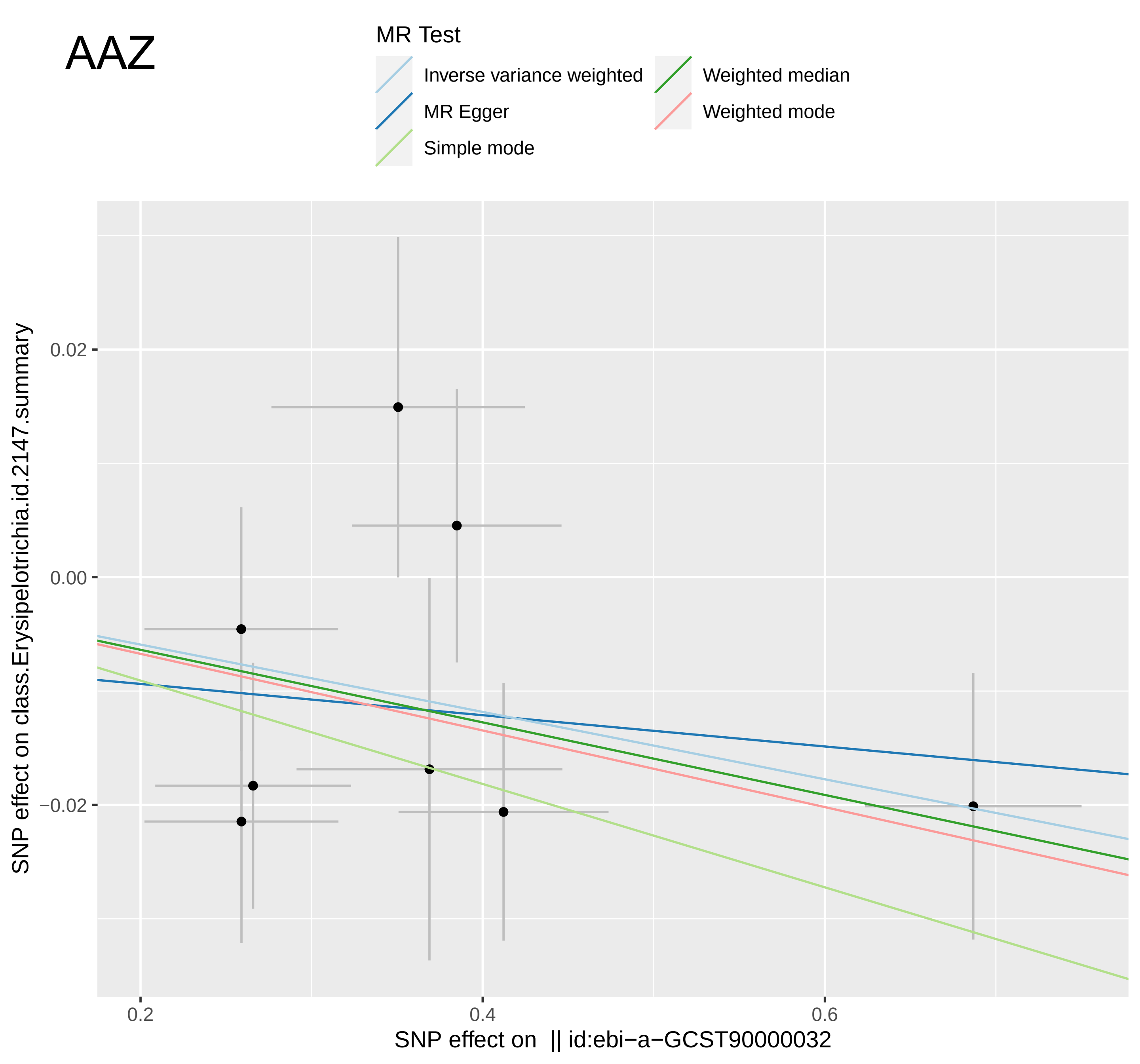
AAZ
MR Test
Inverse variance weighted
MR Egger
Simple mode
Weighted median
Weighted mode
SNP effect on class.Erysipelotrichia.id.2147.summary
0.02
0.00
−0.02
0.2
0.4
0.6
SNP effect on || id:ebi−a−GCST90000032

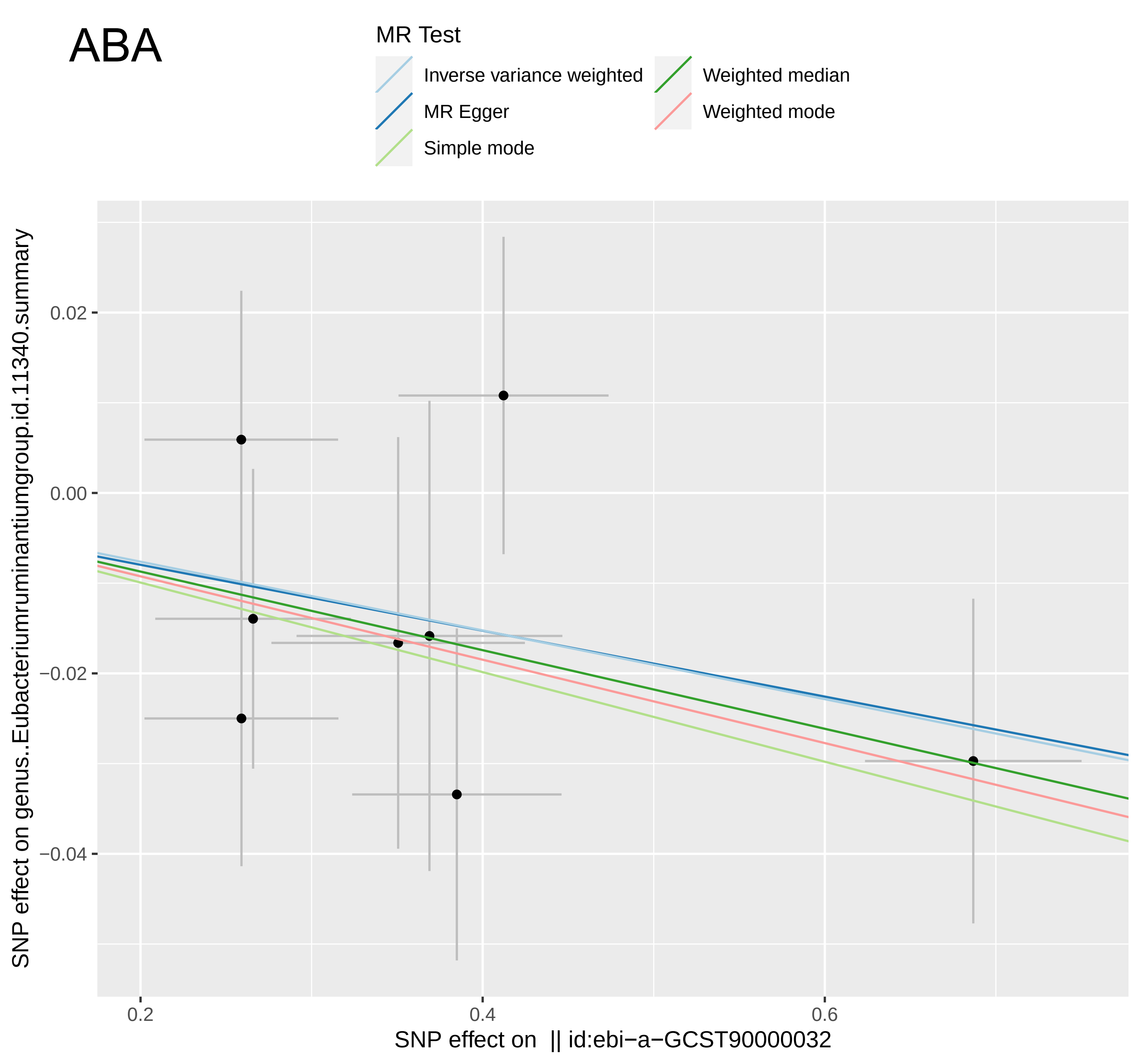
ABA
MR Test
Inverse variance weighted
MR Egger
Simple mode
Weighted median
Weighted mode
SNP effect on genus..Eubacteriumruminantiumgroup.id.11340.summary
0.02
0.00
−0.02
−0.04
0.2
0.4
0.6
SNP effect on || id:ebi−a−GCST90000032

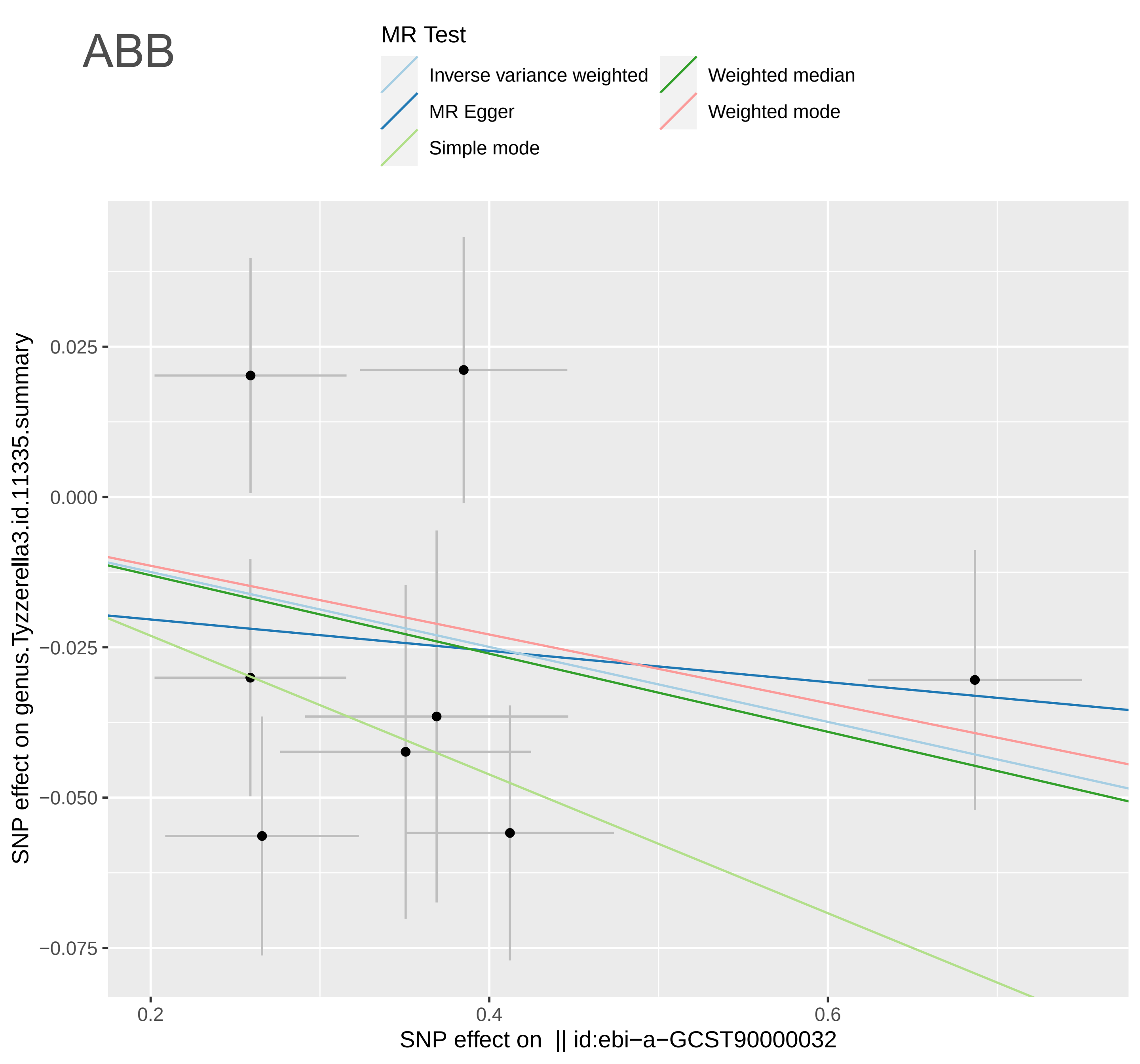
ABB
MR Test
Inverse variance weighted
MR Egger
Simple mode
Weighted median
Weighted mode
0.025
0.000
−0.025
−0.050
−0.075
0.2
0.4
0.6
SNP effect on genus.Tyzzerella3.id.11335.summary
SNP effect on || id:ebi−a−GCST90000032

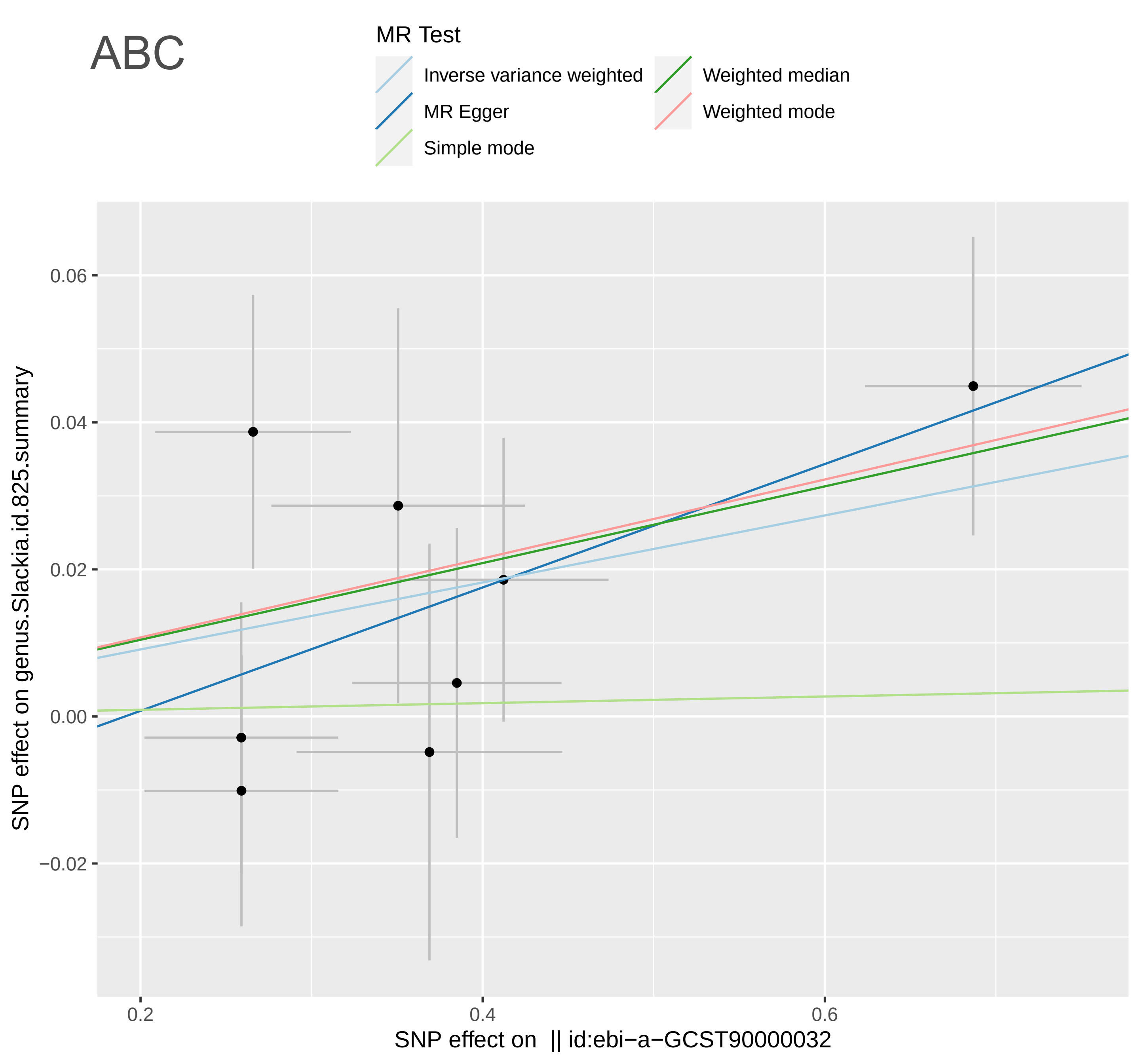

ABC
MR Test
Inverse variance weighted
MR Egger
Simple mode
Weighted median
Weighted mode
SNP effect on genus.Slackia.id.825.summary
0.06
0.04
0.02
0.00
−0.02
0.2
0.4
0.6
SNP effect on || id:ebi−a−GCST90000032

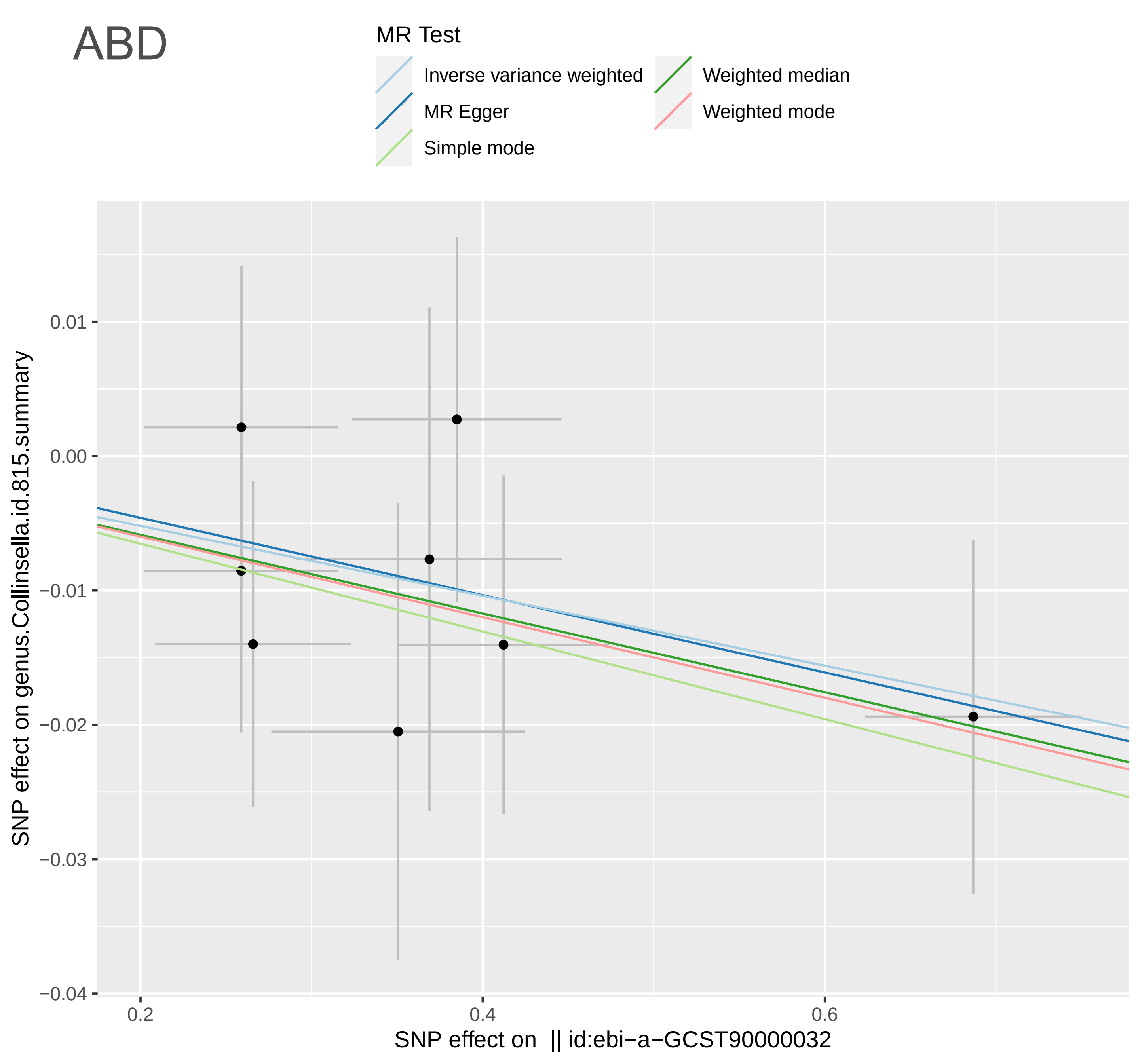

ABD
MR Test
Inverse variance weighted
MR Egger
Simple mode
Weighted median
Weighted mode
SNP effect on genus.Collinsella.id.815.summary
0.01
0.00
−0.01
−0.02
−0.03
−0.04
0.2
0.4
0.6
SNP effect on || id:ebi−a−GCST90000032

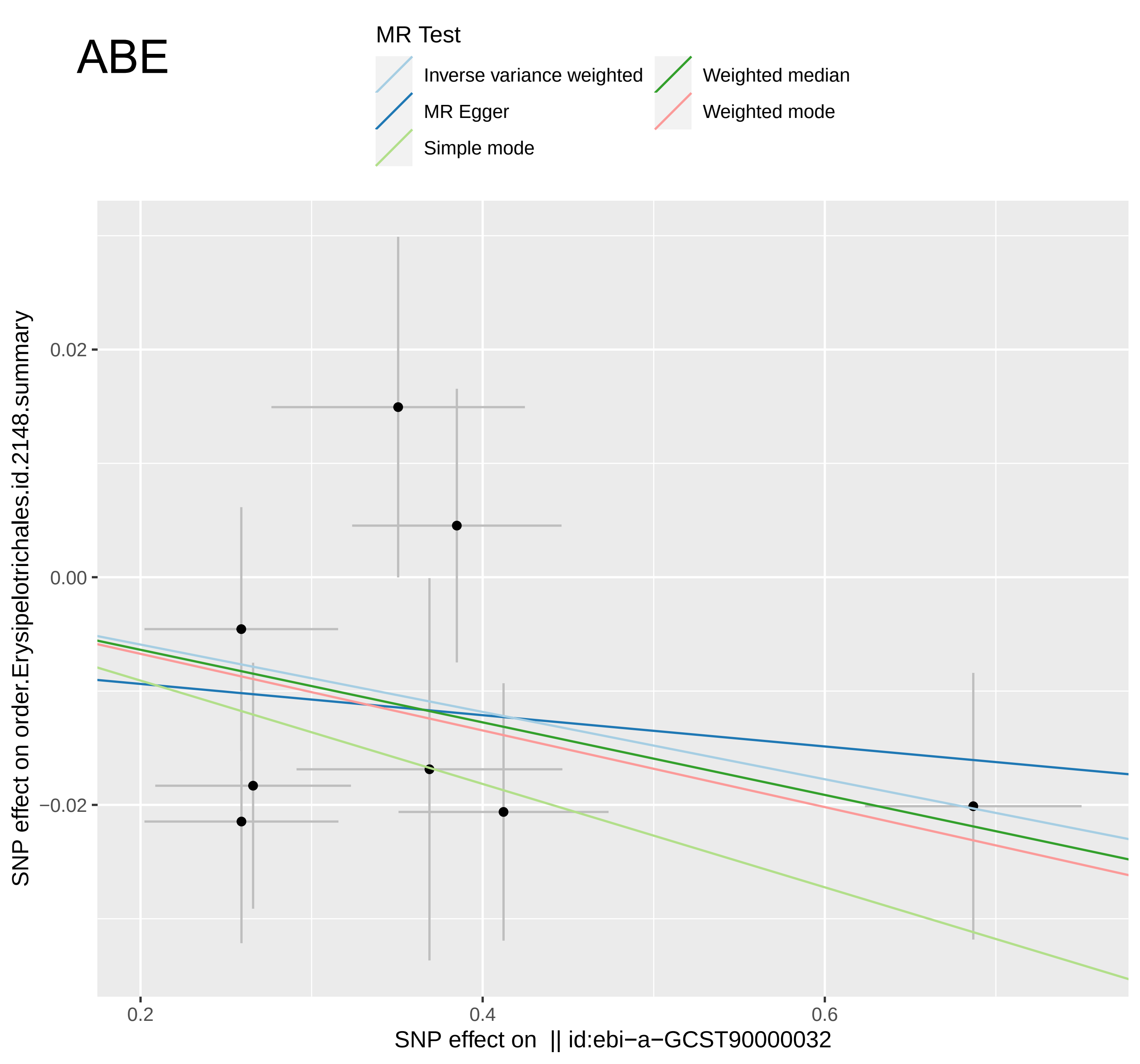
ABE
MR Test
Inverse variance weighted
MR Egger
Simple mode
Weighted median
Weighted mode
SNP effect on order.Erysipelotrichales.id.2148.summary
0.02
0.00
−0.02
0.2
0.4
0.6
SNP effect on || id:ebi−a−GCST90000032

Supplementary figure 8.Sensitivity analysis of the impact of hematologic malignancies on gut microbiota using MR.(scatter plot)

A.Scatter plot between lymphoid leukemia and order.Enterobacteriales

B.Scatter plot between lymphoid leukemia and genus Eubacteriumbrachygroup

C.Scatter plot between lymphoid leukemia and genus.LachnospiraceaeNC2004group

D.Scatter plot between lymphoid leukemia and genus.Anaerofilum.

E.Scatter plot between lymphoid leukemia and genus.Butyrivibrio

F.Scatter plot between lymphoid leukemia and genus.Escherichia.Shigella

G.Scatter plot between lymphoid leukemia and family.Enterobacteriaceae

H.Scatter plot between lymphoid leukemia and genus.Fusicatenibacter

I.Scatter plot between lymphoid leukemia and genus.LachnospiraceaeUCG010

J.Scatter plot between myeloid leukemia and family.Bifidobacteriaceae

K.Scatter plot between myeloid leukemia and class.Actinobacteria

L.Scatter plot between myeloid leukemia and class.Clostridia

M.Scatter plot between myeloid leukemia and phylum.Bacteroidetes

N.Scatter plot between myeloid leukemia and genus..Ruminococcusgauvreauiigroup

O.Scatter plot between myeloid leukemia and order.Clostridiales

P.Scatter plot between myeloid leukemia and phylum.Actinobacteria.

Q.Scatter plot between myeloid leukemia and order.Bacteroidales

R.Scatter plot between myeloid leukemia and class.Bacteroidia

S.Scatter plot between myeloid leukemia and genus.Bifidobacterium

T.Scatter plot between myeloid leukemia and family.Lachnospiraceae

U.Scatter plot between myeloid leukemia and genus.Collinsella

V.Scatter plot between myeloid leukemia and genus.Blautia

W.Scatter plot between myeloid leukemia and genus.Fusicatenibacter

X.Scatter plot between myeloid leukemia and order.Bifidobacteriales

Y.Scatter plot between myeloid leukemia and genus..Ruminococcusgnavusgroup

Z.Scatter plot between myeloid leukemia and genus..Eubacteriumhalliigroup

AA..Scatter plot between myeloid leukemia and genus.Slackia.

AB.Scatter plot between myeloid leukemia and phylum.Firmicutes

AC.Scatter plot between Hodgkin lymphoma and phylum.Verrucomicrobia

AD.Scatter plot between Hodgkin lymphoma and family.ClostridialesvadinBB60group

AE.Scatter plot between Hodgkin lymphoma and genus.RuminococcaceaeUCG010

AF.Scatter plot between Hodgkin lymphoma and genus.Odoribacter

AG.Scatter plot between Hodgkin lymphoma and genus.RuminococcaceaeUCG005

AH.Scatter plot between Hodgkin lymphoma and genus.RuminococcaceaeUCG013

AI. Scatter plot between Hodgkin lymphoma and genus.Ruminiclostridium9

AJ.Scatter plot between Hodgkin lymphoma and genus.RuminococcaceaeNK4A214group

AK.Scatter plot between Hodgkin lymphoma and genus..Eubacteriumventriosumgroup

AL.Scatter plot between Hodgkin lymphoma and genus.RuminococcaceaeUCG003

AM.Scatter plot between malignant plasma cell tumor and family.Defluviitaleaceae

AN. Scatter plot between malignant plasma cell tumor and genus.DefluviitaleaceaeUCG011

AO.Scatter plot between malignant plasma cell tumor and genus..Eubacteriumrectalegroup

AP.Scatter plot between malignant plasma cell tumor and genus.CandidatusSoleaferrea

AQ. Scatter plot between follicular lymphoma and genus.Allisonella

AR.Scatter plot between follicular lymphoma and genus.RikenellaceaeRC9gutgroup

AS.Scatter plot between follicular lymphoma and genus.Faecalibacterium

AT.Scatter plot between follicular lymphoma and family.Clostridiaceae

AU.Scatter plot between follicular lymphoma and genus.DefluviitaleaceaeUCG011.

AV.Scatter plot between follicular lymphoma and genus.Clostridiumsensustricto

AW.Scatter plot between follicular lymphoma and family.Defluviitaleaceae

AX.Scatter plot between diffuse large B-cell lymphomaand family.Veillonellaceae

AY.Scatter plot between diffuse large B-cell lymphomaand genus.Fusicatenibacter

AZ.Scatter plot between diffuse large B-cell lymphomaand genus.Eggerthella

AAA.Scatter plot between diffuse large B-cell lymphomaand genus.Erysipelatoclostridium

AAB.Scatter plot between diffuse large B-cell lymphomaand genus.Blautia

AAC.Scatter plot between diffuse large B-cell lymphomaand genus.RuminococcaceaeUCG009

AAD.Scatter plot between mature T/NK-cell lymphomas and order.Verrucomicrobiales

AAE.Scatter plot between mature T/NK-cell lymphomas and phylum.Tenericutes

AAF.Scatter plot between mature T/NK-cell lymphomas and genus.Lachnoclostridium

AAG.Scatter plot between mature T/NK-cell lymphomas and phylum.Bacteroidetes

AAH.Scatter plot between mature T/NK-cell lymphomas and class.Verrucomicrobiae

AAI.Scatter plot between mature T/NK-cell lymphomas and genus..Eubacteriumrectalegroup

AAJ.Scatter plot between mature T/NK-cell lymphomas and family.Verrucomicrobiaceae

AAK.Scatter plot between mature T/NK-cell lymphomas and order.Bacteroidales

AAL. Scatter plot between mature T/NK-cell lymphomas and genus.Akkermansia

AAM. Scatter plot between mature T/NK-cell lymphomas and genus.RuminococcaceaeUCG005

AAN.Scatter plot between mature T/NK-cell lymphomas and class.Mollicutes.

AAO. Scatter plot between mature T/NK-cell lymphomas and class.Bacteroidia

AAP.Scatter plot between mature T/NK-cell lymphomas and family.Lachnospiraceae

AAQ.Scatter plot between mature T/NK-cell lymphomas and order.Pasteurellales

AAR. Scatter plot between mature T/NK-cell lymphomas and family.Pasteurellaceae.

AAS. Scatter plot between mature T/NK-cell lymphomas and phylum.Verrucomicrobia

AAT.Scatter plot between mature T/NK-cell lymphomas and genus.RuminococcaceaeUCG010

AAU.Scatter plot between mature T/NK-cell lymphomas and family.Veillonellaceae.

AAV.Scatter plot between myeloproliferative neoplasms and genus.Sellimonas

AAW.Scatter plot between myeloproliferative neoplasms and genus.Coprococcus1

AAX.Scatter plot between myeloproliferative neoplasms and genus.Dorea

AAY.Scatter plot between myeloproliferative neoplasms and family.Erysipelotrichaceae

AAZ.Scatter plot between myeloproliferative neoplasms and class.Erysipelotrichia

ABA.Scatter plot between myeloproliferative neoplasms and genus..Eubacteriumruminantiumgroup

ABB.Scatter plot between myeloproliferative neoplasms and genus.Tyzzerella3

ABC.Scatter plot between myeloproliferative neoplasms and genus.Slackia

ABD.Scatter plot between myeloproliferative neoplasms and genus.Collinsella

ABE.Scatter plot between myeloproliferative neoplasms and order.Erysipelotrichales
